# A divergent intermediate strategy yields biologically diverse pseudo-natural products

**DOI:** 10.1038/s41557-024-01458-4

**Published:** 2024-02-16

**Authors:** Sukdev Bag, Jie Liu, Sohan Patil, Jana Bonowski, Sandra Koska, Beate Schölermann, Ruirui Zhang, Lin Wang, Axel Pahl, Sonja Sievers, Lukas Brieger, Carsten Strohmann, Slava Ziegler, Michael Grigalunas, Herbert Waldmann

**Affiliations:** 1 Department of Chemical Biology, Max Planck Institute of Molecular Physiology, Dortmund, Germany; 2 Faculty of Chemistry and Chemical Biology, TU Dortmund University, Dortmund, Germany; 3 Compound Management and Screening Center, Dortmund, Germany; 4 Faculty of Chemistry and Chemical Biology, Inorganic Chemistry, TU Dortmund University, Dortmund, Germany

**Keywords:** Chemical libraries, Chemical libraries

## Abstract

The efficient exploration of biologically relevant chemical space is essential for the discovery of bioactive compounds. A molecular design principle that possesses both biological relevance and structural diversity may more efficiently lead to compound collections that are enriched in diverse bioactivities. Here the diverse pseudo-natural product (PNP) strategy, which combines the biological relevance of the PNP concept with synthetic diversification strategies from diversity-oriented synthesis, is reported. A diverse PNP collection was synthesized from a common divergent intermediate through developed indole dearomatization methodologies to afford three-dimensional molecular frameworks that could be further diversified via intramolecular coupling and/or carbon monoxide insertion. In total, 154 PNPs were synthesized representing eight different classes. Cheminformatic analyses showed that the PNPs are structurally diverse between classes. Biological investigations revealed the extent of diverse bioactivity enrichment of the collection in which four inhibitors of Hedgehog signalling, DNA synthesis, de novo pyrimidine biosynthesis and tubulin polymerization were identified from four different PNP classes.

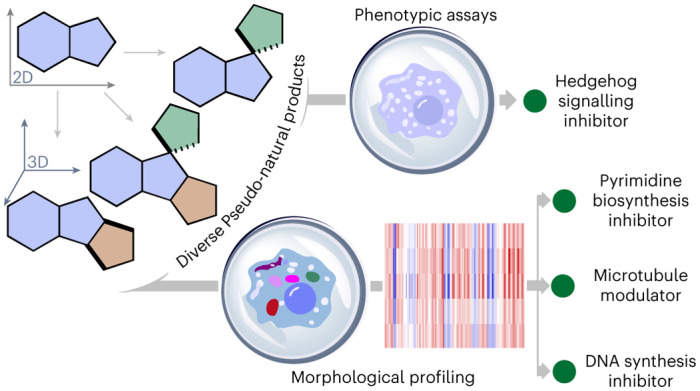

## Abstract (web summary)

Design strategies that possess both biological relevance and structural diversity may lead to compound collections that are enriched in diverse bioactivities. Now a diverse pseudo-natural product design principle has been established to efficiently explore biologically relevant chemical space. Through dearomatization reactions, a compound collection enriched in both structural and biological diversity was rapidly generated.

## Main

The strategic navigation of biologically relevant chemical space for the discovery of bioactive small molecules is a core principle of chemical biology and drug discovery programmes^1,2^. Through evolution, nature has explored biologically relevant chemical space to afford current natural product (NP) structures that represent biologically prevalidated chemical matter. NPs have been and continue to be a rich source of chemical probes and therapeutics^3^; however, the development of NPs into chemical probes and/or therapeutics can be restricted by low availability and insufficient access to derivatives.

Several molecular design principles based on NP structures, such as function-oriented synthesis^4–6^, biology-oriented synthesis^7,8^, dynamic retrosynthetic analysis^9,10^ and others^11^, have successfully addressed these issues by providing compounds that retain the bioactivities of the NPs of interest but are more synthetically tractable than the guiding NPs. Nevertheless, these methods are inherently limited since the resulting compounds have core scaffolds that are present in the guiding NPs and therefore are likely to have the same bioactivities as the guiding NPs. NPs, as well as design principles that are derivatives of NP scaffolds, are also limited due to evolutionary constraints. Natural evolution is a very slow process that has resulted in NPs occupying only a fraction of theoretical NP-like chemical space^12^. Therefore, relying solely on current NP scaffolds, which are synthesized via existing biosynthetic pathways, has limitations in molecular discovery.

**Fig. 1:**
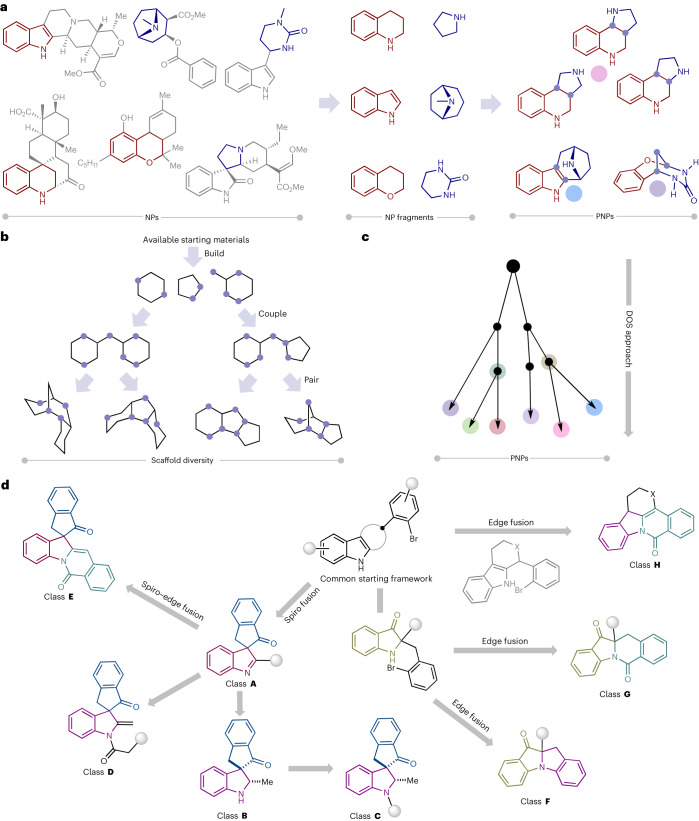
General depiction of the PNP and DOS concepts. **a**, PNP logic: the combination of NP fragments in unprecedented arrangements. **b**, DOS logic: scaffold diversity using a build–couple–pair strategy from available starting materials. **c**, Merging the two concepts of PNPs and DOS, generation of dPNPs. **d**, An overview of dPNP compound class syntheses from common indole-based starting materials.

Employing methods that are inspired by nature and go beyond current biosynthetic pathways may facilitate the exploration of biologically relevant NP-like chemical space^13,14^. The complexity-to-diversity approach is a chemical extension of natural biosynthetic pathways in which suitable NPs or synthetic NP-like compounds are subjected to ring distortion reactions to afford compound collections with diverse scaffolds that are distinct from the starting NP yet retain the biological relevance and complexity of NPs^15–17^. We have recently introduced a design principle for pseudo-natural products (PNPs) through the de novo combination of NP fragments (Fig. 1a)^18–24^. The PNP concept identifies fragments characteristic in different NP classes and combines them in arrangements that are novel and not accessible by known biosynthetic pathways. The PNP scaffolds are not known to be produced in nature but may retain the biological relevance of NPs and therefore have diverse bioactivity profiles and targets. Different NP fragments can be combined in different arrangements and connectivity patterns to access various PNP scaffolds that have diverse bioactivity profiles^25–27^. However, access to diverse PNPs can be laborious in which typically specific starting materials must be made for each scaffold.

To access diverse scaffolds more rapidly, we envisioned developing a design principle for PNP compound collections based on a common divergent intermediate, whereby one common intermediate could give rise to a variety of PNP scaffolds that may possess various bioactivities. In this context, the diversity-oriented synthesis (DOS) strategy^28–32^ can cover a wide area of chemical space to generate compound collections with diverse and complex scaffolds, with a build–couple–pair approach as one example^31^ (Fig. 1b). DOS does lead to diverse compound collections that may have NP-like features that positively contribute to biological performance^33^, such as high fraction of *sp*^3^-hybridized centres and stereogenic content. However, in our perspective, DOS, unlike the PNP design principle, is not directly based on NP structures, and therefore scaffolds resulting from DOS do not necessarily include fragments of biological relevance^18^. Thus, we envisioned combining the two logics of DOS and PNPs to generate a compound collection that incorporates both scaffold diversity and biological relevance, termed as diverse PNPs (dPNPs, Fig. 1c).

In this Article, we report a strategy to access dPNP scaffolds from a common divergent intermediate. Chemical methodologies for indole dearomatization^34–38^ were developed to streamline scaffold construction including intramolecular indole dearomatizations via a σ-acylpalladium intermediate through in situ-generated carbon monoxide (CO). Dearomatization is a powerful approach to access underexplored three-dimensional topologies^39^ from flat aromatic compounds. Furthermore, dearomatization can introduce new stereogenic centre(s) and increase the fraction of *sp*^3^ carbons, both of which are favourable properties for target-binding selectivity and clinical progression of drug candidates^33,40^. The dearomatization of indoles and coupling and/or pairing of intermediates resulted in a total of 154 PNPs constituting eight different classes with diverse combinations and orientations of NP fragments (Fig. 1d). The PNP collection was assessed by cheminformatic methods and was found to be chemically diverse between compound classes. Phenotypic screening and morphological profiling revealed diverse bioactivity profiles within the collection. In particular, unprecedented chemotypes for Hedgehog (Hh) signalling inhibition, DNA synthesis inhibition, de novo pyrimidine biosynthesis inhibition and tubulin modulation were identified.

## Results

### PNP collection synthesis via indole dearomatizations

**Fig. 2:**
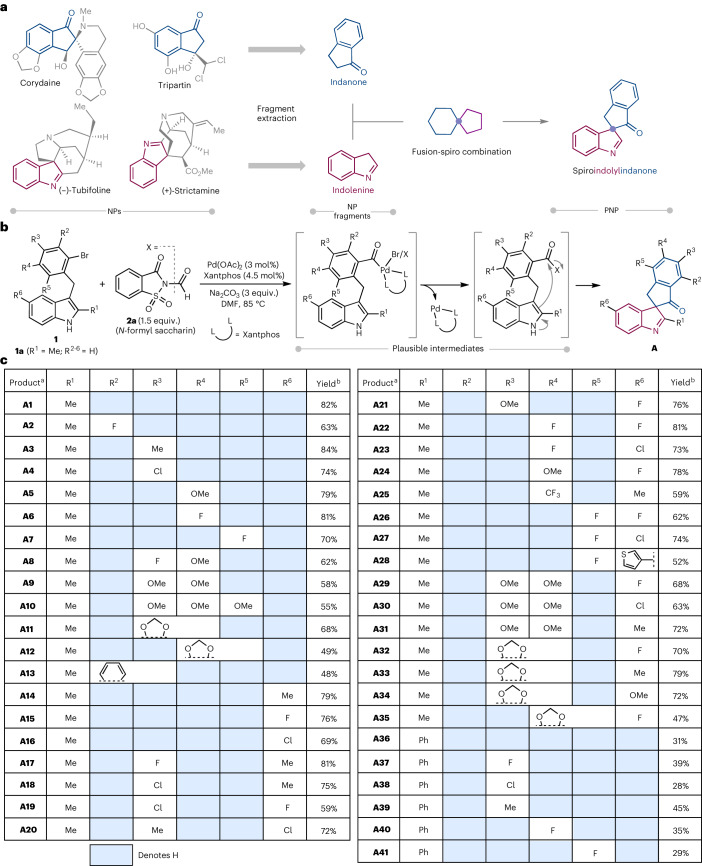
Design, fragment combination and synthesis of spiroindolylindanone PNPs. **a**, Indolenine and indanone fragment combination to construct spiroindolylindanone PNPs. **b**, Method development and optimization of a CO insertion reaction to construct spiroindolylindanone PNPs using *N*-formyl saccharin (**2a**) as a non-toxic bench-stable solid and inexpensive CO surrogate. **c**, Substrate scope for the spiroindolylindanone synthesis. ^a^The PNPs in this manuscript are identified by the letter of the compound class followed by the compound number within the compound class, that is, **A1** is class **A**, compound 1. ^b^Isolated yields are shown.

The initial design was the construction of a scaffold containing indolenine and indanone fragments (Fig. 2a). Indolenine and indanone fragments are commonly found in various bioactive NPs; however, known biosynthetic pathways do not produce NP scaffolds that have both indolenine and indanone fragments (Supplementary Fig. 1). We envisioned that the combination of these fragments would be possible by dearomatizing planar indole starting materials with a C3-tethered electrophile via a palladium-catalysed intramolecular carbonylation/indole dearomatization cascade. The dearomatative nature of the reaction and resulting spirocyclic fusion pattern should result in compounds with higher degrees of chirality and three-dimensionality^41,42^.

Initial attempts started with **1a** (R^1^ = Me, R^2–6^ = H) as a model substrate (Fig. 2b) and CO gas as a carbonyl source with the goal of obtaining dearomatized compound **A1** (PNPs are identified by the letter of the compound class followed by the compound number within the compound class (**A1** is class **A**, compound **1**)) (refs. ^43–47^). Treatment of **1a** with palladium acetate as a catalyst and 1,1′-bis(diphenylphosphino)ferrocene as a ligand in the presence of triethylamine in benzene at 1 atm. CO gas at 80 °C delivered the desired product **A1** in 23% yield. Optimization of different reaction parameters such as solvent, ligand and base in the presence of CO gas did not lead to an improved yield (Supplementary Tables 1–3). We hypothesized that superstoichiometric amounts of CO gas in the medium may be hindering the reaction and focused on CO surrogates that can generate CO gas in a controlled manner in situ^48–50^. Gratifyingly, the use of *N*-formyl saccharin (**2a**) in the presence of palladium acetate, Xantphos (4,5-bis(diphenylphosphino)-9,9-dimethylxanthene) and sodium carbonate in *N,N*-dimethyl formamide (DMF) provided **A1** in an excellent yield of 86%. *N*-formyl saccharin (**2a**) was developed by Manabe et al. and is an inexpensive, safe, efficient and environmentally friendly CO surrogate^49,50^. Employing other CO surrogates in place of **2a**, such as 1,1′-carbonyldiimidazole, 2,4,6-trichlorophenyl formate, molybdenum hexacarbonyl and dicobaltoctacarbonyl, provided little or no desired product (Supplementary Table 4).

The scope of the developed dearomatization reaction was explored and led to the construction of PNP class **A** termed spiroindolylindanones (Fig. 2c). Substrates having either electron-withdrawing or electron-rich groups afforded the desired products in moderate to high yields (**A2**, **A4**, **A6**, **A7**, **A22** and **A25**–**A27** and **A3**, **A5**, **A20** and **A21**, respectively). Multi-substituted and heterocycle-based aryl bromides also reacted to afford the dearomatized products in good yields (**A8**, **A10** and **A29**–**A31** and **A11**, **A12** and **A32**–**A35**, respectively). C5-substituted indoles reacted well regardless of the substituents and substitution pattern of the tethered aryl bromide to furnish spiroindolylindanones **A14**–**A35** in good to excellent yields. C2-phenyl-substituted indoles reacted to afforded the dearomatized products **A36**–**A41** in relatively lower yields than their methyl-substituted derivatives, possibly due to steric and/or electronic reasons. Overall, the developed methodology is an unprecedented example of a carbonylation/intramolecular indole dearomatization via a σ-acylpalladium intermediate cascade. The reaction is operationally simple and robust, which allowed for the rapid synthesis of class **A**.

**Fig. 3:**
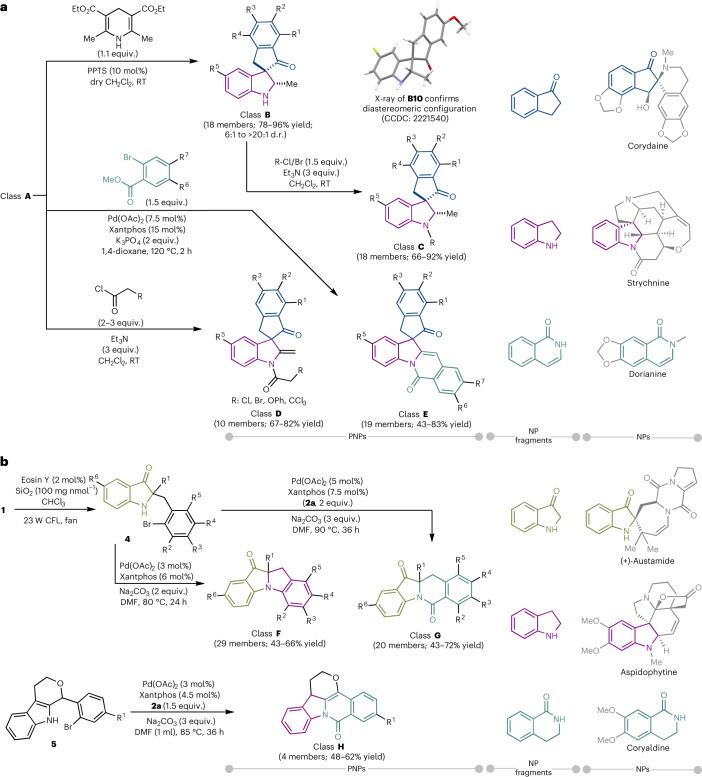
Synthetic routes to classes **B**–**H**. **a**, Access to classes **B**–**H** from class A. The relative configuration of **B10** was determined by X-ray crystallography (Supplementary Information), and the structures of other products (including class **C**) were drawn by analogy. Diastereomeric ratios of class **B** were determined by ^1^H nuclear magnetic resonance. For individual diastereomeric ratios, see Supplementary Information. **b**, Synthesis of classes **F**, **G** and **H** from **1** and **5**, respectively, utilizing the NH of indole derivatives. Isolated yields are shown. Parent NP examples and the corresponding NP fragments from which the PNPs are derived are shown on the right side of the figures. RT, room temperature; CFL, compact fluorescent lamp; fan, 3 W cooling fan.

Derivatization of compounds belonging to class **A** was possible through various reaction pathways and sequences (Fig. 3a). Reduction of the indolenine moiety was performed with Hantzsch ester and a catalytic amount of pyridinium *p*-toluenesulfonate (PPTS) to generate the spiro-indoline–indanone class **B** with good to excellent diastereoselectivities (6:1 ≥ 20:1 diastereomeric ratio (d.r.))^51^. Single-crystal analysis of the major diastereomer of compound **B10** revealed a *cis*-relationship between the methyl and carbonyl groups.

Functional group installation at the free amine group of class **B** generated a new class of compounds (class **C**). Treatment of 2-methyl indolenines with α-halo-acetyl chloride derivatives furnished exocyclic-olefinic α-halo-amides (class **D**). The isoquinolinone fragment, a common motif in a variety of biologically important alkaloids, was fused to the spiroindolylindanone scaffold (class **A**) by employing methyl 2-bromobenzoate in the presence of a palladium/Xantphos catalyst and K_3_PO_4_ as the base^51^. The final indoline–indanone–isoquinolinone scaffold defines a PNP class with both spiro- and edge-fusion combinations (class **E**).

To establish further scaffold diversity, we envisioned rearranging **1** and subjecting it to different variations of the developed Pd-catalysed reaction conditions. Inspired by previous reports on photocatalytic oxidative semipinacolic rearrangement^52,53^, we developed a seminal reaction by employing 2 mol% Eosin Y as a photocatalyst and SiO_2_ as solid additive to induce the rearrangement of **1** to indolin-3-one derivatives **4**. By employing the developed Pd-catalysed conditions, indolin-3-one derivatives (**4**) underwent direct C–N cross couplings in the absence of the CO surrogate **2a** to yield 3-oxindole–indoline-fused PNPs (class **F**, Fig. 3b). In the presence of **2a**, a carbonylation/cyclization cascade resulted in a fusion-edge combination of 3-oxindole and dihydroisoquinolone fragments (class **G**, Fig. 3b). Finally, compounds **5**, stemming from oxa-Pictet-Spengler reactions of tryptophol and benzaldehyde derivatives (Supplementary Fig. 2), were treated with *N*-formyl saccharin for CO insertion. In this case, dearomatization of the indole and formation of the isoquinolinone fragment yielded indoline–isoquinolinone–tetrahydropyran PNPs (class **H**, Fig. 3b).

In total, 154 PNPs representing eight subclasses with different NP fragment combinations and fusion patterns were synthesized from divergent intermediates **1** or compounds **5**. Searches in the Dictionary of Natural Products revealed that neither the NP fragment combinations nor the scaffolds of the subclasses are observed in known NPs (Supplementary Fig. 1). By employing the combination of diverse PNP design with developing and implementing synthetic chemical methodologies, we were able to readily access a diverse range of PNPs that are not possible through existing biosynthetic pathways.

### Cheminformatic analysis of the PNP classes

**Fig. 4:**
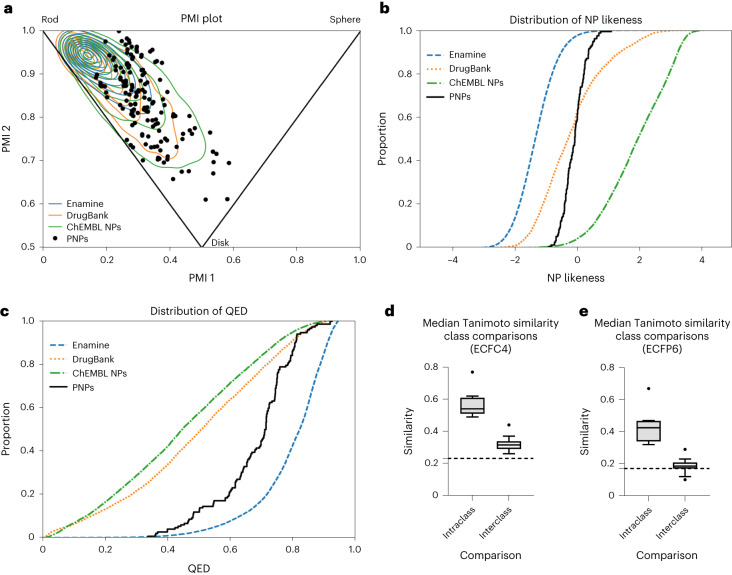
Cheminformatic analyses of the diverse PNP collection. **a**, A PMI plot for the shape of the PNPs (black circles). The corners of the triangle within the plot indicate a rod-like shape (top left), disk-like shape (bottom middle) and sphere-like shape (top right). The contour lines represent a Gaussian kernel density estimation with ten steps. For a PMI plot with individual PNP subclasses, see Supplementary Fig. 3. **b**, NP-likeness scores of the PNPs (black curve) compared with the DrugBank compound collection (orange curve), ChEMBL NPs (green curve) and Enamine building blocks (blue curve). **c**, QED of the PNPs (black curve) compared with the DrugBank compound collection (orange curve), ChEMBL NPs (green curve) and Enamine building blocks (blue curve). **d**, Box plot of intra- and interclass Tanimoto similarity calculations of Morgan fingerprints (ECFC4) following Tukey’s definitions with outliers^83^. Centre line, median; box limits, upper and lower quartiles; whiskers, 1.5× interquartile range; and points, outliers. The dashed line indicates the 95th percentile median (0.23) of random reference compound subsets. For full cross-similarity values, see Supplementary Figs. 9–10. **e**, Box plot of intra- and interclass Tanimoto similarity calculations of Morgan fingerprints (ECFP6) following Tukey’s definitions with outliers^83^. Centre line, median; box limits, upper and lower quartiles; whiskers, 1.5× interquartile range; and points, outliers. The dashed line indicates the 95th percentile median (0.17) of random reference compound subsets. For full cross-similarity values, see Supplementary Figs. 11 and 12. The number of compounds in reference sets is 527,411 (50,000 random compounds were selected for PMI analysis) for Enamine, 4,866 for DrugBank and 45,679 for ChEMBL NPs.

For the characterization of structural and physiochemical properties of the PNP classes, characteristic properties were computed using the open-source software RDKit^54^. The PNPs were compared with reference sets including NPs in the ChEMBL29 database (representing bioactive NPs, 45,679 compounds)^55^, DrugBank compounds (representing marketed and investigational drugs, 4,866 compounds)^56^ and the Enamine Advanced Screening Collection (representing a synthetic drug-like screening library, 527,411 compounds)^57^. To analyse the molecular shapes of the PNP collection and reference sets, principal moments of inertia (PMI) were calculated^58^. The PMI calculation revealed that the PNP collection has shape diversity and overlaps with concentrated areas of bioactive compounds (Fig. 4a). Atom connectivities were evaluated by a NP-likeness score^59^ calculation and were found to notably overlap with DrugBank compounds (Fig. 4b). A lack of considerable overlap with ChEMBL NPs may be due to the PNPs representing fragment combinations and arrangements that are not found in nature. A quantitative estimation of drug-likeness^60^ (QED) analysis suggested that the PNP collection has favourable drug-like properties, which are similar to the properties calculated for the Enamine Advanced Screening Collection (Fig. 4c). A principal component analysis^61^ of 17 molecular descriptors shows that the PNP collection occupies chemical space that is shared by high-density regions of all three reference sets (Supplementary Figs. 4–6). Interestingly, 77% of the PNPs have lead-like sizes, that is, 14 ≤ heavy atoms ≤ 26 (refs. ^62,63^) and, in combination with drug-like properties suggested by QED analysis, indicates that the PNP collection may be lead like. Future compound classes could be designed by combining NP fragments with appropriate molecular properties to afford other lead-like PNP collections.

Intra- and interclass Tanimoto similarities of the Morgan fingerprints of two different designs (ECFC4 and ECFP6) were calculated to determine structural diversity (Fig. 4d,e, and for full comparisons, see Supplementary Figs. 9–12). Within compound classes, the median similarities were high (ECFC4 of 0.49–0.77 and ECFP6 of 0.32–0.67), whereas between compound classes the median similarities are distinctly lower (ECFC4 of 0.26–0.44 and ECFP6 of 0.10–0.29). For comparison, a threshold of randomness was determined by calculating the 95th percentile median similarities from 100 subsets with 100 random members from the Enamine Advanced Screening Collection (ECFC4 of 0.23 and ECFP6 of 0.17; for more details, see Supplementary Information). Several interclass PNP similarities are near but do not fall below the randomness thresholds. This may be expected as similar NP fragments are conserved throughout the compound collection, albeit in different arrangements. It can therefore be concluded that the majority of interclass structures of the PNP collection are diverse.

### Identification of an inhibitor of Hh signalling

The bioactivity of the PNP collection was investigated by employing cell-based assays monitoring different cellular pathways and signalling cascades including autophagy, kynurenine production and Hh-dependent osteoblast differentiation. Gratifyingly, spiroindolylindanone class **A** was enriched with inhibitors of Hh-dependent osteoblast differentiation. No compounds from the PNP collection were identified as hits, that is, >50% inhibition at 10 µM, in assays monitoring autophagy or kynurenine production.

The Hh signalling pathway plays an important role during vertebrate embryonic and post-embryonic development including tissue homeostasis and regeneration^64^. Hh signalling is activated through the binding of Hh ligands to the membrane receptor Patched1, which in turn relieves Patched1-mediated inhibition of the membrane protein Smoothened (SMO). This activation ultimately leads to the transcription of Hh target genes, such as *Gli1* and *Ptch1*, by the transcription factors glioma-associated oncogene homologues 2 and 3 (GLI2 and GLI3) (ref. ^65^). Unregulated Hh signalling has been connected to cancers such as basal cell carcinoma and medulloblastoma. Therefore, novel therapeutics for inhibiting the Hh pathway are in high demand^66^.

**Fig. 5:**
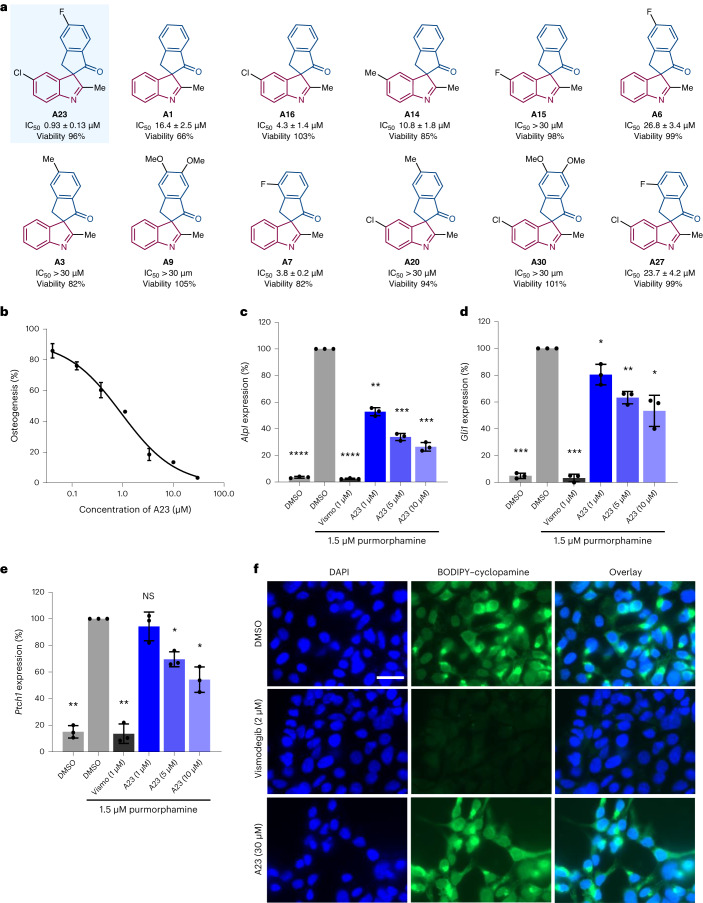
Biological investigation of PNP A23 for Hh signalling inhibition. **a**, Selected compounds with structural variation to the most active compound (**A23**) in an osteoblast differentiation assay. Cell viability was assessed using a CellTiter-Glo Luminescent Cell Viability Assay and treating C3H10T1/2 cells with compound (30 µM) in the absence of purmorphamine for 96 h. The viability of cells treated with DMSO was set to 100%. IC_50_, half-maximal inhibitory concentration. **b**, C3H10T1/2 cells were treated for 96 h with 1.5 µM purmorphamine, DMSO as a control or compound **A23**. The activity of alkaline phosphatase was measured to determine Hh pathway activity. Values for cells treated with purmorphamine and DMSO were set to 100%. The data are the mean values ± s.d. of three biological replicates (*n* = 3). **c**, *Alpl g*ene expression; C3H10T1/2 cells were incubated for 96 h with 1.5 µM purmorphamine and DMSO, 1 µM vismodegib (vismo) or 1, 5 or 10 µM of **A23** before RT–qPCR. Data are mean values ± s.d. of three biological replicates (*n* = 3). The *P* values relative to cells treated with DMSO and purmorphamine are <0.0001 for DMSO-treated, <0.0001 for vismo (1 µM)-treated, 0.0014 for **A23** (1 µM)-treated, 0.0005 for **A23** (5 µM)-treated and 0.0006 for **A23** (10 µM)-treated cells. **d**,**e**, Expression of the Hh target gene *Gli1* (**d**) and *Ptch1* (**e**). C3H10T1/2 cells were incubated with 1.5 µM of purmorphamine and DMSO, 1 µM vismo or **A23** (1 µM, 5 µM or 10 µM) for 96 h before RT–qPCR. Data are mean values ± s.d. of three biological replicates (*n* = 3). For Hh target gene *Gli1*, the *P* values relative to cells treated with DMSO and purmorphamine are 0.0001 for DMSO-treated, 0.0003 for vismo (1 µM)-treated, 0.0477 for **A23** (1 µM)-treated, 0.0052 for **A23** (5 µM)-treated and 0.0203 for **A23** (10 µM)-treated cells. For Hh target gene *Ptch1*, the *P* values relative to cells treated with DMSO and purmorphamine are 0.0010 for DMSO-treated, 0.0024 for vismo (1 µM)-treated, 0.4559 for **A23** (1 µM)-treated, 0.0111 for **A23** (5 µM)-treated and 0.0142 for **A23** (10 µM)-treated cells. **f**, SMO binding assay. HEK293T cells were transfected with a SMO-expressing plasmid. After 48 h, the cells were fixed and incubated with BODIPY–cyclopamine (green, 5 nM) and treated with either DMSO, vismo or **A23** for 4 h. The nuclei were visualized by staining the cells with 4,6-diamidino-2-phenylindole (DAPI) (blue). The images are representative of three biological replicates (*n* = 3). Scale bar, 30 µm. For **c**–**e**, statistical analyses were performed relative to DMSO/purmorphamine by employing unpaired two-tailed *t*-tests with Welch’s correction (*P < 0.05, **P < 0.01, ***P < 0.001, ****P < 0.0001; NS, not significant).

To identify novel inhibitors of Hh signalling, we employed an osteoblast differentiation assay using C3H/10T1/2 mesenchymal progenitor cells upon stimulation with purmophamine^67^. Stimulation of the Hh pathway induces osteoblast differentiation, which can be detected through monitoring of alkaline phosphatase expression and activity. The screen revealed compound **A23** is an inhibitor of Hh-dependent cell differentiation with a half-maximal inhibitory concentration of 0.93 ± 0.13 μM and no effect on cell viability (Fig. 5a).

Variation of the substitution pattern on **A23** to establish a structure–activity correlation did not uncover more potent compounds (Fig. 5a). Removal of chlorine and fluorine substituents led to a loss in potency (**A1**) and affected cell viability. Reintroduction of a chlorine (**A16**) to the indolenine moiety at R^6^ led to a more potent activity relative to **A1** without affecting cell viability, whereas a methyl (**A14**) or a fluorine (**A15**) at the same position led to similar and weaker activities relative to **A1**, respectively. Addition of a fluorine to the indanone moiety at R^4^ (**A6**) led to a loss in potency relative to **A1** as did the introduction of a methyl (**A3**) or dimethoxy substituents (**A9**). Moving the fluorine substituent to R^5^ (**A7**) led to better activity. When compounds with a chlorine at R^6^ and similar substitution patterns on the indanone moiety as above were evaluated, only the activity of **A23** increased, whereas **A20**, **A30** and **A27** became less active. Compounds **A6**, **A16**, **A23** and two other derivatives (**A8** and **A21**) were resynthesized and re-evaluation in an osteoblast differentiation assay resulted in similar activities to the original batches (Supplementary Fig. 13). Compound **A23** was the most active inhibitor (Fig. 5b) and was also found to suppress alkaline phosphatase gene expression (*Alpl*) in a dose-dependent manner (Fig. 5c), which is in line with inhibition of osteoblast differentiation.

Evaluation of Hh genes by quantitative reverse transcription PCR (RT–qPCR)^68^ showed that **A23** reduced expression of the Hh target genes *Ptch1* and *Gli1* (reduction of 46% and 47% at 10 µM, respectively, Fig. 5d,e). As SMO is frequently targeted by small molecules, the binding of **A23** to SMO was explored in a competition assay using fluorinated boron-dipyrromethene (BODIPY)–cyclopamine^69^. Cyclopamine is a NP that binds to the heptahelical bundle in SMO^70^ that is targeted by the most SMO modulators. Compound **A23** did not compete with BODIPY–cyclopamine and, therefore, most likely does not bind to SMO (Fig. 5f). All together, these results indicate that **A23** is inhibiting Hh signalling independent of direct SMO modulation. It should be noted that current therapies affecting Hh signalling target SMO, which can cause mutations leading to drug resistance^71^. Therefore, novel inhibitors of the Hh pathway that do not target SMO are of particular interest.

### PNP biological evaluation via the CPA

Beyond monitoring specific biological processes or signalling pathways, the PNP library was evaluated by morphological profiling via the cell painting assay (CPA)^72,73^. This unbiased morphological profiling method monitors phenotypic changes in cells upon compound treatment by employing six fluorescent dyes that selectively stain various compartments. High-content imaging via multi-channel fluorescence microscopy and automated image analysis extracts and quantifies the changes of cellular morphologies into 579 cellular features to generate a characteristic profile.

An induction value, representing the percentage of significantly changed features relative to dimethylsulfoxide (DMSO) controls, is a measure for compound activity in the CPA^74^. Investigation of the PNP collection at concentrations ranging from 1 to 50 µM revealed that 78% of the compounds are characterized by an induction value ≥5% and are considered bioactive (Supplementary Fig. 14). Furthermore, all compound classes contained CPA-active compounds. For comparison, 42% of compounds with annotated bioactivities (2,230 of 5,307) and 45% of our in-house, non-annotated compounds (6,581 of 14,784) have induction values ≥5% at concentrations up to 50 µM.

The CPA can enable target or mode-of-action (MoA) identification by directly comparing full CPA profiles of uncharacterized compounds with reference compounds with annotated bioactivities^75^. We have recently introduced the concept of a CPA subprofile analysis to facilitate rapid target or MoA prediction^76^. In short, subprofiles are defined by reducing the number of profile features to only those that are shared by annotated compounds in a particular bioactivity cluster. High similarities (>85%) to bioactivity cluster profiles can facilitate the annotation of uncharacterized compounds without prior knowledge of the top biosimilar reference compounds. So far, 12 bioactivity cluster subprofiles have been defined that include biological targets related to AKT/PI3K/MTOR, Aurora kinases, bromodomain and extra-terminal (BET) domain, de novo pyrimidine biosynthesis, DNA synthesis, histone deacetylase (HDAC), HSP90, Na^+^/K^+^ ATPases and tubulin, or processes such as lysosomotropism/cholesterol homeostasis regulation, protein synthesis and uncoupling of the mitochondrial proton gradient.

**Fig. 6:**
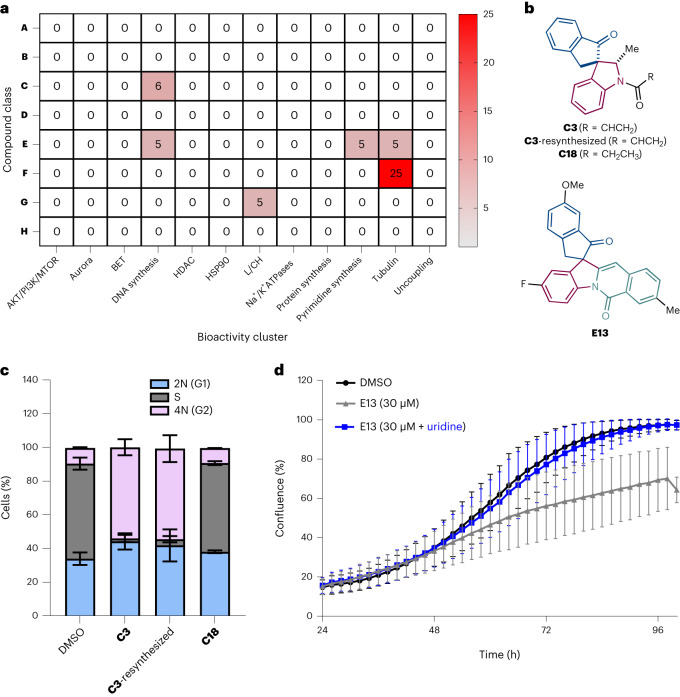
CPA subprofile analysis of the dPNP collection and bioactivity validation of C3 and E13. **a**, A heat map showing the percentage of PNPs in each class that have >85% similarity to a bioactivity cluster profile and induction values >15%. The PNPs were measured at various concentrations (≤50 µM), generating several profiles for each PNP. For each PNP, the profile with the highest similarity to a bioactivity cluster was selected for this analysis. L/CH, lysosomotropism/cholesterol homeostasis. For heat maps of bioactivity cluster similarities of individual profiles of hit compounds, see Supplementary Figs. 15 and 16. **b**, The chemical structure of **C3,** **C3**-resynthesized, **C18** and **E13**. **c**, The influence of **C3,** **C3**-resynthesized and **C18** on DNA content and the cell cycle. U2OS cells were treated with DMSO or compound (30 µM) for 22 h followed by the addition of 10 µM EdU and incubated for an additional 2 h. DNA-incorporated EdU was labelled with Alexa Fluor 488 via click reaction and DNA was stained with propidium iodide. Single-cell analysis via flow cytometry measuring EdU incorporation and total DNA content was used to determine the percentage of cells in either G1 (2N), S (2N–4N) or G2 (4N) phase. Data are mean values ± s.d. (*n* = 10,000 cells examined over three biologically independent samples). Histograms of the FACS analysis can be found in Supplementary Fig. 19. **d**, Uridine rescue assay. HCT116 cells were treated with either DMSO (control), **E13** or **E13** in the presence of uridine (100 µM). Cell confluence was used as a measure of cell proliferation and was monitored over a 96 h period using an IncuCyte ZOOM/S3. Data are mean values ± s.d. of four independent replicates (*n* = 4).

All CPA PNPs profiles with induction values >15% and relative cell counts >50% were subjected to a subprofile analysis. Overall, several PNPs from different compound classes induced subprofiles that were similar to reference subprofiles including inhibition of DNA synthesis, inhibition of de novo pyrimidine biosynthesis and modulation of tubulin (Fig. 6a and Supplementary Figs. 15 and 16).

Compound **C3** (Fig. 6b) was identified by the CPA subprofile analysis as a potential inhibitor of DNA synthesis and was the only compound in class **C** that induced a profile with >85% similarity to the DNA synthesis cluster profile with all other compounds in the class, having <60% similarity (Supplementary Fig. 17). A brief structure–phenotype relationship^74^ study of **C3** was conducted with respect to the DNA synthesis cluster. The indolenine and N-unsubstituted indoline synthetic precursors to **C3** (**A1** and **B1**, respectively) both have weak activity in the CPA and low similarity to the DNA synthesis profile (Supplementary Fig. 18). Introduction of an acrylamide moiety to **B1** affords **C3**, which has a high similarity to the DNA synthesis profile (92% similarity). Other N-functionalized derivatives of **B1** including α-halo amide compounds (**C1** and **C2**), an ethyl carbamate compound (**C4**), an *N*-allylated compound (**C5**) and an *N*-tosylated compound all have very low similarities to the DNA synthesis profile. These results suggest that the acrylamide moiety of **C3** is crucial for producing a highly similar phenotype to the DNA synthesis profile.

The influence of **C3** on DNA synthesis and the cell cycle was investigated by fluorescence-activated cell sorting (FACS) flow cytometry employing 5-ethynyl-2’-deoxyuridine (EdU) and propidium iodide. EdU is an alkynyl thymidine analog that can be incorporated into newly synthesized DNA, and the attachment of a fluorophore via click chemistry and subsequent microscopy can be used to quantify DNA synthesis^77^. Additional staining with propidium iodide, which quantifies overall DNA content, and single-cell analysis via FACS flow cytometry can determine the influence of compounds on the cell cycle. At 30 µM, compound **C3** led to a remarkable decrease in DNA-incorporated EdU (Supplementary Figs. 19 and 20) and a depletion of cells in the S phase relative to DMSO controls (Fig. 6c). These results indicate that **C3** prevents DNA synthesis and confirms the CPA-generated MoA hypothesis. Compound **C3** was resynthesized (**C3**-resynthesized) and reverified to be an inhibitor of DNA synthesis (Fig. 6c and Supplementary Figs. 19 and 20). Since **C3** has an acrylamide moiety that may act as a Michael acceptor, a saturated ethyl amide derivative (**C18**, Fig. 6b) was synthesized and evaluated. Interestingly, **C18** provided similar results to a DMSO control via FACS flow cytometry (Fig. 6c and Supplementary Figs. 19 and 20) and confirms that the acrylamide moiety of **C3** is necessary for its activity as a DNA synthesis inhibitor.

The CPA subprofile analysis (Fig. 6a) also suggested that **E13** (Fig. 6b) may affect a target related to de novo pyrimidine biosynthesis (85% biosimilarity to the de novo pyrimidine biosynthesis profile and 28% induction at 50 µM) (ref. ^78^). Pyrimidine biosynthesis is one facet of the biosynthesis of nucleic acids and nucleotide cofactors that are required for DNA and RNA synthesis as well as several other essential cellular processes^79^. The dysregulation of nucleotide metabolism is prevalent in various types of cancers as well as viral infections, and small molecules that target nucleotide biosynthetic pathways have been successful therapeutics to combat these diseases^79^.

To examine whether **E13** affects de novo pyrimidine biosynthesis, a uridine rescue assay was employed (Fig. 6d). If pyrimidine biosynthesis is inhibited, pyrimidine nucleotides will be depleted, leading to the suppression of cellular growth due to the lack of DNA synthesis and transcription. The supplementation of excess uridine can restore normal cell growth by bypassing the need for de novo pyrimidine biosynthesis. When treated with **E13** at 30 µM, the growth of HCT116 cells was impaired over a 96 h time frame (Fig. 6d). Co-treatment of cells with **E13** and supplemental uridine (100 µM) completely restored cell growth relative to the DMSO control, confirming the CPA-guided hypothesis that **E13** is inhibiting de novo pyrimidine biosynthesis. Compound **E13** was resynthesized and its bioactivity as an inhibitor of de novo pyrimidine biosynthesis was reconfirmed (Supplementary Fig. 21a).

Besides **E13**, no other compounds in class **E** had similarities to the de novo pyrimidine biosynthesis bioactivity profile >85% and induction values >20%, suggesting that they either weakly inhibit or do not inhibit de novo pyrimidine biosynthesis (Supplementary Fig. 22). Compounds **E18** and **E4**, which had moderate similarities to the de novo pyrimidine biosynthesis bioactivity profile (84% and 79% similarity at 50 µM, respectively) and weak induction values (10% and 15% at 50 µM, respectively), were resynthesized and subjected to the uridine rescue assay. At 50 µM concentrations, **E18** had weak activity, whereas **E4** inhibited cell proliferation but could not be rescued by supplemental uridine and therefore is probably not affecting de novo pyrimidine biosynthesis (Supplementary Fig. 21b,c).

**Fig. 7:**
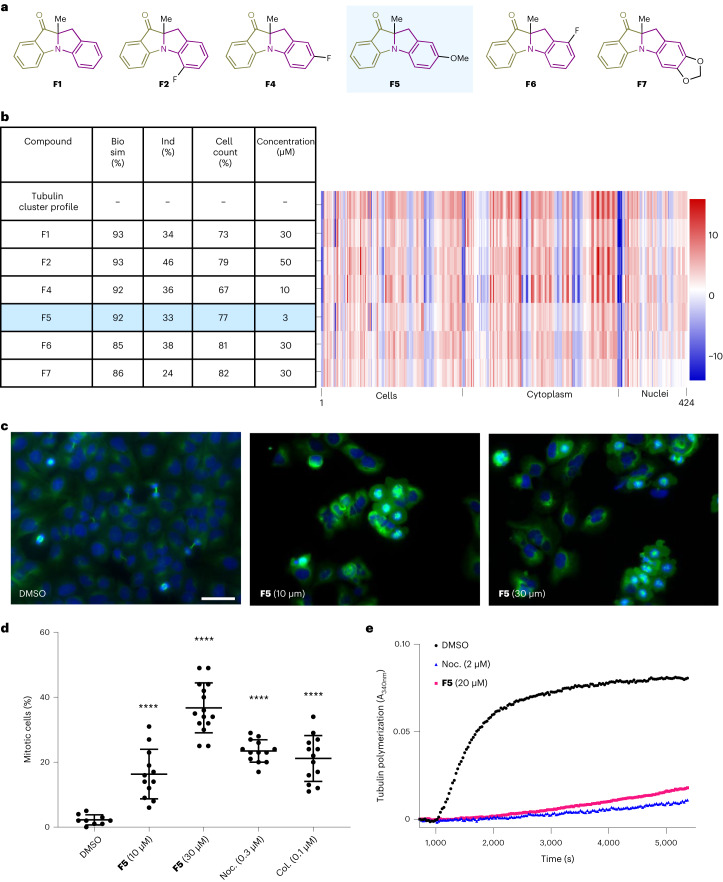
Validation of F5 as a tubulin-targeting compound. **a**, Structures of the six class **F** compounds that have >85% similarity to the tubulin cluster profile. **b**, Tubulin cluster profile relative to class **F** compounds that have >85% similarity to the tubulin cluster profile. The selected profiles are those that have >85% similarity to the tubulin cluster profile and >20% induction at the lowest concentrations per compound. The reference profile is the first profile (100% biosimilarity) for which all subsequent profiles are compared. The tubulin profile has 424 features and is divided into three segments: cell, cytoplasm and nuclei. Biosim, biosimilarity to the tubulin cluster profile; ind, induction (percentage of significantly changed features relative to DMSO controls); and conc, concentration. The induction value reported is relative to the full CPA profiles with 579 features. **c**, Influence on the microtubule network. U2OS cells were treated for 24 h with DMSO (control) or **F5** before staining for tubulin (green) and DNA (blue). Scale bar, 50 µm. **d**, Quantification of mitotic cells via immunocytochemistry. U2OS cells were treated with DMSO (negative control), **F5**, nocodazole (noc, positive control) or colchicine (col, positive control) for 24 h before staining of cells for phospho-histone H3 and DNA. Cells in mitosis were quantified as the percentage of phospho-histone H3-positive cells. Data are mean values ± s.d. of three independent replicates (*n* = 3). Statistical analyses were performed relative to the DMSO control by employing unpaired two-tailed *t*-tests (****P < 0.0001). The *P* values relative to cells treated with DMSO are <0.0001 for **F5** (10 µM)-treated, <0.0001 for **F5** (30 µM)-treated, <0.0001 for noc-treated and <0.0001 for col-treated cells. **e**, In vitro tubulin polymerization assay. The polymerization was initiated upon addition of guanosine triphosphate (GTP) to porcine tubulin and quantified by means of turbidity measurement at 340 nm and 37 °C. DMSO was used as a negative control and noc was used as a positive control for tubulin destabilization. Data are representative of three independent experiments (*n* = 3).

From the CPA subprofile analysis, class **F** was found to be enriched in profiles that had >85% similarity to the tubulin-targeting cluster (25% of all compounds in class **F**). Closer examination of these profiles resulted in the identification of six possible tubulin-targeting compounds that characteristically reduce cell count (Fig. 7a,b). A structure–phenotype relationship study^74^ of class **F** was conducted by comparing similarity with the tubulin cluster profile and induction relative to concentration (Supplementary Fig. 23). Unsubstituted compound **F1** gave a moderate induction value (30%) with a high similarity to the tubulin cluster profile at 30 µM (93% similarity). Installation of a fluoride to the eastern hemisphere either gave similar (**F2** and **F6**) or more potent activity (**F4**), whereas the introduction of a methyl (**F3**) or a fused-phenyl (**F8**) group resulted in lower induction values and low similarities to the tubulin cluster profile relative to **F1**. Methoxy-substituted compound **F5** gave a high tubulin cluster profile similarity at a relatively low concentration of 3 µM. A similar dioxole derivative (**F7**) retained high tubulin cluster similarity but only at a concentration of 30 µM or higher. Incorporation of a methyl (**F15** versus **F6**) or chloride (**F14** versus **F4**) to the western hemisphere of the scaffold or an *α*-phenyl ketone (**F21** versus **F4**) led to relatively lower inductions and low tubulin cluster profile similarities. A closely related 2*H*-isoquinolinone derivative (**G4**) was also less active than its indoline counterpart (**F5**).

Compound **F5** was selected for further investigation since it has a good induction value at a relatively low concentration (3 µM) while retaining a high similarity to the tubulin subcluster, indicating that it may be the most potent compound. To further corroborate the tubulin-targeting prediction, the full profile of **F5** at 3 µM was compared with the full profiles of reference compounds. Gratifyingly, 11 of the first 12 most similar reference compounds are in the tubulin-targeting bioactivity cluster (Supplementary Fig. 24).

To investigate the predicted bioactivity, U2OS cells were treated with **F5** for 24 h followed by immunostaining for tubulin and DNA. Upon treatment of **F5**, there was an accumulation of round cells with condensed microtubules and multipolar spindles (Fig. 7c), which are consistent with a tubulin-targeting phenotype^80^. Notably, upon treatment of **F5** (10 µM), the abundance of miotic cells increased by ninefold relative to the DMSO control as quantified by means of phospho-histone H3-positive cells (Fig. 7d). A concentration-dependent response was observed with a nearly two-fold increase in mitotic cells upon treatment with 10 μM to 30 μM of **F5**. Additionally, compound **F5** suppressed in vitro tubulin polymerization at 20 μM and parallels the activity of the known tubulin polymerization inhibitor nocodazole (Fig. 7e). These results confirm that the PNP **F5** is a microtubule modulator and validates the target prediction of the CPA subprofile analysis. Compound **F5** was resynthesized and functionalized to afford four additional derivatives (Supplementary Fig. 25a). The bioactivity of **F5** was reconfirmed in a tubulin polymerization assay, and the derivatives were either inactive or moderately active relative to the parent compound (Supplementary Fig. 25b). Furthermore, **F5** was more potent than **F4**, and **F4** was more potent than **F15** (Supplementary Fig. 25b), as was suggested by the structure–phenotype analysis (Supplementary Fig. 23).

## Conclusions

In molecular discovery programmes, several design strategies have been applied with the goal of efficiently exploring biologically relevant chemical space. DOS focuses on the rapid synthesis of compound collections that have high chemical diversity with the intention of probing a wide range of biological space; however, the compounds generated may not necessarily be biologically relevant. The PNP principle provides structurally focused collections by combining NP fragments in arrangements not found in existing NP structures to afford NP-like scaffolds that are endowed with the inherent biological relevance of NPs.

We have proposed the diverse PNP concept that combines the chemical diversity of DOS collections with the inherent biological relevance of the PNP concept to afford compound collections that may be enriched with both structural and biological diversity. The diverse PNP concept has been demonstrated by employing a divergent intermediate strategy along with the development and implementation of synthetic methodologies for the rapid access to eight chemically diverse PNP classes. Of note, dearomatization methodologies were employed to convert aromatic ring systems into more three-dimensional moieties with new stereogenic centre(s) and increased fraction of *sp*^3^ carbons (Supplementary Fig. 26). The biological diversity of the collection was exemplified by phenotypic screening and morphological profiling in which four compounds from different classes were identified to have unique bioactivities as either an inhibitor of Hh signalling (**A23**), tubulin polymerization (**F5**), DNA synthesis (**C3**) or de novo pyrimidine biosynthesis (**E13**).

The diverse PNP concept may help guide the future design of NP-like compound collections that embody both chemical diversity and biological relevance. Incorporation of both of these features may lead to high-quality compound collections that more effectively probe diverse biologically relevant chemical space and, in conjunction with suitable screening technologies, lead to the identification of unexpected or novel bioactivities as demonstrated here. Recently, virtual screenings of ultra-large make on-demand compound collections have been used to expand the exploration of biologically relevant chemical space^81,82^. While this approach can rapidly evaluate billions of compounds and lead to the discovery of new chemotypes, it is limited to known targets and binding sites, whereas assessing compounds, such as the collection described here, in an unbiased manner may lead to the identification of novel targets, binding pockets or molecular glues. Further biological screening campaigns of these and other PNPs may provide context for the biological relevance of PNPs relative to NPs and collections of different designs as well as the importance of chemical diversity within PNP design.

## Methods

### Procedure for CO insertion/indole dearomatization cascade

An oven-dried screw-capped reaction tube with a magnetic stir bar was charged with 3-(2-bromobenzyl)-2-methyl-1*H*-indole (0.5 mmol), *N*-formyl saccharin (**2a**, 1.5 equiv., 0.75 mmol), Pd(OAc)_2_ (3 mol%, 0.015 mmol, 3.4 mg), Xantphos (4.5 mol%, 13 mg) and Na_2_CO_3_ (3 equiv., 159 mg). A screw cap fitted with a rubber septum was attached to the reaction tube then degassed and refilled with argon; the process was repeated two additional times. Dry DMF (5 ml) was added to the reaction tube, and the reaction tube was placed in metal block and vigorously stirred at room temperature for 10 min followed by heating to 85 °C. The reaction mixture was cooled to room temperature after 36 h, diluted with 10 ml of ethyl acetate and 25 ml of water. The organic layer was separated and concentrated under vacuum. The crude mixture was purified by column chromatography using silica gel (100–200 mesh size) and petroleum ether/ethyl acetate as the eluent.

### Cheminformatic analyses

All details related to the cheminformatic analyses can be found in the repository at ref. ^84^.

### Biological assays

All details pertaining to the biological assays can be found in Supplementary Information.

Primers used for RT–qPCR:

**Table:** 

Primer	Forward (5′–3′)	Reverse (5′–3′)
*Ptch1*	CTCTGGAGCAGATTTCCAAGG	TGCCGCAGTTCTTTTGAATG
*Gli1*	CACCGTGGGAGTAAACAGGCCTTCC	CCAGAGCGTTACACACCTGCCCTTC
*Alpl*	ATCTTTGGTCTGGCTCCCATG	TTTCCCGTTCACCGTCCAC
*Gapdh*	CAGTGCCAGCCTCGTC	CAATCTCCACTTTGCCACTG
*Ap3dl*	CAGAGGGCTCATCGGTACAC	GCCGGAAGTCCAACTTCTCA

### Reporting summary

Further information on research design is available in the Nature Portfolio Reporting Summary linked to this article.

## Online content

Any methods, additional references, Nature Portfolio reporting summaries, source data, extended data, supplementary information, acknowledgements, peer review information; details of author contributions and competing interests; and statements of data and code availability are available at 10.1038/s41557-024-01458-4.

### Supplementary information

Supplementary Information Supplementary Tables 1–13; Figs. 1–27; cheminformatic, biological experimental and chemistry experimental details; and compound characterization data and spectra.

Reporting Summary

Supplementary Data 1 Crystallographic data for compound **B10**; CCDC reference 2221540.

## Data Availability

The data that support this study are available within the article and Supplementary Information. The reference datasets used on the manuscript (stemming from Enamine Advanced Screening Collection, https://enamine.net/hit-finding/compound-collections/screening-collection/advanced-collection downloaded on 7 December 2020; Drugbank approved and investigational drugs, v.5.1.8; ChEMBL v30) are not included, but the steps to generate them are described in the code repository. All structures covered in this manuscript and their calculated properties are included in the GitHub repository at ref. ^84^. Crystallographic data for the structure reported in the article have been deposited at the Cambridge Crystallographic Data Centre, under deposition number CCDC 2221540 (**B10**). Copies of the data can be obtained free of charge via https://www.ccdc.cam.ac.uk/structures. Further datasets generated and analysed during the current study are available from the corresponding authors upon request.

## Code availability

The code for the standardization of chemical structures, the extraction of NPs from ChEMBL (one of the reference datasets), the calculation of chemical descriptors for the principal component analysis and for the calculation of PMI is available as a GitHub repository at ref. ^84^.

## References

[1] Dobson CM (2004). Chemical space and biology. Nature.

[2] Grygorenko OO, Volochnyuk DM, Ryabukhin SV, Judd DB (2020). The symbiotic relationship between drug discovery and organic chemistry. Chem. Eur. J..

[3] Newman DJ, Cragg GM (2020). Natural products as sources of new drugs over the nearly four decades from 01/1981 to 09/2019. J. Nat. Prod..

[4] Wender PA, Verma VA, Paxton TJ, Pillow TH (2008). Function-oriented synthesis, step economy, and drug design. Acc. Chem. Res..

[5] Micalizio GC, Hale SB (2015). Reaction design, discovery, and development as a foundation to function-oriented synthesis. Acc. Chem. Res..

[6] Crane EA, Gademann K (2016). Capturing biological activity in natural product fragments by chemical synthesis. Angew. Chem. Int. Ed..

[7] Wetzel S, Bon RS, Kumar K, Waldmann H (2011). Biology-oriented synthesis. Angew. Chem. Int. Ed..

[8] Van Hattum H, Waldmann H (2014). Biology-oriented synthesis: harnessing the power of evolution. J. Am. Chem. Soc..

[9] Huffman BJ, Shenvi RA (2019). Natural products in the ‘marketplace’: interfacing synthesis and biology. J. Am. Chem. Soc..

[10] Woo S, Shenvi RA (2021). Natural product synthesis through the lens of informatics. Acc. Chem. Res..

[11] McLeod MC (2014). Probing chemical space with alkaloid-inspired libraries. Nat. Chem..

[12] Pye CR, Bertin MJ, Lokey RS, Gerwick WH, Linington RG (2017). Retrospective analysis of natural products provides insights for future discovery trends. Proc. Natl Acad. Sci. USA.

[13] Grigalunas M, Burhop A, Christoforow A, Waldmann H (2020). Pseudo-natural products and natural product-inspired methods in chemical biology and drug discovery. Curr. Opin. Chem. Biol..

[14] Alkubaisi BO (2023). Complexity-to-diversity and pseudo-natural product strategies as powerful platforms for deciphering next-generation therapeutics. ChemMedChem.

[15] Huigens RW (2013). A ring-distortion strategy to construct stereochemically complex and structurally diverse compounds from natural products. Nat. Chem..

[16] Morrison KC, Hergenrother PJ (2014). Natural products as starting points for the synthesis of complex and diverse compounds. Nat. Prod. Rep..

[17] Motika SE, Hergenrother PJ (2020). Re-engineering natural products to engage new biological targets. Nat. Prod. Rep..

[18] Karageorgis G, Foley DJ, Laraia L, Waldmann H (2020). Principle and design of pseudo-natural products. Nat. Chem..

[19] Karageorgis G, Foley DJ, Laraia L, Brakmann S, Waldmann H (2021). Pseudo natural products—chemical evolution of natural product structure. Angew. Chem. Int. Ed..

[20] Grigalunas M, Brakmann S, Waldmann H (2022). Chemical evolution of natural product structure. J. Am. Chem. Soc..

[21] Akbarzadeh M (2022). The pseudo-natural product rhonin targets RHOGDI. Angew. Chem. Int. Ed..

[22] Grigalunas M (2022). Unprecedented combination of polyketide natural product fragments identifies the new Hedgehog signaling pathway inhibitor grismonone. Chem. Eur. J..

[23] Davies C (2022). Identification of a novel pseudo‐natural product type IV IDO1 inhibitor chemotype. Angew. Chem. Int. Ed..

[24] Yao R (2023). Identification of 5-HT2 serotonin receptor modulators through the synthesis of a diverse, tropane- and quinuclidine-alkaloid-inspired compound library. J. Med. Chem..

[25] Liu J (2021). Design, synthesis, and biological evaluation of chemically and biologically diverse pyrroquinoline pseudo natural products. Angew. Chem. Int. Ed..

[26] Grigalunas M (2021). Natural product fragment combination to performance-diverse pseudo-natural products. Nat. Commun..

[27] Liu J (2021). Combination of pseudo-natural product design and formal natural product ring distortion yields stereochemically and biologically diverse pseudo-sesquiterpenoid alkaloids. Angew. Chem. Int. Ed..

[28] Burke MD, Schreiber SL (2004). A planning strategy for diversity-oriented synthesis. Angew. Chem. Int. Ed..

[29] Cordier C, Morton D, Murrison S, Nelson A, O’Leary-Steele C (2008). Natural products as an inspiration in the diversity-oriented synthesis of bioactive compound libraries. Nat. Prod. Rep..

[30] Schreiber SL (2009). Molecular diversity by design. Nature.

[31] Morton D, Leach S, Cordier C, Warriner S, Nelson A (2009). Synthesis of natural-product-like molecules with over eighty distinct scaffolds. Angew. Chem. Int. Ed..

[32] O’ Connor CJ, Beckmann HSG, Spring DR (2012). Diversity-oriented synthesis: producing chemical tools for dissecting biology. Chem. Soc. Rev..

[33] Clemons PA (2010). Small molecules of different origins have distinct distributions of structural complexity that correlate with protein-binding profiles. Proc. Natl Acad. Sci. USA.

[34] Petrone DA, Yen A, Zeidan N, Lautens M (2015). Dearomative indole bisfunctionalization via a diastereoselective palladium-catalyzed arylcyanation. Org. Lett..

[35] Zeidan N, Beisel T, Ross R, Lautens M (2018). Palladium-catalyzed arylation/heteroarylation of indoles: access to 2,3-functionalized indolines. Org. Lett..

[36] Zheng C, You SL (2019). Catalytic asymmetric dearomatization (CADA) reaction-enabled total synthesis of indole-based natural products. Nat. Prod. Rep..

[37] Abou-Hamdan H, Kouklovsky C, Vincent G (2020). Dearomatization reactions of indoles to access 3D indoline structures. Synlett.

[38] Liu Y-Z, Song H, Zheng C, You S-L (2022). Cascade asymmetric dearomative cyclization reactions via transition-metal-catalysis. Nat. Synth..

[39] Bauer RA, Wurst JM, Tan DS (2010). Expanding the range of ‘druggable’ targets with natural product-based libraries: an academic perspective. Curr. Opin. Chem. Biol..

[40] Lovering F, Bikker J, Humblet C (2009). Escape from flatland: Increasing saturation as an approach to improving clinical success. J. Med. Chem..

[41] Müller G, Berkenbosch T, Benningshof JCJ, Stumpfe D, Bajorath J (2017). Charting biologically relevant spirocyclic compound space. Chem. Eur. J..

[42] Talele TT (2020). Opportunities for tapping into three-dimensional chemical space through a quaternary carbon. J. Med. Chem..

[43] Bera S, Daniliuc CG, Studer A (2017). Oxidative *N*-heterocyclic carbene catalyzed dearomatization of indoles to spirocyclic indolenines with a quaternary carbon stereocenter. Angew. Chem. Int. Ed..

[44] Bai Y, Davis DC, Dai M (2017). Natural product synthesis via palladium-catalyzed carbonylation. J. Org. Chem..

[45] Ma K, Martin BS, Yin X, Dai M (2019). Natural product syntheses: via carbonylative cyclizations. Nat. Prod. Rep..

[46] Breuers CBJ, Daniliuc CG, Studer A (2022). Dearomatizing cyclization of 2-iodoindoles by oxidative NHC catalysis to access spirocyclic indolenines and oxindoles bearing an all carbon quaternary stereocenter. Org. Lett..

[47] Yasui M (2023). Synthesis of spiro[indole-3,3′-pyrrolidine]-2′-(thi)ones. J. Org. Chem..

[48] Ren W, Yamane M (2010). Mo(CO)_6_-mediated carbamoylation of aryl halides. J. Org. Chem..

[49] Ueda T, Konishi H, Manabe K (2013). Palladium-catalyzed reductive carbonylation of aryl halides with *N*-formylsaccharin as a CO source. Angew. Chem. Int. Ed..

[50] Tan Y (2021). *N*-formylsaccharin as a CO source: applications and recent developments. ChemistrySelect.

[51] You SL (2007). Recent developments in asymmetric transfer hydrogenation with Hantzsch esters: a biomimetic approach. Chem. Asian. J..

[52] Susanti D, Ng LLR, Chan PWH (2014). Silica gel-mediated hydroamination/semipinacol rearrangement of 2-alkylaminophenylprop-1-yn-3-ols: synthesis of 2-oxindoles from alkynes and 1-(2-aminophenyl) ketones. Adv. Synth. Catal..

[53] Bu L (2018). Organocatalytic asymmetric cascade aerobic oxidation and semipinacol rearrangement reaction: a visible light-induced approach to access chiral 2,2-disubstituted indolin-3-ones. Chem. Asian J..

[54] RDKit: Open-source cheminformatics v2022.03.5 http://www.rdkit.org

[55] Tanwar S (2022). A new ChEMBL dataset for the similarity-based target fishing engine FastTargetPred: annotation of an exhaustive list of linear tetrapeptides. Data Brief.

[56] Wishart DS (2018). DrugBank 5.0: a major update to the DrugBank database for 2018. Nucleic Acids Res..

[57] Advanced collection. *Enamine* https://enamine.net/hit-finding/compound-collections/screening-collection/advanced-collection (2020).

[58] Sauer WHB, Schwarz MK (2003). Molecular shape diversity of combinatorial libraries: a prerequisite for broad bioactivity. J. Chem. Inf. Comp. Sci..

[59] Ertl P, Roggo S, Schuffenhauer A (2008). Natural product-likeness score and its application for prioritization of compound libraries. J. Chem. Inf. Model..

[60] Bickerton GR, Paolini GV, Besnard J, Muresan S, Hopkins AL (2012). Quantifying the chemical beauty of drugs. Nat. Chem..

[61] Pedregosa F (2011). Scikit-learn: machine learning in Python. J. Mach. Learn Res..

[62] Doveston R, Marsden S, Nelson A (2014). Towards the realisation of lead-oriented synthesis. Drug Discov. Today.

[63] Drugbank appoved and experimental drugs *Drugbank* https://www.drugbank.ca/releases/latest#structures (2020).

[64] Wu F, Zhang Y, Sun B, McMahon AP, Wang Y (2017). Hedgehog signaling: from basic biology to cancer therapy. Cell Chem. Biol..

[65] Hui CC, Angers S (2011). Gli proteins in development and disease. Annu. Rev. Cell Dev. Biol..

[66] Peukert S, Miller-Moslin K (2010). Small-molecule inhibitors of the hedgehog signaling pathway as cancer therapeutics. ChemMedChem.

[67] Wu X, Walker J, Zhang J, Ding S, Schultz PG (2004). Purporphamine induces osteogenesis by activation of the Hedgehog signaling pathway. Chem. Biol..

[68] Kremer L (2017). Discovery of a novel inhibitor of the Hedgehog signaling pathway through cell-based compound discovery and target prediction. Angew. Chem. Int. Ed..

[69] Sinha S, Chen JK (2006). Purmorphamine activates the Hedgehog pathway by targeting Smoothened. Nat. Chem. Biol..

[70] Chen JK, Taipale J, Cooper MK, Beachy PA (2002). Inhibition of Hedgehog signaling by direct binding of cyclopamine to Smoothened. Genes Dev..

[71] Yauch RL (2009). Smoothened mutation confers resistance to a Hedgehog pathway inhibitor in medulloblastoma. Science.

[72] Bray M-A (2016). Cell Painting, a high-content image-based assay for morphological profiling using multiplexed fluorescent dyes. Nat. Protoc..

[73] Caicedo JC, Singh S, Carpenter AE (2016). Applications in image-based profiling of perturbations. Curr. Opin. Biotechnol..

[74] Christoforow A (2019). Design, synthesis, and phenotypic profiling of pyrano-furo-pyridone pseudo natural products. Angew. Chem. Int. Ed..

[75] Ziegler S, Sievers S, Waldmann H (2021). Morphological profiling of small molecules. Cell Chem. Biol..

[76] Pahl A (2023). Morphological subprofile analysis for bioactivity annotation of small molecules. Cell Chem. Biol..

[77] Salic A, Mitchison TJ (2008). A chemical method for fast and sensitive detection of DNA synthesis in vivo. Proc. Natl Acad. Sci. USA.

[78] Schölermann B (2022). Identification of dihydroorotate dehydrogenase inhibitors using the cell painting assay. ChemBioChem.

[79] Wu HL (2022). Targeting nucleotide metabolism: a promising approach to enhance cancer immunotherapy. J. Hematol. Oncol..

[80] Akbarzadeh M (2022). Morphological profiling by means of the cell painting assay enables identification of tubulin-targeting compounds. Cell Chem. Biol..

[81] Lyu J (2019). Ultra-large library docking for discovering new chemotypes. Nature.

[82] Sadybekov AA (2022). Synthon-based ligand discovery in virtual libraries of over 11 billion compounds. Nature.

[83] Tukey, J. W. *Explanatory Data Analysis* (Addison-Wesley, 1977).

[84] mpimp-comas/2023_bag_bio_diverse_pnps: revision. *Zenodo*10.5281/zenodo.8320827 (2023).

