# Supplementary material for: A divergent intermediate strategy yields biologically diverse pseudo-natural products
Source: Nat Chem. 2024 Feb 16;16(6):945–58. doi: 10.1038/s41557-024-01458-4 (PMC11164679; doi:10.1038/s41557-024-01458-4)
Supplement: Supplementary file 1 — Supplementary Tables 1–13; Figs. 1–27; cheminformatic, biological experimental and chemistry experimental details; and compound characterization data and spectra. [file 41557_2024_1458_MOESM1_ESM.pdf]

nature chemistry

Article https://doi.org/10.1038/s41557-024-01458-4

A divergent intermediate strategy yields biologically diverse pseudo-natural products

In the format provided by the authors and unedited

Contents

All Compounds' Identification 3
Supplementary Tables 17
Supplementary Figures 30
Cheminformatic Analysis 56
Biological Experimental Details 58
General Biological Experimental Details 58
Cell Painting Assay 59
Hedgehog-Dependent Osteoblast Differentiation Assay 62
Reverse Transcription Quantitative PCR (RT-qPCR) 64
Smoothened Binding Assay 65
Immunocytochemistry 66
In Vitro Tubulin Polymerization Assay 67
Flow Cytometry 68
Uridine Rescue Assay 69
Statistical Analyses 70
X-Ray Structure Analysis of B10 71
Chemistry Experimental Section 74
General Information 74
General Procedure A: Synthesis of 3-(2-bromobenzyl)-indole derivatives 75
General procedure B: Procedure for CO insertion followed by spiro-cyclization 101
Characterization data for Class A products 102
General procedure C: Synthesis of Class B from Class A 131
General procedure D: Synthesis of *N*-functional-group-appended indoline-indanone PNP (Class C) 146
Characterization data for Class C products 146
General procedure E: Synthesis of Class D 161
Characterization data for Class D products 161
General procedure G: Synthesis of 2,2'-disubstituted-3-oxindole derivatives 186
Characterization data for Intermediates of Class F & G 186
General procedure H: Synthesis of Class F 202

Characterization data for Class F products ... 202
Synthesis and characterization data of F26-F29 ... 220
General procedure I: Synthesis of Class G ... 224
Characterization data for Class G products ... 224
Synthesis of Compounds 5a-5d via oxa-Pictet-Spengler reactions of tryptophol and benzaldehyde derivatives. ... 238
General procedure J: Synthesis of Class H ... 241
Characterization data for Class H products ... 241
Supplementary References ... 245
NMR Spectra ... 247

All Compounds' Identification

Compounds included in the 159-membered diverse-Pseudo Natural Product (dPNP) collection with their corresponding synthetic yields. **Class A**: 41 members; **Class B**: 18 members; **Class C**: 18 members; **Class D**: 10 members; **Class E**: 19 members; **Class F**: 29 members; **Class G**: 20 members; **Class H**: 4 members.

CLASS-A

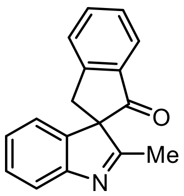	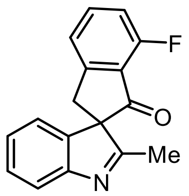	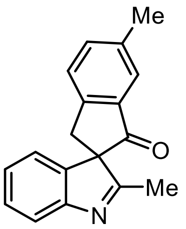	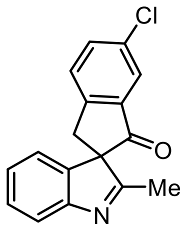
**A1** (82%)	**A2** (63%)	**A3** (84%)	**A4** (74%)
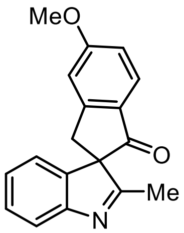	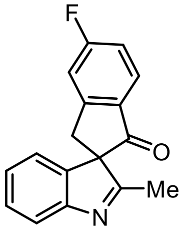	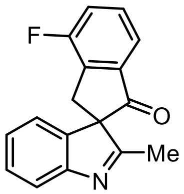	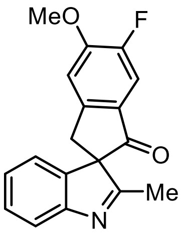
**A5** (79%)	**A6** (81%)	**A7** (70%)	**A8** (62%)
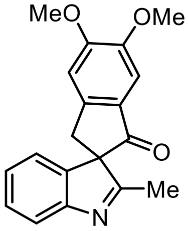	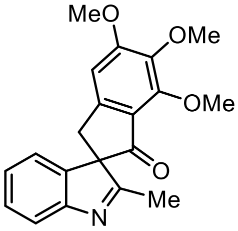	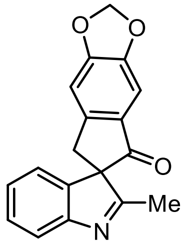	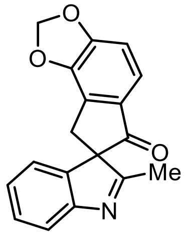
**A9** (58%)	**A10** (55%)	**A11** (68%)	**A12** (49%)
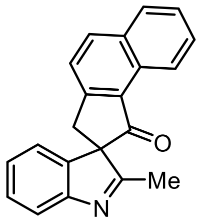	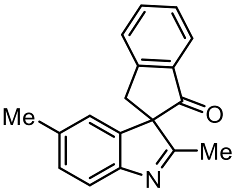	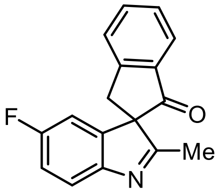	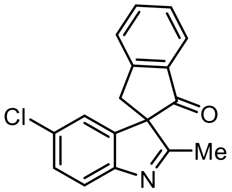
**A13** (48%)	**A14** (79%)	**A15** (76%)	**A16** (69%)

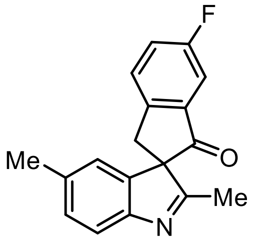	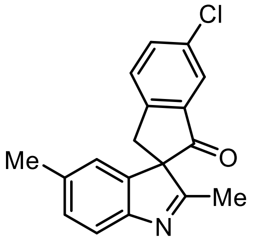	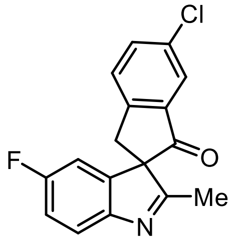	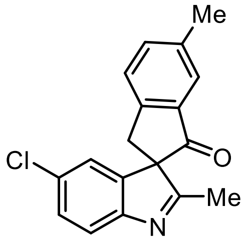
**A17** (81%)	**A18** (75%)	**A19** (59%)	**A20** (72%)
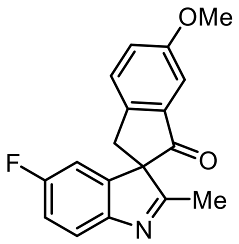	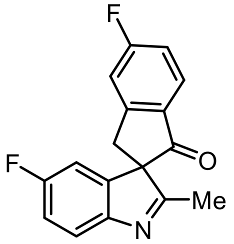	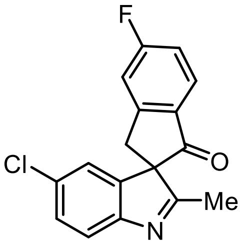	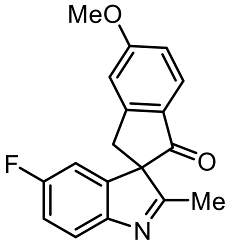
**A21** (76%)	**A22** (81%)	**A23** (73%)	**A24** (78%)
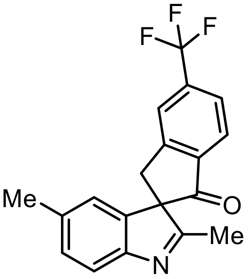	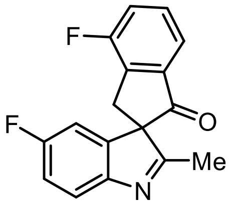	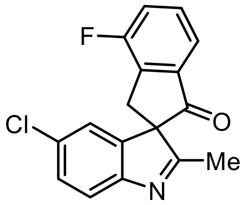	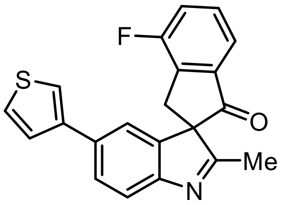
**A25** (59%)	**A26** (62%)	**A27** (74%)	**A28** (52%)
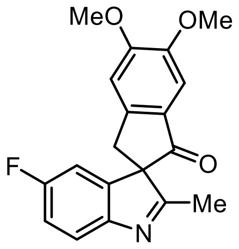	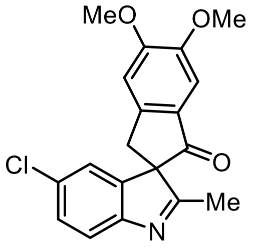	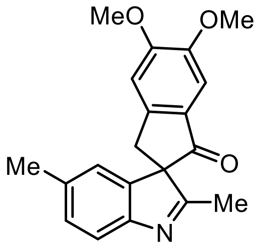	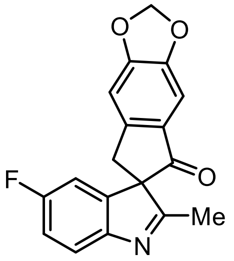
**A29** (68%)	**A30** (63%)	**A31** (72%)	**A32** (70%)

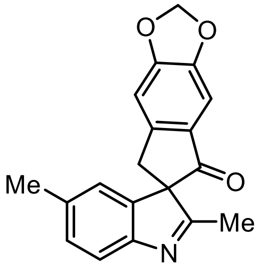	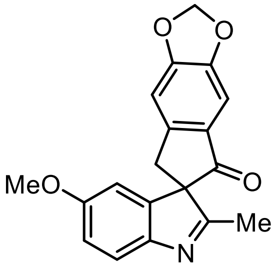	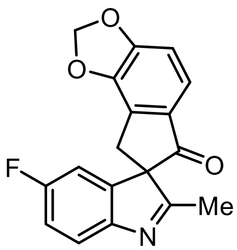	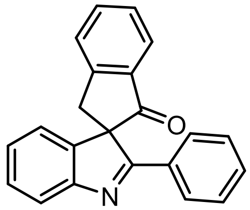
**A33** (79%)	**A34** (72%)	**A35** (47%)	**A36** (31%)
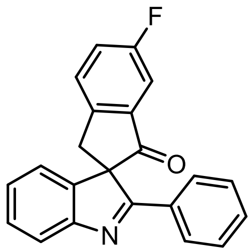	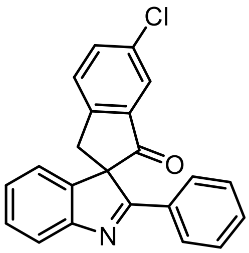	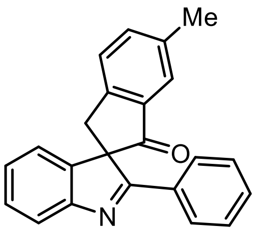	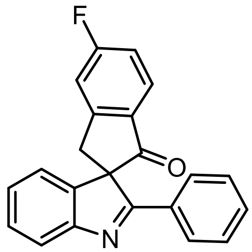
**A37** (39%)	**A38** (28%)	**A39** (45%)	**A40** (35%)
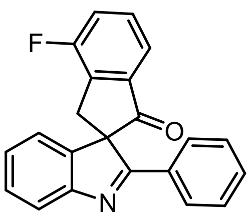			
**A41** (29%)			

CLASS-B

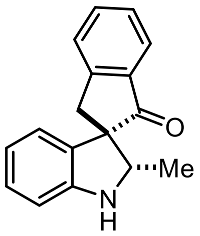	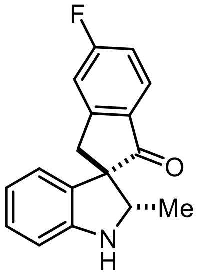	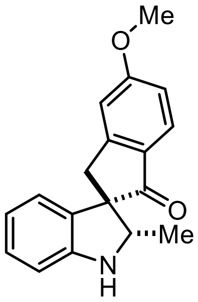	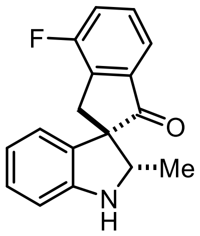
**B1** (88%; dr: 20:1)	**B2** (92%; dr: >20:1)	**B3** (85%; dr: 10:1)	**B4** (79%; dr: 15:1)
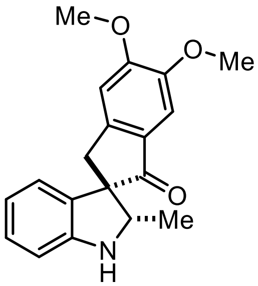	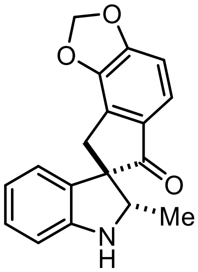	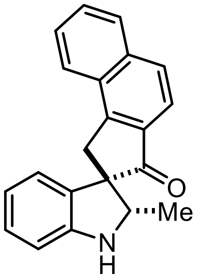	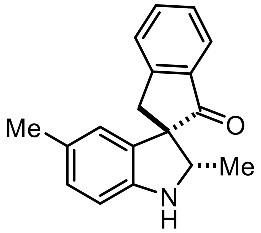
**B5** (80%; dr: >20:1)	**B6** (78%; dr: 11:1)	**B7** (81%; dr: 8:1)	**B8** (96%; dr: 15:1)
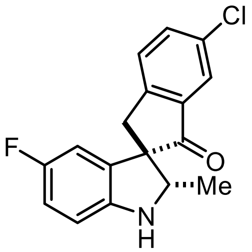	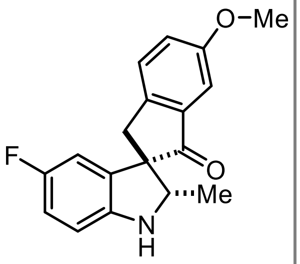	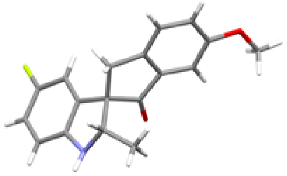	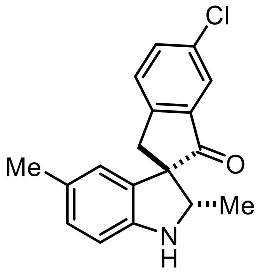
**B9** (83%; dr: >20:1)	**B10** (79%; dr: >20:1)	**B10 (CCDC: 2221540)**	**B11** (90%; dr: 8:1)
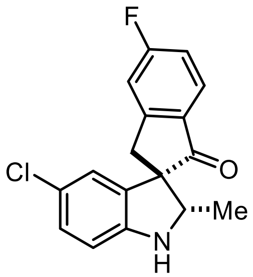	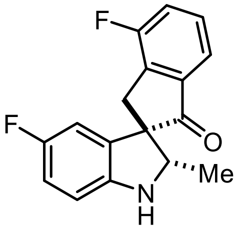	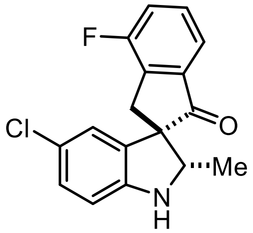	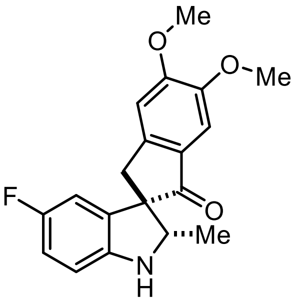
**B12** (78%; dr: >20:1)	**B13** (88%; dr: 20:1)	**B14** (90%; dr: 15:1)	**B15** (84%; dr: 12:1)

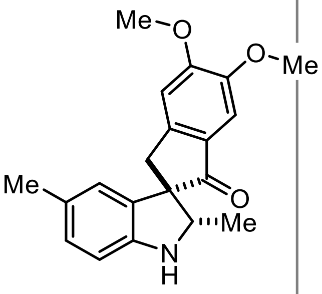	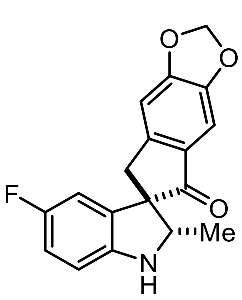	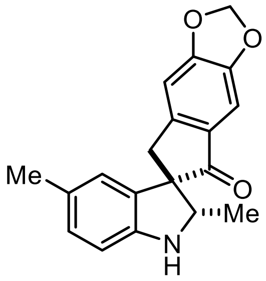
**B16** (78%; dr: 6:1)	**B17** (81%; dr: 10:1)	**B18** (87%; dr: 8:1)

CLASS-C

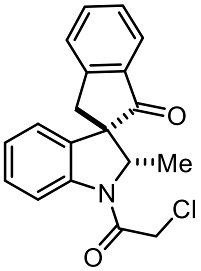	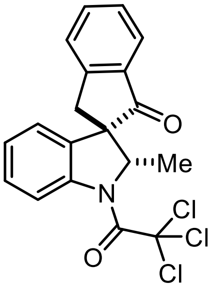	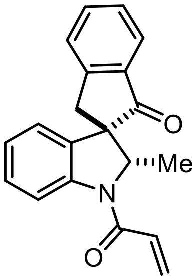	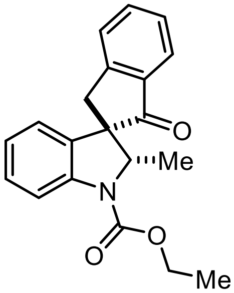
**C1** (86%)	**C2** (71%)	**C3** (82%)	**C4** (92%)
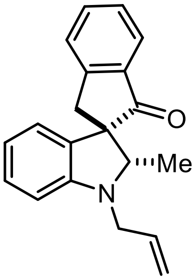	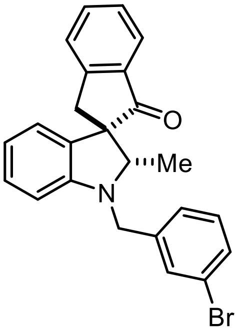	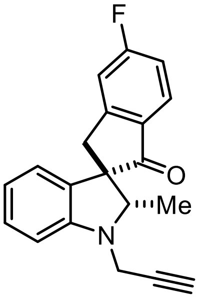	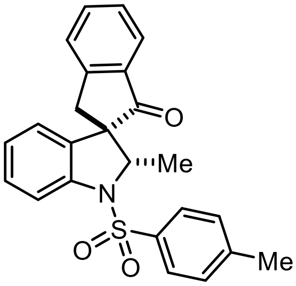
**C5** (79%)	**C6** (88%)	**C7** (80%)	**C8** (83%)
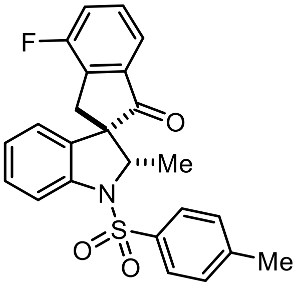	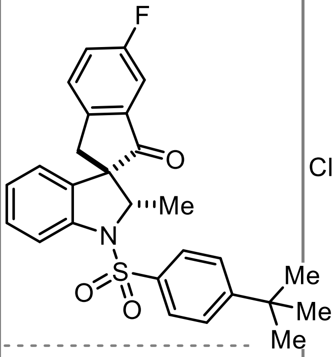	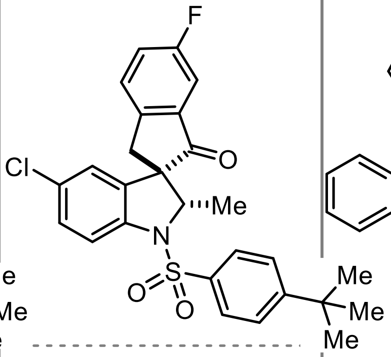	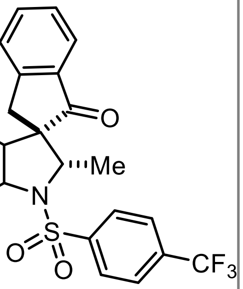
**C9** (88%)	**C10** (73%)	**C11** (78%)	**C12** (85%)
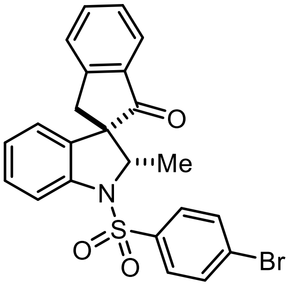	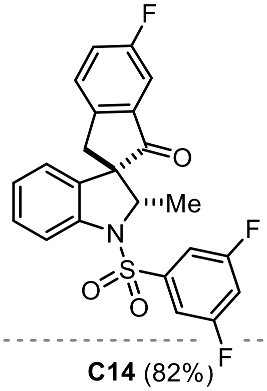	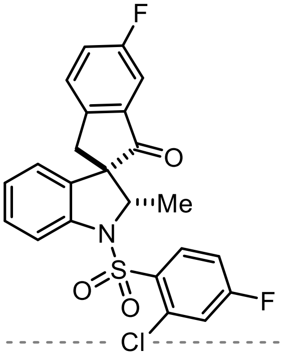	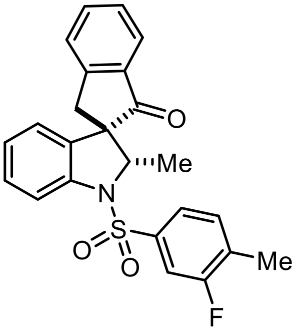
**C13** (80%)	**C14** (82%)	**C15** (66%)	**C16** (75%)

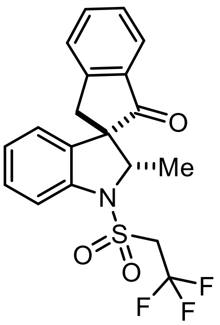	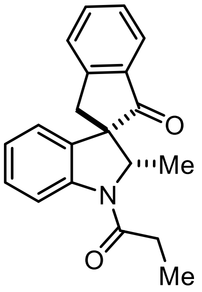
**C17** (72%)	**C18** (84%)

CLASS-D

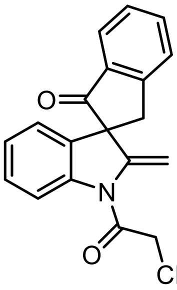<br>**D1** (71%)	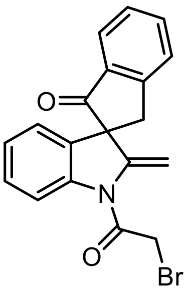<br>**D2** (67%)	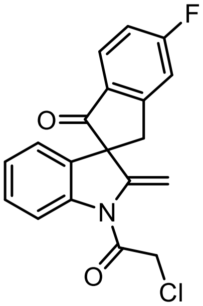<br>**D3** (78%)	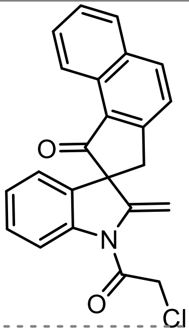<br>**D4** (74%)
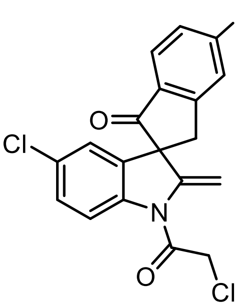<br>**D5** (81%)	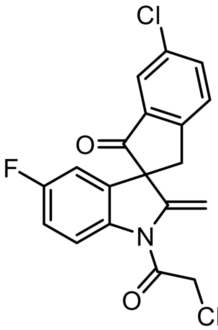<br>**D6** (80%)	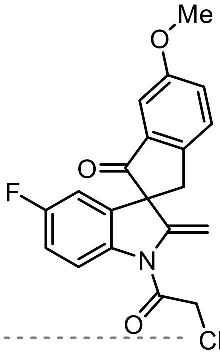<br>**D7** (75%)	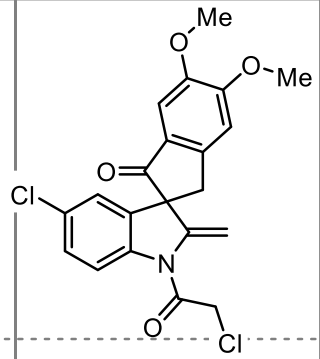<br>**D8** (72%)
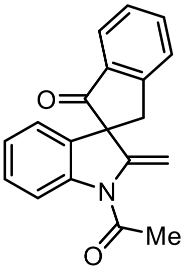<br>**D9** (82%)	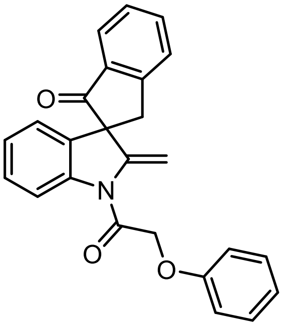<br>**D10** (69%)		

CLASS-E

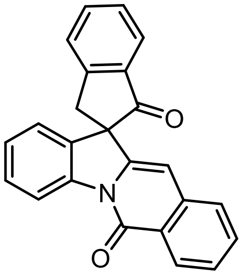	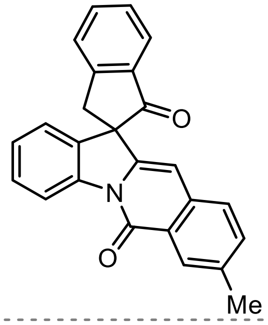	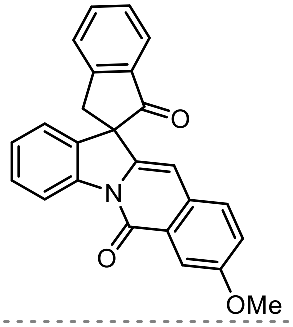	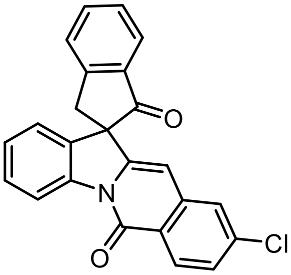
**E1** (78%)	**E2** (83%)	**E3** (76%)	**E4** (59%)
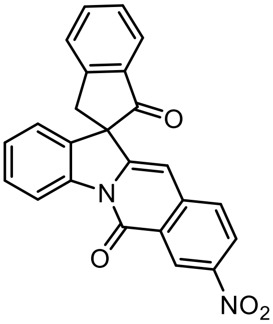	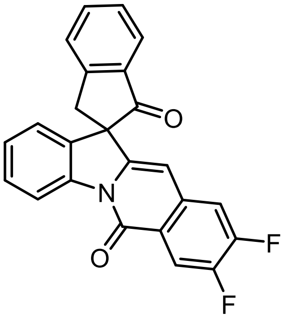	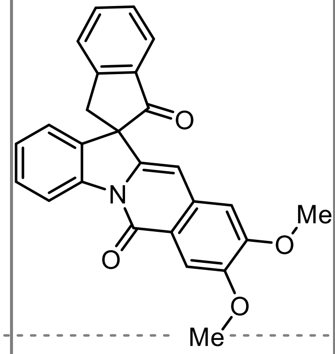	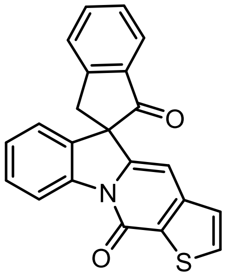
**E5** (43%)	**E6** (56%)	**E7** (67%)	**E8** (72%)
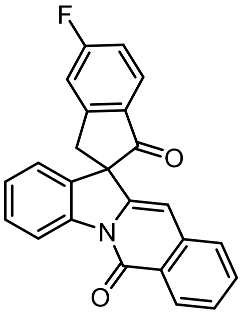	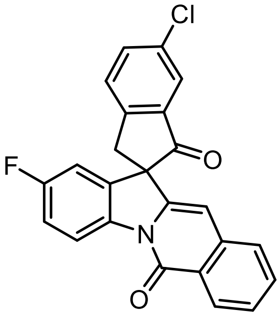	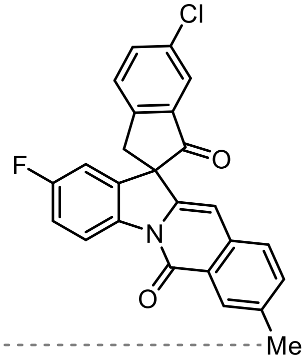	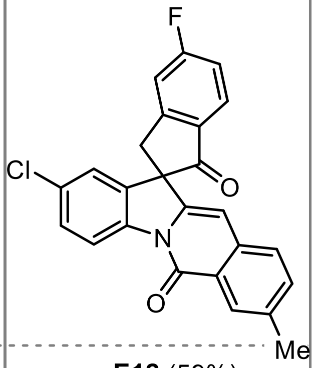
**E9** (77%)	**E10** (65%)	**E11** (68%)	**E12** (59%)
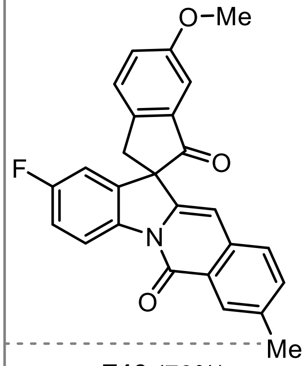	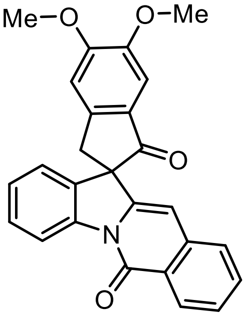	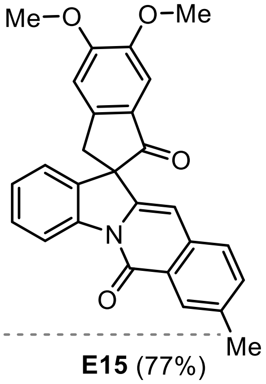	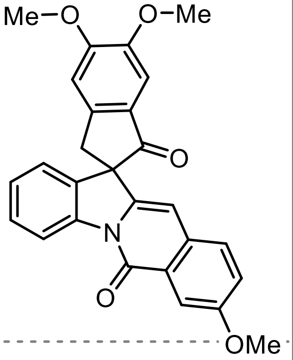
**E13** (70%)	**E14** (72%)	**E15** (77%)	**E16** (66%)

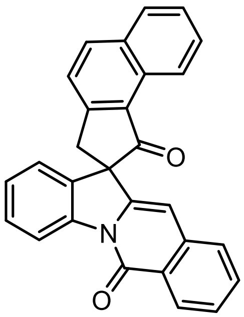	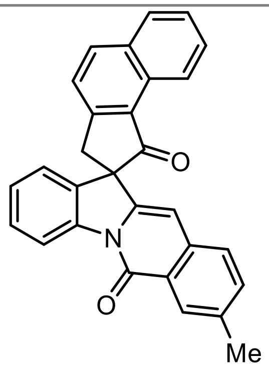	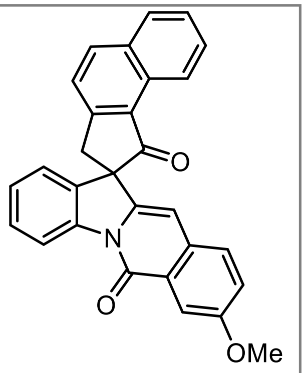
**E17** (60%)	**E18** (65%)	**E19** (63%)

CLASS-F

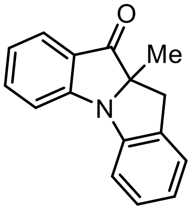	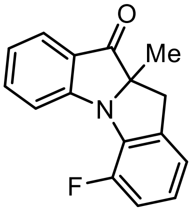	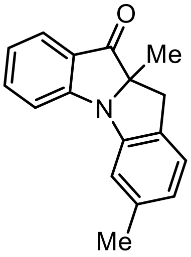	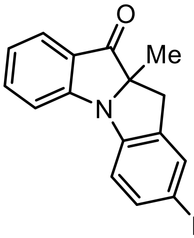
**F1** (59%)	**F2** (45%)	**F3** (65%)	**F4** (52%)
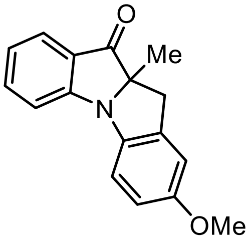	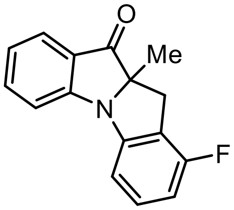	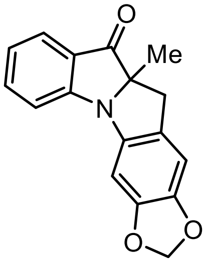	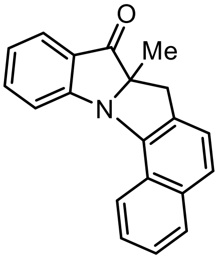
**F5** (57%)	**F6** (51%)	**F7** (56%)	**F8** (44%)
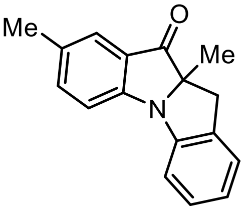	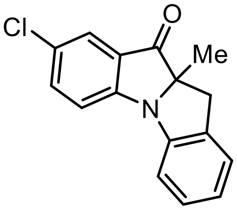	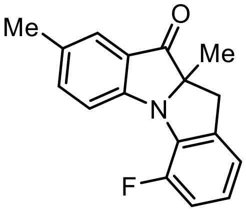	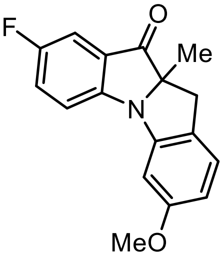
**F9** (66%)	**F10** (48%)	**F11** (45%)	**F12** (65%)
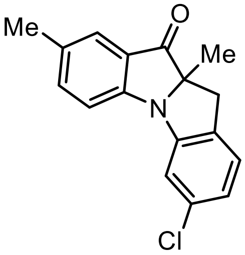	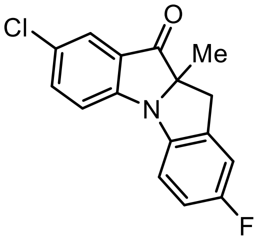	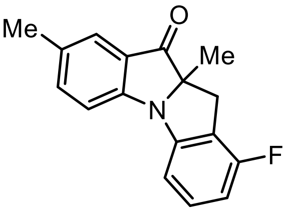	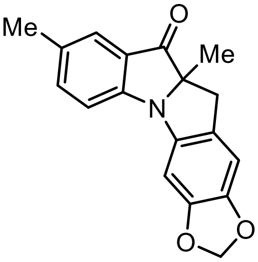
**F13** (53%)	**F14** (49%)	**F15** (52%)	**F16** (43%)

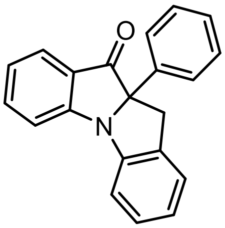	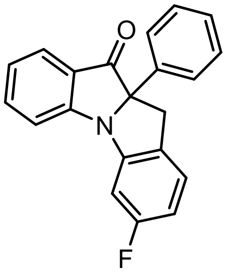	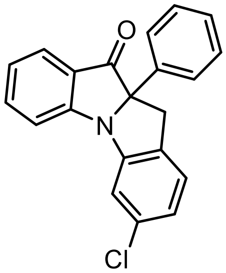	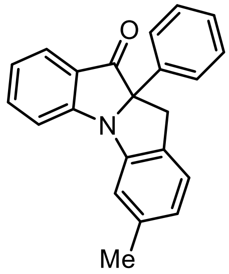
**F17** (52%)	**F18** (61%)	**F19** (48%)	**F20** (65%)
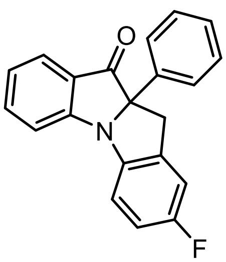	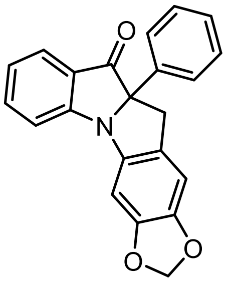	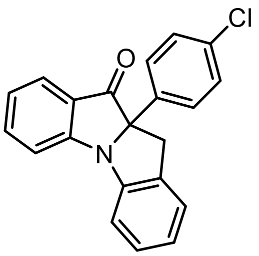	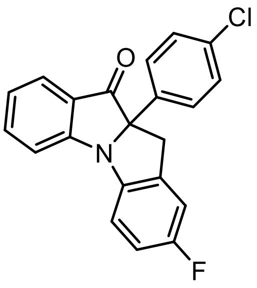
**F21** (60%)	**F22** (55%)	**F23** (43%)	**F24** (48%)
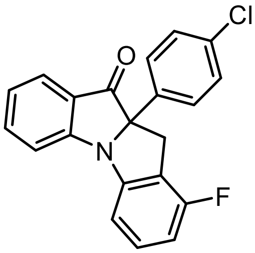	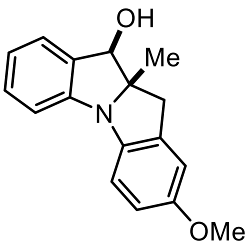	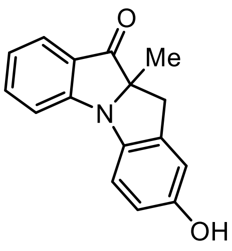	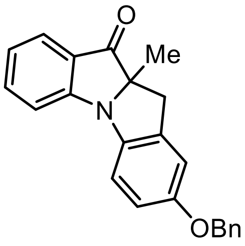
**F25** (45%)	**F26** (from **F5**, 62%)	**F27** (from **F5**, 99%)	**F28** (from **F27**, 70%)
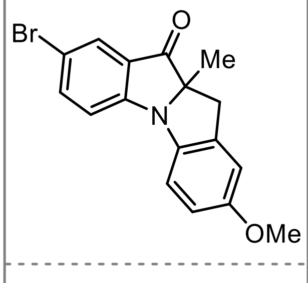			
**F29** (from **F5**, 73%)			

CLASS-G

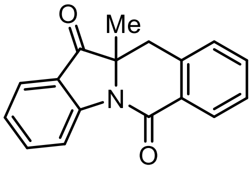	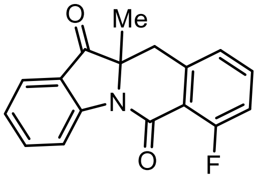	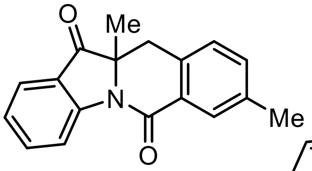	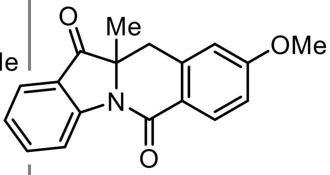
**G1** (68%)	**G2** (51%)	**G3** (63%)	**G4** (66%)
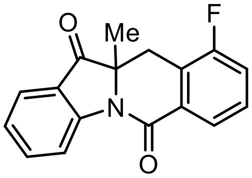	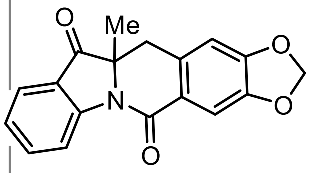	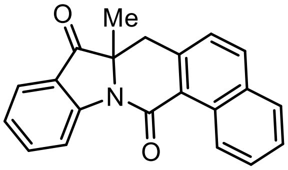	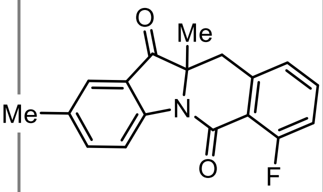
**G5** (53%)	**G6** (49%)	**G7** (47%)	**G8** (55%)
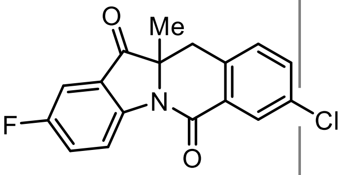	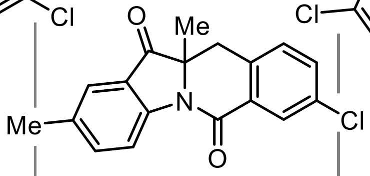	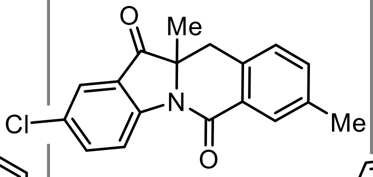	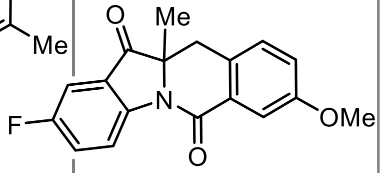
**G9** (54%)	**G10** (51%)	**G11** (48%)	**G12** (61%)
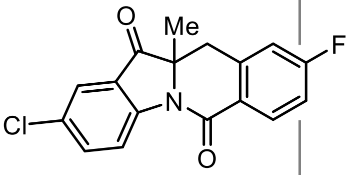	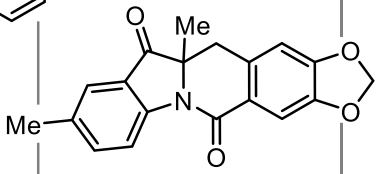	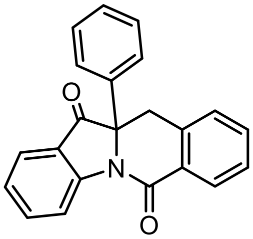	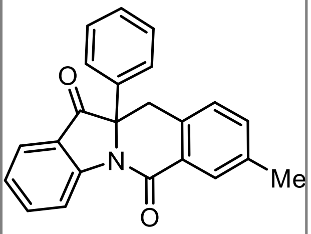
**G13** (52%)	**G14** (65%)	**G15** (63%)	**G16** (72%)

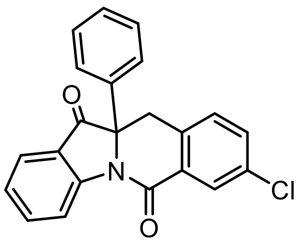	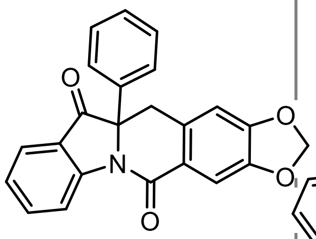	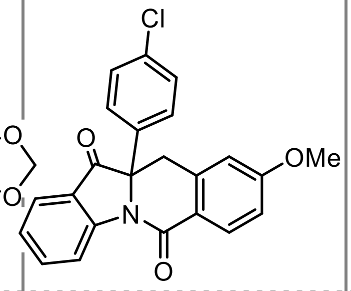	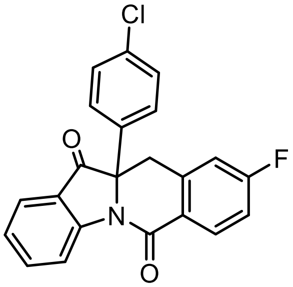
**G17** (58%)	**G18** (66%)	**G19** (43%)	**G20** (62%)

CLASS-H

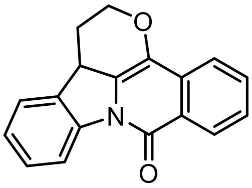	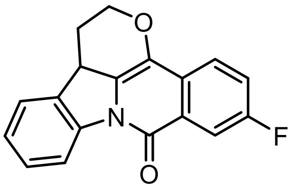	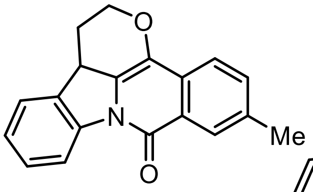	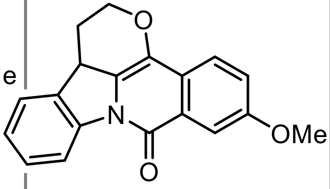
**H1** (53%)	**H2** (48%)	**H3** (62%)	**H4** (56%)

Supplementary Tables

Optimization details for CO insertion reaction:

Supplementary Table 1: Solvent optimization

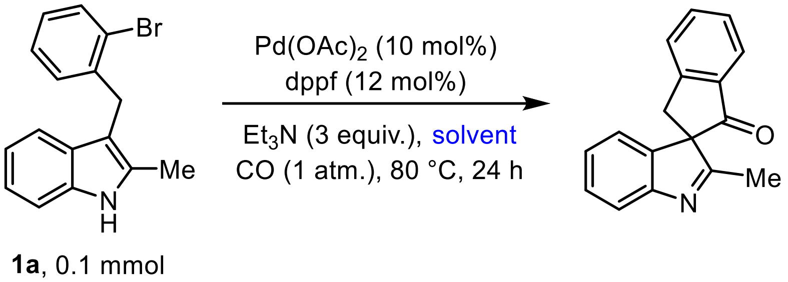

Entry	solvent	Yield[a]
1	Benzene	23%
2	Toluene	6%
3	*p*-Xylene	6%
4	MeCN	8%
5	THF	9%
6	1,4-Dioxane	4%
7	Et_2O	nd
8	DMF	nd
9	DMSO	7%
10	1,2-Dichloroethane	15%

[a]yields are based on ^{1}H NMR of the crude reaction mixture using 1,3,5-trimethoxybenzene as internal standard**.**

Supplementary Table 2: Ligand optimization

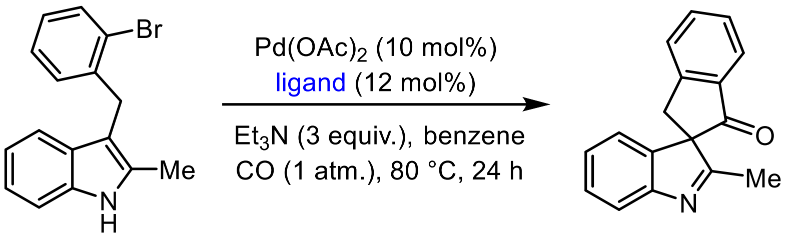

1a, 0.1 mmol

Entry	Ligand (12 mol%)	Yield[a]
1	dppf	23%
2	Xantphos	16%
3	BINAP	13%
4	dcpe	10%
5	dppe	<2%
6[b]	(dppf)$PdCl_2$ (10 mol%)	14%
7	dcpp	6%
8	dppp	5%
9	dppbe	nd

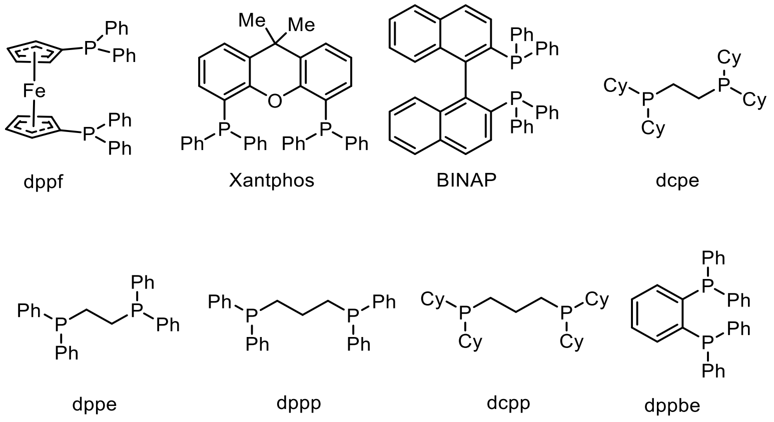

[a]yields are based on ^{1}H NMR of the crude reaction mixture using 1,3,5-trimethoxybenzene as internal standard. [b]in the absence of $Pd(OAc)_2$.

Supplementary Table 3: Base optimization

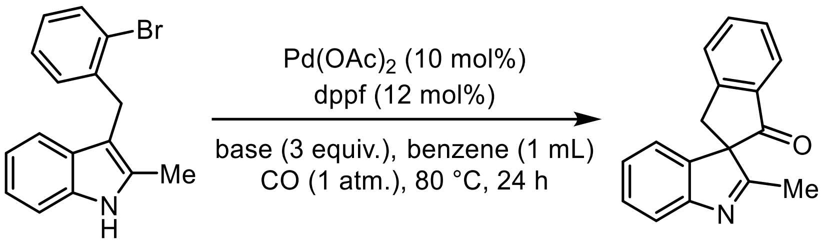

1a, 0.1 mmol

Entry	Base (3 equiv.)	Yield[a]
1	Et_3N	23%
2	K_2CO_3	22%
3	Na_2CO_3	12%
4	Cs_2CO_3	20%
5	Li_2CO_3	10%
6	$(^{i}Pr)_2NEt$	8%
7	TMEDA	<2%
8	DABCO	<2%

[a]yields are based on ^{1}H NMR of the crude reaction mixture using 1,3,5-trimethoxybenzene as internal standard**.**

Supplementary Table 4: Optimization of different CO sources:

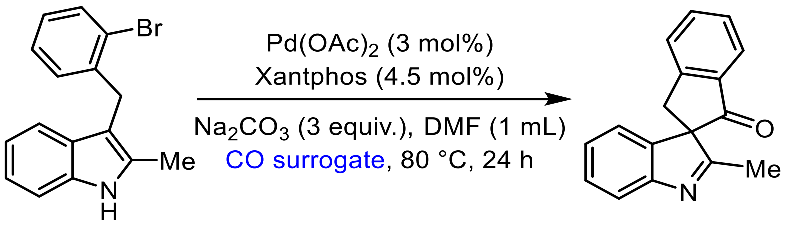

1a, 0.1 mmol

Entry	CO surrogate	Yield[a]
1	**2a** (1.5 equiv.)	66%
2	**2b** (1.5 equiv.)	nd
3	**2c** (1.5 equiv.)	nd
4[c]	**2d** (0.5 equiv.)	<5%
5[c]	**2e** (0.5 equiv.)	nd

CO sources:

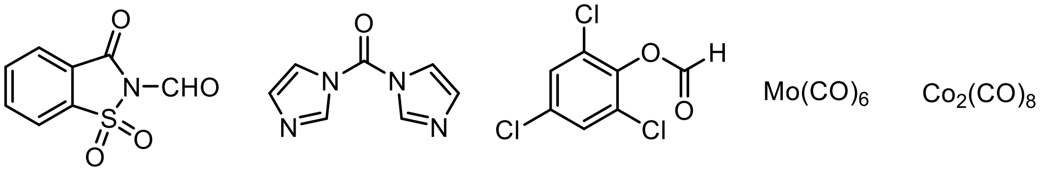

2a **2b** **2c** **2d** **2e**

[a]yields are based on ^{1}H NMR of the crude reaction mixture using 1,3,5-trimethoxybenzene as internal standard**.**
[c]DBU (1 equiv.) is used in addition.
nd: not detected.

Supplementary Table 5: Optimization of ligands:

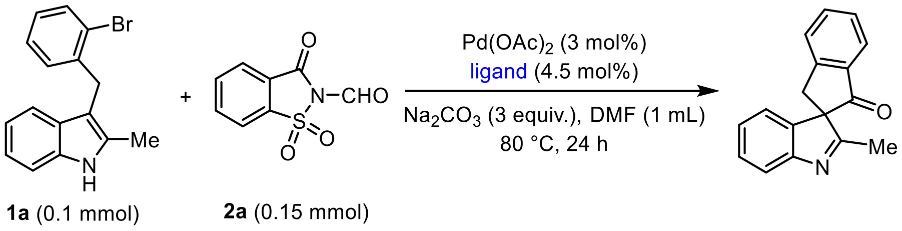

Entry	Ligand (4.5 mol%)	Yield[a]
1	dppf	34%
**2**	**Xantphos**	**66%**
3	dppb	57%
4	dcpb	11%
5	dppp	43%
6	dcpp	4%
7	DPEPhos	45%
8	(±) BINAP	42%

[a]yields are based on ^{1}H NMR of the crude reaction mixture using 1,3,5-trimethoxybenzene as internal standard**.**

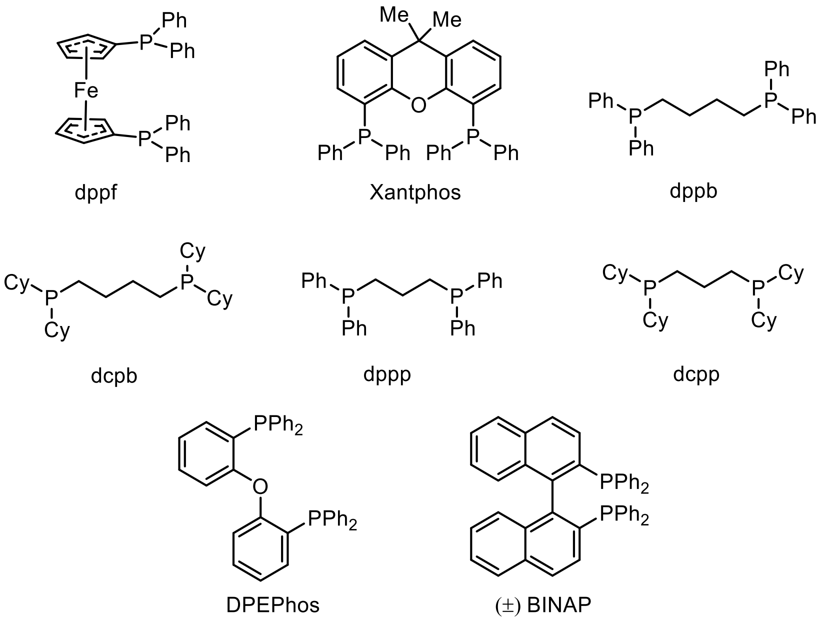

Supplementary Table 6: Optimization of catalyst/ligand ratio:

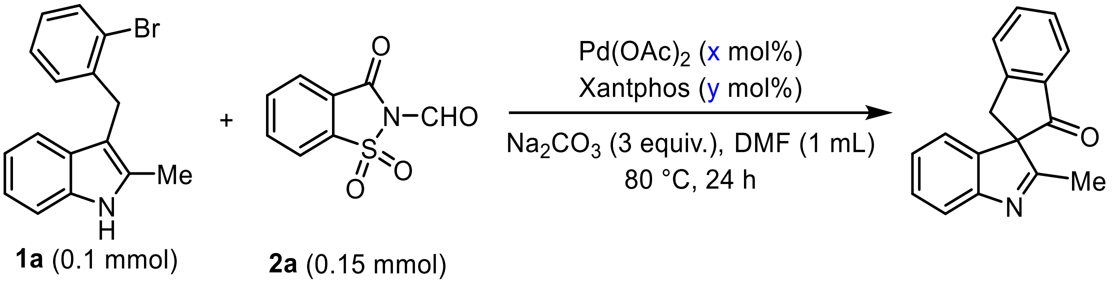

Entry	$Pd(OAc)_2$/Xantphos	Yield[a]
**1**	**3 mol%/4.5 mol%**	**66%**
2	3 mol%/6 mol%	57%
3	5 mol%/7.5 mol%	67%
4	5 mol%/10 mol%	62%

[a]yields are based on ^{1}H NMR of the crude reaction mixture using 1,3,5-trimethoxybenzene as internal standard**.**

Supplementary Table 7: Temperature optimization:

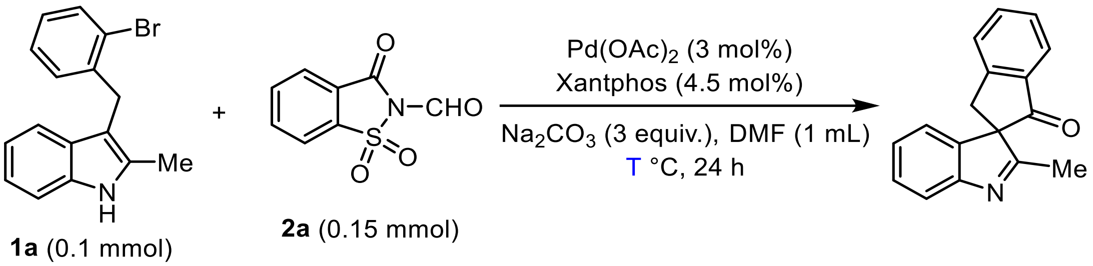

Entry	Temperature (˚C)	Yield[a]
1	75	51%
2	80	66%
**3**	**85**	**71%**
4	90	70%
5	100	68%

[a]yields are based on ^{1}H NMR of the crude reaction mixture using 1,3,5-trimethoxybenzene as internal standard**.**

Supplementary Table 8: Reaction time optimization:

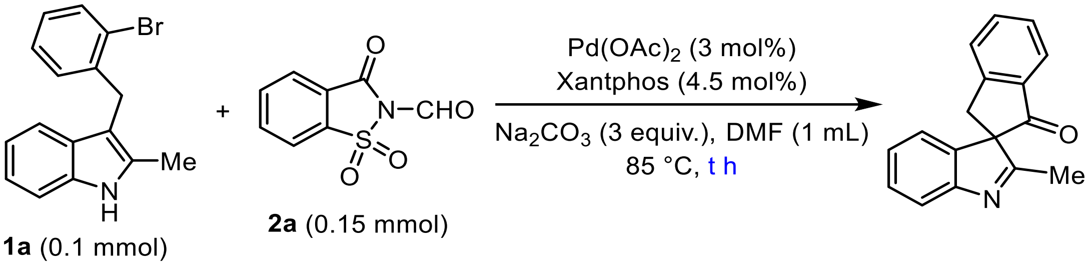

Entry	time (h)	Yield[a]
1	24	71%
2	30	75%
**3**	**36**	**86%**
4	48	83%

[a]yields are based on ^{1}H NMR of the crude reaction mixture using 1,3,5-trimethoxybenzene as internal standard**.**

Optimization details for Heck-type coupling followed by cyclization[1]

Supplementary Table 9: Optimization details for Class E

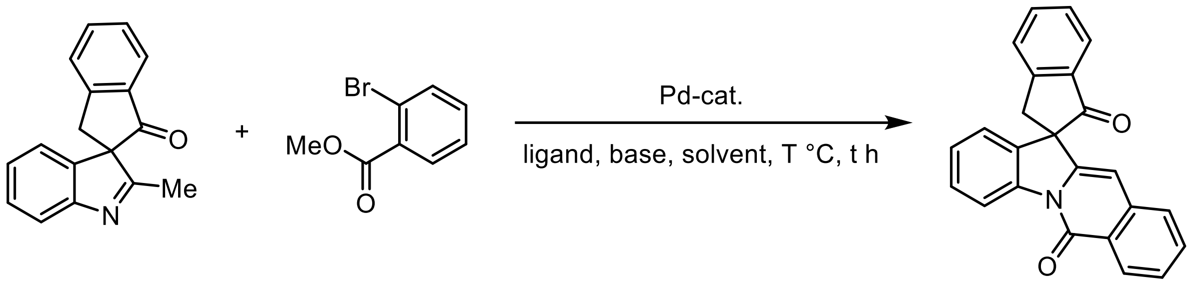

A1 (0.1 mmol) **3a** (1.5 equiv.) **E1**

Entry	$Pd(OAc)_2$	ligand	base	solvent	Temperature (˚C)	time (h)	Yield[a]
1	$Pd(OAc)_2$ (10 mol%)	Xantphos (20 mol%)	K_3PO_4 (3 equiv.)	1,4-dioxane	120	2	68%
2	$Pd(OAc)_2$ (10 mol%)	Xantphos (20 mol%)	K_3PO_4 (3 equiv.)	1,4-dioxane	120	3	82%
3	$Pd(OAc)_2$ (10 mol%)	Xantphos (20 mol%)	K_3PO_4 (3 equiv.)	1,4-dioxane	120	4	85%
4	$Pd(OAc)_2$ (7.5 mol%)	Xantphos (15 mol%)	K_3PO_4 (3 equiv.)	1,4-dioxane	120	3	78%
**5**	**$Pd(OAc)_2$ (7.5 mol%)**	**Xantphos (15 mol%)**	**K_3PO_4 (3 equiv.)**	**1,4-dioxane**	**120**	**4**	**84%**
6	$Pd(OAc)_2$ (5 mol%)	Xantphos (10 mol%)	K_3PO_4 (3 equiv.)	1,4-dioxane	120	4	66%
7	$Pd(OAc)_2$ (7.5 mol%)	Xantphos (15 mol%)	K_3PO_4 (3 equiv.)	Toluene	120	4	21%
8	$Pd(OAc)_2$ (7.5 mol%)	Xantphos (15 mol%)	K_2CO_3 (3 equiv.)	1,4-dioxane	120	4	46%
9	$Pd(OAc)_2$ (7.5 mol%)	Xantphos (15 mol%)	Et_3N (3 equiv.)	1,4-dioxane	120	4	nd
10	$Pd(OAc)_2$ (7.5 mol%)	BINAP (15 mol%)	K_3PO_4 (3 equiv.)	1,4-dioxane	120	4	11%
11	$Pd(OAc)_2$ (7.5 mol%)	PPh_3 (15 mol%)	K_3PO_4 (3 equiv.)	1,4-dioxane	120	4	5%

12	$Pd(OAc)_2$ (7.5 mol%)	DPPP (15 mol%)	K_3PO_4 (3 equiv.)	1,4-dioxane	120	4	nd
13	$Pd(OAc)_2$ (7.5 mol%)	dppf (15 mol%)	K_3PO_4 (3 equiv.)	1,4-dioxane	120	4	41%

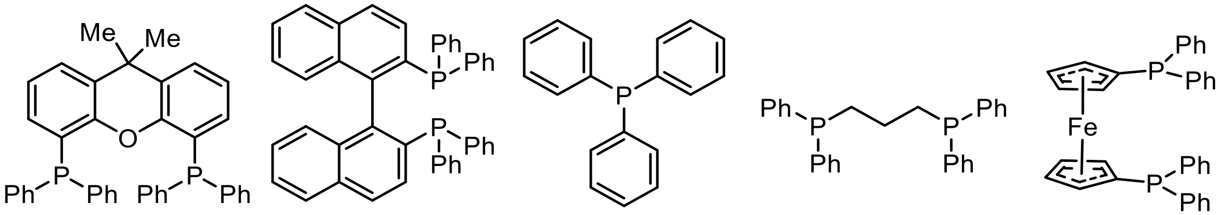

Xantphos BINAP P(Ph)$_3$ DPPP dppf

[a]yields are based on ^{1}H NMR of the crude reaction mixture using 1,3,5-trimethoxybenzene as internal standard**.**

Optimization details of semi-pinacol rearrangement:

Supplementary Table 10: Optimization details for visible-light photoredox-catalyzed semi-pinacol-type rearrangement

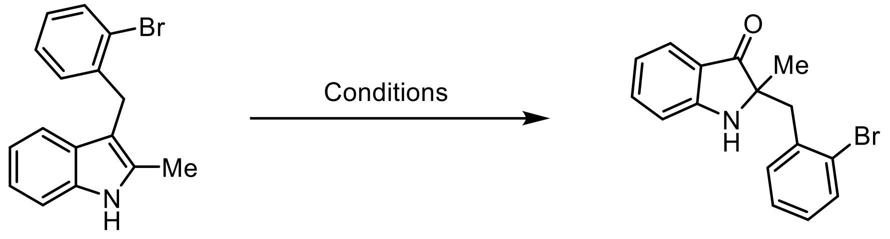

1a (0.25 mmol) **4a**

Entry	Conditions	Yield[a]
1	**Eosin Y** (5 mol%), $CHCl_3$ (1 mL), 23 W CFL, cooling fan, 36 h	58%
2	**Rhodamine B** (5 mol%), $CHCl_3$ (1 mL), 23 W CFL, cooling fan, 36 h	54%
3	**Rose Bengal** (5 mol%), $CHCl_3$ (1 mL), 23 W CFL, cooling fan, 36 h	38%
4	**Fluorescein** (5 mol%), $CHCl_3$ (1 mL), 23 W CFL, cooling fan, 36 h	42%
5	**[Ir(dtbbpy)(ppy)$_2$]PF$_6$** (2 mol%), $CHCl_3$ (1 mL), 23 W CFL, cooling fan, 36 h	36%
6	Eosin Y (5 mol%), **CH_2Cl_2 (1 mL)**, 23 W CFL, cooling fan, 36 h	43%
7	Eosin Y (**5 mol%**), SiO_2 (100 mg/0.1 mmol), $CHCl_3$ (1.5 mL), 23 W CFL, cooling fan, 36 h	66%
**8**	Eosin Y (**2 mol%**), SiO_2 (100 mg/0.1 mmol), $CHCl_3$ (1.5 mL), 23 W CFL, cooling fan, 36 h	**71%**[b]
9	Eosin Y (**1 mol%**), SiO_2 (100 mg/0.1 mmol), $CHCl_3$ (1.5 mL), 23 W CFL, cooling fan, 36 h	52%
10	Eosin Y (2 mol%), SiO_2 (100 mg/0.1 mmol), $CHCl_3$ (1.5 mL), 23 W CFL, cooling fan, **O_2**, 36 h	72%[b]

[a]yields are based on ^{1}H NMR of the crude reaction mixture using 1,3,5-trimethoxybenzene as internal standard**.** [b]As air and O_2 provided almost same results, all reactions were carried out under air atmosphere.

Optimization details for CO insertion followed by nucleophilic attack from *N*-center of 3-oxindole moiety:

Supplementary Table 11: Optimization details for synthesis of Class G

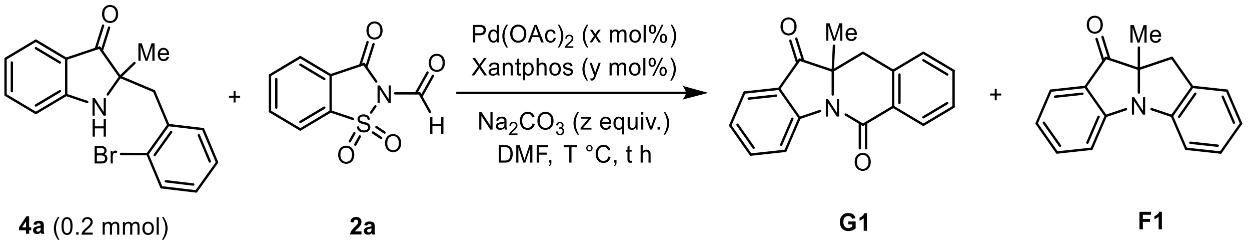

Entry	Conditions	Isolated yields of G1 & F1
1	**2a** (0.15 mmol), $Pd(OAc)_2$ (3 mol%), Xantphos (4.5 mol%), Na_2CO_3 (3 equiv.), DMF (1 mL), 85 ºC, 36 h	46% & 37%
2	**2a** (**0.2 mmol**), $Pd(OAc)_2$ (3 mol%), Xantphos (4.5 mol%), Na_2CO_3 (3 equiv.), DMF (1 mL), 85 ºC, 36 h	50% & 33%
3	**2a** (**0.2 mmol**), $Pd(OAc)_2$ (3 mol%), Xantphos (4.5 mol%), Na_2CO_3 (3 equiv.), DMF (1 mL), **90 ºC**, 36 h	51% & 26%
**4**	**2a** (**0.2 mmol**), $Pd(OAc)_2$ (**5 mol%**), Xantphos (**7.5 mol%**), Na_2CO_3 (3 equiv.), DMF (1 mL), **90 ºC**, 36 h	**68% & 29%**
5	**2a** (**0.2 mmol**), $Pd(OAc)_2$ (**5 mol%**), Xantphos (**7.5 mol%**), Na_2CO_3 (3 equiv.), DMF (1 mL), **100 ºC**, 36 h	62% & 28%
6	**2a** (**0.2 mmol**), $Pd(OAc)_2$ (**7.5 mol%**), Xantphos (**10 mol%**), Na_2CO_3 (3 equiv.), DMF (1 mL), **90 ºC**, 36 h	66% & 31%

Supplementary Table 12: Average values of descriptors per dataset.

Descriptor[a]	Enamine	DrugBank	ChEMBL NPs	Class A	Class B	Class C	Class D	Class E	Class F	Class G	Class H
NP_Like	-1.27	-0.12	1.93	0.02	0.32	-0.29	-0.17	-0.06	-0.09	-0.14	-0.07
QED	0.79	0.50	0.45	0.73	0.82	0.64	0.71	0.44	0.68	0.72	0.65
NumHA	23.01	30.11	32.46	22.20	21.61	27.76	25.20	29.90	21.83	23.45	22.00
MW	326.87	434.72	455.44	299.60	293.22	397.80	361.27	396.66	292.76	316.89	290.60
NumRings	2.97	3.17	3.74	4.33	4.22	4.59	4.20	6.15	4.63	4.50	5.00
NumRingsArom	1.84	1.91	1.21	2.15	2.06	2.59	2.20	4.15	2.50	2.35	3.00
NumRingsAli	1.13	1.25	2.53	2.18	2.17	2.00	2.00	2.00	2.13	2.15	2.00
NumHDon	1.29	3.09	2.66	0.00	1.00	0.00	0.00	0.00	0.00	0.00	0.00
NumHAcc	5.75	7.65	6.95	2.70	2.78	3.47	3.40	3.80	2.29	3.45	3.25
LogP	2.17	2.44	3.62	3.98	3.49	4.37	3.91	4.49	4.20	3.55	3.34
TPSA	71.13	110.13	104.93	35.89	36.28	44.95	41.07	47.23	23.00	41.53	33.54
NumRotBd	4.08	6.99	5.60	0.60	0.44	1.65	1.40	0.70	0.46	0.45	0.25
NumAtOx	2.36	4.39	6.02	1.70	1.78	2.47	2.40	2.75	1.29	2.45	2.25
NumAtN	3.39	3.26	0.93	1.00	1.00	1.00	1.00	1.05	1.00	1.00	1.00
NumAtHal	0.58	0.68	0.12	0.83	0.78	1.35	1.50	0.55	0.71	0.70	0.25
NumAtBridgehead	0.04	0.18	0.49	0.00	0.00	0.00	0.00	0.00	0.00	0.00	0.00
FCsp3	0.46	0.43	0.55	0.21	0.27	0.24	0.16	0.12	0.17	0.18	0.19

[a]Definitions of abbreviations: NP_Like = Natural product-likeness score; QED = Quantitative estimation of drug-likeness; NumHA = Number of heavy atoms, i.e. number of non-hydrogen atoms; MW = Molecular weight; NumRings = Number of rings; NumRingsArom = Number of aromatic rings; NumRingsAli = Number of aliphatic rings; NumHDon = Number of hydrogen donors; NumHAcc = Number of hydrogen acceptors; TPSA = Total polar surface area; NumRotBd = Number of rotatable bonds; NumAtOx = Number of oxygen atoms; NumAtN = Number of nitrogen atoms; NumAtHal = Number of halogen atoms; NumAtBridgehead = Number of bridgehead atoms; FCsp3 = Fraction of sp^3-hybridized carbons (number of sp^3-hybridized carbons/total number of carbons).

Supplementary Figures

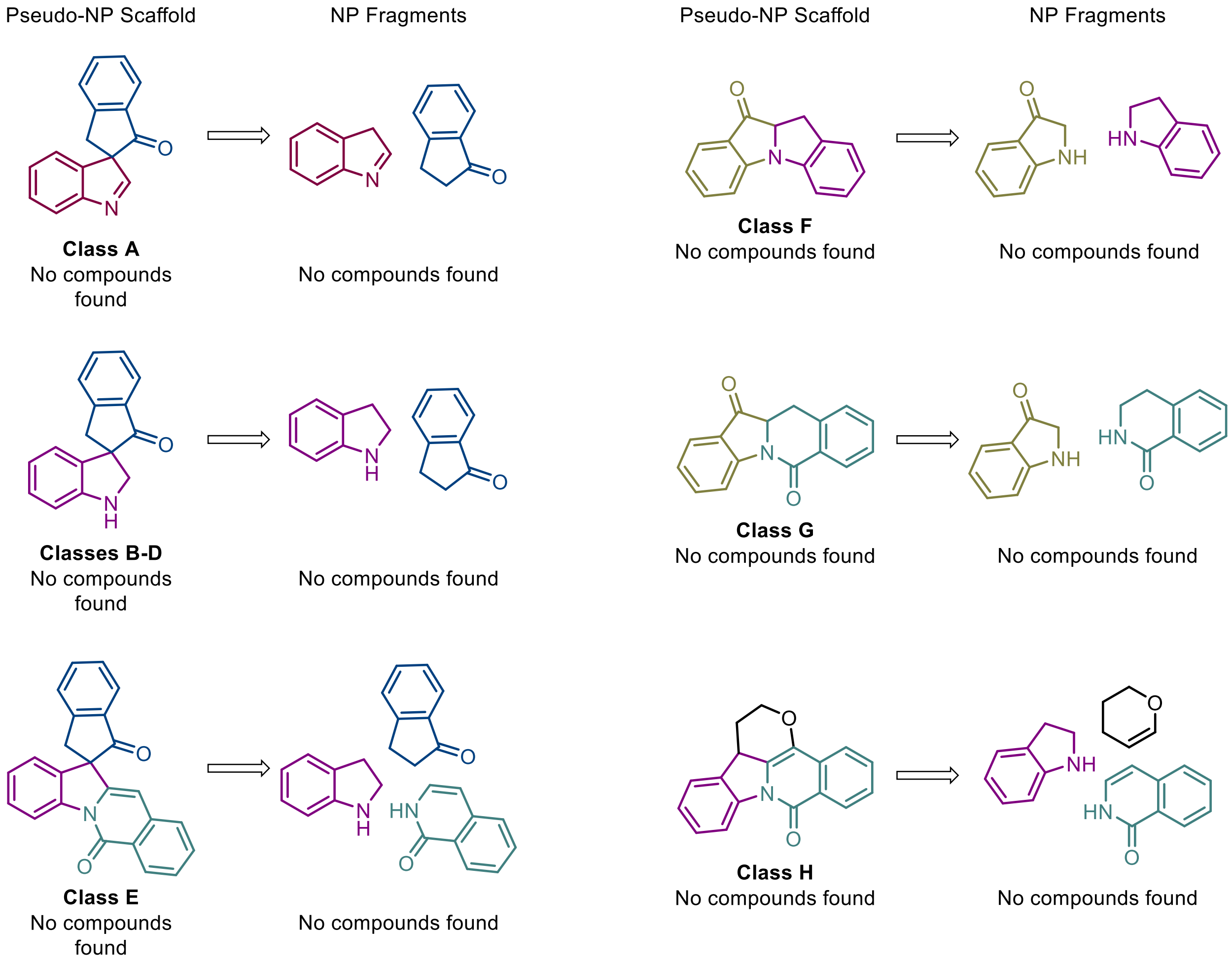

Supplementary Fig. 1: Substructure and fragment combination searches in the Dictionary of Natural Products database. The pseudo-NP scaffolds and their fragment combinations were searched in the Dictionary of Natural Products with tautomers included (https://dnp.chemnetbase.com/faces/chemical/ChemicalSearch.xhtml, accessed on 21.9.2022). A fragment combination search for **Class F** yielded 50 hits; however, none of these NPs contained both the indolin-3-one and indoline fragments.

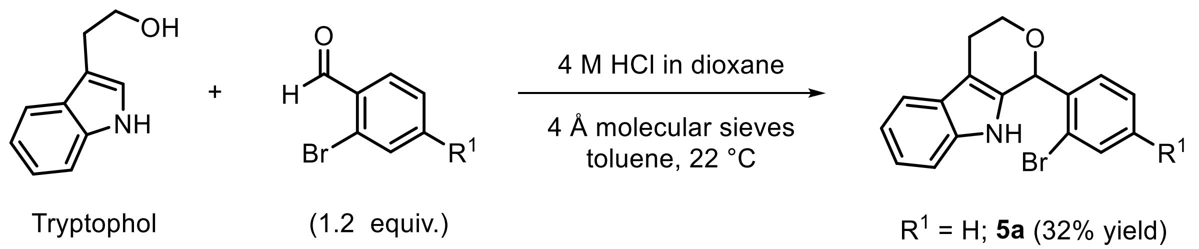

R[1] = H; **5a** (32% yield)
R[1] = F; **5b** (12% yield)
R[1] = Me; **5c** (13% yield)
R[1] = OMe; **5d** (35% yield)

Supplementary Fig. 2: Synthesis of compounds 5a-5d via oxa-Pictet-Spengler reactions of tryptophol and benzaldehyde derivatives.

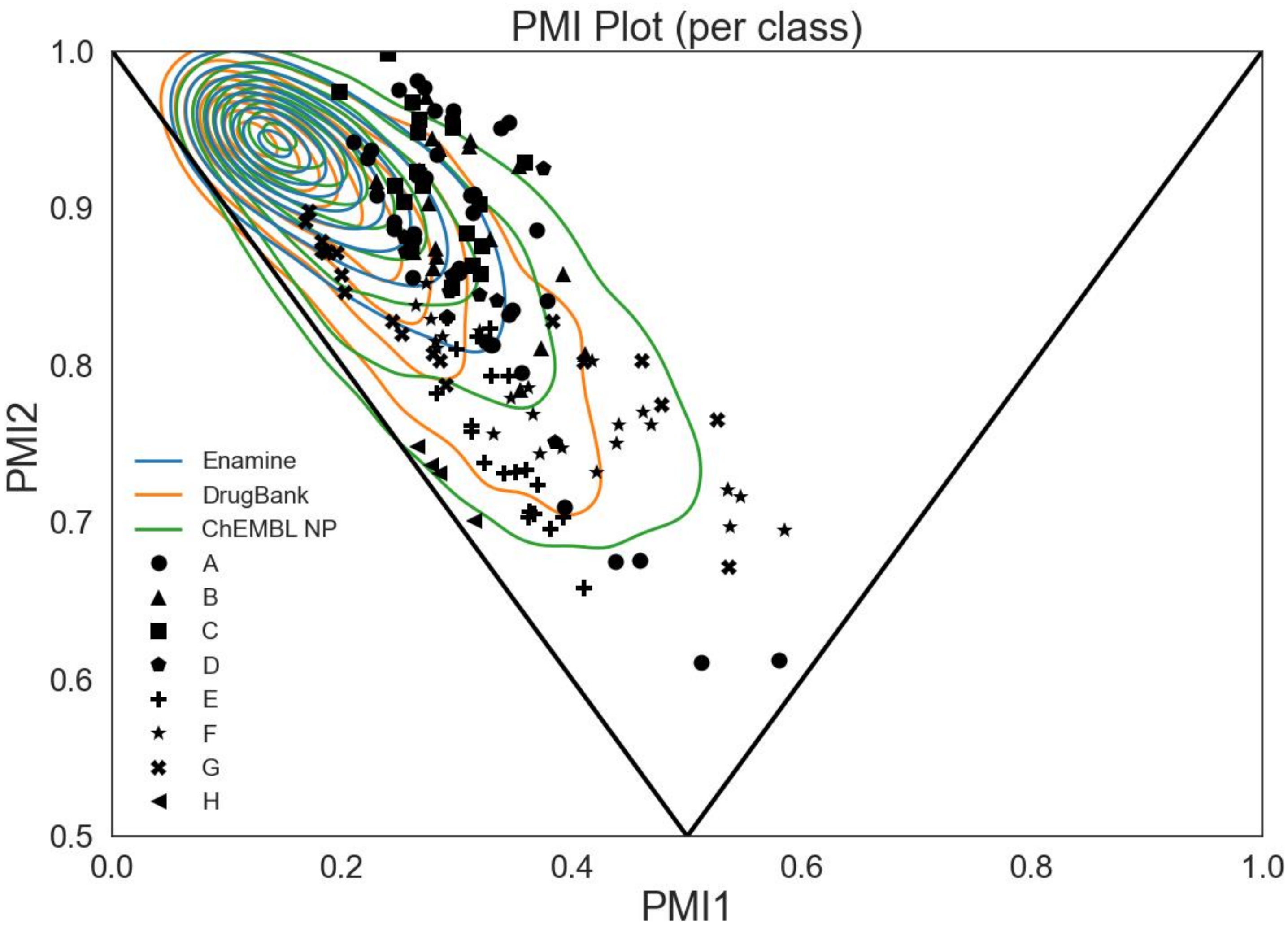

Supplementary Fig. 3: Principal moments of inertia plot per class. Principal moments of inertia (PMI) plot for the shape of the PNPs (154 members, 8 subclasses, different shaped black spots) and reference sets (shown as a contour plot: Enamine = blue, DrugBank = orange, ChEMBL NPs = green). The corners of the triangle within the plot indicate a rod-like shape (top left), disk-like shape (bottom middle), and sphere-like shape (top right). The contour lines represent a Gaussian kernel density estimation with 10 steps.

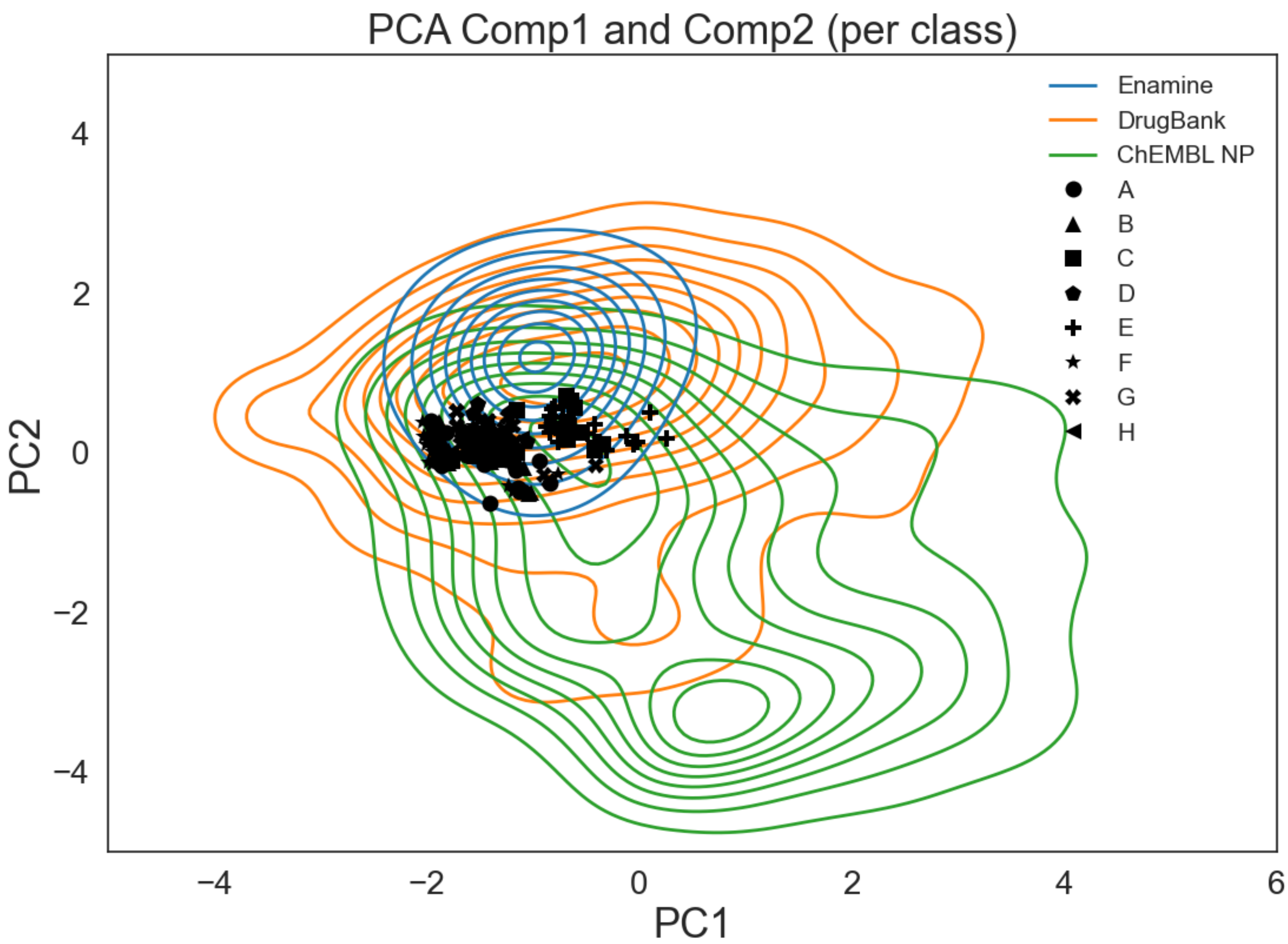

Supplementary Fig. 4: Principal component analysis of molecular descriptors for the pseudo-NPs and reference sets (PC1 vs PC2). The compounds in the analysis are pseudo-NPs from the manuscript (black shapes) and reference sets (shown as a contour plot: Enamine = blue, DrugBank = orange, ChEMBL NPs = green). For a list of the 17 molecular descriptors, see Supplementary Table 12. Explained variance: PC1 = 40.7%, PC2 = 19.8%, PC3 = 10.1%. The contour lines represent a Gaussian kernel density estimation with 10 steps.

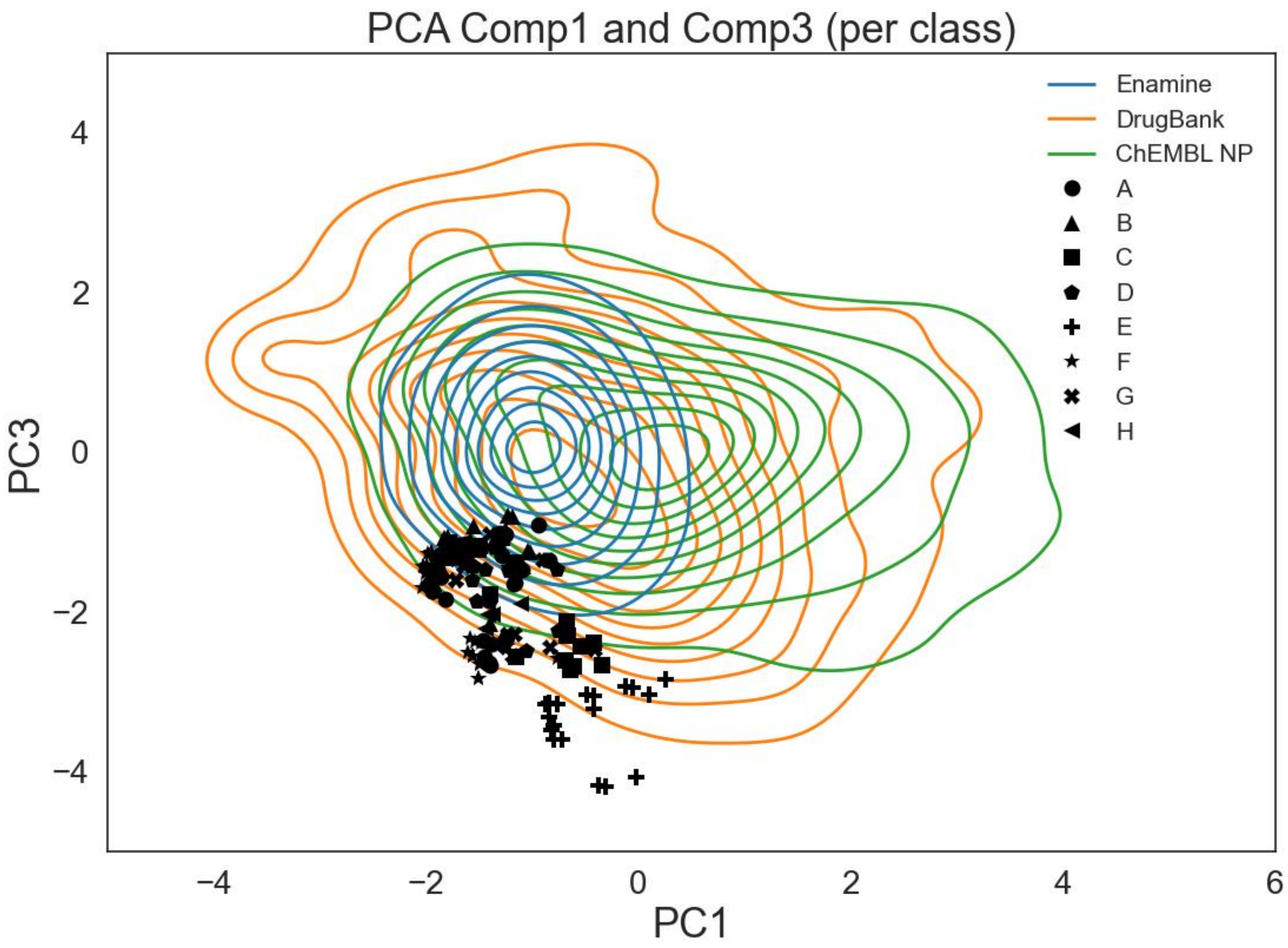

Supplementary Fig. 5: Principal component analysis of molecular descriptors for the pseudo-NPs and reference sets (PC1 vs PC3). The compounds in the analysis are pseudo-NPs from the manuscript (black shapes) and reference sets (shown as a contour plot: Enamine = blue, DrugBank = orange, ChEMBL NPs = green. For a list of the 17 molecular descriptors, see Supplementary Table 12. Explained variance: PC1 = 40.7%, PC2 = 19.8%, PC3 = 10.1%. The contour lines represent a Gaussian kernel density estimation with 10 steps.

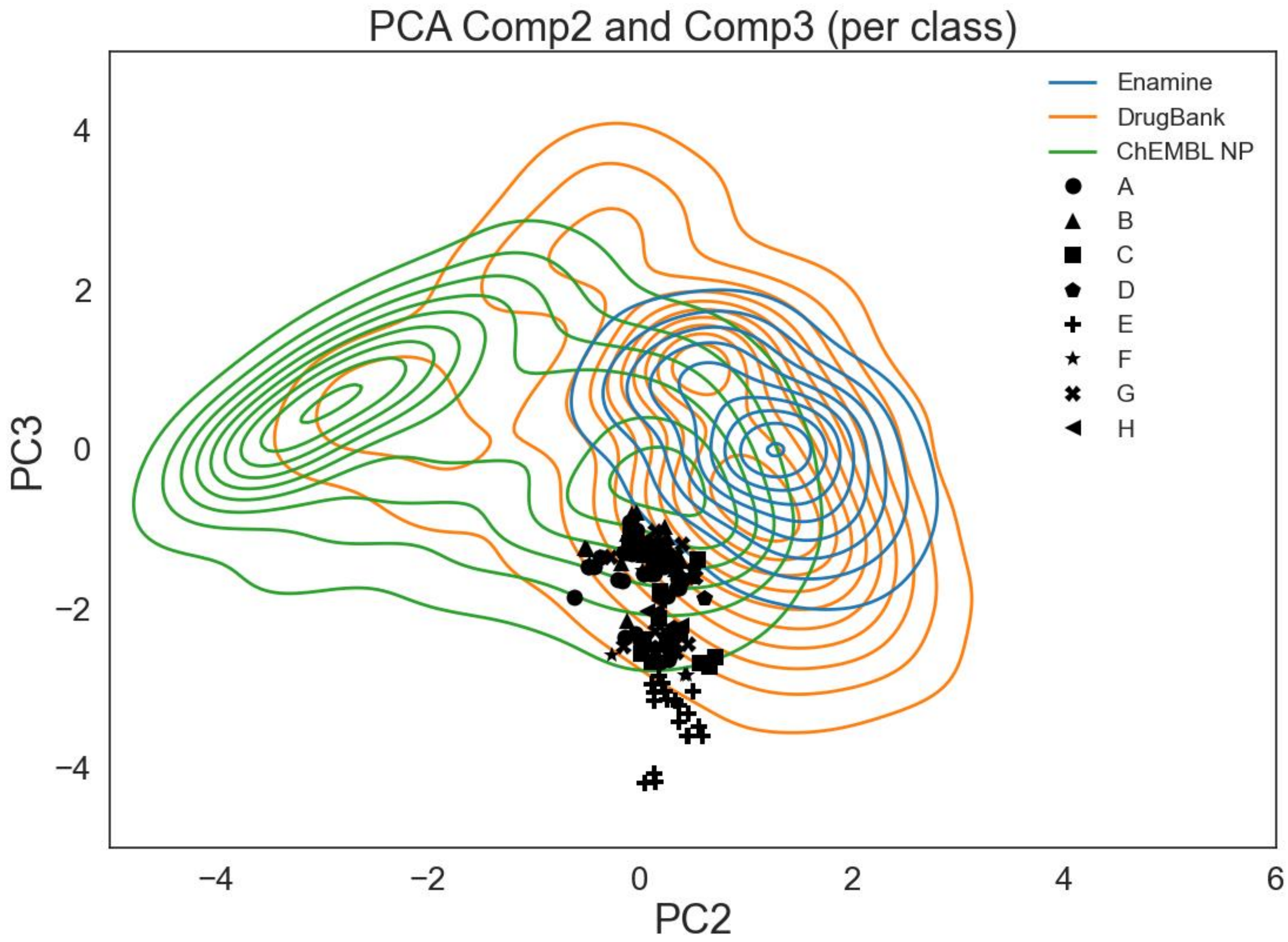

Supplementary Fig. 6: Principal component analysis of molecular descriptors for the pseudo-NPs and reference sets (PC2 vs PC3). The compounds in the analysis are pseudo-NPs from the manuscript (black shapes) and reference sets (shown as a contour plot: Enamine = blue, DrugBank = orange, ChEMBL NPs = green. For a list of the 17 molecular descriptors, see Supplementary Table 12. Explained variance: PC1 = 40.7%, PC2 = 19.8%, PC3 = 10.1%. The contour lines represent a Gaussian kernel density estimation with 10 steps.

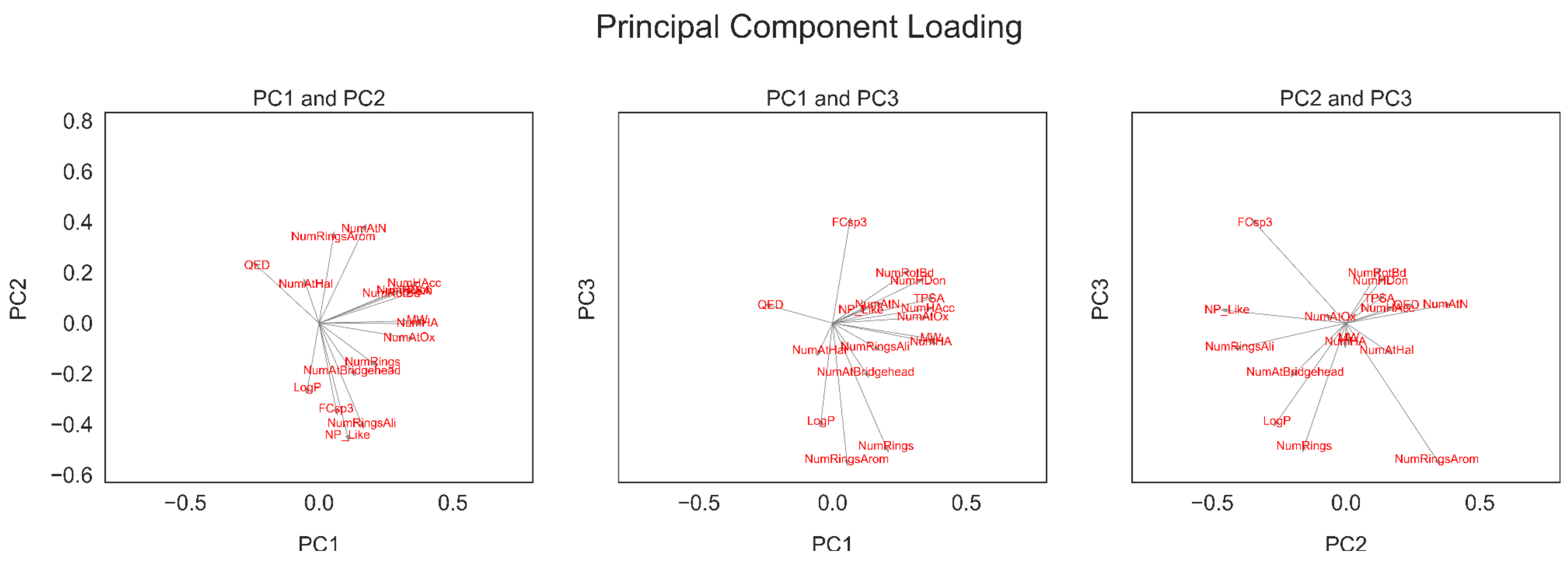

Supplementary Fig. 7: Principal component loading for Supplementary Figs. 4-6.

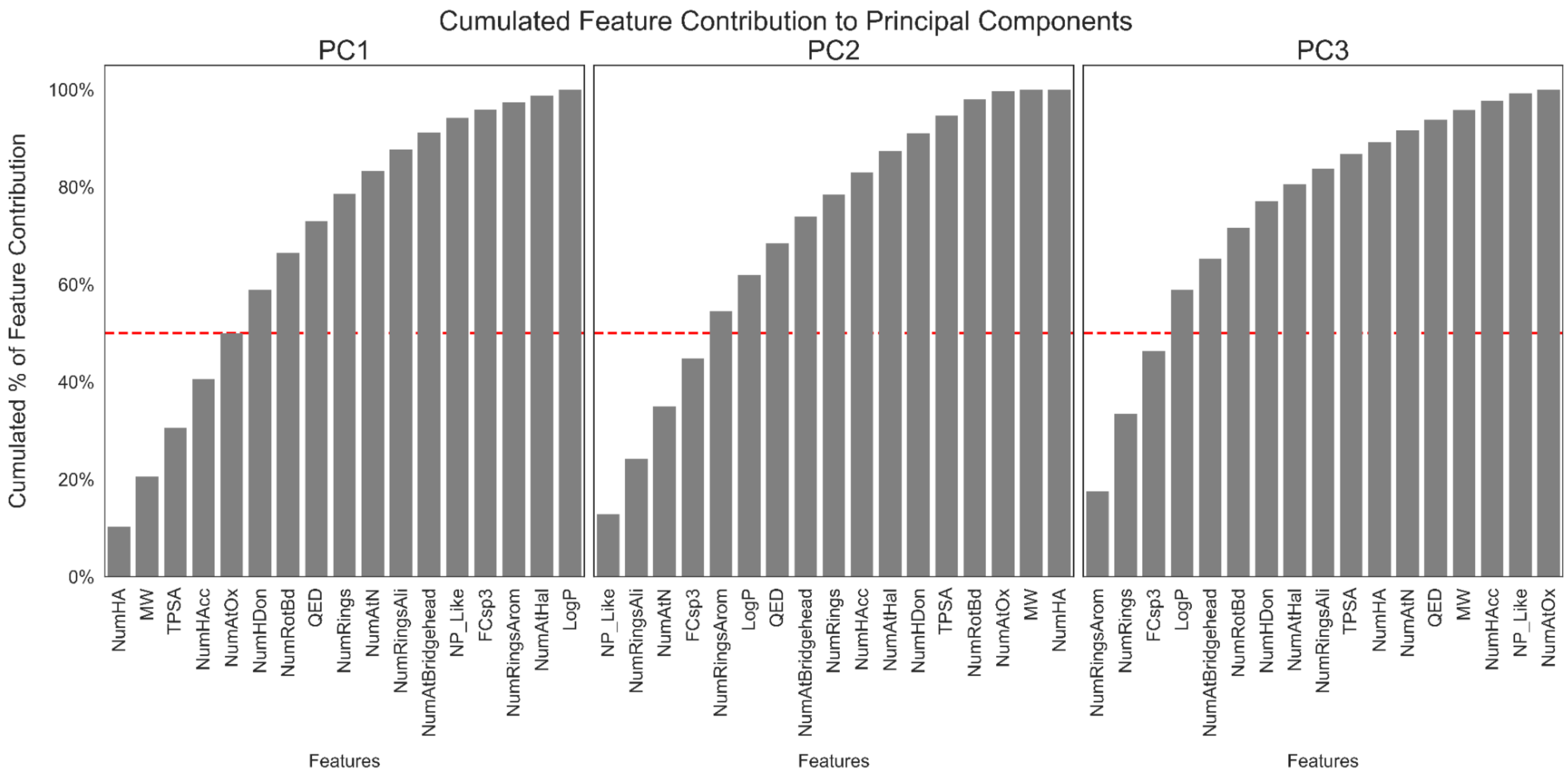

Supplementary Fig. 8: Cumulated feature contribution to principal components of the PCA of 17 molecular descriptors for Supplementary Figs. 4-6.

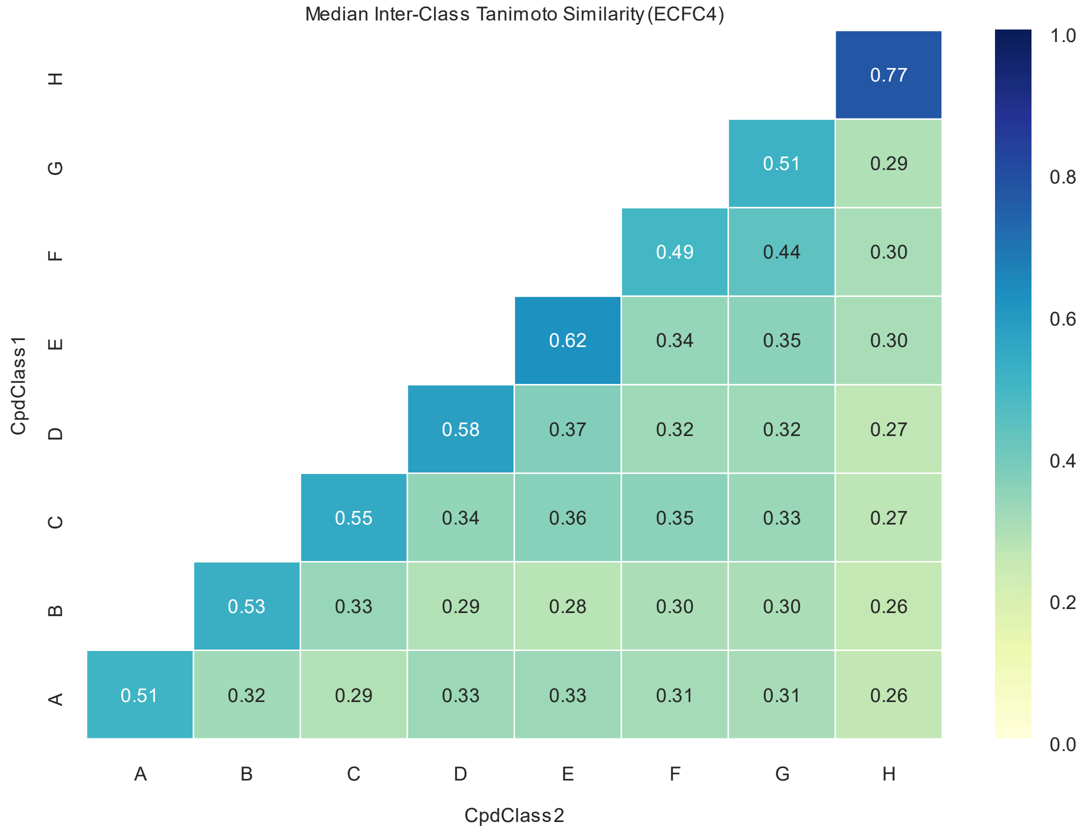

Supplementary Fig. 9: Compound class median cross-similarities for Tanimoto similarities of Morgan fingerprints (ECFC4, count fingerprint, radius 2). A similarity color scale can be seen on the right.

a)

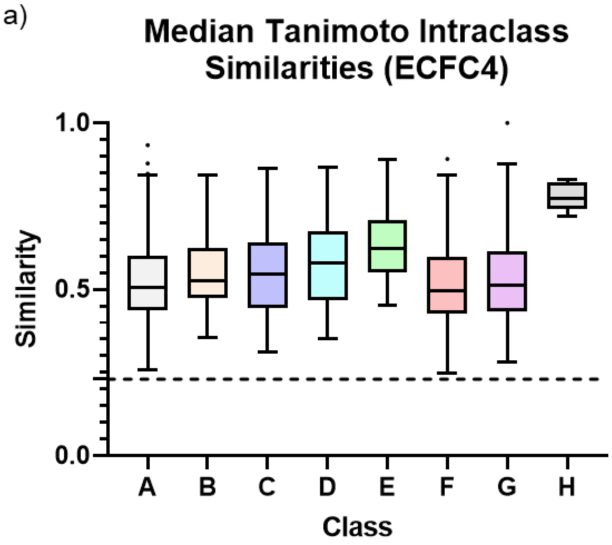

b)

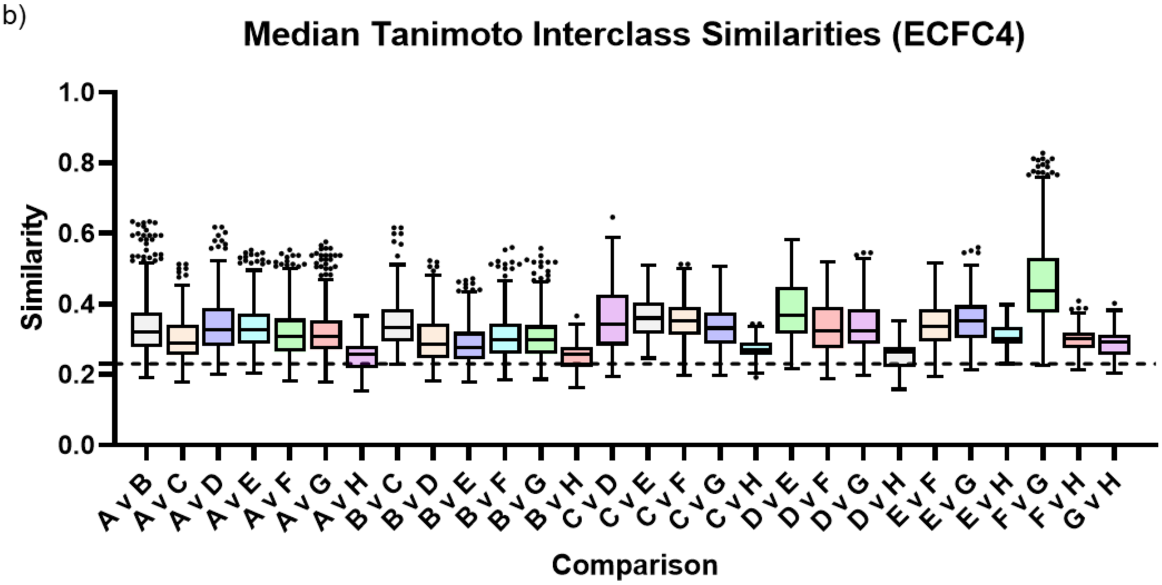

Supplementary Fig. 10: Box plots of Tanimoto similarity calculations of Morgan fingerprints (ECFC4, count fingerprint, radius 2). a) Intraclass comparisons. b) Interclass comparisons. The dashed line indicates the 95th percentile median (0.23) of random reference compound subsets. Outliers are based on Tukey's definitions[2]. Center line, median; box limits, upper and lower quartiles; whiskers, 1.5x interquartile range; points, outliers. Sample sizes are as following: Class A = 41 compounds; Class B = 18 compound; Class C = 18 compounds; Class D = 10 compounds; Class E = 19 compounds; Class F = 29 compounds; Class G = 20 compound; Class H = 4 compounds.

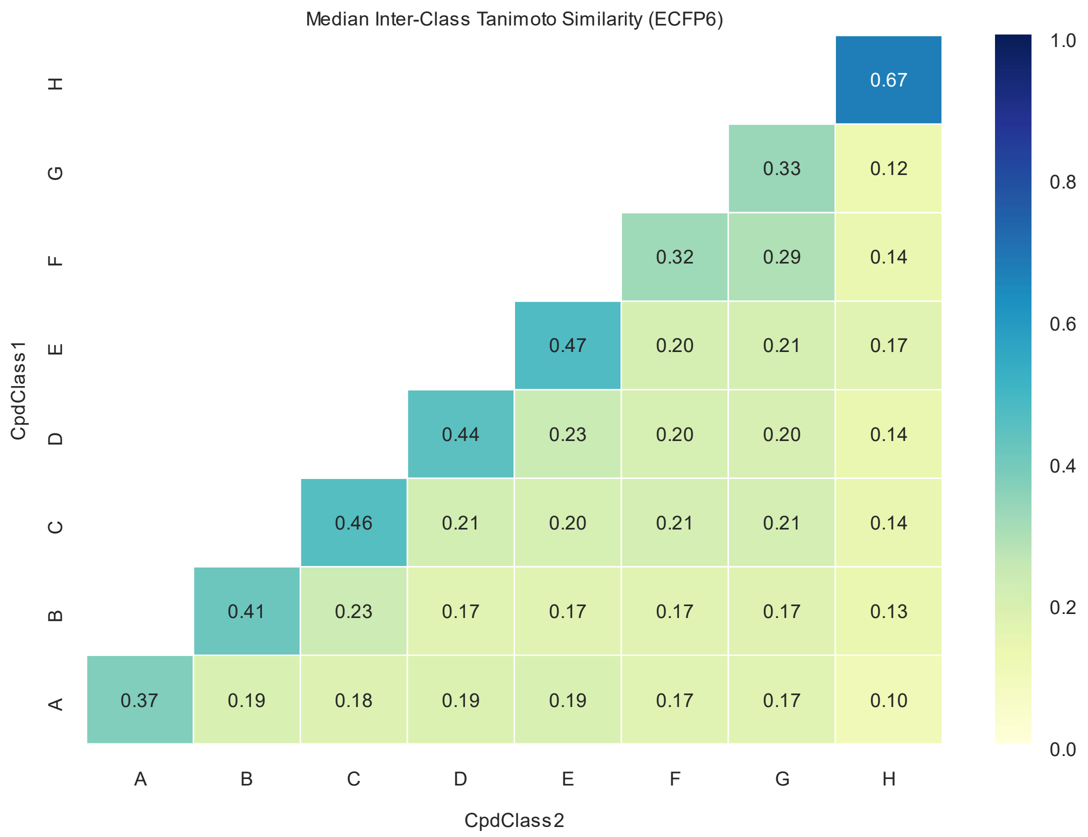

Supplementary Fig. 11: Compound class median cross-similarities for Tanimoto similarities of Morgan fingerprints (ECFP6, bit fingerprint of length 1024, radius 3). A similarity color scale can be seen on the right.

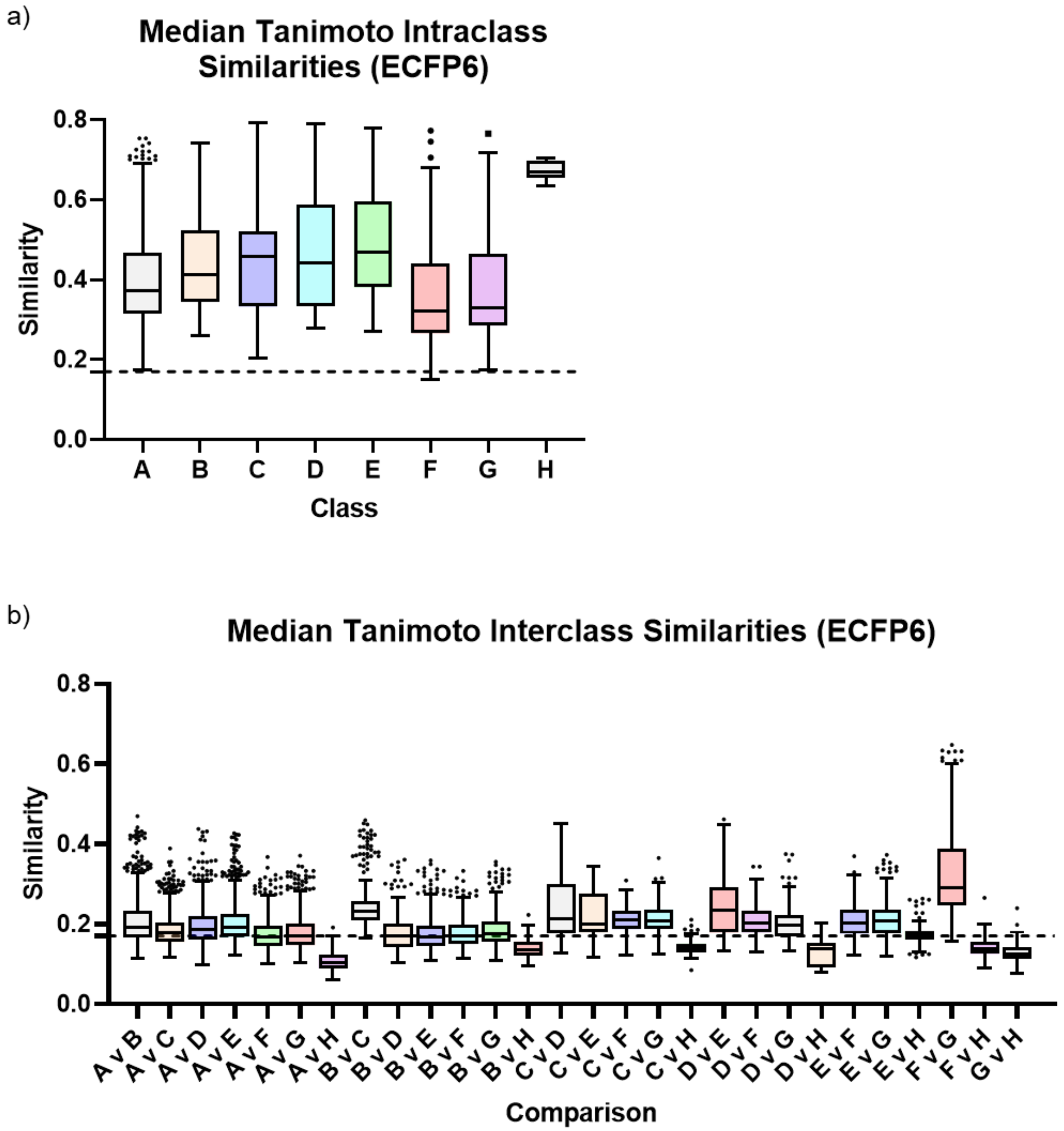

Supplementary Fig. 12: Box plots of Tanimoto similarity calculations of Morgan fingerprints (ECFP6, bit fingerprint of length 1024, radius 3). a) Intraclass comparisons. b) Interclass comparisons. The dashed line indicates the 95th percentile median (0.17) of random reference compound subsets. Outliers are based on Tukey's definitions[2]. Center line, median; box limits, upper and lower quartiles; whiskers, 1.5x interquartile range; points, outliers. Sample sizes are as following: Class A = 41 compounds; Class B = 18 compound; Class C = 18 compounds; Class D = 10 compounds; Class E = 19 compounds; Class F = 29 compounds; Class G = 20 compound; Class H = 4 compounds.

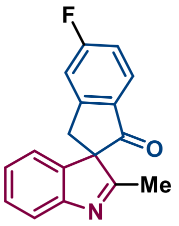

A6
IC_{50} = 26.8 ± 3.4 μM
Viability > 90%

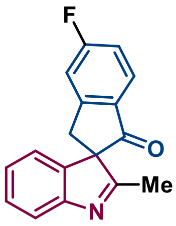

A6-resynthesized
IC_{50} > 30 μM
Viability > 90%

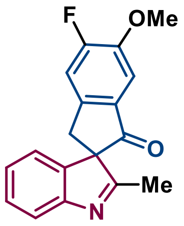

A8
IC_{50} = 7.4 ± 0.8 μm
Viability > 90%

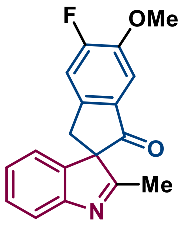

A8-resynthesized
IC_{50} = 5.1 ± 0.2 μm
Viability > 90%

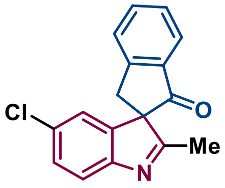

A16
IC_{50} = 4.3 ± 1.4 μM
Viability > 90%

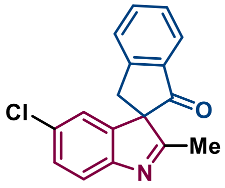

A16-resynthesized
IC_{50} = 7.3 ± 2.7 μM
Viability > 90%

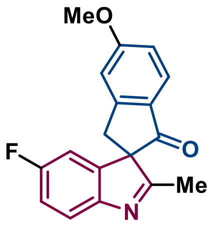

A21
IC_{50} = 22.9 ± 1.3 μM
Viability > 90%

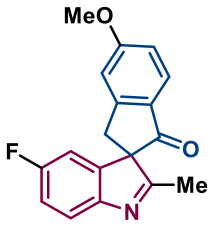

A21-resynthesized
IC_{50} = 23.7 ± 4.2 μM
Viability > 90%

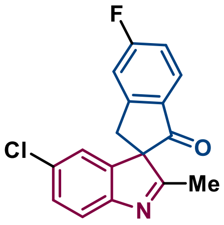

A23
IC_{50} = 0.93 ± 0.13 μM
Viability > 90%

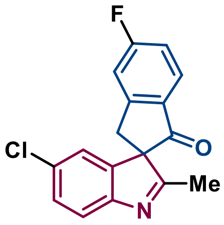

A23-resynthesized
IC_{50} = 1.4 ± 0.3 μM
Viability > 90%

Supplementary Fig. 13: Osteoblast differentiation assay IC_{50} values of selected compounds in Class A and their resynthesized batches. IC_{50} values are averages of three independent replicates ± standard deviation (*n* = 3). Cell viability was assessed using a CellTiter-Glo Luminescent Cell Viability Assay and treating C3H10T1/2 cells with compound (30 μM) in the absence of purmorphamine for 96 h. The viability of cells treated with DMSO were set to 100%.

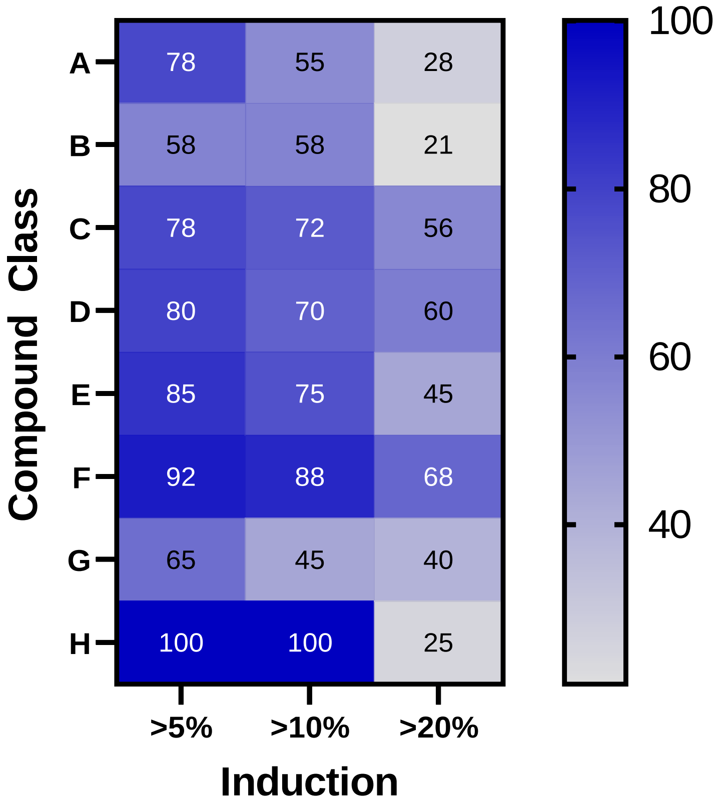

Supplementary Fig. 14: Percentage of compounds within compound classes with CPA induction values > 5, > 10, and > 20% at concentrations up to 50 μM. Values are percentages.

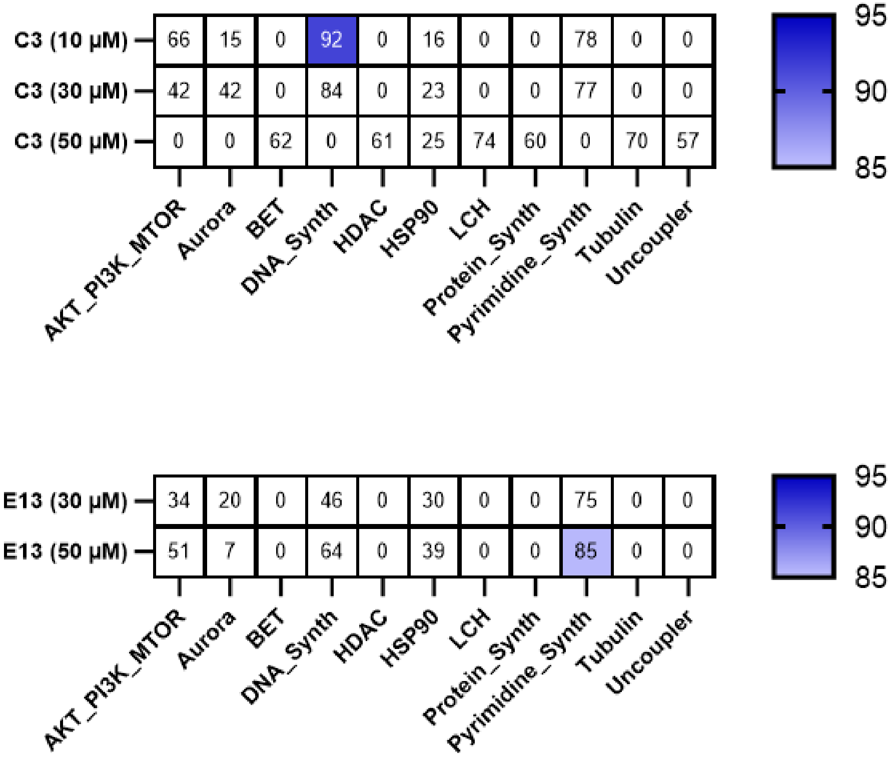

Supplementary Fig. 15: Profiles from compounds in Classes C and E that have > 85% biosimilarity to a bioactivity cluster. Only profiles with inductions > 5% are shown. The biosimilarities to bioactivity clusters are shown in percentages. LCH = lysosomotropism/cholesterol homeostasis; Pyrimidine_Synth = *de novo* pyrimidine biosynthesis.

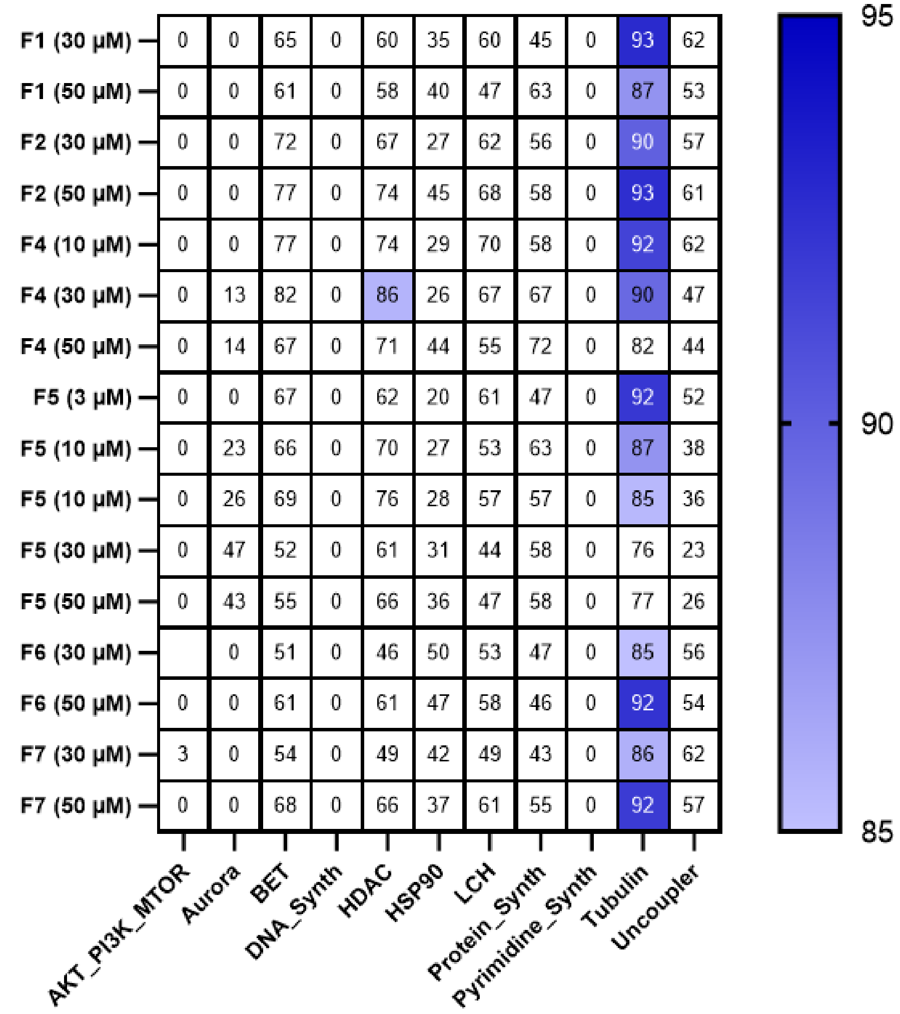

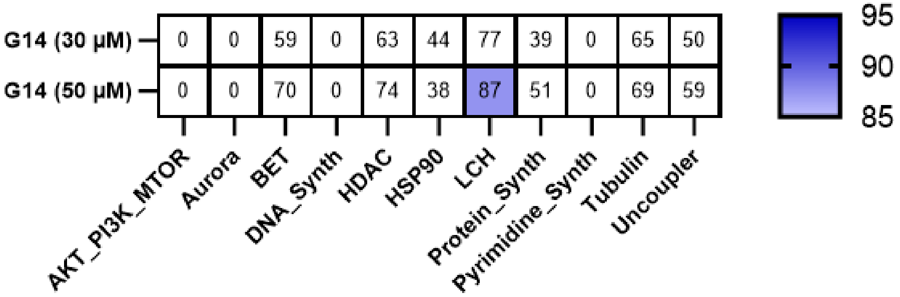

Supplementary Fig. 16: Profiles from compounds in Classes F and G that have > 85% biosimilarity to a bioactivity cluster. Only profiles with inductions > 5% are shown. The biosimilarities to bioactivity clusters are shown in percentages. LCH = lysosomotropism/cholesterol homeostasis; Pyrimidine_Synth = *de novo* pyrimidine biosynthesis.

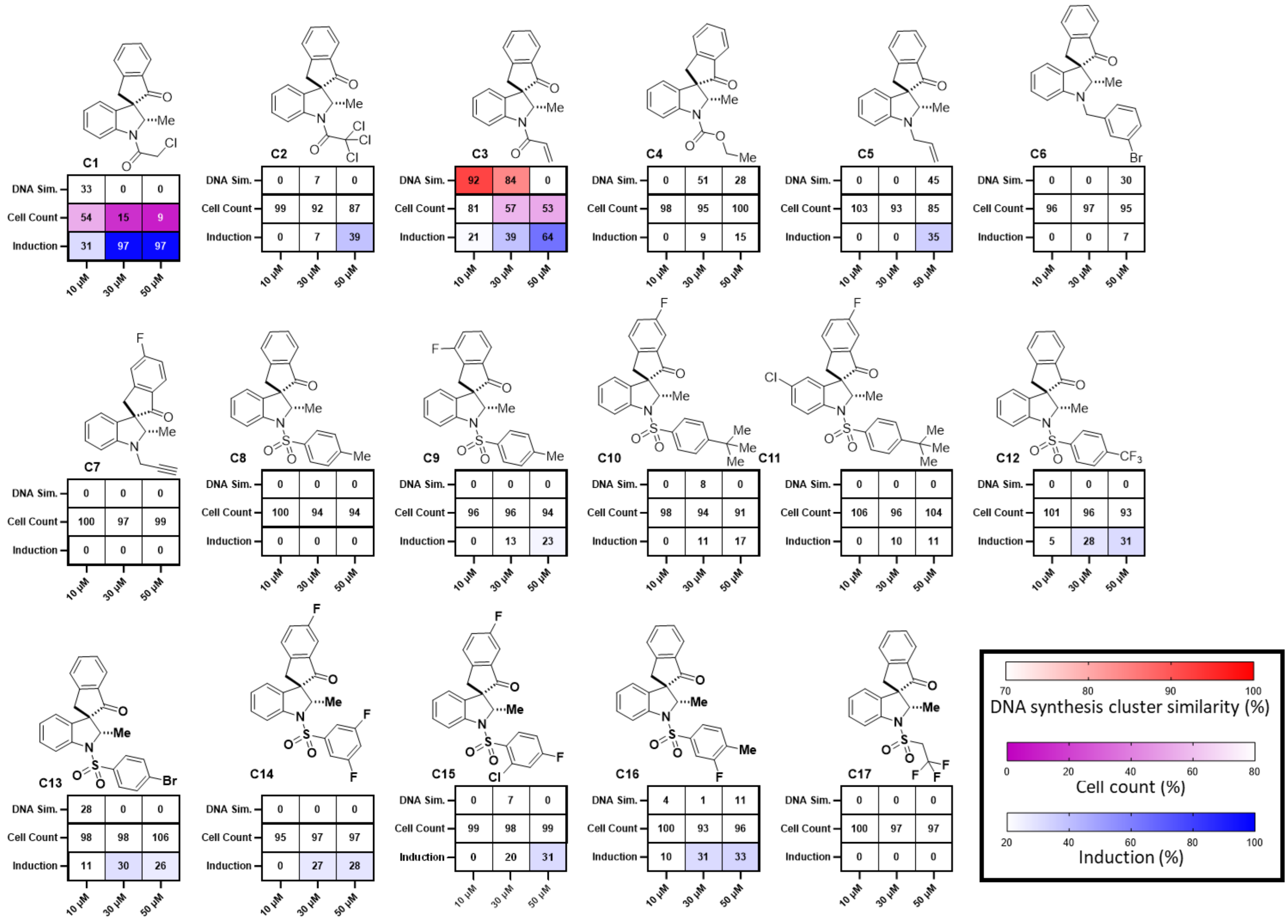

Supplementary Fig. 17. Structure-phenotype relationship of Class C compounds relative to the DNA synthesis cluster profile. DNA Sim. = percent similarity to the DNA synthesis cluster profile.

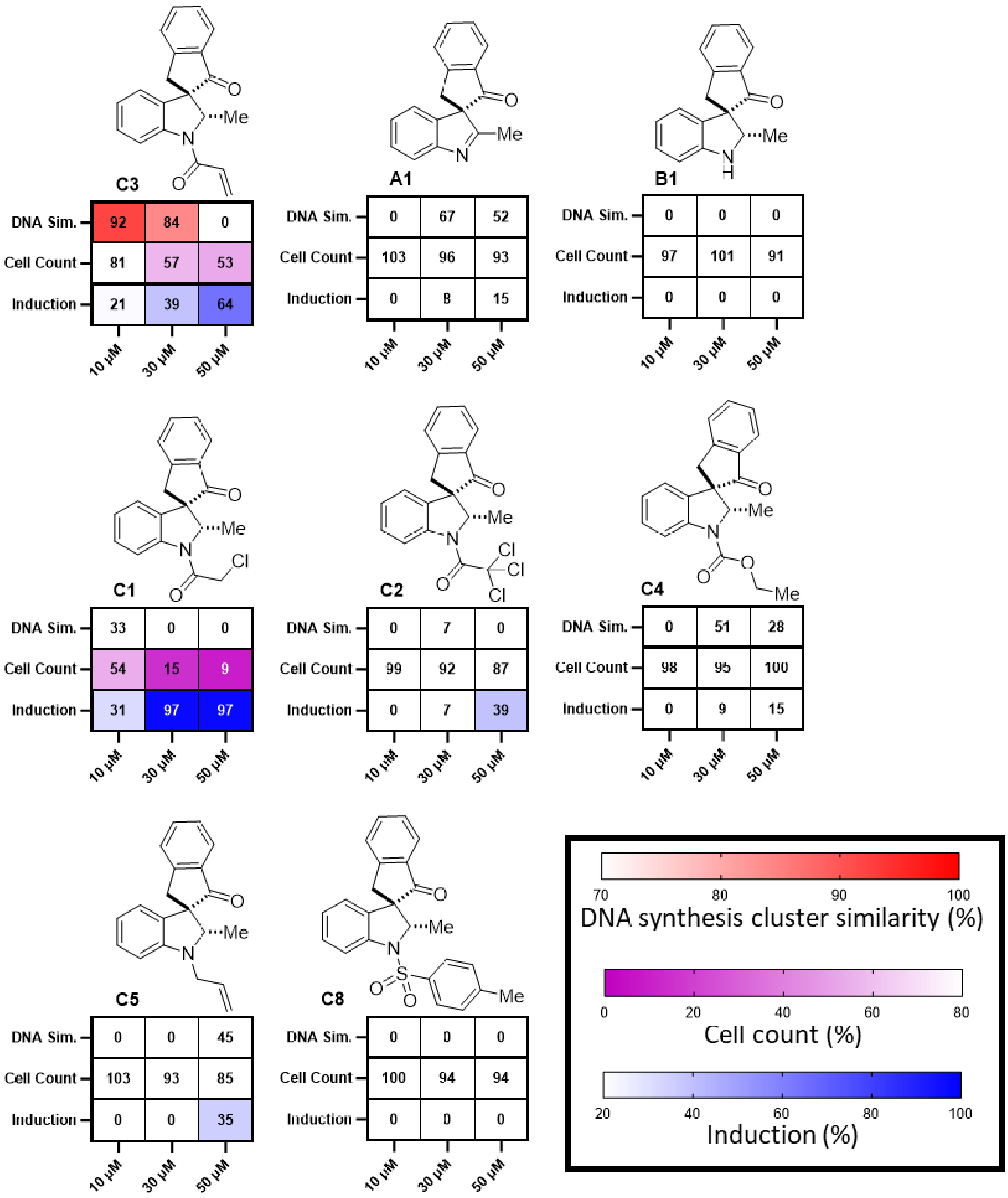

Supplementary Fig. 18. Structure-phenotype relationship of selected compounds relative to the DNA synthesis cluster profile. DNA Sim. = percent similarity to the DNA synthesis cluster profile.

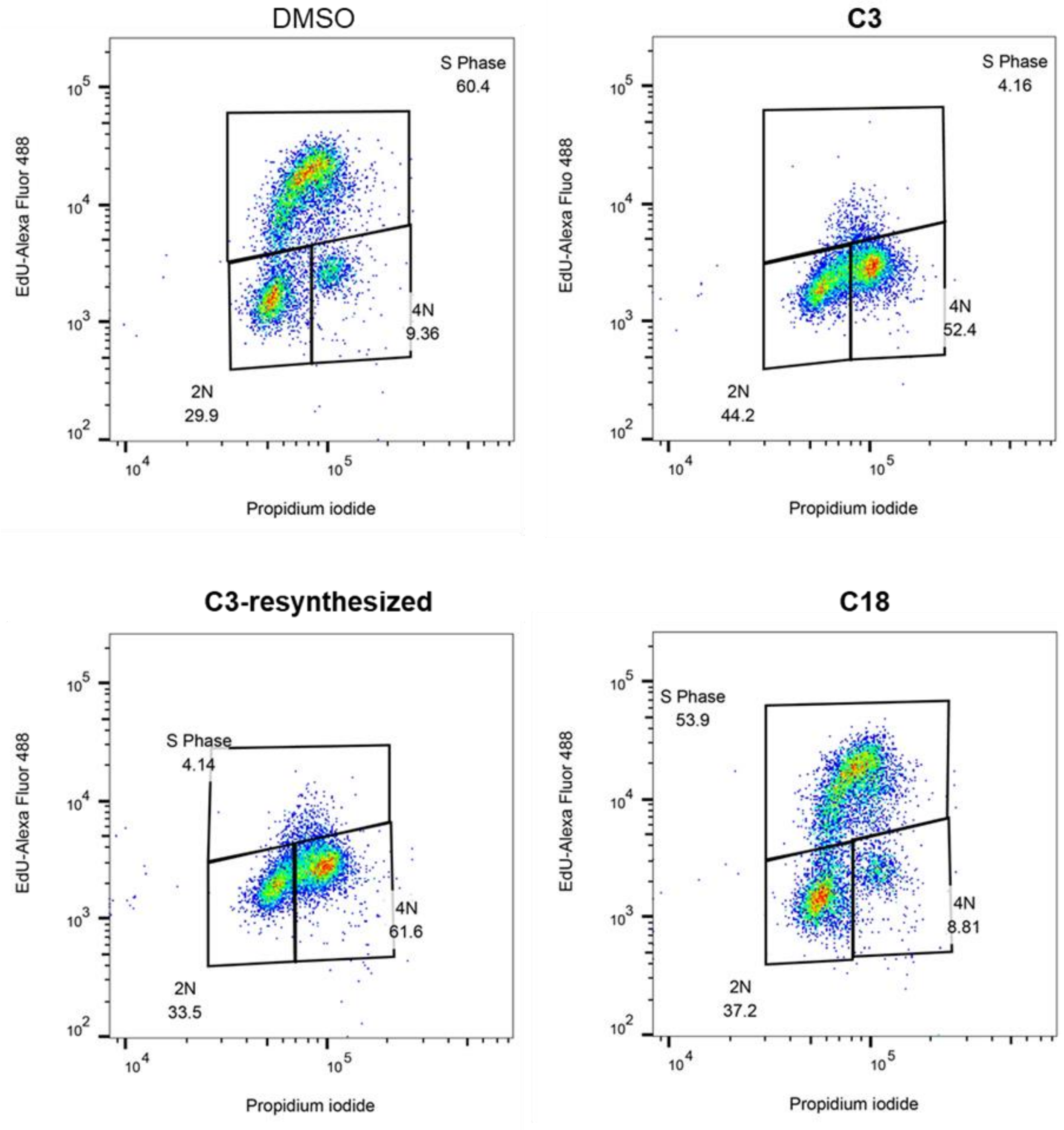

Supplementary Fig. 19. Histograms of the percentage of cells in either G1, S, or G2 phase upon compound treatment. U2OS cells were treated with DMSO or compound (30 µM) for 22 h followed by the addition of 10 µM EdU (5-ethynyl-2'-deoxyuridine) and incubated for an additional 2 h. DNA-incorporated EdU was labeled with Alexa Fluor 488 via click reaction and DNA was stained with propidium iodide. Single cell analysis via flow cytometry measuring EdU incorporation and total DNA content was used to determine the percentage of cells in either G1 (2N), S (2N-4N), or G2 (4N) phase. Data are representative of three biological replicates (n = 3).

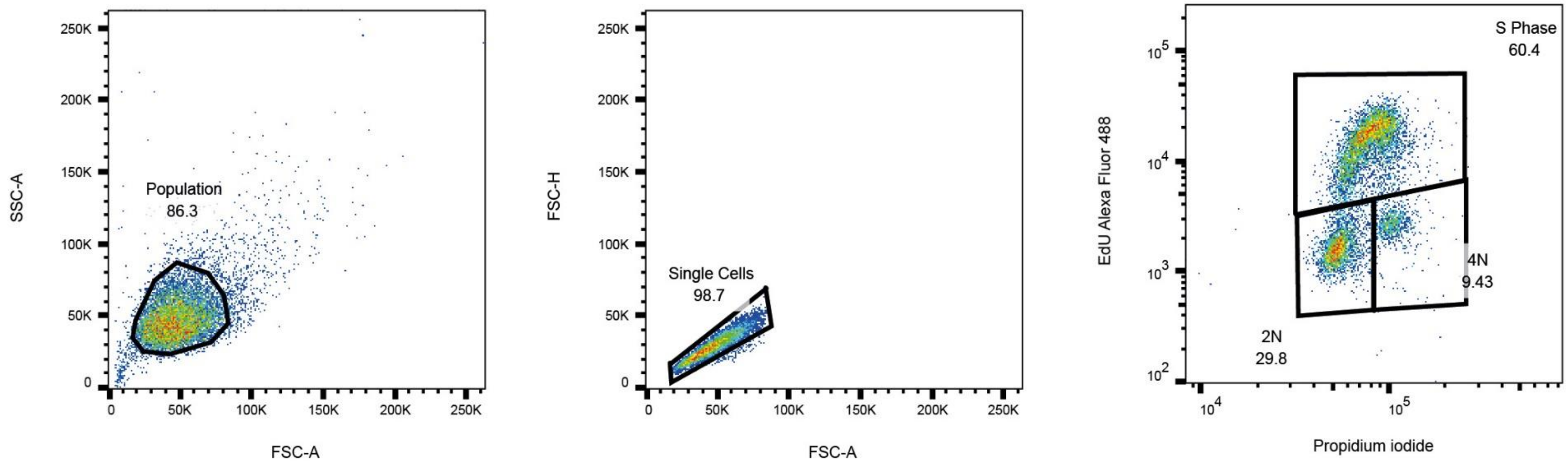

Supplementary Fig. 20. Gating strategy for flow cytometry analysis for cells that were treated with DMSO.

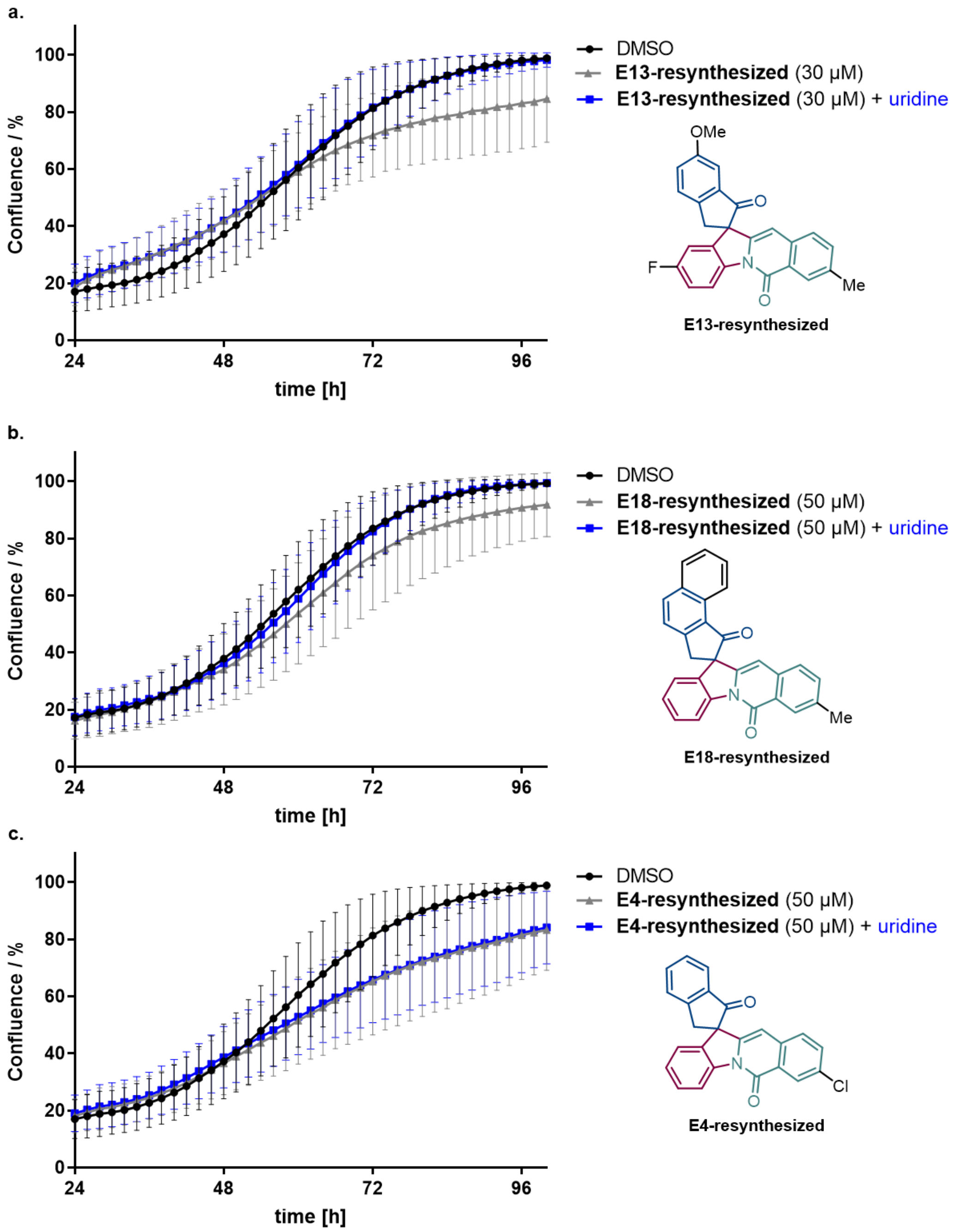

Supplementary Fig. 21. Uridine rescue assay of Class E-resynthesized compounds. HCT116 cells were treated with either DMSO (control), compound (**E13-resynthesized**, **E18-resynthesized**, or **E4-resynthesized**), or in the presence of uridine (100 µM). Cell confluence was used as a measure of cell proliferation and was monitored over a 96 h period using an IncuCyte ZOOM/S3. Data are mean values ± SD of three independent replicates (*n* = 3).

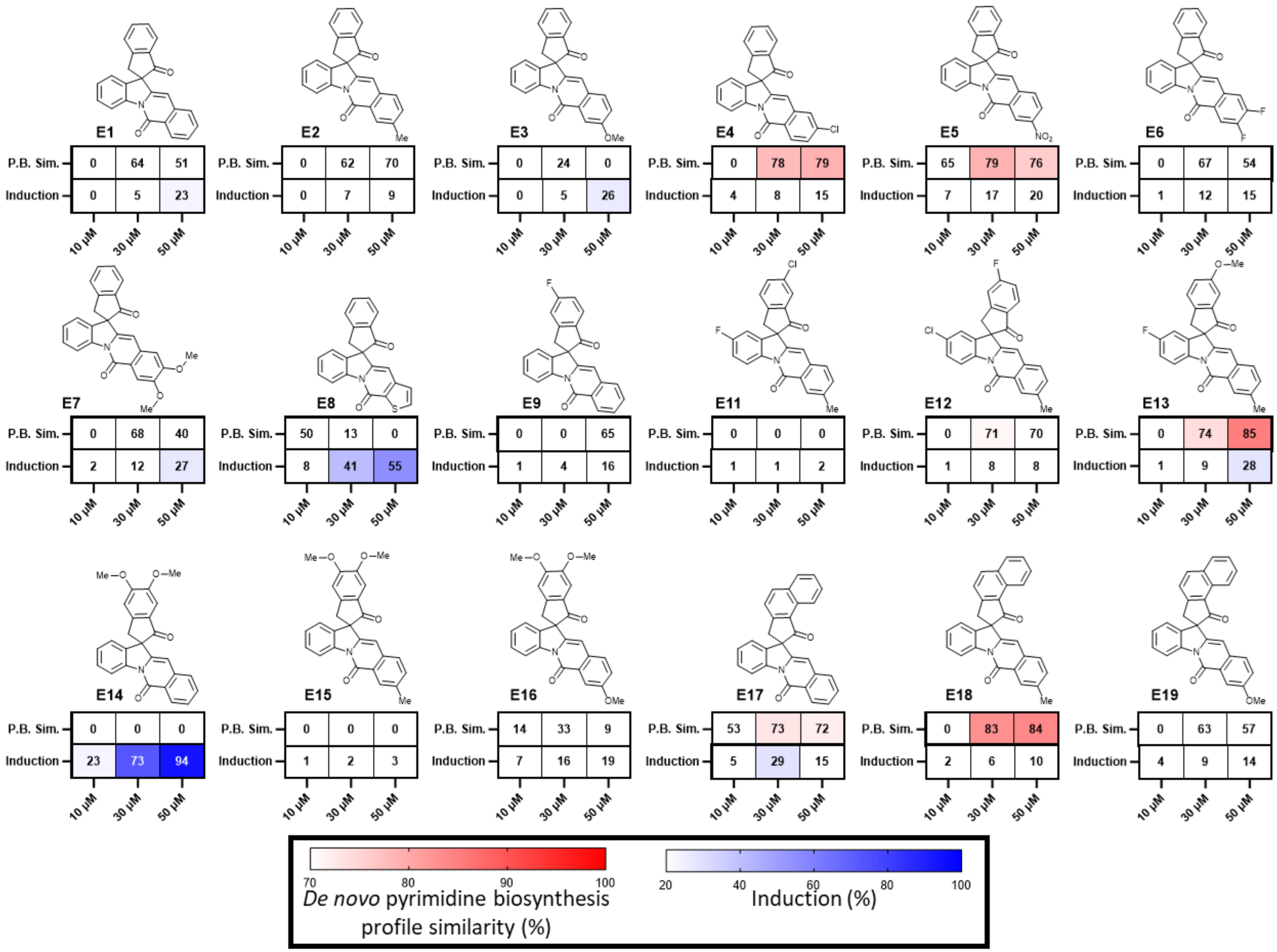

Supplementary Fig. 22. Structure-phenotype relationship of Class E compounds relative to the de novo pyrimidine biosynthesis cluster profile. P.B. Sim. = percent similarity to the *de novo* pyrimidine biosynthesis cluster profile.

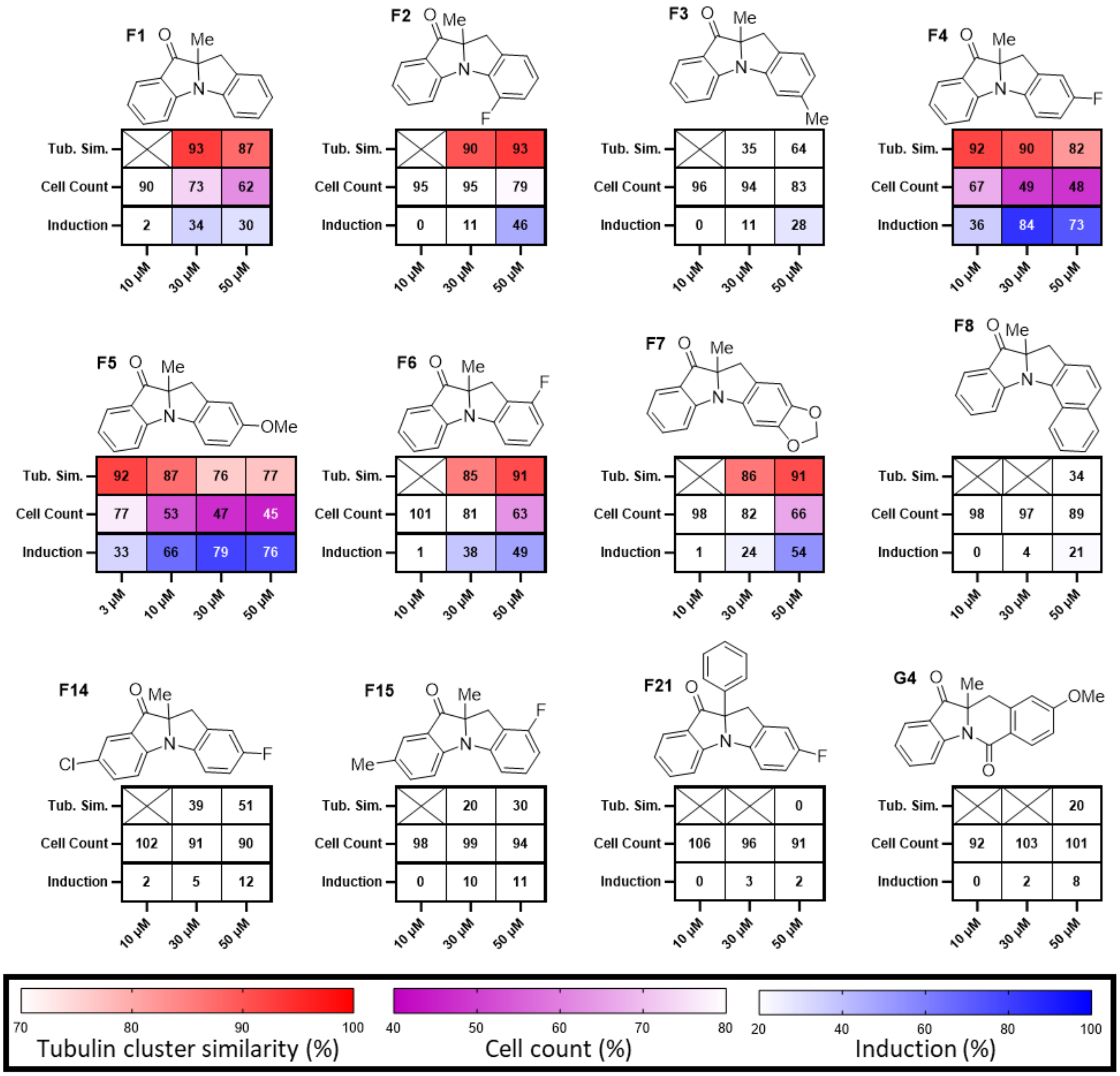

Supplementary Fig. 23: Structure-phenotype relationship study of selected Class F compounds relative to the tubulin cluster profile. Tub. Sim. = Tubulin cluster profile similarity. Tubulin cluster profile similarity, cell count, and induction are reported in percent. For profiles that are CPA inactive, i.e. induction < 5%, a tubulin cluster profile similarity is not reported.

Compound	BioSim [%]	Ind [%]	Cell Count [%]	Conc [µM]
**F5**	--	33	77	3
Rotenone	91	36	77	0.3
TCS JNK 5a	89	38	77	0.2
7-Chloro-4-hydroxy-2-phenyl-1,8-naphthyridine	87	61	63	30
Flubendazole	87	28	69	30
TP-472	87	64	72	10
Anlotinib dihydrochloride	86	53	85	2
Oxfendazole	86	57	66	30
Albendazole	85	66	58	1
Nocodazole	85	61	69	0.1
2-Methoxyestradiol	85	66	72	2
Mebendazole	84	51	71	0.6

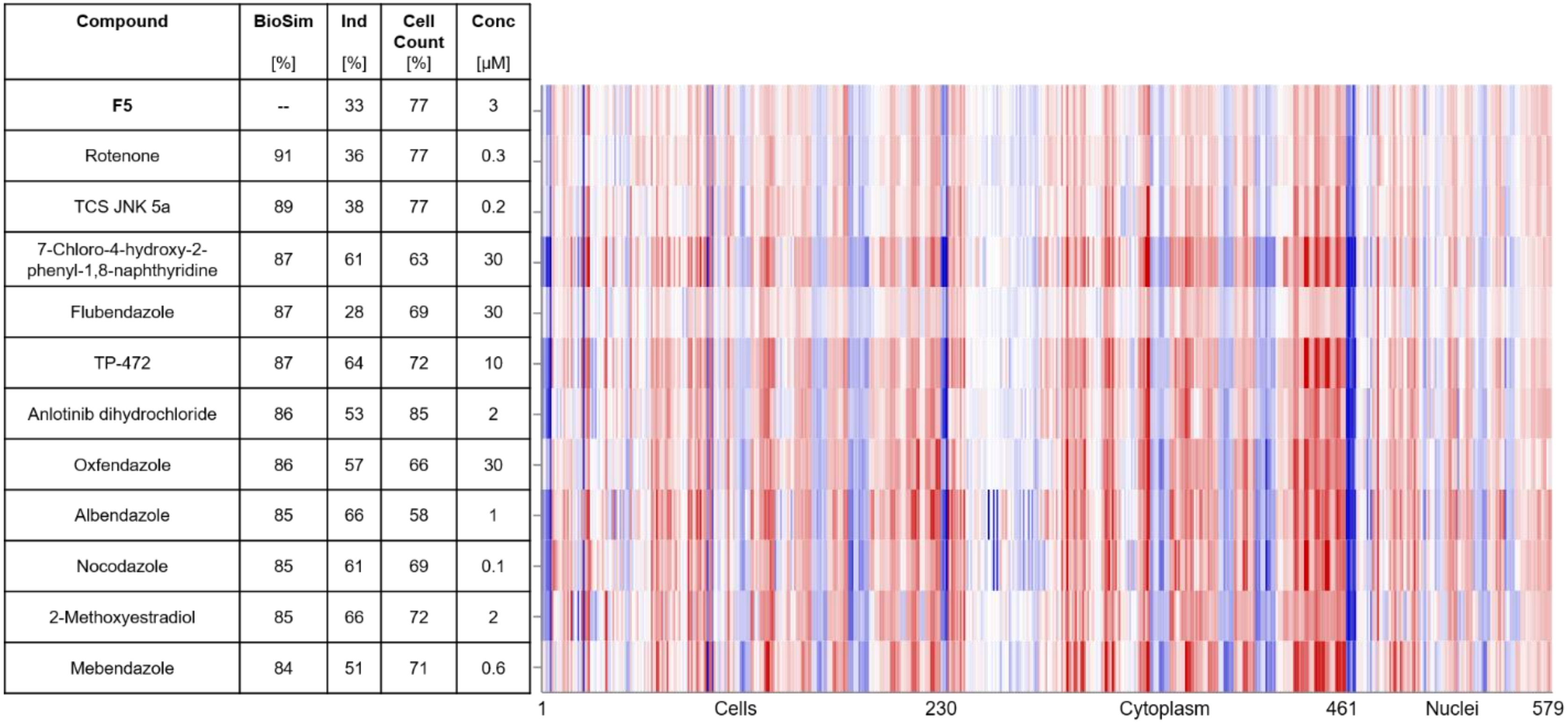

Supplementary Fig. 24: Similarity search of the full profile of F5 at 3 µM compared to reference compounds visualized as heatmaps. Of the first 12 profiles found in the similarity search, 11 are of compounds that are in the defined tubulin bioactivity cluster[3]. These 11 profiles are shown. The first profile is set as the reference profile (100% biosimilarity) for which the following profiles are compared. The 597 morphological features are divided in to features related to the cell (1-229), cytoplasm (230-461), and the nuclei (462-579). Blue color represents decreased feature while the red color represents an increased parameter. The magnitude of feature change relative to DMSO is represented by the intensity of either blue or red. BioSim = Biosimilarity; Ind = Induction; Conc = Concentration

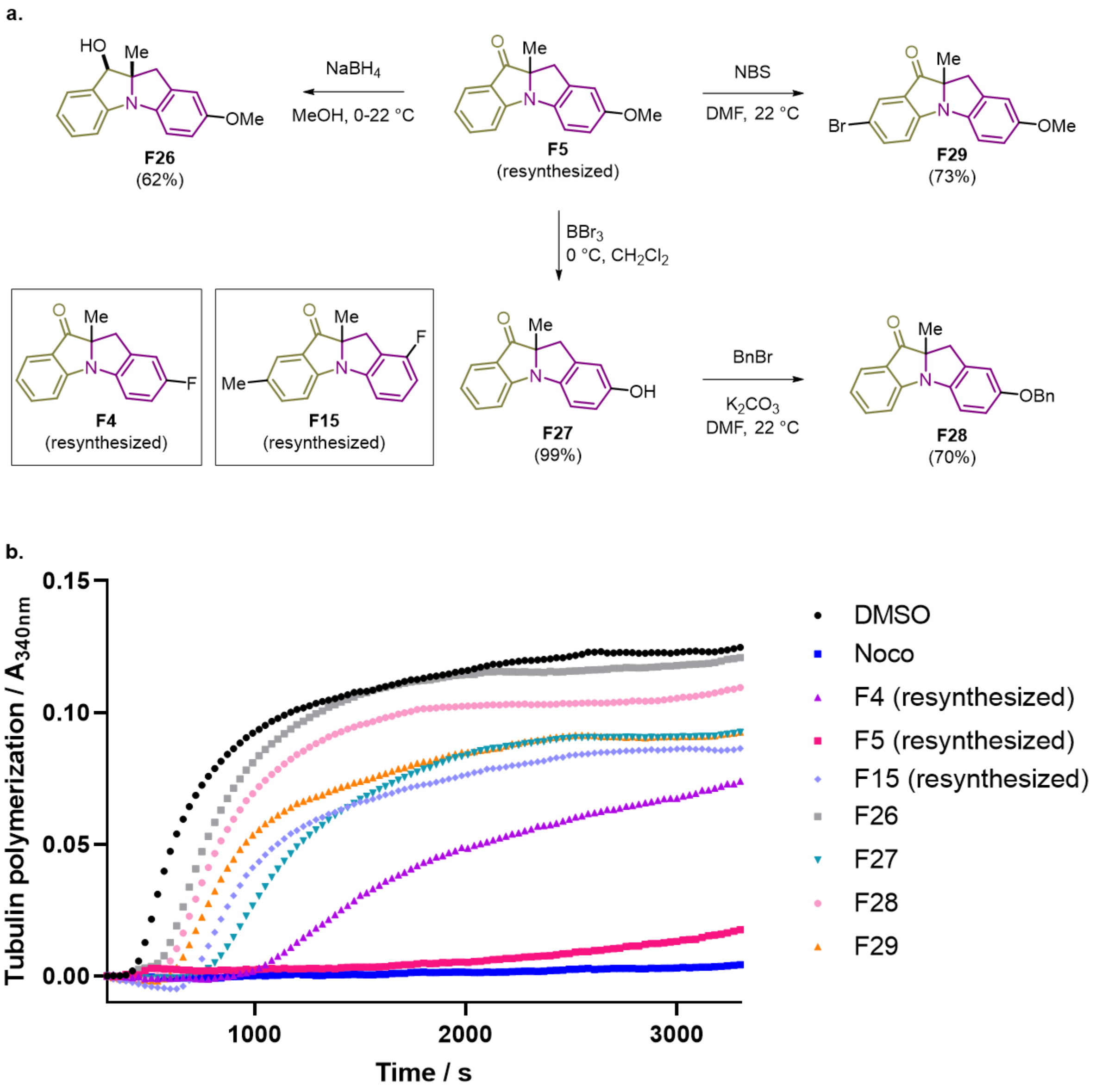

Supplementary Fig. 25: Derivatization of F5 and biological evaluation. a Synthesis of derivatives **F26-F29**. **b** *In vitro* tubulin polymerization assay. DMSO was used as a negative control and Nocodazole (0.3 µM) was used as a positive control for tubulin destabilization. All compounds **F** were assayed at a concentration of 20 µM. Data are representative of three independent experiments (n = 3). Compounds **F4**, **F5**, and **F15** were resynthesized and are independent from the batches evaluated in the CPA and in Fig. 7.

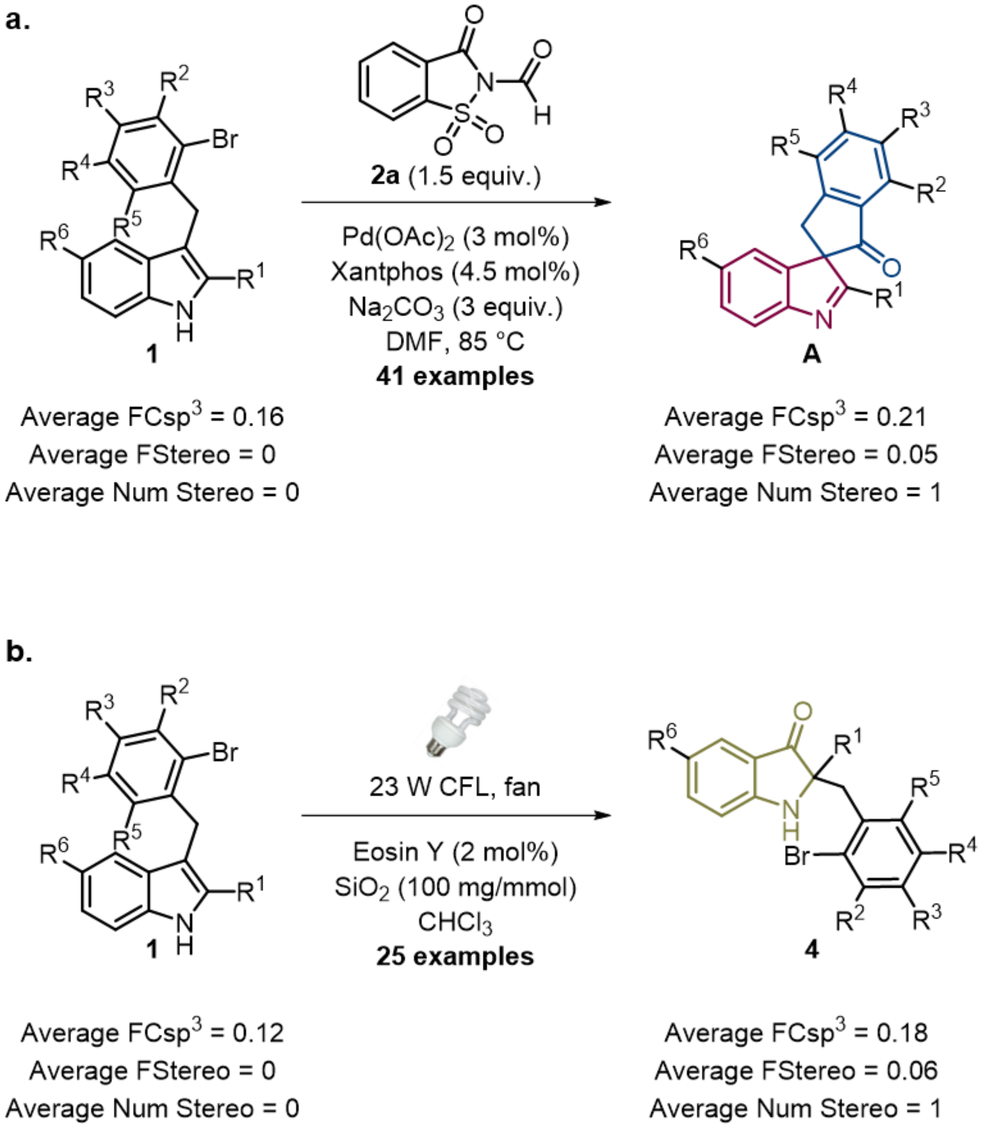

Supplementary Fig. 26. Analysis of the impact of dearomatization reactions on FCsp^3, FStereo, and Num Stereo. **a** Palladium-catalyzed intramolecular carbonylation/indole dearomatization cascade of substrates **1** to form **Class A**. **b** Photocatalyzed oxidative semipinacolic rearrangement of substrates **1** to form dearomatized products **4**. FCsp^3 = (number of sp^3-hybridized carbons / total number of carbons); FStereo = (number of carbon stereocenters / total number of carbons); Num Stereo = number of carbon stereocenters.

Cheminformatic Analysis

The full code and data sets used in the study are available in the online repository: https://zenodo.org/records/8320827.

Determination of a structural randomness threshold:

One hundred subsets from the Enamine Advanced Screening Library with 100 members each were randomly selected. Intra-subset Tanimoto similarities of the Morgan fingerprints of two different designs (ECFC4, count fingerprint, radius 2 and ECFP6, bit fingerprint of length 1024, radius 3) were calculated and the median, 5th percentile, and 95th percentile of each subset was determined. From these intra-subset values, inter-subset medians, 5th percentile medians, and 95th percentile medians were determined.

	**Median**	**5th Percentile**	**95th Percentile**
**Fingerprint**			
ECFC4	0.144	0.069	0.232
ECFP6	0.111	0.062	0.168

Details of data sets used in Fig. 5 ("Cheminformatic analyses…")[in Manuscript]

All downloaded data sets were standardized and deduplicated using this script:
https://github.com/apahl/jupy_tools/blob/main/python_scripts/stand_struct.py

Enamine Advanced Screening Collection (527411 compounds) was downloaded from https://enamine.net/hit-finding/compound-collections/screening-collection/advanced-collection on 07-Dec-2020. 526897 compounds remained after standardization. For the PMI calculations, a random subset of 50k compounds was used.

Drugbank approved and investigational drugs, v. 5.1.8, were downloaded and combined. 4866 compounds remained after standardization.

ChEMBL Natural Products were obtained by downloading the SD file and the SQLite version of ChEMBL v30 from the FTP server (https://ftp.ebi.ac.uk/pub/databases/chembl/ChEMBLdb/releases/chembl_30/), executing the standardization on the SD file using stand_struct.py again and running this script to extract the NPs: https://github.com/apahl/jupy_tools/blob/main/python_scripts/extract_nps_from_sqlite.py. This script also applies a deglycosylation step to the structures. 45679 compounds were identified.

Calculation of molecular descriptors, PCA, and PMI

The full code for molecular descriptors, PCA, and PMI are included in the accompanying repository (https://zenodo.org/records/8320827). For all descriptors, the RDKit was used (v2022.03.5) and is listed in the "environment.yml" file of the repository.

For PMI value calculations, compounds with more than one stereocenter were omitted from the calculation, when they had 1 or more centers of undefined stereochemistry.

Biological Experimental Details

General Biological Experimental Details

Cell lines

The C3H10T1/2 cell line (ATCC, CCL-226) are murine mesenchymal stem cells. The cells were cultured in Dulbecco's Modified Eagle's medium (DMEM with 4.5 g/L glucose, L-glutamine and 3.7 g/L sodium bicarbonate; PAN Biotech, #P04-03550) supplemented with 10% of Fetal Bovine Serum Australian Origin (FBS, CellSera Australia #AU-FBS/PG, heat inactivated), 1 mM sodium pyruvate (PAN, #P04-43100). U2OS and HCT116 cells were cultured in Dulbecco's modified eagle medium (DMEM) (PAN Biotech, cat# P04-03550) with the addition of 10% fetal bovine serum (FBS) (Invitrogen, cat# 10500-084), sodium pyruvate (PAN Biotech, cat# P04-43100), nonessential amino acids (PAN Biotech, cat# P08-32100).

Cells were maintained at 37 °C and 5% CO_2 in a humidified atmosphere. Mycoplasma contaminations were checked on a regular basis, and cells were found to be free of contamination.

Cell Painting Assay

The described assay follows closely the method described by Bray et al[4]. Initially, 5 µl U2OS medium were added to each well of a 384-well plate (PerkinElmer CellCarrier-384 Ultra). Subsequently, U2OS cell were seeded with a density of 1600 cells per well in 20 µl medium. The plate was incubated for 10 min at the ambient temperature, followed by an additional 4 h incubation (37 °C, 5% CO2). Compound treatment was performed with the Echo 520 acoustic dispenser (Labcyte) at final concentrations of 10 µM, 3 µM or 1 µM. Incubation with compound was performed for 20 h (37 °C, 5% CO2). Subsequently, mitochondria were stained with Mito Tracker Deep Red (Thermo Fisher Scientific, Cat. No. M22426). The Mito Tracker Deep Red stock solution (1 mM) was diluted to a final concentration of 100 nM in prewarmed medium. The medium was removed from the plate leaving 10 µl residual volume and 25 µl of the Mito Tracker solution were added to each well. The plate was incubated for 30 min in darkness (37 °C, 5% CO2). To fix the cells 7 µl of 18.5 % formaldehyde in PBS were added, resulting in a final formaldehyde concentration of 3.7 %. Subsequently, the plate was incubated for another 20 min in darkness (RT) and washed three times with 70 µl of PBS. (Biotek Washer Elx405). Cells were permeabilized by addition of 25 µl 0.1% Triton X-100 to each well, followed by 15 min incubation (RT) in darkness. The cells were washed three times with PBS leaving a final volume of 10 µl. To each well 25 µl of a staining solution were added, which contains 1% BSA, 5 µl/ml Phalloidin (Alexa594 conjugate, Thermo Fisher Scientific, A12381), 25 µg/ml Concanavalin A (Alexa488 conjugate, Thermo Fisher Scientific, Cat. No. C11252), 5 µg/ml Hoechst 33342 (Sigma, Cat. No. B2261-25mg), 1.5 µg/ml WGA-Alexa594 conjugate (Thermo Fisher Scientific, Cat. No. W11262) and 1.5 µM SYTO 14 solution (Thermo Fisher Scientific, Cat. No. S7576). The plate is incubated for 30 min (RT) in darkness and washed three times with 70 µl PBS. After the final washing step, the PBS was not aspirated. The plates were sealed and centrifuged for 1 min at 50 x g.

The plates were prepared in triplicates with shifted layouts to reduce plate effects and imaged using a Micro XL High-Content Screening System (Molecular Devices) in 5 channels (DAPI: Ex350-400/ Em410-480; FITC: Ex470-500/ Em510-540; Spectrum Gold: Ex520-545/ Em560-585; TxRed: Ex535-585/ Em600-650; Cy5: Ex605-650/ Em670-715) with 9 sites per well and 20x magnification (binning 2).

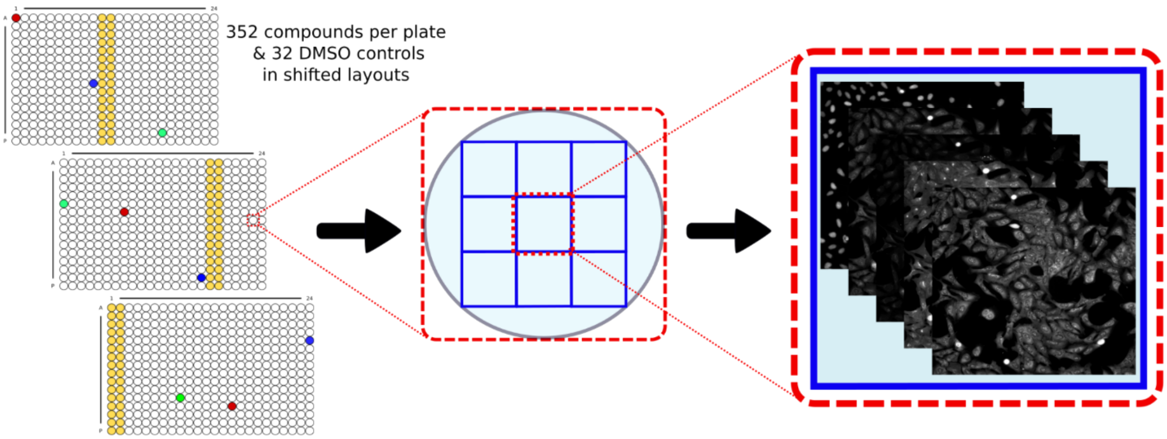

The generated images were processed with the *CellProfiler* package (https://cellprofiler.org/, version 3.0.0) on a computing cluster of the Max Planck Society to extract 1716 cell features per microscope site. The data was then further aggregated as medians per well (9 sites -> 1 well), then over the three replicates.
Further analysis was performed with custom *Python* (https://www.python.org/) scripts using the *Pandas* (https://pandas.pydata.org/) and *Dask* (https://dask.org/) data processing libraries as well as the *Scientific Python* (https://scipy.org/) package (separate publication to follow).

From the total set of 1716 features, a subset of highly reproducible and robust features was determined using the procedure described by Woehrmann et al[5]. in the following way: Two biological repeats of one plate containing reference compounds were analysed. For every feature, its full profile over each whole plate was calculated. If the profiles from the two repeats showed a similarity >= 0.8 (see below), the feature was added to the set.

This procedure was only performed once and resulted in a set of 579 robust features out of the total of 1716 that was used for all further analyses.

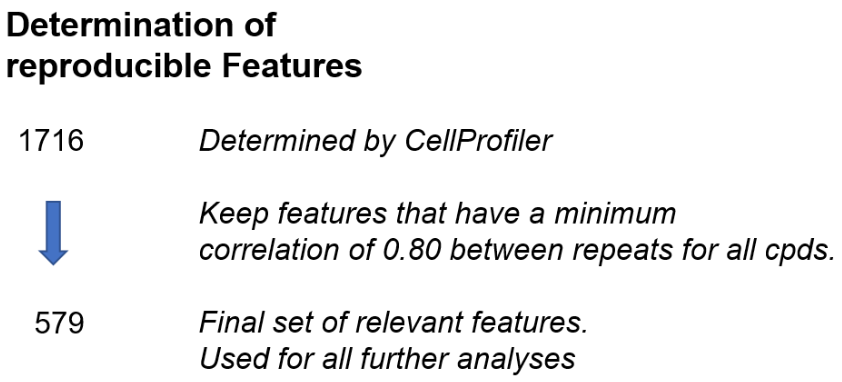

The phenotypic profiles were compiled from the Z-scores of all individual cellular features, where the Z-score is a measure of how far away a data point is from a median value.

Specifically, Z-scores of test compounds were calculated relative to the Median of DMSO controls. Thus, the Z-score of a test compound defines how many MADs (Median Absolute Deviations) the measured value is away from the Median of the controls as illustrated by the following formula:

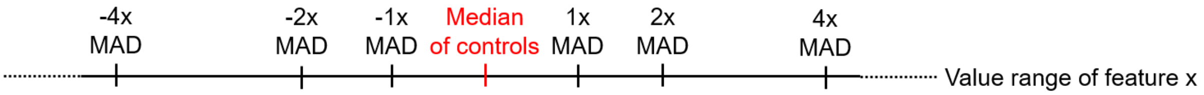

$$z\_score = \frac{value_{meas.} - Median_{Controls}}{MAD_{Controls}}$$

The phenotypic compound profile is then determined as the list of Z-scores of all features for one compound.

In addition to the phenotypic profile, an induction value was determined for each compound as the fraction of significantly changed features, in percent:

$$Induction\ [\%] = \frac{number\ of\ features\ with\ abs.\ values > 3}{total\ number\ of\ features}$$

Similarities of phenotypic profiles were calculated from the correlation distances between two profiles (https://docs.scipy.org/doc/scipy/reference/generated/scipy.spatial.distance.correlation.html; Similarity = 1 - Correlation Distance).

An example for two compounds with highly similar profiles (96% similarity):

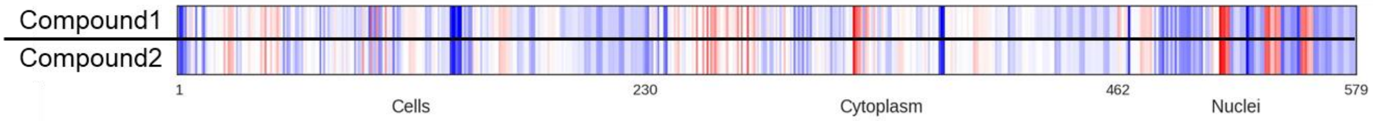

An example for two compounds with low similarity profiles (0% similarity):

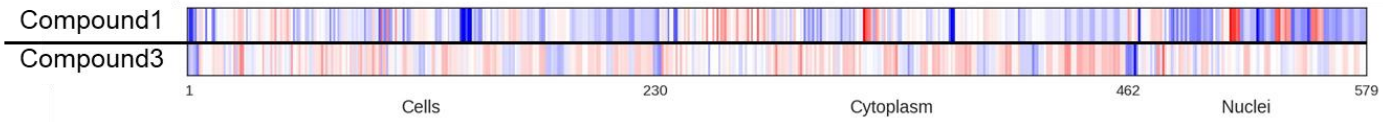

Each colored band represents one Z-score of a feature.

Hedgehog-Dependent Osteoblast Differentiation Assay

For the assay measuring the signal transduction through the Hedgehog (Hh) pathway, mouse embryonic mesoderm fibroblast C3H10T1/2 cells were employed. These multipotent mesenchymal progenitor cells differentiate into osteoblasts upon treatment with the Purmorphamine, antagonist of the protein Smoothened (SMO). During differentiation, osteoblast-specific genes, such as alkaline phosphatase (*Alpl*), are highly expressed. The activity of alkaline phosphatase can directly be monitored by following substrate hydrolysis that yields a highly luminescent product. Inhibition of the pathway leads to the reduction of luminescence and can be used to identify chemical inhibitors.

The medium-throughput evaluation of potential inhibitors of the Hh pathway was performed by the Compound Management and Screening Center in Dortmund, Germany in 384-well format. In short, 800 cells per well were seeded in 25 µl medium (high glucose DMEM, 10% heat inactivated FBS, 1 mM sodium pyruvate, 6 mM L-glutamine, 100 U/ml penicillin and 0.1 mg/ml streptomycin) and were grown overnight. The next day, the compounds were added to arrive at a final concentration of 10 µM using an acoustic nanoliter dispenser ECHO 520 (Beckman). After one hour, 10 µl of Purmorphamine in medium was added to afford a final concentration of 1.5 µM using a Multidrop Combi (Thermofisher Scientific); the control cells were not treated with Purmorphamine. After 96 h, the cell culture medium was aspirated using the aspiration function of the Elx405 cell washer (Biotek) and 25 µl of a commercial luminogenic alkaline phosphatase substrate (CDP-Star, Roche) was added. After one hour, the luminescence was read out.

Cell viability measurements were carried out in parallel to identify toxic compounds which also result in a reduction in the luminescent signal. The cell viability assay followed the same workflow as the Hh assay, except that only 200 cells per well were seeded. Cell culture medium alone served as control for the cell viability assay. For the measurement of cell viability, 15 µl of CellTiterGlo reagent (Promega), which determines the cellular ATP content, were added after aspiration of the medium.

Hit compounds were those that have at least a 50% reduction in the luminescent signal in the Hh assay at 10 µM and are above the minimum threshold of cell viability of 80%. Dose-response

analysis for hit compounds was done using a three-fold dilution curve starting from 10 µM. IC_{50} values were calculated using the Quattro software suite (Quattro Research GmbH).

In a low-throughput manner, the Hh-dependent osteoblast differentiation activity was determined by adopting the previously described procedure reported by Wu et al[6]. In short, 6000 C3H10T1/2 cells per well were seeded in white 96-well plates with a clear flat bottom (Greiner Bio-One, # 655098) and incubated in 5% CO_2 at 37 °C for 16 h. Next, the cells were treated with 1.5 µM purmorphamine (Cayman Chemical #10009634) and different concentrations of the compounds or DMSO (<0.5%) as a control. The plate was sealed with a gas permeable membrane and incubated in 5% CO_2 at 37 °C. After four days, the cell culture medium was aspirated and 50 µL per well lysis buffer (100mM Tris pH 9.5, 250 mM NaCl, 25 mM $MgCl_2$ and 1% Triton X-100) containing luminogenic alkaline phosphatase substrate CDP-Star (Roche, #11685627001) 1:100 dilution was added and incubated for 1 h at 22 °C with gentle shaking in the dark. Finally, the luminescence signal was measured using the Spark® plate reader (Tecan). The alkaline phosphatase activity of cells that were treated with DMSO and purmorphamine was set to 100%. Calculations of the IC_{50} values were conducted using the GraphPad Prism 9 (GraphPad Software, USA).

Reverse Transcription Quantitative PCR (RT-qPCR)

Sixty-thousand C3H10T1/2 cells were seeded into 12-well plates and incubated at 37 °C in 5% CO_2 for 48 hours to achieve 80% cell confluency. Hh signaling was then activated by treating the cells with 1.5 µM purmorphamine, and different concentrations of the compounds or DMSO as control for 96 h. After this time, the total RNA was isolated using the RNAeasy Kit (Qiagen, #74104) including a DNase digestion step. The RNA concentration was measured using NanoDrop 2000 (Thermo Scientific), and the QuantiTect Reverse Transcription Kit (Qiagen #205313) was used to generate cDNA. The relative mRNA amount of the target genes *Ptch1*[7], *Gli1*[8], and *Alpl* along with the reference genes *Gapdh* and *Ap3d1* was assessed using SsoAdvanced™ Universal SYBR® Green Supermix, template cDNA, and primers. The SYBR Green signal was detected using the CFX96 Real-Time PCR Detection System (Bio-Rad, Germany) and relative gene expression levels were calculated using the $2^{-\Delta\Delta Ct}$ method[9] with *Gapdh* and *Ap3dl* as reference genes. Gene expression levels in DMSO/purmorphamine-treated samples were set to 100%. *Alpl*, *Ptch1*, and *Gli1* expression levels in compound-treated samples were calculated relative to the respective positive control.

Used primers:

Primer	Forward (5’-3’)	Reverse (5’-3’)
Ptch1	CTCTGGAGCAGATTTCCAAGG	TGCCGCAGTTCTTTTGAATG
Gli1	CACCGTGGGAGTAAACAGGCCTTCC	CCAGAGCGTTACACACCTGCCCTTC
Alpl	ATCTTTGGTCTGGCTCCCATG	TTTCCCGTTCACCGTCCAC
Gapdh	CAGTGCCAGCCTCGTC	CAATCTCCACTTTGCCACTG
Ap3dl	CAGAGGGCTCATCGGTACAC	GCCGGAAGTCCAACTTCTCA

Smoothened Binding Assay

The Smoothened binding assay was adopted from a previously described procedure[10]. Sixty-thousand HEK293T cells per well were seeded on poly-D-lysine-coated coverslips (Neuvitro, 12 mm, #GG-12-1.5-PDL), placed in a 24-well plate, and incubated at 37 °C in 5% CO_2 for 24 h. The cells were transfected with the SMO-expressing plasmid pGEN-mSMO (pGEN-mSmo was a gift from Philip Beachy (Addgene plasmid # 37673; http://n2t.net/addgene:37673; RRID:Addgene_37673))[11] in OptiMEM medium using FuGENE® HD transfection reagent (Promega, # E2311) according to the manufacturer's protocol. Cells were incubated at 37 °C in 5% CO_2 for 48h. The cells were then washed once with PBS, fixed with 3.7% paraformaldehyde in PBS for 10 min at 22 °C, and subsequently treated with PBS containing 10 mM glycine and 0.2% NaN_3 for 5 min. The fixed cells were then washed three times with PBS and treated with the compounds, Vismodegib (Selleckchem #1082) or DMSO in DMEM containing 0.5% FBS (assay medium), and 5 nM BODIPY-Cyclopamine S26 (Carbosynth Limited, FB18988) for four hours at 22 °C in the dark. After this time, the cover slips were washed with PBS and incubated for 10 min at 22 °C with 1 g/ml 4',6 diamidino-2-phenylindole (DAPI, Sigma Aldrich, Roche, #10236276001) in PBS. The cover slips were washed again and then mounted onto glass slides using Aqua Polymount (Polysciences). Zeiss Observer Z1 microscope (Carl Zeiss, Germany) was used to acquire the images using a Plan-Apochromat 63x/1.40 Oil DIC M27 objective.

Immunocytochemistry

In a 96 well plate (Cellvis P96-1-N, 0.13-0.16 mm thickness), 5,000 U2OS cells were seeded in each well and incubated for 16 h (37 °C, 5% CO_2). The cells were treated with compounds or DMSO for 24 h. The cells were then fixed using 3.7% paraformaldehyde in PBS and permeabilized with 0.1% Triton X100 (in PBS). The cells were washed with PBS-T followed by the addition of 2% BSA in PBS-T and incubation for 1 h prior to staining with DAPI (Sigma Aldrich, D9542-10MG, 1:1000 dilution) to visualize DNA and anti-tubulin-FITC antibody (Thermo Fisher, MA119581, 1:500 dilution) or anti-phospho-histone H3 antibody (Cell Signalling, #8481, 1:500 dilution) overnight at 4 °C. The images were acquired using Observer Z1 (Carl Zeiss, Germany) using 63X objectives (LD Plan-Neofluar). An Axiovert 200M microscope (Carl Zeiss, Germany) equipped with 10X objective was used to detect phospho-histone H3-positive cells and could be quantified by using MetaMorph 7. For the automated image analysis, the percentage of phospho-histone H3-positive cells was determined using the DNA stain to assess the total number of cells by the software CellProfiler.

In Vitro Tubulin Polymerization Assay

In vitro tubulin polymerization assay was performed as described previously by Akbarzadeh et al[3]. Briefly, porcine α/β-tubulin was diluted in a buffer containing 80 mM PIPES (pH 6.9), 2 mM $MgCl_2$, and 0.5 mM EGTA. α/β-Tubulin was then added to a solution containing $MgCl_2$ and glutamate with a final concentration of 0.88 µM and 0.8 mM, respectively, and added to a 96-well plate. Subsequently, compounds at a final concentration of 20 µM were added to the tubulin solution (final concentration of 10 µM) and incubated at 22 °C for 20 min. The plate was then incubated on ice for another 20 min followed by the addition of GTP to provide a final concentration of 500 µM. Tubulin polymerization was monitored for 60 min by means of turbidity measurements at 340 nm using an Infinite M200 plate reader (Tecan).

Flow Cytometry

For cell cycle analysis by flow cytometry, the Click-it™ Plus EdU Alexa Fluor™ 488 Flow Cytometry Assay Kit (Thermo Fisher Scientific, Cat. No. C10632) was used according to the manufacturer's protocol. For this, 1.25 x 10^5 U2OS cells were seeded per well in a 6-well plate and incubated overnight. The following day, cells were treated with the compounds or with DMSO as a control and incubated for 22 h. Afterwards, cells were pulsed with 10 µM EdU (5-ethynyl-2´-deoxyuridine) or medium as a control and incubated for another 2 h. Cells were washed with PBS, detached using trypsin, re-suspended in PBS and centrifuged at 300 x g for 7 min at room temperature. After another washing step with 1 % BSA in PBS, cells were fixed with 4 % paraformaldehyde (PFA) in PBS, permeabilized and subjected to a click-reaction to label the incorporated EdU. All centrifugation steps after fixation were performed at 900 x g for 7 min at room temperature. The DNA content was stained with a propidium iodide solution (100 µg/mL propidium iodide, 0.1 % (v/v) Triton X-100 and 100 µg/mL DNase-free RNase A in PBS) for 30 min at room temperature. Before analysis, the cell suspensions were filtered into FACS tubes through a nylon mesh. For each sample, 10,000 cells were analyzed by the BD LSRII analyzer (Becton Dickinson, USA). FlowJo 10.7.2 software was used for analysis and quantification of all data. For every analysis, FSC and SSC gating was performed to exclude debris and to select single cells. All experiments were performed in three biological replicates.

Uridine Rescue Assay

2,000 HCT116 cells were seeded per well in a 96-well plate (Falcon, 96 well, all clear) and incubated overnight (37 °C, 5% CO_2). On the next day, the medium was replaced by fresh compound or DMS- containing medium supplemented with solvent or uridine (100 µM). The cell growth was recorded every 2 h for a total of 96 h after treatment start using an IncuCyte Zoom (Essen BioScience). Cell confluence was determined as a measure of cell growth using the IncuCyte Zoom software (Essen BioScience).

Statistical Analyses

Statistical analyses for biological experiments where appropriate were performed using GraphPad Prism 9.2.0 software. Statistical significance was determined using either unpaired two-tailed *t*-tests or unpaired two-tailed *t*-tests with Welch's correction. * $p < 0.05$, ** $p < 0.01$, *** $p < 0.001$, **** $p < 0.0001$, ns = not significant.

X-Ray Structure Analysis of B10

The crystal structure of compound **B10** was determined using the *Bruker D8 Venture* four-circle diffractometer equipped with a *PHOTON II* CPAD detector by *Bruker AXS GmbH*. The X-ray radiation was generated by the *IμS* microfocus source Cu (λ = 1.54178 Å) or Mo (λ = 0.71073 Å) from *Incoatec GmbH* equipped with HELIOS mirror optics and a single-hole collimator by *Bruker AXS GmbH*. The selected single crystal of **B10** was covered with an inert oil (perfluoropolyalkyl ether) and mounted on the *MicroMount* from *MiTeGen*. The APEX 4 Suite (v.2021.10-0) software integrated with SAINT (integration) and SADABS (adsorption correction) programs by *Bruker AXS GmbH* were used for data collection. The processing and finalization of the crystal structure were performed using the Olex2 program[12]. The crystal structures were solved by the ShelXT[13] structure solution program using the Intrinsic Phasing option, which were further refined by the ShelXL[13] refinement package using Least Squares minimization. The non-hydrogen atoms were anisotropically refined. The C-bound H atoms were placed in geometrically calculated positions, and a fixed isotropic displacement parameter was assigned to each atom according to the riding-model: C–H = 0.95–1.00 Å with Uiso(H) = 1.5Ueq(CH_3) and 1.2Ueq(CH_2, CH) for other hydrogen atoms. The N-bound hydrogen atoms on N1 were located on the Difference-Fourier-Map and refined independently in every structure. The crystallographic data for the structure of **B10** has been published as supplementary publication number 2221540, in the Cambridge Crystallographic Data Centre. A copy of these data can be obtained for free by applying to CCDC, 12 Union Road, Cambridge CB2 IEZ, UK, fax: 144-(0)1223-336033 or e-mail: deposit@ccdc.cam.ac.uk.

Compound **B10** was recrystallized in dichloromethane at 23 °C via slow evaporation.

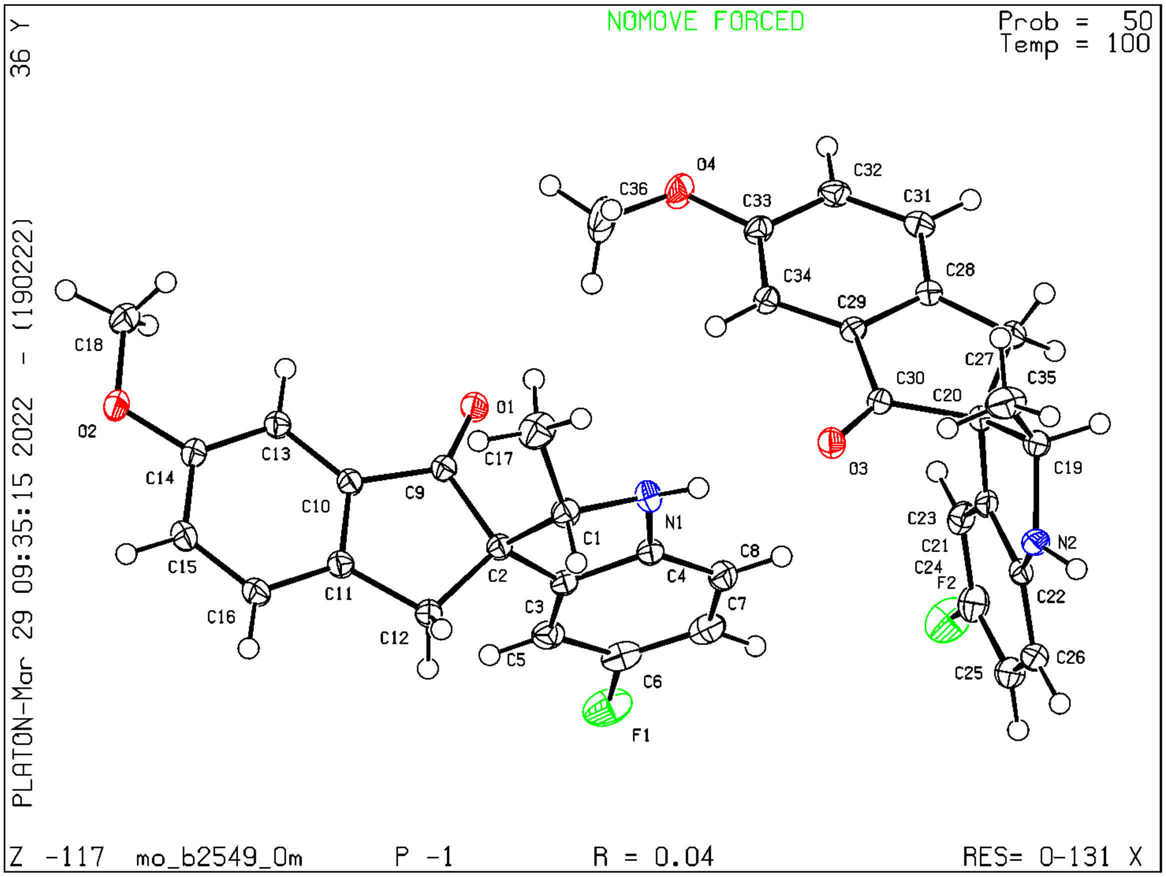

Supplementary Fig. 27. X-ray crystal structure of **B10**. Ellipsoid plot of **B10** [$C_{18}H_{16}FNO_2$ (M =297.32 g/mol)] at the 50% probability level. See Supplementary Table 12 for additional details. Crystallographic data have been deposited at the Cambridge Crystallographic Data Centre and copies can be obtained on request, free of charge, by quoting the publication citation and the deposition number **CCDC: 2221540**.

Supplementary Table 13. Crystal data and structure refinement for **B10**.

Empirical formula	$C_{18}H_{16}FNO_2$
Formula weight	297.32
Temperature/K	100.00
Crystal system	triclinic
Space group	P-1
a/Å	8.9543(6)
b/Å	11.4503(6)
c/Å	15.6757(9)
α/°	88.070(3)
β/°	81.722(2)
γ/°	68.323(2)
Volume/Å^3	1477.67(15)
Z	4
ρ_{calc}g/cm^3	1.336
μ/mm^{-1}	0.096
F(000)	624.0
Crystal size/mm^3	0.957 × 0.625 × 0.452
Radiation	MoKα (λ = 0.71073)
2Θ range for data collection/°	3.828 to 61.114
Index ranges	-12 ≤ h ≤ 12, -16 ≤ k ≤ 16, -21 ≤ l ≤ 22
Reflections collected	39897
Independent reflections	9062 [R_{int} = 0.0260, R_{sigma} = 0.0223]
Data/restraints/parameters	9062/0/409
Goodness-of-fit on F^2	1.054
Final R indexes [I>=2σ (I)]	R_1 = 0.0439, wR_2 = 0.1056
Final R indexes [all data]	R_1 = 0.0521, wR_2 = 0.1119
Largest diff. peak/hole / e Å^{-3}	0.44/-0.26

Chemistry Experimental Section

General Information

Unless otherwise noted, all commercially available compounds were used as provided without further purifications. Solvents for chromatography were technical grade. Analytical thin-layer chromatography (TLC) was performed using petroleum-ether and ethyl acetate as gradient eluents, based on Merck aluminium TLC sheets (silica gel $60F_{254}$). Compounds were visualized by irradiation with UV light. Column chromatography was performed using silica gel Merck 60 (particle size 0.040-0.063 mm).

^1H-NMR and ^{13}C-NMR were recorded on a Bruker DRX400 (400 MHz), Bruker DRX500 (500 MHz), INOVA500 (500 MHz) and Bruker DRX700 using CD_2Cl_2, $CDCl_3$, or CD_3COCD_3 as solvent. Data are reported in the following order: chemical shift (δ) values are reported in ppm with the solvent resonance as internal standard (CD_2Cl_2: δ = 5.32 ppm for ^1H, δ = 53.84 ppm for ^{13}C; $CDCl_3$: δ = 7.26 ppm for ^1H, δ = 77.23 ppm for ^{13}C); multiplicities are indicated s (singlet), d (doublet), t (triplet), q (quartet), m (multiplet); coupling constants (J) are given in Hertz (Hz).

High resolution mass spectra were recorded on a LTQ Orbitrap mass spectrometer coupled to an Accela HPLC-System (HPLC column: Hypersyl GOLD, 50 mm x 1 mm, particle size 1.9 µm, ionization method: electron spray ionization).

Data collection for single crystal X-ray structure analyses was conducted on a Bruker D8 Venture fourcircle diffractometer by Bruker AXS GmbH using a PHOTON II CPAD detector by Bruker AXS GmbH. X-ray radiation was generated by microfocus sources IµS 3.0 Mo by Incoatec GmbH with HELIOS mirror optics and a single-hole collimator by Bruker AXS GmbH. For the data collection, the programs APEX 3 Suite (v.2018.7-2) with the integrated programs SAINT (integration) and SADABS (adsorption correction) by Bruker AXS GmbH were used.

General Procedure A: Synthesis of 3-(2-bromobenzyl)-indole derivatives[14]

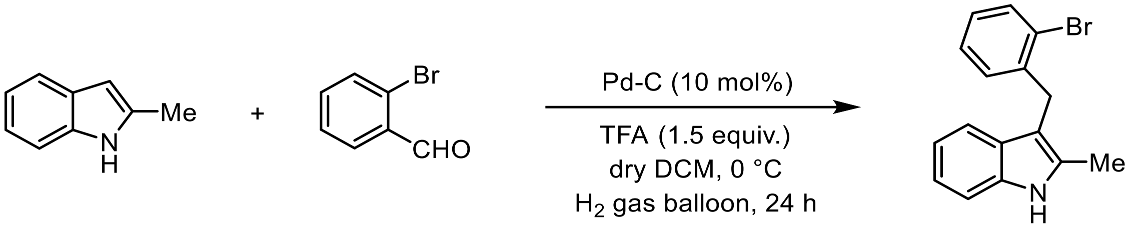

3-(2-bromobenzyl)-2-methyl-1H-indole [**1a**]: In an oven dried 250 ml two-neck round bottom flask with a magnetic stirrer, 10% Pd-C (75 mg) was taken. The flask was degassed and filled with Argon (three times) carefully as Pd-C can go out by vacuum. Then, dry DCM (50 mL) and followed by TFA (1.15 mL, 15 mmol, 1.5 equiv.) was added under Ar at 0 °C; the mixture was stirred for 10 min. Then, a solution of indole derivative (10 mmol, 1 equiv.) and the corresponding 2-bromobenzaldehyde derivative (11 mmol, 1.1 equiv.) in 100 mL RB in dry DCM (50 mL) [Ar atm.] was added dropwise to the reaction mixture. Then, the reaction tube was flashed with H_2 gas and connected with one/two H_2 balloon. After stirring for 4 hours at 0 °C, the reaction mixture was allowed to warm to room temperature and the stirring was continued for additional 20 hours. Next, the reaction mixture was filtered through filter paper and concentrated in a rotary evaporator. The crude reaction mixture was purified through a silica gel column.

Yield: 86% (2.57 g).

Eluent: Petroleum ether/Ethyl acetate (97/3, v/v).

TLC: R_f = 0.35 (95:5 petroleum ether:EtOAc).

Physical State: Brown oil.

^{1}H NMR (500 MHz, Chloroform-*d*) δ 7.85 (s, 1H), 7.58 (dd, *J* = 7.8, 1.4 Hz, 1H), 7.36 – 7.29 (m, 2H), 7.11 (dtd, *J* = 17.8, 7.3, 1.3 Hz, 2H), 7.03 (tt, *J* = 7.1, 1.1 Hz, 2H), 6.95 (dd, *J* = 7.7, 1.9 Hz, 1H), 4.14 (s, 2H), 2.36 (s, 3H) ppm.

^{13}C NMR (126 MHz, $CDCl_3$) δ 140.47, 135.52, 132.60, 130.15, 129.08, 128.47, 127.61, 127.49, 124.78, 121.33, 119.56, 118.62, 110.38, 109.11, 30.59, 12.05 ppm.

HRMS (m/z): [M + H^+] calcd for $C_{16}H_{15}NBr$, 300.03824; found, 300.03840.

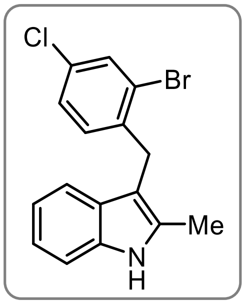

3-(2-bromo-4-chlorobenzyl)-2-methyl-1H-indole [**1b**]: Compound **1b** was prepared according to the general procedure A (5 mmol scale).

Yield: 84% (1.4 g).

Eluent: Petroleum ether/Ethyl acetate (97/3, v/v).

TLC: R_f = 0.38 (95:5 petroleum ether:EtOAc).

Physical State: Brown oil.

^{1}H NMR (500 MHz, Chloroform-*d*) δ 7.88 (s, 1H), 7.60 (d, *J* = 2.2 Hz, 1H), 7.30 (td, *J* = 8.3, 1.0 Hz, 2H), 7.14 (ddd, *J* = 8.1, 7.0, 1.2 Hz, 1H), 7.08 – 7.02 (m, 2H), 6.87 – 6.83 (m, 1H), 4.08 (s, 2H), 2.36 (s, 3H) ppm.

^{13}C NMR (126 MHz, $CDCl_3$) δ 139.12, 135.52, 132.64, 132.32, 132.09, 130.84, 128.86, 127.66, 124.81, 121.48, 119.68, 118.45, 110.46, 108.65, 30.06, 12.01 ppm.

HRMS (m/z): [M + H^+] calcd for $C_{16}H_{14}NBrCl$, 333.999; found, 333.997.

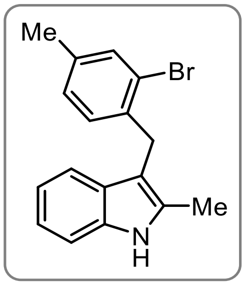

3-(2-bromo-4-methylbenzyl)-2-methyl-1H-indole [**1c**]: Compound **1c** was prepared according to the general procedure A (5 mmol scale).

Yield: 90% (1.4 g).

Eluent: Petroleum ether/Ethyl acetate (98/2, v/v).

TLC: R_f = 0.4 (95:5 petroleum ether:EtOAc).

Physical State: Brown solid.

^{1}H NMR (500 MHz, Chloroform-*d*) δ 7.86 (s, 1H), 7.43 – 7.39 (m, 1H), 7.35 – 7.32 (m, 1H), 7.30 (dt, *J* = 8.1, 0.9 Hz, 1H), 7.12 (ddd, *J* = 8.2, 7.1, 1.3 Hz, 1H), 7.04 – 6.99 (m, 1H), 6.91 – 6.87 (m, 1H), 6.82 (d, *J* = 7.8 Hz, 1H), 4.09 (s, 2H), 2.36 (s, 3H), 2.27 (s, 3H) ppm.

^{13}C NMR (126 MHz, $CDCl_3$) δ 137.52, 137.29, 135.52, 133.04, 132.47, 129.84, 129.11, 128.28, 124.46, 121.28, 119.52, 118.65, 110.34, 109.36, 30.13, 20.74, 12.05 ppm.

HRMS (m/z): [M + H^+] calcd for $C_{17}H_{17}NBr$, 314.05389; found, 314.05413.

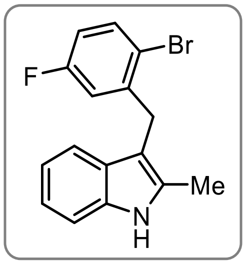

3-(2-bromo-5-fluorobenzyl)-2-methyl-1H-indole [**1d**]: Compound **1d** was prepared according to the general procedure A (10 mmol scale).

Yield: 82% (1.3 g).

Eluent: Petroleum ether/Ethyl acetate (97/3, v/v).

TLC: R_f = 0.36 (95:5 petroleum ether:EtOAc).

Physical State: Brown oil.

^{1}H NMR (500 MHz, Chloroform-*d*) δ 7.85 (s, 1H), 7.55 (ddd, *J* = 8.7, 5.3, 2.4 Hz, 1H), 7.38 – 7.30 (m, 2H), 7.17 (tdd, *J* = 8.1, 6.9, 4.2, 1.6 Hz, 1H), 7.08 (qd, *J* = 7.2, 6.4, 2.7 Hz, 1H), 6.79 (td, *J* = 8.2, 3.2 Hz, 1H), 6.66 (dq, *J* = 9.6, 3.1 Hz, 1H), 4.12 (s, 2H), 2.36 (s, 3H) ppm.

^{13}C NMR (126 MHz, $CDCl_3$) δ 163.35, 161.40 [162.37 (d, *J* = 245.7 Hz)], 143.02, 142.96 [142.99 (d, *J* = 6.9 Hz)], 135.54, 133.60, 133.53 [133.57 (d, *J* = 7.9 Hz)], 132.74, 128.80, 121.50, 119.72, 118.60, 118.58 [118.59 (d, *J* = 3.0 Hz)], 118.37, 117.16, 116.98 [117.07 (d, *J* = 23.6 Hz)], 114.85, 114.67 [114.76 (d, *J* = 22.8 Hz)], 110.49, 108.38, 30.67, 11.94 ppm.

^{19}F NMR (470 MHz, Chloroform-*d*) δ -114.80 (qd, *J* = 9.5, 8.8, 4.9 Hz) ppm.

HRMS (m/z): [M + H$^+$] calcd for $C_{16}H_{14}NBrF$, 318.0288; found, 318.0287.

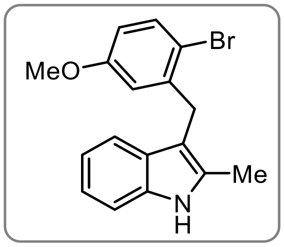

3-(2-bromo-5-methoxybenzyl)-2-methyl-1H-indole [**1e**]: Compound **1e** was prepared according to the general procedure A (10 mmol scale).

Yield: 78% (1.283 g).

Eluent: Petroleum ether/Ethyl acetate (97/3, v/v).

TLC: R_f = 0.35 (95:5 petroleum ether:EtOAc).

Physical State: Amorphous solid.

^{1}H NMR (500 MHz, Chloroform-*d*) δ 7.86 (s, 1H), 7.46 (d, *J* = 8.7 Hz, 1H), 7.36 – 7.33 (m, 1H), 7.29 (dt, *J* = 8.0, 1.0 Hz, 1H), 7.11 (ddd, *J* = 8.1, 7.0, 1.2 Hz, 1H), 7.02 (ddd, *J* = 7.9, 7.1, 1.0 Hz, 1H), 6.60 (dd, *J* = 8.8, 3.1 Hz, 1H), 6.52 (dd, *J* = 3.1, 0.8 Hz, 1H), 4.08 (s, 2H), 3.59 (s, 2H), 2.36 (s, 3H) ppm.

^{13}C NMR (126 MHz, $CDCl_3$) δ 159.14, 141.70, 135.56, 133.04, 132.53, 129.06, 121.33, 119.58, 118.58, 116.54, 115.28, 112.61, 110.35, 109.02, 55.45, 30.71, 12.09 ppm.

HRMS (m/z): [M + H^+] calcd for $C_{17}H_{17}NBr$, 330.0488; found, 330.0489.

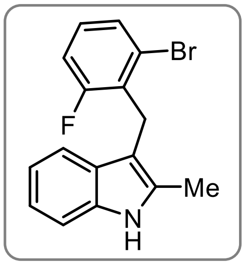

3-(2-bromo-6-fluorobenzyl)-2-methyl-1H-indole [**1f**]: Compound **1f** was prepared according to the general procedure A (10 mmol scale).

Yield: 71% (1.125 g).

Eluent: Petroleum ether/Ethyl acetate (97/3, v/v).

TLC: R_f = 0.36 (95:5 petroleum ether:EtOAc).

Physical State: Brown oil.

^{1}H NMR (500 MHz, Chloroform-*d*) δ 7.81 (s, 1H), 7.43 (dt, *J* = 7.9, 1.0 Hz, 1H), 7.28 (dt, *J* = 8.1, 0.9 Hz, 1H), 7.14 – 7.08 (m, 2H), 7.08 – 7.02 (m, 3H), 6.95 (td, *J* = 7.5, 1.3 Hz, 1H), 4.08 (s, 2H), 2.41 (s, 3H) ppm.

^{13}C NMR (126 MHz, $CDCl_3$) δ 162.09, 160.14 [161.11 (d, *J* = 244.5 Hz)], 135.43, 132.15, 130.57, 130.53 [130.55 (d, *J* = 4.9 Hz)], 128.97, 128.54, 128.41 [128.47 (d, *J* = 15.4 Hz)], 127.53, 127.47 [127.50 (d, *J* = 7.9 Hz)], 124.09, 124.06 [124.07 (d, *J* = 3.6 Hz)], 121.26, 119.51, 118.45, 115.24, 115.06 [115.15 (d, *J* = 22.0 Hz)], 110.35, 109.24, 22.97, 22.94 [22.96 (d, *J* = 4.1 Hz)], 11.93 ppm.

^{19}F NMR (470 MHz, Chloroform-*d*) δ -118.15 (q, *J* = 8.0, 7.2 Hz) ppm.

HRMS (m/z): [M + H^+] calcd for $C_{16}H_{14}NBrF$, 318.02882; found, 318.02887.

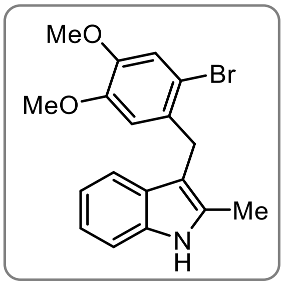

3-(2-bromo-4,5-dimethoxybenzyl)-2-methyl-1H-indole [**1g**]: Compound **1g** was prepared according to the general procedure A (10 mmol scale).

Yield: 62% (2.23 g).

Eluent: Petroleum ether/Ethyl acetate (80/20, v/v).

TLC: R_f = 0.22 (80:20 petroleum ether:EtOAc).

Physical State: Amorphous solid.

^{1}H NMR (600 MHz, Chloroform-*d*) δ 8.02 – 7.90 (m, 1H), 7.40 (td, *J* = 8.6, 8.1, 3.3 Hz, 1H), 7.30 – 7.23 (m, 1H), 7.12 (dddd, *J* = 9.5, 7.3, 3.1, 1.3 Hz, 1H), 7.10 – 7.02 (m, 2H), 6.62 – 6.56 (m, 1H), 4.10 (d, *J* = 3.9 Hz, 2H), 3.85 (s, 3H), 3.56 (s, 3H), 2.34 (s, 3H) ppm.

^{13}C NMR (151 MHz, $CDCl_3$) δ 148.45, 147.88, 135.49, 132.71, 132.26, 128.94, 121.15, 119.40, 118.50, 115.50, 114.20, 113.22, 110.35, 109.40, 56.27, 55.97, 30.15, 11.97 ppm.

HRMS (m/z): [M + H^+] calcd for $C_{18}H_{19}NO_2Br$, 360.059; found, 360.059.

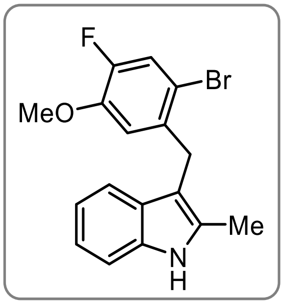

3-(2-bromo-4-fluoro-5-methoxybenzyl)-2-methyl-1H-indole [**1h**]: Compound **1h** was prepared according to the general procedure A (5 mmol scale).

Yield: 54% (937 mg).

Eluent: Petroleum ether/Ethyl acetate (87/13, v/v).

TLC: R_f = 0.55 (80:20 petroleum ether:EtOAc).

Physical State: Brown solid.

^{1}H NMR (500 MHz, Chloroform-*d*) δ 7.88 (s, 1H), 7.35 – 7.29 (m, 3H), 7.12 (ddd, *J* = 8.1, 7.0, 1.2 Hz, 1H), 7.04 (ddd, *J* = 8.1, 7.0, 1.1 Hz, 1H), 6.61 (d, *J* = 9.0 Hz, 1H), 4.06 (s, 3H), 3.57 (s, 3H), 2.37 (s, 3H) ppm.

^{13}C NMR (126 MHz, $CDCl_3$) δ <u>151.67, 149.69</u> [150.68 (d, *J* = 248.2 Hz)], <u>147.07, 146.99</u> [147.03 (d, *J* = 10.2 Hz)], <u>136.61, 136.58</u> [136.60 (d, *J* = 3.6 Hz)], 135.53, 132.47, 128.88, 121.45, <u>120.12, 119.95</u> [120.03 (d, *J* = 21.3 Hz)], 119.66, 118.50, <u>115.07, 115.06</u> [115.06 (d, *J* = 2.1 Hz)], <u>113.59, 113.52</u> [113.55 (d, *J* = 8.2 Hz)], 110.44, 109.09, 56.51, 30.27, 12.11 ppm.

^{19}F NMR (470 MHz, Chloroform-*d*) δ -136.83 (t, *J* = 9.8 Hz) ppm.

HRMS (m/z): [M + H$^+$] calcd for $C_{17}H_{16}NBrF$, 348.0394; found, 348.0393.

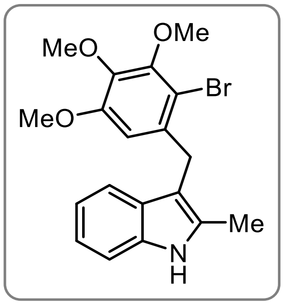

3-(2-bromo-3,4,5-trimethoxybenzyl)-2-methyl-1H-indole [**1i**]: Compound **1i** was prepared according to the general procedure A (5 mmol scale).

Yield: 39% (759 mg).

Eluent: Petroleum ether/Ethyl acetate (70/30, v/v).

TLC: R_f = 0.22 (70:30 petroleum ether:EtOAc).

Physical State: Amorphous solid.

^{1}H NMR (500 MHz, Chloroform-*d*) δ 7.88 (s, 1H), 7.37 (dt, *J* = 7.8, 0.9 Hz, 1H), 7.30 (dt, *J* = 8.1, 0.9 Hz, 1H), 7.12 (ddd, *J* = 8.2, 7.0, 1.2 Hz, 1H), 7.03 (ddd, *J* = 8.0, 7.1, 1.0 Hz, 1H), 6.38 (s, 1H), 4.10 (s, 2H), 3.93 (s, 3H), 3.85 (s, 3H), 3.53 (s, 3H), 2.38 (s, 3H) ppm.

^{13}C NMR (126 MHz, $CDCl_3$) δ 152.60, 150.81, 141.40, 136.24, 135.53, 132.50, 129.07, 121.35, 119.56, 118.65, 110.75, 110.38, 109.17, 109.11, 61.30, 61.17, 56.15, 30.75, 12.14 ppm.

HRMS (m/z): [M + H$^+$] calcd for $C_{19}H_{21}O_3NBr$, 390.0699; found, 390.06996.

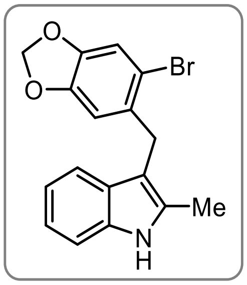

3-((6-bromobenzo[d][1,3]dioxol-5-yl)methyl)-2-methyl-1H-indole [**1j**]: Compound **1j** was prepared according to the general procedure A (5 mmol scale).

Yield: 58% (995 mg).

Eluent: Petroleum ether/Ethyl acetate (85/15, v/v).

TLC: R_f = 0.3 (80:20 petroleum ether:EtOAc).

Physical State: Amorphous solid.

^{1}H NMR (500 MHz, Chloroform-*d*) δ 7.84 (s, 1H), 7.38 – 7.28 (m, 2H), 7.13 (ddd, *J* = 8.1, 7.0, 1.2 Hz, 1H), 7.08 – 7.02 (m, 2H), 6.42 (s, 1H), 5.86 (s, 2H), 4.04 (s, 2H), 2.36 (s, 3H) ppm.

^{13}C NMR (126 MHz, $CDCl_3$) δ 147.50, 146.60, 135.52, 133.87, 132.47, 128.94, 121.38, 119.62, 118.54, 114.39, 112.53, 110.38, 109.94, 109.45, 101.60, 30.29, 11.99 ppm.

HRMS (m/z): [M + H^+] calcd for C17H15NBr, 344.0281; found, 344.0281.

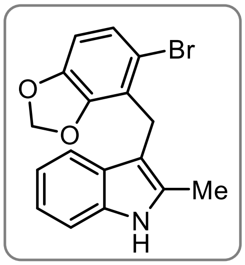

3-((5-bromobenzo[d][1,3]dioxol-4-yl)methyl)-2-methyl-1H-indole [**1k**]: Compound **1k** was prepared according to the general procedure A (5 mmol scale).

Yield: 41% (703 mg).

Eluent: Petroleum ether/Ethyl acetate (85/15, v/v).

TLC: R_f = 0.3 (80:20 petroleum ether:EtOAc).

Physical State: Amorphous solid.

^{1}H NMR (500 MHz, Chloroform-*d*) δ 7.73 (s, 1H), 7.55 – 7.51 (m, 1H), 7.23 (dt, *J* = 8.0, 0.9 Hz, 1H), 7.07 (ddd, *J* = 8.1, 7.0, 1.2 Hz, 1H), 7.04 – 6.97 (m, 2H), 6.57 (d, *J* = 8.2 Hz, 1H), 5.98 (s, 2H), 4.09 (s, 2H), 2.42 (s, 3H) ppm.

^{13}C NMR (126 MHz, $CDCl_3$) δ 147.36, 146.76, 135.24, 132.09, 128.79, 125.60, 122.92, 121.00, 119.32, 118.86, 116.46, 110.20, 108.27, 107.82, 101.55, 25.47, 12.56 ppm.

HRMS (m/z): [M + H$^+$] calcd for $C_{17}H_{15}NO_2Br$, 344.0281; found, 344.0282.

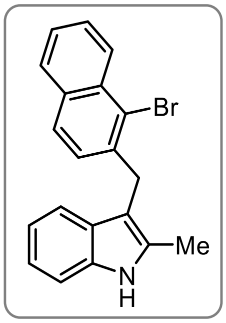

3-((1-bromonaphthalen-2-yl)methyl)-2-methyl-1H-indole [**1l**]: Compound **1l** was prepared according to the general procedure A (5 mmol scale).

Yield: 66% (1.152 g).

Eluent: Petroleum ether/Ethyl acetate (97/3, v/v).

TLC: R_f = 0.38 (95:5 petroleum ether:EtOAc).

Physical State: Brown oil.

^{1}H NMR (700 MHz, Chloroform-*d*) δ 8.41 (dd, *J* = 8.6, 1.1 Hz, 1H), 7.82 (s, 1H), 7.76 (dd, *J* = 8.2, 1.3 Hz, 1H), 7.64 – 7.56 (m, 2H), 7.48 (ddd, *J* = 8.1, 6.8, 1.2 Hz, 1H), 7.39 (dd, *J* = 8.0, 1.1 Hz, 1H), 7.31 (dd, *J* = 8.1, 0.9 Hz, 1H), 7.14 (t, *J* = 7.7 Hz, 2H), 7.04 (ddd, *J* = 8.0, 7.0, 1.0 Hz, 1H), 4.43 (s, 2H), 2.38 (s, 3H) ppm.

^{13}C NMR (176 MHz, $CDCl_3$) δ 138.83, 135.54, 133.50, 132.69, 132.55, 129.20, 128.21, 127.74, 127.55, 127.47, 127.43, 126.02, 123.80, 121.38, 119.64, 118.64, 110.40, 109.71, 31.69, 12.10 ppm.

HRMS (m/z): [M + H$^+$] calcd for $C_{20}H_{17}NBr$, 350.0539; found, 350.0539.

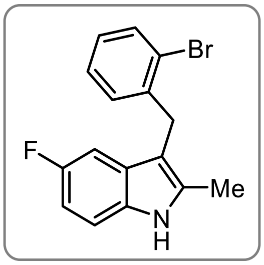

3-(2-bromobenzyl)-5-fluoro-2-methyl-1H-indole [**1m**]: Compound **1m** was prepared according to the general procedure A (10 mmol scale).

Yield: 87% (1.38 g).

Eluent: Petroleum ether/Ethyl acetate (97/3, v/v).

TLC: R_f = 0.38 (95:5 petroleum ether:EtOAc).

Physical State: Brown oil.

^{1}H NMR (500 MHz, Chloroform-*d*) δ 7.84 (s, 1H), 7.58 (dd, *J* = 7.9, 1.4 Hz, 1H), 7.19 (dd, *J* = 8.7, 4.4 Hz, 1H), 7.12 (td, *J* = 7.5, 1.4 Hz, 1H), 7.05 (td, *J* = 7.6, 1.8 Hz, 1H), 6.96 (dd, *J* = 9.8, 2.5 Hz, 1H), 6.93 (dd, *J* = 7.7, 1.7 Hz, 1H), 6.85 (td, *J* = 9.0, 2.5 Hz, 1H), 4.09 (s, 2H), 2.36 (s, 3H) ppm.

^{13}C NMR (126 MHz, $CDCl_3$) δ 158.93, 157.07 [158.00 (d, *J* = 233.9 Hz)], 140.04, 134.57, 132.73, 131.93, 130.03, 129.56, 129.48 [129.52 (d, *J* = 9.9 Hz)], 127.77, 127.54 [127.66 (d, *J* = 29.2 Hz)], 124.76, 110.89, 110.82 [110.86 (d, *J* = 9.6 Hz)], 109.46, 109.42, 109.44, 109.23 [109.34 (d, *J* = 26.0 Hz)], 103.81, 103.62 [103.71 (d, *J* = 23.6 Hz)], 30.63, 12.18 ppm.

^{19}F NMR (470 MHz, Chloroform-*d*) δ -124.76 (td, *J* = 9.7, 4.6 Hz) ppm.

HRMS (m/z): [M + H$^+$] calcd for $C_{16}H_{14}NBrF$, 318.02882; found, 318.02914.

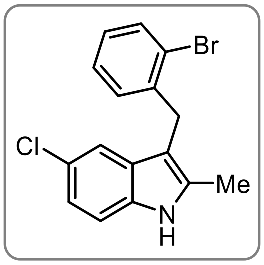

3-(2-bromobenzyl)-5-chloro-2-methyl-1H-indole [**1n**]: Compound **1n** was prepared according to the general procedure A (5 mmol scale).

Yield: 80% (1.332 g).

Eluent: Petroleum ether/Ethyl acetate (97/3, v/v).

TLC: R_f = 0.38 (95:5 petroleum ether:EtOAc).

Physical State: Brown oil.

^{1}H NMR (500 MHz, Chloroform-*d*) δ 7.87 (s, 1H), 7.59 (dd, *J* = 7.9, 1.4 Hz, 1H), 7.29 (d, *J* = 2.0 Hz, 1H), 7.20 (d, *J* = 8.5 Hz, 1H), 7.12 (td, *J* = 7.5, 1.4 Hz, 1H), 7.09 – 7.03 (m, 2H), 6.90 (ddt, *J* = 7.7, 1.9, 0.9 Hz, 1H), 4.09 (s, 2H), 2.34 (s, 3H) ppm.

^{13}C NMR (126 MHz, $CDCl_3$) δ 139.95, 134.23, 133.83, 132.73, 130.22, 129.93, 127.80, 127.56, 125.25, 124.74, 121.51, 118.05, 111.36, 108.97, 30.48, 12.10 ppm.

HRMS (m/z): [M + H^+] calcd for $C_{16}H_{14}NBrCl$, 333.999; found, 333.999.

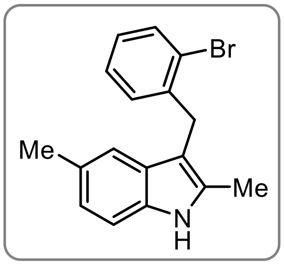

3-(2-bromobenzyl)-2,5-dimethyl-1H-indole [**1o**]: Compound **1o** was prepared according to the general procedure A (5 mmol scale).

Yield: 89% (1.393 g).

Eluent: Petroleum ether/Ethyl acetate (98/2, v/v).

TLC: R_f = 0.4 (95:5 petroleum ether:EtOAc).

Physical State: Brown oil.

^{1}H NMR (500 MHz, Chloroform-*d*) δ 7.71 (s, 1H), 7.65 (dd, *J* = 7.8, 1.5 Hz, 1H), 7.24 – 7.19 (m, 2H), 7.13 (td, *J* = 7.5, 1.5 Hz, 1H), 7.08 (td, *J* = 7.6, 1.9 Hz, 1H), 7.02 (dd, *J* = 8.1, 1.7 Hz, 1H), 6.98 (dd, *J* = 7.6, 1.9 Hz, 1H), 4.17 (s, 2H), 2.46 (s, 3H), 2.34 (s, 3H) ppm.

^{13}C NMR (126 MHz, $CDCl_3$) δ 140.52, 133.78, 132.76, 132.51, 130.05, 129.31, 128.68, 127.53, 127.47, 124.74, 122.76, 118.26, 110.10, 108.43, 30.49, 21.68, 11.93 ppm.

HRMS (m/z): [M + H^+] calcd for $C_{17}H_{17}NBr$, 314.054; found, 314.054.

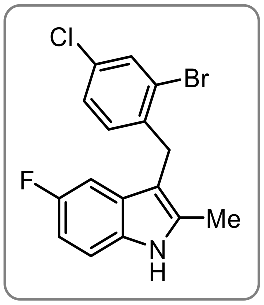

3-(2-bromo-4-chlorobenzyl)-5-fluoro-2-methyl-1H-indole [**1p**]: Compound **1p** was prepared according to the general procedure A (5 mmol scale).

Yield: 75% (1.316 g).

Eluent: Petroleum ether/Ethyl acetate (98/2, v/v).

TLC: R_f = 0.4 (95:5 petroleum ether:EtOAc).

Physical State: Brown oil.

^{1}H NMR (500 MHz, Chloroform-*d*) δ 7.87 (s, 1H), 7.60 (d, *J* = 2.2 Hz, 1H), 7.20 (dd, *J* = 8.7, 4.3 Hz, 1H), 7.12 – 7.05 (m, 1H), 6.92 (dd, *J* = 9.6, 2.5 Hz, 1H), 6.89 – 6.81 (m, 2H), 4.03 (s, 2H), 2.35 (s, 3H) ppm.

^{13}C NMR (126 MHz, $CDCl_3$) δ 158.97, 157.10 [158.04 (d, *J* = 234.3 Hz)], 138.70, 134.64, 132.50, 132.23, 131.93, 130.70, 129.35, 129.28 [129.32 (d, *J* = 9.8 Hz)], 127.72, 124.82, 111.02, 110.94 [110.98 (d, *J* = 9.6 Hz)], 109.61, 109.40 [109.51 (d, *J* = 26.2 Hz)], 109.01, 108.97 [108.99 (d, *J* = 4.5 Hz)], 103.65, 103.46 [103.56 (d, *J* = 23.6 Hz)], 30.08, 12.16 ppm.

^{19}F NMR (470 MHz, Chloroform-*d*) δ -124.50 (td, *J* = 9.5, 4.3 Hz) ppm.

HRMS (m/z): [M + H^+] calcd for $C_{16}H_{13}NBrClF$, 351.9898; found, 351.9898.

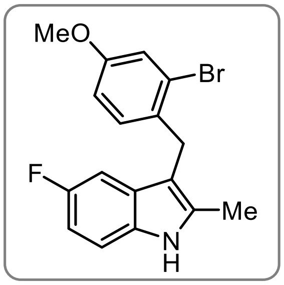

3-(2-bromo-4-methoxybenzyl)-5-fluoro-2-methyl-1H-indole [**1q**]: Compound **1q** was prepared according to the general procedure A (5 mmol scale).

Yield: 70% (1.215 g).

Eluent: Petroleum ether/Ethyl acetate (90/10, v/v).

TLC: R_f = 0.42 (80:20 petroleum ether:EtOAc).

Physical State: Brown solid.

^{1}H NMR (500 MHz, Chloroform-*d*) δ 7.82 (s, 1H), 7.18 (dd, *J* = 8.7, 4.4 Hz, 1H), 7.14 (d, *J* = 2.6 Hz, 1H), 6.95 (dd, *J* = 9.8, 2.5 Hz, 1H), 6.87 – 6.80 (m, 2H), 6.68 (dd, *J* = 8.6, 2.6 Hz, 1H), 4.01 (s, 2H), 3.76 (s, 3H), 2.36 (s, 3H) ppm.

^{13}C NMR (126 MHz, $CDCl_3$) δ 158.51, 158.92, 157.06 [157.99 (d, *J* = 233.8 Hz)], 134.38, 132.01, 131.93, 130.35, 129.56, 129.48 [129.52 (d, *J* = 9.8 Hz)], 124.66, 117.93, 113.64, 110.85, 110.77 [110.81 (d, *J* = 9.6 Hz)], 109.96, 109.93 [109.94 (d, *J* = 4.5 Hz)], 109.41, 109.20 [109.30 (d, *J* = 26.1 Hz)], 103.86, 103.67 [103.76 (d, *J* = 23.6 Hz)], 55.72, 29.71, 12.20 ppm.

^{19}F NMR (470 MHz, Chloroform-*d*) δ -124.83 (td, *J* = 9.4, 4.4 Hz) ppm.

HRMS (m/z): [M + H^+] calcd for $C_{17}H_{16}ONBrF$, 348.0394; found, 348.0395.

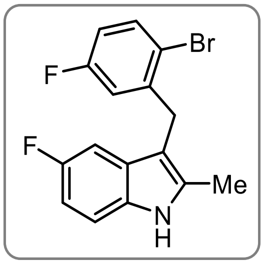

3-(2-bromo-5-fluorobenzyl)-5-fluoro-2-methyl-1H-indole [**1r**]: Compound **1r** was prepared according to the general procedure A (5 mmol scale).

Yield: 83% (1.39 g).

Eluent: Petroleum ether/Ethyl acetate (98/2, v/v).

TLC: R_f = 0.4 (95:5 petroleum ether:EtOAc).

Physical State: Brown oil.

^{1}H NMR (500 MHz, Chloroform-*d*) δ 7.88 (s, 1H), 7.53 (tt, *J* = 7.7, 2.9 Hz, 1H), 7.21 (dd, *J* = 8.7, 4.3 Hz, 1H), 6.94 (qd, *J* = 9.7, 8.7, 3.8 Hz, 1H), 6.87 (ddd, *J* = 8.9, 5.4, 2.2 Hz, 1H), 6.79 (td, *J* = 8.2, 2.9 Hz, 1H), 6.67 – 6.55 (m, 1H), 4.05 (s, 2H), 2.36 (s, 3H) ppm.

^{13}C NMR (126 MHz, $CDCl_3$) δ 163.38, 161.43 [162.41 (d, *J* = 246.0 Hz)], 159.04, 157.18 [158.11 (d, *J* = 234.3 Hz], 142.60, 142.55 [142.57 (d, *J* = 7.2 Hz)], 134.77, 134.72 [134.74 (d, *J* = 5.8 Hz)], 133.76, 133.70 [133.73 (d, *J* = 7.7 Hz)], 131.99, 129.34, 129.27 [129.31 (d, *J* = 9.6 Hz)], 118.63, 117.05, 116.86 [116.96 (d, *J* = 23.5 Hz)], 115.02, 114.84 [114.93 (d, *J* = 22.7 Hz)], 111.06, 111.01 [111.04 (d, *J* = 6.8 Hz)], 109.70, 109.49 [109.59 (d, *J* = 26.0 Hz)], 108.79, 108.75 [108.77 (d, *J* = 4.5 Hz)], 103.61, 103.43 [103.52 (d, *J* = 23.4 Hz)], 77.49, 77.23, 76.98, 30.70, 12.12 ppm.

^{19}F NMR (470 MHz, Chloroform-*d*) δ -112.96 – -116.30 (m), -122.84 – -125.96 (m) ppm.

HRMS (m/z): [M + H$^+$] calcd for $C_{16}H_{13}NBrF_2$, 336.0194; found, 336.0195.

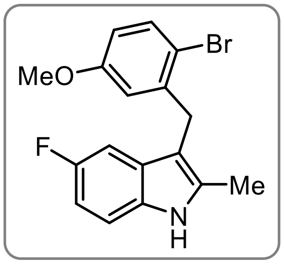

3-(2-bromo-5-methoxybenzyl)-5-fluoro-2-methyl-1H-indole [**1s**]: Compound **1s** was prepared according to the general procedure A (5 mmol scale).

Yield: 69% (1.197 g).

Eluent: Petroleum ether/Ethyl acetate (90/10, v/v).

TLC: R_f = 0.4 (80:20 petroleum ether:EtOAc).

Physical State: Brown solid.

^{1}H NMR (500 MHz, Chloroform-*d*) δ 7.85 (s, 1H), 7.47 (d, *J* = 8.7 Hz, 1H), 7.18 (dd, *J* = 8.7, 4.3 Hz, 1H), 6.97 (dd, *J* = 9.7, 2.5 Hz, 1H), 6.84 (td, *J* = 9.1, 2.5 Hz, 1H), 6.61 (dd, *J* = 8.7, 3.1 Hz, 1H), 6.51 – 6.47 (m, 1H), 4.03 (s, 2H), 3.62 (s, 3H), 2.35 (s, 3H) ppm.

^{13}C NMR (126 MHz, $CDCl_3$) δ 159.14, 158.96, 157.10 [158.03 (d, *J* = 233.9 Hz)], 141.26, 134.55, 133.16, 131.96, 129.54, 129.46 [129.50 (d, *J* = 9.8 Hz)], 116.52, 115.25, 112.59, 110.88, 110.80 [110.84 (d, *J* = 9.8 Hz)], 109.46, 109.25 [109.35 (d, *J* = 26.3 Hz)], 109.34, 109.31 [109.32 (d, *J* = 4.5 Hz)], 103.77, 103.58 [103.68 (d, *J* = 23.6 Hz)], 55.47, 30.71, 12.19 ppm.

^{19}F NMR (470 MHz, Chloroform-*d*) δ -124.84 (td, *J* = 9.5, 4.3 Hz) ppm.

HRMS (m/z): [M + H$^+$] calcd for $C_{17}H_{16}ONBrF$, 348.0394; found, 348.0395.

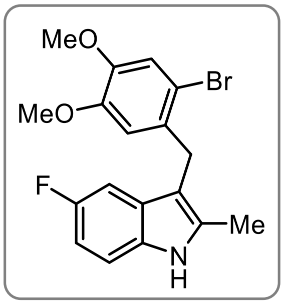

3-(2-bromo-4,5-dimethoxybenzyl)-5-fluoro-2-methyl-1H-indole [**1t**]: Compound **1t** was prepared according to the general procedure A (5 mmol scale).

Yield: 69% (1.3 g).

Eluent: Petroleum ether/Ethyl acetate (80/20, v/v).

TLC: R_f = 0.23 (80:20 petroleum ether:EtOAc).

Physical State: Amorphous white solid.

^{1}H NMR (600 MHz, Chloroform-*d*) δ 7.87 (s, 1H), 7.19 – 7.14 (m, 1H), 7.06 (s, 1H), 6.99 (dd, *J* = 9.8, 2.5 Hz, 1H), 6.86 – 6.81 (m, 1H), 6.53 (s, 1H), 4.02 (s, 2H), 3.85 (s, 3H), 3.59 (s, 3H), 2.36 (s, 3H) ppm.

^{13}C NMR (151 MHz, $CDCl_3$) δ 158.75, 157.20 [157.97 (d, *J* = 233.9 Hz)], 148.55, 148.07, 134.30, 132.22, 131.93, 129.49, 129.42 [129.46 (d, *J* = 9.8 Hz)], 115.62, 114.28, 113.12, 110.89, 110.83 [110.86 (d, *J* = 9.5 Hz)], 109.94, 109.92 [109.93 (d, *J* = 4.4 Hz)], 109.39, 109.21 [109.30 (d, *J* = 26.3 Hz)], 103.75, 103.60 [103.68 (d, *J* = 23.6 Hz)], 56.36, 56.11, 30.22, 12.23 ppm.

^{19}F NMR (565 MHz, Chloroform-*d*) δ -124.77 (td, *J* = 9.6, 4.5 Hz) ppm.

HRMS (m/z): [M + H^+] calcd for $C_{18}H_{18}O_2NBrF$, 378.0499; found, 378.0499.

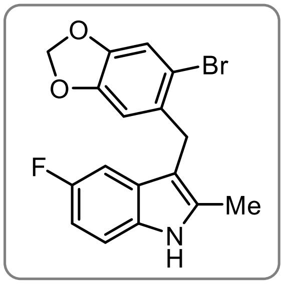

3-((6-bromobenzo[d][1,3]dioxol-5-yl)methyl)-5-fluoro-2-methyl-1H-indole [**1u**]: Compound **1u** was prepared according to the general procedure A (5 mmol scale).

Yield: 71% (1.28 g).

Eluent: Petroleum ether/Ethyl acetate (85/15, v/v).

TLC: R_f = 0.42 (80:20 petroleum ether:EtOAc).

Physical State: Off-white solid.

^{1}H NMR (500 MHz, Chloroform-*d*) δ 7.86 (s, 1H), 7.19 (dd, *J* = 8.7, 4.3 Hz, 1H), 7.04 (s, 1H), 6.96 (dd, *J* = 9.7, 2.5 Hz, 1H), 6.85 (td, *J* = 9.1, 2.5 Hz, 1H), 6.39 (s, 1H), 5.88 (s, 2H), 3.98 (s, 2H), 2.36 (s, 3H) ppm.

^{13}C NMR (126 MHz, $CDCl_3$) δ 158.97, 157.11 [158.04 (d, *J* = 234.0 Hz)], 147.54, 146.72, 134.47, 133.41, 131.95, 129.43, 129.36 [129.40 (d, *J* = 9.7 Hz)], 114.43, 113.50, 113.44 [113.47 (d, *J* = 7.3 Hz)], 112.65, 110.91, 110.84 [110.87 (d, *J* = 9.6 Hz)], 109.77, 109.51, 109.31 [109.41 (d, *J* = 26.0 Hz)], 103.76, 103.57 [103.66 (d, *J* = 23.6 Hz)], 101.67, 30.33, 12.13 ppm.

^{19}F NMR (470 MHz, Chloroform-*d*) δ -124.71 (td, *J* = 9.7, 4.4 Hz) ppm.

HRMS (m/z): [M + H$^+$] calcd for $C_{17}H_{14}O_2NBrF$, 362.01865; found, 362.01875.

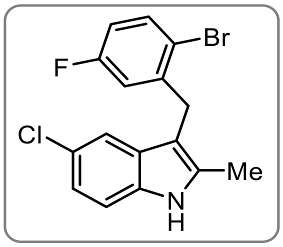

3-(2-bromo-5-fluorobenzyl)-5-chloro-2-methyl-1H-indole [**1v**]: Compound **1v** was prepared according to the general procedure A (10 mmol scale).

Yield: 86% (3.02 g).

Eluent: Petroleum ether/Ethyl acetate (98/2, v/v).

TLC: R_f = 0.4 (95:5 petroleum ether:EtOAc).

Physical State: Brown solid.

^{1}H NMR (500 MHz, Chloroform-*d*) δ 7.98 – 7.86 (m, 1H), 7.53 (ddd, *J* = 8.7, 5.3, 1.8 Hz, 1H), 7.30 – 7.26 (m, 1H), 7.22 (dd, *J* = 8.6, 2.3 Hz, 1H), 7.08 (dt, *J* = 8.5, 2.2 Hz, 1H), 6.79 (td, *J* = 8.1, 2.8 Hz, 1H), 6.65 – 6.50 (m, 1H), 4.04 (s, 2H), 2.34 (s, 3H) ppm.

^{13}C NMR (126 MHz, $CDCl_3$) δ 163.39, 161.44 [162.41 (d, *J* = 245.8 Hz)], 142.51, 142.46 [142.49 (d, *J* = 7.3 Hz)], 134.44, 134.42 [134.43 (d, *J* = 2.8 Hz)], 133.90, 133.77 [133.84 (d, *J* = 16.3 Hz)], 133.71, 130.00, 125.47, 121.77, 118.63, 117.86, 116.98, 116.79 [116.88 (d, *J* = 23.6 Hz)], 115.05, 114.87 [114.96 (d, *J* = 22.6 Hz)], 111.51, 108.31, 30.56, 12.05 ppm.

^{19}F NMR (470 MHz, Chloroform-*d*) δ -113.30 – -115.98 (m) ppm.

HRMS (m/z): [M + H$^+$] calcd for $C_{16}H_{13}NBrClF$, 351.9898; found, 351.9898.

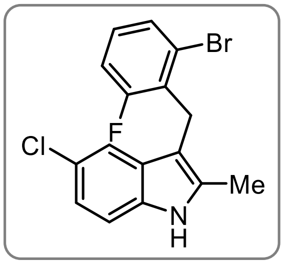

3-(2-bromo-6-fluorobenzyl)-5-chloro-2-methyl-1H-indole [**1w**]: Compound **1w** was prepared according to the general procedure A (5 mmol scale).

Yield: 65% (1.14 mg).

Eluent: Petroleum ether/Ethyl acetate (97/3, v/v).

TLC: R_f = 0.35 (95:5 petroleum ether:EtOAc).

Physical State: Brown oil.

^{1}H NMR (700 MHz, Chloroform-*d*) δ 7.55 (dd, *J* = 2.2, 0.6 Hz, 1H), 7.35 (dt, *J* = 8.0, 1.1 Hz, 1H), 7.31 (dd, *J* = 8.7, 2.2 Hz, 1H), 7.08 (td, *J* = 8.1, 5.9 Hz, 1H), 6.98 (ddd, *J* = 9.5, 8.2, 1.2 Hz, 1H), 6.69 (dd, *J* = 8.6, 0.6 Hz, 1H), 4.86 (s, 1H), 3.27 – 3.16 (m, 2H), 1.37 (d, *J* = 1.5 Hz, 3H) ppm.

^{13}C NMR (176 MHz, $CDCl_3$) δ 202.48, 162.43, 161.01 [161.72 (d, *J* = 249.7 Hz)], 158.05, 137.36, 129.70, 129.65 [129.68 (d, *J* = 9.4 Hz)], 129.13, 129.11 [129.12 (d, *J* = 3.3 Hz)], 126.68, 126.66 [126.67 (d, *J* = 4.9 Hz)], 124.60, 124.49 [124.55 (d, *J* = 18.8 Hz)], 124.42, 124.05, 121.18, 120.65, 115.09, 114.95 [115.02 (d, *J* = 23.7 Hz)], 113.34, 68.31, 36.55, 22.86, 22.85 [22.85 (d, *J* = 2.0 Hz)] ppm.

^{19}F NMR (470 MHz, Chloroform-*d*) δ -106.84 (t, *J* = 7.7 Hz) ppm.

HRMS (m/z): [M + H$^+$] calcd for $C_{16}H_{13}NBrClF$, 351.9898; found, 351.9899.

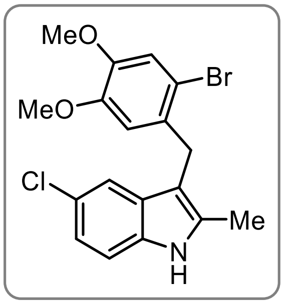

3-(2-bromo-4,5-dimethoxybenzyl)-5-chloro-2-methyl-1H-indole [**1x**]: Compound **1x** was prepared according to the general procedure A (5 mmol scale).

Yield: 71% (1.4 g).

Eluent: Petroleum ether/Ethyl acetate (80/20, v/v).

TLC: R_f = 0.42 (75:25 petroleum ether:EtOAc).

Physical State: Off-white solid.

^{1}H NMR (500 MHz, Chloroform-*d*) δ 7.96 (s, 1H), 7.33 (d, *J* = 2.0 Hz, 1H), 7.15 (d, *J* = 8.5 Hz, 1H), 7.09 – 7.01 (m, 2H), 6.52 (s, 1H), 4.01 (s, 2H), 3.85 (s, 3H), 3.59 (s, 3H), 2.33 (s, 3H) ppm.

^{13}C NMR (126 MHz, $CDCl_3$) δ 148.44, 147.98, 133.98, 133.77, 132.09, 130.06, 125.07, 121.36, 117.97, 115.56, 114.23, 113.02, 111.35, 109.31, 56.29, 56.06, 30.05, 12.09 ppm.

HRMS (m/z): [M + H$^+$] calcd for $C_{18}H_{18}O_2NBrCl$, 394.0204; found, 394.0204.

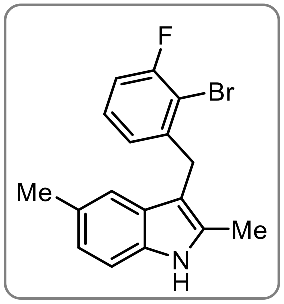

3-(2-bromo-3-fluorobenzyl)-2,5-dimethyl-1H-indole [**1y**]: Compound **1y** was prepared according to the general procedure A (5 mmol scale).

Yield: 54% (894 mg).

Eluent: Petroleum ether/Ethyl acetate (98/2, v/v).

TLC: R_f = 0.4 (95:5 petroleum ether:EtOAc).

Physical State: Brown oil.

^{1}H NMR (600 MHz, Chloroform-*d*) δ 7.74 (s, 1H), 7.21 (dd, *J* = 8.2, 0.6 Hz, 1H), 7.12 (dd, *J* = 1.8, 0.9 Hz, 1H), 7.06 (td, *J* = 7.9, 5.6 Hz, 1H), 7.00 – 6.93 (m, 2H), 6.74 (ddq, *J* = 7.7, 1.7, 0.9 Hz, 1H), 4.13 (s, 2H), 2.41 (s, 3H), 2.34 (s, 3H) ppm.

^{13}C NMR (151 MHz, $CDCl_3$) δ 160.05, 158.42 [159.24 (d, *J* = 245.7 Hz)], 143.16, 133.83, 132.81, 129.25, 128.88, 128.14, 128.08 [128.11 (d, *J* = 8.2 Hz)], 125.18, 125.16 [125.17 (d, *J* = 2.8 Hz)], 122.93, 118.20, 113.91, 113.76 [113.83 (d, *J* = 22.8 Hz)], 111.45, 111.32, 110.13, 108.24, 77.44, 77.23, 77.02, 30.34, 30.32, 21.69, 12.00 ppm.

^{19}F NMR (565 MHz, Chloroform-*d*) δ -105.62 (dd, *J* = 8.3, 5.4 Hz) ppm.

HRMS (m/z): [M + H$^+$] calcd for $C_{17}H_{16}NBrF$, 332.0445; found, 332.0445.

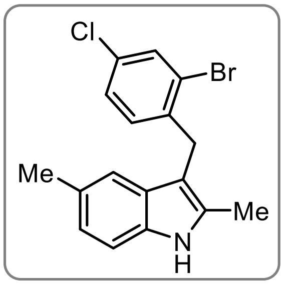

3-(2-bromo-4-chlorobenzyl)-2,5-dimethyl-1H-indole [**1z**]: Compound **1z** was prepared according to the general procedure A (5 mmol scale).

Yield: 77% (1.34 g).

Eluent: Petroleum ether/Ethyl acetate (98/2, v/v).

TLC: R_f = 0.4 (95:5 petroleum ether:EtOAc).

Physical State: Brown oil.

^{1}H NMR (500 MHz, Chloroform-*d*) δ 7.78 (s, 1H), 7.60 (d, *J* = 2.1 Hz, 1H), 7.20 (d, *J* = 8.2 Hz, 1H), 7.09 – 7.04 (m, 2H), 6.96 (dd, *J* = 8.2, 1.6 Hz, 1H), 6.82 (dd, *J* = 8.3, 0.9 Hz, 1H), 4.04 (s, 2H), 2.38 (s, 3H), 2.32 (s, 3H) ppm.

^{13}C NMR (126 MHz, $CDCl_3$) δ 139.21, 133.82, 132.80, 132.26, 132.05, 130.79, 129.15, 128.94, 127.67, 124.82, 122.98, 118.16, 110.13, 108.13, 30.00, 21.70, 12.02 ppm.

HRMS (m/z): [M + H$^+$] calcd for $C_{17}H_{16}NBrCl$, 348.0149; found, 348.0147.

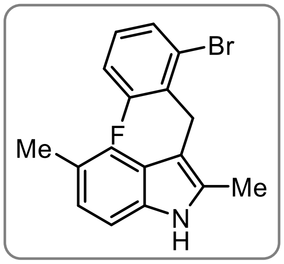

3-(2-bromo-6-fluorobenzyl)-2,5-dimethyl-1H-indole [**1aa**]: Compound **1aa** was prepared according to the general procedure A (5 mmol scale).

Yield: 57% (943 mg).

Eluent: Petroleum ether/Ethyl acetate (98/2, v/v).

TLC: R_f = 0.4 (95:5 petroleum ether:EtOAc).

Physical State: Brown oil.

^{1}H NMR (500 MHz, Chloroform-*d*) δ 7.63 (s, 1H), 7.36 (dt, *J* = 7.9, 1.2 Hz, 1H), 7.29 (d, *J* = 1.7 Hz, 1H), 7.12 (d, *J* = 8.2 Hz, 1H), 7.05 (td, *J* = 8.1, 6.0 Hz, 1H), 7.00 (ddd, *J* = 9.6, 8.2, 1.4 Hz, 1H), 6.90 (dd, *J* = 8.2, 1.6 Hz, 1H), 4.18 (d, *J* = 2.0 Hz, 2H), 2.39 (s, 3H), 2.35 (s, 3H) ppm.

^{13}C NMR (126 MHz, $CDCl_3$) δ 162.87, 160.90 [161.89 (d, *J* = 248.9 Hz)], 133.44, 132.15, 128.94, 128.81 [128.88 (d, *J* = 17.3 Hz)], 128.93, 128.87, 128.84 [128.85 (d, *J* = 3.5 Hz)], 128.45, 128.37, 125.79, 125.75 [125.77 (d, *J* = 5.0 Hz)], 122.48, 118.49, 118.47 [118.48 (d, *J* = 2.3 Hz)], 114.90, 114.71 [114.80 (d, *J* = 23.3 Hz)], 109.89, 107.80, 25.03, 25.00 [25.01 (d, *J* = 3.6 Hz)], 21.83, 12.43, 12.41 [12.42 (d, *J* = 2.2 Hz)] ppm.

^{19}F NMR (470 MHz, Chloroform-*d*) δ -110.13 – -110.20 (m) ppm.

HRMS (m/z): [M + H$^+$] calcd for $C_{17}H_{16}NBrF$, 332.0445; found, 332.0445.

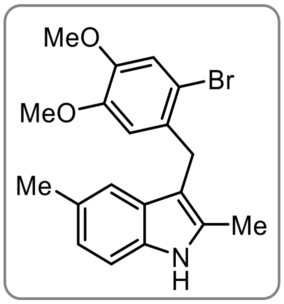

3-(2-bromo-4,5-dimethoxybenzyl)-2,5-dimethyl-1H-indole [**1ab**]: Compound **1ab** was prepared according to the general procedure A (5 mmol scale).

Yield: 71% (1.32 g).

Eluent: Petroleum ether/Ethyl acetate (80/20, v/v).

TLC: R_f = 0.32 (75:25 petroleum ether:EtOAc).

Physical State: Brown solid.

^{1}H NMR (500 MHz, Chloroform-*d*) δ 7.75 (s, 1H), 7.20 – 7.14 (m, 2H), 7.06 (s, 1H), 6.93 (dd, *J* = 8.2, 1.7 Hz, 1H), 6.55 (s, 1H), 4.04 (s, 2H), 3.85 (s, 3H), 3.57 (s, 3H), 2.38 (s, 3H), 2.34 (s, 3H) ppm.

^{13}C NMR (126 MHz, $CDCl_3$) δ 148.45, 147.87, 133.81, 132.73, 132.44, 129.30, 128.70, 122.78, 118.35, 115.45, 114.22, 113.12, 110.00, 109.05, 56.35, 56.07, 30.13, 21.72, 12.17 ppm.

HRMS (m/z): [M + H^+] calcd for $C_{19}H_{21}NO_2NBr$, 374.075; found, 374.0749.

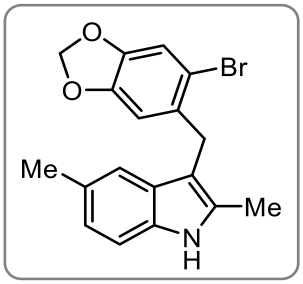

3-((6-bromobenzo[d][1,3]dioxol-5-yl)methyl)-2,5-dimethyl-1H-indole [**1ac**]: Compound **1ac** was prepared according to the general procedure A (5 mmol scale).

Yield: 63% (1.125 g).

Eluent: Petroleum ether/Ethyl acetate (80/20, v/v).

TLC: R_f = 0.35 (75:25 petroleum ether:EtOAc).

Physical State: Brown solid.

^{1}H NMR (500 MHz, Chloroform-*d*) δ 7.75 (s, 1H), 7.21 – 7.17 (m, 1H), 7.14 – 7.11 (m, 1H), 7.05 (s, 1H), 6.95 (dd, *J* = 8.2, 1.7 Hz, 1H), 6.40 (s, 1H), 5.86 (s, 2H), 4.00 (s, 2H), 2.39 (s, 3H), 2.33 (s, 3H) ppm.

^{13}C NMR (126 MHz, $CDCl_3$) δ 147.50, 146.56, 133.99, 133.82, 132.65, 129.23, 128.84, 122.89, 118.23, 114.37, 112.52, 110.05, 109.91, 108.91, 101.58, 30.21, 21.72, 12.00 ppm.

HRMS (m/z): [M + H^+] calcd for $C_{18}H_{17}O_2NBr$, 358.0437; found, 358.0437.

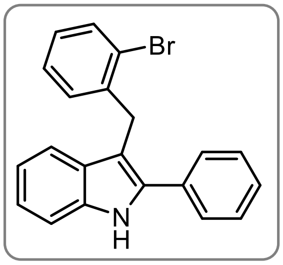

3-(2-bromobenzyl)-2-phenyl-1H-indole [**1ad**]: Compound **1ad** was prepared according to the general procedure A (10 mmol scale).

Yield: 89% (3.21 g).

Eluent: Petroleum ether/Ethyl acetate (97/3, v/v).

TLC: R_f = 0.32 (95:5 petroleum ether:EtOAc).

Physical State: Yellow semi-solid.

^{1}H NMR (500 MHz, Chloroform-*d*) δ 8.22 (s, 1H), 7.63 (dd, *J* = 7.6, 1.6 Hz, 1H), 7.48 – 7.40 (m, 5H), 7.37 (dd, *J* = 9.1, 7.5 Hz, 2H), 7.24 (ddd, *J* = 8.1, 7.0, 1.1 Hz, 1H), 7.14 – 7.04 (m, 3H), 7.01 (dd, *J* = 7.4, 2.1 Hz, 1H), 4.31 (s, 2H) ppm.

^{13}C NMR (126 MHz, $CDCl_3$) δ 140.41, 136.21, 136.11, 132.82, 132.67, 130.02, 129.62, 129.21, 128.06, 127.78, 127.74, 127.67, 124.87, 122.76, 120.13, 119.68, 111.04, 110.04, 31.30 ppm.

HRMS (m/z): [M + H^+] calcd for $C_{21}H_{17}NBr$, 362.05389; found, 362.05401.

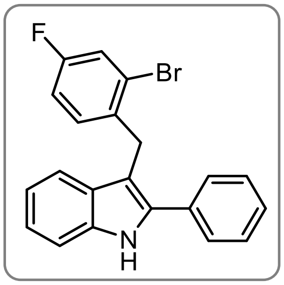

3-(2-bromo-4-fluorobenzyl)-2-phenyl-1H-indole [**1ae**]: Compound **1ae** was prepared according to the general procedure A (5 mmol scale).

Yield: 85% (1.61 g).

Eluent: Petroleum ether/Ethyl acetate (97/3, v/v).

TLC: R_f = 0.35 (95:5 petroleum ether:EtOAc).

Physical State: Yellow semi-solid.

^{1}H NMR (500 MHz, Chloroform-*d*) δ 8.23 (s, 1H), 7.46 – 7.42 (m, 5H), 7.40 – 7.33 (m, 3H), 7.26 – 7.21 (m, 1H), 7.09 (ddd, *J* = 8.0, 7.0, 1.0 Hz, 1H), 6.98 – 6.93 (m, 1H), 6.82 (td, *J* = 8.3, 2.6 Hz, 1H), 4.25 (s, 2H) ppm.

^{13}C NMR (126 MHz, $CDCl_3$) δ 162.01, 160.04 [161.03 (d, *J* = 248.1 Hz)], 136.23, 136.21, 136.14 [136.18 (d, *J* = 8.8 Hz)], 132.72, 130.70, 130.64 [130.67 (d, *J* = 8.2 Hz)], 129.44, 129.25, 128.92, 128.16, 127.76, 126.00, 124.41, 124.33 [124.37 (d, *J* = 9.5 Hz)], 122.86, 120.22, 119.94, 119.75 [119.85 (d, *J* = 24.5 Hz)], 119.53, 114.70, 114.53 [114.62 (d, *J* = 20.5 Hz)], 111.11, 109.90, 30.53 ppm.

^{19}F NMR (470 MHz, Chloroform-*d*) δ -115.53 (q, *J* = 7.3 Hz) ppm.

HRMS (m/z): [M + H^+] calcd for $C_{21}H_{16}NBrF$, 380.0445; found, 380.0427.

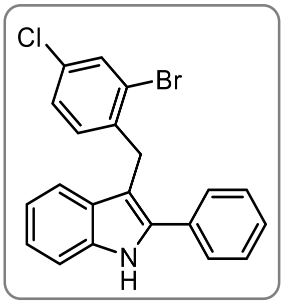

3-(2-bromo-4-chlorobenzyl)-2-phenyl-1H-indole [**1af**]: Compound **1af** was prepared according to the general procedure A (5 mmol scale).

Yield: 78% (1.54 g).

Eluent: Petroleum ether/Ethyl acetate (97/3, v/v).

TLC: R_f = 0.32 (95:5 petroleum ether:EtOAc).

Physical State: Yellow semi-solid.

^{1}H NMR (700 MHz, Chloroform-*d*) δ 8.22 (s, 1H), 7.64 (d, *J* = 2.2 Hz, 1H), 7.45 – 7.41 (m, 5H), 7.39 – 7.36 (m, 1H), 7.35 – 7.33 (m, 1H), 7.24 (ddd, *J* = 8.1, 7.0, 1.1 Hz, 1H), 7.11 – 7.06 (m, 2H), 6.92 (dd, *J* = 8.4, 0.9 Hz, 1H), 4.25 (s, 2H) ppm.

^{13}C NMR (176 MHz, CDCl$_3$) δ 139.11, 136.23, 136.22, 132.69, 132.52, 132.23, 130.76, 129.41, 129.26, 128.20, 127.84, 127.77, 124.93, 122.90, 120.28, 119.49, 111.13, 109.54, 30.81 ppm.

HRMS (m/z): [M + H$^+$] calcd for $C_{21}H_{16}NBrCl$, 396.0149; found, 396.0148.

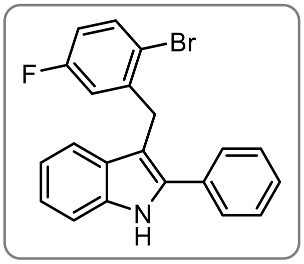

3-(2-bromo-5-fluorobenzyl)-2-phenyl-1H-indole [**1ag**]: Compound **1ag** was prepared according to the general procedure A (5 mmol scale).

Yield: 82% (1.554 g).

Eluent: Petroleum ether/Ethyl acetate (97/3, v/v).

TLC: R_f = 0.32 (95:5 petroleum ether:EtOAc).

Physical State: Yellow semi-solid.

^{1}H NMR (500 MHz, Chloroform-*d*) δ 8.22 (s, 1H), 7.56 (ddd, *J* = 7.3, 5.3, 1.8 Hz, 1H), 7.47 – 7.40 (m, 5H), 7.39 – 7.34 (m, 2H), 7.27 – 7.21 (m, 1H), 7.14 – 7.07 (m, 1H), 6.80 (td, *J* = 8.2, 2.9 Hz, 1H), 6.72 (dd, *J* = 9.9, 3.2 Hz, 1H), 4.27 (s, 2H) ppm.

^{13}C NMR (126 MHz, CDCl$_3$) δ <u>163.47, 161.52</u> [162.49 (d, *J* = 245.9 Hz)], <u>143.02, 142.96</u> [142.99 (d, *J* = 7.2 Hz)], 136.27, 136.21, <u>133.71, 133.65</u> [133.68 (d, *J* = 8.1 Hz)], 132.63, 129.35, 129.26, 128.20, 127.77, 122.91, 120.31, 119.43, <u>118.69, 118.66</u> [118.68 (d, *J* = 3.0 Hz)], <u>117.24, 117.05</u> [117.15 (d, *J* = 23.9 Hz)], <u>115.01, 114.82</u> [114.92 (d, *J* = 22.7 Hz)], 111.15, 109.26, 31.45 ppm.

^{19}F NMR (470 MHz, Chloroform-*d*) δ -113.33 – -115.90 (m) ppm.

HRMS (m/z): [M + H$^+$] calcd for $C_{21}H_{16}NBrF$, 380.0445; found, 380.0444.

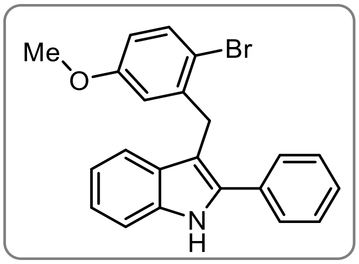

3-(2-bromo-5-methoxybenzyl)-2-phenyl-1H-indole [**1ah**]: Compound **1ah** was prepared according to the general procedure A (5 mmol scale).

Yield: 44% (834 mg).

Eluent: Petroleum ether/Ethyl acetate (85/15, v/v).

TLC: R_f = 0.48 (80:20 petroleum ether:EtOAc).

Physical State: Yellow semi-solid.

^{1}H NMR (500 MHz, Chloroform-*d*) δ 8.19 (s, 1H), 7.52 (d, *J* = 8.7 Hz, 1H), 7.49 – 7.39 (m, 6H), 7.38 – 7.32 (m, 1H), 7.23 (ddd, *J* = 8.1, 7.0, 1.1 Hz, 1H), 7.13 – 7.06 (m, 1H), 6.64 (dd, *J* = 8.6, 3.1 Hz, 1H), 6.60 (d, *J* = 3.1 Hz, 1H), 4.28 (s, 2H), 3.56 (s, 3H) ppm.

^{13}C NMR (126 MHz, $CDCl_3$) δ 159.24, 141.65, 136.21, 136.04, 133.10, 132.83, 129.60, 129.20, 128.04, 127.77, 122.75, 120.15, 119.64, 116.51, 115.28, 112.68, 111.01, 109.95, 55.42, 31.37 ppm.

HRMS (m/z): [M + H^+] calcd for $C_{21}H_{16}NBrF$, 392.065; found, 392.059.

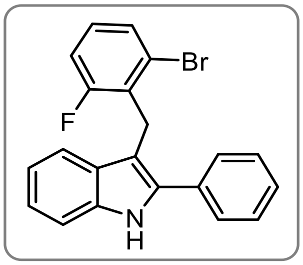

3-(2-bromo-6-fluorobenzyl)-2-phenyl-1H-indole [**1ai**]: Compound **1ai** was prepared according to the general procedure A (5 mmol scale).

Yield: 68% (1.29 g).

Eluent: Petroleum ether/Ethyl acetate (97/3, v/v).

TLC: R_f = 0.30 (95:5 petroleum ether:EtOAc).

Physical State: Yellow oil.

^{1}H NMR (500 MHz, Chloroform-*d*) δ 8.03 (s, 1H), 7.61 – 7.56 (m, 2H), 7.47 (t, *J* = 7.7 Hz, 2H), 7.42 – 7.36 (m, 2H), 7.32 (dd, *J* = 26.1, 8.0 Hz, 2H), 7.18 – 7.12 (m, 1H), 7.04 – 6.97 (m, 2H), 6.93 (ddd, *J* = 9.4, 8.2, 1.2 Hz, 1H), 4.44 (d, *J* = 1.6 Hz, 2H) ppm.

^{13}C NMR (126 MHz, $CDCl_3$) δ 162.81, 160.83 [161.82 (d, *J* = 249.3 Hz)], 135.95, 135.90, 133.65, 130.19, 128.94, 128.86, 128.81, 128.78 [128.79 (d, *J* = 3.4 Hz)], 128.66, 128.59 [128.63 (d, *J* = 9.4 Hz)], 128.56, 128.51, 128.43, 128.02, 126.03, 125.99 [126.01 (d, *J* = 5.1 Hz)], 122.20, 119.87, 119.71, 119.69 [119.70 (d, *J* = 1.9 Hz)], 114.86, 114.68 [114.77 (d, *J* = 23.1 Hz)], 110.92, 109.42, 25.75, 25.72 [25.74 (d, *J* = 3.5 Hz)] ppm.

^{19}F NMR (470 MHz, Chloroform-*d*) δ -109.87 (t, *J* = 7.9 Hz) ppm.

HRMS (m/z): [M + H$^+$] calcd for $C_{21}H_{16}NBrF$, 380.0447; found, 380.0446.

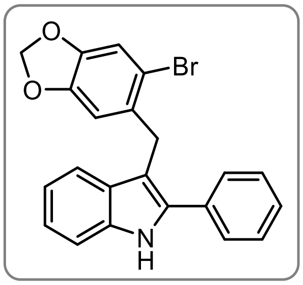

3-((6-bromobenzo[d][1,3]dioxol-5-yl)methyl)-2-phenyl-1H-indole [**1aj**]: Compound **1aj** was prepared according to the general procedure A (5 mmol scale).

Yield: 52% (1.05 g).

Eluent: Petroleum ether/Ethyl acetate (85/15, v/v).

TLC: R_f = 0.28 (80:20 petroleum ether:EtOAc).

Physical State: Yellow semi-solid.

^{1}H NMR (400 MHz, Chloroform-*d*) δ 8.18 (s, 1H), 7.48 – 7.36 (m, 7H), 7.23 (ddt, *J* = 8.0, 7.1, 1.0 Hz, 1H), 7.13 – 7.07 (m, 2H), 6.50 (d, *J* = 0.8 Hz, 1H), 5.87 (d, *J* = 0.7 Hz, 2H), 4.22 (s, 2H) ppm.

^{13}C NMR (126 MHz, $CDCl_3$) δ 147.62, 146.71, 136.20, 136.00, 133.79, 132.75, 129.52, 129.21, 128.08, 127.78, 122.80, 120.20, 119.61, 114.54, 112.70, 111.05, 110.32, 109.96, 101.64, 31.01 ppm.

HRMS (m/z): [M + H$^+$] calcd for $C_{22}H_{17}NBr$, 406.0437; found, 406.0438.

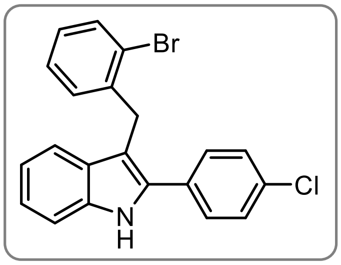

3-(2-bromobenzyl)-2-(4-chlorophenyl)-1H-indole [**1ak**]: Compound **1ak** was prepared according to the general procedure A (5 mmol scale).

Yield: 59% (1.165 g).

Eluent: Petroleum ether/Ethyl acetate (97/3, v/v).

TLC: R_f = 0.32 (95:5 petroleum ether:EtOAc).

Physical State: Yellow semi-solid.

^{1}H NMR (500 MHz, Chloroform-*d*) δ 8.06 (s, 1H), 7.53 – 7.50 (m, 1H), 7.28 (t, *J* = 7.9 Hz, 2H), 7.25 – 7.20 (m, 4H), 7.16 – 7.11 (m, 1H), 7.01 – 6.94 (m, 3H), 6.88 – 6.83 (m, 1H), 4.16 (s, 2H) ppm.

^{13}C NMR (126 MHz, $CDCl_3$) δ 140.10, 136.23, 134.81, 133.93, 132.73, 131.17, 129.88, 129.53, 129.35, 128.90, 127.85, 127.68, 124.80, 123.02, 120.29, 119.68, 111.13, 110.48, 31.21 ppm.

HRMS (m/z): [M + H^+] calcd for $C_{21}H_{16}NBrCl$, 396.015; found, 395.998.

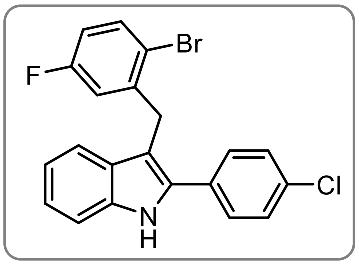

3-(2-bromo-5-fluorobenzyl)-2-(4-chlorophenyl)-1H-indole [**1al**]: Compound **1al** was prepared according to the general procedure A (5 mmol scale).

Yield: 61% (1.26 g).

Eluent: Petroleum ether/Ethyl acetate (97/3, v/v).

TLC: R_f = 0.35 (95:5 petroleum ether:EtOAc).

Physical State: Yellow semi-solid.

^{1}H NMR (500 MHz, Chloroform-*d*) δ 8.10 (s, 1H), 7.48 (dd, *J* = 8.7, 5.3 Hz, 1H), 7.35 (dt, *J* = 8.2, 0.9 Hz, 1H), 7.33 – 7.30 (m, 2H), 7.28 (dt, *J* = 8.0, 0.9 Hz, 1H), 7.26 (d, *J* = 2.1 Hz, 1H), 7.19 – 7.15 (m, 1H), 7.03 (ddd, *J* = 8.0, 7.0, 0.9 Hz, 1H), 6.75 – 6.68 (m, 1H), 6.60 (ddd, *J* = 9.9, 3.1, 0.9 Hz, 1H), 4.14 (s, 1H) ppm.

^{13}C NMR (126 MHz, $CDCl_3$) δ 163.47, 161.51 [162.49 (d, *J* = 246.2 Hz)], 142.71, 142.65 [142.68 (d, *J* = 6.9 Hz)], 136.27, 135.01, 134.18, 133.82, 133.75 [133.79 (d, *J* = 8.1 Hz)], 131.06, 129.49, 129.29, 128.94, 123.23, 120.52, 119.48, 118.66, 118.64 [118.65 (d, *J* = 2.9 Hz)], 117.15, 116.96 [117.05 (d, *J* = 23.7 Hz)], 115.13, 114.95 [115.04 (d, *J* = 22.7 Hz)], 111.22, 109.79, 31.38 ppm.

^{19}F NMR (470 MHz, Chloroform-*d*) δ -113.78 – -115.19 (m) ppm.

HRMS (m/z): [M + H^+] calcd for $C_{21}H_{15}BrClFN$, 414.00604; found, 414.00522.

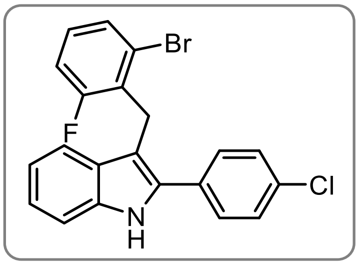

3-(2-bromo-6-fluorobenzyl)-2-(4-chlorophenyl)-1H-indole [**1am**]: Compound **1am** was prepared according to the general procedure A (5 mmol scale).

Yield: 29% (599 mg).

Eluent: Petroleum ether/Ethyl acetate (97/3, v/v).

TLC: R_f = 0.32 (95:5 petroleum ether:EtOAc).

Physical State: Yellow oil.

^{1}H NMR (500 MHz, Chloroform-*d*) δ 8.01 (s, 1H), 7.51 – 7.46 (m, 2H), 7.45 – 7.40 (m, 2H), 7.39 (dd, *J* = 7.8, 2.1 Hz, 1H), 7.34 (d, *J* = 8.2 Hz, 1H), 7.31 – 7.27 (m, 1H), 7.16 (tt, *J* = 8.1, 1.3 Hz, 1H), 7.04 – 6.98 (m, 2H), 6.93 (ddd, *J* = 9.6, 8.3, 1.2 Hz, 1H), 4.39 (s, 2H) ppm.

^{13}C NMR (126 MHz, $CDCl_3$) δ 162.76, 160.78 [161.77 (d, *J* = 249.3 Hz)], 136.02, 134.60, 134.01, 132.08, 130.17, 129.06, 128.85, 128.83 [128.84 (d, *J* = 3.4 Hz)], 128.71, 128.63, 128.59 [128.61 (d, *J* = 5.0 Hz)], 128.31, 128.18 [128.24 (d, *J* = 17.1 Hz)], 125.99, 125.95 [125.97 (d, *J* = 5.0 Hz)], 122.51, 120.05, 119.77, 119.75 [119.76 (d, *J* = 2.1 Hz)], 114.88, 114.70 [114.79 (d, *J* = 23.1 Hz)], 110.97, 109.95, 25.66, 25.63 [25.64 (d, *J* = 3.3 Hz)] ppm.

^{19}F NMR (470 MHz, Chloroform-*d*) δ -109.15 – -110.75 (m) ppm.

HRMS (m/z): [M + H^+] calcd for $C_{21}H_{15}NBrClF$, 414.0055; found, 414.0055.

CLASS-A

Optimized reaction condition:

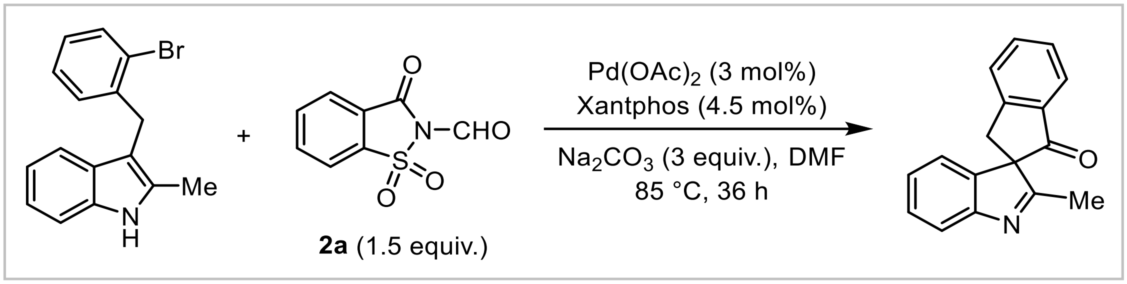

General procedure B: Procedure for CO insertion followed by spiro-cyclization

An oven-dried screw capped reaction tube (pictorial description below) with a magnetic stir-bar was charged with 3-(2-bromobenzyl)-2-methyl-1H-indole (0.5 mmol), *N*-formyl saccharin (**2a**, 1.5 equiv., 0.75 mmol), $Pd(OAc)_2$ (3 mol%, 0.015 mmol, 3.4 mg), Xantphos (4.5 mol%, 13 mg), and Na_2CO_3 (3 equiv., 159 mg). A screw cap fitted with a rubber septum was attached to the reaction tube, then degassed and refilled with argon; the process was repeated two additional times. Dry *N*,*N*-dimethyl formamide (DMF, 5 mL) was added to the reaction tube, and the reaction tube was placed in metal block and vigorously stirred at room temperature for 10 min followed by heating to 85 °C. The reaction mixture was cooled to room temperature after 36 h, diluted with 10 mL of ethyl acetate and 25 mL of water. The organic layer was separated and concentrated under vacuum. The crude mixture was purified by column chromatography using silica gel (100-200 mesh size) and petroleum ether/ethyl acetate as the eluent.

Description of Reaction Tube:

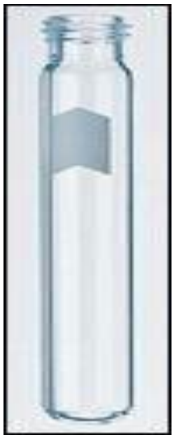

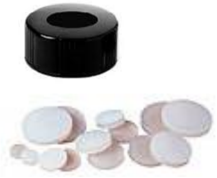

Pictorial description of reaction tube: Fisherbrand Disposable Borosilicate Glass Tubes (16*125mm) with Threaded End (Fisher Scientific Order No. 1495935A) [left]; Kimble Black Phenolic Screw Thread Closures with Open Tops (Fisher Scientific Order No. 033407E);Thermo Scientific National PTFE/Silicone Septa for Sample Screw Thread Caps (Fisher Scientific Order No. 03394A).

Characterization data for Class A products

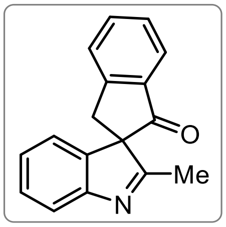

2'-methylspiro[indene-2,3'-indol]-1(3H)-one [**A1**]: Compound **A1** was prepared according to the general procedure B.

Yield: 82% (isolated 101.5 mg for 0.5 mmol scale); 71% (isolated 878 mg for 5 mmol scale).

Eluent: Petroleum ether/Ethyl acetate (70/30, v/v).

TLC: R_f = 0.23 (70:30 petroleum ether:EtOAc).

Physical state: yellowish solid.

^{1}H NMR (500 MHz, Acetone-*d*$_6$) δ 7.86 – 7.81 (m, 1H), 7.81 – 7.76 (m, 2H), 7.57 (ddt, *J* = 7.9, 7.1, 1.1 Hz, 1H), 7.51 (dt, *J* = 7.7, 0.9 Hz, 1H), 7.35 (td, *J* = 7.5, 1.4 Hz, 1H), 7.14 (tt, *J* = 7.3, 1.0 Hz, 1H), 7.10 (dt, *J* = 7.4, 1.5 Hz, 1H), 3.78 (d, *J* = 17.9 Hz, 1H), 3.54 (d, *J* = 17.8 Hz, 1H), 2.09 (s, 3H) ppm.

^{13}C NMR (126 MHz, Acetone-d$_6$) δ 201.07, 180.15, 157.95, 154.39, 142.23, 137.19, 136.71, 129.20, 129.06, 128.28, 126.39, 125.53, 122.22, 120.64, 73.50, 35.59, 16.40 ppm.

135 DEPT NMR (126 MHz, Acetone-d$_6$) δ 136.72, 129.20, 129.06, 128.28, 126.39, 125.52, 122.22, 120.64, 35.59, 16.40 ppm.

HRMS (m/z): [M + H$^+$] calcd for $C_{17}H_{14}ON$, 248.10699; found, 248.10695.

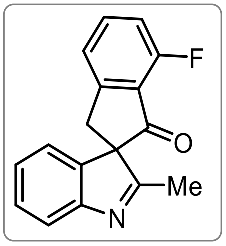

7-fluoro-2'-methylspiro[indene-2,3'-indol]-1(3H)-one [**A2**]: Compound **A2** was prepared according to the general procedure B (0.5 mmol scale).

Yield: 63% (isolated 83.6 mg).

Eluent: Petroleum ether/Ethyl acetate (70/30, v/v).

TLC: R_f = 0.24 (70:30 petroleum ether:EtOAc).

Physical state: Oily liquid.

^{1}H NMR (600 MHz, Methylene Chloride-*d*$_2$) δ 7.74 (td, *J* = 7.9, 5.0 Hz, 1H), 7.56 (d, *J* = 7.8 Hz, 1H), 7.46 (dd, *J* = 7.5, 1.1 Hz, 1H), 7.37 (td, *J* = 7.7, 1.2 Hz, 1H), 7.17 (td, *J* = 7.5, 1.0 Hz, 1H), 7.13 (t, *J* = 8.7 Hz, 1H), 7.06 (d, *J* = 7.4 Hz, 1H), 3.63 (d, *J* = 17.9 Hz, 1H), 3.50 (d, *J* = 17.9 Hz, 1H), 2.15 (s, 3H) ppm.

^{13}C NMR (151 MHz, CD$_2$Cl$_2$) δ 196.60, 196.59 [196.60 (d, *J* = 2.2 Hz)], 178.96, 160.56, 158.80 [159.68 (d, *J* = 264.7 Hz)], 156.70, 154.95, 154.94 [154.95 (d, *J* = 2.1 Hz)], 140.60, 137.90, 137.85 [137.87 (d, *J* = 8.7 Hz)], 128.77, 125.81, 124.27, 124.19 [124.23 (d, *J* = 13.2 Hz)], 122.98, 122.95 [122.96 (d, *J* = 4.3 Hz)], 121.28, 120.16, 115.06, 114.93 [114.99 (d, *J* = 18.8 Hz)], 72.85, 34.98, 34.97 [34.98 (d, *J* = 1.6 Hz)], 16.25 ppm.

^{19}F NMR (565 MHz, Methylene Chloride-*d*$_2$) δ -114.06 (dd, *J* = 9.4, 5.1 Hz) ppm.

HRMS (m/z): [M + H$^+$] calcd for $C_{17}H_{13}ONF$, 266.09757; found, 266.09757.

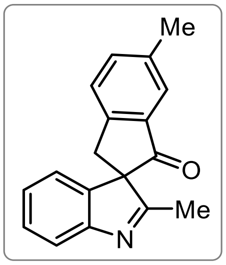

2',6-dimethylspiro[indene-2,3'-indol]-1(3H)-one [**A3**]: Compound **A3** was prepared according to the general procedure B (0.5 mmol scale).

Yield: 84% (isolated 109.6 mg).

Eluent: Petroleum ether/Ethyl acetate (70/30, v/v).

TLC: R$_f$ = 0.36 (70:30 petroleum ether:EtOAc).

Physical State: Amorphous solid.

^{1}H NMR (600 MHz, Chloroform-*d*) δ 7.64 (s, 1H), 7.60 (dt, *J* = 7.7, 0.9 Hz, 1H), 7.57 – 7.52 (m, 2H), 7.34 (td, *J* = 7.7, 1.2 Hz, 1H), 7.12 (td, *J* = 7.5, 1.1 Hz, 1H), 7.00 (ddd, *J* = 7.4, 1.2, 0.7 Hz, 1H), 3.59 – 3.41 (m, 2H), 2.46 (s, 3H), 2.16 (s, 3H) ppm.

^{13}C NMR (151 MHz, CDCl$_3$) δ 200.32, 179.66, 156.16, 150.16, 140.70, 138.45, 137.15, 136.60, 128.64, 126.60, 125.86, 125.33, 121.12, 120.23, 72.82, 34.89, 21.11, 16.39 ppm.

HRMS (m/z): [M + H$^+$] calcd for $C_{18}H_{16}ON$, 262.12264; found, 262.12264.

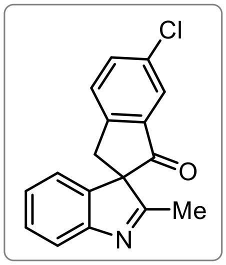

6-chloro-2'-methylspiro[indene-2,3'-indol]-1(3H)-one [**A4**]: Compound **A4** was prepared according to the general procedure B (0.5 mmol).

Yield: 74% (isolated 104.2 mg).

Eluent: Petroleum ether/Ethyl acetate (70/30, v/v).

TLC: R_f = 0.32 (70:30 petroleum ether:EtOAc).

Physical State: Amorphous solid.

^{1}H NMR (500 MHz, Acetone-*d*$_6$) δ 7.86 – 7.82 (m, 2H), 7.73 (t, *J* = 1.3 Hz, 1H), 7.51 (dt, *J* = 7.8, 1.0 Hz, 1H), 7.39 – 7.33 (m, 1H), 7.18 – 7.13 (m, 2H), 3.78 (d, *J* = 18.1 Hz, 1H), 3.55 (d, *J* = 17.9 Hz, 1H), 2.11 (s, 3H) ppm.

^{13}C NMR (126 MHz, Acetone) δ 200.08, 179.70, 157.87, 153.02, 141.83, 138.84, 136.47, 134.79, 130.00, 129.39, 126.52, 124.92, 122.38, 120.71, 73.91, 35.21, 16.42 ppm.

HRMS (m/z): [M + H$^+$] calcd for $C_{17}H_{13}ONCl$, 282.06802; found, 282.06844.

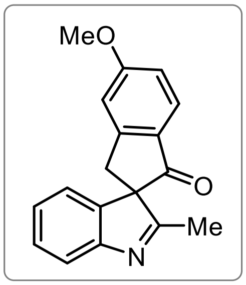

5-methoxy-2'-methylspiro[indene-2,3'-indol]-1(3H)-one [**A5**]: Compound **A5** was prepared according to the general procedure B (0.5 mmol scale).

Yield: 79% (isolated 109.5 mg).

Eluent: Petroleum ether/Ethyl acetate (60/40, v/v).

TLC: R_f = 0.23 (60:40 petroleum ether:EtOAc).

Physical State: Amorphous solid.

^{1}H NMR (600 MHz, Chloroform-*d*) δ 7.76 (d, *J* = 8.5 Hz, 1H), 7.58 (d, *J* = 7.8 Hz, 1H), 7.33 (td, *J* = 7.6, 1.2 Hz, 1H), 7.12 (td, *J* = 7.5, 1.1 Hz, 1H), 7.05 (d, *J* = 2.2 Hz, 1H), 7.01 (ddd, *J* = 8.6, 3.0, 1.6 Hz, 2H), 3.94 (s, 3H), 3.56 – 3.40 (m, 2H), 2.15 (s, 3H) ppm.

^{13}C NMR (151 MHz, $CDCl_3$) δ 198.31, 179.72, 166.18, 156.32, 155.88, 140.86, 129.55, 128.58, 127.10, 125.78, 121.11, 120.22, 116.33, 110.05, 72.64, 55.92, 35.15, 16.34 ppm.

135-DEPT NMR (151 MHz, $CDCl_3$) δ 128.58, 127.10, 125.78, 121.11, 120.22, 116.33, 110.04, 55.92, 35.15, 16.34 ppm.

HRMS (m/z): [M + H$^+$] calcd for $C_{18}H_{16}O_2N$, 278.11756; found, 278.11758.

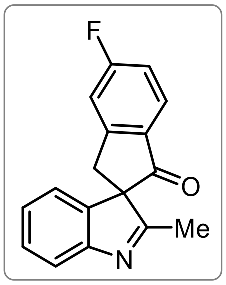

5-fluoro-2'-methylspiro[indene-2,3'-indol]-1(3H)-one [**A6**]: Compound **A6** was prepared according to the general procedure B (0.5 mmol scale).

Yield: 81% (isolated 107.4 mg).

Eluent: Petroleum ether/Ethyl acetate (70/30, v/v).

TLC: R_f = 0.24 (70:30 petroleum ether:EtOAc).

Physical State: Yellowish solid.

^{1}H NMR (600 MHz, Chloroform-*d*) δ 7.85 (dd, *J* = 8.5, 5.2 Hz, 1H), 7.60 (dt, *J* = 7.7, 0.9 Hz, 1H), 7.35 (td, *J* = 7.7, 1.2 Hz, 1H), 7.31 (dtd, *J* = 8.2, 1.4, 0.7 Hz, 1H), 7.23 – 7.18 (m, 1H), 7.14 (td, *J* = 7.5, 1.1 Hz, 1H), 7.00 (ddd, *J* = 7.4, 1.2, 0.7 Hz, 1H), 3.62 – 3.44 (m, 2H), 2.16 (s, 3H) ppm.

^{13}C NMR (151 MHz, $CDCl_3$) δ 198.35, 178.97, 168.58, 166.87 [167.73 (d, *J* = 259.0 Hz)], 156.17, 155.74, 155.67 [155.70 (d, *J* = 10.2 Hz)], 140.31, 132.74, 132.72 [132.73 (d, *J* = 2.0 Hz)], 128.87, 127.80, 127.73 [127.76 (d, *J* = 10.5 Hz)], 125.99, 121.07, 120.37, 116.87, 116.71 [116.79 (d, *J* = 23.9 Hz)], 113.82, 113.67 [113.75 (d, *J* = 22.7 Hz)], 72.52, 34.99, 34.97 [34.98 (d, *J* = 2.3 Hz)], 16.39 ppm.

^{19}F NMR (565 MHz, $CDCl_3$) δ -100.32, -100.33, -100.33, -100.34, -100.35, -100.36 [-100.34 (td, *J* = 8.5, 5.4 Hz)] ppm.

HRMS (m/z): [M + H$^+$] calcd for $C_{17}H_{13}ONF$, 266.09757; found, 266.09754.

Compound **A6** was resynthesized (**A6-resynthesized**) and retested in biological assays. The ^{1}H NMR spectra of the resynthesized batch is analogous to the original batch.

1H NMR (400 MHz, Chloroform-d) δ 7.85 (ddd, J = 8.3, 5.3, 2.6 Hz, 1H), 7.60 (dd, J = 7.9, 2.6 Hz, 1H), 7.34 (ddd, J = 19.5, 7.8, 4.2 Hz, 2H), 7.17 (dtd, J = 24.5, 8.0, 7.3, 2.5 Hz, 2H), 7.00 (dd, J = 7.6, 2.5 Hz, 1H), 3.60 (d, J = 15.6 Hz, 1H), 3.47 (d, J = 15.5 Hz, 1H), 2.17 (s, 3H).

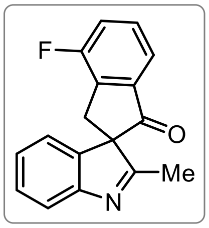

4-fluoro-2'-methylspiro[indene-2,3'-indol]-1(3H)-one [**A7**]: Compound **A7** was prepared according to the general procedure B (0.5 mmol scale).

Yield: 70% (isolated 92.9 mg).

Eluent: Petroleum ether/Ethyl acetate (70/30, v/v).

TLC: R_f = 0.24 (70:30 petroleum ether:EtOAc).

Physical State: Semi-solid.

^{1}H NMR (600 MHz, Chloroform-*d*) δ 7.67 (dd, *J* = 7.5, 0.9 Hz, 1H), 7.62 (dt, *J* = 7.7, 0.9 Hz, 1H), 7.54 – 7.49 (m, 1H), 7.44 (td, *J* = 8.3, 1.0 Hz, 1H), 7.37 (td, *J* = 7.7, 1.2 Hz, 1H), 7.16 (td, *J* = 7.5, 1.1 Hz, 1H), 7.02 (dt, *J* = 7.3, 0.9 Hz, 1H), 3.69 – 3.48 (m, 2H), 2.19 (s, 3H) ppm.

^{13}C NMR (151 MHz, $CDCl_3$) δ 199.04, 199.02, 178.81, 161.03, 159.36 [160.20 (d, *J* = 251.5 Hz)], 156.04, 140.12, 139.07, 139.04 [139.06 (d, *J* = 4.6 Hz)], 138.60, 138.47 [138.54 (d, *J* = 19.6 Hz)], 130.44, 130.40 [130.42 (d, *J* = 6.2 Hz)], 128.98, 126.10, 122.06, 121.93 [121.99 (d, *J* = 19.8 Hz)], 121.22, 121.19 [121.21 (d, *J* = 4.1 Hz)], 121.12, 120.43, 72.01, 30.96, 16.41 ppm.

135-DEPT NMR (151 MHz, $CDCl_3$) δ 130.44, 130.40 [130.42 (d, *J* = 6.3 Hz)], 128.98, 126.10, 122.06, 121.93 [122.00 (d, *J* = 19.9 Hz)], 121.22, 121.19 [121.21 (d, *J* = 4.1 Hz)], 121.12, 120.43, 30.96, 16.42 ppm.

^{19}F NMR (565 MHz, $CDCl_3$) δ -117.68, -117.68, -117.69, -117.70 [-117.69 (dd, *J* = 8.8, 4.5 Hz)] ppm.
HRMS (m/z): [M + H^+] calcd for $C_{17}H_{13}ONF$, 266.09757; found, 266.09753.

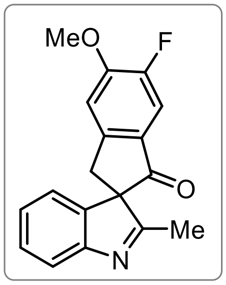

6-fluoro-5-methoxy-2'-methylspiro[indene-2,3'-indol]-1(3H)-one [**A8**]: Compound **A8** was prepared according to the general procedure B (0.5 mmol scale).

Yield: 62% (isolated 91.5 mg).

Eluent: Petroleum ether/Ethyl acetate (60/40, v/v).

TLC: R_f = 0.3 (60:40 petroleum ether:EtOAc).

Physical State: Yellowish solid.

^{1}H NMR (400 MHz, Chloroform-*d*) δ 7.59 (dd, *J* = 7.8, 1.0 Hz, 1H), 7.49 (d, *J* = 9.4 Hz, 1H), 7.34 (td, *J* = 7.6, 1.3 Hz, 1H), 7.18 – 7.09 (m, 2H), 7.01 (ddt, *J* = 7.5, 1.2, 0.5 Hz, 1H), 4.03 (s, 3H), 3.57 – 3.36 (m, 2H), 2.15 (s, 3H) ppm.

^{13}C NMR (151 MHz, Acetone-d_6) δ 198.85, 198.83 [198.84 (d, *J* = 2.8 Hz)], 180.20, 157.87, 155.71, 155.63 [155.67 (d, *J* = 12.0 Hz)], 154.45, 152.79 [153.62 (d, *J* = 249.3 Hz)], 152.62, 152.61 [152.61 (d, *J* = 2.2 Hz)], 142.14, 129.61, 129.57 [129.59 (d, *J* = 6.4 Hz)], 129.23, 126.42, 122.23, 120.63, 111.43, 111.42 [111.42 (d, *J* = 1.9 Hz)], 111.32, 111.20 [111.26 (d, *J* = 18.8 Hz)], 73.77, 57.25, 35.38, 16.27 ppm.

^{19}F NMR (470 MHz, Acetone-d_6) δ -135.29 – -135.37 (m) ppm.

HRMS (m/z): [M + H$^+$] calcd for $C_{18}H_{15}O_2NF$, 296.10813; found, 296.10839.

Compound **A8** was resynthesized (**A8-resynthesized**) and retested in biological assays. The ^{1}H NMR spectra of the resynthesized batch is analogous to the original batch.

^{1}H NMR (400 MHz, Chloroform-d) δ 7.65 (d, J = 7.7 Hz, 1H), 7.51 (d, J = 9.8 Hz, 1H), 7.38 (t, J = 7.8 Hz, 1H), 7.23 – 7.11 (m, 2H), 7.04 (d, J = 7.5 Hz, 1H), 4.05 (s, 3H), 3.58 (d, J = 17.6 Hz, 1H), 3.45 (d, J = 17.5 Hz, 1H), 2.24 (s, 3H).

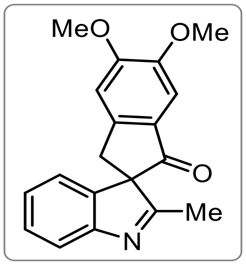

5,6-dimethoxy-2'-methylspiro[indene-2,3'-indol]-1(3H)-one [**A9**]: Compound **A9** was prepared according to the general procedure B (0.5 mmol scale).

Yield: 58% (isolated 89 mg).

Eluent: Petroleum ether/Ethyl acetate (55/45, v/v).

TLC: R_f = 0.36 (50:50 petroleum ether:EtOAc).

Physical State: Amorphous solid.

^{1}H NMR (500 MHz, Methylene Chloride-*d*$_2$) δ 7.54 (d, *J* = 7.7 Hz, 1H), 7.35 (td, *J* = 7.6, 1.2 Hz, 1H), 7.21 (s, 1H), 7.15 (td, *J* = 7.5, 1.1 Hz, 1H), 7.07 (s, 1H), 7.05 (dt, *J* = 7.3, 1.0 Hz, 1H), 3.99 (s, 2H), 3.89 (s, 2H), 3.51 (d, *J* = 17.8 Hz, 1H), 3.40 (d, *J* = 17.5 Hz, 1H), 2.10 (s, 2H) ppm.

^{13}C NMR (126 MHz, CD_2Cl_2) δ 198.72, 179.97, 156.76, 156.56, 150.19, 148.59, 141.12, 129.02, 128.38, 125.55, 121.20, 119.88, 107.72, 105.18, 72.87, 56.34, 56.08, 34.70, 16.00 ppm.

HRMS (m/z): [M + H$^+$] calcd for $C_{19}H_{18}O_3N$, 308.12812; found, 308.12822.

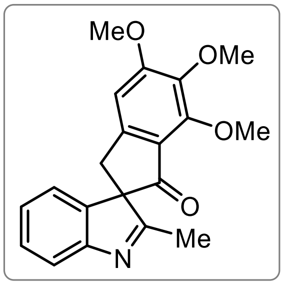

5,6,7-trimethoxy-2'-methylspiro[indene-2,3'-indol]-1(3H)-one [**A10**]: Compound **A10** was prepared according to the general procedure B (0.5 mmol scale).

Yield: 55% (isolated 92.8 mg).

Eluent: Petroleum ether/Ethyl acetate (50/50, v/v).

TLC: R_f = 0.25 (40:60 petroleum ether:EtOAc).

Physical State: Semi-solid.

^{1}H NMR (700 MHz, Methylene Chloride-d_2) δ 7.53 (dt, J = 7.8, 0.8 Hz, 1H), 7.35 (td, J = 7.6, 1.2 Hz, 1H), 7.15 (td, J = 7.4, 1.1 Hz, 1H), 7.06 (ddd, J = 7.4, 1.2, 0.6 Hz, 1H), 6.84 (s, 1H), 3.99 (s, 3H), 3.96 (s, 3H), 3.86 (s, 3H), 3.48 (dd, J = 17.5, 1.0 Hz, 1H), 3.35 (dd, J = 17.5, 1.0 Hz, 1H), 2.13 (s, 3H) ppm.

^{13}C NMR (176 MHz, CD_2Cl_2) δ 195.96, 180.00, 160.87, 156.71, 152.59, 150.93, 141.34, 141.25, 128.46, 125.60, 122.04, 121.17, 119.97, 104.07, 73.22, 61.93, 61.28, 56.52, 34.70, 16.16 ppm.

HRMS (m/z): [M + H$^+$] calcd for $C_{20}H_{20}O_4N$, 338.13868; found, 338.13883.

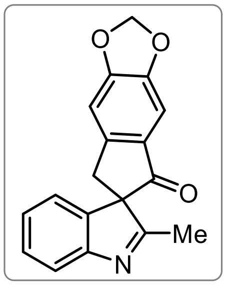

2'-methylspiro[indeno[5,6-d][1,3]dioxole-6,3'-indol]-5(7H)-one [**A11**]: Compound **A11** was prepared according to the general procedure B (0.5 mmol scale).

Yield: 68% (isolated 99 mg).

Eluent: Petroleum ether/Ethyl acetate (60/40, v/v).

TLC: R_f = 0.32 (60:40 petroleum ether:EtOAc).

Physical State:

^{1}H NMR (600 MHz, Acetone-d_6) δ 7.49 (dt, J = 7.8, 0.9 Hz, 1H), 7.33 (td, J = 7.4, 1.6 Hz, 1H), 7.19 (d, J = 1.0 Hz, 1H), 7.17 – 7.10 (m, 2H), 7.07 (s, 1H), 6.26 – 6.24 (m, 2H), 3.63 (dd, J = 17.7, 0.9 Hz, 1H), 3.41 (dd, J = 17.7, 0.9 Hz, 1H), 2.08 (s, 3H) ppm.

^{13}C NMR (151 MHz, Acetone) δ 198.52, 180.38, 157.85, 156.28, 152.31, 150.08, 142.27, 131.70, 129.13, 126.35, 122.17, 120.57, 107.05, 104.07, 103.31, 73.90, 35.32, 16.22 ppm.

HRMS (m/z): [M + H$^+$] calcd for $C_{18}H_{14}O_3N$, 292.09682; found, 292.09695.

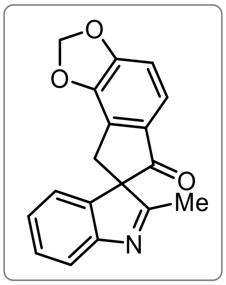

2'-methylspiro[indeno[4,5-d][1,3]dioxole-7,3'-indol]-6(8H)-one [**A12**]: Compound **A12** was prepared according to the general procedure B (0.5 mmol scale).

Yield: 49% (isolated 71.4 mg).

Eluent: Petroleum ether/Ethyl acetate (60/40, v/v).

TLC: R_f = 0.34 (60:40 petroleum ether:EtOAc).

Physical State: Semi-solid.

^{1}H NMR (500 MHz, Methylene Chloride-*d*$_2$) δ 7.55 (dt, *J* = 7.8, 0.9 Hz, 1H), 7.45 (d, *J* = 8.1 Hz, 1H), 7.36 (td, *J* = 7.7, 1.3 Hz, 1H), 7.16 (td, *J* = 7.5, 1.1 Hz, 1H), 7.07 (dt, *J* = 7.4, 1.0 Hz, 1H), 7.01 (dt, *J* = 8.1, 0.7 Hz, 1H), 6.19 (q, *J* = 1.2 Hz, 2H), 3.57 – 3.35 (m, 2H), 2.13 (s, 3H) ppm.

^{13}C NMR (126 MHz, CD_2Cl_2) δ 197.94, 179.45, 156.70, 153.61, 144.49, 141.04, 131.80, 131.74, 128.58, 125.68, 121.25, 120.86, 120.01, 109.37, 102.96, 72.50, 30.60, 16.15 ppm.

HRMS (m/z): [M + H^+] calcd for $C_{18}H_{14}O_3N$, 292.09682; found, 292.09674.

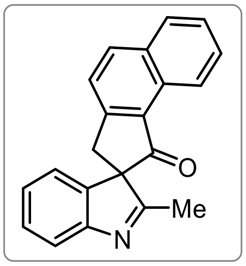

2'-methylspiro[cyclopenta[a]naphthalene-2,3'-indol]-1(3H)-one [**A13**]: Compound **A13** was prepared according to the general procedure B (0.5 mmol scale).

Yield: 48% (isolated 71.4 mg).

Eluent: Petroleum ether/Ethyl acetate (70/30, v/v).

TLC: R_f = 0.45 (70:30 petroleum ether:EtOAc).

Physical State: Oily liquid.

^{1}H NMR (500 MHz, Methylene Chloride-*d*$_2$) δ 9.00 (d, *J* = 8.4 Hz, 1H), 8.24 (d, *J* = 8.3 Hz, 1H), 8.02 (d, *J* = 8.2 Hz, 1H), 7.75 – 7.69 (m, 2H), 7.65 (ddd, *J* = 8.2, 6.9, 1.3 Hz, 1H), 7.61 (d, *J* = 7.8 Hz, 1H), 7.38 (td, *J* = 7.7, 1.2 Hz, 1H), 7.15 (td, *J* = 7.5, 1.1 Hz, 1H), 7.06 (dt, *J* = 7.5, 1.0 Hz, 1H), 3.73 (d, *J* = 18.2 Hz, 1H), 3.61 (d, *J* = 18.2 Hz, 1H), 2.15 (s, 3H) ppm.

^{13}C NMR (126 MHz, CD_2Cl_2) δ 200.47, 179.72, 156.95, 156.91, 141.01, 136.99, 133.03, 130.66, 129.79, 129.50, 128.51, 128.47, 127.22, 125.63, 124.01, 123.72, 121.22, 120.03, 72.96, 35.38, 16.16 ppm.

HRMS (m/z): [M + H^+] calcd for $C_{21}H_{16}ON$, 298.12264; found, 298.12255.

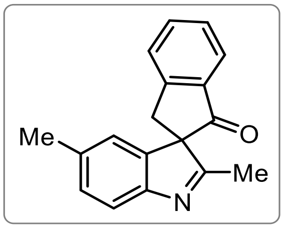

2',5'-dimethylspiro[indene-2,3'-indol]-1(3H)-one [**A14**]: Compound **A14** was prepared according to the general procedure B (0.5 mmol scale).

Yield: 79% (isolated 103.2 mg).

Eluent: Petroleum ether/Ethyl acetate (70/30, v/v).

TLC: R_f = 0.32 (70:30 petroleum ether:EtOAc).

Physical State: Amorphous solid.

^{1}H NMR (400 MHz, Chloroform-*d*) δ 7.84 (dd, *J* = 7.7, 1.2 Hz, 1H), 7.76 – 7.70 (m, 1H), 7.66 – 7.62 (m, 1H), 7.52 – 7.44 (m, 2H), 7.13 (ddt, *J* = 7.9, 1.6, 0.8 Hz, 1H), 6.82 – 6.78 (m, 1H), 3.67 – 3.41 (m, 2H), 2.28 (s, 3H), 2.13 (s, 3H) ppm.

^{13}C NMR (101 MHz, Chloroform-*d*) δ 200.58, 178.30, 154.07, 152.80, 140.78, 136.42, 135.77, 135.75, 129.22, 128.23, 126.92, 125.38, 121.84, 119.78, 72.23, 35.30, 21.32, 16.32 ppm.

HRMS (m/z): [M + H^+] calcd for $C_{18}H_{16}ON$, 262.12264; found, 262.12269.

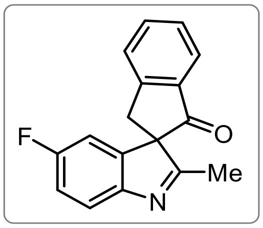

5'-fluoro-2'-methylspiro[indene-2,3'-indol]-1(3H)-one [**A15**]: Compound **A15** was prepared according to the general procedure B (0.5 mmol scale).

Yield: 76% (isolated 101 mg).

Eluent: Petroleum ether/Ethyl acetate (70/30, v/v).

TLC: R_f = 0.32 (70:30 petroleum ether:EtOAc).

Physical State: Solid.

^{1}H NMR (600 MHz, Methylene Chloride-d_2) δ 7.73 (ddd, *J* = 7.7, 1.3, 0.7 Hz, 1H), 7.67 (td, *J* = 7.5, 1.2 Hz, 1H), 7.59 (dt, *J* = 7.7, 0.9 Hz, 1H), 7.45 – 7.39 (m, 2H), 6.96 (ddd, *J* = 9.4, 8.5, 2.6 Hz, 1H), 6.66 (dd, *J* = 8.0, 2.6 Hz, 1H), 3.60 – 3.37 (m, 2H), 2.00 (s, 3H) ppm.

^{13}C NMR (151 MHz, CD_2Cl_2) δ 199.71, 179.36, 179.34 [179.35 (d, *J* = 3.4 Hz)], 161.95, 160.33 [161.14 (d, *J* = 243.8 Hz), 152.86, 152.85 [152.85 (d, *J* = 2.5 Hz)], 152.82, 142.59, 142.53, 136.11, 135.98, 128.32, 127.09, 125.23, 120.71, 120.65 [120.68 (d, *J* = 9.0 Hz)], 115.02, 114.86 [114.94 (d, *J* = 23.7 Hz)], 109.17, 109.00 [109.09 (d, *J* = 25.3 Hz)], 72.94, 72.92 [72.93 (d, *J* = 2.6 Hz)], 35.02, 16.13 ppm.

^{19}F NMR (565 MHz, CD_2Cl_2) δ -117.28, -117.28, -117.29, -117.30, -117.31, -117.32, -117.33, -117.33 [δ -117.31 (qd, *J* = 8.8, 4.4 Hz)] ppm.

HRMS (m/z): [M + H^+] calcd for $C_{17}H_{13}ONF$, 266.09757; found, 266.09757.

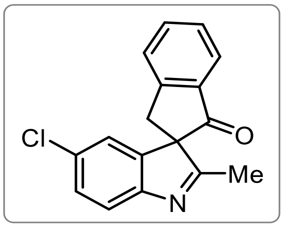

5'-chloro-2'-methylspiro[indene-2,3'-indol]-1(3H)-one [**A16**]: Compound **A16** was prepared according to the general procedure B (0.2 mmol scale).

Yield: 69% (isolated 97.2 mg).

Eluent: Petroleum ether/Ethyl acetate (70/30, v/v).

TLC: R_f = 0.35 (70:30 petroleum ether:EtOAc).

Physical State: Yellowish solid.

^{1}H NMR (600 MHz, Methylene Chloride-d_2) δ 7.73 (d, *J* = 7.7 Hz, 1H), 7.68 (td, *J* = 7.5, 1.2 Hz, 1H), 7.60 (dt, *J* = 7.7, 1.0 Hz, 1H), 7.46 – 7.42 (m, 1H), 7.40 (d, *J* = 8.2 Hz, 1H), 7.25 (dd, *J* = 8.3, 2.1 Hz, 1H), 6.92 (d, *J* = 2.1 Hz, 1H), 3.55 (d, *J* = 17.7 Hz, 1H), 3.41 (d, *J* = 17.7 Hz, 1H), 2.02 (s, 3H) ppm.

^{13}C NMR (151 MHz, CD_2Cl_2) δ 199.52, 180.15, 155.35, 152.82, 142.59, 136.06, 136.01, 131.12, 128.56, 128.34, 127.09, 125.27, 121.75, 120.88, 72.75, 34.93, 16.20 ppm.

HRMS (m/z): [M + H^+] calcd for $C_{17}H_{13}ONCl$, 282.06802; found, 282.06822.

Compound **A16** was resynthesized (**A16-resynthesized**) and retested in biological assays. The ^{1}H NMR spectra of the resynthesized batch is analogous to the original batch.

^{1}H NMR (400 MHz, Methylene Chloride-d2) δ 7.82 (ddt, J = 7.7, 1.4, 0.7 Hz, 1H), 7.80 – 7.74 (m, 1H), 7.69 (dp, J = 7.7, 0.9 Hz, 1H), 7.56 – 7.46 (m, 2H), 7.34 (ddd, J = 8.4, 2.1, 0.7 Hz, 1H), 7.01 (dt, J = 2.2, 0.6 Hz, 1H), 3.64 (d, J = 17.7 Hz, 1H), 3.50 (d, J = 17.7 Hz, 1H), 2.11 (s, 3H) ppm.

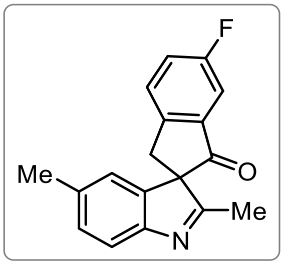

6-fluoro-2',5'-dimethylspiro[indene-2,3'-indol]-1(3H)-one [**A17**]: Compound **A17** was prepared according to the general procedure B (0.5 mmol scale).

Yield: 81% (isolated 113 mg).

Eluent: Petroleum ether/Ethyl acetate (70/30, v/v).

TLC: R_f = 0.5 (70:30 petroleum ether:EtOAc).

Physical State: Amorphous solid.

^{1}H NMR (500 MHz, Methylene Chloride-d_2) δ 7.66 (ddq, *J* = 8.4, 4.6, 0.9 Hz, 1H), 7.52 – 7.45 (m, 2H), 7.43 (d, *J* = 7.9 Hz, 1H), 7.17 (ddd, *J* = 7.9, 1.7, 0.8 Hz, 1H), 6.85 (dt, *J* = 1.6, 0.8 Hz, 1H), 3.58 (d, *J* = 17.5 Hz, 1H), 3.45 (d, *J* = 17.5 Hz, 1H), 2.30 (s, 3H), 2.10 (s, 3H) ppm.

^{13}C NMR (126 MHz, CD_2Cl_2) δ <u>199.92, 199.90</u> [199.91 (d, *J* = 3.0 Hz)], 177.92, <u>163.69, 161.71</u> [162.70 (d, *J* = 248.7 Hz)], 154.30, <u>148.67, 148.65</u> [148.66 (d, *J* = 2.2 Hz)], 140.67, <u>138.14, 138.08</u> [138.11 (d, *J* = 7.4 Hz)], 135.89, 129.20, <u>128.59, 128.53</u> [128.56 (d, *J* = 8.0 Hz)], <u>123.58, 123.39</u> [123.48 (d, *J* = 23.8 Hz)], 121.98, 119.58, <u>110.81, 110.64</u> [110.72 (d, *J* = 22.3 Hz)], 73.08, 34.60, 21.04, 16.12 ppm.

^{19}F NMR (470 MHz, Methylene Chloride-d_2) δ -110.41 – -117.35 (m) ppm.

HRMS (m/z): [M + H^+] calcd for $C_{18}H_{15}ONF$, 280.11322; found, 280.11328.

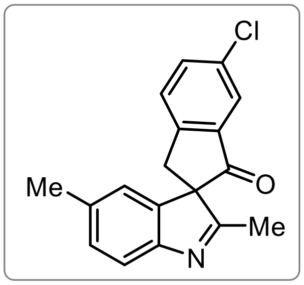

6-chloro-2',5'-dimethylspiro[indene-2,3'-indol]-1(3H)-one [**A18**]: Compound **A18** was prepared according to the general procedure B (0.5 mmol scale).

Yield: 75% (isolated 111 mg).

Eluent: Petroleum ether/Ethyl acetate (70/30, v/v).

TLC: R_f = 0.5 (70:30 petroleum ether:EtOAc).

Physical State: Oily liquid.

^{1}H NMR (600 MHz, Methylene Chloride-*d*$_2$) δ 7.78 (d, *J* = 1.8 Hz, 1H), 7.72 (dd, *J* = 8.2, 2.1 Hz, 1H), 7.63 (dq, *J* = 8.3, 0.9 Hz, 1H), 7.43 (d, *J* = 7.9 Hz, 1H), 7.17 (ddd, *J* = 7.9, 1.7, 0.9 Hz, 1H), 6.83 (dt, *J* = 1.6, 0.8 Hz, 1H), 3.58 (d, *J* = 17.8 Hz, 1H), 3.44 (d, *J* = 17.8 Hz, 1H), 2.30 (s, 3H), 2.09 (s, 3H) ppm.

^{13}C NMR (151 MHz, CD_2Cl_2) δ 199.66, 177.77, 154.44, 151.35, 140.70, 137.94, 135.93, 135.73, 134.54, 129.26, 128.36, 124.76, 121.99, 119.65, 72.78, 34.82, 21.07, 16.18 ppm.

HRMS (m/z): [M + H$^+$] calcd for $C_{18}H_{15}ONCl$, 296.08367; found, 296.08354.

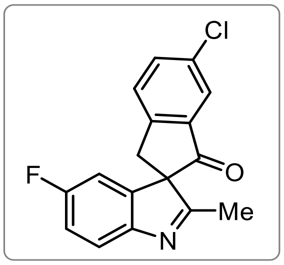

6-chloro-5'-fluoro-2'-methylspiro[indene-2,3'-indol]-1(3H)-one [**A19**]: Compound **A19** was prepared according to the general procedure B (0.5 mmol scale).

Yield: 59% (isolated 88.4 mg).

Eluent: Petroleum ether/Ethyl acetate (70/30, v/v).

TLC: R_f = 0.45 (70:30 petroleum ether:EtOAc).

Physical State: Yellow solid.

^{1}H NMR (500 MHz, Chloroform-*d*) δ 7.82 (d, *J* = 2.1 Hz, 1H), 7.72 (dd, *J* = 8.2, 2.1 Hz, 1H), 7.63 – 7.58 (m, 1H), 7.55 (dd, *J* = 8.5, 4.6 Hz, 1H), 7.06 (td, *J* = 8.9, 2.6 Hz, 1H), 6.73 (dd, *J* = 7.7, 2.5 Hz, 1H), 3.59 (d, *J* = 17.8 Hz, 1H), 3.45 (d, *J* = 17.8 Hz, 1H), 2.16 (s, 3H) ppm.

^{13}C NMR (126 MHz, $CDCl_3$) δ 198.45, 178.86, 162.52, 160.56 [161.54 (d, *J* = 245.6 Hz)], 152.24, 150.67, 141.89, 141.81 [141.85 (d, *J* = 9.0 Hz)], 137.77, 136.32, 135.28, 128.30, 125.47, 121.34, 121.27 [121.30 (d, *J* = 8.9 Hz)], 115.84, 115.66 [115.75 (d, *J* = 23.5 Hz)], 109.43, 109.23 [109.33 (d, *J* = 25.3 Hz)], 73.24, 73.22 [73.23 (d, *J* = 2.5 Hz)], 34.87, 16.61 ppm.

^{19}F NMR (470 MHz, $CDCl_3$) δ -115.70 ppm.

HRMS (m/z): [M + H^+] calcd for $C_{17}H_{12}ONClF$, 300.05860; found, 300.05872.

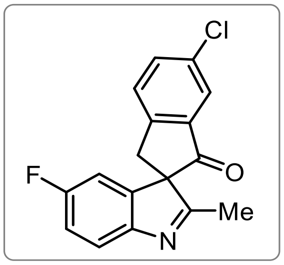

5'-chloro-2',6-dimethylspiro[indene-2,3'-indol]-1(3H)-one [**A20**]: Compound **A20** was prepared according to the general procedure B (0.5 mmol).

Yield: 72% (108 mg isolated).

Eluent: Petroleum ether/Ethyl acetate (70/30, v/v).

TLC: R_f = 0.40 (70:30 petroleum ether:EtOAc).

Physical State:

^{1}H NMR (500 MHz, Methylene Chloride-*d_2*) δ 7.63 – 7.54 (m, 3H), 7.48 (d, *J* = 8.3 Hz, 1H), 7.33 (dd, *J* = 8.3, 2.1 Hz, 1H), 7.00 (d, *J* = 2.1 Hz, 1H), 3.58 (d, *J* = 17.5 Hz, 1H), 3.44 (d, *J* = 17.6 Hz, 1H), 2.46 (s, 3H), 2.10 (s, 3H) ppm.

^{13}C NMR (126 MHz, CD_2Cl_2) δ 199.58, 180.40, 155.29, 150.24, 142.70, 138.73, 137.35, 136.27, 131.12, 128.54, 126.73, 125.14, 121.78, 120.86, 73.16, 34.61, 20.85, 16.22 ppm.

HRMS (m/z): [M + H^+] calcd for $C_{18}H_{15}ONCl$, 296.08367; found, 296.08387.

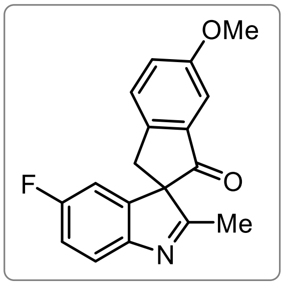

5'-fluoro-6-methoxy-2'-methylspiro[indene-2,3'-indol]-1(3H)-one [**A21**]: Compound **A21** was prepared according to the general procedure B (0.5 mmol).

Yield: 76% (112 mg isolated).

Eluent: Petroleum ether/Ethyl acetate (70/30, v/v).

TLC: R_f = 0.35 (60:40 petroleum ether:EtOAc).

Physical State:

^{1}H NMR (500 MHz, Methylene Chloride-*d*$_2$) δ 7.59 – 7.54 (m, 1H), 7.50 (dd, *J* = 8.5, 4.6 Hz, 1H), 7.35 (dd, *J* = 8.4, 2.6 Hz, 1H), 7.25 (d, *J* = 2.6 Hz, 1H), 7.05 (ddd, *J* = 9.3, 8.5, 2.6 Hz, 1H), 6.77 (dd, *J* = 8.0, 2.6 Hz, 1H), 3.87 (s, 3H), 3.55 (d, *J* = 17.5 Hz, 1H), 3.41 (d, *J* = 17.5 Hz, 1H), 2.09 (s, 3H) ppm.

^{13}C NMR (126 MHz, CD_2Cl_2) δ 199.62, <u>179.44, 179.41</u> [179.43 (d, *J* = 3.6 Hz)], <u>162.11, 160.17</u> [161.14 (d, *J* = 243.8 Hz)], 160.14, <u>152.86, 152.84</u> [152.85 (d, *J* = 2.4 Hz)], 145.60, <u>142.61, 142.54</u> [142.58 (d, *J* = 9.1 Hz)], 137.42, 127.71, 125.26, <u>120.69, 120.61</u> [120.65 (d, *J* = 9.0 Hz)], <u>115.02, 114.83</u> [114.93 (d, *J* = 23.5 Hz)], <u>109.23, 109.03</u> [109.13 (d, *J* = 25.2 Hz)], 106.32, <u>73.71, 73.69</u> [73.70 (d, *J* = 2.3 Hz)], 55.79, 34.30, 16.12 ppm.

^{19}F NMR (470 MHz, Methylene Chloride-*d*$_2$) δ -117.21 – -117.33 (m) ppm.

HRMS (m/z): [M + H^+] calcd for $C_{18}H_{15}O_2NF$, 296.10813; found, 296.10815.

Compound **A21** was resynthesized (**A21-resynthesized**) and retested in biological assays. The ^{1}H NMR spectra of the resynthesized batch is analogous to the original batch.

^{1}H NMR (400 MHz, Methylene Chloride-d2) δ 7.56 (dq, J = 8.4, 0.8 Hz, 1H), 7.50 (dd, J = 8.5, 4.7 Hz, 1H), 7.35 (dd, J = 8.4, 2.6 Hz, 1H), 7.25 (d, J = 2.5 Hz, 1H), 7.05 (ddd, J = 9.3, 8.5, 2.6 Hz, 1H), 6.76 (ddd, J = 8.0, 2.6, 0.4 Hz, 1H), 3.88 (s, 3H), 3.55 (d, J = 17.8 Hz, 1H), 3.40 (d, J = 17.4 Hz, 1H), 2.09 (s, 3H) ppm.

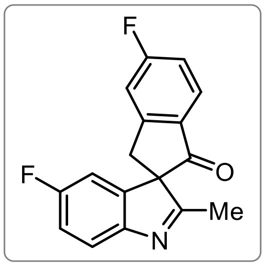

5,5'-difluoro-2'-methylspiro[indene-2,3'-indol]-1(3H)-one [**A22**]: Compound **A22** was prepared according to the general procedure B (0.5 mmol).

Yield: 81% (114.7 mg isolated).

Eluent: Petroleum ether/Ethyl acetate (70/30, v/v).

TLC: R_f = 0.35 (70:30 petroleum ether:EtOAc).

Physical State: Brown solid.

^{1}H NMR (600 MHz, Methylene Chloride-*d*$_2$) δ 7.84 (dd, *J* = 8.5, 5.3 Hz, 1H), 7.50 (dd, *J* = 8.5, 4.7 Hz, 1H), 7.35 (ddt, *J* = 8.3, 2.2, 1.1 Hz, 1H), 7.24 (tt, *J* = 8.6, 1.5 Hz, 1H), 7.06 (ddd, *J* = 9.3, 8.5, 2.6 Hz, 1H), 6.77 (dd, *J* = 7.9, 2.6 Hz, 1H), 3.62 (d, *J* = 18.0 Hz, 1H), 3.48 (d, *J* = 18.0 Hz, 1H), 2.10 (s, 3H) ppm.

^{13}C NMR (151 MHz, CD_2Cl_2) δ 197.85, <u>179.00, 178.98</u> [178.99 (d, *J* = 3.4 Hz)], <u>168.71, 167.00</u> [167.86 (d, *J* = 258.2 Hz)], <u>162.03, 160.42</u> [161.23 (d, *J* = 244.0 Hz)], <u>155.89, 155.82</u> [155.85 (d, *J* = 10.5 Hz)], <u>152.83, 152.82</u> [152.83 (d, *J* = 2.5 Hz)], <u>142.31, 142.25</u> [142.28 (d, *J* = 9.3 Hz)], <u>132.58, 132.57</u> [132.57 (d, *J* = 1.7 Hz)], <u>127.74, 127.67</u> [127.71 (d, *J* = 10.6 Hz)], <u>120.85, 120.79</u> [120.82 (d, *J* = 8.9 Hz)], <u>116.89, 116.73</u> [116.81 (d, *J* = 24.0 Hz)], <u>115.23, 115.07</u> [115.15 (d, *J* = 23.6 Hz)], <u>113.92, 113.77</u> [113.84 (d, *J* = 22.7 Hz)], <u>109.23, 109.07</u> [109.15 (d, *J* = 25.3 Hz)], <u>73.07, 73.05</u> [73.06 (d, *J* = 2.5 Hz)], <u>34.82, 34.81</u> [34.81 (d, *J* = 2.2 Hz)], 16.14 ppm.

^{19}F NMR (565 MHz, Methylene Chloride-*d*$_2$) δ -101.18 (td, *J* = 8.5, 5.2 Hz), -117.09 (td, *J* = 8.5, 4.6 Hz) ppm.

HRMS (m/z): [M + H^+] calcd for $C_{17}H_{12}ONF_2$, 284.08815; found, 284.08822.

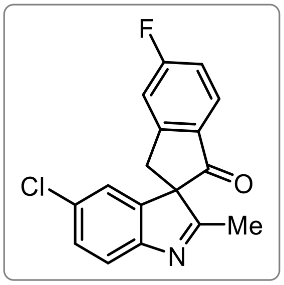

5'-chloro-5-fluoro-2'-methylspiro[indene-2,3'-indol]-1(3H)-one [**A23**]: Compound **A23** was prepared according to the general procedure B (0.5 mmol).

Yield: 73% (109.4 mg isolated).

Eluent: Petroleum ether/Ethyl acetate (70/30, v/v).

TLC: R_f = 0.35 (70:30 petroleum ether:EtOAc).

Physical State: Yellowish solid.

^{1}H NMR (600 MHz, Methylene Chloride-*d*$_2$) δ 7.84 (dd, *J* = 8.5, 5.3 Hz, 1H), 7.49 (d, *J* = 8.2 Hz, 1H), 7.35 (td, *J* = 8.2, 7.7, 1.7 Hz, 2H), 7.24 (tt, *J* = 8.6, 1.5 Hz, 1H), 7.03 (d, *J* = 2.1 Hz, 1H), 3.62 (d, *J* = 18.0 Hz, 1H), 3.48 (d, *J* = 18.0 Hz, 1H), 2.12 (s, 3H) ppm.

^{13}C NMR (151 MHz, CD_2Cl_2) δ 197.64, 179.75, <u>168.72, 167.01</u> [167.86 (d, *J* = 258.2 Hz)], <u>155.88, 155.81</u> [155.84 (d, *J* = 10.5 Hz)], 155.34, 142.32, <u>132.53, 132.52</u> [132.53 (d, *J* = 1.7 Hz)], 131.26, 128.75, <u>127.77, 127.70</u> [127.74 (d, *J* = 10.7 Hz)], 121.80, 121.01, <u>116.91, 116.75</u> [116.83 (d, *J* = 24.1 Hz)], <u>113.92, 113.77</u> [113.85 (d, *J* = 22.8 Hz)], 72.88, <u>34.73, 34.72</u> [34.72 (d, *J* = 2.2 Hz)], 16.21 ppm.

^{19}F NMR (565 MHz, Methylene Chloride-*d*$_2$) δ -101.09 (td, *J* = 8.5, 5.2 Hz) ppm.

HRMS (m/z): [M + H$^+$] calcd for $C_{17}H_{12}ONClF$, 300.05860; found, 300.05886.

Compound **A23** was resynthesized (**A23-resynthesized**) and retested in biological assays. The ^{1}H NMR spectra of the resynthesized batch is analogous to the original batch.

^{1}H NMR (400 MHz, Methylene Chloride-d2) δ 7.84 (dd, J = 8.5, 5.3 Hz, 1H), 7.50 (d, J = 8.2 Hz, 1H), 7.38 – 7.32 (m, 2H), 7.27 – 7.20 (m, 1H), 7.02 (dd, J = 1.7, 1.1 Hz, 1H), 3.62 (d, J = 18.8 Hz, 1H), 3.48 (d, J = 17.2 Hz, 1H), 2.12 (s, 3H) ppm.

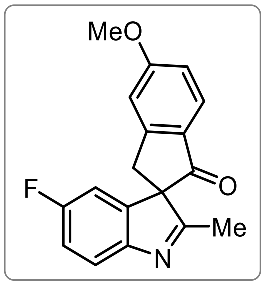

5'-fluoro-5-methoxy-2'-methylspiro[indene-2,3'-indol]-1(3H)-one [**A24**]: Compound **A24** was prepared according to the general procedure B (0.5 mmol scale).

Yield: 78% (115 mg).

Eluent: Petroleum ether/Ethyl acetate (70/30, v/v).

TLC: R_f = 0.35 (60:40 petroleum ether:EtOAc).

Physical State: Amorphous solid.

^{1}H NMR (600 MHz, Methylene Chloride-*d*$_2$) δ 7.74 (d, *J* = 8.6 Hz, 1H), 7.49 (dd, *J* = 8.5, 4.6 Hz, 1H), 7.11 – 7.08 (m, 1H), 7.07 – 7.01 (m, 2H), 6.77 (dd, *J* = 8.0, 2.6 Hz, 1H), 3.95 (s, 3H), 3.57 (d, *J* = 17.7 Hz, 1H), 3.43 (d, *J* = 17.7 Hz, 1H), 2.10 (s, 3H) ppm.

^{13}C NMR (151 MHz, CD_2Cl_2) δ 197.56, 179.81, 179.78 [179.79 (d, *J* = 3.4 Hz)], 166.42, 162.00, 160.39 [161.20 (d, *J* = 243.7 Hz)], 155.98, 152.84, 152.83 [152.83 (d, *J* = 2.5 Hz)], 142.89, 142.83 [142.86 (d, *J* = 9.3 Hz)], 129.25, 126.92, 120.65, 120.60 [120.62 (d, *J* = 8.8 Hz)], 116.45, 114.96, 114.80 [114.88 (d, *J* = 23.6 Hz)], 110.16, 109.19, 109.03 [109.11 (d, *J* = 24.9 Hz)], 73.21, 73.20 [73.21 (d, *J* = 2.3 Hz)], 56.04, 34.97, 16.08 ppm.

^{19}F NMR (565 MHz, Methylene Chloride-*d*$_2$) δ -117.48 (td, *J* = 8.7, 4.6 Hz) ppm.

HRMS (m/z): [M + H$^+$] calcd for $C_{18}H_{15}O_2NF$, 296.10813; found, 296.10824.

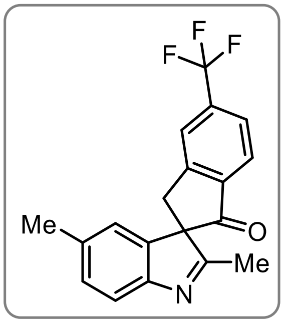

2',5'-dimethyl-5-(trifluoromethyl)spiro[indene-2,3'-indol]-1(3H)-one [**A25**]: Compound **A25** was prepared according to the general procedure B (0.2 mmol scale).

Yield: 59% (38.9 mg isolated).

Eluent: Petroleum ether/Ethyl acetate (70/30, v/v).

TLC: R_f = 0.5 (70:30 petroleum ether:EtOAc).

Physical State: Yellowish oily liquid.

^{1}H NMR (500 MHz, Chloroform-*d*) δ 7.53 (ddd, *J* = 8.2, 2.0, 1.0 Hz, 1H), 7.50 (dt, *J* = 1.8, 0.9 Hz, 1H), 7.46 (dd, *J* = 8.4, 1.9 Hz, 1H), 7.42 (s, 1H), 7.34 (d, *J* = 8.3 Hz, 1H), 7.32 (d, *J* = 8.3 Hz, 1H), 3.40 (dt, *J* = 16.6, 1.2 Hz, 1H), 3.02 (d, *J* = 16.6 Hz, 1H), 2.36 (s, 3H), 1.54 (s, 3H) ppm.

^{13}C NMR (126 MHz, $CDCl_3$) δ 204.62, 157.38, 149.04, 138.81, 133.89, 133.02, 125.95, 125.92, 125.89, 125.86 [125.90 (q, *J* = 4.0 Hz)], 126.03, 125.77, 125.51, 125.26 [125.64 (q, *J* = 32.5 Hz)], **125.18**, **124.84**, 127.75, 125.59, 123.43, 121.27 [124.51 (q, *J* = 271.6 Hz)], 122.97, 122.94, 122.91, 122.88 [122.92 (q, *J* = 3.7 Hz)], 114.96, 114.52, 76.10, 37.55, 24.51, 20.87 ppm.

135-DEPT NMR (126 MHz, $CDCl_3$) δ 138.63, 125.77, 125.74, 125.71, 125.68 [125.72 (q, *J* = 3.9 Hz)], 125.00, 122.79, 122.76, 122.73, 122.70 [122.74 (q, *J* = 3.6 Hz)], 114.78, 114.34, 37.38, 24.33, 20.70 ppm.

^{19}F NMR (470 MHz, $CDCl_3$) δ -61.55 ppm.

HRMS (m/z): [M + H^+] calcd for $C_{19}H_{15}ONF_3$, 330.11003; found, 330.11000.

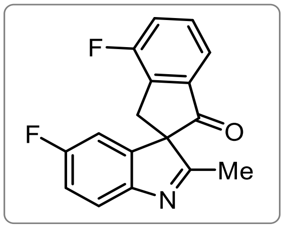

4,5'-difluoro-2'-methylspiro[indene-2,3'-indol]-1(3H)-one [**A26**]: Compound **A26** was prepared according to the general procedure B (0.2 mmol scale).

Yield: 62% (35.1 mg).

Eluent: Petroleum ether/Ethyl acetate (70/30, v/v).

TLC: R_f = 0.35 (70:30 petroleum ether:EtOAc).

Physical State: Amorphous white solid.

^{1}H NMR (500 MHz, Methylene Chloride-d_2) δ 7.65 (dd, *J* = 7.5, 1.0 Hz, 1H), 7.58 – 7.49 (m, 2H), 7.47 (ddd, *J* = 8.9, 8.1, 1.0 Hz, 1H), 7.07 (ddd, *J* = 9.4, 8.5, 2.6 Hz, 1H), 6.78 (dd, *J* = 7.9, 2.6 Hz, 1H), 3.65 (dd, *J* = 18.0, 0.8 Hz, 1H), 3.50 (dd, *J* = 18.0, 0.8 Hz, 1H), 2.11 (s, 3H) ppm.

^{13}C NMR (126 MHz, CD_2Cl_2) δ 198.59, 198.56 [198.57 (d, *J* = 2.7 Hz),], 178.68, 178.66 [178.67 (d, *J* = 3.6 Hz)], 162.18, 160.24 [161.18 (d, *J* = 244.3 Hz)], 161.26, 159.27 [d, *J* = 251.0 Hz], 152.86, 152.84 [152.85 (d, *J* = 2.3 Hz)], 142.12, 142.05 [142.09 (d, *J* = 9.1 Hz)], 138.83, 138.79 [138.81 (d, *J* = 4.5 Hz)], 138.63, 138.47 [138.55 (d, *J* = 19.6 Hz)], 130.62, 130.57 [130.60 (d, *J* = 6.4 Hz)], 122.19, 122.03 [122.11 (d, *J* = 19.9 Hz)], 121.15, 121.12 [121.14 (d, *J* = 4.0 Hz)], 120.93, 120.86 [120.90 (d, *J* = 9.0 Hz)], 115.34, 115.15 [115.25 (d, *J* = 23.6 Hz)], 109.29, 109.09 [109.19 (d, *J* = 25.3 Hz)], 72.50, 72.48 [72.49 (d, *J* = 2.3 Hz)], 30.74, 16.19 ppm.

^{19}F NMR (470 MHz, Methylene Chloride-d_2) δ -116.96 (td, *J* = 8.6, 4.6 Hz), -118.46 (dd, *J* = 8.7, 4.7 Hz) ppm.

HRMS (m/z): $[M + H^+]$ calcd for $C_{17}H_{12}ONF_2$, 284.08815; found, 284.08820.

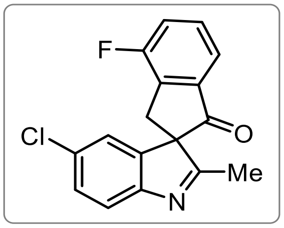

5'-chloro-4-fluoro-2'-methylspiro[indene-2,3'-indol]-1(3H)-one [**A27**]: Compound **A27** was prepared according to the general procedure B (0.5 mmol scale).

Yield: 74% (111 mg isolated).

Eluent: Petroleum ether/Ethyl acetate (70/30, v/v).

TLC: R_f = 0.35 (70:30 petroleum ether:EtOAc).

Physical State: Yellow solid.

^{1}H NMR (500 MHz, Methylene Chloride-d_2) δ 7.65 (dd, *J* = 7.5, 1.0 Hz, 1H), 7.54 (tdt, *J* = 7.5, 4.6, 0.9 Hz, 1H), 7.50 (d, *J* = 8.3 Hz, 1H), 7.47 (ddd, *J* = 8.9, 8.0, 1.0 Hz, 1H), 7.35 (dd, *J* = 8.3, 2.1 Hz, 1H), 7.04 (d, *J* = 2.1 Hz, 1H), 3.65 (d, *J* = 18.0 Hz, 1H), 3.51 (d, *J* = 18.0 Hz, 1H), 2.13 (s, 3H) ppm.

^{13}C NMR (126 MHz, CD_2Cl_2) δ 198.39, 198.36 [198.37 (d, *J* = 2.7 Hz)], 179.45, 161.24, 159.24 [160.24 (d, *J* = 251.1 Hz)], 155.33, 142.12, 138.76, 138.73 [138.75 (d, *J* = 4.6 Hz)], 138.62, 138.47, 131.30, 130.64, 130.59 [130.61 (d, *J* = 6.4 Hz)], 128.84, 122.22, 122.06 [122.14 (d, *J* = 19.7 Hz)], 121.82, 121.18, 121.15 [121.17 (d, *J* = 3.8 Hz)], 121.06, 72.30, 30.66, 16.25 ppm.

^{19}F NMR (470 MHz, Methylene Chloride-*d*$_2$) δ -118.38 (dd, *J* = 8.7, 4.6 Hz) ppm.

HRMS (m/z): [M + H$^+$] calcd for $C_{17}H_{12}ONClF$, 300.05860; found, 300.05902.

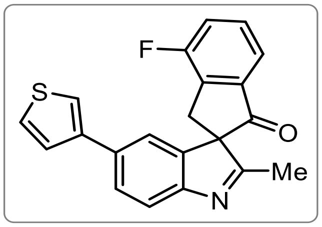

4-fluoro-2'-methyl-5'-(thiophen-3-yl)spiro[indene-2,3'-indol]-1(3H)-one [**A28**]: Compound **A28** was prepared according to the general procedure B (0.2 mmol scale).

Yield: 52% (36 mg isolated).

Eluent: Petroleum ether/Ethyl acetate (60/40, v/v).

TLC: R_f = 0.35 (60:40 petroleum ether:EtOAc).

Physical State: Yellowish liquid.

^{1}H NMR (500 MHz, Acetonitrile-*d*$_3$) δ 7.68 (dd, *J* = 8.1, 1.8 Hz, 1H), 7.64 (dd, *J* = 7.3, 1.3 Hz, 1H), 7.59 (dt, *J* = 7.3, 3.6 Hz, 1H), 7.57 – 7.53 (m, 2H), 7.53 (dd, *J* = 3.0, 1.4 Hz, 1H), 7.48 (d, *J* = 1.8 Hz, 1H), 7.43 (dd, *J* = 5.1, 3.0 Hz, 1H), 7.39 (dd, *J* = 5.1, 1.4 Hz, 1H), 3.73 (d, *J* = 18.1 Hz, 1H), 3.63 (d, *J* = 18.2 Hz, 1H), 2.09 (s, 3H) ppm.

^{13}C NMR (126 MHz, CD_3CN) δ 201.07, 201.05 [201.06 (d, *J* = 2.8 Hz)], 180.82, 162.62, 160.63 [161.62 (d, *J* = 249.2 Hz)], 157.24, 143.00, 142.84, 140.98, 140.82 [140.90 (d, *J* = 20.0 Hz)], 140.29, 140.25 [140.27 (d, *J* = 5.0 Hz)], 134.86, 131.92, 131.87 [131.89 (d, *J* = 6.4 Hz)], 128.10, 128.00, 127.61, 123.36, 123.20 [123.28 (d, *J* = 20.3 Hz)], 122.20, 122.17 [122.18 (d, *J* = 3.9 Hz)], 121.86, 121.36, 121.25, 73.62, 31.68, 16.92 ppm.

^{19}F NMR (470 MHz, Acetonitrile-*d*$_3$) δ -119.75 (dd, *J* = 8.8, 4.8 Hz) ppm.

HRMS (m/z): [M + H$^+$] calcd for $C_{21}H_{16}O_2NS$, 348.08542; found, 348.08541.

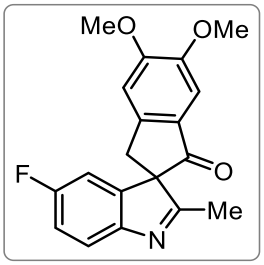

5'-fluoro-5,6-dimethoxy-2'-methylspiro[indene-2,3'-indol]-1(3H)-one [**A29**]: Compound **A29** was prepared according to the general procedure B (0.5 mmol scale).

Yield: 68% (111 mg isolated).

Eluent: Petroleum ether/Ethyl acetate (60/40, v/v).

TLC: R_f = 0.45 (50:50 petroleum ether:EtOAc).

Physical State: Amorphous colorless solid.

^{1}H NMR (500 MHz, Methylene Chloride-*d*$_2$) δ 7.49 (dd, *J* = 8.5, 4.7 Hz, 1H), 7.20 (s, 1H), 7.06 (s, 1H), 7.06 – 7.01 (m, 1H), 6.78 (dd, *J* = 8.0, 2.6 Hz, 1H), 3.99 (s, 3H), 3.90 (s, 3H), 3.52 (d, *J* = 17.6 Hz, 1H), 3.38 (d, *J* = 17.5 Hz, 1H), 2.08 (s, 3H) ppm.

^{13}C NMR (126 MHz, CD_2Cl_2) δ 197.93, 179.93, 179.90 [179.92 (d, *J* = 3.6 Hz)], 162.13, 160.19 [161.16 (d, *J* = 243.8 Hz)], 156.74, 152.85, 152.83 [152.84 (d, *J* = 2.2 Hz)], 150.32, 148.41, 142.86, 142.79 [142.82 (d, *J* = 9.1 Hz)], 128.81, 120.61, 120.54 [120.57 (d, *J* = 9.0 Hz)], 114.91, 114.72 [114.82 (d, *J* = 23.6 Hz)], 109.19, 108.99 [109.09 (d, *J* = 25.2 Hz)], 107.72, 105.27, 73.26, 73.24 [73.25 (d, *J* = 2.4 Hz)], 56.39, 56.13, 34.65, 16.00 ppm.

^{19}F NMR (470 MHz, Methylene Chloride-*d*$_2$) δ -117.53 (td, *J* = 8.6, 4.7 Hz) ppm.

HRMS (m/z): [M + H$^+$] calcd for $C_{19}H_{17}O_3NF$, 326.11870; found, 326.11872.

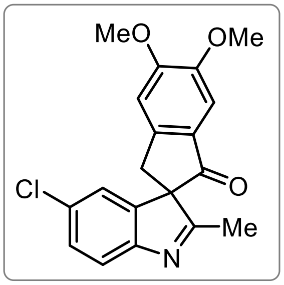

5'-chloro-5,6-dimethoxy-2'-methylspiro[indene-2,3'-indol]-1(3H)-one [**A30**]: Compound **A30** was prepared according to the general procedure B (0.5 mmol scale).

Yield: 63% (108 mg isolated).

Eluent: Petroleum ether/Ethyl acetate (60/40, v/v).

TLC: R_f = 0.45 (50:50 petroleum ether:EtOAc).

Physical State: Colorless solid.

^{1}H NMR (500 MHz, Methylene Chloride-*d*$_2$) δ 7.48 (d, *J* = 8.3 Hz, 1H), 7.32 (dd, *J* = 8.3, 2.1 Hz, 1H), 7.20 (s, 1H), 7.06 (s, 1H), 7.03 (d, *J* = 2.1 Hz, 1H), 4.00 (s, 3H), 3.90 (s, 3H), 3.52 (dd, *J* = 17.5, 0.9 Hz, 1H), 3.38 (dd, *J* = 17.5, 1.0 Hz, 1H), 2.10 (s, 3H) ppm.

^{13}C NMR (126 MHz, CD_2Cl_2) δ 197.73, 180.69, 156.77, 155.37, 150.34, 148.42, 142.86, 131.05, 128.77, 128.44, 121.77, 120.80, 107.73, 105.29, 73.09, 56.40, 56.14, 34.57, 16.09 ppm.

HRMS (m/z): [M + H^+] calcd for $C_{19}H_{17}O_3NCl$, 342.08915; found, 342.08915.

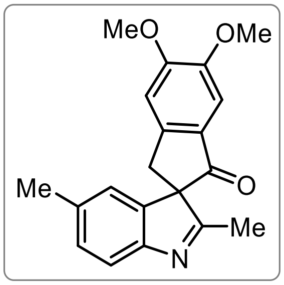

5,6-dimethoxy-2',5'-dimethylspiro[indene-2,3'-indol]-1(3H)-one [**A31**]: Compound **A31** was prepared according to the general procedure B (0.5 mmol scale).

Yield: 72% (116 mg isolated).

Eluent: Petroleum ether/Ethyl acetate (60/40, v/v).

TLC: R_f = 0.5 (50:50 petroleum ether:EtOAc).

Physical State: Yellowish solid.

^{1}H NMR (500 MHz, Methylene Chloride-*d*$_2$) δ 7.41 (d, *J* = 7.8 Hz, 1H), 7.20 (s, 1H), 7.15 (ddd, *J* = 7.9, 1.7, 0.8 Hz, 1H), 7.06 (s, 1H), 6.85 (dt, *J* = 1.6, 0.7 Hz, 1H), 3.99 (s, 3H), 3.90 (s, 3H), 3.49 (d, *J* = 17.5 Hz, 1H), 3.38 (d, *J* = 17.5 Hz, 1H), 2.30 (s, 3H), 2.07 (s, 3H) ppm.

^{13}C NMR (126 MHz, CD_2Cl_2) δ 199.03, 178.87, 156.54, 154.50, 150.19, 148.65, 141.28, 135.61, 129.08, 128.90, 121.98, 119.41, 107.72, 105.18, 72.69, 56.36, 56.11, 34.84, 21.08, 15.97 ppm.

HRMS (m/z): [M + H^+] calcd for $C_{20}H_{20}O_3N$, 322.14377; found, 322.14372.

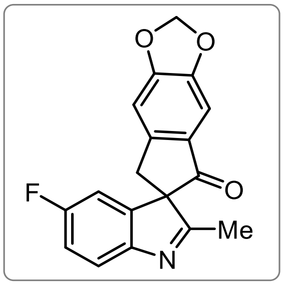

5'-fluoro-2'-methylspiro[indeno[5,6-d][1,3]dioxole-6,3'-indol]-5(7H)-one [**A32**]: Compound **A32** was prepared according to the general procedure B (0.5 mmol scale).

Yield: 70% (108 mg isolated).

Eluent: Petroleum ether/Ethyl acetate (60/40, v/v).

TLC: R_f = 0.5 (50:50 petroleum ether:EtOAc).

Physical State: Brownish oil.

^{1}H NMR (500 MHz, Methylene Chloride-*d*$_2$) δ 7.39 (dd, *J* = 8.5, 4.7 Hz, 1H), 7.02 (s, 1H), 6.97 – 6.93 (m, 1H), 6.93 – 6.91 (m, 1H), 6.70 (dd, *J* = 8.0, 2.6 Hz, 1H), 6.08 – 6.05 (m, 2H), 3.39 (d, *J* = 18.0 Hz, 1H), 3.25 (d, *J* = 18.1 Hz, 1H), 2.00 (s, 3H) ppm.

^{13}C NMR (126 MHz, CD_2Cl_2) δ 197.25, 179.57, 179.54 [179.55 (d, *J* = 3.5 Hz)], 162.10, 160.17 [161.13 (d, *J* = 243.8 Hz)], 155.52, 152.76, 152.74 [152.75 (d, *J* = 2.3 Hz)], 150.80, 149.20, 142.60, 142.53 [142.57 (d, *J* = 9.1 Hz)], 130.70, 120.62, 120.55 [120.58 (d, *J* = 8.8 Hz)], 114.97, 114.78 [114.87 (d, *J* = 23.6 Hz)], 109.18, 108.98 [109.08 (d, *J* = 25.2 Hz)], 105.93, 103.12, 103.10, 73.35, 73.33 [73.34 (d, *J* = 2.5 Hz)], 34.70, 15.98 ppm.

^{19}F NMR (470 MHz, Methylene Chloride-*d*$_2$) δ -117.37 (td, *J* = 8.7, 4.7 Hz) ppm.

HRMS (m/z): [M + H$^+$] calcd for $C_{18}H_{13}O_3NF$, 310.08740; found, 310.08746.

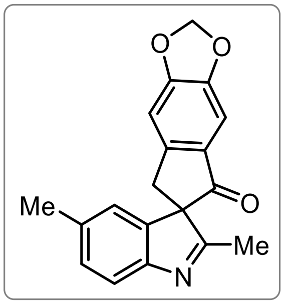

2',5'-dimethylspiro[indeno[5,6-d][1,3]dioxole-6,3'-indol]-5(7H)-one [**A33**]: Compound **A33** was prepared according to the general procedure B (0.5 mmol scale).

Yield: 79% (121 mg isolated).

Eluent: Petroleum ether/Ethyl acetate (60/40, v/v).

TLC: R_f = 0.5 (50:50 petroleum ether:EtOAc).

Physical State: Brownish oil.

^{1}H NMR (600 MHz, Methylene Chloride-*d_2*) δ 7.45 (d, *J* = 7.9 Hz, 1H), 7.19 (ddd, *J* = 7.9, 1.7, 0.8 Hz, 1H), 7.16 (s, 1H), 7.06 (q, *J* = 0.8 Hz, 1H), 6.91 (dt, *J* = 1.6, 0.7 Hz, 1H), 6.21 – 6.17 (m, 2H), 3.50 (d, *J* = 17.5 Hz, 1H), 3.38 (d, *J* = 17.5 Hz, 1H), 2.34 (s, 3H), 2.12 (s, 3H). [little amount of DMF present]

^{13}C NMR (151 MHz, CD_2Cl_2) δ 198.29, 178.53, 155.32, 154.35, 151.01, 149.07, 141.03, 135.65, 130.98, 128.95, 121.94, 119.40, 105.90, 103.04, 102.99, 72.80, 34.90, 21.03, 15.92 ppm.

HRMS (m/z): [M + H^+] calcd for $C_{19}H_{16}O_3N$, 306.11247; found, 306.11261.

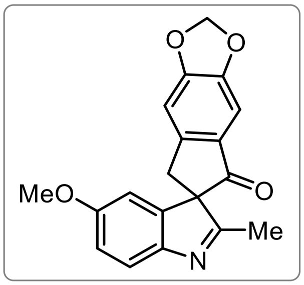

5'-methoxy-2'-methylspiro[indeno[5,6-d][1,3]dioxole-6,3'-indol]-5(7H)-one [**A34**]: Compound **A34** was prepared according to the general procedure B (0.5 mmol scale).

Yield: 72% (116 mg isolated).

Eluent: Petroleum ether/Ethyl acetate (50/50, v/v).

TLC: R_f = 0.45 (30:70 petroleum ether:EtOAc).

Physical State: Amorphous solid.

^{1}H NMR (600 MHz, Methylene Chloride-*d_2*) δ 7.43 (d, *J* = 8.5 Hz, 1H), 7.12 (s, 1H), 7.02 (s, 1H), 6.86 (dd, *J* = 8.5, 2.5 Hz, 1H), 6.62 (d, *J* = 2.5 Hz, 1H), 6.16 (d, *J* = 2.5 Hz, 2H), 3.73 (s, 3H), 3.47 (d, *J* = 17.6 Hz, 1H), 3.34 (d, *J* = 17.6 Hz, 1H), 2.07 (s, 3H) ppm.

^{13}C NMR (151 MHz, CD_2Cl_2) δ 198.07, 177.41, 158.37, 155.40, 150.97, 150.06, 149.15, 142.34, 130.97, 120.16, 113.19, 107.87, 105.95, 103.12, 103.05, 73.10, 55.69, 35.09, 15.87 ppm.

HRMS (m/z): [M + H^+] calcd for $C_{19}H_{16}O_4N$, 322.10738; found, 322.10749.

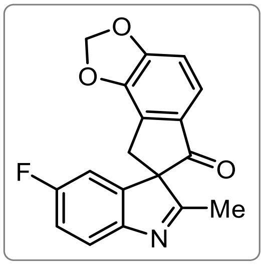

5'-fluoro-2'-methylspiro[indeno[4,5-d][1,3]dioxole-7,3'-indol]-6(8H)-one [**A35**]: Compound **A35** was prepared according to the general procedure B (0.2 mmol scale).

Yield: 47% (29 mg isolated).

Eluent: Petroleum ether/Ethyl acetate (60/40, v/v).

TLC: R_f = 0.4 (60:40 petroleum ether:EtOAc).

Physical State: Yellowish oil.

^{1}H NMR (600 MHz, Methylene Chloride-d_2) δ 7.50 (dd, J = 8.5, 4.7 Hz, 1H), 7.46 (d, J = 8.1 Hz, 1H), 7.05 (ddd, J = 9.3, 8.5, 2.6 Hz, 1H), 7.01 (d, J = 8.1 Hz, 1H), 6.80 (dd, J = 8.0, 2.6 Hz, 1H), 6.20 (s, 2H), 3.53 (d, J = 17.8 Hz, 1H), 3.39 (d, J = 17.7 Hz, 1H), 2.12 (s, 3H) ppm.

^{13}C NMR (151 MHz, CD_2Cl_2) δ 197.05, <u>179.52, 179.50</u> [179.51 (d, J = 3.4 Hz)], <u>162.06, 160.45</u> [161.26 (d, J = 243.8 Hz)], 153.83, 152.59, 144.57, <u>142.69, 142.63</u> [142.66 (d, J = 8.9 Hz)], 131.52, 131.44, 121.13, <u>120.75, 120.69</u> [120.72 (d, J = 8.9 Hz)], <u>115.16, 115.01</u> [115.08 (d, J = 23.6 Hz)], 109.57, <u>109.28, 109.11</u> [109.20 (d, J = 25.3 Hz)], 103.06, <u>72.91, 72.90</u> [72.91 (d, J = 2.3 Hz)], 30.57, 16.13 ppm.

^{19}F NMR (565 MHz, Methylene Chloride-d_2) δ -117.19 ppm.

HRMS (m/z): [M + H^+] calcd for $C_{18}H_{13}O_3NF$, 310.08740; found, 310.08751.

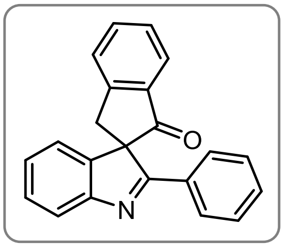

2'-phenylspiro[indene-2,3'-indol]-1(3H)-one [**A36**]: Compound **A36** was prepared according to the general procedure B (0.5 mmol scale).

Yield: 31% (48 mg isolated).

Eluent: Petroleum ether/Ethyl acetate (80/20, v/v).

TLC: R_f = 0.6 (70:30 petroleum ether:EtOAc).

Physical State: Amorphous colorless solid.

^{1}H NMR (700 MHz, Methylene Chloride-*d*$_2$) δ 7.92 (d, *J* = 7.7 Hz, 1H), 7.82 (td, *J* = 7.5, 1.2 Hz, 1H), 7.72 (dd, *J* = 12.4, 7.8 Hz, 2H), 7.68 – 7.64 (m, 2H), 7.59 (t, *J* = 7.5 Hz, 1H), 7.47 – 7.43 (m, 1H), 7.41 (td, *J* = 7.6, 1.1 Hz, 1H), 7.36 (t, *J* = 7.8 Hz, 2H), 7.16 (td, *J* = 7.5, 1.0 Hz, 1H), 6.93 (dd, *J* = 7.5, 1.1 Hz, 1H), 3.85 (d, *J* = 17.8 Hz, 1H), 3.54 (d, *J* = 17.8 Hz, 1H) ppm.

^{13}C NMR (176 MHz, CD_2Cl_2) δ 200.29, 177.18, 155.93, 152.78, 142.23, 136.27, 135.93, 131.79, 131.15, 128.96, 128.76, 128.36, 127.80, 127.45, 126.28, 125.66, 121.08, 120.20, 70.58, 37.58 ppm.

HRMS (m/z): [M + H$^+$] calcd for $C_{22}H_{16}ON$, 310.12264; found, 310.12304.

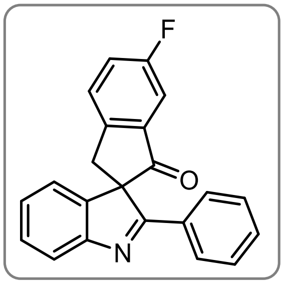

6-fluoro-2'-phenylspiro[indene-2,3'-indol]-1(3H)-one [**A37**]: Compound **A37** was prepared according to the general procedure B (0.2 mmol scale).

Yield: 39% (26 mg isolated).

Eluent: Petroleum ether/Ethyl acetate (80/20, v/v).

TLC: R_f = 0.6 (70:30 petroleum ether:EtOAc).

Physical State: Colorless solid.

^{1}H NMR (600 MHz, Acetone-*d*$_6$) δ 7.91 (dd, *J* = 8.3, 4.8 Hz, 1H), 7.75 – 7.68 (m, 4H), 7.57 (dd, *J* = 7.5, 2.6 Hz, 1H), 7.51 – 7.47 (m, 1H), 7.45 – 7.40 (m, 3H), 7.20 (td, *J* = 7.5, 1.0 Hz, 1H), 7.03 (dt, *J* = 7.4, 0.9 Hz, 1H), 3.89 (dt, *J* = 17.9, 1.5 Hz, 1H), 3.60 (dt, *J* = 17.8, 1.2 Hz, 1H) ppm.

^{13}C NMR (151 MHz, Acetone) δ <u>200.04, 200.02</u> [200.03 (d, *J* = 3.1 Hz)], 177.46, <u>164.62, 162.98</u> [163.80 (d, *J* = 247.4 Hz)], 156.92, <u>149.70, 149.68</u> [149.69 (d, *J* = 2.0 Hz)], 143.13, <u>138.97, 138.92</u> [138.94 (d, *J* = 7.6 Hz)], 132.77, 132.16, <u>130.66, 130.61</u> [130.63 (d, *J* = 8.3 Hz)], 129.95, 129.73, 128.65, 127.36, <u>124.69, 124.53</u> [124.61 (d, *J* = 23.9 Hz)], 121.91, 121.23, <u>111.64, 111.49</u> [111.56 (d, *J* = 22.6 Hz)], 72.12, 37.66 ppm.

^{19}F NMR (565 MHz, Acetone-*d*$_6$) δ -115.14 (td, *J* = 8.1, 5.7 Hz) ppm.

HRMS (m/z): [M + H$^+$] calcd for $C_{22}H_{15}ONF$, 328.11322; found, 328.11349.

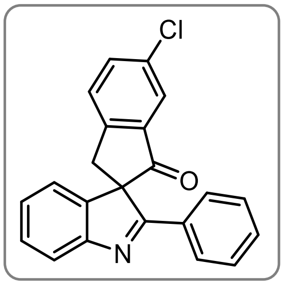

6-chloro-2'-phenylspiro[indene-2,3'-indol]-1(3H)-one [**A38**]: Compound **A38** was prepared according to the general procedure B (0.2 mmol scale).

Yield: 28% (19 mg isolated).

Eluent: Petroleum ether/Ethyl acetate (80/20, v/v).

TLC: R_f = 0.62 (70:30 petroleum ether:EtOAc).

Physical State: Off-white solid.

^{1}H NMR (600 MHz, Chloroform-*d*) δ 7.92 (d, *J* = 2.0 Hz, 1H), 7.79 – 7.74 (m, 2H), 7.66 – 7.60 (m, 3H), 7.48 – 7.39 (m, 2H), 7.38 – 7.34 (m, 2H), 7.15 (td, *J* = 7.5, 1.0 Hz, 1H), 6.92 (dt, *J* = 7.4, 1.0 Hz, 1H), 3.85 – 3.42 (m, 2H) ppm.

^{13}C NMR (151 MHz, $CDCl_3$) δ 199.08, 176.93, 155.91, 150.77, 141.74, 138.08, 136.17, 135.19, 131.68, 131.53, 129.29, 129.26, 128.63, 128.08, 126.68, 125.79, 121.65, 120.28, 70.98, 37.43 ppm.

HRMS (m/z): [M + H^+] calcd for $C_{22}H_{15}ONCl$, 344.08367; found, 344.08394.

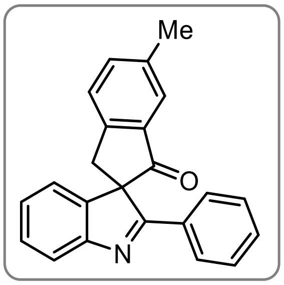

6-methyl-2'-phenylspiro[indene-2,3'-indol]-1(3H)-one [**A39**]: Compound **A39** was prepared according to the general procedure B (0.2 mmol scale).

Yield: 45% (29 mg isolated).

Eluent: Petroleum ether/Ethyl acetate (80/20, v/v).

TLC: R_f = 0.45 (80:20 petroleum ether:EtOAc).

Physical State: Off-white solid.

^{1}H NMR (500 MHz, Methylene Chloride-d_2) δ 7.74 – 7.70 (m, 2H), 7.69 – 7.62 (m, 3H), 7.59 (dd, *J* = 7.8, 0.9 Hz, 1H), 7.46 – 7.42 (m, 1H), 7.40 (td, *J* = 7.6, 1.2 Hz, 1H), 7.38 – 7.34 (m, 2H), 7.16 (td, *J* = 7.5, 1.1 Hz, 1H), 6.94 (dt, *J* = 7.4, 0.9 Hz, 1H), 3.79 (d, *J* = 17.7 Hz, 1H), 3.48 (d, *J* = 17.7 Hz, 1H) ppm.

^{13}C NMR (126 MHz, CD_2Cl_2) δ 200.36, 177.27, 155.91, 150.16, 142.36, 138.70, 137.25, 136.44, 131.84, 131.15, 128.97, 128.72, 127.80, 127.08, 126.26, 125.53, 121.04, 120.21, 70.93, 37.23, 20.92 ppm.

135-DEPT NMR (126 MHz, CD_2Cl_2) δ 137.22, 131.11, 128.93, 128.68, 127.77, 127.05, 126.23, 125.50, 121.01, 120.18, 37.20, 20.89 ppm.

HRMS (m/z): [M + H^+] calcd for $C_{23}H_{18}ON$, 324.13829; found, 324.13841.

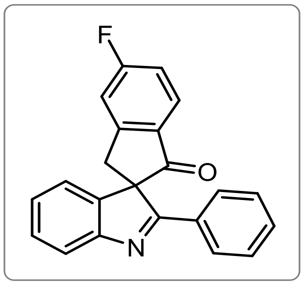

5-fluoro-2'-phenylspiro[indene-2,3'-indol]-1(3H)-one [**A40**]: Compound **A40** was prepared according to the general procedure B (0.2 mmol scale).

Yield: 35% (23 mg isolated).

Eluent: Petroleum ether/Ethyl acetate (80/20, v/v).

TLC: R_f = 0.5 (80:20 petroleum ether:EtOAc).

Physical State: Yellowish oil.

^{1}H NMR (500 MHz, Methylene Chloride-d_2) δ 7.94 (dd, *J* = 8.5, 5.3 Hz, 1H), 7.72 (dt, *J* = 7.8, 0.9 Hz, 1H), 7.68 – 7.63 (m, 2H), 7.48 – 7.41 (m, 2H), 7.41 – 7.35 (m, 3H), 7.30 (td, *J* = 8.6, 2.2 Hz, 1H), 7.18 (td, *J* = 7.5, 1.1 Hz, 1H), 6.96 (dt, *J* = 7.5, 0.9 Hz, 1H), 3.83 (d, *J* = 18.3 Hz, 1H), 3.52 (d, *J* = 18.0 Hz, 1H) ppm.

^{13}C NMR (126 MHz, CD_2Cl_2) δ 198.44, 176.80, <u>168.89, 166.84</u> [167.87 (d, *J* = 257.6 Hz)], 155.89, <u>155.86, 155.77</u> [155.81 (d, *J* = 10.5 Hz)], 141.94, <u>132.66, 132.65</u> [132.65 (d, *J* = 1.8 Hz)], 131.68, 131.27, 129.05, 128.94, <u>128.12, 128.04</u> [128.08 (d, *J* = 10.8 Hz)], 127.78, 126.39, 121.16, 120.21, <u>116.91, 116.72</u> [116.82 (d, *J* = 24.1 Hz)], <u>114.26, 114.08</u> [114.17 (d, *J* = 22.9 Hz)], 70.66, <u>37.37, 37.35</u> [37.36 (d, *J* = 2.2 Hz)] ppm.

^{19}F NMR (470 MHz, Methylene Chloride-d_2) δ -101.51 (td, *J* = 8.7, 5.7 Hz) ppm.

HRMS (m/z): [M + H^+] calcd for $C_{22}H_{15}ONF$, 328.11322; found, 328.11333.

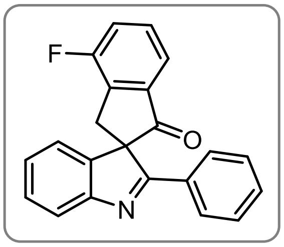

4-fluoro-2'-phenylspiro[indene-2,3'-indol]-1(3H)-one [**A41**]: Compound **A41** was prepared according to the general procedure B (0.2 mmol scale).

Yield: 29% (19 mg isolated).

Eluent: Petroleum ether/Ethyl acetate (80/20, v/v).

TLC: R_f = 0.32 (80:20 petroleum ether:EtOAc).

Physical State: Brown oil.

^{1}H NMR (700 MHz, Acetone-*d*$_6$) δ 7.77 – 7.70 (m, 5H), 7.68 (ddd, *J* = 9.0, 7.4, 1.7 Hz, 1H), 7.51 – 7.47 (m, 1H), 7.46 – 7.40 (m, 3H), 7.20 (td, *J* = 7.5, 1.1 Hz, 1H), 7.07 (dt, *J* = 7.4, 0.9 Hz, 1H), 3.90 (d, *J* = 18.2 Hz, 1H), 3.66 (d, *J* = 18.1 Hz, 1H) ppm.

^{13}C NMR (176 MHz, Acetone) δ 199.60, 199.59 [199.60 (d, *J* = 2.7 Hz)], 177.34, 162.17, 160.75 [161.46 (d, *J* = 249.9 Hz)], 156.89, 142.92, 139.91, 139.89 [139.90 (d, *J* = 4.6 Hz)], 139.45, 139.33 [139.39 (d, *J* = 20.0 Hz)], 132.68, 132.19, 131.99, 131.96 [131.98 (d, *J* = 6.6 Hz)], 129.98, 129.81, 128.65, 127.41, 123.14, 123.03 [123.09 (d, *J* = 20.1 Hz)], 122.19, 122.17 [122.18 (d, *J* = 3.9 Hz)], 121.94, 121.33, 71.01, 33.90 ppm.

^{19}F NMR (470 MHz, Acetone-*d*$_6$) δ -118.93 (dd, *J* = 9.8, 3.8 Hz) ppm.

HRMS (m/z): [M + H^+] calcd for $C_{22}H_{15}ONF$, 328.11322; found, 328.11332.

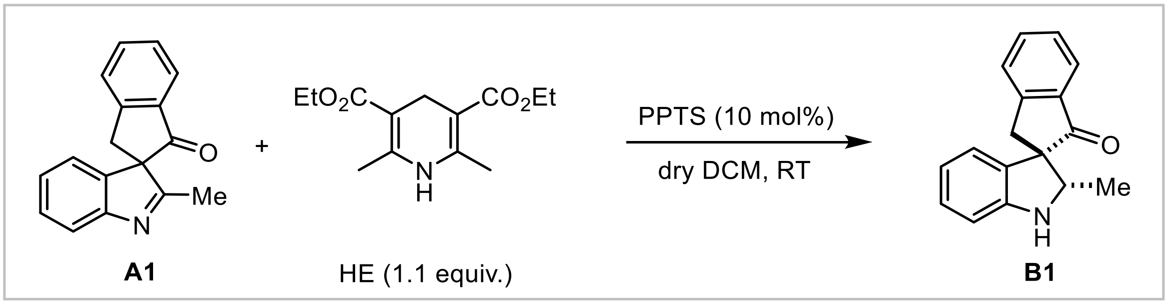

General procedure C: Synthesis of Class B from Class A[15]

An oven-dried screw capped reaction tube with a magnetic stir-bar was charged with 2'-methylspiro[indene-2,3'-indol]-1(3H)-one (0.1 mmol), Hantzsch ester (1.1 equiv., 1.1 mmol, 28 mg) and pyridinium *p*-toluenesulfonate (PPTS, 10 mol%, 2.5 mg). A screw cap fitted with a rubber septum was attached to the reaction tube. Dry dichloromethane (2 mL) was added in the reaction mixture and vigorously stirred at room temperature for 12 h. The reaction mixture was taken out after 12 h, diluted with 10 mL of dichloromethane and transferred to a round bottom flask. The solvent was evaporated by rotavapor. The crude mixture was purified by column chromatography using silica gel (100-200 mesh size) and petroleum ether/ethyl acetate as the eluent.

Characterization data for Class B products

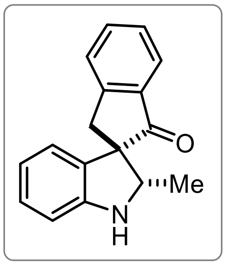

2'-methylspiro[indene-2,3'-indolin]-1(3H)-one [**B1**]: Compound **B1** was prepared according to the general procedure C (0.1 mmol scale).

Yield: 88% (22 mg isolated) [diastereomeric ratio (d.r.), 20:1].

Eluent: Petroleum ether/Ethyl acetate (75/25, v/v).

TLC: R_f = 0.46 (70:30 petroleum ether:EtOAc).

Physical State: Yellowish solid.

^{1}H NMR (400 MHz, Chloroform-*d*) δ 7.78 (ddd, *J* = 7.7, 1.4, 0.8 Hz, 1H), 7.65 (td, *J* = 7.4, 1.2 Hz, 1H), 7.53 (dp, *J* = 7.7, 1.0 Hz, 1H), 7.47 – 7.38 (m, 1H), 7.10 (ddd, *J* = 7.9, 7.1, 1.6 Hz, 1H), 6.82 (dt, *J* = 7.9, 0.8 Hz, 1H), 6.80 – 6.68 (m, 2H), 4.13 (q, *J* = 6.6 Hz, 1H), 3.59 (d, *J* = 16 Hz, 1H), 3.54 (s, 2H), 3.25 (d, *J* = 17.6 Hz, 1H), 1.28 (d, *J* = 6.6 Hz, 3H) ppm.

^{13}C NMR (176 MHz, CDCl$_3$) δ 204.05, 152.79, 151.07, 136.21, 135.18, 133.42, 128.76, 128.20, 126.26, 124.90, 122.75, 120.18, 111.53, 65.55, 61.92, 41.00, 15.68 ppm.

135-DEPT NMR (176 MHz, CDCl$_3$) δ 135.00, 128.58, 128.02, 126.08, 124.72, 122.57, 120.00, 111.35, 65.37, 40.82, 15.50 ppm.

HRMS (m/z): [M + H$^+$] calcd for $C_{17}H_{16}ON$, 250.12264; found, 250.12270.

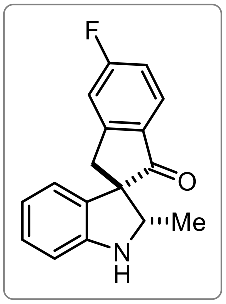

5-fluoro-2'-methylspiro[indene-2,3'-indolin]-1(3H)-one [**B2**]: Compound **B2** was prepared according to the general procedure C (0.1 mmol scale).

Yield: 92% (24.6 mg isolated) [d.r., >20:1].

Eluent: Petroleum ether/Ethyl acetate (75/25, v/v).

TLC: R_f = 0.48 (70:30 petroleum ether:EtOAc).

Physical State: Brown oil.

^{1}H NMR (500 MHz, Chloroform-*d*) δ 7.78 (dd, *J* = 8.4, 5.4 Hz, 1H), 7.19 (dd, *J* = 8.5, 2.2 Hz, 1H), 7.15 – 7.06 (m, 2H), 6.79 (d, *J* = 7.8 Hz, 1H), 6.78 – 6.68 (m, 2H), 4.12 (q, *J* = 6.6 Hz, 1H), 3.76 (s, 1H), 3.58 (d, *J* = 17.8 Hz, 1H), 3.22 (d, *J* = 17.8 Hz, 1H), 1.27 (d, *J* = 6.6 Hz, 3H) ppm.

^{13}C NMR (126 MHz, CDCl$_3$) δ 202.21, 168.59, 166.55 [167.57 (d, *J* = 256.8 Hz)], 155.64, 155.56 [155.60 (d, *J* = 10.1 Hz)], 151.19, 132.91, 132.49, 132.48 [132.49 (d, *J* = 1.9 Hz)], 128.88, 127.26, 127.18 [127.22 (d, *J* = 10.5 Hz)], 122.65, 120.03, 116.64, 116.45 [116.54 (d, *J* = 23.8 Hz)], 113.07, 112.90 [112.99 (d, *J* = 22.3 Hz)], 111.39, 65.50, 62.14, 40.76, 40.74 [40.75 (d, *J* = 2.1 Hz)], 15.63 ppm.

^{19}F NMR (470 MHz, Chloroform-*d*) δ -102.28 (td, *J* = 8.6, 5.4 Hz) ppm.

HRMS (m/z): [M + H$^+$] calcd for $C_{17}H_{15}ONF$, 268.11322; found, 268.11337.

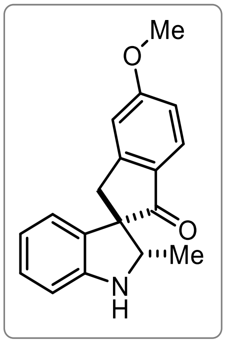

5-methoxy-2'-methylspiro[indene-2,3'-indolin]-1(3H)-one [**B3**]: Compound **B3** was prepared according to the general procedure C (0.1 mmol scale).

Yield: 85% (23.7 mg isolated) [d.r., 10:1].

Eluent: Petroleum ether/Ethyl acetate (70/30, v/v).

TLC: R_f = 0.25 (70:30 petroleum ether:EtOAc).

Physical State: Amorphous white solid.

^{1}H NMR (500 MHz, Chloroform-*d*) δ 7.71 (d, *J* = 9.1 Hz, 1H), 7.10 (td, *J* = 7.6, 1.4 Hz, 1H), 6.98 – 6.92 (m, 2H), 6.82 (dd, *J* = 7.8, 1.0 Hz, 1H), 6.78 (dd, *J* = 7.5, 1.4 Hz, 1H), 6.74 (td, *J* = 7.4, 1.0 Hz, 1H), 4.12 (q, *J* = 6.6 Hz, 1H), 3.92 (s, 3H), 3.61 – 3.51 (m, 2H [1NH]), 3.18 (d, *J* = 17.6 Hz, 1H), 1.29 (d, *J* = 6.6 Hz, 3H) ppm.

^{13}C NMR (126 MHz, $CDCl_3$) δ 202.23, 165.79, 155.72, 150.68, 133.89, 129.25, 128.70, 126.67, 122.74, 120.43, 116.14, 111.76, 109.54, 65.51, 62.02, 55.93, 40.95, 15.55 ppm.

135-DEPT NMR (126 MHz, $CDCl_3$) δ 128.51, 126.47, 122.55, 120.24, 115.95, 111.57, 109.35, 65.32, 55.74, 40.76, 15.36 ppm.

HRMS (m/z): [M + H^+] calcd for $C_{18}H_{18}O_2N$, 280.13321; found, 280.13333.

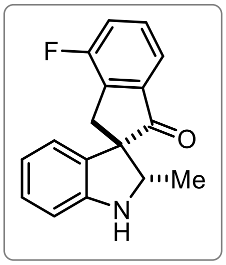

4-fluoro-2'-methylspiro[indene-2,3'-indolin]-1(3H)-one [**B4**]: Compound **B4** was prepared according to the general procedure C (0.1 mmol scale).

Yield: 79% (21.1 mg isolated) [d.r., 15:1].

Eluent: Petroleum ether/Ethyl acetate (80/20, v/v).

TLC: R_f = 0.45 (70:30 petroleum ether:EtOAc).

Physical State: Brown oil.

^{1}H NMR (500 MHz, Methylene Chloride-*d*$_2$) δ 7.57 – 7.53 (m, 1H), 7.45 (tdt, *J* = 7.5, 4.6, 0.8 Hz, 1H), 7.36 (ddd, *J* = 8.9, 8.0, 0.9 Hz, 1H), 7.09 (td, *J* = 7.7, 1.4 Hz, 1H), 6.75 (ddt, *J* = 10.2, 7.9, 0.8 Hz, 2H), 6.69 (td, *J* = 7.4, 1.0 Hz, 1H), 4.14 (q, *J* = 6.6 Hz, 1H), 3.62 (d, *J* = 17.9 Hz, 1H), 3.24 (d, *J* = 17.8 Hz, 1H), 1.20 (d, *J* = 6.6 Hz, 3H) ppm.

^{13}C NMR (126 MHz, CD_2Cl_2) δ 203.25, 203.23 [203.24 (d, *J* = 3.0 Hz)], 161.12, 159.14 [160.13 (d, *J* = 249.8 Hz)], 152.30, 139.34, 139.30 [139.32 (d, *J* = 4.7 Hz)], 139.08, 138.92 [139.00 (d, *J* = 19.7 Hz)], 132.74, 130.61, 130.56 [130.58 (d, *J* = 6.3 Hz)], 129.17, 123.05, 121.57, 121.41 [121.49 (d, *J* = 20.2 Hz)], 120.61, 120.58 [120.59 (d, *J* = 3.9 Hz)], 119.63, 110.83, 66.09, 62.13, 37.01, 27.45, 15.82 ppm.

^{19}F NMR (470 MHz, Methylene Chloride-*d*$_2$) δ -119.52 (dd, *J* = 8.8, 4.6 Hz) ppm.

HRMS (m/z): [M + H^+] calcd for $C_{17}H_{15}ONF$, 268.11322; found, 268.11297.

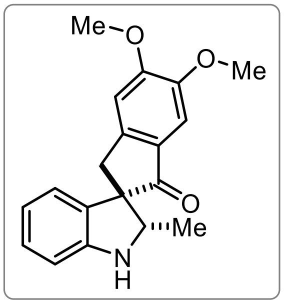

5,6-dimethoxy-2'-methylspiro[indene-2,3'-indolin]-1(3H)-one [**B5**]: Compound **B5** was prepared according to the general procedure C (0.1 mmol scale).

Yield: 80% (25 mg isolated) [d.r., >20:1].

Eluent: Petroleum ether/Ethyl acetate (70/30, v/v).

TLC: R_f = 0.25 (60:40 petroleum ether:EtOAc).

Physical State: Off-white solid.

^{1}H NMR (500 MHz, Chloroform-*d*) δ 7.18 (s, 1H), 7.08 (td, *J* = 7.6, 1.4 Hz, 1H), 6.94 (s, 1H), 6.77 (ddd, *J* = 9.0, 7.6, 1.1 Hz, 2H), 6.71 (td, *J* = 7.4, 1.0 Hz, 1H), 4.11 (q, *J* = 6.6 Hz, 1H), 4.00 (s, 3H), 3.90 (s, 3H), 3.51 (d, *J* = 17.4 Hz, 1H), 3.31 (s, 1H), 3.13 (d, *J* = 17.3 Hz, 1H), 1.28 (d, *J* = 6.6 Hz, 3H) ppm.

^{13}C NMR (126 MHz, $CDCl_3$) δ 202.73, 155.84, 151.21, 150.04, 148.12, 133.60, 128.68, 128.61, 122.62, 119.96, 111.33, 107.16, 105.13, 65.31, 62.11, 56.48, 56.32, 40.89, 15.59 ppm.

135-DEPT NMR (126 MHz, $CDCl_3$) δ 128.46, 122.48, 119.81, 111.18, 107.01, 104.98, 65.17, 56.34, 56.17, 40.74, 15.44 ppm.

HRMS (m/z): [M + H^+] calcd for $C_{19}H_{20}O_3N$, 310.14377; found, 310.14407.

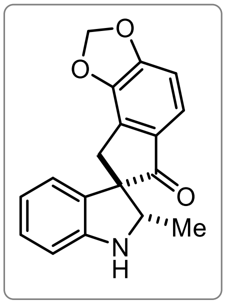

2'-methylspiro[indeno[4,5-d][1,3]dioxole-7,3'-indolin]-6(8H)-one [**B6**]: Compound **B6** was prepared according to the general procedure C (0.1 mmol scale).

Yield: 78% (22.9 mg isolated) [d.r., 11:1].

Eluent: Petroleum ether/Ethyl acetate (70/30, v/v).

TLC: R_f = 0.22 (70:30 petroleum ether:EtOAc).

Physical State: Brown oil.

^{1}H NMR (500 MHz, Chloroform-*d*) δ 7.41 (d, *J* = 8.1 Hz, 1H), 7.13 – 7.06 (m, 1H), 6.92 (d, *J* = 8.1 Hz, 1H), 6.83 – 6.77 (m, 2H), 6.73 (dd, *J* = 8.0, 6.9 Hz, 1H), 6.14 (dt, *J* = 4.7, 1.2 Hz, 2H), 4.11 (q, *J* = 6.5 Hz, 1H), 3.65 – 3.37 (m, 3H), 3.14 (d, *J* = 17.7 Hz, 1H), 1.28 (d, *J* = 6.6 Hz, 3H) ppm.

^{13}C NMR (126 MHz, $CDCl_3$) δ 201.66, 153.03, 151.06, 143.66, 133.41, 131.81, 131.67, 128.79, 122.75, 120.43, 120.14, 111.48, 109.51, 102.48, 77.48, 77.23, 76.98, 65.68, 62.03, 36.43, 15.58 ppm.

135-DEPT NMR (126 MHz, $CDCl_3$) δ 128.63, 122.59, 120.26, 119.97, 111.31, 109.34, 102.31, 65.52, 36.26, 15.41 ppm.

HRMS (m/z): [M + H^+] calcd for $C_{18}H_{16}O_3N$, 294.11247; found, 294.11255.

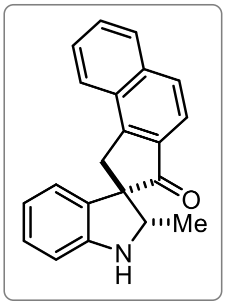

2'-methylspiro[cyclopenta[a]naphthalene-2,3'-indolin]-3(1H)-one [**B7**]: Compound **B7** was prepared according to the general procedure C (0.1 mmol scale).

Yield: 81% (24.2 mg isolated) [d.r., 8:1].

Eluent: Petroleum ether/Ethyl acetate (80/20, v/v).

TLC: R_f = 0.45 (70:30 petroleum ether:EtOAc).

Physical State: Yellowish oil.

^{1}H NMR (700 MHz, Chloroform-*d*) δ 9.12 (dq, *J* = 8.4, 0.9 Hz, 1H), 8.11 (d, *J* = 8.3 Hz, 1H), 7.92 (dt, *J* = 8.1, 0.8 Hz, 1H), 7.66 (ddd, *J* = 8.3, 6.9, 1.3 Hz, 1H), 7.61 – 7.55 (m, 2H), 7.12 (td, *J* = 7.6, 1.3 Hz, 1H), 6.85 (dt, *J* = 7.9, 0.8 Hz, 1H), 6.81 (dt, *J* = 7.4, 0.9 Hz, 1H), 6.74 (td, *J* = 7.4, 1.0 Hz, 1H), 4.19 (q, *J* = 6.6 Hz, 1H), 3.72 (d, *J* = 17.8 Hz, 1H), 3.58 (s, 1H), 3.32 (d, *J* = 17.8 Hz, 1H), 1.32 (d, *J* = 6.6 Hz, 3H) ppm.

^{13}C NMR (176 MHz, $CDCl_3$) δ 204.65, 156.06, 151.21, 136.33, 133.66, 133.31, 129.91, 129.81, 129.35, 128.73, 128.38, 126.99, 124.49, 123.63, 122.75, 120.24, 111.67, 65.67, 62.16, 41.24, 15.62 ppm.

135 DEPT NMR (176 MHz, $CDCl_3$) δ 136.15, 129.17, 128.56, 128.20, 126.81, 124.32, 123.45, 122.58, 120.07, 111.49, 65.49, 41.07, 15.45 ppm.

HRMS (m/z): [M + H^+] calcd for $C_{21}H_{18}ON$, 300.13829; found, 300.13837.

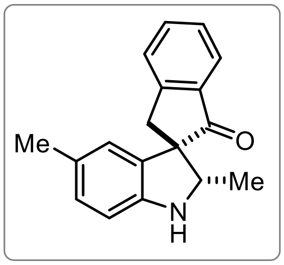

2',5'-dimethylspiro[indene-2,3'-indolin]-1(3H)-one [**B8**]: Compound **B8** was prepared according to the general procedure C (0.1 mmol scale).

Yield: 96% (25.3 mg) [d.r., 15:1].

Eluent: Petroleum ether/Ethyl acetate (80/20, v/v).

TLC: R_f = 0.5 (70:30 petroleum ether:EtOAc).

Physical State: Brown solid.

^{1}H NMR (700 MHz, Chloroform-*d*) δ 7.78 (d, *J* = 7.6 Hz, 1H), 7.65 (td, *J* = 7.4, 1.3 Hz, 1H), 7.55 – 7.52 (m, 1H), 7.43 (t, *J* = 7.5 Hz, 1H), 6.93 – 6.90 (m, 1H), 6.76 (d, *J* = 8.0 Hz, 1H), 6.58 (d, *J* = 1.7 Hz, 1H), 4.10 (q, *J* = 6.6 Hz, 1H), 3.60 (d, *J* = 17.5 Hz, 1H), 3.23 (d, *J* = 17.5 Hz, 1H), 2.18 (s, 3H), 1.27 (d, *J* = 6.6 Hz, 3H) ppm.

^{13}C NMR (176 MHz, $CDCl_3$) δ 204.39, 152.92, 148.51, 136.23, 135.23, 134.09, 130.11, 129.27, 128.22, 126.29, 124.94, 123.41, 112.00, 65.83, 62.07, 40.73, 21.05, 15.56 ppm.

HRMS (m/z): [M + H^+] calcd for $C_{18}H_{18}ON$, 264.13829; found, 264.13818.

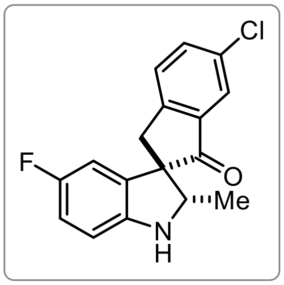

6-chloro-5'-fluoro-2'-methylspiro[indene-2,3'-indolin]-1(3H)-one [**B9**]: Compound **B9** was prepared according to the general procedure C (0.1 mmol scale).

Yield: 83% (25 mg isolated) [d.r., >20:1].

Eluent: Petroleum ether/Ethyl acetate (80/20, v/v).

TLC: R_f = 0.35 (80:20 petroleum ether:EtOAc).

Physical State: Brown semi-solid.

^{1}H NMR (600 MHz, Chloroform-*d*) δ 7.73 (dd, *J* = 2.1, 0.6 Hz, 1H), 7.61 (dd, *J* = 8.2, 2.1 Hz, 1H), 7.47 (dq, *J* = 8.1, 0.9 Hz, 1H), 6.79 (ddd, *J* = 9.0, 8.5, 2.6 Hz, 1H), 6.71 (dd, *J* = 8.6, 4.3 Hz, 1H), 6.48 (dd, *J* = 8.1, 2.6 Hz, 1H), 4.11 (q, *J* = 6.6 Hz, 1H), 3.68 (s, 1H), 3.52 (d, *J* = 17.7 Hz, 1H), 3.19 (d, *J* = 17.8 Hz, 1H), 1.23 (d, *J* = 6.6 Hz, 3H) ppm.

^{13}C NMR (151 MHz, $CDCl_3$) δ 202.36, <u>158.56, 156.98</u> [157.77 (d, *J* = 237.7 Hz)], 150.55, <u>147.35, 147.34</u> [147.34 (d, *J* = 1.9 Hz)], 137.40, 135.38, 134.76, <u>134.61, 134.55</u> [134.58 (d, *J* = 7.9 Hz)], 127.53, 124.62, <u>115.19, 115.03</u> [115.11 (d, *J* = 23.3 Hz)], <u>112.23, 112.18</u> [112.21 (d, *J* = 8.0 Hz)], <u>110.27, 110.11</u> [110.19 (d, *J* = 24.2 Hz)], 66.26, <u>62.88, 62.87</u> [62.87 (d, *J* = 1.7 Hz)], 40.06, 15.55 ppm.

135-DEPT NMR (151 MHz, $CDCl_3$) δ 135.22, 127.37, 124.45, 115.02, 114.87 [114.95 (d, *J* = 23.4 Hz)], 112.07, 112.01 [112.04 (d, *J* = 8.2 Hz)], 110.10, 109.94 [110.02 (d, *J* = 24.2 Hz)], 66.09, 39.89, 15.39 ppm.

^{19}F NMR (565 MHz, Chloroform-*d*) δ -124.16 (td, *J* = 8.6, 4.3 Hz) ppm.

HRMS (m/z): [M + H^+] calcd for $C_{17}H_{14}ONClF$, 302.07425; found, 302.07423.

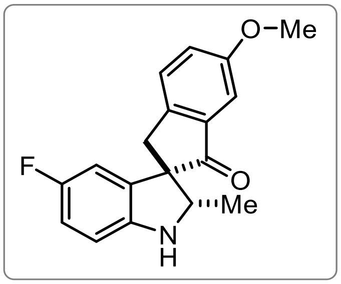

5'-fluoro-6-methoxy-2'-methylspiro[indene-2,3'-indolin]-1(3H)-one [**B10**]: Compound **B10** was prepared according to the general procedure C (0.1 mmol scale). Compound **B10** was recrystallized in dichloromethane at 23 °C via slow evaporation.

Yield: 79% (23.5 mg isolated) [d.r., >20:1].

Eluent: Petroleum ether/Ethyl acetate (75/25, v/v).

TLC: R_f = 0.35 (70:30 petroleum ether:EtOAc).

Physical State: Yellowish semi-solid.

^{1}H NMR (500 MHz, Chloroform-*d*) δ 7.41 (dq, *J* = 8.4, 0.8 Hz, 1H), 7.25 (dd, *J* = 8.4, 2.6 Hz, 1H), 7.19 (d, *J* = 2.6 Hz, 1H), 6.79 (td, *J* = 8.8, 2.6 Hz, 1H), 6.75 (dd, *J* = 8.5, 4.5 Hz, 1H), 6.50 (dd, *J* = 8.2, 2.5 Hz, 1H), 4.13 (q, *J* = 6.6 Hz, 1H), 3.84 (s, 3H), 3.64 (s, 1H), 3.48 (dd, *J* = 17.2, 0.9 Hz, 1H), 3.15 (dd, *J* = 17.3, 0.9 Hz, 1H), 1.27 (d, *J* = 6.7 Hz, 3H) ppm.

^{13}C NMR (126 MHz, $CDCl_3$) δ 203.60, 160.16, 158.95, 157.06 [158.00 (d, *J* = 237.8 Hz)], 146.88, 146.86 [146.87 (d, *J* = 1.8 Hz)], 145.43, 136.99, 135.52, 135.46 [135.49 (d, *J* = 7.7 Hz)], 127.03, 124.96, 115.03, 114.85 [114.94 (d, *J* = 23.2 Hz)], 112.53, 112.46 [112.50 (d, *J* = 8.3 Hz)], 110.33, 110.14 [110.23 (d, *J* = 24.1 Hz)], 105.84, 66.09, 62.99, 62.98 [62.99 (d, *J* = 1.8 Hz)], 55.85, 39.89, 15.44 ppm.

135-DEPT NMR (126 MHz, $CDCl_3$) δ 126.86, 124.79, 114.86, 114.68 [114.77 (d, *J* = 23.3 Hz)], 112.36, 112.30 [112.33 (d, *J* = 8.4 Hz)], 110.16, 109.96 [110.06 (d, *J* = 24.1 Hz)], 105.67, 65.92, 55.68, 39.72, 15.27 ppm.

^{19}F NMR (470 MHz, Chloroform-*d*) δ -123.86 (q, *J* = 7.8, 7.0 Hz) ppm.

HRMS (m/z): [M + H^+] calcd for $C_{18}H_{17}O_2NF$, 298.12378; found, 298.12379.

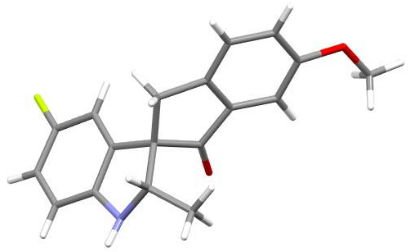

CCDC: 2221540

*See Supplementary Fig. 27 and Supplementary Table 13 for more details of X-ray structure analysis of **B10.***

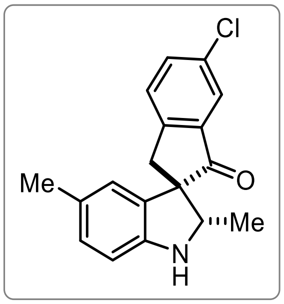

6-chloro-2',5'-dimethylspiro[indene-2,3'-indolin]-1(3H)-one [**B11**]: Compound **B11** was prepared according to the general procedure C (0.1 mmol scale).

Yield: 90% (26.8 mg isolated) [d.r., 8:1].

Eluent: Petroleum ether/Ethyl acetate (80/20, v/v).

TLC: R_f = 0.55 (70:30 petroleum ether:EtOAc).

Physical State: Colorless solid.

^{1}H NMR (500 MHz, Chloroform-*d*) δ 7.74 (d, *J* = 2.1 Hz, 1H), 7.61 (dd, *J* = 8.2, 2.1 Hz, 1H), 7.48 (dq, *J* = 8.1, 0.9 Hz, 1H), 6.94 (ddd, *J* = 7.9, 1.7, 0.8 Hz, 1H), 6.81 (d, *J* = 8.0 Hz, 1H), 6.58 (dt, *J* = 1.6, 0.7 Hz, 1H), 4.12 (q, *J* = 6.6 Hz, 1H), 3.56 (d, *J* = 17.8 Hz, 1H), 3.27 – 3.13 (m, 2H), 2.19 (s, 3H), 1.28 (d, *J* = 6.7 Hz, 3H) ppm.

^{13}C NMR (126 MHz, $CDCl_3$) δ 203.03, 150.89, 147.49, 137.57, 135.30, 134.65, 133.77, 130.82, 129.53, 127.53, 124.64, 123.34, 112.59, 65.72, 62.57, 39.97, 21.06, 15.30 ppm.

HRMS (m/z): [M + H^+] calcd for $C_{18}H_{17}ONCl$, 298.09932; found, 298.09921.

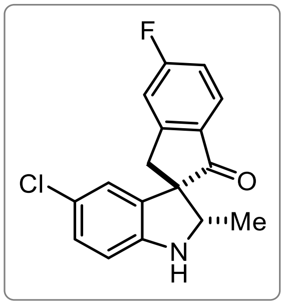

5'-chloro-5-fluoro-2'-methylspiro[indene-2,3'-indolin]-1(3H)-one [**B12**]: Compound **B12** was prepared according to the general procedure C (0.1 mmol scale).

Yield: 78% (23.5 mg isolated) [d.r., >20:1].

Eluent: Petroleum ether/Ethyl acetate (80/20, v/v).

TLC: R_f = 0.5 (70:30 petroleum ether:EtOAc).

Physical State: Brown semi-solid.

^{1}H NMR (500 MHz, Chloroform-*d*) δ 7.78 (dd, *J* = 8.5, 5.3 Hz, 1H), 7.18 (dd, *J* = 8.4, 2.2 Hz, 1H), 7.13 (td, *J* = 8.6, 2.3 Hz, 1H), 7.03 (dd, *J* = 8.3, 2.1 Hz, 1H), 6.70 (d, *J* = 2.2 Hz, 1H), 6.67 (d, *J* = 8.4 Hz, 1H), 4.11 (q, *J* = 6.6 Hz, 1H), 3.61 – 3.46 (m, 2H [-NH]), 3.20 (d, *J* = 17.8 Hz, 1H), 1.24 (d, *J* = 6.6 Hz, 3H) ppm.

^{13}C NMR (126 MHz, $CDCl_3$) δ 201.59, <u>168.69, 166.65</u> [167.67 (d, *J* = 257.3 Hz)], <u>155.37, 155.29</u> [155.33 (d, *J* = 10.1 Hz)], 149.98, 134.52, <u>132.19, 132.17</u> [132.18 (d, *J* = 1.8 Hz)], 128.65, <u>127.37, 127.28</u> [127.33 (d, *J* = 10.5 Hz)], 124.34, 122.95, <u>116.85, 116.66</u> [116.75 (d, *J* = 23.7 Hz)], <u>113.14, 112.96</u> [113.05 (d, *J* = 22.3 Hz)], 111.92, 65.86, 62.04, <u>40.60, 40.58</u> [40.59 (d, *J* = 2.1 Hz)], 15.57 ppm.

135-DEPT NMR (126 MHz, $CDCl_3$) δ 128.50, <u>127.22, 127.14</u> [127.18 (d, *J* = 10.5 Hz)], 122.80, <u>116.70, 116.51</u> [116.60 (d, *J* = 23.8 Hz)], <u>112.99, 112.81</u> [112.90 (d, *J* = 22.4 Hz)], 111.77, 65.71, <u>40.45, 40.44</u> [40.44 (d, *J* = 2.0 Hz)], 15.42 ppm.

^{19}F NMR (470 MHz, Chloroform-*d*) δ -101.67 (td, *J* = 8.7, 5.3 Hz) ppm.

HRMS (m/z): [M + H^+] calcd for $C_{17}H_{14}ONClF$, 302.07425; found, 302.07424.

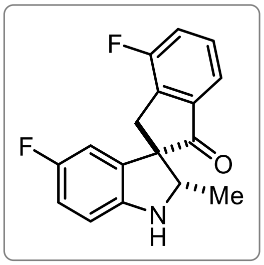

4,5'-difluoro-2'-methylspiro[indene-2,3'-indolin]-1(3H)-one [**B13**]: Compound **B13** was prepared according to the general procedure C (0.1 mmol scale).

Yield: 88% (25.1 mg isolated) [d.r., 20:1].

Eluent: Petroleum ether/Ethyl acetate (80/20, v/v).

TLC: R_f = 0.5 (70:30 petroleum ether:EtOAc).

Physical State: Yellow oil.

^{1}H NMR (500 MHz, Chloroform-*d*) δ 7.61 – 7.57 (m, 1H), 7.44 (td, *J* = 7.8, 4.6 Hz, 1H), 7.34 (td, *J* = 8.4, 1.0 Hz, 1H), 6.81 (td, *J* = 8.8, 2.6 Hz, 1H), 6.75 (dd, *J* = 8.5, 4.4 Hz, 1H), 6.51 (dd, *J* = 8.1, 2.6 Hz, 1H), 4.16 (q, *J* = 6.6 Hz, 1H), 3.71 – 3.53 (m, 2H), 3.24 (d, *J* = 18.0 Hz, 1H), 1.26 (d, *J* = 6.6 Hz, 3H) ppm.

^{13}C NMR (126 MHz, $CDCl_3$) δ 202.44, 202.41 [202.42 (d, *J* = 2.9 Hz)], 160.71, 158.71 [159.71 (d, *J* = 251.0 Hz)], 158.90, 157.01 [157.95 (d, *J* = 238.0 Hz)], 147.01, 147.00 [147.01 (d, *J* = 1.4 Hz)], 138.58, 138.54 [138.56 (d, *J* = 4.6 Hz)], 138.41, 138.25 [138.33 (d, *J* = 19.5 Hz)], 134.72, 134.65 [134.69 (d, *J* = 7.8 Hz)], 130.45, 130.40 [130.42 (d, *J* = 6.2 Hz)], 121.64, 121.48 [121.56 (d, *J* = 19.9 Hz)], 120.73, 120.70 [120.72 (d, *J* = 4.0 Hz)], 115.32, 115.14 [115.23 (d, *J* = 23.2 Hz)], 112.55, 112.48 [112.51 (d, *J* = 8.2 Hz)], 110.36, 110.16 [110.26 (d, *J* = 24.3 Hz)], 66.26, 62.17, 62.15 [62.16 (d, *J* = 1.8 Hz)], 36.06, 15.43 ppm.

^{19}F NMR (470 MHz, Chloroform-*d*) δ -118.42 (dt, *J* = 7.9, 3.6 Hz), -123.78 (tt, *J* = 11.7, 5.8 Hz) ppm.

HRMS (m/z): [M + H^+] calcd for $C_{17}H_{14}ONF_2$, 286.10380; found, 286.10376.

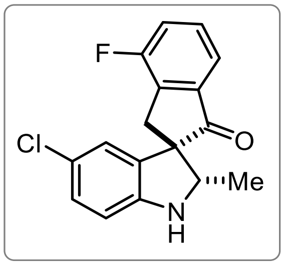

5'-chloro-4-fluoro-2'-methylspiro[indene-2,3'-indolin]-1(3H)-one [**B14**]: Compound **B14** was prepared according to the general procedure C (0.1 mmol scale).

Yield: 90% (27.2 mg) [d.r., 15:1].

Eluent: Petroleum ether/Ethyl acetate (80/20, v/v).

TLC: R_f = 0.52 (70:30 petroleum ether:EtOAc).

Physical State: Yellow oil.

^{1}H NMR (600 MHz, Chloroform-*d*) δ 7.60 (d, *J* = 7.5 Hz, 1H), 7.45 (td, *J* = 7.8, 4.6 Hz, 1H), 7.36 (t, *J* = 8.3 Hz, 1H), 7.10 (dd, *J* = 8.4, 2.1 Hz, 1H), 6.85 (d, *J* = 8.4 Hz, 1H), 6.77 (d, *J* = 2.1 Hz, 1H), 4.21 (q, *J* = 6.6 Hz, 1H), 3.59 (d, *J* = 18.0 Hz, 1H), 3.49 (s, 1H), 3.26 (d, *J* = 18.0 Hz, 1H), 1.32 (d, *J* = 6.6 Hz, 3H) ppm.

^{13}C NMR (151 MHz, $CDCl_3$) δ <u>202.07, 202.05</u> [202.06 (d, *J* = 2.8 Hz)], <u>160.57, 158.91</u> [159.74 (d, *J* = 251.0 Hz)], 147.88, <u>138.42, 138.39</u> [138.40 (d, *J* = 4.5 Hz)], <u>138.34, 138.21</u> [138.28 (d, *J* = 19.6 Hz)], 135.21, <u>130.58, 130.54</u> [130.56 (d, *J* = 6.1 Hz)], 129.05, 126.31, 123.17, <u>121.82, 121.69</u> [121.76 (d, *J* = 19.8 Hz)], <u>120.87, 120.84</u> [120.86 (d, *J* = 3.9 Hz)], 113.58, 65.92, 61.66, 35.96, 15.18 ppm.

135-DEPT NMR (151 MHz, $CDCl_3$) δ <u>130.38, 130.34</u> [130.36 (d, *J* = 6.3 Hz)], 128.84, 122.97, <u>121.62, 121.49</u> [121.55 (d, *J* = 19.9 Hz)], <u>120.67, 120.64</u> [120.65 (d, *J* = 3.9 Hz)], 113.37, 65.72, 35.76, 14.97 ppm.

^{19}F NMR (470 MHz, Chloroform-*d*) δ -118.02 (dd, *J* = 8.3, 4.5 Hz) ppm.

HRMS (m/z): [M + H^+] calcd for $C_{17}H_{14}ONClF$, 302.07425; found, 302.07436.

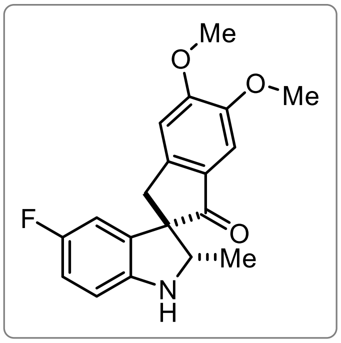

5'-fluoro-5,6-dimethoxy-2'-methylspiro[indene-2,3'-indolin]-1(3H)-one [**B15**]: Compound **B15** was prepared according to the general procedure C (0.1 mmol scale).

Yield: 84% (27.5 mg isolated) [d.r., 12:1].

Eluent: Petroleum ether/Ethyl acetate (60/40, v/v).

TLC: R_f = 0.35 (60:40 petroleum ether:EtOAc).

Physical State: Off-white solid.

^{1}H NMR (500 MHz, Chloroform-*d*) δ 7.17 (s, 1H), 6.93 (s, 1H), 6.78 (td, *J* = 8.8, 2.6 Hz, 1H), 6.72 (dd, *J* = 8.6, 4.5 Hz, 1H), 6.49 (dd, *J* = 8.2, 2.5 Hz, 1H), 4.11 (q, *J* = 6.5 Hz, 1H), 4.00 (s, 3H), 3.90 (s, 3H), 3.47 (d, *J* = 17.4 Hz, 1H), 3.21 (s, 1H), 3.12 (d, *J* = 17.3 Hz, 1H), 1.26 (d, *J* = 6.6 Hz, 3H) ppm.

^{13}C NMR (126 MHz, $CDCl_3$) δ 202.22, <u>158.87, 156.99</u> [157.93 (d, *J* = 237.4 Hz)], 156.07, 150.19, 147.95, 147.09, <u>135.74, 135.67</u> [135.70 (d, *J* = 7.6 Hz)], 128.38, <u>114.89, 114.71</u> [114.80 (d, *J* =

23.2 Hz)], 112.37, 112.31 [112.34 (d, *J* = 8.2 Hz)], 112.36, 112.29 [112.32 (d, *J* = 8.3 Hz)], 110.22, 110.02 [110.12 (d, *J* = 24.1 Hz)], 107.19, 105.12, 65.94, 62.49, 62.47 [62.48 (d, *J* = 1.8 Hz)], 56.52, 56.34, 40.44, 15.42 ppm.

^{19}F NMR (470 MHz, Chloroform-*d*) δ -124.06 – -124.20 (m) ppm.

HRMS (m/z): [M + H^+] calcd for $C_{19}H_{19}O_3NF$, 328.13435; found, 328.13430.

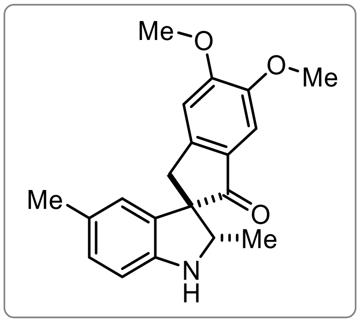

5,6-dimethoxy-2',5'-dimethylspiro[indene-2,3'-indolin]-1(3H)-one [**B16**]: Compound **B16** was prepared according to the general procedure C (0.1 mmol scale).

Yield: 78% (25.2 mg isolated) [d.r., 6:1].

Eluent: Petroleum ether/Ethyl acetate (70/30, v/v).

TLC: R_f = 0.4 (60:40 petroleum ether:EtOAc).

Physical State: Off-white solid.

^{1}H NMR (500 MHz, Chloroform-*d*) δ 7.19 (s, 1H), 6.95 (s, 1H), 6.91 (ddt, *J* = 7.9, 1.8, 0.9 Hz, 1H), 6.77 (dt, *J* = 8.0, 2.0 Hz, 1H), 6.59 (d, *J* = 1.9 Hz, 1H), 4.10 (q, *J* = 6.7 Hz, 1H), 4.01 (s, 3H), 3.91 (s, 3H), 3.50 (d, *J* = 17.4 Hz, 1H), 3.13 (d, *J* = 17.4 Hz, 1H), 2.18 (s, 3H), 1.29 (d, *J* = 6.6 Hz, 3H) ppm.

^{13}C NMR (126 MHz, $CDCl_3$) δ 202.94, 155.94, 150.09, 148.23, 134.52, 129.17, 128.67, 123.30, 122.06, 119.97, 112.25, 107.21, 105.16, 65.53, 62.25, 56.52, 56.37, 40.52, 21.07, 15.38 ppm.

HRMS (m/z): [M + H^+] calcd for $C_{20}H_{22}O_3N$, 324.15942; found, 324.15969.

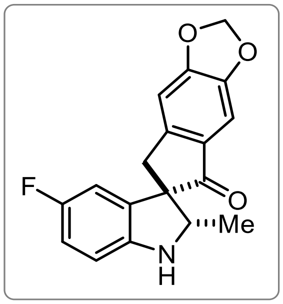

5'-fluoro-2'-methylspiro[indeno[5,6-d][1,3]dioxole-6,3'-indolin]-5(7H)-one [**B17**]: Compound **B17** was prepared according to the general procedure C (0.1 mmol scale).

Yield: 81% (25.2 mg isolated) [d.r., 10:1].

Eluent: Petroleum ether/Ethyl acetate (70/30, v/v).

TLC: R_f = 0.45 (60:40 petroleum ether:EtOAc).

Physical State: Yellow oil.

^{1}H NMR (500 MHz, Chloroform-*d*) δ 7.10 (s, 1H), 6.89 (q, *J* = 0.8 Hz, 1H), 6.78 (td, *J* = 8.8, 2.6 Hz, 1H), 6.73 (ddd, *J* = 8.6, 4.4, 1.1 Hz, 1H), 6.50 (dd, *J* = 8.2, 2.6 Hz, 1H), 6.09 (q, *J* = 1.1 Hz, 2H), 4.09 (q, *J* = 6.6, 6.1 Hz, 1H), 3.68 (s, 1H), 3.43 (d, *J* = 17.6 Hz, 1H), 3.09 (d, *J* = 17.6 Hz, 1H), 1.26 (d, *J* = 6.7 Hz, 3H) ppm.

^{13}C NMR (126 MHz, $CDCl_3$) δ 201.50, <u>158.97, 157.08</u> [158.02 (d, *J* = 238.0 Hz)], 155.02, 150.19, 149.06, <u>146.87, 146.82</u> [146.84 (d, *J* = 5.5 Hz)], <u>135.66, 135.60</u> [135.63 (d, *J* = 7.9 Hz)], 130.16, <u>114.99, 114.81</u> [114.90 (d, *J* = 23.2 Hz)], <u>112.60, 112.53</u> [112.56 (d, *J* = 8.2 Hz)], <u>110.23, 110.04</u> [110.14 (d, *J* = 24.1 Hz)], 105.52, 103.25, 102.59, 65.99, <u>62.71, 62.69</u> [62.70 (d, *J* = 1.8 Hz)], 40.41, 15.35 ppm.

135-DEPT NMR (126 MHz, Chloroform-*d*) δ 114.73 (d, *J* = 23.4 Hz), 112.38 (d, *J* = 8.4 Hz), 109.97 (d, *J* = 24.1 Hz) ppm.

^{19}F NMR (470 MHz, Chloroform-*d*) δ -123.83 – -124.00 (m) ppm.

HRMS (m/z): [M + H$^+$] calcd for $C_{18}H_{15}O_3NF$, 312.10305; found, 312.10302.

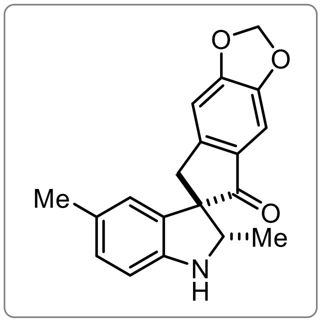

2',5'-dimethylspiro[indeno[5,6-d][1,3]dioxole-6,3'-indolin]-5(7H)-one [**B18**]: Compound **B18** was prepared according to the general procedure C (0.1 mmol scale).

Yield: 87% (26.7 mg isolated) [d.r., 8:1].

Eluent: Petroleum ether/Ethyl acetate (70/30, v/v).

TLC: R_f = 0.48 (60:40 petroleum ether:EtOAc).

Physical State: Amorphous white solid.

^{1}H NMR (700 MHz, Chloroform-*d*) δ 7.11 (s, 1H), 6.92 – 6.88 (m, 2H), 6.74 (d, J = 7.9 Hz, 1H), 6.61 – 6.58 (m, 1H), 6.10 – 6.07 (m, 2H), 4.06 (q, J = 6.6 Hz, 1H), 3.69 (s, 1H), 3.46 (d, J = 17.5 Hz, 1H), 3.09 (d, J = 17.4 Hz, 1H), 2.18 (s, 3H), 1.27 (d, J = 6.6 Hz, 3H) ppm.

^{13}C NMR (176 MHz, $CDCl_3$) δ 202.21, 154.81, 150.41, 148.92, 148.36, 134.25, 130.52, 130.06, 129.17, 123.27, 112.02, 105.51, 103.24, 102.49, 65.58, 62.54, 40.66, 21.03, 15.42 ppm.

135-DEPT NMR (176 MHz, $CDCl_3$) δ 128.99, 123.09, 111.85, 105.33, 103.06, 102.31, 65.40, 40.48, 20.85, 15.24 ppm.

HRMS (m/z): [M + H$^+$] calcd for $C_{19}H_{18}O_3N$, 308.12812; found, 308.12827.

CLASS-C

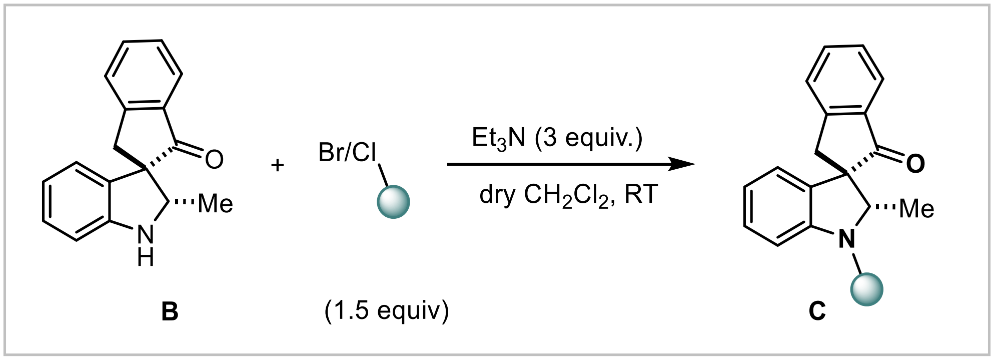

General procedure D: Synthesis of *N*-functional-group-appended indoline-indanone PNP (Class C)

An oven-dried screw capped reaction tube with a magnetic stir-bar was charged with 2'-methylspiro[indene-2,3'-indolin]-1(3H)-one (0.1 mmol) and bromo/chloro derivative of functional group (1.5 equiv., 0.15 mmol). A screw cap fitted with a rubber septum was attached to the reaction tube, then degassed and refilled with argon. Dry dichloromethane (2 mL) was added in the reaction tube followed by triethylamine (3 equiv., 0.3 mmol) added dropwise. The mixture was stirred at room temperature for 24 h. After maximum conversion (checked by TLC), the mixture was taken out and diluted with 10 mL of dichloromethane and transferred to a round bottom flask. The solvent was evaporated by rotavapor. The crude mixture was purified by column chromatography using silica gel (100-200 mesh size) and petroleum ether/ethyl acetate as the eluent.

Characterization data for Class C products:

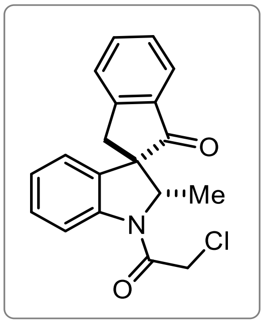

1'-(2-chloroacetyl)-2'-methylspiro[indene-2,3'-indolin]-1(3H)-one [**C1**]: Compound **C1** was prepared according to the general procedure D (0.1 mmol scale).

Yield: 86% (28 mg isolated).

Eluent: Petroleum ether/Ethyl acetate (80/20, v/v).

TLC: R_f = 0.45 (70:30 petroleum ether:EtOAc).

Physical State: Yellowish oil.

^{1}H NMR (500 MHz, Chloroform-*d*) δ 8.52 – 7.91 (m, 1H), 7.87 (d, *J* = 7.6 Hz, 1H), 7.71 (td, *J* = 7.5, 1.2 Hz, 1H), 7.50 (t, *J* = 7.8 Hz, 2H), 7.35 (td, *J* = 7.9, 1.4 Hz, 1H), 7.16 (td, *J* = 7.5, 1.0 Hz, 1H), 7.04 (d, *J* = 7.6 Hz, 1H), 4.71 – 4.42 (m, 1H), 4.37 – 3.87 (m, 2H), 3.61 (d, *J* = 17.0 Hz, 1H), 3.22 (d, *J* = 17.0 Hz, 1H), 1.32 (d, *J* = 18.1 Hz, 3H) ppm.

^{13}C NMR (126 MHz, $CDCl_3$) δ 204.09, 163.84, 151.15, 141.52, 137.10, 135.99, 129.02, 128.40, 126.90, 125.85, 125.84, 124.35, 124.16, 118.60, 67.73, 62.57, 44.44, 42.47, 19.07 ppm.

HRMS (m/z): [M + H$^+$] calcd for $C_{19}H_{17}O_2NCl$, 326.09423; found, 326.09435.

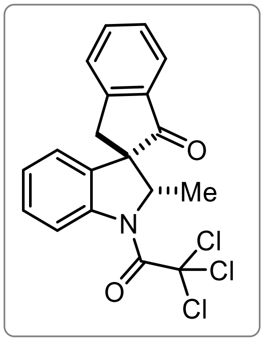

2'-methyl-1'-(2,2,2-trichloroacetyl)spiro[indene-2,3'-indolin]-1(3H)-one [**C2**]: Compound **C2** was prepared according to the general procedure D (0.1 mmol scale).

Yield: 71% (28 mg isolated).

Eluent: Petroleum ether/Ethyl acetate (85/15, v/v).

TLC: R_f = 0.42 (80:20 petroleum ether:EtOAc).

Physical State: Colorless liquid.

^{1}H NMR (500 MHz, Chloroform-*d*) δ 8.09 (dt, *J* = 8.1, 0.8 Hz, 1H), 7.87 (dt, *J* = 7.7, 1.0 Hz, 1H), 7.69 (td, *J* = 7.5, 1.3 Hz, 1H), 7.52 – 7.44 (m, 2H), 7.40 – 7.34 (m, 1H), 7.21 (td, *J* = 7.5, 1.1 Hz, 1H), 7.06 (dd, *J* = 7.6, 1.3 Hz, 1H), 5.05 (q, *J* = 6.3 Hz, 1H), 3.82 (d, *J* = 17.0 Hz, 1H), 3.15 (d, *J* = 17.1 Hz, 1H), 1.45 (d, *J* = 6.3 Hz, 3H) ppm.

^{13}C NMR (126 MHz, $CDCl_3$) δ 203.76, 157.84, 150.93, 142.49, 137.31, 136.06, 135.70, 128.81, 128.42, 126.86, 126.82, 124.45, 124.35, 120.39, 93.84, 68.94, 63.59, 42.53, 18.35 ppm.

135-DEPT NMR (126 MHz, $CDCl_3$) δ 135.89, 128.64, 128.25, 126.69, 126.64, 124.28, 124.18, 120.21, 68.76, 42.35, 18.17 ppm.

HRMS (m/z): [M + H$^+$] calcd for $C_{19}H_{15}O_2NCl_3$, 394.01629; found, 394.01619.

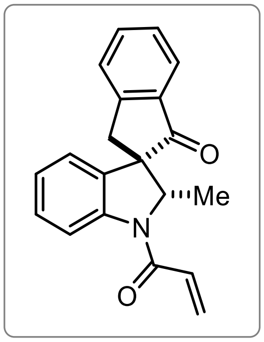

1'-acryloyl-2'-methylspiro[indene-2,3'-indolin]-1(3H)-one [**C3**]: Compound **C3** was prepared according to the general procedure D (0.1 mmol scale).

Yield: 82% (24.9 mg isolated).

Eluent: Petroleum ether/Ethyl acetate (70/30, v/v).

TLC: R_f = 0.5 (60:40 petroleum ether:EtOAc).

Physical State: Colorless liquid.

^{1}H NMR (500 MHz, Chloroform-*d*) δ 8.28 – 7.87 (m, 1H), 7.87 – 7.81 (m, 1H), 7.68 (td, *J* = 7.5, 1.2 Hz, 1H), 7.51 – 7.43 (m, 2H), 7.31 (td, *J* = 7.8, 1.3 Hz, 1H), 7.10 (td, *J* = 7.5, 1.1 Hz, 1H), 7.00 (dd, *J* = 7.6, 1.3 Hz, 1H), 6.77 – 6.46 (m, 2H), 5.88 – 5.76 (m, 1H), 4.68 – 4.37 (m, 1H), 3.52 (d, *J* = 16.9 Hz, 1H), 3.22 (d, *J* = 16.9 Hz, 1H), 1.31 (d, *J* = 6.5 Hz, 3H) ppm.

^{13}C NMR (126 MHz, $CDCl_3$) δ 204.31, 163.70, 151.16, 141.94, 137.20, 135.86, 134.88, 129.74, 128.89, 128.85, 128.36, 126.86, 125.22, 124.35, 124.29, 117.95, 68.05, 62.38, 44.69, 18.63 ppm.

HRMS (m/z): [M + H^+] calcd for $C_{20}H_{18}O_2N$, 304.13321; found, 304.13316.

Compound **C3** was resynthesized (**C3-resynthesized**) and retested in biological assays. The ^{1}H NMR spectra of the resynthesized batch is analogous to the original batch.

^{1}H NMR (400 MHz, Chloroform-*d*) δ 7.88 – 7.81 (m, 1H), 7.68 (td, *J* = 7.5, 1.2 Hz, 1H), 7.51 – 7.43 (m, 2H), 7.31 (td, *J* = 7.8, 1.4 Hz, 1H), 7.10 (td, *J* = 7.5, 1.1 Hz, 1H), 7.00 (dd, *J* = 7.5, 1.3 Hz, 1H), 6.70 – 6.48 (m, 2H), 5.87 – 5.78 (m, 1H), 4.51 (d, *J* = 6.7 Hz, 1H), 3.52 (d, *J* = 17.0 Hz, 1H), 3.22 (d, *J* = 16.9 Hz, 1H), 1.32 (d, *J* = 6.6 Hz, 3H).

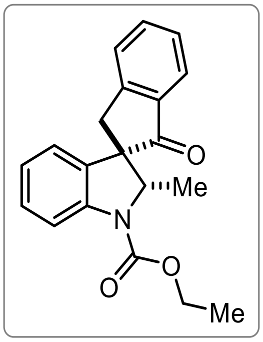

ethyl 2'-methyl-1-oxo-1,3-dihydrospiro[indene-2,3'-indoline]-1'-carboxylate [**C4**]: Compound **C4** was prepared according to the general procedure D (0.1 mmol scale).

Yield: 92% (29.6 mg isolated).

Eluent: Petroleum ether/Ethyl acetate (70/30, v/v).

TLC: R_f = 0.36 (60:40 petroleum ether:EtOAc).

Physical State: Colorless liquid.

^{1}H NMR (500 MHz, Chloroform-*d*) δ 7.82 (dt, *J* = 7.8, 1.0 Hz, 1H), 7.80 – 7.70 (m, 1H), 7.66 (td, *J* = 7.5, 1.2 Hz, 1H), 7.49 – 7.41 (m, 2H), 7.26 (ddd, *J* = 8.3, 6.9, 1.4 Hz, 1H), 6.99 (td, *J* = 7.5, 1.1 Hz, 1H), 6.91 (dd, *J* = 7.5, 1.4 Hz, 1H), 4.41 (q, *J* = 6.5 Hz, 1H), 4.37 – 4.23 (m, 2H), 3.51 (d, *J* = 16.9 Hz, 1H), 3.26 (d, *J* = 17.0 Hz, 1H), 1.36 (t, *J* = 7.0 Hz, 3H), 1.29 (d, *J* = 6.5 Hz, 3H) ppm.

^{13}C NMR (126 MHz, $CDCl_3$) δ 204.72, 153.13, 151.51, 142.17, 137.06, 135.66, 133.70, 128.89, 128.24, 126.71, 124.36, 123.94, 123.75, 115.87, 67.28, 62.01, 61.91, 45.13, 17.35, 14.77 ppm.

HRMS (m/z): [M + H^+] calcd for $C_{20}H_{20}O_3N$, 322.14377; found, 322.14381.

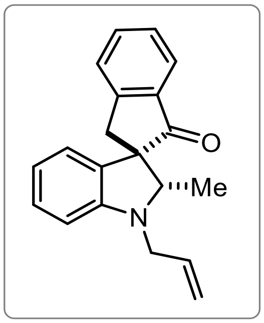

1'-allyl-2'-methylspiro[indene-2,3'-indolin]-1(3H)-one [**C5**]: Compound **C5** was prepared according to the general procedure D (0.1 mmol scale) using allyl bromide (1.5 equiv.).

Yield: 79% (22.9 mg isolated).

Eluent: Petroleum ether/Ethyl acetate (80/20, v/v).

TLC: R_f = 0.55 (70:30 petroleum ether:EtOAc).

Physical State: Yellow liquid.

^{1}H NMR (500 MHz, Chloroform-*d*) δ 7.77 (d, *J* = 7.5 Hz, 1H), 7.64 (td, *J* = 7.5, 1.3 Hz, 1H), 7.52 (dt, *J* = 7.7, 1.0 Hz, 1H), 7.42 (td, *J* = 7.4, 1.0 Hz, 1H), 7.11 (td, *J* = 7.7, 1.3 Hz, 1H), 6.70 (dd, *J* = 7.3, 1.3 Hz, 1H), 6.65 – 6.58 (m, 2H), 5.87 (dddd, *J* = 17.3, 10.2, 7.3, 4.5 Hz, 1H), 5.33 (dq, *J* = 17.2, 1.7 Hz, 1H), 5.21 (dq, *J* = 10.2, 1.4 Hz, 1H), 4.01 (ddt, *J* = 16.3, 4.6, 1.7 Hz, 1H), 3.83 (q, *J* = 6.5 Hz, 1H), 3.68 (ddt, *J* = 16.4, 7.4, 1.4 Hz, 1H), 3.63 (d, *J* = 17.6 Hz, 1H), 3.19 (d, *J* = 17.6 Hz, 1H), 1.21 (d, *J* = 6.6 Hz, 3H) ppm.

^{13}C NMR (126 MHz, $CDCl_3$) δ 203.84, 152.82, 152.35, 136.27, 135.10, 133.03, 132.21, 128.83, 128.17, 126.14, 124.98, 122.16, 118.35, 118.13, 108.77, 68.10, 60.89, 48.63, 41.41, 13.04 ppm.

135-DEPT NMR (126 MHz, $CDCl_3$) δ 134.93, 132.86, 128.66, 128.00, 125.97, 124.80, 121.98, 118.18, 117.96, 108.60, 67.93, 48.45, 41.24, 12.87 ppm.

HRMS (m/z): [M + H^+] calcd for $C_{20}H_{20}ON$, 290.15394; found, 290.15400.

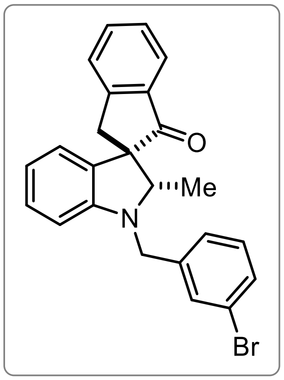

1'-(3-bromobenzyl)-2'-methylspiro[indene-2,3'-indolin]-1(3H)-one [**C6**]: Compound **C6** was prepared according to the general procedure D (0.1 mmol scale).

Yield: 88% (36.8 mg isolated).

Eluent: Petroleum ether/Ethyl acetate (80/20, v/v).

TLC: R_f = 0.45 (70:30 petroleum ether:EtOAc).

Physical State: Yellow liquid.

^{1}H NMR (600 MHz, Chloroform-*d*) δ 7.79 (d, *J* = 7.6 Hz, 1H), 7.65 (td, *J* = 7.4, 1.2 Hz, 1H), 7.57 (s, 1H), 7.53 (d, *J* = 7.7 Hz, 1H), 7.45 – 7.41 (m, 2H), 7.39 (d, *J* = 8.1 Hz, 1H), 7.21 (t, *J* = 7.8 Hz, 1H), 7.07 (td, *J* = 7.7, 1.3 Hz, 1H), 6.73 (dd, *J* = 7.4, 1.3 Hz, 1H), 6.63 (td, *J* = 7.4, 1.0 Hz, 1H), 6.44 (d, *J* = 7.9 Hz, 1H), 4.42 (d, *J* = 16.5 Hz, 1H), 4.21 (d, *J* = 16.4 Hz, 1H), 3.85 (q, *J* = 6.5 Hz, 1H), 3.65 (d, *J* = 17.5 Hz, 1H), 3.21 (d, *J* = 17.5 Hz, 1H), 1.23 (d, *J* = 6.6 Hz, 3H) ppm.

^{13}C NMR (151 MHz, CDCl$_3$) δ 203.56, 152.72, 152.63, 141.39, 136.25, 135.17, 131.93, 130.45, 130.40, 130.38, 128.99, 128.26, 126.21, 126.17, 124.94, 122.84, 122.21, 118.51, 108.15, 69.45, 61.06, 50.24, 41.25, 13.45 ppm.

HRMS (m/z): [M + H$^+$] calcd for $C_{24}H_{21}ONBr$, 418.08010; found, 418.07957.

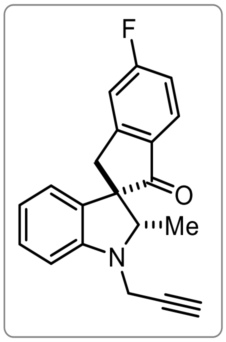

5-fluoro-2'-methyl-1'-(prop-2-yn-1-yl)spiro[indene-2,3'-indolin]-1(3H)-one [**C7**]: Compound **C7** was prepared according to the general procedure D (0.1 mmol scale).

Yield: 80% (24.4 mg isolated).

Eluent: Petroleum ether/Ethyl acetate (80/20, v/v).

TLC: R_f = 0.52 (70:30 petroleum ether:EtOAc).

Physical State: Colorless liquid.

^{1}H NMR (500 MHz, Chloroform-*d*) δ 7.78 (dd, *J* = 8.5, 5.3 Hz, 1H), 7.24 – 7.16 (m, 2H), 7.13 (td, *J* = 8.7, 2.3 Hz, 1H), 6.77 – 6.70 (m, 3H), 4.28 (dd, *J* = 18.3, 2.4 Hz, 1H), 3.93 – 3.84 (m, 2H), 3.64 (d, *J* = 17.9 Hz, 1H), 3.22 (d, *J* = 18.0 Hz, 1H), 2.08 (t, *J* = 2.3 Hz, 1H), 1.24 (d, *J* = 6.6 Hz, 3H) ppm.

^{13}C NMR (126 MHz, CDCl$_3$) δ 201.95, 168.63, 166.58 [167.61 (d, *J* = 257.1 Hz)], 155.68, 155.60 [155.64 (d, *J* = 10.1 Hz)], 151.06, 132.68, 132.47, 132.45 [132.46 (d, *J* = 1.8 Hz)], 128.88, 127.44, 127.36 [127.40 (d, *J* = 10.5 Hz)], 122.11, 120.13, 116.69, 116.51 [116.60 (d, *J* = 23.7 Hz)], 113.02, 112.84 [112.93 (d, *J* = 22.3 Hz)], 110.29, 77.64, 72.78, 67.27, 60.95, 40.66, 40.64 [40.65 (d, *J* = 2.0 Hz)], 35.08, 12.32 ppm.

^{19}F NMR (470 MHz, Chloroform-*d*) δ -102.16 (td, *J* = 8.5, 5.4 Hz) ppm.

HRMS (m/z): [M + H$^+$] calcd for $C_{20}H_{17}ONF$, 306.12887; found, 306.12890.

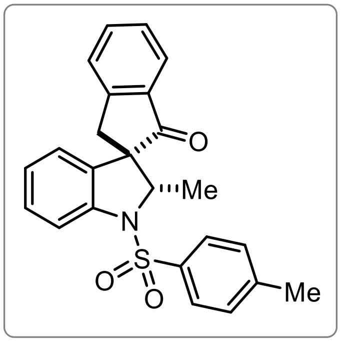

2'-methyl-1'-tosylspiro[indene-2,3'-indolin]-1(3H)-one [**C8**]: Compound **C8** was prepared according to the general procedure D (0.1 mmol scale).

Yield: 83% (33.5 mg isolated).

Eluent: Petroleum ether/Ethyl acetate (70/30, v/v).

TLC: R_f = 0.38 (60:40 petroleum ether:EtOAc).

Physical State: Colorless amorphous solid.

^{1}H NMR (700 MHz, Chloroform-*d*) δ 7.76 (dt, *J* = 7.7, 1.0 Hz, 1H), 7.74 (dt, *J* = 8.2, 0.8 Hz, 1H), 7.69 – 7.66 (m, 2H), 7.61 (td, *J* = 7.5, 1.2 Hz, 1H), 7.42 (td, *J* = 7.5, 1.0 Hz, 1H), 7.31 – 7.26 (m, 3H), 7.25 (d, *J* = 0.9 Hz, 1H), 7.03 (td, *J* = 7.5, 1.0 Hz, 1H), 6.77 (ddd, *J* = 7.5, 1.3, 0.6 Hz, 1H), 4.15 (q, *J* = 6.7 Hz, 1H), 2.85 (d, *J* = 17.3 Hz, 1H), 2.50 (d, *J* = 17.2 Hz, 1H), 2.44 (s, 3H), 1.41 (d, *J* = 6.7 Hz, 3H) ppm.

^{13}C NMR (176 MHz, $CDCl_3$) δ 204.12, 151.10, 144.41, 142.27, 136.69, 135.68, 135.44, 134.83, 129.91, 129.23, 128.39, 127.47, 126.35, 125.18, 124.53, 124.04, 116.92, 69.19, 62.35, 44.97, 21.82, 19.69 ppm.

HRMS (m/z): [M + H^+] calcd for $C_{24}H_{22}O_3NS$, 404.13149; found, 404.13140.

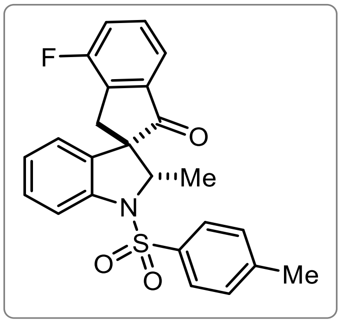

4-fluoro-2'-methyl-1'-tosylspiro[indene-2,3'-indolin]-1(3H)-one [**C9**]: Compound **C9** was prepared according to the general procedure D (0.1 mmol scale).

Yield: 88% (37.1 mg isolated).

Eluent: Petroleum ether/Ethyl acetate (70/30, v/v).

TLC: R_f = 0.4 (60:40 petroleum ether:EtOAc).

Physical State: Off-white solid.

^{1}H NMR (700 MHz, Chloroform-*d*) δ 7.77 (dt, *J* = 8.1, 0.9 Hz, 1H), 7.68 – 7.63 (m, 2H), 7.56 (dd, *J* = 7.5, 0.9 Hz, 1H), 7.44 – 7.39 (m, 1H), 7.32 (ddd, *J* = 8.2, 7.5, 1.3 Hz, 1H), 7.30 – 7.27 (m, 3H), 7.05 (td, *J* = 7.5, 1.0 Hz, 1H), 6.77 (ddd, *J* = 7.5, 1.3, 0.6 Hz, 1H), 4.11 (q, *J* = 6.7 Hz, 1H), 2.69 (d, *J* = 17.5 Hz, 1H), 2.43 (s, 3H), 2.34 (d, *J* = 17.5 Hz, 1H), 1.40 (d, *J* = 6.7 Hz, 3H) ppm.

^{13}C NMR (176 MHz, $CDCl_3$) δ 203.07, 160.42, 159.00 [159.71 (d, *J* = 250.9 Hz)], 144.81, 142.22, 139.37, 139.34 [139.35 (d, *J* = 4.5 Hz)], 137.00, 136.89 [136.94 (d, *J* = 19.5 Hz)], 135.07, 134.44, 130.39, 130.35 [130.37 (d, *J* = 6.2 Hz)], 130.04, 129.48, 127.40, 125.42, 124.03, 121.85, 121.73 [121.79 (d, *J* = 20.0 Hz)], 120.23, 120.21 [120.22 (d, *J* = 3.9 Hz)], 117.30, 77.41, 77.23, 77.05, 69.06, 62.32, 40.29, 21.74, 19.76 ppm.

^{19}F NMR (565 MHz, Chloroform-*d*) δ -119.18 (dd, *J* = 8.7, 4.6 Hz) ppm.

HRMS (m/z): [M + H^+] calcd for $C_{24}H_{21}O_3NFS$, 422.12207; found, 422.12191.

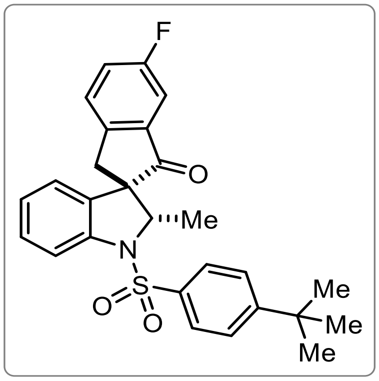

1'-((4-(tert-butyl)phenyl)sulfonyl)-6-fluoro-2'-methylspiro[indene-2,3'-indolin]-1(3H)-one [**C10**]: Compound **C10** was prepared according to the general procedure D (0.1 mmol scale).

Yield: 73% (33.8 mg isolated).

Eluent: Petroleum ether/Ethyl acetate (75/25, v/v).

TLC: R_f = 0.4 (70:30 petroleum ether:EtOAc).

Physical State: Yellow oil.

^{1}H NMR (600 MHz, Chloroform-*d*) δ 7.79 – 7.74 (m, 2H), 7.70 – 7.67 (m, 2H), 7.54 – 7.47 (m, 2H), 7.31 (ddd, *J* = 8.4, 7.5, 1.3 Hz, 1H), 7.10 (td, *J* = 8.6, 2.3 Hz, 1H), 7.06 (td, *J* = 7.5, 1.0 Hz, 1H), 6.82 (dd, *J* = 8.2, 2.2 Hz, 1H), 6.77 (ddd, *J* = 7.6, 1.3, 0.6 Hz, 1H), 4.10 (q, *J* = 6.7 Hz, 1H), 2.70 (d, *J* = 17.5 Hz, 1H), 2.27 (d, *J* = 17.5 Hz, 1H), 1.40 (d, *J* = 6.7 Hz, 3H), 1.35 (s, 9H) ppm.

^{13}C NMR (151 MHz, CDCl$_3$) δ 202.24, 168.64, 166.93 [167.78 (d, *J* = 258.1 Hz)], 157.62, 153.97, 153.91 [153.94 (d, *J* = 10.0 Hz)], 142.21, 135.24, 134.74, 133.08, 133.07 [133.07 (d, *J* = 1.9 Hz)], 129.38, 127.24, 126.91, 126.84 [126.87 (d, *J* = 10.4 Hz)], 126.30, 125.37, 123.96, 117.21, 116.76, 116.60 [116.68 (d, *J* = 23.7 Hz)], 113.02, 112.87 [112.95 (d, *J* = 22.4 Hz)], 69.03, 62.48, 44.58, 44.56 [44.57 (d, *J* = 2.0 Hz)], 35.47, 31.32, 19.67 ppm.

135-DEPT NMR (151 MHz, CDCl$_3$) δ 129.18, 127.04, 126.71, 126.64 [126.67 (d, *J* = 10.4 Hz)], 126.10, 125.17, 123.76, 117.01, 116.55, 116.40 [116.47 (d, *J* = 23.7 Hz)], 112.82, 112.67 [112.74 (d, *J* = 22.3 Hz)], 68.82, 44.37, 44.36 [44.37 (d, *J* = 1.7 Hz)], 31.12, 19.47 ppm.

^{19}F NMR (565 MHz, Chloroform-*d*) δ -100.99 (td, *J* = 8.5, 5.1 Hz) ppm.

HRMS (m/z): [M + H$^+$] calcd for $C_{27}H_{27}O_3NFS$, 464.16902; found, 464.16837.

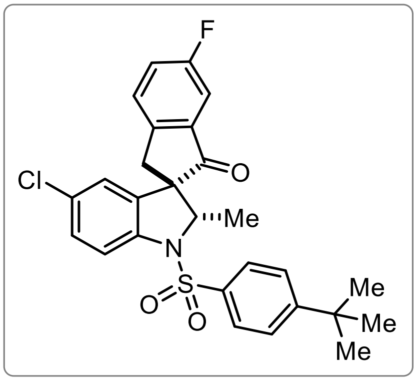

1'-((4-(tert-butyl)phenyl)sulfonyl)-5'-chloro-6-fluoro-2'-methylspiro[indene-2,3'-indolin]-1(3H)-one [**C11**]: Compound **C11** was prepared according to the general procedure D (0.1 mmol scale).

Yield: 78% (38.8 mg isolated).

Eluent: Petroleum ether/Ethyl acetate (70/30, v/v).

TLC: R_f = 0.42 (70:30 petroleum ether:EtOAc).

Physical State: Yellowish oil.

^{1}H NMR (500 MHz, Chloroform-*d*) δ 7.78 (dd, *J* = 8.5, 5.3 Hz, 1H), 7.73 – 7.66 (m, 3H), 7.57 – 7.52 (m, 2H), 7.32 – 7.26 (m, 1H), 7.13 (td, *J* = 8.6, 2.3 Hz, 1H), 6.84 (dd, *J* = 8.2, 2.2 Hz, 1H), 6.75 (d, *J* = 2.1 Hz, 1H), 4.11 (q, *J* = 6.7 Hz, 1H), 2.71 (d, *J* = 17.6 Hz, 1H), 2.26 (d, *J* = 17.5 Hz, 1H), 1.41 (d, *J* = 6.7 Hz, 3H), 1.38 (s, 9H) ppm.

^{13}C NMR (126 MHz, CDCl$_3$) δ 201.44, 168.87, 166.82 [167.85 (d, *J* = 258.7 Hz)], 157.90, 153.73, 153.65 [153.69 (d, *J* = 10.1 Hz)], 140.95, 136.41, 134.73, 132.72, 132.70 [132.71 (d, *J* = 1.8 Hz)], 130.39, 129.48, 127.21, 127.05, 126.97 [127.01 (d, *J* = 10.5 Hz)], 126.42, 124.22, 118.17, 116.97, 116.78 [116.87 (d, *J* = 23.7 Hz)], 113.06, 112.88 [112.97 (d, *J* = 22.6 Hz)], 69.31, 62.16, 44.26, 44.25 [44.26 (d, *J* = 1.8 Hz)], 35.50, 31.28, 19.58 ppm.

^{19}F NMR (470 MHz, Chloroform-*d*) δ -100.36 (td, *J* = 8.4, 5.4 Hz) ppm.

HRMS (m/z): [M + H$^+$] calcd for $C_{27}H_{26}O_3NClFS$, 498.13005; found, 498.12956.

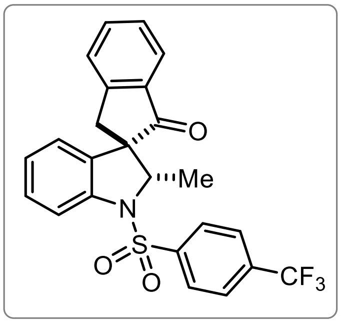

2'-methyl-1'-((4-(trifluoromethyl)phenyl)sulfonyl)spiro[indene-2,3'-indolin]-1(3H)-one [**C12**]: Compound **C12** was prepared according to the general procedure D (0.1 mmol scale).

Yield: 85% (38.9 mg isolated).

Eluent: Petroleum ether/Ethyl acetate (70/30, v/v).

TLC: R_f = 0.5 (60:40 petroleum ether:EtOAc).

Physical State: Off-white solid.

^{1}H NMR (500 MHz, Chloroform-*d*) δ 7.96 – 7.92 (m, 2H), 7.81 – 7.73 (m, 5H), 7.63 (td, *J* = 7.5, 1.2 Hz, 1H), 7.43 (td, *J* = 7.5, 1.0 Hz, 1H), 7.32 (ddd, *J* = 8.4, 7.5, 1.3 Hz, 1H), 7.27 – 7.24 (m, 2H), 7.07 (td, *J* = 7.5, 1.1 Hz, 1H), 6.80 (dd, *J* = 7.5, 1.3 Hz, 1H), 4.16 (q, *J* = 6.7 Hz, 1H), 2.86 (d, *J* = 17.1 Hz, 1H), 2.42 (d, *J* = 17.1 Hz, 1H), 1.44 (d, *J* = 6.7 Hz, 3H) ppm.

^{13}C NMR (126 MHz, $CDCl_3$) δ 203.62, 150.86, 141.79, 141.61, 136.44, 135.91, 135.58, 135.31, 135.05, 134.78 [134.99 (q, *J* = 33.2 Hz)], 134.82, 129.45, 128.55, 127.85, 126.55, 126.52, 126.49, 126.46 [126.32 (q, *J* = 3.7 Hz)], 126.36, 125.72, 124.62, 124.21, 126.60, 124.43, 122.26, 120.09 [123.15 (q, *J* = 273.0 Hz)] 116.68, 77.48, 77.23, 76.98, 69.44, 62.01, 44.71, 19.44 ppm.

135-DEPT NMR (126 MHz, $CDCl_3$) δ 135.72, 129.26, 128.36, 127.66, 126.36, 126.33, 126.30, 126.27 [126.32 (q, *J* = 3.7 Hz)], 126.17, 125.53, 124.43, 124.02, 116.49, 69.25, 44.52, 19.25 ppm.

^{19}F NMR (470 MHz, Chloroform-*d*) δ -63.09 ppm.

HRMS (m/z): [M + H$^+$] calcd for $C_{24}H_{18}O_3NF_3NaS$, 480.08517; found, 480.08475.

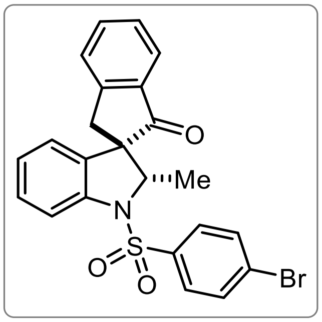

1'-((4-bromophenyl)sulfonyl)-2'-methylspiro[indene-2,3'-indolin]-1(3H)-one [**C13**]: Compound **C13** was prepared according to the general procedure D (0.1 mmol scale).

Yield: 80% (37.5 mg isolated).

Eluent: Petroleum ether/Ethyl acetate (70/30, v/v).

TLC: R_f = 0.35 (60:40 petroleum ether:EtOAc).

Physical State: Colorless solid.

^{1}H NMR (500 MHz, Chloroform-*d*) δ 7.76 (dt, *J* = 7.7, 1.0 Hz, 1H), 7.72 (dt, *J* = 8.2, 0.8 Hz, 1H), 7.69 – 7.61 (m, 5H), 7.43 (td, *J* = 7.6, 1.0 Hz, 1H), 7.35 – 7.28 (m, 2H), 7.05 (td, *J* = 7.5, 1.1 Hz, 1H), 6.79 (dd, *J* = 7.5, 1.3 Hz, 1H), 4.14 (q, *J* = 6.7 Hz, 1H), 2.93 (d, *J* = 17.2 Hz, 1H), 2.54 (d, *J* = 17.2 Hz, 1H), 1.43 (d, *J* = 6.7 Hz, 3H) ppm.

^{13}C NMR (126 MHz, $CDCl_3$) δ 203.80, 150.99, 141.82, 137.20, 136.48, 135.84, 134.80, 132.62, 129.35, 128.84, 128.58, 128.50, 126.44, 125.52, 124.58, 124.14, 116.70, 77.49, 77.23, 76.98, 69.33, 62.10, 44.89, 19.53 ppm.

HRMS (m/z): [M + H^+] calcd for $C_{23}H_{19}O_3NBrS$, 468.02635; found, 468.02604.

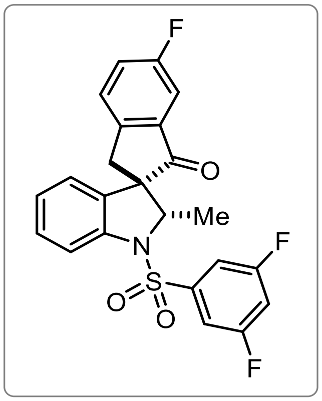

1'-((3,5-difluorophenyl)sulfonyl)-6-fluoro-2'-methylspiro[indene-2,3'-indolin]-1(3H)-one [**C14**]: Compound **C14** was prepared according to the general procedure D (0.1 mmol scale).

Yield: 82% (36.4 mg isolated).

Eluent: Petroleum ether/Ethyl acetate (70/30, v/v).

TLC: R_f = 0.5 (70:30 petroleum ether:EtOAc).

Physical State: Off-white solid.

^{1}H NMR (500 MHz, Chloroform-*d*) δ 7.78 (dd, *J* = 8.5, 5.2 Hz, 1H), 7.70 (d, *J* = 8.2 Hz, 1H), 7.37 – 7.29 (m, 3H), 7.13 (td, *J* = 8.6, 2.2 Hz, 1H), 7.10 – 7.04 (m, 2H), 7.02 (dd, *J* = 8.2, 2.2 Hz, 1H), 6.80 (dd, *J* = 7.6, 1.3 Hz, 1H), 4.19 (q, *J* = 6.7 Hz, 1H), 3.02 (d, *J* = 16.9 Hz, 1H), 2.68 (d, *J* = 17.4 Hz, 1H), 1.44 (d, *J* = 6.8 Hz, 3H) ppm.

^{13}C NMR (126 MHz, $CDCl_3$) δ 201.56, 168.92, 166.87 [167.90 (d, *J* = 258.4 Hz)], 164.02, 161.99 [163.00 (d, *J* = 255.6 Hz)], 163.93, 161.89 [162.91 (d, *J* = 255.6 Hz)], 153.89, 153.81 [153.85 (d, *J* = 10.2 Hz)], 141.38, 141.32, 141.26, 141.19 [141.26 (t, *J* = 8.0 Hz)], 134.34, 132.73, 132.71 [132.72 (d, *J* = 1.9 Hz)], 129.64, 127.10, 127.02 [127.06 (d, *J* = 10.7 Hz)], 125.80, 124.10, 117.03, 116.84 [116.93 (d, *J* = 23.7 Hz)], 116.45, 113.35, 113.18 [113.26 (d, *J* = 22.6 Hz)], 111.03, 110.81 [110.92 (d, *J* = 28.5 Hz)], 110.97, 110.87 [110.92 (d, *J* = 13.5 Hz)], 109.35, 109.15, 108.95 [109.15 (t, *J* = 24.9 Hz)], 69.43, 62.12, 44.47, 44.45 [44.46 (d, *J* = 1.9 Hz)], 19.21 ppm.

^{19}F NMR (470 MHz, Chloroform-*d*) δ -100.51 (td, *J* = 8.6, 5.6 Hz), -104.70 – -105.00 (m) ppm.

HRMS (m/z): [M + H$^+$] calcd for $C_{23}H_{17}O_3NF_3S$, 444.08758; found, 444.08728.

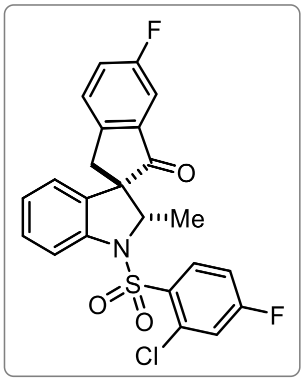

1'-((2-chloro-4-fluorophenyl)sulfonyl)-6-fluoro-2'-methylspiro[indene-2,3'-indolin]-1(3H)-one [**C15**]: Compound **C15** was prepared according to the general procedure D (0.1 mmol scale).

Yield: 66% (30.4 mg isolated).

Eluent: Petroleum ether/Ethyl acetate (75/25, v/v).

TLC: R_f = 0.45 (70:30 petroleum ether:EtOAc).

Physical State: Brown solid.

^{1}H NMR (700 MHz, Chloroform-*d*) δ 8.23 (dd, *J* = 8.9, 5.7 Hz, 1H), 7.79 (dd, *J* = 8.4, 5.2 Hz, 1H), 7.47 (dt, *J* = 8.2, 0.7 Hz, 1H), 7.30 (dd, *J* = 8.1, 2.5 Hz, 1H), 7.23 (ddd, *J* = 8.4, 7.5, 1.3 Hz, 1H), 7.14 (ddt, *J* = 9.0, 7.5, 2.4 Hz, 2H), 7.10 (dd, *J* = 8.2, 2.2 Hz, 1H), 7.02 (td, *J* = 7.5, 1.0 Hz,

1H), 6.85 (dd, J = 7.7, 1.3 Hz, 1H), 4.50 (q, J = 6.6 Hz, 1H), 3.30 – 3.19 (m, 2H), 1.35 (d, J = 6.6 Hz, 3H) ppm.

^{13}C NMR (176 MHz, CDCl$_3$) δ 201.92, 168.66, 167.20 [167.93 (d, J = 258.4 Hz)], 165.61, 164.14 [164.87 (d, J = 259.3 Hz)], 154.32, 154.26 [154.29 (d, J = 10.1 Hz)], 141.97, 134.88, 134.82 [134.85 (d, J = 11.0 Hz)], 134.21, 134.15 [134.18 (d, J = 9.9 Hz)], 134.12, 134.10 [134.11 (d, J = 3.5 Hz)], 133.81, 132.97, 132.96 [132.96 (d, J = 1.7 Hz)], 129.10, 127.02, 126.96 [126.99 (d, J = 10.5 Hz)], 124.79, 123.93, 120.07, 119.92 [120.00 (d, J = 25.3 Hz)], 116.90, 116.76 [116.83 (d, J = 23.8 Hz)], 116.11, 114.73, 114.60 [114.67 (d, J = 21.5 Hz)], 113.45, 113.32 [113.38 (d, J = 22.4 Hz)], 69.36, 62.44, 43.87, 43.86 [43.86 (d, J = 1.9 Hz)], 18.28 ppm.

^{19}F NMR (565 MHz, Chloroform-*d*) δ -100.70 (td, J = 8.7, 5.7 Hz), -102.72 (q, J = 7.5 Hz) ppm.

HRMS (m/z): [M + H$^+$] calcd for $C_{23}H_{17}O_3NClF_2S$, 460.05802; found, 460.05774.

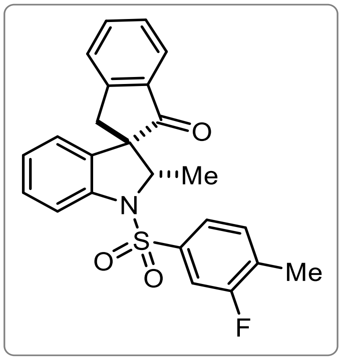

1'-((3-fluoro-4-methylphenyl)sulfonyl)-2'-methylspiro[indene-2,3'-indolin]-1(3H)-one [**C16**]: Compound **C16** was prepared according to the general procedure D (0.1 mmol scale).

Yield: 75% (31.6 mg isolated).

Eluent: Petroleum ether/Ethyl acetate (75/25, v/v).

TLC: R_f = 0.45 (70:30 petroleum ether:EtOAc).

Physical State: White semi-solid.

^{1}H NMR (500 MHz, Chloroform-*d*) δ 7.79 – 7.74 (m, 1H), 7.72 (dt, J = 8.1, 0.7 Hz, 1H), 7.63 (td, J = 7.5, 1.2 Hz, 1H), 7.48 (dd, J = 7.9, 1.9 Hz, 1H), 7.46 – 7.40 (m, 2H), 7.35 – 7.27 (m, 3H), 7.04 (td, J = 7.5, 1.0 Hz, 1H), 6.79 (dd, J = 7.7, 1.3 Hz, 1H), 4.17 (q, J = 6.7 Hz, 1H), 2.95 (d, J = 17.1 Hz, 1H), 2.61 (d, J = 17.1 Hz, 1H), 2.35 (d, J = 2.0 Hz, 3H), 1.43 (d, J = 6.7 Hz, 3H) ppm.

^{13}C NMR (126 MHz, CDCl$_3$) δ 203.85, 161.94, 159.95 [160.94 (d, J = 250.5 Hz)], 151.03, 141.89, 137.20, 137.15 [137.17 (d, J = 6.2 Hz)], 136.49, 135.79, 134.68, 132.36, 132.32 [132.34 (d, J = 4.9 Hz)], 131.60, 131.46 [131.53 (d, J = 17.2 Hz)], 129.30, 128.45, 126.40, 125.36, 124.53, 124.06,

122.98, 122.95 [122.96 (d, J = 3.8 Hz)], 116.61, 114.43, 114.23 [114.33 (d, J = 25.5 Hz)], 77.48, 77.23, 76.98, 69.27, 62.14, 44.86, 19.51, 15.06, 15.03 [15.05 (d, J = 3.5 Hz)] ppm.

135-DEPT NMR (126 MHz, $CDCl_3$) δ 135.64, 132.21, 132.17 [132.19 (d, J = 5.0 Hz)], 129.15, 128.30, 126.25, 125.21, 124.38, 123.91, 122.82, 122.79 [122.81 (d, J = 3.7 Hz)], 116.46, 114.28, 114.08 [114.18 (d, J = 25.5 Hz)], 69.12, 44.70, 19.35, 14.91, 14.88 [14.90 (d, J = 3.4 Hz)] ppm.

^{19}F NMR (470 MHz, Chloroform-*d*) δ -113.35 (t, J = 8.2 Hz) ppm.

HRMS (m/z): [M + Na^+] calcd for $C_{24}H_{20}O_3NFNaS$, 444.10401; found, 444.10340.

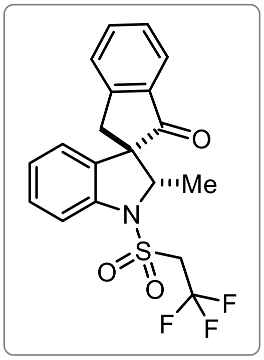

2'-methyl-1'-((2,2,2-trifluoroethyl)sulfonyl)spiro[indene-2,3'-indolin]-1(3H)-one [**C17**]: Compound **C17** was prepared according to the general procedure D (0.1 mmol scale).

Yield: 72% (28.5 mg).

Eluent: Petroleum ether/Ethyl acetate (70/30, v/v).

TLC: R_f = 0.45 (70:30 petroleum ether:EtOAc).

Physical State: Colorless solid.

^{1}H NMR (700 MHz, Chloroform-*d*) δ 7.79 (dt, J = 7.7, 1.0 Hz, 1H), 7.70 (td, J = 7.5, 1.3 Hz, 1H), 7.53 (dt, J = 7.7, 1.0 Hz, 1H), 7.49 – 7.45 (m, 2H), 7.29 (ddd, J = 8.4, 7.4, 1.3 Hz, 1H), 7.06 (td, J = 7.5, 1.0 Hz, 1H), 6.88 (dd, J = 7.6, 1.3 Hz, 1H), 4.51 (q, J = 6.7 Hz, 1H), 3.96 (dq, J = 15.0, 8.9 Hz, 1H), 3.88 (dq, J = 15.0, 9.3 Hz, 1H), 3.51 (d, J = 17.3 Hz, 1H), 3.44 (d, J = 17.3 Hz, 1H), 1.50 (d, J = 6.7 Hz, 3H) ppm.

^{13}C NMR (176 MHz, $CDCl_3$) δ 203.32, 151.65, 141.13, 136.06, 136.01, 134.13, 129.52, 128.65, 126.59, 125.32, 124.82, 124.09, 124.21, 122.63, 121.05, 119.47 [121.84 (q, J = 277.9 Hz)], 114.72, 69.74, 61.58, 53.38, 53.20, 53.01, 52.85 [53.11 (q, J = 29.6, 26.8 Hz)], 43.67, 18.13 ppm.

^{19}F NMR (470 MHz, Chloroform-*d*) δ -61.02 (t, J = 9.1 Hz) ppm.

HRMS (m/z): [M + H^+] calcd for $C_{19}H_{17}O_3NF_3S$, 396.08758; found, 396.08744.

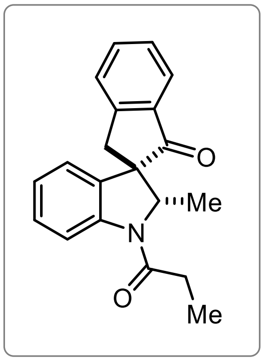

2'-methyl-1'-propionylspiro[indene-2,3'-indolin]-1(3H)-one [**C18**]: Compound **C18** was prepared according to the general procedure D (0.1 mmol scale).

Yield: 84% (25.7 mg isolated).

Eluent: Petroleum ether/Ethyl acetate (70/30, v/v).

TLC: R_f = 0.5 (60:40 petroleum ether:EtOAc).

Physical State: Colorless liquid.

^{1}H NMR (500 MHz, DMSO-*d*$_6$) δ 7.77 (t, *J* = 7.4 Hz, 2H), 7.59 (dt, *J* = 7.4, 1.1 Hz, 1H), 7.55 – 7.48 (m, 1H), 7.26 (ddd, *J* = 8.3, 7.4, 1.5 Hz, 1H), 7.03 (td, *J* = 7.4, 1.1 Hz, 1H), 6.98 (dd, *J* = 7.5, 1.4 Hz, 1H), 4.56 (q, *J* = 6.5 Hz, 1H), 3.67 (d, *J* = 16.9 Hz, 1H), 3.10 (d, *J* = 16.8 Hz, 1H), 2.78 – 2.61 (m, 1H), 2.48 – 2.33 (m, 1H), 1.11 (d, *J* = 6.5 Hz, 3H), 1.08 (t, *J* = 7.3 Hz, 3H).

^{13}C NMR (126 MHz, DMSO-*d*6) δ 204.25, 171.21, 151.59, 142.05, 136.52, 135.70, 134.62, 128.11, 128.01, 127.05, 124.30, 123.92, 123.40, 116.78, 66.03, 61.74, 43.95, 27.40, 18.08, 9.10.

HRMS (m/z): [M + H^+] calcd for $C_{20}H_{20}O_2N$ 306.14886; found, 306.14886.

CLASS-D

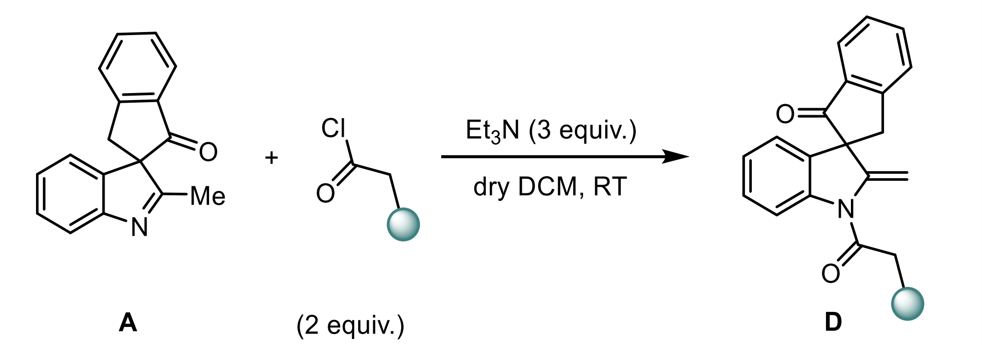

General procedure E: Synthesis of Class D

An oven-dried screw capped reaction tube with a magnetic stir-bar was charged with 2'-methylspiro[indene-2,3'-indol]-1(3H)-one derivative (0.1 mmol) and acyl chloride derivative (2 equiv., 0.2 mmol). A screw cap fitted with a rubber septum was attached to the reaction tube. Dry dichloromethane (2 mL) was added in the reaction tube followed by triethylamine (3 equiv., 0.3 mmol) added dropwise under positive flow of argon. The mixture was stirred at room temperature for 24 h. After maximum conversion (checked by TLC), the mixture was taken out and diluted with 10 mL of dichloromethane and transferred to a round bottom flask. The solvent was evaporated by rotavapor. The crude mixture was purified by column chromatography using silica gel (100-200 mesh size) and petroleum ether/ethyl acetate as the eluent.

Characterization data for Class D products

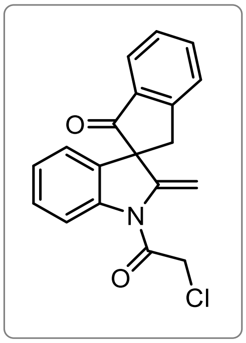

1'-(2-chloroacetyl)-2'-methylenespiro[indene-2,3'-indolin]-1(3H)-one [**D1**]: Compound **D1** was prepared according to the general procedure E (0.1 mmol scale).

Yield: 71% (23 mg isolated).

Eluent: Petroleum ether/Ethyl acetate (80/20, v/v).

TLC: R_f = 0.4 (75:25 petroleum ether:EtOAc).

Physical State: Yellow oil.

^{1}**H NMR (500 MHz, Chloroform-*d*) δ** 8.08 – 8.00 (m, 1H), 7.81 (dt, *J* = 7.7, 1.0 Hz, 1H), 7.73 (td, *J* = 7.5, 1.2 Hz, 1H), 7.60 (dt, *J* = 7.6, 1.0 Hz, 1H), 7.50 (td, *J* = 7.4, 0.9 Hz, 1H), 7.31 (ddd, *J* = 8.4, 7.5, 1.3 Hz, 1H), 7.09 (td, *J* = 7.5, 1.0 Hz, 1H), 6.89 (dd, *J* = 7.5, 1.3 Hz, 1H), 5.32 (d, *J* = 3.3 Hz, 1H), 4.70 (d, *J* = 3.3 Hz, 1H), 4.55 (d, *J* = 12.9 Hz, 1H), 4.45 (d, *J* = 12.8 Hz, 1H), 3.68 (d, *J* = 17.7 Hz, 1H), 3.57 (d, *J* = 17.8 Hz, 1H) ppm.

^{13}**C NMR (126 MHz, $CDCl_3$) δ** 201.21, 165.37, 152.71, 150.46, 142.94, 135.96, 133.92, 133.04, 129.07, 128.80, 126.51, 126.04, 125.66, 122.00, 117.27, 97.21, 77.48, 77.23, 76.98, 61.88, 43.20, 42.64 ppm.

HRMS (m/z): [M + H$^+$] calcd for $C_{19}H_{15}O_2NCl$, 324.07858; found, 324.07891.

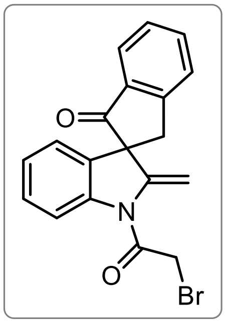

1'-(2-bromoacetyl)-2'-methylenespiro[indene-2,3'-indolin]-1(3H)-one [**D2**]: Compound **D2** was prepared according to the general procedure E (0.1 mmol scale).

Yield: 67% (24.7 mg isolated).

Eluent: Petroleum ether/Ethyl acetate (80/20, v/v).

TLC: R_f = 0.4 (75:25 petroleum ether:EtOAc).

Physical State: Yellow oil.

^{1}**H NMR (500 MHz, Methylene Chloride-d_2) δ** 8.02 (d, *J* = 8.0 Hz, 1H), 7.79 (dt, *J* = 7.7, 1.0 Hz, 1H), 7.75 (td, *J* = 7.5, 1.2 Hz, 1H), 7.64 (dt, *J* = 7.7, 1.0 Hz, 1H), 7.51 (td, *J* = 7.4, 1.0 Hz, 1H), 7.33 (ddd, *J* = 8.4, 7.5, 1.3 Hz, 1H), 7.12 (td, *J* = 7.6, 1.0 Hz, 1H), 6.93 (dd, *J* = 7.5, 1.3 Hz, 1H), 5.27 (d, *J* = 3.4 Hz, 1H), 4.70 (d, *J* = 3.3 Hz, 1H), 4.57 (d, *J* = 13.1 Hz, 1H), 4.48 (d, *J* = 13.1 Hz, 1H), 3.70 (d, *J* = 17.8 Hz, 1H), 3.58 (d, *J* = 17.8 Hz, 1H) ppm.

^{13}**C NMR (126 MHz, CD_2Cl_2) δ** 200.98, 165.07, 152.75, 150.37, 142.88, 135.71, 133.67, 133.05, 128.71, 128.52, 126.45, 125.53, 125.35, 121.94, 116.85, 96.77, 61.76, 43.33, 42.26 ppm.

135 DEPT NMR (126 MHz, CD_2Cl_2) δ 135.75, 128.75, 128.55, 126.48, 125.56, 125.38, 121.97, 116.88, 96.81, 43.36, 42.29 ppm.

HRMS (m/z): [M + H$^+$] calcd for $C_{19}H_{15}O_2NBr$, 368.02807; found, 368.02813.

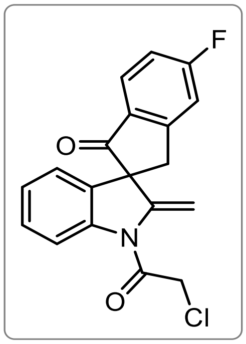

1'-(2-chloroacetyl)-5-fluoro-2'-methylenespiro[indene-2,3'-indolin]-1(3H)-one [**D3**]: Compound **D3** was prepared according to the general procedure E (0.1 mmol scale).

Yield: 78% (26.7 mg isolated).

Eluent: Petroleum ether/Ethyl acetate (80/20, v/v).

TLC: R_f = 0.42 (75:25 petroleum ether:EtOAc).

Physical State: Colorless oil.

^{1}H NMR (500 MHz, Chloroform-*d*) δ 8.04 (d, *J* = 8.1 Hz, 1H), 7.82 (dd, *J* = 8.5, 5.3 Hz, 1H), 7.32 (ddd, *J* = 8.4, 7.5, 1.3 Hz, 1H), 7.30 – 7.26 (m, 1H), 7.20 (td, *J* = 8.6, 2.3 Hz, 1H), 7.10 (td, *J* = 7.5, 1.0 Hz, 1H), 6.89 (dd, *J* = 7.6, 1.3 Hz, 1H), 5.35 (d, *J* = 3.4 Hz, 1H), 4.69 (d, *J* = 3.3 Hz, 1H), 4.55 (d, *J* = 12.8 Hz, 1H), 4.44 (d, *J* = 12.8 Hz, 1H), 3.70 – 3.52 (m, 2H) ppm.

^{13}C NMR (126 MHz, $CDCl_3$) δ 199.35, <u>168.96, 166.90</u> [167.93 (d, *J* = 258.8 Hz)], 165.34, <u>155.63, 155.55</u> [155.59 (d, *J* = 10.4 Hz)], 150.29, 142.90, 132.71, <u>130.25, 130.24</u> [130.25 (d, *J* = 1.9 Hz)], 129.26, <u>128.44, 128.36</u> [128.40 (d, *J* = 10.6 Hz)], 125.72, 121.96, 117.31, <u>117.36, 117.17</u> [117.27 (d, *J* = 23.9 Hz)], <u>113.48, 113.30</u> [113.39 (d, *J* = 22.7 Hz)], 97.31, 62.10, 43.17, 42.47, 42.46 [42.46 (d, *J* = 2.1 Hz)] ppm.

DEPT-135 NMR (126 MHz, $CDCl_3$) δ 129.10, <u>128.29, 128.20</u> [128.24 (d, *J* = 10.5 Hz)], 125.56, 121.81, 117.15, <u>117.21, 117.02</u> [117.11 (d, *J* = 23.9 Hz)], <u>113.32, 113.14</u> [113.23 (d, *J* = 22.6 Hz)], 97.15, 43.02, <u>42.31, 42.30</u> [42.31 (d, *J* = 2.0 Hz)] ppm.

^{19}F NMR (470 MHz, Chloroform-*d*) δ -100.41 (td, *J* = 8.6, 5.2 Hz) ppm.

HRMS (m/z): [M + H^+] calcd for $C_{19}H_{14}O_2NClF$, 342.06916; found, 342.06939.

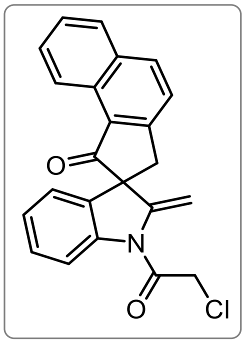

1'-(2-chloroacetyl)-2'-methylenespiro[cyclopenta[a]naphthalene-2,3'-indolin]-1(3H)-one [**D4**]: Compound **D4** was prepared according to the general procedure E (0.1 mmol scale).

Yield: 74% (27.7 mg isolated).

Eluent: Petroleum ether/Ethyl acetate (80/20, v/v).

TLC: R_f = 0.4 (75:25 petroleum ether:EtOAc).

Physical State: Colorless oil.

^{1}H NMR (700 MHz, CD_2Cl_2) δ 8.97 (dq, J = 8.4, 1.0 Hz, 1H), 8.24 – 8.21 (m, 1H), 8.08 – 8.04 (m, 1H), 8.02 – 7.98 (m, 1H), 7.72 – 7.67 (m, 2H), 7.63 (ddd, J = 8.2, 6.9, 1.3 Hz, 1H), 7.38 – 7.32 (m, 1H), 7.12 (td, J = 7.5, 1.0 Hz, 1H), 6.96 (ddd, J = 7.5, 1.4, 0.5 Hz, 1H), 5.30 (d, J = 3.4 Hz, 1H), 4.75 (d, J = 3.4 Hz, 1H), 4.61 (d, J = 13.1 Hz, 1H), 4.53 (d, J = 13.1 Hz, 1H), 3.79 (d, J = 18.1 Hz, 1H), 3.69 (d, J = 18.1 Hz, 1H) ppm.

^{13}C NMR (176 MHz, CD_2Cl_2) δ 201.59, 165.22, 156.58, 150.54, 143.08, 136.98, 133.38, 130.12, 129.47, 128.76, 128.54, 127.63, 127.16, 125.42, 123.85, 123.66, 121.99, 116.97, 96.64, 62.20, 43.48, 42.91 ppm.

135 DEPT NMR (176 MHz, CD_2Cl_2) δ 136.93, 129.42, 128.71, 128.49, 127.11, 125.37, 123.80, 123.61, 121.94, 116.92, 96.60, 43.43, 42.86 ppm.

HRMS (m/z): [M + H^+] calcd for $C_{23}H_{17}O_2NCl$, 374.09423; found, 374.09434.

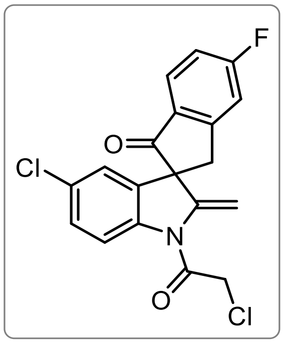

5'-chloro-1'-(2-chloroacetyl)-5-fluoro-2'-methylenespiro[indene-2,3'-indolin]-1(3H)-one [**D5**]: Compound **D5** was prepared according to the general procedure E (0.1 mmol scale).

Yield: 81% (30.5 mg).

Eluent: Petroleum ether/Ethyl acetate (80/20, v/v).

TLC: R_f = 0.52 (75:25 petroleum ether:EtOAc).

Physical State: Yellow liquid.

^{1}H NMR (500 MHz, Chloroform-*d*) δ 8.06 (d, *J* = 8.7 Hz, 1H), 7.83 (dd, *J* = 8.5, 5.3 Hz, 1H), 7.29 (dd, *J* = 8.8, 2.2 Hz, 2H), 7.22 (td, *J* = 8.6, 2.3 Hz, 1H), 6.86 (d, *J* = 2.2 Hz, 1H), 5.27 (d, *J* = 3.7 Hz, 1H), 4.71 (d, *J* = 3.7 Hz, 1H), 4.53 (d, *J* = 12.7 Hz, 1H), 4.41 (d, *J* = 12.8 Hz, 1H), 3.69 – 3.51 (m, 2H) ppm.

^{13}C NMR (126 MHz, $CDCl_3$) δ 198.56, <u>169.11, 167.04</u> [168.07 (d, *J* = 259.5 Hz)], 165.30, <u>155.34, 155.26</u> [155.30 (d, *J* = 10.4 Hz)], 149.92, 141.62, 134.42, 130.94, <u>129.91, 129.90</u> [129.91 (d, *J* = 1.8 Hz)], 129.21, <u>128.65, 128.56</u> [128.60 (d, *J* = 10.6 Hz)], 122.20, 118.64, <u>117.63, 117.44</u> [117.54 (d, *J* = 23.8 Hz)], <u>113.58, 113.40</u> [113.49 (d, *J* = 22.9 Hz)], 97.41, 61.95, 42.95, <u>42.16, 42.14</u> [42.15 (d, *J* = 2.1 Hz)] ppm.

135-DEPT NMR (126 MHz, $CDCl_3$) δ 129.04, <u>128.47, 128.39</u> [128.43 (d, *J* = 10.6 Hz)], 122.02, 118.46, <u>117.46, 117.27</u> [117.37 (d, *J* = 23.8 Hz)], <u>113.40, 113.22</u> [113.31 (d, *J* = 22.7 Hz)], 97.23, 42.78, <u>41.98, 41.97</u> [41.98 (d, *J* = 2.0 Hz)] ppm.

^{19}F NMR (470 MHz, Chloroform-*d*) δ -99.77 (td, *J* = 8.3, 5.1 Hz) ppm.

HRMS (m/z): [M + H^+] calcd for $C_{19}H_{13}O_2NCl_2F$, 376.03019; found, 376.02985.

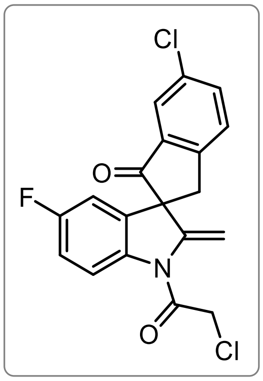

6-chloro-1'-(2-chloroacetyl)-5'-fluoro-2'-methylenespiro[indene-2,3'-indolin]-1(3H)-one [**D6**]: Compound **D6** was prepared according to the general procedure E (0.1 mmol scale).

Yield: 80% (30 mg isolated).

Eluent: Petroleum ether/Ethyl acetate (80/20, v/v).

TLC: R_f = 0.52 (75:25 petroleum ether:EtOAc).

Physical State: Yellow oil.

^{1}H NMR (500 MHz, Chloroform-*d*) δ 8.10 (dd, *J* = 9.0, 4.6 Hz, 1H), 7.77 (d, *J* = 2.0 Hz, 1H), 7.70 (dd, *J* = 8.1, 2.1 Hz, 1H), 7.56 (dt, *J* = 8.2, 0.9 Hz, 1H), 7.01 (td, *J* = 8.9, 2.7 Hz, 1H), 6.60 (dd, *J* = 7.6, 2.7 Hz, 1H), 5.27 (d, *J* = 3.7 Hz, 1H), 4.70 (d, *J* = 3.6 Hz, 1H), 4.55 – 4.37 (m, 2H), 3.65 – 3.51 (m, 2H) ppm.

^{13}C NMR (126 MHz, $CDCl_3$) δ 199.13, 165.13, <u>161.45, 159.50</u> [160.48 (d, *J* = 245.6 Hz)], 150.40, 149.94, <u>139.14, 139.12</u> [139.13 (d, *J* = 2.5 Hz)], 136.22, 135.47, 135.21, <u>134.29, 134.22</u> [134.25 (d, *J* = 8.4 Hz)], 127.72, 125.82, <u>118.94, 118.87</u> [118.90 (d, *J* = 8.2 Hz)], <u>115.81, 115.63</u> [115.72 (d, *J* = 22.8 Hz)], <u>109.60, 109.40</u> [109.50 (d, *J* = 24.9 Hz)], 97.60, <u>62.40, 62.38</u> [62.39 (d, *J* = 2.1 Hz)], 42.89, 41.80 ppm.

^{19}F NMR (470 MHz, Chloroform-*d*) δ -115.93 (td, *J* = 8.2, 4.6 Hz) ppm.

HRMS (m/z): [M + H^+] calcd for $C_{19}H_{13}Cl_2FNO_2$, 376.0307; found, 376.00907.

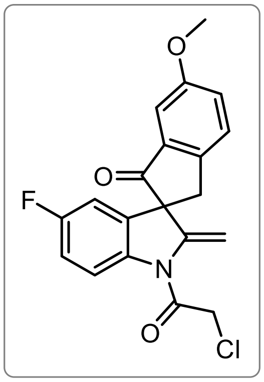

1'-(2-chloroacetyl)-5'-fluoro-6-methoxy-2'-methylenespiro[indene-2,3'-indolin]-1(3H)-one [**D7**]: Compound **D7** was prepared according to the general procedure E (0.1 mmol scale).

Yield: 75% (27.9 mg isolated).

Eluent: Petroleum ether/Ethyl acetate (70/30, v/v).

TLC: R_f = 0.3 (70:30 petroleum ether:EtOAc).

Physical State: Colorless solid.

^{1}H NMR (500 MHz, Chloroform-*d*) δ 8.11 (dd, *J* = 9.0, 4.6 Hz, 1H), 7.49 (dd, *J* = 8.4, 0.8 Hz, 1H), 7.32 (dd, *J* = 8.4, 2.6 Hz, 1H), 7.22 (d, *J* = 2.5 Hz, 1H), 7.00 (td, *J* = 8.9, 2.7 Hz, 1H), 6.61 (dd, *J* = 7.7, 2.7 Hz, 1H), 5.24 (d, *J* = 3.5 Hz, 1H), 4.72 (d, *J* = 3.6 Hz, 1H), 4.54 (d, *J* = 12.7 Hz, 1H), 4.42 (d, *J* = 12.8 Hz, 1H), 3.87 (s, 3H), 3.57 (dd, *J* = 17.4, 0.9 Hz, 1H), 3.49 (dd, *J* = 17.4, 0.9 Hz, 1H) ppm.

^{13}C NMR (126 MHz, CDCl$_3$) δ 200.49, 165.18, 160.61, 161.49, 159.54 [160.51 (d, *J* = 245.2 Hz)], 150.35, 145.25, 139.16, 139.14 [139.15 (d, *J* = 2.5 Hz)], 134.92, 134.94, 134.87 [134.91 (d, *J* = 8.3 Hz)], 127.25, 125.70, 118.86, 118.80 [118.83 (d, *J* = 8.2 Hz)], 115.56, 115.38 [115.47 (d, *J* = 22.8 Hz)], 109.59, 109.40 [109.50 (d, *J* = 24.8 Hz)], 106.95, 97.31, 62.71, 62.69 [62.70 (d, *J* = 2.0 Hz)], 55.95, 42.99, 41.89 ppm.

^{19}F NMR (470 MHz, Chloroform-*d*) δ -116.21 (td, *J* = 8.2, 4.5 Hz).

HRMS (m/z): [M + H$^+$] calcd for $C_{20}H_{16}O_3NClF$, 372.07973; found, 372.07991.

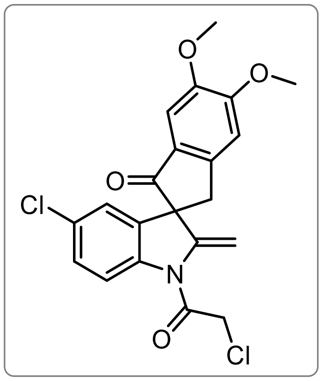

5'-chloro-1'-(2-chloroacetyl)-5,6-dimethoxy-2'-methylenespiro[indene-2,3'-indolin]-1(3H)-one [**D8**]: Compound **D8** was prepared according to the general procedure E (0.1 mmol scale).

Yield: 72% (30 mg isolated).

Eluent: Petroleum ether/Ethyl acetate (60/40, v/v).

TLC: R_f = 0.28 (60:40 petroleum ether:EtOAc).

Physical State: Off-white solid.

^{1}H NMR (700 MHz, Chloroform-*d*) δ 8.06 (d, *J* = 8.7 Hz, 1H), 7.29 – 7.26 (m, 1H), 7.19 (s, 1H), 7.00 (s, 1H), 6.86 (d, *J* = 2.2 Hz, 1H), 5.24 (d, *J* = 3.6 Hz, 1H), 4.71 (d, *J* = 3.6 Hz, 1H), 4.54 (d, *J* = 12.8 Hz, 1H), 4.42 (d, *J* = 12.9 Hz, 1H), 4.04 (s, 3H), 3.93 (s, 3H), 3.58 – 3.44 (m, 2H) ppm.

^{13}C NMR (176 MHz, CDCl$_3$) δ 199.23, 165.37, 156.80, 150.75, 150.46, 148.04, 141.68, 135.22, 130.88, 128.95, 126.24, 122.20, 118.55, 107.40, 105.98, 96.94, 62.12, 56.70, 56.46, 43.08, 42.71 ppm.

HRMS (m/z): [M + H$^+$] calcd for $C_{21}H_{18}O_4NCl_2$, 418.06074; found, 418.06079.

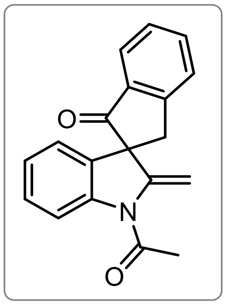

1'-acetyl-2'-methylenespiro[indene-2,3'-indolin]-1(3H)-one [**D9**]: Compound **D9** was prepared according to the general procedure E (0.1 mmol scale).

Yield: 82% (23.7 mg isolated).

Eluent: Petroleum ether/Ethyl acetate (75/25, v/v).

TLC: R_f = 0.35 (70:30 petroleum ether:EtOAc).

Physical State: Colorless solid.

^{1}H NMR (500 MHz, Chloroform-*d*) δ 8.00 (d, *J* = 8.2 Hz, 1H), 7.83 (dt, *J* = 7.7, 1.0 Hz, 1H), 7.71 (td, *J* = 7.5, 1.2 Hz, 1H), 7.59 (dt, *J* = 7.7, 0.9 Hz, 1H), 7.49 (td, *J* = 7.4, 1.0 Hz, 1H), 7.28 (ddd, *J* = 8.5, 7.5, 1.4 Hz, 1H), 7.04 (td, *J* = 7.5, 1.0 Hz, 1H), 6.86 (dd, *J* = 7.6, 1.4 Hz, 1H), 5.21 (d, *J* = 2.9 Hz, 1H), 4.57 (d, *J* = 2.9 Hz, 1H), 3.73 – 3.51 (m, 2H), 2.54 (s, 3H) ppm.

^{13}C NMR (126 MHz, $CDCl_3$) δ 201.75, 169.77, 152.93, 151.12, 143.75, 135.77, 134.31, 132.83, 128.85, 128.66, 126.48, 126.01, 124.83, 121.93, 117.08, 96.12, 62.07, 43.70, 26.27 ppm.

DEPT-135 NMR (126 MHz, $CDCl_3$) δ 135.61, 128.70, 128.51, 126.33, 125.86, 124.67, 121.78, 116.93, 95.97, 43.54, 26.12 ppm.

HRMS (m/z): [M + H^+] calcd for $C_{19}H_{16}O_2N$, 290.11756; found, 290.11769.

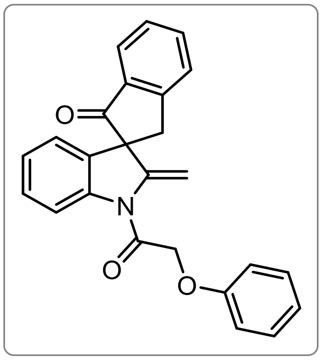

2'-methylene-1'-(2-phenoxyacetyl)spiro[indene-2,3'-indolin]-1(3H)-one [**D10**]: Compound **D10** was prepared according to the general procedure E (0.1 mmol scale).

Yield: 69% (26.3 mg isolated).

Eluent: Petroleum ether/Ethyl acetate (70/30, v/v).

TLC: R_f = 0.25 (70:30 petroleum ether:EtOAc).

Physical State: Colorless solid.

^{1}H NMR (500 MHz, Chloroform-*d*) δ 8.04 (d, *J* = 8.1 Hz, 1H), 7.84 (dd, *J* = 7.8, 1.0 Hz, 1H), 7.73 (td, *J* = 7.5, 1.2 Hz, 1H), 7.61 (dt, *J* = 7.8, 1.0 Hz, 1H), 7.51 (td, *J* = 7.5, 1.0 Hz, 1H), 7.34 – 7.27 (m, 3H), 7.09 (td, *J* = 7.5, 1.0 Hz, 1H), 7.02 – 6.96 (m, 3H), 6.90 (dd, *J* = 7.5, 1.3 Hz, 1H), 5.21 (d, *J* = 3.3 Hz, 1H), 5.10 – 5.02 (m, 2H), 4.68 (d, *J* = 3.3 Hz, 1H), 3.70 (d, *J* = 17.7 Hz, 1H), 3.58 (d, *J* = 17.7 Hz, 1H) ppm.

^{13}C NMR (126 MHz, $CDCl_3$) δ 201.32, 167.26, 157.97, 152.78, 150.26, 143.06, 135.91, 134.04, 132.95, 129.83, 129.08, 128.81, 126.51, 126.14, 125.45, 121.97, 121.88, 117.44, 114.89, 96.78, 67.92, 62.08, 42.80 ppm.

HRMS (m/z): [M + H^+] calcd for $C_{25}H_{20}O_3N$, 382.14377; found, 382.14365.

CLASS-E

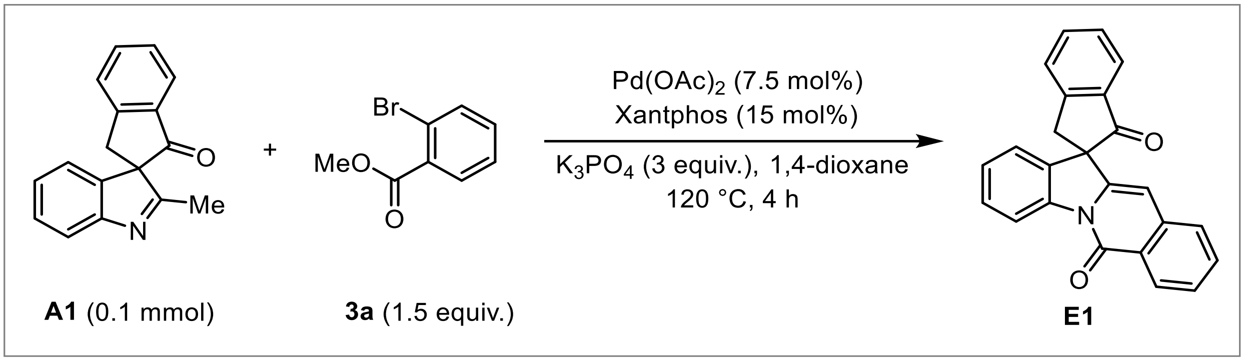

General procedure F: Synthesis of Class E

An oven-dried screw capped reaction tube with a magnetic stir-bar was charged with 2'-methylspiro[indene-2,3'-indol]-1(3H)-one (0.1 mmol), **3a** (1.5 equiv.), $Pd(OAc)_2$ (7.5 mol%, 1.7 mg), Xantphos (15 mol%, 8.7 mg), K_3PO_4 (3 equiv., 63.6 mg). A screw cap fitted with a rubber septum was attached to the reaction tube, then degassed and refilled with argon. 1,4-Dioxane (1 mL) was added in the reaction tube, placed in metal block, and vigorously stirred at room temperature for 10 min followed by heated to 120 °C. The reaction mixture was taken out after 4 h, diluted with 10 mL of ethyl acetate. The mixture was concentrated under vacuum. The crude mixture was purified by column chromatography using silica gel (100-200 mesh size) and petroleum ether/ethyl acetate as the eluent.

Characterization data for Class E products

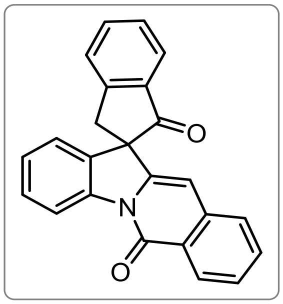

6'H-spiro[indene-2,12'-indolo[1,2-b]isoquinoline]-1,6'(3H)-dione [**E1**]: Compound **E1** was prepared according to the general procedure G (0.1 mmol scale).

Yield: 78% (27.3 mg isolated).

Eluent: Petroleum ether/Ethyl acetate (70/30, v/v).

TLC: R_f = 0.32 (70:30 petroleum ether:EtOAc).

Physical State: Off-white solid.

^{1}H NMR (600 MHz, Chloroform-*d*) δ 8.80 (dt, *J* = 8.1, 0.9 Hz, 1H), 8.53 (ddt, *J* = 8.1, 1.5, 0.7 Hz, 1H), 7.90 (dt, *J* = 7.6, 1.0 Hz, 1H), 7.79 (td, *J* = 7.5, 1.2 Hz, 1H), 7.67 (dt, *J* = 7.7, 1.0 Hz,

1H), 7.61 (ddd, J = 7.8, 7.1, 1.4 Hz, 1H), 7.56 (td, J = 7.5, 0.9 Hz, 1H), 7.52 – 7.48 (m, 1H), 7.48 – 7.44 (m, 1H), 7.41 (dt, J = 7.9, 0.8 Hz, 1H), 7.21 (td, J = 7.5, 1.1 Hz, 1H), 7.03 (ddd, J = 7.5, 1.3, 0.6 Hz, 1H), 6.36 (d, J = 0.7 Hz, 1H), 3.85 – 3.76 (m, 2H) ppm.

^{13}C NMR (151 MHz, $CDCl_3$) δ 201.08, 161.04, 152.82, 146.15, 142.78, 136.47, 136.19, 135.04, 133.69, 132.72, 129.57, 128.90, 128.14, 127.05, 126.85, 126.56, 126.36, 126.33, 126.21, 122.07, 118.20, 101.60, 62.02, 43.48 ppm.

HRMS (m/z): [M + H^+] calcd for $C_{24}H_{16}O_2N$, 350.11756; found, 350.11748.

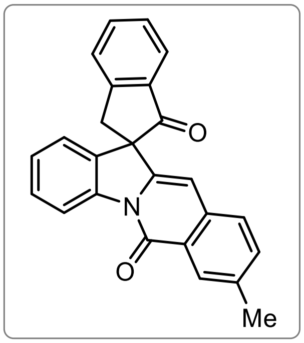

8'-methyl-6'H-spiro[indene-2,12'-indolo[1,2-b]isoquinoline]-1,6'(3H)-dione [**E2**]: Compound **E2** was prepared according to the general procedure G (0.1 mmol scale).

Yield: 83% (30.2 mg isolated).

Eluent: Petroleum ether/Ethyl acetate (70/30, v/v).

TLC: R_f = 0.35 (70:30 petroleum ether:EtOAc).

Physical State: Off-white solid.

^{1}H NMR (500 MHz, Chloroform-*d*) δ 8.80 (dt, J = 8.1, 0.8 Hz, 1H), 8.33 (dd, J = 1.8, 0.9 Hz, 1H), 7.89 (dt, J = 7.6, 1.0 Hz, 1H), 7.79 (td, J = 7.5, 1.2 Hz, 1H), 7.67 (dt, J = 7.8, 1.0 Hz, 1H), 7.55 (td, J = 7.5, 1.0 Hz, 1H), 7.50 – 7.41 (m, 2H), 7.31 (d, J = 8.0 Hz, 1H), 7.20 (td, J = 7.5, 1.1 Hz, 1H), 7.02 (dd, J = 7.5, 1.3 Hz, 1H), 6.33 (s, 1H), 3.85 – 3.73 (m, 2H), 2.50 (s, 3H) ppm.

^{13}C NMR (126 MHz, $CDCl_3$) δ 201.26, 161.05, 152.86, 145.12, 142.84, 137.17, 136.14, 135.05, 134.19, 134.09, 133.73, 129.52, 128.85, 127.76, 126.83, 126.45, 126.26, 126.23, 126.17, 122.06, 118.21, 101.55, 61.89, 43.53, 21.71 ppm.

HRMS (m/z): [M + H^+] calcd for $C_{25}H_{18}O_2N$, 364.13321; found, 364.13294.

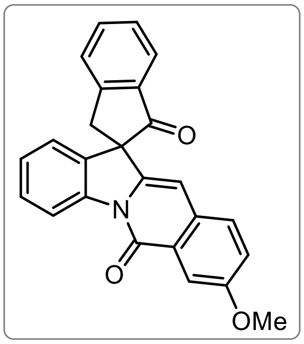

8'-methoxy-6'H-spiro[indene-2,12'-indolo[1,2-b]isoquinoline]-1,6'(3H)-dione [**E3**]: Compound **E3** was prepared according to the general procedure G (0.1 mmol scale).

Yield: 76% (28.8 mg isolated).

Eluent: Petroleum ether/Ethyl acetate (60/40, v/v).

TLC: R_f = 0.35 (60:40 petroleum ether:EtOAc).

Physical State: Off-white solid.

^{1}H NMR (600 MHz, Chloroform-*d*) δ 8.81 (dt, *J* = 8.1, 0.8 Hz, 1H), 7.94 (d, *J* = 2.7 Hz, 1H), 7.89 (dt, *J* = 7.7, 1.0 Hz, 1H), 7.78 (td, *J* = 7.5, 1.2 Hz, 1H), 7.66 (dt, *J* = 7.7, 1.0 Hz, 1H), 7.55 (td, *J* = 7.5, 1.0 Hz, 1H), 7.46 (ddd, *J* = 8.3, 7.5, 1.3 Hz, 1H), 7.34 (d, *J* = 8.6 Hz, 1H), 7.24 – 7.19 (m, 2H), 7.02 (ddd, *J* = 7.6, 1.2, 0.6 Hz, 1H), 6.33 (d, *J* = 0.6 Hz, 1H), 3.94 (s, 3H), 3.78 (q, *J* = 17.6 Hz, 2H) ppm.

^{13}C NMR (151 MHz, $CDCl_3$) δ 201.30, 160.71, 159.02, 152.83, 143.74, 142.82, 136.13, 135.05, 133.94, 130.43, 129.51, 128.84, 127.88, 127.75, 126.83, 126.33, 126.15, 123.16, 122.09, 118.22, 108.08, 101.45, 61.83, 55.87, 43.50 ppm.

HRMS (m/z): [M + H^+] calcd for $C_{25}H_{18}O_3N$, 380.12812; found, 380.12777.

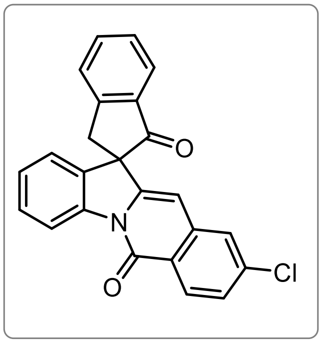

9'-chloro-6'H-spiro[indene-2,12'-indolo[1,2-b]isoquinoline]-1,6'(3H)-dione [**E4**]: Compound **E4** was prepared according to the general procedure G (0.1 mmol scale).

Yield: 59% (22.6 mg isolated).

Eluent: Petroleum ether/Ethyl acetate (70/30, v/v).

TLC: R_f = 0.35 (70:30 petroleum ether:EtOAc).

Physical State: Yellow solid.

^{1}H NMR (700 MHz, Chloroform-*d*) δ 8.76 (dt, *J* = 8.2, 0.8 Hz, 1H), 8.45 (d, *J* = 8.6 Hz, 1H), 7.90 (dt, *J* = 7.6, 1.0 Hz, 1H), 7.80 (td, *J* = 7.5, 1.2 Hz, 1H), 7.68 (dt, *J* = 7.6, 1.0 Hz, 1H), 7.57 (td, *J* = 7.5, 1.0 Hz, 1H), 7.47 (ddd, *J* = 8.4, 7.5, 1.2 Hz, 1H), 7.43 (dd, *J* = 8.6, 2.0 Hz, 1H), 7.40 (d, *J* = 2.0 Hz, 1H), 7.22 (td, *J* = 7.5, 1.1 Hz, 1H), 7.03 (ddd, *J* = 7.6, 1.3, 0.6 Hz, 1H), 6.27 (s, 1H), 3.89 – 3.70 (m, 2H) ppm.

^{13}C NMR (176 MHz, $CDCl_3$) δ 200.69, 160.37, 152.69, 147.73, 142.57, 139.17, 137.73, 136.30, 134.94, 133.54, 129.90, 129.65, 129.00, 127.50, 126.88, 126.54, 126.29, 125.65, 124.90, 122.12, 118.19, 100.53, 62.19, 43.32 ppm.

HRMS (m/z): [M + H^+] calcd for $C_{24}H_{15}O_2NCl$, 384.07858; found, 384.07842.

Compound **E4** was resynthesized (**E4-resynthesized**) and retested in biological assays. The ^{1}H NMR spectra of the resynthesized batch is analogous to the original batch.

^{1}H NMR (500 MHz, Chloroform-*d*) δ 8.77 (d, *J* = 8.1 Hz, 1H), 8.45 (d, *J* = 8.6 Hz, 1H), 7.90 (d, *J* = 7.7 Hz, 1H), 7.81 (td, *J* = 7.5, 1.2 Hz, 1H), 7.68 (dt, *J* = 7.7, 0.9 Hz, 1H), 7.57 (td, *J* = 7.5, 0.9 Hz, 1H), 7.47 (ddd, *J* = 8.4, 7.5, 1.3 Hz, 1H), 7.44 (dd, *J* = 8.6, 2.0 Hz, 1H), 7.40 (d, *J* = 2.0 Hz, 1H), 7.22 (td, *J* = 7.5, 1.1 Hz, 1H), 7.03 (dd, *J* = 7.5, 1.2 Hz, 1H), 6.28 (s, 1H), 3.97 – 3.63 (m, 2H).

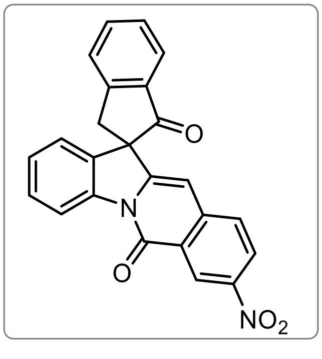

8'-nitro-6'H-spiro[indene-2,12'-indolo[1,2-b]isoquinoline]-1,6'(3H)-dione [**E5**]: Compound **E5** was prepared according to the general procedure G (0.1 mmol scale).

Yield: 43% (17 mg isolated).

Eluent: Petroleum ether/Ethyl acetate (60/40, v/v).

TLC: R_f = 0.32 (60:40 petroleum ether:EtOAc).

Physical State: Yellow solid.

^{1}H NMR (700 MHz, Chloroform-*d*) δ 9.39 (d, *J* = 2.4 Hz, 1H), 8.78 (dt, *J* = 8.2, 0.8 Hz, 1H), 8.40 (dd, *J* = 8.7, 2.4 Hz, 1H), 7.91 (dt, *J* = 7.6, 1.0 Hz, 1H), 7.83 (td, *J* = 7.6, 1.2 Hz, 1H), 7.70 (dt, *J* = 7.7, 1.0 Hz, 1H), 7.59 (td, *J* = 7.5, 1.0 Hz, 1H), 7.55 (d, *J* = 8.7 Hz, 1H), 7.51 (ddd, *J* = 8.4, 7.5, 1.3 Hz, 1H), 7.28 (dd, *J* = 7.5, 1.1 Hz, 1H), 7.08 – 7.04 (m, 1H), 6.45 (d, *J* = 0.6 Hz, 1H), 3.90 – 3.79 (m, 2H) ppm.

^{13}C NMR (176 MHz, $CDCl_3$) δ 200.17, 159.78, 152.59, 150.61, 146.30, 142.11, 141.07, 136.54, 134.79, 133.47, 129.89, 129.18, 127.60, 127.14, 126.94, 126.78, 126.56, 126.43, 124.69, 122.17, 118.39, 100.60, 77.41, 77.23, 77.05, 62.58, 43.05 ppm.

HRMS (m/z): [M + H^+] calcd for $C_{24}H_{15}O_4N_2$, 395.10263; found, 395.10235.

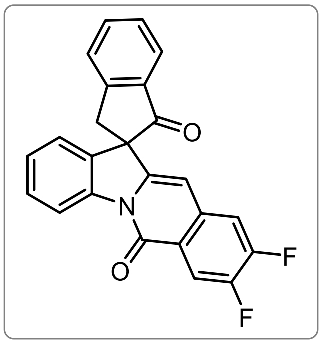

8',9'-difluoro-6'H-spiro[indene-2,12'-indolo[1,2-b]isoquinoline]-1,6'(3H)-dione [**E6**]: Compound **E6** was prepared according to the general procedure G (0.1 mmol scale).

Yield: 56% (21.6 mg isolated).

Eluent: Petroleum ether/Ethyl acetate (70/30, v/v).

TLC: R_f = 0.45 (70:30 petroleum ether:EtOAc).

Physical State: Yellow solid.

^{1}H NMR (600 MHz, Chloroform-*d*) δ 8.75 (dt, *J* = 8.1, 0.9 Hz, 1H), 8.29 (dd, *J* = 10.7, 8.1 Hz, 1H), 7.92 – 7.87 (m, 1H), 7.81 (td, *J* = 7.5, 1.3 Hz, 1H), 7.68 (dp, *J* = 7.7, 0.9 Hz, 1H), 7.59 – 7.55 (m, 1H), 7.47 (ddd, *J* = 8.2, 7.5, 1.3 Hz, 1H), 7.23 (td, *J* = 7.5, 1.1 Hz, 1H), 7.18 (dd, *J* = 10.3, 7.1 Hz, 1H), 7.03 (ddd, *J* = 7.6, 1.3, 0.6 Hz, 1H), 6.29 (s, 1H), 3.88 – 3.72 (m, 2H) ppm.

^{13}C NMR (151 MHz, $CDCl_3$) δ 200.72, 159.46, 159.44 [159.45 (d, *J* = 3.1 Hz)], 154.94, 154.85, 153.24, 153.15 [154.05 (dd, *J* = 256.3, 14.5 Hz)],152.67, 150.91, 150.82, 149.24, 149.15 [150.03 (dd, *J* = 251.2, 14.0 Hz)], 147.16, 147.14 [147.15 (d, *J* = 2.7 Hz)], 142.40, 136.37, 134.87, 134.17, 134.15, 134.11 [134.13 (dd, *J* = 8.8, 2.7 Hz)], 134.09, 133.53, 129.95, 129.69, 129.04, 126.88, 126.73, 126.30, 123.57, 123.56, 123.53, 123.52 [123.55 (dd, *J* = 6.1, 2.1 Hz)], 122.12, 118.21,

116.40, 116.38, 116.27, 116.26 [116.33 (dd, *J* = 18.8, 2.1 Hz)], 113.76, 113.64 [113.70 (d, *J* = 18.1 Hz)], 100.36, 100.35, 100.33 [100.35 (t, *J* = 2.2 Hz)], 62.11, 43.28 ppm.

^{19}F NMR (565 MHz, Chloroform-*d*) δ -129.39 (ddd, *J* = 21.4, 10.6, 8.3 Hz), -136.43 (ddd, *J* = 21.5, 10.6, 7.1 Hz) ppm.

HRMS (m/z): [M + H$^+$] calcd for $C_{24}H_{14}O_2NF_2$, 386.09871; found, 386.09846.

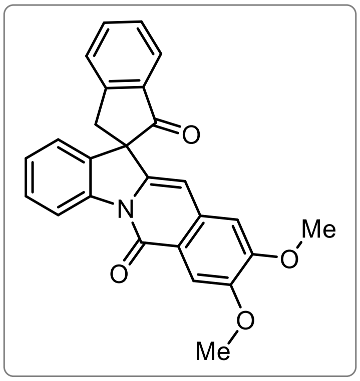

8',9'-dimethoxy-6'H-spiro[indene-2,12'-indolo[1,2-b]isoquinoline]-1,6'(3H)-dione [**E7**]: Compound **E7** was prepared according to the general procedure G (0.1 mmol scale).

Yield: 67% (27.4 mg isolated).

Eluent: Petroleum ether/Ethyl acetate (60/40, v/v).

TLC: R$_f$ = 0.28 (60:40 petroleum ether:EtOAc).

Physical State: Off-white solid.

^{1}H NMR (700 MHz, Chloroform-*d*) δ 8.79 (dt, *J* = 8.1, 0.8 Hz, 1H), 7.91 (s, 1H), 7.89 (dt, *J* = 7.7, 1.0 Hz, 1H), 7.79 (td, *J* = 7.5, 1.2 Hz, 1H), 7.66 (dt, *J* = 7.8, 1.0 Hz, 1H), 7.55 (td, *J* = 7.5, 1.0 Hz, 1H), 7.47 (ddd, *J* = 8.3, 7.5, 1.3 Hz, 1H), 7.20 (td, *J* = 7.5, 1.1 Hz, 1H), 7.03 (ddd, *J* = 7.6, 1.3, 0.6 Hz, 1H), 6.78 (s, 1H), 6.29 (s, 1H), 4.03 (s, 3H), 3.92 (s, 3H), 3.85 – 3.72 (m, 2H) ppm.

^{13}C NMR (176 MHz, $CDCl_3$) δ 201.23, 160.47, 153.75, 152.84, 149.42, 144.81, 143.04, 136.12, 135.15, 133.70, 131.92, 129.56, 128.85, 126.83, 126.18, 126.18, 122.11, 120.37, 118.14, 108.07, 106.61, 101.15, 61.95, 56.43, 56.26, 43.49 ppm.

HRMS (m/z): [M + H$^+$] calcd for $C_{26}H_{20}O_4N$, 410.13868; found, 410.13830.

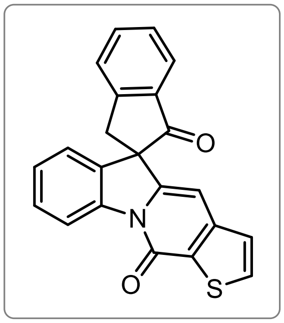

11'H-spiro[indene-2,5'-thieno[3',2':4,5]pyrido[1,2-a]indole]-1,11'(3H)-dione [**E8**]: Compound **E8** was prepared according to the general procedure G (0.1 mmol scale).

Yield: 72% (25.6 mg isolated).

Eluent: Petroleum ether/Ethyl acetate (70/30, v/v).

TLC: R_f = 0.48 (60:40 petroleum ether:EtOAc).

Physical State: Yellow solid.

^{1}H NMR (700 MHz, Chloroform-*d*) δ 8.80 (dt, *J* = 8.2, 0.8 Hz, 1H), 7.90 (dt, *J* = 7.7, 1.0 Hz, 1H), 7.79 (td, *J* = 7.5, 1.2 Hz, 1H), 7.70 (d, *J* = 5.1 Hz, 1H), 7.67 (dt, *J* = 7.7, 1.0 Hz, 1H), 7.56 (td, *J* = 7.5, 1.0 Hz, 1H), 7.47 (ddd, *J* = 8.3, 7.5, 1.3 Hz, 1H), 7.22 (td, *J* = 7.5, 1.1 Hz, 1H), 7.12 (d, *J* = 5.1 Hz, 1H), 7.03 (ddd, *J* = 7.5, 1.3, 0.6 Hz, 1H), 6.52 (s, 1H), 3.88 – 3.72 (m, 2H) ppm.

^{13}C NMR (176 MHz, $CDCl_3$) δ 200.89, 157.37, 152.72, 148.11, 144.58, 142.71, 136.24, 135.08, 134.18, 134.16, 130.65, 129.64, 128.93, 126.88, 126.52, 126.26, 124.44, 122.11, 118.42, 98.63, 62.05, 43.21 ppm.

HRMS (m/z): [M + H^+] calcd for $C_{22}H_{14}O_2NS$, 356.07398; found, 356.07406.

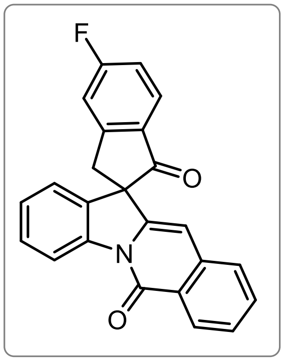

5-fluoro-6'H-spiro[indene-2,12'-indolo[1,2-b]isoquinoline]-1,6'(3H)-dione [**E9**]: Compound **E9** was prepared according to the general procedure G (0.1 mmol scale).

Yield: 77% (28.3 mg isolated).

Eluent: Petroleum ether/Ethyl acetate (70/30, v/v).

TLC: R_f = 0.3 (70:30 petroleum ether:EtOAc).

Physical State: Yellow solid.

^{1}H NMR (700 MHz, Chloroform-*d*) δ 8.80 (dt, *J* = 8.2, 0.8 Hz, 1H), 8.53 (ddt, *J* = 8.2, 1.4, 0.7 Hz, 1H), 7.91 (dd, *J* = 8.4, 5.2 Hz, 1H), 7.62 (ddd, *J* = 7.8, 7.1, 1.3 Hz, 1H), 7.51 (ddd, *J* = 8.2, 7.1, 1.2 Hz, 1H), 7.48 (ddd, *J* = 8.3, 7.5, 1.3 Hz, 1H), 7.44 – 7.41 (m, 1H), 7.37 – 7.33 (m, 1H), 7.27 (dd, *J* = 8.6, 2.3 Hz, 1H), 7.22 (td, *J* = 7.5, 1.1 Hz, 1H), 7.03 (ddd, *J* = 7.5, 1.3, 0.6 Hz, 1H), 6.36 (d, *J* = 0.6 Hz, 1H), 3.84 – 3.74 (m, 2H) ppm.

^{13}C NMR (176 MHz, $CDCl_3$) δ 199.19, 168.85, 167.38 [168.12 (d, *J* = 259.3 Hz)], 161.00, 155.76, 155.70 [155.73 (d, *J* = 10.2 Hz)], 145.86, 142.76, 136.38, 133.34, 132.80, 131.36, 131.35 [131.36 (d, *J* = 1.7 Hz)], 129.75, 128.58, 128.52 [128.55 (d, *J* = 10.6 Hz)], 128.19, 127.18, 126.61, 126.38, 122.01, 118.27, 117.44, 117.31 [117.37 (d, *J* = 23.8 Hz)], 113.78, 113.66 [113.72 (d, *J* = 22.7 Hz)], 101.66, 62.19, 43.24, 43.23 [43.24 (d, *J* = 2.0 Hz)] ppm.

^{19}F NMR (470 MHz, Chloroform-*d*) δ -99.99 (td, *J* = 8.6, 5.4 Hz) ppm.

HRMS (m/z): [M + H^+] calcd for $C_{24}H_{15}O_2NF$, 368.10813; found, 368.10797.

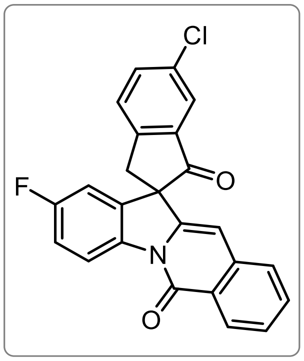

6-chloro-2'-fluoro-6'H-spiro[indene-2,12'-indolo[1,2-b]isoquinoline]-1,6'(3H)-dione [**E10**]: Compound **E10** was prepared according to the general procedure G (0.1 mmol scale).

Yield: 65% (26.1 mg isolated).

Eluent: Petroleum ether/Ethyl acetate (70/30, v/v).

TLC: R_f = 0.35 (70:30 petroleum ether:EtOAc).

Physical State: Yellow solid.

^{1}H NMR (700 MHz, Chloroform-*d*) δ 8.78 (dd, *J* = 8.9, 4.7 Hz, 1H), 8.52 (dd, *J* = 8.1, 1.3 Hz, 1H), 7.87 (d, *J* = 2.0 Hz, 1H), 7.77 (dd, *J* = 8.2, 2.1 Hz, 1H), 7.66 – 7.61 (m, 2H), 7.52 (ddd, *J* = 8.2, 7.1, 1.2 Hz, 1H), 7.43 (d, *J* = 7.9 Hz, 1H), 7.17 (td, *J* = 8.9, 2.6 Hz, 1H), 6.74 (dd, *J* = 7.6, 2.6 Hz, 1H), 6.36 (s, 1H), 3.80 – 3.72 (m, 2H) ppm.

^{13}C NMR (176 MHz, CDCl$_3$) δ 199.10, 161.77, 160.38 [161.07 (d, J = 246.3 Hz)], 160.76, 150.53, 145.49, 138.91, 138.90 [138.91 (d, J = 2.3 Hz)], 136.48, 136.31, 136.23, 135.66, 134.95, 134.90 [134.93 (d, J = 8.4 Hz)], 132.93, 128.15, 128.04, 127.41, 126.49, 126.48, 125.99, 119.49, 119.44 [119.47 (d, J = 8.3 Hz)], 116.44, 116.31 [116.38 (d, J = 22.9 H z)], 109.85, 109.71 [109.78 (d, J = 25.1 Hz)], 102.00, 62.35, 62.34 [62.35 (d, J = 2.2 Hz)], 42.77 ppm.

^{19}F NMR (470 MHz, Chloroform-*d*) δ -114.32 – -115.49 (m) ppm.

HRMS (m/z): [M + H$^+$] calcd for $C_{24}H_{14}O_2NClF$, 402.06916; found, 402.06910.

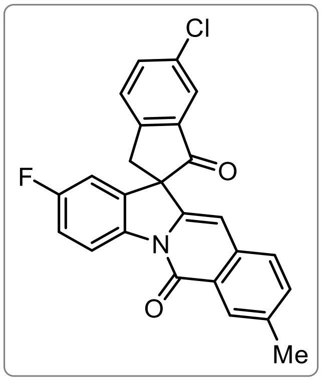

6-chloro-2'-fluoro-8'-methyl-6'H-spiro[indene-2,12'-indolo[1,2-b]isoquinoline]-1,6'(3H)-dione [**E11**]: Compound **E11** was prepared according to the general procedure G (0.1 mmol scale).

Yield: 68% (28.3 mg isolated).

Eluent: Petroleum ether/Ethyl acetate (75/25, v/v).

TLC: R_f = 48 (70:30 petroleum ether:EtOAc).

Physical State: Yellow solid.

^{1}H NMR (500 MHz, Chloroform-*d*) δ 8.78 (dd, J = 8.9, 4.8 Hz, 1H), 8.32 (dq, J = 1.7, 0.8 Hz, 1H), 7.86 (d, J = 2.0 Hz, 1H), 7.76 (dd, J = 8.2, 2.1 Hz, 1H), 7.62 (dd, J = 8.2, 0.8 Hz, 1H), 7.45 (ddd, J = 8.1, 1.9, 0.7 Hz, 1H), 7.33 (d, J = 8.1 Hz, 1H), 7.17 (td, J = 8.9, 2.6 Hz, 1H), 6.73 (dd, J = 7.7, 2.6 Hz, 1H), 6.32 (d, J = 0.6 Hz, 1H), 3.75 (d, J = 3.0 Hz, 2H), 2.51 (s, 3H) ppm.

^{13}C NMR (126 MHz, CDCl$_3$) δ 199.29, 161.94, 159.99 [160.97 (d, J = 246.1 Hz)], 160.70, 150.59, 144.46, 138.99, 138.98 [138.99 (d, J = 2.3 Hz)], 137.56, 136.41, 136.30, 135.56, 134.96, 134.90 [134.93 (d, J = 8.2 Hz)], 134.35, 133.82, 128.02, 127.74, 126.40, 126.35, 125.92, 119.43, 119.36 [119.40 (d, J = 8.3 Hz)], 116.36, 116.18 [116.27 (d, J = 22.9 Hz)], 109.85, 109.65 [109.75 (d, J = 25.0 Hz)], 101.86, 77.49, 77.23, 76.98, 62.23, 62.21 [62.22 (d, J = 2.1 Hz)], 42.79, 21.72 ppm.

^{19}F NMR (470 MHz, Chloroform-*d*) δ -115.11 (td, J = 8.2, 4.6 Hz) ppm.

HRMS (m/z): [M + H$^+$] calcd for $C_{25}H_{16}O_2NClF$, 416.08481; found, 416.08448.

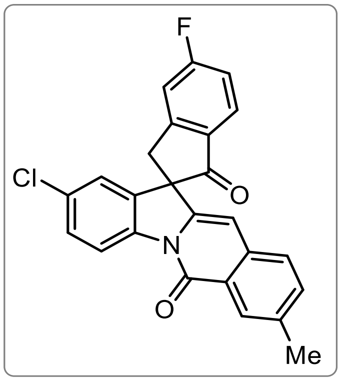

2'-chloro-5-fluoro-8'-methyl-6'H-spiro[indene-2,12'-indolo[1,2-b]isoquinoline]-1,6'(3H)-dione [**E12**]: Compound **E12** was prepared according to the general procedure G (0.1 mmol scale).

Yield: 59% (24.5 mg isolated).

Eluent: Petroleum ether/Ethyl acetate (75/25, v/v).

TLC: R_f = 0.5 (70:30 petroleum ether:EtOAc).

Physical State: Yellow amorphous solid.

^{1}H NMR (500 MHz, Chloroform-*d*) δ 8.71 (d, *J* = 8.7 Hz, 1H), 8.29 (dd, *J* = 1.8, 0.9 Hz, 1H), 7.90 (dd, *J* = 8.5, 5.2 Hz, 1H), 7.43 (ddd, *J* = 12.4, 8.4, 2.0 Hz, 2H), 7.34 (dd, *J* = 8.2, 2.2 Hz, 1H), 7.31 (d, *J* = 8.1 Hz, 1H), 7.27 (td, *J* = 8.6, 2.3 Hz, 1H), 6.98 (d, *J* = 2.1 Hz, 1H), 6.32 (s, 1H), 3.83 – 3.70 (m, 2H), 2.49 (s, 3H) ppm.

^{13}C NMR (126 MHz, $CDCl_3$) δ 198.63, <u>169.21, 167.14</u> [168.18 (d, *J* = 259.8 Hz)], 160.81, <u>155.56, 155.48</u> [155.52 (d, *J* = 10.2 Hz)], 144.38, 141.36, 137.56, 135.10, 134.42, 133.89, 131.50, <u>131.01, 131.00</u> [131.01 (d, *J* = 1.8 Hz)], 129.68, <u>128.70, 128.62</u> [128.66 (d, *J* = 10.6 Hz)], 127.76, 126.36, 126.31, 122.42, 119.12, <u>117.64, 117.45</u> [117.54 (d, *J* = 23.7 Hz)], <u>113.86, 113.68</u> [113.77 (d, *J* = 22.7 Hz)], 101.82, 61.78, <u>43.03, 43.01</u> [43.02 (d, *J* = 2.0 Hz)], 21.71 ppm.

135-DEPT NMR (126 MHz, $CDCl_3$) δ 134.25, 129.51, <u>128.53, 128.44</u> [128.49 (d, *J* = 10.7 Hz)], <u>127.59, 126.19</u> [117.37 (d, *J* = 23.9 Hz)], 122.25, 118.94, 117.46, 117.27, <u>113.69, 113.50</u> [113.60 (d, *J* = 22.8 Hz)], 101.65, 42.85, 42.84 [42.85 (d, *J* = 1.8 Hz)], 21.53 ppm.

^{19}F NMR (470 MHz, Chloroform-*d*) δ -99.49 (td, *J* = 8.5, 5.3 Hz) ppm.

HRMS (m/z): [M + H^+] calcd for $C_{25}H_{16}O_2NClF$, 416.08481; found, 416.08432.

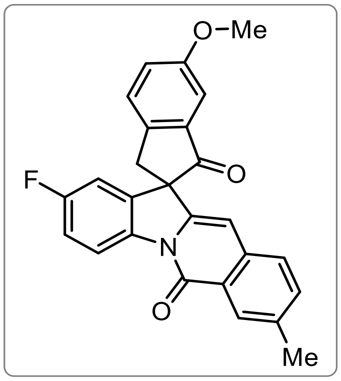

2'-fluoro-6-methoxy-8'-methyl-6'H-spiro[indene-2,12'-indolo[1,2-b]isoquinoline]-1,6'(3H)-dione [**E13**]: Compound **E13** was prepared according to the general procedure G (0.1 mmol scale).

Yield: 70% (28.8 mg isolated).

Eluent: Petroleum ether/Ethyl acetate (70/30, v/v).

TLC: R_f = 0.32 (70:30 petroleum ether:EtOAc).

Physical State: Amorphous brown solid.

^{1}H NMR (500 MHz, Chloroform-*d*) δ 8.77 (dd, *J* = 8.9, 4.7 Hz, 1H), 8.31 (dd, *J* = 1.8, 0.9 Hz, 1H), 7.55 (d, *J* = 8.3 Hz, 1H), 7.44 (dt, *J* = 8.0, 1.2 Hz, 1H), 7.38 (dd, *J* = 8.4, 2.6 Hz, 1H), 7.34 – 7.28 (m, 2H), 7.14 (td, *J* = 8.9, 2.6 Hz, 1H), 6.74 (dd, *J* = 7.7, 2.6 Hz, 1H), 6.33 (d, *J* = 0.6 Hz, 1H), 3.90 (s, 3H), 3.74 – 3.65 (m, 2H), 2.50 (s, 3H) ppm.

^{13}C NMR (176 MHz, $CDCl_3$) δ 200.49, 161.72, 160.32 [161.02 (d, *J* = 245.7 Hz)], 160.78, 160.65, 145.42, 145.03, 139.01, 138.99 [139.00 (d, *J* = 2.3 Hz)], 137.35, 136.06, 135.64, 135.59 [135.62 (d, *J* = 8.4 Hz)], 134.26, 134.03, 127.73, 127.53, 126.39, 126.33, 125.91, 119.34, 119.30 [119.32 (d, *J* = 8.3 Hz)], 116.09, 115.96 [116.02 (d, *J* = 22.9 Hz)], 109.82, 109.68 [109.75 (d, *J* = 24.9 Hz)], 107.07, 101.70, 62.61, 62.60 [62.60 (d, *J* = 2.1 Hz)], 56.02, 42.77, 21.71 ppm.

^{19}F NMR (470 MHz, Chloroform-*d*) δ -115.35 – -115.42 (m) ppm.

HRMS (m/z): [M + H^+] calcd for $C_{26}H_{19}O_3NF$, 412.13435; found, 412.13394.

Compound **E13** was resynthesized (**E13-resynthesized**) and retested in biological assays. The ^{1}H NMR spectra of the resynthesized batch is analogous to the original batch.

^{1}H NMR (400 MHz, Chloroform-*d*) δ 8.78 (dd, *J* = 9.0, 4.7 Hz, 1H), 8.32 (s, 1H), 7.55 (d, *J* = 8.3 Hz, 1H), 7.44 (d, *J* = 8.1 Hz, 1H), 7.39 (dd, *J* = 8.3, 2.4 Hz, 1H), 7.35 – 7.28 (m, 2H), 7.15 (dd, *J* = 10.0, 7.8 Hz, 1H), 6.74 (d, *J* = 6.7 Hz, 1H), 6.33 (s, 1H), 3.90 (s, 3H), 3.70 (s, 2H), 2.50 (s, 3H).

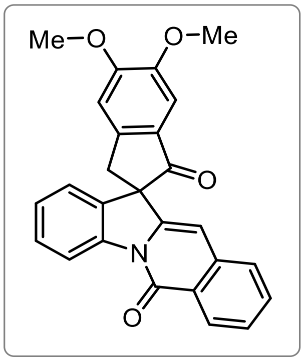

5,6-dimethoxy-6'H-spiro[indene-2,12'-indolo[1,2-b]isoquinoline]-1,6'(3H)-dione [**E14**]: Compound **E14** was prepared according to the general procedure G (0.1 mmol scale).

Yield: 72% (29.5 mg isolated).

Eluent: Petroleum ether/Ethyl acetate (60/40, v/v).

TLC: R_f = 0.31 (60:40 petroleum ether:EtOAc).

Physical State: Off-white solid.

^{1}H NMR (500 MHz, Chloroform-*d*) δ 8.79 (dt, *J* = 8.1, 0.8 Hz, 1H), 8.52 (dd, *J* = 8.1, 1.3 Hz, 1H), 7.61 (ddd, *J* = 8.2, 7.2, 1.4 Hz, 1H), 7.47 (dddd, *J* = 14.0, 8.3, 7.3, 1.2 Hz, 2H), 7.42 (dd, *J* = 7.8, 1.2 Hz, 1H), 7.28 (s, 1H), 7.21 (td, *J* = 7.5, 1.1 Hz, 1H), 7.09 – 7.04 (m, 2H), 6.37 (s, 1H), 4.07 (s, 3H), 3.96 (s, 3H), 3.76 – 3.63 (m, 2H) ppm.

^{13}C NMR (126 MHz, $CDCl_3$) δ 199.69, 161.04, 156.71, 150.57, 148.47, 146.52, 142.76, 136.54, 133.97, 132.68, 129.46, 128.12, 127.63, 126.95, 126.51, 126.32, 122.07, 118.15, 107.58, 106.00, 101.40, 62.29, 56.73, 56.49, 43.44 ppm.

HRMS (m/z): [M + H^+] calcd for $C_{26}H_{20}O_4N$, 410.13868; found, 410.13868.

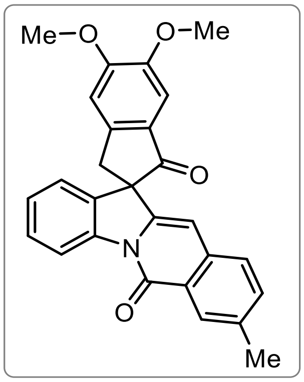

5,6-dimethoxy-8'-methyl-6'H-spiro[indene-2,12'-indolo[1,2-b]isoquinoline]-1,6'(3H)-dione [**E15**]: Compound **E15** was prepared according to the general procedure G (0.1 mmol scale).

Yield: 77% (32.6 mg isolated).

Eluent: Petroleum ether/Ethyl acetate (60/40, v/v).

TLC: R_f = 0.33 (60:40 petroleum ether:EtOAc).

Physical State: Brown amorphous solid.

^{1}H NMR (500 MHz, Chloroform-*d*) δ 8.79 (dt, *J* = 8.2, 0.8 Hz, 1H), 8.32 (dd, *J* = 1.8, 0.9 Hz, 1H), 7.49 – 7.40 (m, 2H), 7.31 (d, *J* = 8.0 Hz, 1H), 7.27 (s, 1H), 7.21 (td, *J* = 7.5, 1.1 Hz, 1H), 7.08 – 7.02 (m, 2H), 6.34 (d, *J* = 0.6 Hz, 1H), 4.07 (s, 3H), 3.96 (s, 3H), 3.77 – 3.58 (m, 2H), 2.50 (s, 3H) ppm.

^{13}C NMR (126 MHz, $CDCl_3$) δ 199.86, 161.05, 156.66, 150.53, 148.49, 145.52, 142.85, 137.06, 134.18, 134.15, 134.03, 129.41, 127.75, 127.67, 126.44, 126.22, 122.06, 118.17, 107.57, 105.98, 101.32, 62.19, 56.72, 56.48, 43.52, 21.70 ppm.

HRMS (m/z): [M + H^+] calcd for $C_{27}H_{22}O_4N$, 424.15433; found, 424.15400.

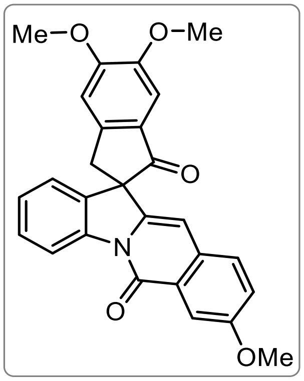

5,6,8'-trimethoxy-6'H-spiro[indene-2,12'-indolo[1,2-b]isoquinoline]-1,6'(3H)-dione [**E16**]: Compound **E16** was prepared according to the general procedure G (0.1 mmol scale).

Yield: 66% (29 mg).

Eluent: Petroleum ether/Ethyl acetate (55/35, v/v).

TLC: R_f = 0.35 (50:50 petroleum ether:EtOAc).

Physical State: Off-white solid.

^{1}H NMR (500 MHz, Chloroform-*d*) δ 8.79 (dt, *J* = 8.1, 0.7 Hz, 1H), 7.91 (d, *J* = 2.7 Hz, 1H), 7.48 – 7.41 (m, 1H), 7.32 (d, *J* = 8.6 Hz, 1H), 7.25 (s, 1H), 7.22 – 7.17 (m, 2H), 7.07 – 7.01 (m, 2H), 6.33 (s, 1H), 4.04 (s, 3H), 3.94 (s, 3H), 3.92 (s, 3H), 3.71 – 3.59 (m, 2H) ppm.

^{13}C NMR (126 MHz, $CDCl_3$) δ 199.88, 160.66, 158.87, 156.62, 150.47, 148.47, 144.06, 142.76, 134.20, 130.46, 129.34, 127.80, 127.63, 127.55, 126.28, 123.07, 122.07, 118.11, 107.97, 107.55, 105.89, 101.21, 62.05, 56.67, 56.42, 55.81, 43.39 ppm.

HRMS (m/z): [M + H^+] calcd for $C_{27}H_{22}O_5N$, 440.14925; found, 440.14863.

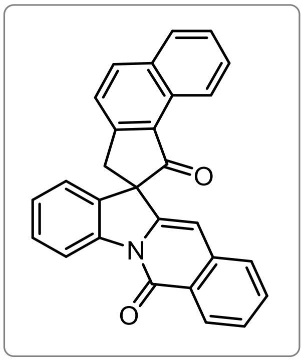

6'H-spiro[cyclopenta[a]naphthalene-2,12'-indolo[1,2-b]isoquinoline]-1,6'(3H)-dione [**E17**]: Compound **E17** was prepared according to the general procedure G (0.1 mmol scale).

Yield: 60% (24 mg isolated).

Eluent: Petroleum ether/Ethyl acetate (75/25, v/v).

TLC: R_f = 0.35 (70:30 petroleum ether:EtOAc).

Physical State: Yellow semi-solid.

^{1}H NMR (600 MHz, Chloroform-*d*) δ 9.03 (dd, *J* = 8.4, 1.2 Hz, 1H), 8.83 (dt, *J* = 8.1, 0.8 Hz, 1H), 8.57 – 8.51 (m, 1H), 8.24 (d, *J* = 8.4 Hz, 1H), 8.00 (dt, *J* = 8.3, 1.0 Hz, 1H), 7.73 – 7.67 (m, 2H), 7.64 (ddd, *J* = 8.2, 6.9, 1.3 Hz, 1H), 7.58 (ddd, *J* = 8.1, 7.1, 1.4 Hz, 1H), 7.47 (dtd, *J* = 8.1, 7.1, 1.2 Hz, 2H), 7.40 – 7.35 (m, 1H), 7.20 (td, *J* = 7.5, 1.1 Hz, 1H), 7.06 (dd, *J* = 7.6, 1.2 Hz, 1H), 6.40 (s, 1H), 3.95 – 3.84 (m, 2H) ppm.

^{13}C NMR (151 MHz, $CDCl_3$) δ 201.32, 161.06, 156.55, 146.30, 142.89, 137.47, 136.50, 133.84, 133.45, 132.66, 130.24, 129.90, 129.49, 129.10, 128.65, 128.08, 127.57, 126.95, 126.52, 126.34, 126.32, 124.31, 123.75, 122.08, 118.18, 101.48, 62.35, 43.79 ppm.

HRMS (m/z): [M + H^+] calcd for $C_{28}H_{18}O_2N$, 400.13321; found, 400.13287.

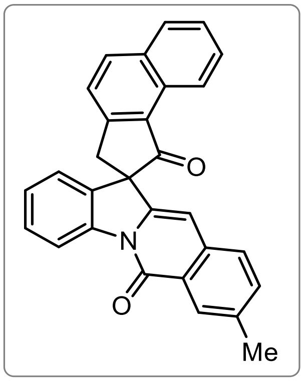

8'-methyl-6'H-spiro[cyclopenta[a]naphthalene-2,12'-indolo[1,2-b]isoquinoline]-1,6'(3H)-dione [**E18**]: Compound **E18** was prepared according to the general procedure G (0.1 mmol scale).

Yield: 65% (26.9 mg isolated).

Eluent: Petroleum ether/Ethyl acetate (80/20, v/v).

TLC: R_f = 0.4 (70:30 petroleum ether:EtOAc).

Physical State: Brown solid.

^{1}H NMR (500 MHz, Chloroform-*d*) δ 9.03 (dd, *J* = 8.4, 1.2 Hz, 1H), 8.83 (dt, *J* = 8.2, 0.8 Hz, 1H), 8.34 (dd, *J* = 1.8, 0.9 Hz, 1H), 8.24 (d, *J* = 8.4 Hz, 1H), 8.00 (d, *J* = 8.0 Hz, 1H), 7.72 – 7.68 (m, 2H), 7.64 (ddd, *J* = 8.3, 7.0, 1.3 Hz, 1H), 7.47 (ddd, *J* = 8.4, 7.5, 1.3 Hz, 1H), 7.40 (dd, *J* = 8.2, 1.9 Hz, 1H), 7.27 (d, *J* = 8.1 Hz, 1H), 7.19 (td, *J* = 7.5, 1.1 Hz, 1H), 7.07 – 7.04 (m, 1H), 6.36 (d, *J* = 0.6 Hz, 1H), 3.95 – 3.81 (m, 2H), 2.49 (s, 3H) ppm.

^{13}C NMR (126 MHz, $CDCl_3$) δ 201.36, 160.92, 156.43, 145.12, 142.82, 137.26, 136.92, 133.98, 133.74, 133.27, 130.07, 129.72, 129.30, 128.95, 128.49, 127.56, 127.39, 126.28, 126.09, 126.07, 124.15, 123.61, 121.91, 118.04, 101.26, 62.08, 43.71, 21.53 ppm.

HRMS (m/z): [M + H^+] calcd for $C_{29}H_{20}O_2N$, 414.14886; found, 414.14870.

Compound **E18** was resynthesized (**E18-resynthesized**) and retested in biological assays. The ^{1}H NMR spectra of the resynthesized batch is analogous to the original batch.

^{1}H NMR (500 MHz, Chloroform-*d*) δ 9.03 (dd, *J* = 8.4, 1.2 Hz, 1H), 8.83 (dt, *J* = 8.2, 0.7 Hz, 1H), 8.34 (dd, *J* = 1.9, 0.9 Hz, 1H), 8.25 (d, *J* = 8.4 Hz, 1H), 8.01 (dt, *J* = 8.2, 0.9 Hz, 1H), 7.74 – 7.69 (m, 2H), 7.65 (ddd, *J* = 8.2, 6.9, 1.3 Hz, 1H), 7.48 (ddd, *J* = 8.4, 7.5, 1.3 Hz, 1H), 7.42 (dd, *J* = 8.2, 1.9 Hz, 1H), 7.29 (d, *J* = 8.0 Hz, 1H), 7.20 (td, *J* = 7.5, 1.1 Hz, 1H), 7.06 (dd, *J* = 7.6, 1.2 Hz, 1H), 6.37 (s, 1H), 3.97 – 3.81 (m, 2H), 2.50 (s, 3H).

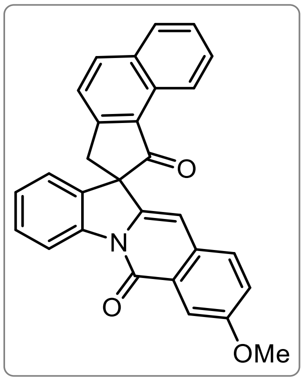

8'-methoxy-6'H-spiro[cyclopenta[a]naphthalene-2,12'-indolo[1,2-b]isoquinoline]-1,6'(3H)-dione [**E19**]: Compound **E19** was prepared according to the general procedure G (0.1 mmol scale).

Yield: 63% (27 mg isolated).

Eluent: Petroleum ether/Ethyl acetate (70/30, v/v).

TLC: R_f = 0.38 (60:40 petroleum ether:EtOAc).

Physical State: Yellow solid.

^{1}H NMR (500 MHz, Chloroform-*d*) δ 9.06 – 9.00 (m, 1H), 8.84 (dt, *J* = 8.2, 0.8 Hz, 1H), 8.23 (dd, *J* = 8.5, 0.7 Hz, 1H), 7.99 (dt, *J* = 8.1, 1.0 Hz, 1H), 7.94 (d, *J* = 2.8 Hz, 1H), 7.73 – 7.67 (m, 2H), 7.63 (ddd, *J* = 8.2, 7.0, 1.3 Hz, 1H), 7.47 (ddd, *J* = 8.5, 7.5, 1.3 Hz, 1H), 7.30 (d, *J* = 8.7 Hz, 1H), 7.23 – 7.17 (m, 2H), 7.06 (dd, *J* = 7.4, 1.2 Hz, 1H), 6.37 (d, *J* = 0.6 Hz, 1H), 3.94 (s, 3H), 3.93 – 3.81 (m, 2H) ppm.

^{13}C NMR (126 MHz, $CDCl_3$) δ 201.44, 160.59, 158.78, 156.42, 143.72, 142.79, 137.26, 133.93, 133.26, 130.31, 130.06, 129.72, 129.31, 128.94, 128.48, 127.71, 127.55, 127.39, 126.18, 124.14, 123.60, 122.96, 121.96, 118.07, 107.85, 101.19, 62.00, 55.69, 43.69 ppm.

HRMS (m/z): [M + H^+] calcd for $C_{29}H_{20}O_3N$, 430.14377; found, 430.14333.

Intermediates of CLASS-F & G

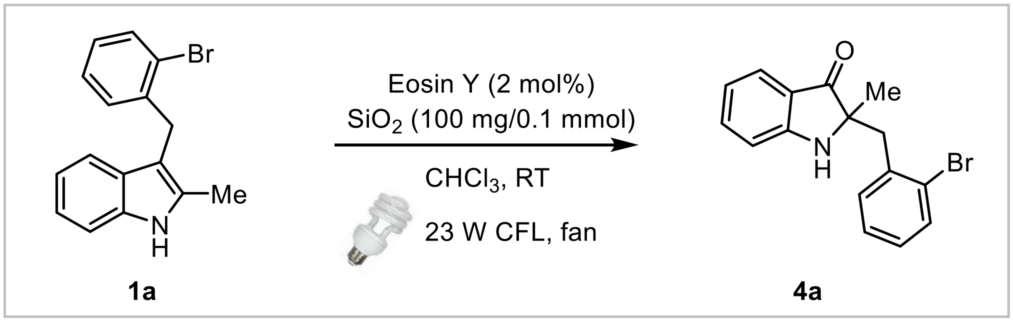

General procedure G: Synthesis of 2,2'-disubstituted-3-oxindole derivatives

An oven-dried screw capped reaction tube with a magnetic stir-bar was charged with 3-(2-bromobenzyl)-2-methyl-1H-indole (0.25 mmol), Eosin Y (2 mol%, 3.5 mg), SiO_2 (250 mg). 4 mL of Chloroform was added to the reaction tube and a screw cap fitted with a rubber septum was attached to the reaction tube. The reaction tube was placed close to light source from 23 W CFL. To keep the reaction mixture cool, a fan was set nearby. The reaction was monitored by TLC checking. The reaction mixture was taken out after 36 h, then, solid particles were filtered off by a filter paper and washed with dichloromethane. The solvent was evaporated under vacuum. The crude mixture was purified by column chromatography using silica gel (100-200 mesh size) and petroleum ether/ethyl acetate as the eluent.

Characterization data for Intermediates of Class F & G

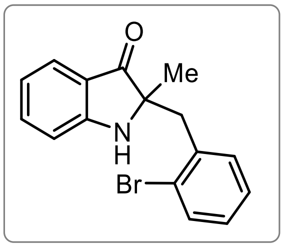

2-(2-bromobenzyl)-2-methylindolin-3-one [**4a**]: Compound **4a** was prepared according to the general procedure G (four set of 0.25 mmol scale).

Yield: 63% (overall isolated yield) [*The same is followed for the rest of the entries*].

Eluent: Petroleum ether/Ethyl acetate (97/3, v/v).

TLC: R_f = 0.58 (90:10 petroleum ether:EtOAc).

Physical State: Yellow liquid.

^{1}H NMR (500 MHz, Chloroform-*d*) δ 7.56 – 7.52 (m, 1H), 7.48 (dd, *J* = 8.0, 1.3 Hz, 1H), 7.33 (ddd, *J* = 9.1, 6.8, 1.4 Hz, 1H), 7.19 (dd, *J* = 7.7, 1.8 Hz, 1H), 7.10 (td, *J* = 7.5, 1.4 Hz, 1H), 6.99 (td, *J* = 7.7, 1.8 Hz, 1H), 6.71 (dd, *J* = 7.9, 6.8 Hz, 2H), 3.28 (d, *J* = 13.7 Hz, 1H), 3.11 (d, *J* = 13.7 Hz, 1H), 1.38 (s, 3H) ppm.

^{13}C NMR (126 MHz, CDCl$_3$) δ 204.44, 160.04, 137.43, 136.12, 132.96, 132.22, 128.67, 127.45, 125.75, 124.86, 120.05, 118.73, 112.21, 68.10, 42.45, 23.35 ppm.

HRMS (m/z): [M + H$^+$] calcd for $C_{16}H_{15}ONBr$, 316.03315; found, 316.03282.

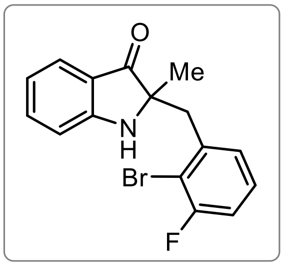

2-(2-bromo-3-fluorobenzyl)-2-methylindolin-3-one [**4b**]: Compound **4b** was prepared according to the general procedure G (four set of 0.25 mmol scale).

Yield: 56%.

Eluent: Petroleum ether/Ethyl acetate (97/3, v/v).

TLC: R_f = 0.6 (90:10 petroleum ether:EtOAc).

Physical State: Yellow liquid.

^{1}H NMR (600 MHz, Chloroform-*d*) δ 7.53 (ddd, *J* = 7.9, 1.4, 0.6 Hz, 1H), 7.34 (ddd, *J* = 8.3, 7.1, 1.4 Hz, 1H), 7.07 (td, *J* = 7.9, 5.5 Hz, 1H), 6.99 (dt, *J* = 7.8, 1.3 Hz, 1H), 6.90 (td, *J* = 8.3, 1.6 Hz, 1H), 6.74 – 6.69 (m, 2H), 4.81 (s, 1H), 3.32 (d, *J* = 13.7 Hz, 1H), 3.16 (d, *J* = 13.7 Hz, 1H), 1.38 (s, 3H) ppm.

^{13}C NMR (151 MHz, CDCl$_3$) δ 204.10, 159.98, 159.94, 158.31 [159.12 (d, *J* = 246.1 Hz)], 138.76, 137.54, 128.20, 128.15 [128.17 (d, *J* = 8.2 Hz)], 127.25, 127.23 [127.24 (d, *J* = 3.1 Hz)], 124.90, 119.96, 118.87, 115.00, 114.85 [114.92 (d, *J* = 23.0 Hz)], 112.94, 112.81 [112.87 (d, *J* = 20.2 Hz)], 112.26, 67.97, 41.99, 41.97 [41.98 (d, *J* = 2.3 Hz)], 23.51 ppm.

^{19}F NMR (565 MHz, Chloroform-*d*) δ -103.16 (dd, *J* = 8.4, 5.6 Hz) ppm.

HRMS (m/z): [M + H$^+$] calcd for $C_{16}H_{14}ONBrF$, 334.02373; found, 334.02419.

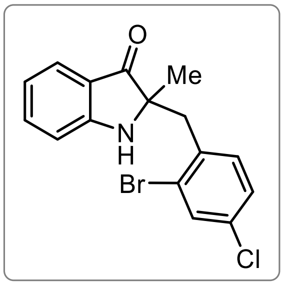

2-(2-bromo-4-chlorobenzyl)-2-methylindolin-3-one [**4c**]: Compound **4c** was prepared according to the general procedure G (four set of 0.25 mmol scale).

Yield: 65%.

Eluent: Petroleum ether/Ethyl acetate (97/3, v/v).

TLC: R_f = 0.6 (90:10 petroleum ether:EtOAc).

Physical State: Yellow liquid.

^{1}H NMR (500 MHz, Chloroform-*d*) δ 7.56 – 7.51 (m, 1H), 7.49 (d, *J* = 2.1 Hz, 1H), 7.36 (ddd, *J* = 8.3, 7.1, 1.4 Hz, 1H), 7.15 – 7.06 (m, 2H), 6.77 – 6.70 (m, 2H), 3.25 (d, *J* = 13.8 Hz, 1H), 3.10 (d, *J* = 13.8 Hz, 1H), 1.38 (s, 3H) ppm.

^{13}C NMR (126 MHz, $CDCl_3$) δ 204.12, 159.99, 137.62, 134.84, 133.44, 132.73, 132.46, 127.70, 126.01, 124.91, 119.98, 118.98, 112.29, 67.99, 41.87, 23.63 ppm.

HRMS (m/z): [M + H^+] calcd for $C_{16}H_{14}ONBrCl$, 349.99418; found, 349.99392.

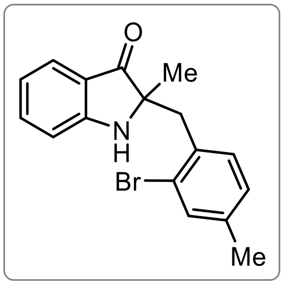

2-(2-bromo-4-methylbenzyl)-2-methylindolin-3-one [**4d**]: Compound **4d** was prepared according to the general procedure G (four set of 0.25 mmol scale).

Yield: 70%.

Eluent: Petroleum ether/Ethyl acetate (97/3, v/v).

TLC: R_f = 0.63 (90:10 petroleum ether:EtOAc).

Physical State: Yellow liquid.

^{1}H NMR (500 MHz, Chloroform-*d*) δ 7.57 – 7.52 (m, 1H), 7.38 – 7.32 (m, 1H), 7.31 (s, 1H), 7.06 (d, *J* = 7.7 Hz, 1H), 6.94 – 6.89 (m, 2H), 6.72 (dd, *J* = 7.8, 6.7 Hz, 2H), 3.22 (d, *J* = 13.8 Hz, 1H), 3.06 (d, *J* = 13.7 Hz, 1H), 2.22 (s, 3H), 1.36 (s, 3H) ppm.

^{13}C NMR (126 MHz, $CDCl_3$) δ 204.60, 160.08, 138.76, 137.41, 133.40, 132.93, 131.92, 128.36, 125.47, 124.91, 120.09, 118.71, 112.29, 68.15, 41.94, 23.32, 20.77 ppm.

HRMS (m/z): [M + H^+] calcd for $C_{17}H_{17}ONBr$, 330.0488; found, 330.0485.

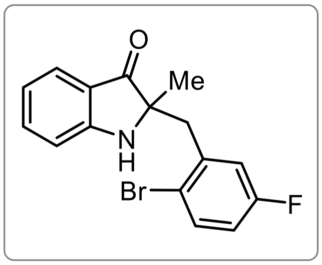

2-(2-bromo-5-fluorobenzyl)-2-methylindolin-3-one [**4e**]: Compound **4e** was prepared according to the general procedure G (four set of 0.25 mmol scale).

Yield: 63%.

Eluent: Petroleum ether/Ethyl acetate (97/3, v/v).

TLC: R_f = 0.6 (90:10 petroleum ether:EtOAc).

Physical State: Yellow liquid.

^{1}H NMR (500 MHz, Chloroform-*d*) δ 7.56 (ddd, *J* = 7.9, 1.4, 0.7 Hz, 1H), 7.43 (dd, *J* = 8.8, 5.4 Hz, 1H), 7.36 (ddd, *J* = 8.4, 7.0, 1.4 Hz, 1H), 6.95 (dd, *J* = 9.3, 3.1 Hz, 1H), 6.77 – 6.71 (m, 3H), 4.73 (s, 1H), 3.27 (d, *J* = 13.8 Hz, 1H), 3.06 (d, *J* = 13.7 Hz, 1H), 1.38 (s, 3H) ppm.

^{13}C NMR (126 MHz, $CDCl_3$) δ 204.03, 162.61, 160.64 [161.62 (d, *J* = 247.4 Hz)], 159.99, 138.41, 138.35 [138.38 (d, *J* = 7.5 Hz)], 137.59, 134.03, 133.97 [134.00 (d, *J* = 8.1 Hz)], 125.00, 120.00, 119.98, 119.95 [119.96 (d, *J* = 3.2 Hz)], 119.02, 118.98, 118.79 [118.89 (d, *J* = 22.7 Hz)], 116.01, 115.84 [115.93 (d, *J* = 22.4 Hz)], 112.30, 67.88, 42.55, 42.53 [42.54 (d, *J* = 1.7 Hz)], 23.43 ppm.

^{19}F NMR (470 MHz, Chloroform-*d*) δ -110.08 – -116.68 (m) ppm.

HRMS (m/z): [M + H^+] calcd for $C_{16}H_{14}ONBrF$, 334.02373; found, 334.02346.

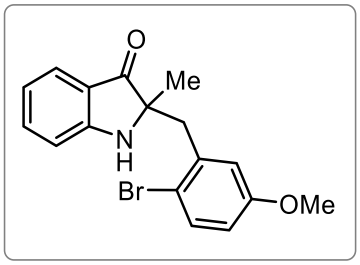

2-(2-bromo-5-methoxybenzyl)-2-methylindolin-3-one [**4f**]: Compound **4f** was prepared according to the general procedure G (four set of 0.25 mmol scale).

Yield: 52%.

Eluent: Petroleum ether/Ethyl acetate (90/10, v/v).

TLC: R_f = 0.45 (80:20 petroleum ether:EtOAc).

Physical State: Yellow liquid.

^{1}H NMR (700 MHz, Chloroform-*d*) δ 7.55 (ddd, *J* = 7.7, 1.4, 0.8 Hz, 1H), 7.37 – 7.32 (m, 2H), 6.75 – 6.70 (m, 3H), 6.58 (dd, *J* = 8.8, 3.1 Hz, 1H), 3.67 (s, 3H), 3.25 (d, *J* = 13.8 Hz, 1H), 3.08 (d, *J* = 13.8 Hz, 1H), 1.39 (s, 3H) ppm.

^{13}C NMR (176 MHz, CDCl$_3$) δ 204.49, 160.13, 158.78, 137.46, 137.11, 133.45, 124.83, 120.31, 118.86, 117.10, 116.19, 115.13, 112.31, 68.09, 55.65, 42.82, 23.49 ppm.

HRMS (m/z): [M + H$^+$] calcd for $C_{17}H_{17}O_2NBr$, 346.0437; found, 346.0434.

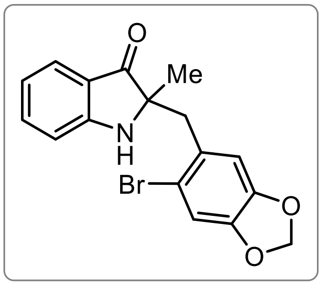

2-((6-bromobenzo[d][1,3]dioxol-5-yl)methyl)-2-methylindolin-3-one [**4g**]: Compound **4g** was prepared according to the general procedure G (four set of 0.25 mmol scale).

Yield: 59%.

Eluent: Petroleum ether/Ethyl acetate (85/15, v/v).

TLC: R$_f$ = 0.45 (80:20 petroleum ether:EtOAc).

Physical State: Yellow solid.

^{1}H NMR (500 MHz, Chloroform-*d*) δ 7.60 – 7.53 (m, 1H), 7.37 (ddd, *J* = 9.0, 6.8, 1.4 Hz, 1H), 6.93 (s, 1H), 6.75 (dd, *J* = 8.2, 7.1 Hz, 2H), 6.69 (s, 1H), 5.89 (dd, *J* = 12.5, 1.4 Hz, 2H), 3.19 (d, *J* = 13.9 Hz, 1H), 2.99 (d, *J* = 13.9 Hz, 1H), 1.36 (s, 3H) ppm.

^{13}C NMR (126 MHz, CDCl$_3$) δ 204.45, 160.11, 147.49, 147.36, 137.49, 129.14, 125.01, 120.14, 118.84, 116.00, 112.76, 112.35, 111.45, 101.89, 68.19, 42.37, 23.40 ppm.

HRMS (m/z): [M + H$^+$] calcd for $C_{17}H_{15}O_3NBr$, 360.02298; found, 360.02265.

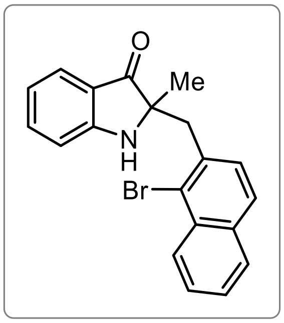

2-((1-bromonaphthalen-2-yl)methyl)-2-methylindolin-3-one [**4h**]: Compound **4h** was prepared according to the general procedure G (four set of 0.25 mmol scale).

Yield: 62%.

Eluent: Petroleum ether/Ethyl acetate (97/3, v/v).

TLC: R_f = 0.6 (90:10 petroleum ether:EtOAc).

Physical State: Yellow liquid.

^{1}H NMR (500 MHz, Chloroform-*d*) δ 8.31 (dq, *J* = 8.6, 0.9 Hz, 1H), 7.74 (dt, *J* = 8.2, 0.9 Hz, 1H), 7.62 (d, *J* = 8.4 Hz, 1H), 7.60 – 7.55 (m, 2H), 7.48 (ddd, *J* = 8.0, 6.8, 1.2 Hz, 1H), 7.35 – 7.28 (m, 2H), 6.74 – 6.65 (m, 2H), 4.88 (s, 1H), 3.57 (d, *J* = 13.7 Hz, 1H), 3.34 (d, *J* = 13.7 Hz, 1H), 1.43 (s, 3H) ppm.

^{13}C NMR (126 MHz, $CDCl_3$) δ 204.48, 160.07, 137.49, 134.67, 133.71, 132.58, 129.18, 128.25, 127.87, 127.66, 127.59, 126.50, 125.73, 124.96, 119.99, 118.84, 112.37, 68.30, 43.67, 23.65 ppm.

HRMS (m/z): [M + H^+] calcd for $C_{20}H_{17}ONBr$, 366.04880; found, 366.04932.

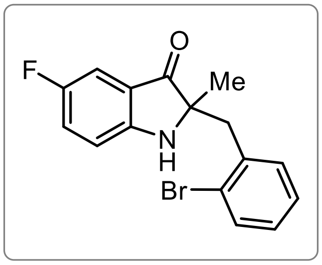

2-(2-bromobenzyl)-5-fluoro-2-methylindolin-3-one [**4i**]: Compound **4i** was prepared according to the general procedure G (four set of 0.25 mmol scale).

Yield: 74%.

Eluent: Petroleum ether/Ethyl acetate (97/3, v/v).

TLC: R_f = 0.6 (90:10 petroleum ether:EtOAc).

Physical State: Yellow liquid.

^{1}H NMR (500 MHz, Chloroform-*d*) δ 7.47 (dd, *J* = 8.0, 1.3 Hz, 1H), 7.17 (td, *J* = 7.4, 2.2 Hz, 2H), 7.13 – 7.07 (m, 2H), 7.00 (ddd, *J* = 7.9, 7.2, 1.8 Hz, 1H), 6.68 (dd, *J* = 8.9, 3.8 Hz, 1H), 4.76 (s, 1H), 3.28 (d, *J* = 13.7 Hz, 1H), 3.12 (d, *J* = 13.7 Hz, 1H), 1.39 (s, 3H) ppm.

^{13}C NMR (126 MHz, $CDCl_3$) δ <u>204.28, 204.25</u> [204.26 (d, *J* = 3.3 Hz)], 156.82, <u>157.33,155.43</u> [156.38 (d, *J* = 239.0 Hz)], 135.89, 133.00, 132.18, 128.80, 127.55, <u>125.68, 125.63</u> [125.65 (d, *J* = 5.8 Hz)], 125.43, <u>120.47, 120.41</u> [120.44 (d, *J* = 7.2 Hz)], <u>113.34, 113.28</u> [113.31 (d, *J* = 7.6 Hz)], <u>109.55, 109.37</u> [109.46 (d, *J* = 22.4 Hz)], 69.38, 42.60, 23.46 ppm.

^{19}F NMR (470 MHz, Chloroform-*d*) δ -125.31 (td, *J* = 8.1, 3.8 Hz) ppm.

HRMS (m/z): [M + H$^+$] calcd for $C_{16}H_{14}ONBrF$, 334.02373; found, 334.02421.

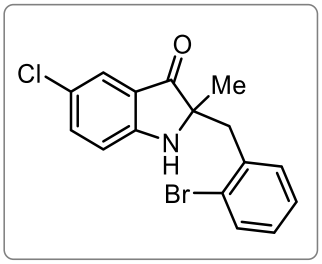

2-(2-bromobenzyl)-5-chloro-2-methylindolin-3-one [**4j**]: Compound **4j** was prepared according to the general procedure G (four set of 0.25 mmol scale).

Yield: 70%.

Eluent: Petroleum ether/Ethyl acetate (97/3, v/v).

TLC: R_f = 0.6 (90:10 petroleum ether:EtOAc).

Physical State: Yellow liquid.

^{1}H NMR (500 MHz, Chloroform-*d*) δ 7.43 – 7.37 (m, 2H), 7.18 (dd, *J* = 8.7, 2.3 Hz, 1H), 7.10 – 7.01 (m, 2H), 6.93 (ddd, *J* = 7.9, 7.1, 1.9 Hz, 1H), 6.59 (dd, *J* = 8.6, 0.6 Hz, 1H), 4.78 (s, 1H), 3.20 (d, *J* = 13.8 Hz, 1H), 3.05 (d, *J* = 13.8 Hz, 1H), 1.31 (s, 3H) ppm.

^{13}C NMR (126 MHz, $CDCl_3$) δ 203.29, 158.29, 137.34, 135.76, 133.00, 132.13, 128.84, 127.61, 125.68, 124.07, 123.87, 120.95, 113.32, 69.04, 42.50, 23.42 ppm.

HRMS (m/z): [M + H$^+$] calcd for $C_{16}H_{14}ONBrCl$, 349.99418; found, 349.99476.

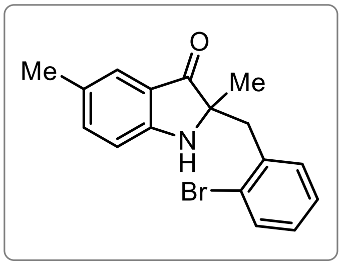

2-(2-bromobenzyl)-2,5-dimethylindolin-3-one [**4k**]: Compound **4k** was prepared according to the general procedure G (four set of 0.25 mmol scale).

Yield: 58%.

Eluent: Petroleum ether/Ethyl acetate (97/3, v/v).

TLC: R_f = 0.63 (90:10 petroleum ether:EtOAc).

Physical State: Yellow liquid.

^{1}H NMR (500 MHz, Chloroform-*d*) δ 7.47 (dd, *J* = 8.0, 1.3 Hz, 1H), 7.34 (dt, *J* = 1.9, 0.8 Hz, 1H), 7.18 (td, *J* = 7.8, 1.8 Hz, 2H), 7.10 (td, *J* = 7.5, 1.3 Hz, 1H), 6.99 (td, *J* = 7.7, 1.8 Hz, 1H), 6.65 (d, *J* = 8.3 Hz, 1H), 3.26 (d, *J* = 13.7 Hz, 1H), 3.09 (d, *J* = 13.7 Hz, 1H), 2.22 (s, 3H), 1.36 (s, 3H) ppm.

^{13}C NMR (126 MHz, $CDCl_3$) δ 204.54, 158.57, 138.92, 136.27, 132.93, 132.23, 128.61, 128.25, 127.44, 125.76, 124.16, 120.20, 112.26, 68.37, 42.38, 23.45, 20.67 ppm.

HRMS (m/z): [M + H$^+$] calcd for $C_{17}H_{17}ONBr$, 330.04880; found, 330.04931.

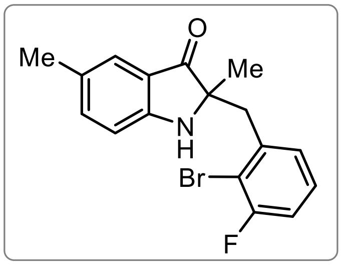

2-(2-bromo-3-fluorobenzyl)-2,5-dimethylindolin-3-one [**4l**]: Compound **4l** was prepared according to the general procedure G (four set of 0.25 mmol scale).

Yield: 55%.

Eluent: Petroleum ether/Ethyl acetate (97/3, v/v).

TLC: R_f = 0.64 (90:10 petroleum ether:EtOAc).

Physical State: Yellow liquid.

^{1}H NMR (500 MHz, Chloroform-*d*) δ 7.34 (d, *J* = 1.9 Hz, 1H), 7.19 (dd, *J* = 8.4, 1.9 Hz, 1H), 7.07 (td, *J* = 7.9, 5.5 Hz, 1H), 6.99 (dt, *J* = 7.8, 1.3 Hz, 1H), 6.91 (td, *J* = 8.2, 1.6 Hz, 1H), 6.66 (d, *J* = 8.3 Hz, 1H), 4.65 (s, 1H), 3.31 (d, *J* = 13.7 Hz, 1H), 3.15 (d, *J* = 13.6 Hz, 1H), 2.23 (s, 3H), 1.37 (s, 3H) ppm.

^{13}C NMR (126 MHz, $CDCl_3$) δ 204.22, 160.08, 158.12 [159.10 (d, *J* = 246.1 Hz)], 158.51, 139.03, 138.90, 128.42, 128.19, 128.13 [128.16 (d, *J* = 8.2 Hz)], 127.25, 127.22 [127.24 (d, *J* = 3.1 Hz)], 124.20, 120.11, 114.95, 114.77 [114.86 (d, *J* = 22.8 Hz)], 112.97, 112.81 [112.89 (d, *J* = 20.0 Hz)], 112.31, 68.26, 41.93, 41.91 [41.92 (d, *J* = 2.4 Hz)], 23.63, 20.67 ppm.

^{19}F NMR (470 MHz, Chloroform-*d*) δ -103.23 (dd, *J* = 8.4, 5.5 Hz) ppm.

HRMS (m/z): [M + H$^+$] calcd for $C_{17}H_{16}ONBrF$, 348.03938; found, 348.03911.

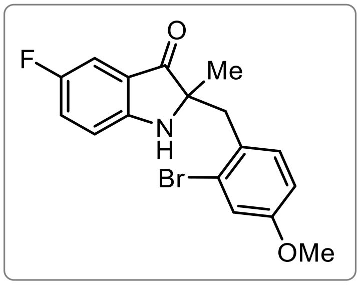

2-(2-bromo-4-methoxybenzyl)-5-fluoro-2-methylindolin-3-one [**4m**]: Compound **4m** was prepared according to the general procedure G (four set of 0.25 mmol scale).

Yield: 62%.

Eluent: Petroleum ether/Ethyl acetate (90/10, v/v).

TLC: R_f = 0.45 (85:15 petroleum ether:EtOAc).

Physical State: Yellow solid.

^{1}H NMR (600 MHz, Chloroform-*d*) δ 7.18 (ddd, *J* = 7.4, 2.7, 0.6 Hz, 1H), 7.11 (td, *J* = 8.7, 2.7 Hz, 1H), 7.07 (d, *J* = 8.5 Hz, 1H), 7.02 (d, *J* = 2.7 Hz, 1H), 6.69 (ddd, *J* = 8.9, 3.8, 0.6 Hz, 1H), 6.67 (dd, *J* = 8.6, 2.7 Hz, 1H), 4.76 (s, 1H), 3.72 (s, 3H), 3.19 (d, *J* = 14.0 Hz, 1H), 3.06 (d, *J* = 14.0 Hz, 1H), 1.37 (s, 3H) ppm.

^{13}C NMR (151 MHz, $CDCl_3$) δ 204.47, 204.45 [204.46 (d, *J* = 3.3 Hz)], 159.11, 156.89, 157.22, 155.64 [156.43 (d, *J* = 239.3 Hz)], 132.51, 127.79, 125.75, 125.59 [125.67 (d, *J* = 24.5 Hz)], 125.42, 120.59, 120.55 [120.57 (d, *J* = 7.2 Hz)], 118.16, 113.66, 113.38, 113.33 [113.36 (d, *J* = 7.6 Hz)], 109.57, 109.42 [109.49 (d, *J* = 22.5 Hz)], 69.54, 55.67, 41.78, 23.41 ppm.

^{19}F NMR (565 MHz, Chloroform-*d*) δ -125.39 (td, *J* = 7.9, 3.7 Hz) ppm.

HRMS (m/z): [M + H$^+$] calcd for $C_{17}H_{16}O_2NBrF$, 364.03430; found, 364.03477.

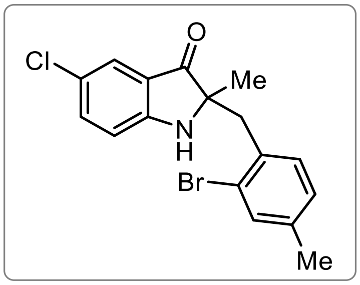

2-(2-bromo-4-methylbenzyl)-5-chloro-2-methylindolin-3-one [**4n**]: Compound **4n** was prepared according to the general procedure G (four set of 0.25 mmol scale).

Yield: 66%.

Eluent: Petroleum ether/Ethyl acetate (97/3, v/v).

TLC: R_f = 0.6 (90:10 petroleum ether:EtOAc).

Physical State: Yellow liquid.

^{1}H NMR (500 MHz, Chloroform-*d*) δ 7.62 (d, *J* = 2.3 Hz, 1H), 7.52 (dd, *J* = 8.7, 2.3 Hz, 1H), 7.34 (d, *J* = 8.7 Hz, 1H), 7.11 (d, *J* = 1.4 Hz, 1H), 7.07 (d, *J* = 7.6 Hz, 1H), 6.90 – 6.84 (m, 1H), 3.30 (d, *J* = 16.2 Hz, 1H), 2.91 (d, *J* = 16.1 Hz, 1H), 2.41 (s, 3H), 1.51 (s, 3H) ppm.

^{13}C NMR (126 MHz, $CDCl_3$) δ 203.99, 158.54, 145.32, 138.38, 137.19, 130.28, 127.67, 125.68, 125.61, 124.85, 124.76, 116.68, 115.61, 76.73, 37.64, 23.94, 21.85 ppm.

HRMS (m/z): [M + H^+] calcd for $C_{17}H_{16}ONBrCl$, 364.00983; found, 364.01047.

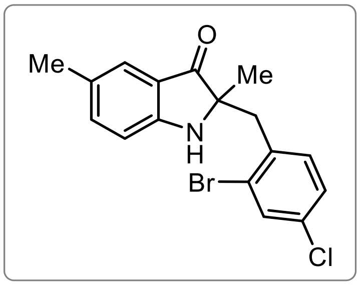

2-(2-bromo-4-chlorobenzyl)-2,5-dimethylindolin-3-one [**4o**]: Compound **4o** was prepared according to the general procedure G (four set of 0.25 mmol scale).

Yield: 60%.

Eluent: Petroleum ether/Ethyl acetate (98/2, v/v).

TLC: R_f = 0.64 (90:10 petroleum ether:EtOAc).

Physical State: Yellow liquid.

¹H NMR (600 MHz, Chloroform-*d*) δ 7.63 (d, *J* = 2.1 Hz, 1H), 7.48 (dt, *J* = 1.8, 0.9 Hz, 1H), 7.35 (ddd, *J* = 8.3, 1.9, 0.6 Hz, 1H), 7.27 (d, *J* = 8.3 Hz, 1H), 7.23 (dd, *J* = 8.3, 2.1 Hz, 1H), 6.81 (dd, *J* = 8.2, 0.6 Hz, 1H), 4.78 (s, 1H), 3.38 (d, *J* = 13.8 Hz, 1H), 3.23 (d, *J* = 13.8 Hz, 1H), 2.39 (s, 3H), 1.51 (s, 3H) ppm.

¹³C NMR (151 MHz, $CDCl_3$) δ 204.23, 158.55, 139.11, 135.02, 133.37, 132.74, 132.43, 128.54, 127.70, 126.04, 124.22, 120.17, 112.32, 68.28, 41.86, 23.76, 20.69 ppm.

ppm.

HRMS (m/z): [M + H⁺] calcd for $C_{17}H_{16}ONBrCl$, 364.00983; found, 364.00934.

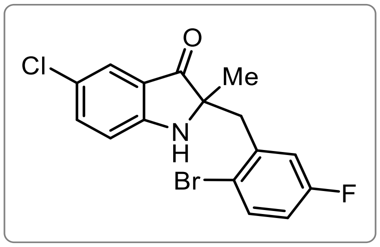

2-(2-bromo-5-fluorobenzyl)-5-chloro-2-methylindolin-3-one [**4p**]: Compound **4p** was prepared according to the general procedure G (four set of 0.25 mmol scale).

Yield: 73%.

Eluent: Petroleum ether/Ethyl acetate (97/3, v/v).

TLC: R_f = 0.6 (90:10 petroleum ether:EtOAc).

Physical State: Yellow liquid.

¹H NMR (500 MHz, Chloroform-*d*) δ 7.51 (d, *J* = 2.3 Hz, 1H), 7.44 (dd, *J* = 8.8, 5.4 Hz, 1H), 7.30 (dd, *J* = 8.7, 2.2 Hz, 1H), 6.91 (dd, *J* = 9.2, 3.1 Hz, 1H), 6.77 (ddd, *J* = 8.8, 7.7, 3.1 Hz, 1H), 6.70 (d, *J* = 8.6 Hz, 1H), 4.83 (s, 1H), 3.26 (d, *J* = 13.7 Hz, 1H), 3.08 (d, *J* = 13.8 Hz, 1H), 1.39 (s, 3H) ppm.

¹³C NMR (126 MHz, $CDCl_3$) δ 202.64, <u>162.49, 160.52</u> [161.50 (d, *J* = 247.8 Hz)], 158.02, <u>137.89, 137.83</u> [137.86 (d, *J* = 7.5 Hz)], 137.33, <u>133.95, 133.88</u> [133.92 (d, *J* = 8.1 Hz)], 124.10, 124.07, 120.78, <u>119.74, 119.71</u> [119.73 (d, *J* = 3.2 Hz)], <u>118.80, 118.62</u> [118.71 (d, *J* = 22.7 Hz)], <u>116.06, 115.88</u> [115.97 (d, *J* = 22.4 Hz)], 113.26, 68.65, <u>42.38, 42.37</u> [42.38 (d, *J* = 1.3 Hz)], 23.38 ppm.
¹⁹F NMR (470 MHz, Chloroform-*d*) δ -114.17 (td, *J* = 8.6, 5.3 Hz) ppm.

HRMS (m/z): [M + H⁺] calcd for $C_{16}H_{13}ONBrClF$, 367.98476; found, 367.98543.

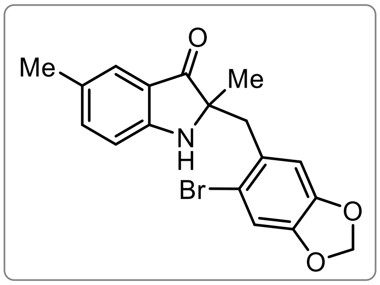

2-((6-bromobenzo[d][1,3]dioxol-5-yl)methyl)-2,5-dimethylindolin-3-one [**4q**]: Compound **4q** was prepared according to the general procedure G (four set of 0.25 mmol scale).

Yield: 43%.

Eluent: Petroleum ether/Ethyl acetate (85/15, v/v).

TLC: R_f = 0.42 (80:20 petroleum ether:EtOAc).

Physical State: Yellow oil.

^{1}H NMR (500 MHz, Chloroform-*d*) δ 7.37 (dt, *J* = 1.8, 0.9 Hz, 1H), 7.22 (dd, *J* = 8.4, 1.9 Hz, 1H), 6.93 (s, 1H), 6.73 – 6.68 (m, 2H), 5.89 (dd, *J* = 9.2, 1.5 Hz, 2H), 3.18 (d, *J* = 13.9 Hz, 1H), 2.98 (d, *J* = 13.9 Hz, 1H), 2.25 (s, 3H), 1.35 (s, 3H) ppm.

^{13}C NMR (126 MHz, $CDCl_3$) δ 204.48, 158.48, 147.45, 147.34, 138.96, 129.27, 128.51, 124.33, 120.38, 116.01, 112.74, 112.51, 111.49, 101.87, 68.50, 42.28, 23.46, 20.72 ppm.

HRMS (m/z): [M + H^+] calcd for $C_{18}H_{17}O_3NBr$, 374.03863; found, 374.03926.

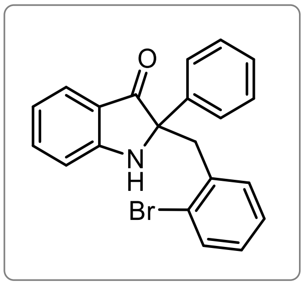

2-(2-bromobenzyl)-2-phenylindolin-3-one [**4r**]: Compound **4r** was prepared according to the general procedure G (four set of 0.25 mmol scale).

Yield: 52%.

Eluent: Petroleum ether/Ethyl acetate (97/3, v/v).

TLC: R_f = 0.6 (90:10 petroleum ether:EtOAc).

Physical State: Yellow liquid.

^{1}H NMR (500 MHz, Chloroform-*d*) δ 7.73 – 7.65 (m, 2H), 7.47 (td, *J* = 8.1, 1.4 Hz, 2H), 7.41 – 7.33 (m, 2H), 7.36 – 7.27 (m, 2H), 7.03 – 6.92 (m, 3H), 6.80 – 6.75 (m, 1H), 6.70 (ddd, *J* = 7.8, 7.0, 0.8 Hz, 1H), 5.37 (s, 1H), 3.73 (d, *J* = 13.7 Hz, 1H), 3.64 (d, *J* = 13.8 Hz, 1H) ppm.

^{13}C NMR (126 MHz, CDCl$_3$) δ 201.54, 160.58, 139.54, 137.57, 135.43, 132.84, 132.08, 128.83, 128.01, 127.47, 126.09, 125.93, 125.13, 119.71, 119.06, 111.86, 72.57, 43.74 ppm.

HRMS (m/z): [M + H$^+$] calcd for $C_{21}H_{17}ONBr$, 378.04880; found, 378.04834.

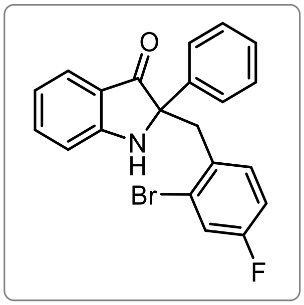

2-(2-bromo-4-fluorobenzyl)-2-phenylindolin-3-one [**4s**]: Compound **4s** was prepared according to the general procedure G (four set of 0.25 mmol scale).

Yield: 56%.

Eluent: Petroleum ether/Ethyl acetate (97/3, v/v).

TLC: R$_f$ = 0.61 (90:10 petroleum ether:EtOAc).

Physical State: Yellow liquid.

^{1}H NMR (500 MHz, Chloroform-*d*) δ 7.68 – 7.63 (m, 2H), 7.48 (dd, *J* = 7.8, 1.3 Hz, 1H), 7.40 – 7.34 (m, 3H), 7.34 – 7.29 (m, 1H), 7.21 (dd, *J* = 8.2, 2.7 Hz, 1H), 6.99 (dd, *J* = 8.6, 6.1 Hz, 1H), 6.81 – 6.77 (m, 1H), 6.75 – 6.69 (m, 2H), 5.28 (s, 1H), 3.74 – 3.56 (m, 2H) ppm.

^{13}C NMR (126 MHz, CDCl$_3$) δ 201.38, 162.34, 160.35 [161.35 (d, *J* = 250.6 Hz)], 160.51, 139.34, 137.75, 132.84, 132.77 [132.80 (d, *J* = 8.3 Hz)], 131.44, 131.41 [131.43 (d, *J* = 3.6 Hz)], 128.92, 128.13, 126.00, 125.75, 125.67 [125.71 (d, *J* = 9.5 Hz)], 125.17, 120.07, 119.88 [119.97 (d, *J* = 24.4 Hz)], 119.65, 119.22, 114.80, 114.64 [114.72 (d, *J* = 20.8 Hz)], 111.83, 72.55, 42.91 ppm.

^{19}F NMR (470 MHz, Chloroform-*d*) δ -112.03 – -113.83 (m) ppm.

HRMS (m/z): [M + H$^+$] calcd for $C_{21}H_{16}ONBrF$, 396.03938; found, 396.03914.

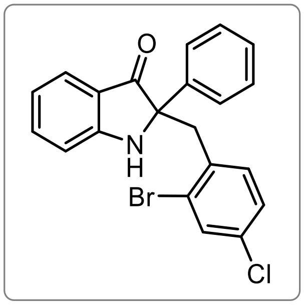

2-(2-bromo-4-chlorobenzyl)-2-phenylindolin-3-one [**4t**]: Compound **4t** was prepared according to the general procedure G (four set of 0.25 mmol scale).

Yield: 61%.

Eluent: Petroleum ether/Ethyl acetate (97/3, v/v).

TLC: R_f = 0.62 (90:10 petroleum ether:EtOAc).

Physical State: Yellow liquid.

^{1}H NMR (700 MHz, Chloroform-*d*) δ 7.70 – 7.62 (m, 2H), 7.49 (dd, *J* = 8.7, 1.6 Hz, 2H), 7.39 – 7.34 (m, 3H), 7.33 – 7.29 (m, 1H), 6.98 (dd, *J* = 8.3, 2.1 Hz, 1H), 6.94 (d, *J* = 8.3 Hz, 1H), 6.80 (dd, *J* = 8.3, 0.8 Hz, 1H), 6.73 (ddd, *J* = 7.8, 7.0, 0.8 Hz, 1H), 5.29 (s, 1H), 3.69 (d, *J* = 14.0 Hz, 1H), 3.60 (d, *J* = 13.9 Hz, 1H) ppm.

^{13}C NMR (176 MHz, $CDCl_3$) δ 201.19, 160.47, 139.26, 137.78, 134.20, 133.65, 132.63, 132.41, 128.93, 128.14, 127.71, 126.19, 125.97, 125.21, 119.58, 119.28, 111.88, 72.42, 43.05 ppm.

HRMS (m/z): [M + H^+] calcd for $C_{21}H_{16}ONBrCl$, 412.0098; found, 412.0096.

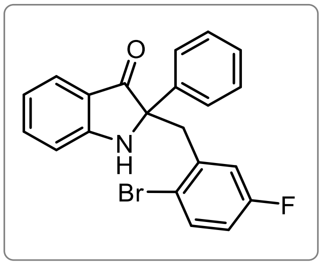

2-(2-bromo-5-fluorobenzyl)-2-phenylindolin-3-one [**4u**]: Compound **4u** was prepared according to the general procedure G (four set of 0.25 mmol scale).

Yield: 57%.

Eluent: Petroleum ether/Ethyl acetate (97/3, v/v).

TLC: R_f = 0.62 (90:10 petroleum ether:EtOAc).

Physical State: Yellow liquid.

^{1}H NMR (500 MHz, Chloroform-*d*) δ 7.68 – 7.64 (m, 2H), 7.51 (dt, *J* = 7.7, 1.2 Hz, 1H), 7.45 – 7.29 (m, 5H), 6.79 (dt, *J* = 8.2, 0.8 Hz, 1H), 6.77 – 6.69 (m, 3H), 5.31 (s, 1H), 3.69 (d, *J* = 13.8 Hz, 1H), 3.61 (d, *J* = 13.8 Hz, 1H) ppm.

^{13}C NMR (126 MHz, $CDCl_3$) δ 201.12, 162.55, 160.58 [161.57 (d, *J* = 247.6 Hz)], 160.46, 139.17, 137.74, 137.71, 137.65 [137.68 (d, *J* = 7.7 Hz)], 133.90, 133.83 [133.87 (d, *J* = 7.9 Hz)], 128.96, 128.19, 125.96, 125.31, 120.12, 120.10 [120.11 (d, *J* = 3.2 Hz)], 119.61, 119.31, 118.98, 118.80 [118.89 (d, *J* = 22.9 Hz)], 116.21, 116.03 [116.12 (d, *J* = 22.4 Hz)], 111.83, 72.34, 43.73, 43.71 [43.72 (d, *J* = 1.7 Hz)] ppm.

^{19}F NMR (470 MHz, Chloroform-*d*) δ -114.26 (ddd, *J* = 9.6, 7.8, 5.4 Hz) ppm.

HRMS (m/z): [M + H$^+$] calcd for $C_{21}H_{16}ONBrF$, 396.03938; found, 396.03899.

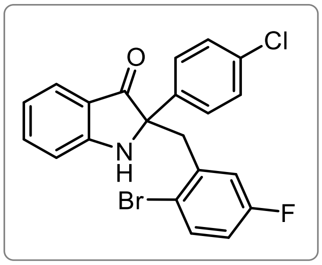

2-(2-bromo-5-fluorobenzyl)-2-(4-chlorophenyl)indolin-3-one [**4v**]: Compound **4v** was prepared according to the general procedure G (four set of 0.25 mmol scale).

Yield: 48%.

Eluent: Petroleum ether/Ethyl acetate (97/3, v/v).

TLC: R_f = 0.64 (90:10 petroleum ether:EtOAc).

Physical State: Yellow oil.

^{1}H NMR (500 MHz, Chloroform-*d*) δ 7.64 – 7.59 (m, 2H), 7.51 (ddd, *J* = 7.8, 1.4, 0.7 Hz, 1H), 7.45 – 7.41 (m, 1H), 7.39 – 7.32 (m, 3H), 6.80 (dd, *J* = 8.3, 0.8 Hz, 1H), 6.78 – 6.70 (m, 3H), 5.28 (s, 1H), 3.64 (d, *J* = 13.9 Hz, 1H), 3.56 (d, *J* = 13.8 Hz, 1H) ppm.

^{13}C NMR (126 MHz, $CDCl_3$) δ 200.76, 162.59, 160.62 [161.60 (d, *J* = 248.2 Hz)], 160.40, 137.92, 137.77, 137.31, 137.25 [137.28 (d, *J* = 7.7 Hz)], 134.23, 134.04, 133.98 [134.01 (d, *J* = 8.1 Hz)], 129.04, 128.65, 127.52, 125.36, 119.98, 119.95 [119.96 (d, *J* = 3.2 Hz)], 119.64, 119.53, 118.97, 118.78 [118.87 (d, *J* = 23.1 Hz)], 116.43, 116.25 [116.34 (d, *J* = 22.6 Hz)], 111.99, 71.84, 43.80 ppm.

^{19}F NMR (470 MHz, Chloroform-*d*) δ -113.94 (td, *J* = 8.5, 5.1 Hz) ppm.

HRMS (m/z): [M + H$^+$] calcd for $C_{21}H_{15}ONBrClF$, 430.00041; found, 430.00042.

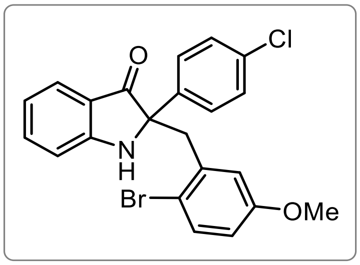

2-(2-bromo-5-methoxybenzyl)-2-(4-chlorophenyl)indolin-3-one [**4w**]: Compound **4w** was prepared according to the general procedure G (four set of 0.25 mmol scale).

Yield: 44%.

Eluent: Petroleum ether/Ethyl acetate (90/10, v/v).

TLC: R_f = 0.36 (80:20 petroleum ether:EtOAc).

Physical State: Yellow oil.

^{1}H NMR (500 MHz, Chloroform-*d*) δ 7.62 – 7.58 (m, 2H), 7.56 (ddd, *J* = 7.8, 1.4, 0.7 Hz, 1H), 7.44 (ddd, *J* = 8.4, 7.1, 1.4 Hz, 1H), 7.33 – 7.29 (m, 2H), 7.08 (dd, *J* = 8.3, 7.5 Hz, 1H), 6.88 (dt, *J* = 8.3, 0.8 Hz, 1H), 6.81 (ddd, *J* = 7.8, 7.1, 0.8 Hz, 1H), 6.71 (ddd, *J* = 8.3, 2.6, 0.9 Hz, 1H), 6.51 (d, *J* = 7.6 Hz, 1H), 6.40 (dd, *J* = 2.5, 1.6 Hz, 1H), 4.93 (s, 1H), 3.62 (s, 3H), 3.47 (d, *J* = 13.7 Hz, 1H), 3.10 (d, *J* = 13.7 Hz, 1H) ppm.

^{13}C NMR (126 MHz, $CDCl_3$) δ 201.03, 159.95, 159.52, 137.75, 137.53, 137.04, 133.86, 129.53, 128.73, 127.96, 125.66, 122.46, 119.65, 119.59, 115.50, 113.10, 112.42, 71.08, 55.24, 44.76 ppm.

HRMS (m/z): [M + H^+] calcd for $C_{22}H_{18}O_2NBrCl$, 442.02040; found, 442.02020.

CLASS-F

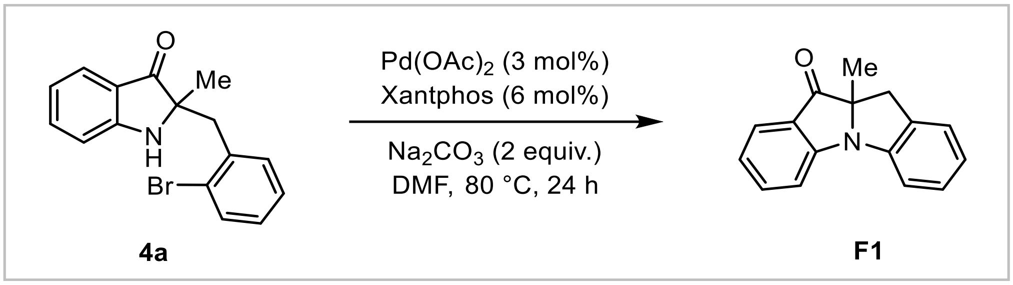

General procedure H: Synthesis of Class F

An oven-dried screw capped reaction tube with a magnetic stir-bar was charged with 2-(2-bromobenzyl)-2-methylindolin-3-one (0.2 mmol), $Pd(OAc)_2$ (3 mol%, 1.4 mg), Xantphos (6 mol%, 7 mg), Na_2CO_3 (2 equiv., 42.4 mg). A screw cap fitted with a rubber septum was attached to the reaction tube, then degassed and refilled with argon. Dry *N,N*-dimethyl formamide (DMF, 2 mL) was added in the reaction tube, placed in metal block, and vigorously stirred at 80 °C. The reaction mixture was taken out after 24 h, diluted with 10 mL of ethyl acetate and 25 mL of water. The organic layer was separated and concentrated under vacuum. The crude mixture was purified by column chromatography using silica gel (100-200 mesh size) and petroleum ether/ethyl acetate as the eluent.

Characterization data for Class F products

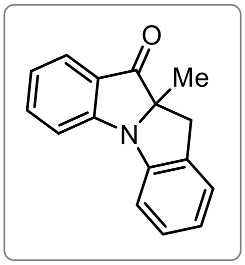

10a-methyl-10a,11-dihydro-10H-indolo[1,2-a]indol-10-one [**F1**]: Compound **F1** was prepared according to the general procedure H (0.2 mmol scale).

Yield: 59% (27.8 mg isolated).

Eluent: Petroleum ether/Ethyl acetate (97/3, v/v).

TLC: R_f = 0.55 (90:10 petroleum ether:EtOAc).

Physical State: Yellow oil.

^{1}H NMR (400 MHz, Chloroform-*d*) δ 7.68 (ddd, *J* = 7.6, 1.4, 0.7 Hz, 1H), 7.58 (ddd, *J* = 8.4, 7.2, 1.4 Hz, 1H), 7.40 (dt, *J* = 8.3, 0.8 Hz, 1H), 7.36 – 7.32 (m, 1H), 7.31 – 7.24 (m, 1H), 7.18 (dtt, *J* = 7.5, 1.3, 0.7 Hz, 1H), 7.09 – 7.00 (m, 2H), 3.35 (d, *J* = 16.3 Hz, 1H), 2.96 (d, *J* = 16.3 Hz, 1H), 1.54 (s, 3H) ppm.

^{13}C NMR (101 MHz, Chloroform-*d*) δ 205.03, 160.25, 145.69, 137.39, 133.44, 128.15, 125.86, 125.36, 124.55, 123.79, 122.27, 115.93, 114.44, 75.60, 37.96, 24.00 ppm.

HRMS (m/z): [M + H$^+$] calcd for $C_{16}H_{14}ON$, 236.10699; found, 236.10696.

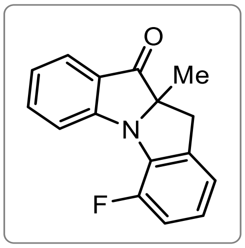

4-fluoro-10a-methyl-10a,11-dihydro-10H-indolo[1,2-a]indol-10-one [**F2**]: Compound **F2** was prepared according to the general procedure H (0.2 mmol scale).

Yield: 45% (22.8 mg).

Eluent: Petroleum ether/Ethyl acetate (97/3, v/v).

TLC: R_f = 0.57 (90:10 petroleum ether:EtOAc).

Physical State: Yellow oil.

^{1}H NMR (500 MHz, Chloroform-*d*) δ 7.68 (ddd, *J* = 7.8, 1.5, 0.7 Hz, 1H), 7.60 (ddd, *J* = 8.4, 7.0, 1.4 Hz, 1H), 7.52 (dd, *J* = 8.2, 0.9 Hz, 1H), 7.10 – 7.03 (m, 2H), 7.03 – 6.95 (m, 2H), 3.34 (d, *J* = 16.2 Hz, 1H), 2.94 (d, *J* = 16.3 Hz, 1H), 1.54 (s, 3H) ppm.

^{13}C NMR (126 MHz, $CDCl_3$) δ 204.05, 160.85, 154.01, 152.05 [153.03 (d, *J* = 245.8 Hz)], 137.91, 137.89 [137.90 (d, *J* = 1.8 Hz)], 137.87, 132.63, 132.54, 125.49, 125.44 [125.46 (d, *J* = 6.8 Hz)], 125.20, 124.53, 122.47, 121.53, 121.50 [121.52 (d, *J* = 3.2 Hz)], 116.46, 116.36 [116.41 (d, *J* = 12.6 Hz)], 115.90, 115.74 [115.82 (d, *J* = 19.8 Hz)], 76.93, 38.40, 38.38 [38.39 (d, *J* = 2.2 Hz)], 23.20 ppm.

^{19}F NMR (470 MHz, Chloroform-*d*) δ -124.33 (dd, *J* = 10.5, 4.8 Hz) ppm.

HRMS (m/z): [M + H$^+$] calcd for $C_{16}H_{13}ONF$, 254.09757; found, 254.09764.

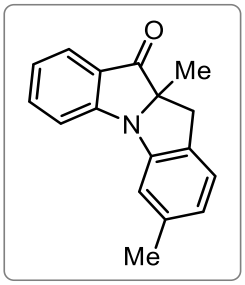

3,10a-dimethyl-10a,11-dihydro-10H-indolo[1,2-a]indol-10-one [**F3**]: Compound **F3** was prepared according to the general procedure H (0.2 mmol scale).

Yield: 65% (32.4 mg).

Eluent: Petroleum ether/Ethyl acetate (97/3, v/v).

TLC: R_f = 0.6 (90:10 petroleum ether:EtOAc).

Physical State: Yellow oil.

^{1}H NMR (500 MHz, Chloroform-*d*) δ 7.67 (ddd, *J* = 7.7, 1.5, 0.7 Hz, 1H), 7.60 (ddd, *J* = 8.5, 7.2, 1.4 Hz, 1H), 7.41 (dt, *J* = 8.3, 0.8 Hz, 1H), 7.16 (d, *J* = 1.5 Hz, 1H), 7.06 (ddd, *J* = 7.9, 7.2, 0.9 Hz, 2H), 6.86 (ddd, *J* = 7.5, 1.6, 0.8 Hz, 1H), 3.30 (d, *J* = 16.1 Hz, 1H), 2.92 (d, *J* = 16.1 Hz, 1H), 2.41 (s, 3H), 1.52 (s, 3H) ppm.

^{13}C NMR (126 MHz, $CDCl_3$) δ 205.32, 160.30, 145.80, 138.22, 137.43, 130.44, 125.52, 125.39, 124.56, 124.52, 122.22, 116.74, 114.48, 75.98, 37.68, 24.03, 21.87 ppm.

HRMS (m/z): [M + H^+] calcd for $C_{17}H_{16}ON$, 250.12264; found, 250.12263.

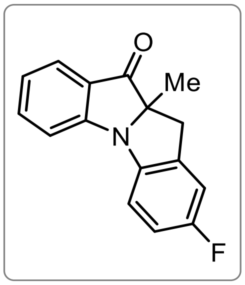

2-fluoro-10a-methyl-10a,11-dihydro-10H-indolo[1,2-a]indol-10-one [**F4**]: Compound **F4** was prepared according to the general procedure H (0.2 mmol scale).

Yield: 52% (26.3 mg).

Eluent: Petroleum ether/Ethyl acetate (97/3, v/v).

TLC: R_f = 0.56 (90:10 petroleum ether:EtOAc).

Physical State: Yellow oil.

^{1}H NMR (600 MHz, Chloroform-*d*) δ 7.69 (ddd, *J* = 7.7, 1.4, 0.7 Hz, 1H), 7.60 (ddd, *J* = 8.4, 7.2, 1.4 Hz, 1H), 7.34 (dt, *J* = 8.3, 0.8 Hz, 1H), 7.26 – 7.24 (m, 1H), 7.08 (td, *J* = 7.5, 0.8 Hz, 1H), 7.01 – 6.94 (m, 1H), 6.91 (ddt, *J* = 8.2, 2.5, 1.1 Hz, 1H), 3.35 (dd, *J* = 16.6, 1.3 Hz, 1H), 2.94 (d, *J* = 16.6 Hz, 1H), 1.53 (s, 3H) ppm.

^{13}C NMR (151 MHz, $CDCl_3$) δ 204.71, 160.45, 160.69, 159.09 [159.89 (d, *J* = 242.0 Hz)], 141.96, 141.94 [141.95 (d, *J* = 2.1 Hz)], 137.56, 135.50, 135.44 [135.47 (d, *J* = 8.6 Hz)], 125.53, 124.42,

122.50, 116.65, 116.59 [116.62 (d, *J* = 8.7 Hz)], 114.75, 114.59 [114.67 (d, *J* = 23.7 Hz)], 114.30, 113.31, 113.15 [113.23 (d, *J* = 24.0 Hz)], 76.28, 38.23, 38.21 [38.22 (d, *J* = 2.0 Hz)], 23.86 ppm.

^{19}F NMR (565 MHz, Chloroform-*d*) δ -119.67 (td, *J* = 8.5, 4.3 Hz) ppm.

HRMS (m/z): [M + H$^+$] calcd for $C_{16}H_{13}ONF$, 254.09757; found, 254.09779.

Compound **F4** was resynthesized (**F4-resynthesized**) and retested in biological assays. The ^{1}H NMR spectra of the resynthesized batch is analogous to the original batch.

^{1}H NMR (400 MHz, Chloroform-*d*) δ 7.71 (d, *J* = 7.7 Hz, 1H), 7.62 (td, *J* = 7.8, 1.8 Hz, 1H), 7.36 (dd, *J* = 8.3, 1.7 Hz, 1H), 7.28 (td, *J* = 4.6, 2.6 Hz, 1H), 7.10 (td, *J* = 7.4, 1.6 Hz, 1H), 7.03 – 6.95 (m, 1H), 6.95 – 6.90 (m, 1H), 3.36 (d, *J* = 16.6 Hz, 1H), 2.96 (d, *J* = 16.6 Hz, 1H), 1.55 (s, 3H).

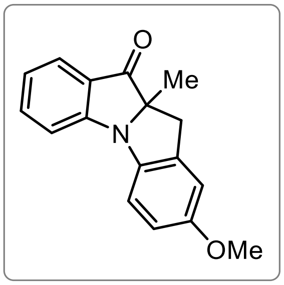

2-methoxy-10a-methyl-10a,11-dihydro-10H-indolo[1,2-a]indol-10-one [**F5**]: Compound **F5** was prepared according to the general procedure H (0.2 mmol scale).

Yield: 57% (30.2 mg).

Eluent: Petroleum ether/Ethyl acetate (90/10, v/v).

TLC: R_f = 0.4 (80:20 petroleum ether:EtOAc).

Physical State: Brown semi-solid.

^{1}H NMR (500 MHz, Chloroform-*d*) δ 7.67 (ddd, *J* = 7.7, 1.4, 0.7 Hz, 1H), 7.58 (ddd, *J* = 8.5, 7.2, 1.4 Hz, 1H), 7.33 (dt, *J* = 8.2, 0.8 Hz, 1H), 7.26 (d, *J* = 8.5 Hz, 1H), 7.04 (ddd, *J* = 7.9, 7.1, 0.8 Hz, 1H), 6.82 (ddt, *J* = 8.5, 2.7, 0.8 Hz, 1H), 6.76 (dt, *J* = 2.5, 1.1 Hz, 1H), 3.77 (s, 3H), 3.33 (d, *J* = 16.4 Hz, 1H), 2.92 (d, *J* = 16.4 Hz, 1H), 1.53 (s, 3H) ppm.

^{13}C NMR (176 MHz, $CDCl_3$) δ 205.12, 161.07, 156.89, 139.40, 137.47, 135.09, 125.40, 124.33, 122.11, 116.72, 114.33, 113.35, 111.91, 76.22, 55.97, 38.49, 23.72 ppm.

HRMS (m/z): [M + H$^+$] calcd for $C_{17}H_{16}O_2N$, 266.11756; found, 266.11759.

Compound **F5** was resynthesized (**F5-resynthesized**) and retested in biological assays. The ^{1}H NMR spectra of the resynthesized batch is analogous to the original batch.

^{1}H NMR (400 MHz, Chloroform-*d*) δ 7.66 (d, *J* = 7.7 Hz, 1H), 7.57 (ddd, *J* = 8.4, 7.1, 1.4 Hz, 1H), 7.35 – 7.30 (m, 1H), 7.28 – 7.21 (m, 1H), 7.04 (t, *J* = 7.5 Hz, 1H), 6.81 (dd, *J* = 8.5, 2.5 Hz, 1H), 6.76 (dd, *J* = 2.6, 1.3 Hz, 1H), 3.77 (s, 3H), 3.33 (d, *J* = 16.4 Hz, 1H), 2.92 (d, *J* = 16.4 Hz, 1H), 1.53 (s, 3H).

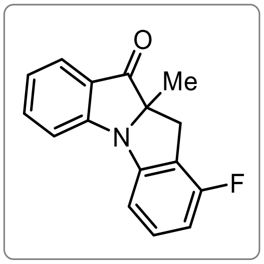

1-fluoro-10a-methyl-10a,11-dihydro-10H-indolo[1,2-a]indol-10-one [**F6**]: Compound **F6** was prepared according to the general procedure H (0.2 mmol scale).

Yield: 51% (25.8 mg).

Eluent: Petroleum ether/Ethyl acetate (97/3, v/v).

TLC: R_f = 0.55 (90:10 petroleum ether:EtOAc).

Physical State: Yellow oil.

^{1}H NMR (500 MHz, Chloroform-d) δ 7.69 (ddd, J = 7.7, 1.4, 0.7 Hz, 1H), 7.61 (ddd, J = 8.5, 7.2, 1.4 Hz, 1H), 7.39 (dt, J = 8.2, 0.8 Hz, 1H), 7.30 – 7.21 (m, 2H), 7.14 – 7.08 (m, 2H), 6.76 (td, J = 8.5, 0.8 Hz, 1H), 3.33 (dt, J = 16.6, 1.3 Hz, 1H), 3.04 (dd, J = 16.6, 0.8 Hz, 1H), 1.55 (s, 3H) ppm.

^{13}C NMR (126 MHz, $CDCl_3$) δ 204.51, 159.77, 160.42, 158.45 [159.43 (d, *J* = 248.0 Hz)], 148.28, 148.23 [148.26 (d, *J* = 7.4 Hz)], 137.57, 130.04, 129.97 [130.00 (d, *J* = 8.2 Hz)], 125.52, 124.59, 122.79, 119.56, 119.40 [119.48 (d, *J* = 20.3 Hz)], 114.65, 111.54, 111.51 [111.53 (d, *J* = 3.2 Hz)], 110.93, 110.77 [110.85 (d, *J* = 20.5 Hz)], 76.15, 34.28, 24.31 ppm.

^{19}F NMR (470 MHz, Chloroform-*d*) δ -116.17 (dd, *J* = 8.7, 5.5 Hz) ppm.

HRMS (m/z): [M + H^{+}] calcd for $C_{16}H_{13}FNO$, 254.0981; found, 254.0981.

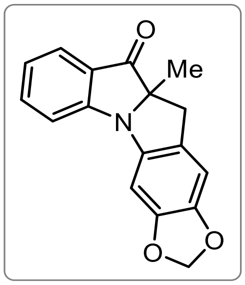

10a-methyl-10a,11-dihydro-10H-[1,3]dioxolo[4,5-f]indolo[1,2-a]indol-10-one [**F7**]: Compound **F7** was prepared according to the general procedure H (0.2 mmol scale).

Yield: 56% (31.3 mg).

Eluent: Petroleum ether/Ethyl acetate (85/15, v/v).

TLC: R_f = 0.3 (80:20 petroleum ether:EtOAc).

Physical State: Yellow oil.

^{1}H NMR (500 MHz, Chloroform-*d*) δ 7.67 (ddd, *J* = 7.7, 1.5, 0.7 Hz, 1H), 7.58 (ddd, *J* = 8.5, 7.2, 1.4 Hz, 1H), 7.31 (dt, *J* = 8.2, 0.8 Hz, 1H), 7.06 (ddd, *J* = 7.8, 7.2, 0.8 Hz, 1H), 6.90 (s, 1H), 6.66 (s, 1H), 5.97 (d, *J* = 1.4 Hz, 1H), 5.93 (d, *J* = 1.4 Hz, 1H), 3.26 (dd, *J* = 15.9, 1.1 Hz, 1H), 2.84 (d, *J* = 16.0 Hz, 1H), 1.52 (s, 3H) ppm.

^{13}C NMR (176 MHz, $CDCl_3$) δ 205.01, 160.80, 147.61, 144.43, 139.66, 137.43, 125.47, 125.31, 124.44, 122.29, 114.36, 106.22, 101.64, 99.01, 76.69, 38.11, 23.75 ppm.

HRMS (m/z): [M + H^+] calcd for $C_{17}H_{14}O_3N$, 280.09682; found, 280.09690.

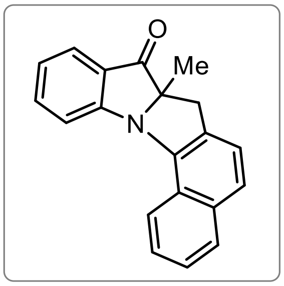

7a-methyl-7,7a-dihydro-8H-benzo[g]indolo[1,2-a]indol-8-one [**F8**]: Compound **F8** was prepared according to the general procedure H (0.2 mmol scale).

Yield: 44% (25.1 mg).

Eluent: Petroleum ether/Ethyl acetate (97/3, v/v).

TLC: R_f = 0.55 (90:10 petroleum ether:EtOAc).

Physical State: Yellow oil.

^{1}H NMR (500 MHz, Chloroform-*d*) δ 8.22 (dd, *J* = 8.5, 1.2 Hz, 1H), 7.91 (d, *J* = 8.3 Hz, 1H), 7.73 (dd, *J* = 7.8, 1.4 Hz, 1H), 7.68 – 7.61 (m, 2H), 7.53 (ddd, *J* = 8.1, 6.8, 1.2 Hz, 1H), 7.45 (ddd, *J* = 8.5, 7.1, 1.4 Hz, 1H), 7.35 (d, *J* = 8.3 Hz, 1H), 7.31 (d, *J* = 8.4 Hz, 1H), 7.08 – 7.00 (m, 1H), 3.49 (d, *J* = 16.1 Hz, 1H), 2.99 (d, *J* = 16.1 Hz, 1H), 1.60 (s, 3H) ppm.

^{13}C NMR (126 MHz, $CDCl_3$) δ 204.13, 161.96, 141.91, 137.28, 134.36, 131.16, 128.78, 126.54, 126.23, 125.84, 125.72, 125.39, 124.63, 124.49, 123.49, 122.01, 115.52, 78.00, 39.51, 21.89 ppm.

HRMS (m/z): [M + H^+] calcd for $C_{20}H_{16}ON$, 286.12264; found, 286.12270.

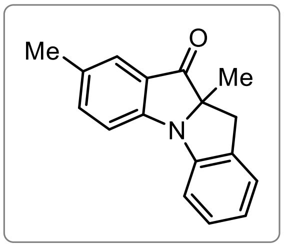

8,10a-dimethyl-10a,11-dihydro-10H-indolo[1,2-a]indol-10-one [**F9**]: Compound **F9** was prepared according to the general procedure H (0.2 mmol scale).

Yield: 66% (32.9 mg).

Eluent: Petroleum ether/Ethyl acetate (97/3, v/v).

TLC: R_f = 0.6 (90:10 petroleum ether:EtOAc).

Physical State: Yellow oil.

^{1}H NMR (400 MHz, Chloroform-*d*) δ 7.40 (dt, *J* = 1.8, 0.9 Hz, 1H), 7.38 – 7.31 (m, 1H), 7.27 – 7.15 (m, 3H), 7.12 – 7.07 (m, 1H), 6.95 (td, *J* = 7.3, 1.3 Hz, 1H), 3.28 (d, *J* = 16.4 Hz, 1H), 2.88 (d, *J* = 16.4 Hz, 1H), 2.27 (s, 3H), 1.45 (s, 3H) ppm.

^{13}C NMR (101 MHz, $CDCl_3$) δ 205.38, 158.74, 146.16, 138.68, 133.32, 132.17, 128.15, 125.84, 124.92, 124.64, 123.66, 115.84, 114.47, 75.93, 38.07, 24.19, 20.84 ppm.

HRMS (m/z): [M + H^+] calcd for $C_{17}H_{16}ON$, 250.12264; found, 250.12265.

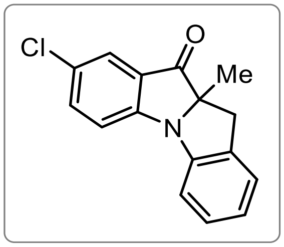

8-chloro-10a-methyl-10a,11-dihydro-10H-indolo[1,2-a]indol-10-one [**F10**]: Compound **F10** was prepared according to the general procedure H (0.2 mmol scale).

Yield: 48% (25.9 mg).

Eluent: Petroleum ether/Ethyl acetate (97/3, v/v).

TLC: R_f = 0.55 (90:10 petroleum ether:EtOAc).

Physical State: Yellow oil.

^{1}H NMR (400 MHz, Chloroform-*d*) δ 7.63 (dd, *J* = 2.2, 0.6 Hz, 1H), 7.53 (dd, *J* = 8.7, 2.3 Hz, 1H), 7.33 (dd, *J* = 8.7, 0.6 Hz, 1H), 7.31 – 7.27 (m, 2H), 7.20 (dt, *J* = 7.4, 1.0 Hz, 1H), 7.06 (ddd, *J* = 7.5, 5.9, 2.6 Hz, 1H), 3.35 (dd, *J* = 16.4, 1.3 Hz, 1H), 2.97 (dd, *J* = 16.3, 0.9 Hz, 1H), 1.53 (s, 3H) ppm.

^{13}C NMR (101 MHz, $CDCl_3$) δ 203.89, 158.57, 145.25, 137.26, 133.35, 128.33, 127.84, 126.01, 125.74, 124.82, 124.15, 115.92, 115.61, 76.43, 37.97, 23.97 ppm.

HRMS (m/z): [M + H^+] calcd for $C_{16}H_{13}ONCl$, 270.06802; found, 270.06818.

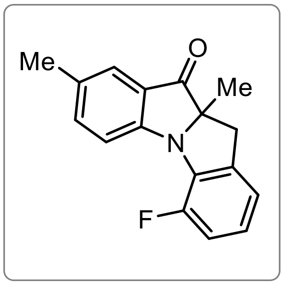

4-fluoro-8,10a-dimethyl-10a,11-dihydro-10H-indolo[1,2-a]indol-10-one [**F11**]: Compound **F11** was prepared according to the general procedure H (0.2 mmol scale).

Yield: 45% (24.1 mg).

Eluent: Petroleum ether/Ethyl acetate (98/2, v/v).

TLC: R_f = 0.6 (90:10 petroleum ether:EtOAc).

Physical State: Yellow oil.

^{1}H NMR (500 MHz, Chloroform-*d*) δ 7.47 (dt, *J* = 1.8, 0.9 Hz, 1H), 7.42 (q, *J* = 1.2 Hz, 2H), 7.07 – 6.98 (m, 2H), 6.96 (dq, *J* = 7.3, 1.4 Hz, 1H), 3.34 (d, *J* = 16.4 Hz, 1H), 2.93 (d, *J* = 16.4 Hz, 1H), 2.34 (s, 3H), 1.53 (s, 3H) ppm.

^{13}C NMR (126 MHz, $CDCl_3$) δ 204.24, 159.21, 154.00, 152.05 [153.02 (d, *J* = 245.7 Hz)], 139.18, 139.16 [139.17 (d, *J* = 1.9 Hz)], 137.76, 137.74 [137.75 (d, *J* = 3.4 Hz)], 133.00, 132.91 [132.96 (d, *J* = 11.9 Hz)], 132.29, 125.31, 125.25 [125.28 (d, *J* = 6.7 Hz)], 124.67, 124.59, 121.47, 121.44

[121.45 (d, J = 3.2 Hz)], <u>116.37, 116.27</u> [116.32 (d, J = 12.4 Hz)], <u>115.83, 115.67</u> [115.75 (d, J = 19.9 Hz)], <u>38.47, 38.45</u> [38.46 (d, J = 2.2 Hz)], 23.34, 20.83 ppm.

^{19}F NMR (470 MHz, Chloroform-*d*) δ -124.61 (dd, J = 10.7, 4.7 Hz) ppm.

HRMS (m/z): [M + H$^+$] calcd for $C_{17}H_{15}ONF$, 268.11322; found, 268.11327.

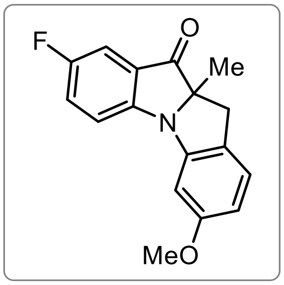

8-fluoro-3-methoxy-10a-methyl-10a,11-dihydro-10H-indolo[1,2-a]indol-10-one [**F12**]: Compound **F12** was prepared according to the general procedure H (0.2 mmol scale).

Yield: 65% (36.8 mg).

Eluent: Petroleum ether/Ethyl acetate (90/10, v/v).

TLC: R_f = 0.4 (80:20 petroleum ether:EtOAc).

Physical State: Yellow oil.

^{1}H NMR (600 MHz, Chloroform-*d*) δ 7.37 – 7.30 (m, 3H), 7.07 (dt, J = 8.2, 1.1 Hz, 1H), 6.86 (d, J = 2.3 Hz, 1H), 6.58 (dd, J = 8.2, 2.3 Hz, 1H), 3.85 (s, 3H), 3.30 (dd, J = 15.9, 1.3 Hz, 1H), 2.90 (d, J = 15.9 Hz, 1H), 1.53 (s, 3H) ppm.

^{13}C NMR (151 MHz, $CDCl_3$) δ <u>204.71, 204.69</u> [204.70 (d, J = 2.9 Hz)], 160.30, <u>159.52, 157.90</u> [158.71 (d, J = 243.6 Hz)], 156.59, 146.91, 126.12, <u>125.46, 125.41</u> [125.43 (d, J = 7.2 Hz)], <u>125.14, 125.14</u> [125.14 (d, J = 1.2 Hz)], 124.98, <u>115.89, 115.84</u> [115.87 (d, J = 7.7 Hz)], <u>110.63, 110.48</u> [110.55 (d, J = 22.6 Hz)], 108.48, 103.10, 77.49, 55.88, 37.36, 24.16 ppm.

^{19}F NMR (565 MHz, Chloroform-*d*) δ -120.70 (td, J = 7.3, 4.2 Hz) ppm.

HRMS (m/z): [M + H$^+$] calcd for $C_{17}H_{15}O_2NF$, 284.10813; found, 284.10817.

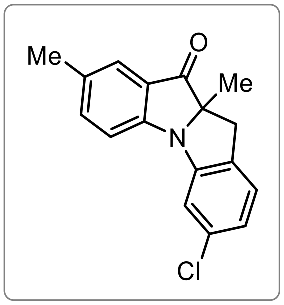

3-chloro-8,10a-dimethyl-10a,11-dihydro-10H-indolo[1,2-a]indol-10-one [**F13**]: Compound **F13** was prepared according to the general procedure H (0.2 mmol scale).

Yield: 53% (30.1 mg).

Eluent: Petroleum ether/Ethyl acetate (97/3, v/v).

TLC: R_f = 0.6 (90:10 petroleum ether:EtOAc).

Physical State: Yellow oil.

^{1}H NMR (700 MHz, Chloroform-*d*) δ 7.41 (dt, *J* = 1.7, 0.8 Hz, 1H), 7.38 (ddd, *J* = 8.3, 1.9, 0.7 Hz, 1H), 7.21 (d, *J* = 8.3 Hz, 1H), 7.20 – 7.18 (m, 1H), 7.00 (dt, *J* = 8.0, 1.2 Hz, 1H), 6.91 (dd, *J* = 7.9, 1.9 Hz, 1H), 3.23 (dd, *J* = 16.5, 1.3 Hz, 1H), 2.85 (d, *J* = 16.4 Hz, 1H), 2.28 (s, 3H), 1.44 (s, 3H) ppm.

^{13}C NMR (176 MHz, $CDCl_3$) δ 204.83, 157.86, 147.34, 138.78, 133.62, 132.74, 131.73, 126.47, 125.06, 124.71, 123.55, 116.00, 114.53, 76.48, 37.50, 24.34, 20.86 ppm.

HRMS (m/z): [M + H^+] calcd for $C_{17}H_{15}ONCl$, 284.08367; found, 284.08388.

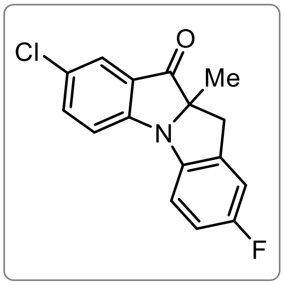

8-chloro-2-fluoro-10a-methyl-10a,11-dihydro-10H-indolo[1,2-a]indol-10-one [**F14**]: Compound **F14** was prepared according to the general procedure H (0.2 mmol scale).

Yield: 49% (28.2 mg).

Eluent: Petroleum ether/Ethyl acetate (97/3, v/v).

TLC: R_f = 0.55 (90:10 petroleum ether:EtOAc).

Physical State: Yellow oil.

^{1}H NMR (700 MHz, Chloroform-*d*) δ 7.63 (d, *J* = 2.2 Hz, 1H), 7.53 (dd, *J* = 8.7, 2.3 Hz, 1H), 7.27 (d, *J* = 8.7 Hz, 1H), 7.22 (dd, *J* = 8.6, 4.3 Hz, 1H), 7.00 – 6.96 (m, 1H), 6.91 (ddt, *J* = 8.1, 2.5, 1.1 Hz, 1H), 3.34 (d, *J* = 16.6 Hz, 1H), 2.94 (d, *J* = 16.6 Hz, 1H), 1.53 (s, 3H) ppm.

^{13}C NMR (176 MHz, $CDCl_3$) δ 203.42, <u>160.71, 159.33</u> [160.02 (d, *J* = 242.6 Hz)], 158.70, <u>141.49, 141.47</u> [141.48 (d, *J* = 2.2 Hz)], 137.36, <u>135.36, 135.31</u> [135.34 (d, *J* = 8.6 Hz)], 128.06, 125.61, 124.92, <u>116.63, 116.58</u> [116.61 (d, *J* = 8.9 Hz)], 115.46, <u>114.90, 114.77</u> [114.84 (d, *J* = 23.7 Hz)], <u>113.43, 113.29</u> [113.36 (d, *J* = 24.0 Hz)], [one peak merged in $CDCl_3$ peaks], <u>38.18, 38.17</u> [38.18 (d, *J* = 1.9 Hz)], 23.79 ppm.

^{19}F NMR (470 MHz, Chloroform-*d*) δ -119.08 (td, *J* = 8.4, 4.3 Hz) ppm.

HRMS (m/z): [M + H^+] calcd for $C_{16}H_{12}ClFNO$, 288.0586; found, 288.05876.

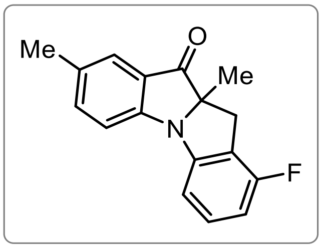

1-fluoro-8,10a-dimethyl-10a,11-dihydro-10H-indolo[1,2-a]indol-10-one [**F15**]: Compound **F15** was prepared according to the general procedure H (0.2 mmol scale).

Yield: 52% (27.8 mg isolated).

Eluent: Petroleum ether/Ethyl acetate (98/2, v/v).

TLC: R_f = 0.6 (90:10 petroleum ether:EtOAc).

Physical State: Yellow oil.

^{1}H NMR (500 MHz, Chloroform-*d*) δ 7.48 (dt, *J* = 1.9, 0.8 Hz, 1H), 7.43 (dd, *J* = 8.3, 1.9 Hz, 1H), 7.29 (d, *J* = 8.3 Hz, 1H), 7.23 (td, *J* = 8.1, 5.6 Hz, 1H), 7.09 (d, *J* = 7.8 Hz, 1H), 6.77 – 6.70 (m, 1H), 3.32 (d, *J* = 16.6 Hz, 1H), 3.02 (d, *J* = 16.6 Hz, 1H), 2.34 (s, 3H), 1.54 (s, 3H) ppm.

^{13}C NMR (126 MHz, $CDCl_3$) δ 204.63, <u>160.33, 158.35</u> [159.34 (d, *J* = 248.1 Hz)], 158.11, <u>148.69, 148.63</u> [148.66 (d, *J* = 7.5 Hz)], 138.70, 132.63, <u>129.94, 129.88</u> [129.91 (d, *J* = 8.2 Hz)], 124.97, 124.62, <u>119.30, 119.14</u> [119.22 (d, *J* = 20.1 Hz)], 114.60, <u>111.35, 111.32</u> [111.33 (d, *J* = 3.2 Hz)], <u>110.66, 110.50</u> [110.58 (d, *J* = 20.5 Hz)], 76.37, 34.27, 24.41, 20.82 ppm.

135-DEPT NMR (126 MHz, $CDCl_3$) δ 138.56, <u>129.81, 129.74</u> [129.77 (d, *J* = 8.1 Hz)], 124.83, 114.46, <u>111.21, 111.18</u> [111.19 (d, *J* = 3.2 Hz)], 110.52, 110.36 [110.44 (d, *J* = 20.6 Hz)], 34.13, 24.27, 20.68 ppm.

^{19}F NMR (470 MHz, Chloroform-*d*) δ -116.23 (dd, *J* = 8.6, 5.7 Hz) ppm.

HRMS (m/z): [M + H$^+$] calcd for $C_{17}H_{15}FNO$, 268.1138; found, 268.1143.

Compound **F15** was resynthesized (**F15-resynthesized**) and retested in biological assays. The ^{1}H NMR spectra of the resynthesized batch is analogous to the original batch.

^{1}H NMR (400 MHz, Chloroform-*d*) δ 7.50 – 7.47 (m, 1H), 7.43 (dd, *J* = 8.3, 1.9 Hz, 1H), 7.29 (d, *J* = 8.3 Hz, 1H), 7.23 (dd, *J* = 8.1, 5.6 Hz, 1H), 7.09 (d, *J* = 7.9 Hz, 1H), 6.74 (t, *J* = 8.4 Hz, 1H), 3.33 (d, *J* = 16.7 Hz, 1H), 3.03 (d, *J* = 16.7 Hz, 1H), 2.35 (s, 3H), 1.54 (s, 3H).

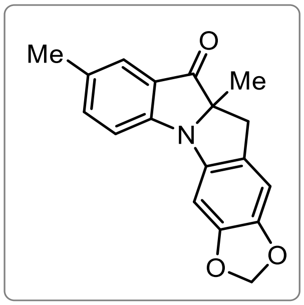

8,10a-dimethyl-10a,11-dihydro-10H-[1,3]dioxolo[4,5-f]indolo[1,2-a]indol-10-one [**F16**]: Compound **F16** was prepared according to the general procedure H (0.2 mmol scale).

Yield: 43% (25.2 mg isolated).

Eluent: Petroleum ether/Ethyl acetate (85/15, v/v).

TLC: R_f = 0.32 (80/20 petroleum ether:EtOAc).

Physical State: Yellow solid.

^{1}H NMR (500 MHz, Chloroform-*d*) δ 7.46 (dt, *J* = 1.7, 0.9 Hz, 1H), 7.42 – 7.38 (m, 1H), 7.22 (d, *J* = 8.3 Hz, 1H), 6.87 (s, 1H), 6.64 (s, 1H), 5.96 (d, *J* = 1.4 Hz, 1H), 5.92 (d, *J* = 1.4 Hz, 1H), 3.26 (dd, *J* = 16.0, 1.2 Hz, 1H), 2.83 (d, *J* = 16.0 Hz, 1H), 2.34 (s, 3H), 1.51 (s, 3H) ppm.

^{13}C NMR (126 MHz, $CDCl_3$) δ 205.26, 159.23, 147.54, 144.29, 140.08, 138.68, 132.15, 125.07, 124.94, 124.46, 114.35, 106.13, 101.59, 98.88, 76.95, 38.16, 23.88, 20.84 ppm.

HRMS (m/z): [M + H$^+$] calcd for $C_{18}H_{16}O_3N$, 294.11247; found, 294.11257.

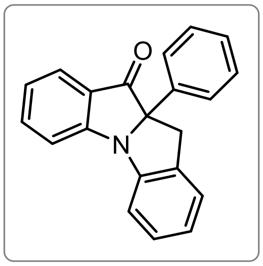

10a-phenyl-10a,11-dihydro-10H-indolo[1,2-a]indol-10-one [**F17**]: Compound **F17** was prepared according to the general procedure H (0.2 mmol scale).

Yield: 52% (30.9 mg).

Eluent: Petroleum ether/Ethyl acetate (97/3, v/v).

TLC: R_f = 0.55 (90:10 petroleum ether:EtOAc).

Physical State: Yellow oil.

^{1}H NMR (500 MHz, Chloroform-*d*) δ 7.79 – 7.73 (m, 2H), 7.62 (dtd, *J* = 7.2, 3.9, 1.4 Hz, 2H), 7.56 – 7.52 (m, 1H), 7.48 – 7.44 (m, 1H), 7.36 – 7.27 (m, 4H), 7.16 – 7.11 (m, 1H), 7.07 (td, *J* = 7.4, 0.9 Hz, 1H), 7.02 (td, *J* = 7.5, 1.0 Hz, 1H), 3.83 (d, *J* = 16.3 Hz, 1H), 3.47 (d, *J* = 16.3 Hz, 1H) ppm.

^{13}C NMR (126 MHz, $CDCl_3$) δ 202.59, 160.54, 145.94, 140.50, 137.51, 133.14, 128.82, 128.22, 128.14, 125.87, 125.77, 125.70, 124.68, 123.92, 122.72, 115.43, 114.50, 80.08, 40.86 ppm.

135-DEPT NMR (126 MHz, $CDCl_3$) δ 137.32, 128.63, 128.03, 127.95, 125.68, 125.58, 125.51, 123.73, 122.53, 115.24, 114.31, 40.68 ppm.

HRMS (m/z): [M + H^+] calcd for $C_{21}H_{16}ON$, 298.12264; found, 298.12276.

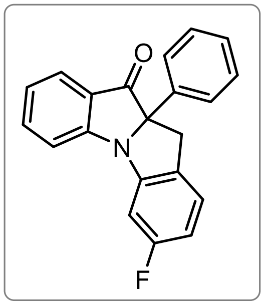

3-fluoro-10a-phenyl-10a,11-dihydro-10H-indolo[1,2-a]indol-10-one [**F18**]: Compound **F18** was prepared according to the general procedure H (0.2 mmol scale).

Yield: 61% (38.5 mg).

Eluent: Petroleum ether/Ethyl acetate (97/3, v/v).

TLC: R_f = 0.56 (90:10 petroleum ether:EtOAc).

Physical State: Yellow oil.

^{1}H NMR (500 MHz, Chloroform-*d*) δ 7.74 – 7.70 (m, 2H), 7.68 – 7.62 (m, 2H), 7.50 (dt, *J* = 8.2, 0.8 Hz, 1H), 7.37 – 7.31 (m, 2H), 7.29 – 7.26 (m, 1H), 7.15 (dd, *J* = 9.2, 2.4 Hz, 1H), 7.11 (td, *J* = 7.5, 0.8 Hz, 1H), 7.05 (ddt, *J* = 8.0, 5.6, 1.1 Hz, 1H), 6.71 (ddd, *J* = 9.1, 8.3, 2.3 Hz, 1H), 3.78 (dt, *J* = 16.1, 1.7 Hz, 1H), 3.43 (dt, *J* = 16.1, 1.2 Hz, 1H) ppm.

^{13}C NMR (126 MHz, $CDCl_3$) δ 202.20, 164.04, 162.09 [163.07 (d, *J* = 244.2 Hz)], 159.76, 147.18, 147.09 [147.13 (d, *J* = 11.3 Hz)], 140.25, 137.60, 128.90, 128.29, 128.27, 126.21, 126.13 [126.17 (d, *J* = 9.9 Hz)], 125.98, 125.62, 124.82, 123.22, 114.57, 110.41, 110.23 [110.32 (d, *J* = 22.5 Hz)], 103.47, 103.26 [103.36 (d, *J* = 26.2 Hz)], 80.96, 40.14 ppm.

^{19}F NMR (470 MHz, Chloroform-*d*) δ -113.79 (td, *J* = 9.0, 4.8 Hz) ppm.

HRMS (m/z): [M + H^+] calcd for $C_{21}H_{15}ONF$, 316.11322; found, 316.11298.

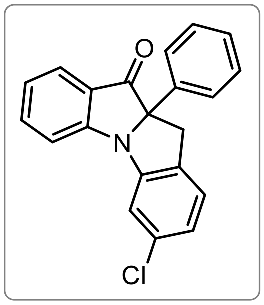

3-chloro-10a-phenyl-10a,11-dihydro-10H-indolo[1,2-a]indol-10-one [**F19**]: Compound **F19** was prepared according to the general procedure H (0.2 mmol scale).

Yield: 48% (31.9 mg).

Eluent: Petroleum ether/Ethyl acetate (97/3, v/v).

TLC: R_f = 0.56 (90:10 petroleum ether:EtOAc).

Physical State: Yellow oil.

^{1}H NMR (500 MHz, Chloroform-*d*) δ 7.77 – 7.70 (m, 2H), 7.70 – 7.61 (m, 2H), 7.52 (dt, *J* = 8.2, 0.9 Hz, 1H), 7.43 (d, *J* = 1.8 Hz, 1H), 7.37 – 7.30 (m, 2H), 7.30 – 7.25 (m, 1H), 7.11 (ddd, *J* = 7.8, 7.3, 0.8 Hz, 1H), 7.04 (dt, *J* = 7.9, 1.2 Hz, 1H), 6.99 (dd, *J* = 7.9, 1.8 Hz, 1H), 3.79 (dd, *J* = 16.4, 1.3 Hz, 1H), 3.43 (d, *J* = 16.4 Hz, 1H) ppm.

^{13}C NMR (126 MHz, $CDCl_3$) δ 202.07, 159.69, 147.08, 140.14, 137.65, 133.68, 131.55, 128.91, 128.30, 126.35, 125.97, 125.61, 124.77, 123.81, 123.25, 115.64, 114.60, 80.56, 40.31 ppm.

HRMS (m/z): [M + H^+] calcd for $C_{21}H_{15}ONCl$, 332.08367; found, 332.08392.

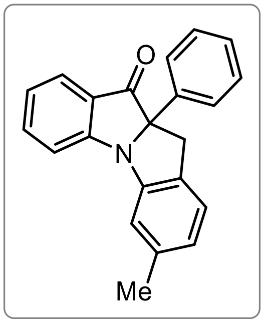

3-methyl-10a-phenyl-10a,11-dihydro-10H-indolo[1,2-a]indol-10-one [**F20**]: Compound **F20** was prepared according to the general procedure H (0.2 mmol scale).

Yield: 65% (40.5 mg isolated).

Eluent: Petroleum ether/Ethyl acetate (97/3, v/v).

TLC: R_f = 0.6 (90:10 petroleum ether:EtOAc).

Physical State: Yellow oil.

^{1}H NMR (500 MHz, Chloroform-*d*) δ 7.77 – 7.72 (m, 2H), 7.66 – 7.60 (m, 2H), 7.54 (dt, *J* = 8.0, 1.0 Hz, 1H), 7.35 – 7.29 (m, 2H), 7.27 (dd, *J* = 4.5, 1.4 Hz, 1H), 7.06 (td, *J* = 7.4, 0.9 Hz, 1H), 7.01 (d, *J* = 7.6 Hz, 1H), 6.83 (ddd, *J* = 7.5, 1.5, 0.8 Hz, 1H), 3.77 (d, *J* = 16.1 Hz, 1H), 3.42 (d, *J* = 16.1 Hz, 1H), 2.42 (s, 3H) ppm.

^{13}C NMR (126 MHz, $CDCl_3$) δ 202.68, 160.53, 146.04, 140.59, 138.23, 137.45, 130.07, 128.79, 128.08, 125.82, 125.76, 125.29, 124.65, 124.64, 122.60, 116.22, 114.51, 80.40, 77.48, 77.23, 76.98, 40.59, 21.89 ppm.

135-DEPT NMR (126 MHz, $CDCl_3$) δ 137.26, 128.60, 127.89, 125.63, 125.57, 125.10, 124.45, 122.41, 116.04, 114.32, 40.40, 21.71 ppm.

HRMS (m/z): [M + H^+] calcd for $C_{22}H_{18}ON$, 312.13829; found, 312.13843.

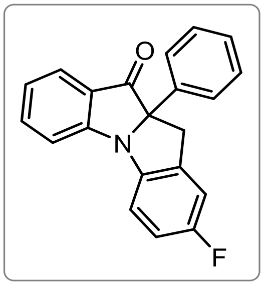

2-fluoro-10a-phenyl-10a,11-dihydro-10H-indolo[1,2-a]indol-10-one [**F21**]: Compound **F21** was prepared according to the general procedure H (0.2 mmol scale).

Yield: 60% (37.8 mg isolated).

Eluent: Petroleum ether/Ethyl acetate (97/3, v/v).

TLC: R_f = 0.56 (90:10 petroleum ether:EtOAc).

Physical State: Yellow oil.

^{1}H NMR (500 MHz, Chloroform-*d*) δ 7.78 – 7.72 (m, 2H), 7.66 – 7.60 (m, 2H), 7.48 (dd, *J* = 8.7, 0.8 Hz, 1H), 7.38 (dd, *J* = 8.6, 4.4 Hz, 1H), 7.36 – 7.31 (m, 2H), 7.30 – 7.26 (m, 1H), 7.08 (td, *J* = 7.4, 0.8 Hz, 1H), 7.01 – 6.94 (m, 1H), 6.85 (ddt, *J* = 8.2, 2.5, 1.1 Hz, 1H), 3.81 (dq, *J* = 16.6, 1.2 Hz, 1H), 3.45 (d, *J* = 16.5 Hz, 1H) ppm.

^{13}C NMR (126 MHz, $CDCl_3$) δ 202.14, 160.61, <u>160.86, 158.93</u> [159.90 (d, *J* = 242.1 Hz)], <u>142.13, 142.11</u> [142.12 (d, *J* = 2.2 Hz)], 140.14, 137.61, <u>135.17, 135.10</u> [135.13 (d, *J* = 8.6 Hz)], 128.88, 128.27, 125.96, 125.73, 124.46, 122.87, <u>116.13, 116.06</u> [116.09 (d, *J* = 8.7 Hz)], <u>114.76, 114.57</u> [114.67 (d, *J* = 23.8 Hz)], 114.30, <u>113.16, 112.97</u> [113.06 (d, *J* = 24.1 Hz)], 80.62, <u>41.02, 41.01</u> [41.01 (d, *J* = 1.9 Hz)] ppm.

^{19}F NMR (470 MHz, Chloroform-*d*) δ -119.52 (td, *J* = 8.5, 4.3 Hz) ppm.

HRMS (m/z): [M + H^+] calcd for $C_{21}H_{15}ONF$, 316.11322; found, 316.11344.

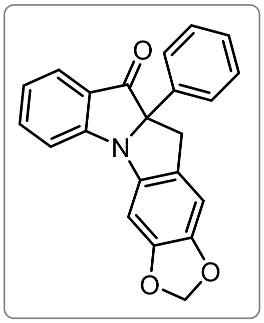

10a-phenyl-10a,11-dihydro-10H-[1,3]dioxolo[4,5-f]indolo[1,2-a]indol-10-one [**F22**]: Compound **F22** was prepared according to the general procedure H (0.2 mmol scale).

Yield: 55% (37.6 mg isolated).

Eluent: Petroleum ether/Ethyl acetate (85/15, v/v).

TLC: R_f = 0.3 (80:20 petroleum ether:EtOAc).

Physical State: Yellow oil.

^{1}H NMR (700 MHz, Chloroform-*d*) δ 8.75 (d, *J* = 8.3 Hz, 1H), 7.77 (ddd, *J* = 8.5, 7.2, 1.4 Hz, 1H), 7.73 (dd, *J* = 7.7, 1.3 Hz, 1H), 7.52 (s, 1H), 7.44 – 7.39 (m, 2H), 7.26 – 7.21 (m, 3H), 7.20 – 7.17 (m, 1H), 6.63 (s, 1H), 5.98 (d, *J* = 1.5 Hz, 1H), 5.94 (d, *J* = 1.5 Hz, 1H), 3.70 (d, *J* = 15.8 Hz, 1H), 3.37 (dd, *J* = 15.8, 1.0 Hz, 1H) ppm.

^{13}C NMR (176 MHz, $CDCl_3$) δ 197.72, 161.97, 152.03, 151.85, 147.70, 137.91, 136.05, 132.33, 129.09, 128.52, 125.64, 125.25, 124.95, 124.00, 122.89, 117.92, 108.44, 102.09, 72.06, 35.93 ppm.

HRMS (m/z): [M + H^+] calcd for $C_{22}H_{16}O_3N$, 342.11247; found, 342.11255.

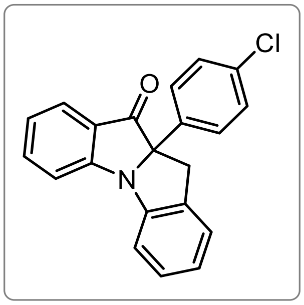

10a-(4-chlorophenyl)-10a,11-dihydro-10H-indolo[1,2-a]indol-10-one [**F23**]: Compound **F23** was prepared according to the general procedure H (0.2 mmol scale).

Yield: 43% (28.5 mg isolated).

Eluent: Petroleum ether/Ethyl acetate (97/3, v/v).

TLC: R_f = 0.55 (90:10 petroleum ether:EtOAc).

Physical State: Yellow oil.

^{1}H NMR (700 MHz, Chloroform-*d*) δ 7.72 – 7.68 (m, 2H), 7.65 – 7.61 (m, 2H), 7.55 – 7.52 (m, 1H), 7.46 (d, *J* = 7.7 Hz, 1H), 7.32 – 7.27 (m, 3H), 7.14 (d, *J* = 7.4 Hz, 1H), 7.10 – 7.07 (m, 1H), 7.03 (td, *J* = 7.5, 1.0 Hz, 1H), 3.81 (dt, *J* = 16.2, 1.2 Hz, 1H), 3.39 (d, *J* = 16.2 Hz, 1H) ppm.

^{13}C NMR (176 MHz, $CDCl_3$) δ 202.03, 160.53, 145.84, 139.08, 137.67, 134.13, 132.85, 128.95, 128.37, 127.29, 125.91, 125.74, 124.51, 124.17, 122.93, 115.60, 114.58, 79.59, 40.99 ppm.

HRMS (m/z): [M + H^+] calcd for $C_{21}H_{15}ONCl$, 332.08367; found, 332.08374.

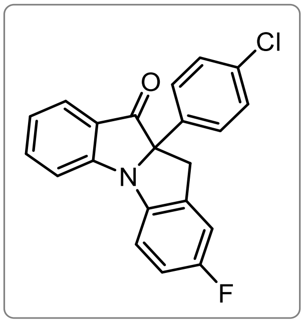

10a-(4-chlorophenyl)-2-fluoro-10a,11-dihydro-10H-indolo[1,2-a]indol-10-one [**F24**]: Compound **F24** was prepared according to the general procedure H (0.2 mmol scale).

Yield: 48% (33.6 mg isolated).

Eluent: Petroleum ether/Ethyl acetate (97/3, v/v).

TLC: R_f = 0.6 (90:10 petroleum ether:EtOAc).

Physical State: Yellow oil.

^{1}H NMR (500 MHz, Chloroform-*d*) δ 7.70 – 7.66 (m, 2H), 7.66 – 7.61 (m, 2H), 7.49 – 7.45 (m, 1H), 7.38 (dd, *J* = 8.6, 4.4 Hz, 1H), 7.33 – 7.27 (m, 2H), 7.10 (td, *J* = 7.4, 0.8 Hz, 1H), 6.99 (tdd, *J* = 8.7, 2.1, 0.8 Hz, 1H), 6.85 (ddd, *J* = 8.0, 2.4, 1.2 Hz, 1H), 3.80 (dq, *J* = 16.5, 1.3 Hz, 1H), 3.37 (dt, *J* = 16.6, 0.7 Hz, 1H) ppm.

^{13}C NMR (126 MHz, $CDCl_3$) δ 201.61, 160.58, 160.98, 159.05 [160.02 (d, *J* = 242.5 Hz)], 141.99, 141.97 [141.98 (d, *J* = 2.2 Hz)], 138.68, 137.80, 134.89, 134.82 [134.86 (d, *J* = 8.7 Hz)], 134.28, 129.01, 127.25, 126.01, 124.26, 123.09, 116.34, 116.27 [116.30 (d, *J* = 8.7 Hz)], 114.94, 114.76 [114.85 (d, *J* = 23.6 Hz)], 114.37, 113.21, 113.02 [113.11 (d, *J* = 24.1 Hz)], 80.14, 41.14, 41.12 [41.13 (d, *J* = 1.8 Hz)] ppm.

^{19}F NMR (470 MHz, Chloroform-*d*) δ -119.11 (td, *J* = 8.4, 4.3 Hz) ppm.

HRMS (m/z): [M + H^+] calcd for $C_{21}H_{14}ClFNO$, 350.0748; found, 350.0743.

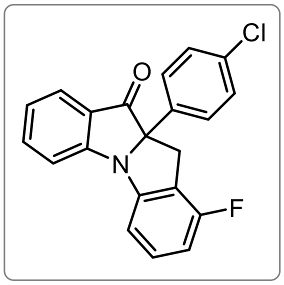

10a-(4-chlorophenyl)-1-fluoro-10a,11-dihydro-10H-indolo[1,2-a]indol-10-one [**F25**]: Compound **F25** was prepared according to the general procedure H (0.2 mmol scale).

Yield: 45% (31.5 mg isolated).

Eluent: Petroleum ether/Ethyl acetate (97/3, v/v).

TLC: R_f = 0.6 (90:10 petroleum ether:EtOAc).

Physical State: Yellow oil.

^{1}H NMR (500 MHz, Chloroform-*d*) δ 7.71 – 7.67 (m, 2H), 7.67 – 7.62 (m, 2H), 7.52 (dt, *J* = 8.1, 0.8 Hz, 1H), 7.32 – 7.29 (m, 2H), 7.29 – 7.26 (m, 1H), 7.24 (dd, *J* = 7.9, 1.0 Hz, 1H), 7.12 (ddd, *J*

= 7.8, 7.2, 0.8 Hz, 1H), 6.75 (td, *J* = 8.3, 1.1 Hz, 1H), 3.79 (dt, *J* = 16.5, 1.1 Hz, 1H), 3.46 (d, *J* = 16.5 Hz, 1H) ppm.

^{13}C NMR (126 MHz, CDCl$_3$) δ 201.43, 159.98, 160.13, 158.15 [159.14 (d, *J* = 248.6 Hz)], 148.33, 148.28 [148.30 (d, *J* = 7.3 Hz)], 138.67, 137.77, 134.34, 130.25, 130.19 [130.22 (d, *J* = 8.2 Hz)], 129.05, 127.18, 126.01, 124.51, 123.41, 119.01, 118.85 [118.93 (d, *J* = 20.2 Hz)], 114.77, 111.29, 111.19 [111.24 (d, *J* = 12.5 Hz)], 111.16, 111.13 [111.14 (d, *J* = 4.5 Hz)], 79.99, 37.28 ppm.

^{19}F NMR (470 MHz, Chloroform-*d*) δ -115.92 (dd, *J* = 8.5, 5.3 Hz) ppm.

HRMS (m/z): [M + H$^+$] calcd for $C_{21}H_{14}ONClF$, 350.07425; found, 350.07453.

Synthesis and characterization data of F26-F29

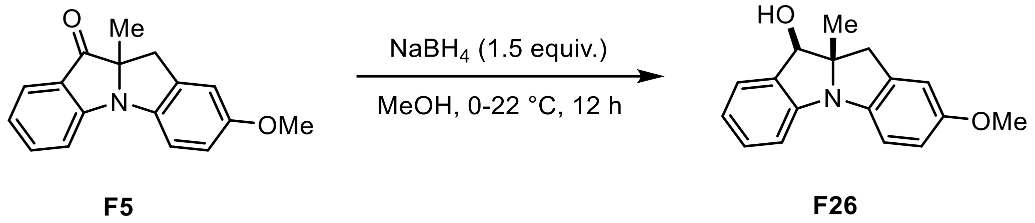

(10R,10aS)-2-methoxy-10a-methyl-10a,11-dihydro-10H-indolo[1,2-a]indol-10-ol **[F26]:** An oven-dried screw capped reaction tube with a magnetic stir-bar was charged with F5 (53 mg, 0.2 mmol). The reaction tube was kept on an ice bath and methanol (3 mL) was added. Then, sodium borohydride (1.5 equiv.) was added under Argon atm. After stirring for 2 hours at 0 °C, the reaction mixture was allowed to warm to room temperature and the stirring was continued for an additional 10 hours. Next, the reaction mixture was quenched with water (10 mL) and concentrated in a rotary evaporator. The organic compound was extracted with ethyl acetate (15 mL) three times. Then, the organic phase was collected together, and ethyl acetate was evaporated. The crude reaction mixture was purified through a silica gel column.

Yield: 62% (33 mg) [d.r., >20:1; *cis*- conformation was confirmed by 2D NOESY].

Eluent: Petroleum ether/Ethyl acetate (70/30, v/v).

TLC: R_f = 0.35 (60:40 petroleum ether:EtOAc).

Physical State: Brown sticky oil.

^{1}H NMR (400 MHz, Chloroform-*d*) δ 7.24 (d, *J* = 7.4 Hz, 1H), 7.20 – 7.13 (m, 2H), 7.08 (d, *J* = 7.8 Hz, 1H), 6.91 (td, *J* = 7.4, 1.0 Hz, 1H), 6.80 (d, *J* = 2.6 Hz, 1H), 6.72 (dd, *J* = 8.5, 2.7 Hz, 1H), 5.17 (s, 1H), 3.69 (s, 3H), 3.16 (d, *J* = 16.3 Hz, 1H), 2.85 (d, *J* = 16.3 Hz, 1H), 1.25 (s, 3H).

^{13}C NMR (101 MHz, CDCl$_3$) δ 155.29, 148.49, 141.29, 134.52, 133.59, 128.93, 125.55, 121.35, 114.57, 112.74, 112.33, 111.33, 78.65, 76.04, 55.36, 21.60.

HRMS (m/z): [M + H$^+$] calcd for $C_{17}H_{18}O_2N$ 268.13321; found, 268.13228.

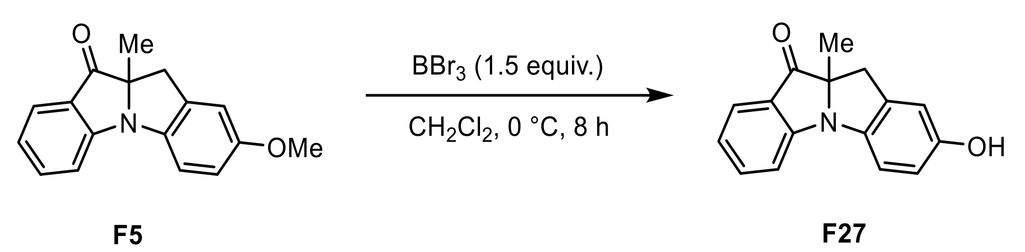

2-hydroxy-10a-methyl-10a,11-dihydro-10H-indolo[1,2-a]indol-10-one **[F27]:** An oven-dried screw capped reaction tube with a magnetic stir-bar was charged with F5 (53 mg, 0.2 mmol). The reaction tube was evacuated and refilled with argon. The reaction tube was kept on an ice bath and dry CH_2Cl_2 (4 mL) was added. Then, BBr_3 (1.5 equiv.) was added dropwise. After stirring for 8 hours at 0 °C, the reaction mixture was quenched with water (10 mL) and concentrated in a rotary evaporator. The organic compound was extracted with CH_2Cl_2 (15 mL) three times. Then, the organic phase was collected together and evaporated. The crude reaction mixture was purified through a silica gel column.

Yield: 99% (50 mg).

Eluent: Petroleum ether/Ethyl acetate (75/25, v/v).

TLC: R_f = 0.4 (70:30 petroleum ether:EtOAc).

Physical State: Yellow semi-solid.

^{1}H NMR (500 MHz, Chloroform-*d*) δ 7.67 (d, *J* = 7.0 Hz, 1H), 7.58 (ddd, *J* = 8.4, 7.2, 1.4 Hz, 1H), 7.32 (d, *J* = 8.3 Hz, 1H), 7.20 (d, *J* = 8.4 Hz, 1H), 7.04 (t, *J* = 7.5 Hz, 1H), 6.77 (dd, *J* = 8.3, 2.6 Hz, 1H), 6.72 (q, *J* = 1.2 Hz, 1H), 5.30 (s, 1H), 3.30 (d, *J* = 16.4 Hz, 1H), 2.90 (d, *J* = 16.4 Hz, 1H), 1.53 (s, 3H).

^{13}C NMR (126 MHz, Chloroform-*d*) δ 205.49, 161.15, 152.83, 139.21, 137.63, 135.28, 125.46, 124.17, 122.15, 116.87, 114.70, 114.33, 113.25, 76.27, 38.32, 23.66.

HRMS (m/z): [M + H^+] calcd for $C_{16}H_{14}O_2N$ 252.10191; found, 252.10122.

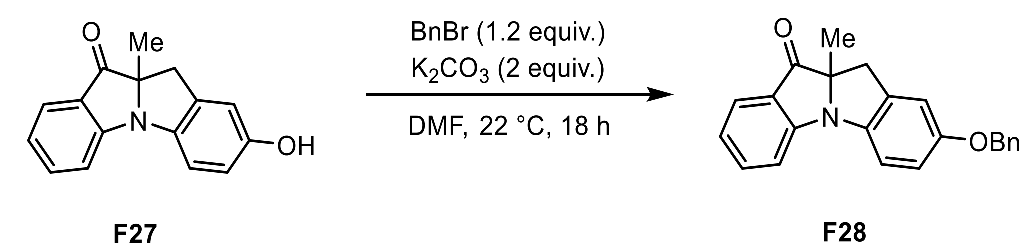

***2-(benzyloxy)-10a-methyl-10a,11-dihydro-10H-indolo[1,2-a]indol-10-one* [F28]:** An oven-dried screw capped reaction tube with a magnetic stir-bar was charged with F27 (37.5 mg, 0.15 mmol) and potassium carbonate (2 equiv.). The reaction tube was evacuated and refilled with argon. 4 mL of DMF was added. Then, benzyl bromide (BnBr) (1.2 equiv.) was added dropwise. After stirring for 18 hours at 22 °C, the reaction mixture was quenched with water (15 mL) and ethyl acetate (7 mL). Then, the organic phase was collected and evaporated. The crude reaction mixture was purified through a silica gel column.

Yield: 70% (35.8 mg).

Eluent: Petroleum ether/Ethyl acetate (90/10, v/v).

TLC: R_f = 0.35 (85:15 petroleum ether:EtOAc).

Physical State: Brownish oil.

^{1}H NMR (400 MHz, Chloroform-*d*) δ 7.67 (dt, *J* = 7.7, 1.0 Hz, 1H), 7.57 (ddd, *J* = 8.4, 7.2, 1.4 Hz, 1H), 7.45 – 7.36 (m, 4H), 7.35 – 7.29 (m, 2H), 7.25 (d, *J* = 8.5 Hz, 1H), 7.08 – 7.01 (m, 1H), 6.89 (dd, *J* = 8.5, 2.6 Hz, 1H), 6.85 – 6.82 (m, 1H), 5.03 (s, 2H), 3.33 (d, *J* = 16.4 Hz, 1H), 2.92 (d, *J* = 16.5 Hz, 1H), 1.53 (s, 3H).

^{13}C NMR (101 MHz, $CDCl_3$) δ 205.08, 160.99, 156.04, 139.61, 137.45, 137.21, 135.06, 128.82, 128.20, 127.62, 125.39, 124.33, 122.13, 116.68, 114.43, 114.34, 112.94, 76.20, 70.83, 38.45, 23.75.

HRMS (m/z): [M + H^+] calcd for $C_{23}H_{20}O_2N$ 342.14886; found, 342.14859.

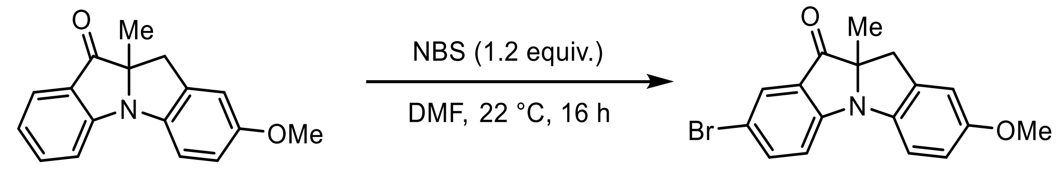

F5 **F29**

***2-(benzyloxy)-10a-methyl-10a,11-dihydro-10H-indolo[1,2-a]indol-10-one* [F29]:** An oven-dried screw capped reaction tube with a magnetic stir-bar was charged with F5 (53 mg, 0.2 mmol). 4 mL of DMF was added. Then, *N*-bromo succinimide (NBS) (1.2 equiv.) was added portion-wise. After stirring for 16 hours at 22 °C, the reaction mixture was quenched with water (15 mL) and ethyl acetate (7 mL). Then, the organic phase was collected and evaporated. The crude reaction mixture was purified through a silica gel column.

Yield: 73% (50.3 mg).

Eluent: Petroleum ether/Ethyl acetate (90/10, v/v).

TLC: R_f = 0.38 (80:20 petroleum ether:EtOAc).

Physical State: Brown solid.

^{1}H NMR (400 MHz, Chloroform-*d*) δ 7.77 (d, *J* = 2.1 Hz, 1H), 7.67 – 7.61 (m, 1H), 7.21 (dd, *J* = 8.6, 1.3 Hz, 1H), 6.81 (dd, *J* = 8.5, 2.6 Hz, 1H), 6.78 – 6.75 (m, 1H), 3.77 (s, 3H), 3.32 (d, *J* = 16.4 Hz, 1H), 2.91 (d, *J* = 16.4 Hz, 1H), 1.51 (s, 3H).

^{13}C NMR (101 MHz, Chloroform-*d*) δ 203.59, 159.60, 157.10, 139.90, 138.75, 134.94, 127.91, 125.97, 116.69, 115.84, 114.62, 113.45, 112.00, 76.84, 55.97, 38.41, 23.61.

HRMS (m/z): [M + H^{+}] calcd for $C_{17}H_{15}O_{2}NBr$ 344.02807 and 346.02602; found, 344.02790 and 346.02573.

CLASS-G

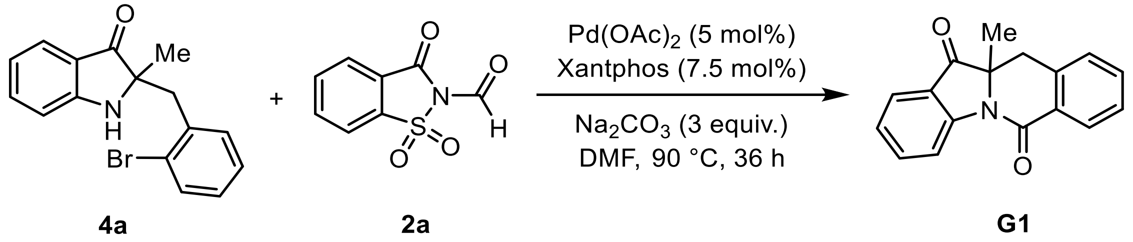

General procedure I: Synthesis of Class G

An oven-dried screw capped reaction tube (pictorial description in below) with a magnetic stir-bar was charged with 2-(2-bromobenzyl)-2-methylindolin-3-one (0.2 mmol), $Pd(OAc)_2$ (5 mol%, 2.3 mg), Xantphos (7.5 mol%, 8.7 mg), *N*-formyl saccharin (**2a**, 2 equiv., 0.4 mmol, 84.5 mg) and Na_2CO_3 (3 equiv., 63.6 mg). A screw cap fitted with a rubber septum was attached to the reaction tube, then degassed and refilled with argon; the process was repeated an additional two times. Dry *N*,*N*-dimethyl formamide (DMF, 2 mL) was added in the reaction tube, placed in metal block, and vigorously stirred at room temperature for 10 min followed by heated to 90 °C. The reaction mixture was taken out after 36 h, diluted with 10 mL of ethyl acetate and 25 mL of water. The organic layer was separated and concentrated under vacuum. The crude mixture was purified by column chromatography using silica gel (100-200 mesh size) and petroleum ether/ethyl acetate as the eluent.

Characterization data for Class G products

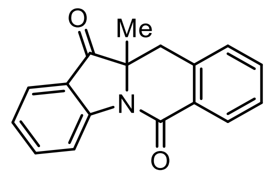

11a-methyl-11,11a-dihydroindolo[1,2-b]isoquinoline-6,12-dione [**G1**]: Compound **G1** was prepared according to the general procedure I (0.2 mmol scale).

Yield: 68% (35.8 mg isolated).

Eluent: Petroleum ether/Ethyl acetate (94/6, v/v).

TLC: R_f = 0.52 (80:20 petroleum ether:EtOAc).

Physical State: Off-white solid.

^{1}H NMR (400 MHz, Chloroform-*d*) δ 8.63 (dq, *J* = 8.3, 0.7 Hz, 1H), 8.17 (dt, *J* = 7.7, 1.0 Hz, 1H), 7.82 (ddt, *J* = 7.7, 1.3, 0.6 Hz, 1H), 7.78 – 7.70 (m, 1H), 7.52 (td, *J* = 7.5, 1.5 Hz, 1H), 7.43

(tdd, J = 7.5, 1.3, 0.6 Hz, 1H), 7.32 (ddt, J = 7.6, 1.6, 0.8 Hz, 1H), 7.30 – 7.24 (m, 1H), 3.18 – 3.05 (m, 2H), 1.37 (s, 3H) ppm.

^{13}C NMR (101 MHz, cdcl$_3$) δ 200.91, 161.50, 151.15, 137.80, 136.85, 133.17, 129.50, 129.08, 129.03, 127.98, 124.77, 124.69, 123.06, 118.37, 67.00, 34.53, 22.31 ppm.

HRMS (m/z): [M + H$^+$] calcd for $C_{17}H_{14}O_2N$, 264.10191; found, 264.10193.

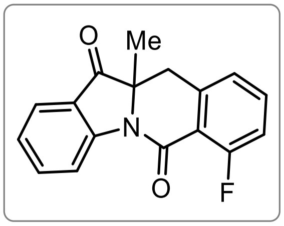

7-fluoro-11a-methyl-11,11a-dihydroindolo[1,2-b]isoquinoline-6,12-dione [**G2**]: Compound **G2** was prepared according to the general procedure I (0.2 mmol scale).

Yield: 51% (28.7 mg).

Eluent: Petroleum ether/Ethyl acetate (95/5, v/v).

TLC: R_f = 0.54 (80:20 petroleum ether:EtOAc).

Physical State: Yellow solid.

^{1}H NMR (700 MHz, Chloroform-*d*) δ 8.67 (dt, J = 8.3, 0.8 Hz, 1H), 7.85 (ddd, J = 7.6, 1.4, 0.7 Hz, 1H), 7.77 (ddd, J = 8.4, 7.2, 1.4 Hz, 1H), 7.53 – 7.48 (m, 1H), 7.31 (ddd, J = 8.0, 7.3, 0.9 Hz, 1H), 7.19 – 7.12 (m, 2H), 3.17 – 3.06 (m, 2H), 1.38 (s, 3H) ppm.

^{13}C NMR (176 MHz, CDCl$_3$) δ 200.53, 163.90, 162.40 [163.15 (d, J = 263.8 Hz)], 158.59, 158.58 [158.59 (d, J = 3.0 Hz)], 151.12, 139.66, 138.03, 134.52, 134.46 [134.49 (d, J = 10.0 Hz)], 125.10, 124.88, 124.86 [124.87 (d, J = 3.7 Hz)], 124.73, 123.15, 118.89, 117.83, 117.79 [117.81 (d, J = 6.0 Hz)], 116.86, 116.74 [116.80 (d, J = 21.8 Hz)], 66.91, 35.17, 35.16 [35.17 (d, J = 2.2 Hz)], 21.77 ppm.

135-DEPT NMR (176 MHz, CDCl$_3$) δ 137.82, 134.30, 134.25 [134.28 (d, J = 9.9 Hz)], 124.88, 124.67, 124.64 [124.66 (d, J = 3.8 Hz)], 124.52, 118.68, 116.65, 116.53 [116.59 (d, J = 21.9 Hz)], 34.96, 34.95 [34.96 (d, J = 2.1 Hz)], 21.56 ppm.

^{19}F NMR (470 MHz, Chloroform-*d*) δ -111.67 (dd, J = 9.3, 3.9 Hz) ppm.

HRMS (m/z): [M + H$^+$] calcd for $C_{17}H_{13}O_2NF$, 282.09248; found, 282.09257.

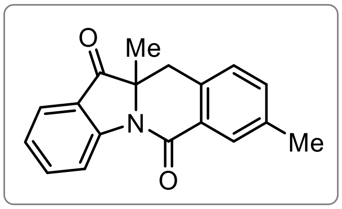

8,11a-dimethyl-11,11a-dihydroindolo[1,2-b]isoquinoline-6,12-dione [**G3**]: Compound **G3** was prepared according to the general procedure I (0.2 mmol scale).

Yield: 63% (35 mg).

Eluent: Petroleum ether/Ethyl acetate (95/5, v/v).

TLC: R_f = 0.55 (80:20 petroleum ether:EtOAc).

Physical State: Brown solid.

^{1}H NMR (500 MHz, Chloroform-*d*) δ 8.64 (dt, *J* = 8.4, 0.9 Hz, 1H), 8.01 – 7.98 (m, 1H), 7.84 (ddd, *J* = 7.8, 1.5, 0.7 Hz, 1H), 7.76 (ddd, *J* = 8.5, 7.3, 1.4 Hz, 1H), 7.34 (ddd, *J* = 7.7, 1.9, 0.8 Hz, 1H), 7.29 (td, *J* = 7.4, 0.9 Hz, 1H), 7.23 (d, *J* = 7.6 Hz, 1H), 3.15 – 3.03 (m, 2H), 2.43 (s, 3H), 1.38 (s, 3H) ppm.

^{13}C NMR (126 MHz, $CDCl_3$) δ 201.19, 161.85, 151.24, 137.90, 137.86, 134.03, 133.87, 129.48, 129.25, 128.97, 124.76, 124.72, 123.10, 118.42, 67.20, 34.15, 22.32, 21.32 ppm.

135-DEPT NMR (126 MHz, $CDCl_3$) δ 137.67, 133.84, 129.29, 128.78, 124.57, 124.53, 118.23, 33.96, 22.13, 21.13 ppm.

HRMS (m/z): [M + H^+] calcd for $C_{18}H_{16}O_2N$, 278.11756; found, 278.11762.

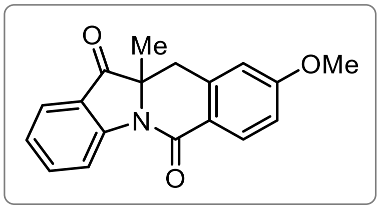

9-methoxy-11a-methyl-11,11a-dihydroindolo[1,2-b]isoquinoline-6,12-dione [**G4**]: Compound **G4** was prepared according to the general procedure I (0.2 mmol scale).

Yield: 66% (38.7 mg).

Eluent: Petroleum ether/Ethyl acetate (85/15, v/v).

TLC: R_f = 0.24 (80:20 petroleum ether:EtOAc).

Physical State: Brown solid.

^{1}H NMR (500 MHz, Chloroform-d) δ 8.62 (dd, J = 8.4, 0.9 Hz, 1H), 8.13 (d, J = 8.6 Hz, 1H), 7.83 (ddd, J = 7.7, 1.5, 0.7 Hz, 1H), 7.75 (ddd, J = 8.5, 7.2, 1.4 Hz, 1H), 7.30 – 7.25 (m, 1H), 6.94 (dd, J = 8.6, 2.5 Hz, 1H), 6.82 (d, J = 2.5 Hz, 1H), 3.90 (s, 3H), 3.15 – 3.02 (m, 2H), 1.39 (s, 3H)

^{13}C NMR (126 MHz, $CDCl_3$) δ 201.21, 163.54, 161.51, 151.44, 139.16, 137.89, 131.26, 124.71, 124.54, 122.91, 122.14, 118.24, 114.28, 113.38, 67.04, 55.77, 34.83, 22.36

HRMS (m/z): [M + H^+] calcd for $C_{18}H_{16}O_3N$, 294.11247; found, 294.11258.

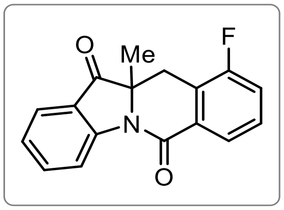

10-fluoro-11a-methyl-11,11a-dihydroindolo[1,2-b]isoquinoline-6,12-dione [**G5**]: Compound **G5** was prepared according to the general procedure I (0.2 mmol scale).

Yield: 53% (29.8 mg).

Eluent: Petroleum ether/Ethyl acetate (94/6, v/v).

TLC: R_f = 0.54 (80:20 petroleum ether:EtOAc).

Physical State: Yellow oil.

^{1}H NMR (700 MHz, Chloroform-d) δ 8.63 (dt, J = 8.3, 0.8 Hz, 1H), 8.02 (dd, J = 7.7, 1.1 Hz, 1H), 7.86 (ddd, J = 7.7, 1.4, 0.7 Hz, 1H), 7.77 (ddd, J = 8.5, 7.2, 1.4 Hz, 1H), 7.43 (tdd, J = 7.9, 5.2, 1.0 Hz, 1H), 7.35 – 7.29 (m, 2H), 3.49 (d, J = 16.2 Hz, 1H), 2.87 (d, J = 16.2 Hz, 1H), 1.41 (s, 3H)

^{13}C NMR (176 MHz, $CDCl_3$) δ 200.38, 160.48, 160.46 [160.47 (d, *J* = 3.3 Hz)], 161.15, 159.74 [160.44 (d, *J* = 247.6 Hz)], 150.97, 137.95, 131.57, 131.55 [131.56 (d, *J* = 3.6 Hz)], 128.96, 128.92 [128.94 (d, *J* = 7.8 Hz)], 125.12, 124.87, 124.77, 124.75 [124.76 (d, *J* = 3.3 Hz)], 123.98, 123.87 [123.93 (d, *J* = 18.6 Hz)], 123.04, 120.08, 119.96 [120.02 (d, *J* = 21.7 Hz)], 118.42, 66.64, 27.47, 27.45 [27.46 (d, *J* = 3.0 Hz)], 22.74

^{19}F NMR (470 MHz, Chloroform-*d*) δ -118.43 (dd, *J* = 8.8, 5.2 Hz)

HRMS (m/z): [M + H^+] calcd for $C_{17}H_{13}O_2NF$, 282.09248; found, 282.09257.

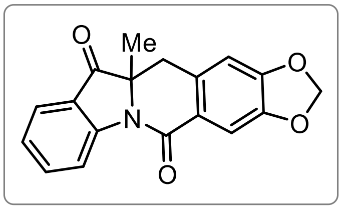

11a-methyl-11a,12-dihydro-[1,3]dioxolo[4,5-g]indolo[1,2-b]isoquinoline-5,11-dione [**G6**]: Compound **G6** was prepared according to the general procedure I (0.2 mmol scale).

Yield: 49% (30 mg).

Eluent: Petroleum ether/Ethyl acetate (80/20, v/v).

TLC: R_f = 0.36 (70:30 petroleum ether:EtOAc).

Physical State: Off-white solid.

^{1}H NMR (700 MHz, Chloroform-*d*) δ 8.61 (dt, *J* = 8.3, 0.8 Hz, 1H), 7.83 (ddd, *J* = 7.7, 1.5, 0.7 Hz, 1H), 7.75 (ddd, *J* = 8.5, 7.2, 1.4 Hz, 1H), 7.61 (s, 1H), 7.31 – 7.27 (m, 1H), 6.77 (d, *J* = 0.8 Hz, 1H), 6.07 (dd, *J* = 9.2, 1.5 Hz, 2H), 3.08 – 2.99 (m, 2H), 1.40 (s, 3H) ppm.

^{13}C NMR (176 MHz, $CDCl_3$) δ 201.07, 161.03, 151.89, 151.29, 147.80, 137.88, 132.87, 124.73, 124.64, 123.53, 123.01, 118.28, 108.92, 108.73, 102.20, 67.04, 34.56, 22.24 ppm.

HRMS (m/z): [M + H^+] calcd for $C_{18}H_{14}O_4N$, 308.09173; found, 308.09189.

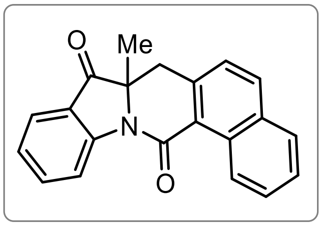

7a-methyl-7,7a-dihydrobenzo[h]indolo[1,2-b]isoquinoline-8,14-dione [**G7**]: Compound **G7** was prepared according to the general procedure I (0.2 mmol scale).

Yield: 47% (29.5 mg).

Eluent: Petroleum ether/Ethyl acetate (94/6, v/v).

TLC: R_f = 0.55 (80:20 petroleum ether:EtOAc).

Physical State: Yellow oil.

^{1}H NMR (700 MHz, Chloroform-*d*) δ 9.37 (dq, *J* = 8.7, 0.9 Hz, 1H), 8.77 (dt, *J* = 8.3, 0.8 Hz, 1H), 8.01 (d, *J* = 8.2 Hz, 1H), 7.92 – 7.85 (m, 2H), 7.80 (ddd, *J* = 8.4, 7.2, 1.4 Hz, 1H), 7.69 (ddd, *J* = 8.5, 6.8, 1.4 Hz, 1H), 7.56 (ddd, *J* = 8.0, 6.7, 1.1 Hz, 1H), 7.42 (d, *J* = 8.2 Hz, 1H), 7.34 – 7.30 (m, 1H), 3.31 (d, *J* = 15.8 Hz, 1H), 3.21 (d, *J* = 15.8 Hz, 1H), 1.40 (s, 3H) ppm.

^{13}C NMR (176 MHz, CDCl$_3$) δ 201.22, 162.27, 151.57, 138.09, 137.90, 134.02, 133.73, 132.15, 128.75, 128.67, 126.62, 126.54, 126.49, 124.81, 124.74, 124.48, 123.39, 119.02, 66.42, 36.09, 21.50 ppm.

HRMS (m/z): [M + H$^+$] calcd for $C_{21}H_{16}O_2N$, 314.11756; found, 314.11771.

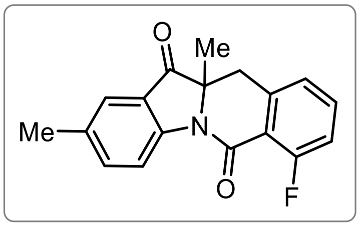

7-fluoro-2,11a-dimethyl-11,11a-dihydroindolo[1,2-b]isoquinoline-6,12-dione [**G8**]: Compound **G8** was prepared according to the general procedure I (0.2 mmol scale).

Yield: 55% (32.5 mg).

Eluent: Petroleum ether/Ethyl acetate (95/5, v/v).

TLC: R_f = 0.6 (80:20 petroleum ether:EtOAc).

Physical State: Brown solid.

^{1}H NMR (500 MHz, Chloroform-*d*) δ 8.54 (d, *J* = 8.5 Hz, 1H), 7.63 (dt, *J* = 1.9, 0.8 Hz, 1H), 7.59 (ddd, *J* = 8.5, 2.0, 0.7 Hz, 1H), 7.53 – 7.45 (m, 1H), 7.16 – 7.10 (m, 2H), 3.18 – 3.03 (m, 2H), 2.43 (s, 3H), 1.36 (s, 3H) ppm.

^{13}C NMR (126 MHz, CDCl$_3$) δ 200.58, <u>164.12, 162.03</u> [163.07 (d, *J* = 263.4 Hz)], <u>158.39, 158.36</u> [158.38 (d, *J* = 3.2 Hz)], 149.20, 139.67, 139.17, 135.19, <u>134.39, 134.31</u> [134.35 (d, *J* = 9.9 Hz)], <u>124.84, 124.81</u> [124.83 (d, *J* = 3.7 Hz)], 124.31, 123.25, 118.61, <u>117.86, 117.81</u> [117.84 (d, *J* = 6.2 Hz)], <u>116.83, 116.66</u> [116.74 (d, *J* = 21.8 Hz)], 67.09, <u>35.16, 35.14</u> [35.15 (d, *J* = 2.3 Hz)], 21.78, 21.16 ppm.

^{19}F NMR (470 MHz, Chloroform-*d*) δ -111.86 (dd, *J* = 11.2, 5.1 Hz) ppm.

HRMS (m/z): [M + H$^+$] calcd for $C_{18}H_{15}O_2NF$, 296.10813; found, 296.10802.

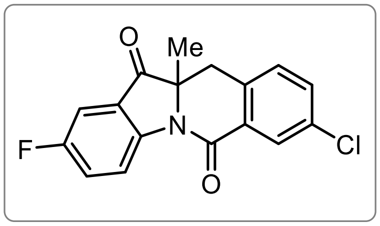

8-chloro-2-fluoro-11a-methyl-11,11a-dihydroindolo[1,2-b]isoquinoline-6,12-dione [**G9**]: Compound **G9** was prepared according to the general procedure I (0.2 mmol scale).

Yield: 54% (34.1 mg).

Eluent: Petroleum ether/Ethyl acetate (95/5, v/v).

TLC: R_f = 0.52 (80:20 petroleum ether:EtOAc).

Physical State: Yellow oil.

^{1}H NMR (500 MHz, Chloroform-*d*) δ 8.62 (dd, *J* = 9.7, 4.2 Hz, 1H), 8.14 (d, *J* = 2.2 Hz, 1H), 7.54 – 7.44 (m, 3H), 7.29 (d, *J* = 8.1 Hz, 1H), 3.17 – 3.03 (m, 2H), 1.38 (s, 3H) ppm.

^{13}C NMR (126 MHz, $CDCl_3$) δ 199.72, 199.70 [199.71 (d, *J* = 2.8 Hz)], 160.00, 160.82, 158.85 [159.83 (d, *J* = 247.7 Hz)], 147.25, 147.24 [147.25 (d, *J* = 1.4 Hz)], 134.94, 134.29, 133.23, 130.80, 130.44, 129.06, 125.42, 125.23 [125.33 (d, *J* = 24.2 Hz)], 124.44, 124.39 [124.41 (d, *J* = 7.4 Hz)], 120.04, 119.98 [120.01 (d, *J* = 7.4 Hz)], 110.41, 110.23 [110.32 (d, *J* = 23.3 Hz)], 67.67, 33.94, 22.42 ppm.

^{19}F NMR (470 MHz, Chloroform-*d*) δ -115.74 (td, *J* = 7.6, 4.1 Hz) ppm.

HRMS (m/z): [M + H^+] calcd for $C_{17}H_{12}O_2NClF$, 316.05351; found, 316.05371

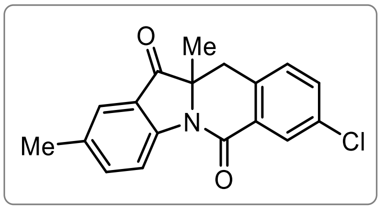

8-chloro-2,11a-dimethyl-11,11a-dihydroindolo[1,2-b]isoquinoline-6,12-dione [**G10**]: Compound **G10** was prepared according to the general procedure I (0.2 mmol scale).

Yield: 51% (31.8 mg).

Eluent: Petroleum ether/Ethyl acetate (95/5, v/v).

TLC: R_f = 0.6 (80:20 petroleum ether:EtOAc).

Physical State: Off-white solid.

^{1}H NMR (700 MHz, Chloroform-*d*) δ 8.50 (d, *J* = 8.4 Hz, 1H), 8.16 (d, *J* = 2.3 Hz, 1H), 7.64 (dt, *J* = 1.8, 0.8 Hz, 1H), 7.59 (ddd, *J* = 8.4, 2.0, 0.7 Hz, 1H), 7.50 (dd, *J* = 8.0, 2.3 Hz, 1H), 7.31 – 7.27 (m, 1H), 3.12 (d, *J* = 15.8 Hz, 1H), 3.06 (d, *J* = 15.8 Hz, 1H), 2.44 (s, 3H), 1.37 (s, 3H) ppm.

^{13}C NMR (176 MHz, $CDCl_3$) δ 200.54, 160.02, 149.01, 139.09, 135.30, 135.17, 134.20, 133.02, 131.20, 130.39, 129.04, 124.45, 123.24, 118.22, 67.20, 34.09, 22.43, 21.19 ppm.

HRMS (m/z): [M + H^+] calcd for $C_{18}H_{15}O_2NCl$, 312.07858; found, 312.07882.

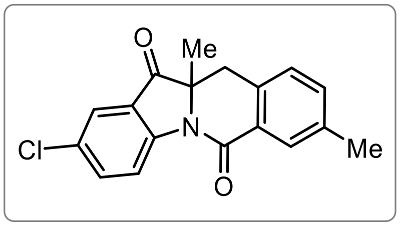

2-chloro-8,11a-dimethyl-11,11a-dihydroindolo[1,2-b]isoquinoline-6,12-dione [**G11**]: Compound **G11** was prepared according to the general procedure I (0.2 mmol scale).

Yield: 48% (30 mg).

Eluent: Petroleum ether/Ethyl acetate (95/5, v/v).

TLC: R_f = 0.6 (80:20 petroleum ether:EtOAc).

Physical State: Off-white solid.

^{1}H NMR (600 MHz, Chloroform-*d*) δ 8.60 (dd, *J* = 8.8, 0.5 Hz, 1H), 8.01 – 7.95 (m, 1H), 7.78 (dd, *J* = 2.3, 0.5 Hz, 1H), 7.69 (dd, *J* = 8.7, 2.3 Hz, 1H), 7.35 (ddd, *J* = 7.6, 2.0, 0.8 Hz, 1H), 7.22 (d, *J* = 7.6 Hz, 1H), 3.14 – 3.04 (m, 2H), 2.43 (s, 3H), 1.37 (s, 3H) ppm.

^{13}C NMR (151 MHz, $CDCl_3$) δ 199.90, 161.70, 149.60, 138.05, 137.57, 134.22, 133.75, 130.36, 129.52, 129.02, 128.97, 124.43, 124.20, 119.63, 67.78, 34.11, 22.33, 21.30 ppm.

HRMS (m/z): [M + H^+] calcd for $C_{18}H_{15}O_2NCl$, 312.07858; found, 312.07875.

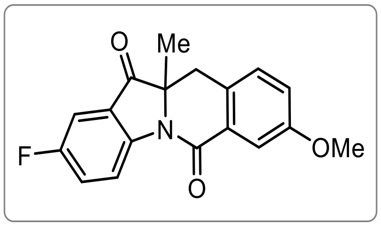

2-fluoro-8-methoxy-11a-methyl-11,11a-dihydroindolo[1,2-b]isoquinoline-6,12-dione [**G12**]: Compound **G12** was prepared according to the general procedure I (0.2 mmol scale).

Yield: 61% (38 mg).

Eluent: Petroleum ether/Ethyl acetate (85/15, v/v).

TLC: R_f = 0.45 (70:30 petroleum ether:EtOAc).

Physical State: Colorless solid.

^{1}H NMR (700 MHz, Chloroform-*d*) δ 8.65 (dd, *J* = 9.7, 4.2 Hz, 1H), 7.69 (d, *J* = 2.8 Hz, 1H), 7.51 – 7.45 (m, 2H), 7.26 – 7.22 (m, 1H), 7.10 (dd, *J* = 8.3, 2.8 Hz, 1H), 3.90 (s, 3H), 3.17 – 3.00 (m, 2H), 1.39 (s, 3H) ppm.

^{13}C NMR (176 MHz, $CDCl_3$) δ 200.32, 200.30 [200.31 (d, *J* = 2.9 Hz)], 161.33, 160.43, 159.02 [159.73 (d, *J* = 247.3 Hz)], 159.54, 147.53, 147.53 [147.53 (d, *J* = 1.2 Hz)], 130.26, 130.18, 128.79, 125.29, 125.15 [125.22 (d, *J* = 24.4 Hz)], 124.54, 124.50 [124.52 (d, *J* = 7.6 Hz)], 120.52, 119.97, 119.93 [119.95 (d, *J* = 7.6 Hz)], 112.54, 110.28, 110.15 [110.22 (d, *J* = 23.2 Hz)], 68.06, 55.85, 33.67, 22.37 ppm.

^{19}F NMR (377 MHz, Chloroform-*d*) δ -115.29 – -117.42 (m) ppm.

HRMS (m/z): [M + H$^+$] calcd for $C_{18}H_{15}O_2NCl$, 312.07858; found, 312.07875.

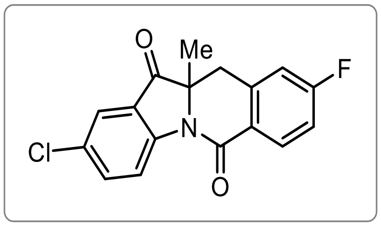

2-chloro-9-fluoro-11a-methyl-11,11a-dihydroindolo[1,2-b]isoquinoline-6,12-dione [**G13**]: Compound **G13** was prepared according to the general procedure I (0.2 mmol scale).

Yield: 52% (32.8 mg).

Eluent: Petroleum ether/Ethyl acetate (95/5, v/v).

TLC: R_f = 0.55 (80:20 petroleum ether:EtOAc).

Physical State: Yellow solid.

^{1}H NMR (500 MHz, Chloroform-*d*) δ 8.57 (d, *J* = 8.7 Hz, 1H), 8.19 (dd, *J* = 8.6, 5.7 Hz, 1H), 7.79 (d, *J* = 2.3 Hz, 1H), 7.70 (dd, *J* = 8.8, 2.3 Hz, 1H), 7.14 (td, *J* = 8.5, 2.5 Hz, 1H), 7.05 (dd, *J* = 8.6, 2.5 Hz, 1H), 3.19 – 3.07 (m, 2H), 1.40 (s, 3H) ppm.

^{13}C NMR (126 MHz, $CDCl_3$) δ 199.35, 166.76, 164.73 [165.74 (d, *J* = 255.7 Hz)], 160.49, 149.43, 139.76, 139.69 [139.72 (d, *J* = 9.2 Hz)], 137.72, 132.00, 131.92 [131.96 (d, *J* = 9.9 Hz)], 130.60, 125.54, 125.52 [125.53 (d, *J* = 2.9 Hz)], 124.31, 124.28, 119.55, 116.25, 116.07 [116.16 (d, *J* = 22.3 Hz)], 115.62, 115.44 [115.53 (d, *J* = 21.9 Hz)], 67.51, 34.53, 34.52 [34.52 (d, *J* = 1.6 Hz)], 22.44 ppm.

135-DEPT NMR (126 MHz, $CDCl_3$) δ 137.54, 131.82, 131.74 [131.78 (d, *J* = 9.9 Hz)], 124.13, 119.36, 116.06, 115.88 [115.97 (d, *J* = 22.6 Hz)], 115.43, 115.26 [115.34 (d, *J* = 21.9 Hz)], 34.34, 34.33 [34.34 (d, *J* = 1.2 Hz)], 22.25 ppm.

^{19}F NMR (470 MHz, Chloroform-*d*) δ -104.62 (td, *J* = 8.3, 5.6 Hz) ppm.

HRMS (m/z): [M + H$^+$] calcd for $C_{17}H_{12}O_2NClF$, 316.05351; found, 316.05366.

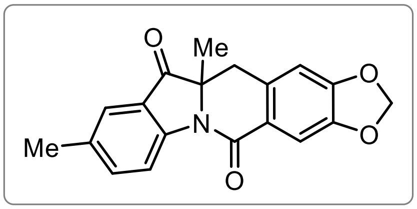

9,11a-dimethyl-11a,12-dihydro-[1,3]dioxolo[4,5-g]indolo[1,2-b]isoquinoline-5,11-dione [**G14**]: Compound **G14** was prepared according to the general procedure I (0.2 mmol scale).

Yield: 65% (41.8 mg).

Eluent: Petroleum ether/Ethyl acetate (80/20, v/v).

TLC: R_f = 0.25 (70:30 petroleum ether:EtOAc).

Physical State: Off-white solid.

^{1}H NMR (500 MHz, Chloroform-*d*) δ 8.47 (d, *J* = 8.4 Hz, 1H), 7.61 (dt, *J* = 1.7, 0.8 Hz, 1H), 7.60 (s, 1H), 7.56 (dd, *J* = 8.4, 1.5 Hz, 1H), 6.75 (s, 1H), 6.09 – 6.01 (m, 2H), 3.11 – 2.95 (m, 2H), 2.42 (s, 3H), 1.38 (s, 3H) ppm.

^{13}C NMR (126 MHz, $CDCl_3$) δ 201.13, 160.80, 151.75, 149.36, 147.72, 139.02, 134.68, 132.82, 124.31, 123.56, 123.10, 117.99, 108.89, 108.64, 102.15, 67.21, 34.53, 22.24, 21.14 ppm.

HRMS (m/z): [M + H$^+$] calcd for $C_{19}H_{16}O_4N$, 322.10738; found, 322.10751.

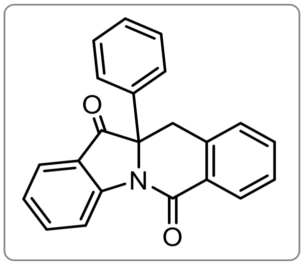

11a-phenyl-11,11a-dihydroindolo[1,2-b]isoquinoline-6,12-dione [**G15**]: Compound **G15** was prepared according to the general procedure I (0.2 mmol scale).

Yield: 63% (41 mg).

Eluent: Petroleum ether/Ethyl acetate (95/5, v/v).

TLC: R_f = 0.6 (80:20 petroleum ether:EtOAc).

Physical State: Off-white solid.

^{1}H NMR (500 MHz, Chloroform-*d*) δ 8.80 (d, *J* = 8.3 Hz, 1H), 8.11 (dd, *J* = 7.7, 1.4 Hz, 1H), 7.79 (ddd, *J* = 8.5, 7.3, 1.4 Hz, 1H), 7.75 (ddd, *J* = 7.7, 1.5, 0.7 Hz, 1H), 7.45 – 7.41 (m, 2H), 7.38 (dd, *J* = 7.5, 1.5 Hz, 1H), 7.32 (tt, *J* = 7.5, 1.1 Hz, 1H), 7.28 (td, *J* = 7.6, 1.0 Hz, 1H), 7.23 – 7.13 (m, 4H), 3.83 (d, *J* = 15.8 Hz, 1H), 3.46 (d, *J* = 15.8 Hz, 1H) ppm.

^{13}C NMR (126 MHz, $CDCl_3$) δ 197.73, 162.56, 151.92, 137.93, 136.26, 135.91, 133.26, 129.88, 129.07, 128.78, 128.52, 128.50, 127.94, 125.64, 125.27, 125.17, 122.94, 118.05, 72.10, 35.95 ppm.

135-DEPT NMR (126 MHz, $CDCl_3$) δ 137.75, 133.07, 128.88, 128.60, 128.34, 128.31, 127.75, 125.45, 125.09, 124.99, 117.86, 35.76 ppm.

HRMS (m/z): [M + H^+] calcd for $C_{22}H_{16}O_2N$, 326.11756; found, 326.11770.

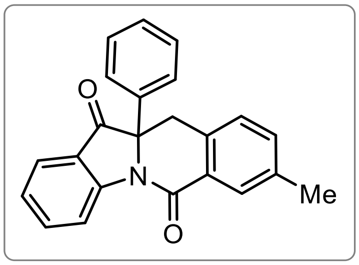

8-methyl-11a-phenyl-11,11a-dihydroindolo[1,2-b]isoquinoline-6,12-dione [**G16**]: Compound **G16** was prepared according to the general procedure I (0.2 mmol scale).

Yield: 72% (48.9 mg).

Eluent: Petroleum ether/Ethyl acetate (95/5, v/v).

TLC: R_f = 0.62 (80:20 petroleum ether:EtOAc).

Physical State: Off-white solid.

^{1}H NMR (500 MHz, Chloroform-*d*) δ 8.81 – 8.78 (m, 1H), 7.91 (d, *J* = 1.8 Hz, 1H), 7.79 (ddd, *J* = 8.4, 7.2, 1.4 Hz, 1H), 7.74 (dt, *J* = 7.6, 1.0 Hz, 1H), 7.46 – 7.39 (m, 2H), 7.31 – 7.24 (m, 1H), 7.23 – 7.13 (m, 4H), 7.09 (dd, *J* = 7.8, 0.9 Hz, 1H), 3.79 (d, *J* = 15.8 Hz, 1H), 3.40 (d, *J* = 15.8 Hz, 1H), 2.33 (s, 3H) ppm.

^{13}C NMR (126 MHz, $CDCl_3$) δ 197.83, 162.79, 152.00, 137.88, 137.74, 136.05, 134.08, 133.28, 129.63, 129.17, 129.04, 128.44, 128.38, 125.65, 125.24, 125.08, 122.94, 118.05, 72.24, 35.52, 21.26 ppm.

135-DEPT NMR (126 MHz, $CDCl_3$) δ 137.70, 133.90, 128.98, 128.86, 128.25, 128.19, 125.46, 125.05, 124.89, 117.86, 35.34, 21.07 ppm.

HRMS (m/z): [M + H^+] calcd for $C_{23}H_{18}O_2N$, 340.13321; found, 340.13339.

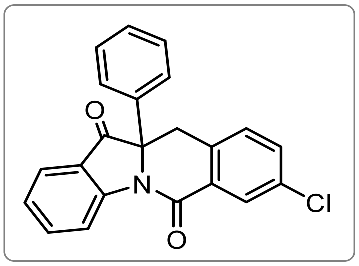

8-chloro-11a-phenyl-11,11a-dihydroindolo[1,2-b]isoquinoline-6,12-dione [**G17**]: Compound **G17** was prepared according to the general procedure I (0.2 mmol scale).

Yield: 58% (41.7 mg).

Eluent: Petroleum ether/Ethyl acetate (95/5, v/v).

TLC: R_f = 0.6 (80:20 petroleum ether:EtOAc).

Physical State: Colorless solid.

^{1}H NMR (500 MHz, Chloroform-*d*) δ 8.77 (d, *J* = 8.3 Hz, 1H), 8.08 (d, *J* = 2.2 Hz, 1H), 7.80 (ddd, *J* = 8.5, 7.2, 1.4 Hz, 1H), 7.75 (dd, *J* = 7.7, 1.3 Hz, 1H), 7.42 – 7.37 (m, 2H), 7.35 (dd, *J* = 8.1, 2.3 Hz, 1H), 7.30 (td, *J* = 7.5, 0.9 Hz, 1H), 7.24 – 7.14 (m, 4H), 3.82 (d, *J* = 15.9 Hz, 1H), 3.40 (d, *J* = 15.7 Hz, 1H) ppm.
^{13}C NMR (126 MHz, $CDCl_3$) δ 197.22, 161.24, 151.61, 138.00, 135.58, 134.57, 134.04, 133.17, 131.42, 129.88, 129.20, 128.75, 128.69, 125.55, 125.48, 125.35, 122.92, 118.07, 72.03, 35.47 ppm.
135-DEPT NMR (126 MHz, $CDCl_3$) δ 137.82, 132.99, 129.70, 129.02, 128.57, 128.51, 125.37, 125.30, 125.16, 117.89, 35.29 ppm.
HRMS (m/z): [M + H$^+$] calcd for $C_{22}H_{15}ClNO_2$, 360.0791; found, 360.083.

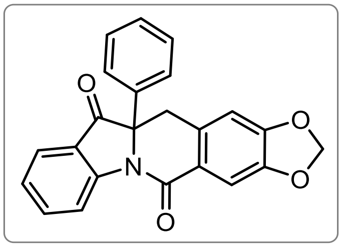

11a-phenyl-11a,12-dihydro-[1,3]dioxolo[4,5-g]indolo[1,2-b]isoquinoline-5,11-dione [**G18**]: Compound **G18** was prepared according to the general procedure I (0.2 mmol scale).

Yield: 66% (48.8 mg).

Eluent: Petroleum ether/Ethyl acetate (85/15, v/v).

TLC: R_f = 0.25 (70:30 petroleum ether:EtOAc).

Physical State: Off-white solid.

^{1}H NMR (700 MHz, Chloroform-d) δ 7.77 – 7.71 (m, 2H), 7.64 – 7.58 (m, 2H), 7.43 (dt, J = 8.2, 0.8 Hz, 1H), 7.36 – 7.31 (m, 2H), 7.28 – 7.24 (m, 1H), 7.08 – 7.04 (m, 1H), 7.01 (s, 1H), 6.60 (s, 1H), 5.93 (dd, J = 8.0, 1.5 Hz, 2H), 3.73 (dd, J = 15.9, 1.1 Hz, 1H), 3.34 (dt, J = 15.9, 0.6 Hz, 1H) ppm.

^{13}C NMR (176 MHz, CDCl3) δ 202.34, 160.94, 147.66, 144.51, 140.43, 139.87, 137.48, 128.80, 128.80, 128.15, 125.90, 125.79, 124.98, 124.52, 122.63, 114.34, 106.07, 101.66, 98.62, 81.07, 40.99 ppm.

HRMS (m/z): [M + H$^+$] calcd for $C_{23}H_{16}O_4N$, 370.10738; found, 370.10751.

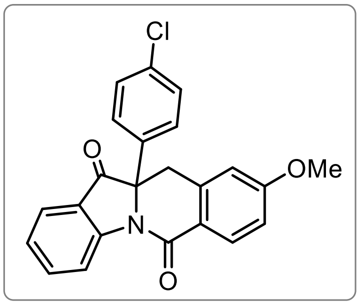

11a-(4-chlorophenyl)-9-methoxy-11,11a-dihydroindolo[1,2-b]isoquinoline-6,12-dione [**G19**]: Compound **G19** was prepared according to the general procedure I (0.2 mmol scale).

Yield: 43% (33.5 mg).

Eluent: Petroleum ether/Ethyl acetate (85/15, v/v).

TLC: R_f = 0.3 (70:30 petroleum ether:EtOAc).

Physical State: Off-white solid.

^{1}H NMR (500 MHz, Chloroform-*d*) δ 8.76 (dt, *J* = 8.3, 0.8 Hz, 1H), 8.05 (d, *J* = 8.6 Hz, 1H), 7.78 (ddd, *J* = 8.5, 7.2, 1.4 Hz, 1H), 7.74 (ddd, *J* = 7.7, 1.5, 0.7 Hz, 1H), 7.41 – 7.33 (m, 2H), 7.30 – 7.23 (m, 1H), 7.20 – 7.16 (m, 2H), 6.83 (ddd, *J* = 8.7, 2.5, 0.7 Hz, 1H), 6.67 (dd, *J* = 2.6, 1.1 Hz, 1H), 3.81 (s, 3H), 3.69 (d, *J* = 15.9 Hz, 1H), 3.41 (dt, *J* = 15.7, 1.1 Hz, 1H) ppm.

^{13}C NMR (126 MHz, $CDCl_3$) δ 197.48, 163.60, 162.32, 152.13, 138.24, 138.13, 134.77, 134.64, 131.07, 129.28, 127.13, 125.28, 125.02, 122.58, 122.42, 117.94, 113.86, 113.38, 71.63, 55.69, 36.27 ppm.

HRMS (m/z): [M + H$^+$] calcd for $C_{23}H_{17}O_3NCl$, 390.08915; found, 390.08908.

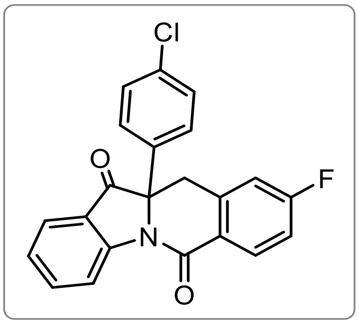

11a-(4-chlorophenyl)-9-fluoro-11,11a-dihydroindolo[1,2-b]isoquinoline-6,12-dione [**G20**]: Compound **G20** was prepared according to the general procedure I (0.2 mmol scale).

Yield: 62% (46.8 mg).

Eluent: Petroleum ether/Ethyl acetate (95/5, v/v).

TLC: R_f = 0.6 (80:20 petroleum ether:EtOAc).

Physical State: Yellow solid.

^{1}H NMR (500 MHz, Methylene Chloride-d_2) δ 8.74 (dt, *J* = 8.3, 0.8 Hz, 1H), 8.09 (dd, *J* = 8.6, 5.7 Hz, 1H), 7.81 (ddd, *J* = 8.5, 7.3, 1.4 Hz, 1H), 7.74 (ddd, *J* = 7.6, 1.4, 0.7 Hz, 1H), 7.39 – 7.34 (m, 2H), 7.31 (td, *J* = 7.5, 0.9 Hz, 1H), 7.24 – 7.17 (m, 2H), 7.03 (tdd, *J* = 8.5, 2.5, 0.9 Hz, 1H), 6.95 (ddd, *J* = 8.7, 2.5, 1.1 Hz, 1H), 3.75 (d, *J* = 16.0 Hz, 1H), 3.45 (dq, *J* = 16.0, 1.1 Hz, 1H) ppm.

^{13}C NMR (126 MHz, CD_2Cl_2) δ 196.59, 166.42, 164.39 [165.40 (d, *J* = 254.7 Hz)], 161.05, 151.69, 139.11, 139.04 [139.07 (d, *J* = 9.4 Hz)], 137.92, 134.55, 134.46 [134.51 (d, *J* = 11.0 Hz)], 131.38, 131.30 [131.34 (d, *J* = 10.0 Hz)], 129.08, 127.02, 126.21, 126.19 [126.20 (d, *J* = 2.7 Hz)], 125.16, 124.99, 122.48, 117.72, 115.48, 115.30, 115.18 [115.24 (d, *J* = 14.1 Hz)], 115.01, 71.37, 35.71 ppm.

^{19}F NMR (470 MHz, Methylene Chloride-d_2) δ -105.80 (td, *J* = 8.7, 5.5 Hz) ppm.

HRMS (m/z): [M + H^+] calcd for $C_{22}H_{14}O_2NClF$ 378.06916; found, 378.06919.

Synthesis of Compounds 5a-5d via oxa-Pictet-Spengler reactions of tryptophol and benzaldehyde derivatives.

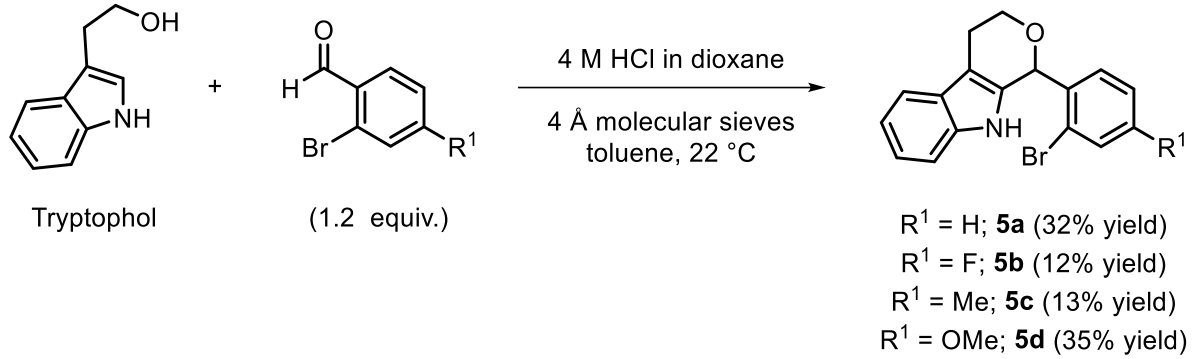

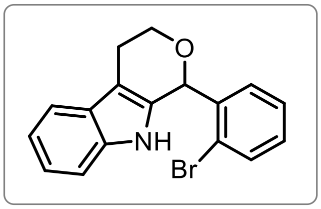

1-(2-bromophenyl)-1,3,4,9-tetrahydropyrano[3,4-b]indole [**5a**]: In an oven dried 50 ml round bottom flask with a magnetic stirrer, tryptophol (806 mg, 5 mmol), 2-bromobenzaldehyde (1.11 g, 6 mmol) and 4 Å molecular sieves (1 g) were taken. The flask was degassed and filled with Argon (three times) carefully as molecular sieves can go out by vacuum. Then, toluene (20 mL) and followed by 4 M HCl in dioxane (500 μL) was added under Ar at 22 °C; the mixture was stirred for overnight. Next, the reaction mixture was filtered through filter paper and concentrated in a rotary evaporator. The crude reaction mixture was purified through a silica gel column.

Yield: 32% (525 mg).

Eluent: Petroleum ether/Ethyl acetate (97/3, v/v).

TLC: R_f = 0.45 (90:10 petroleum ether:EtOAc).

Physical State: Amorphous white solid.

^{1}H NMR (500 MHz, Methylene Chloride-d_2) δ 7.75 (s, 1H), 7.67 (dd, J = 8.0, 1.3 Hz, 1H), 7.53 (dd, J = 7.6, 1.3 Hz, 1H), 7.30 (td, J = 7.8, 1.8 Hz, 2H), 7.28 – 7.25 (m, 1H), 7.25 – 7.21 (m, 1H), 7.13 (ddd, J = 8.1, 7.1, 1.4 Hz, 1H), 7.09 (ddd, J = 8.2, 7.1, 1.2 Hz, 1H), 6.29 (t, J = 2.0 Hz, 1H), 4.30 (ddd, J = 11.3, 5.3, 3.1 Hz, 1H), 4.00 (ddd, J = 11.3, 9.4, 4.0 Hz, 1H), 3.07 (dddd, J = 15.0, 9.4, 5.3, 2.1 Hz, 1H), 2.83 (dddd, J = 15.5, 4.1, 3.1, 1.9 Hz, 1H) ppm.

^{13}C NMR (126 MHz, CD_2Cl_2) δ 139.74, 136.47, 133.42, 133.38, 130.57, 130.48, 128.28, 127.27, 123.66, 122.34, 119.93, 118.63, 111.34, 109.22, 74.74, 65.05, 22.65 ppm.

HRMS (m/z): [M + H^+] calcd for $C_{17}H_{15}ONBr$ 328.03315 and 330.03111; found, 328.03323 and 330.03051.

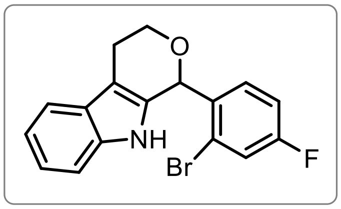

***1-(2-bromo-4-fluorophenyl)-1,3,4,9-tetrahydropyrano[3,4-b]indole* [5b]:** Compound **5b** was prepared according to the above procedure (5 mmol scale).

Yield: 12% (207.7 mg).

Eluent: Petroleum ether/Ethyl acetate (97/3, v/v).

TLC: R_f = 0.50 (90:10 petroleum ether:EtOAc).

Physical State: Amorphous white solid.

^{1}H NMR (500 MHz, Methylene Chloride-*d*$_2$) δ 7.70 (s, 1H), 7.53 (dq, *J* = 7.6, 0.9 Hz, 1H), 7.42 (dd, *J* = 8.3, 2.6 Hz, 1H), 7.34 – 7.23 (m, 2H), 7.14 (ddd, *J* = 8.1, 7.1, 1.4 Hz, 1H), 7.10 (ddd, *J* = 8.1, 7.1, 1.2 Hz, 1H), 7.02 (td, *J* = 8.3, 2.6 Hz, 1H), 6.25 (t, *J* = 2.0 Hz, 1H), 4.27 (ddd, *J* = 11.3, 5.3, 3.3 Hz, 1H), 3.99 (ddd, *J* = 11.3, 9.3, 4.1 Hz, 1H), 3.05 (dddd, *J* = 15.4, 9.3, 5.3, 2.0 Hz, 1H), 2.83 (dddd, *J* = 15.5, 4.1, 3.3, 1.9 Hz, 1H) ppm.

^{13}C NMR (126 MHz, Methylene Chloride-*d*$_2$) δ 162.63 (d, *J* = 251.5 Hz), 135.88 (d, *J* = 3.6 Hz), 131.87 (d, *J* = 8.7 Hz), 123.85 (d, *J* = 9.7 Hz), 120.44 (d, *J* = 24.6 Hz), 115.42 (d, *J* = 21.2 Hz) ppm.

^{19}F NMR (470 MHz, Methylene Chloride-*d*$_2$) δ -111.85 (td, *J* = 8.2, 6.1 Hz) ppm.

HRMS (m/z): [M + H$^+$] calcd for $C_{17}H_{14}ONBrF$ 346.02373 and 348.02168; found, 346.02373 and 348.02141.

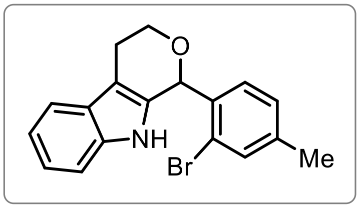

***1-(2-bromo-4-methylphenyl)-1,3,4,9-tetrahydropyrano[3,4-b]indole* [5c]:** Compound **5c** was prepared according to the above procedure (5 mmol scale).

Yield: 13% (222.5 mg).

Eluent: Petroleum ether/Ethyl acetate (97/3, v/v).

TLC: R_f = 0.50 (90:10 petroleum ether:EtOAc).

Physical State: Amorphous white solid.

^{1}H NMR (500 MHz, Methylene Chloride-*d*$_2$) δ 7.72 (s, 1H), 7.53 (dd, *J* = 7.7, 1.4 Hz, 1H), 7.50 (d, *J* = 0.8 Hz, 1H), 7.25 (dt, *J* = 8.0, 1.0 Hz, 1H), 7.17 – 7.11 (m, 2H), 7.11 – 7.07 (m, 2H), 6.24 (t, *J* = 2.0 Hz, 1H), 4.27 (ddd, *J* = 11.3, 5.3, 3.2 Hz, 1H), 3.99 (ddd, *J* = 11.3, 9.3, 4.0 Hz, 1H), 3.05 (dddd, *J* = 15.4, 9.3, 5.4, 2.1 Hz, 1H), 2.82 (dddd, *J* = 15.4, 4.1, 3.3, 1.9 Hz, 1H), 2.33 (s, 3H).

^{13}C NMR (126 MHz, CD_2Cl_2) δ 141.03, 136.58, 136.45, 133.72, 133.64, 130.27, 129.05, 127.31, 123.49, 122.27, 119.90, 118.60, 111.32, 109.18, 74.54, 64.93, 22.66, 20.93.

HRMS (m/z): [M + H^+] calcd for $C_{18}H_{17}ONBr$ 342.04880 and 344.04676; found, 342.04895 and 344.04624.

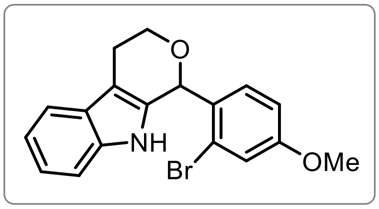

1-(2-bromo-4-methoxyphenyl)-1,3,4,9-tetrahydropyrano[3,4-b]indole **[5d]:** Compound **5d** was prepared according to the above procedure (5 mmol scale).

Yield: 35% (627 mg).

Eluent: Petroleum ether/Ethyl acetate (93/7, v/v).

TLC: R_f = 0.30 (90:10 petroleum ether:EtOAc).

Physical State: Amorphous white solid.

^{1}H NMR (500 MHz, Methylene Chloride-*d*$_2$) δ 7.72 (s, 1H), 7.53 (dd, *J* = 7.7, 1.2 Hz, 1H), 7.26 (dt, *J* = 8.0, 1.0 Hz, 1H), 7.21 (d, *J* = 2.6 Hz, 1H), 7.16 – 7.11 (m, 2H), 7.11 – 7.07 (m, 1H), 6.81 (dd, *J* = 8.7, 2.6 Hz, 1H), 6.22 (t, *J* = 1.9 Hz, 1H), 4.25 (ddd, *J* = 11.3, 5.3, 3.4 Hz, 1H), 3.98 (ddd, *J* = 11.3, 9.1, 4.1 Hz, 1H), 3.80 (s, 3H), 3.04 (dddd, *J* = 15.4, 9.1, 5.3, 2.0 Hz, 1H), 2.86 – 2.77 (m, 1H) ppm.

^{13}C NMR (126 MHz, CD_2Cl_2) δ 160.67, 136.46, 133.72, 131.55, 131.28, 127.33, 124.34, 122.26, 119.89, 118.60, 118.47, 114.16, 111.32, 109.31, 74.30, 64.79, 56.05, 22.67 ppm.

HRMS (m/z): [M + H^+] calcd for $C_{18}H_{17}O_2NBr$ 358.04372 and 360.04167; found, 358.04397 and 360.04167.

CLASS-H

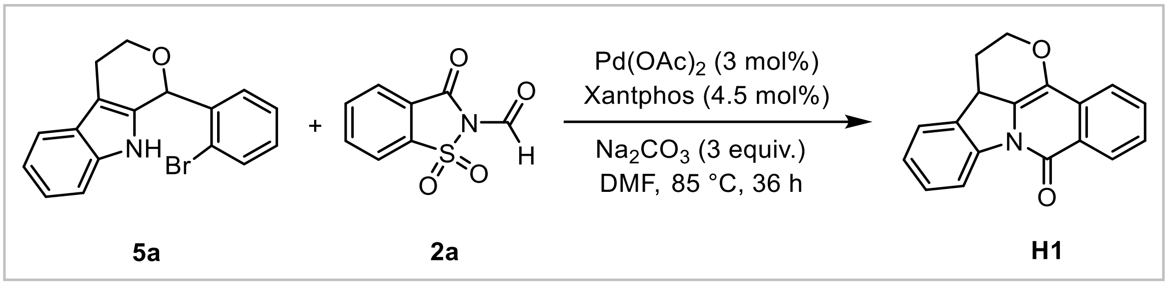

General procedure J: Synthesis of Class H

An oven-dried screw capped reaction tube with a magnetic stir-bar was charged with 1-(2-bromophenyl)-1,3,4,9-tetrahydropyrano[3,4-b]indole (0.2 mmol), *N*-formyl saccharin (**2a**, 1.5 equiv., 0.3 mmol, 63.3 mg), $Pd(OAc)_2$ (3 mol%, 1.34 mg), Xantphos (4.5 mol%, 5.2 mg), Na_2CO_3 (3 equiv., 63.6 mg). A screw cap fitted with a rubber septum was attached the reaction tube, then degassed and refilled with argon; the process was repeated an additional two times. Dry *N*,*N*-dimethyl formamide (DMF, 2 mL) was added in the reaction tube, placed in metal block, and vigorously stirred at room temperature for 10 min followed by heated to 85 °C. The reaction mixture was taken out after 36 h and DMF was removed by rotavapor under high vacuum. [*Note*: *ethyl acetate/water extraction was avoided*] The crude mixture was purified by flash column chromatography using silica gel (100-200 mesh size) and petroleum ether/ethyl acetate as the eluent.

Characterization data for Class H products

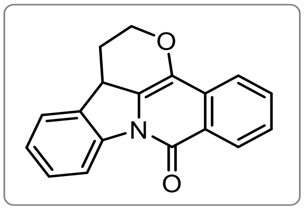

1,13b-dihydrodibenzo[b,f]pyrano[2,3,4-hi]indolizin-8(2H)-one [**H1**]: Compound **H1** was prepared according to the general procedure J (0.2 mmol scale).

Yield: 53% (29.2 mg isolated).

Eluent: Petroleum ether/Ethyl acetate (90/10, v/v).

TLC: R_f = 0.45 (80:20 petroleum ether:EtOAc).

Physical State: Yellow solid.

^{1}H NMR (500 MHz, Methylene Chloride-*d*$_2$) δ 8.53 – 8.48 (m, 1H), 8.43 (ddd, *J* = 7.5, 1.1, 0.6 Hz, 1H), 7.78 – 7.73 (m, 1H), 7.68 (ddd, *J* = 8.1, 7.0, 1.3 Hz, 1H), 7.49 (ddd, *J* = 8.2, 7.0, 1.4 Hz, 1H), 7.47 – 7.41 (m, 1H), 7.38 (tt, *J* = 7.8, 1.1 Hz, 1H), 7.22 (td, *J* = 7.5, 1.1 Hz, 1H), 4.65 (ddd, *J* = 11.3, 4.4, 1.7 Hz, 1H), 4.38 (ddd, *J* = 12.8, 11.2, 2.9 Hz, 1H), 4.24 (dd, *J* = 11.4, 5.0 Hz, 1H), 2.62 (dddd, *J* = 12.8, 4.8, 2.9, 1.7 Hz, 1H), 1.88 (tdd, *J* = 12.9, 11.3, 4.3 Hz, 1H) ppm.

^{13}C NMR (126 MHz, CD_2Cl_2) δ 158.81, 143.17, 132.97, 132.13, 131.21, 131.11, 128.43, 128.18, 126.80, 126.52, 125.18, 124.20, 123.03, 119.79, 116.84, 66.92, 38.50, 25.31 ppm.

135 DEPT NMR (126 MHz, CD_2Cl_2) δ 132.13 (CH), 128.44 (CH), 128.19 (CH), 126.52 (CH), 125.18 (CH), 124.20 (CH), 119.79 (CH), 116.84 (CH), 66.92 (CH_2), 38.50 (CH), 25.31 (CH_2) ppm.

HRMS (m/z): [M + H^+] calcd for $C_{18}H_{14}NO_2$, 276.10186; found, 276.10191.

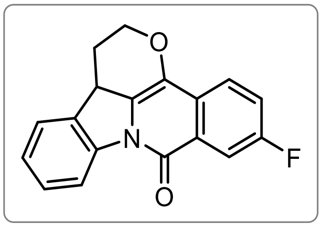

6-fluoro-1,13b-dihydrodibenzo[b,f]pyrano[2,3,4-hi]indolizin-8(2H)-one [**H2**]: Compound **H2** was prepared according to the general procedure J (0.2 mmol scale).

Yield: 48% (28.2 mg isolated).

Eluent: Petroleum ether/Ethyl acetate (90/10, v/v).

TLC: R_f = 0.47 (80:20 petroleum ether:EtOAc).

Physical State: Yellow solid.

^{1}H NMR (700 MHz, Methylene Chloride-d_2) δ 8.50 (d, *J* = 8.0 Hz, 1H), 8.09 (dd, *J* = 9.6, 2.8 Hz, 1H), 7.78 (dd, *J* = 8.7, 5.2 Hz, 1H), 7.46 (dd, *J* = 7.4, 1.5 Hz, 1H), 7.42 (td, *J* = 8.5, 2.8 Hz, 1H), 7.41 – 7.38 (m, 1H), 7.25 (td, *J* = 7.4, 1.1 Hz, 1H), 4.67 (ddd, *J* = 11.1, 4.3, 1.6 Hz, 1H), 4.40 (ddd, *J* = 12.9, 11.1, 2.9 Hz, 1H), 4.26 (dd, *J* = 11.4, 5.1 Hz, 1H), 2.65 (dddd, *J* = 12.9, 4.8, 2.9, 1.6 Hz, 1H), 1.90 (tdd, *J* = 12.8, 11.3, 4.3 Hz, 1H) ppm.

^{13}C NMR (176 MHz, CD_2Cl_2) δ <u>162.25, 160.85</u> [161.55 (d, *J* = 246.3 Hz)], <u>157.87, 157.85</u> [157.86 (d, *J* = 3.4 Hz)], 143.04, 133.07, 131.02, <u>128.77, 128.73</u> [128.75 (d, *J* = 7.7 Hz)], 128.52, <u>127.84, 127.83</u> [127.83 (d, *J* = 2.3 Hz)], 125.43, 124.29, 124.28, <u>122.42, 122.41</u> [122.41 (d, *J* = 2.8 Hz)], <u>122.34, 122.31</u> [122.32 (d, *J* = 7.9 Hz)], 122.30, <u>120.50, 120.37</u> [120.43 (d, *J* = 23.5 Hz)], 116.93, <u>113.69, 113.56</u> [113.63 (d, *J* = 23.3 Hz)], 67.05, 38.44, 25.36 ppm.

^{19}F NMR (470 MHz, Methylene Chloride-d_2) δ -114.33 (td, *J* = 8.8, 5.4 Hz) ppm.

HRMS (m/z): [M + H^+] calcd for $C_{18}H_{13}FNO_2$, 294.0930; found, 294.09248.

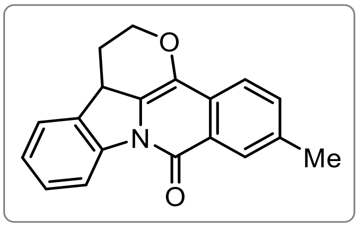

6-methyl-1,13b-dihydrodibenzo[b,f]pyrano[2,3,4-hi]indolizin-8(2H)-one [**H3**]: Compound **H3** was prepared according to the general procedure J (0.2 mmol scale).

Yield: 62% (35.9 mg isolated).

Eluent: Petroleum ether/Ethyl acetate (90/10, v/v).

TLC: R_f = 0.5 (80:20 petroleum ether:EtOAc).

Physical State: Yellow solid.

^{1}H NMR (500 MHz, Chloroform-*d*) δ 8.59 (dd, *J* = 8.0, 1.2 Hz, 1H), 8.31 (dt, *J* = 1.9, 0.8 Hz, 1H), 7.68 (d, *J* = 8.1 Hz, 1H), 7.52 (dd, *J* = 8.2, 1.9 Hz, 1H), 7.40 (ddt, *J* = 10.2, 7.7, 1.4 Hz, 2H), 7.22 (td, *J* = 7.5, 1.1 Hz, 1H), 4.67 (ddd, *J* = 11.2, 4.3, 1.6 Hz, 1H), 4.40 (ddd, *J* = 12.9, 11.3, 2.9 Hz, 1H), 4.26 (dd, *J* = 11.4, 5.0 Hz, 1H), 2.63 (dddd, *J* = 12.9, 4.9, 2.9, 1.7 Hz, 1H), 2.51 (s, 3H), 1.94 (tdd, *J* = 12.9, 11.3, 4.3 Hz, 1H) ppm.

^{13}C NMR (126 MHz, $CDCl_3$) δ 159.37, 143.45, 137.04, 133.82, 132.96, 131.84, 128.93, 128.87, 128.43, 127.03, 125.42, 124.24, 121.92, 120.04, 117.48, 66.95, 38.64, 25.61, 21.73 ppm.

135-DEPT NMR (126 MHz, $CDCl_3$) δ 133.64 (CH), 128.69 (CH), 128.25 (CH), 125.23 (CH), 124.06 (CH), 119.85 (CH), 117.29 (CH), 66.76 (CH_2), 38.45 (CH), 25.43 (CH_2), 21.55(CH_3) ppm.

HRMS (m/z): [M + H^+] calcd for $C_{19}H_{16}NO_2$, 290.1181; found, 290.1176.

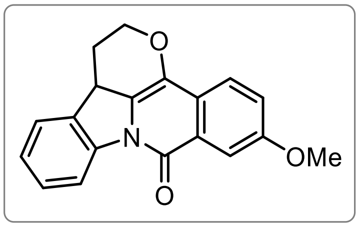

6-methoxy-1,13b-dihydrodibenzo[b,f]pyrano[2,3,4-hi]indolizin-8(2H)-one [**H4**]: Compound **H4** was prepared according to the general procedure J (0.2 mmol scale).

Yield: 56% (34.2 mg isolated).

Eluent: Petroleum ether/Ethyl acetate (85/15, v/v).

TLC: R_f = 0.46 (70:30 petroleum ether:EtOAc).

Physical State: Yellow solid.

^{1}H NMR (500 MHz, Chloroform-*d*) δ 8.59 (d, *J* = 8.0 Hz, 1H), 7.93 (d, *J* = 2.7 Hz, 1H), 7.71 (d, *J* = 8.7 Hz, 1H), 7.46 – 7.38 (m, 2H), 7.29 (dd, *J* = 8.7, 2.7 Hz, 1H), 7.23 (td, *J* = 7.4, 1.1 Hz, 1H), 4.66 (ddd, *J* = 11.2, 4.3, 1.7 Hz, 1H), 4.39 (ddd, *J* = 12.6, 11.2, 2.8 Hz, 1H), 4.25 (dd, *J* = 11.3, 4.9 Hz, 1H), 3.94 (s, 3H), 2.62 (dddd, *J* = 13.0, 4.9, 2.9, 1.7 Hz, 1H), 1.92 (tdd, *J* = 12.7, 11.3, 4.2 Hz, 1H) ppm.

^{13}C NMR (126 MHz, $CDCl_3$) δ 158.97, 158.94, 143.43, 133.19, 131.91, 128.85, 128.43, 125.52, 125.21, 124.28, 122.43, 121.75, 120.61, 117.48, 109.10, 66.99, 55.83, 38.55, 25.63 ppm.

135-DEPT NMR (126 MHz, $CDCl_3$) δ 128.68 (CH), 125.35 (CH), 124.11 (CH), 122.26 (CH), 121.58 (CH), 117.31 (CH), 108.92 (CH), 66.82 (CH_2), 55.66 (CH_3), 38.37 (CH), 25.45 (CH_2) ppm.

HRMS (m/z): [M + H^+] calcd for $C_{19}H_{16}O_3N$, 306.11247; found, 306.11230.

Supplementary References

1. Li, S., Nie, H., Duan, M., Wang, W., Zhu, C. & Song, C. Construction of a Protoberberine Alkaloid Skeleton via the Palladium-Catalyzed α-Arylation−Amide Formation Cascade. *Org. Lett*. **23**, 9631−9634 (2021).
2. Tukey, J. W. Explanatory data analysis. *Addison-Wesley, Reading, MA* (1977).
3. Akbarzadeh, M., Deipenwisch, I., Schoelermann, B., Pahl, A., Sievers, S., Ziegler, S., Waldmann, H. Morphological profiling by means of the Cell Painting assay enables identification of tubulin-targeting compounds. *Cell Chem. Biol.* **29**, 1053-1064 (2022).
4. Bray, M. A., Singh, S., Han, H., Davis, C. T., Borgeson, B., Hartland, C., Kost-Alimova, M., Gustafsdottir, S. M., Gibson, C. C., and Carpenter, A. E. Cell Painting, a high-content image-based assay for morphological profiling using multiplexed fluorescent dyes. *Nat. Protoc*. **11**, 1757–1774 (2016).
5. Woehrmann, M. H., Bray, W. M., Durbin, J. K., Nisam, S. C., Michael, A. K., Glassey, E., Stuart, J. M. & Lokey, R. S. Large-scale cytological profiling for functional analysis of bioactive compounds. *Mol. BioSyst*. **9**, 2604–2617 (2013).
6. Wu, X., Ding, S., Ding, Q., Gray, N. S. & Schultz, P. G. A small molecule with osteogenesis-inducing activity in multipotent mesenchymal progenitor cells. *J. Am. Chem. Soc.* **124**, 14520 (2002).
7. Lipinski, R. J., Gipp, J. J., Zhang, J., Doles, J. D. & Bushman, W. Unique and complimentary activities of the Gli transcription factors in Hedgehog signaling. *Exp. Cell Res*. **312**, 1925 (2006).
8. Regl, G., Neill, G. W., Eichberger, T., Kasper, M., Ikram, M. S., Koller, J., Hintner, H., Quinn, A. G., Frischauf, A. M. & Aberger, F. Human GLI2 and GLI1 are part of a positive feedback mechanism in Basal Cell Carcinoma. *Oncogene* **21**, 5529 (2002).
9. Pfaffl, M. W. A new mathematical model for relative quantification in real-time RT-PCR. *Nucleic Acids Res.* **29**, e45 (2001).
10. Sinha, S. & Chen, J. K. Purmorphamine activates the Hedgehog pathway by targeting Smoothened. *Nat. Chem. Biol.* **2**, 29 (2006).
11. Talpale, J., Chen, J. K., Cooper, M. K., Wang, B., Mann, R. K., Milenkovic, L., Scott, M. P. & Beachy, P. A. Effects of oncogenic mutations in Smoothened and Patched can be reversed by cyclopamine. *Nature* **406**, 1005 (2000).

12. Dolomanov, O. V., Bourhis, L. J., Gildea, R. J., Howard, J. A. K., Puschmann, H. OLEX2: a complete structure solution, refinement and analysis program. *J. Appl. Cryst*. **42**, 339–341 (2009).
13. Sheldrick, G. M. SHELXT-Integrated Space-Group and Crystal-Structure Determination. *Acta Cryst*. **A71**, 3–8 (2015).
14. Bera, S., Daniliuc, C. G. & Studer, A. Oxidative *N*-Heterocyclic Carbene Catalyzed Dearomatization of Indoles to Spirocyclic Indolenines with a Quaternary Carbon Stereocenter. *Angew. Chem. Int. Ed*. **56**, 7402–7406 (2017).
15. Grugel, C. P. & Breit, B. Rhodium-Catalyzed Diastereo- and Enantioselective Tandem Spirocyclization/Reduction of 3-Allenylindoles: Access to Functionalized Vinylic Spiroindolines. *Org. Lett*. **21**, 9672-9676 (2019).

NMR Spectra

Starting Materials

***3-(2-bromobenzyl)-2-methyl-1H-indole* [1a]:**

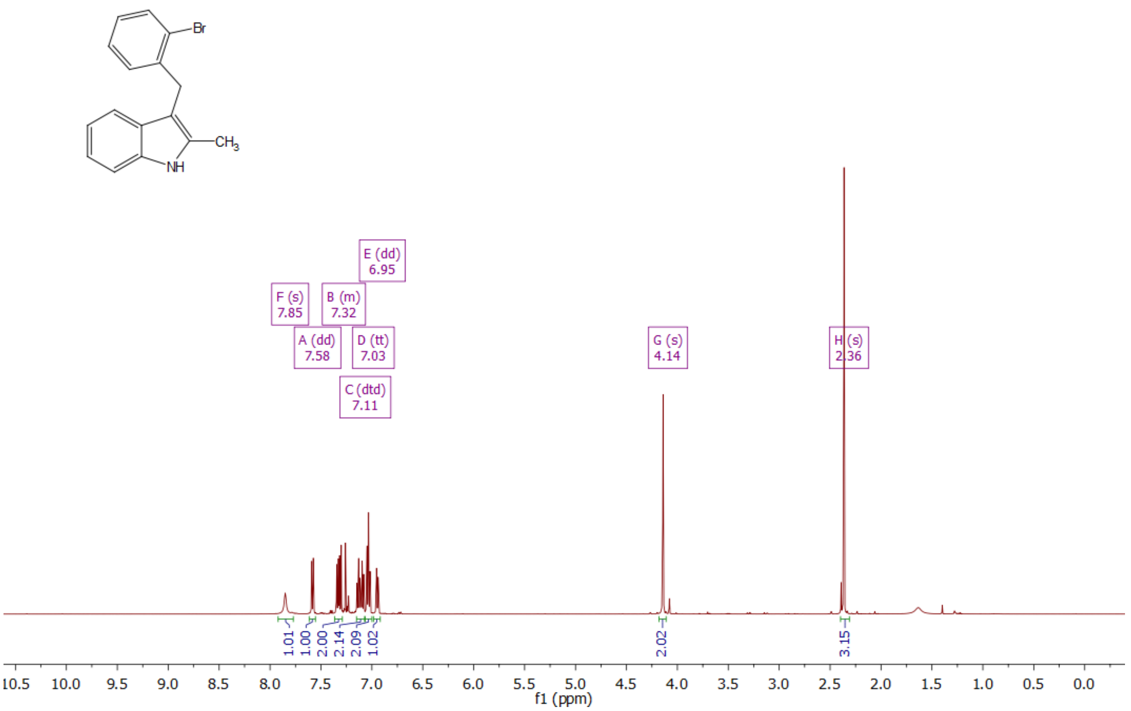

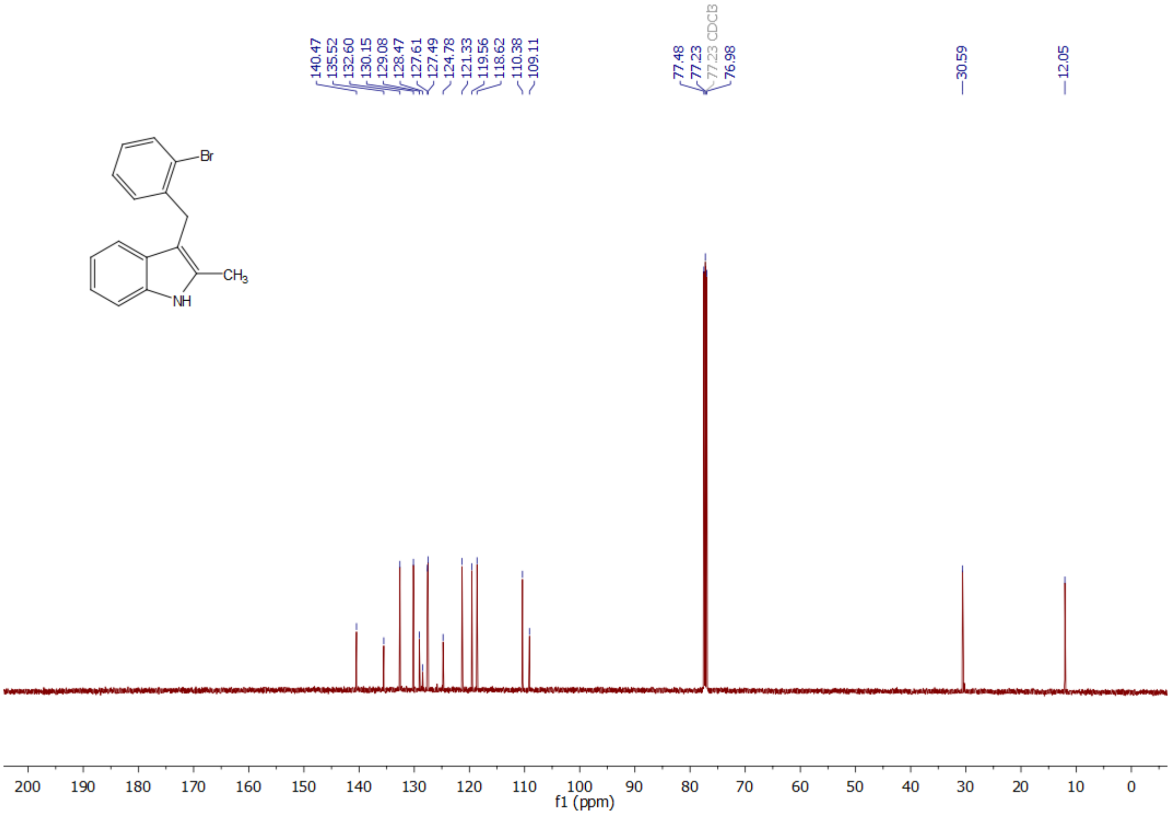

3-(2-bromo-4-chlorobenzyl)-2-methyl-1H-indole [**1b**]:

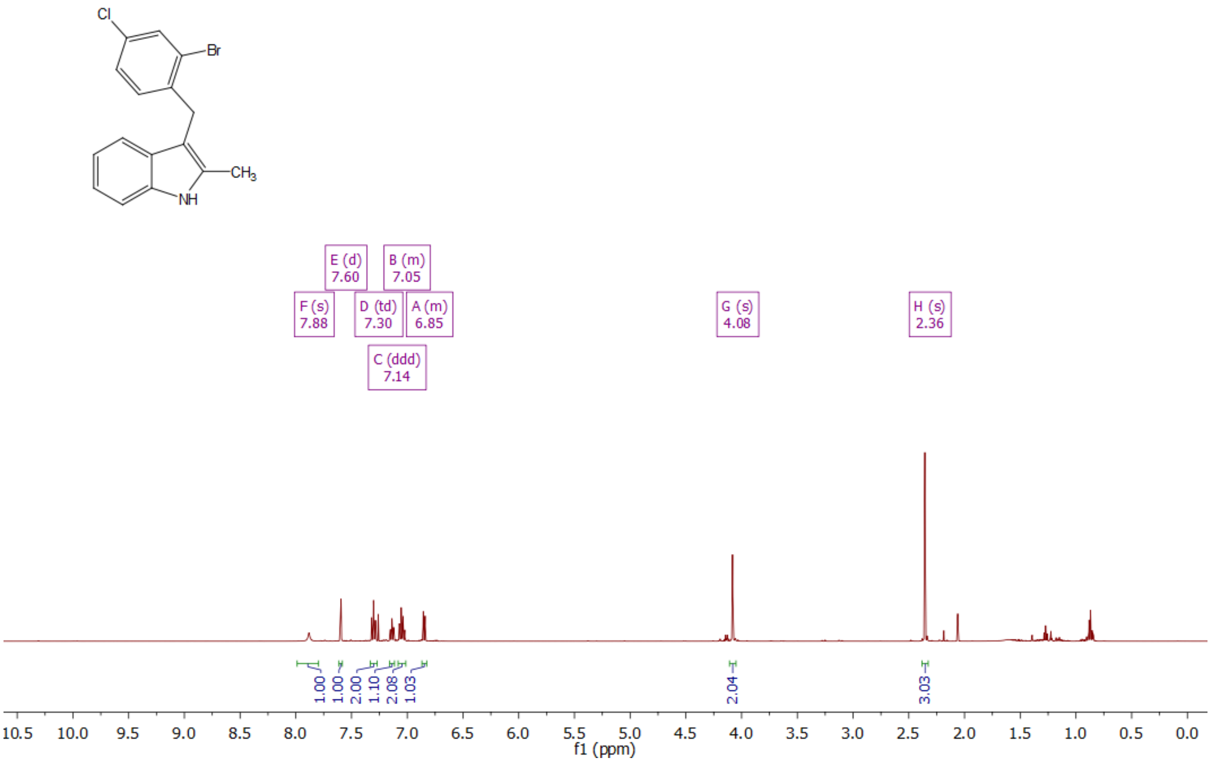

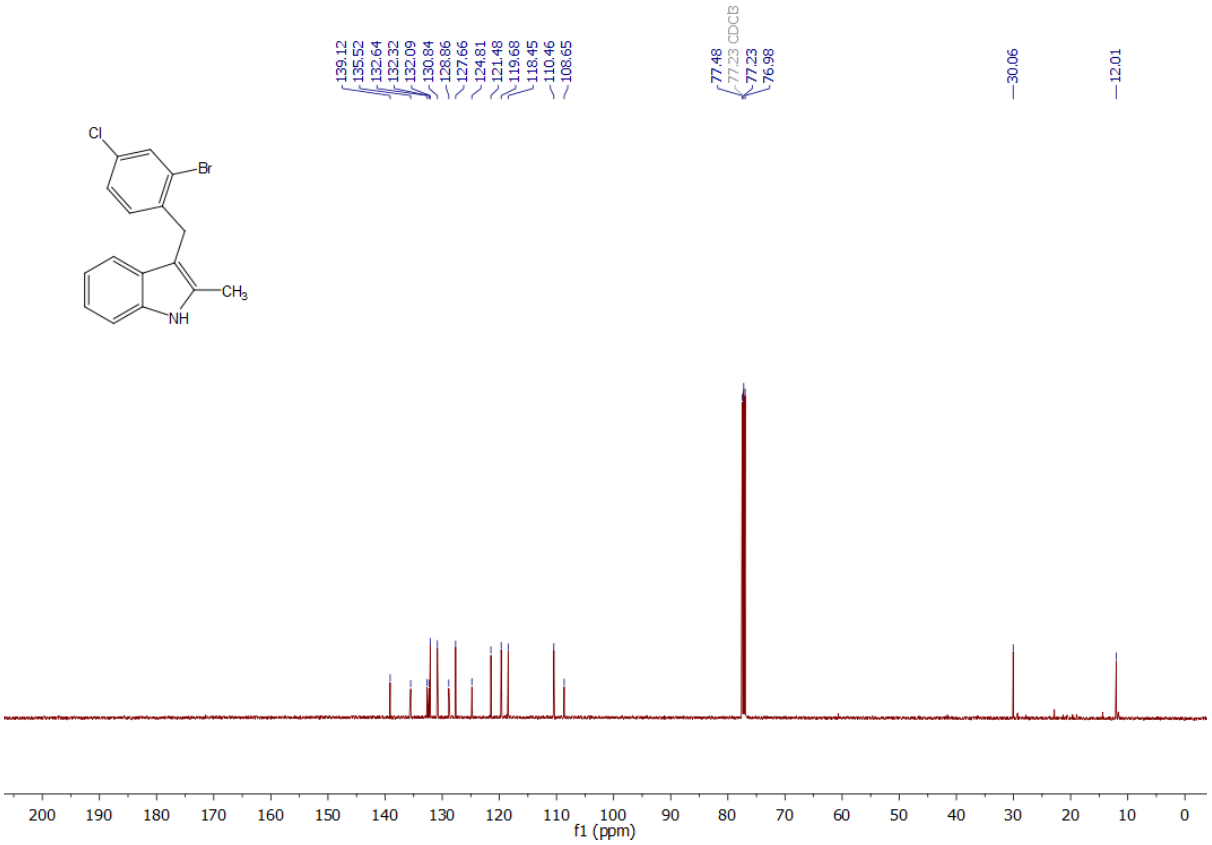

3-(2-bromo-4-methylbenzyl)-2-methyl-1H-indole [**1c**]:

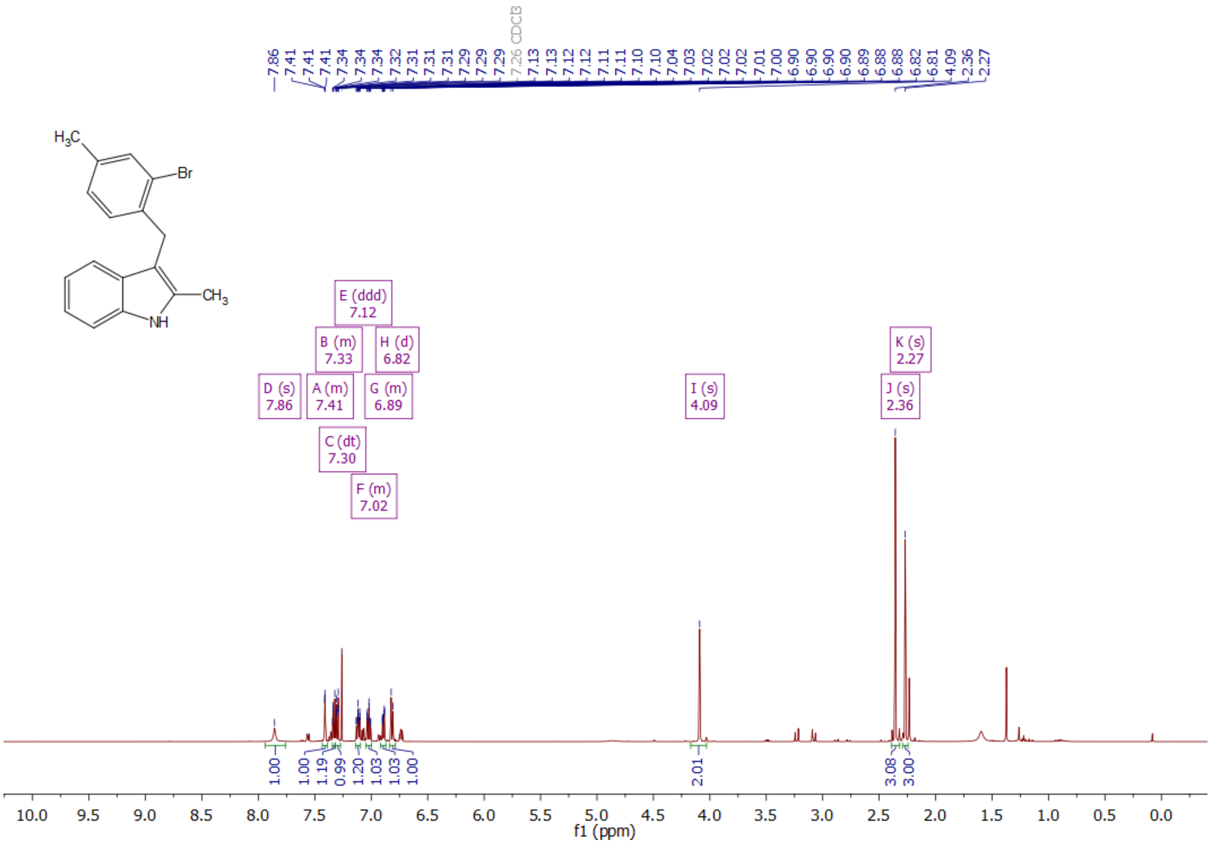

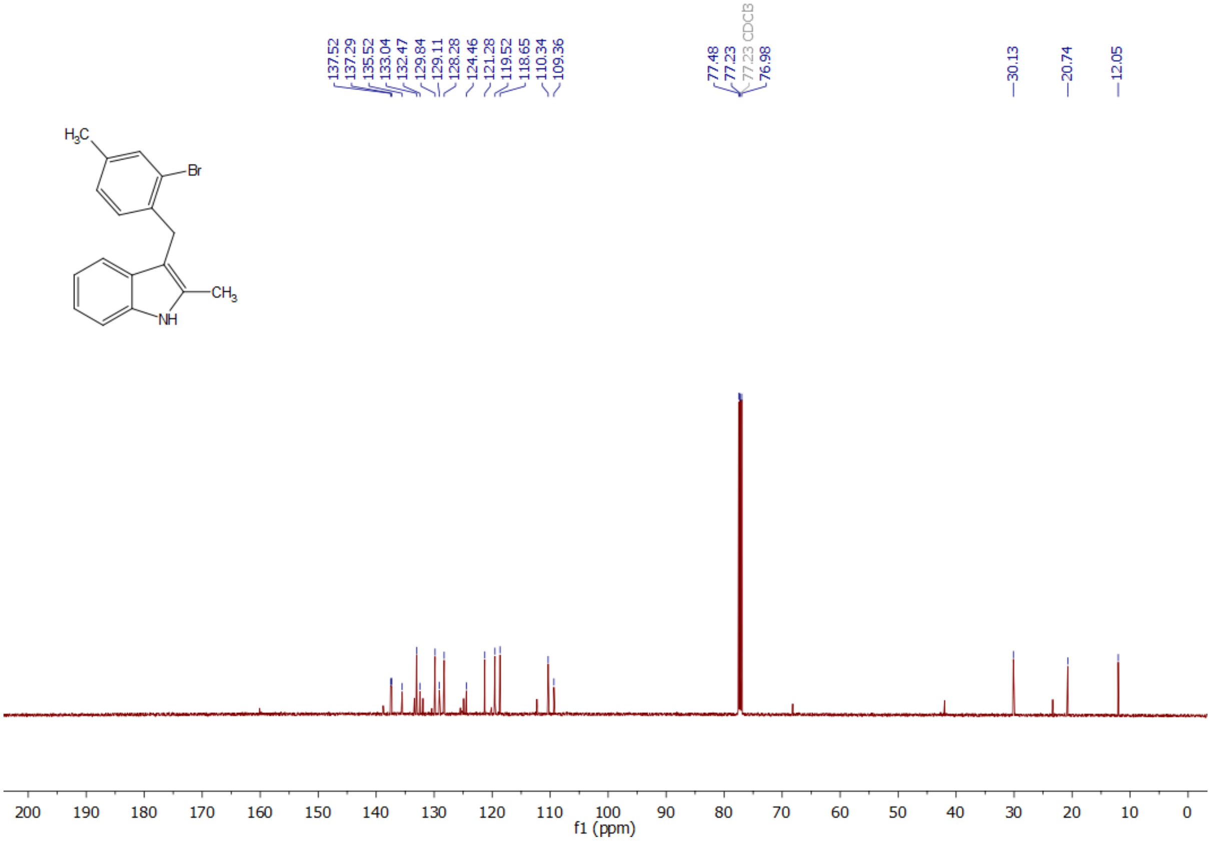

3-(2-bromo-5-fluorobenzyl)-2-methyl-1H-indole [**1d**]:

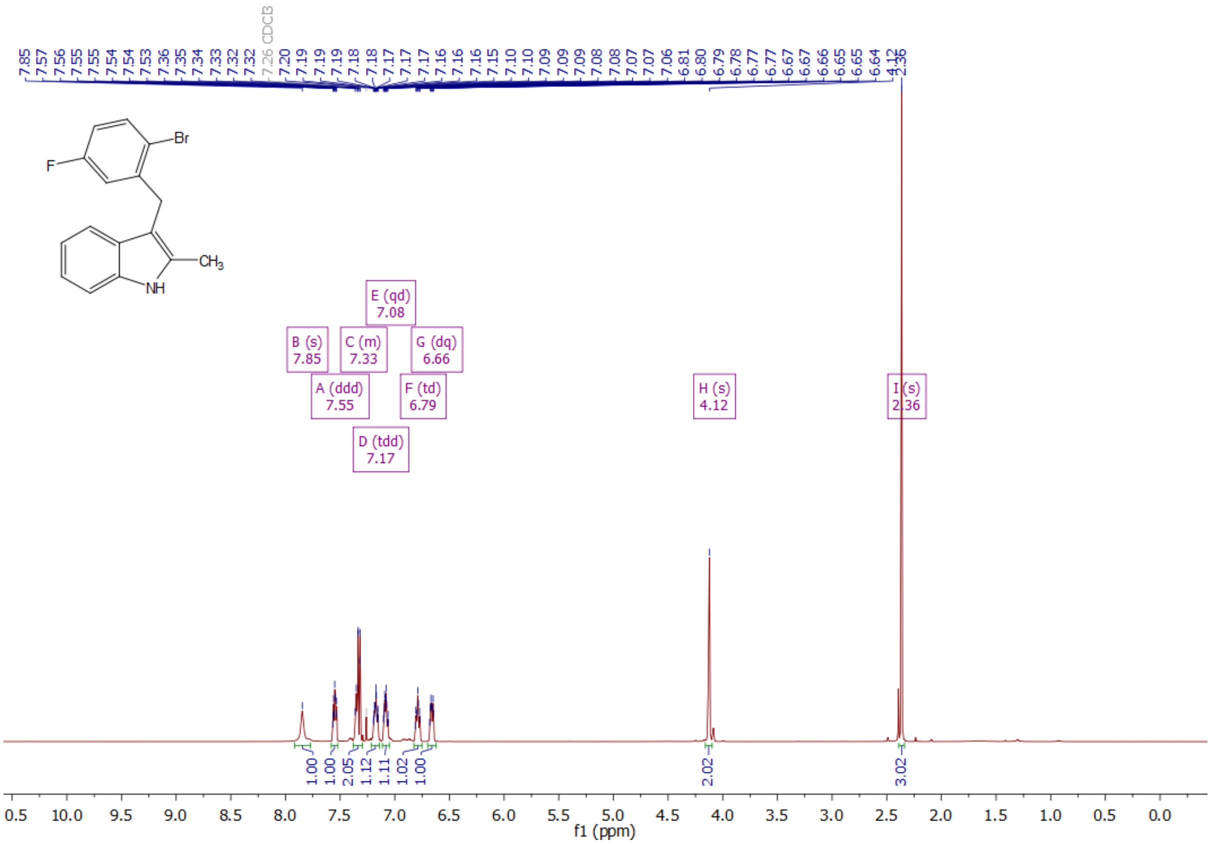

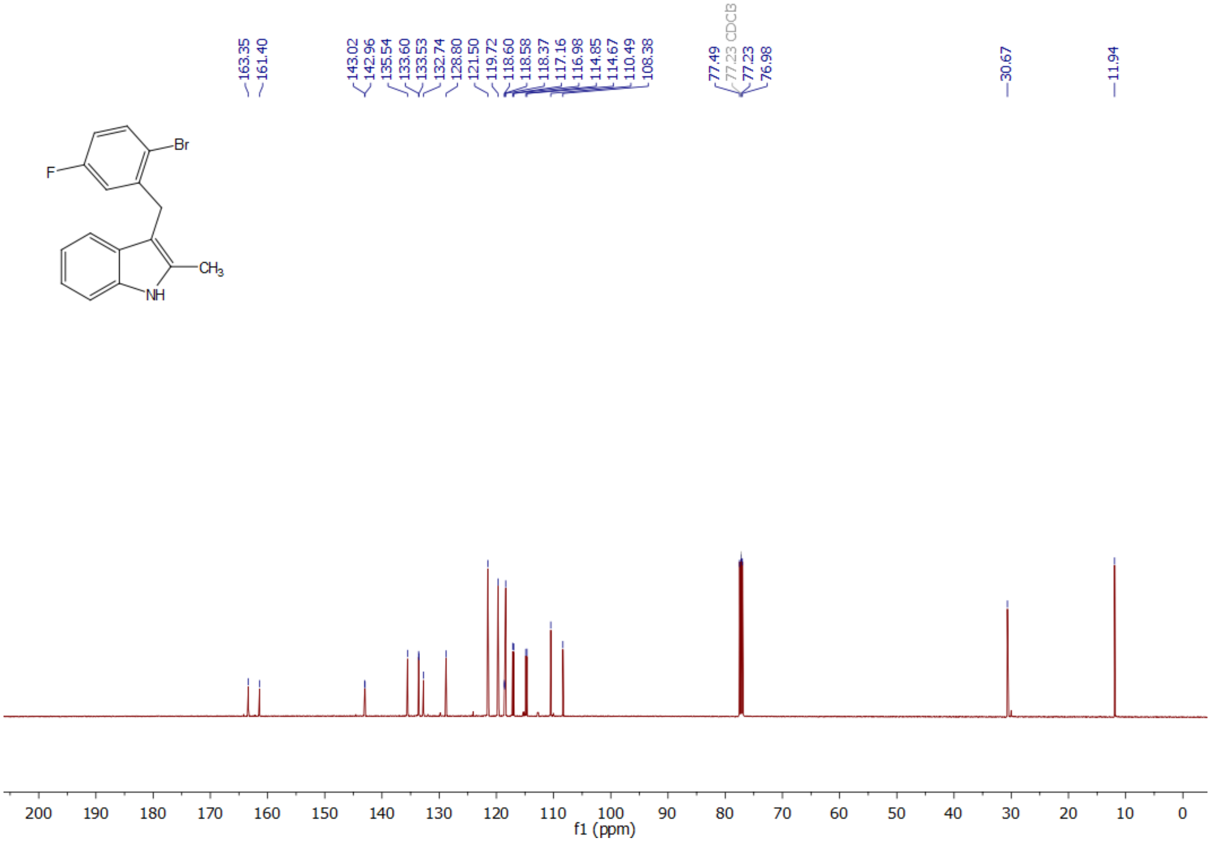

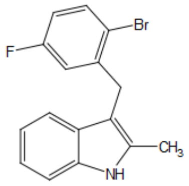
Br
F
CH3
NH

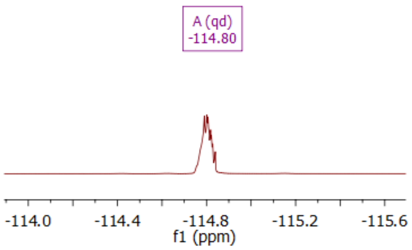
A (qd)
-114.80
-114.0
-114.4
-114.8
-115.2
-115.6
f1 (ppm)

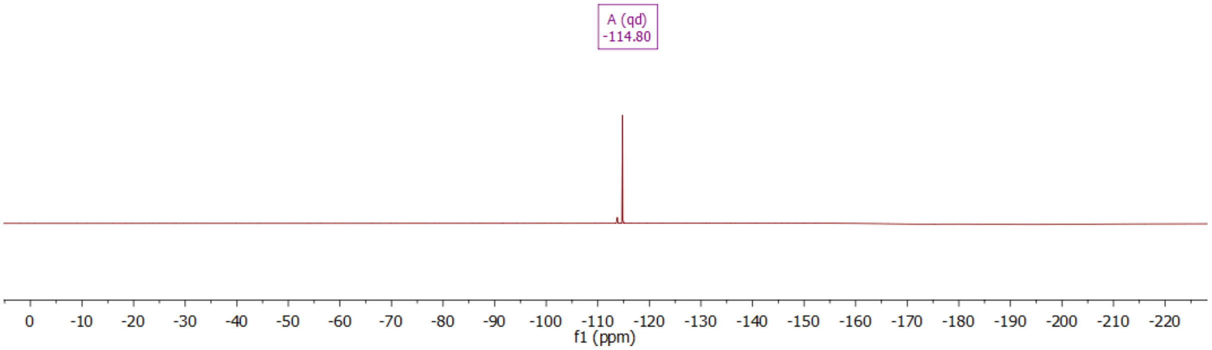
A (qd)
-114.80
0
-10
-20
-30
-40
-50
-60
-70
-80
-90
-100
-110
-120
-130
-140
-150
-160
-170
-180
-190
-200
-210
-220
f1 (ppm)

3-(2-bromo-5-methoxybenzyl)-2-methyl-1H-indole [**1e**]:

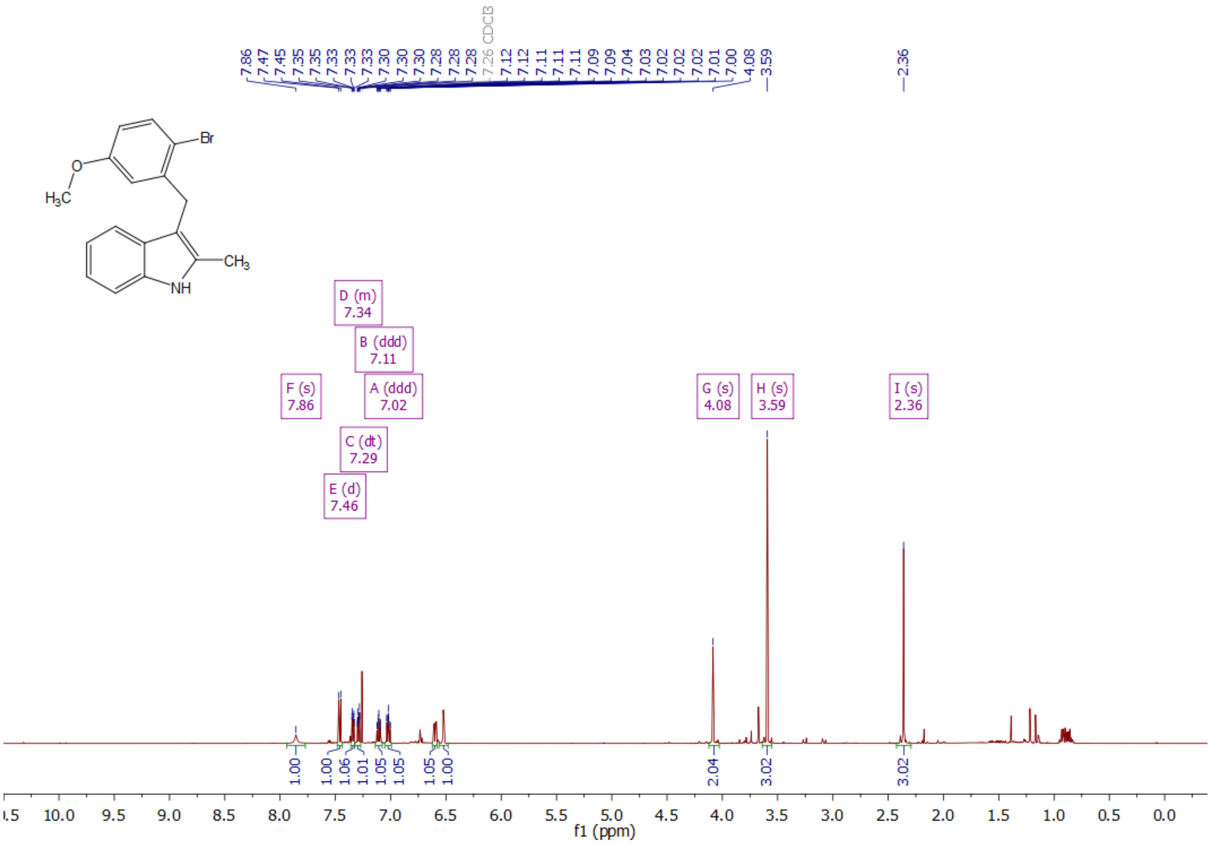

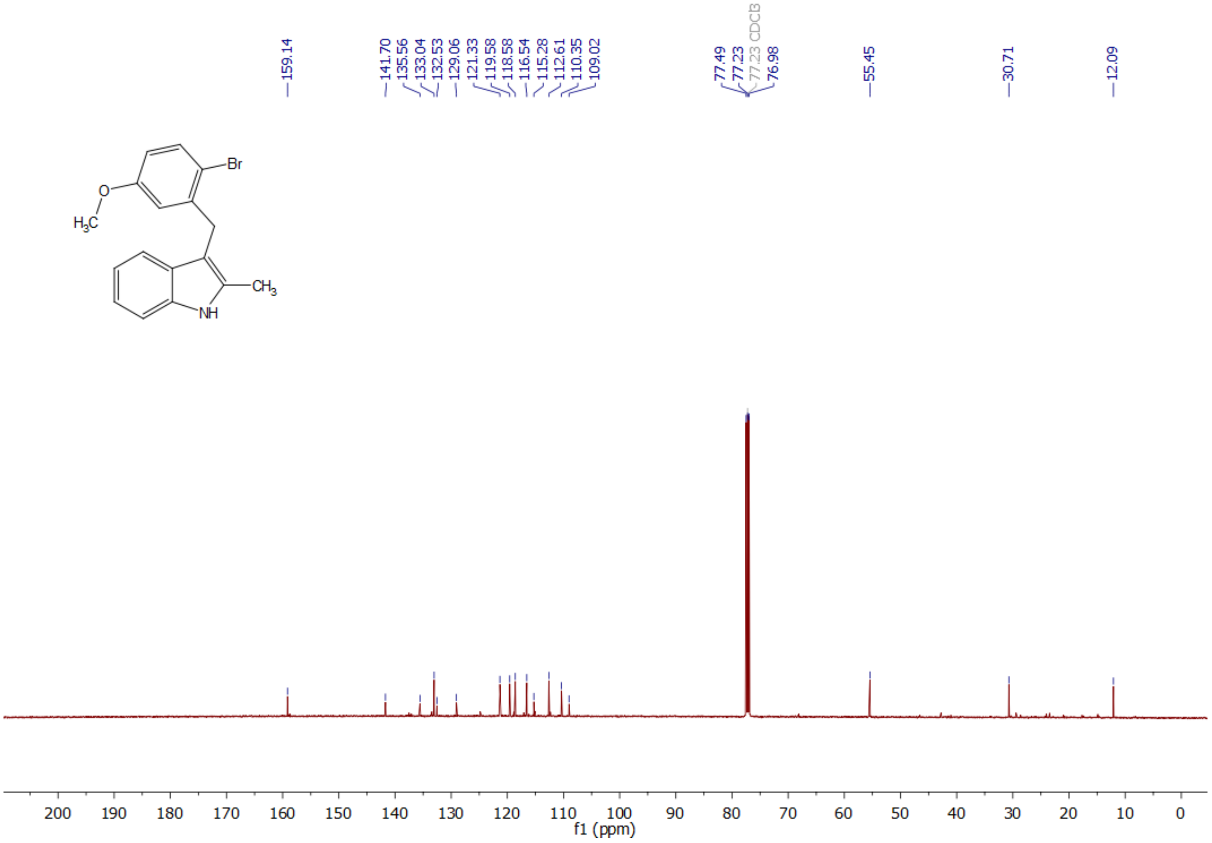

3-(2-bromo-6-fluorobenzyl)-2-methyl-1H-indole [**1f**]:

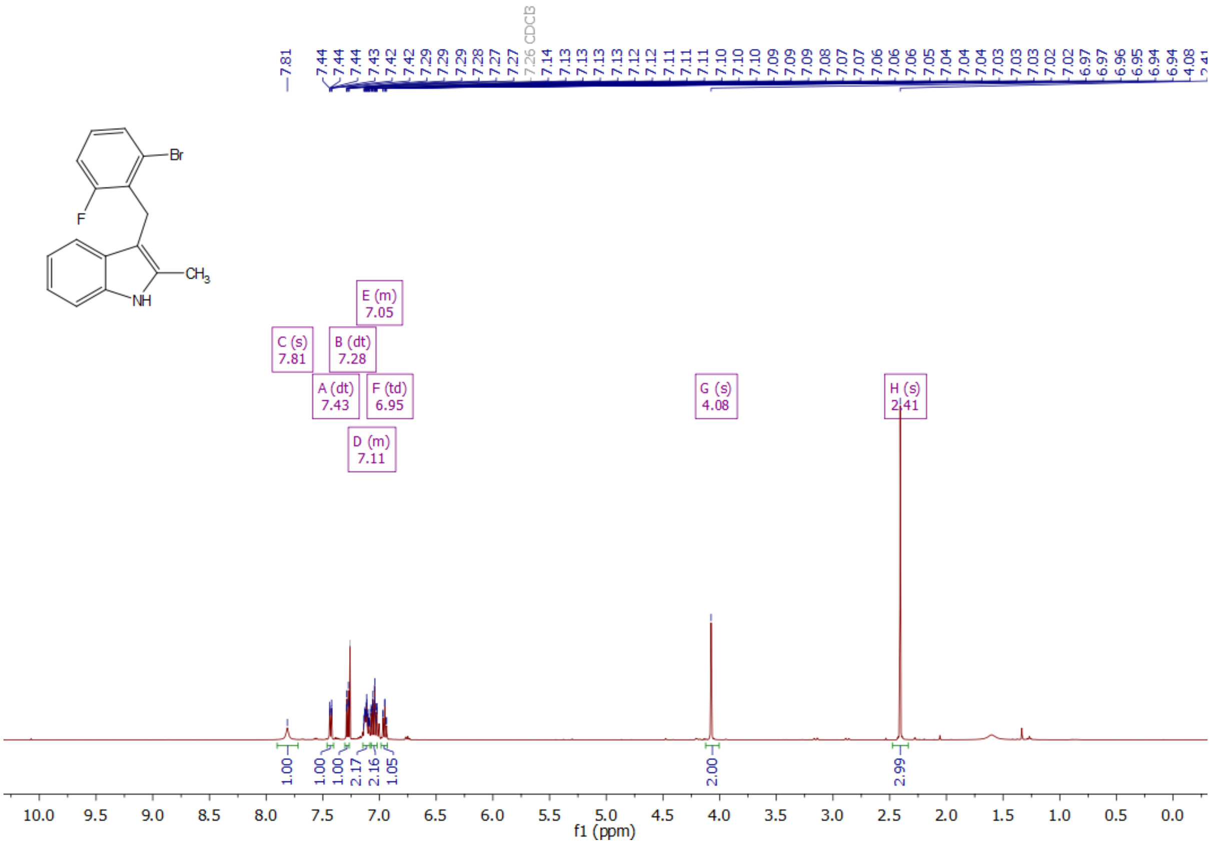

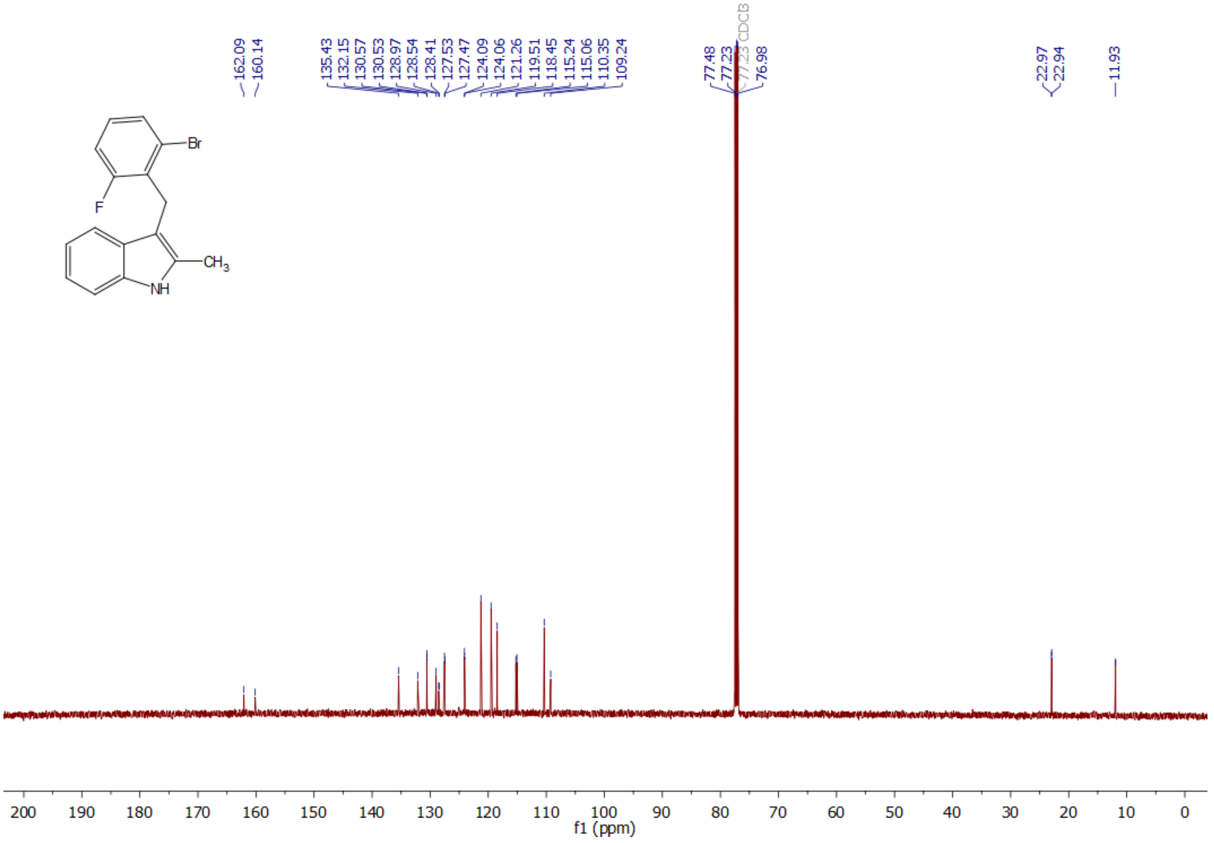

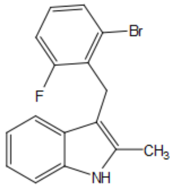
Br
F
CH3
NH

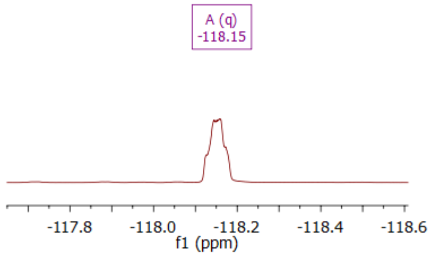
A (q)
-118.15
-117.8
-118.0
-118.2
-118.4
-118.6
f1 (ppm)

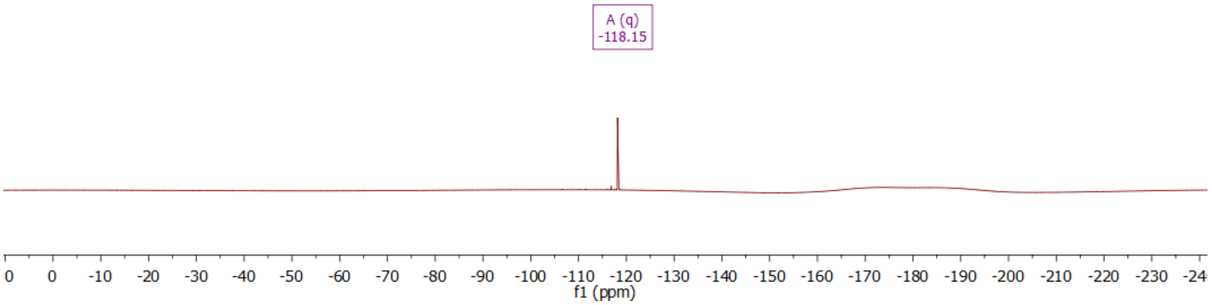
A (q)
-118.15
0 0 -10 -20 -30 -40 -50 -60 -70 -80 -90 -100 -110 -120 -130 -140 -150 -160 -170 -180 -190 -200 -210 -220 -230
f1 (ppm)

3-(2-bromo-4,5-dimethoxybenzyl)-2-methyl-1H-indole [**1g**]:

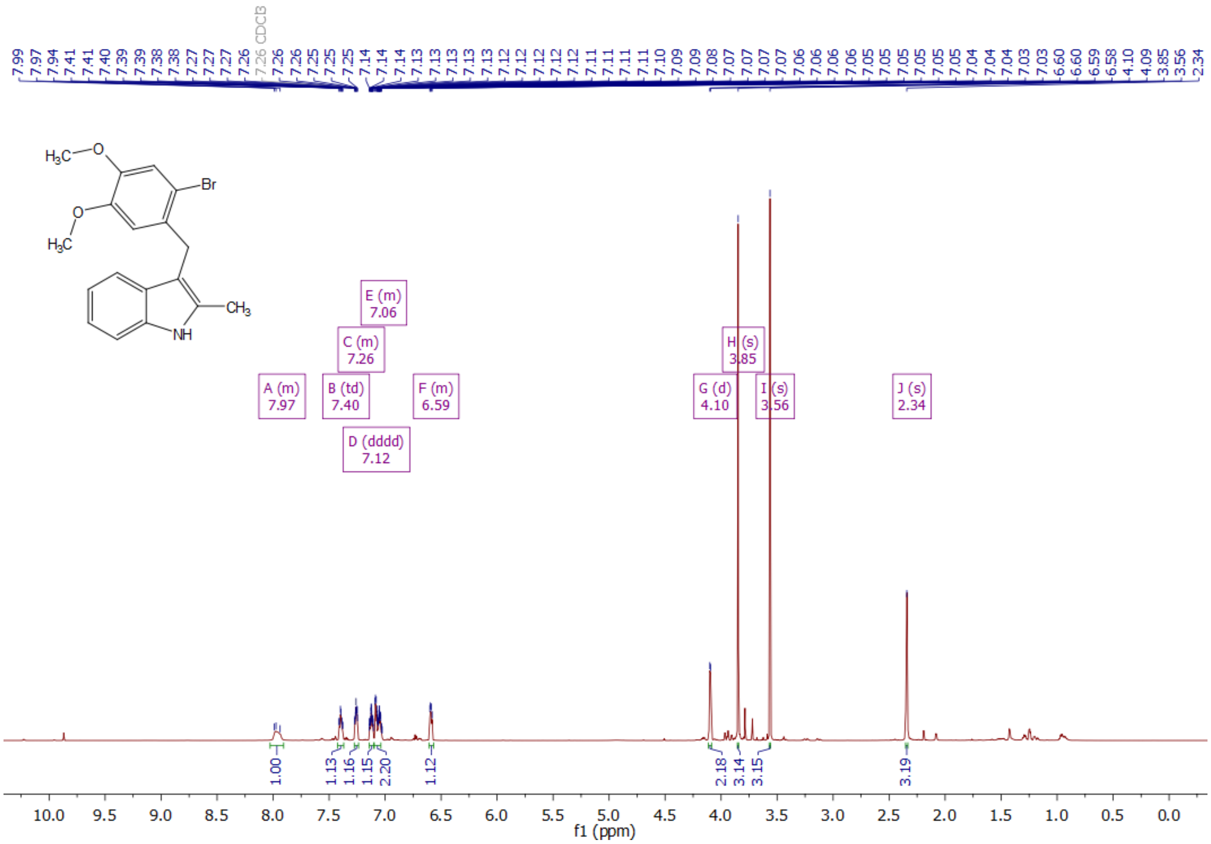

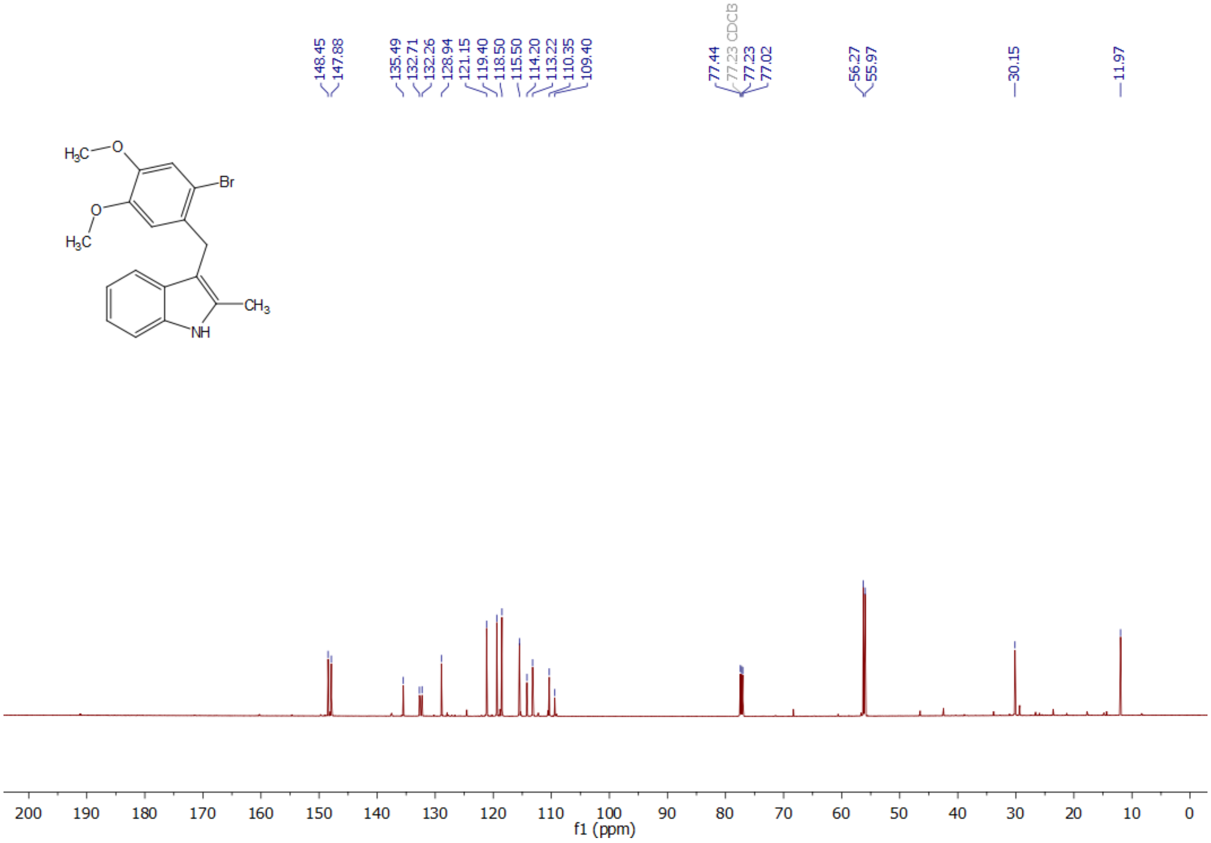

3-(2-bromo-4-fluoro-5-methoxybenzyl)-2-methyl-1H-indole [**1h**]:

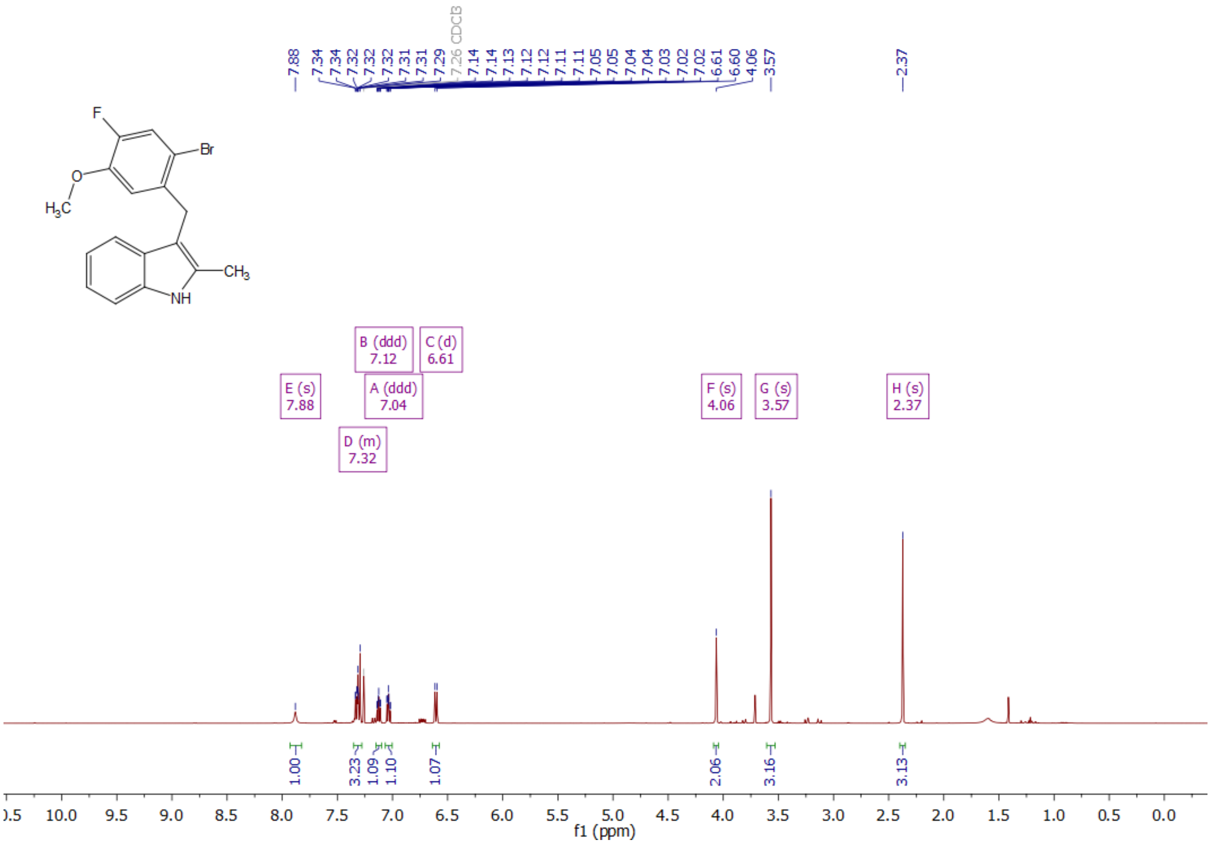

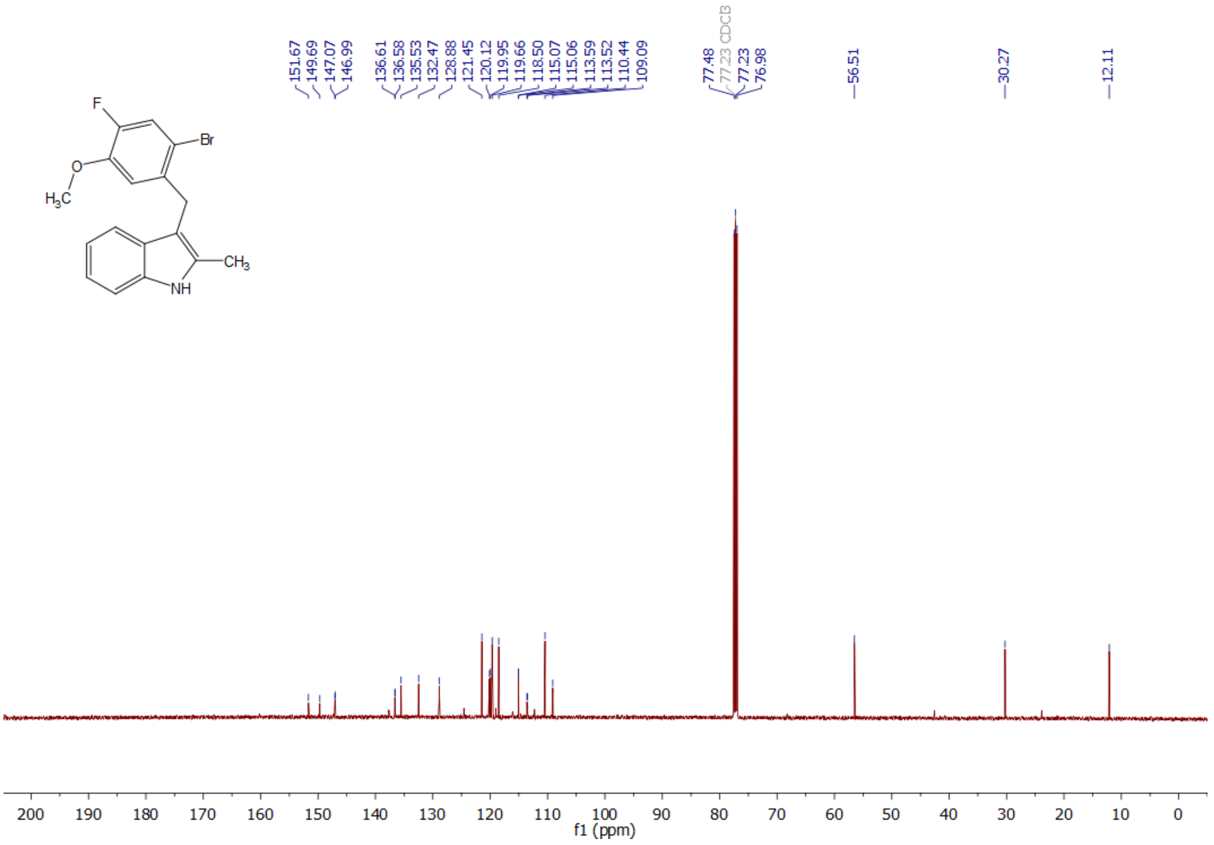

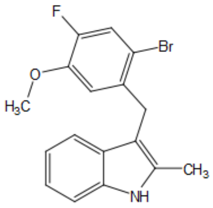
F
Br
O
H3C
CH3
NH

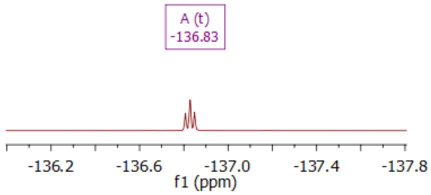
A (t)
-136.83
-136.2
-136.6
-137.0
-137.4
-137.8
f1 (ppm)

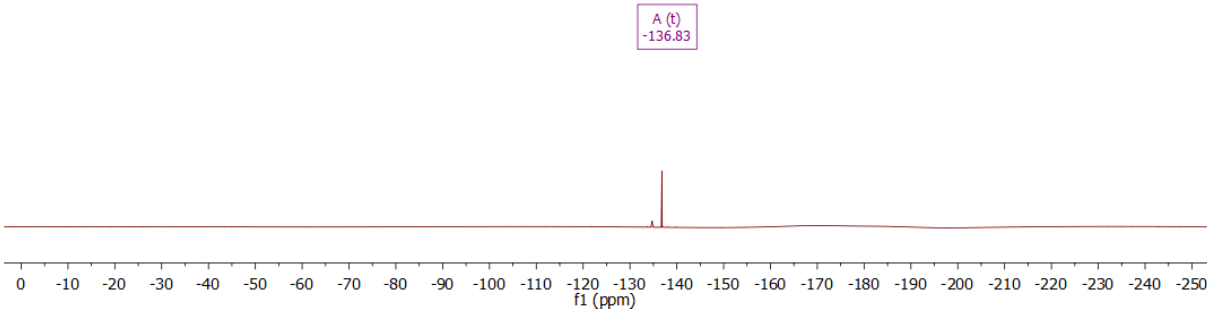
A (t)
-136.83
0 -10 -20 -30 -40 -50 -60 -70 -80 -90 -100 -110 -120 -130 -140 -150 -160 -170 -180 -190 -200 -210 -220 -230 -240 -250
f1 (ppm)

3-(2-bromo-3,4,5-trimethoxybenzyl)-2-methyl-1H-indole [**1i**]:

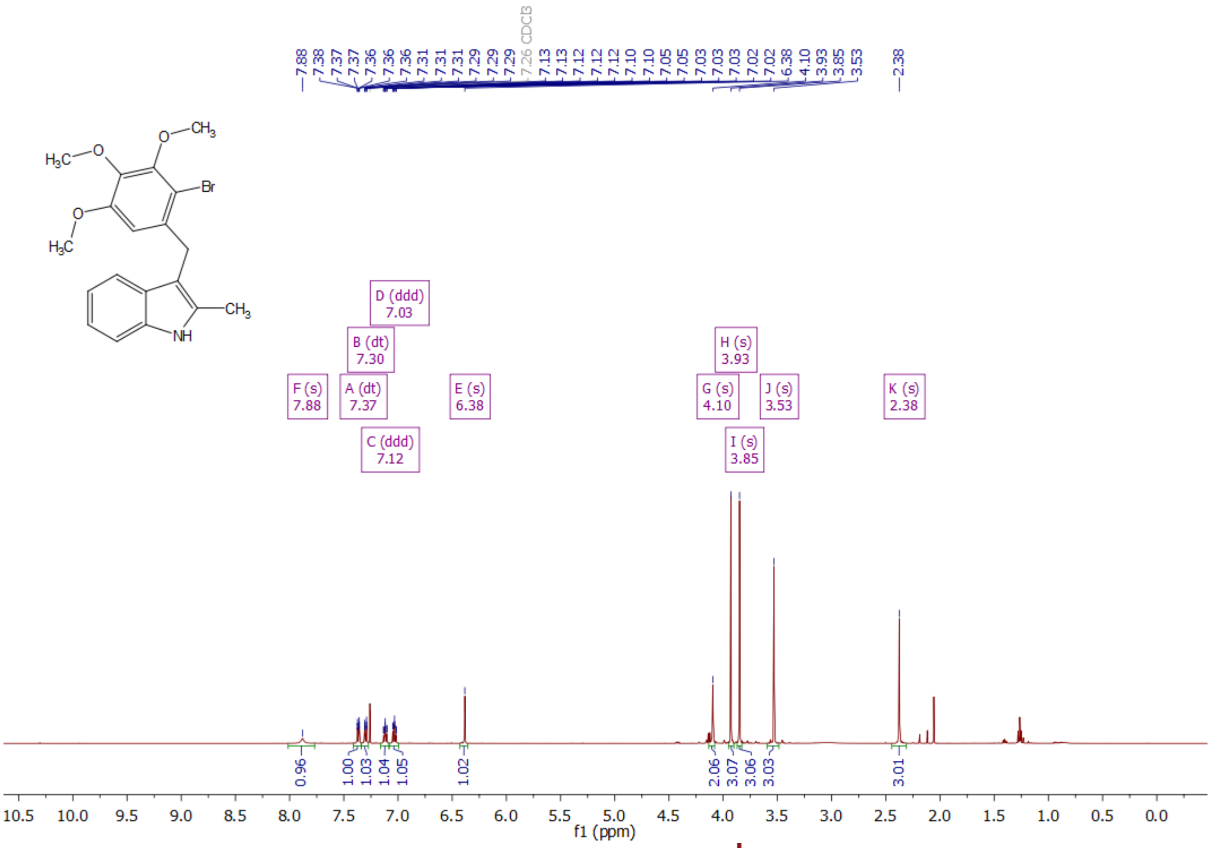

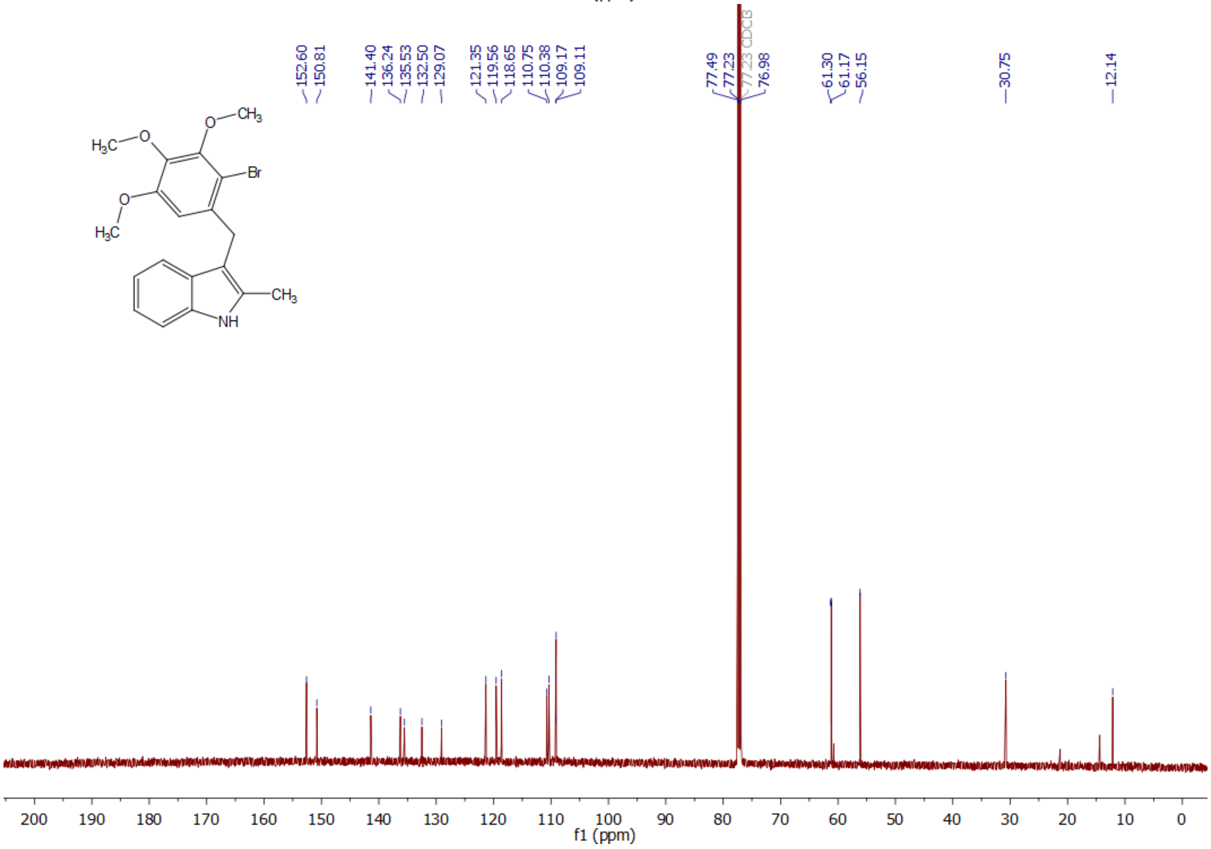

3-((6-bromobenzo[d][1,3]dioxol-5-yl)methyl)-2-methyl-1H-indole **[1j]**:

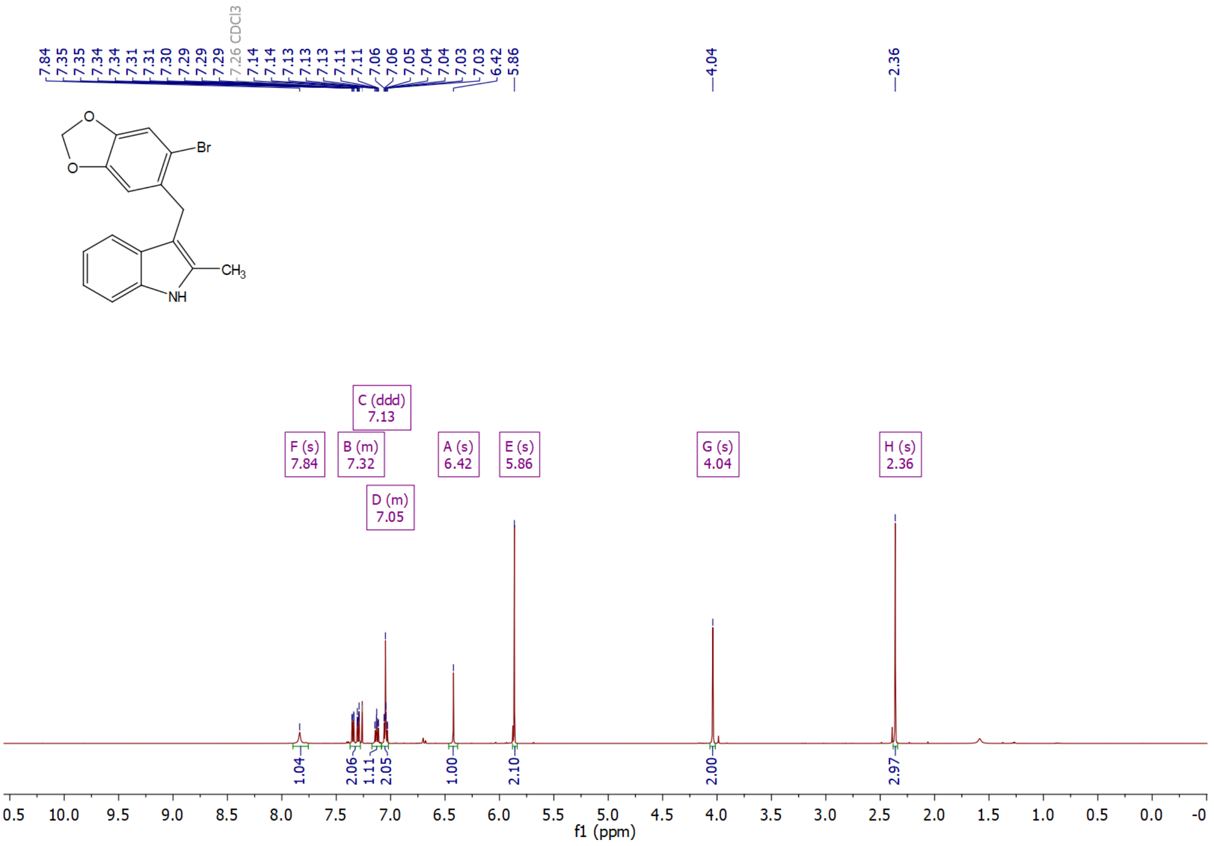

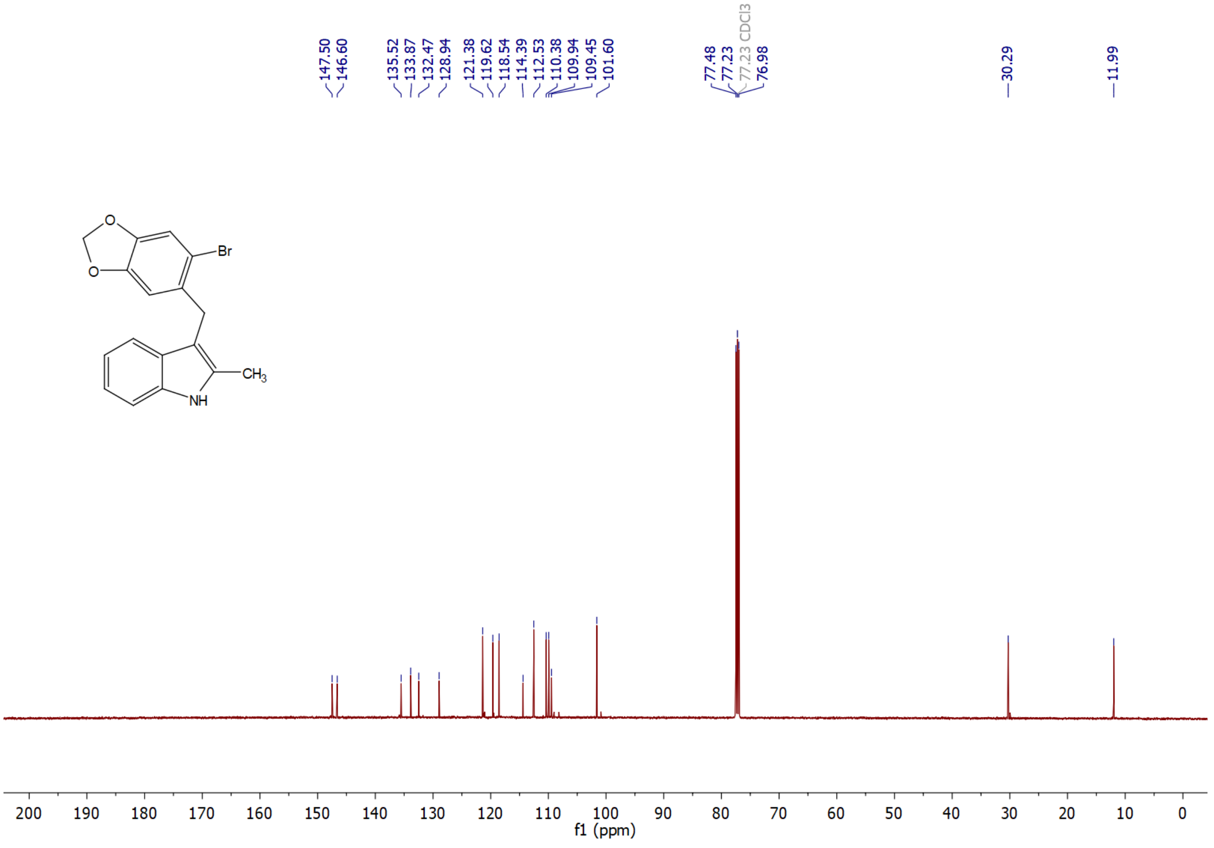

3-((5-bromobenzo[d][1,3]dioxol-4-yl)methyl)-2-methyl-1H-indole [**1k**]:

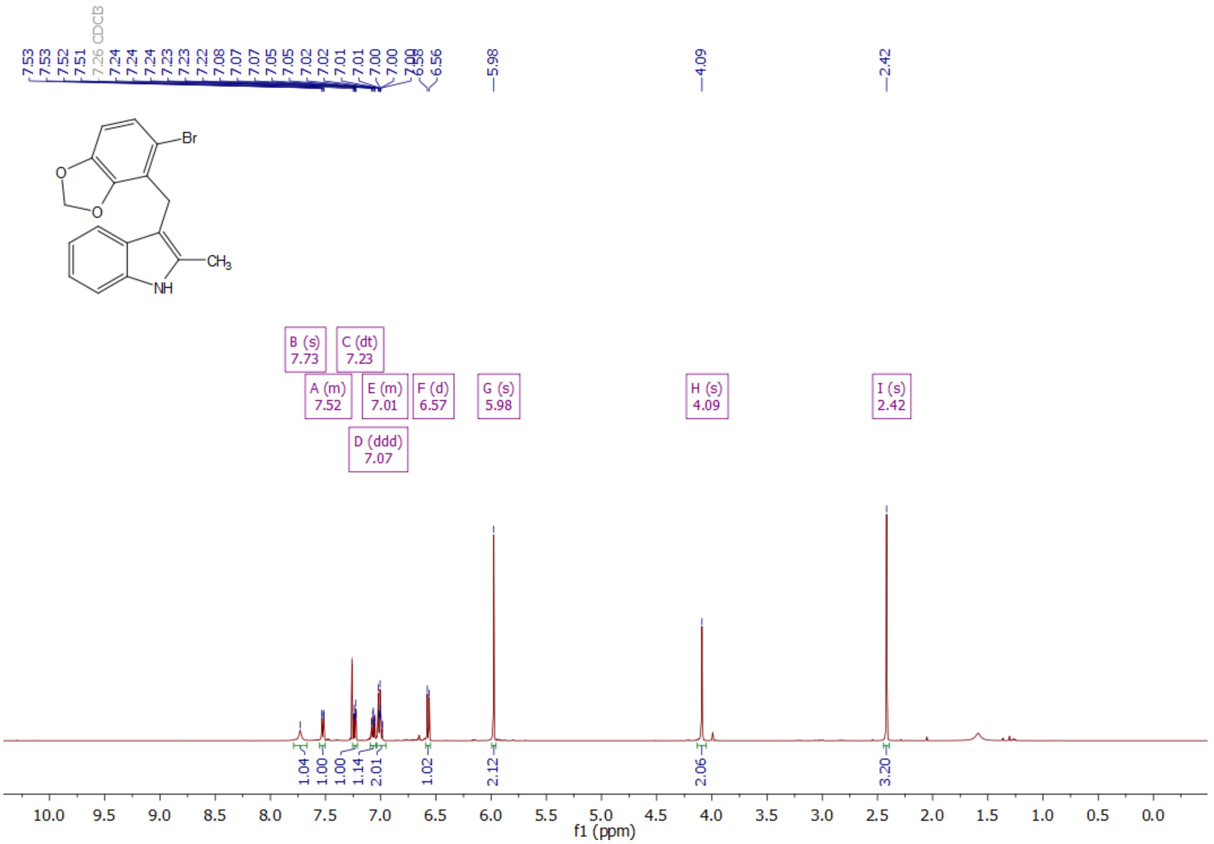

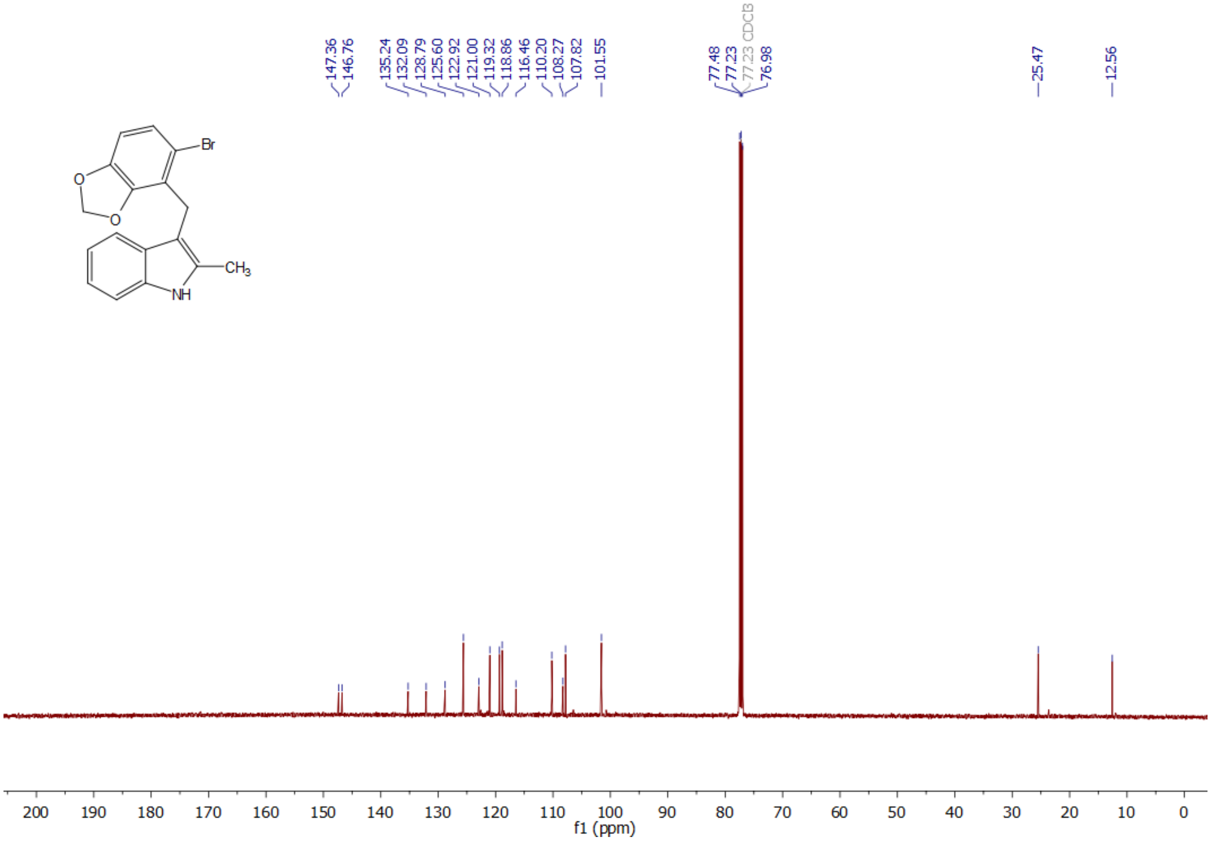

3-((1-bromonaphthalen-2-yl)methyl)-2-methyl-1H-indole [**1l**]:

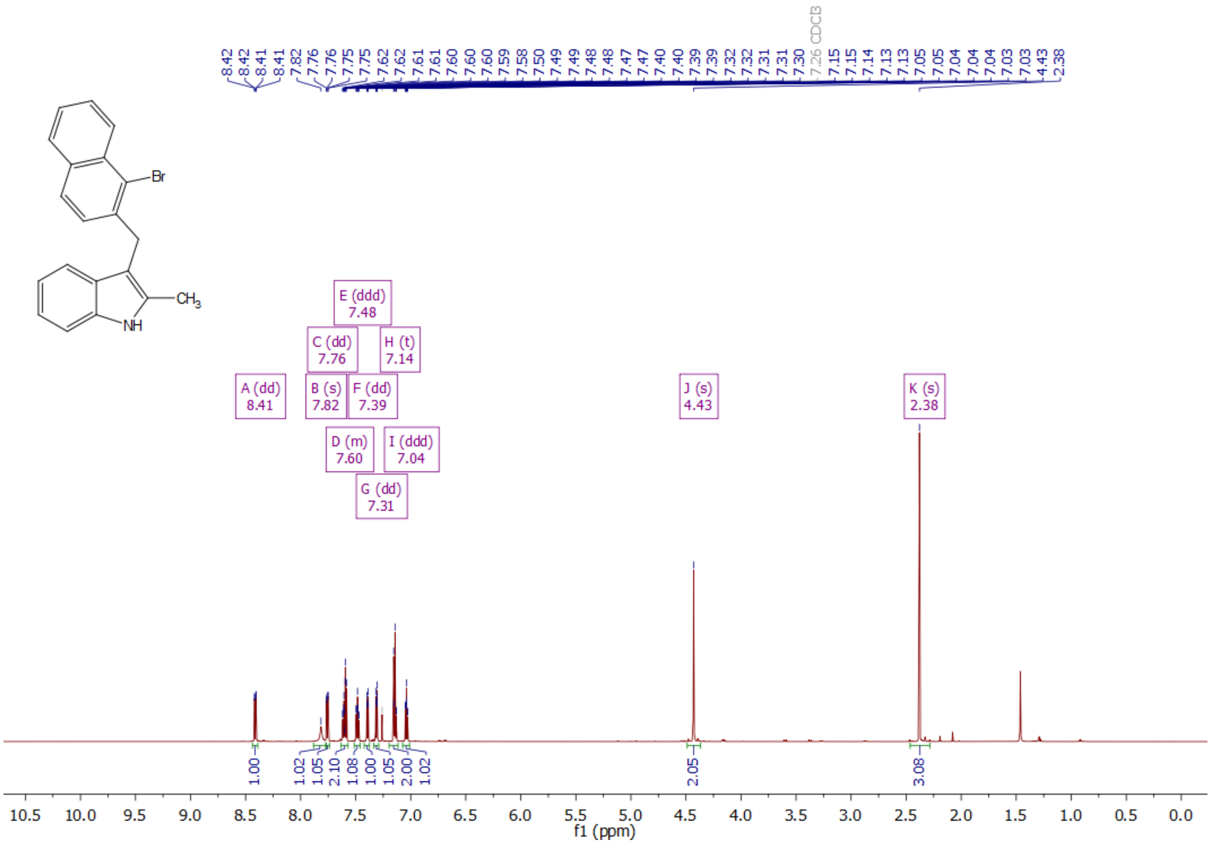

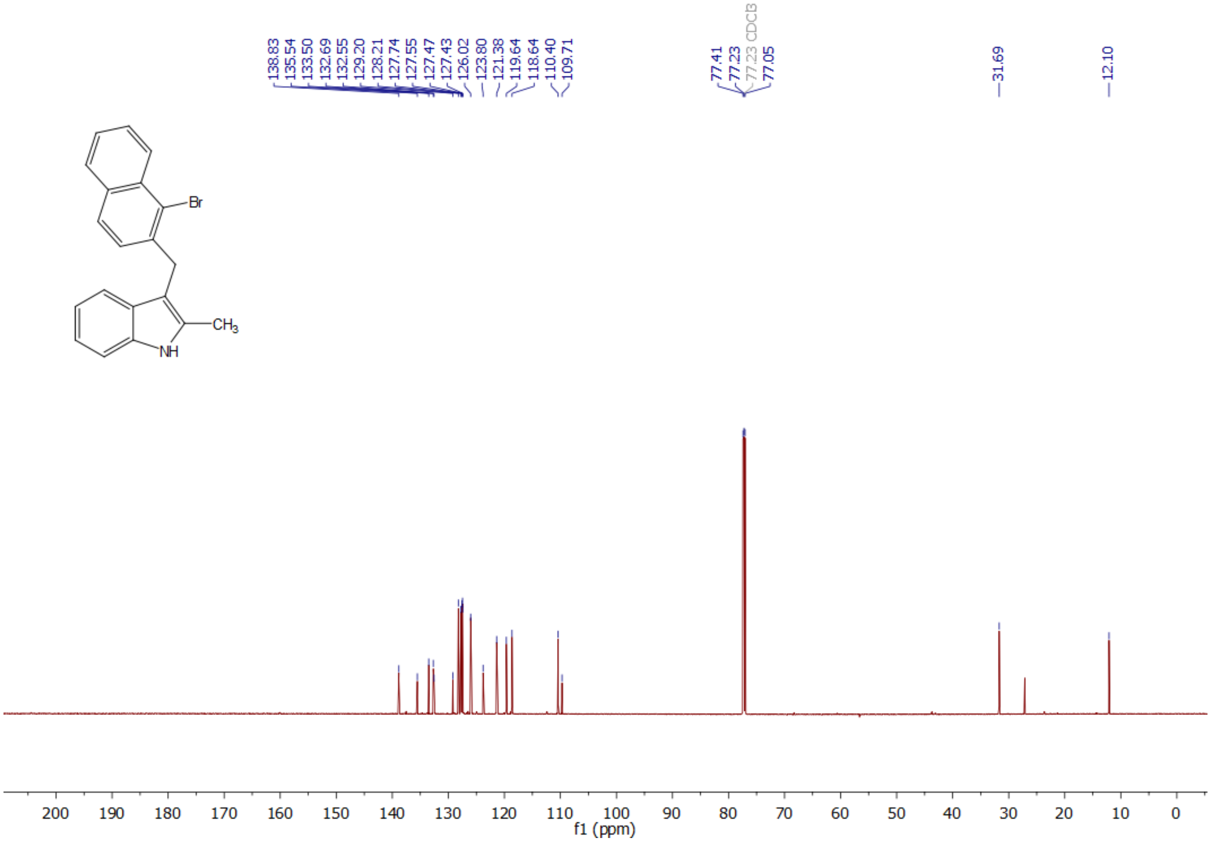

3-(2-bromobenzyl)-5-fluoro-2-methyl-1H-indole [**1m**]:

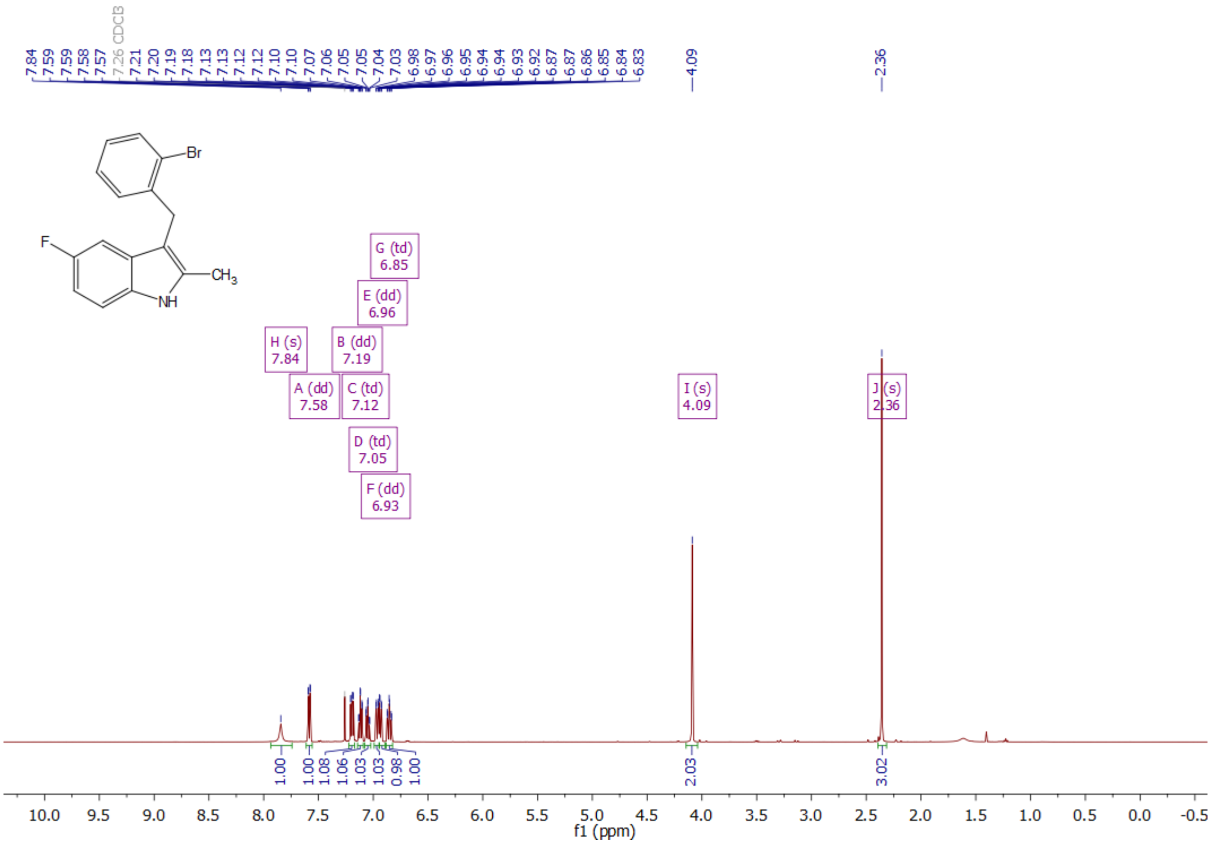

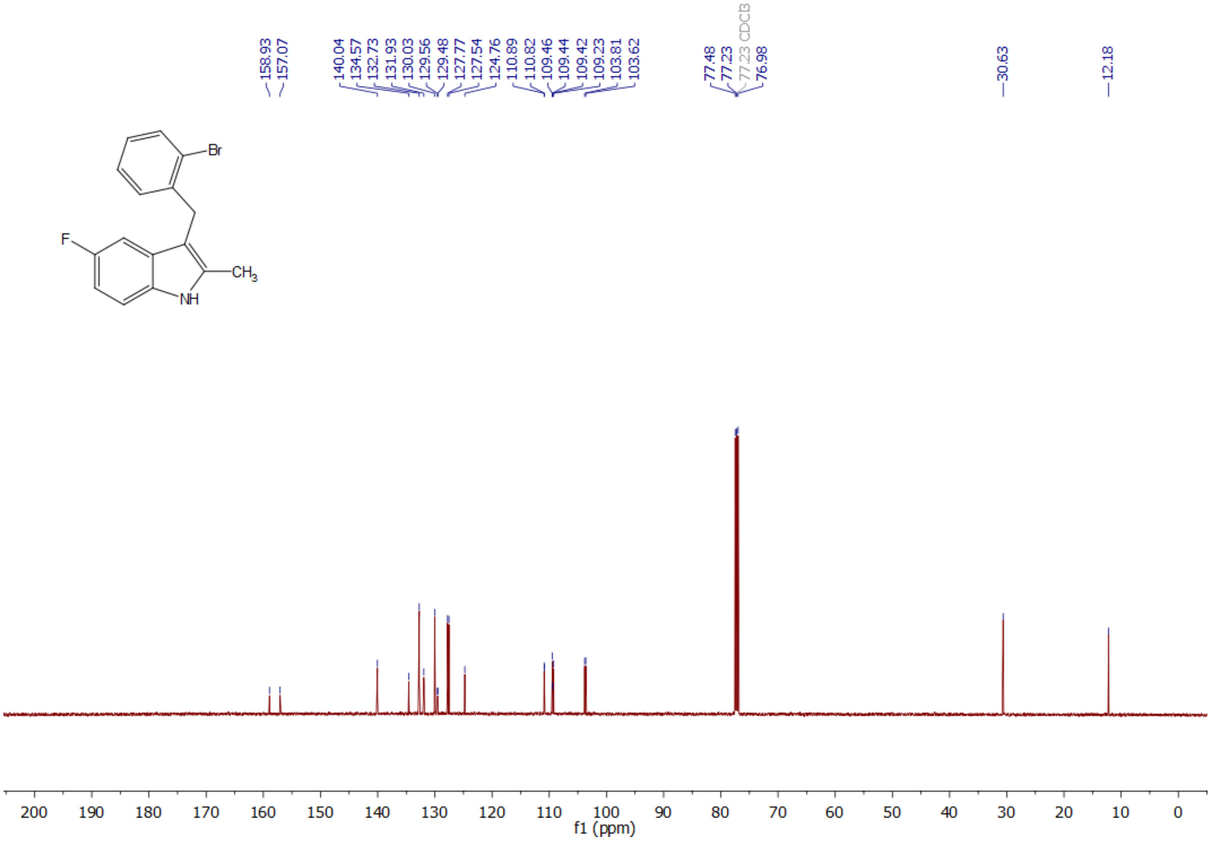

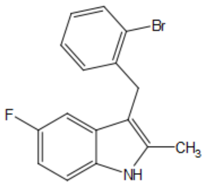
Br
F
CH3
NH

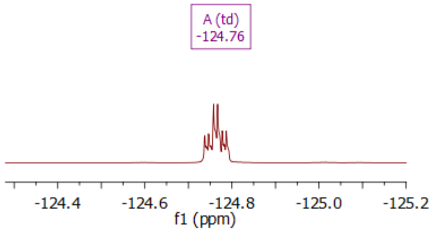
A (td)
-124.76
-124.4
-124.6
-124.8
-125.0
-125.2
f1 (ppm)

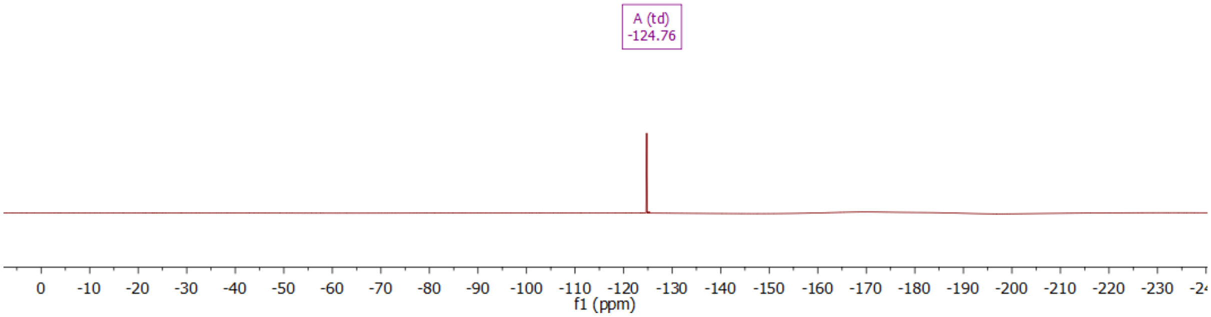
A (td)
-124.76
0 -10 -20 -30 -40 -50 -60 -70 -80 -90 -100 -110 -120 -130 -140 -150 -160 -170 -180 -190 -200 -210 -220 -230
f1 (ppm)

3-(2-bromobenzyl)-5-chloro-2-methyl-1H-indole [**1n**]:

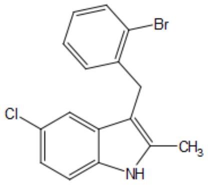

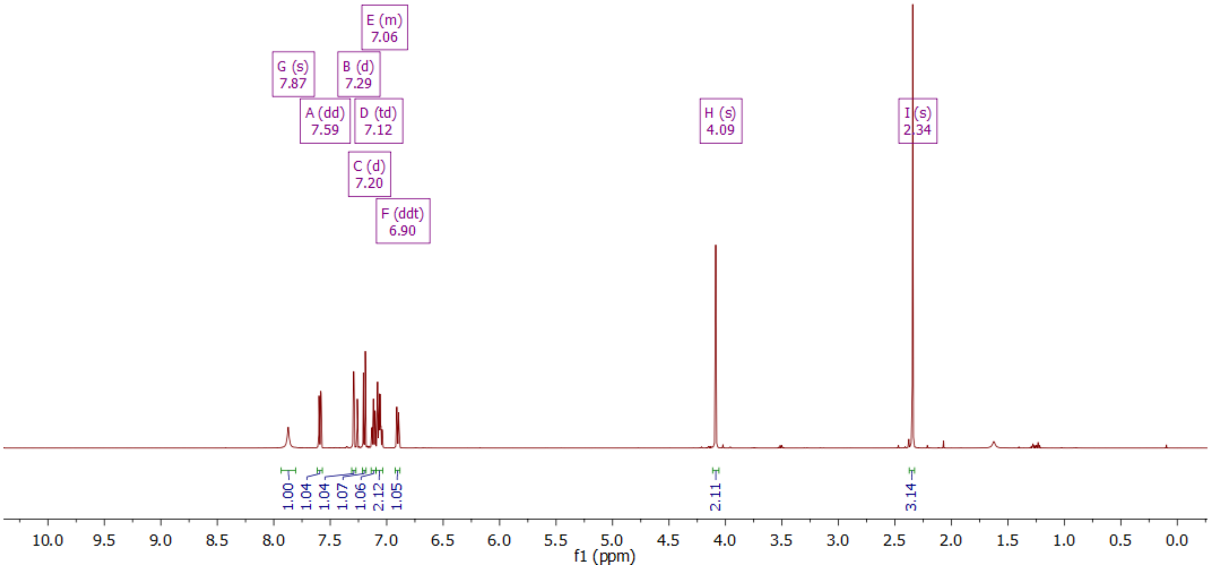

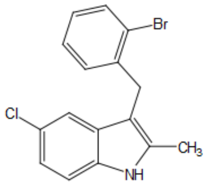

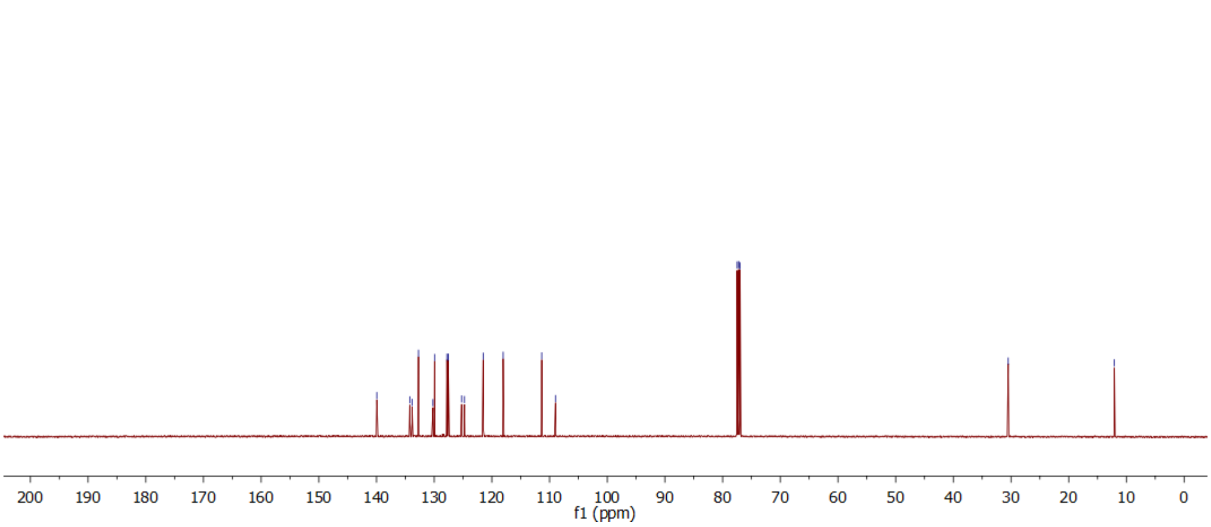

3-(2-bromobenzyl)-2,5-dimethyl-1H-indole [**1o**]:

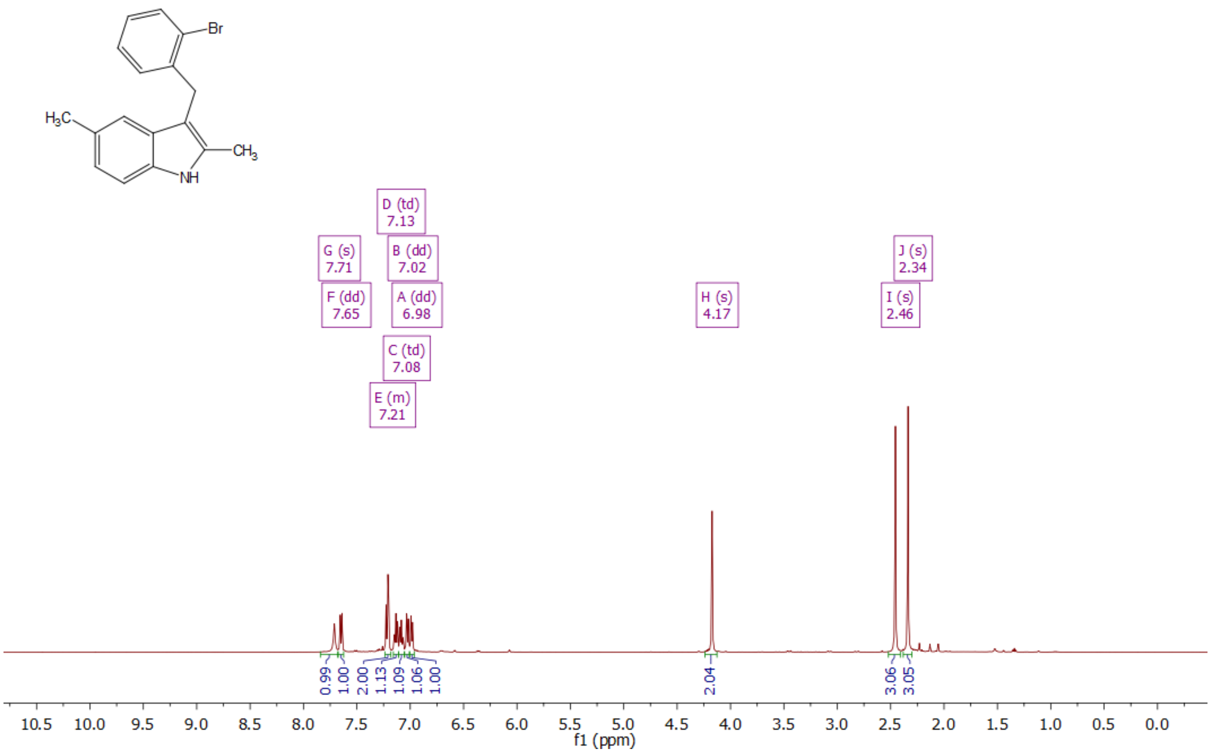

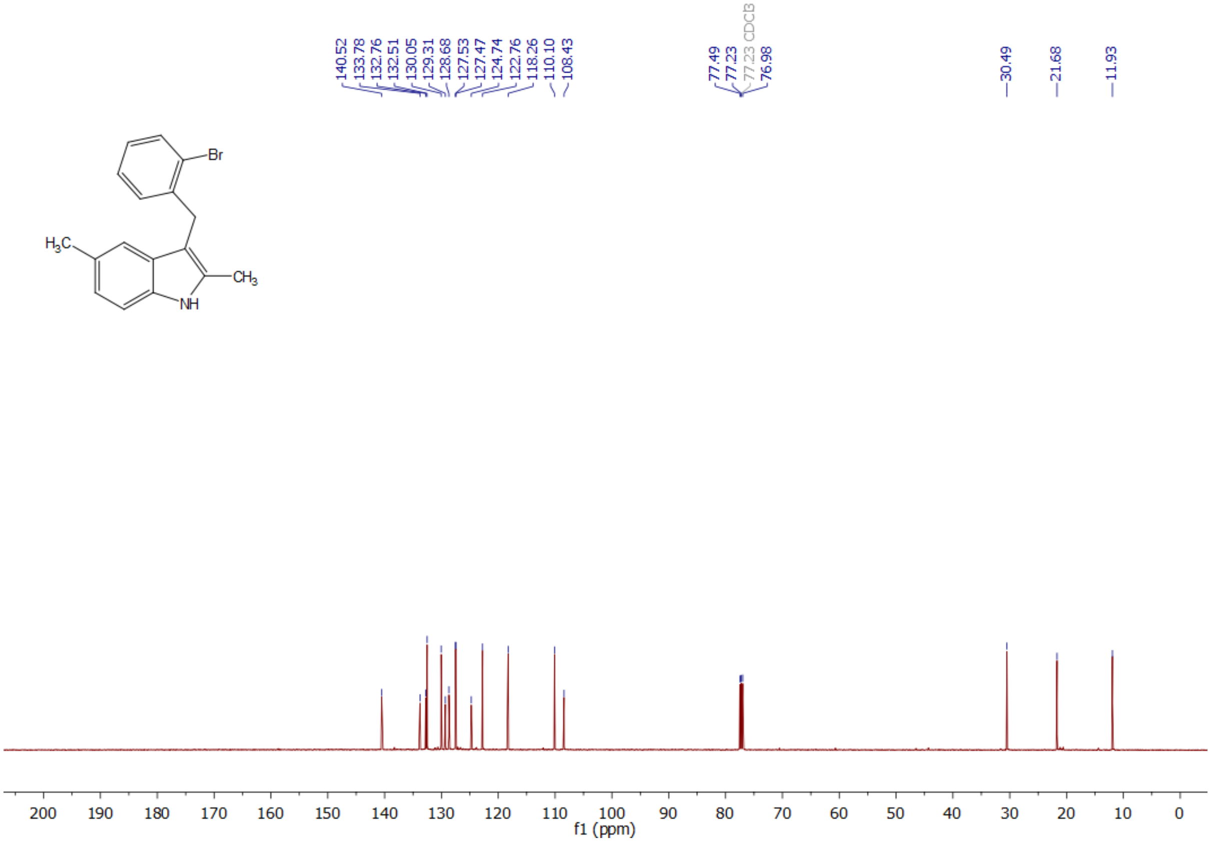

3-(2-bromo-4-chlorobenzyl)-5-fluoro-2-methyl-1H-indole [**1p**]:

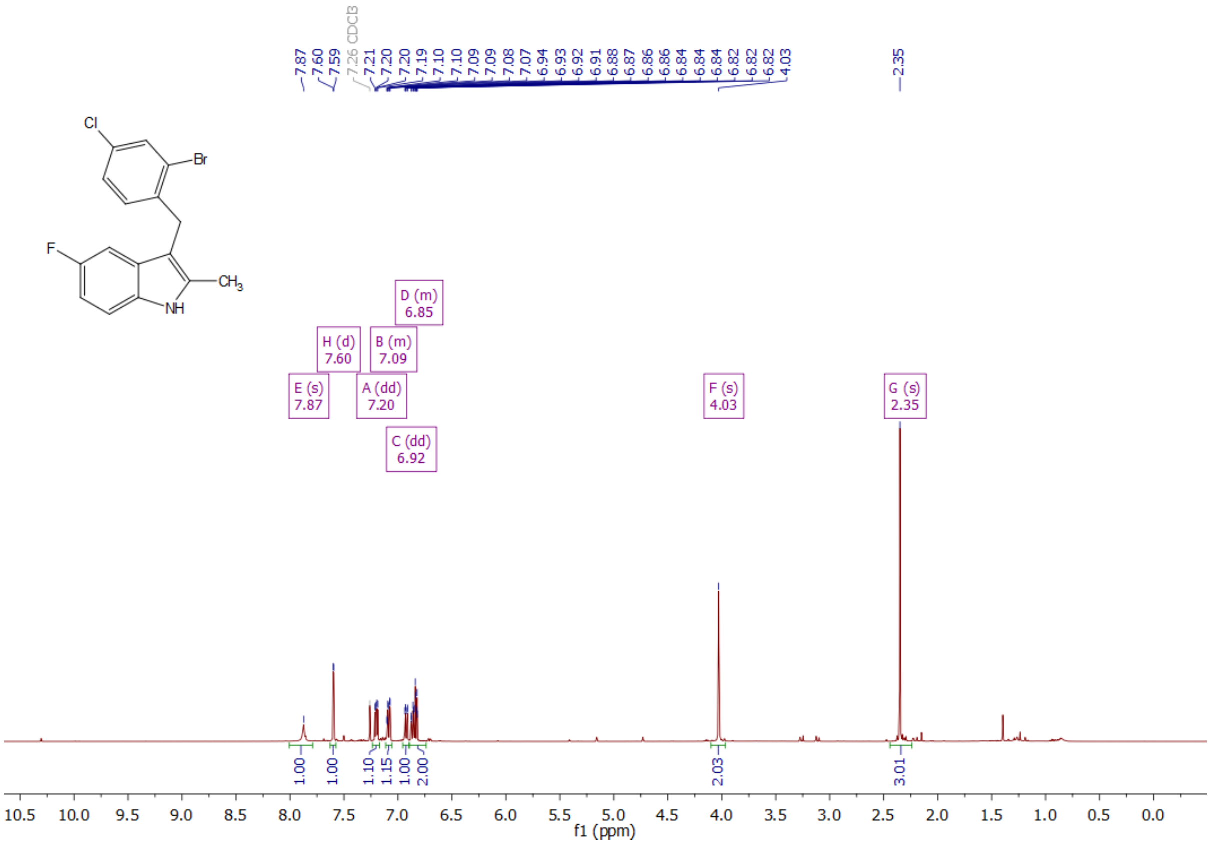

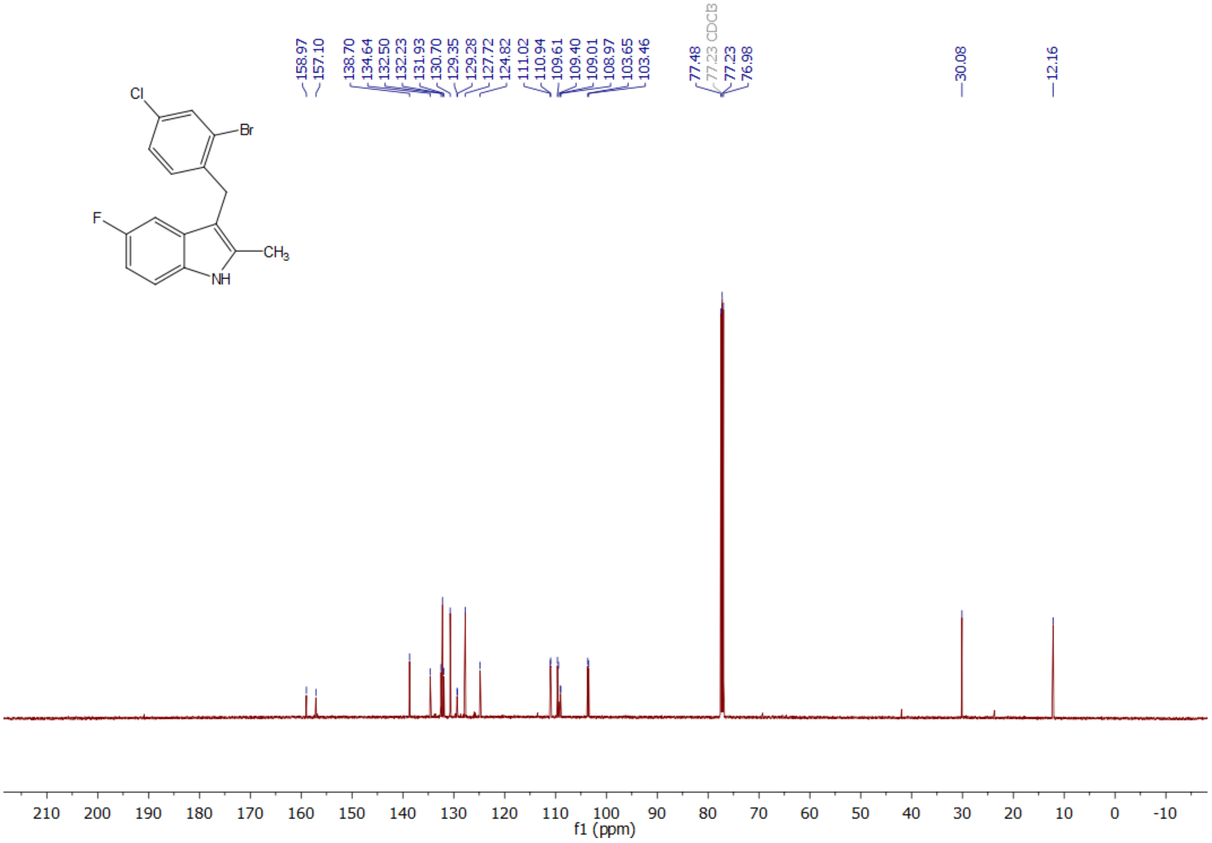

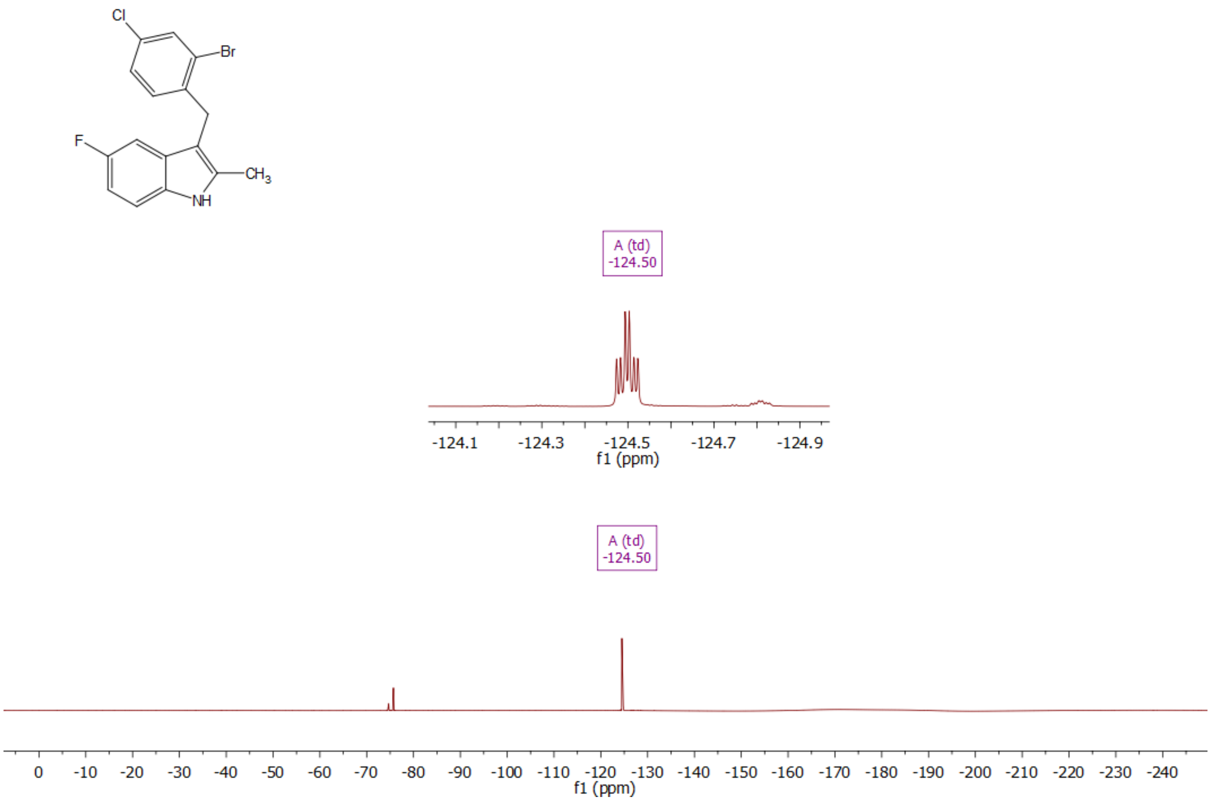

Cl
Br
F
CH3
NH
A (td)
-124.50
-124.1
-124.3
-124.5
-124.7
-124.9
f1 (ppm)
A (td)
-124.50
0
-10
-20
-30
-40
-50
-60
-70
-80
-90
-100
-110
-120
-130
-140
-150
-160
-170
-180
-190
-200
-210
-220
-230
-240
f1 (ppm)

***3-(2-bromo-4-methoxybenzyl)-5-fluoro-2-methyl-1H-indole* [1q]:**

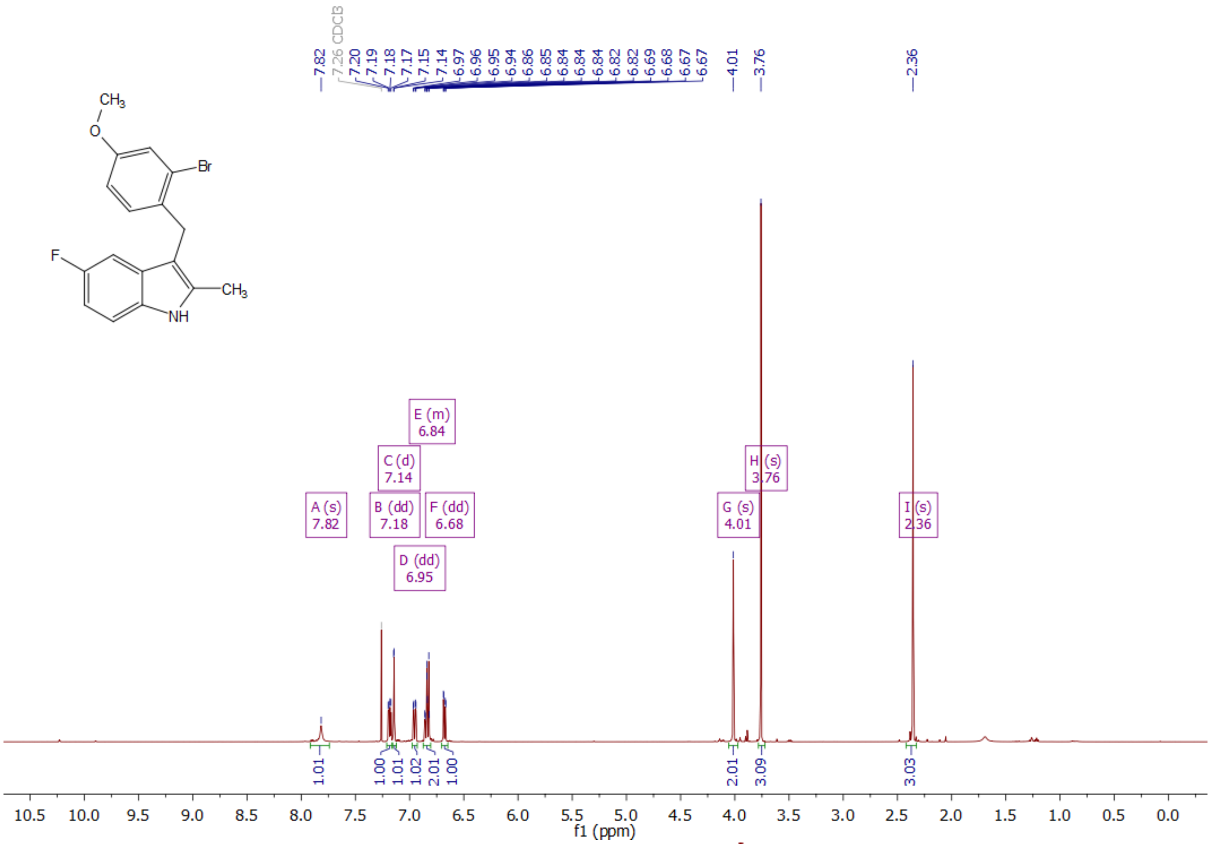

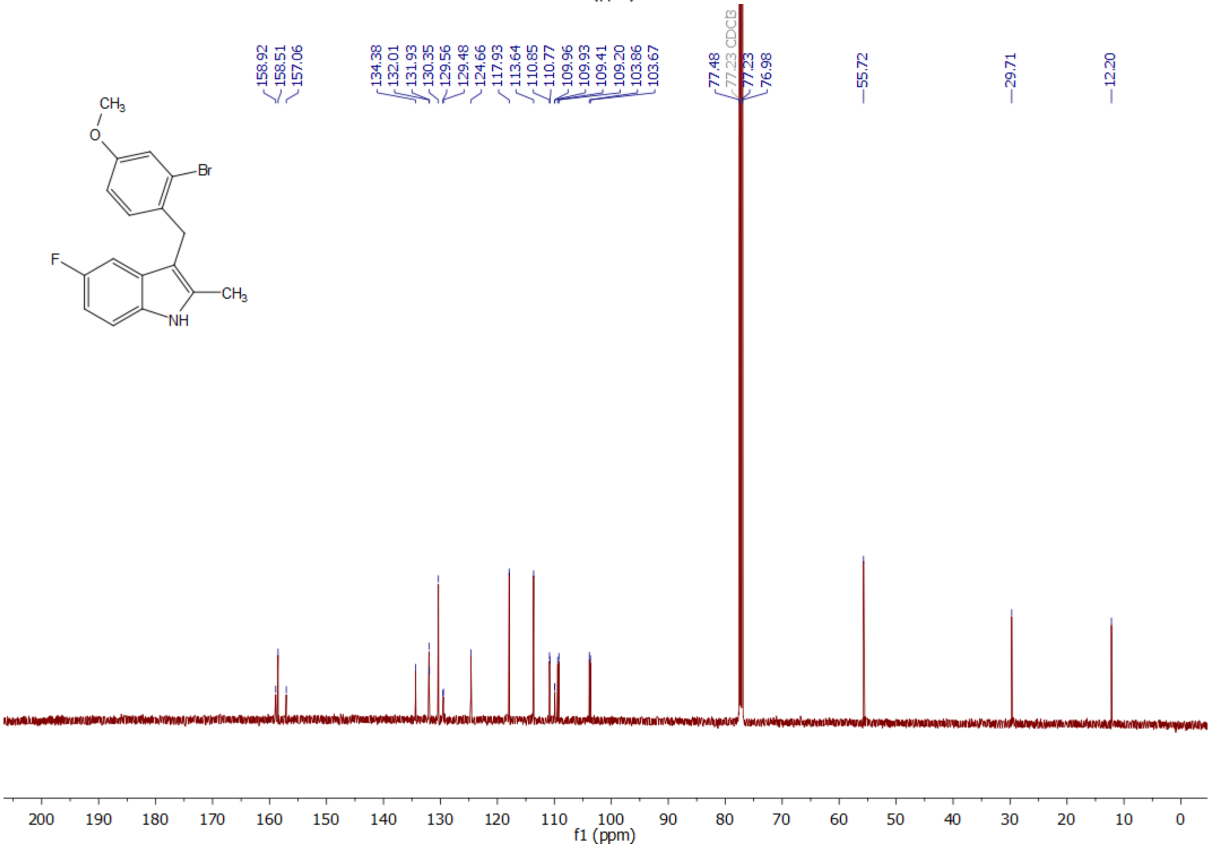

3-(2-bromo-5-fluorobenzyl)-5-fluoro-2-methyl-1H-indole [**1r**]:

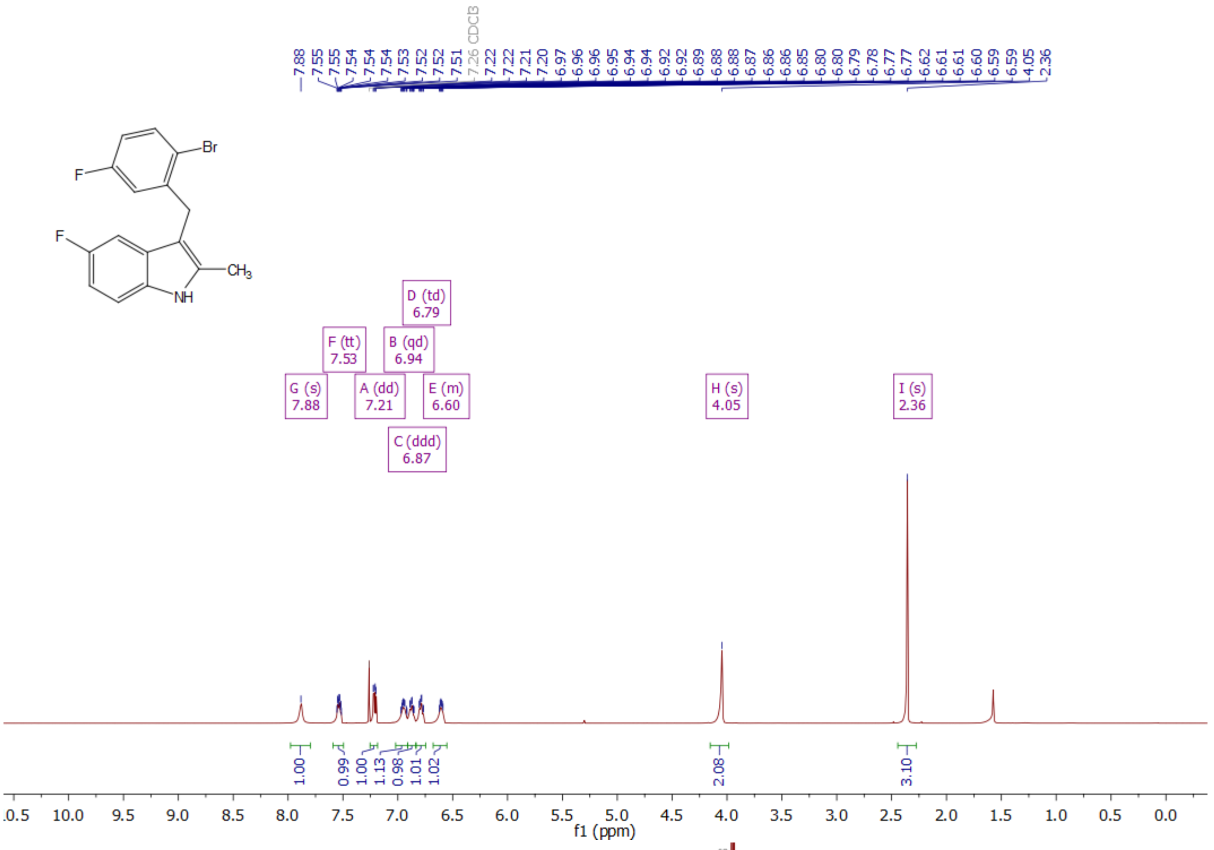

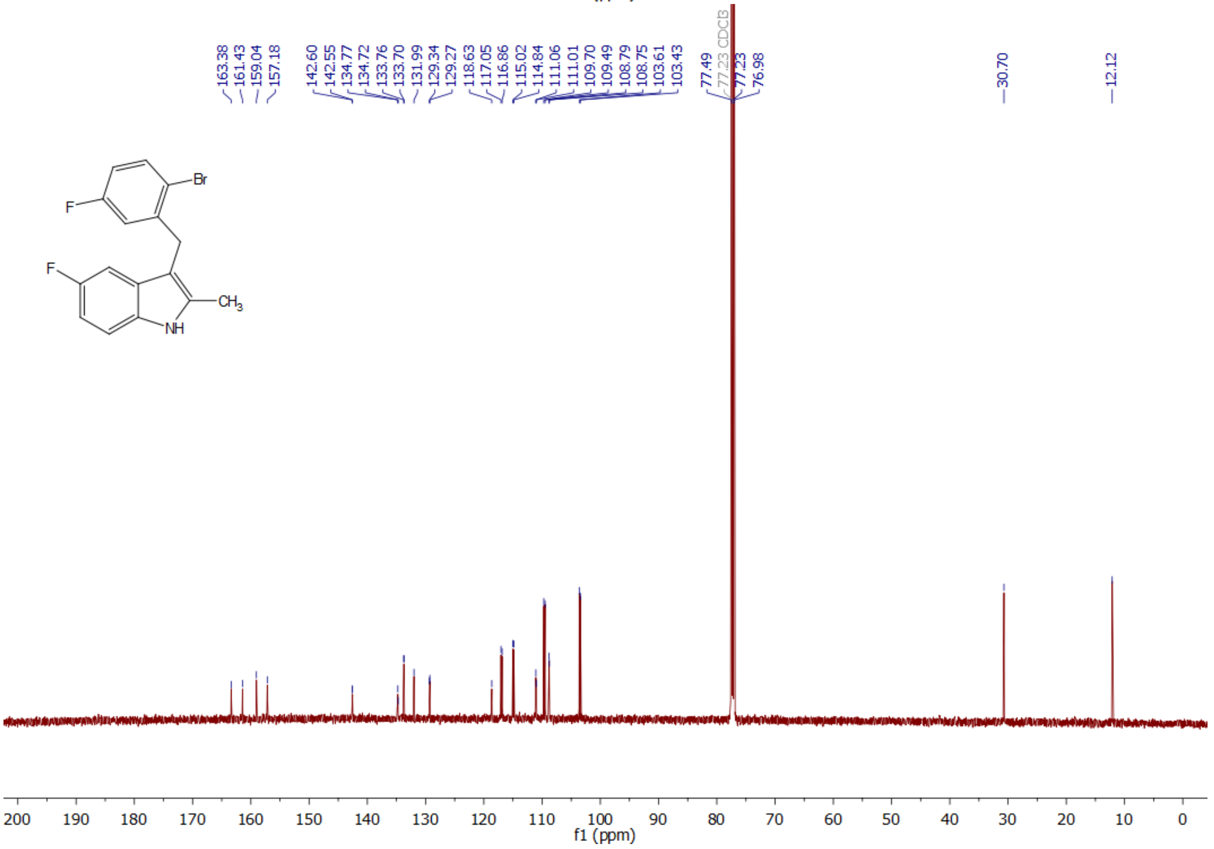

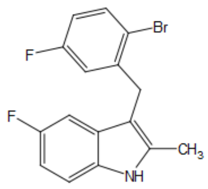

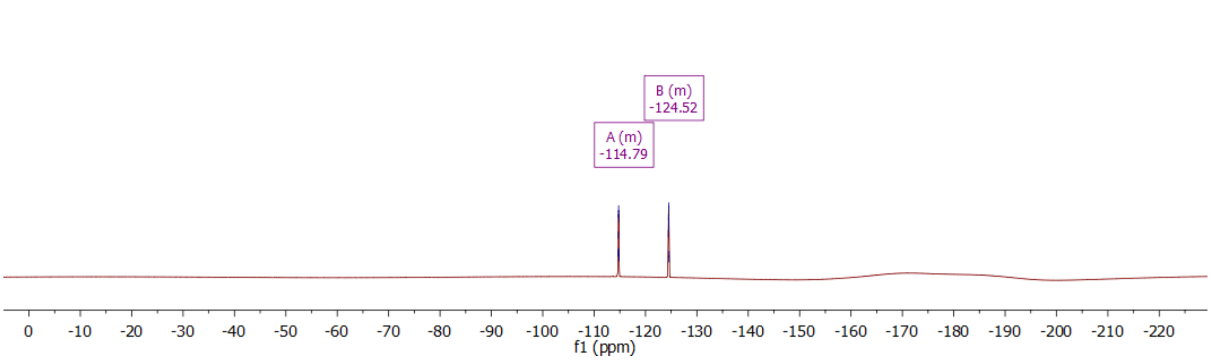
-114.68
-114.71
-114.75
-114.77
-114.78
-114.80
-114.83
-114.85
-114.85
-124.46
-124.49
-124.51
-124.53
-124.54
-124.56
-124.58
-124.58
B (m)
-124.52
A (m)
-114.79
0 -10 -20 -30 -40 -50 -60 -70 -80 -90 -100 -110 -120 -130 -140 -150 -160 -170 -180 -190 -200 -210 -220
f1 (ppm)

3-(2-bromo-5-methoxybenzyl)-5-fluoro-2-methyl-1H-indole **[1s]**:

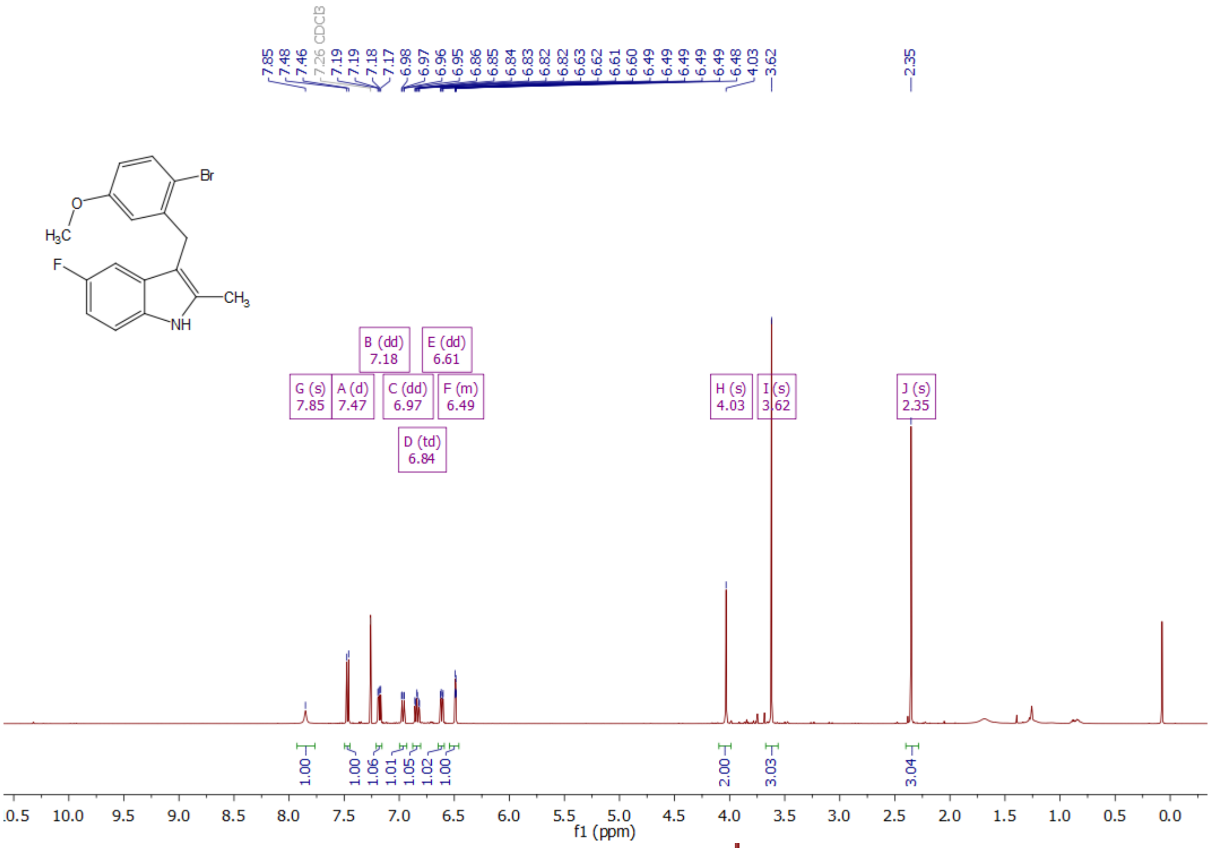

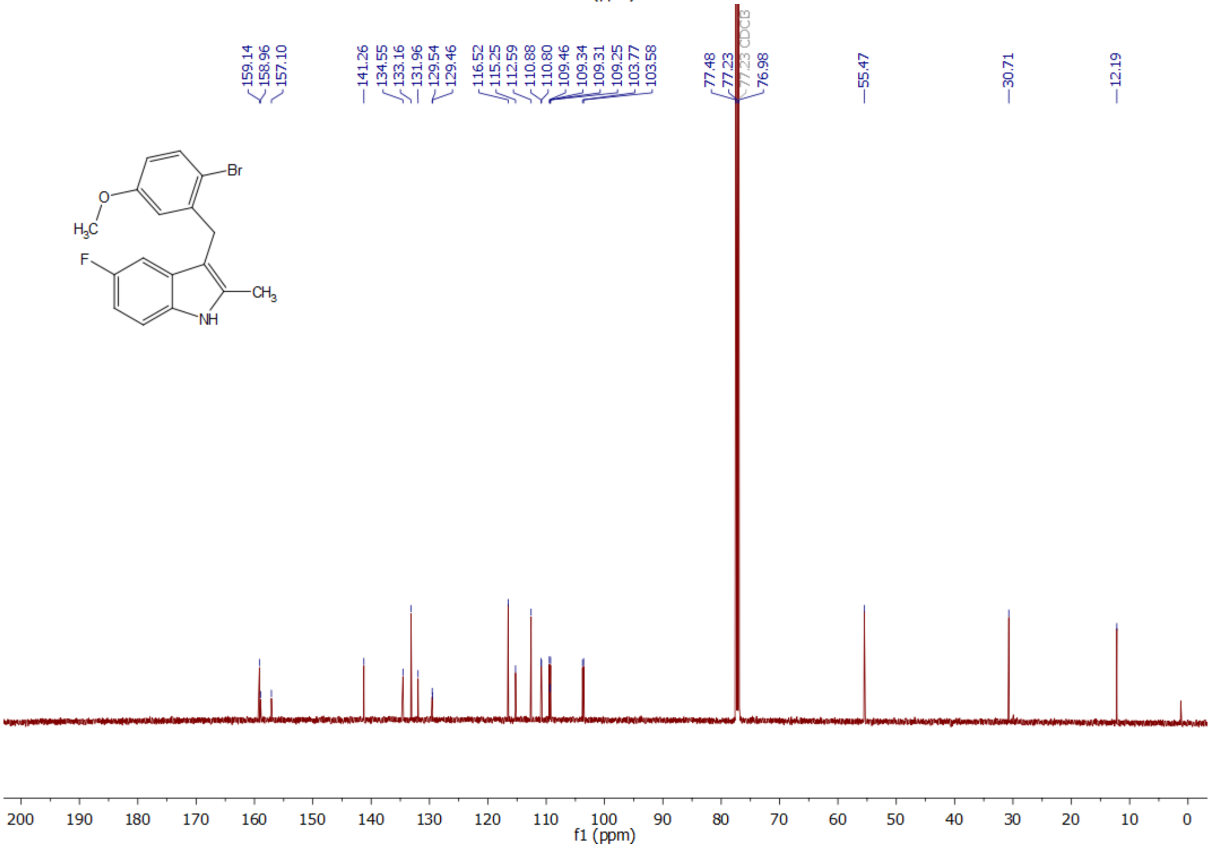

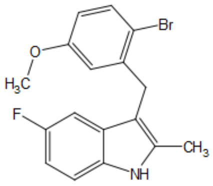
Br
O
H3C
F
CH3
NH

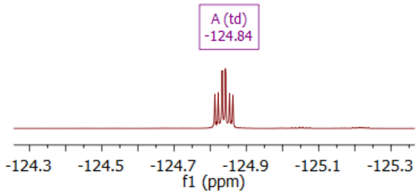
A (td)
-124.84
-124.3
-124.5
-124.7
-124.9
-125.1
-125.3
f1 (ppm)

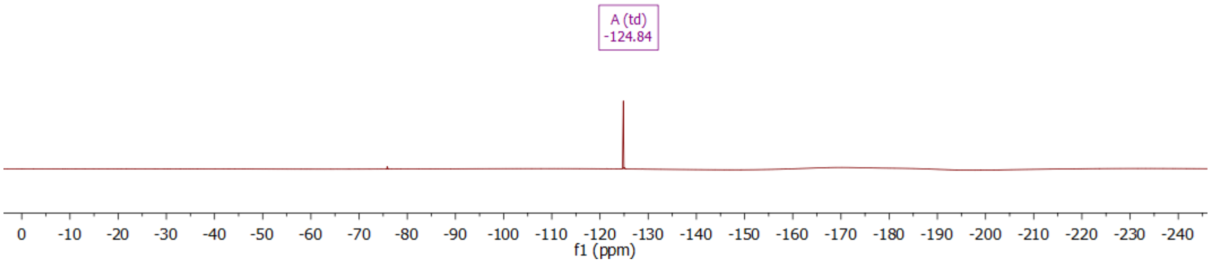
A (td)
-124.84
0 -10 -20 -30 -40 -50 -60 -70 -80 -90 -100 -110 -120 -130 -140 -150 -160 -170 -180 -190 -200 -210 -220 -230 -240
f1 (ppm)

3-(2-bromo-4,5-dimethoxybenzyl)-5-fluoro-2-methyl-1H-indole **[1t]**:

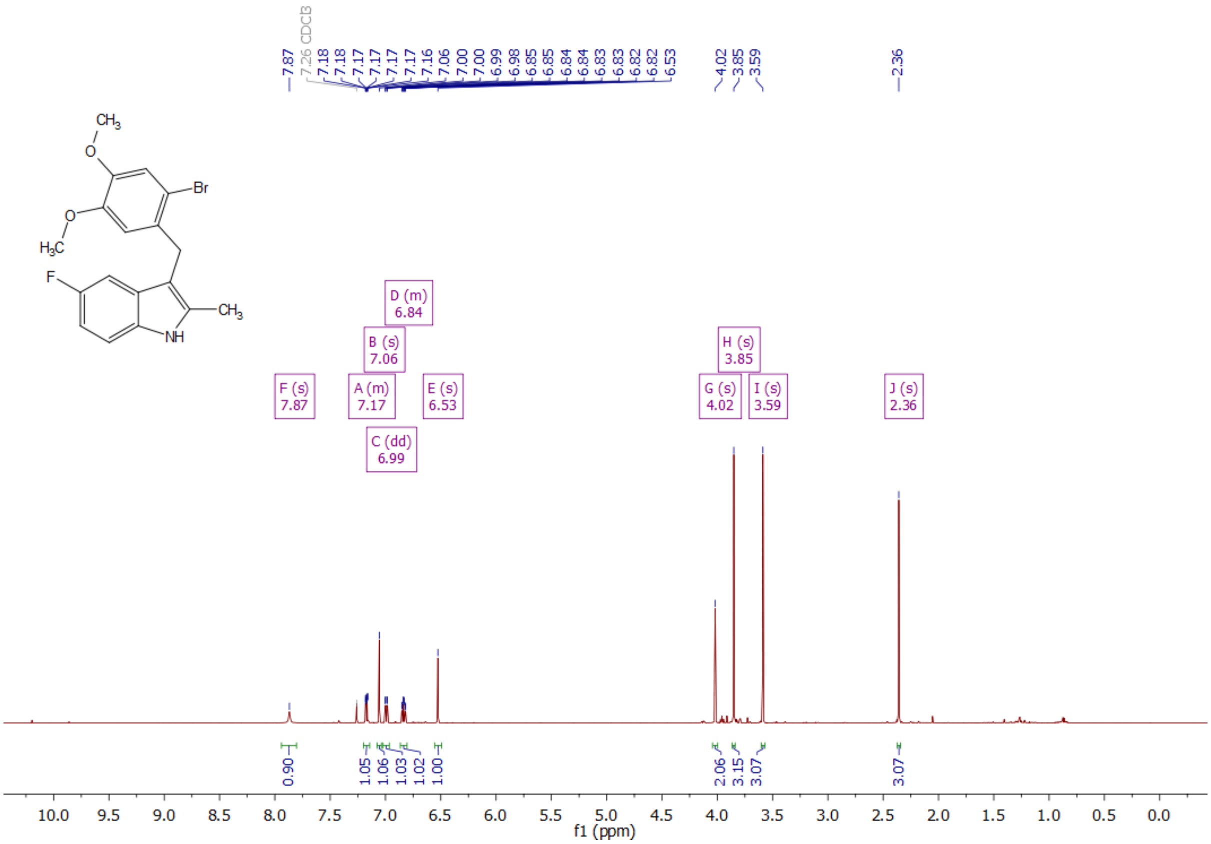

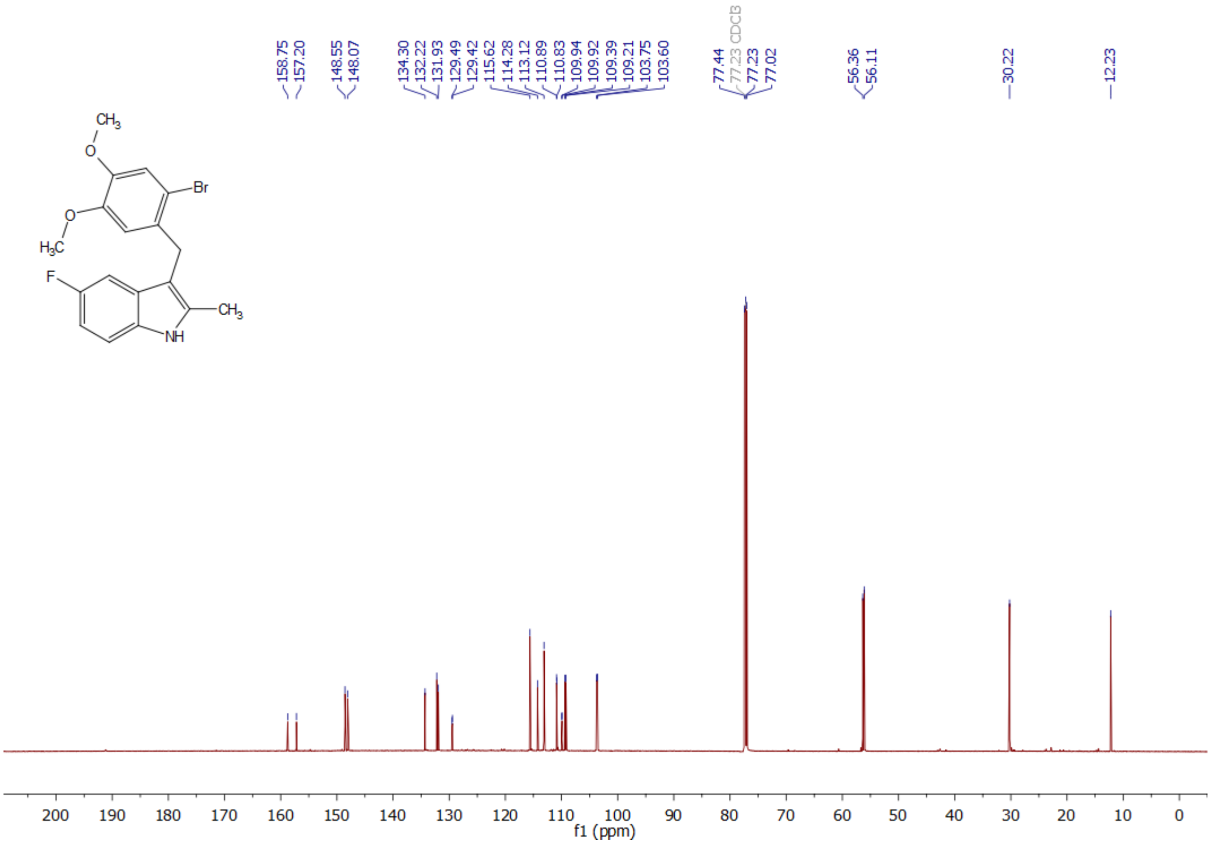

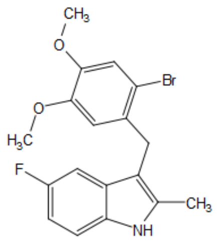

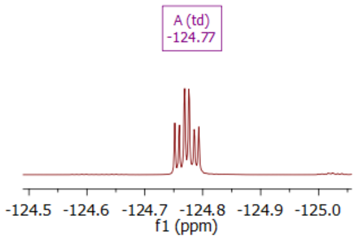
A (td)
-124.77
-124.5 -124.6 -124.7 -124.8 -124.9 -125.0
f1 (ppm)

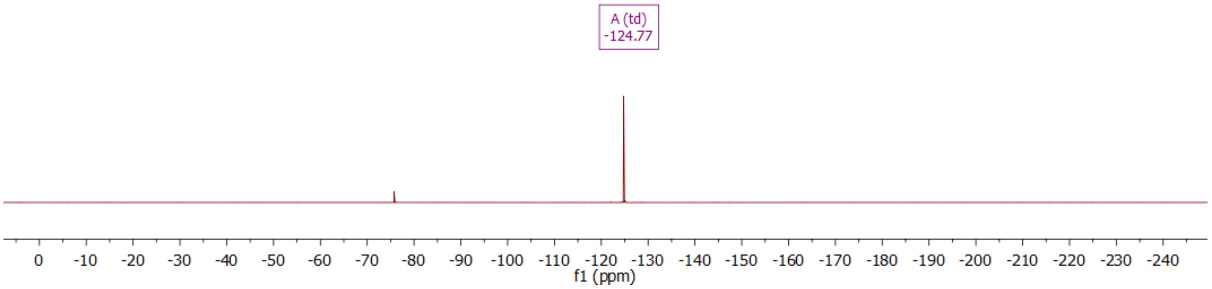
A (td)
-124.77
0 -10 -20 -30 -40 -50 -60 -70 -80 -90 -100 -110 -120 -130 -140 -150 -160 -170 -180 -190 -200 -210 -220 -230 -240
f1 (ppm)

3-((6-bromobenzo[d][1,3]dioxol-5-yl)methyl)-5-fluoro-2-methyl-1H-indole [**1u**]:

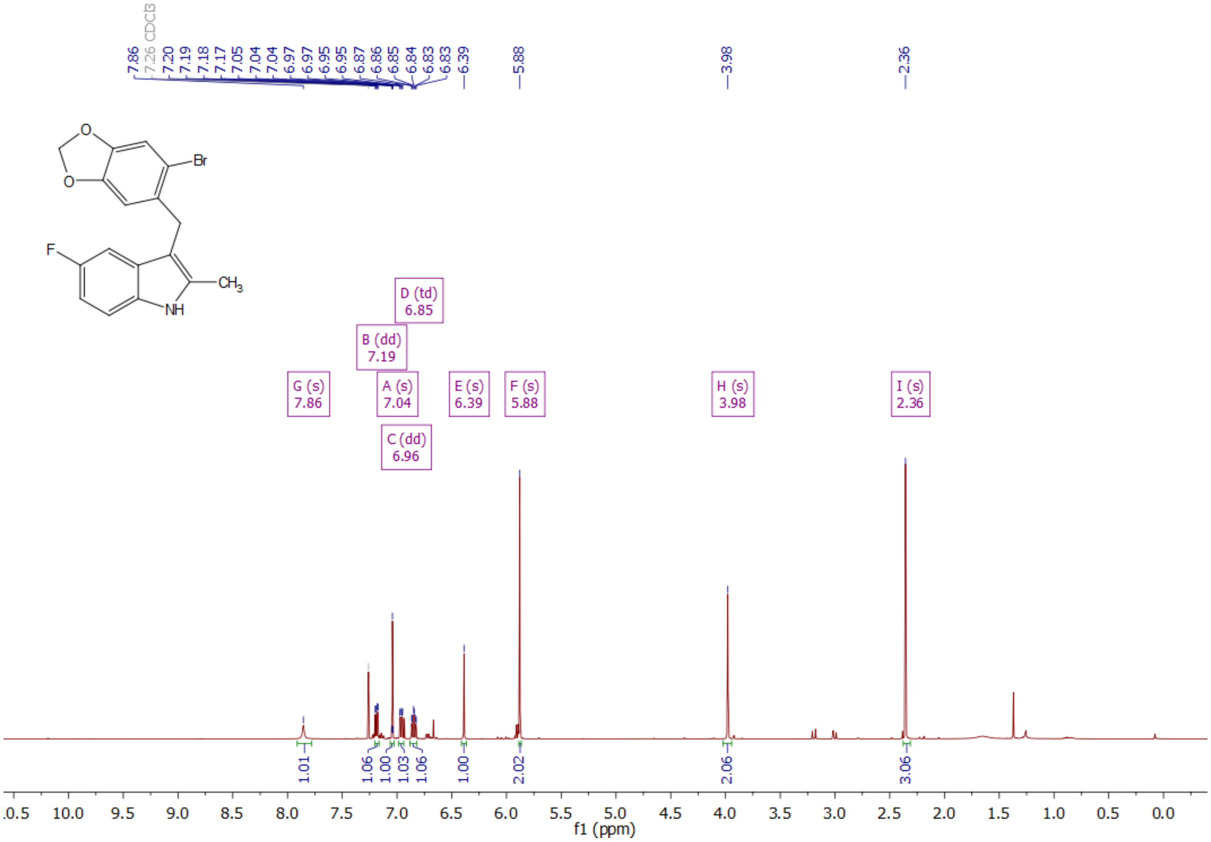

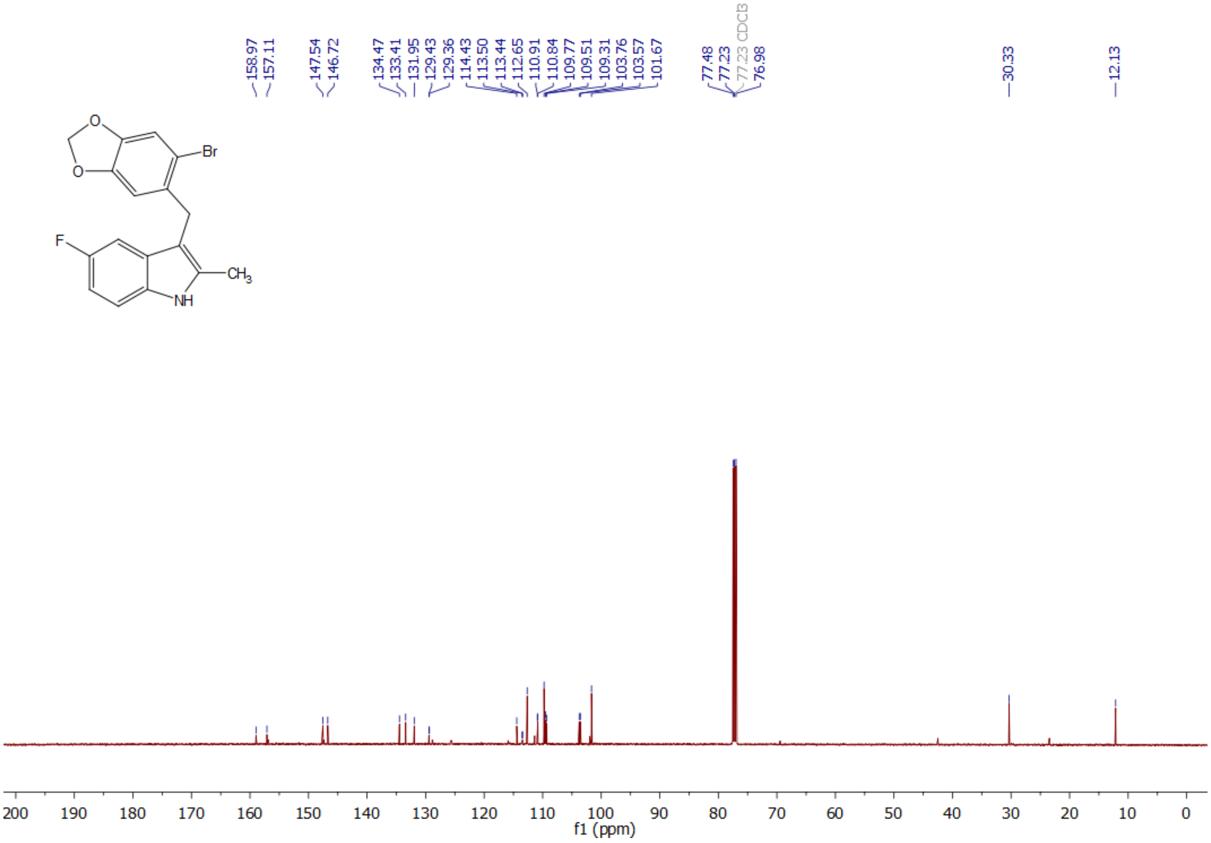

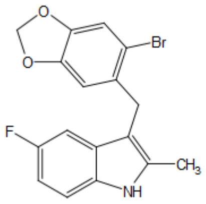

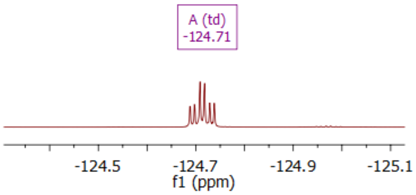

A (td)
-124.71
-124.5
-124.7
-124.9
-125.1
f1 (ppm)

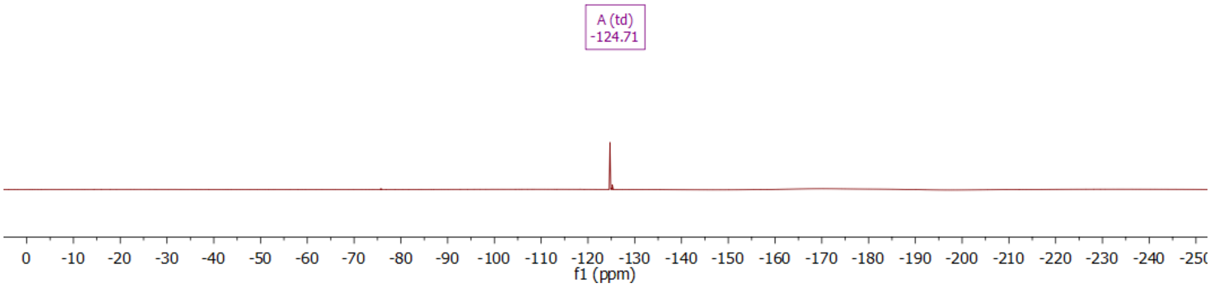

A (td)
-124.71
0
-10
-20
-30
-40
-50
-60
-70
-80
-90
-100
-110
-120
-130
-140
-150
-160
-170
-180
-190
-200
-210
-220
-230
-240
f1 (ppm)

3-(2-bromo-5-fluorobenzyl)-5-chloro-2-methyl-1H-indole [**1v**]:

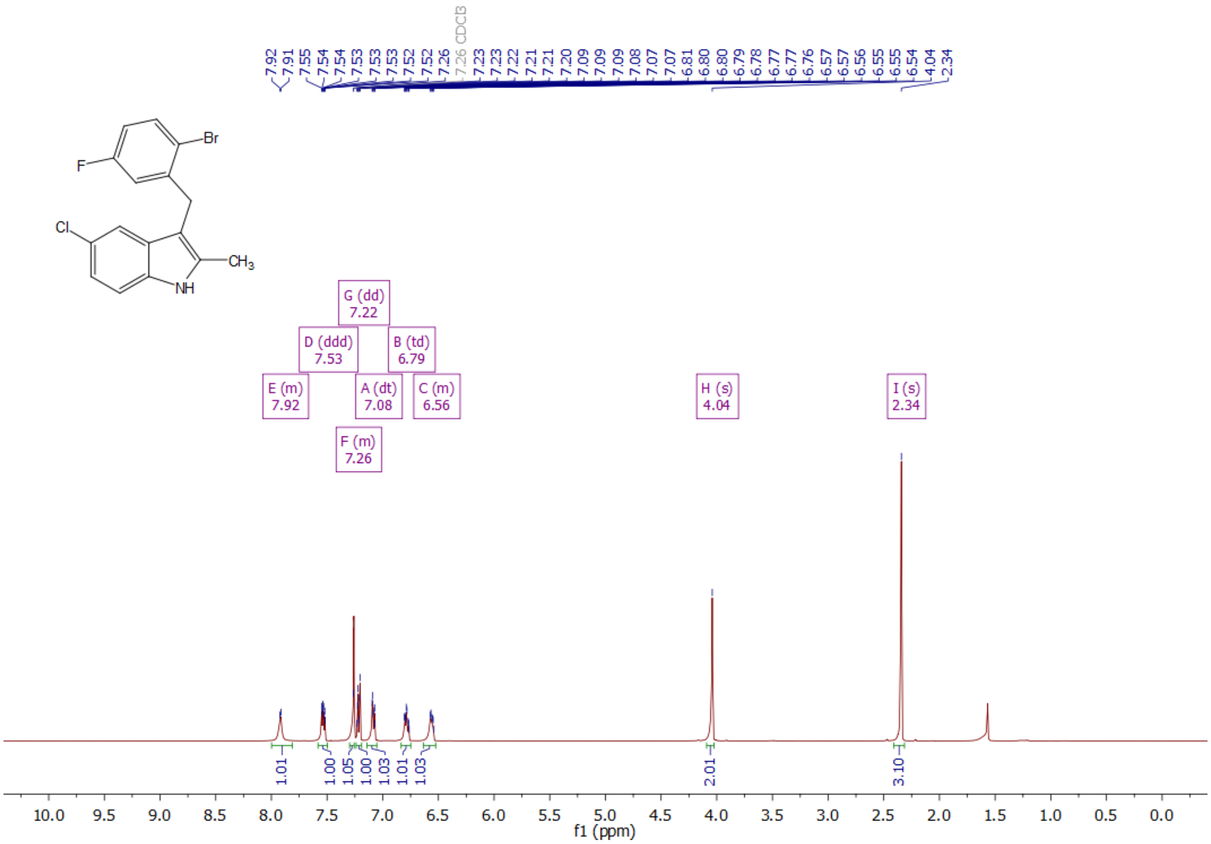

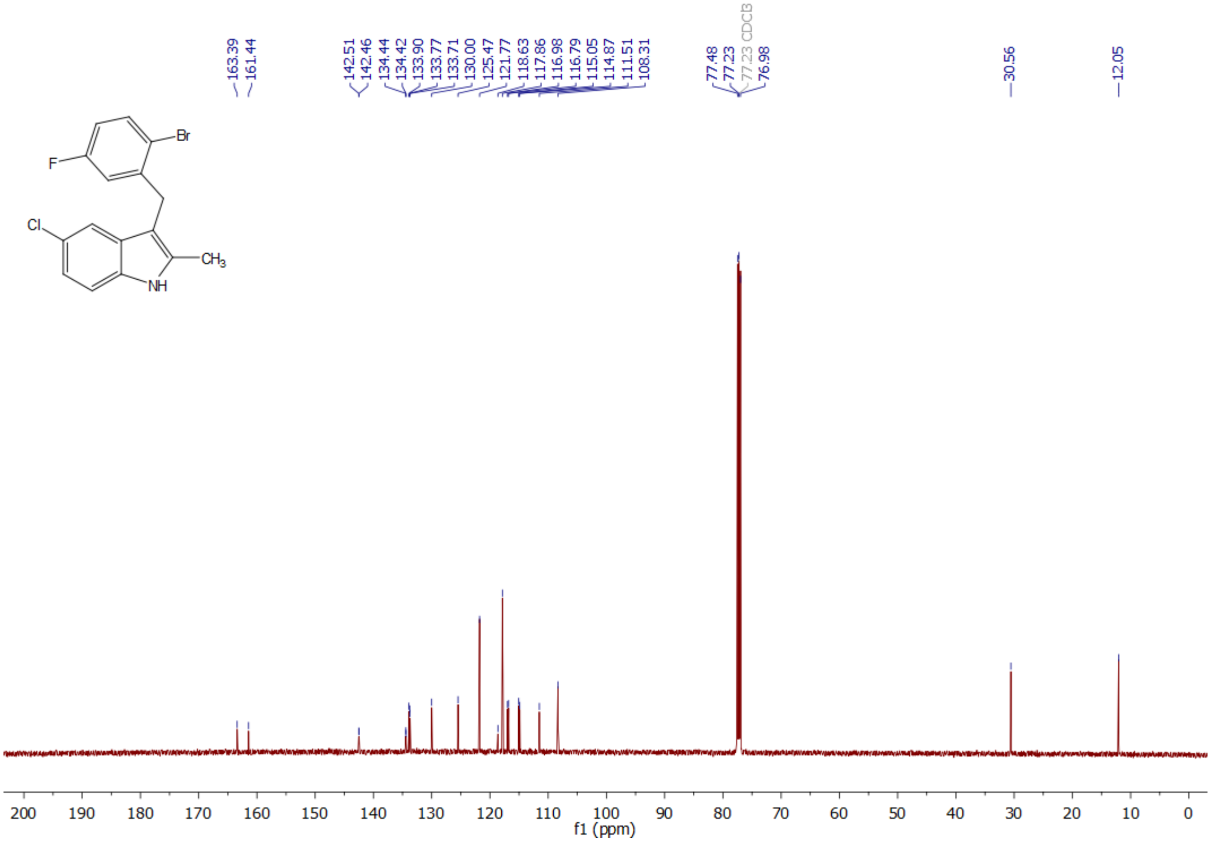

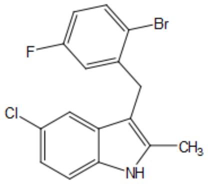
F
Br
Cl
CH3
NH

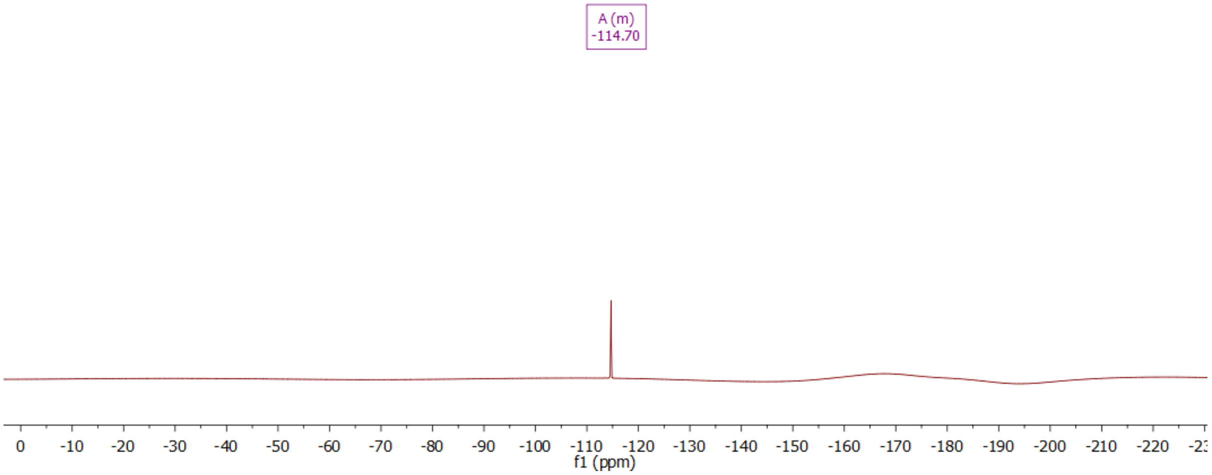
A (m)
-114.70
0
-10
-20
-30
-40
-50
-60
-70
-80
-90
-100
-110
-120
-130
-140
-150
-160
-170
-180
-190
-200
-210
-220
f1 (ppm)

3-(2-bromo-6-fluorobenzyl)-5-chloro-2-methyl-1H-indole [**1w**]:

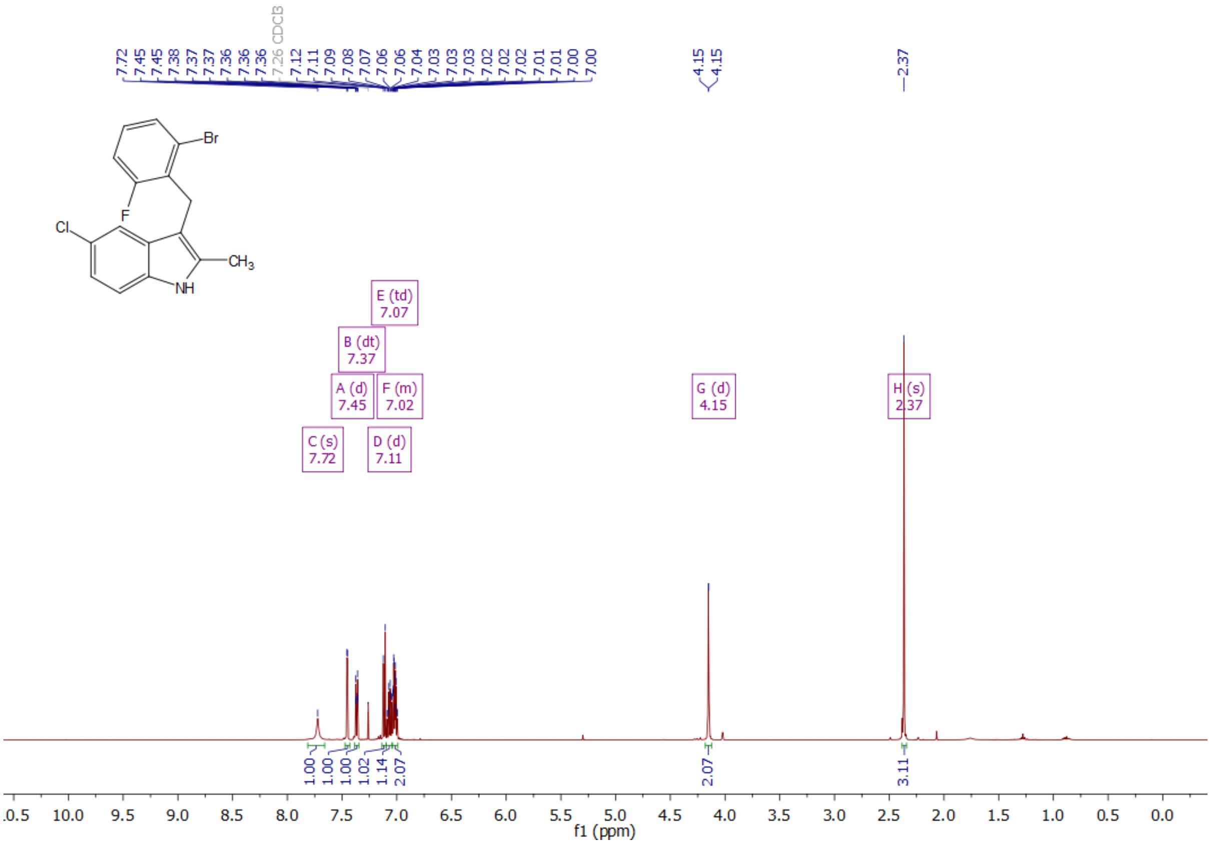

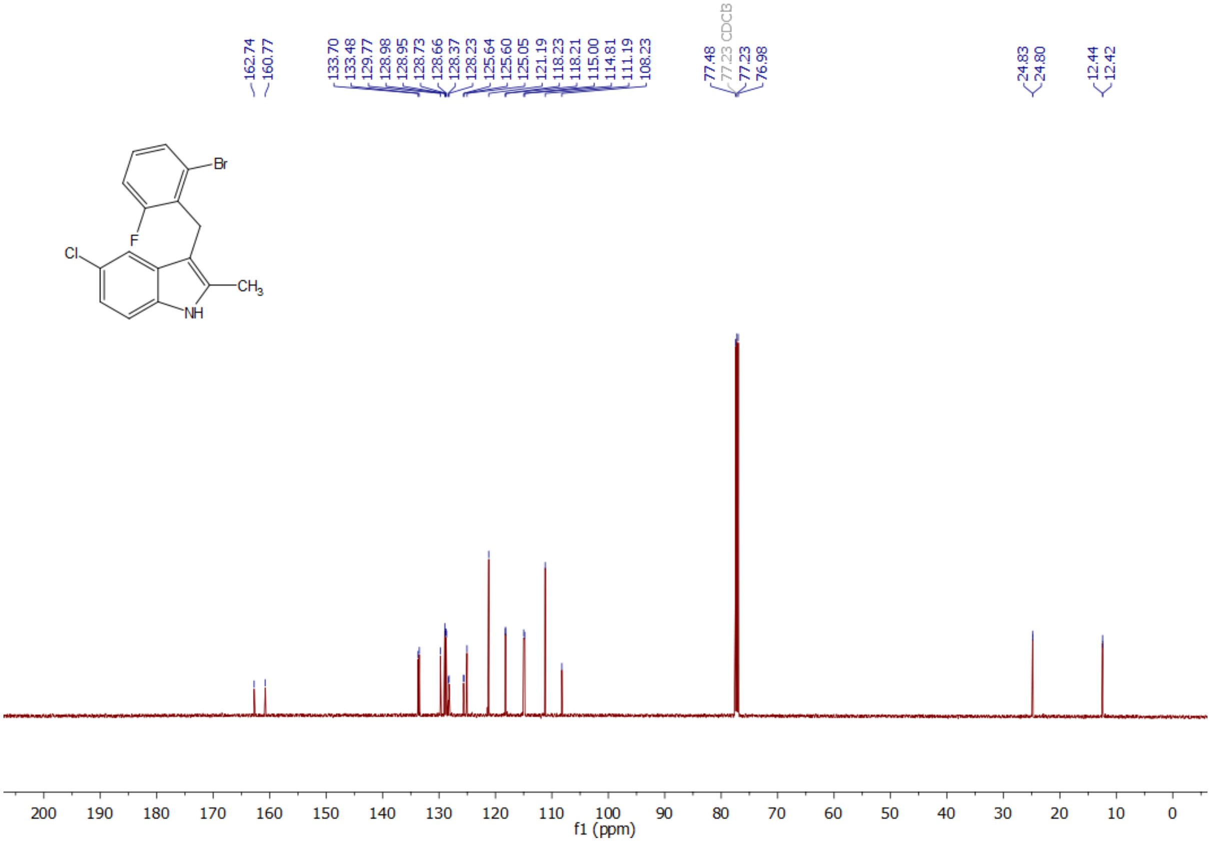

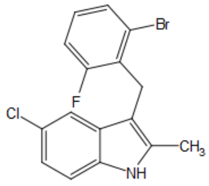
Br
F
Cl
CH3
NH

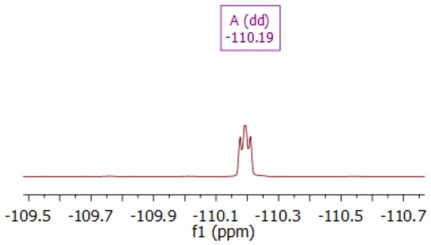
A (dd)
-110.19
-109.5 -109.7 -109.9 -110.1 -110.3 -110.5 -110.7
f1 (ppm)

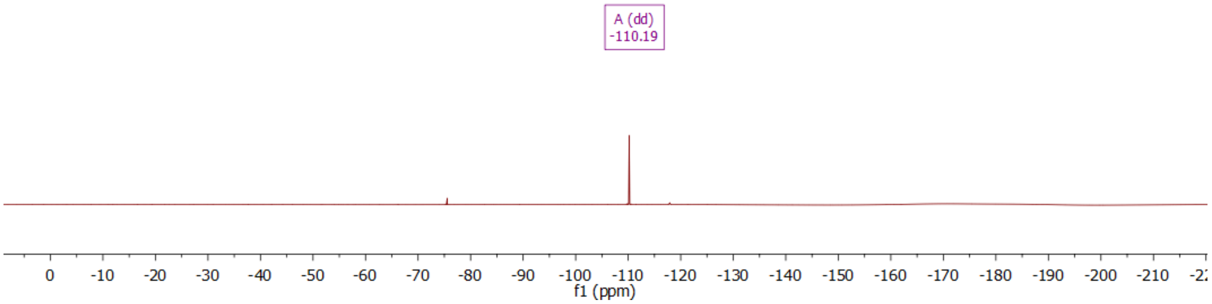
A (dd)
-110.19
0 -10 -20 -30 -40 -50 -60 -70 -80 -90 -100 -110 -120 -130 -140 -150 -160 -170 -180 -190 -200 -210
f1 (ppm)

3-(2-bromo-4,5-dimethoxybenzyl)-5-chloro-2-methyl-1H-indole [**1x**]:

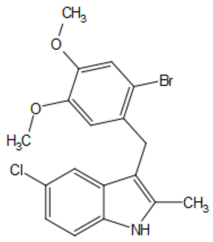

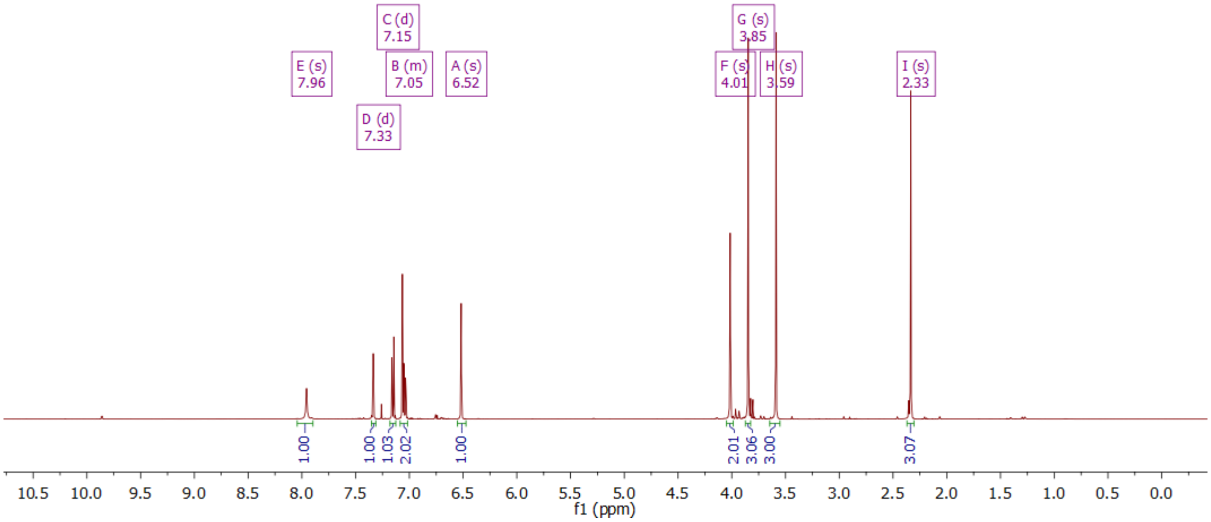

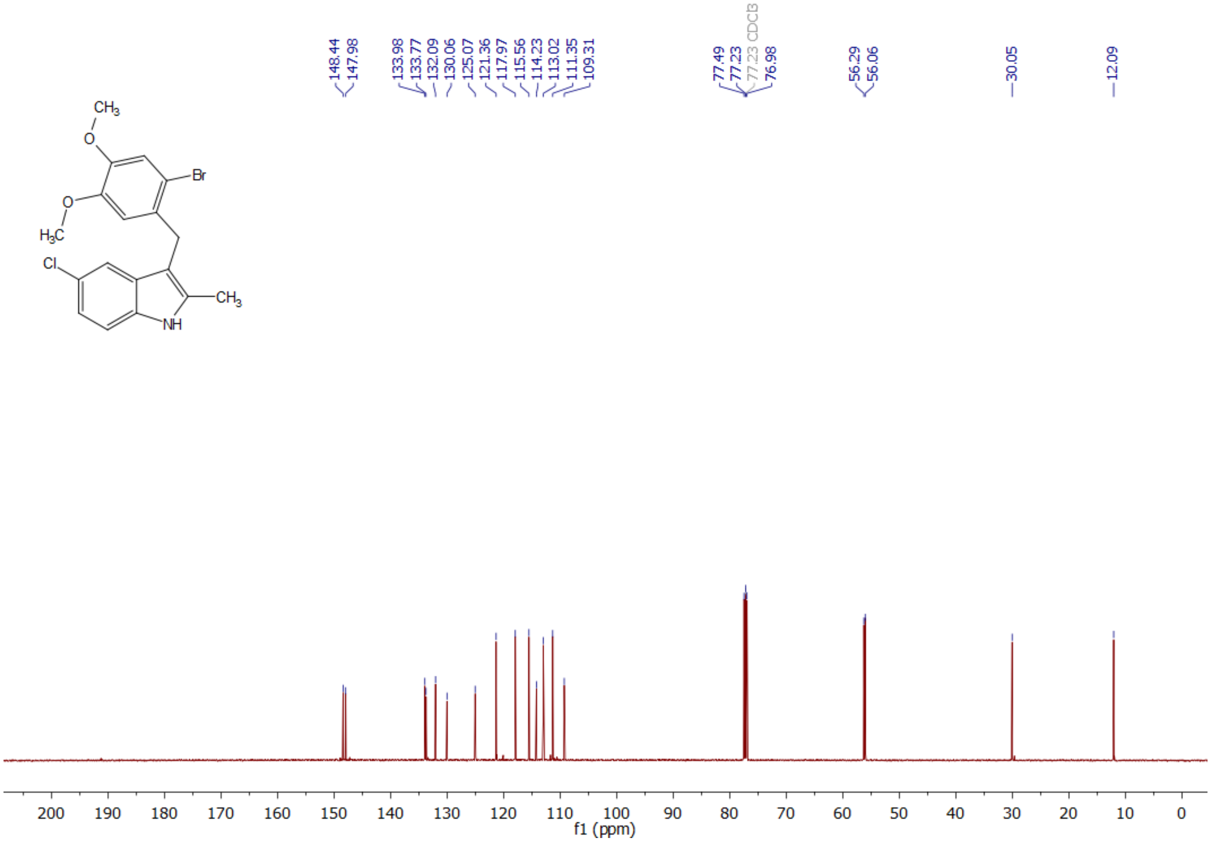

3-(2-bromo-3-fluorobenzyl)-2,5-dimethyl-1H-indole [**1y**]:

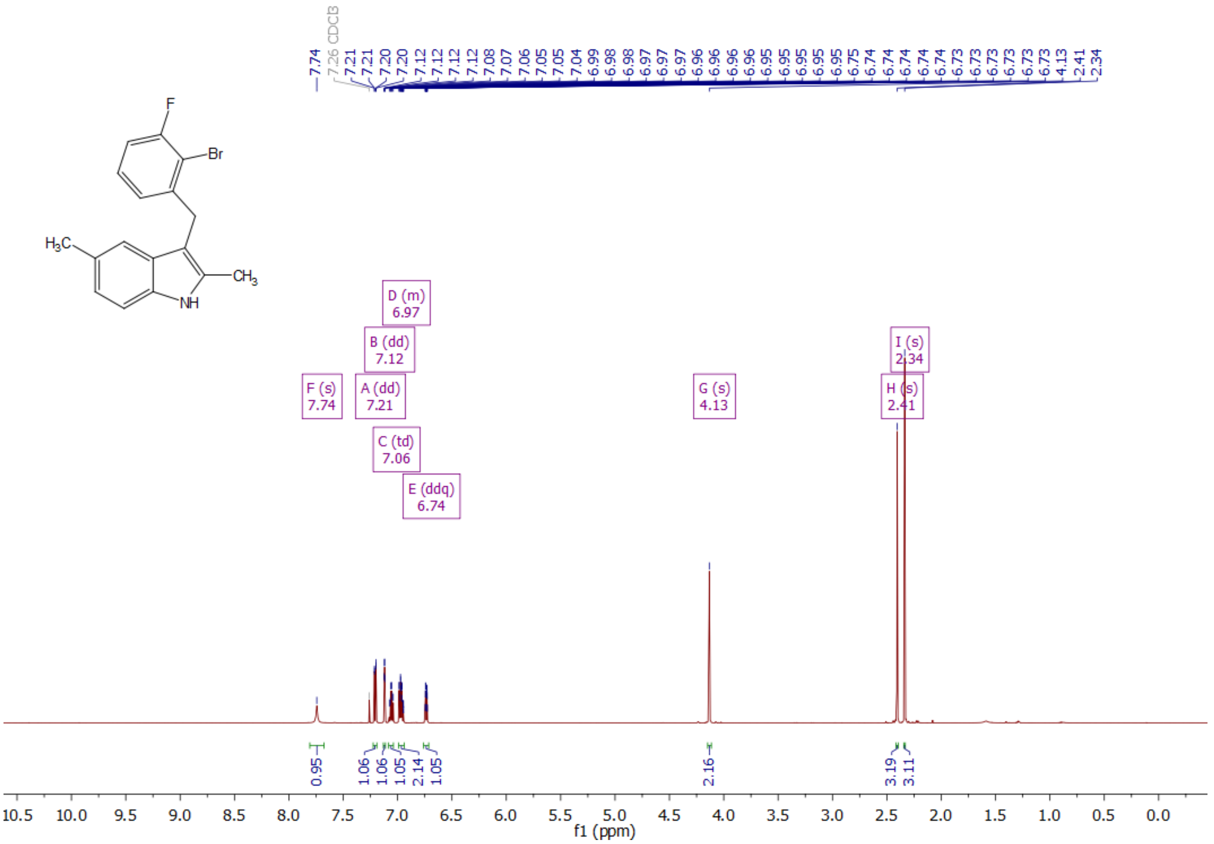

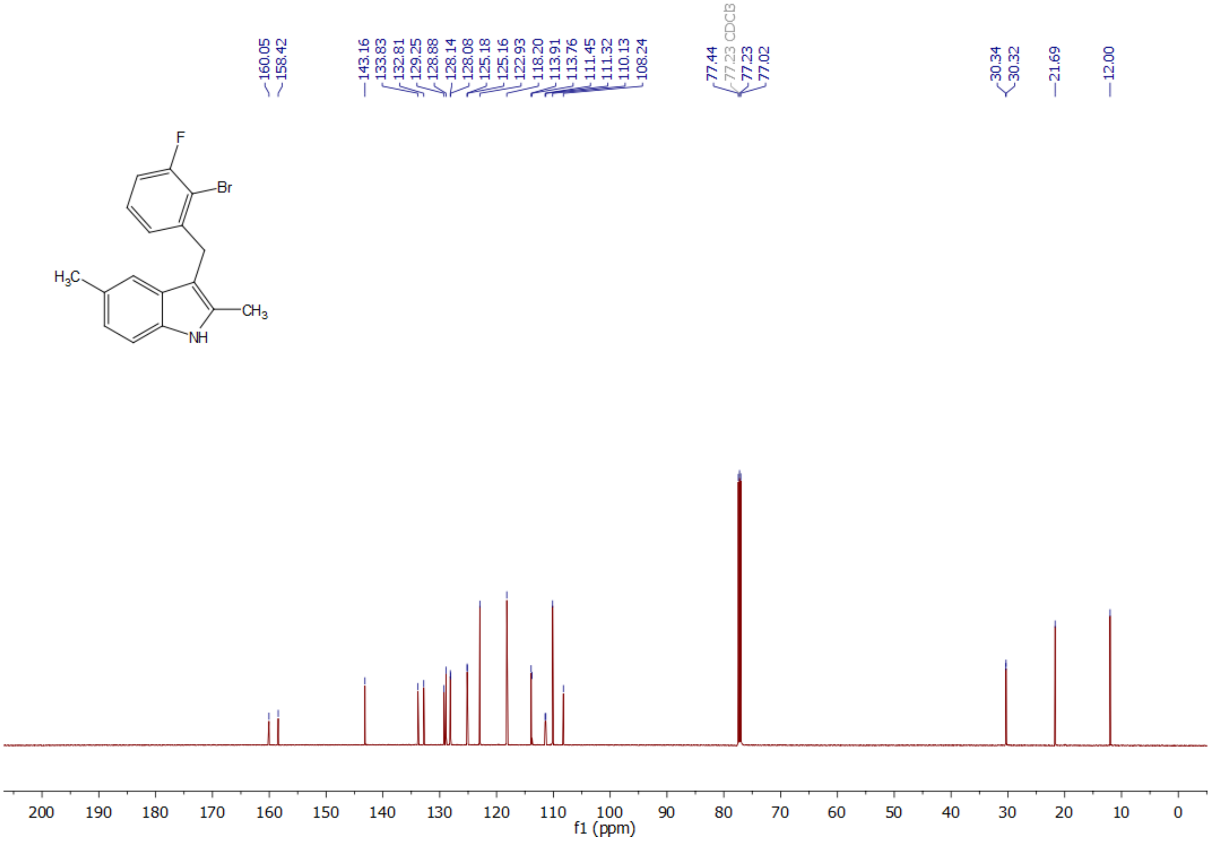

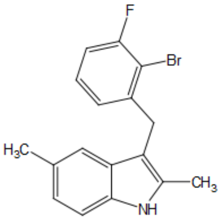
F
Br
H3C
CH3
NH

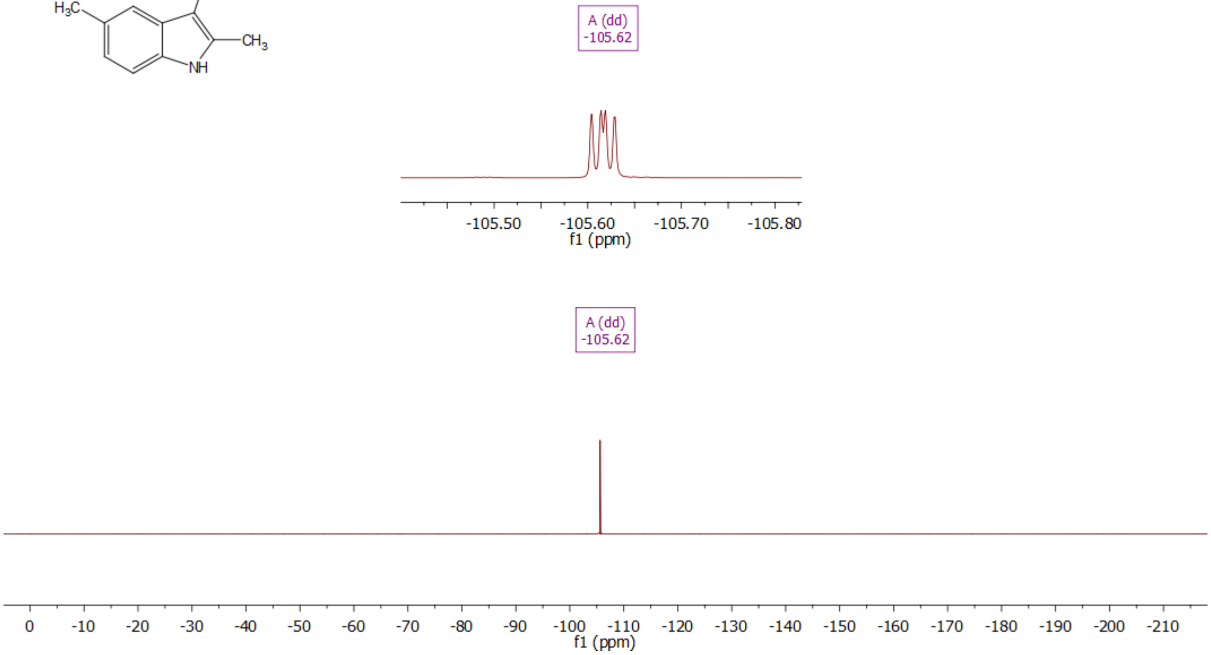
A (dd)
-105.62
-105.50
-105.60
-105.70
-105.80
f1 (ppm)
A (dd)
-105.62
0
-10
-20
-30
-40
-50
-60
-70
-80
-90
-100
-110
-120
-130
-140
-150
-160
-170
-180
-190
-200
-210
f1 (ppm)

***3-(2-bromo-4-chlorobenzyl)-2,5-dimethyl-1H-indole* [1z]:**

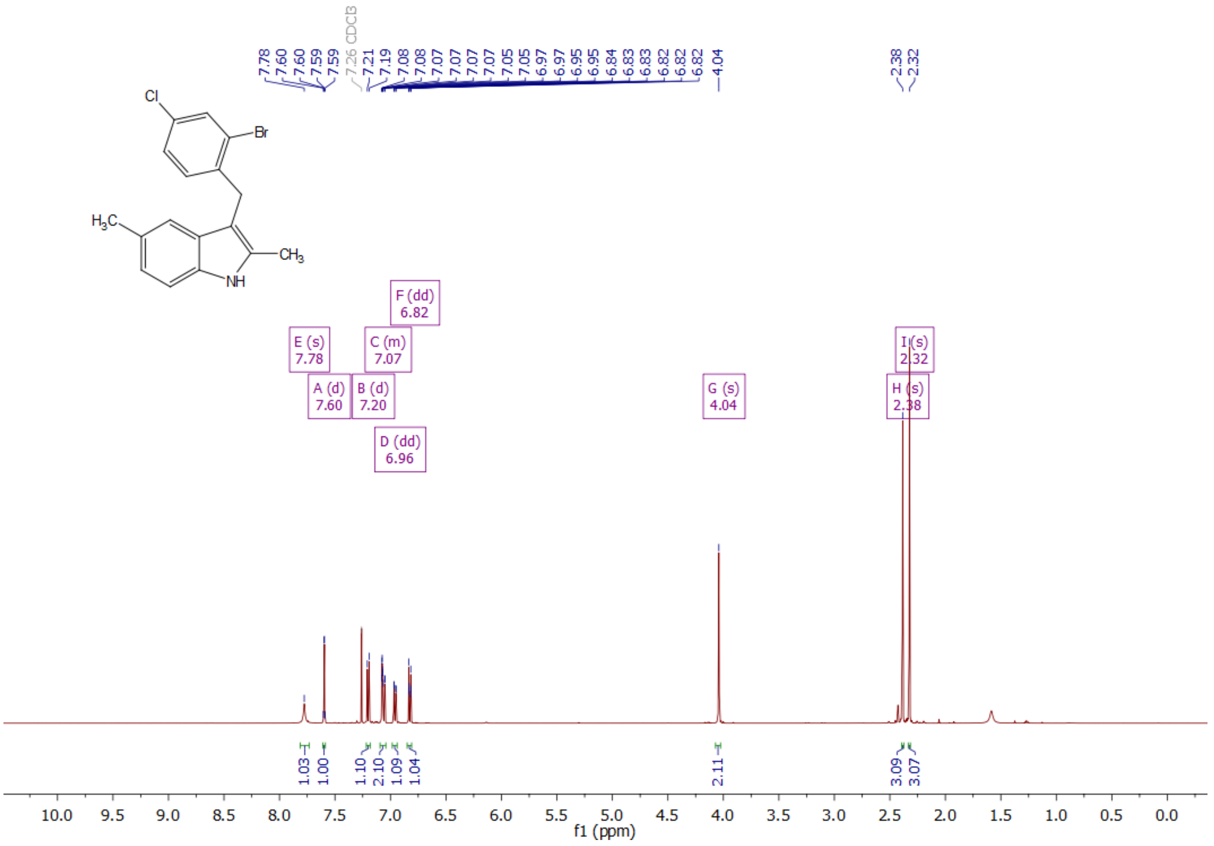

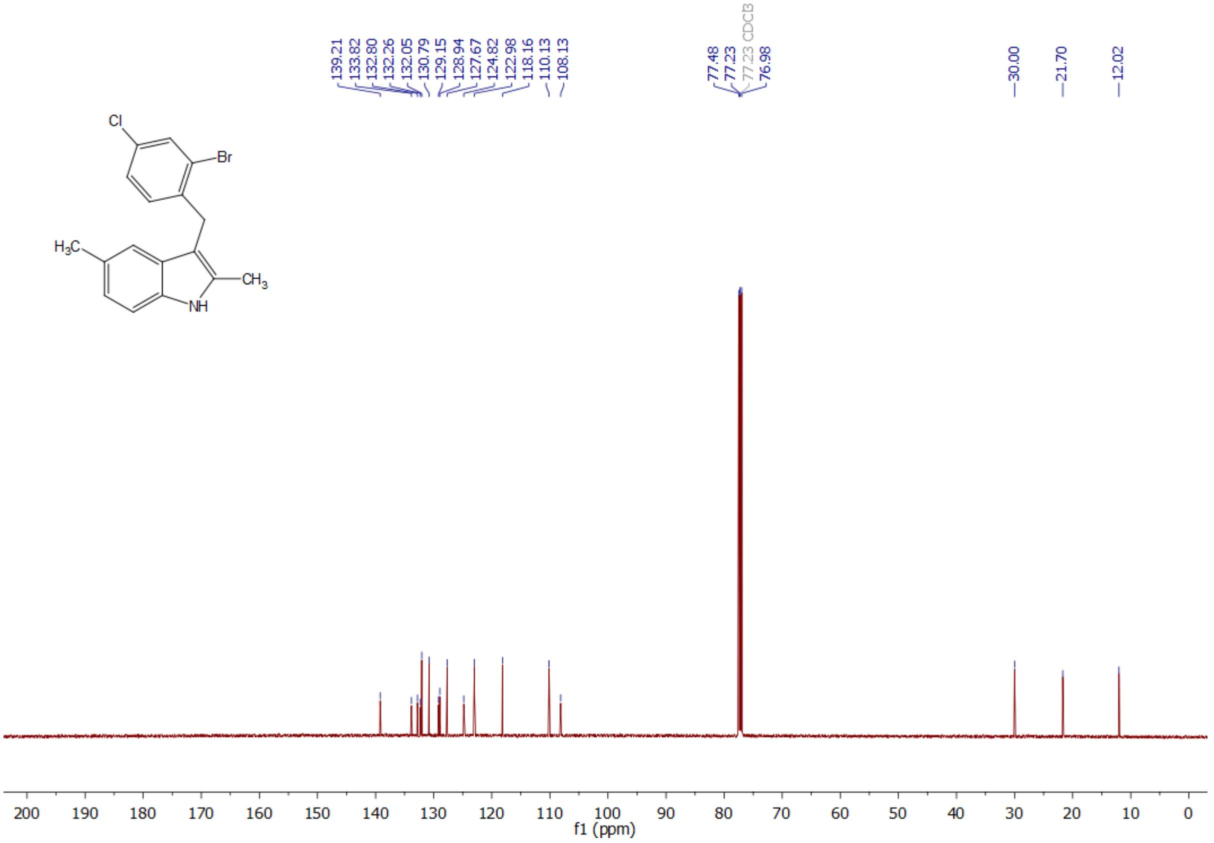

3-(2-bromo-6-fluorobenzyl)-2,5-dimethyl-1H-indole [**1aa**]:

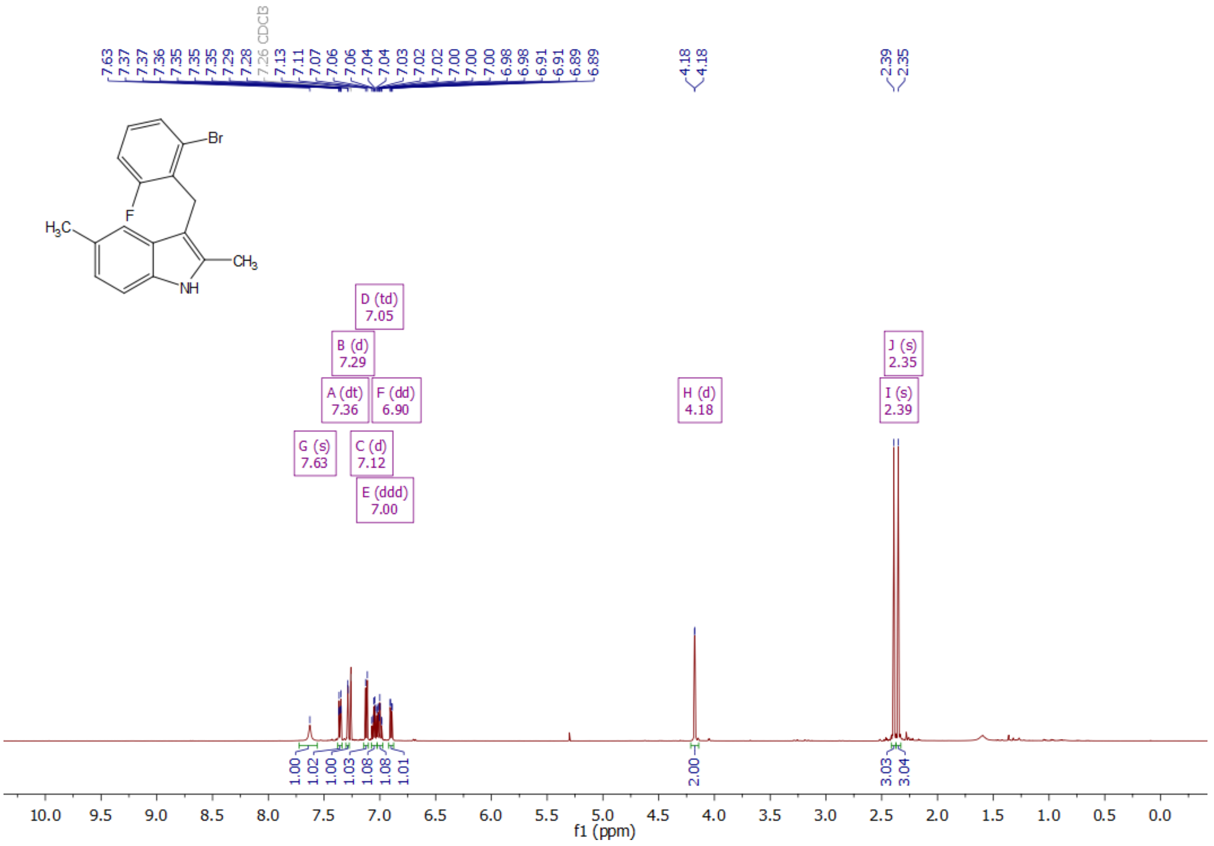

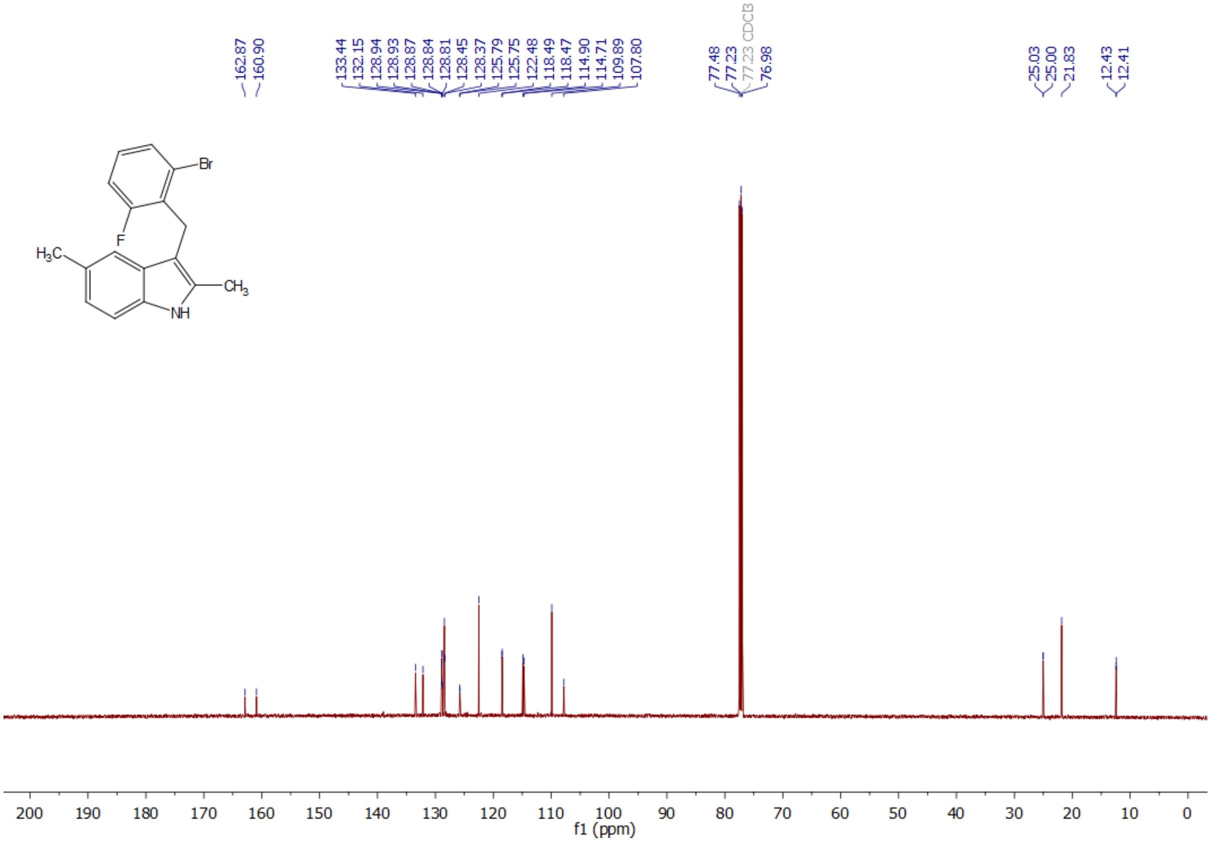

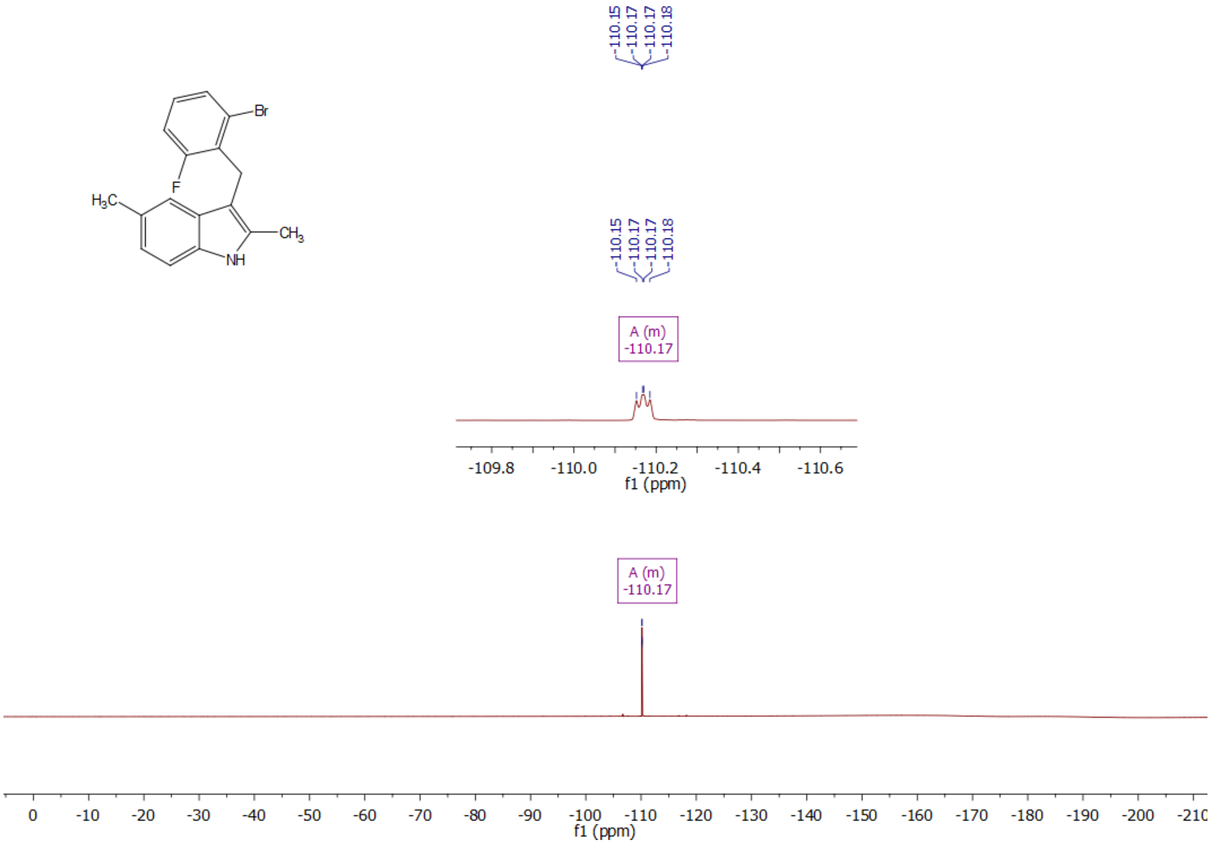
Br
F
H3C
CH3
NH
-110.15
-110.17
-110.17
-110.18
A (m)
-110.17
-109.8
-110.0
-110.2
-110.4
-110.6
f1 (ppm)
0
-10
-20
-30
-40
-50
-60
-70
-80
-90
-100
-110
-120
-130
-140
-150
-160
-170
-180
-190
-200
-210
f1 (ppm)

3-(2-bromo-4,5-dimethoxybenzyl)-2,5-dimethyl-1H-indole [**1ab**]:

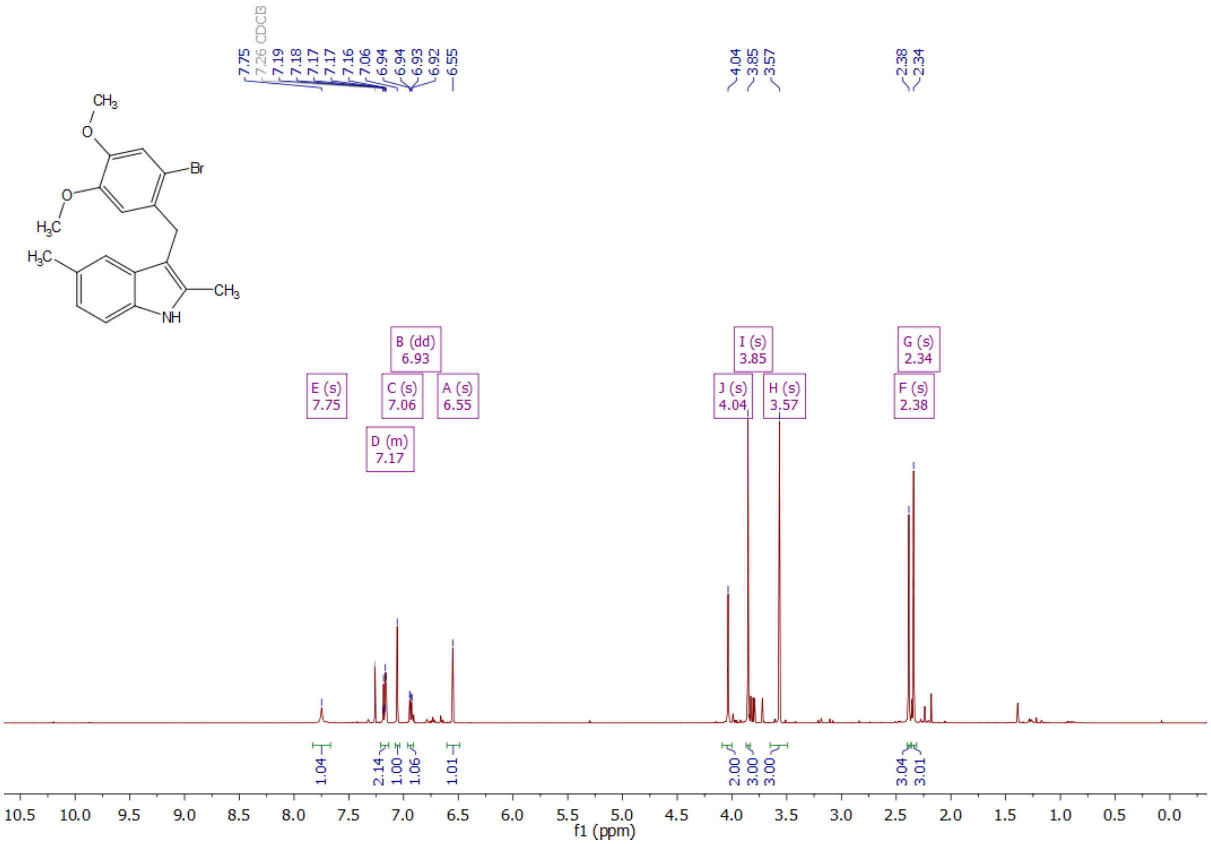

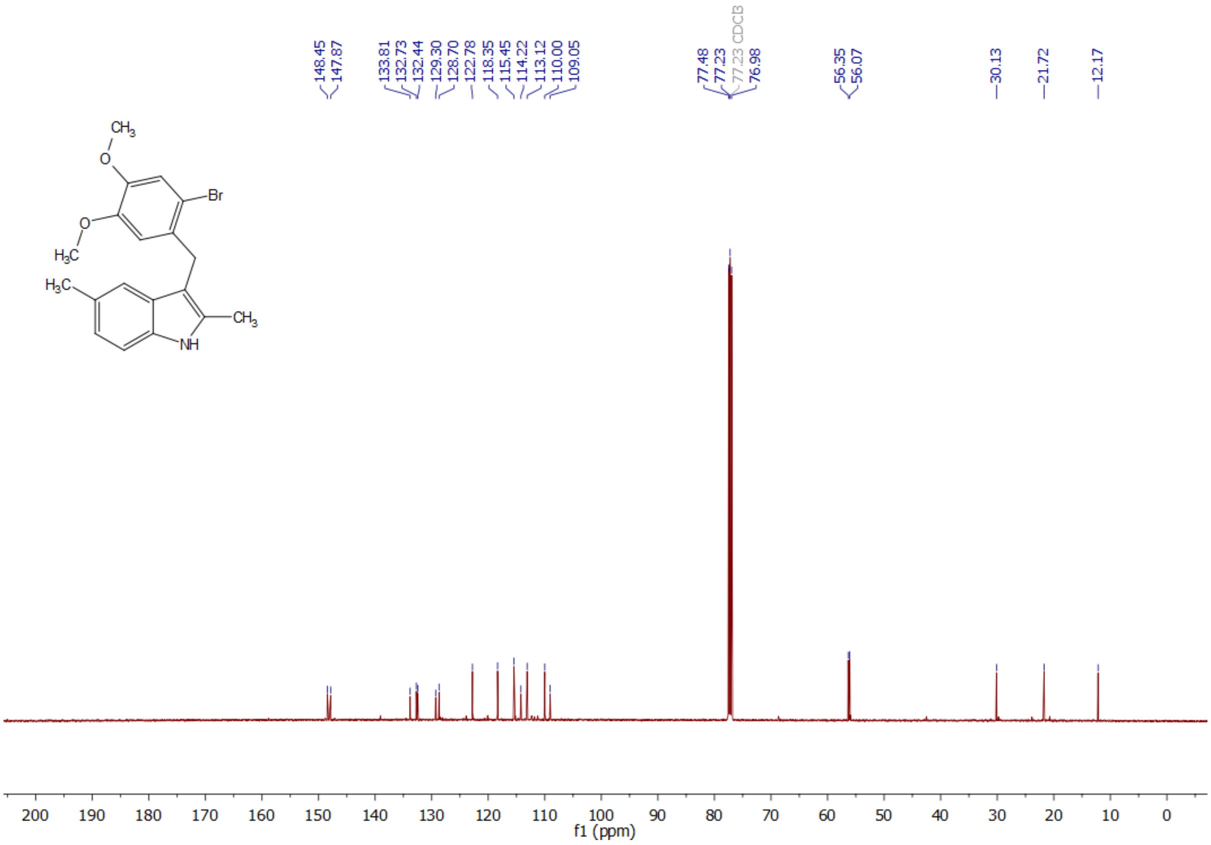

3-((6-bromobenzo[d][1,3]dioxol-5-yl)methyl)-2,5-dimethyl-1H-indole [**1ac**]:

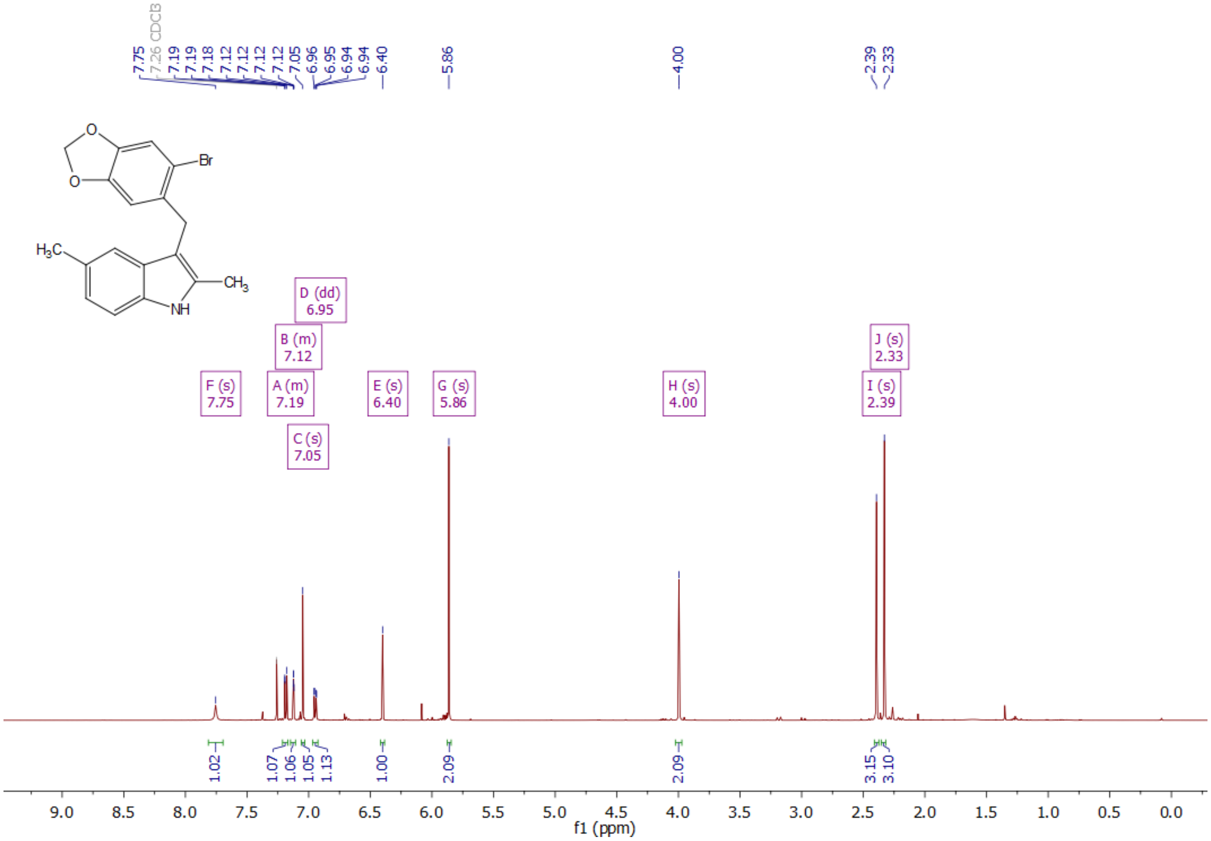

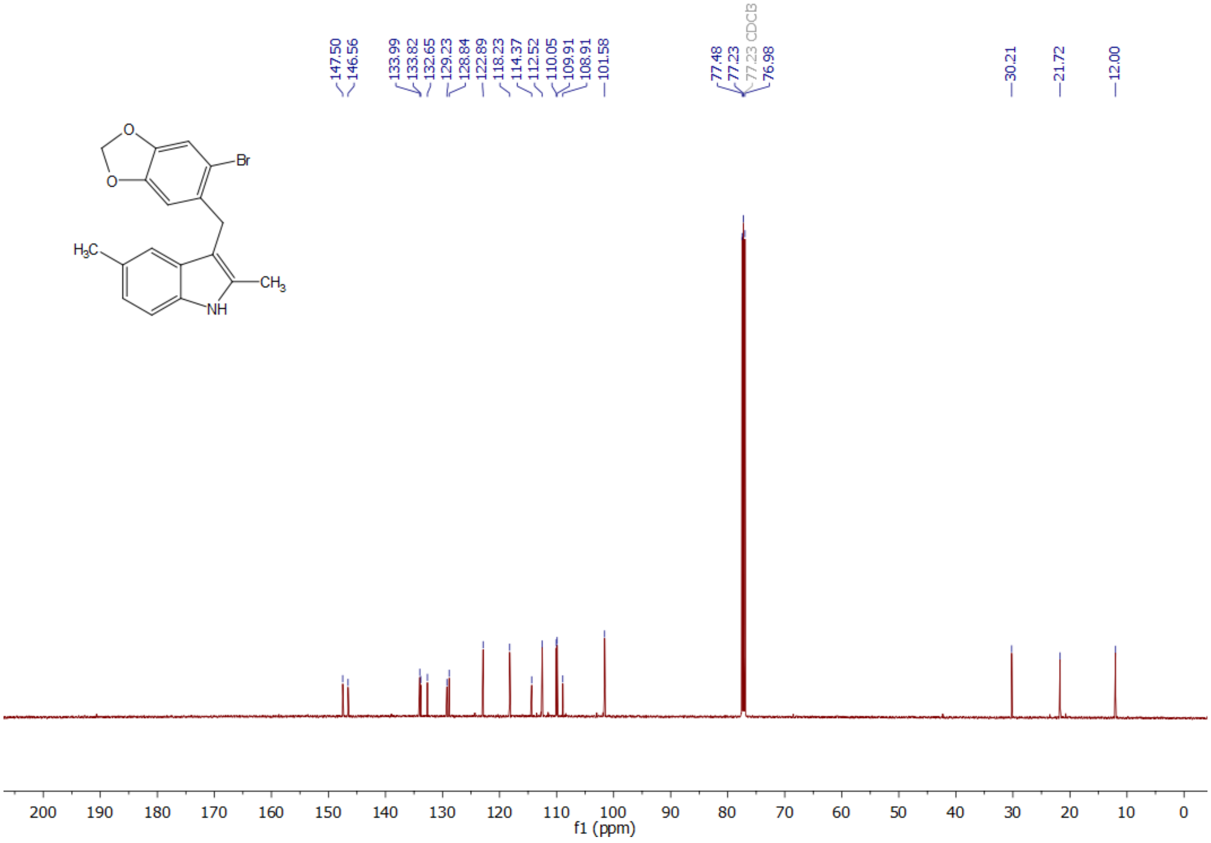

3-(2-bromobenzyl)-2-phenyl-1H-indole [**1ad**]:

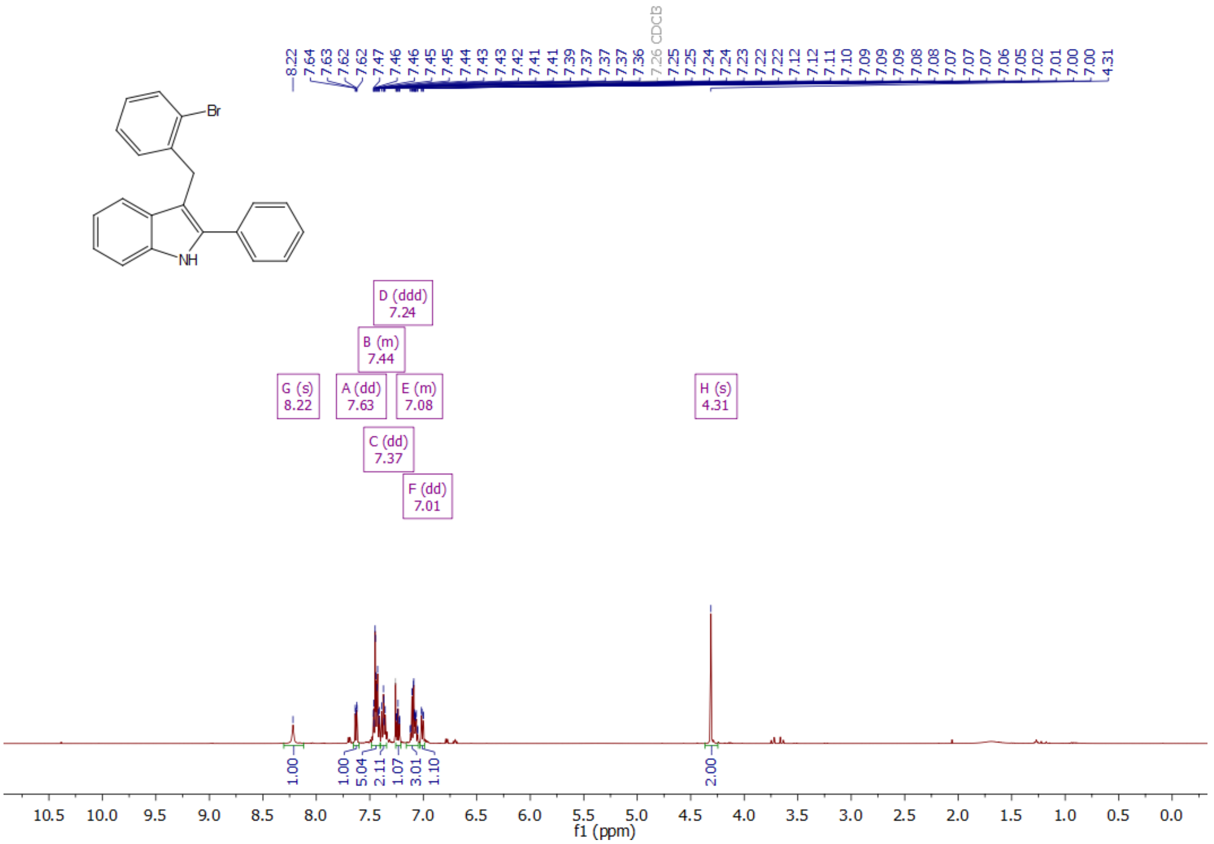

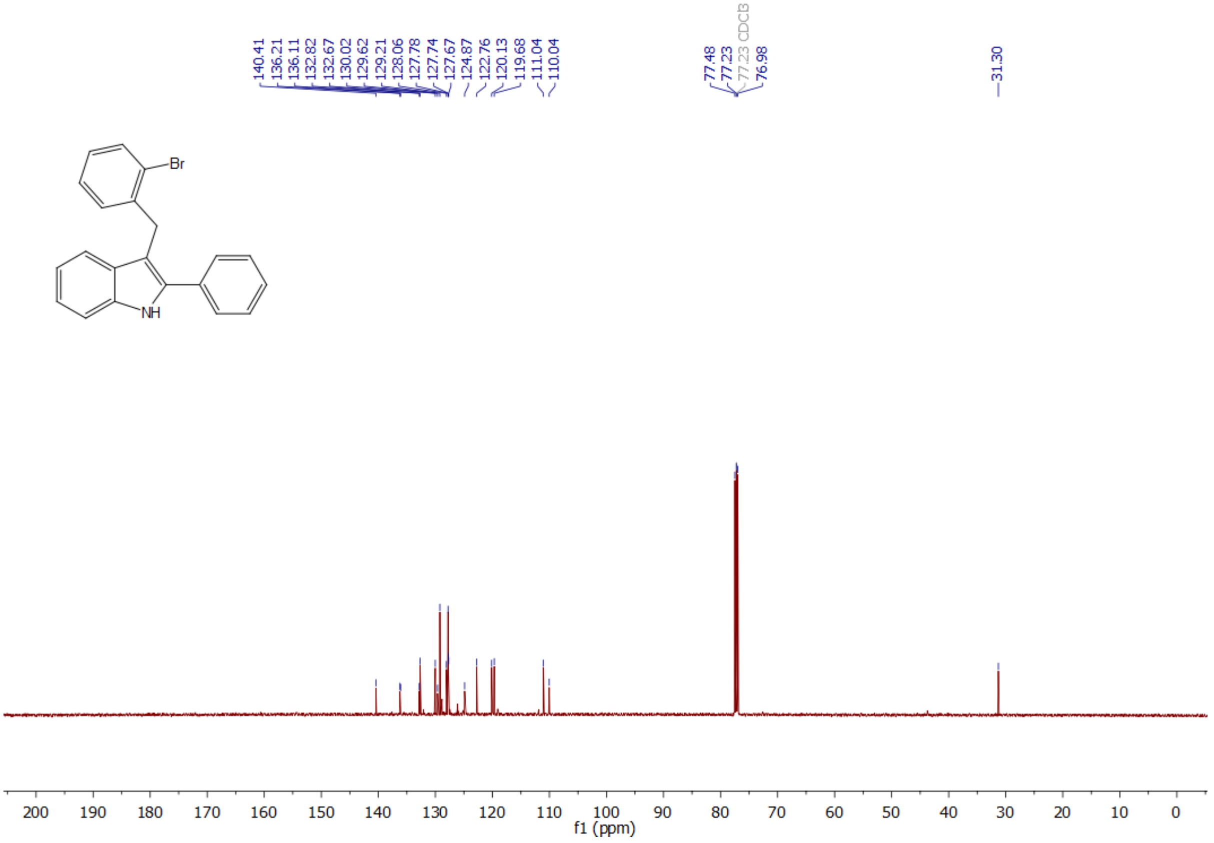

3-(2-bromo-4-fluorobenzyl)-2-phenyl-1H-indole [**1ae**]:

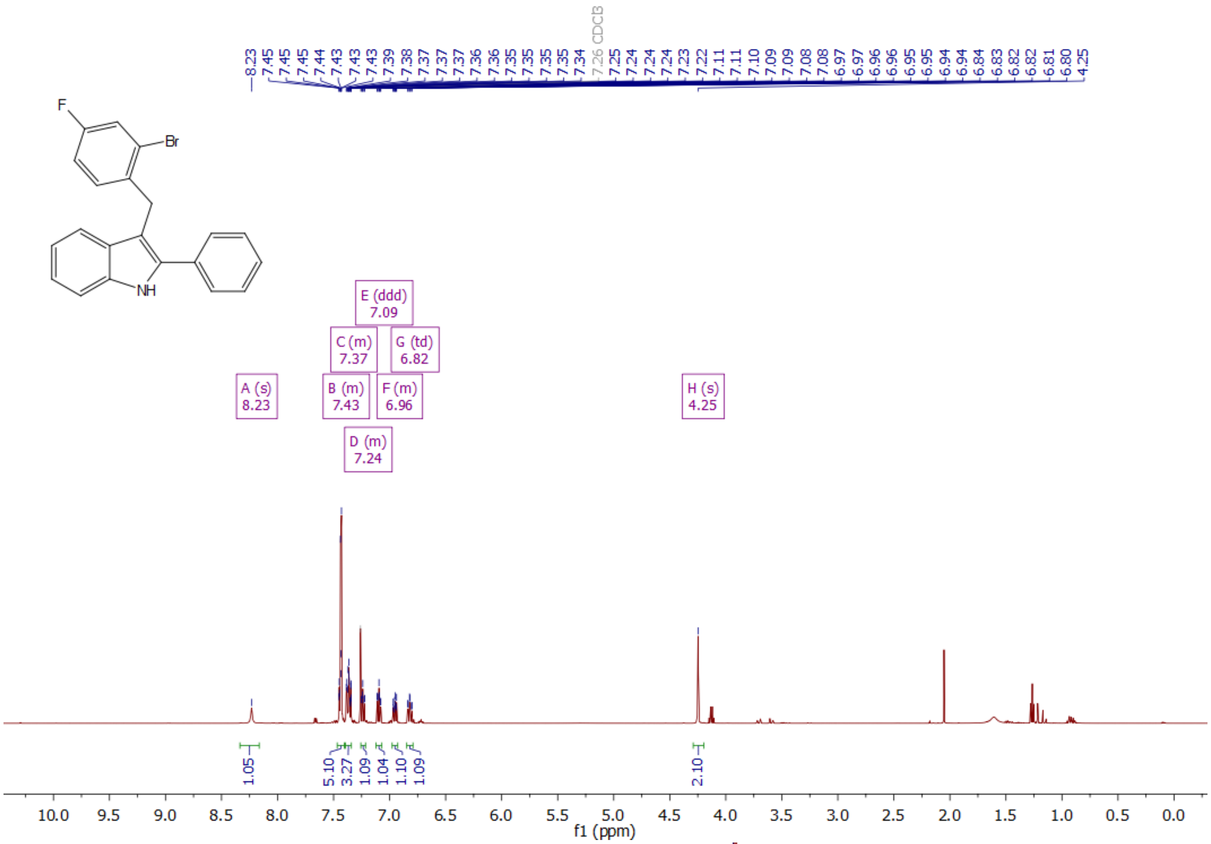

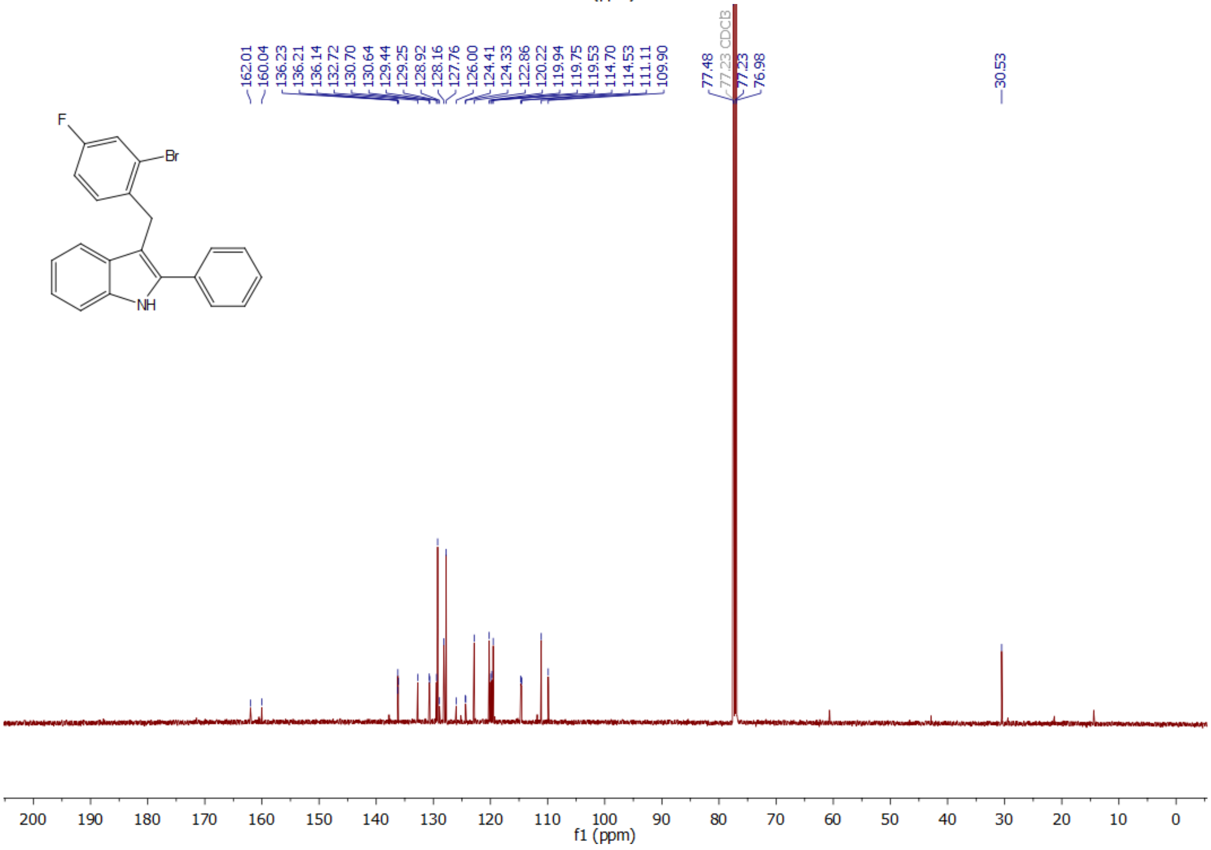

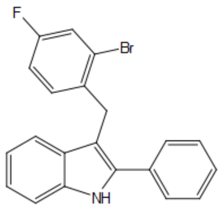
F
Br
NH

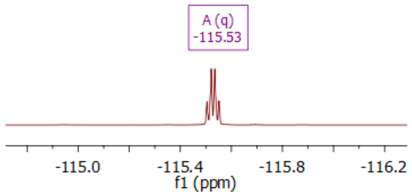
A (q)
-115.53
-115.0
-115.4
-115.8
-116.2
f1 (ppm)

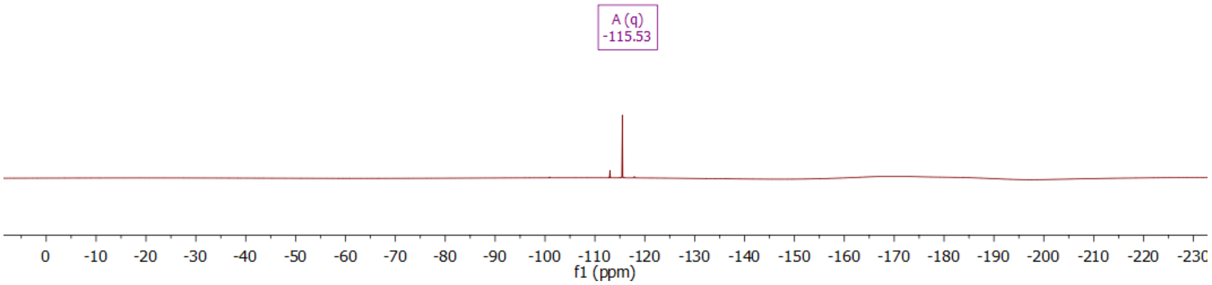
A (q)
-115.53
0 -10 -20 -30 -40 -50 -60 -70 -80 -90 -100 -110 -120 -130 -140 -150 -160 -170 -180 -190 -200 -210 -220 -230
f1 (ppm)

***3-(2-bromo-4-chlorobenzyl)-2-phenyl-1H-indole* [1af]:**

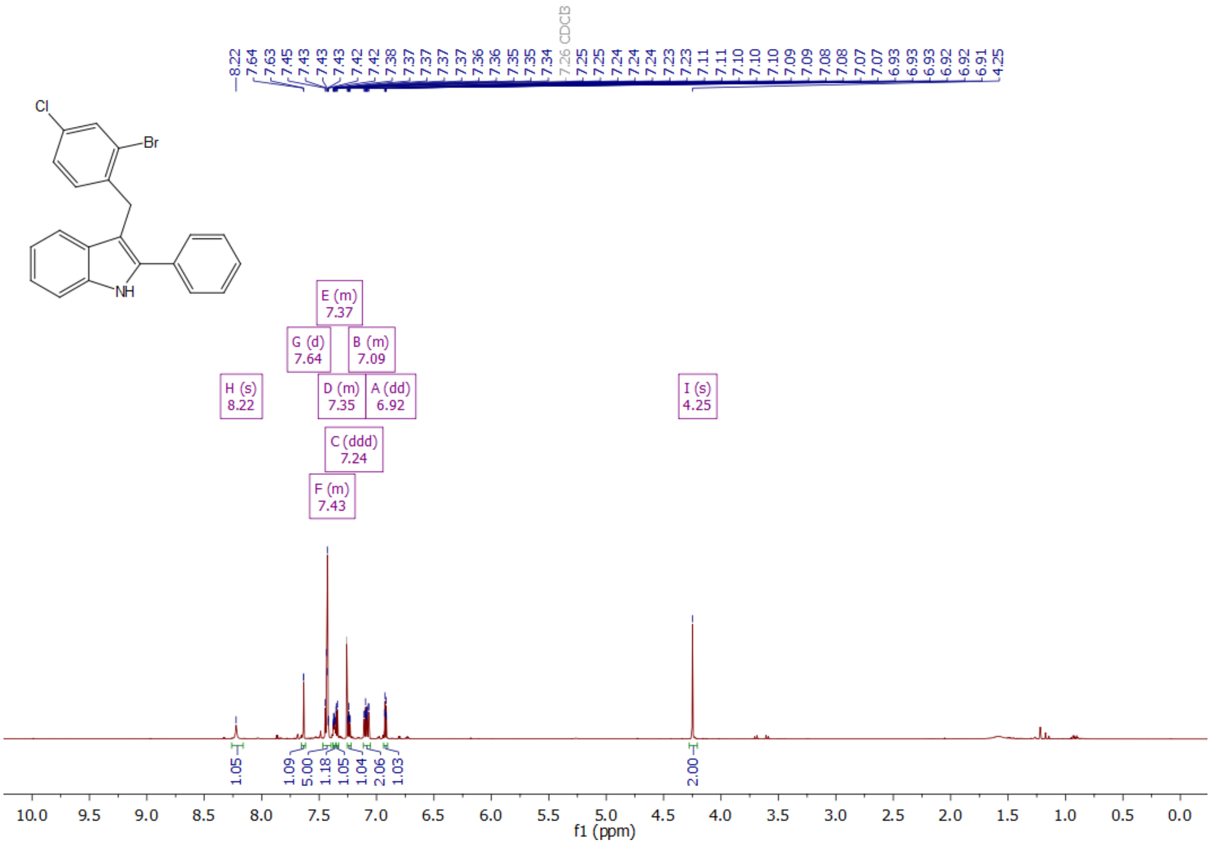

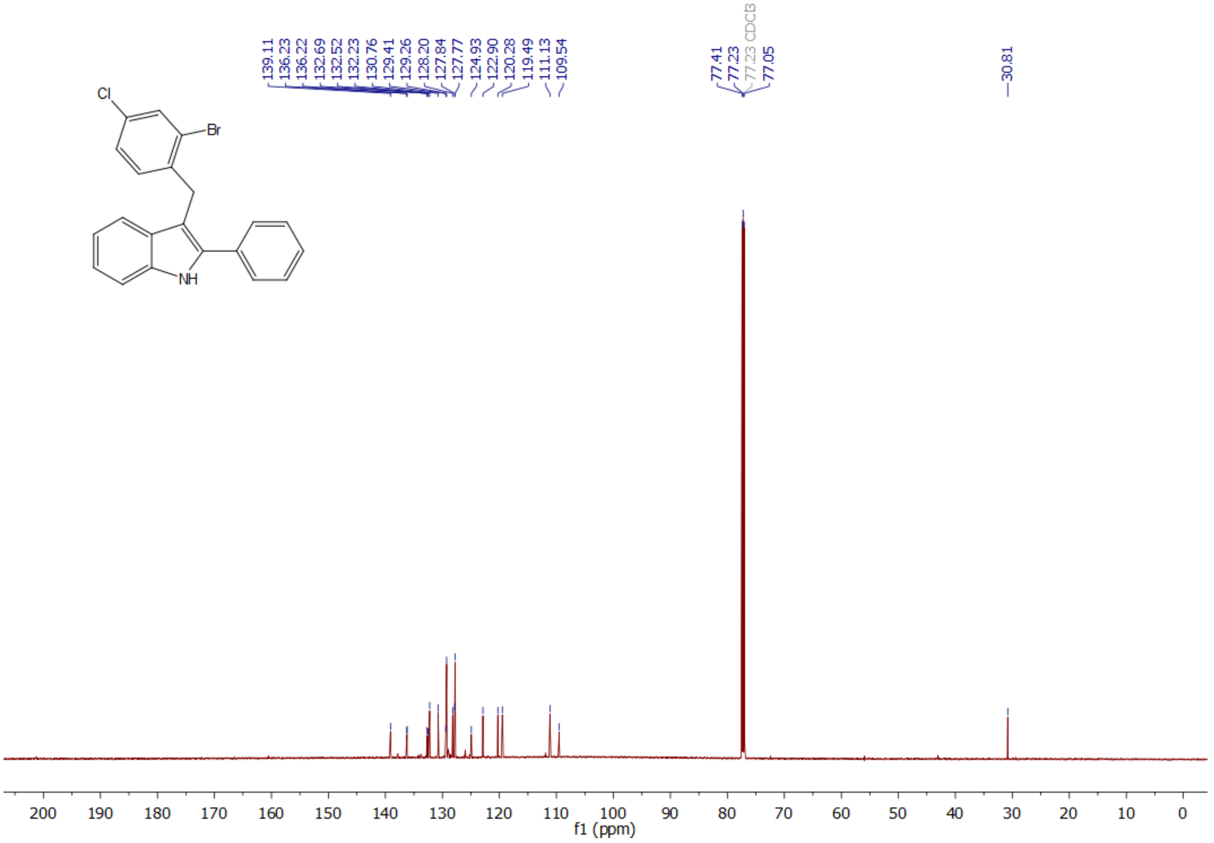

3-(2-bromo-5-fluorobenzyl)-2-phenyl-1H-indole [**1ag**]:

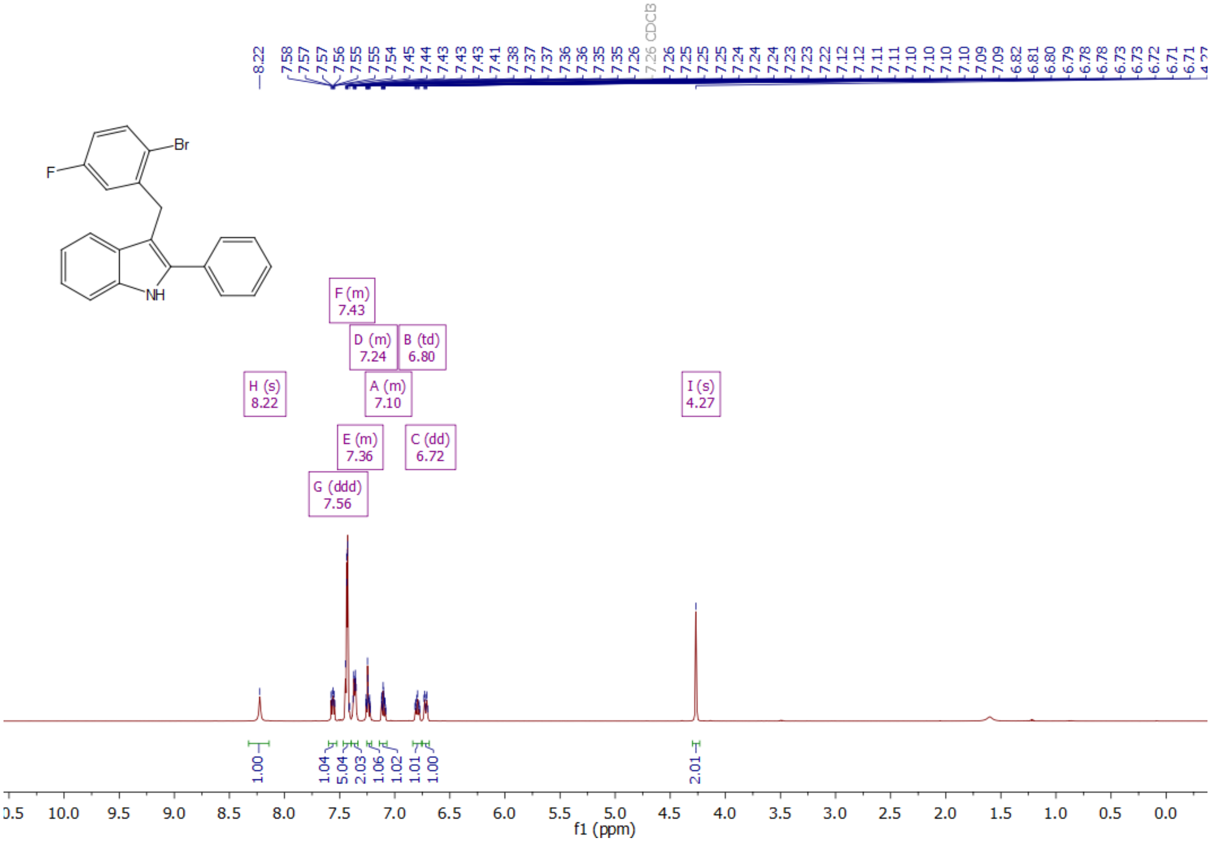

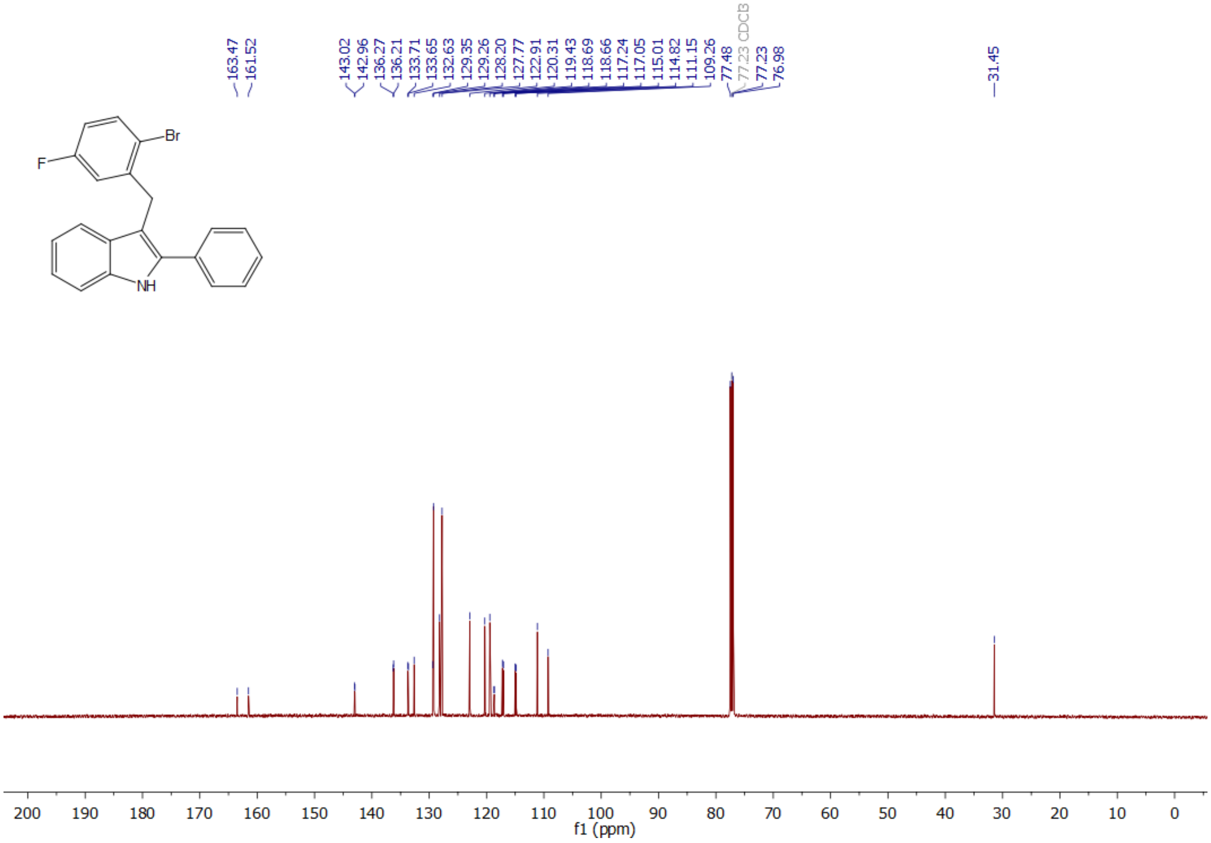

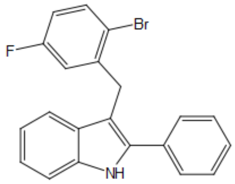
F
Br
NH

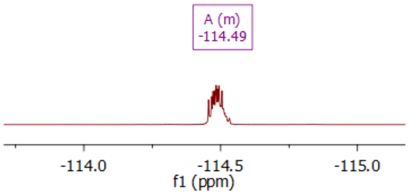
A (m)
-114.49
-114.0
-114.5
-115.0
f1 (ppm)

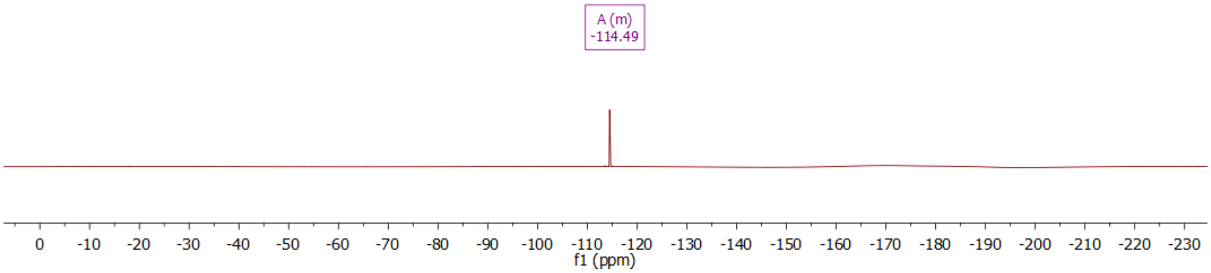
A (m)
-114.49
0 -10 -20 -30 -40 -50 -60 -70 -80 -90 -100 -110 -120 -130 -140 -150 -160 -170 -180 -190 -200 -210 -220 -230
f1 (ppm)

3-(2-bromo-5-methoxybenzyl)-2-phenyl-1H-indole [**1ah**]:

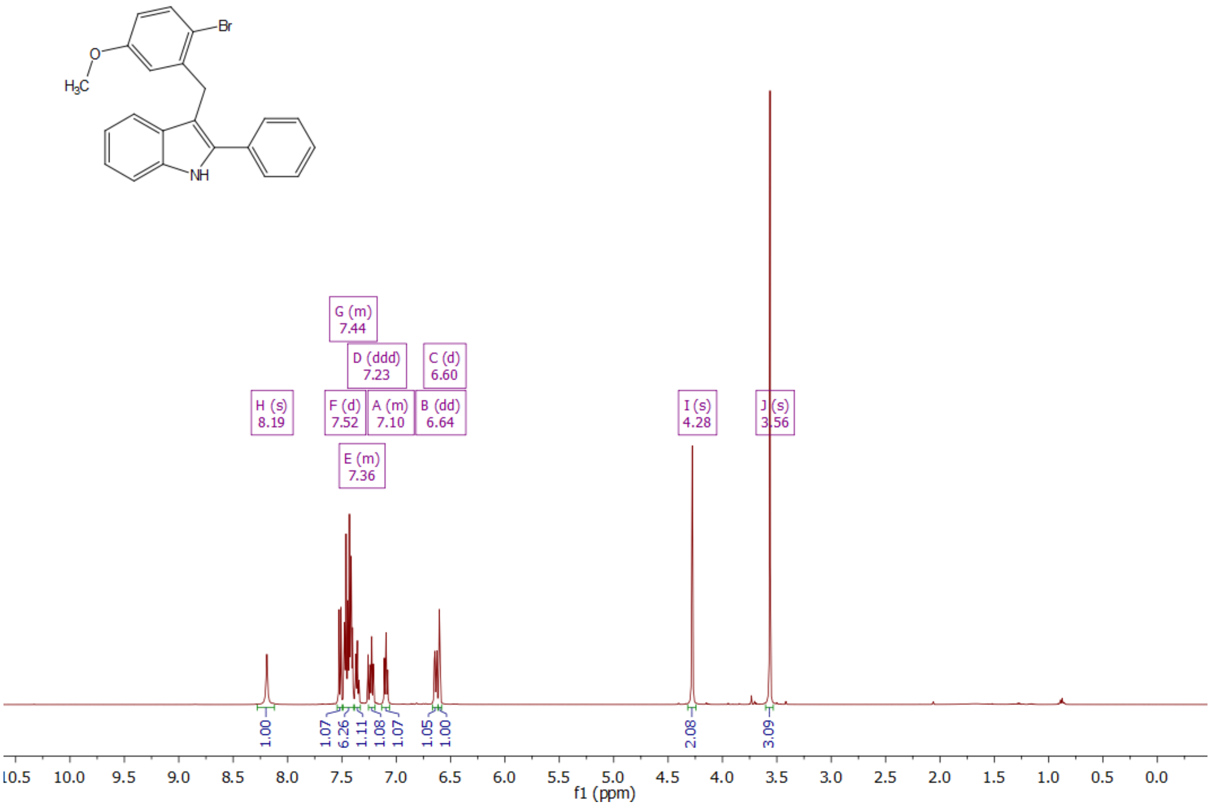

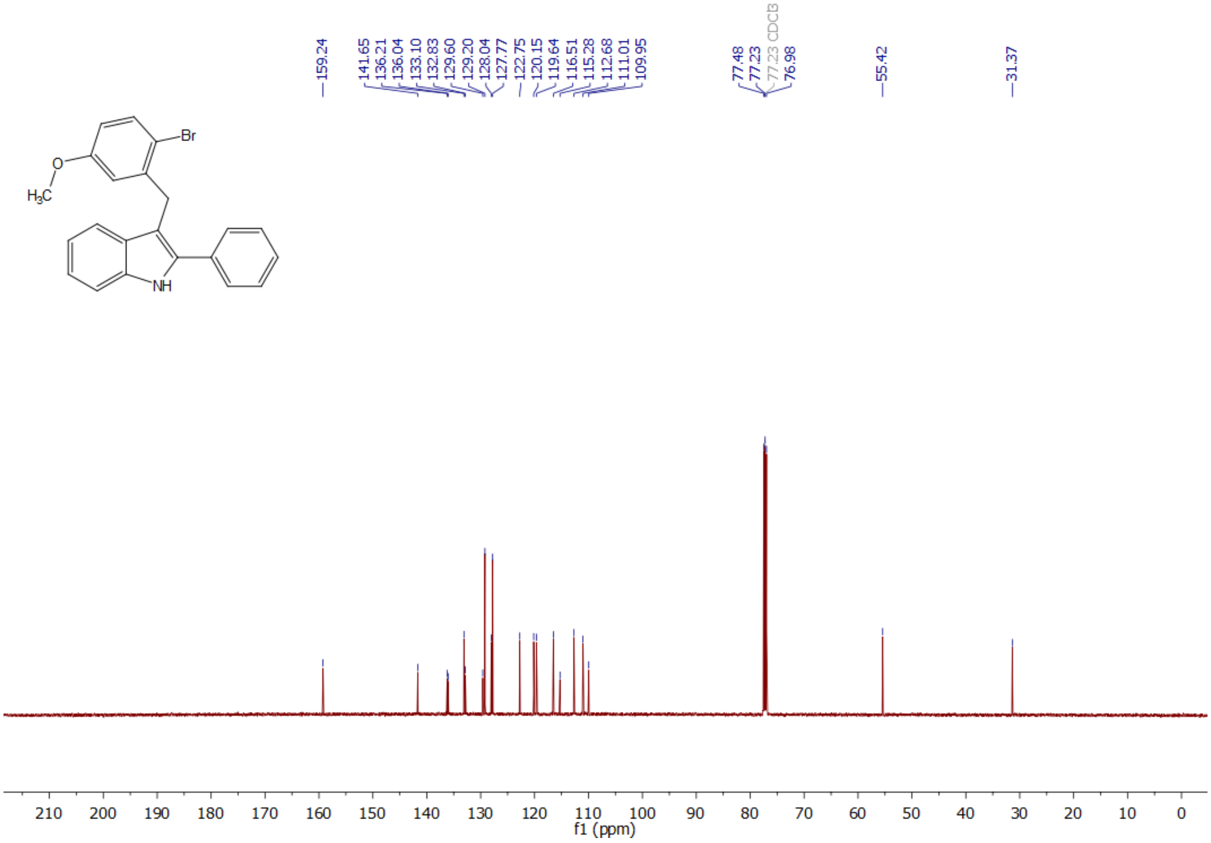

3-(2-bromo-6-fluorobenzyl)-2-phenyl-1H-indole [**1ai**]:

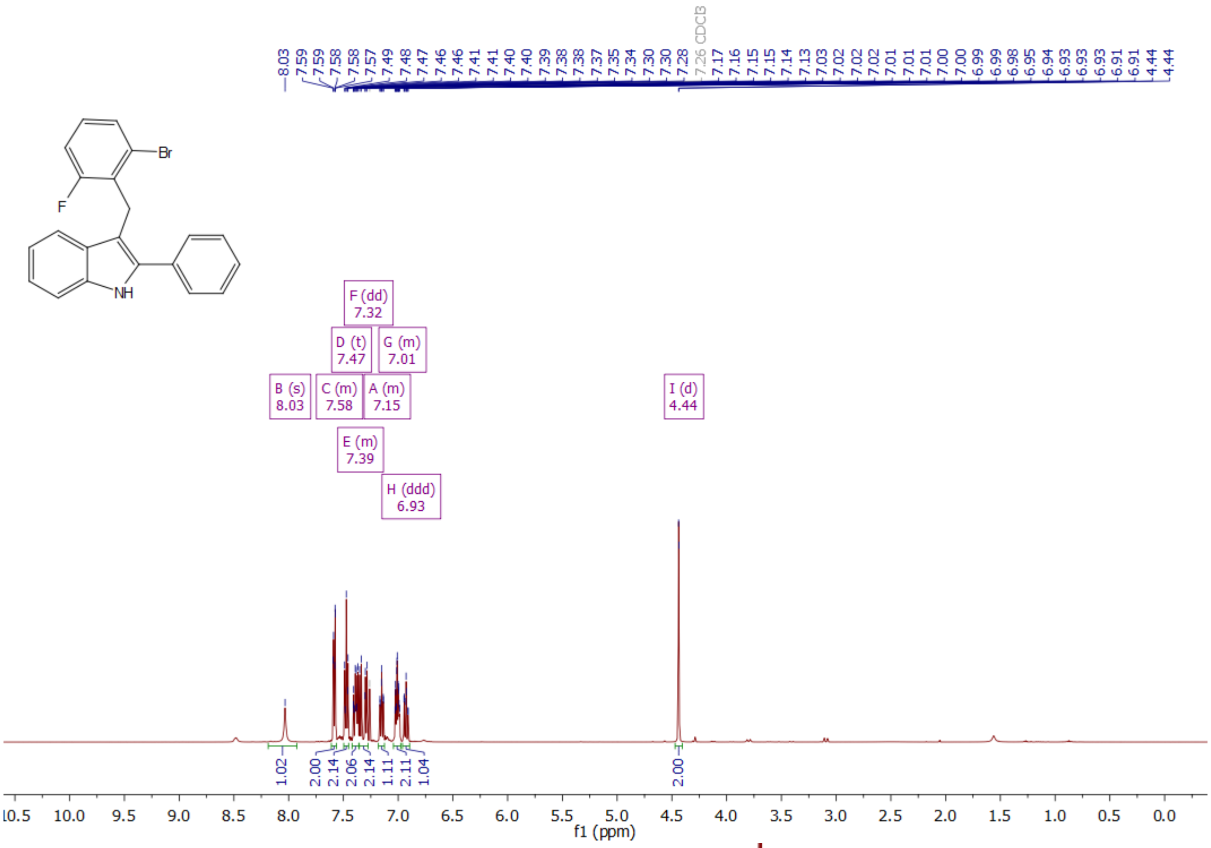

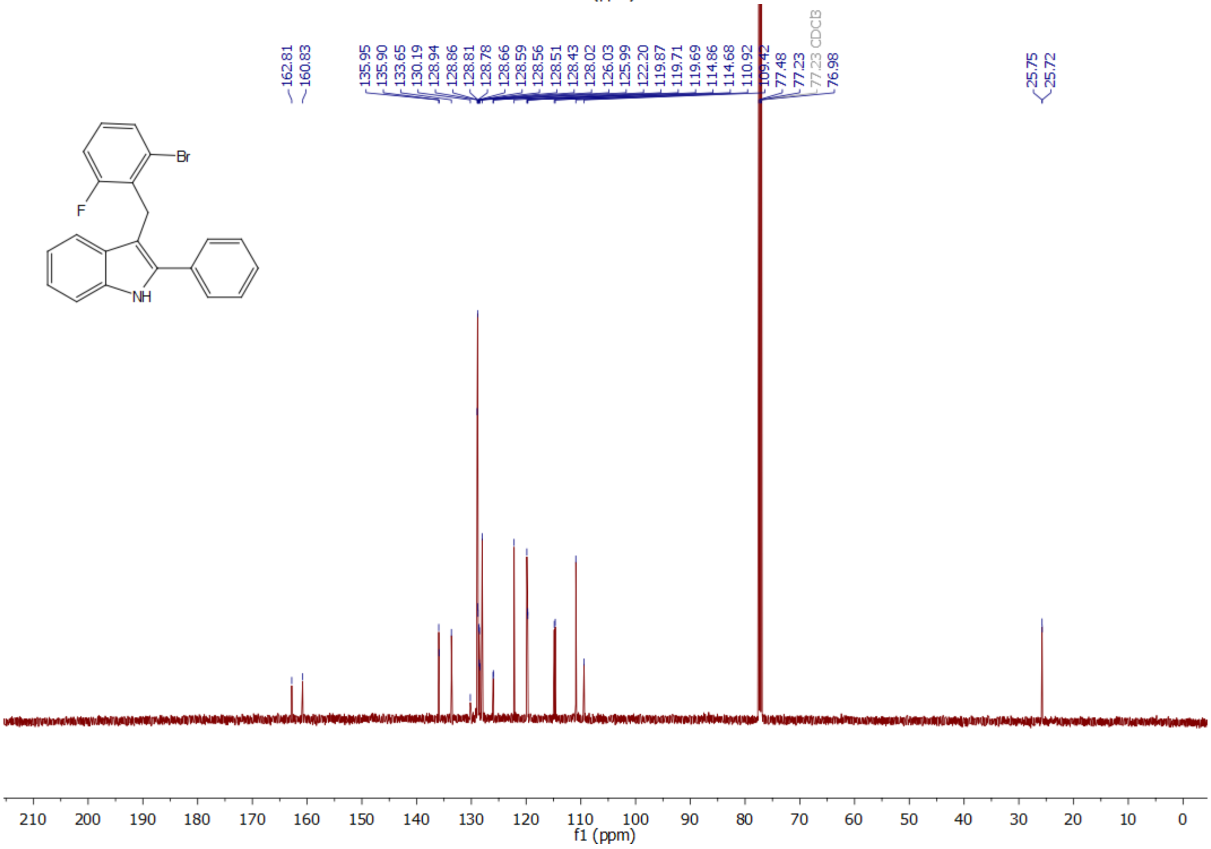

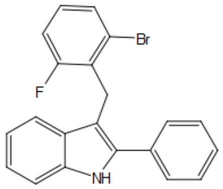
Br
F
NH

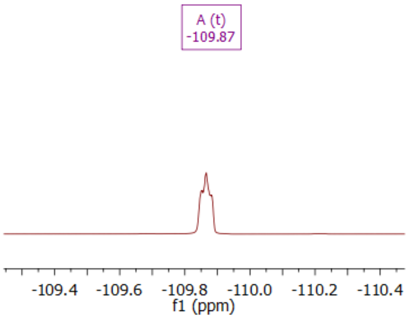
A (t)
-109.87
-109.4 -109.6 -109.8 -110.0 -110.2 -110.4
f1 (ppm)

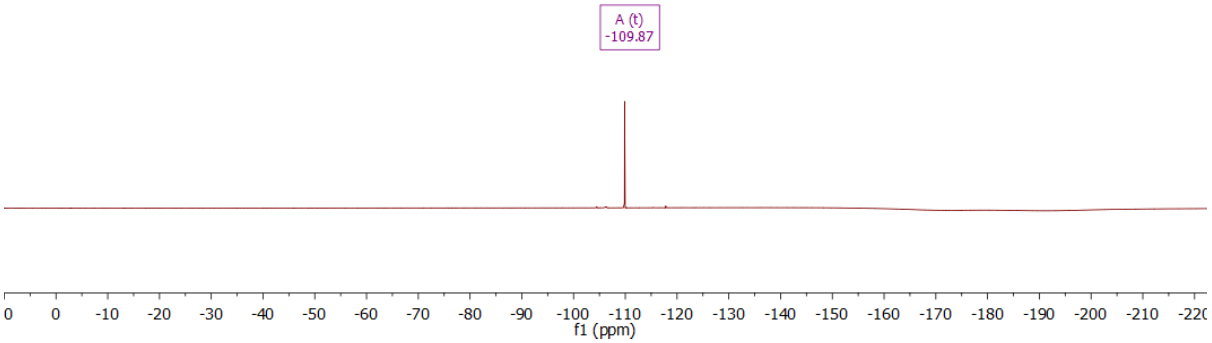
A (t)
-109.87
0 0 -10 -20 -30 -40 -50 -60 -70 -80 -90 -100 -110 -120 -130 -140 -150 -160 -170 -180 -190 -200 -210 -220
f1 (ppm)

3-((6-bromobenzo[d][1,3]dioxol-5-yl)methyl)-2-phenyl-1H-indole [**1aj**]:

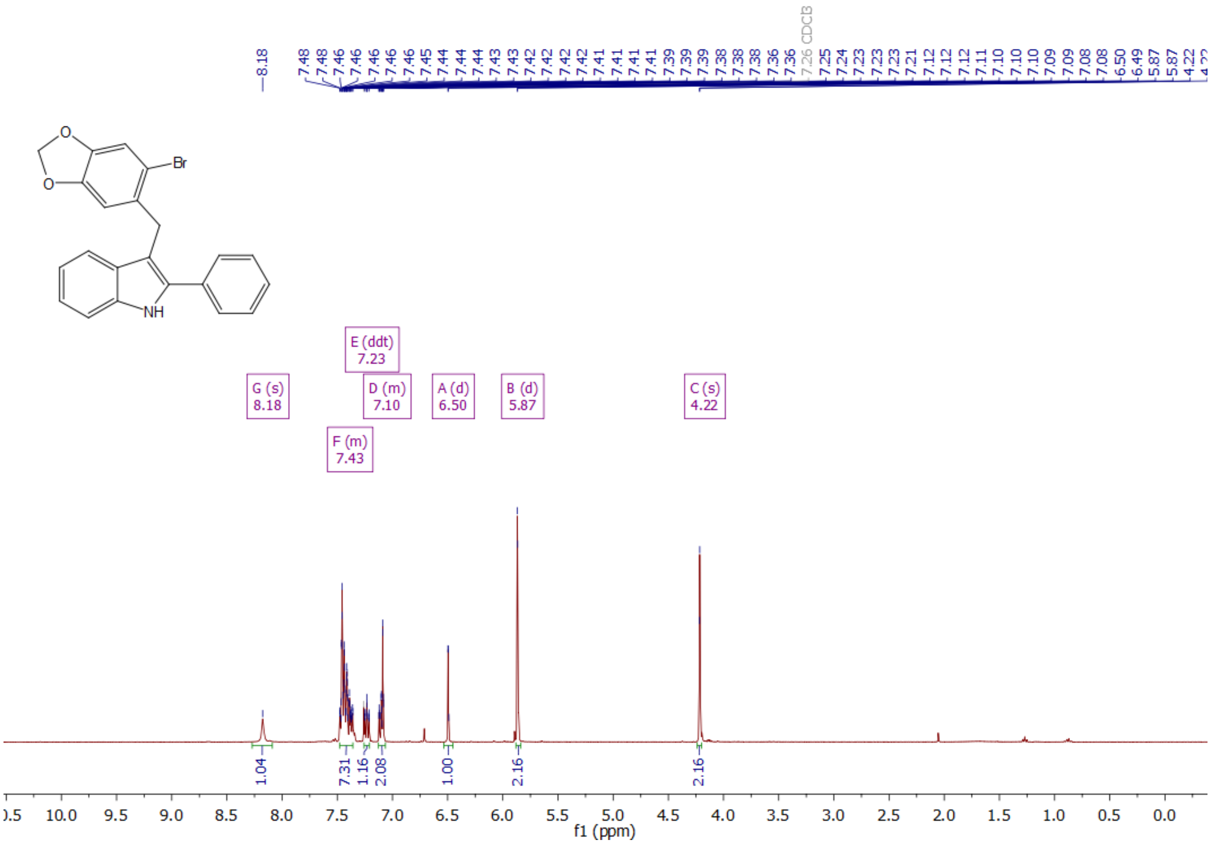

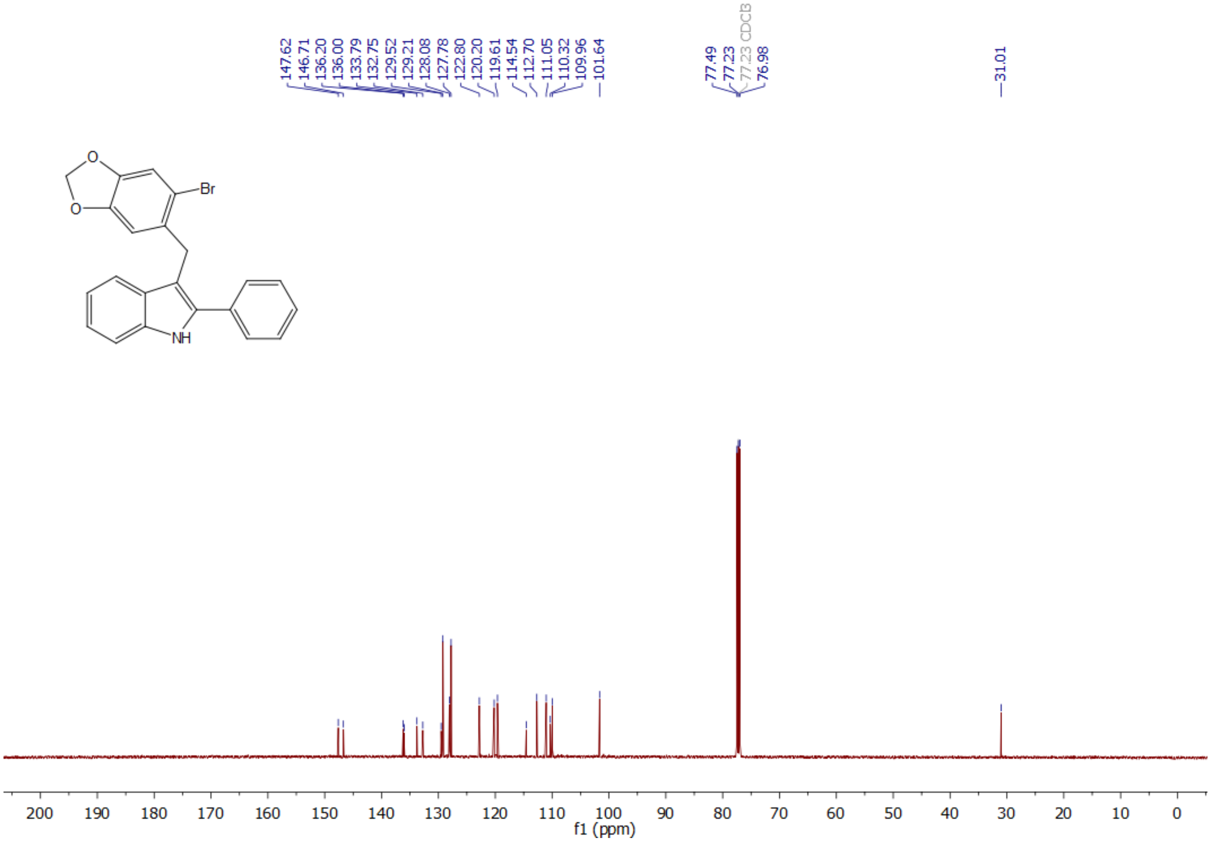

3-(2-bromobenzyl)-2-(4-chlorophenyl)-1H-indole [**1ak**]:

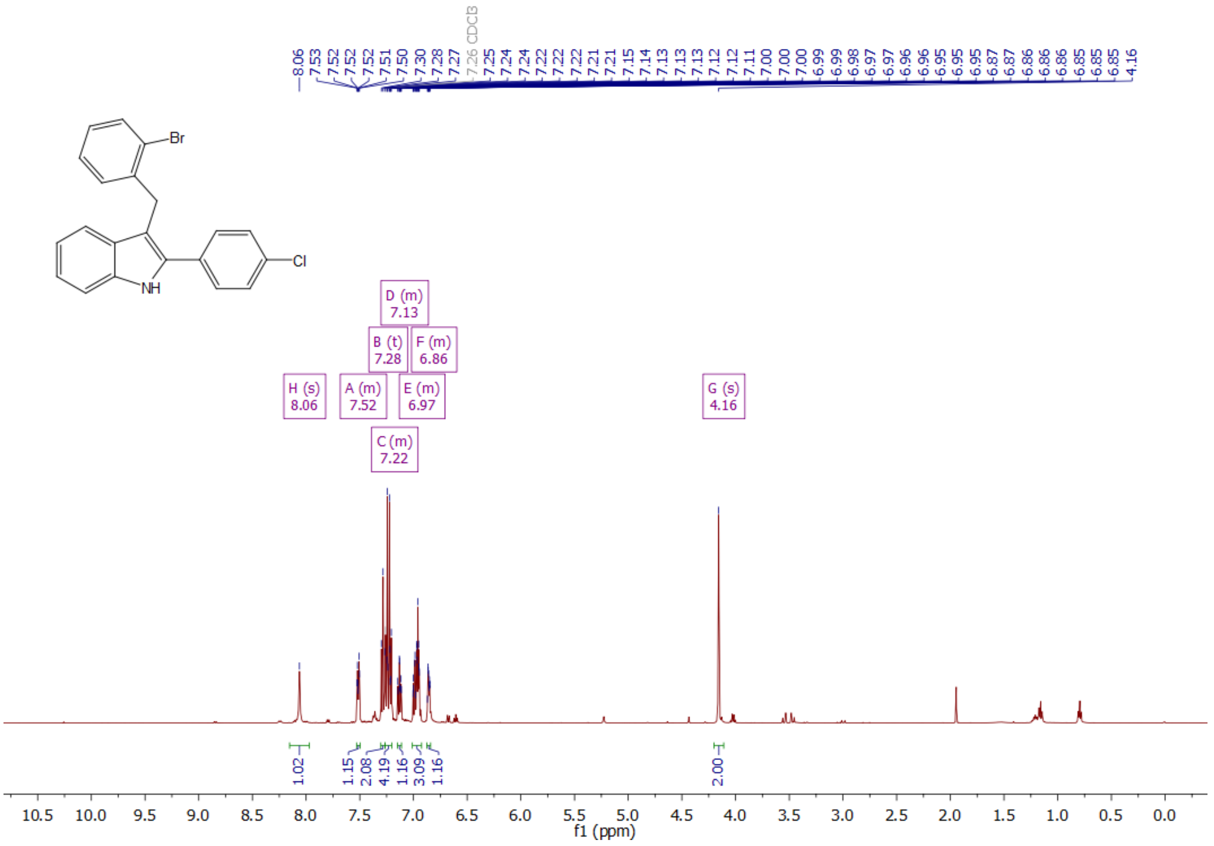

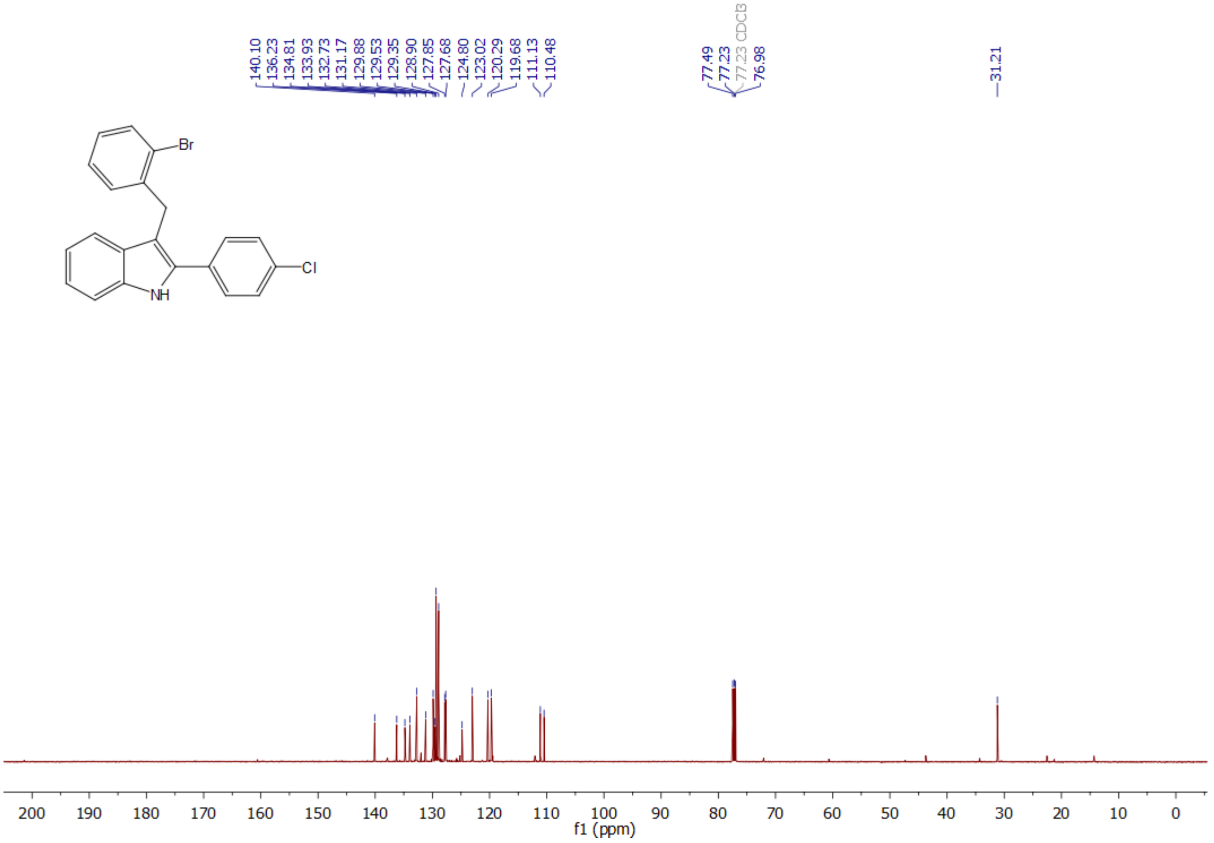

***3-(2-bromo-5-fluorobenzyl)-2-(4-chlorophenyl)-1H-indole* [1al]:**

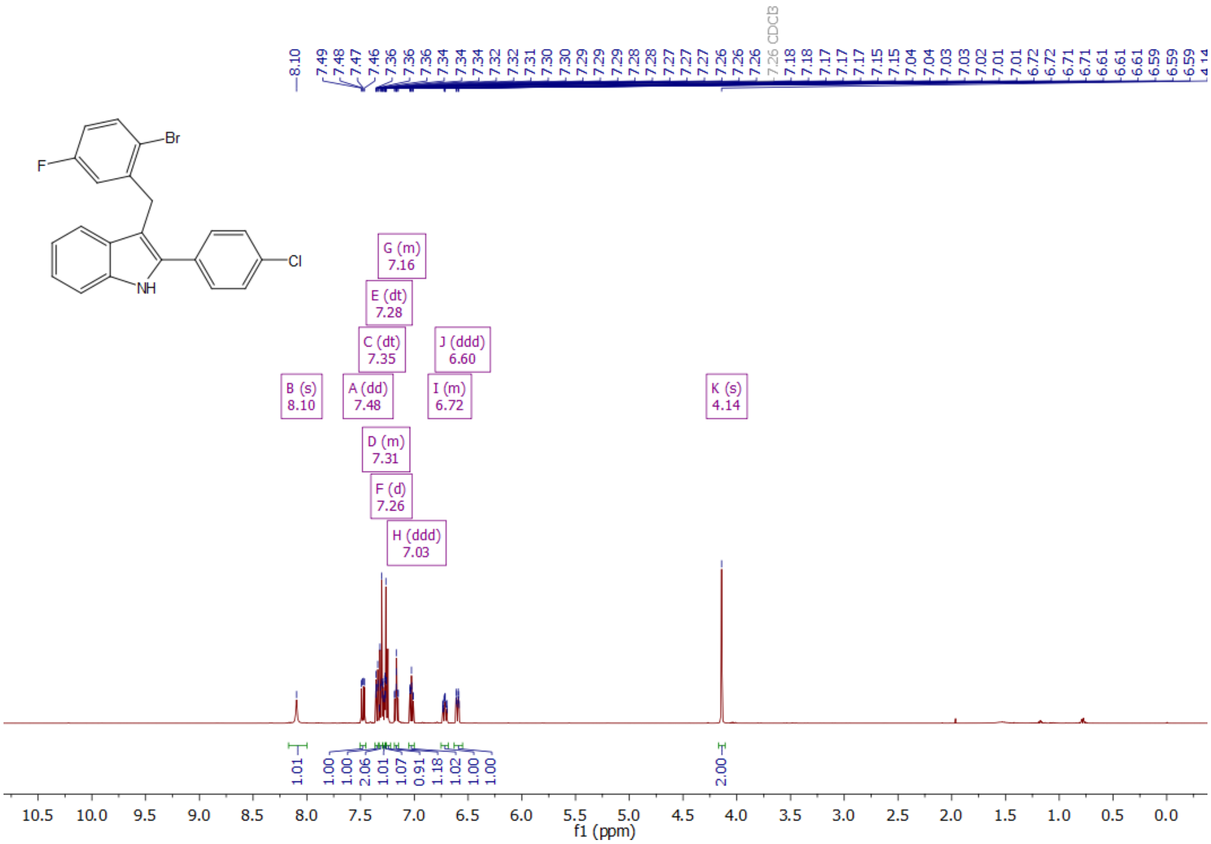

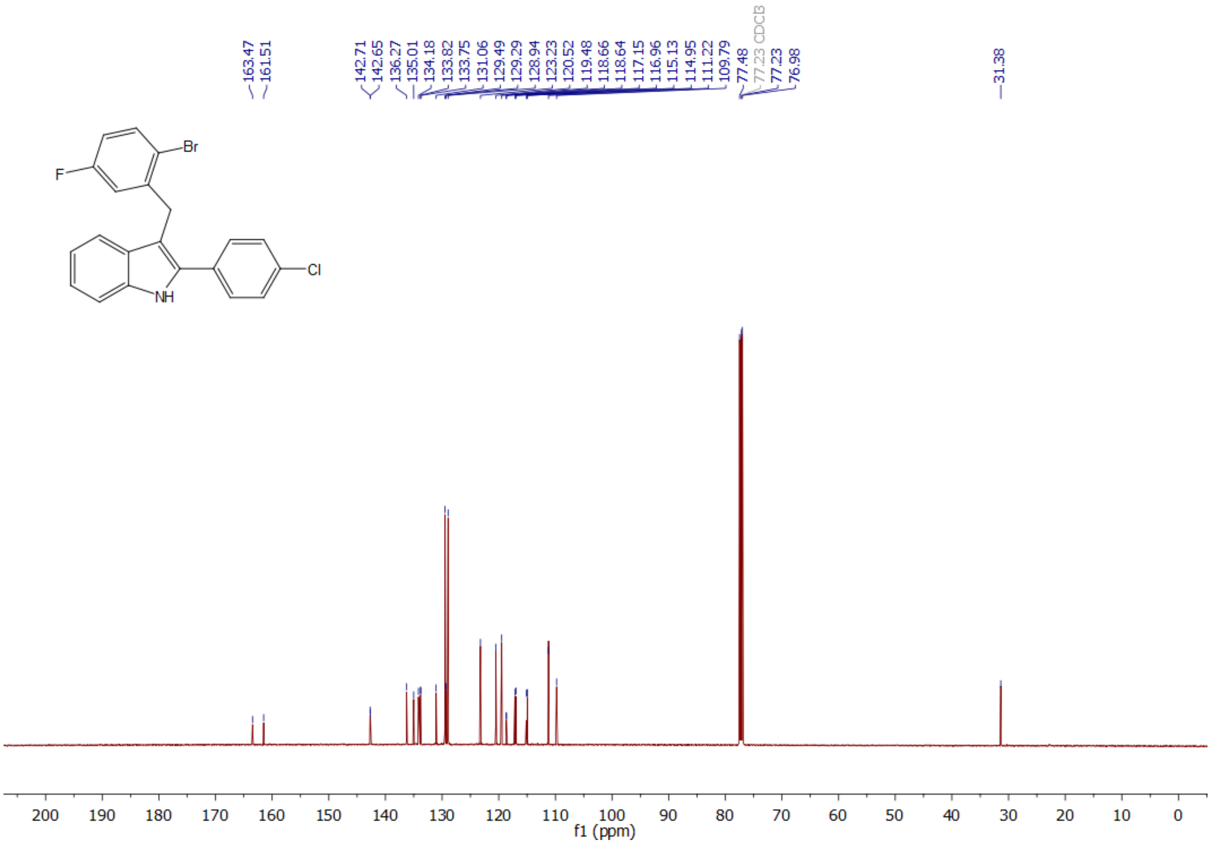

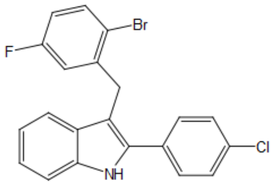
F
Br
NH
Cl

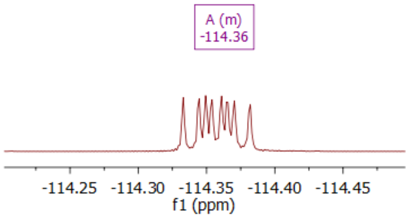
A (m)
-114.36
-114.25 -114.30 -114.35 -114.40 -114.45
f1 (ppm)

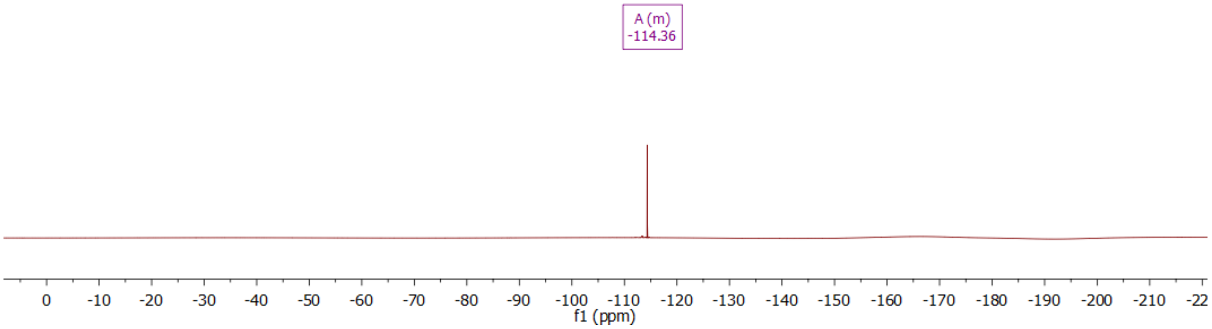
A (m)
-114.36
0 -10 -20 -30 -40 -50 -60 -70 -80 -90 -100 -110 -120 -130 -140 -150 -160 -170 -180 -190 -200 -210 -22
f1 (ppm)

3-(2-bromo-6-fluorobenzyl)-2-(4-chlorophenyl)-1H-indole [**1am**]:

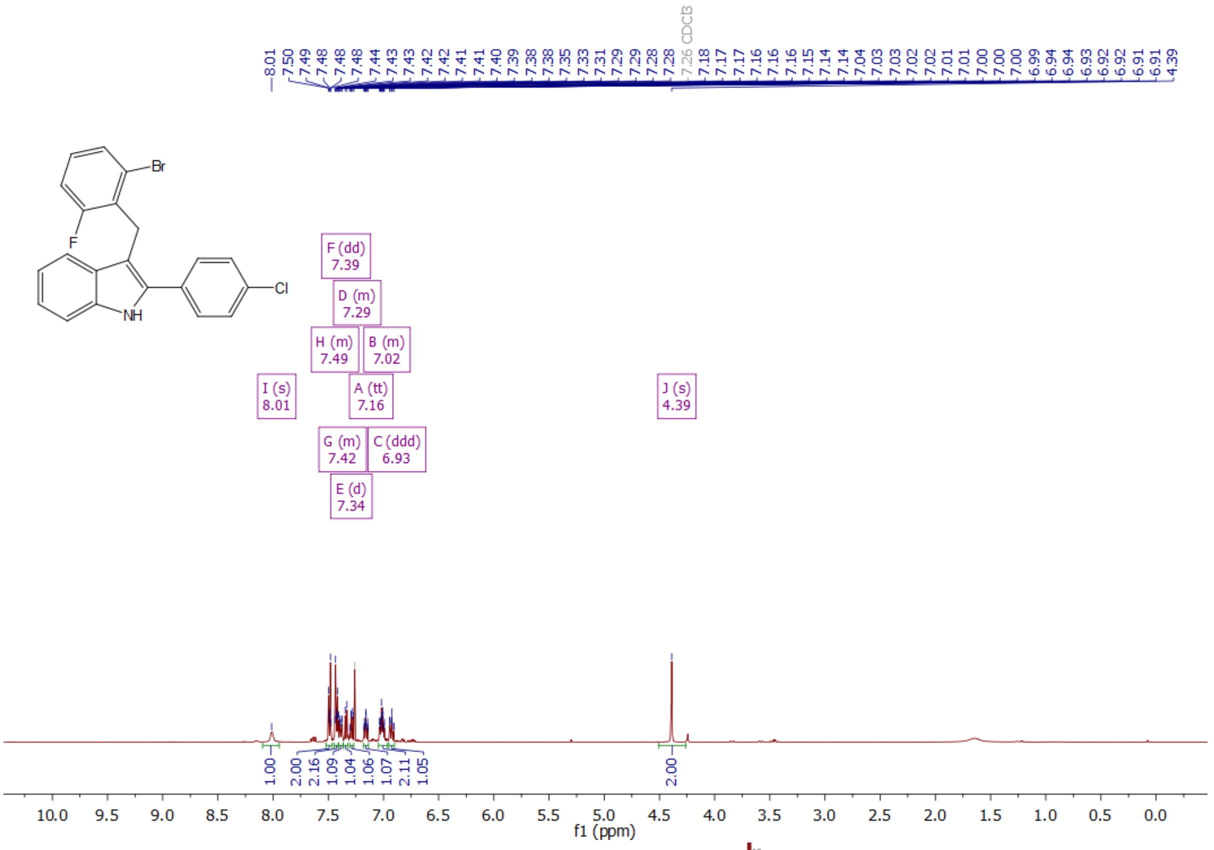

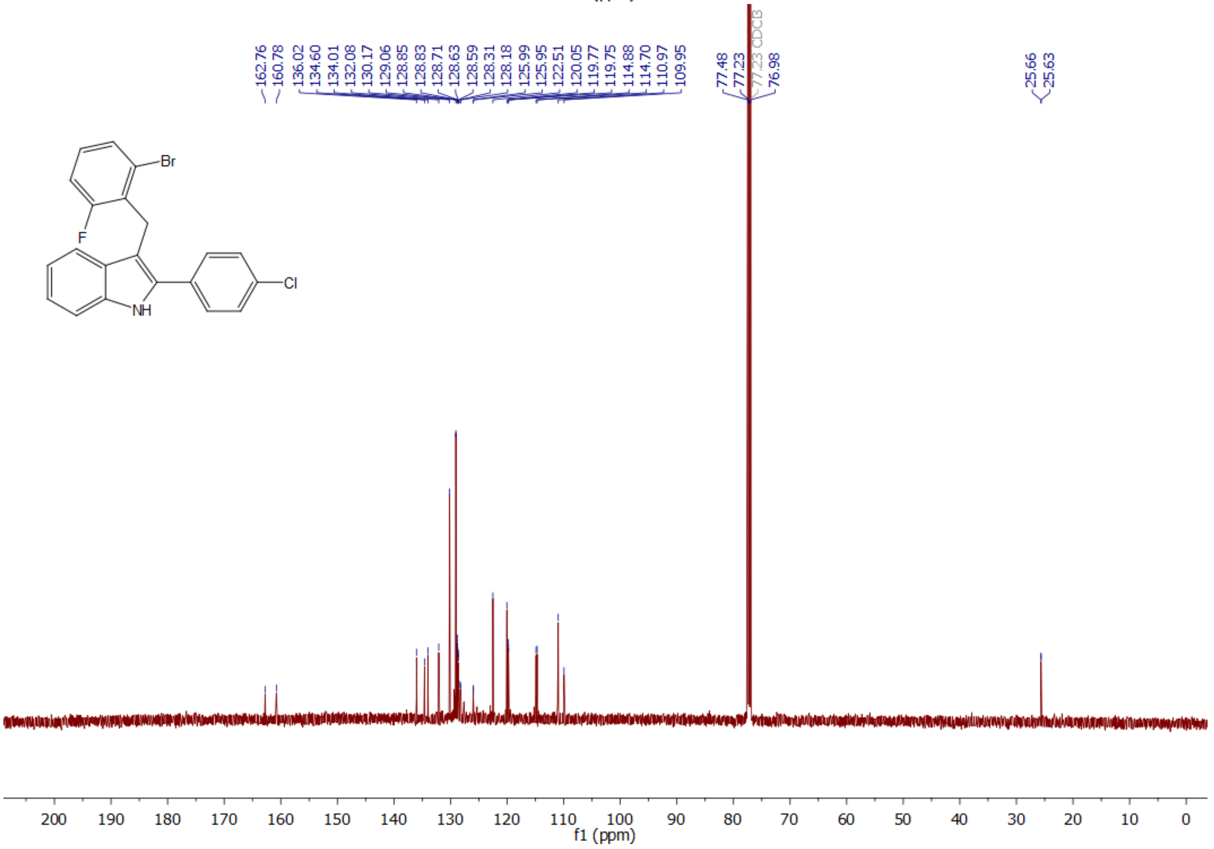

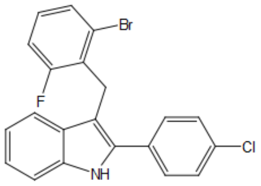
Br
F
NH
Cl

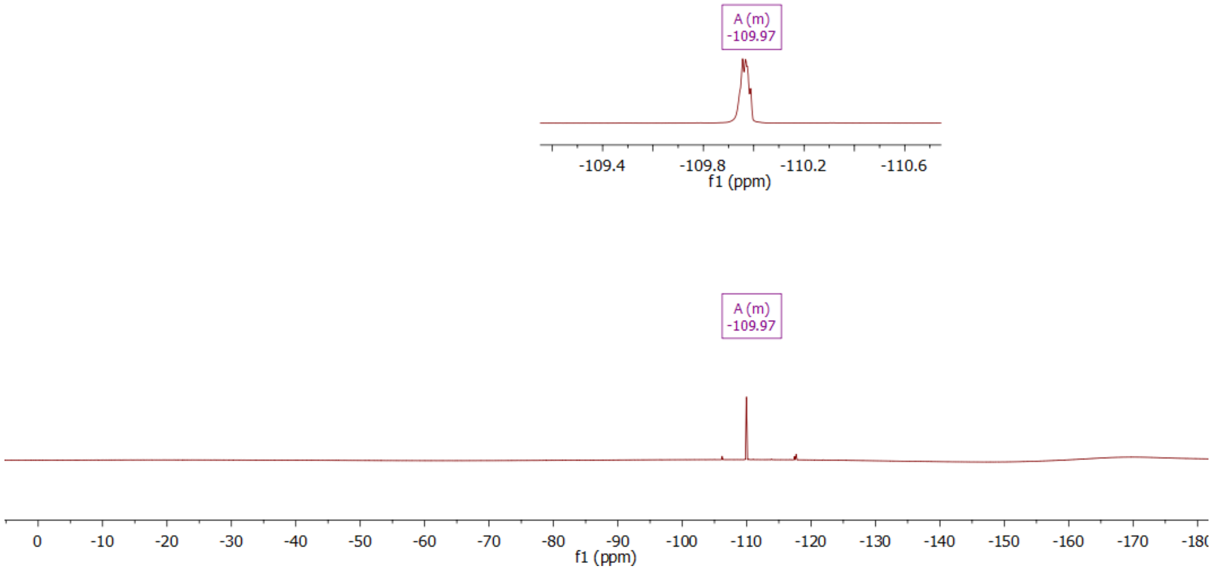
A (m)
-109.97
-109.4
-109.8
-110.2
-110.6
f1 (ppm)
A (m)
-109.97
0
-10
-20
-30
-40
-50
-60
-70
-80
-90
-100
-110
-120
-130
-140
-150
-160
-170
f1 (ppm)

CLASS-A

2'-methylspiro[indene-2,3'-indol]-1(3H)-one [**A1**]:

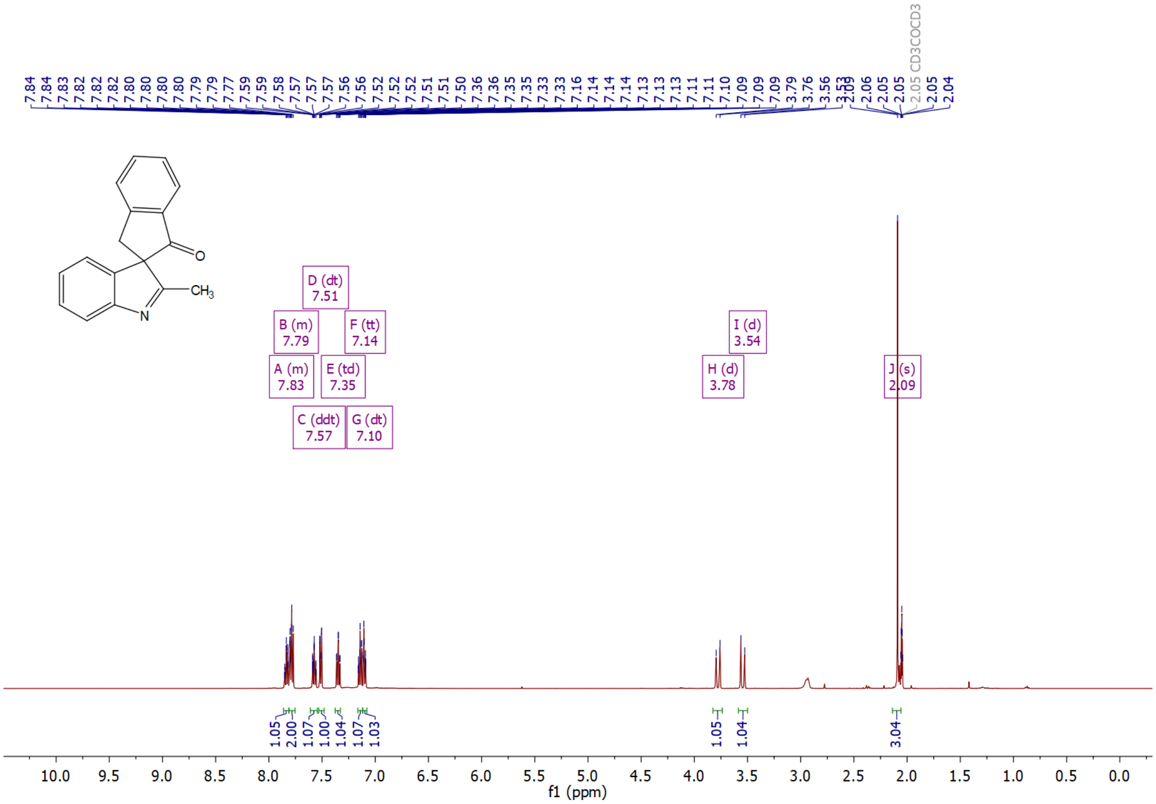

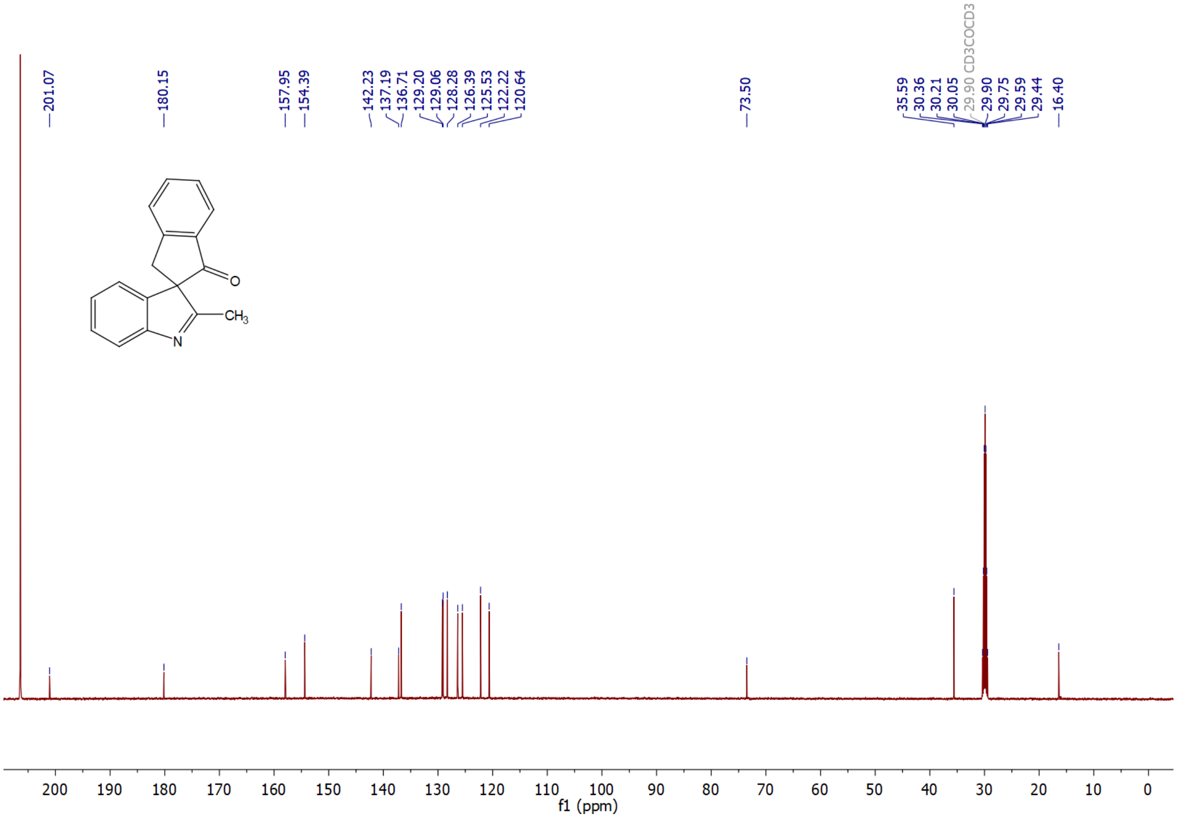

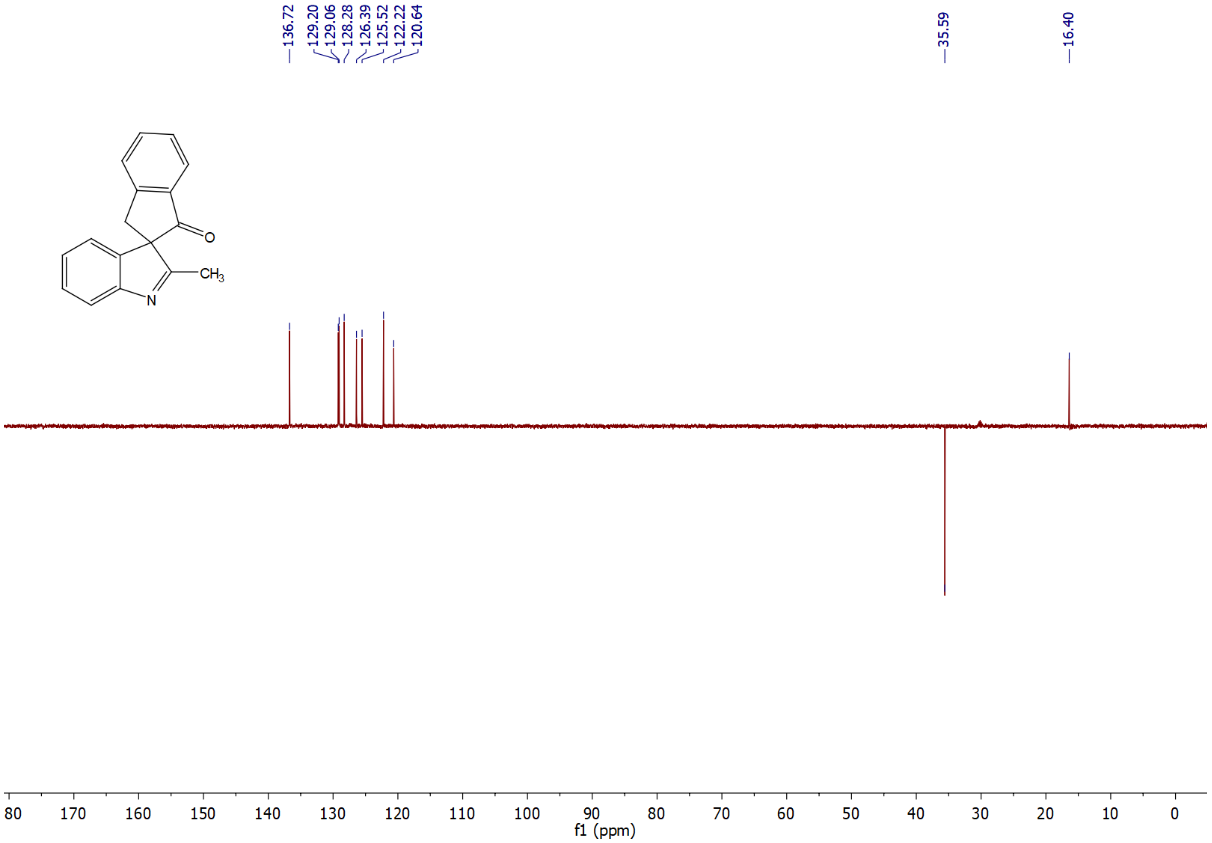
136.72
129.20
129.06
128.28
126.39
125.52
122.22
120.64
35.59
16.40
O
CH3
N
80
170
160
150
140
130
120
110
100
90
80
70
60
50
40
30
20
10
0
f1 (ppm)

7-fluoro-2'-methylspiro[indene-2,3'-indol]-1(3H)-one [A2]:

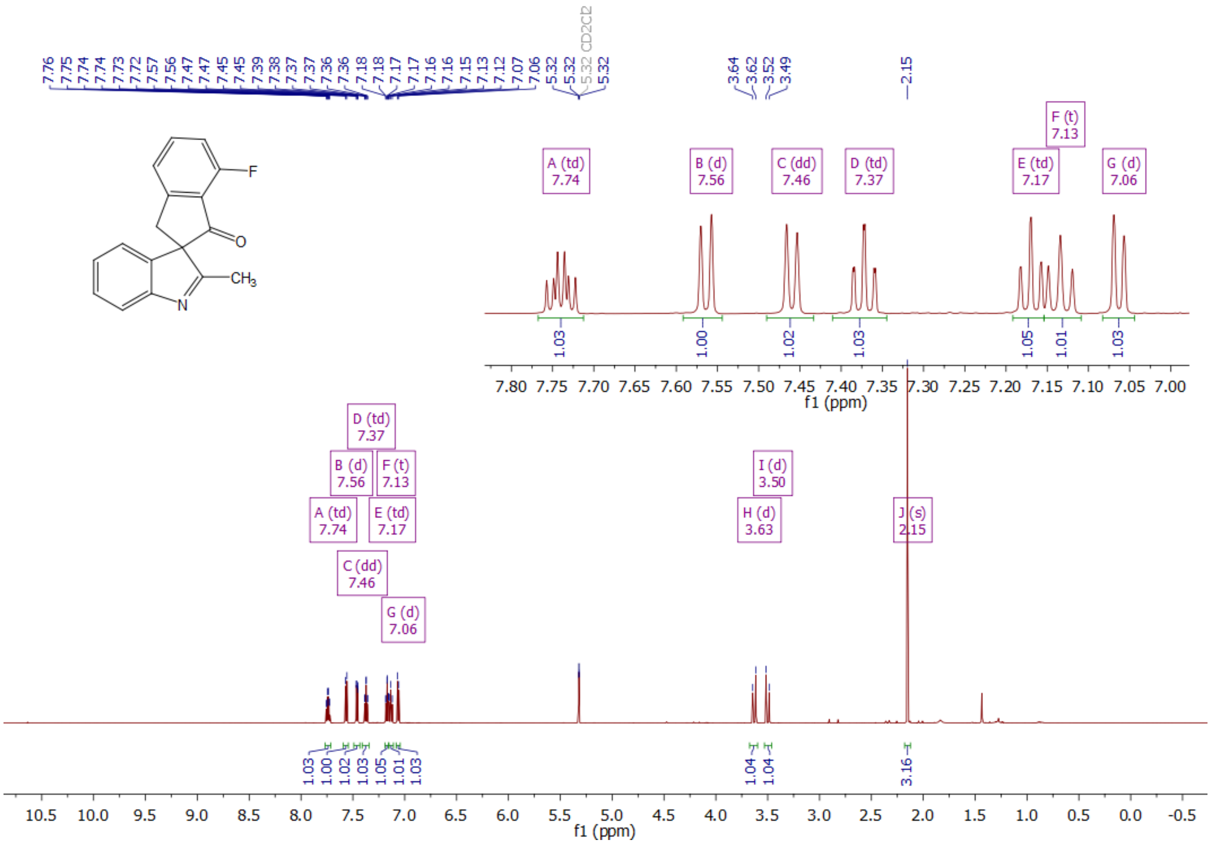

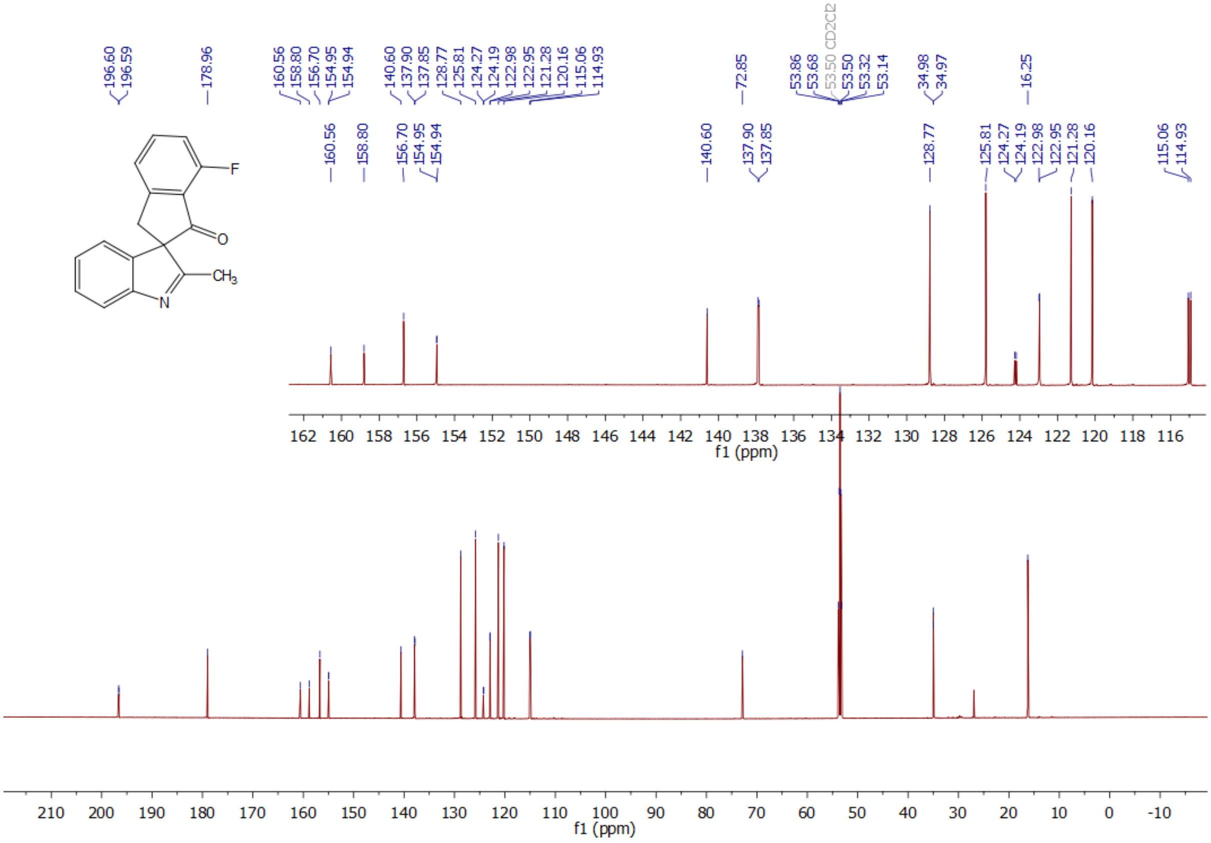

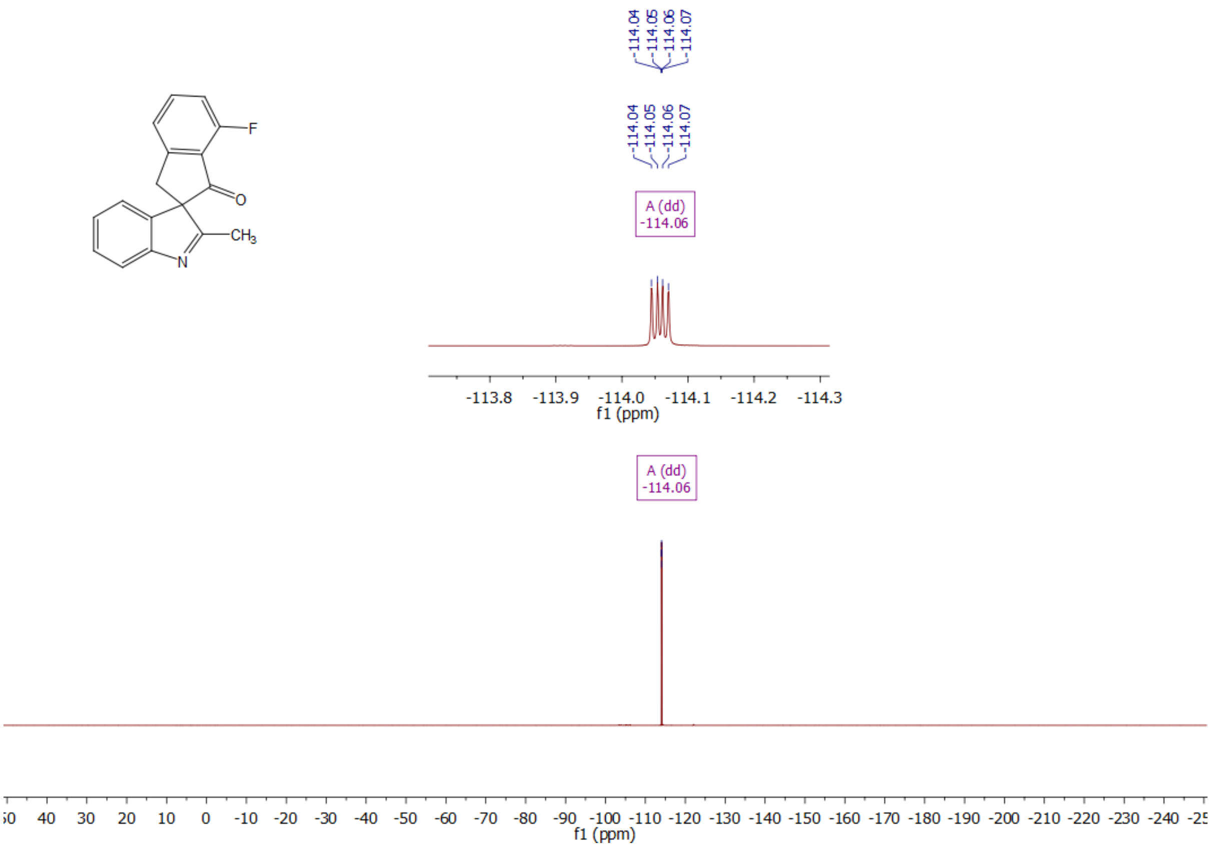
F
O
CH3
N
-114.04
-114.05
-114.06
-114.07
-114.04
-114.05
-114.06
-114.07
A (dd)
-114.06
-113.8 -113.9 -114.0 -114.1 -114.2 -114.3
f1 (ppm)
A (dd)
-114.06
40 30 20 10 0 -10 -20 -30 -40 -50 -60 -70 -80 -90 -100 -110 -120 -130 -140 -150 -160 -170 -180 -190 -200 -210 -220 -230 -240
f1 (ppm)

***2',6-dimethylspiro[indene-2,3'-indol]-1(3H)-one* [A3]:**

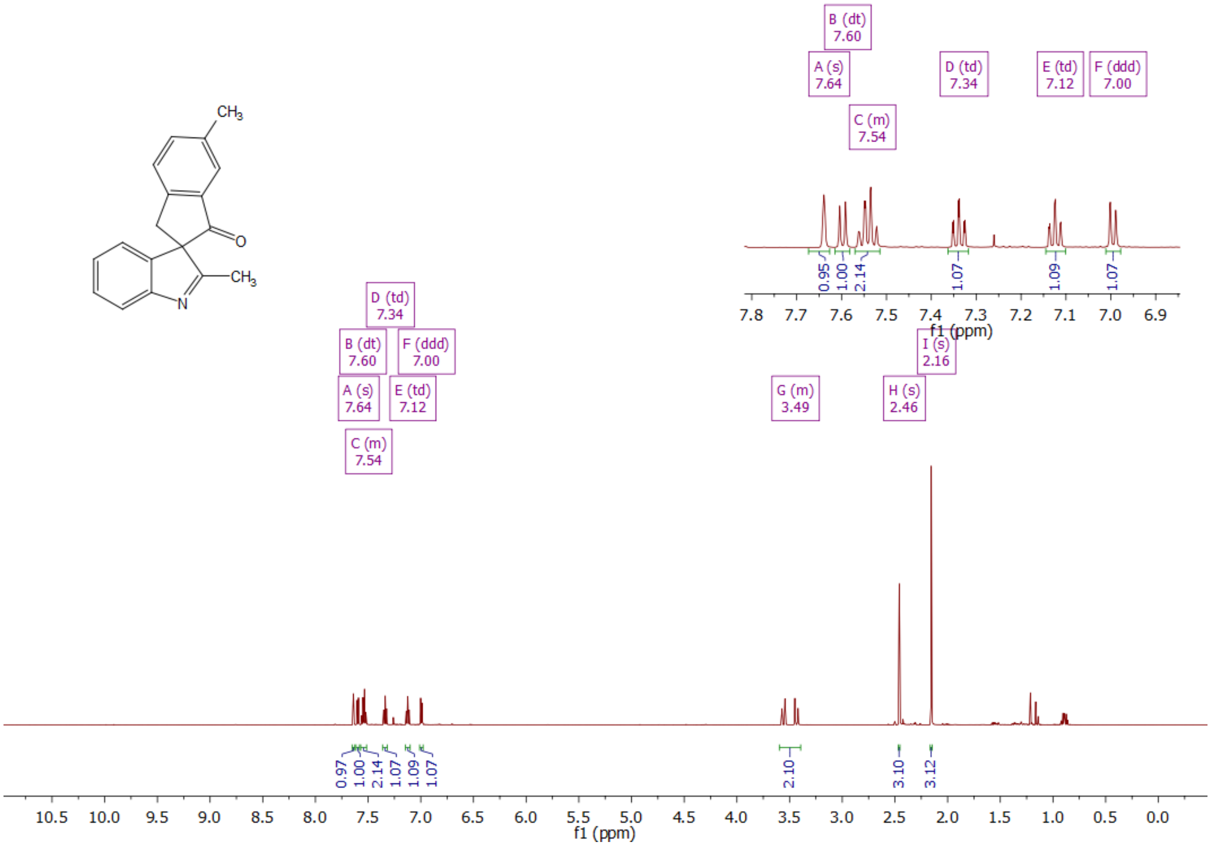

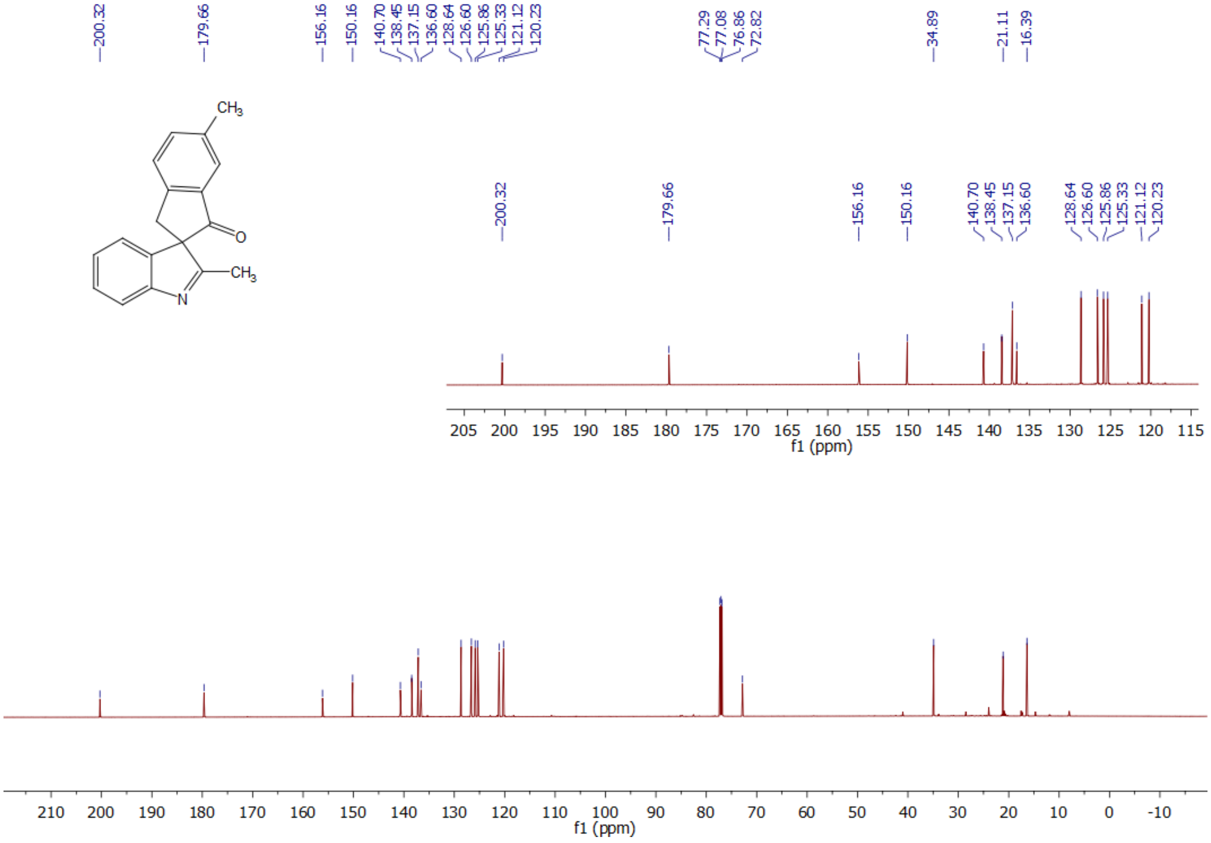

6-chloro-2'-methylspiro[indene-2,3'-indol]-1(3H)-one [**A4**]:

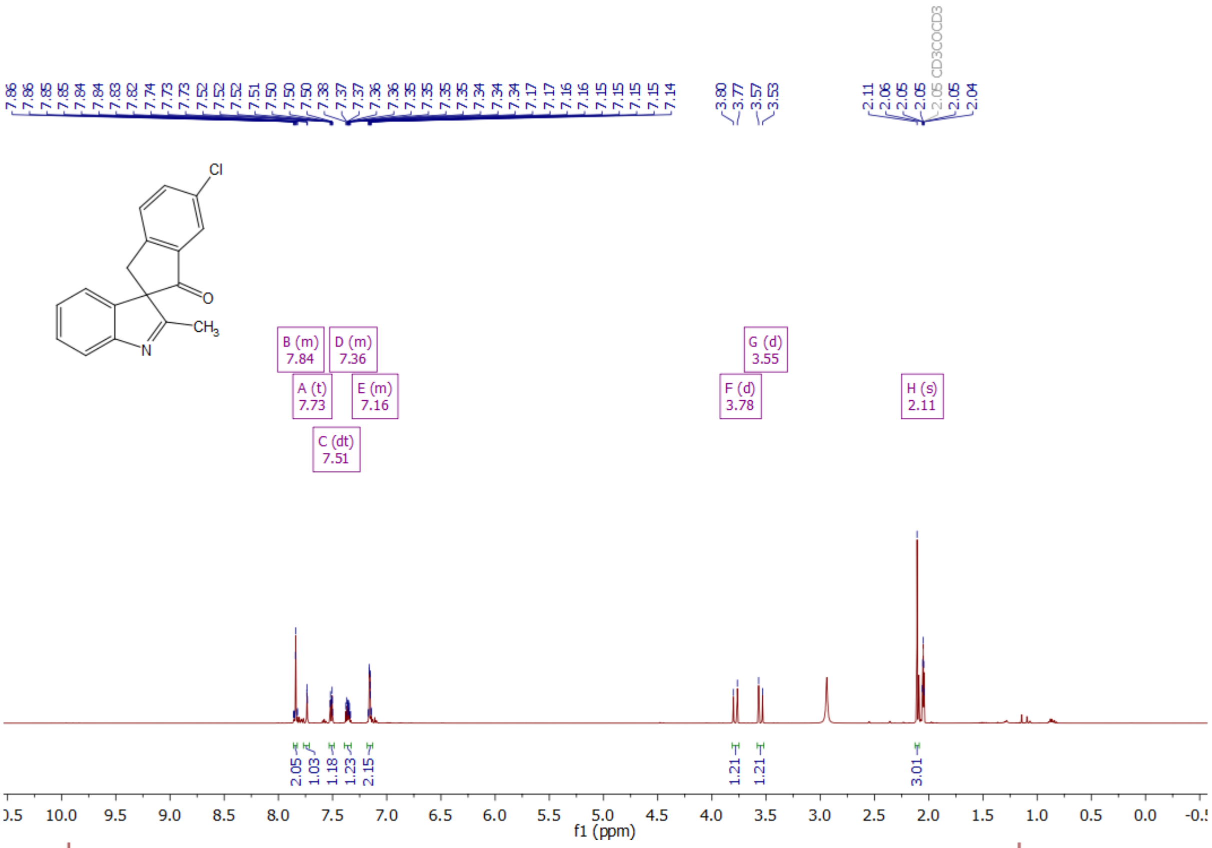

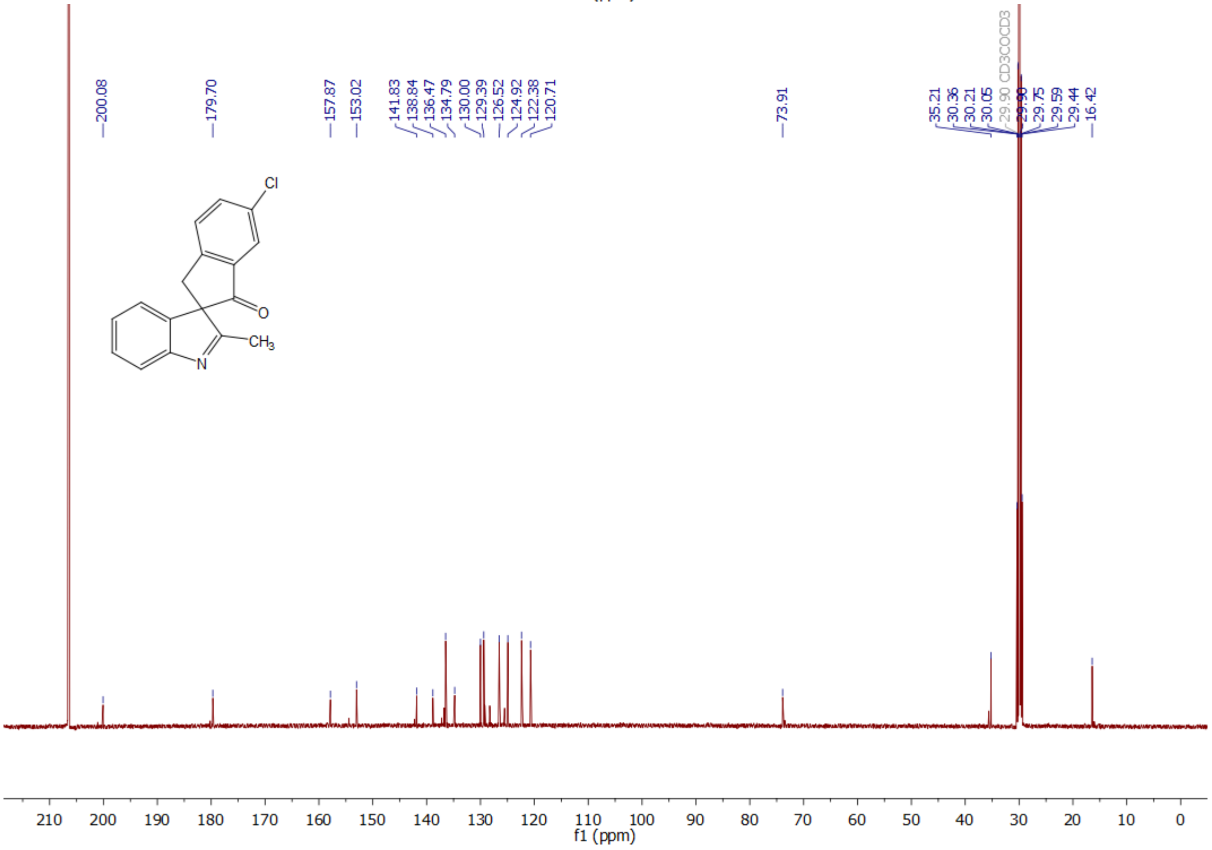

5-methoxy-2'-methylspiro[indene-2,3'-indol]-1(3H)-one [**A5**]:

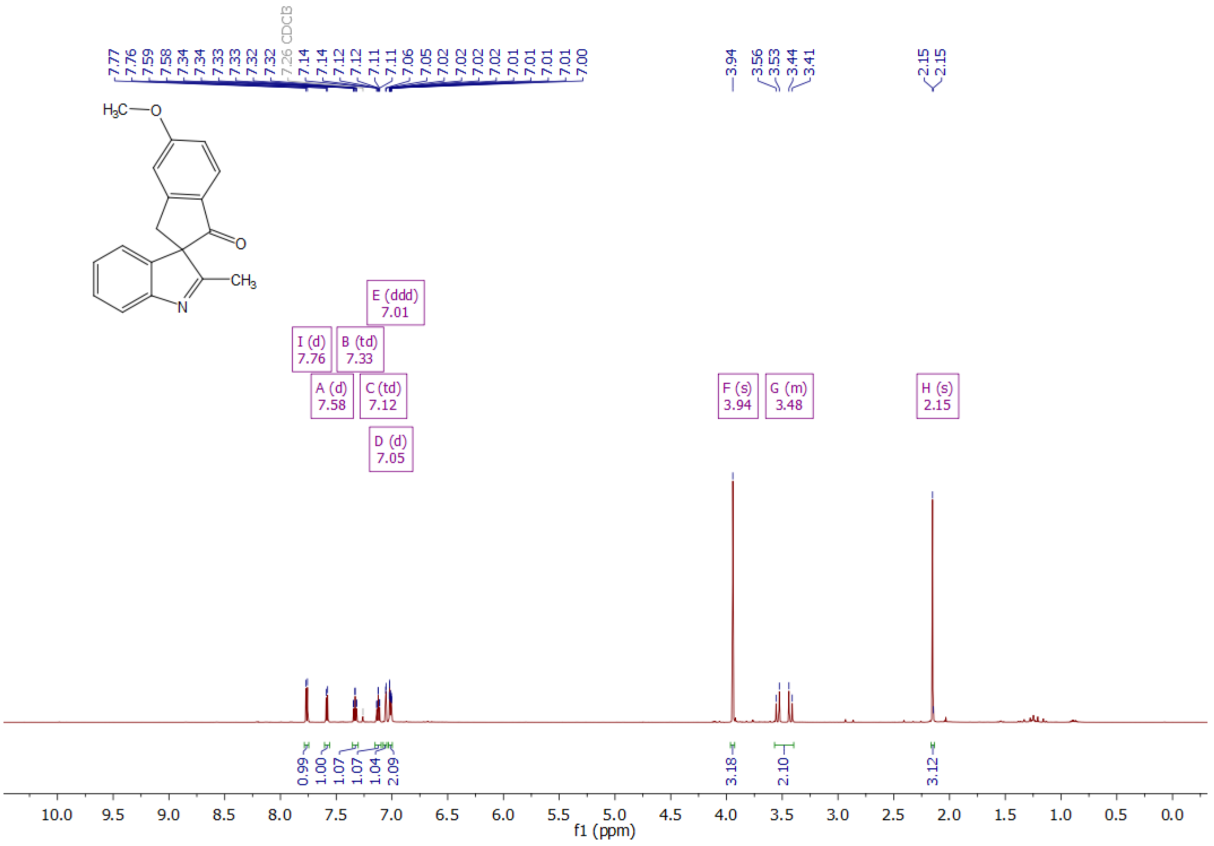

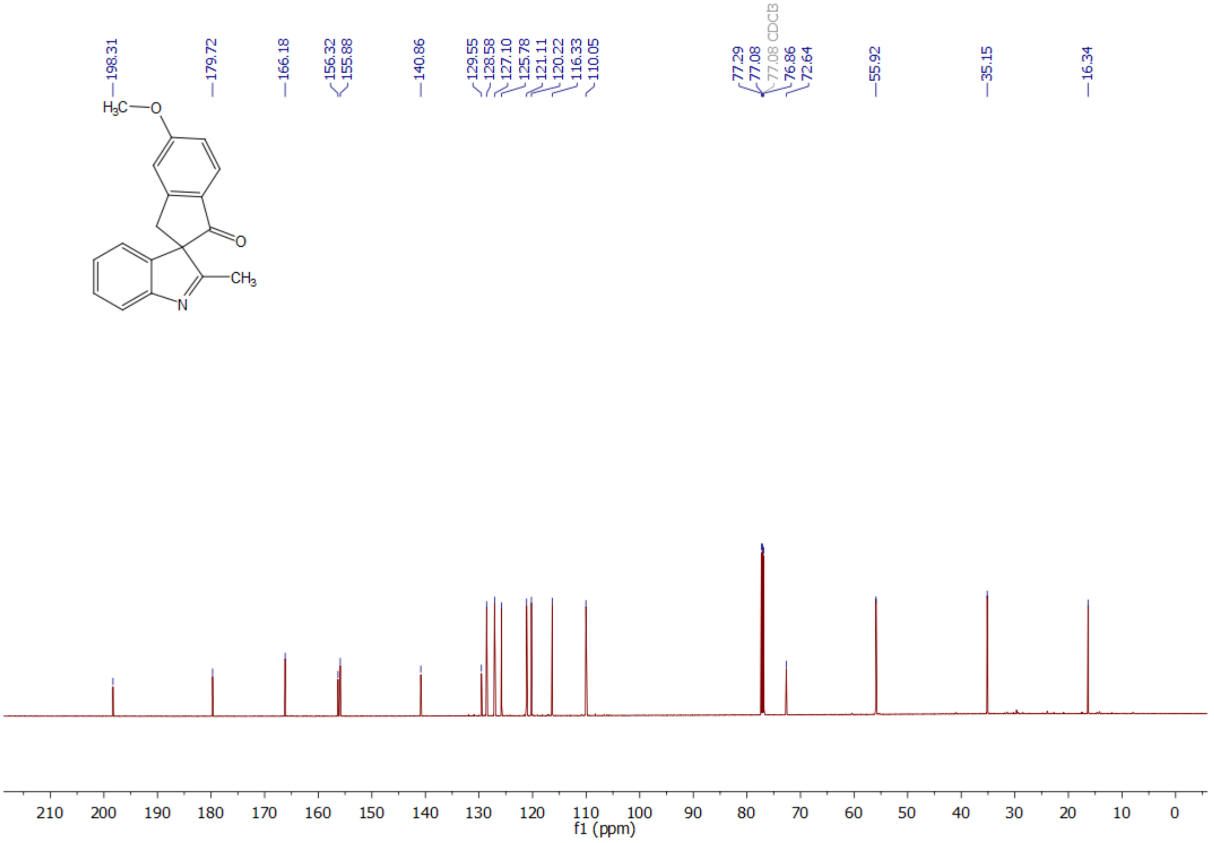

5-fluoro-2'-methylspiro[indene-2,3'-indol]-1(3H)-one [**A6**]:

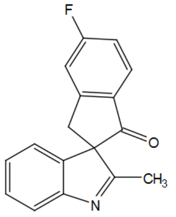

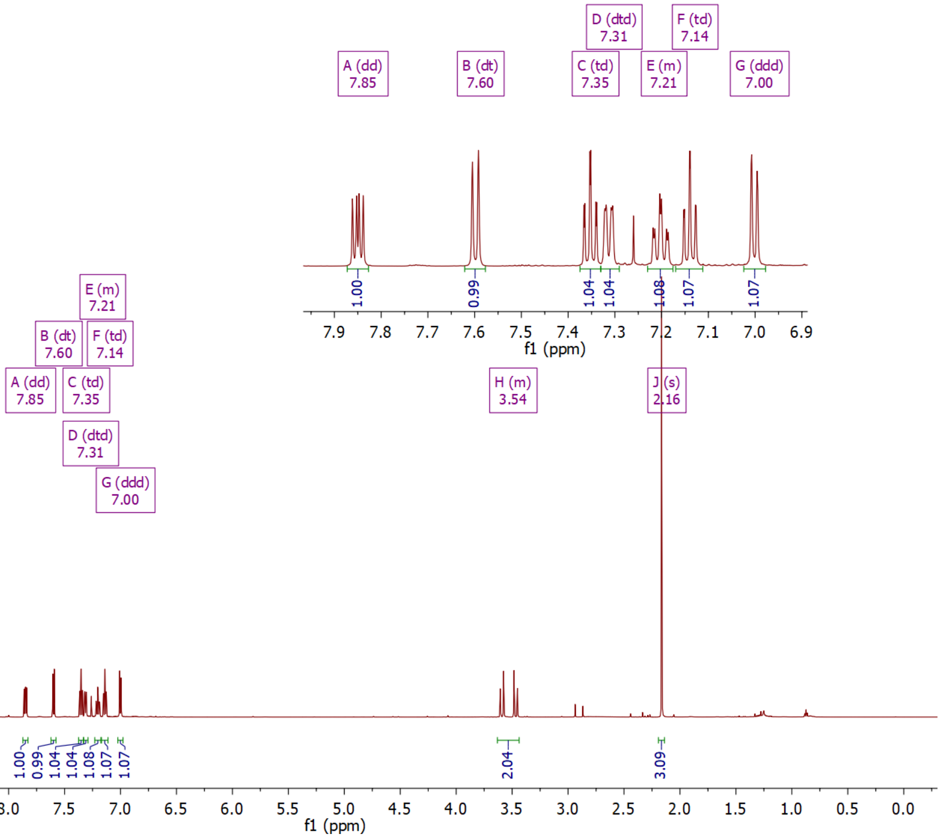

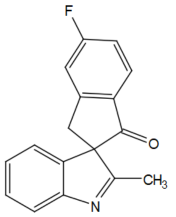

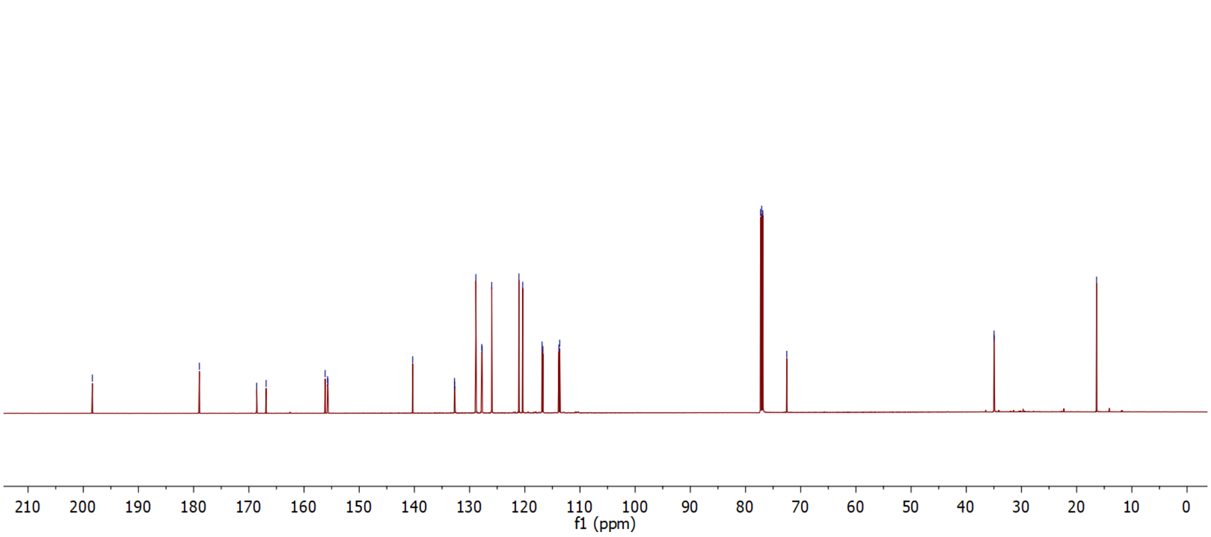

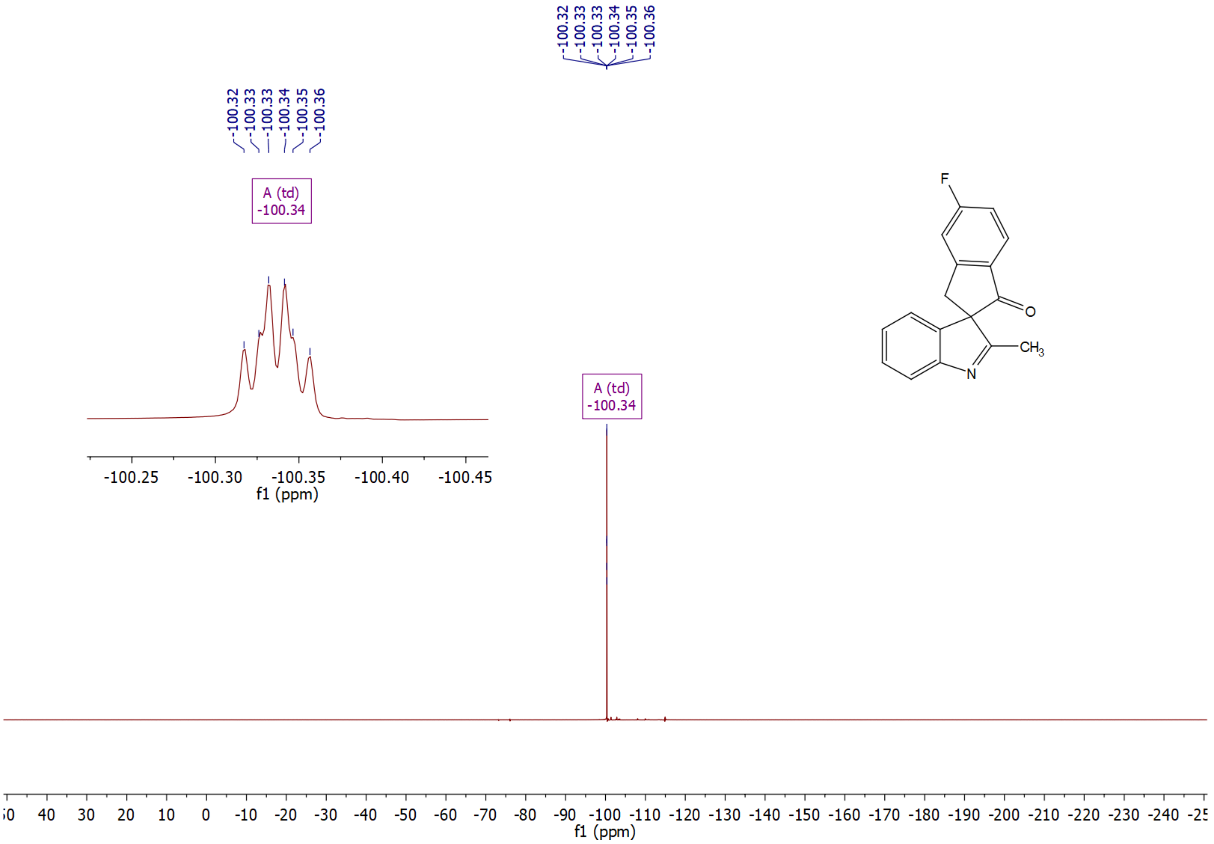

5-fluoro-2'-methylspiro[indene-2,3'-indol]-1(3H)-one [A6-resynthesized]:

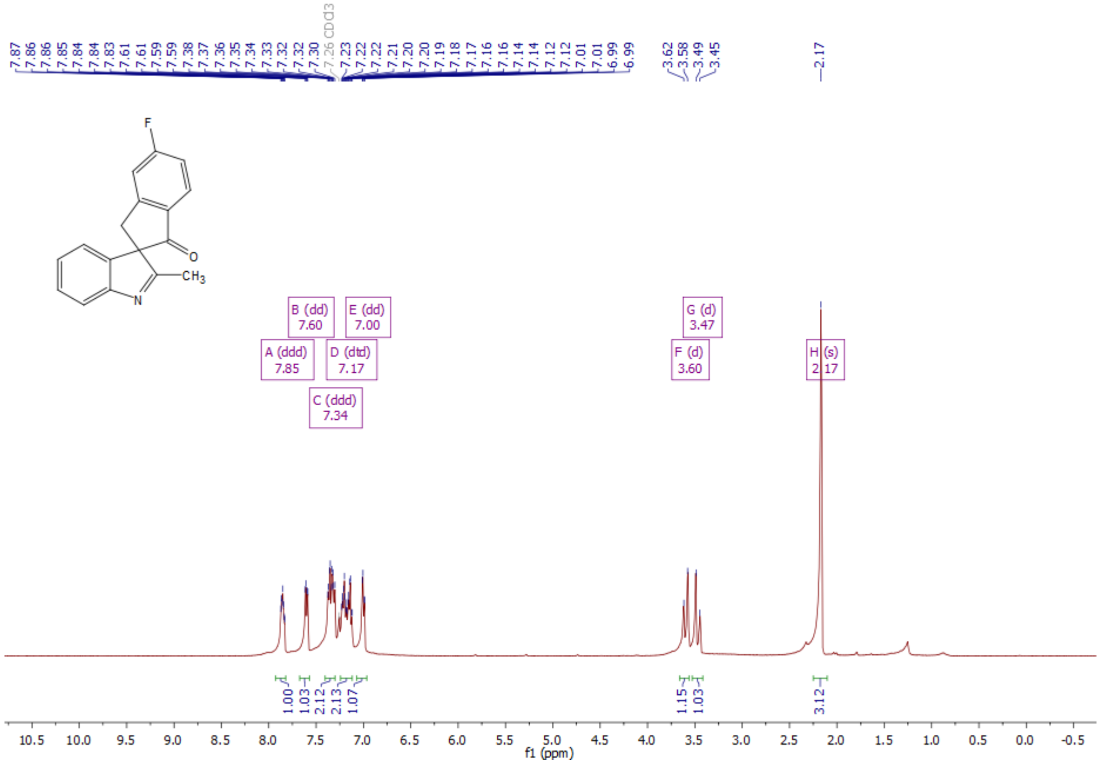

4-fluoro-2'-methylspiro[indene-2,3'-indol]-1(3H)-one [**A7**]:

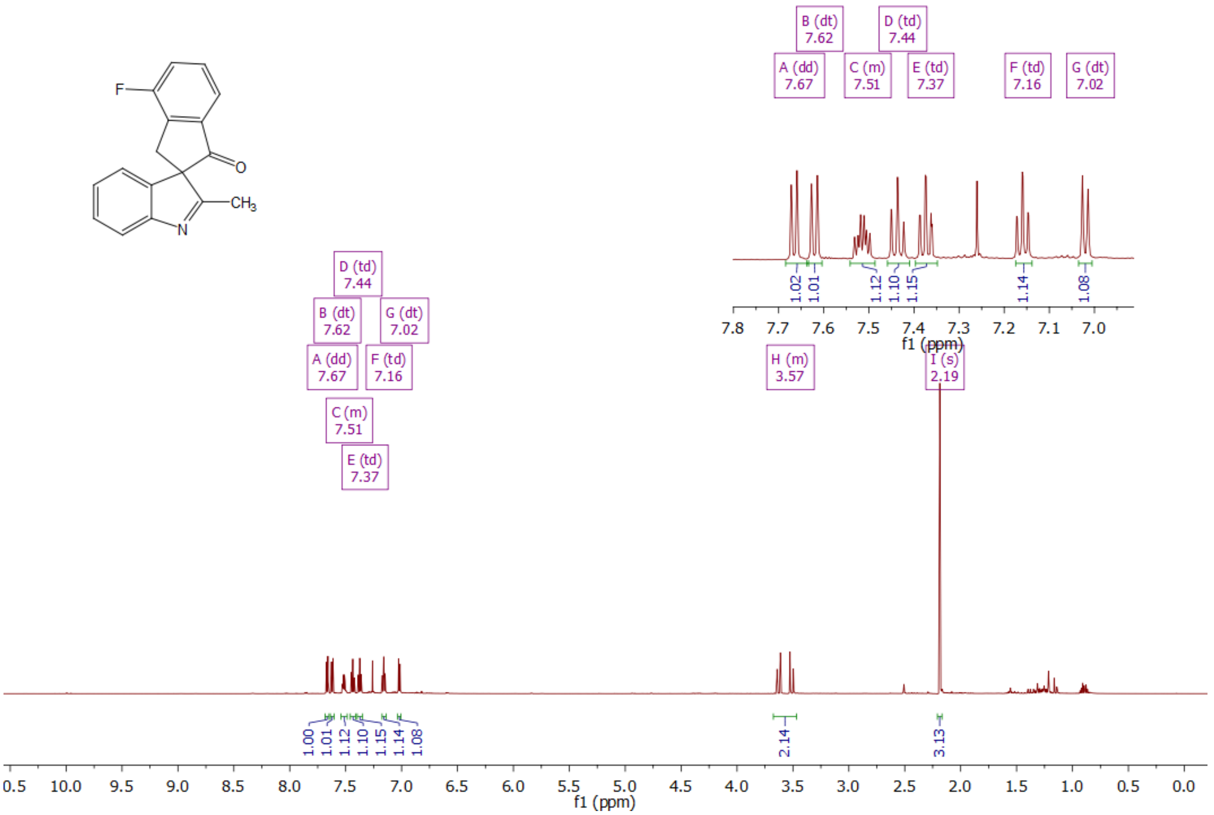

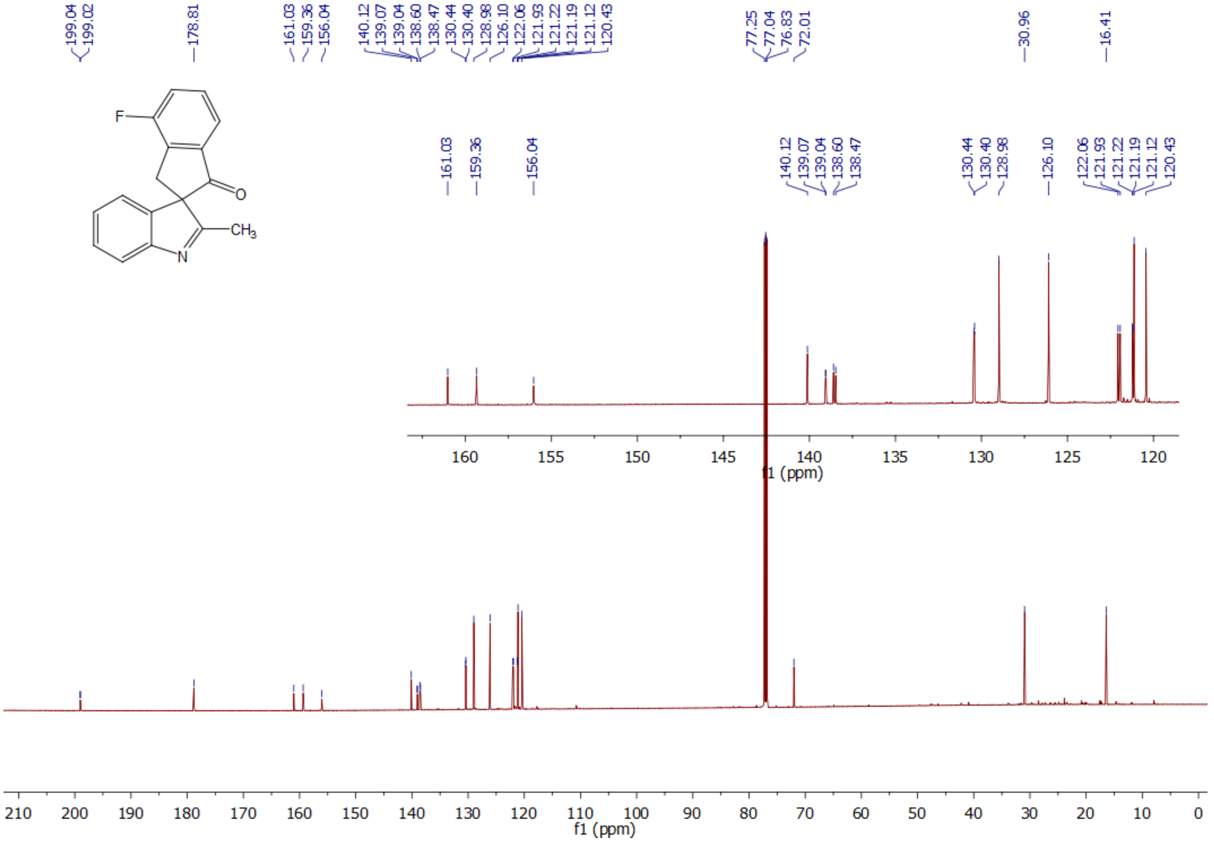

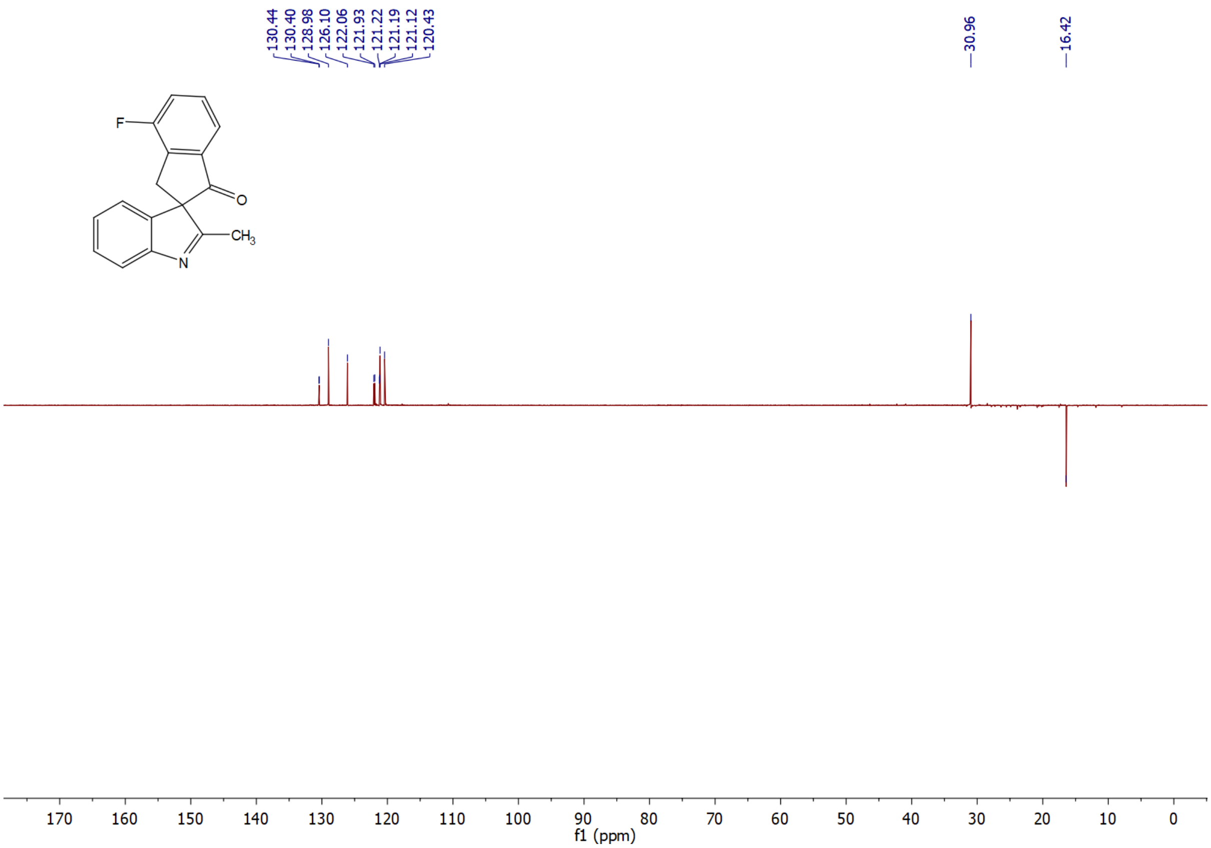
130.44
130.40
128.98
126.10
122.06
121.93
121.22
121.19
121.12
120.43
30.96
16.42
F
O
CH3
N
f1 (ppm)

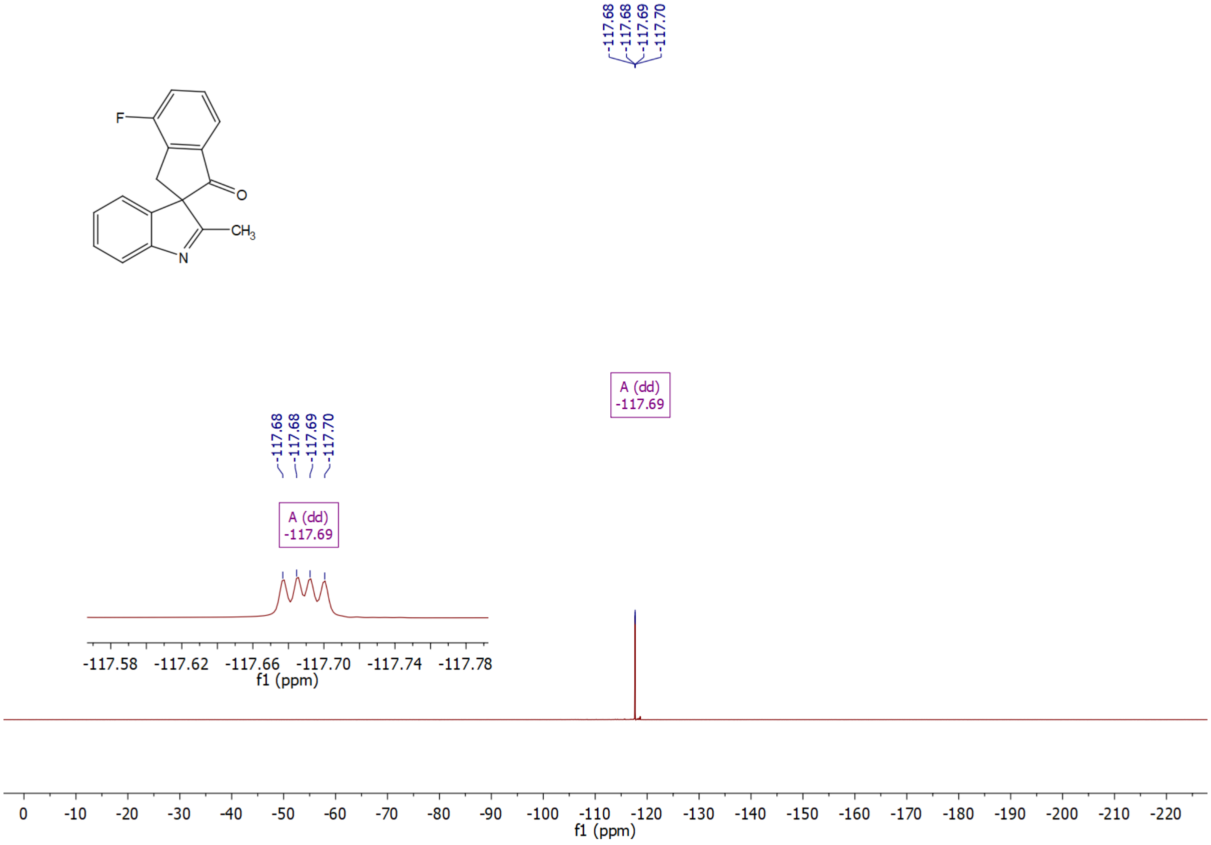
-117.68
-117.68
-117.69
-117.70
F
O
CH3
N
A (dd)
-117.69
f1 (ppm)

6-fluoro-5-methoxy-2'-methylspiro[indene-2,3'-indol]-1(3H)-one [**A8**]:

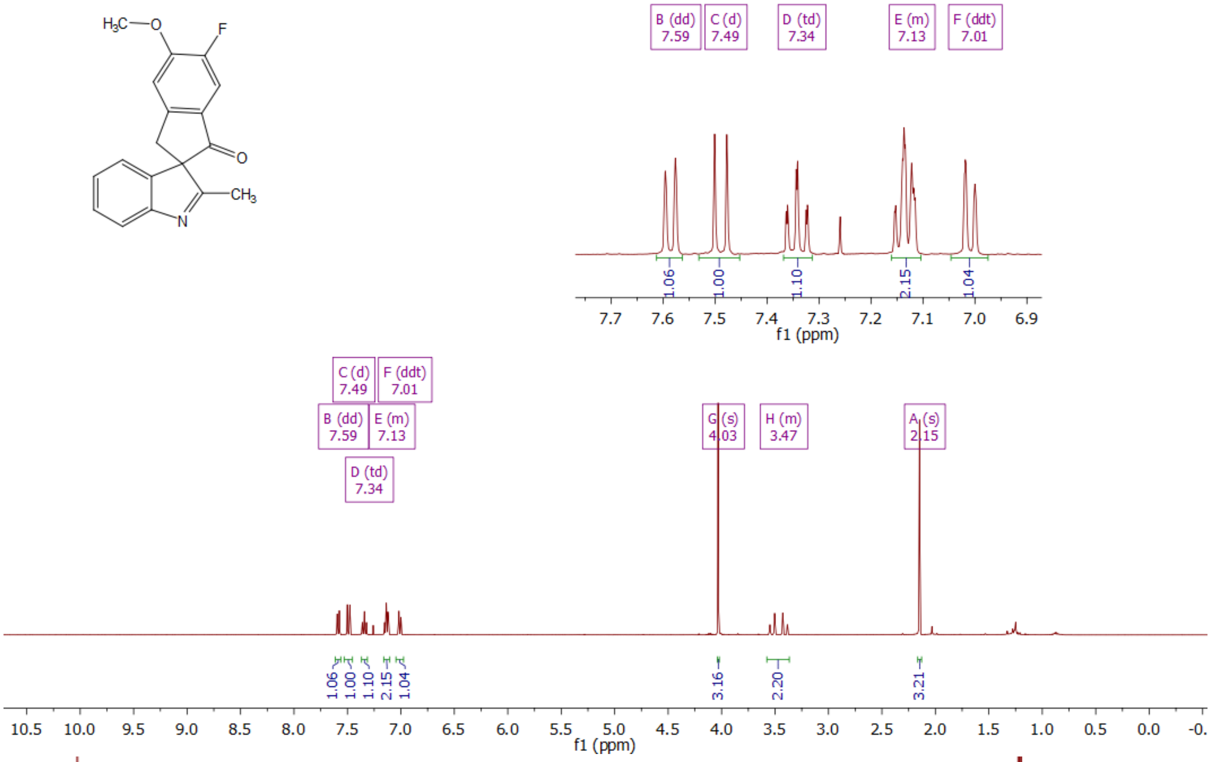

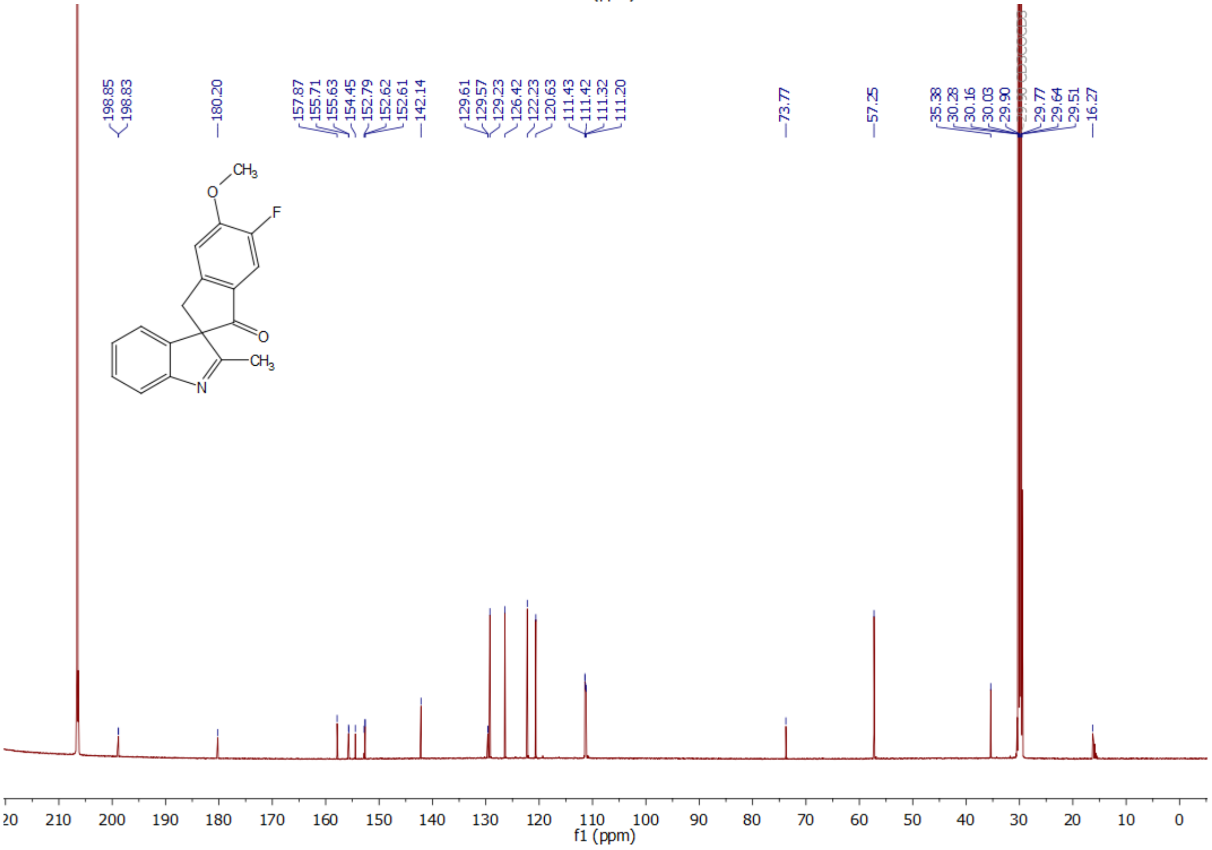

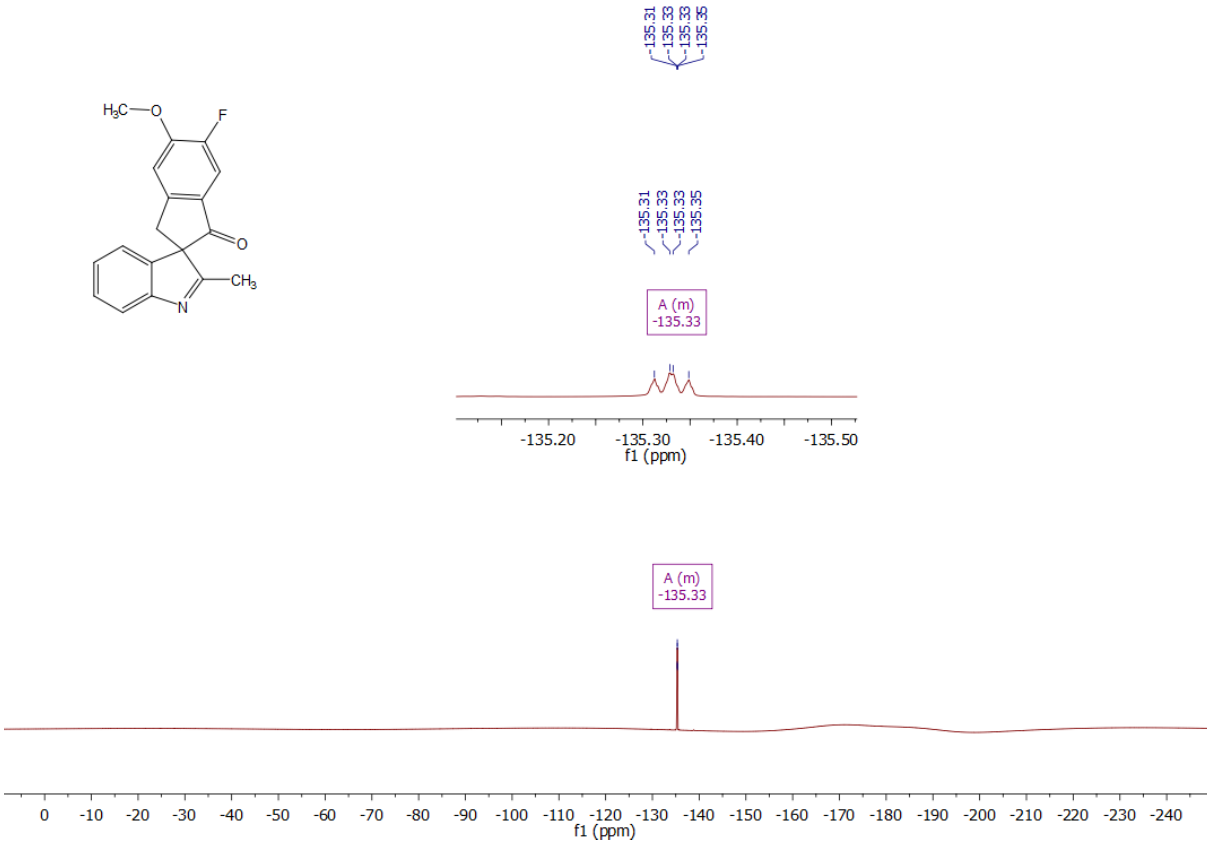

6-fluoro-5-methoxy-2'-methylspiro[indene-2,3'-indol]-1(3H)-one **[A8-resynthesized]**:

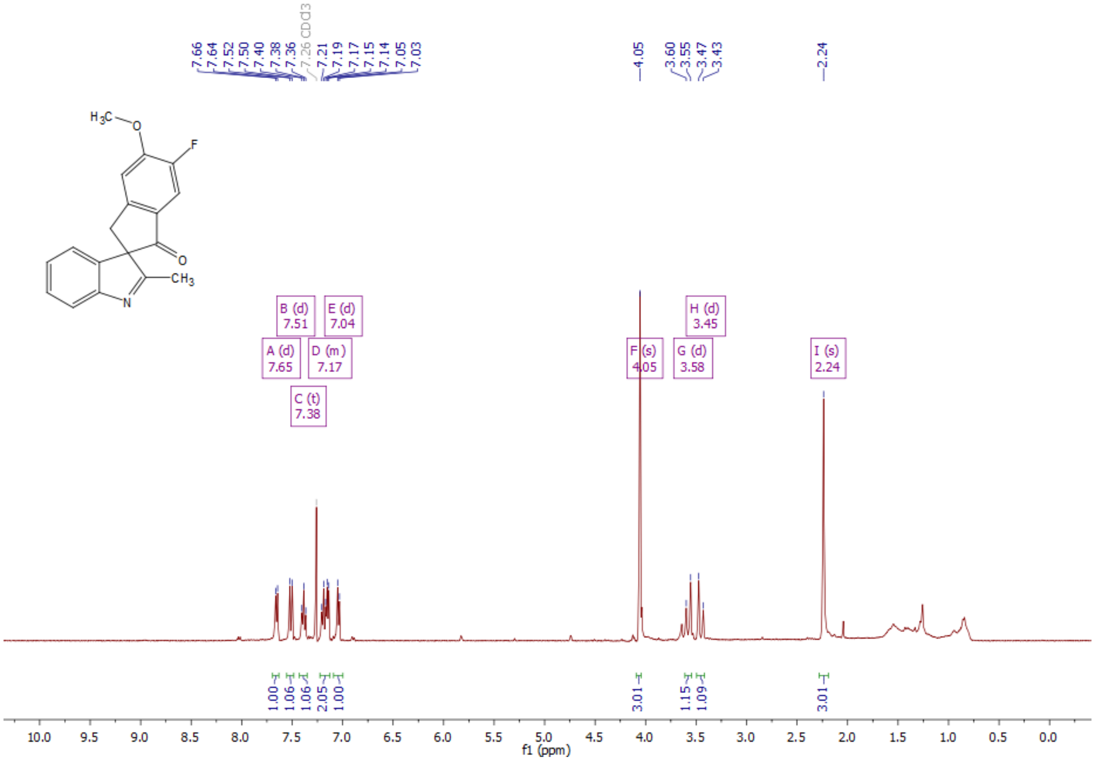

5,6-dimethoxy-2'-methylspiro[indene-2,3'-indol]-1(3H)-one [A9]:

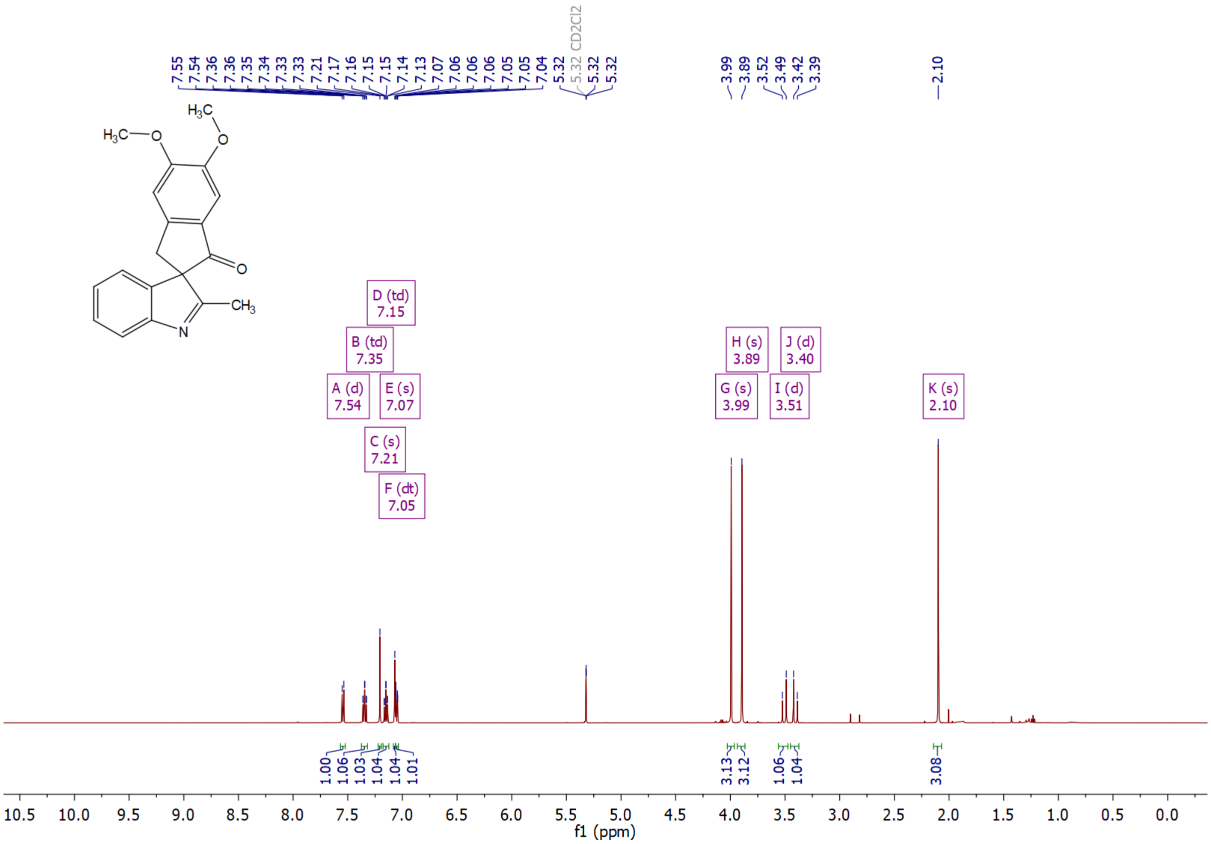

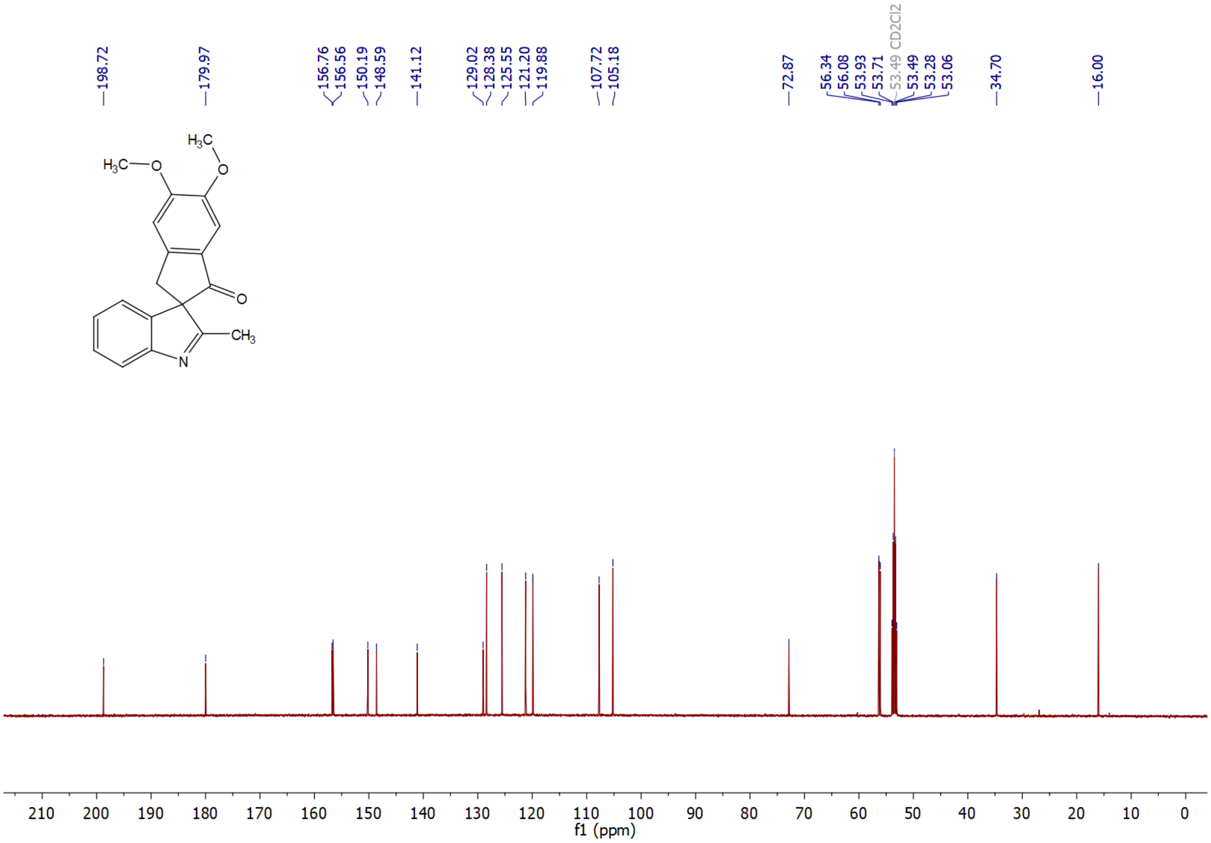

5,6,7-trimethoxy-2'-methylspiro[indene-2,3'-indol]-1(3H)-one [**A10**]:

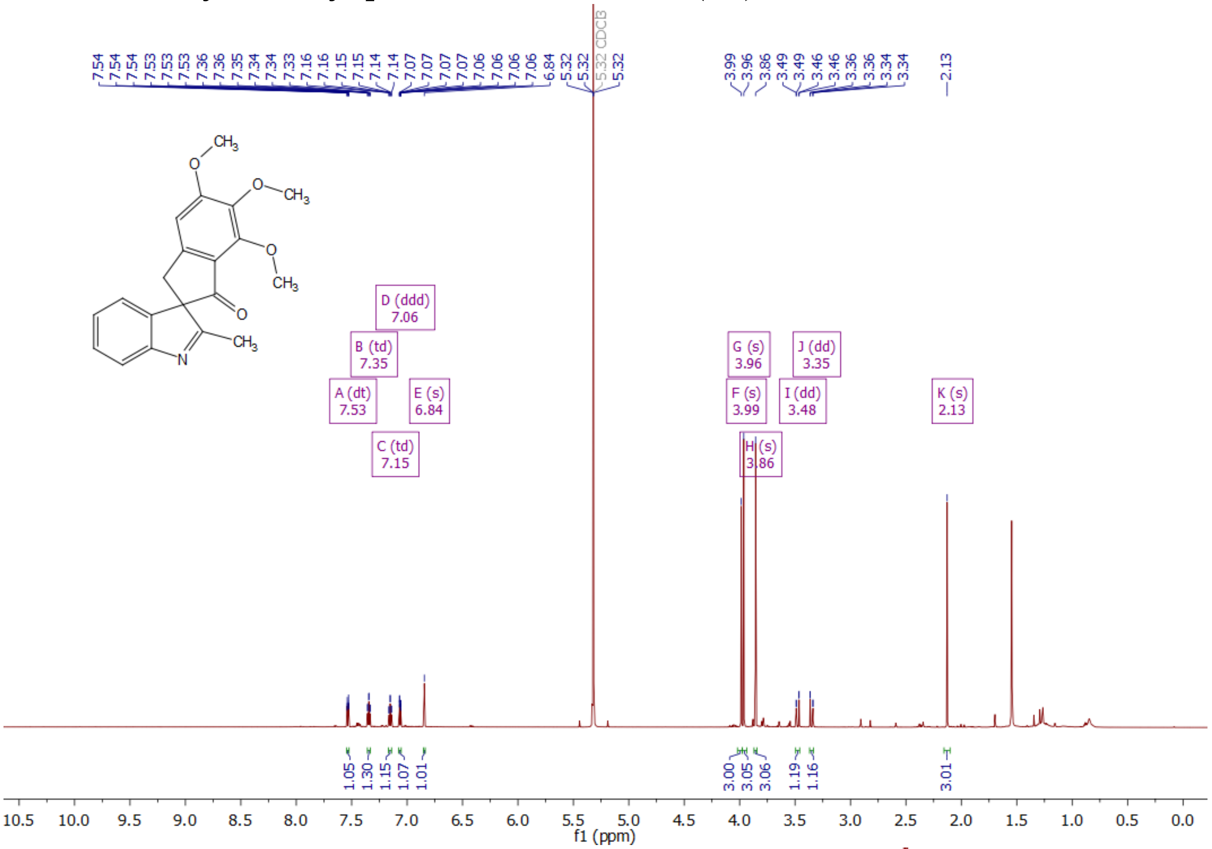

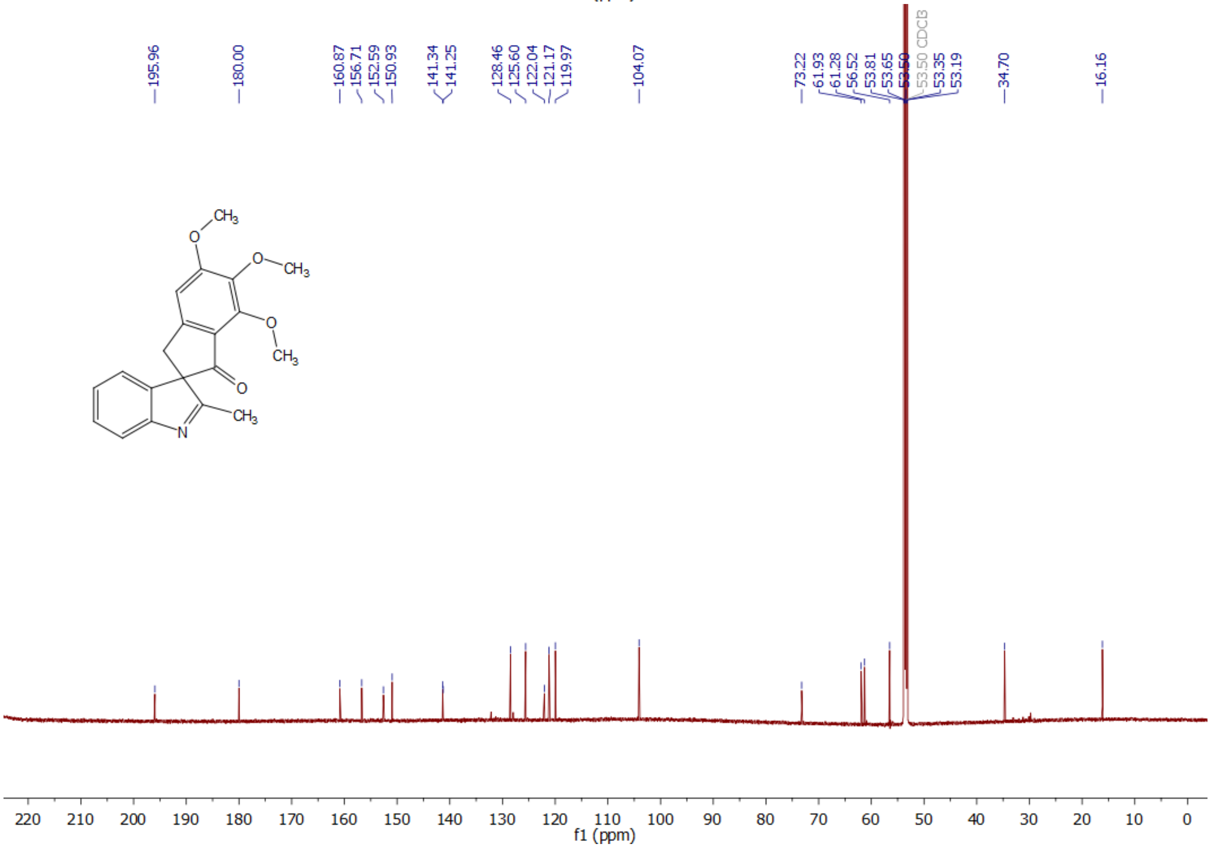

2'-methylspiro[indeno[5,6-d][1,3]dioxole-6,3'-indol]-5(7H)-one [**A11**]:

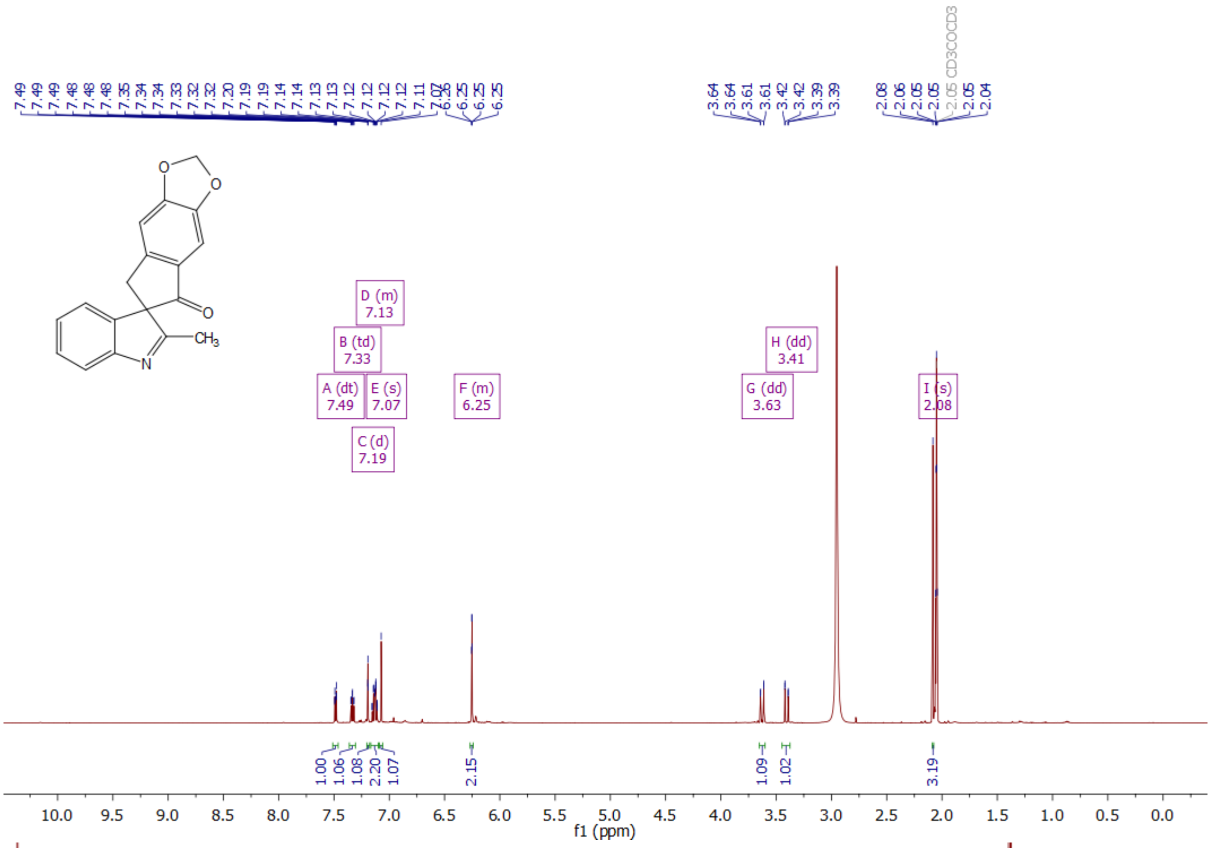

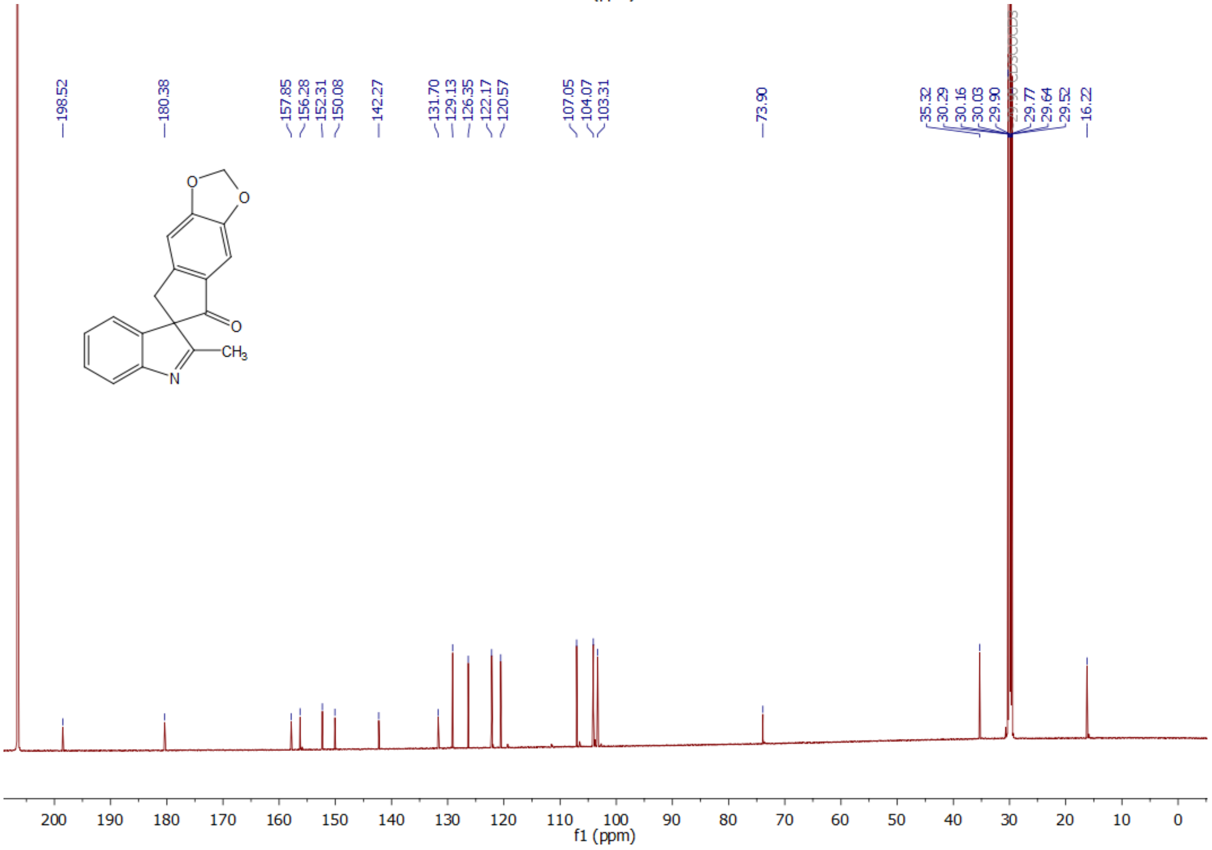

2'-methylspiro[indeno[4,5-d][1,3]dioxole-7,3'-indol]-6(8H)-one [**A12**]:

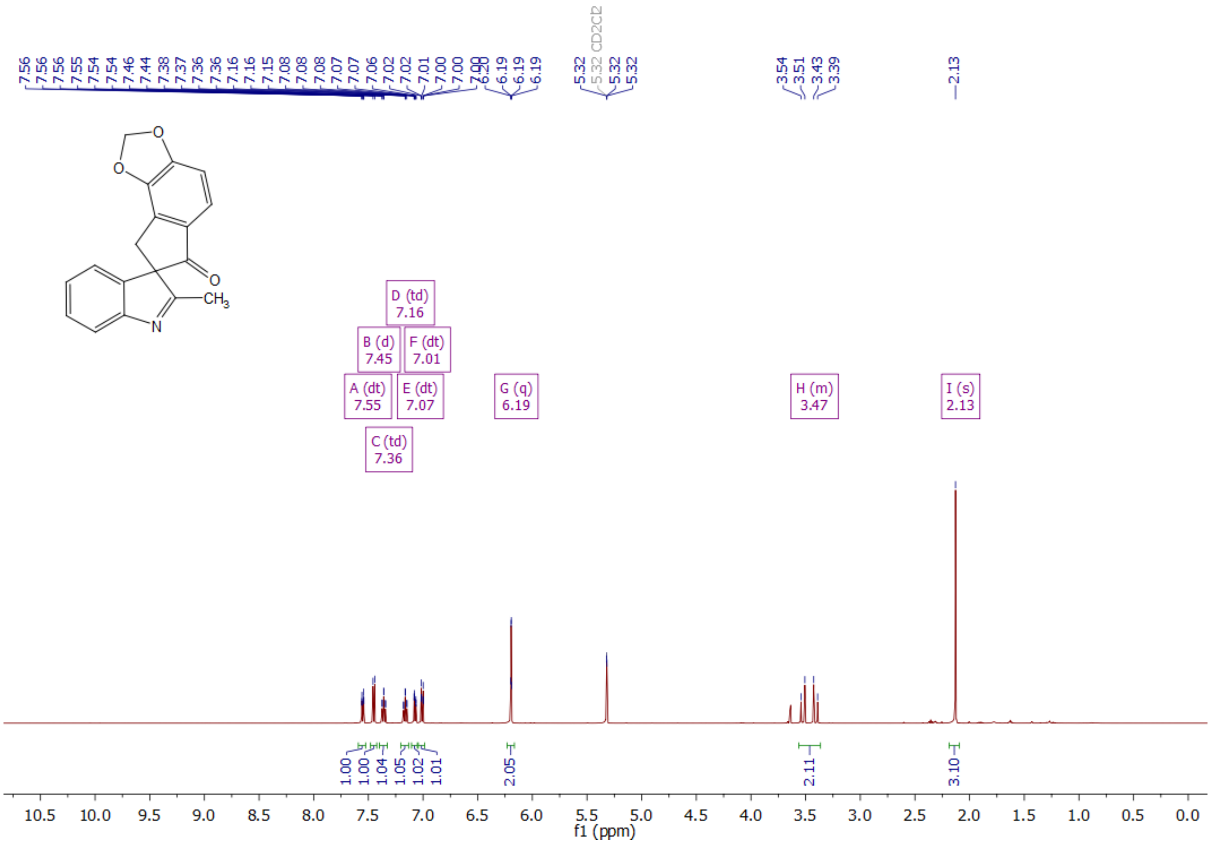

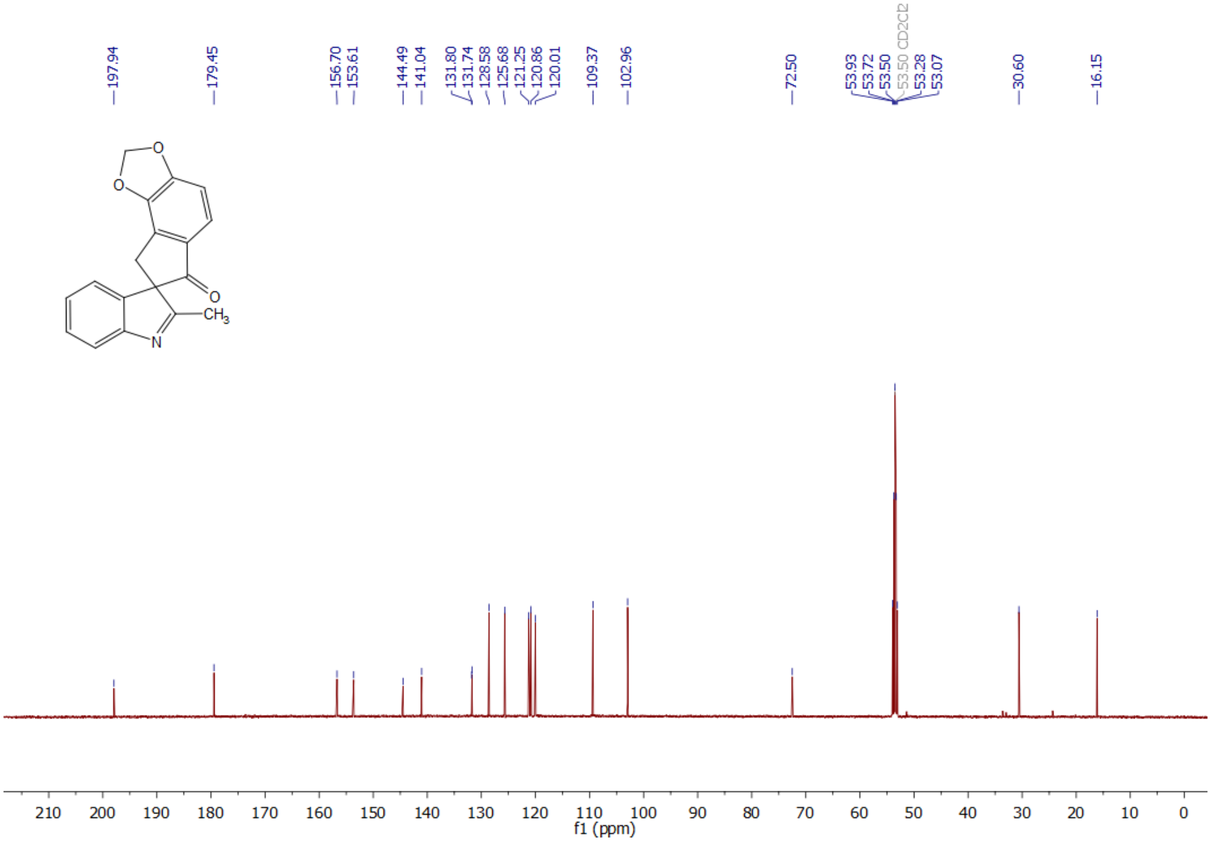

2'-methylspiro[cyclopenta[a]naphthalene-2,3'-indol]-1(3H)-one [**A13**]:

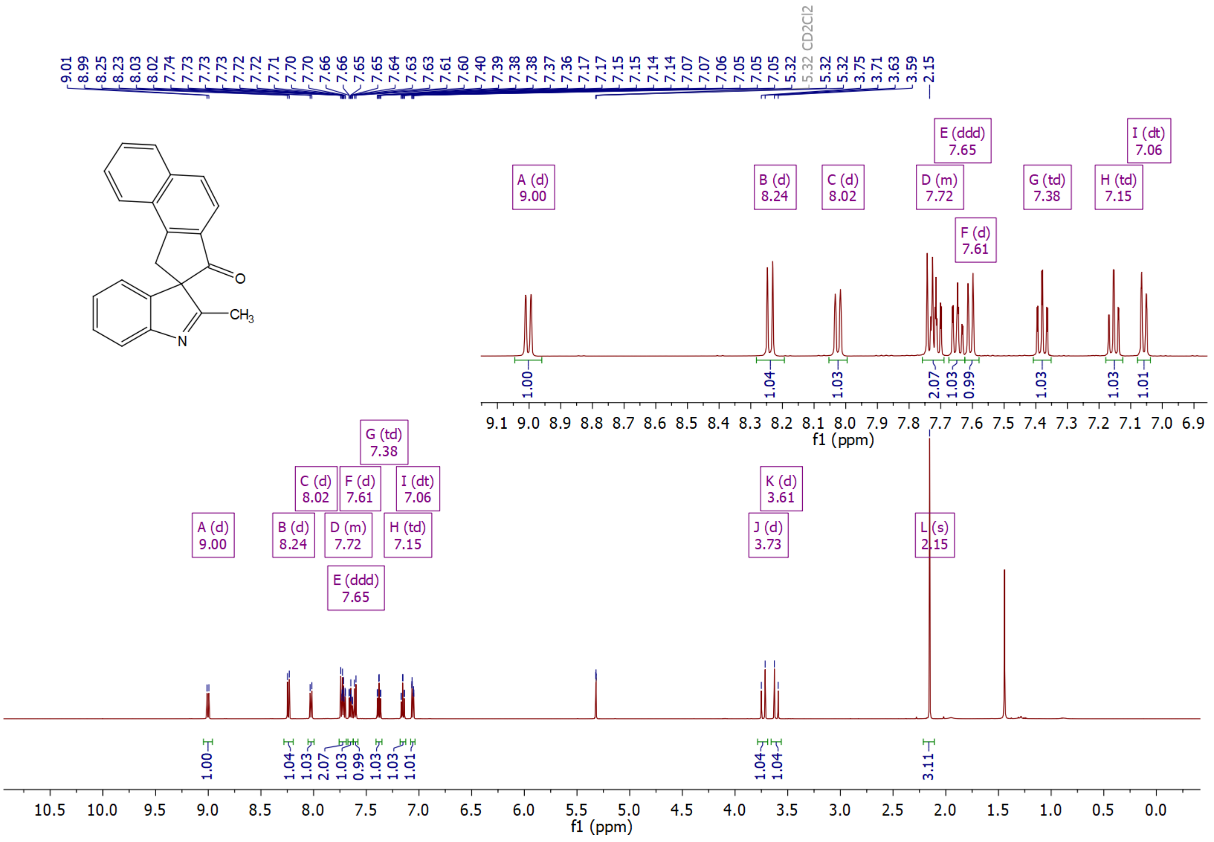

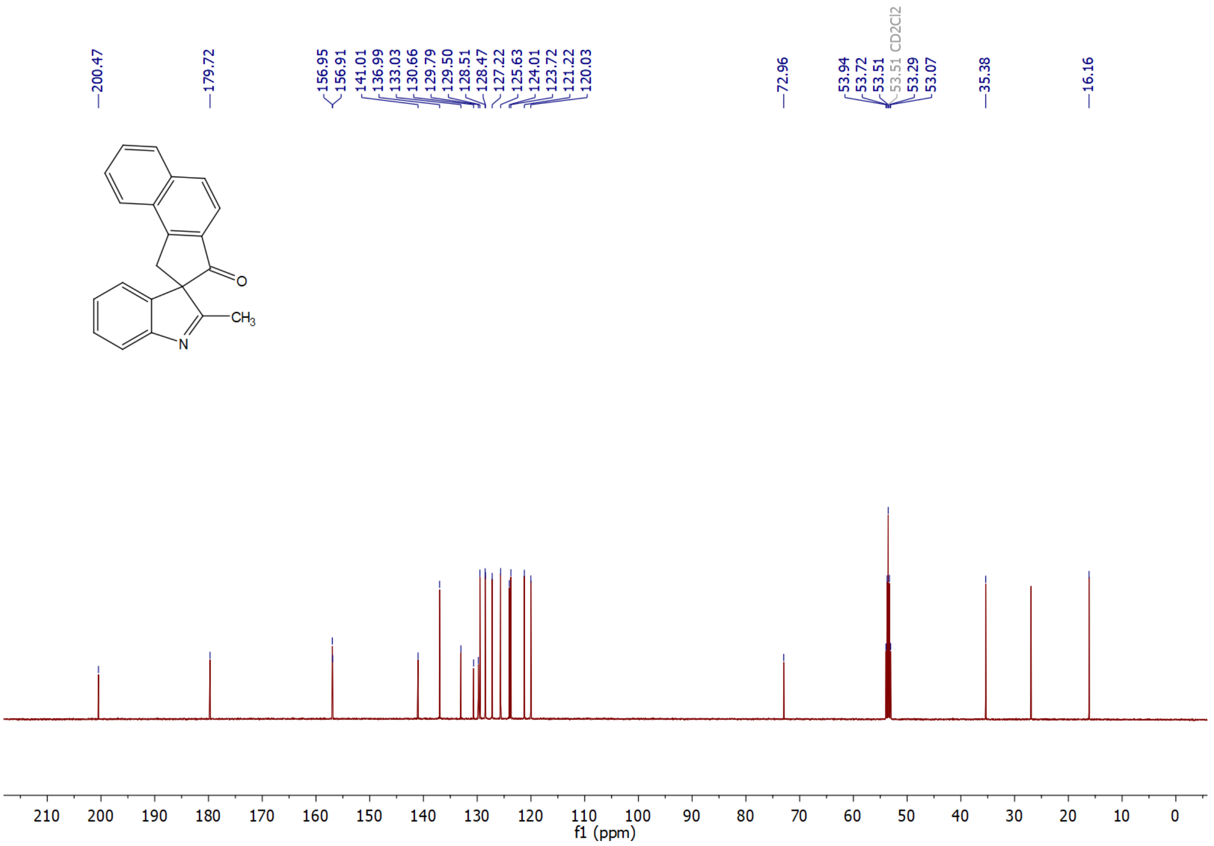

2',5'-dimethylspiro[indene-2,3'-indol]-1(3H)-one [**A14**]:

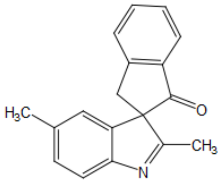

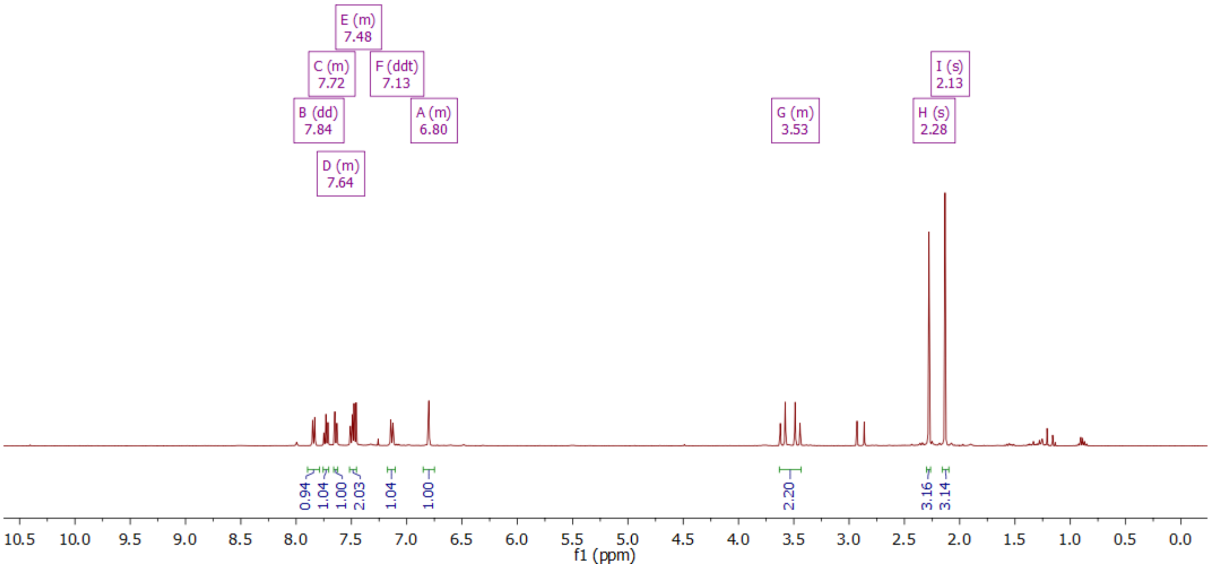

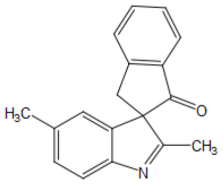

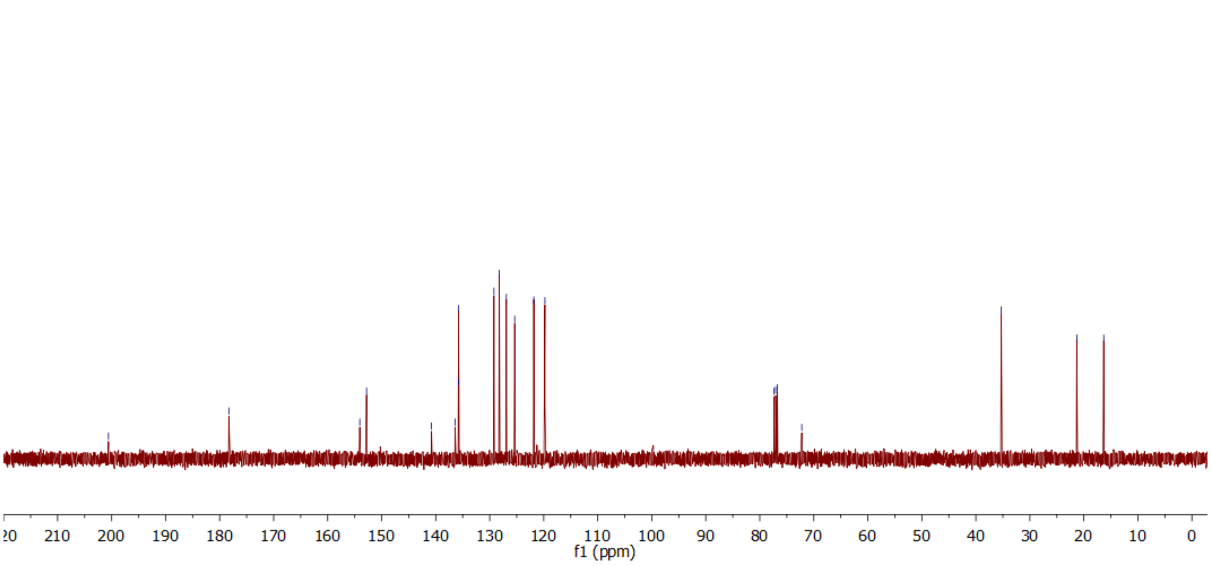

5'-fluoro-2'-methylspiro[indene-2,3'-indol]-1(3H)-one [**A15**]:

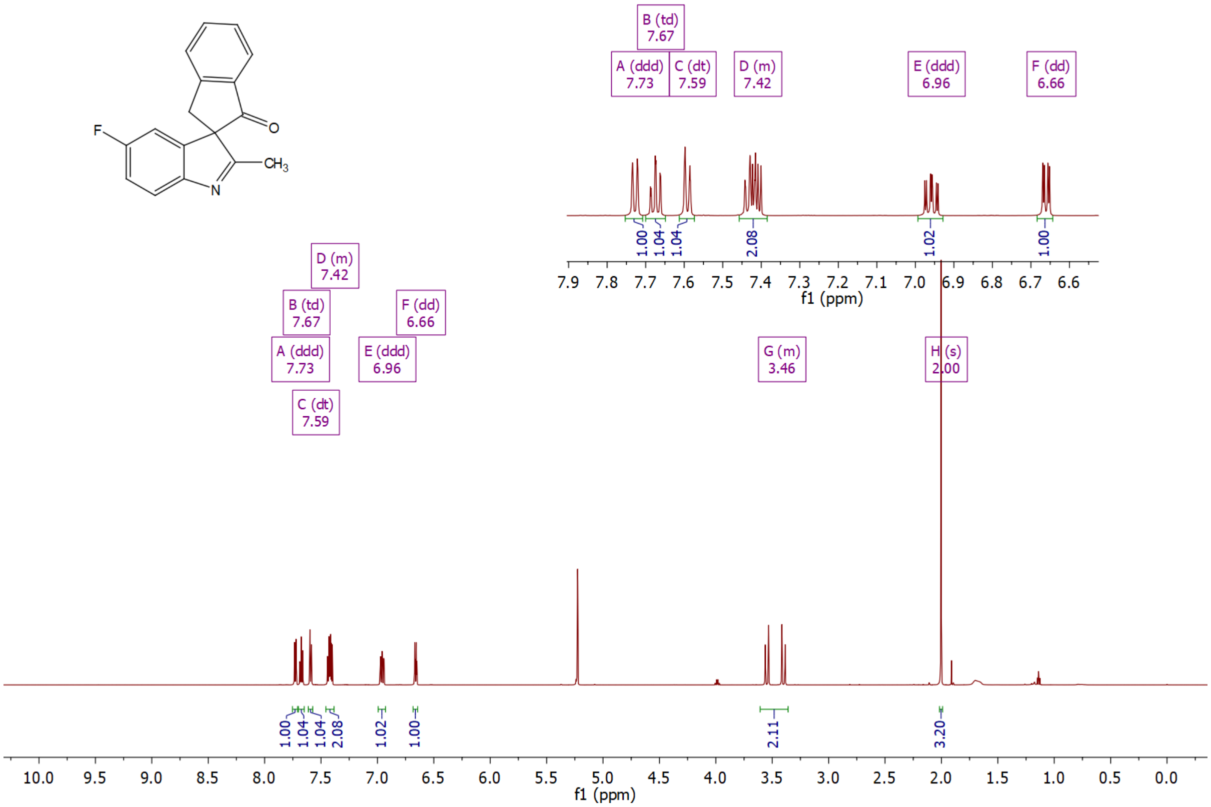

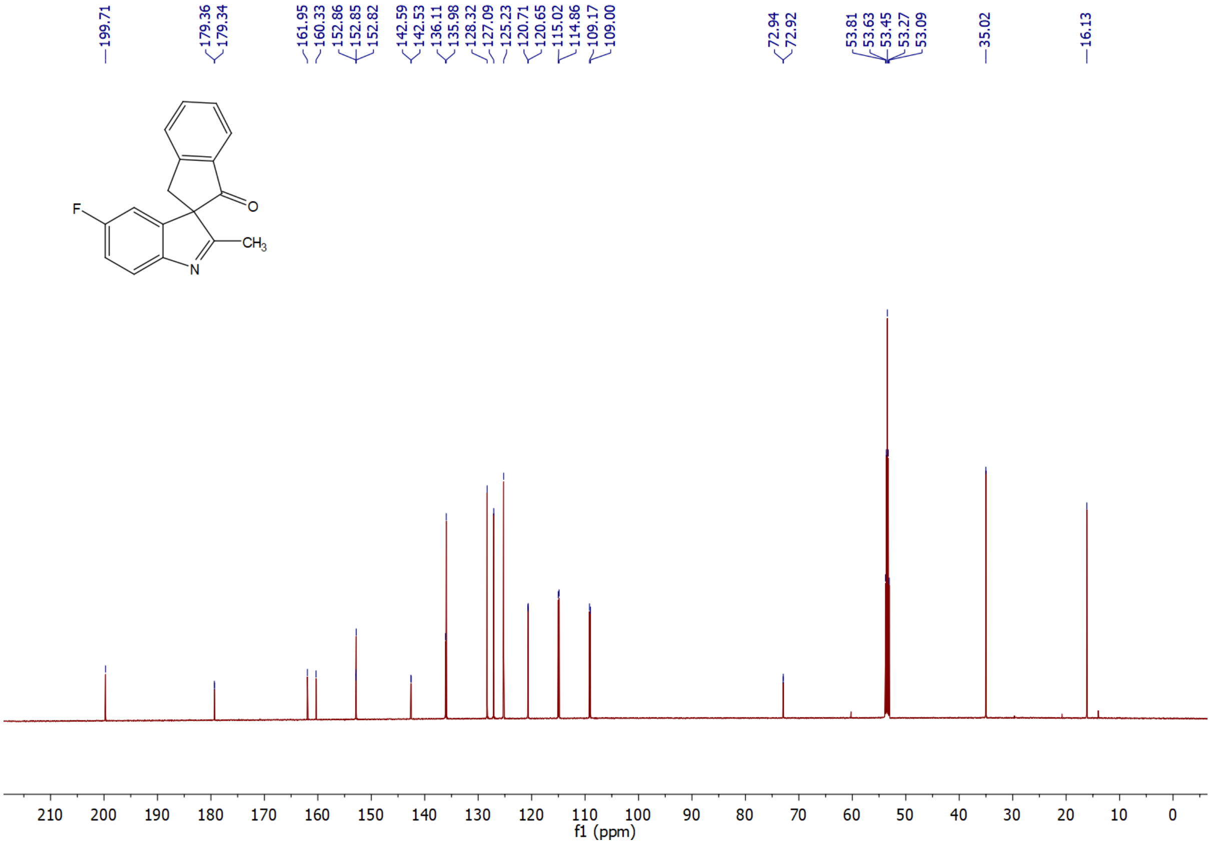

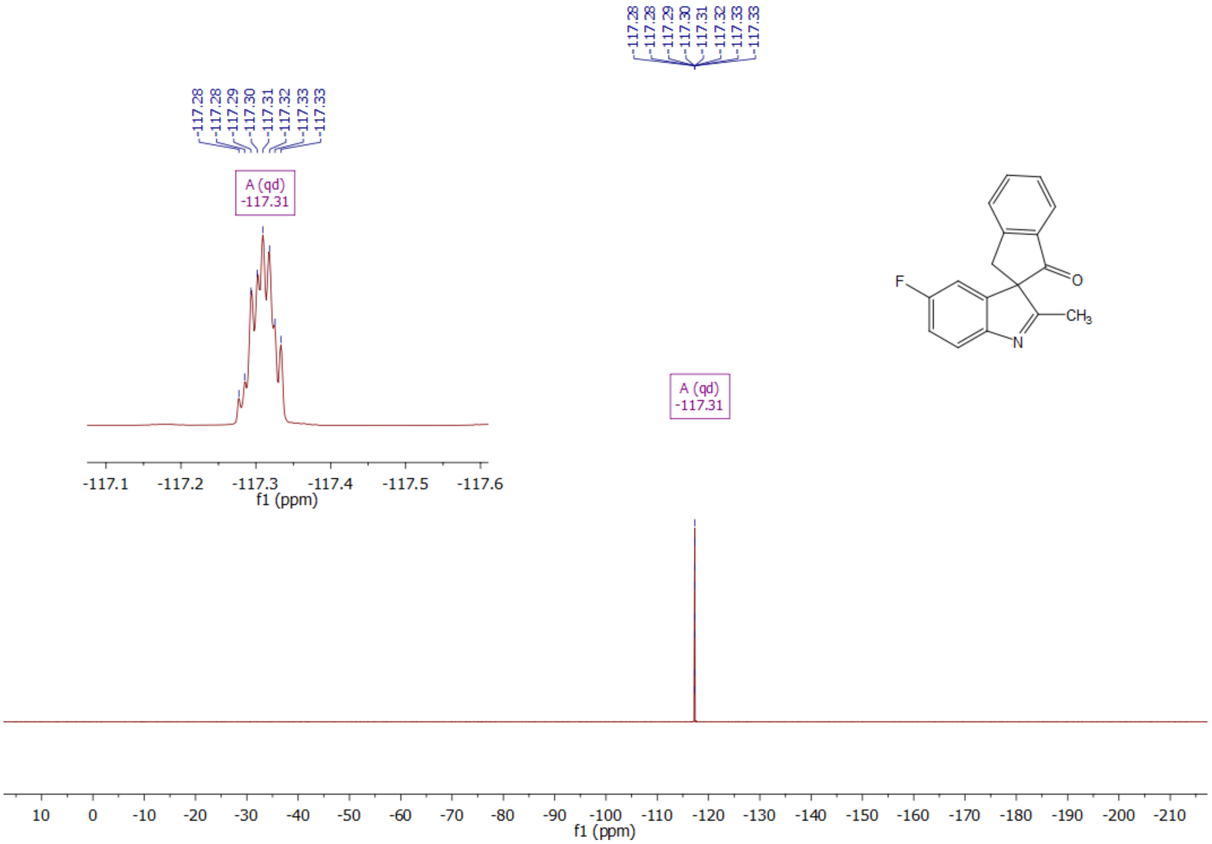
-117.28
-117.28
-117.29
-117.30
-117.31
-117.32
-117.33
-117.33
A (qd)
-117.31
-117.1
-117.2
-117.3
-117.4
-117.5
-117.6
f1 (ppm)
F
O
CH3
N
10
0
-10
-20
-30
-40
-50
-60
-70
-80
-90
-100
-110
-120
-130
-140
-150
-160
-170
-180
-190
-200
-210

5'-chloro-2'-methylspiro[indene-2,3'-indol]-1(3H)-one [**A16**]:

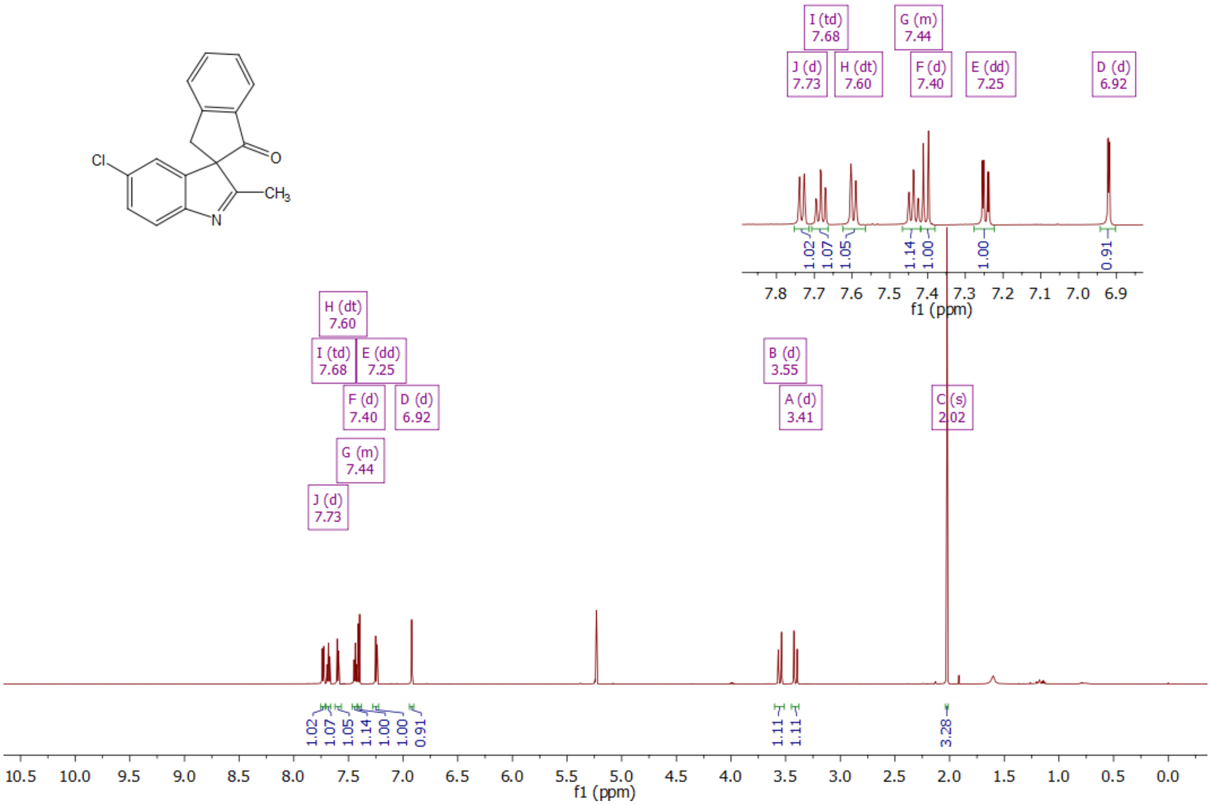

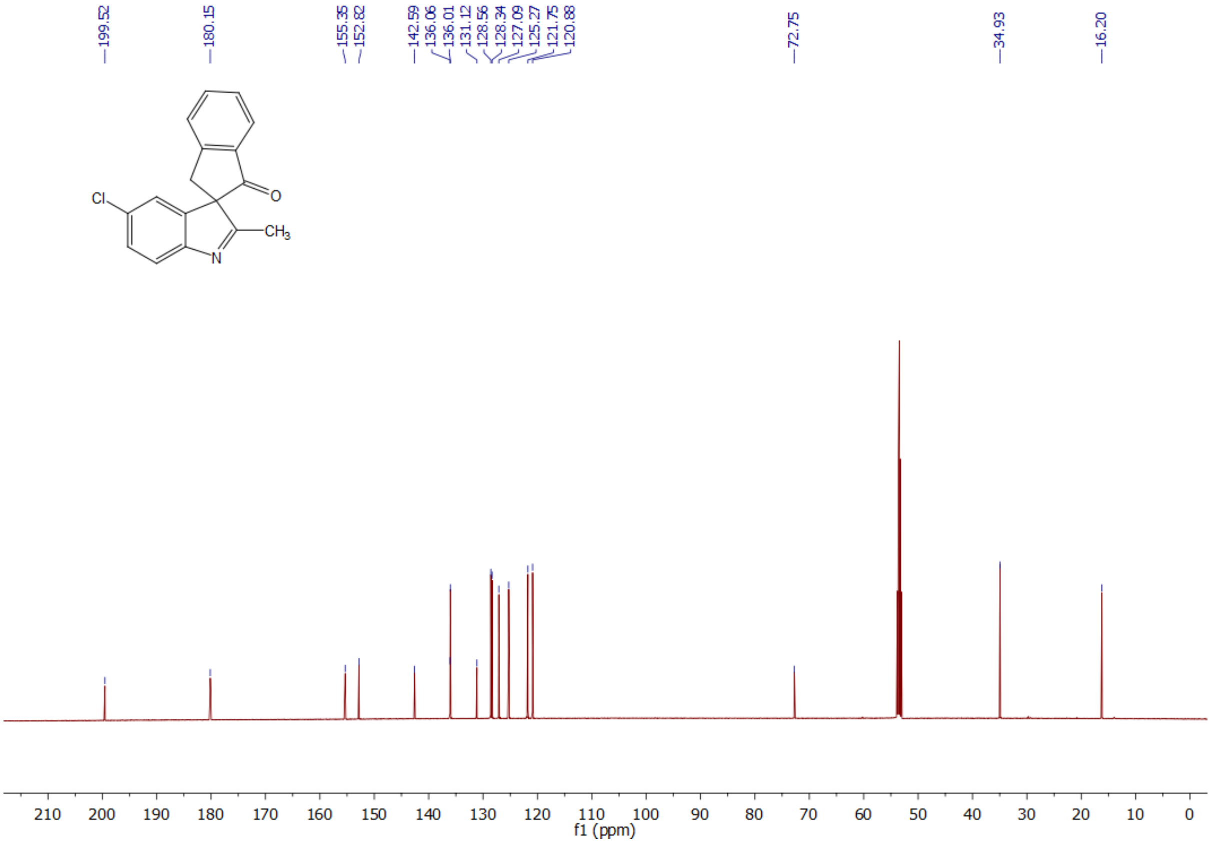

5'-chloro-2'-methylspiro[indene-2,3'-indol]-1(3H)-one [**A16-resynthesized**]:

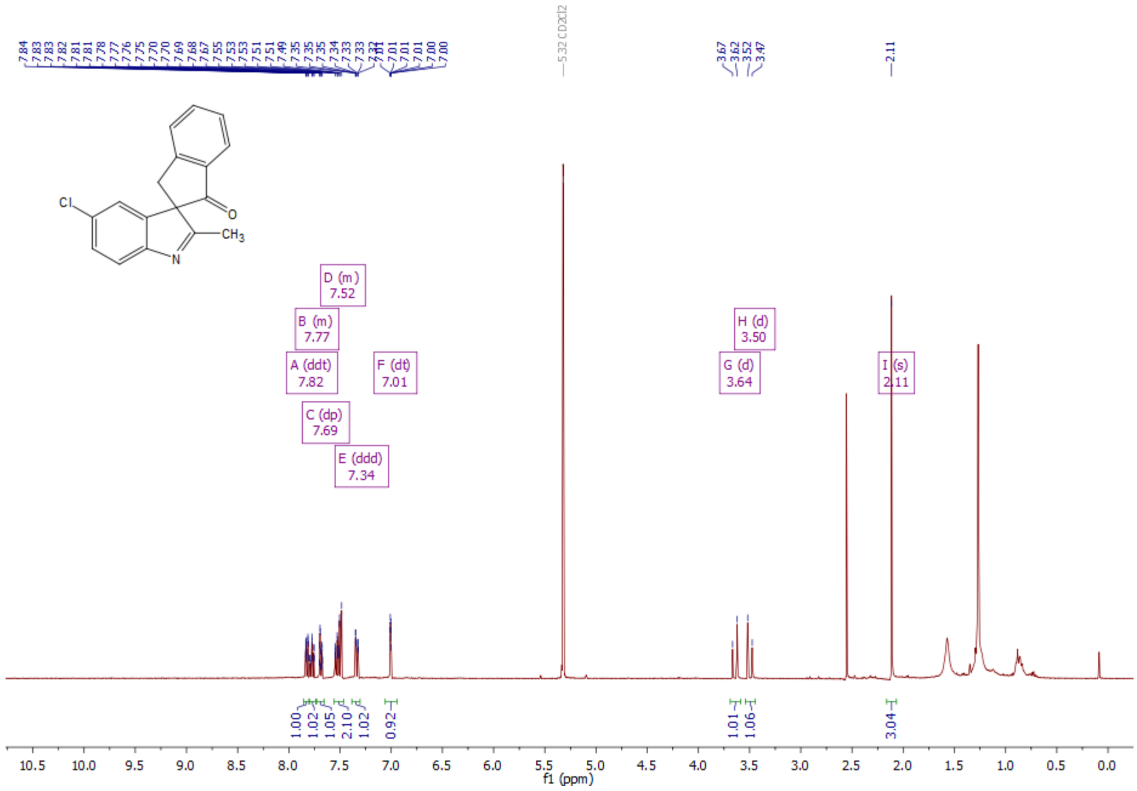

6-fluoro-2',5'-dimethylspiro[indene-2,3'-indol]-1(3H)-one [**A17**]:

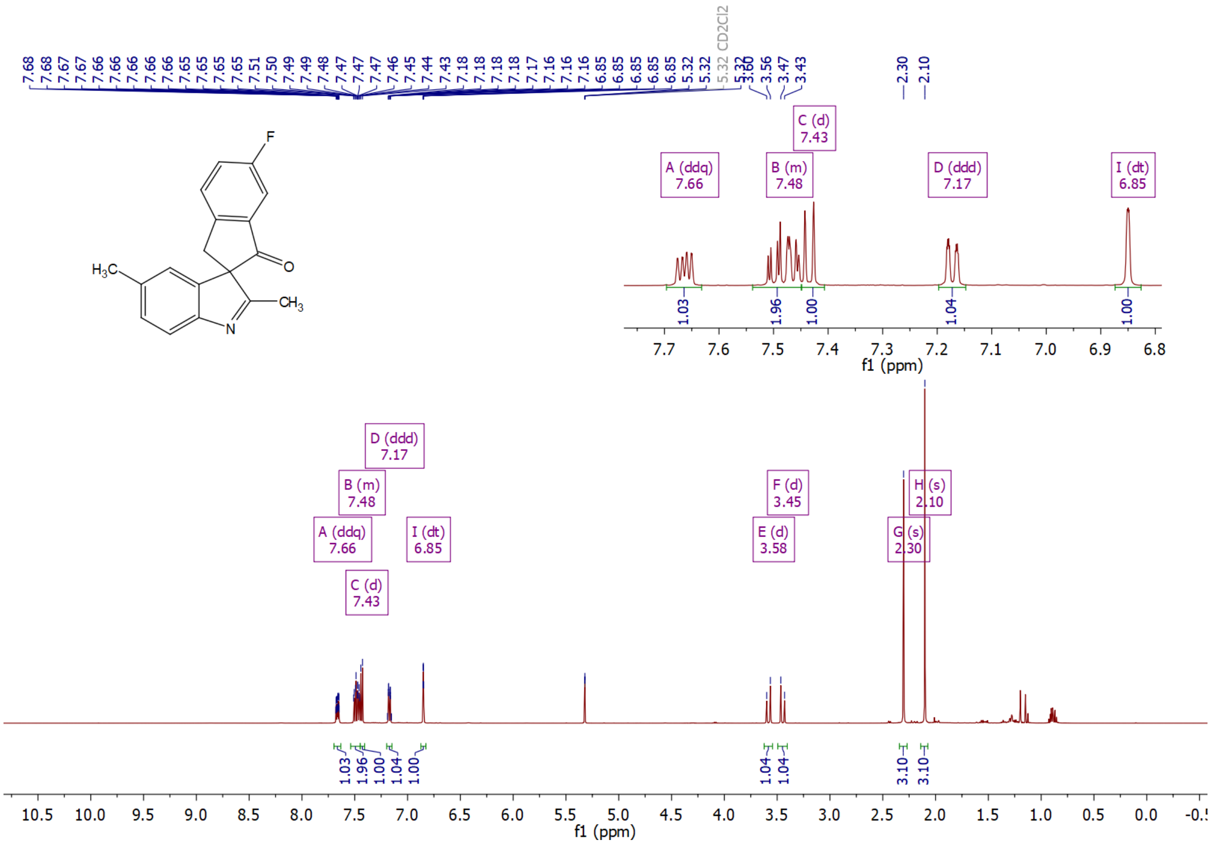

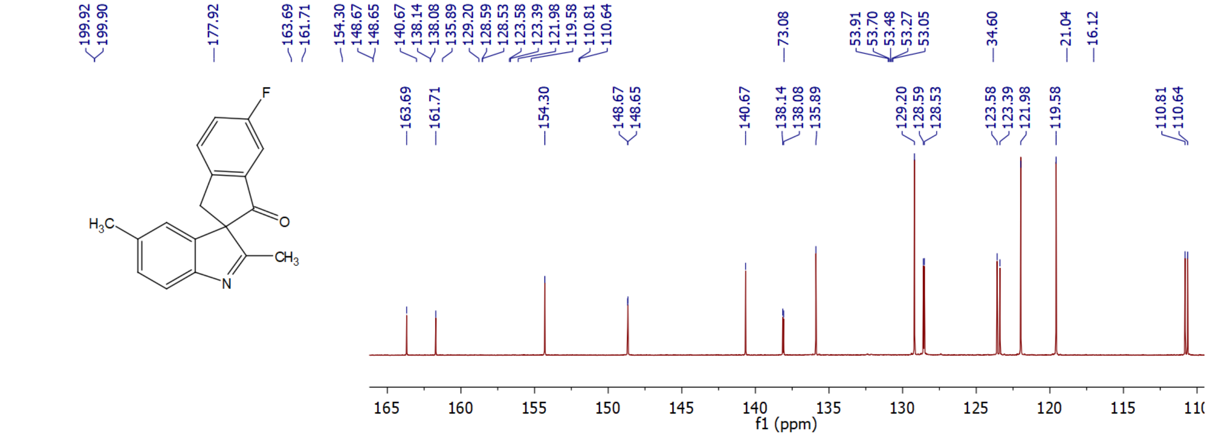

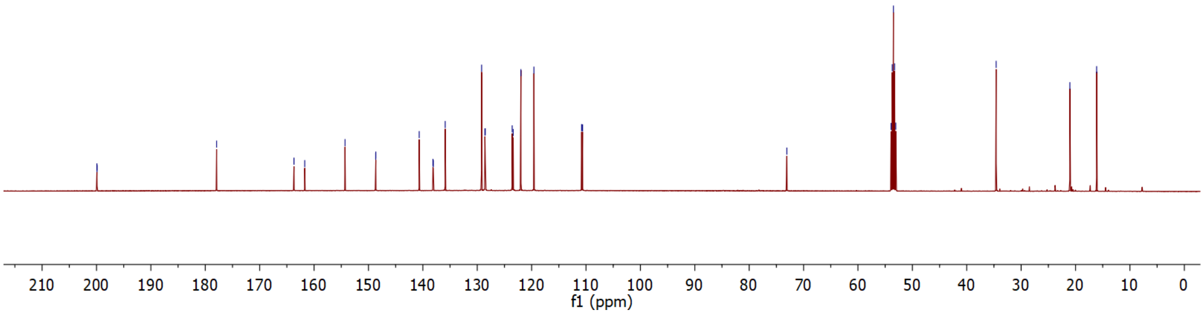

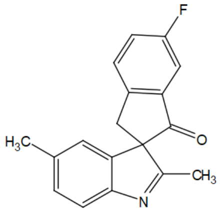

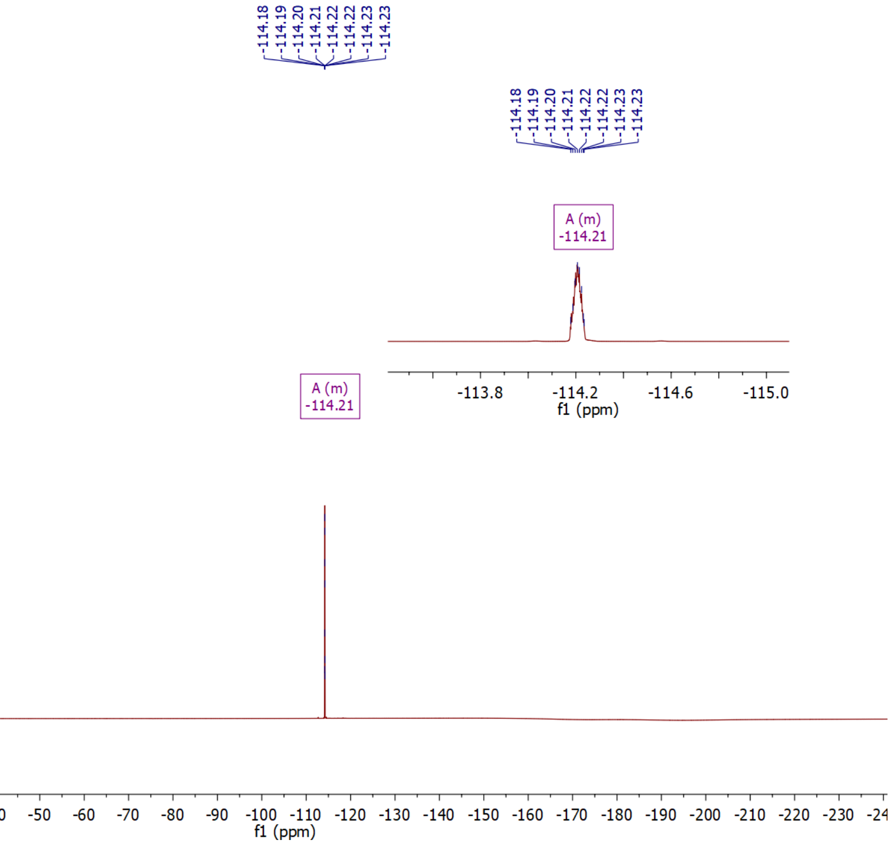
-114.18
-114.19
-114.20
-114.21
-114.22
-114.22
-114.22
-114.23
-114.23
A (m)
-114.21
-113.8
-114.2
-114.6
-115.0
f1 (ppm)
0
20
10
0
-10
-20
-30
-40
-50
-60
-70
-80
-90
-100
-110
-120
-130
-140
-150
-160
-170
-180
-190
-200
-210
-220
-230
-24

6-chloro-2',5'-dimethylspiro[indene-2,3'-indol]-1(3H)-one [**A18**]:

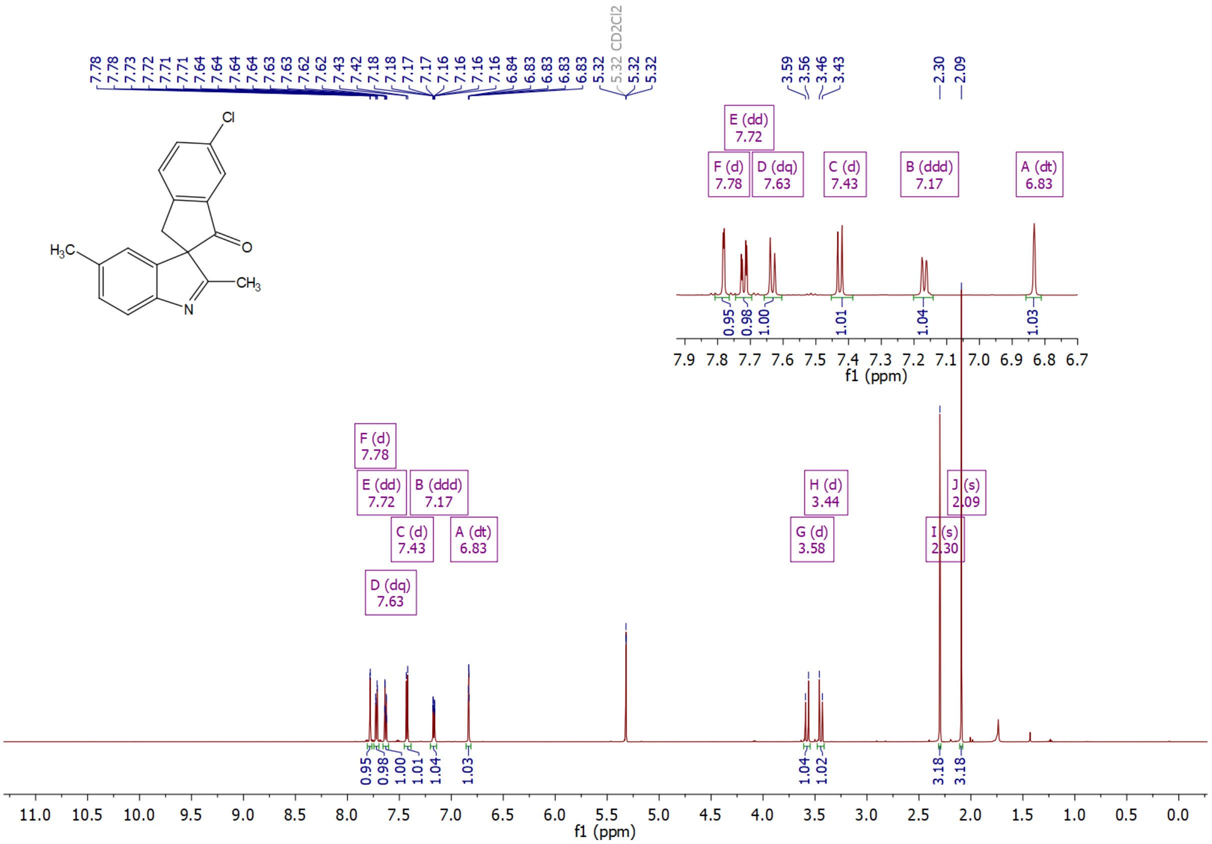

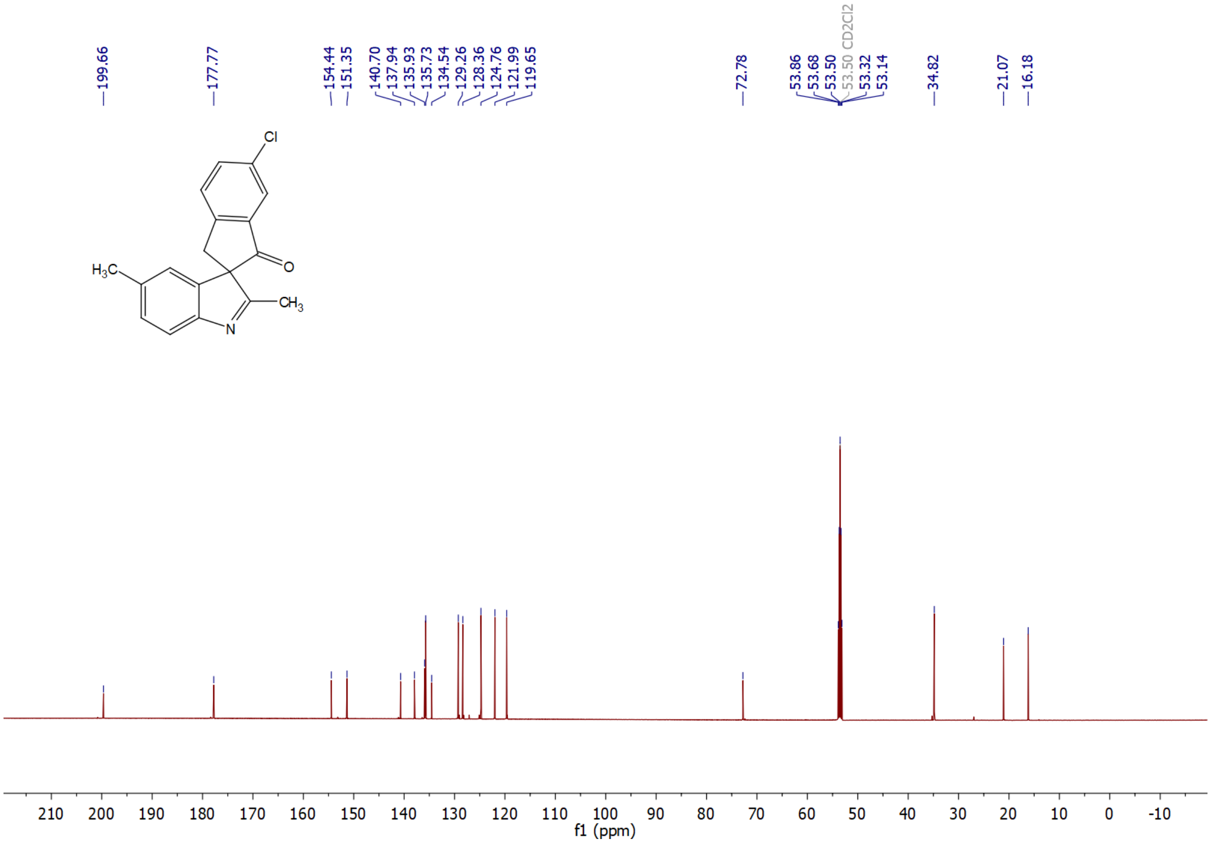

6-chloro-5'-fluoro-2'-methylspiro[indene-2,3'-indol]-1(3H)-one [**A19**]:

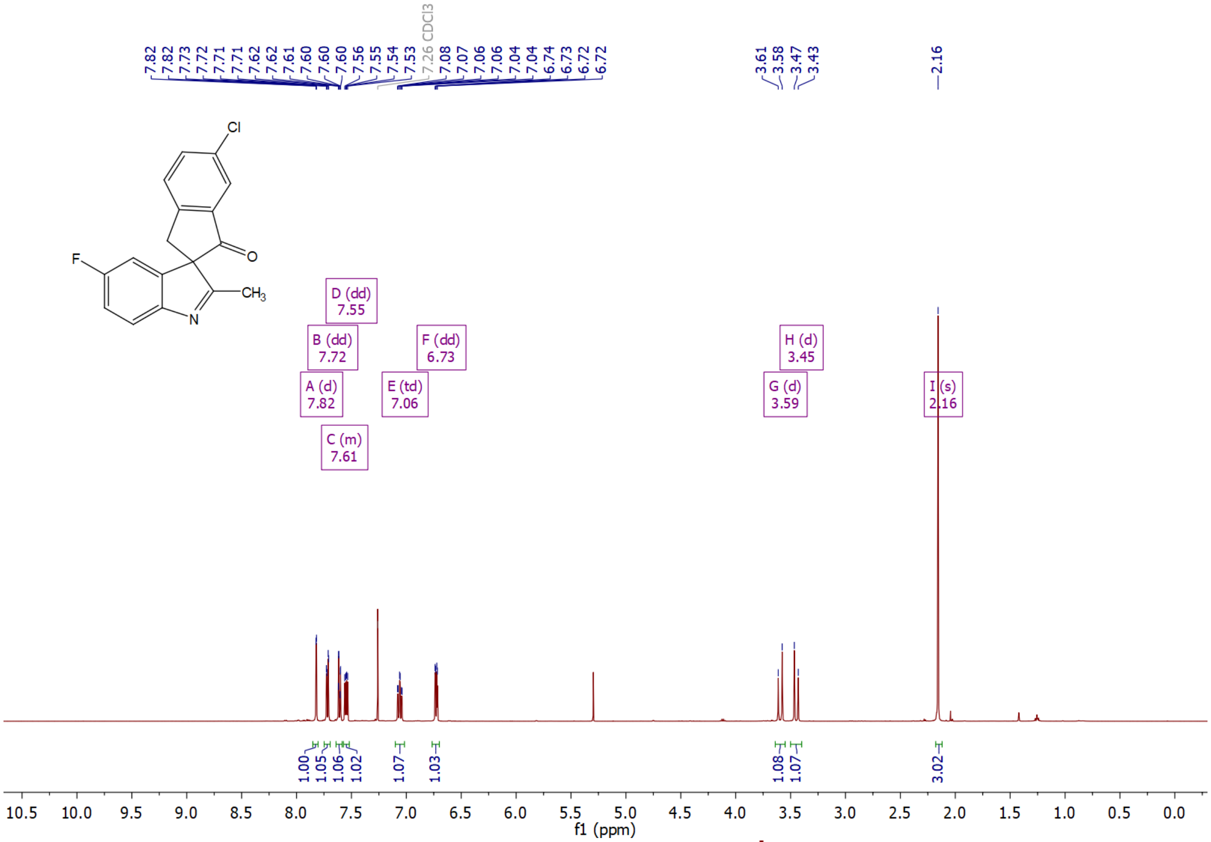

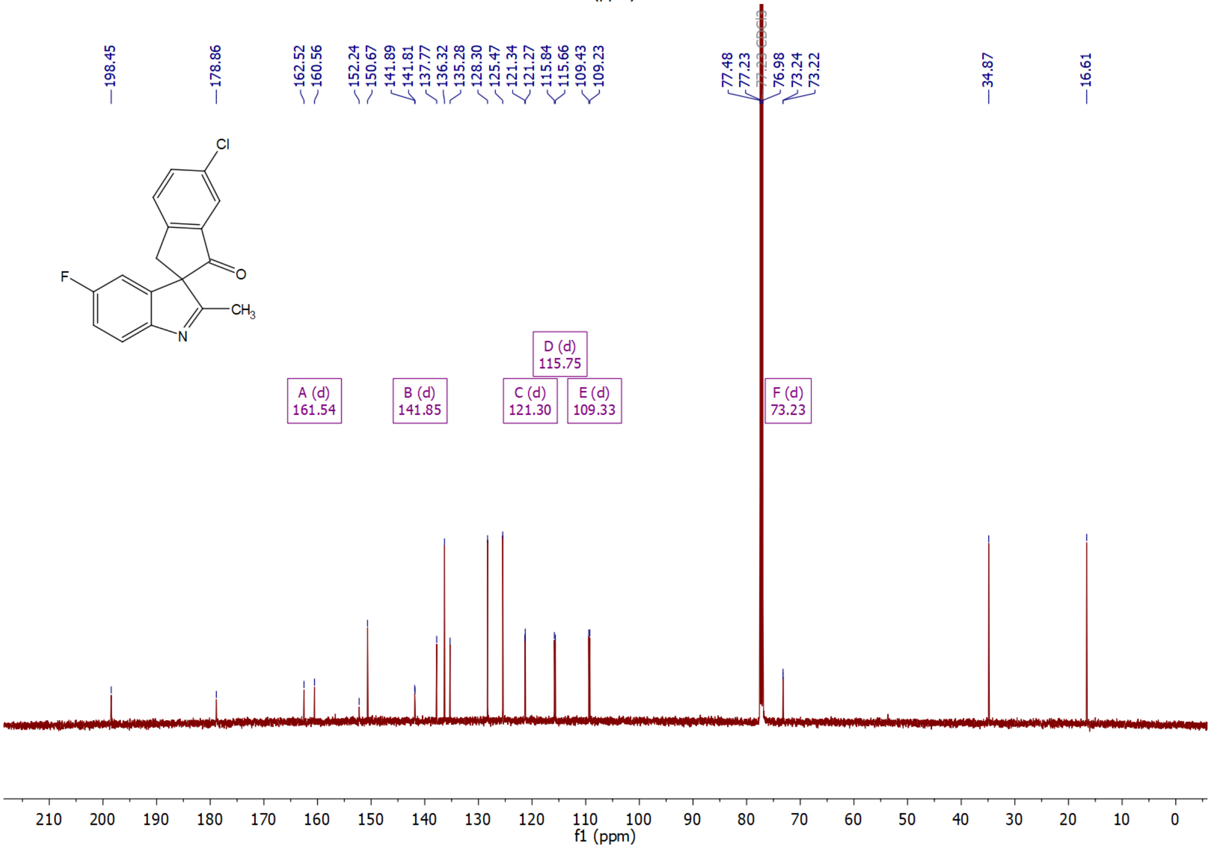

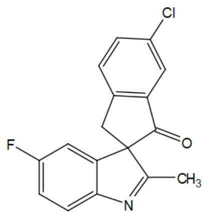
Cl
O
F
CH3
N

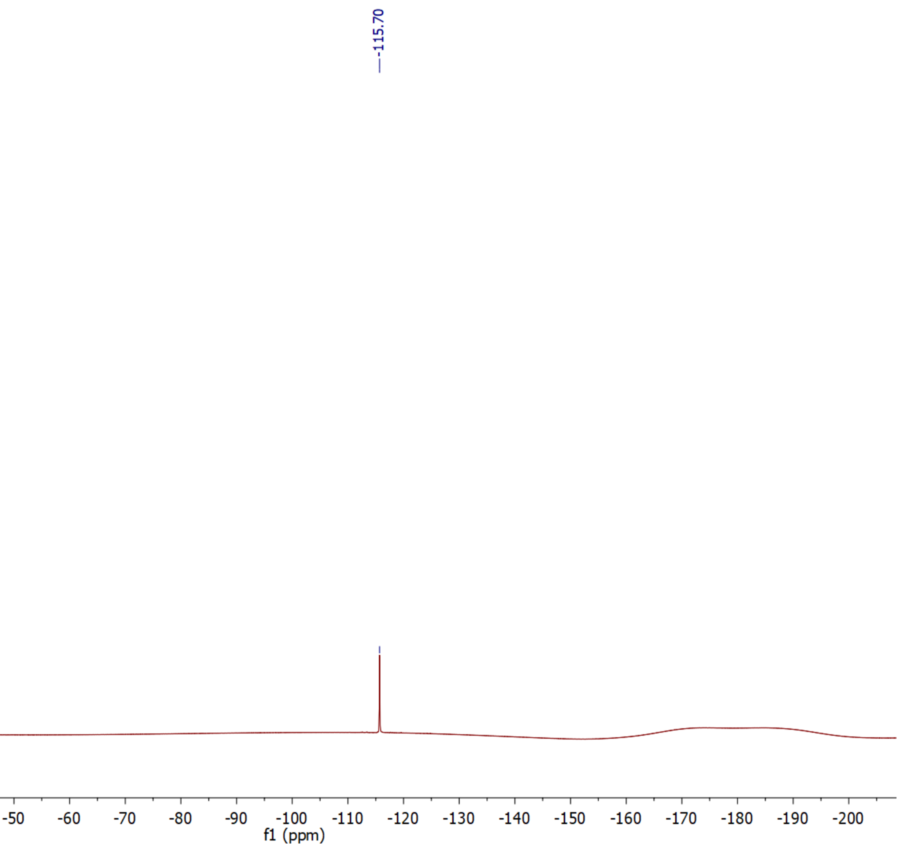
-115.70
0
-10
-20
-30
-40
-50
-60
-70
-80
-90
-100
-110
-120
-130
-140
-150
-160
-170
-180
-190
-200
f1 (ppm)

5'-chloro-2',6-dimethylspiro[indene-2,3'-indol]-1(3H)-one [**A20**]:

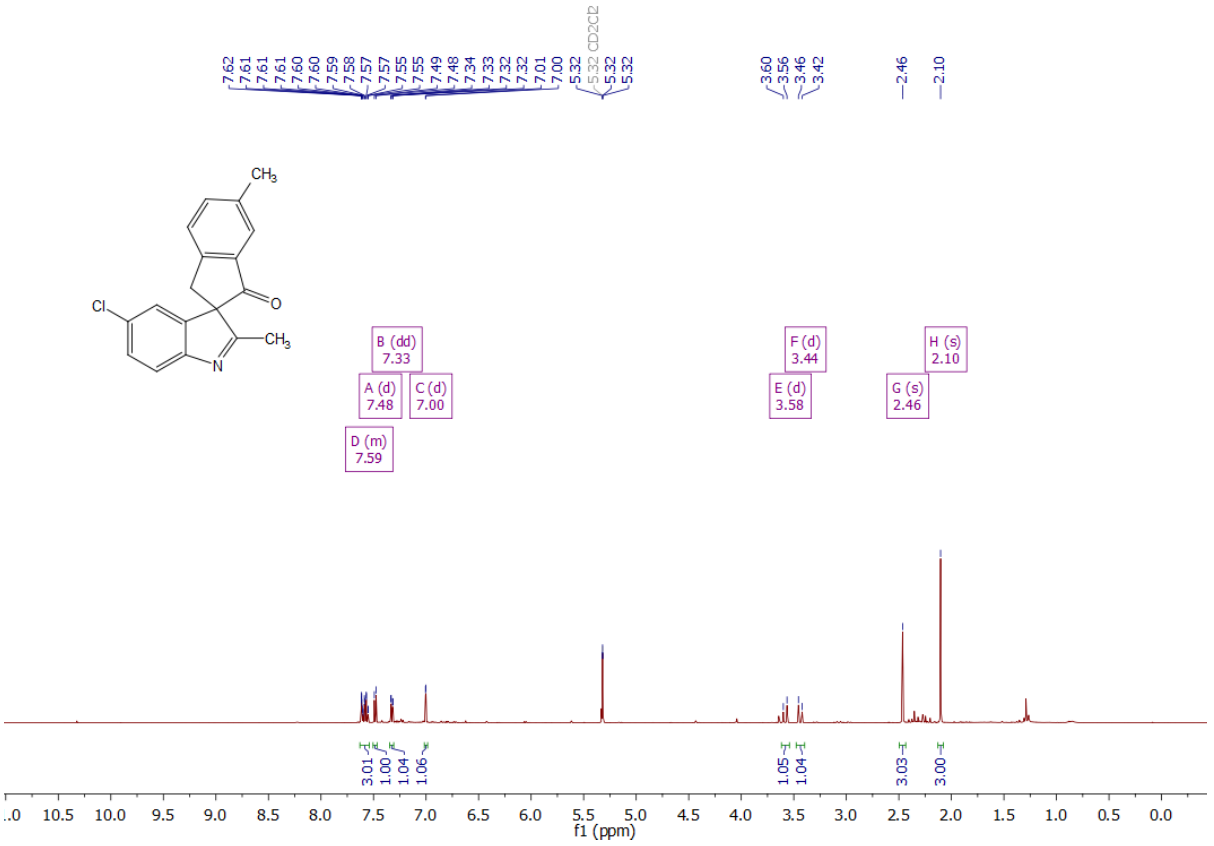

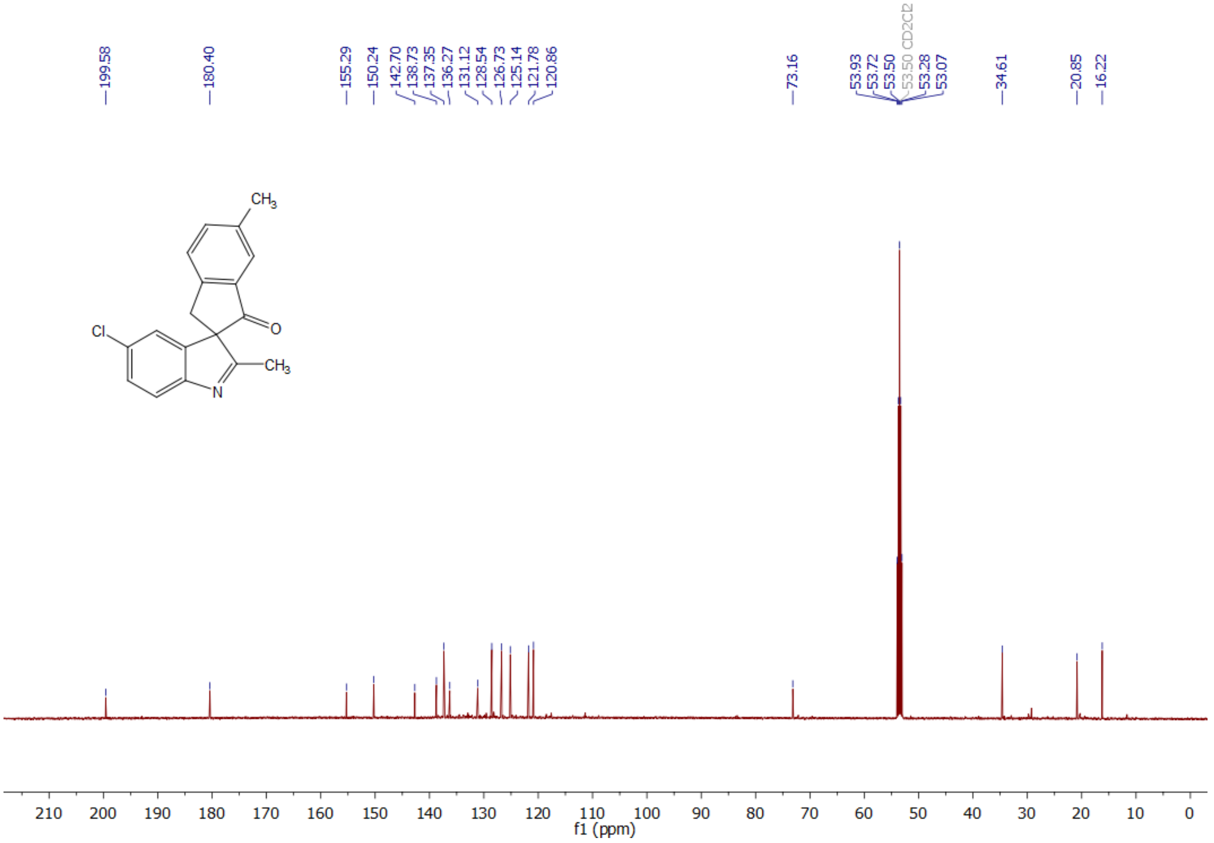

5'-fluoro-6-methoxy-2'-methylspiro[indene-2,3'-indol]-1(3H)-one [A21]:

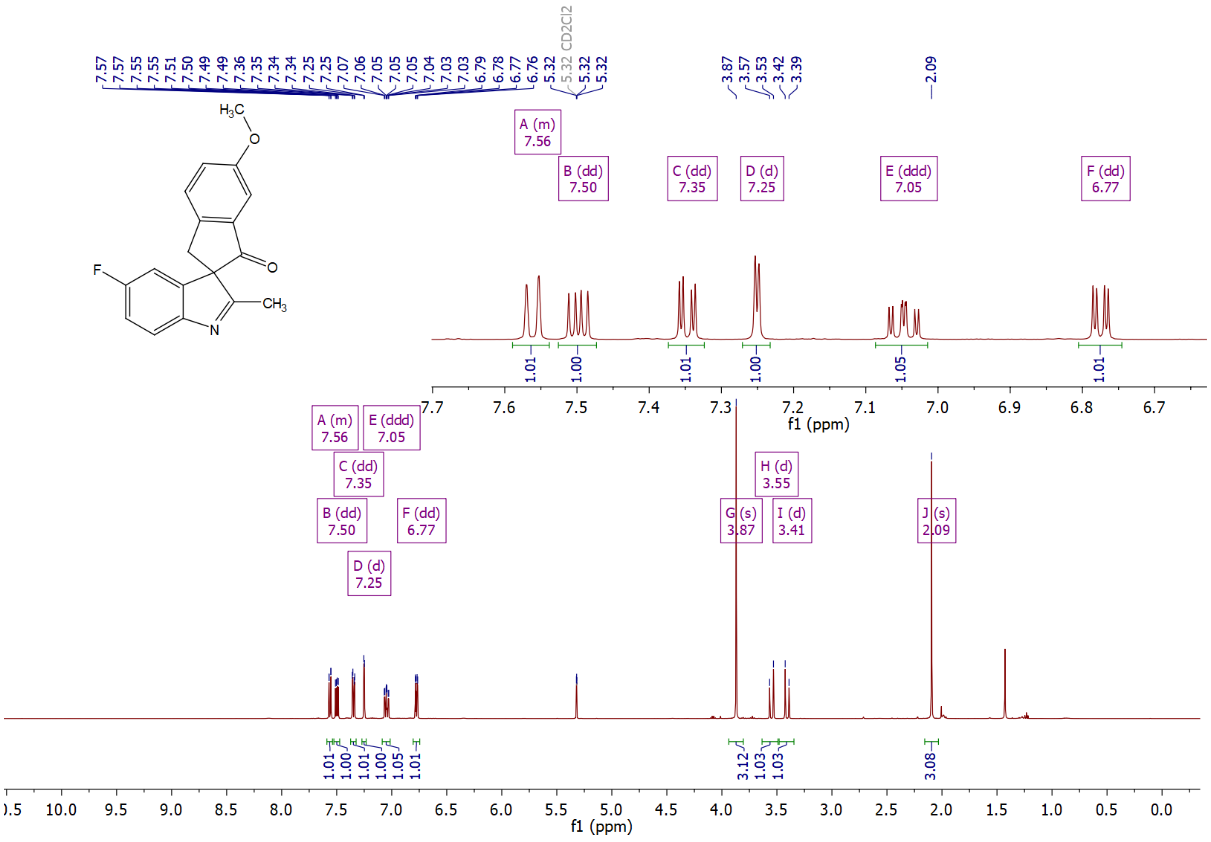

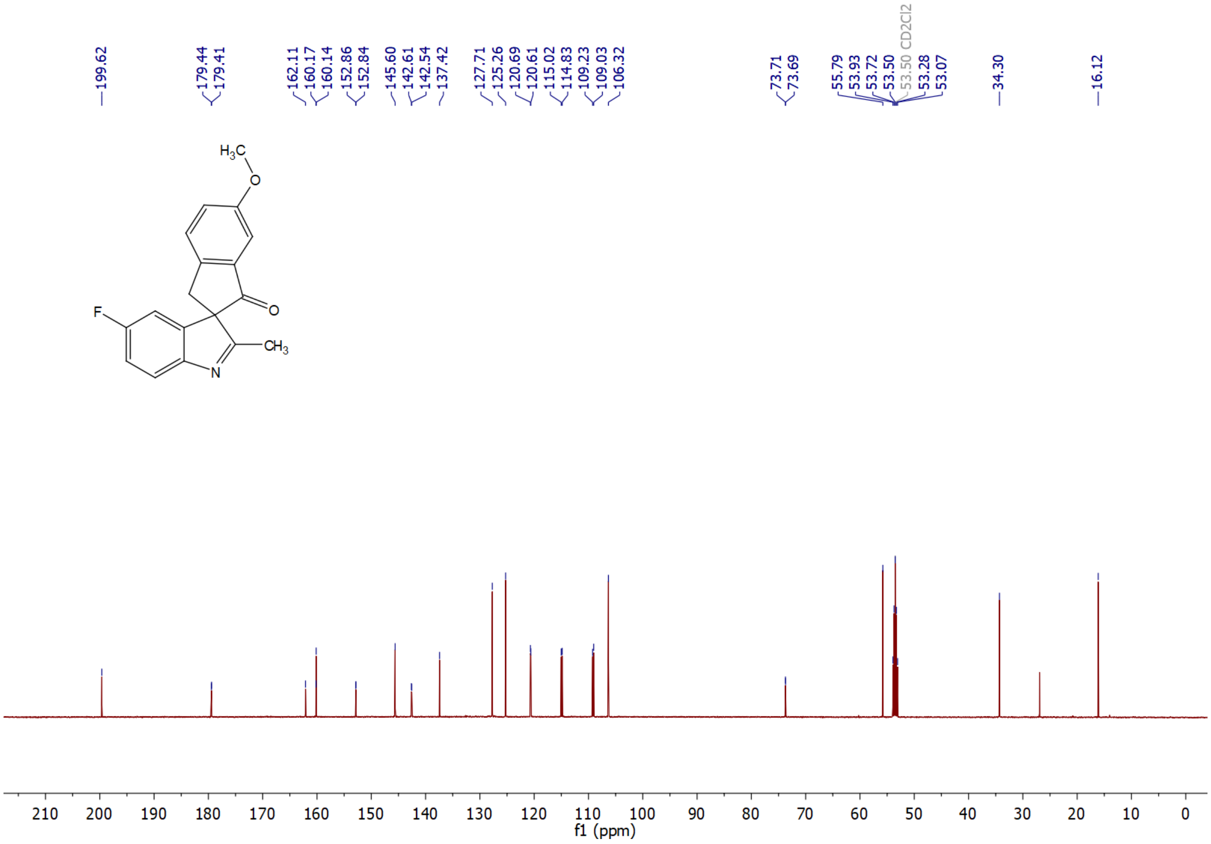

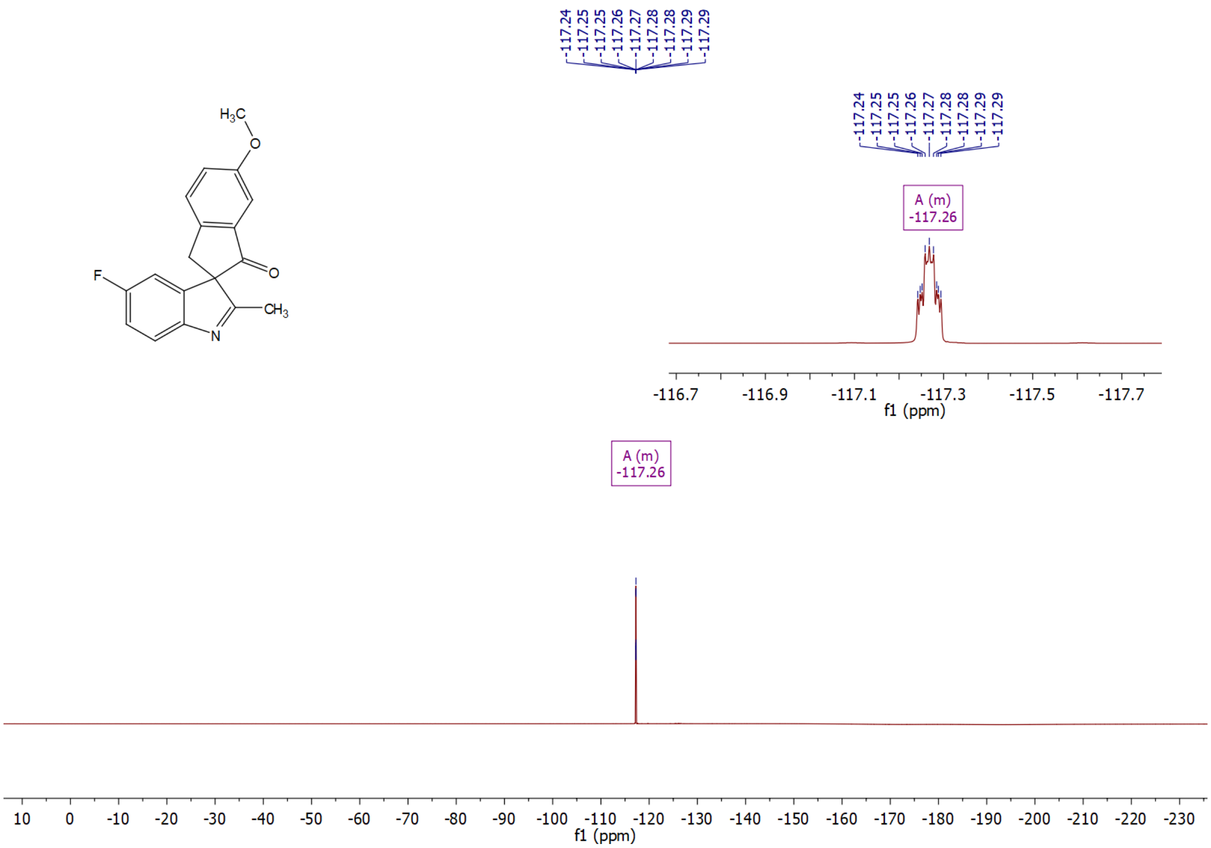

5'-fluoro-6-methoxy-2'-methylspiro[indene-2,3'-indol]-1(3H)-one **[A21-resynthesized]**:

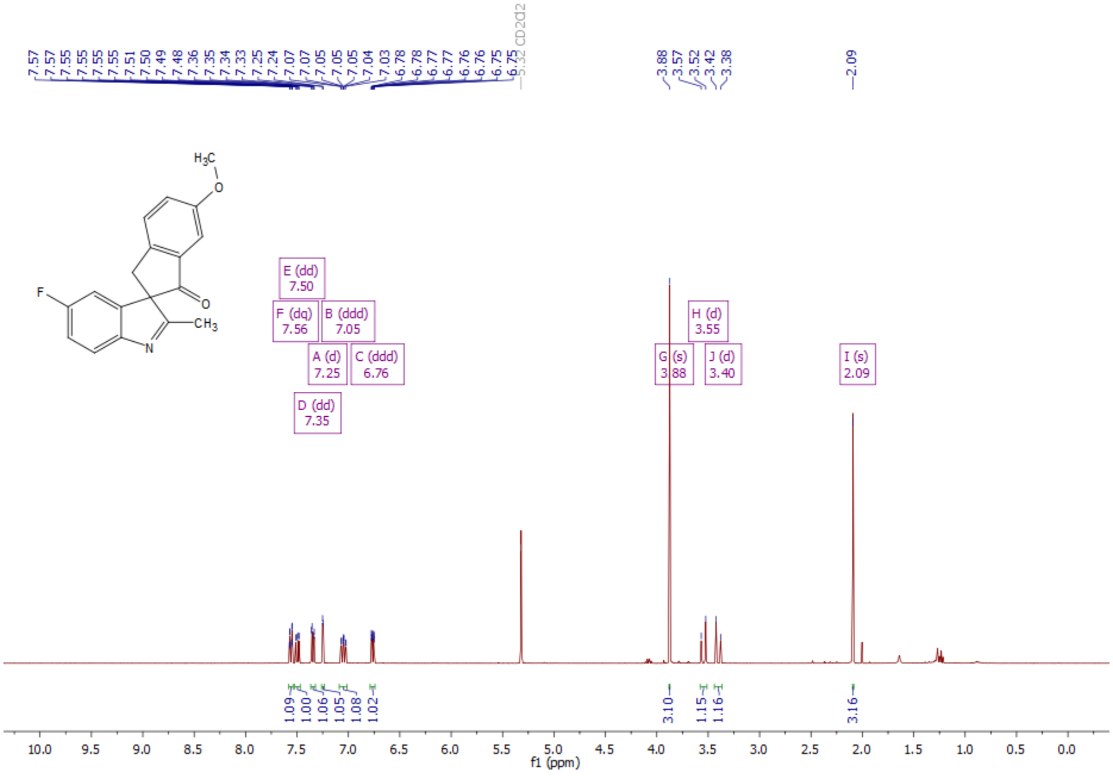

5,5'-difluoro-2'-methylspiro[indene-2,3'-indol]-1(3H)-one [**A22**]:

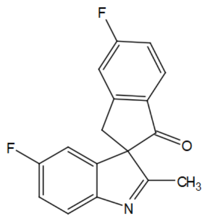

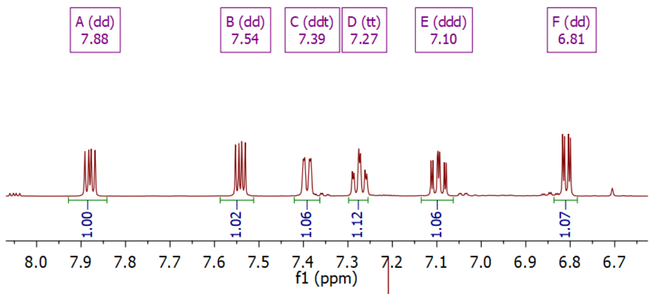

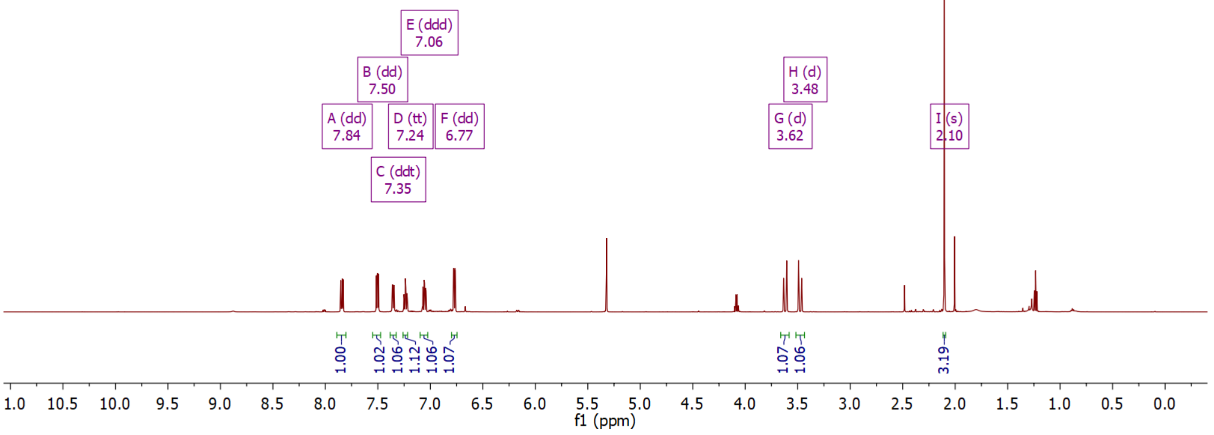

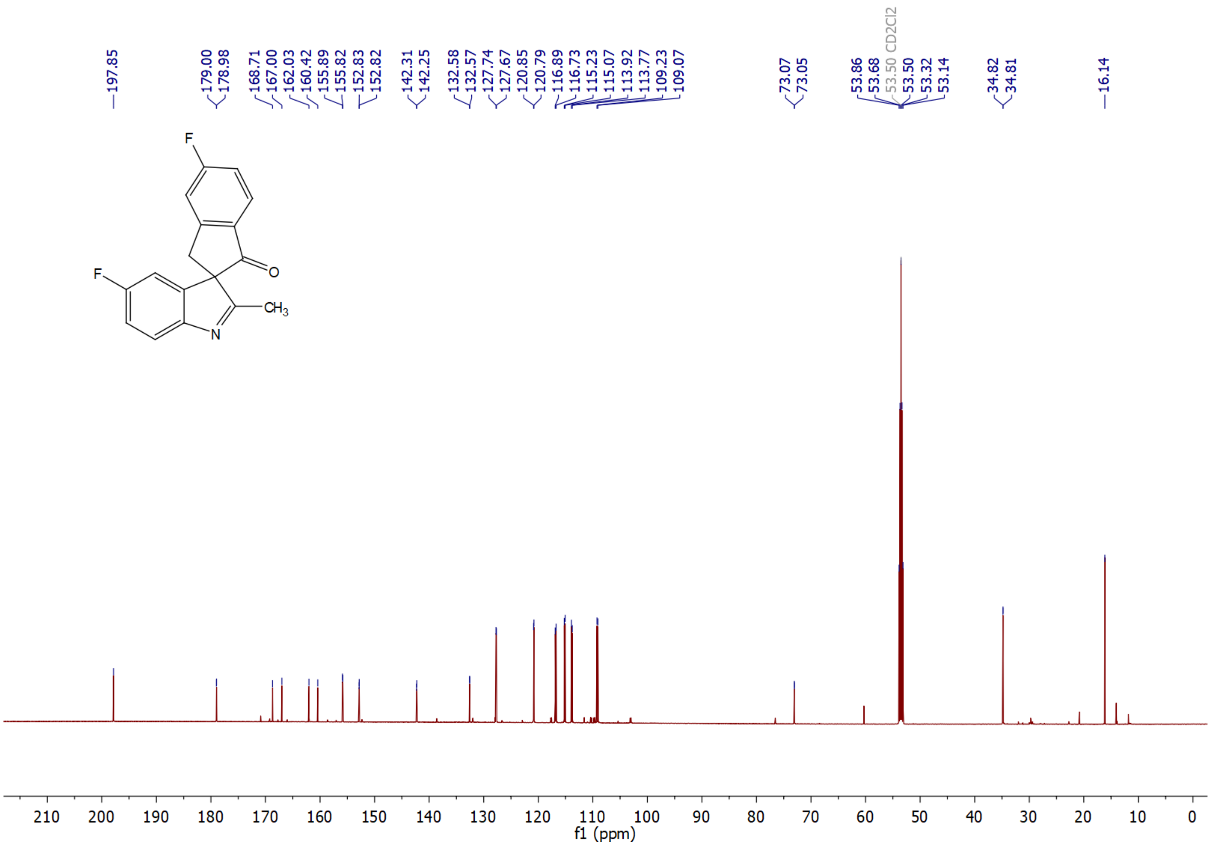

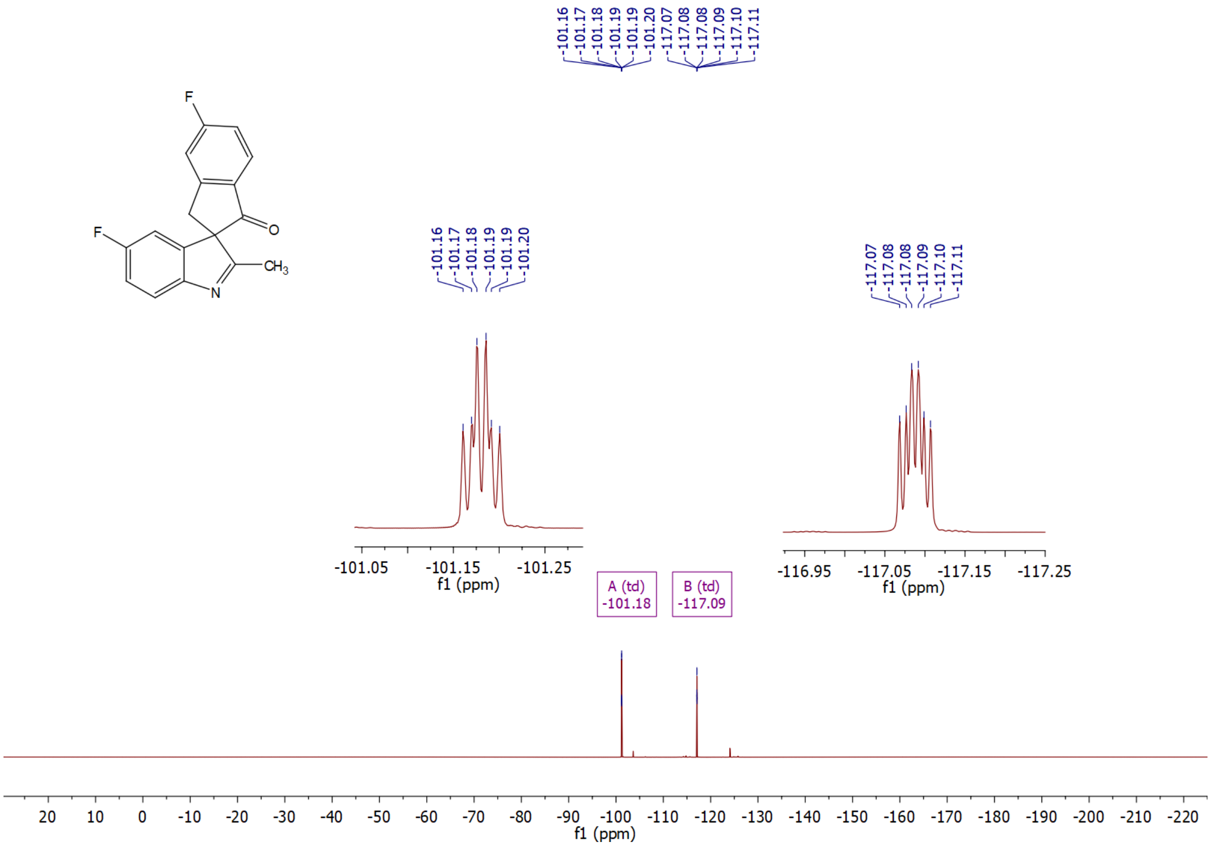

-101.16
-101.17
-101.18
-101.19
-101.19
-101.20
-117.07
-117.08
-117.08
-117.09
-117.10
-117.11
F
F
O
CH3
N
A (td)
-101.18
B (td)
-117.09
-101.05
-101.15
-101.25
f1 (ppm)
-116.95
-117.05
-117.15
-117.25
f1 (ppm)
20 10 0 -10 -20 -30 -40 -50 -60 -70 -80 -90 -100 -110 -120 -130 -140 -150 -160 -170 -180 -190 -200 -210 -220
f1 (ppm)

5'-chloro-5-fluoro-2'-methylspiro[indene-2,3'-indol]-1(3H)-one **[A23]**:

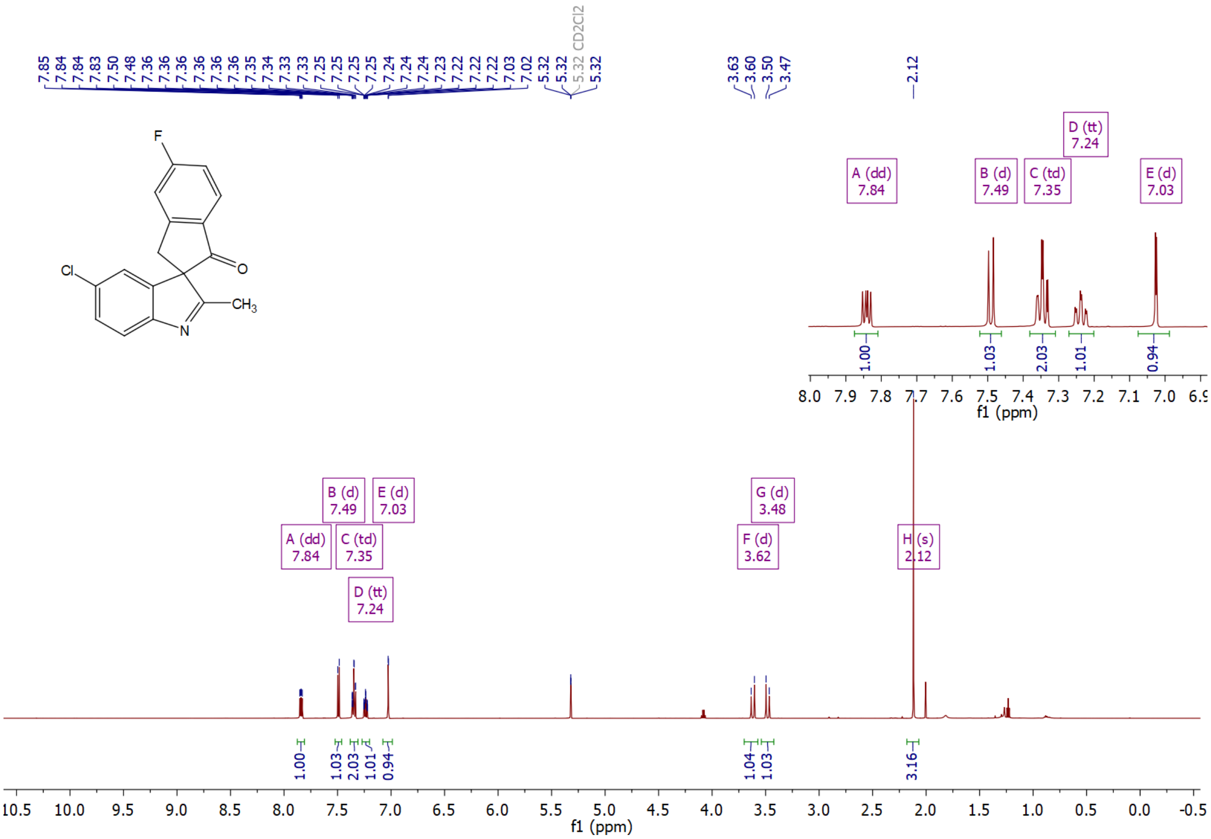

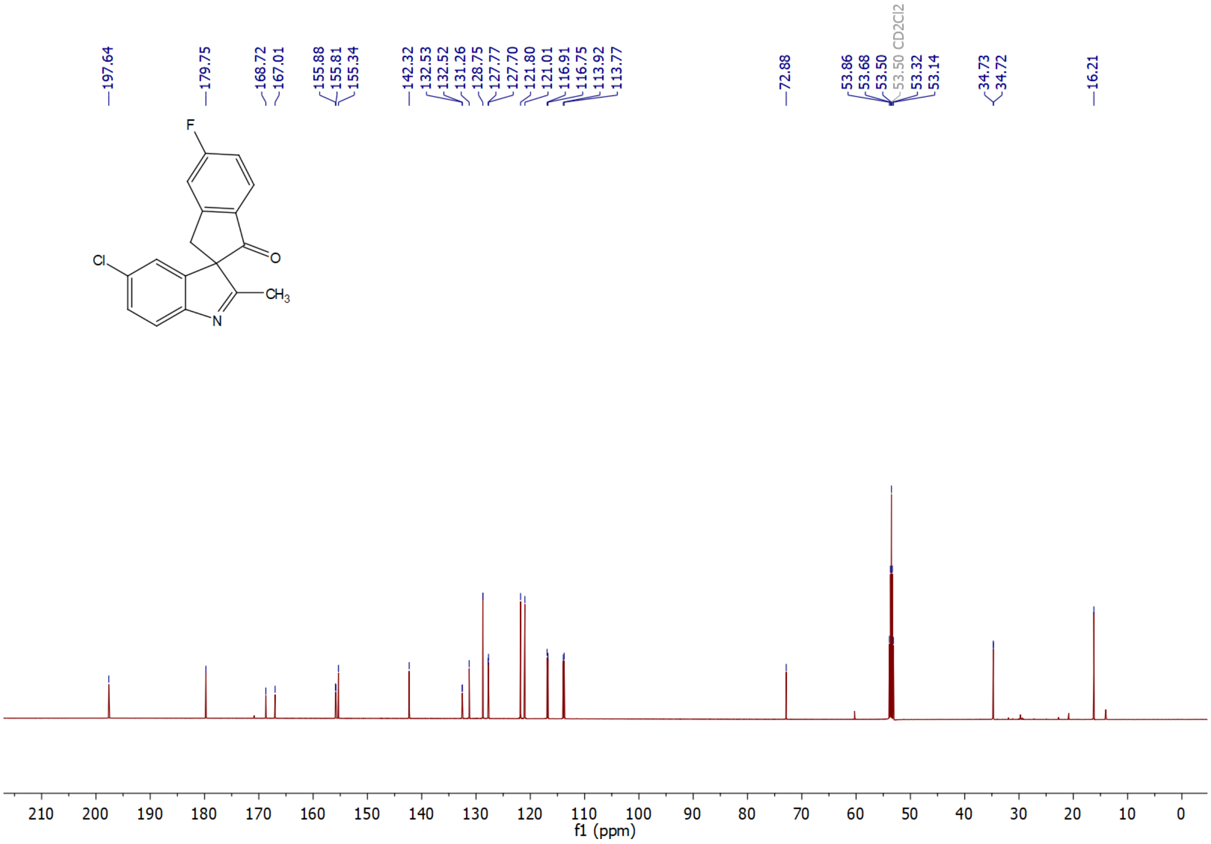

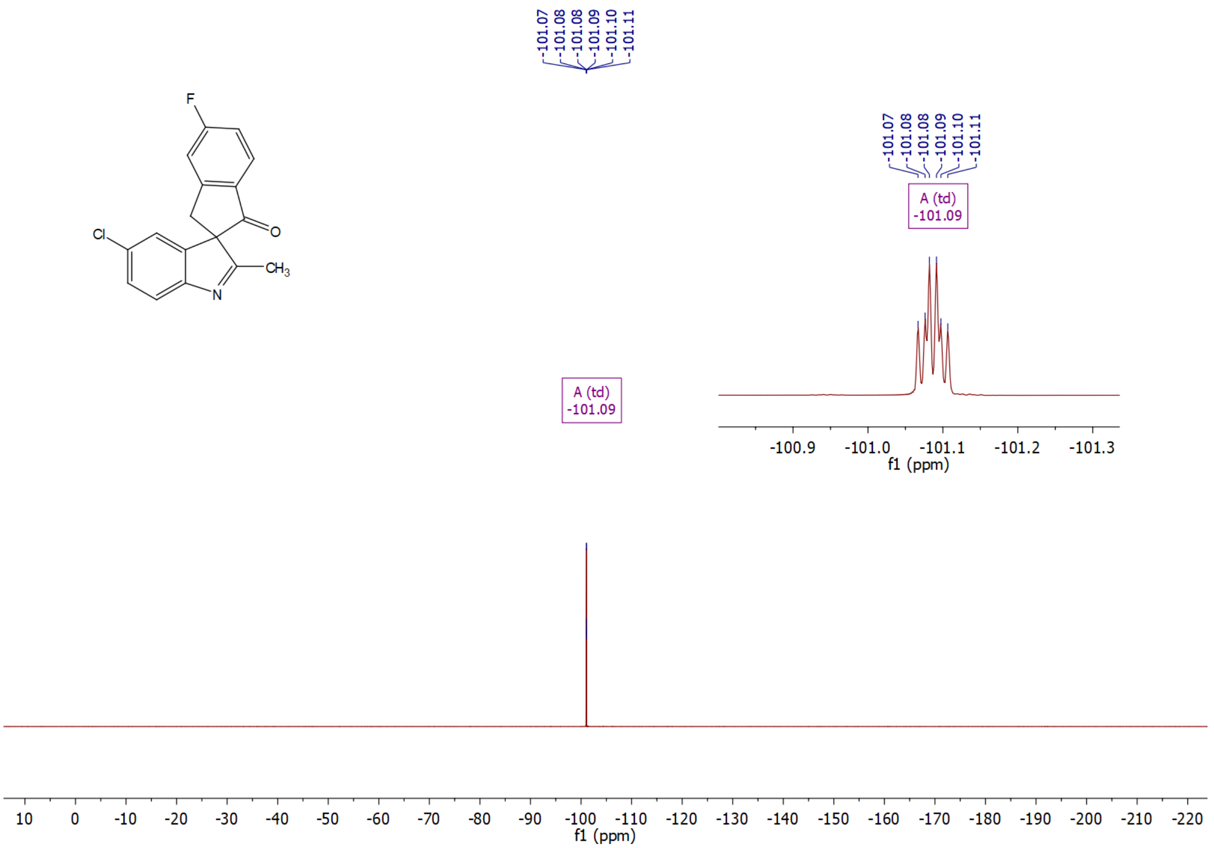

5'-chloro-5-fluoro-2'-methylspiro[indene-2,3'-indol]-1(3H)-one [**A23-resynthesized**]:

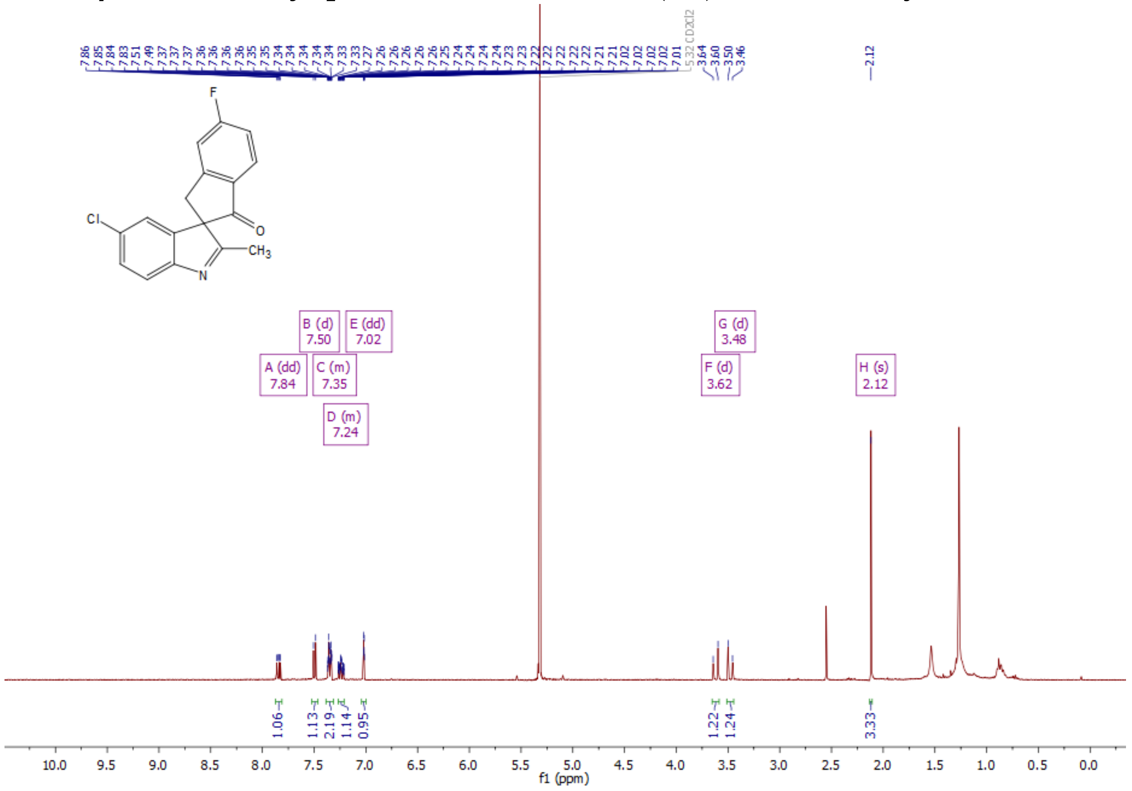

5'-fluoro-5-methoxy-2'-methylspiro[indene-2,3'-indol]-1(3H)-one [**A24**]:

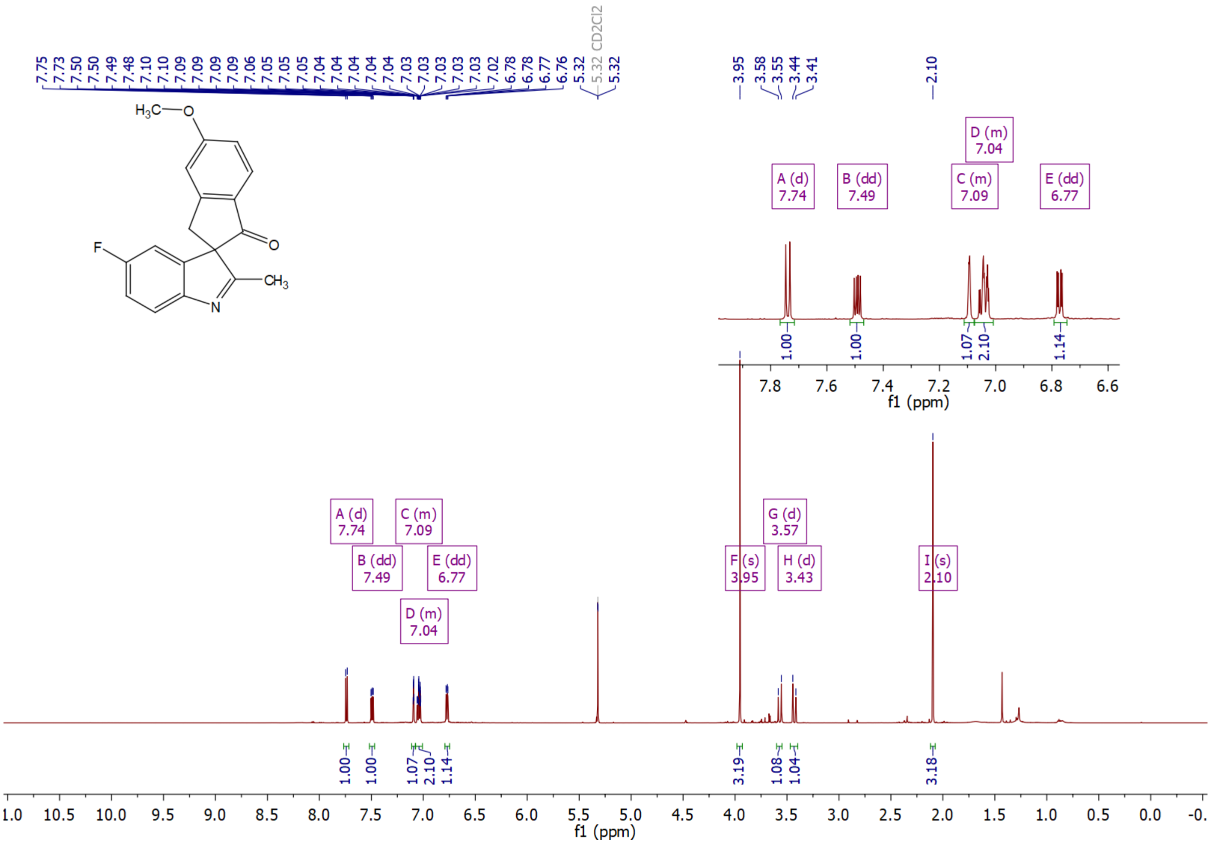

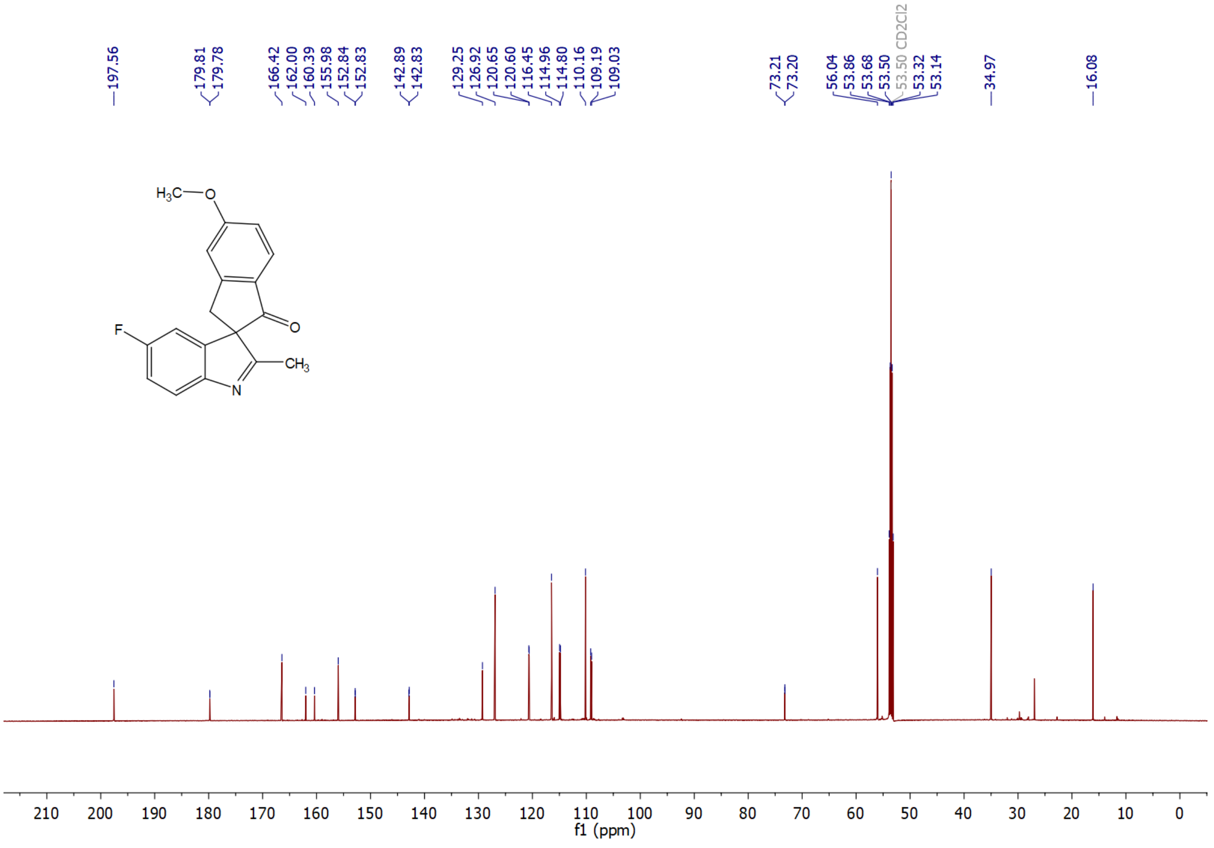

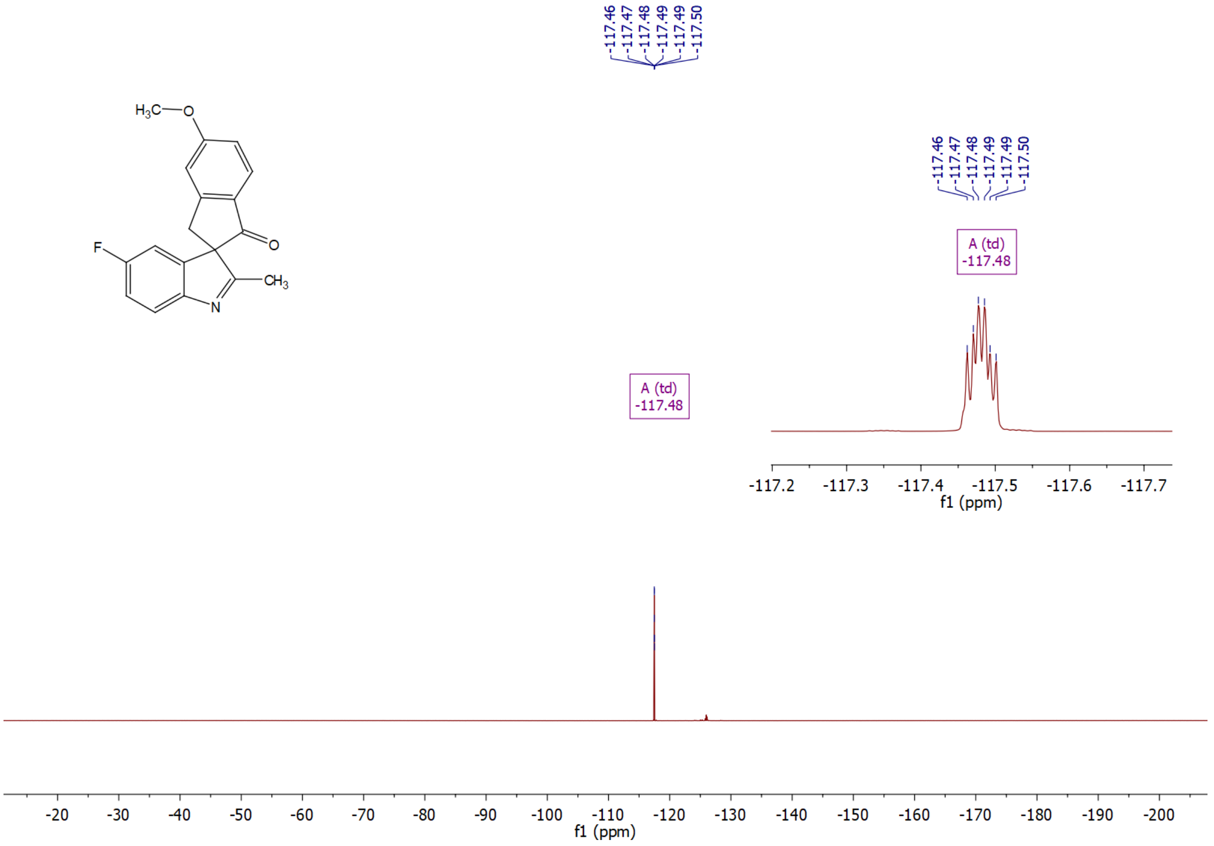
H3C—O
F
CH3
N
O
-117.46
-117.47
-117.48
-117.49
-117.49
-117.50
A (td)
-117.48
-117.2 -117.3 -117.4 -117.5 -117.6 -117.7
f1 (ppm)
-20 -30 -40 -50 -60 -70 -80 -90 -100 -110 -120 -130 -140 -150 -160 -170 -180 -190 -200
f1 (ppm)

2',5'-dimethyl-5-(trifluoromethyl)spiro[indene-2,3'-indol]-1(3H)-one [**A25**]:

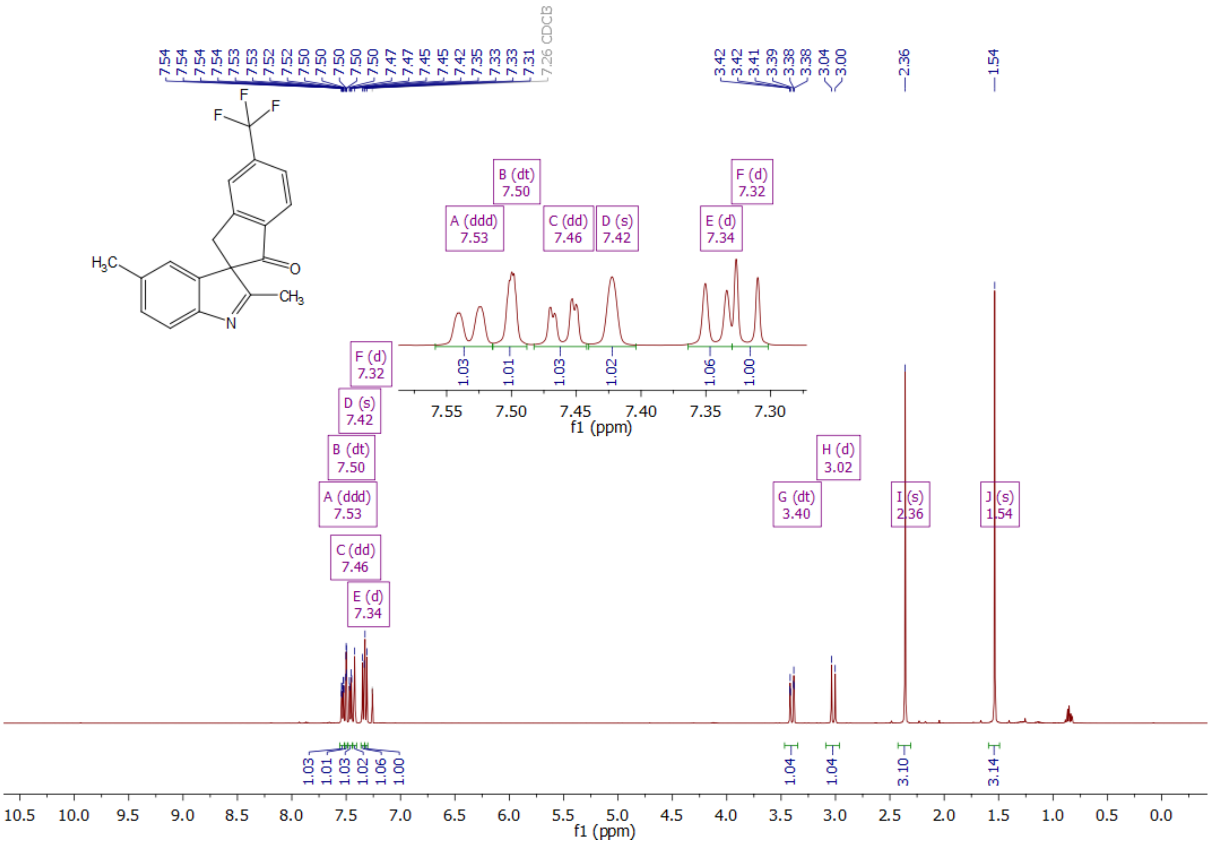

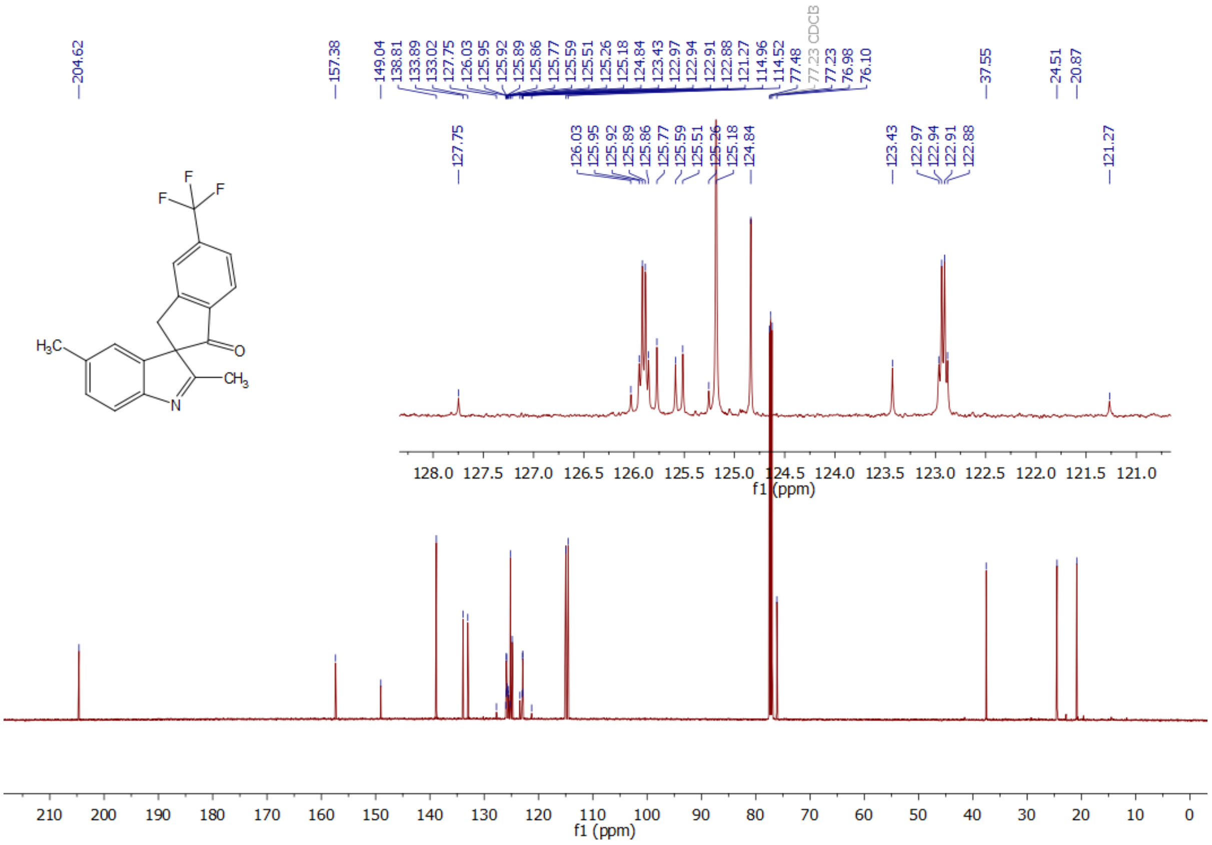

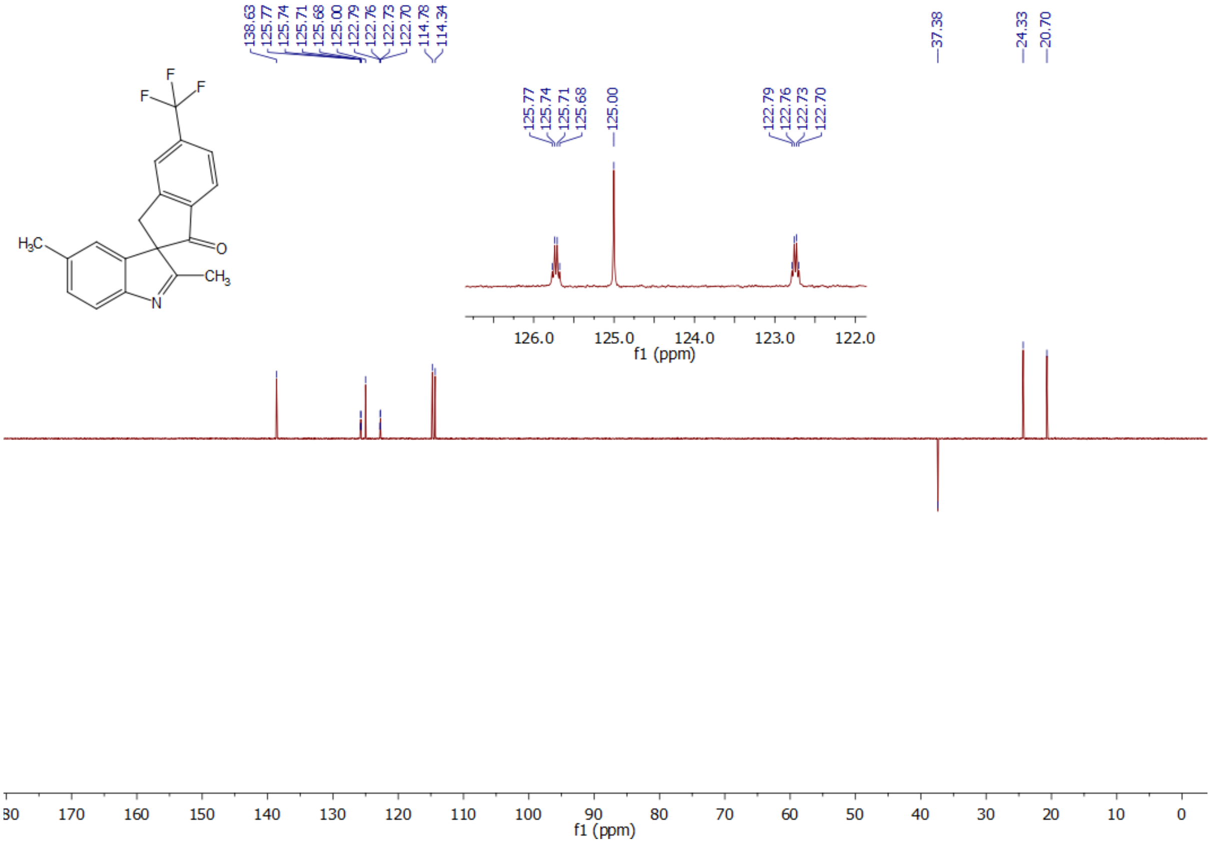
138.63
125.77
125.74
125.71
125.68
125.00
122.79
122.76
122.73
122.70
114.78
114.34
37.38
24.33
20.70
F
F
F
H3C
O
CH3
N
126.0
125.0
124.0
123.0
122.0
f1 (ppm)
170
160
150
140
130
120
110
100
90
80
70
60
50
40
30
20
10
0
f1 (ppm)

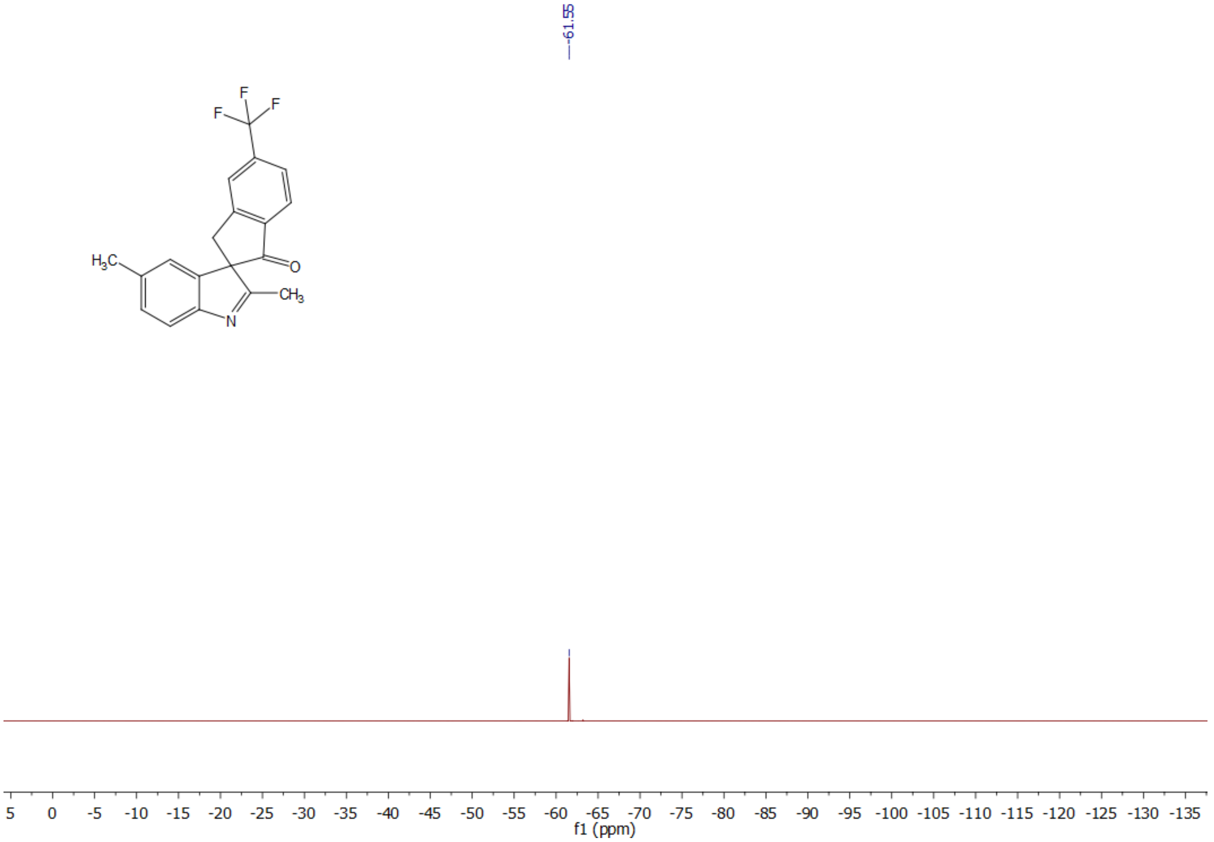
-61.55
F
F
F
H3C
O
CH3
N
5
0
-5
-10
-15
-20
-25
-30
-35
-40
-45
-50
-55
-60
-65
-70
-75
-80
-85
-90
-95
-100
-105
-110
-115
-120
-125
-130
-135
f1 (ppm)

4,5'-difluoro-2'-methylspiro[indene-2,3'-indol]-1(3H)-one [**A26**]:

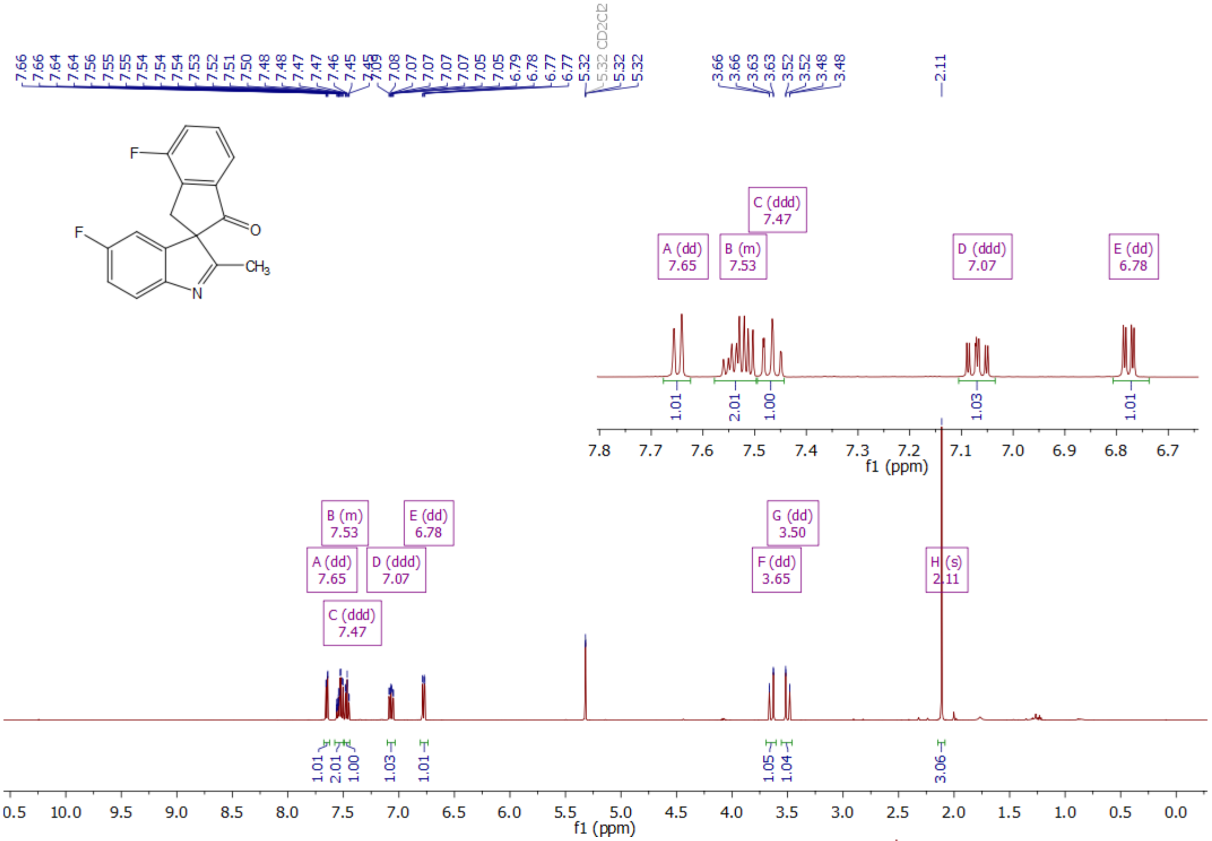

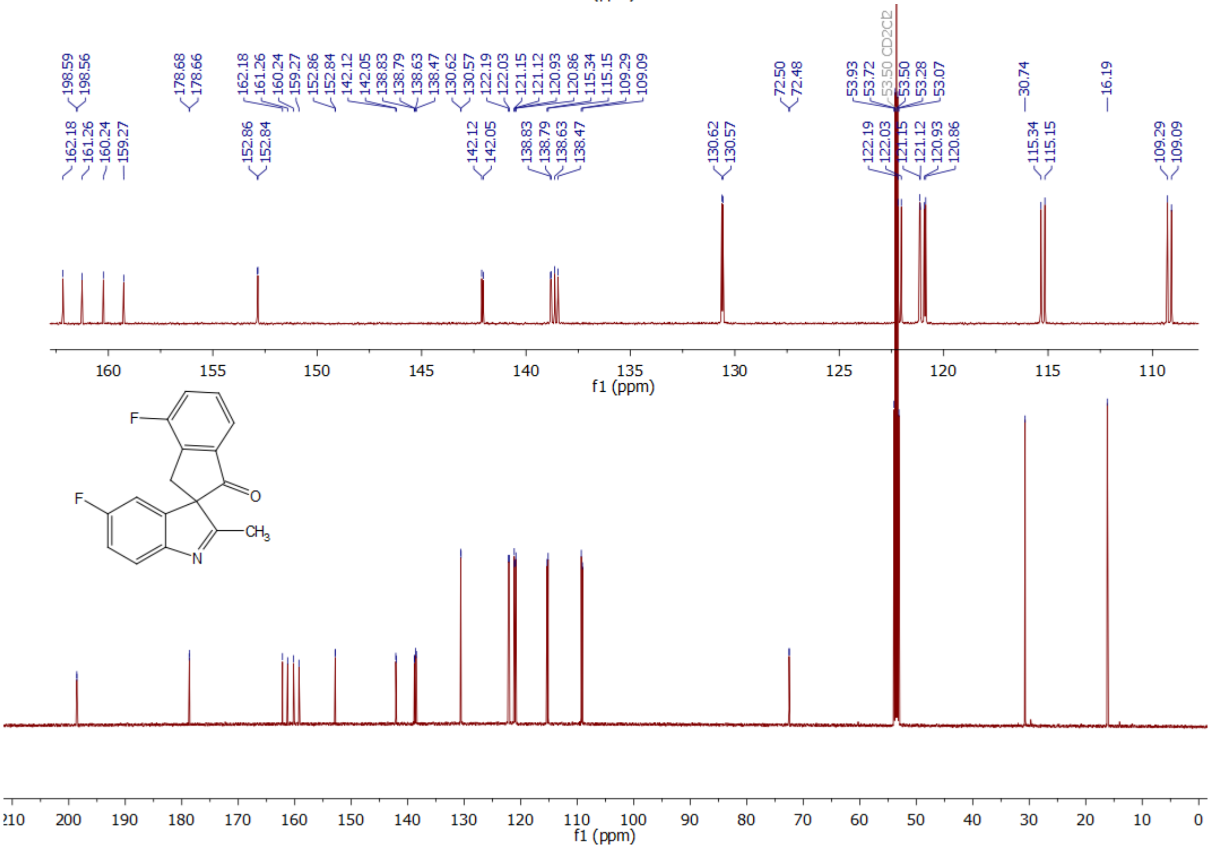

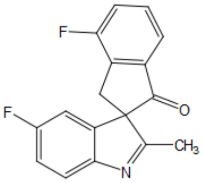
F
F
O
CH3
N

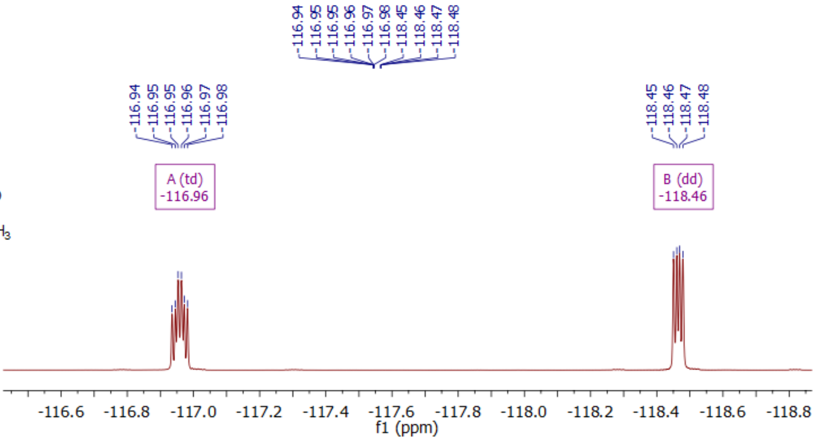
-116.94
-116.95
-116.95
-116.96
-116.97
-116.98
-118.45
-118.46
-118.47
-118.48
A (td)
-116.96
B (dd)
-118.46
-116.6 -116.8 -117.0 -117.2 -117.4 -117.6 -117.8 -118.0 -118.2 -118.4 -118.6 -118.8
f1 (ppm)

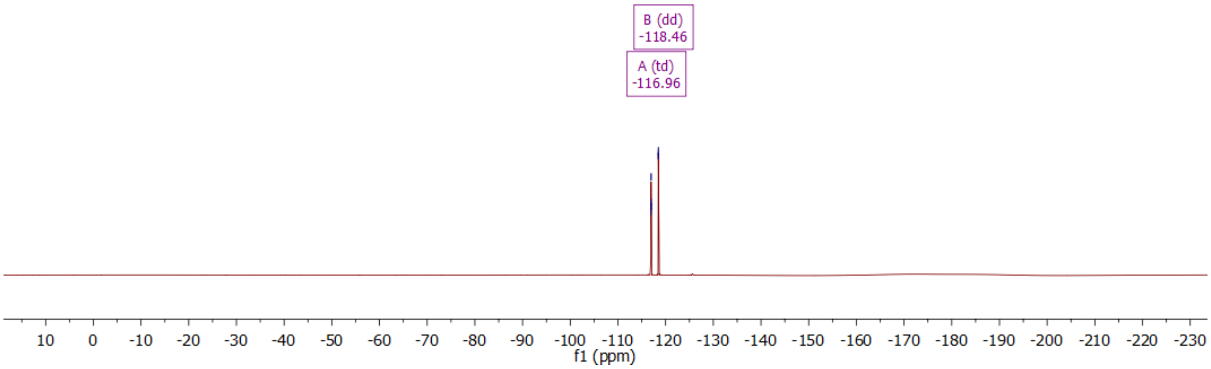
B (dd)
-118.46
A (td)
-116.96
10 0 -10 -20 -30 -40 -50 -60 -70 -80 -90 -100 -110 -120 -130 -140 -150 -160 -170 -180 -190 -200 -210 -220 -230
f1 (ppm)

5'-chloro-4-fluoro-2'-methylspiro[indene-2,3'-indol]-1(3H)-one [**A27**]:

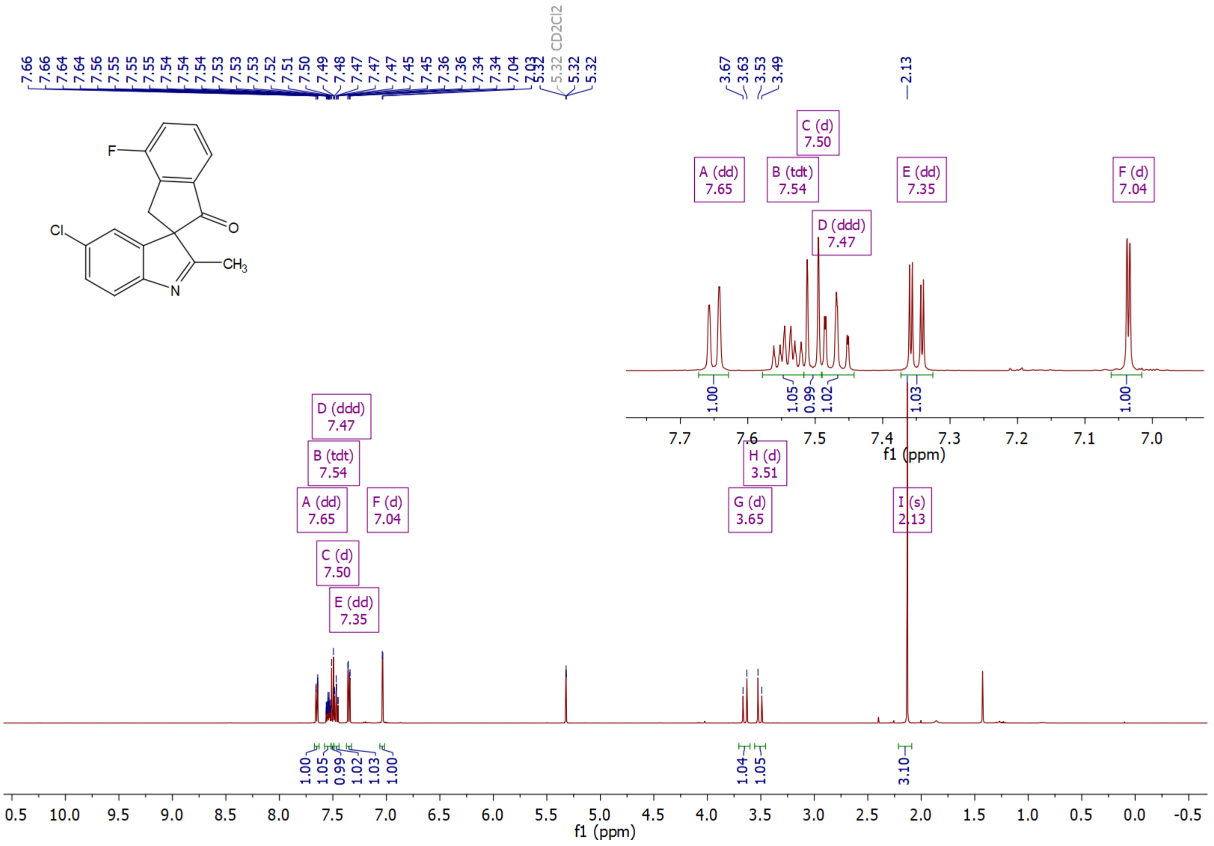

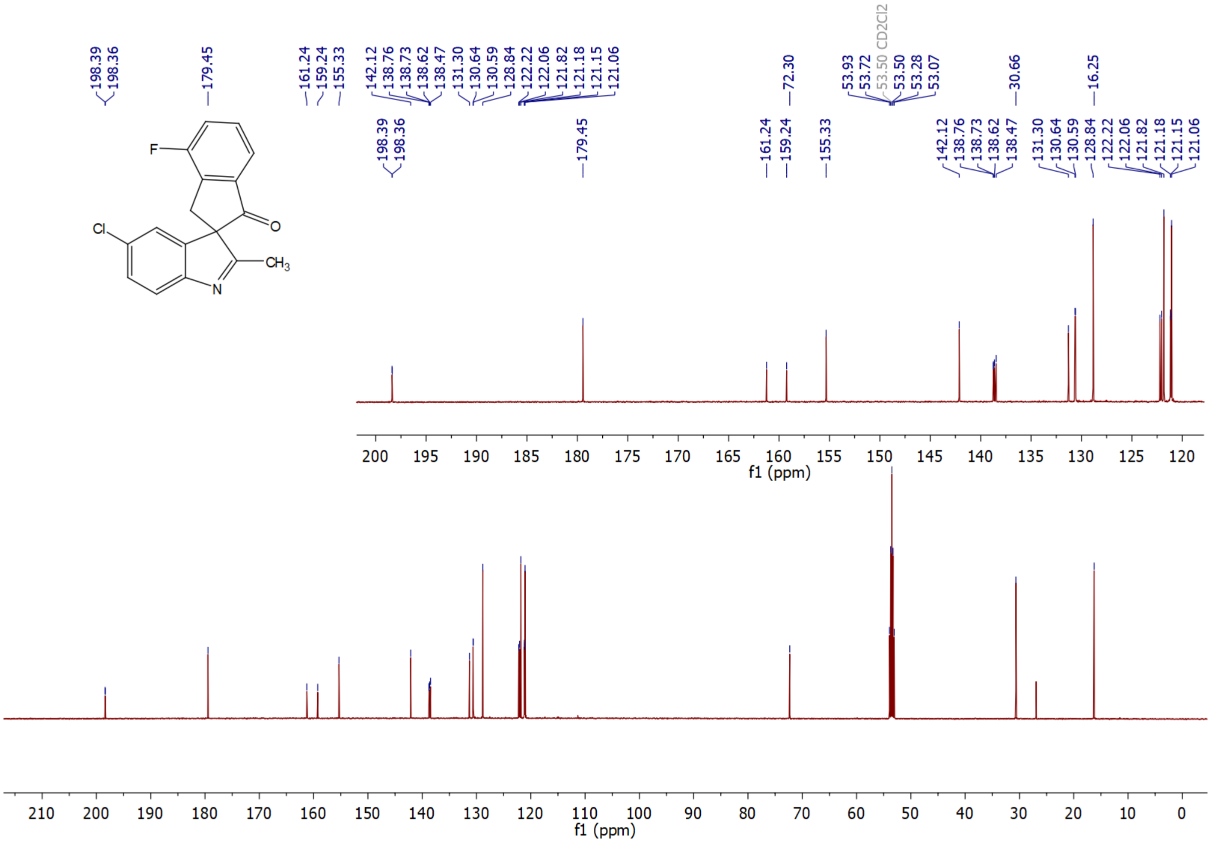

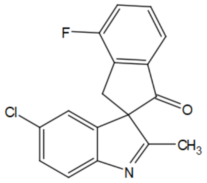
F
Cl
O
CH_3
N

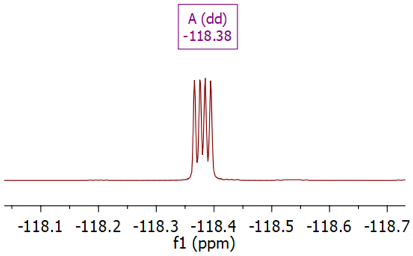
A (dd)
-118.38
-118.1 -118.2 -118.3 -118.4 -118.5 -118.6 -118.7
f1 (ppm)

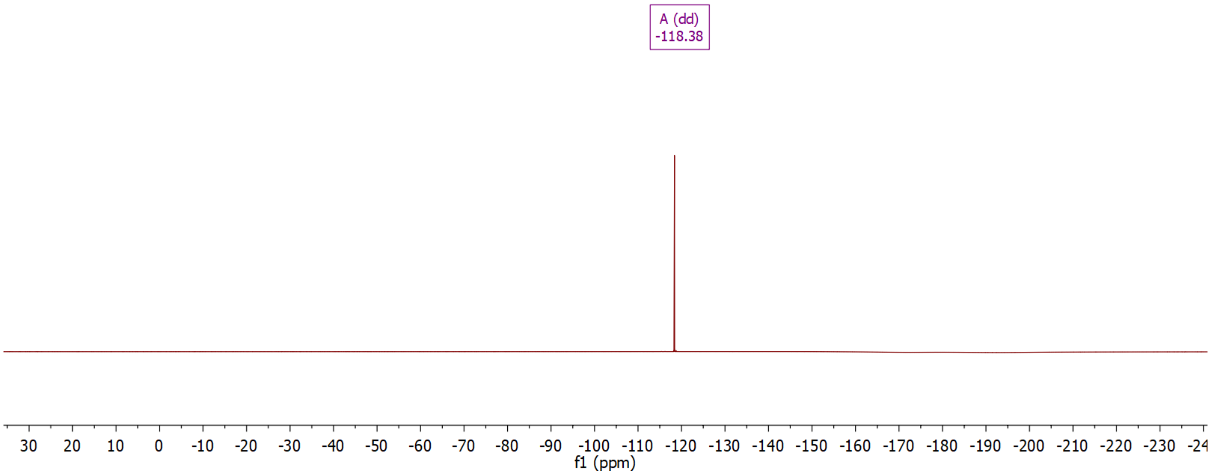
A (dd)
-118.38
30 20 10 0 -10 -20 -30 -40 -50 -60 -70 -80 -90 -100 -110 -120 -130 -140 -150 -160 -170 -180 -190 -200 -210 -220 -230 -24
f1 (ppm)

4-fluoro-2'-methyl-5'-(thiophen-3-yl)spiro[indene-2,3'-indol]-1(3H)-one [**A28**]:

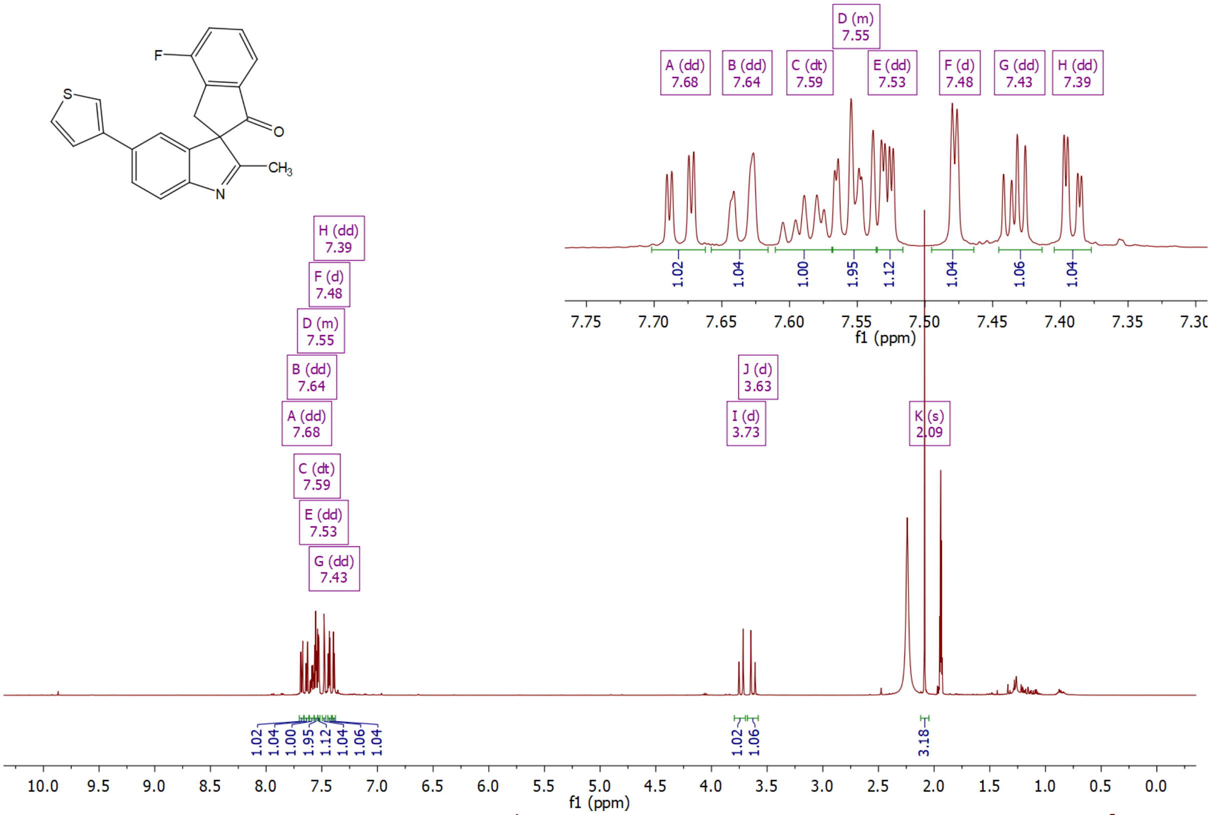

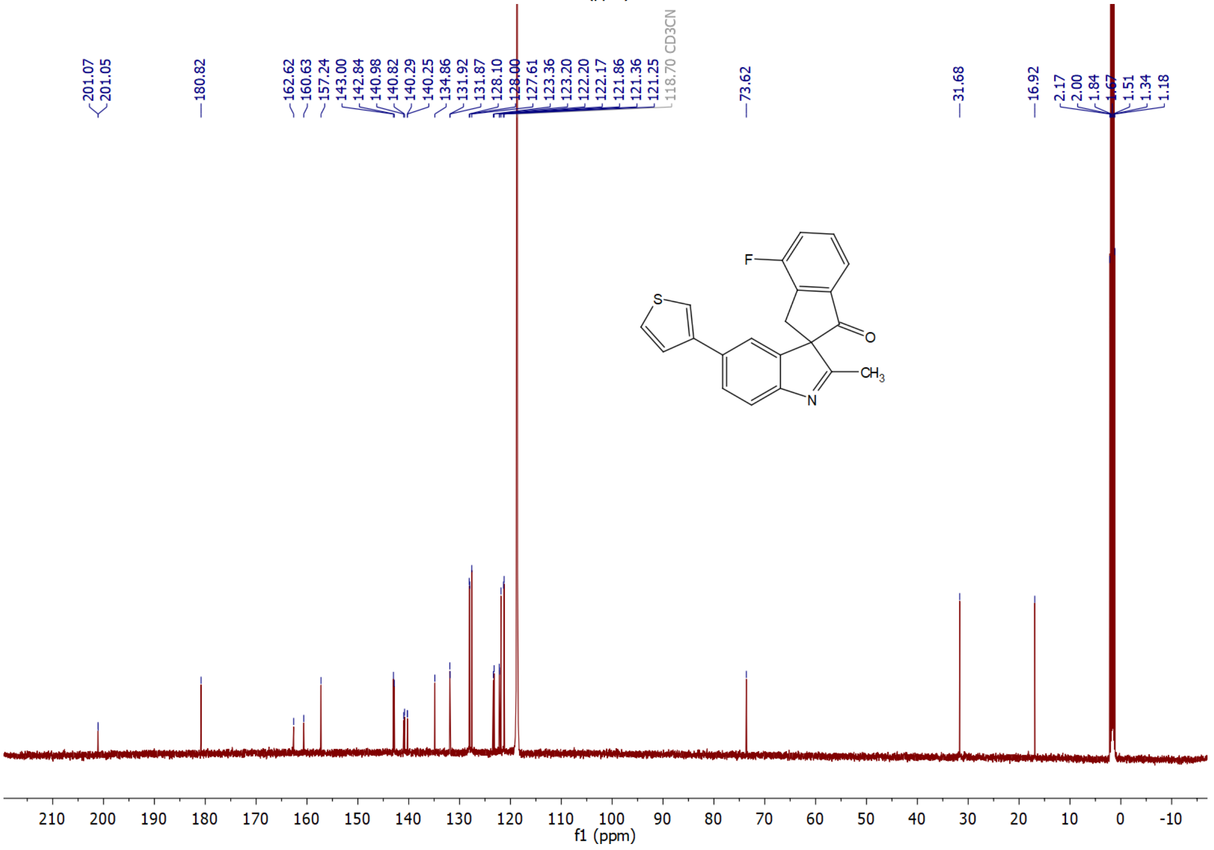

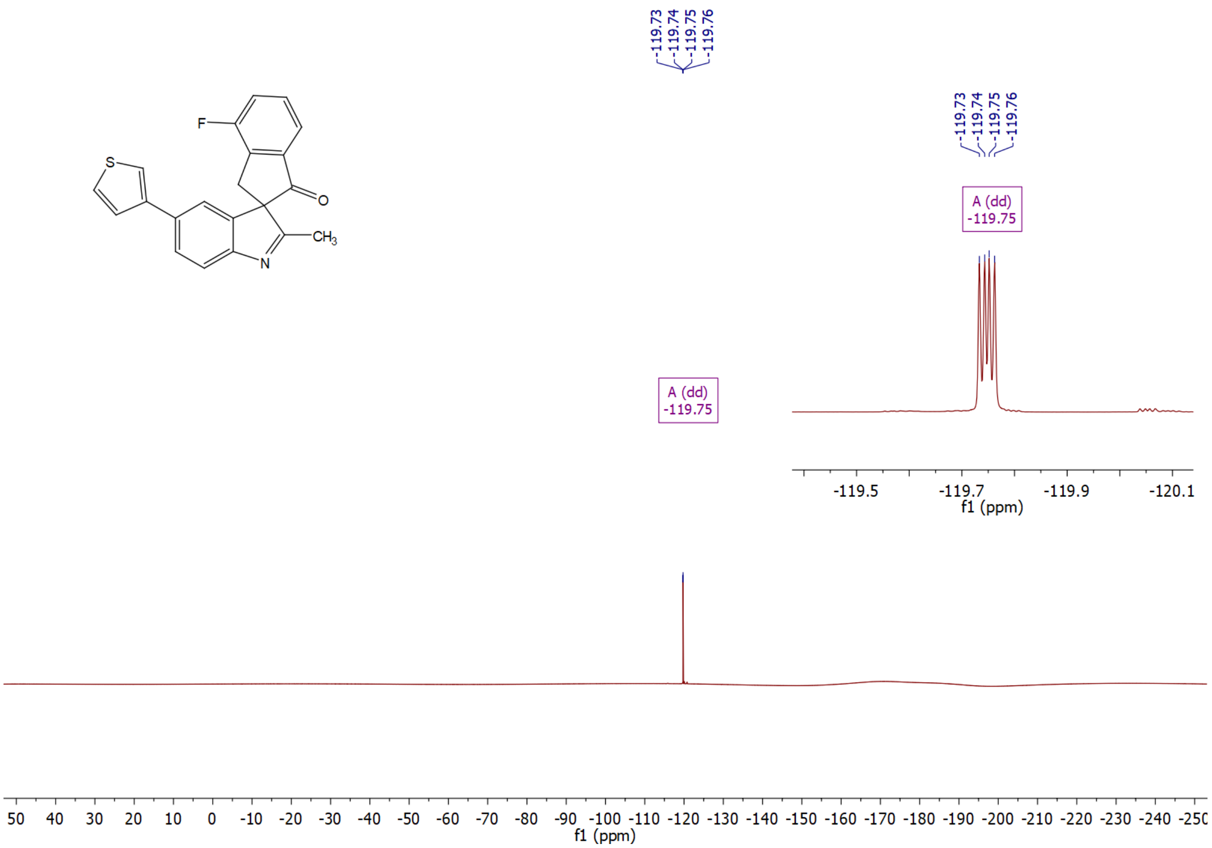
F
S
O
CH3
N
-119.73
-119.74
-119.75
-119.76
A (dd)
-119.75
-119.5
-119.7
-119.9
-120.1
f1 (ppm)
50 40 30 20 10 0 -10 -20 -30 -40 -50 -60 -70 -80 -90 -100 -110 -120 -130 -140 -150 -160 -170 -180 -190 -200 -210 -220 -230 -240 -250
f1 (ppm)

5'-fluoro-5,6-dimethoxy-2'-methylspiro[indene-2,3'-indol]-1(3H)-one [**A29**]:

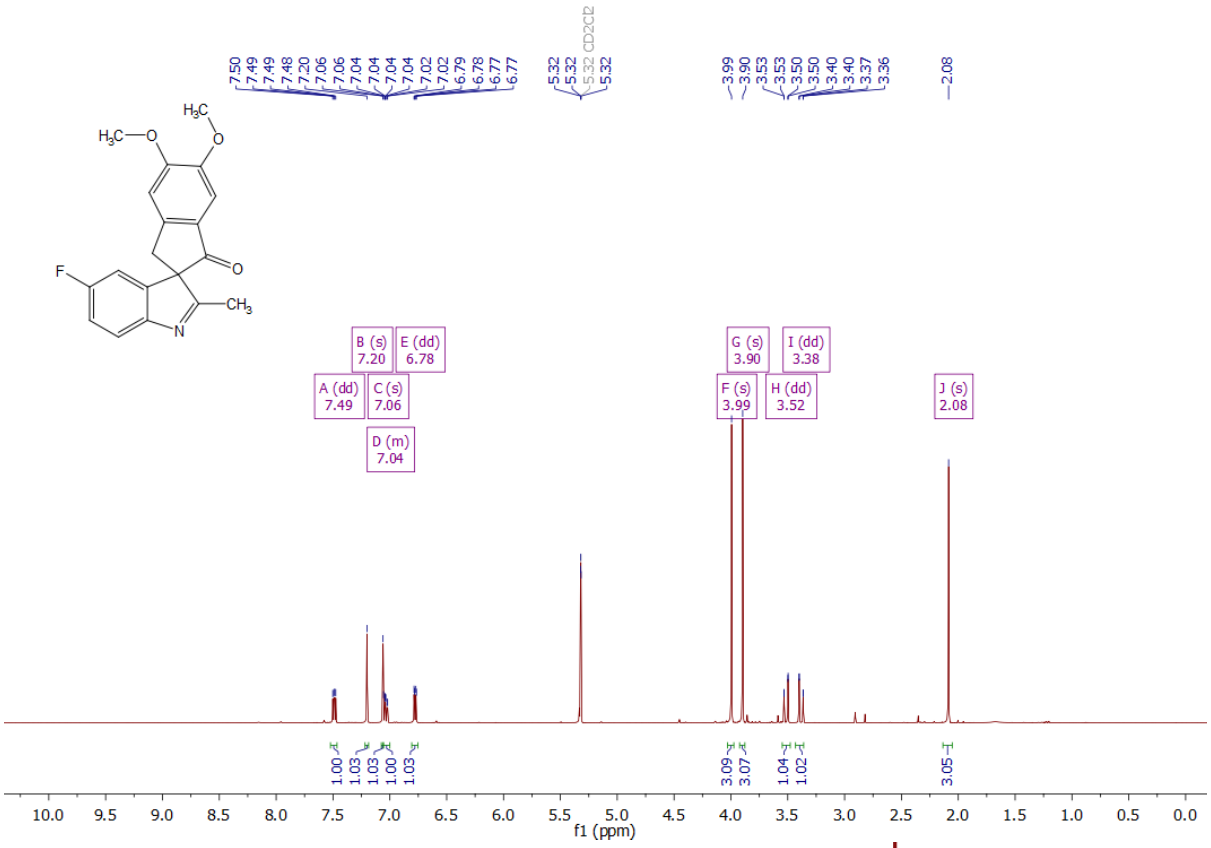

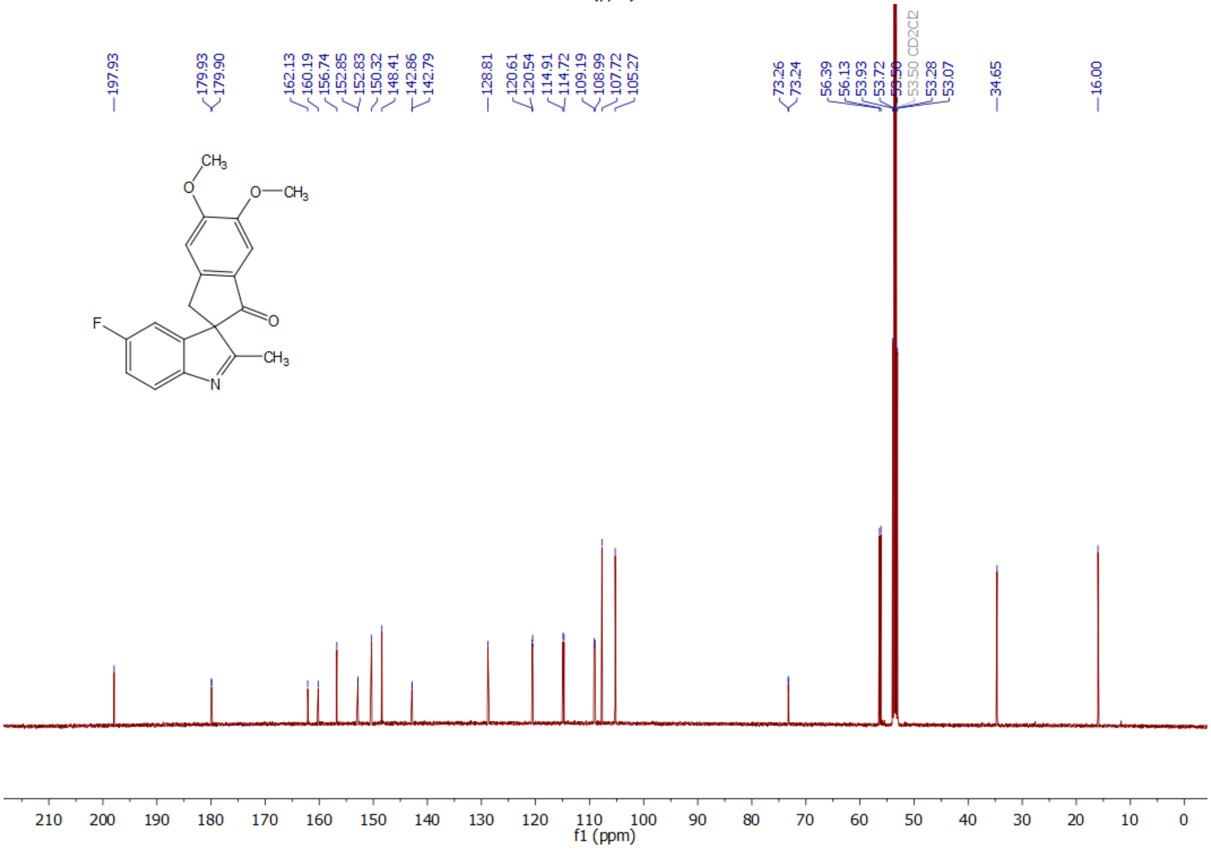

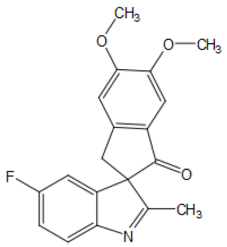

CH3
O
O—CH3
O
F
CH3
N

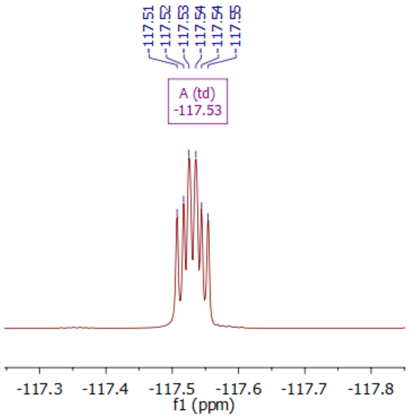

-117.51
-117.52
-117.53
-117.54
-117.54
-117.55
A (td)
-117.53
-117.3 -117.4 -117.5 -117.6 -117.7 -117.8
f1 (ppm)

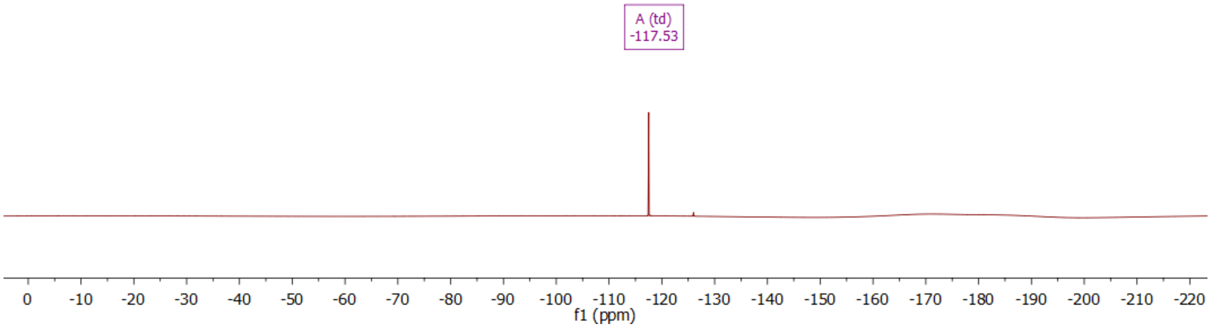

A (td)
-117.53
0 -10 -20 -30 -40 -50 -60 -70 -80 -90 -100 -110 -120 -130 -140 -150 -160 -170 -180 -190 -200 -210 -220
f1 (ppm)

5'-chloro-5,6-dimethoxy-2'-methylspiro[indene-2,3'-indol]-1(3H)-one **[A30]**:

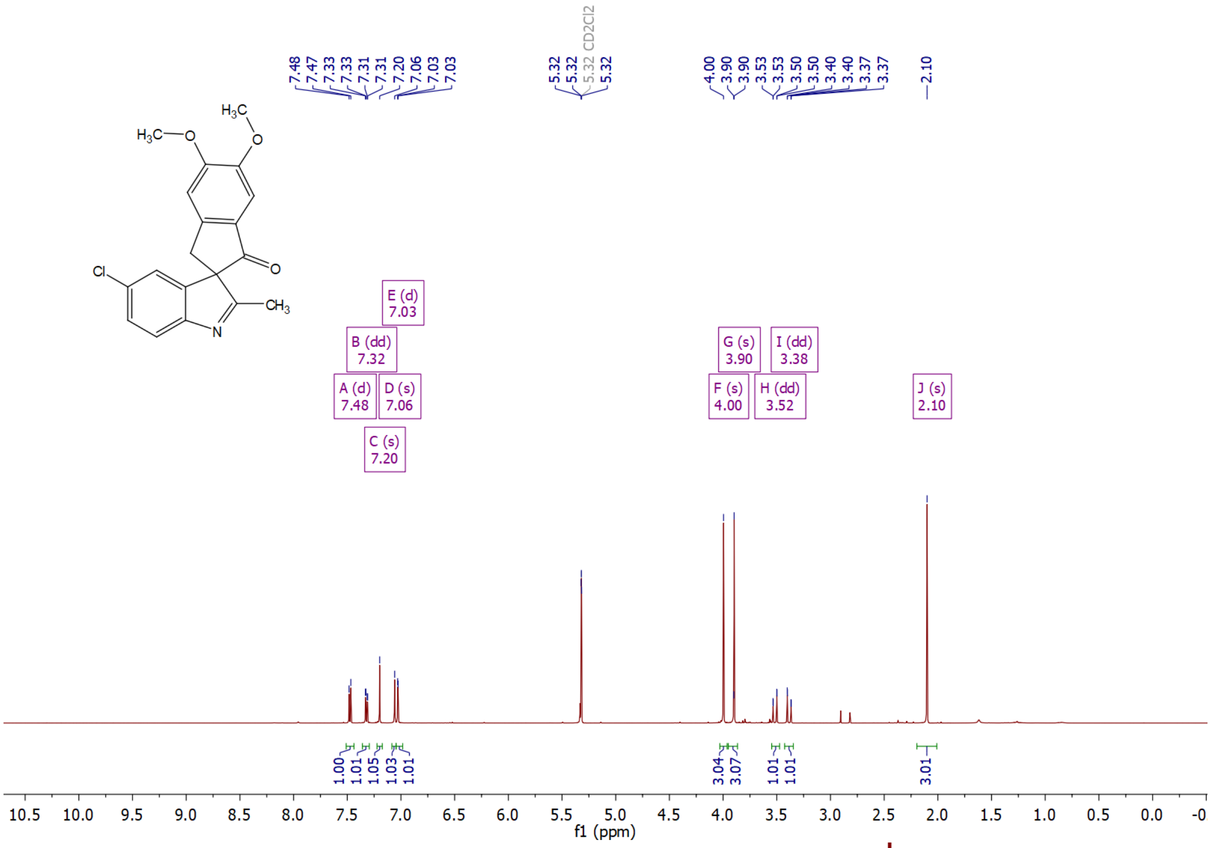

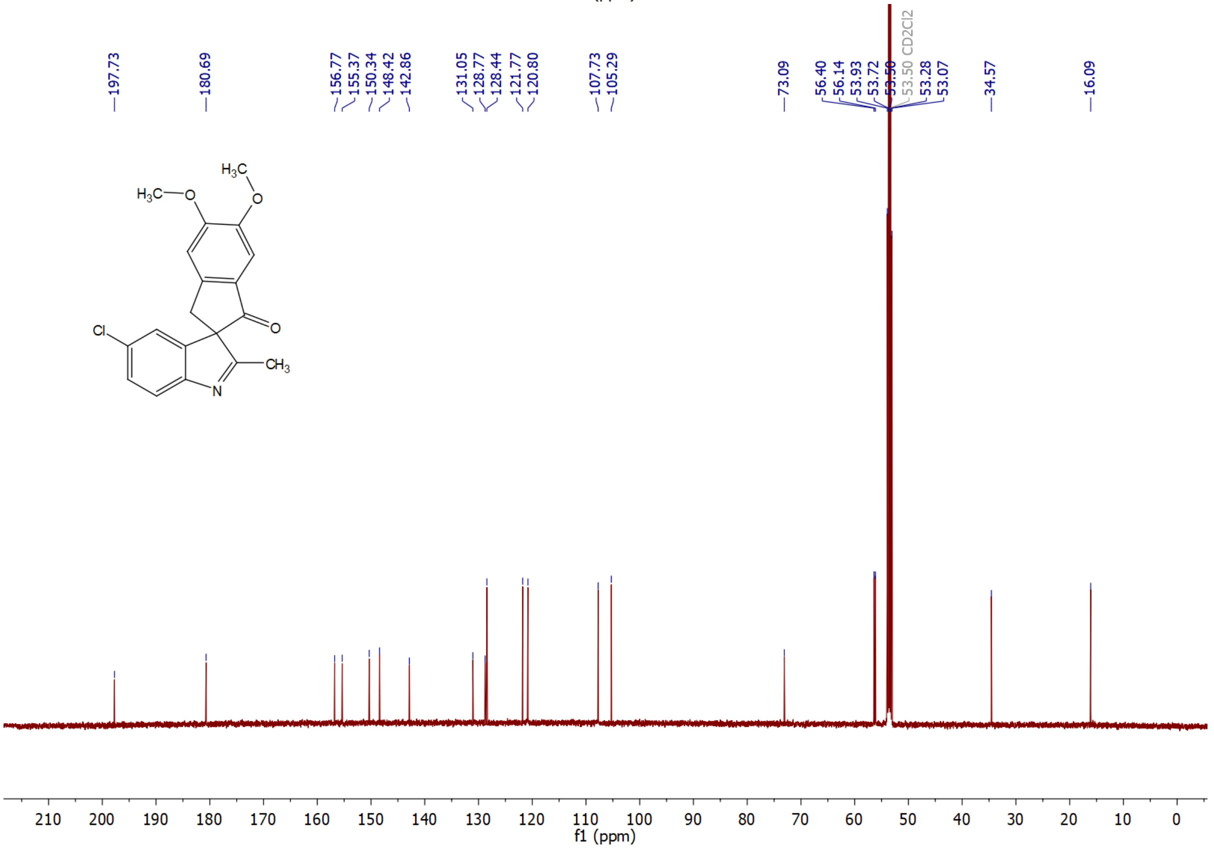

5,6-dimethoxy-2',5'-dimethylspiro[indene-2,3'-indol]-1(3H)-one [**A31**]:

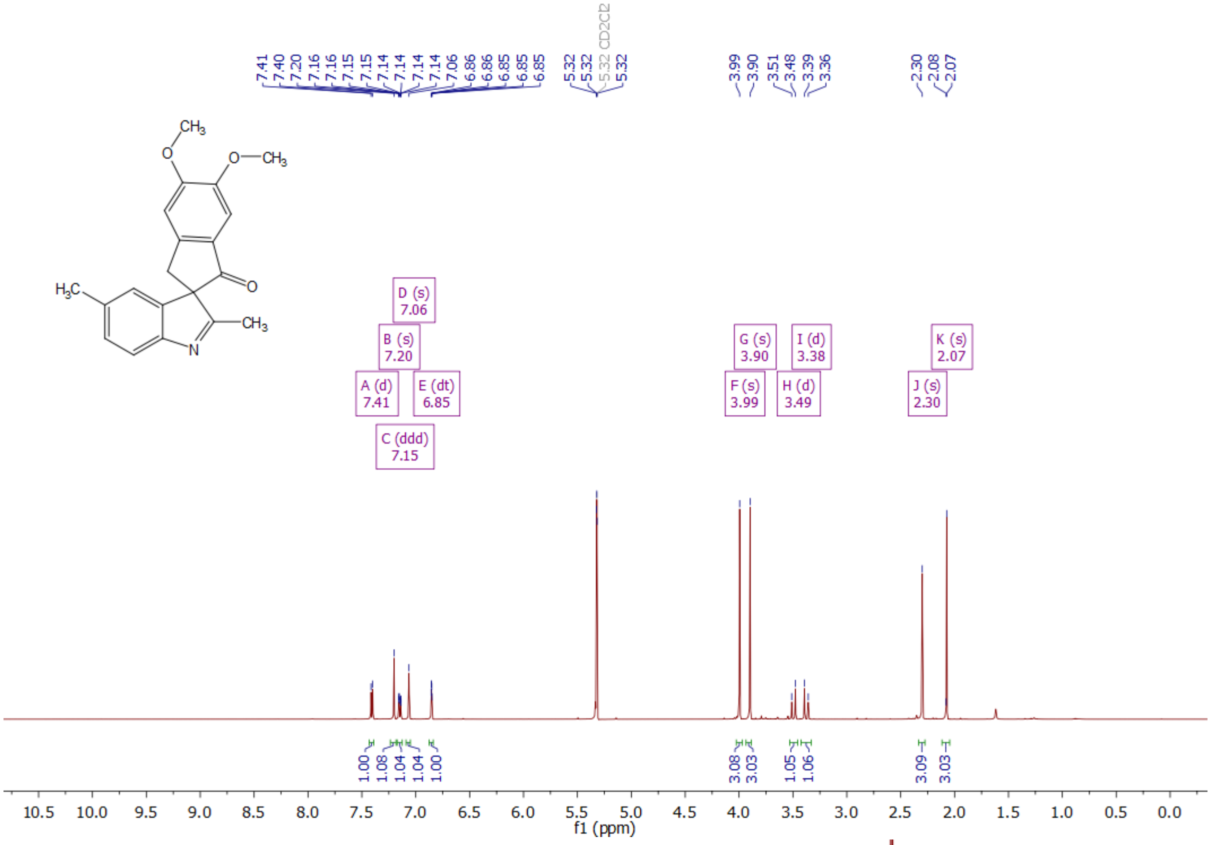

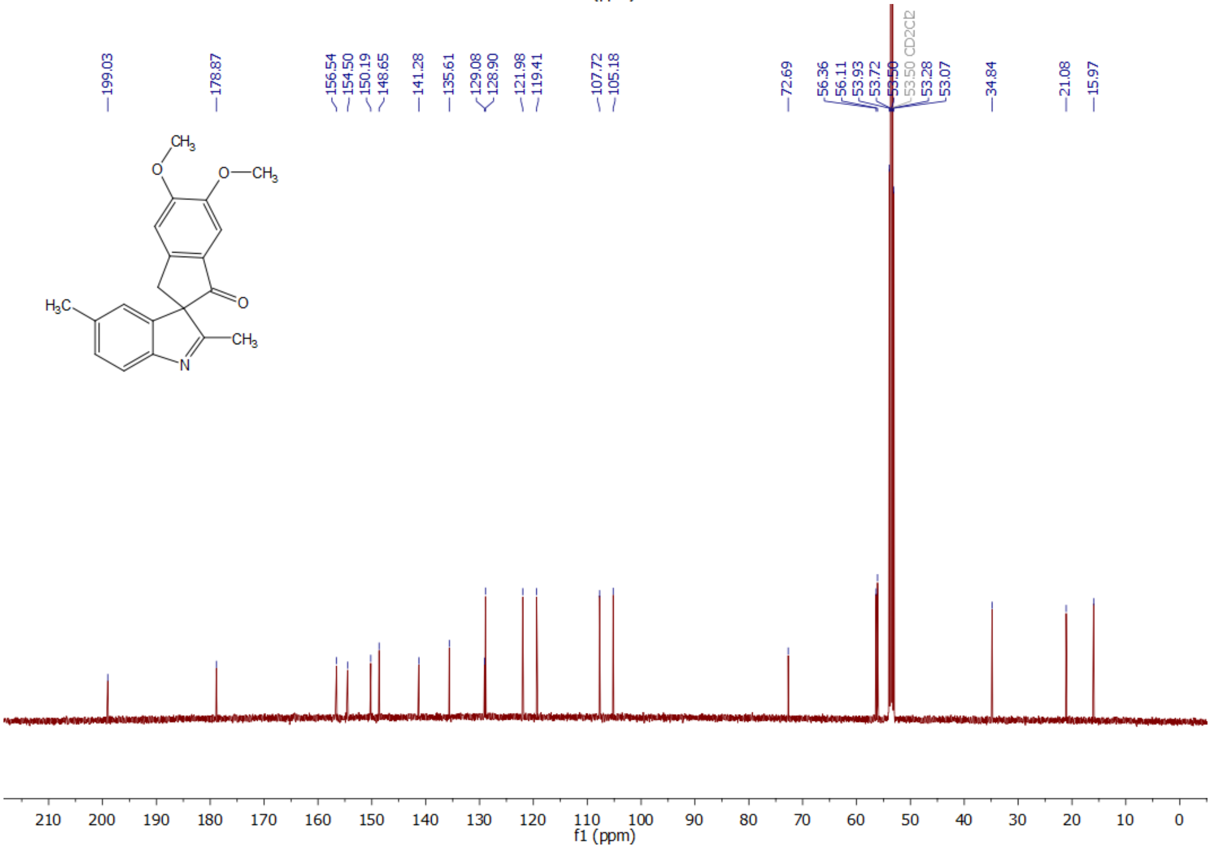

5'-fluoro-2'-methylspiro[indeno[5,6-d][1,3]dioxole-6,3'-indol]-5(7H)-one [A32]:

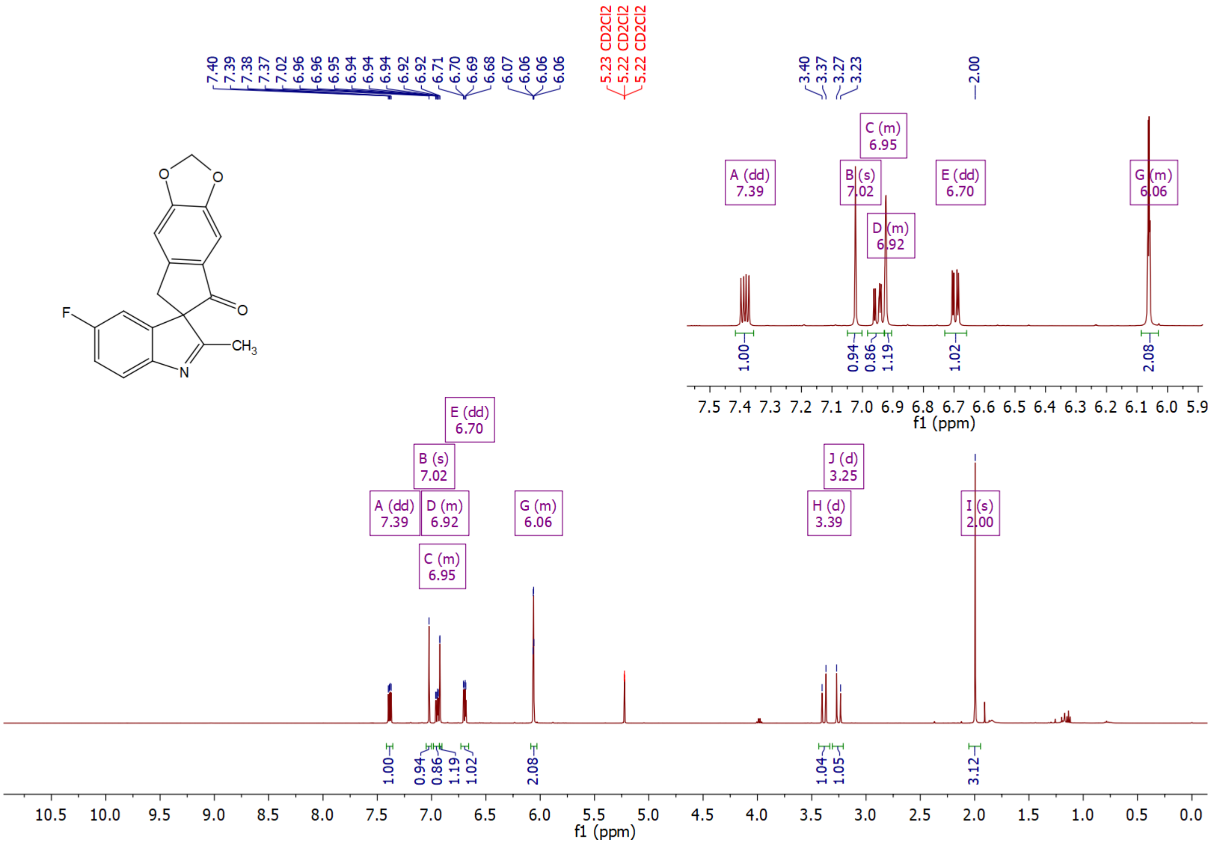

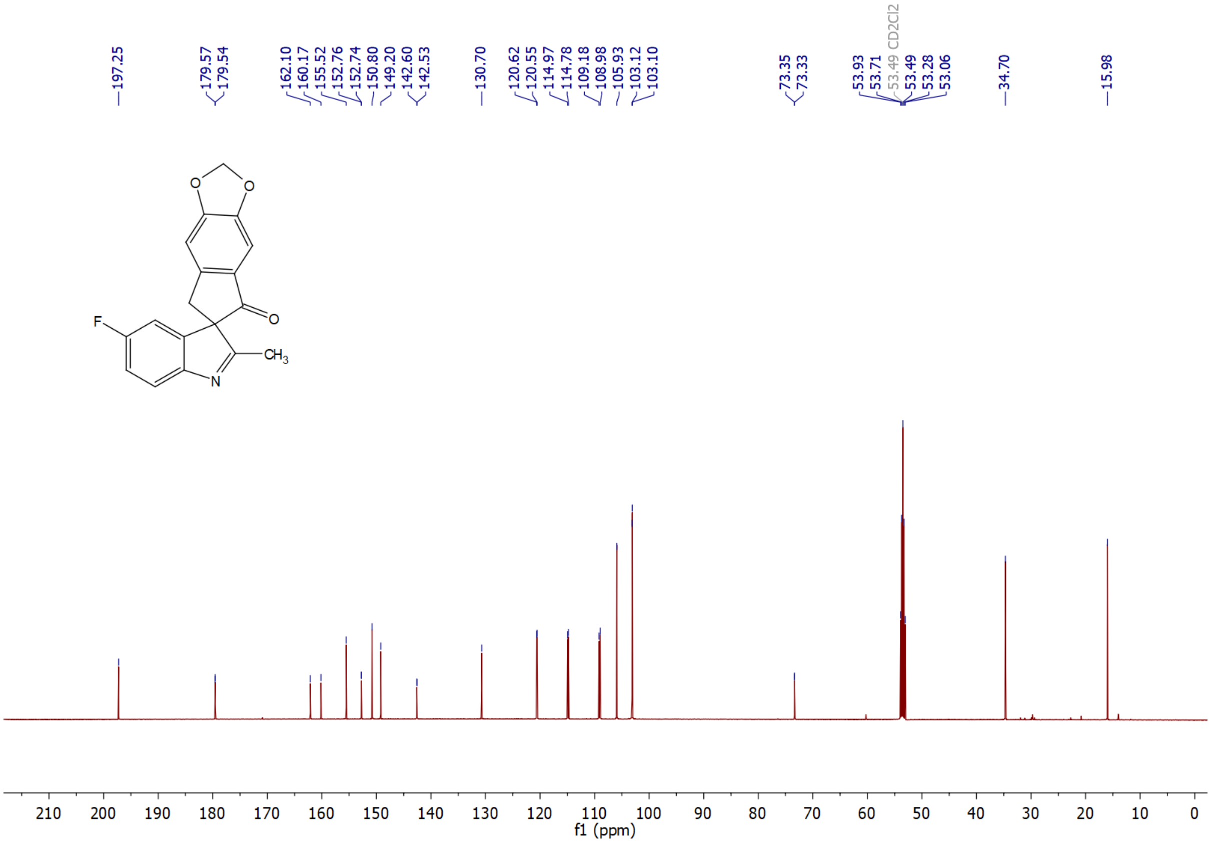

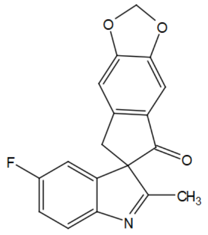
F
O
O
O
CH3
N

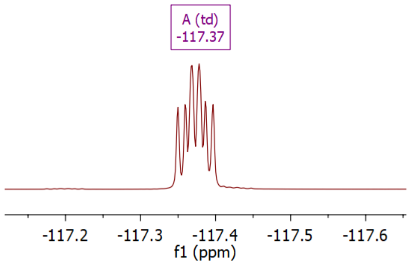
A (td)
-117.37
-117.2
-117.3
-117.4
-117.5
-117.6
f1 (ppm)

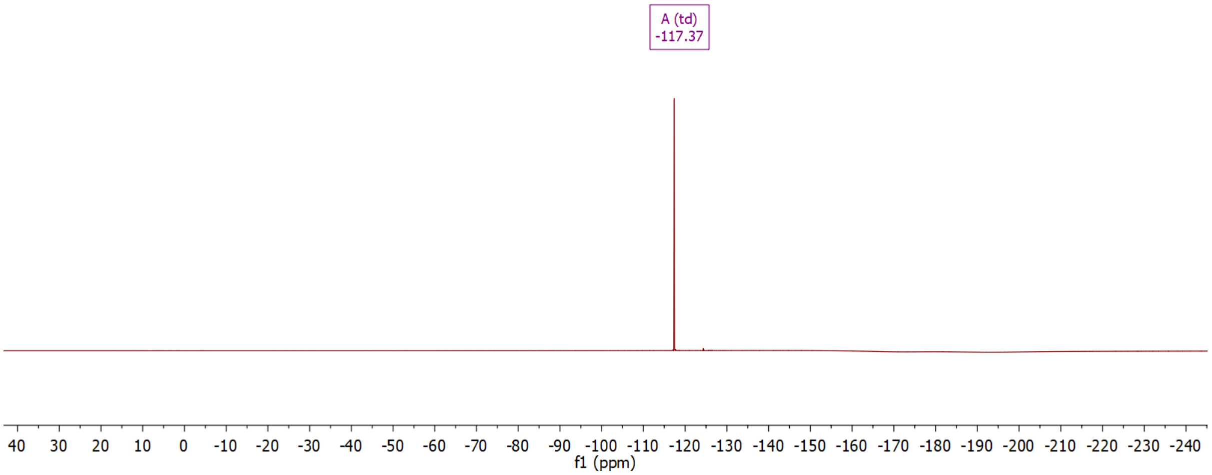
A (td)
-117.37
40 30 20 10 0 -10 -20 -30 -40 -50 -60 -70 -80 -90 -100 -110 -120 -130 -140 -150 -160 -170 -180 -190 -200 -210 -220 -230 -240
f1 (ppm)

2',5'-dimethylspiro[indeno[5,6-d][1,3]dioxole-6,3'-indol]-5(7H)-one [A33]:

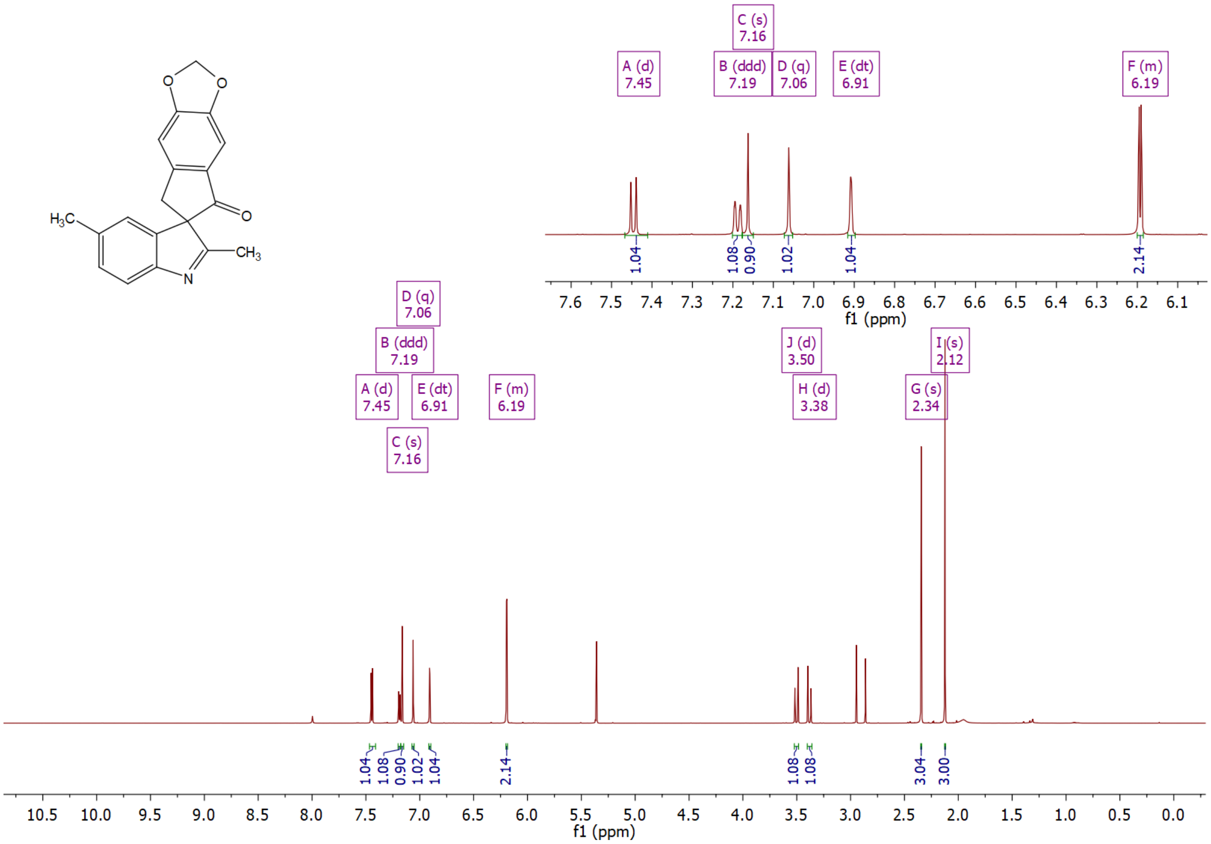

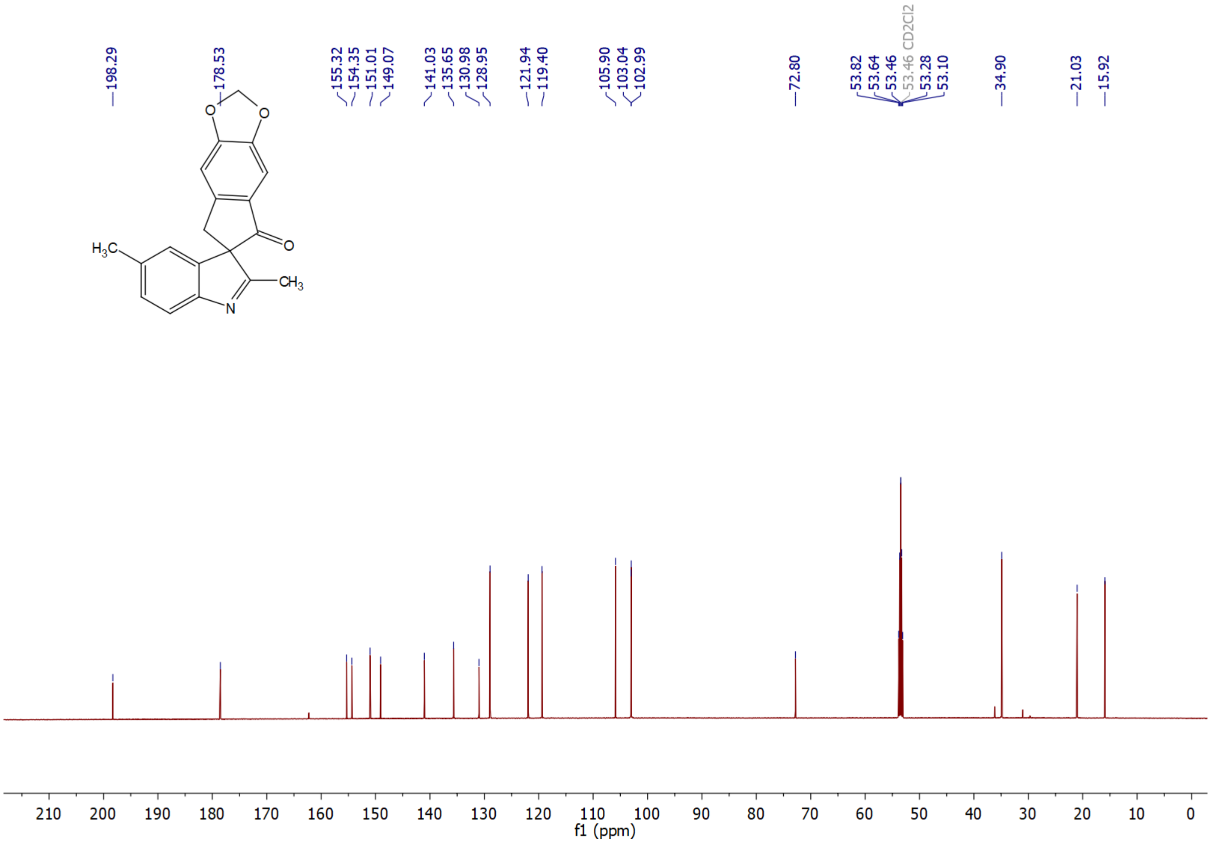

5'-methoxy-2'-methylspiro[indeno[5,6-d][1,3]dioxole-6,3'-indol]-5(7H)-one **[A34]**:

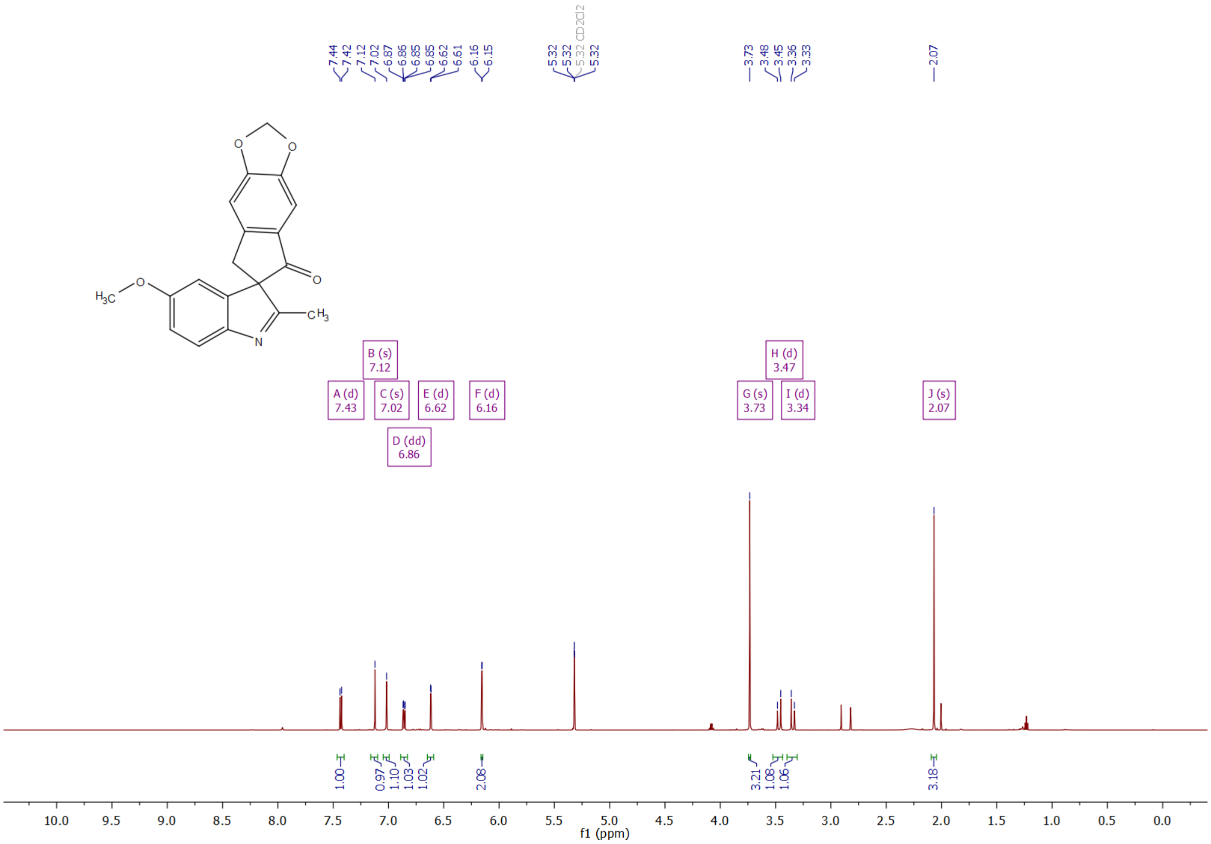

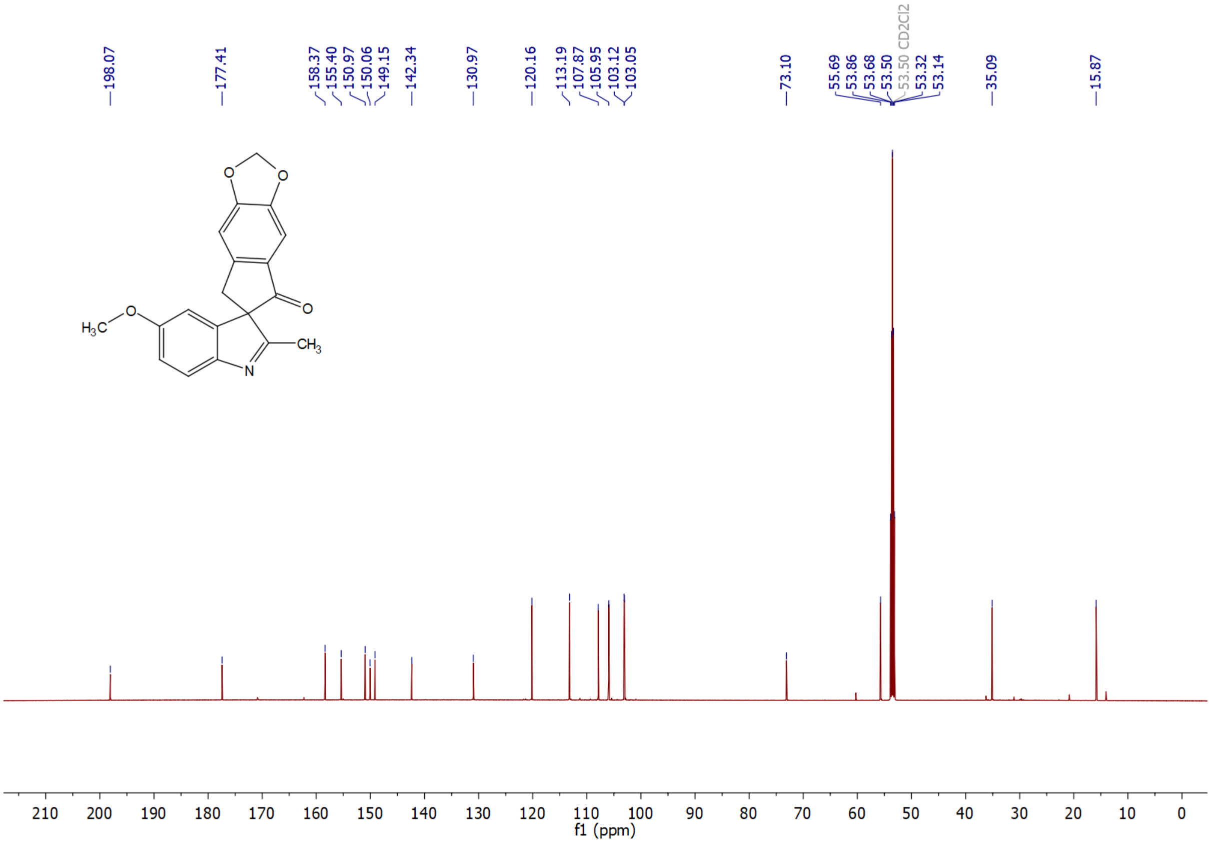

5'-fluoro-2'-methylspiro[indeno[4,5-d][1,3]dioxole-7,3'-indol]-6(8H)-one [A35]:

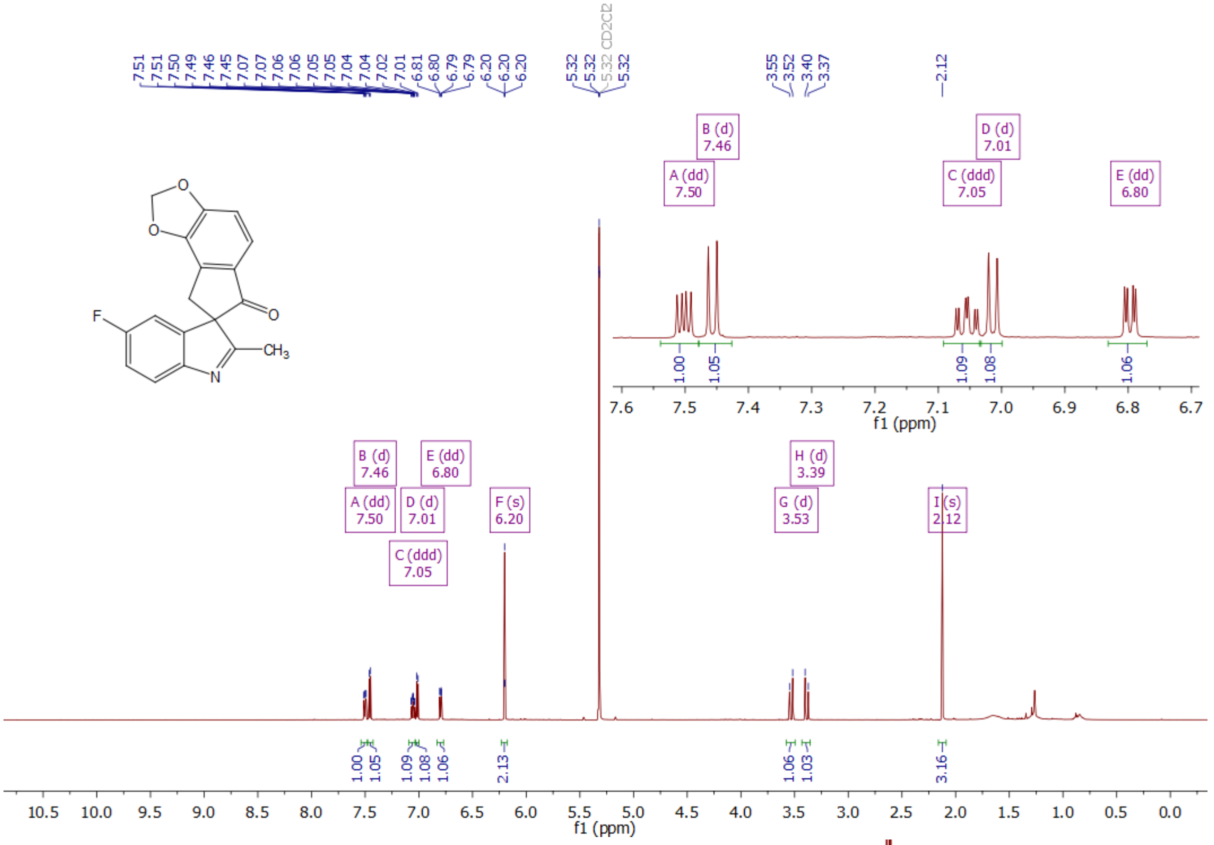

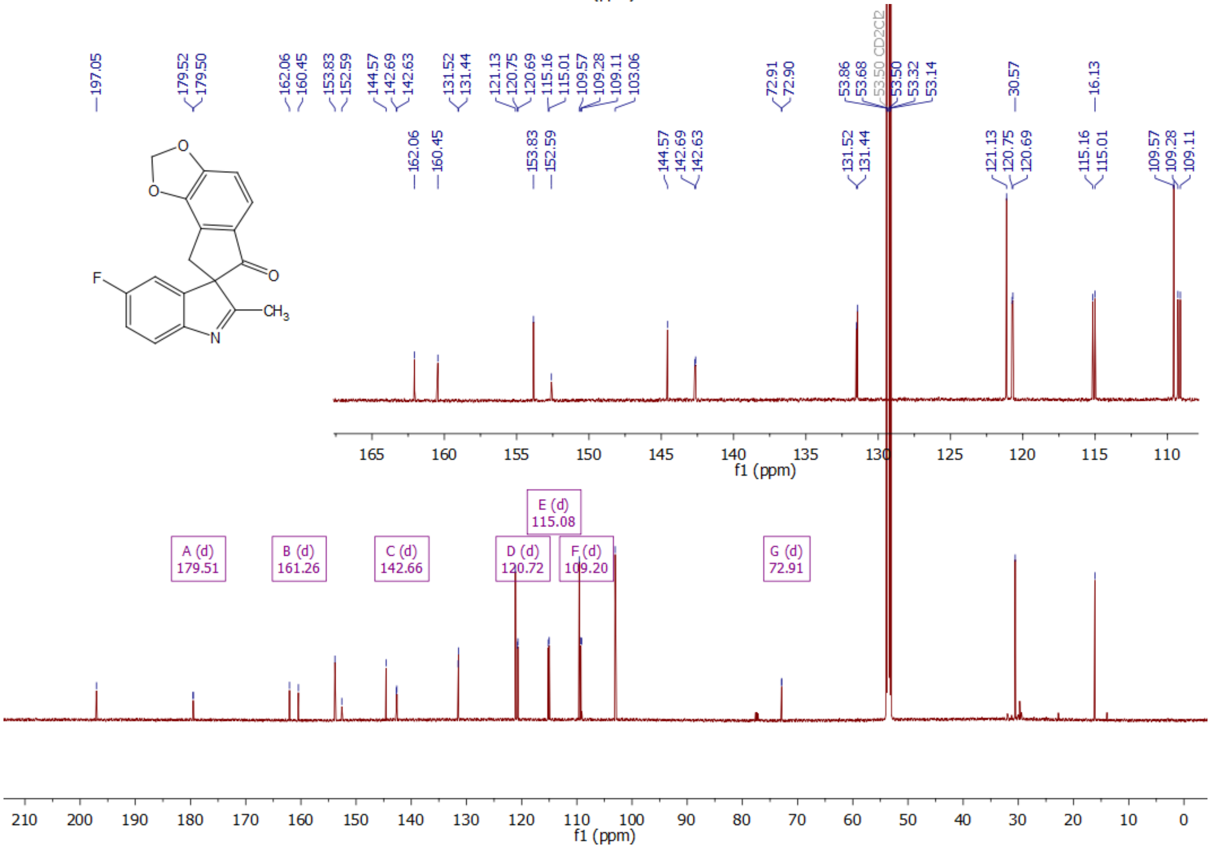

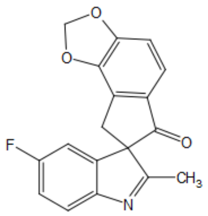
F
O
O
O
N
CH3

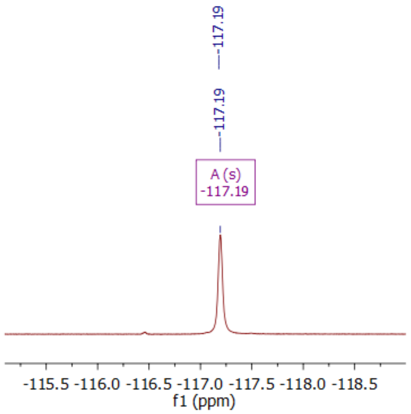
-117.19
-117.19
A (s)
-117.19
-115.5 -116.0 -116.5 -117.0 -117.5 -118.0 -118.5
f1 (ppm)

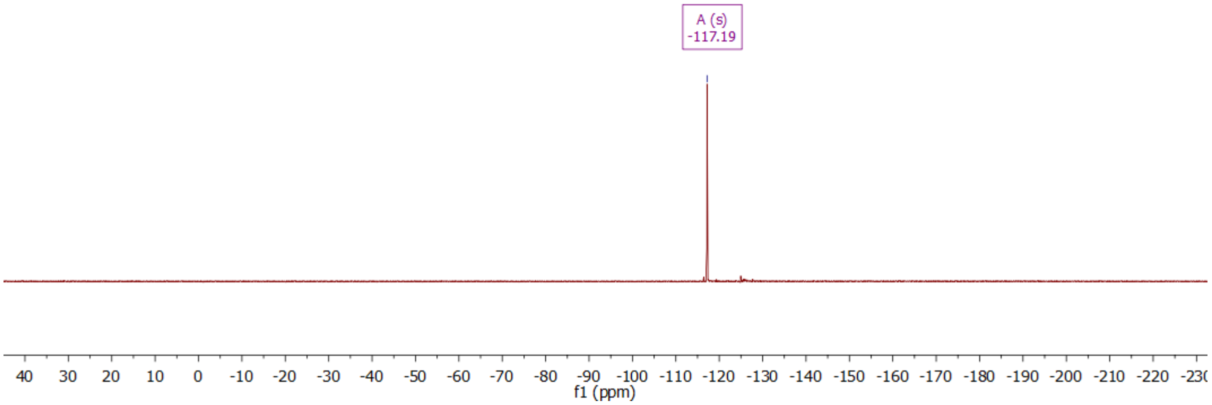
A (s)
-117.19
40 30 20 10 0 -10 -20 -30 -40 -50 -60 -70 -80 -90 -100 -110 -120 -130 -140 -150 -160 -170 -180 -190 -200 -210 -220 -230
f1 (ppm)

2'-phenylspiro[indene-2,3'-indol]-1(3H)-one [**A36**]:

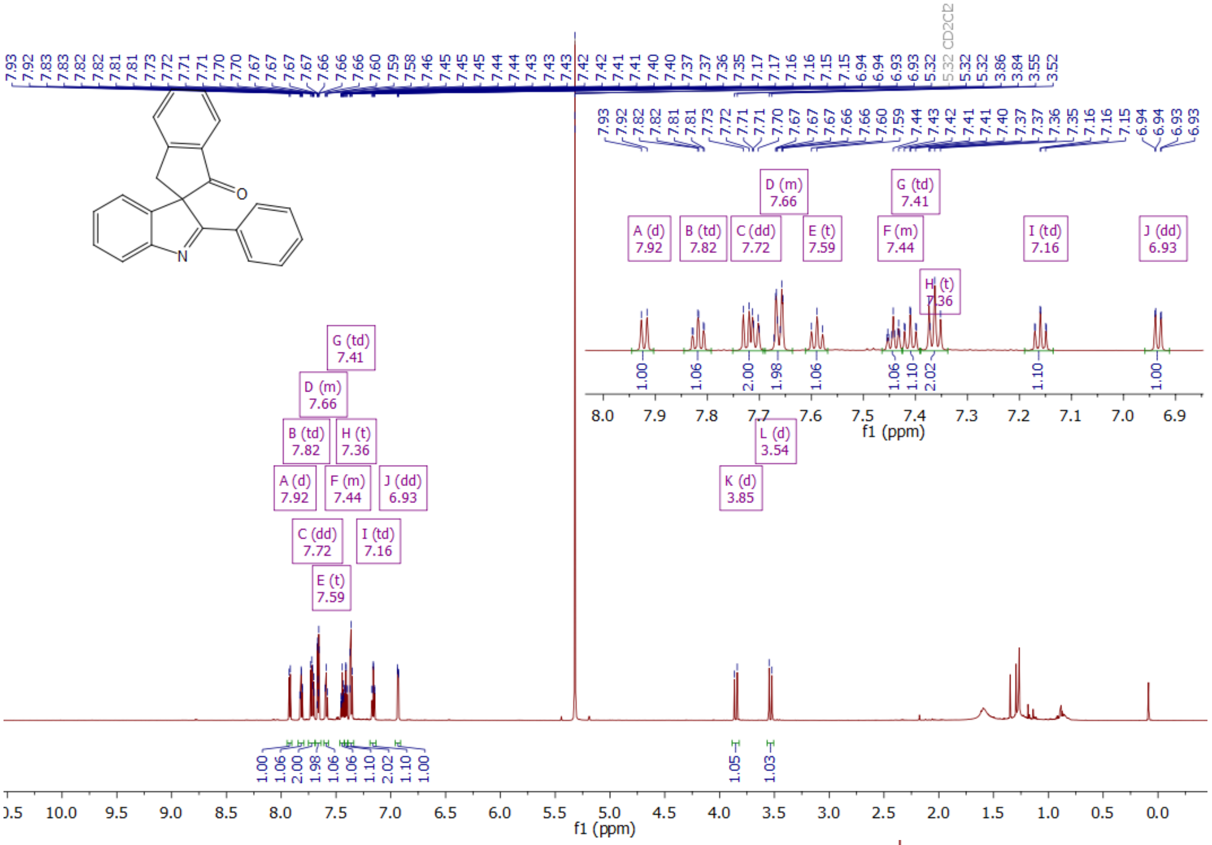

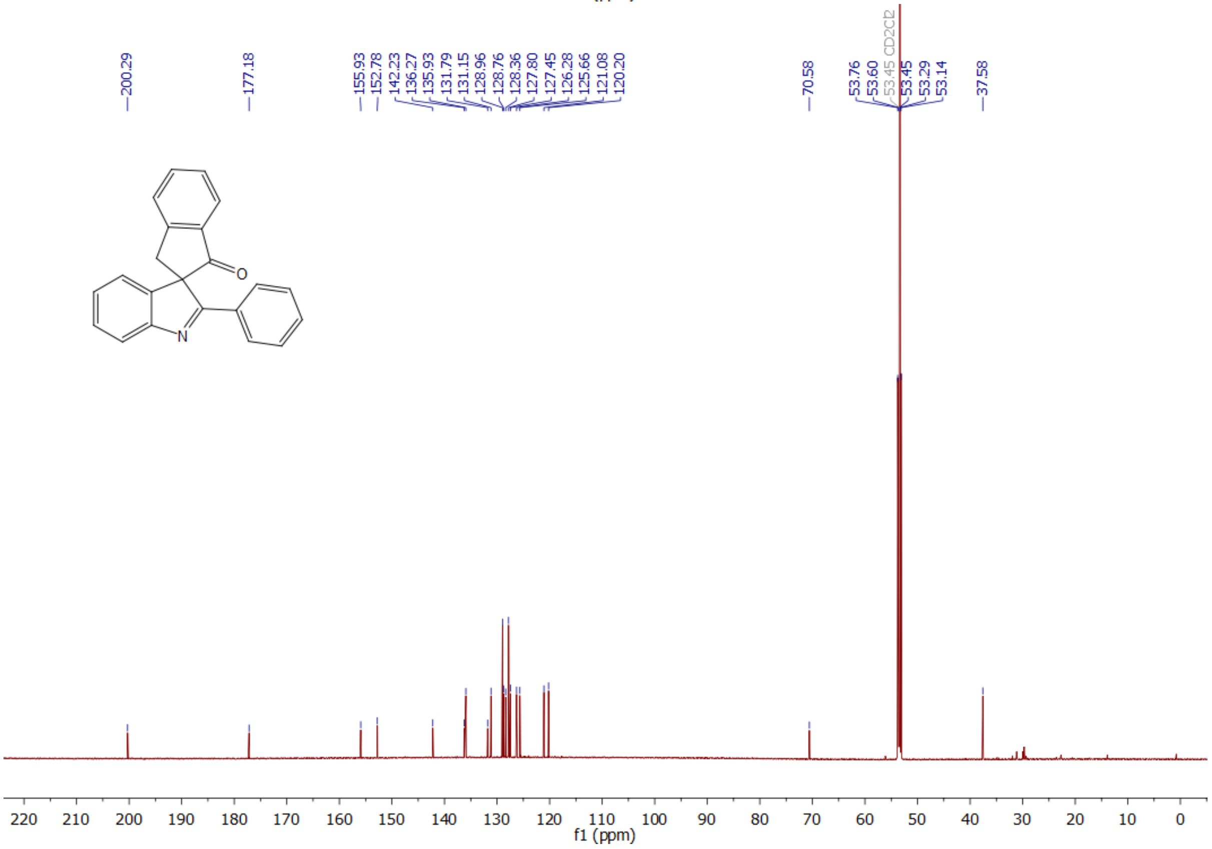

6-fluoro-2'-phenylspiro[indene-2,3'-indol]-1(3H)-one [**A37**]:

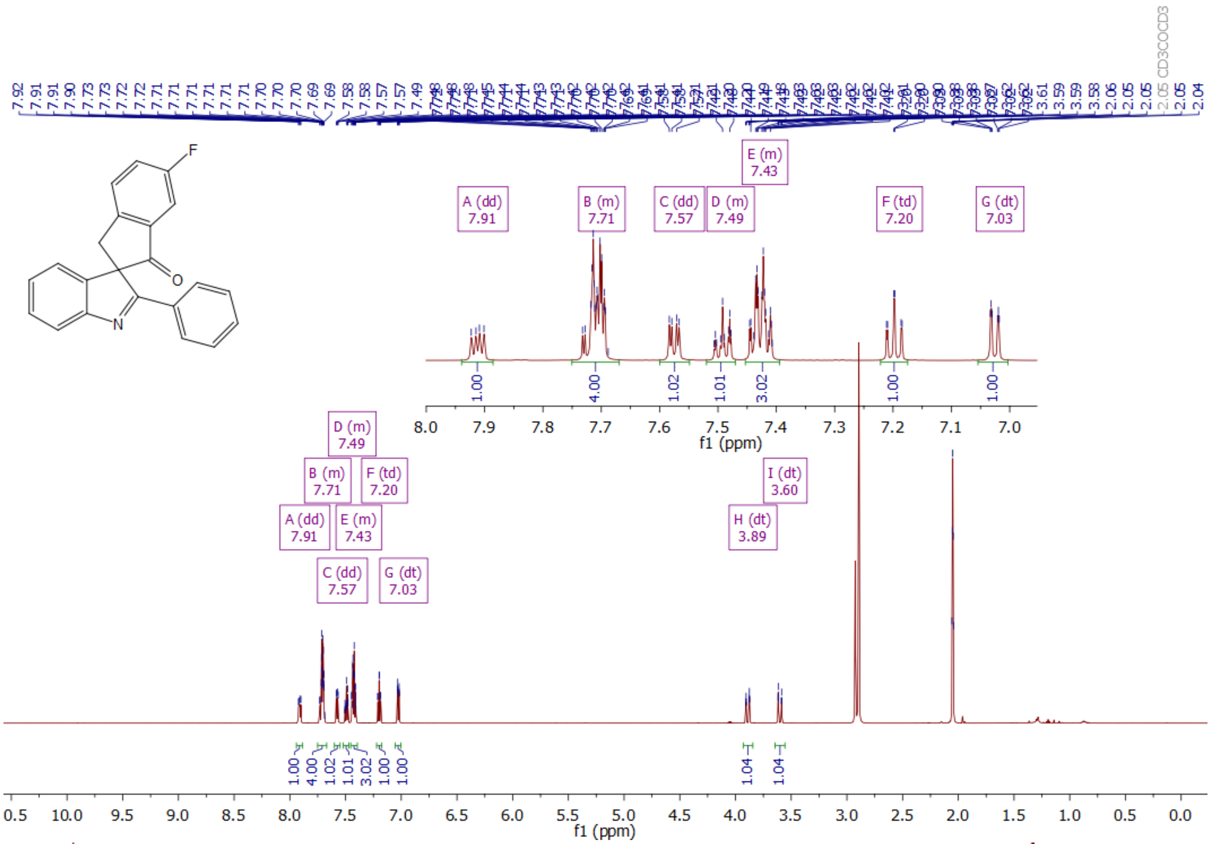

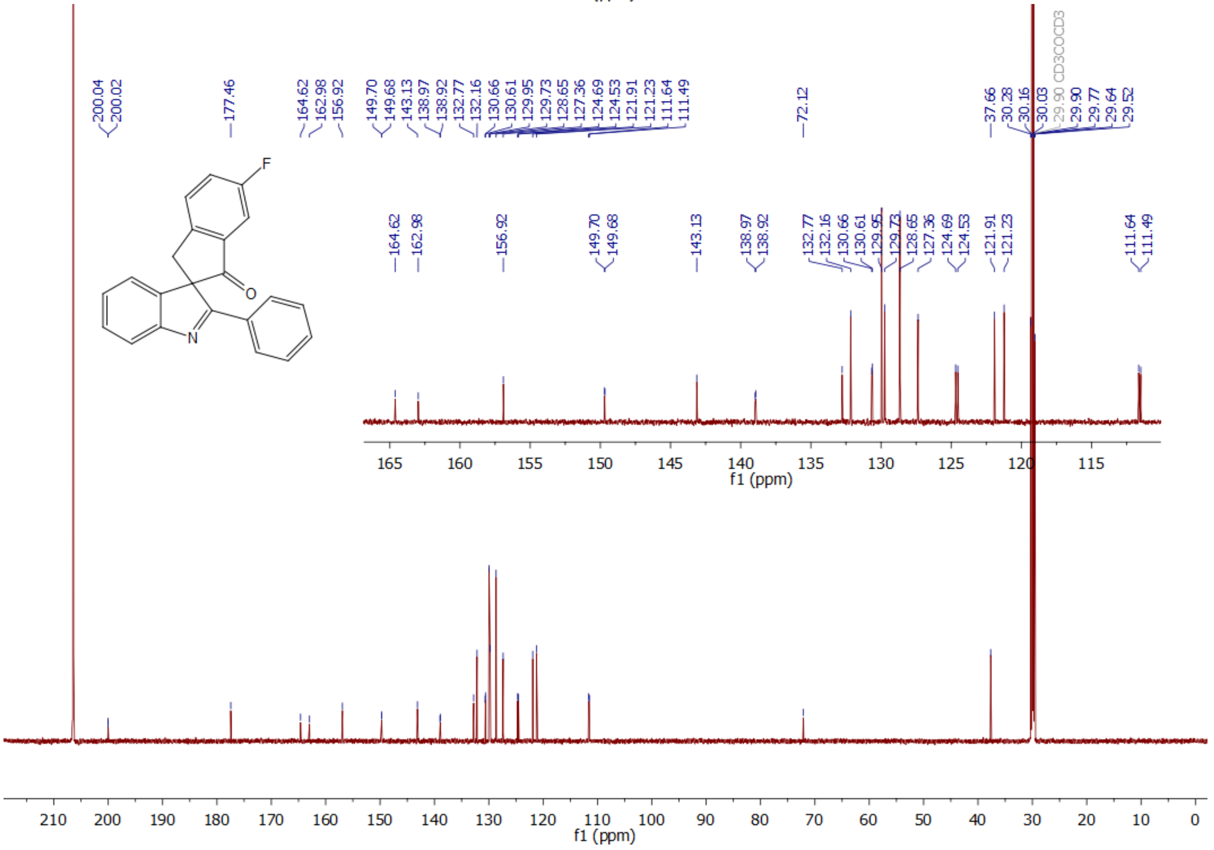

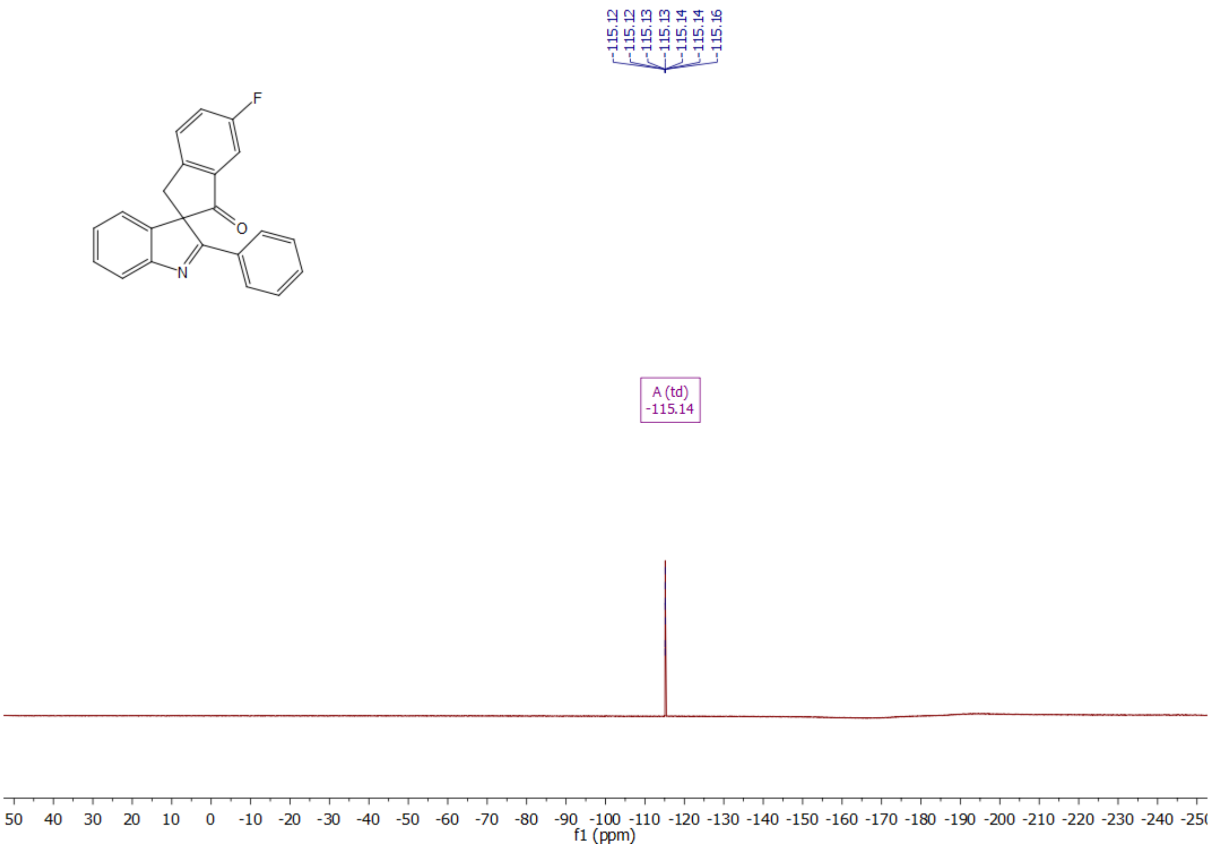

-115.12
-115.12
-115.13
-115.13
-115.14
-115.14
-115.16
F
O
N
A (td)
-115.14
50 40 30 20 10 0 -10 -20 -30 -40 -50 -60 -70 -80 -90 -100 -110 -120 -130 -140 -150 -160 -170 -180 -190 -200 -210 -220 -230 -240
f1 (ppm)

6-chloro-2'-phenylspiro[indene-2,3'-indol]-1(3H)-one [**A38**]:

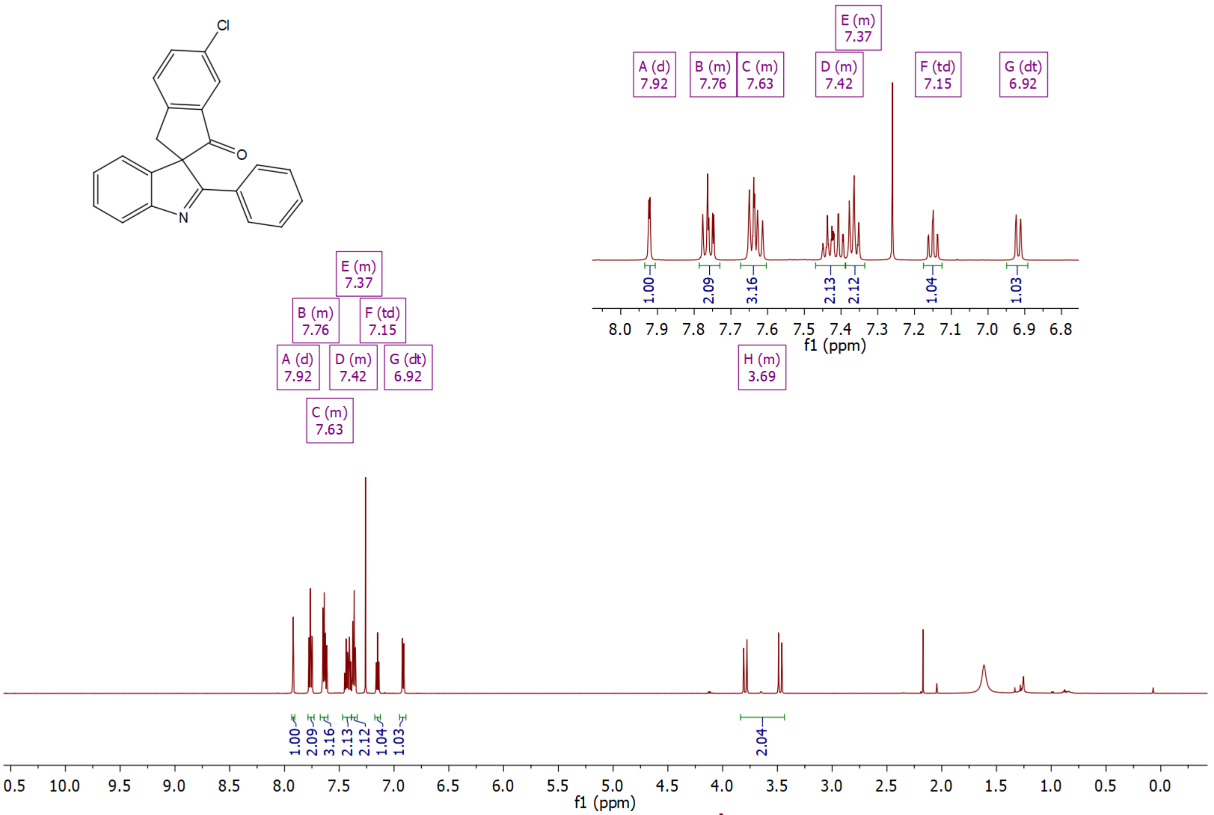

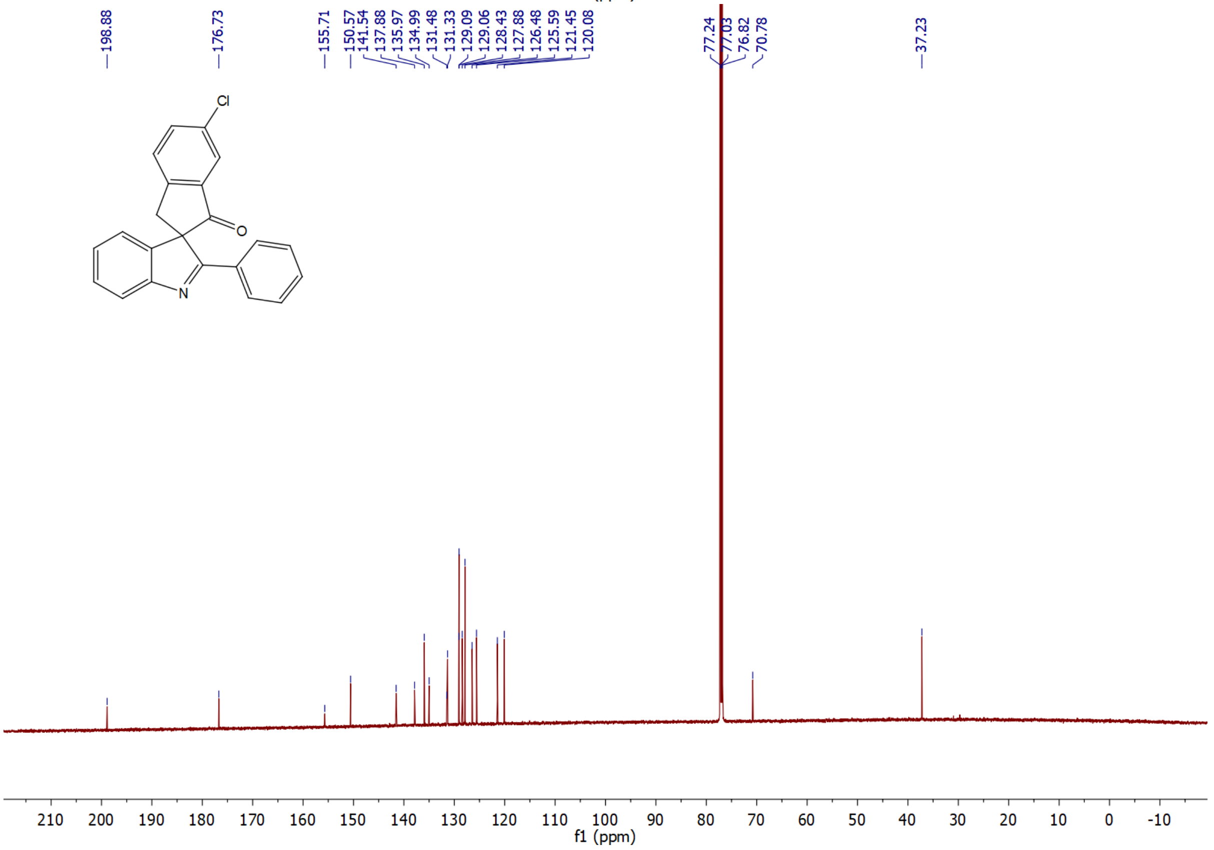

6-methyl-2'-phenylspiro[indene-2,3'-indol]-1(3H)-one [**A39**]:

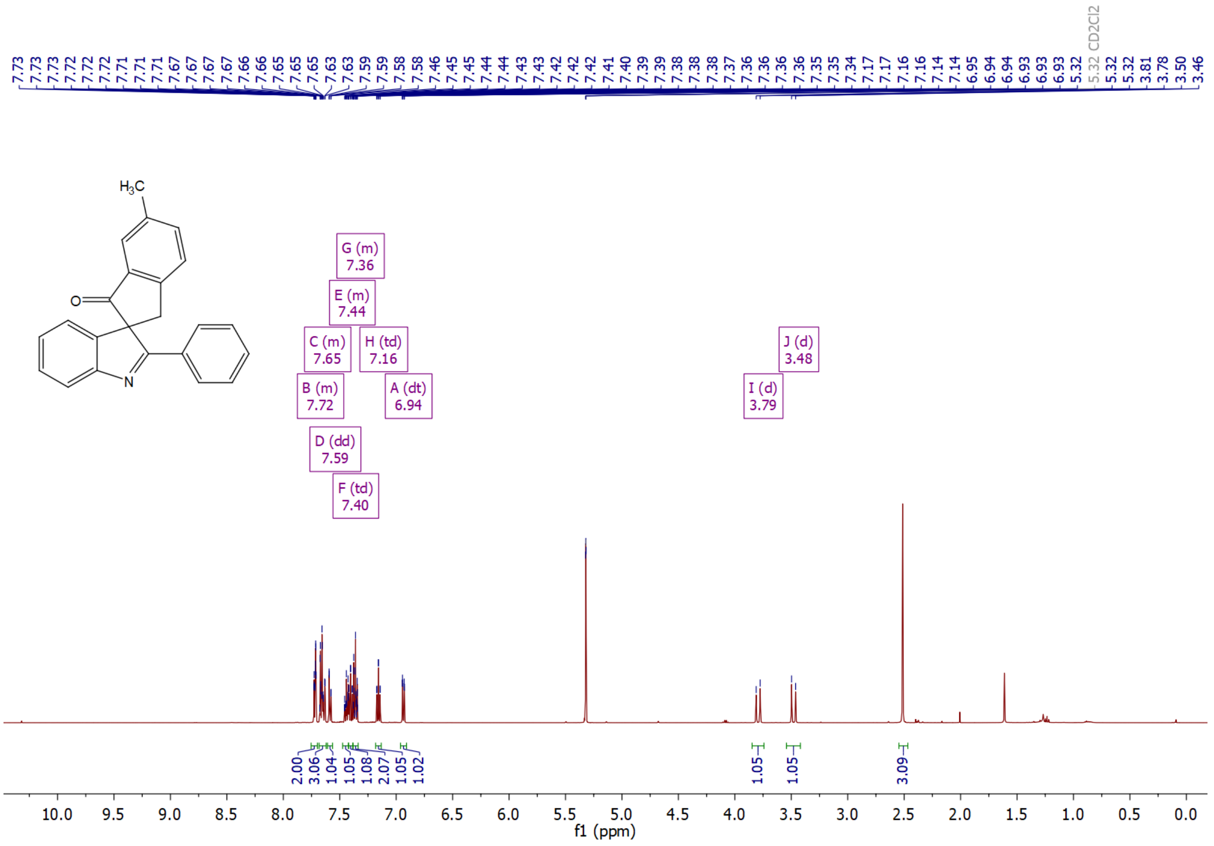

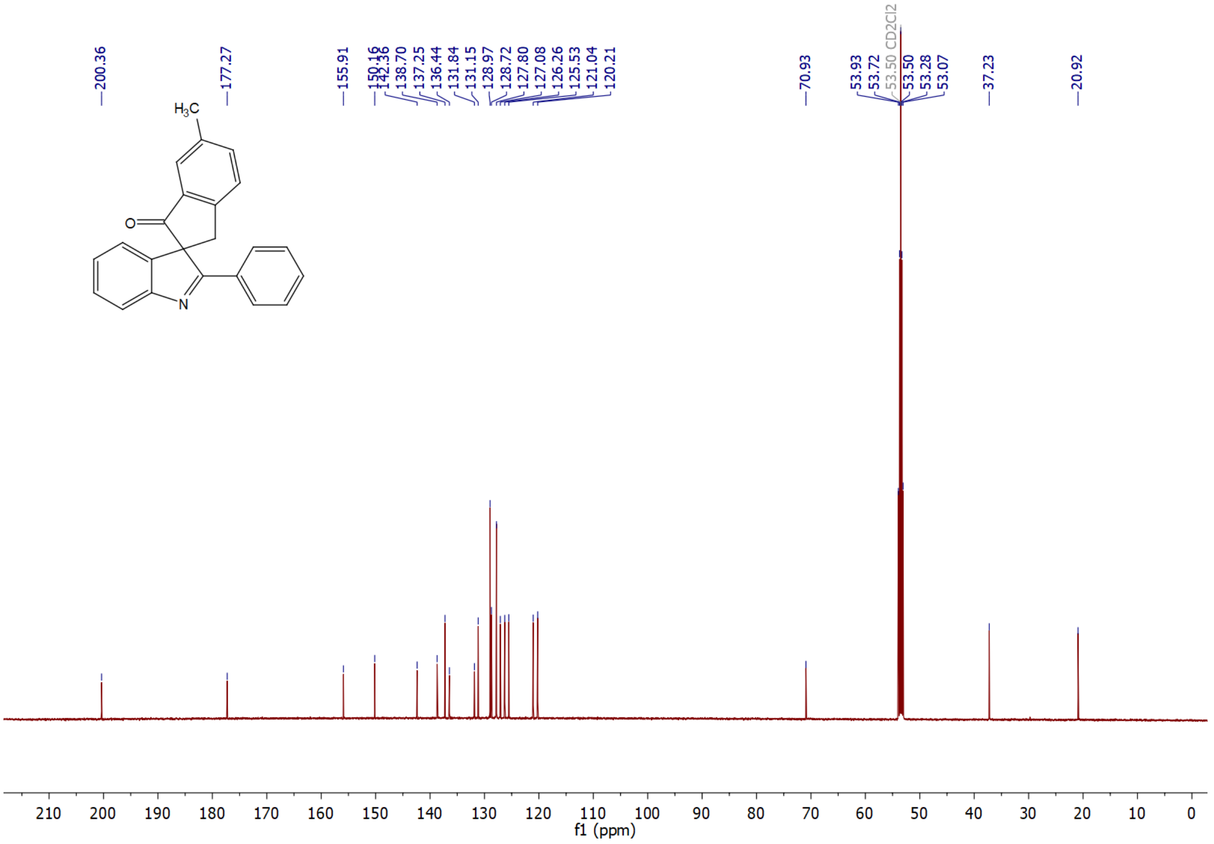

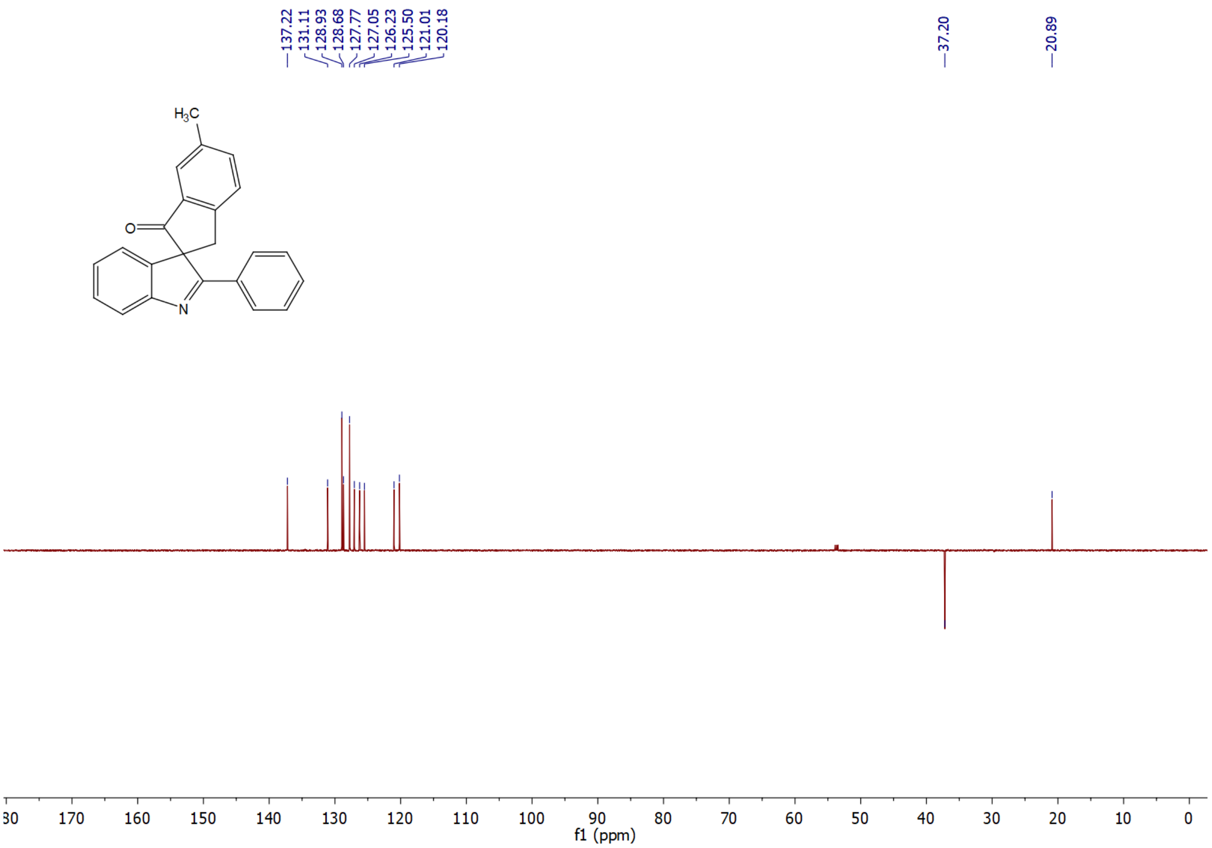

137.22
131.11
128.93
128.68
127.77
127.05
126.23
125.50
121.01
120.18
37.20
20.89
H3C
O
N
180
170
160
150
140
130
120
110
100
90
80
70
60
50
40
30
20
10
0
f1 (ppm)

5-fluoro-2'-phenylspiro[indene-2,3'-indol]-1(3H)-one [**A40**]:

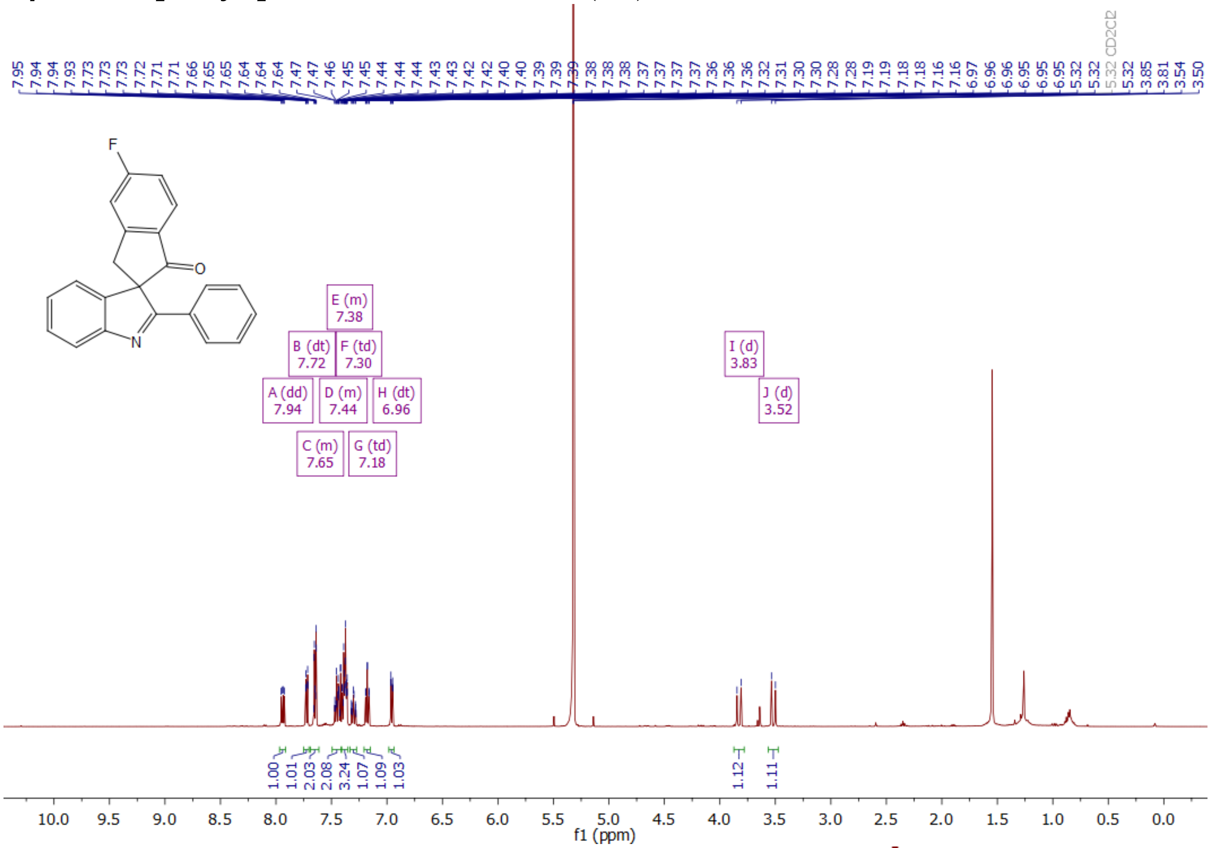

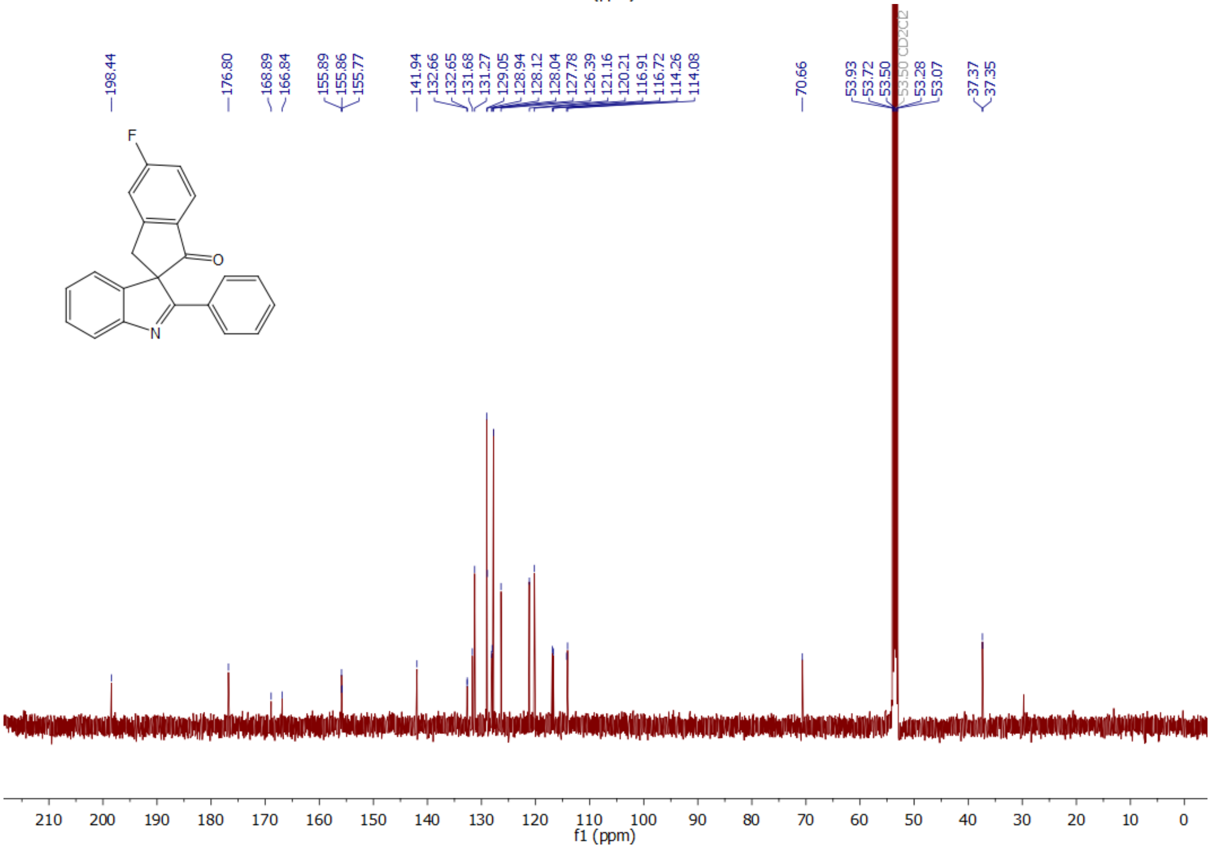

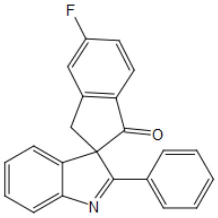

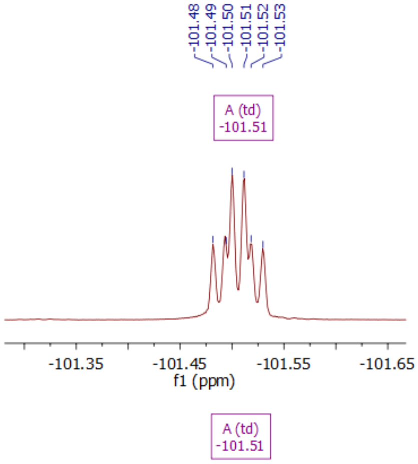

-101.48
-101.49
-101.50
-101.51
-101.52
-101.53
A (td)
-101.51
-101.35
-101.45
-101.55
-101.65
f1 (ppm)
A (td)
-101.51

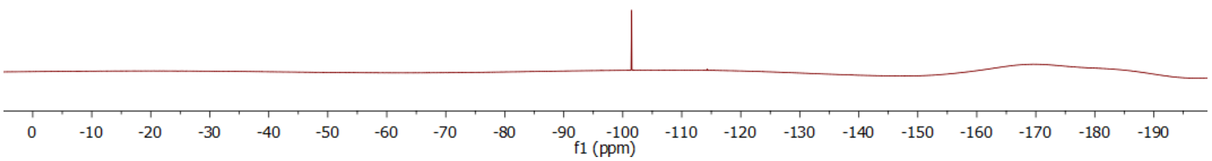

0
-10
-20
-30
-40
-50
-60
-70
-80
-90
-100
-110
-120
-130
-140
-150
-160
-170
-180
-190
f1 (ppm)

4-fluoro-2'-phenylspiro[indene-2,3'-indol]-1(3H)-one [**A41**]:

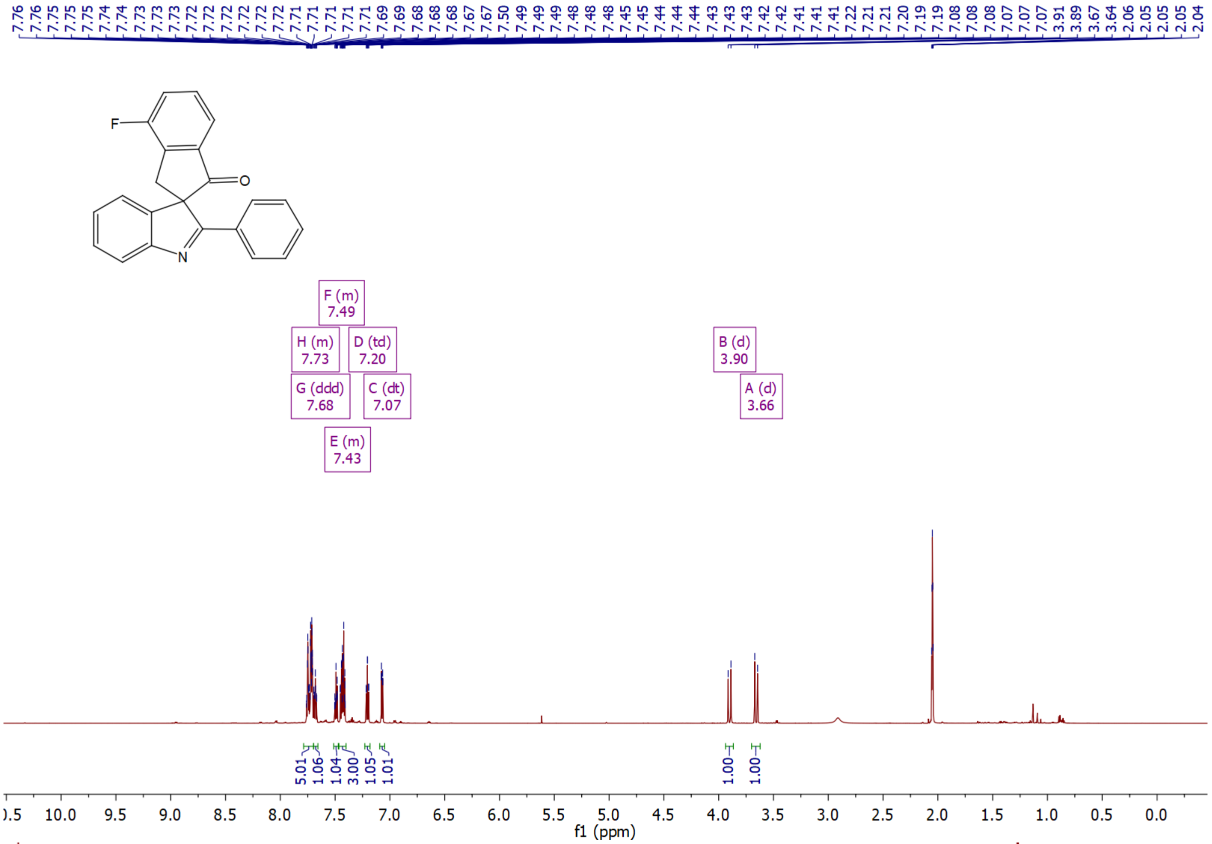

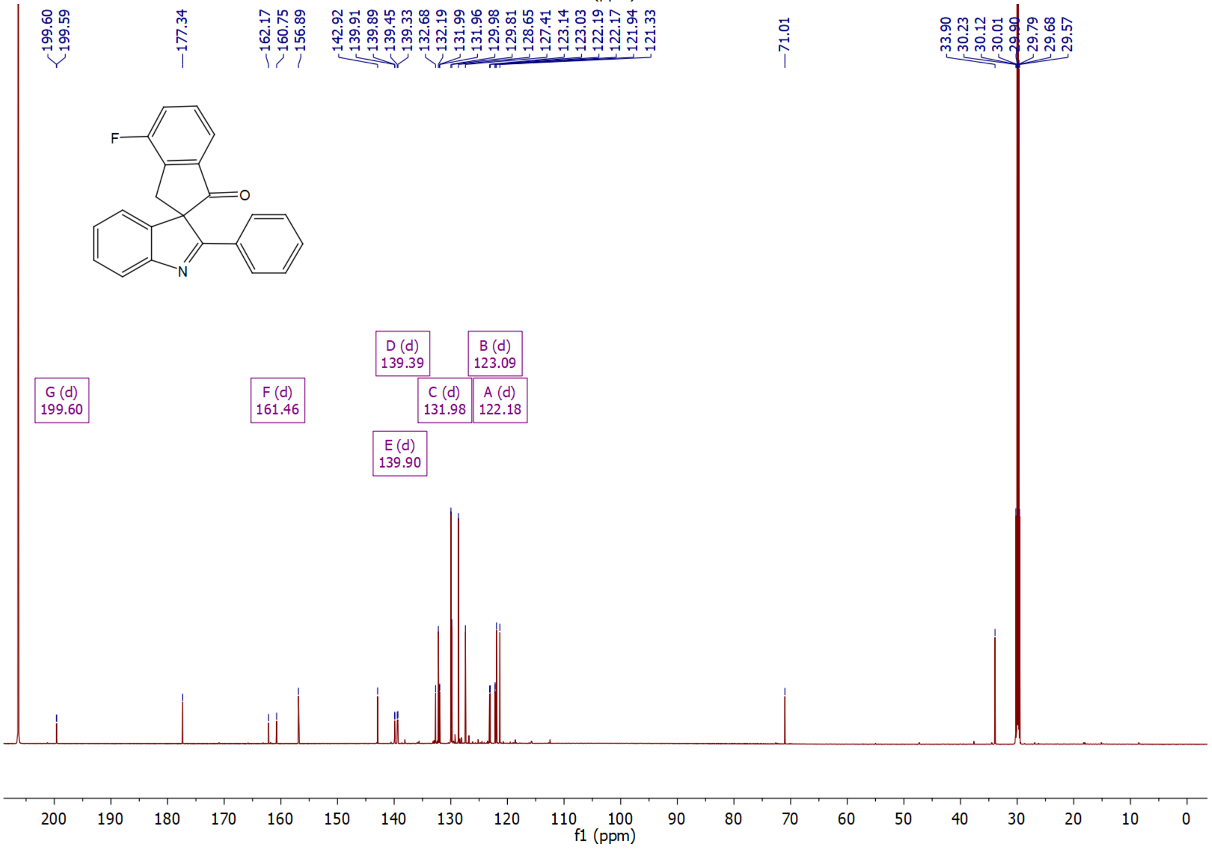

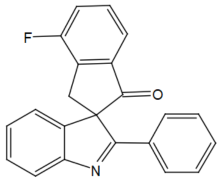
F
O
N

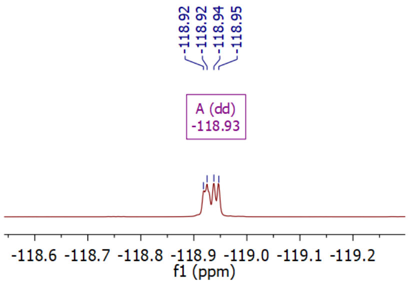
-118.92
-118.92
-118.94
-118.95
A (dd)
-118.93
-118.6 -118.7 -118.8 -118.9 -119.0 -119.1 -119.2
f1 (ppm)

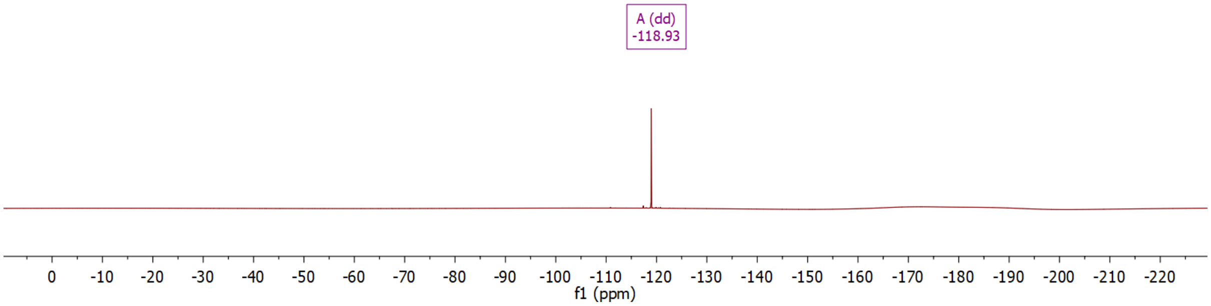
A (dd)
-118.93
0 -10 -20 -30 -40 -50 -60 -70 -80 -90 -100 -110 -120 -130 -140 -150 -160 -170 -180 -190 -200 -210 -220
f1 (ppm)

CLASS-B

2'-methylspiro[indene-2,3'-indolin]-1(3H)-one [**B1**]:

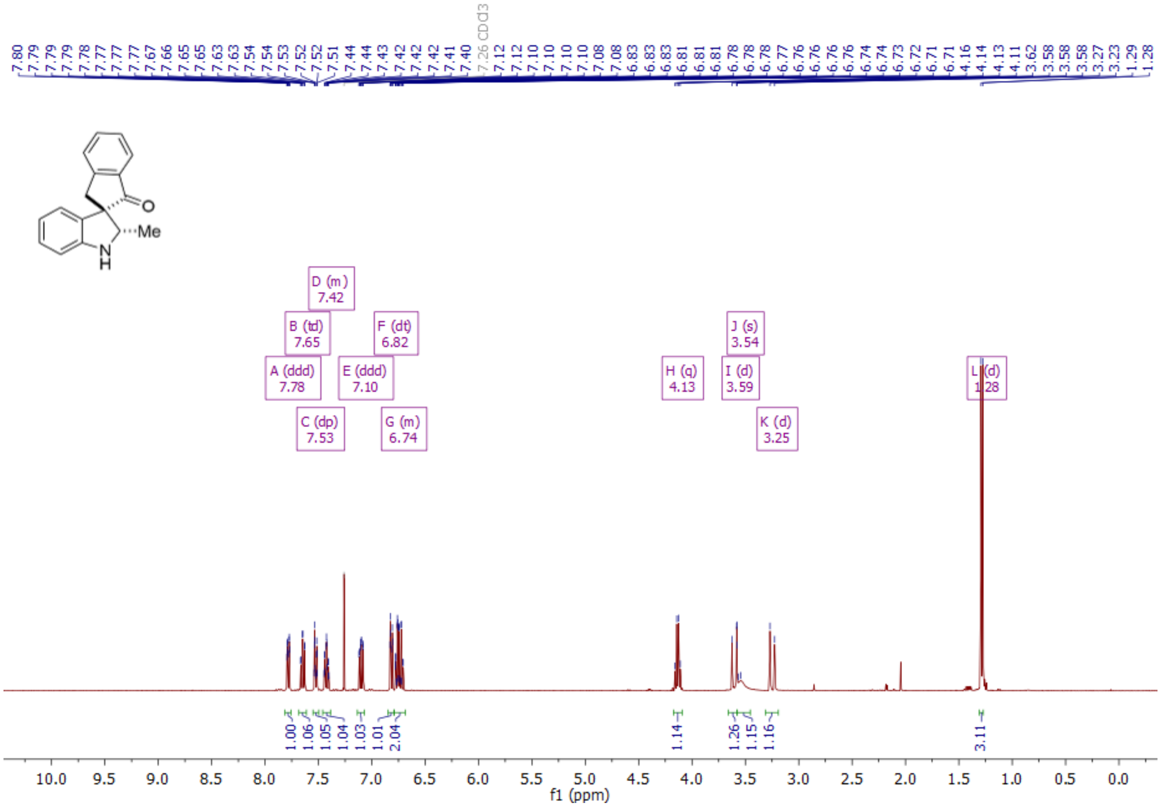

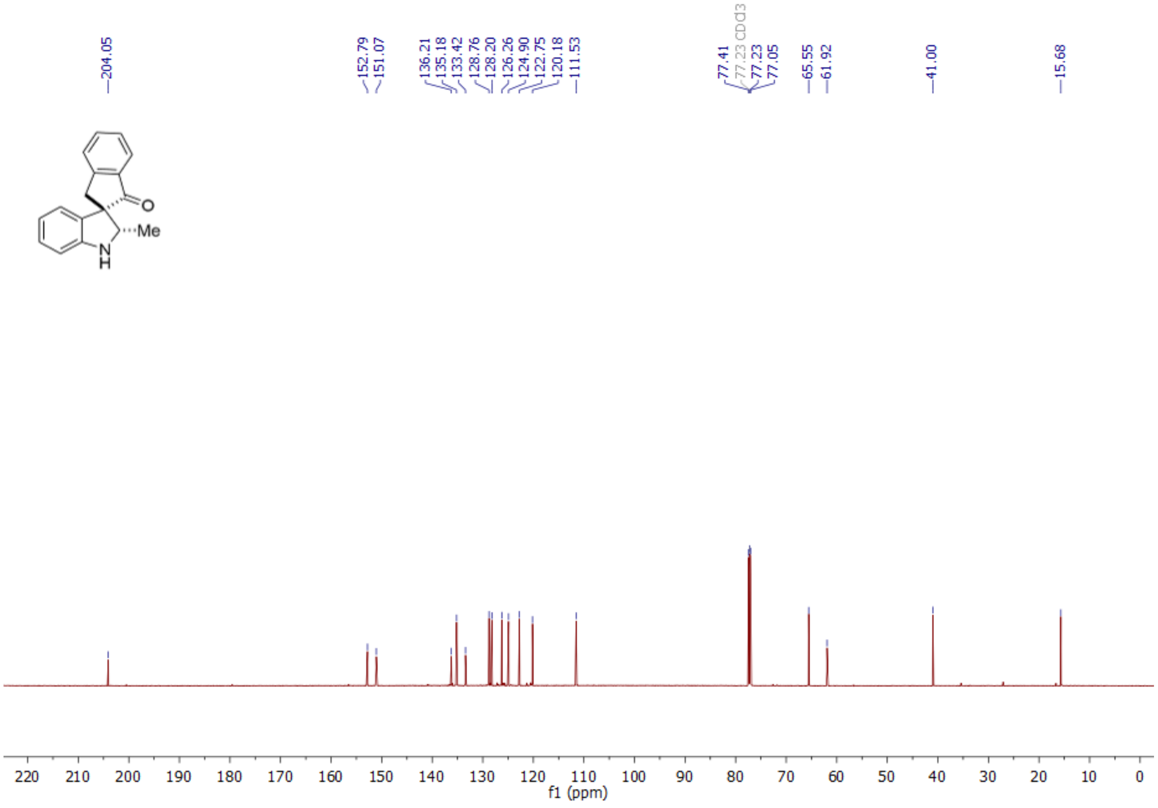

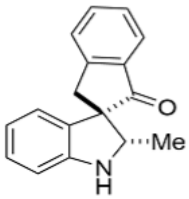

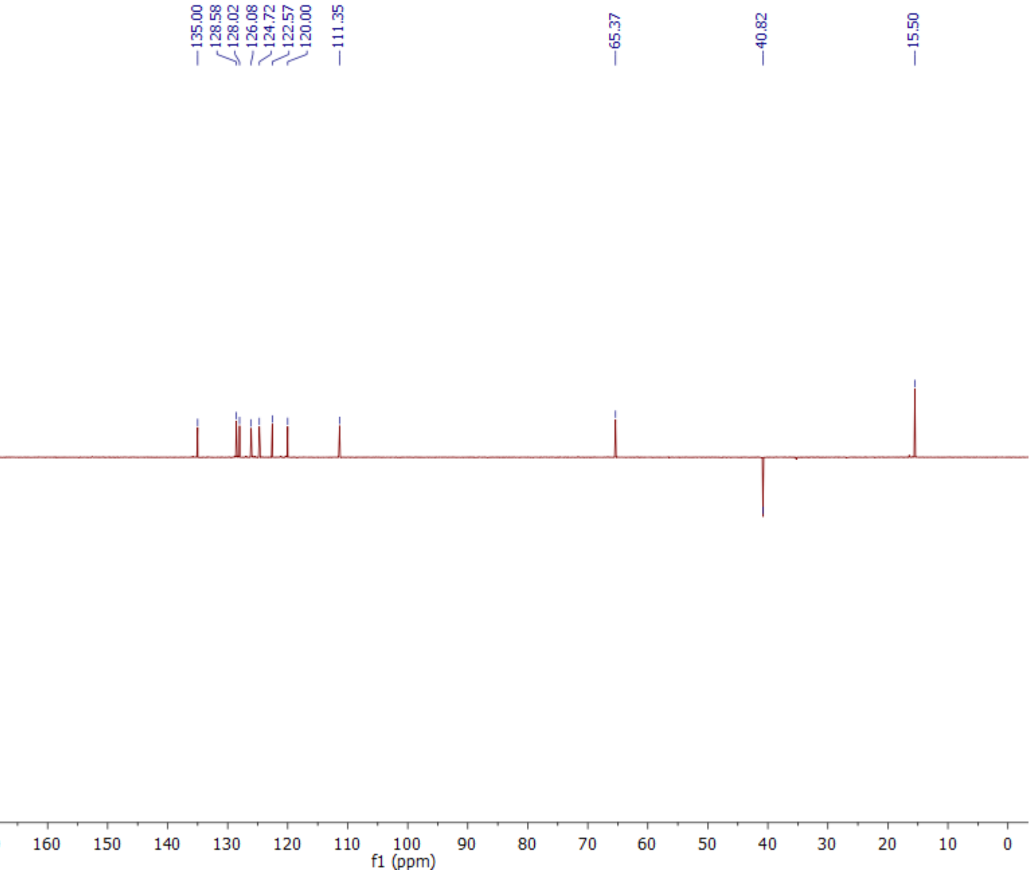

135.00
128.58
128.02
126.08
124.72
122.57
120.00
111.35
65.37
40.82
15.50
200
190
180
170
160
150
140
130
120
110
100
90
80
70
60
50
40
30
20
10
0
f1 (ppm)

***5-fluoro-2'-methylspiro[indene-2,3'-indolin]-1(3H)-one* [B2]:**

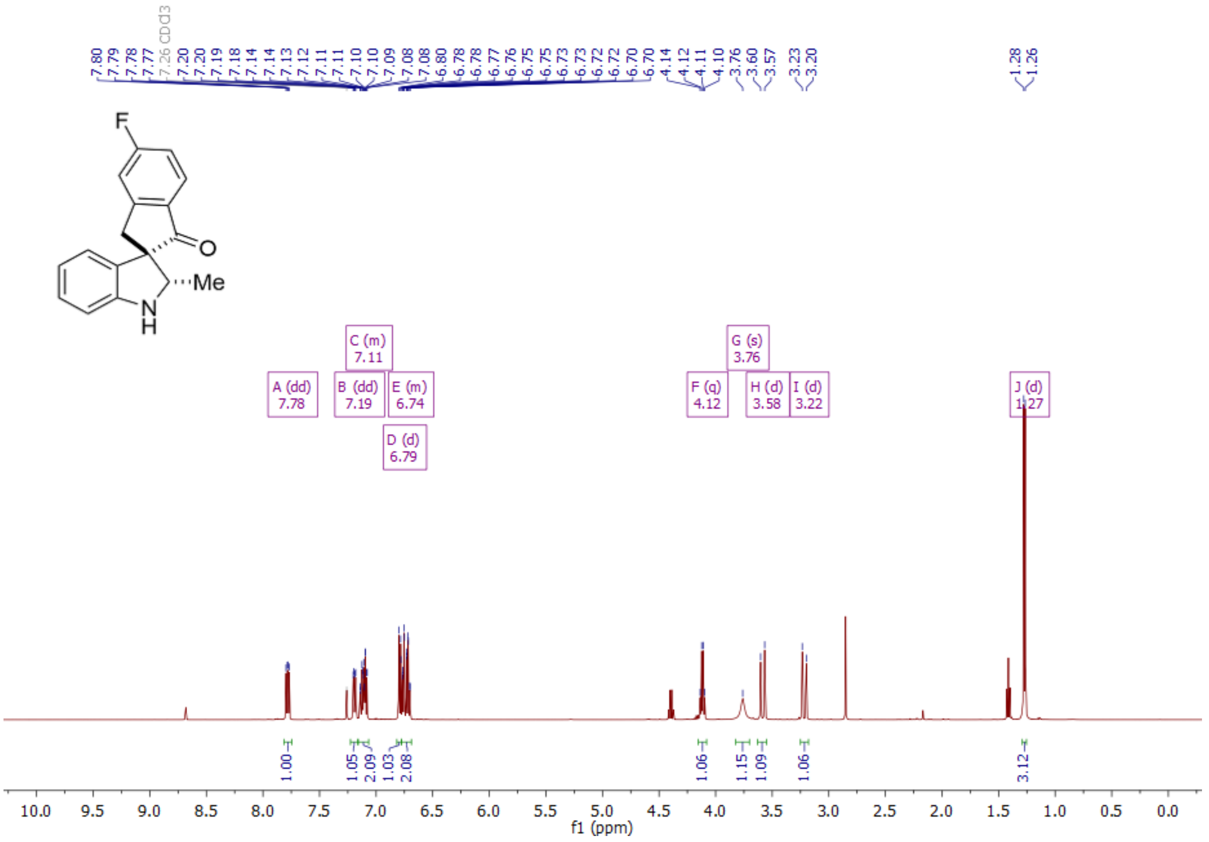

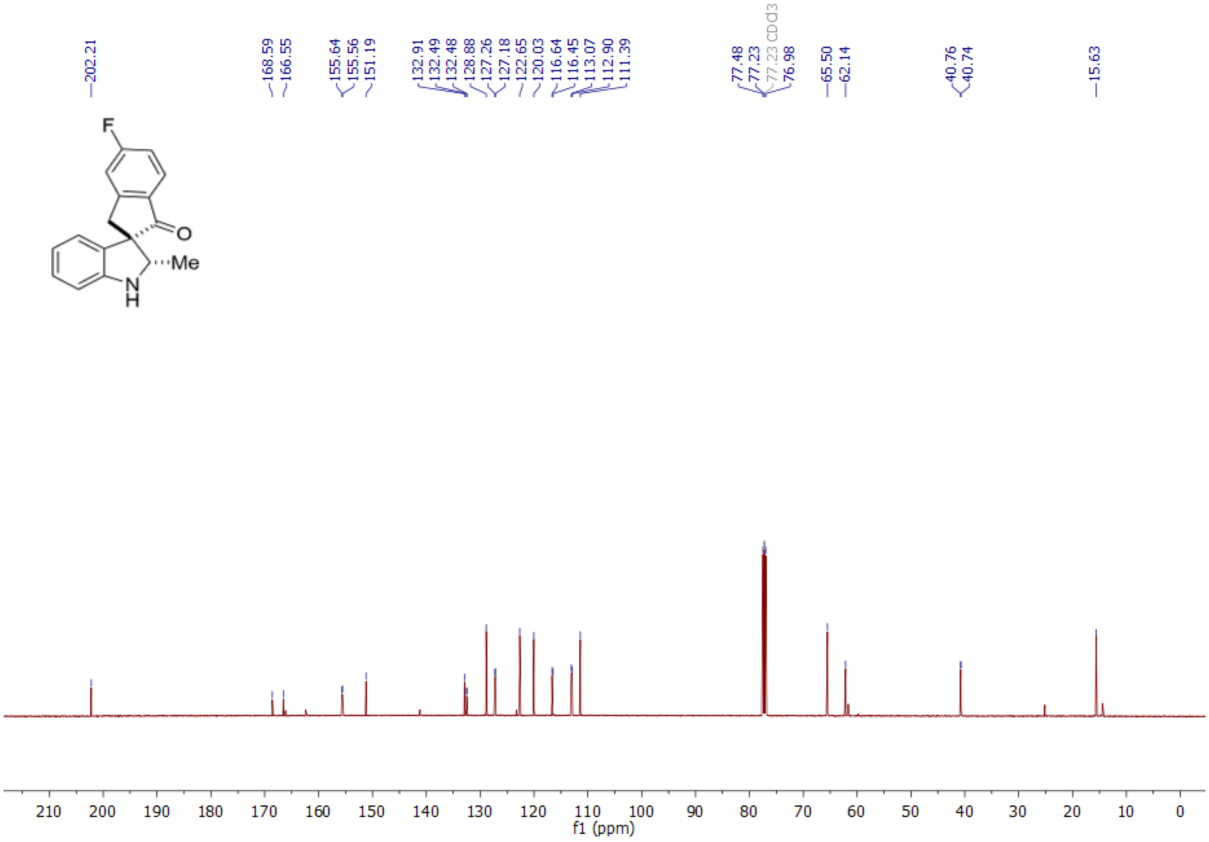

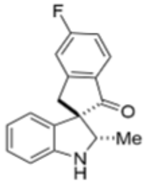

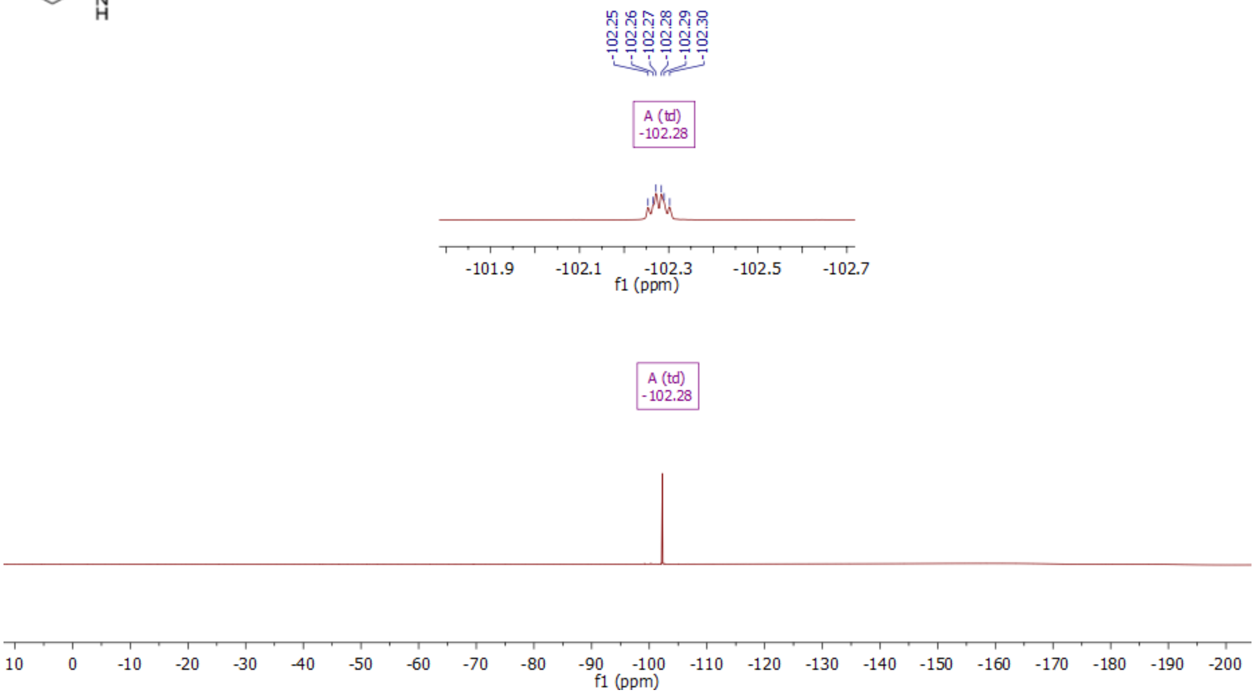
-102.25
-102.26
-102.27
-102.28
-102.29
-102.30
A (td)
-102.28
-101.9
-102.1
-102.3
-102.5
-102.7
f1 (ppm)
A (td)
-102.28
10
0
-10
-20
-30
-40
-50
-60
-70
-80
-90
-100
-110
-120
-130
-140
-150
-160
-170
-180
-190
-200
f1 (ppm)

5-methoxy-2'-methylspiro[indene-2,3'-indolin]-1(3H)-one [**B3**]:

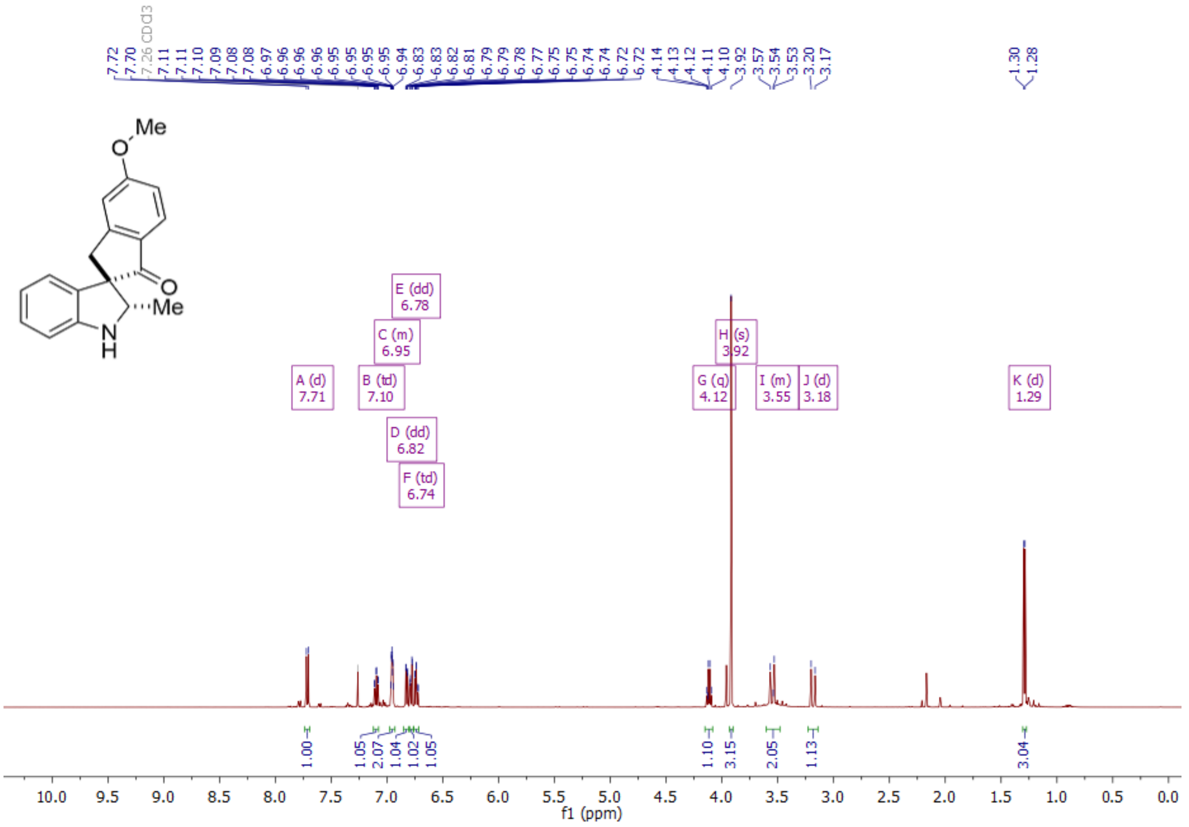

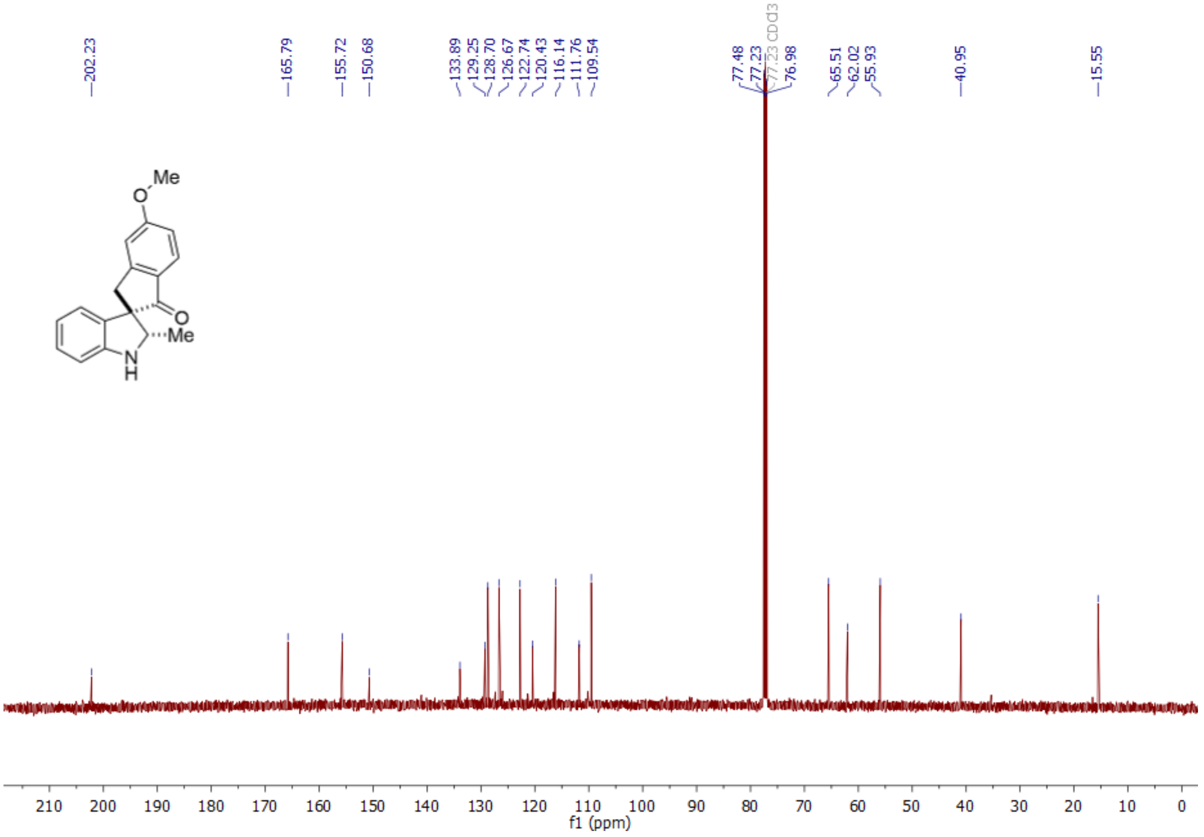

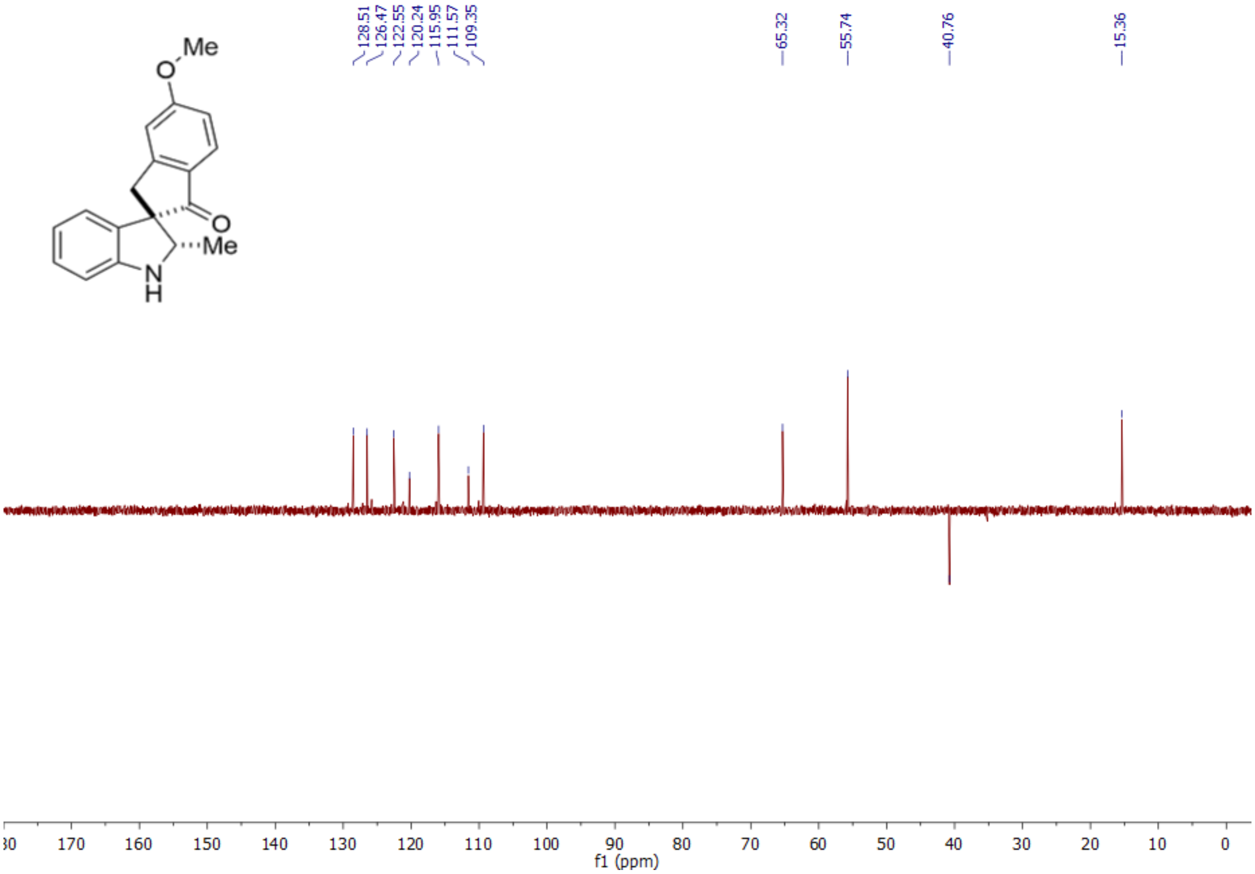

128.51
126.47
122.55
120.24
115.95
111.57
109.35
65.32
55.74
40.76
15.36
170
160
150
140
130
120
110
100
90
80
70
60
50
40
30
20
10
0
f1 (ppm)

4-fluoro-2'-methylspiro[indene-2,3'-indolin]-1(3H)-one [**B4**]:

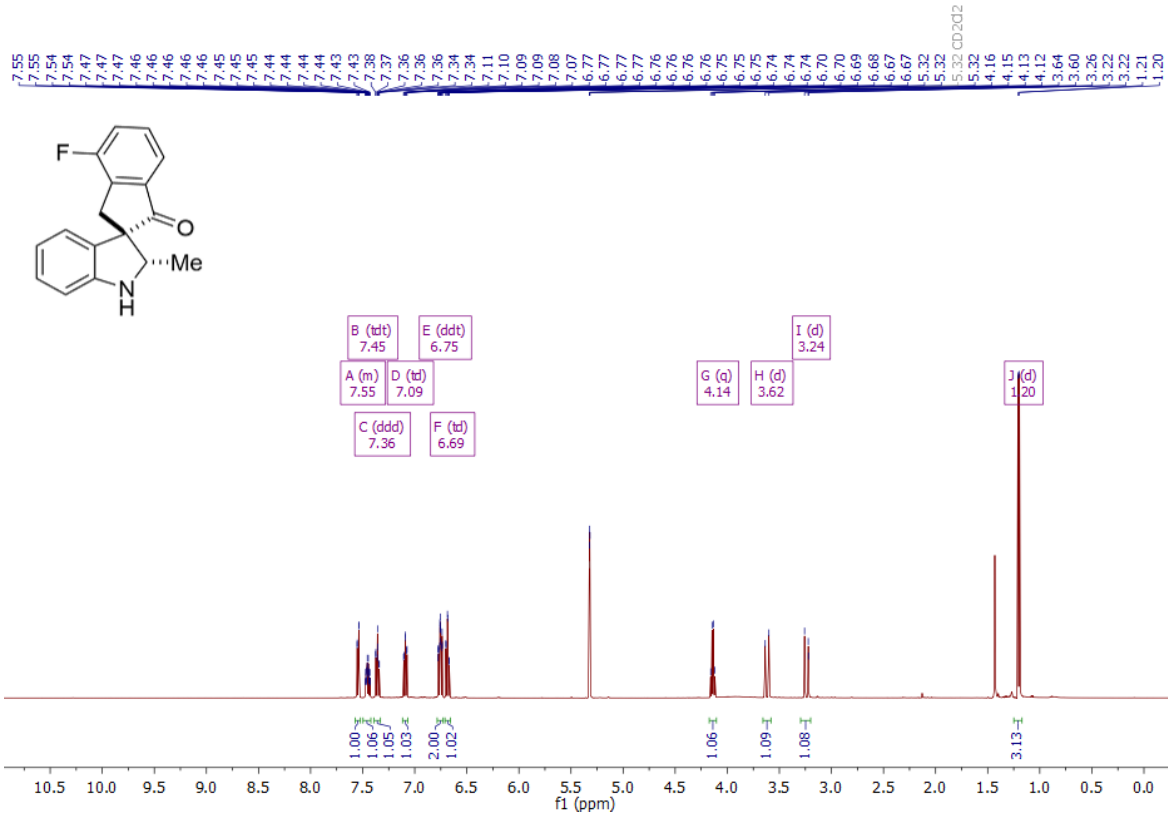

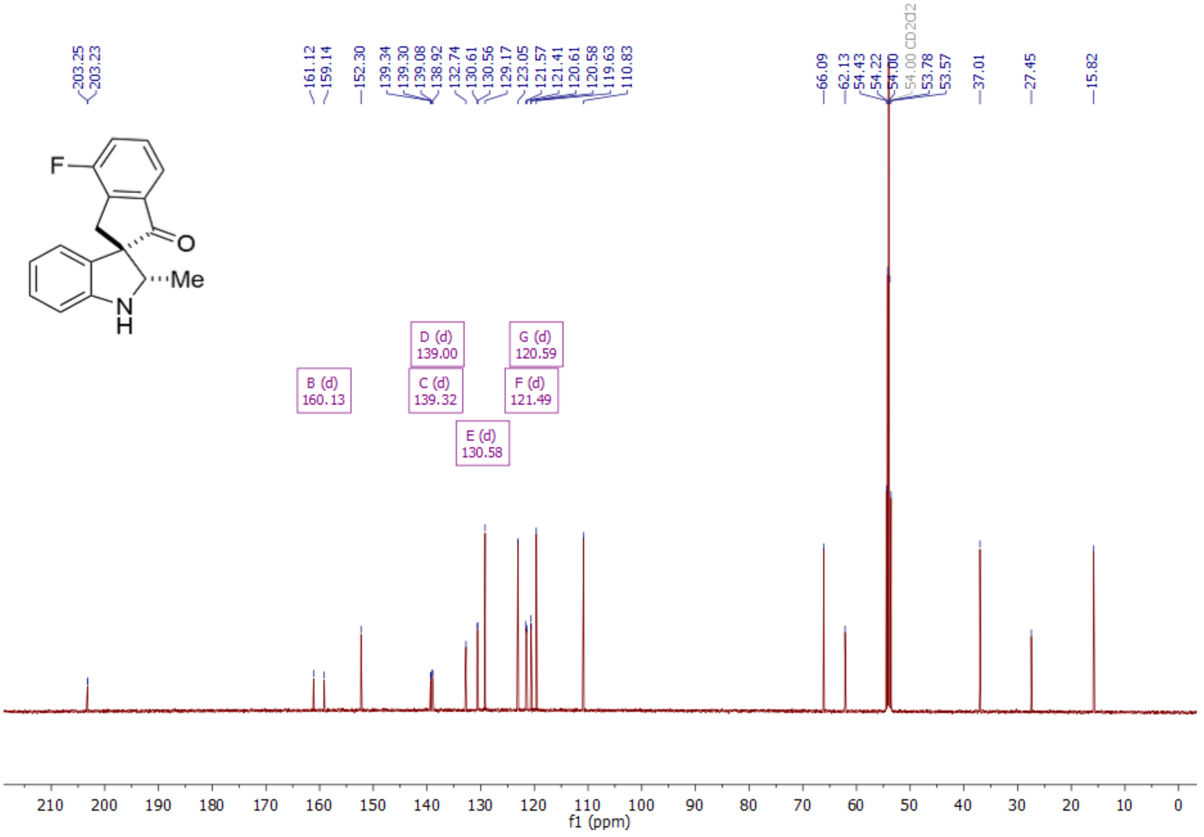

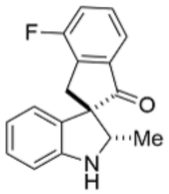
F
O
Me
N
H

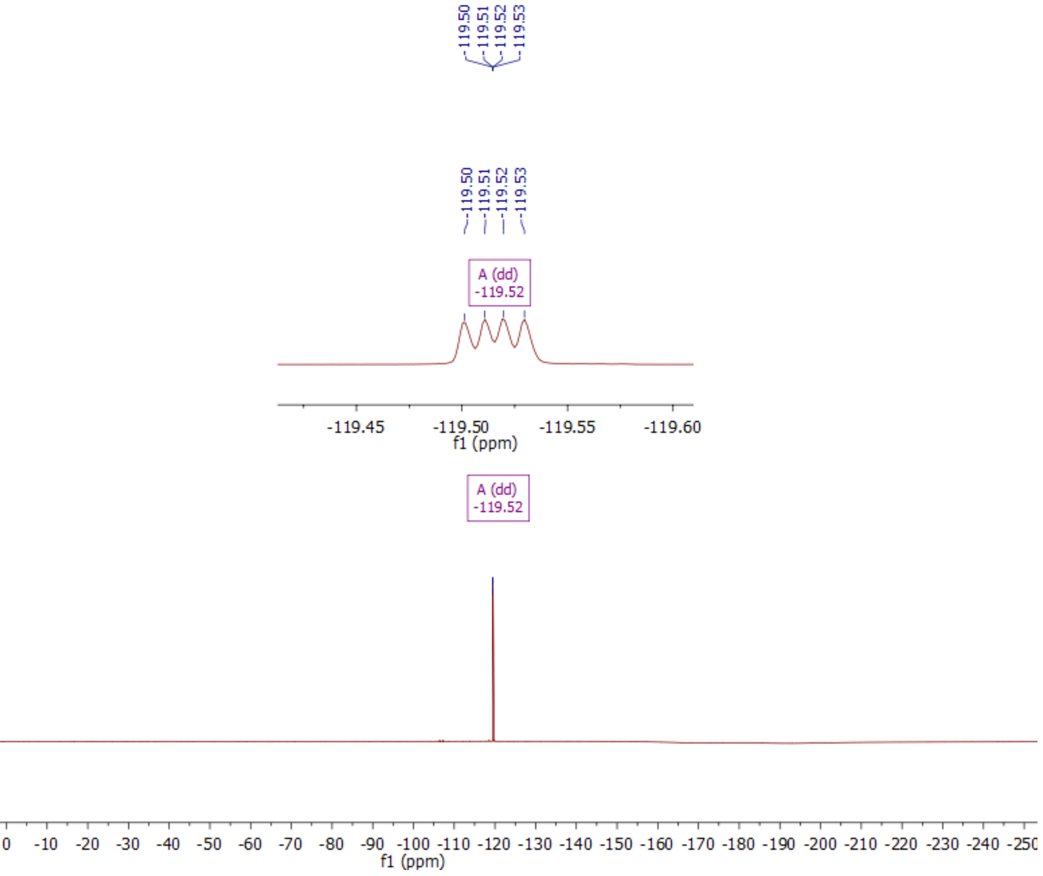
-119.50
-119.51
-119.52
-119.53
A (dd)
-119.52
-119.45
-119.50
-119.55
-119.60
f1 (ppm)
50 40 30 20 10 0 -10 -20 -30 -40 -50 -60 -70 -80 -90 -100 -110 -120 -130 -140 -150 -160 -170 -180 -190 -200 -210 -220 -230 -240 -250
f1 (ppm)

5,6-dimethoxy-2'-methylspiro[indene-2,3'-indolin]-1(3H)-one [**B5**]:

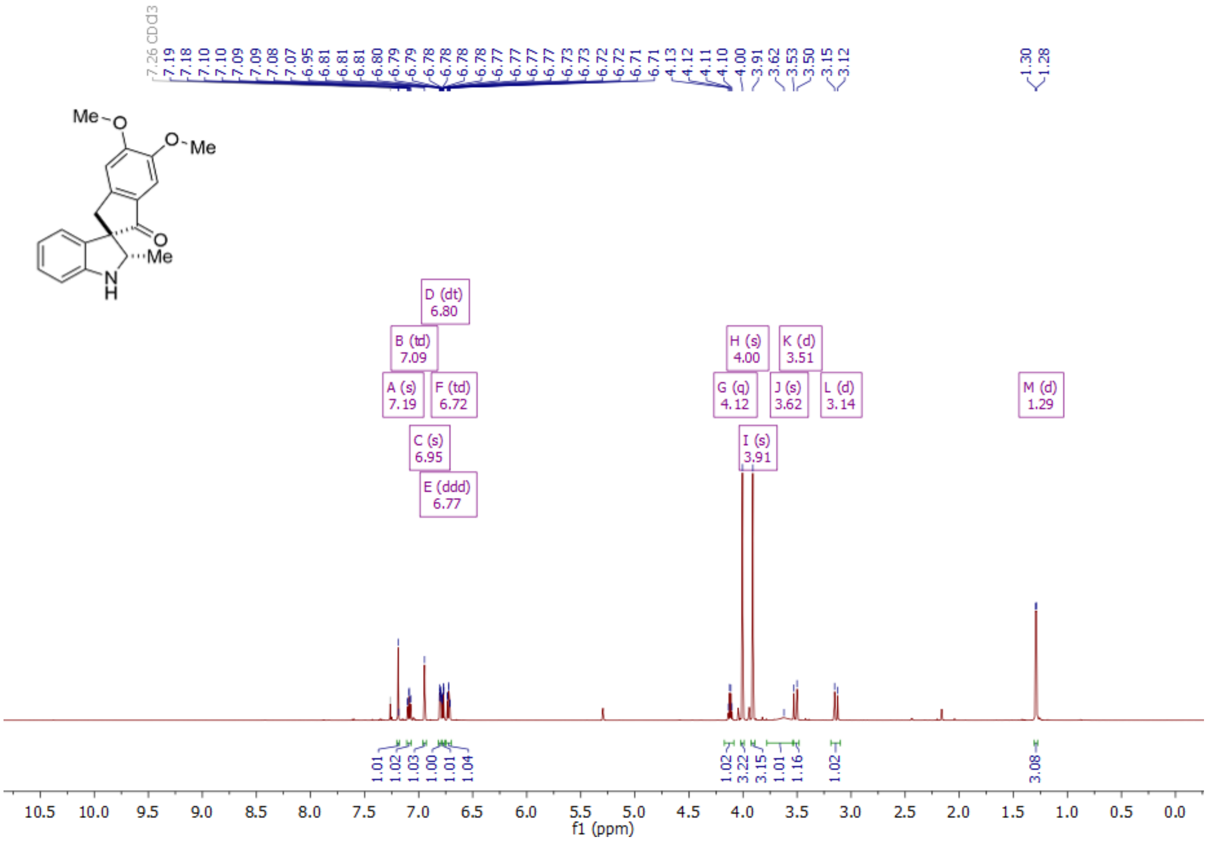

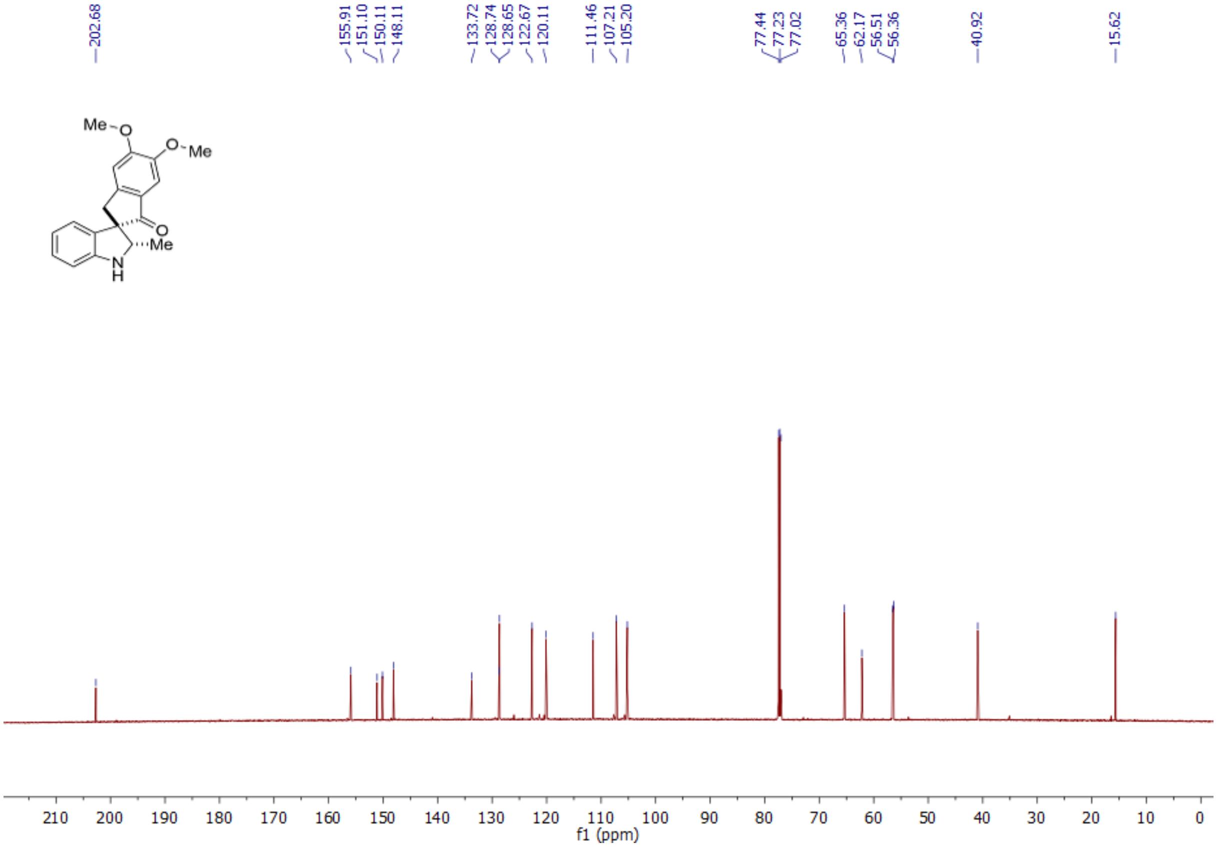

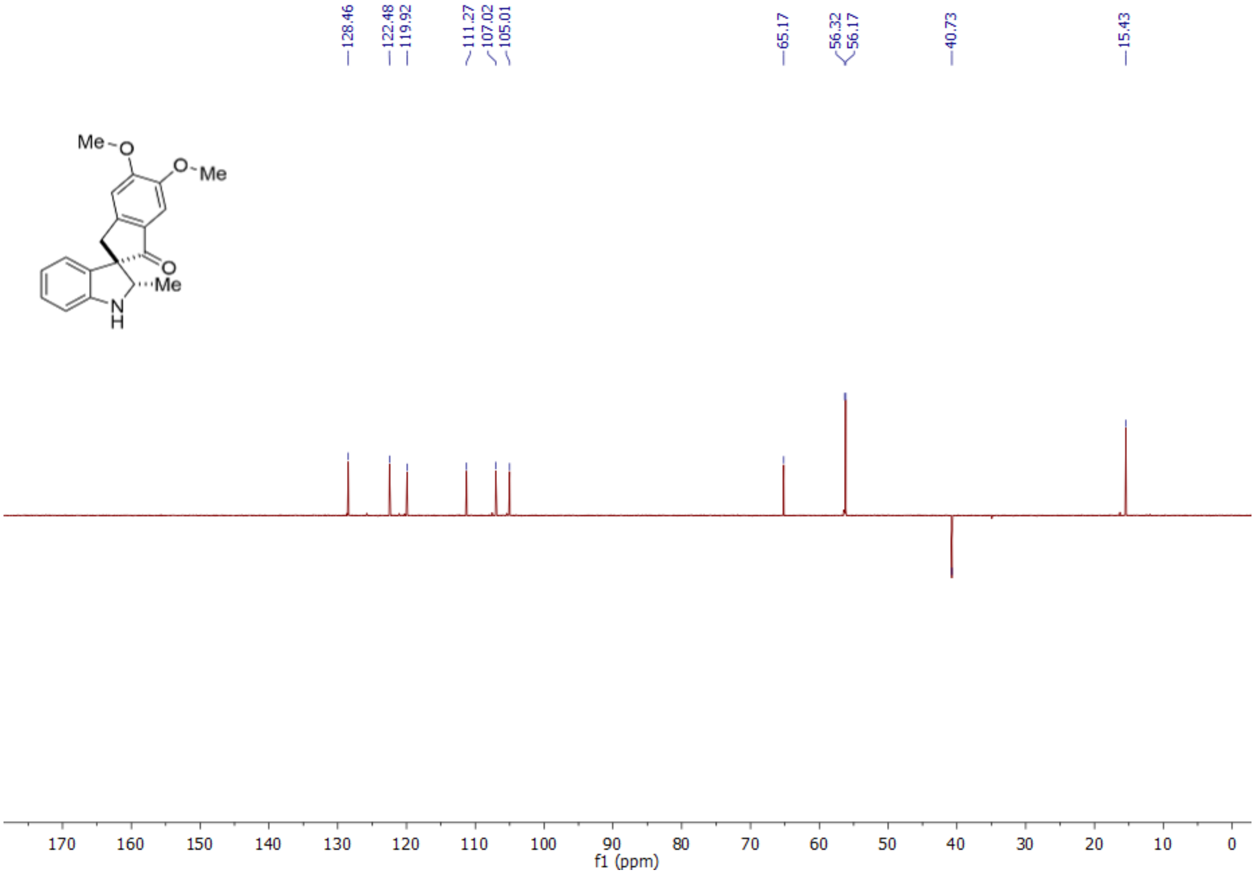

128.46
122.48
119.92
111.27
107.02
105.01
65.17
56.32
56.17
40.73
15.43
Me
O
O
Me
O
Me
N
H
170
160
150
140
130
120
110
100
90
80
70
60
50
40
30
20
10
0
f1 (ppm)

2'-methylspiro[indeno[4,5-d][1,3]dioxole-7,3'-indolin]-6(8H)-one [**B6**]:

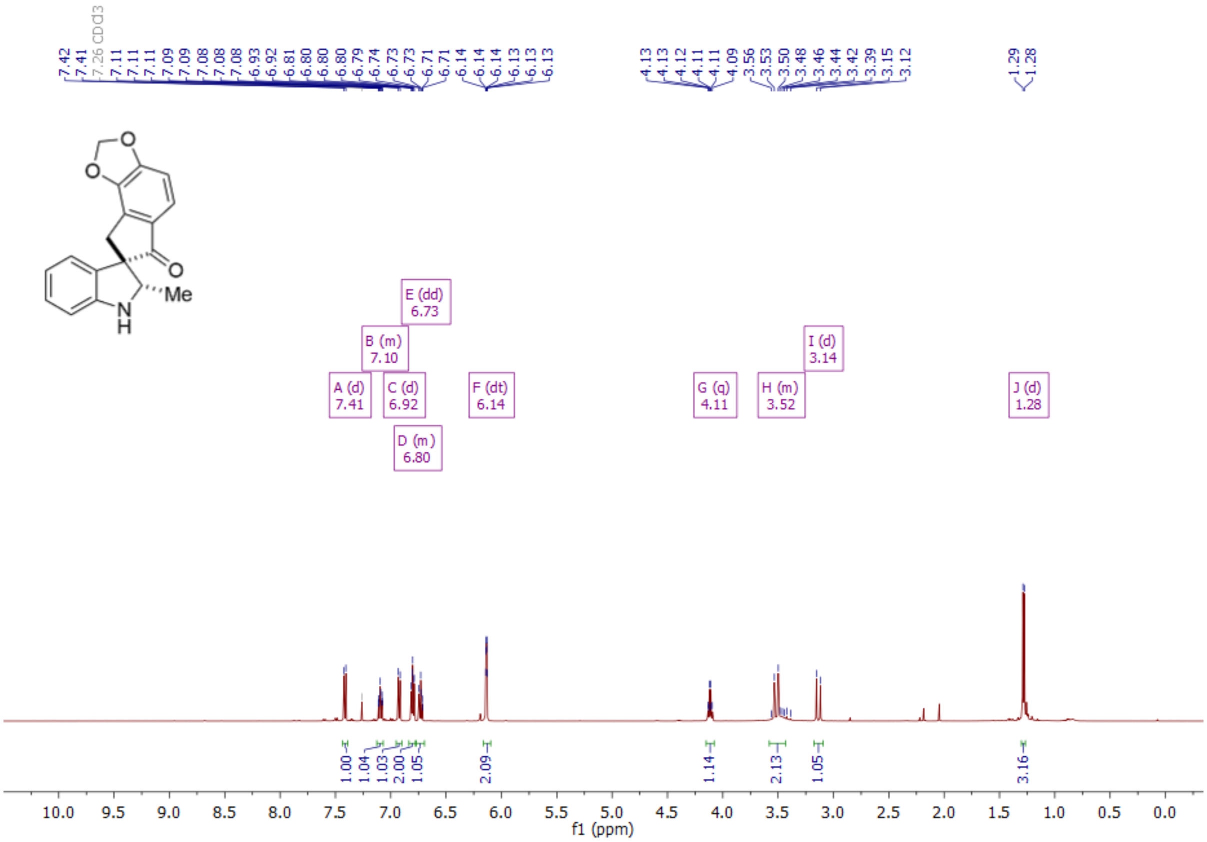

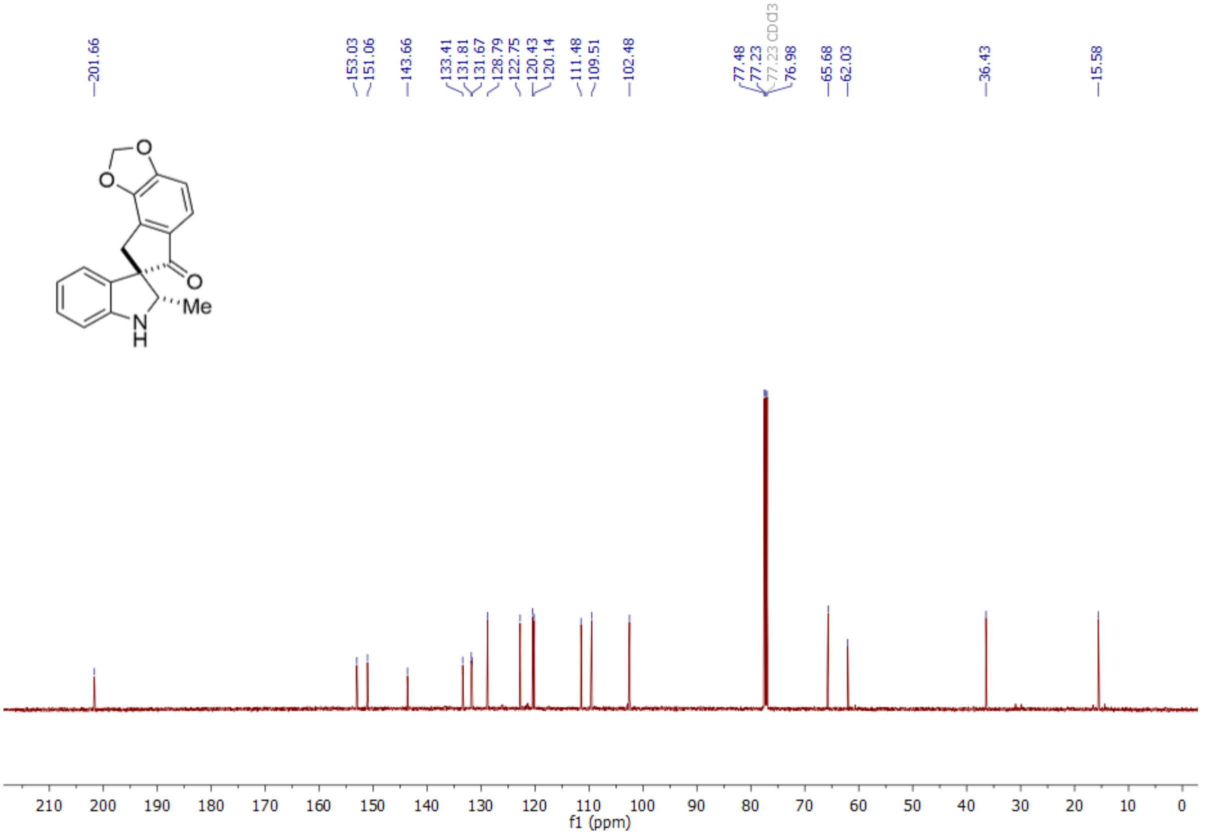

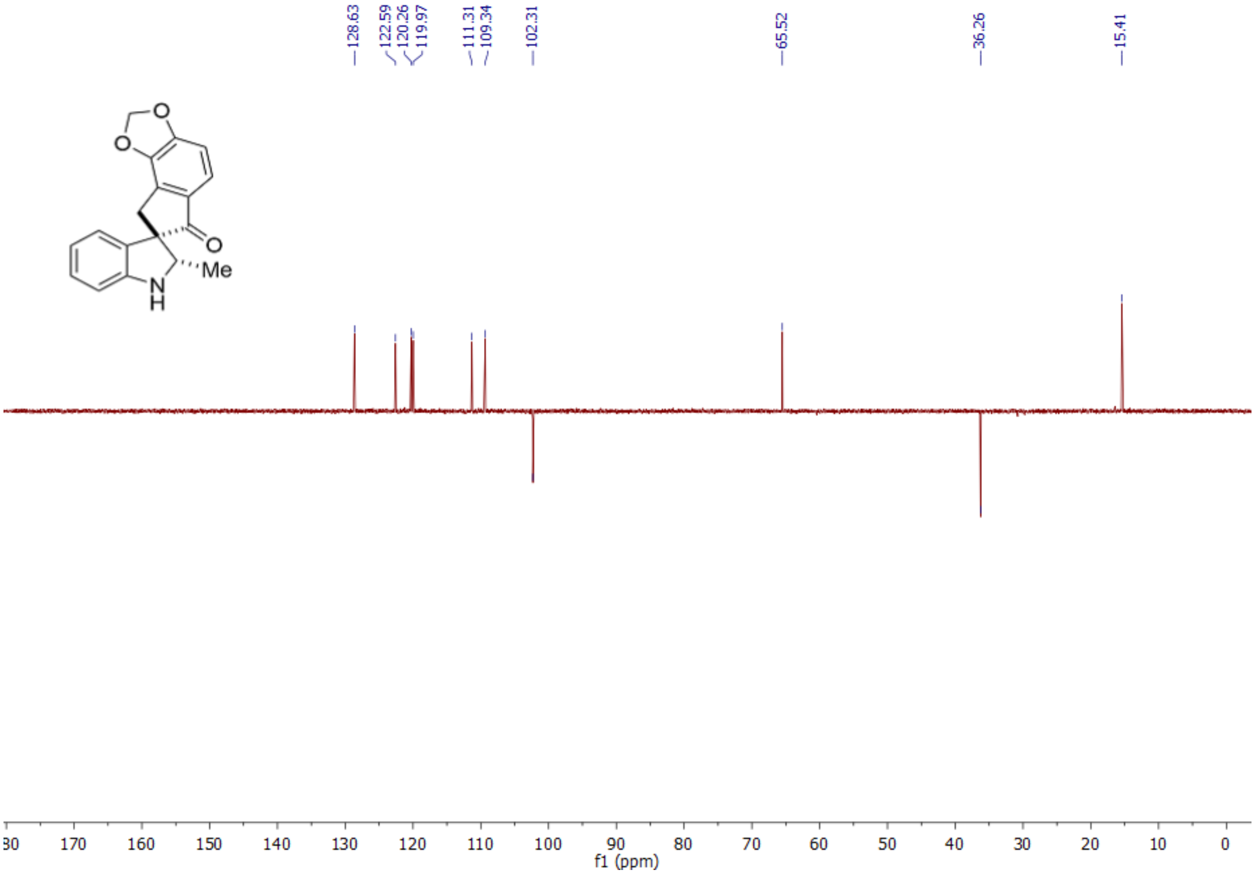

128.63
122.59
120.26
119.97
111.31
109.34
102.31
65.52
36.26
15.41
O
O
O
N
H
Me
30
170
160
150
140
130
120
110
100
90
80
70
60
50
40
30
20
10
0
f1 (ppm)

2'-methylspiro[cyclopenta[a]naphthalene-2,3'-indolin]-3(1H)-one **[B7]**:

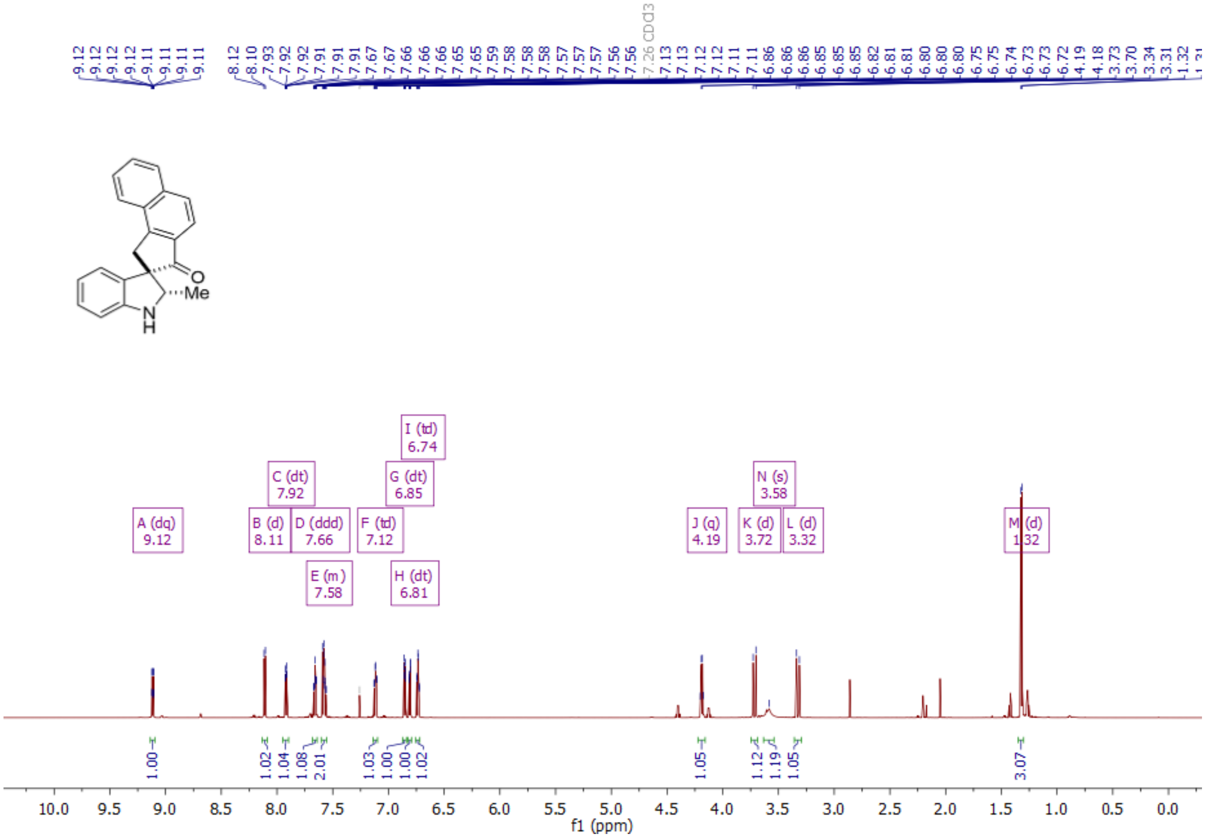

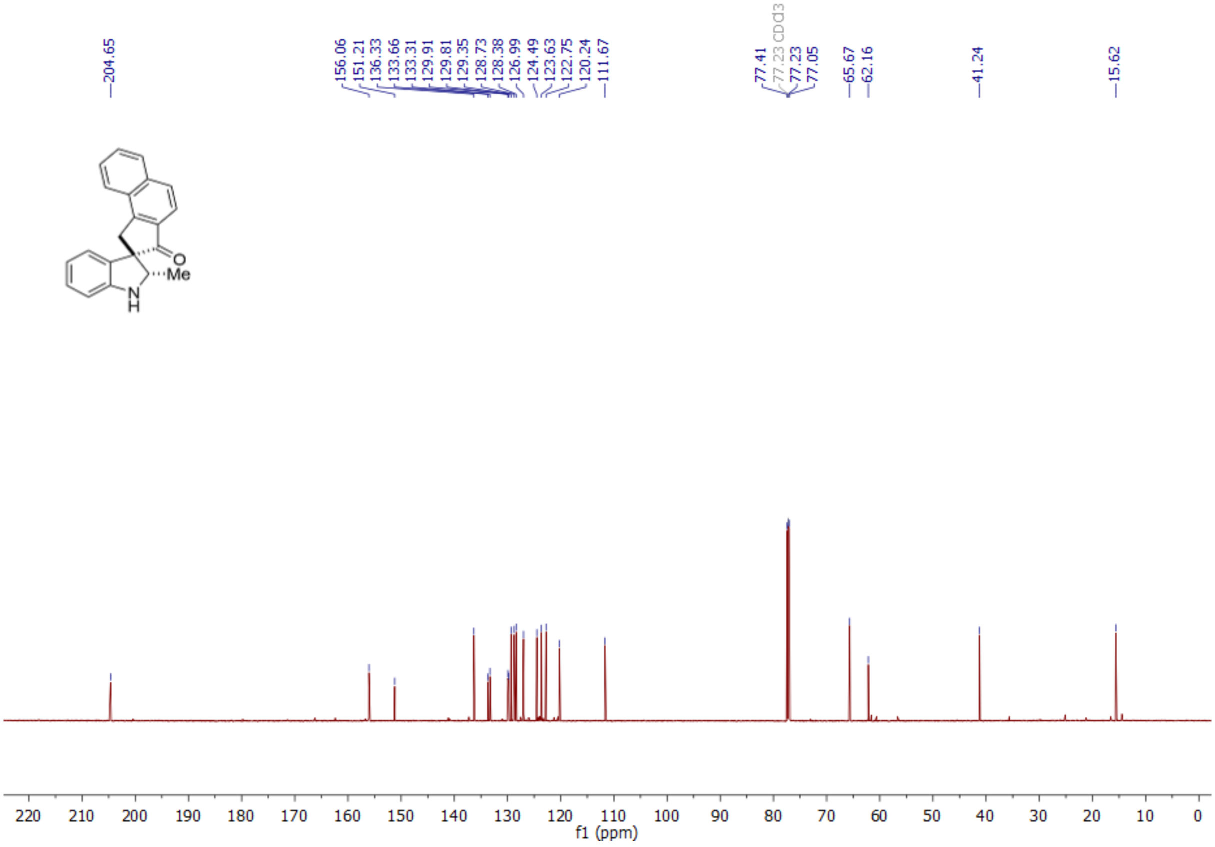

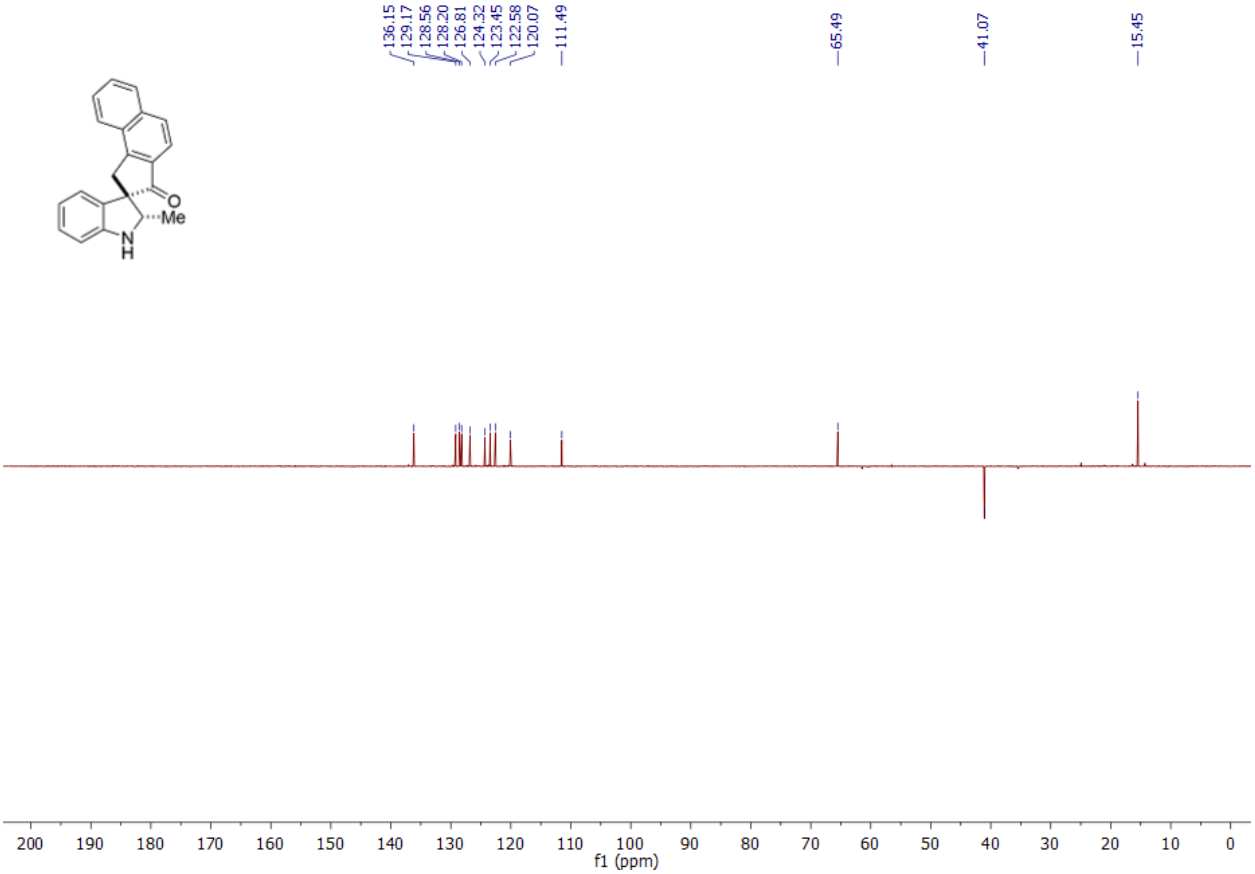
136.15
129.17
128.56
128.20
126.81
124.32
123.45
122.58
120.07
111.49
65.49
41.07
15.45
Me
O
N
H
200 190 180 170 160 150 140 130 120 110 100 90 80 70 60 50 40 30 20 10 0
f1 (ppm)

2',5'-dimethylspiro[indene-2,3'-indolin]-1(3H)-one [**B8**]:

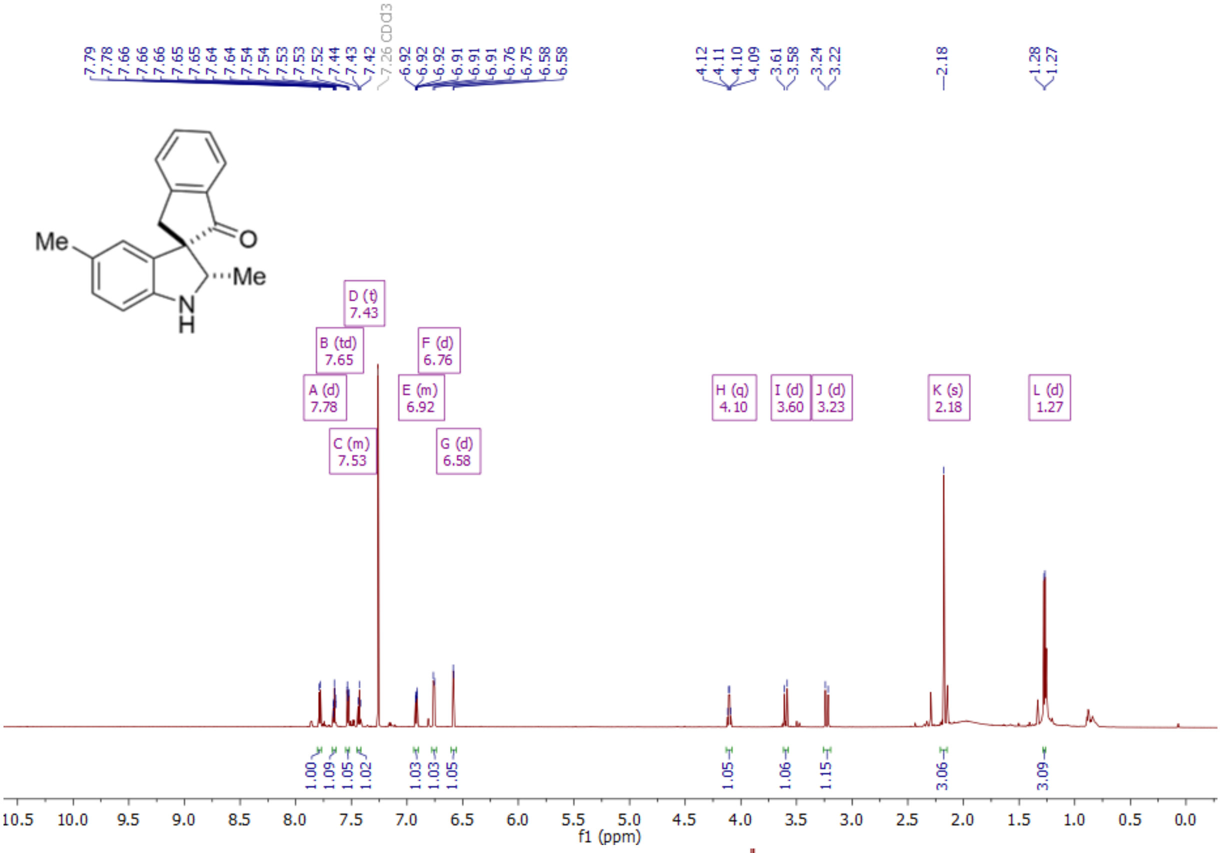

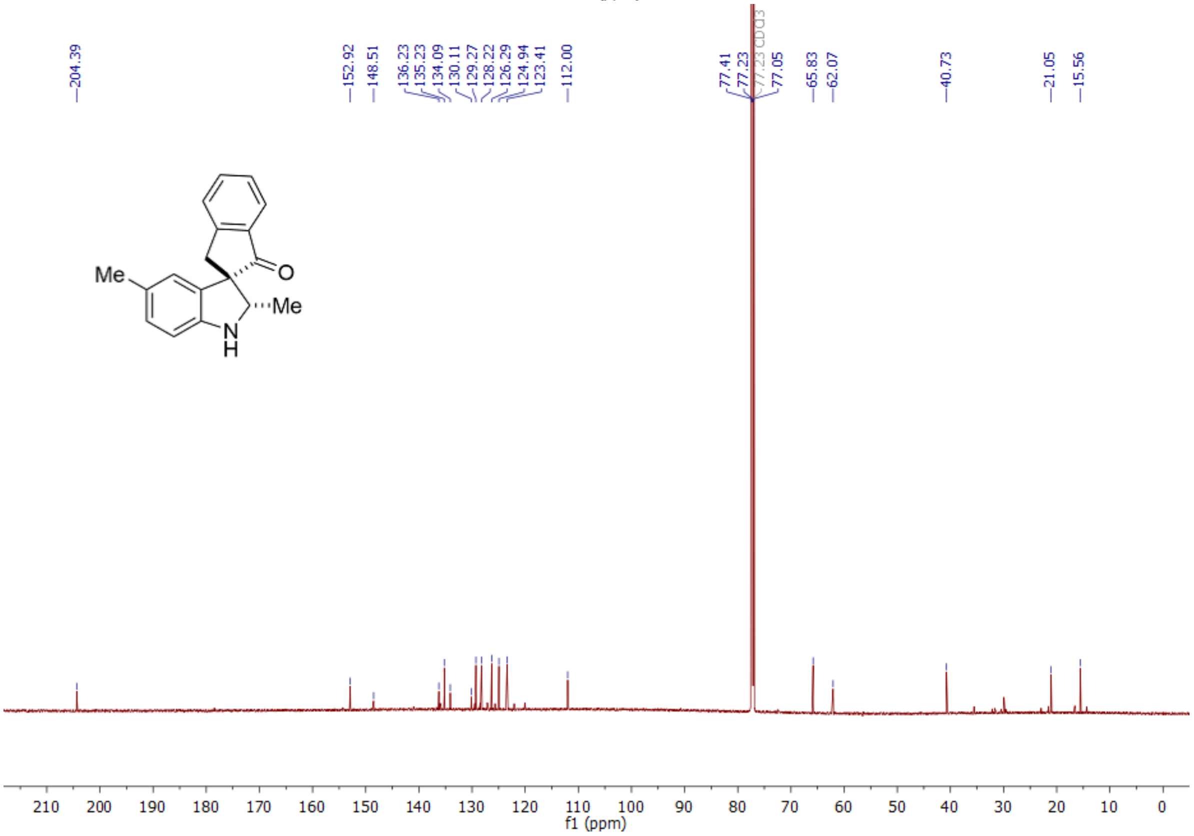

6-chloro-5'-fluoro-2'-methylspiro[indene-2,3'-indolin]-1(3H)-one [**B9**]:

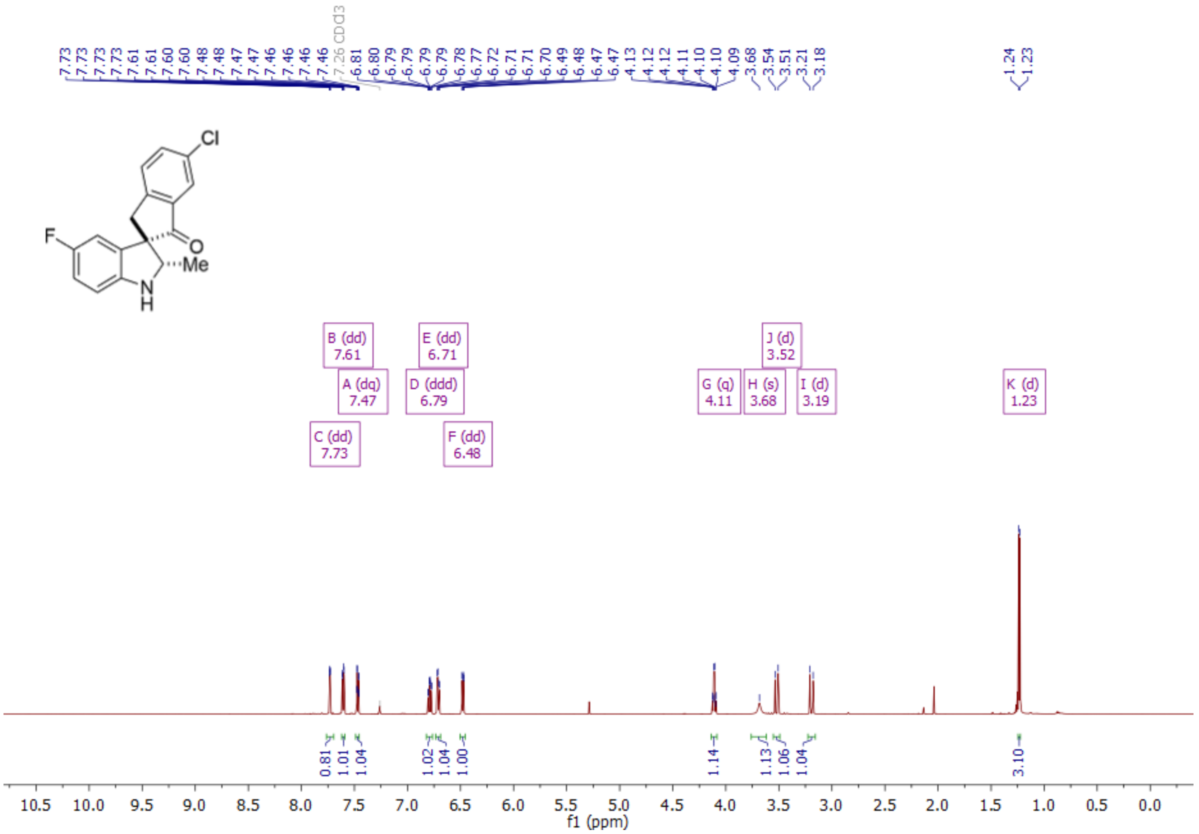

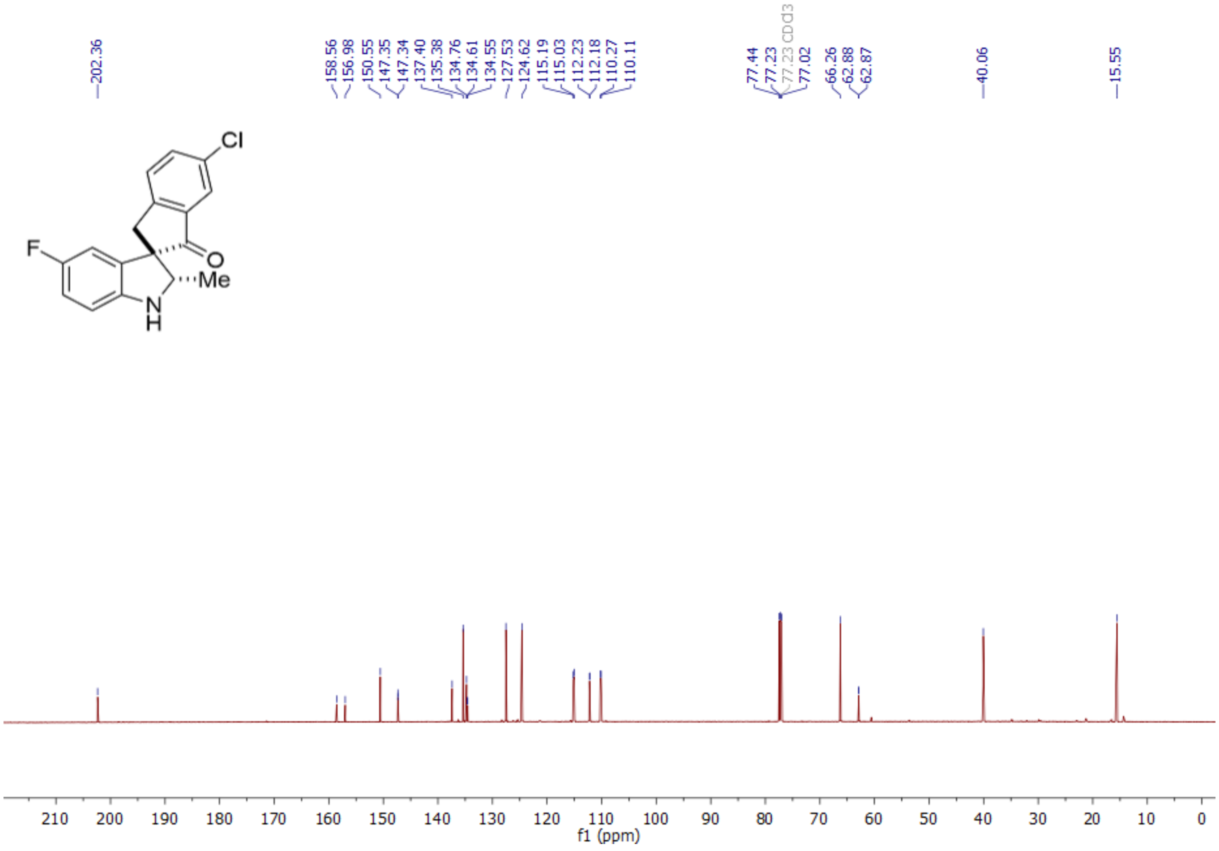

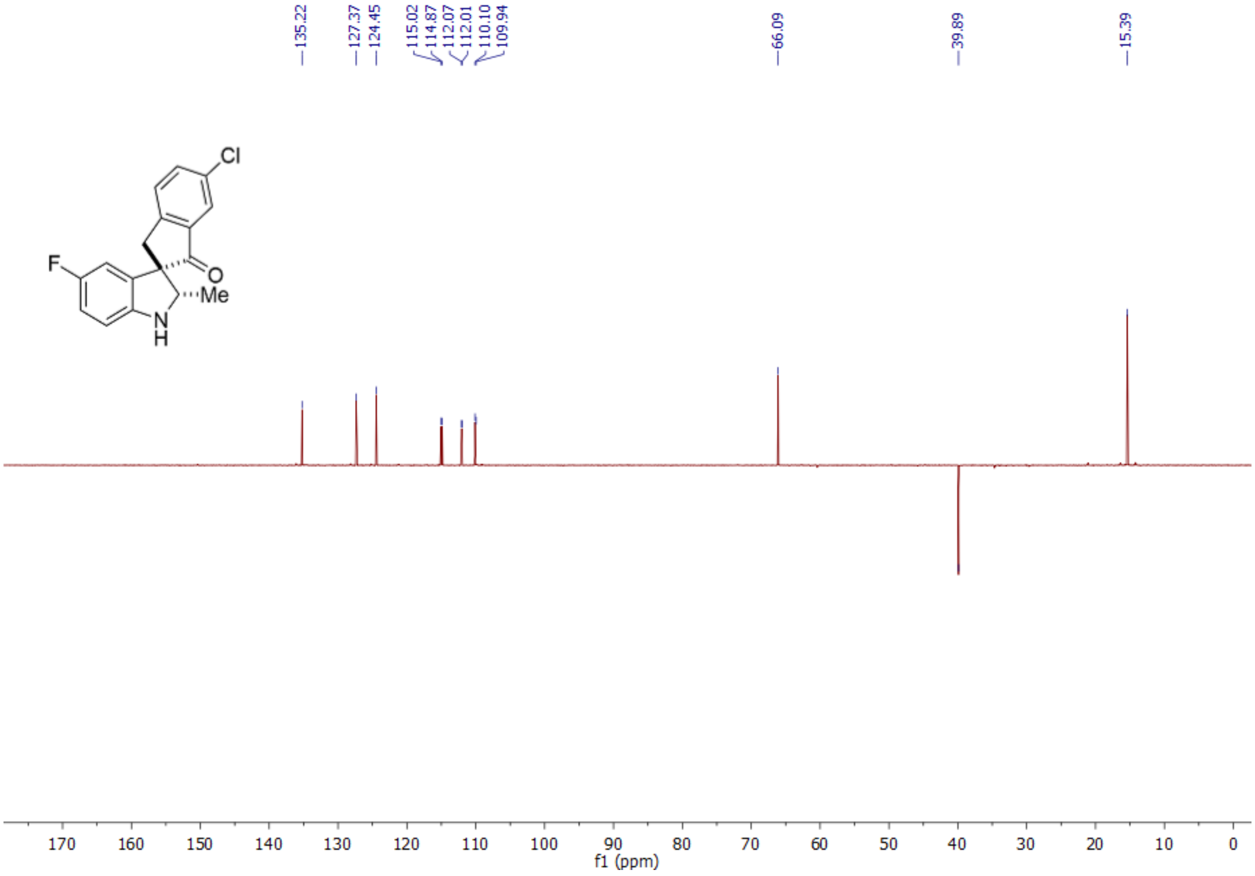

135.22
127.37
124.45
115.02
114.87
112.07
112.01
110.10
109.94
66.09
39.89
15.39
Cl
F
O
Me
N
H
170 160 150 140 130 120 110 100 90 80 70 60 50 40 30 20 10 0
f1 (ppm)

5'-fluoro-6-methoxy-2'-methylspiro[indene-2,3'-indolin]-1(3H)-one [**B10**]:

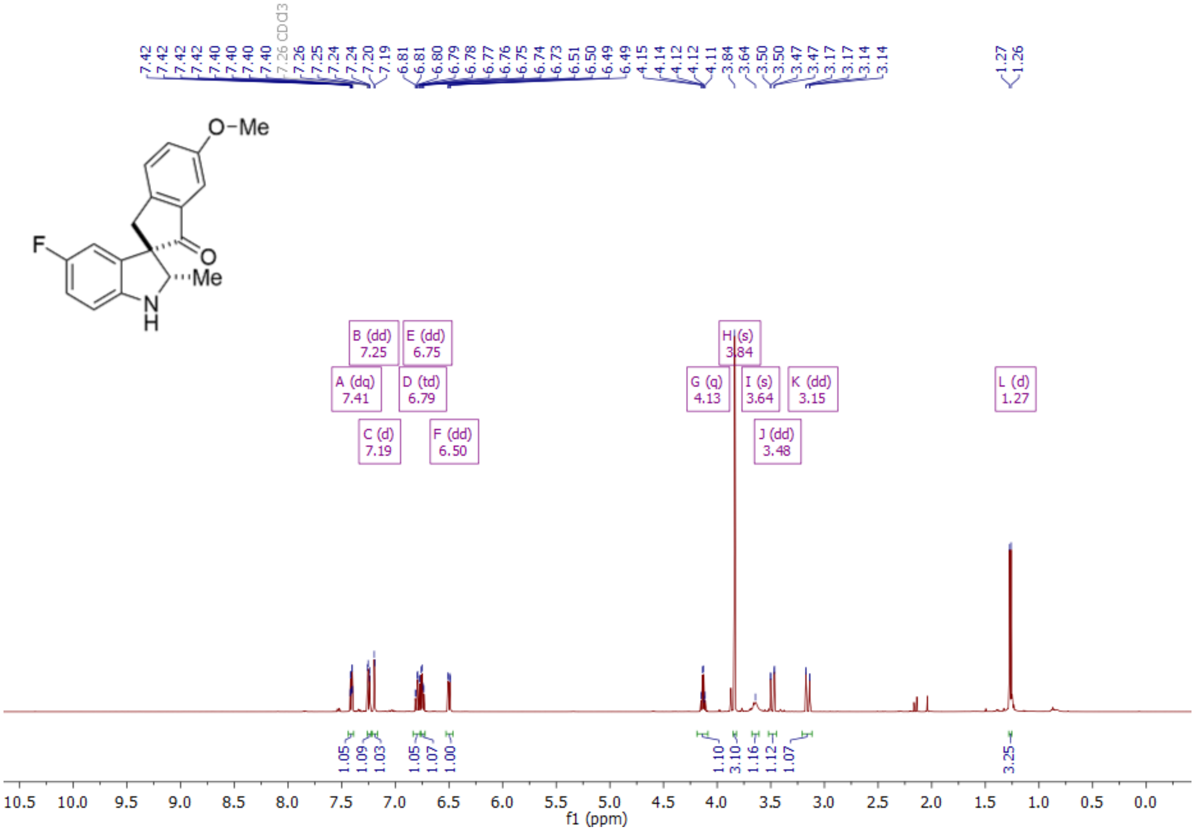

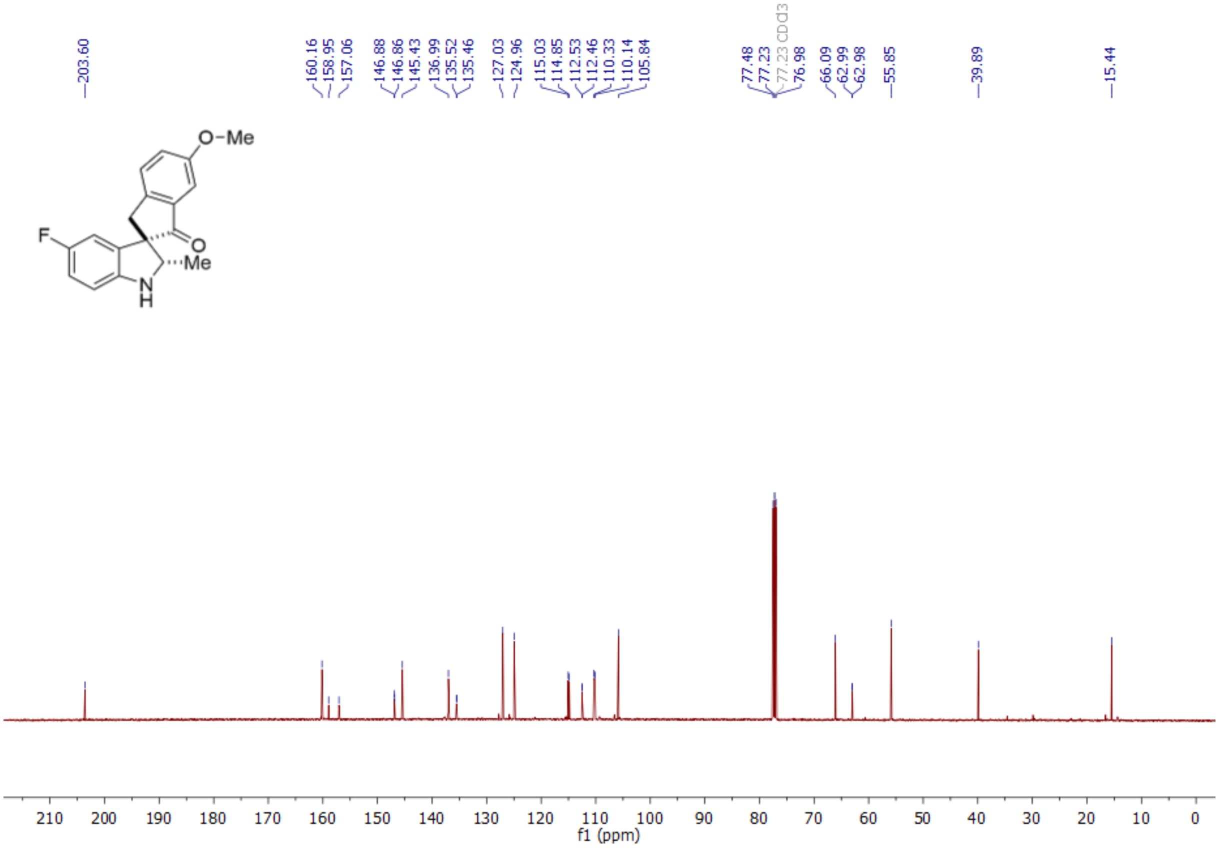

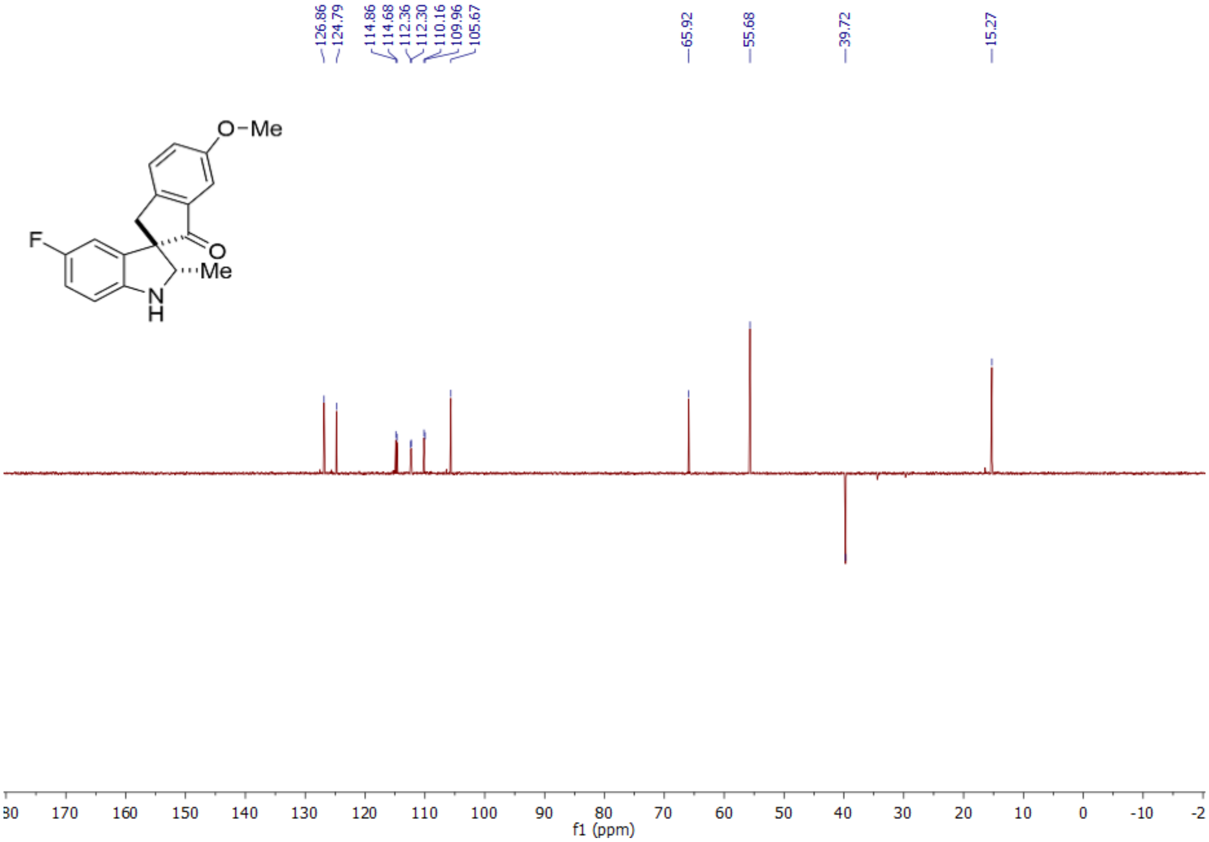
126.86
124.79
114.86
114.68
112.36
112.30
110.16
109.96
105.67
65.92
55.68
39.72
15.27
O−Me
F
O
Me
N
H
f1 (ppm)

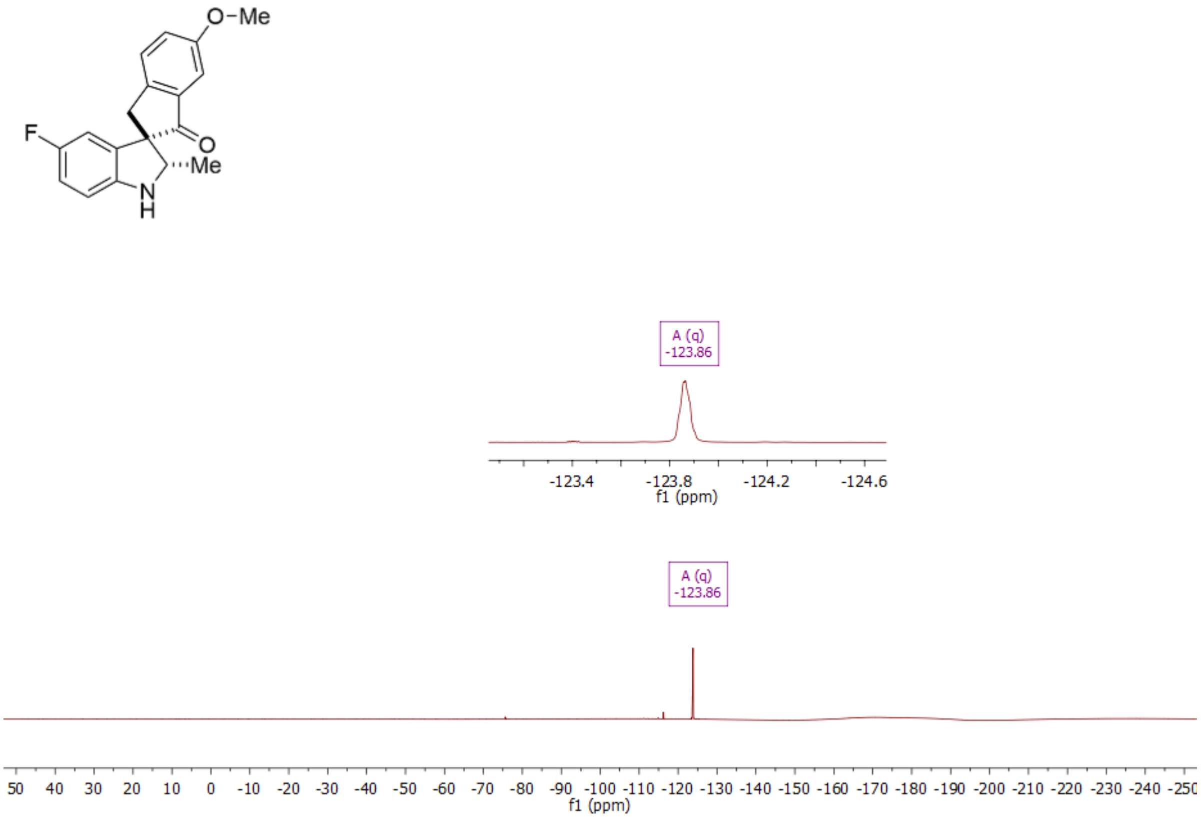
O−Me
F
O
Me
N
H
A (q)
-123.86
-123.4
-123.8
-124.2
-124.6
f1 (ppm)
A (q)
-123.86
f1 (ppm)

6-chloro-2',5'-dimethylspiro[indene-2,3'-indolin]-1(3H)-one [**B11**]:

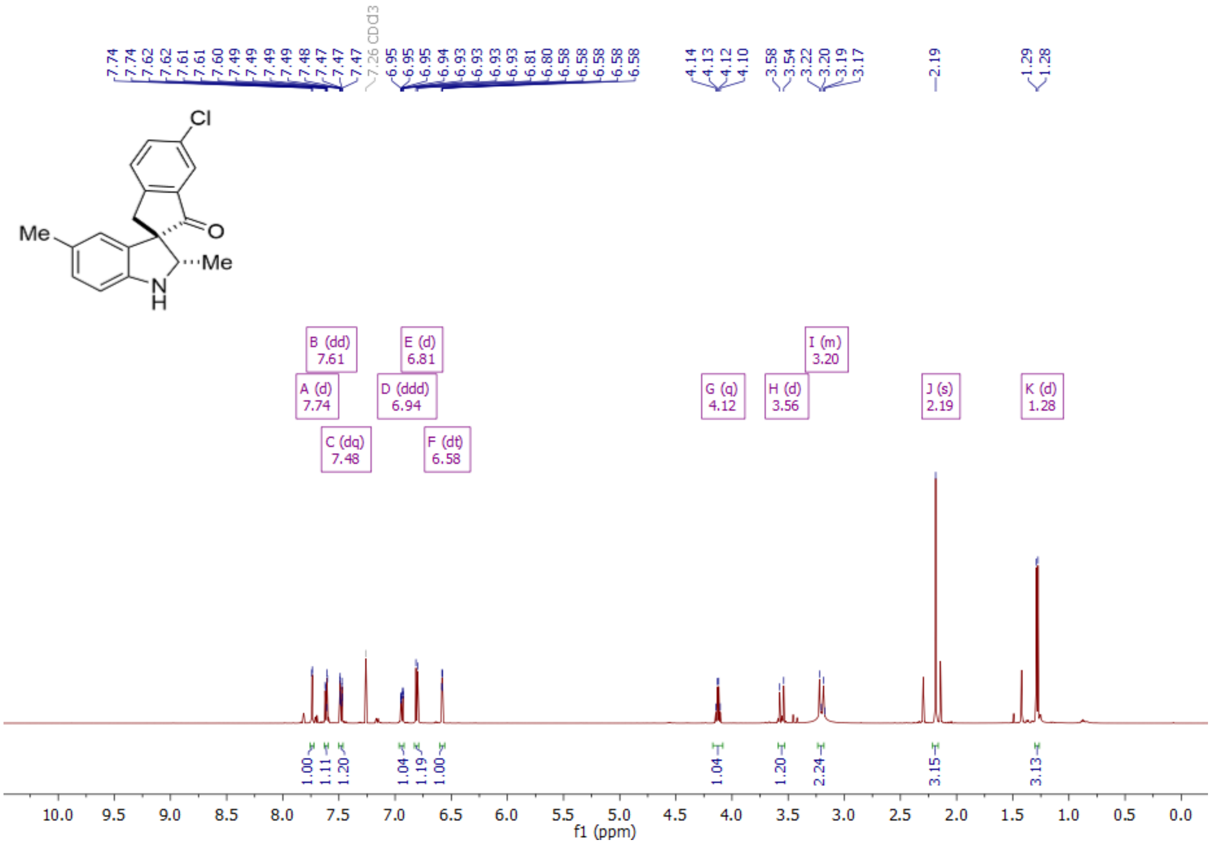

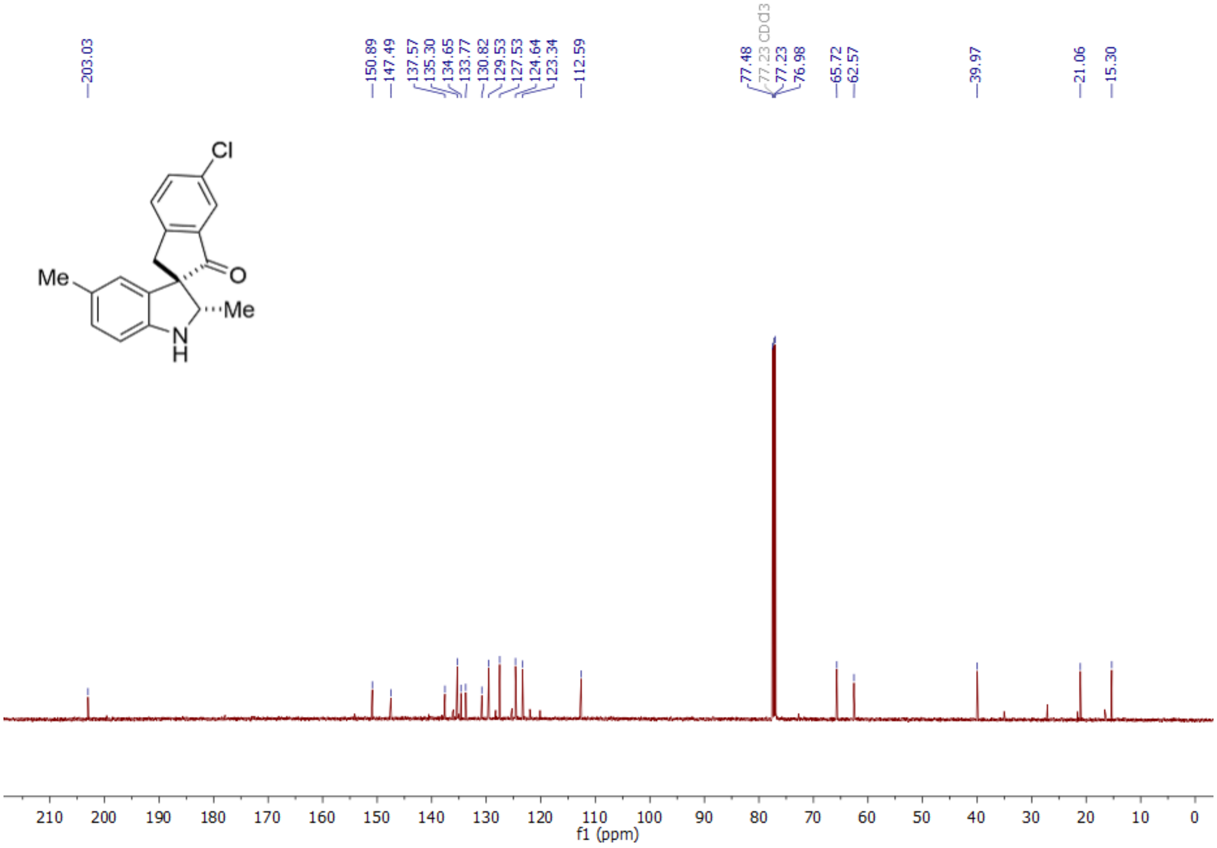

5'-chloro-5-fluoro-2'-methylspiro[indene-2,3'-indolin]-1(3H)-one [**B12**]:

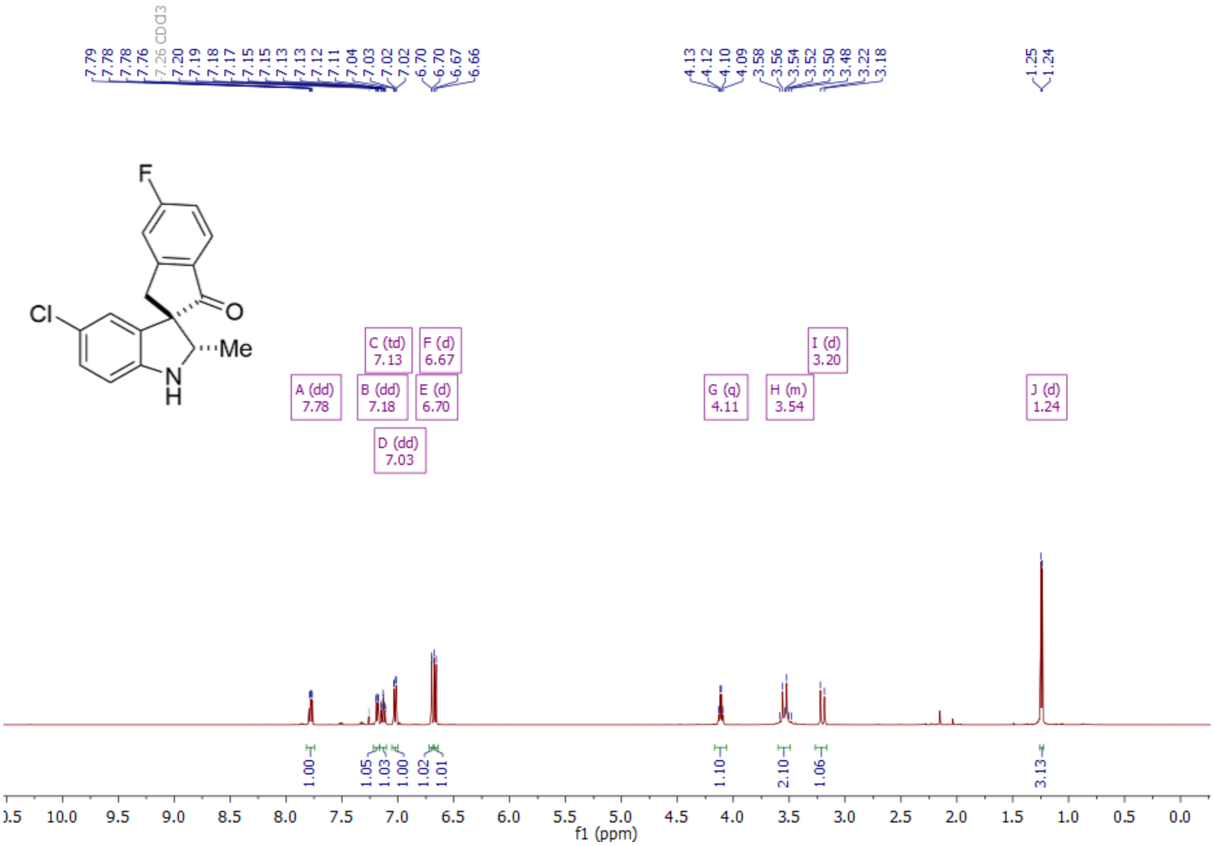

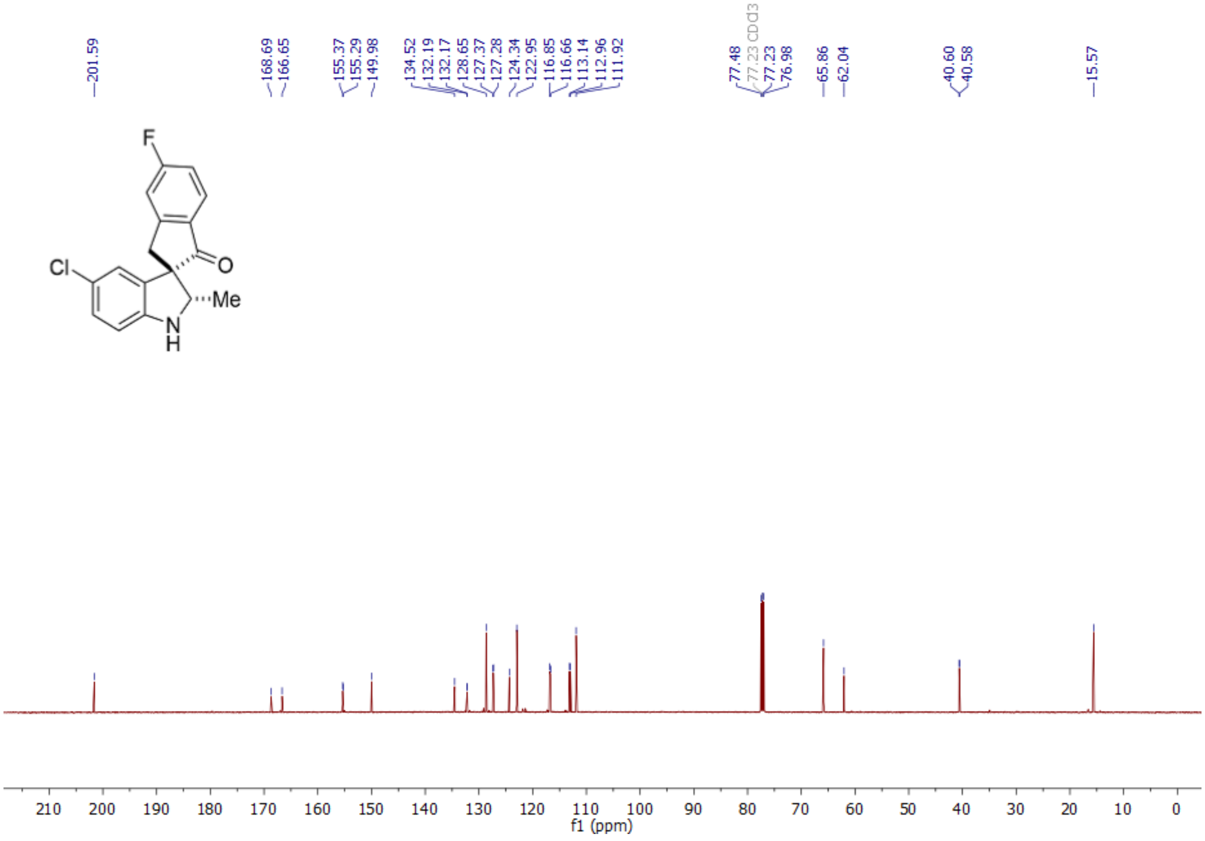

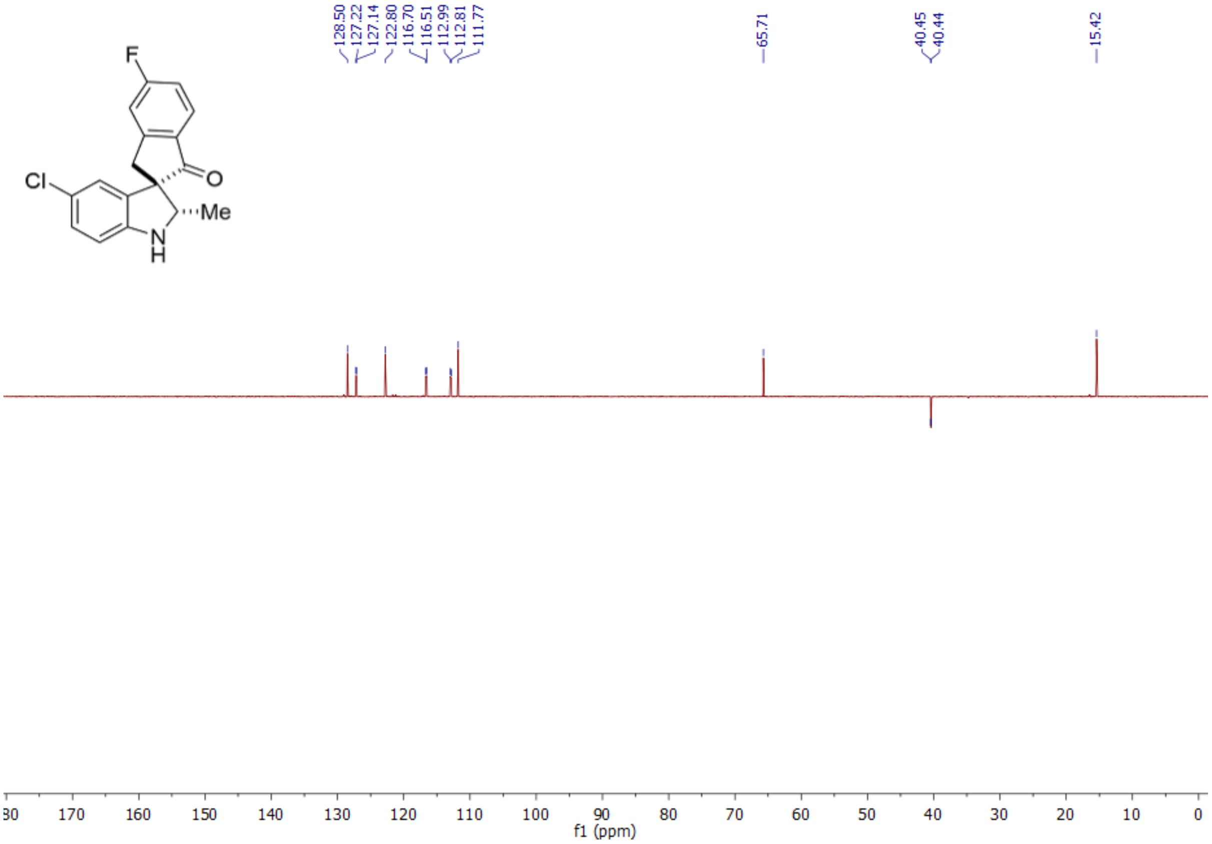
128.50
127.22
127.14
122.80
116.70
116.51
112.99
112.81
111.77
65.71
40.45
40.44
15.42
F
Cl
O
Me
N
H
f1 (ppm)

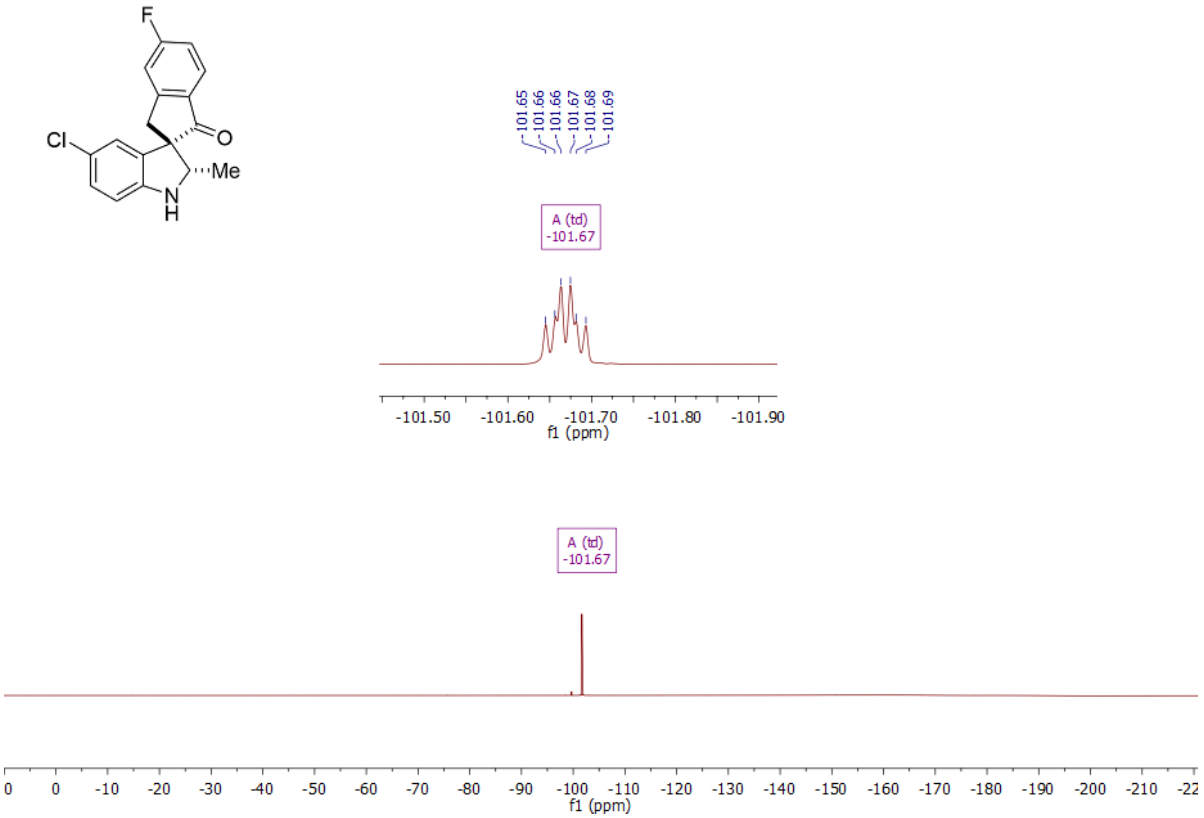
F
Cl
O
Me
N
H
-101.65
-101.66
-101.66
-101.67
-101.68
-101.69
A (td)
-101.67
-101.50
-101.60
-101.70
-101.80
-101.90
f1 (ppm)
A (td)
-101.67
f1 (ppm)

4,5'-difluoro-2'-methylspiro[indene-2,3'-indolin]-1(3H)-one [**B13**]:

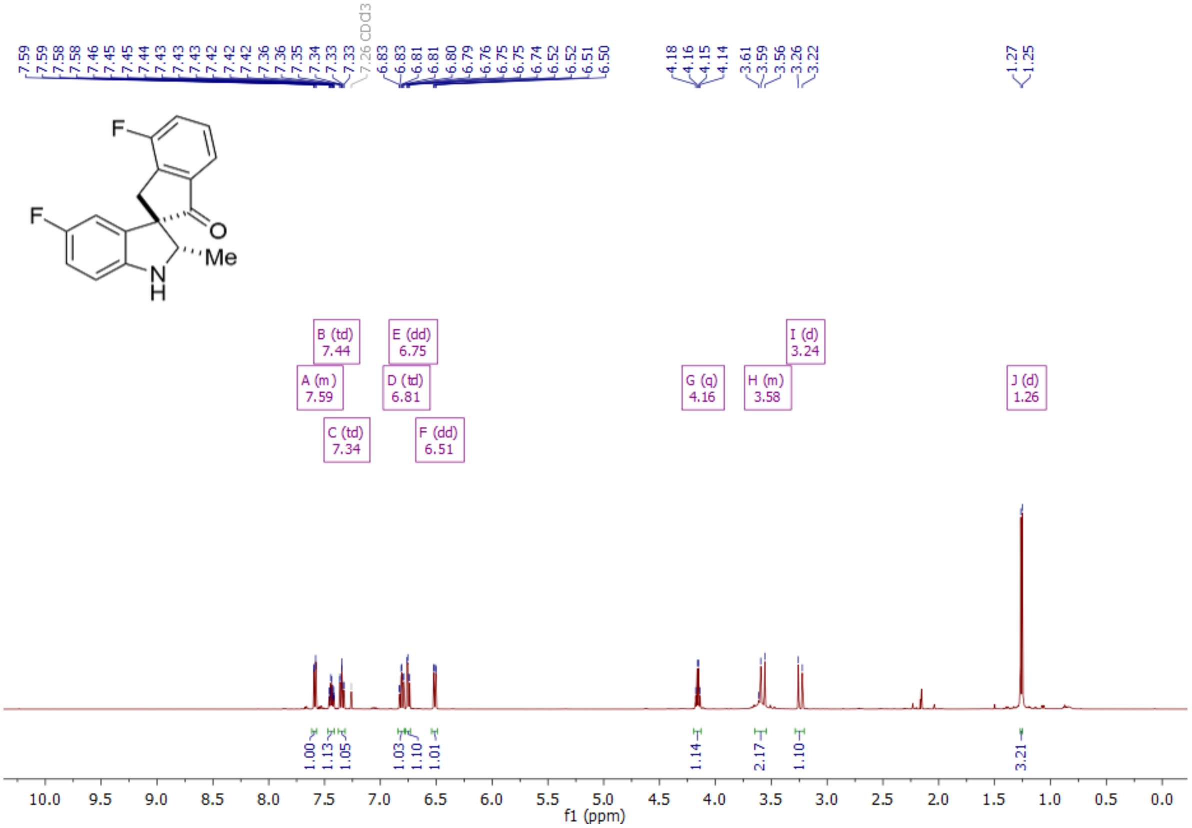

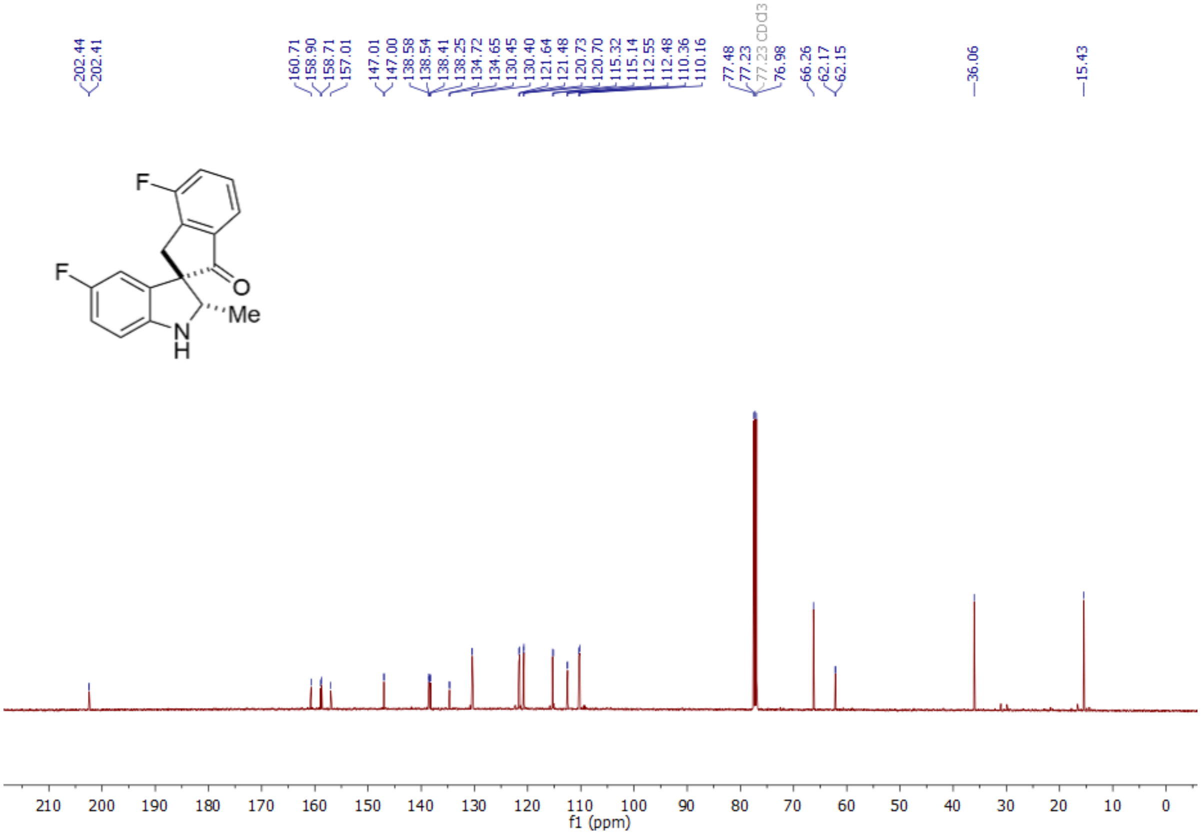

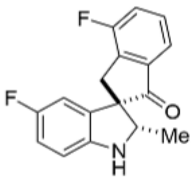

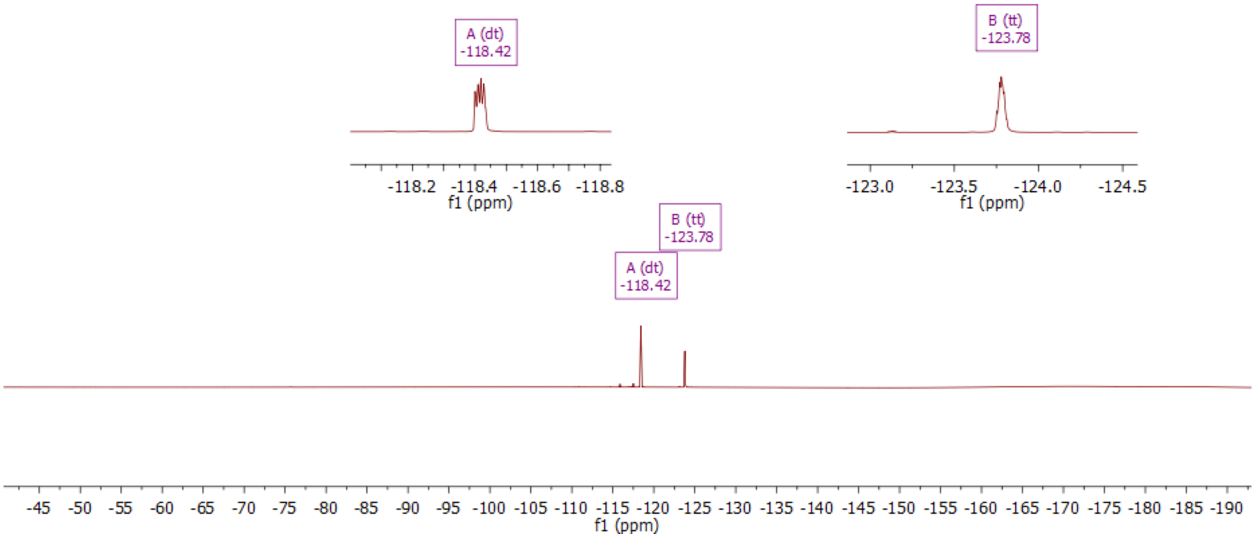

A (dt)
-118.42
B (tt)
-123.78
-118.2 -118.4 -118.6 -118.8
f1 (ppm)
-123.0 -123.5 -124.0 -124.5
f1 (ppm)
B (tt)
-123.78
A (dt)
-118.42
-45 -50 -55 -60 -65 -70 -75 -80 -85 -90 -95 -100 -105 -110 -115 -120 -125 -130 -135 -140 -145 -150 -155 -160 -165 -170 -175 -180 -185 -190
f1 (ppm)

5'-chloro-4-fluoro-2'-methylspiro[indene-2,3'-indolin]-1(3H)-one [**B14**]:

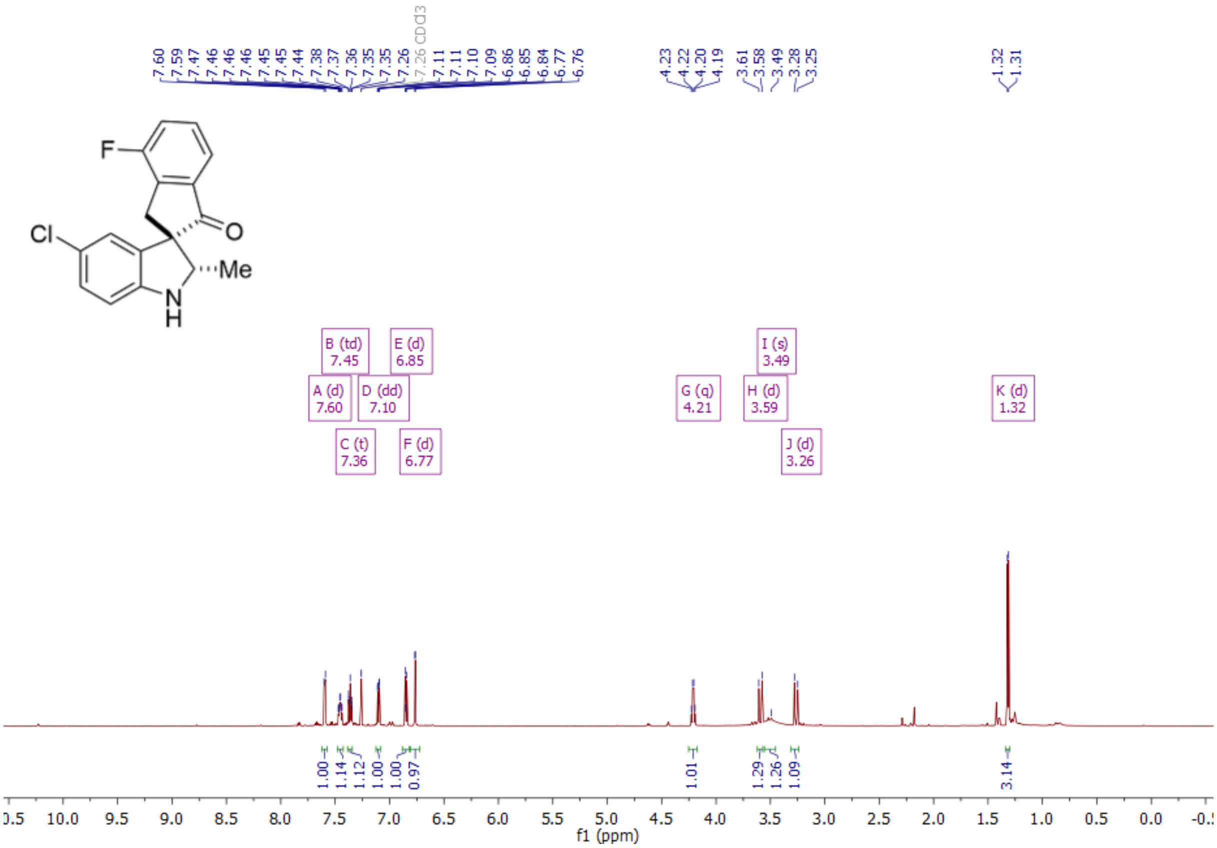

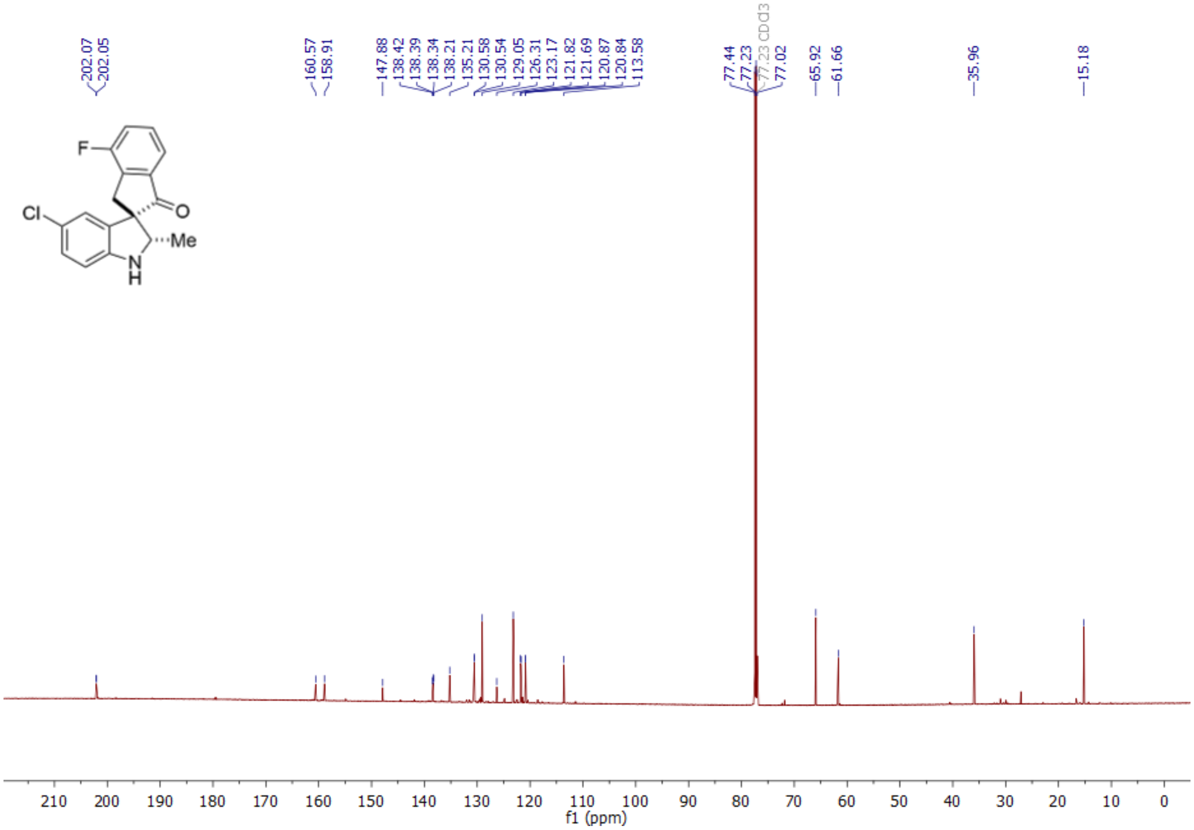

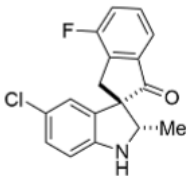
F
Cl
O
Me
N
H

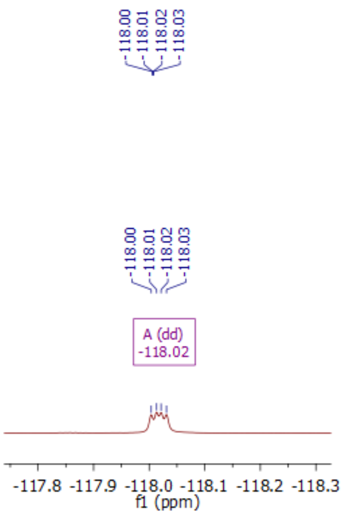
-118.00
-118.01
-118.02
-118.03
A (dd)
-118.02
-117.8 -117.9 -118.0 -118.1 -118.2 -118.3
f1 (ppm)

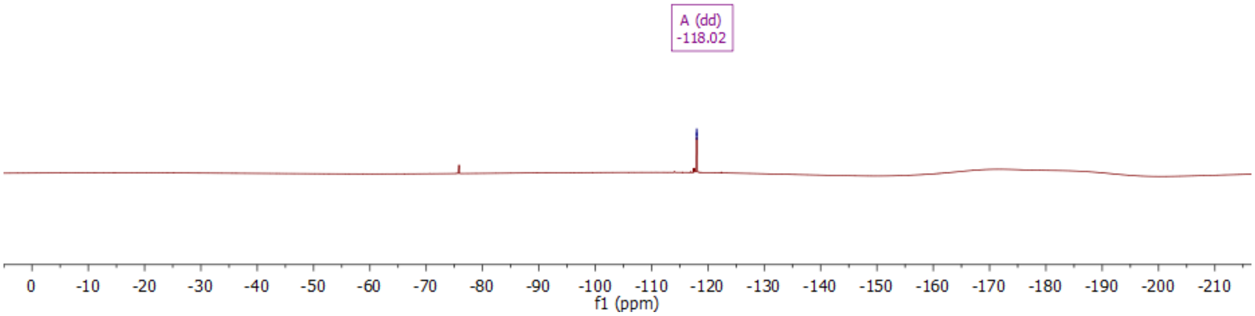
A (dd)
-118.02
0 -10 -20 -30 -40 -50 -60 -70 -80 -90 -100 -110 -120 -130 -140 -150 -160 -170 -180 -190 -200 -210
f1 (ppm)

5'-fluoro-5,6-dimethoxy-2'-methylspiro[indene-2,3'-indolin]-1(3H)-one [**B15**]:

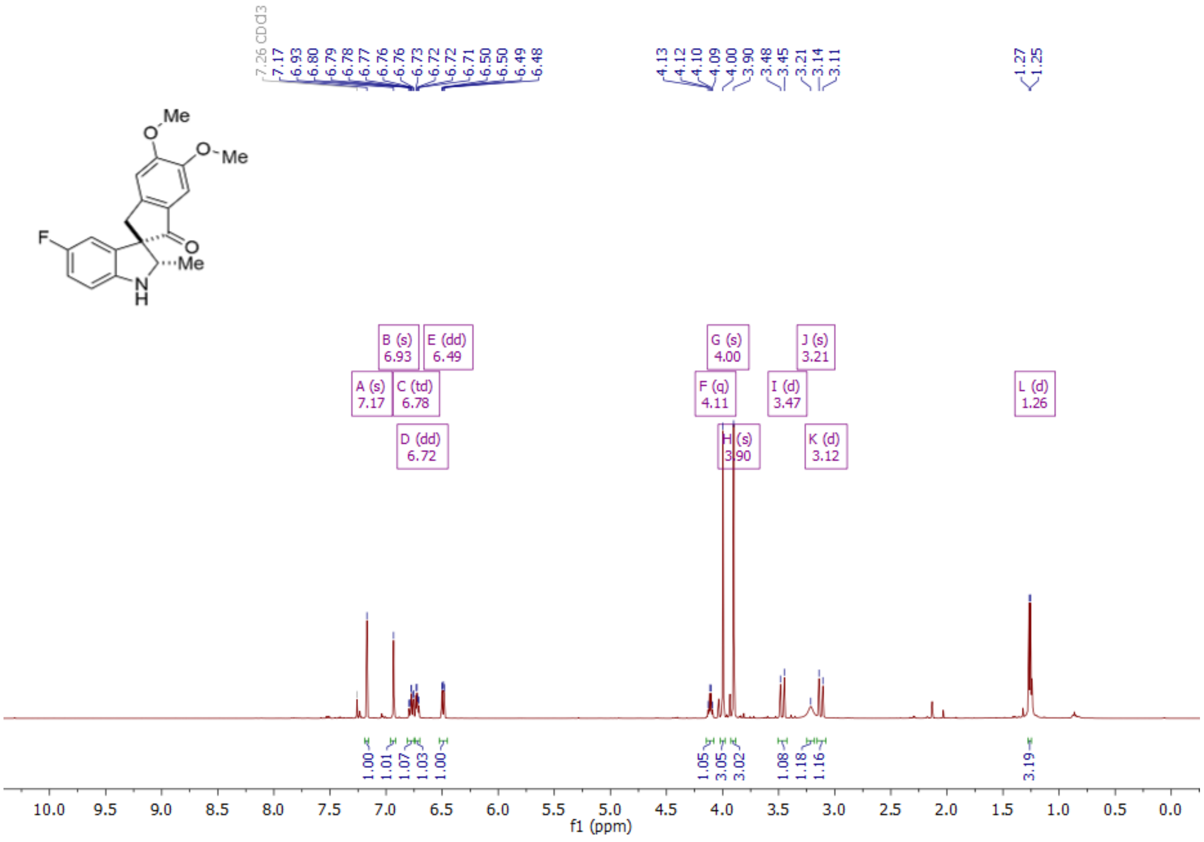

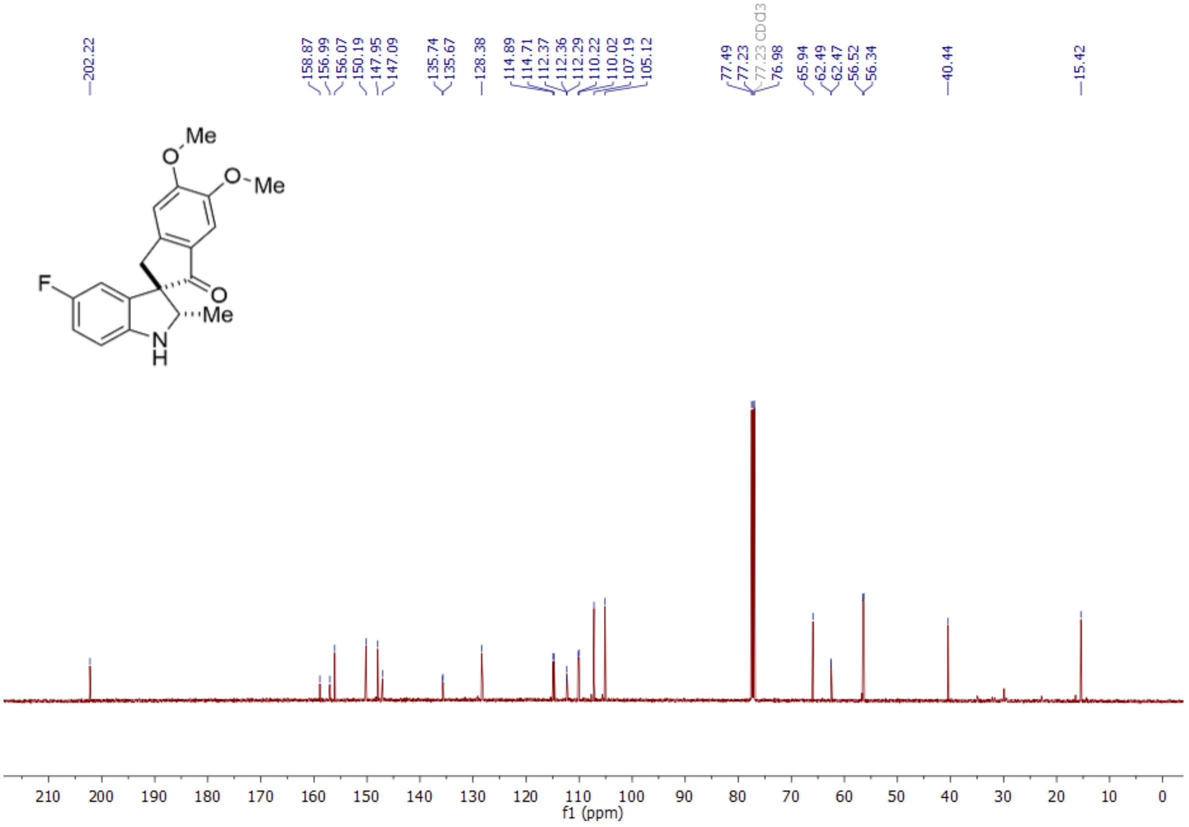

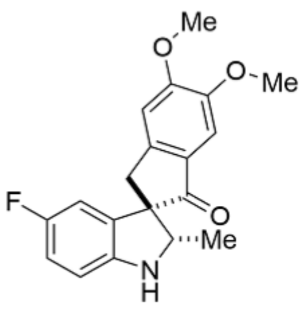
Me
O
O
Me
F
O
Me
N
H

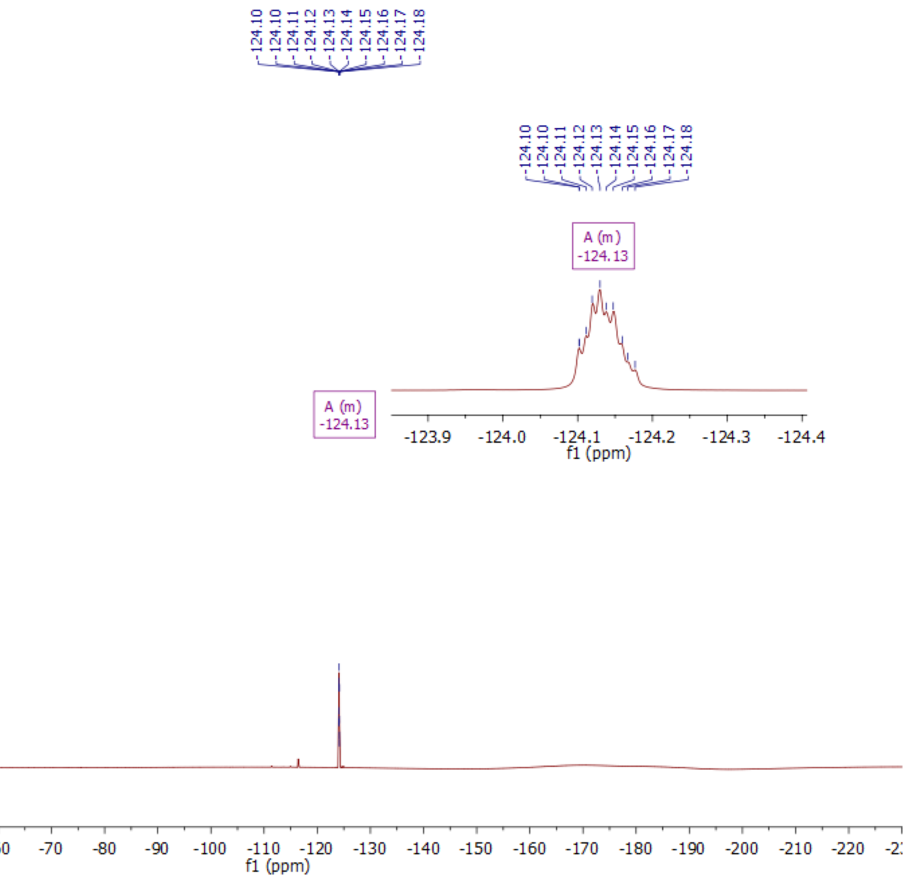
-124.10
-124.10
-124.11
-124.12
-124.13
-124.14
-124.15
-124.16
-124.17
-124.18
A (m)
-124.13
-123.9 -124.0 -124.1 -124.2 -124.3 -124.4
f1 (ppm)
0 -10 -20 -30 -40 -50 -60 -70 -80 -90 -100 -110 -120 -130 -140 -150 -160 -170 -180 -190 -200 -210 -220
f1 (ppm)

5,6-dimethoxy-2',5'-dimethylspiro[indene-2,3'-indolin]-1(3H)-one [**B16**]:

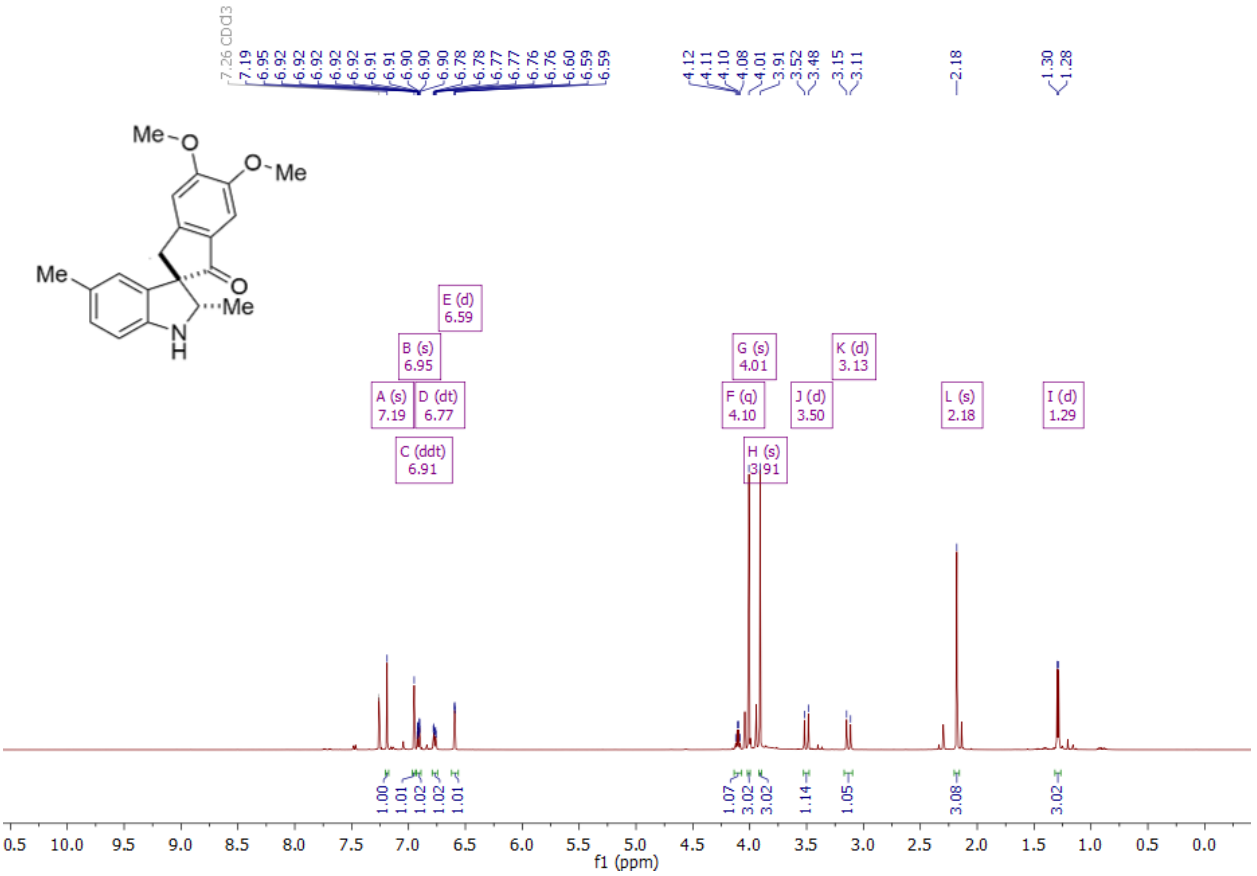

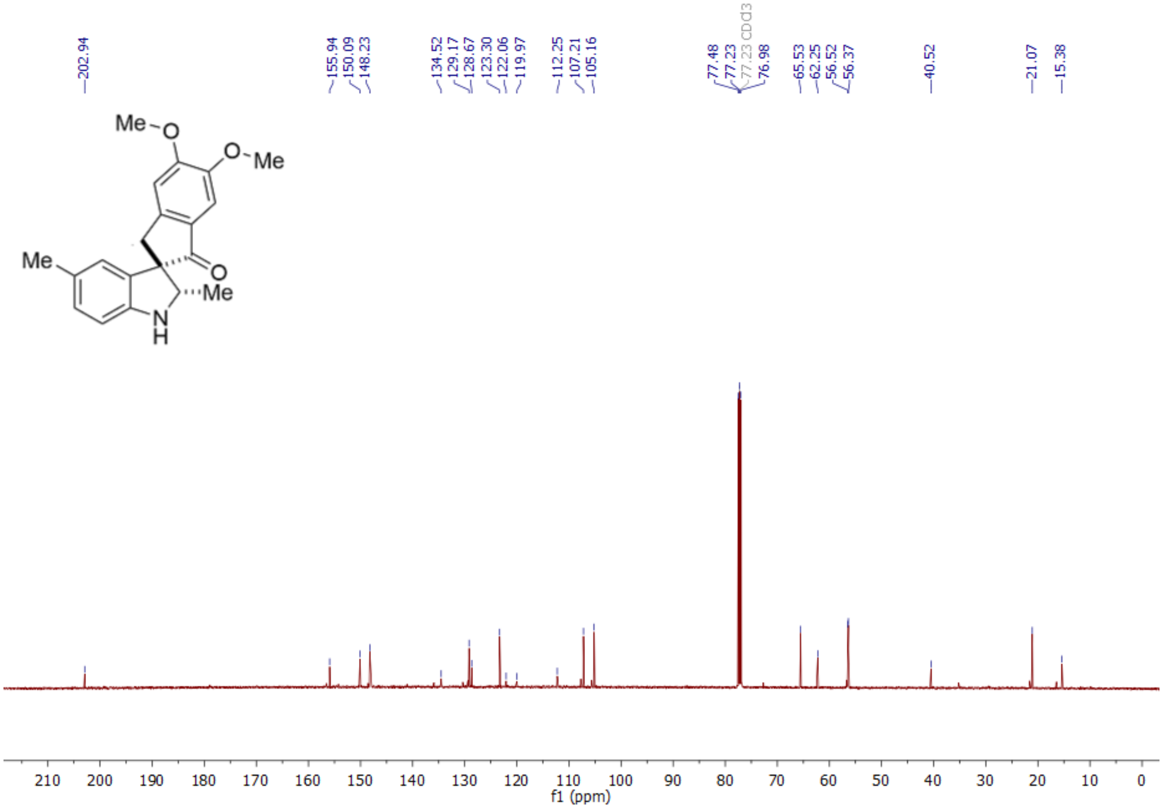

5'-fluoro-2'-methylspiro[indeno[5,6-d][1,3]dioxole-6,3'-indolin]-5(7H)-one [**B17**]:

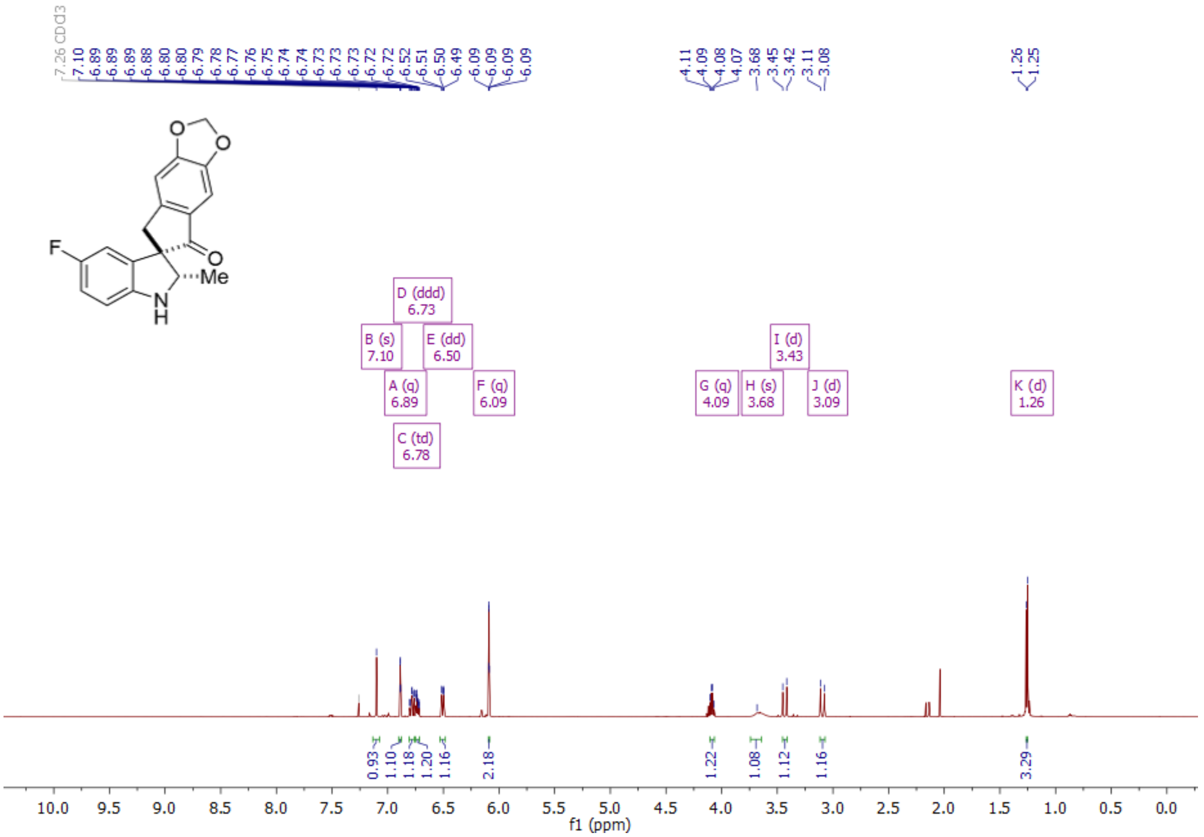

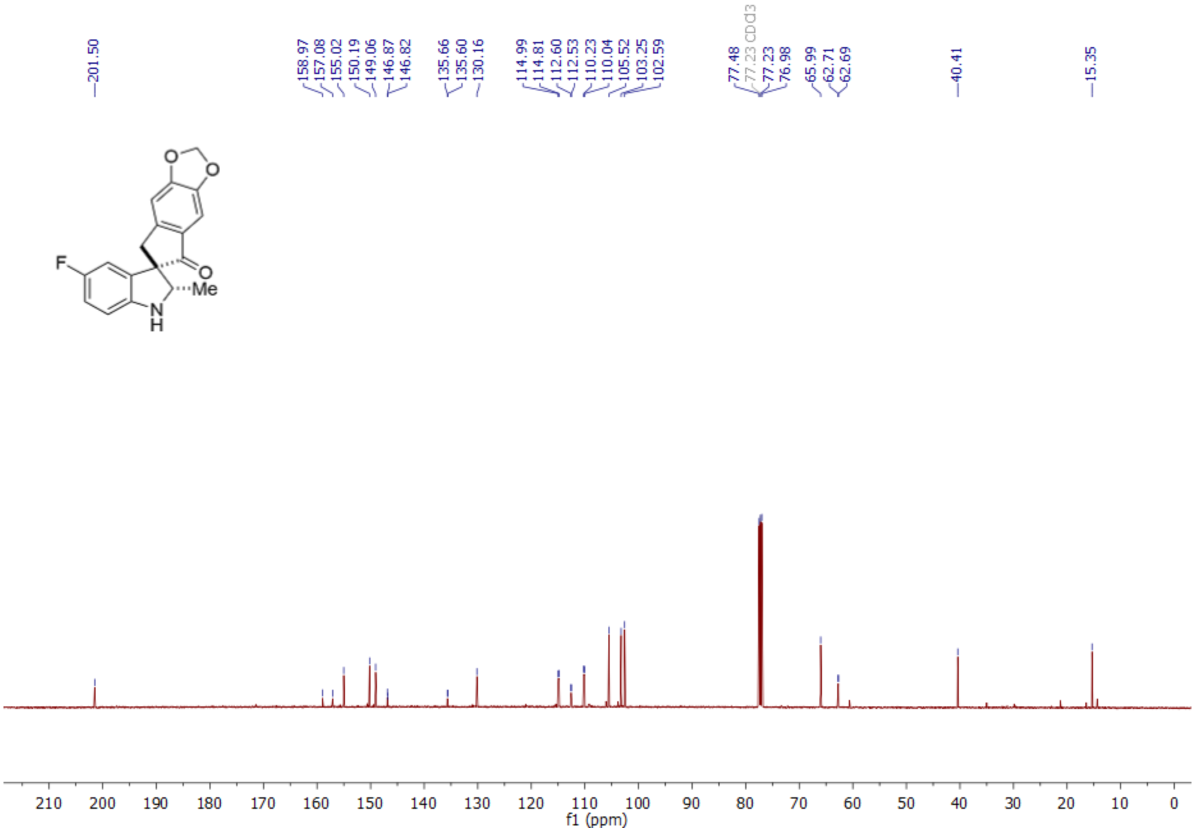

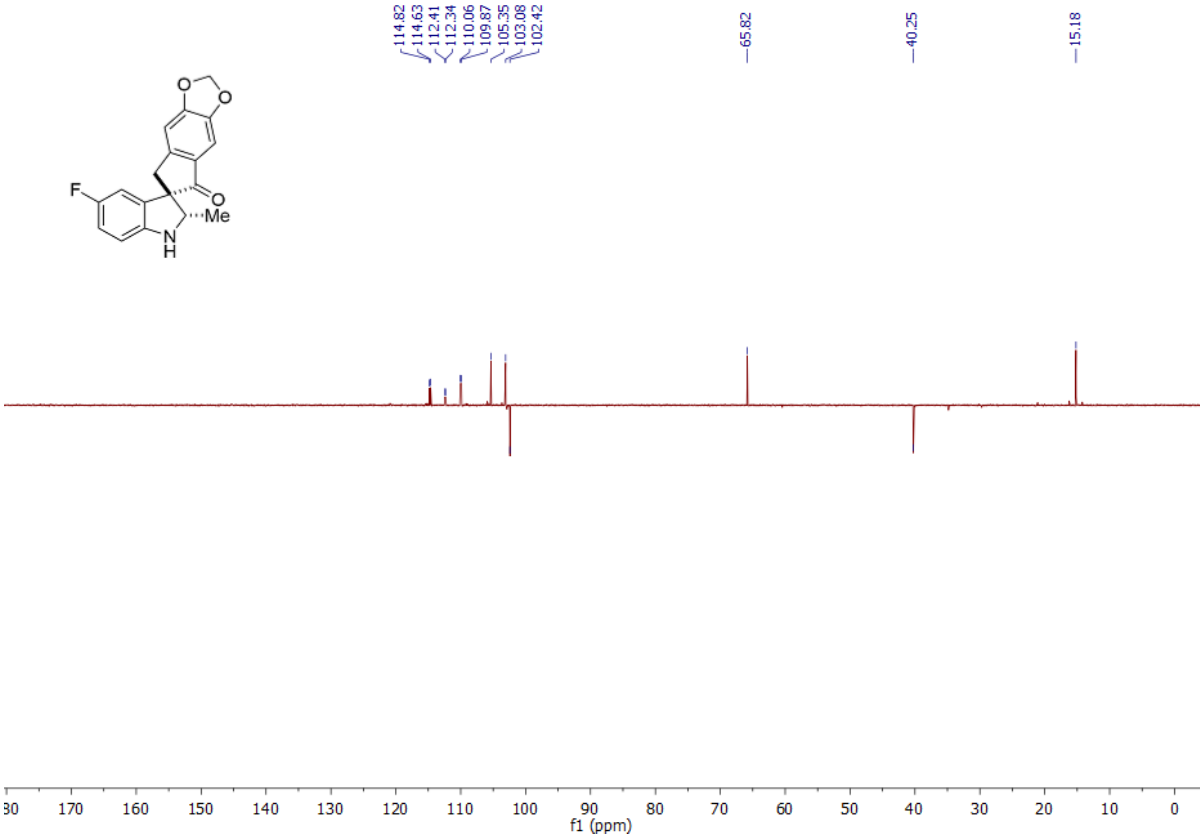
114.82
114.63
112.41
112.34
110.06
109.87
105.35
103.08
102.42
65.82
40.25
15.18
F
O
O
O
Me
N
H
f1 (ppm)

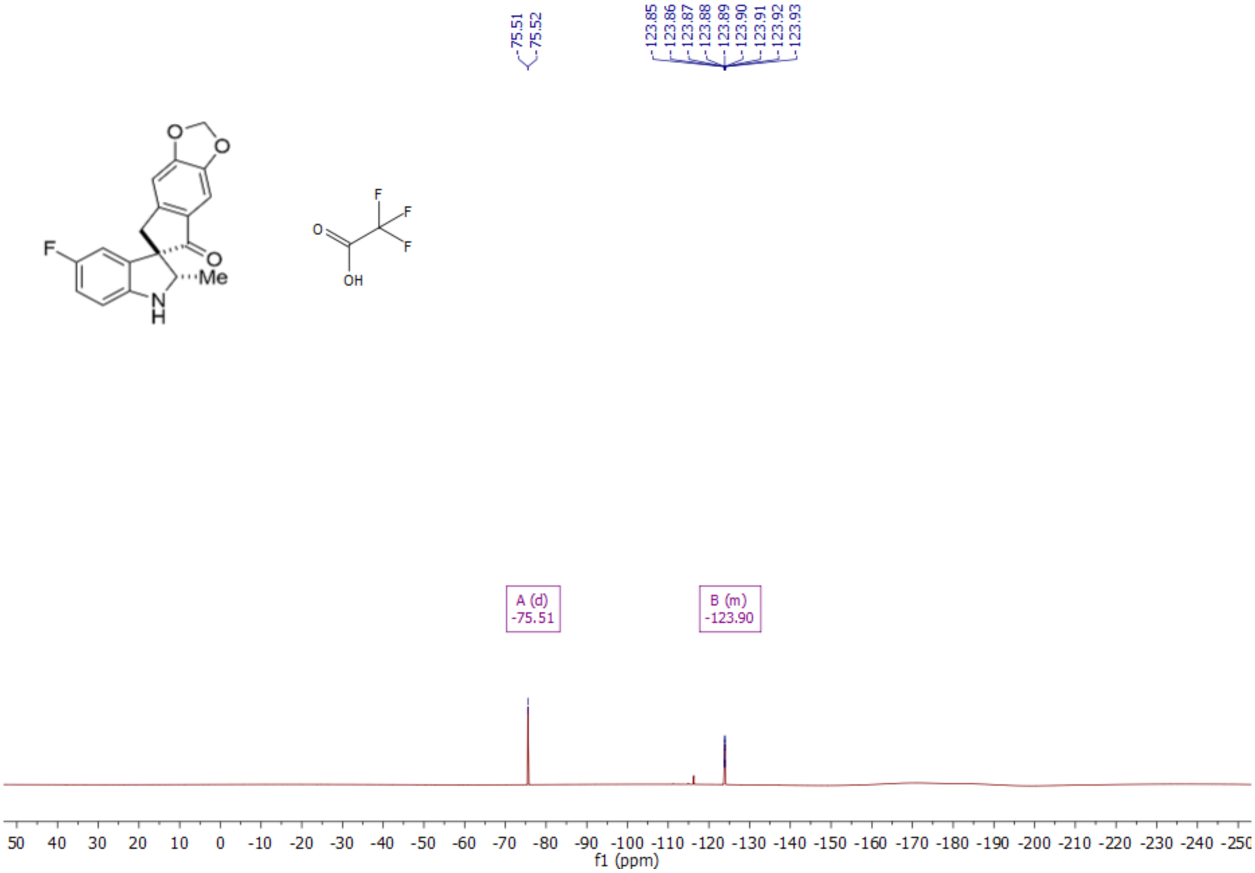
-75.51
-75.52
-123.85
-123.86
-123.87
-123.88
-123.89
-123.90
-123.91
-123.92
-123.93
F
O
O
O
Me
N
H
O
OH
F
F
F
A (d)
-75.51
B (m)
-123.90
f1 (ppm)

2',5'-dimethylspiro[indeno[5,6-d][1,3]dioxole-6,3'-indolin]-5(7H)-one [**B18**]:

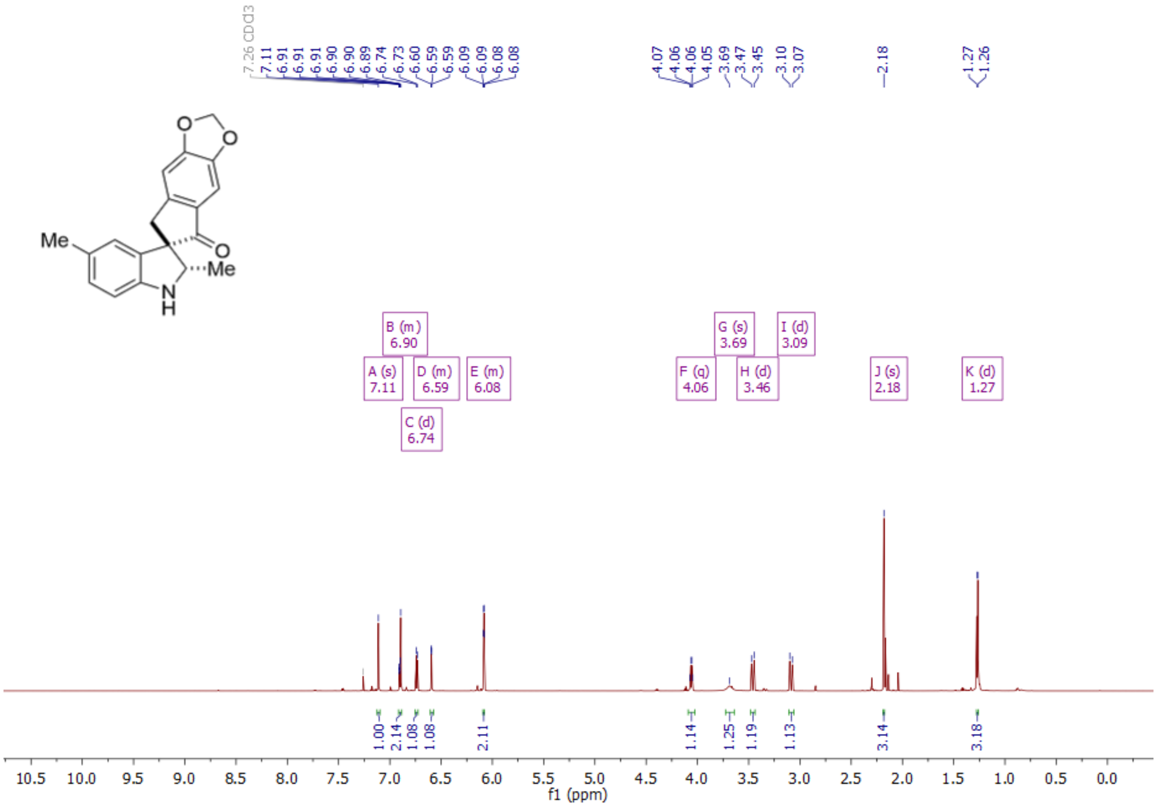

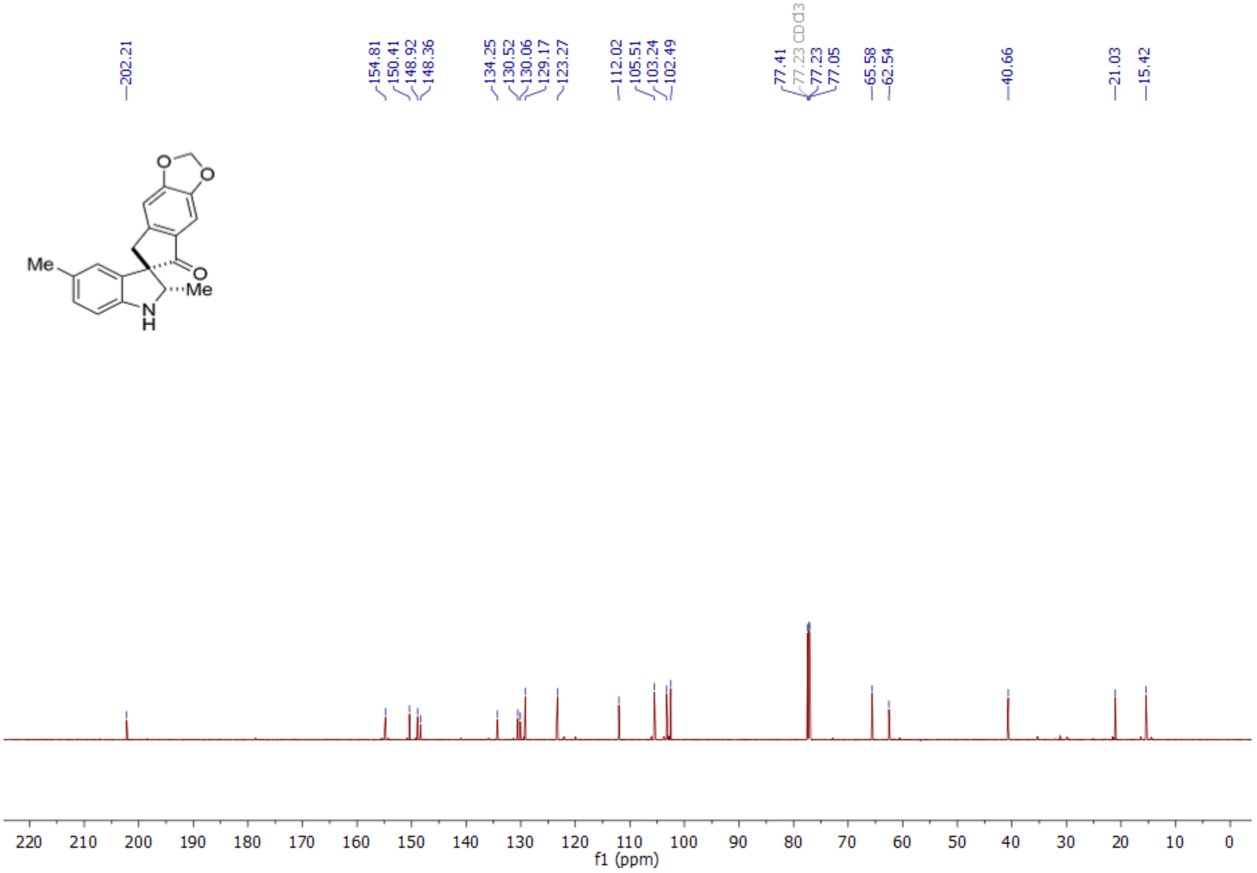

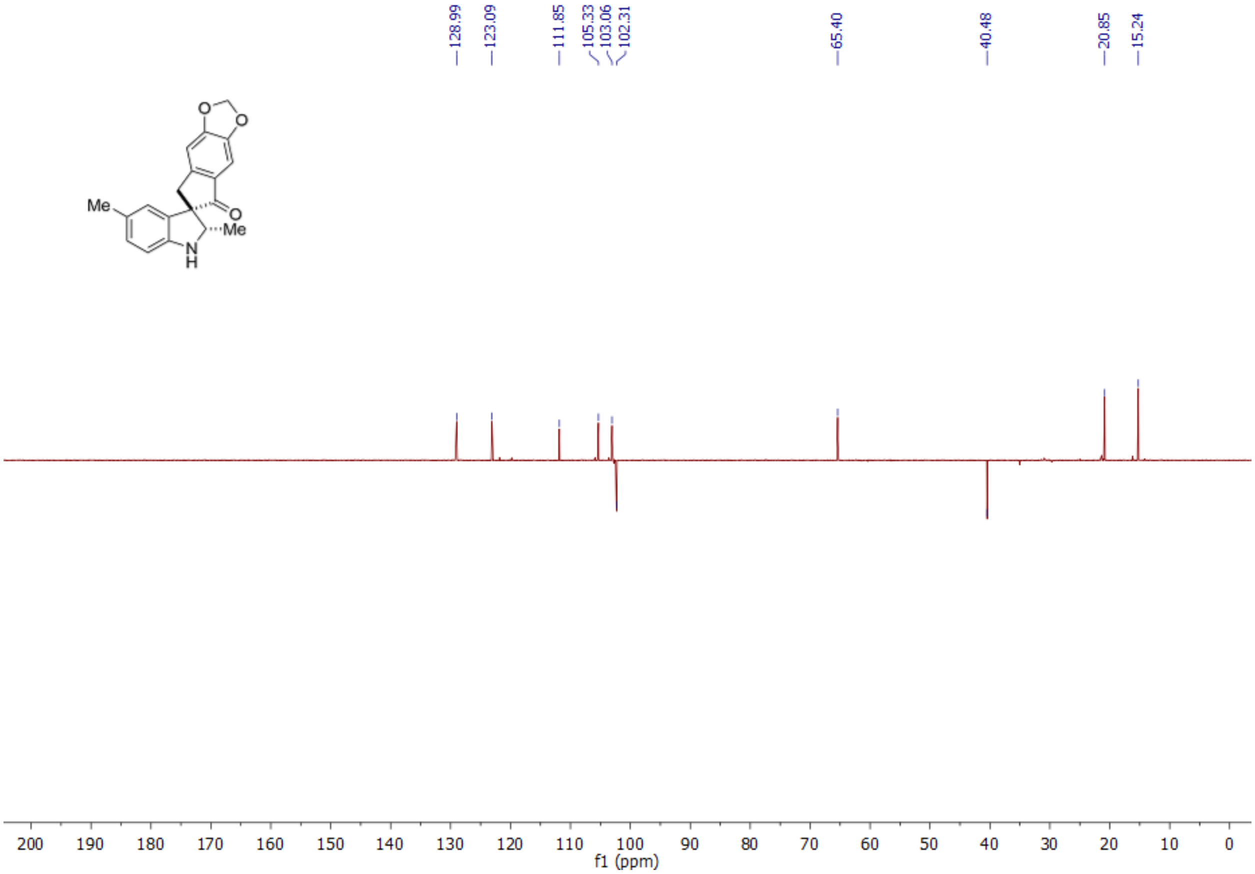
O
O
Me
O
Me
N
H
128.99
123.09
111.85
105.33
103.06
102.31
65.40
40.48
20.85
15.24
200 190 180 170 160 150 140 130 120 110 100 90 80 70 60 50 40 30 20 10 0
f1 (ppm)

CLASS-C

1'-(2-chloroacetyl)-2'-methylspiro[indene-2,3'-indolin]-1(3H)-one [C1]:

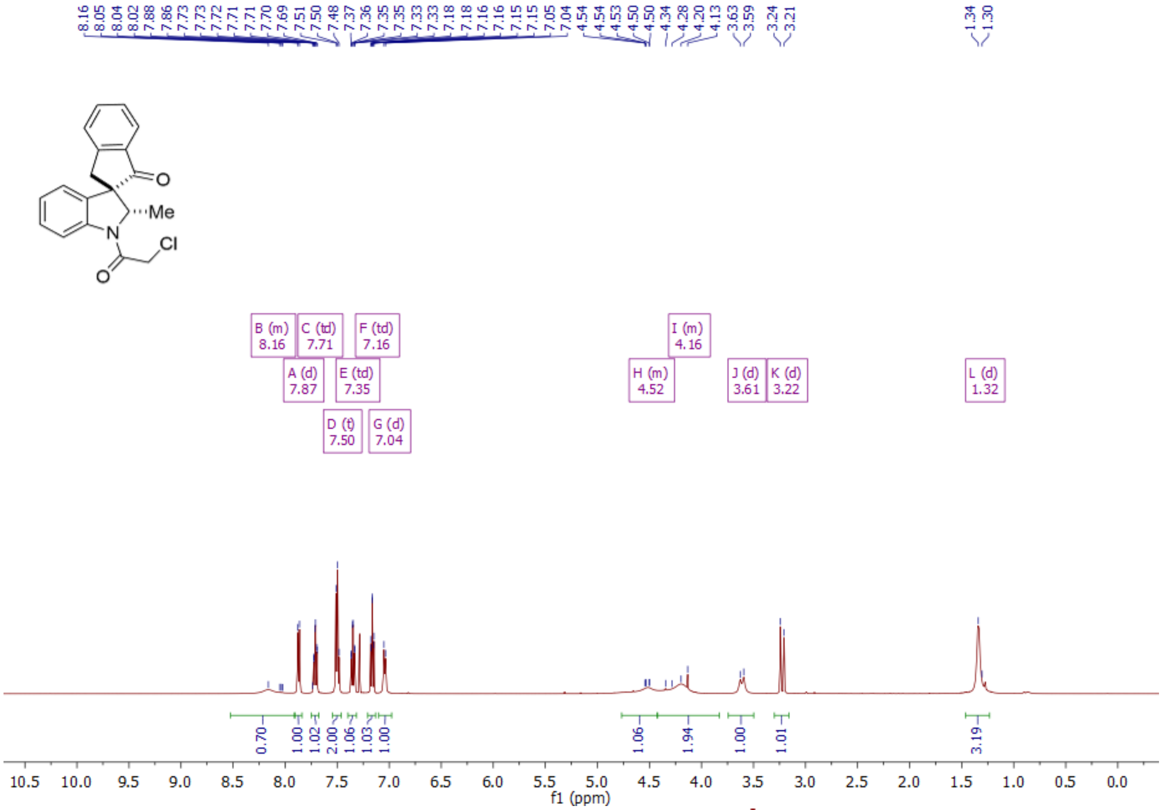

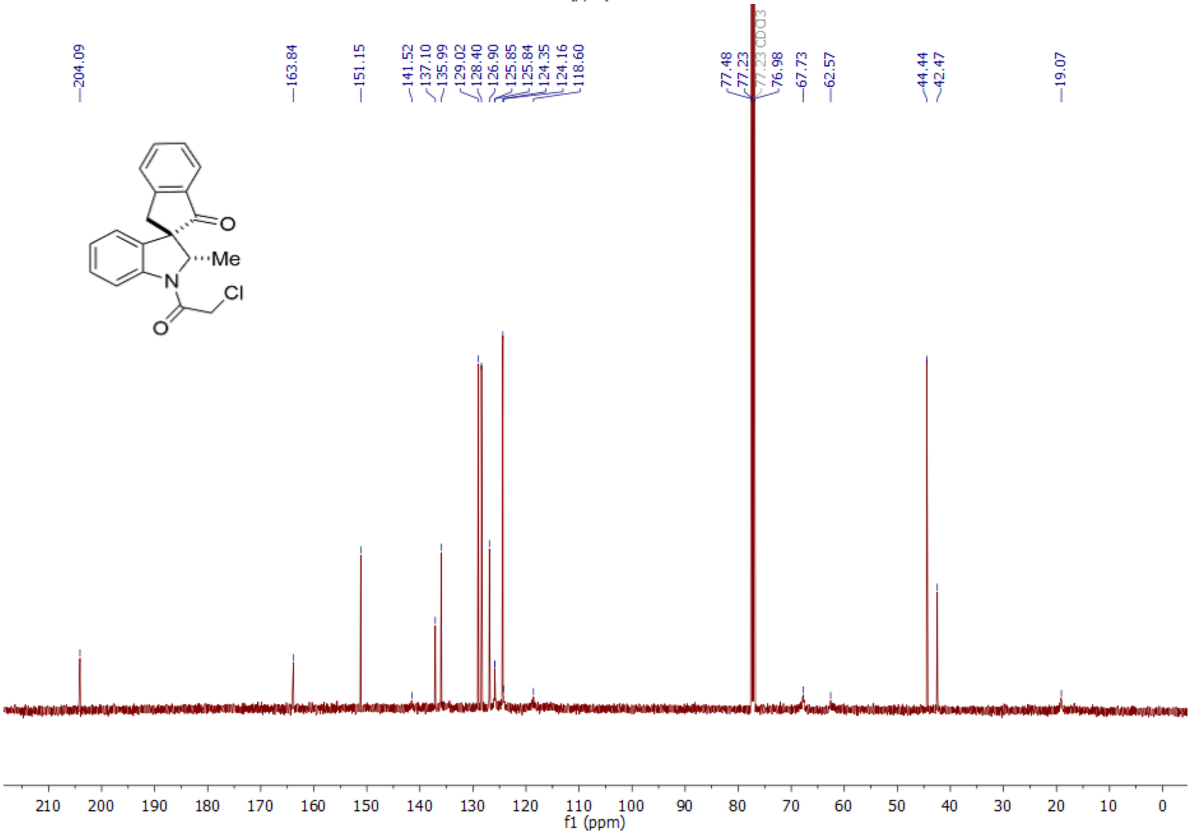

2'-methyl-1'-(2,2,2-trichloroacetyl)spiro[indene-2,3'-indolin]-1(3H)-one [C2]:

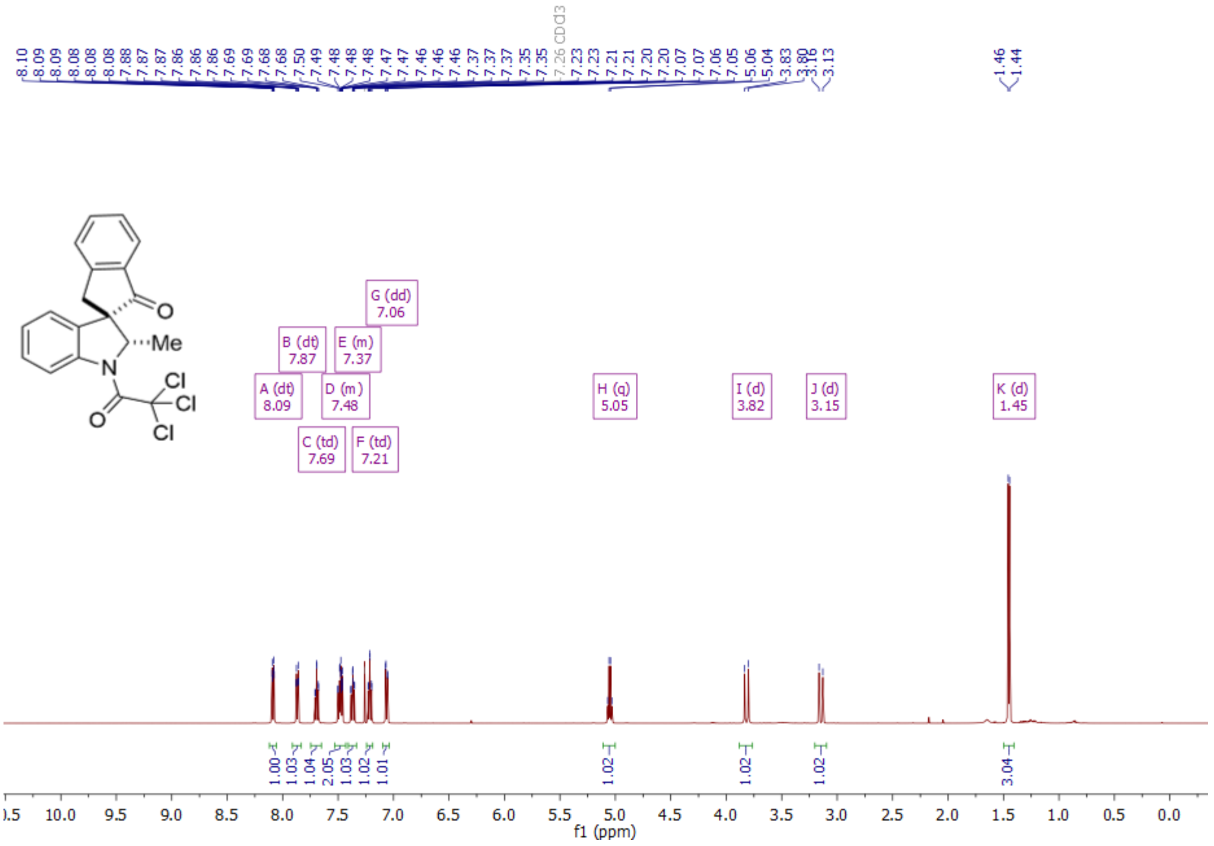

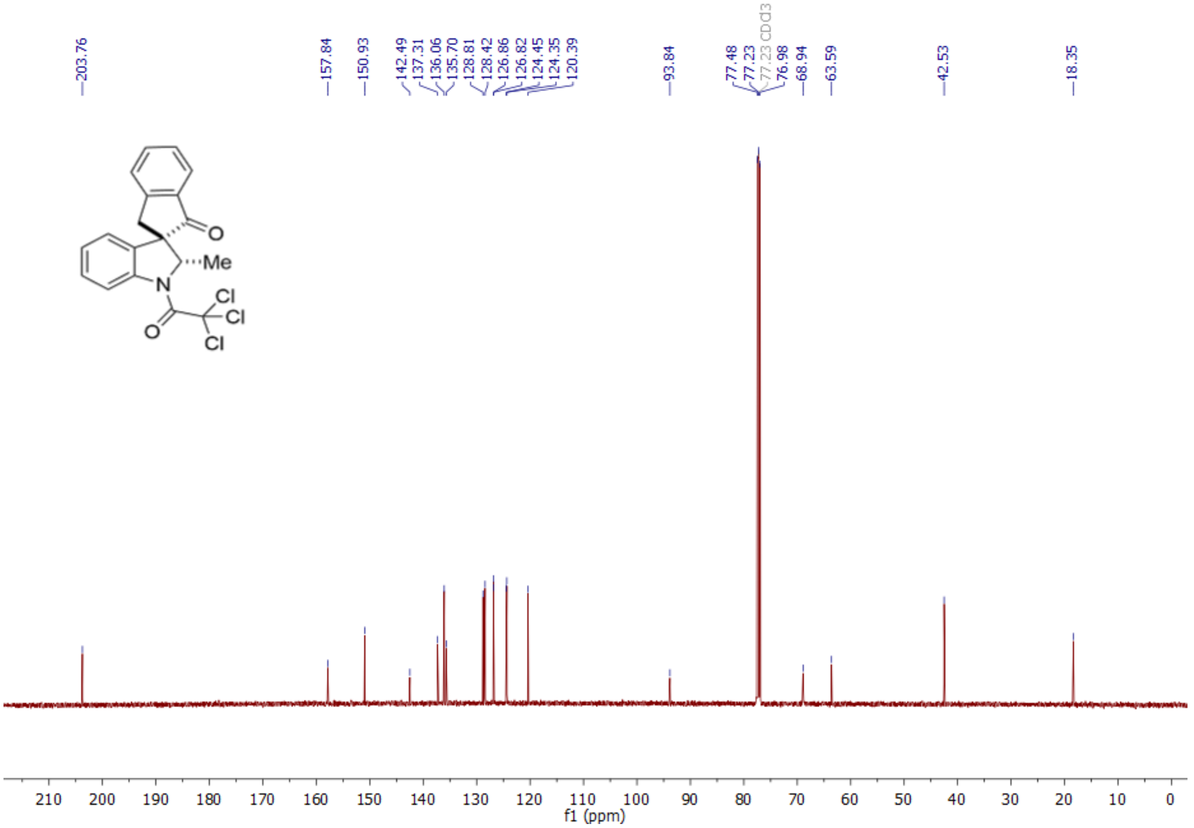

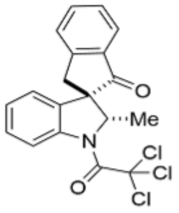

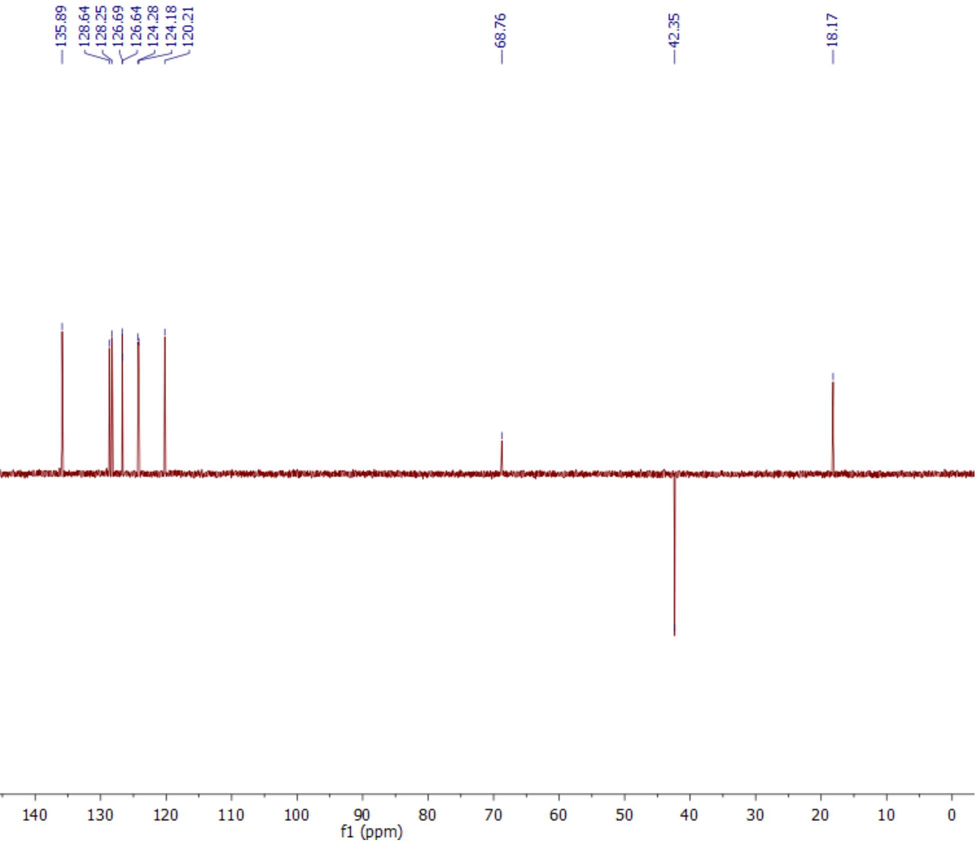

135.89
128.64
128.25
126.69
126.64
124.28
124.18
120.21
68.76
42.35
18.17
30 170 160 150 140 130 120 110 100 90 80 70 60 50 40 30 20 10 0
f1 (ppm)

1'-acryloyl-2'-methylspiro[indene-2,3'-indolin]-1(3H)-one [**C3**]:

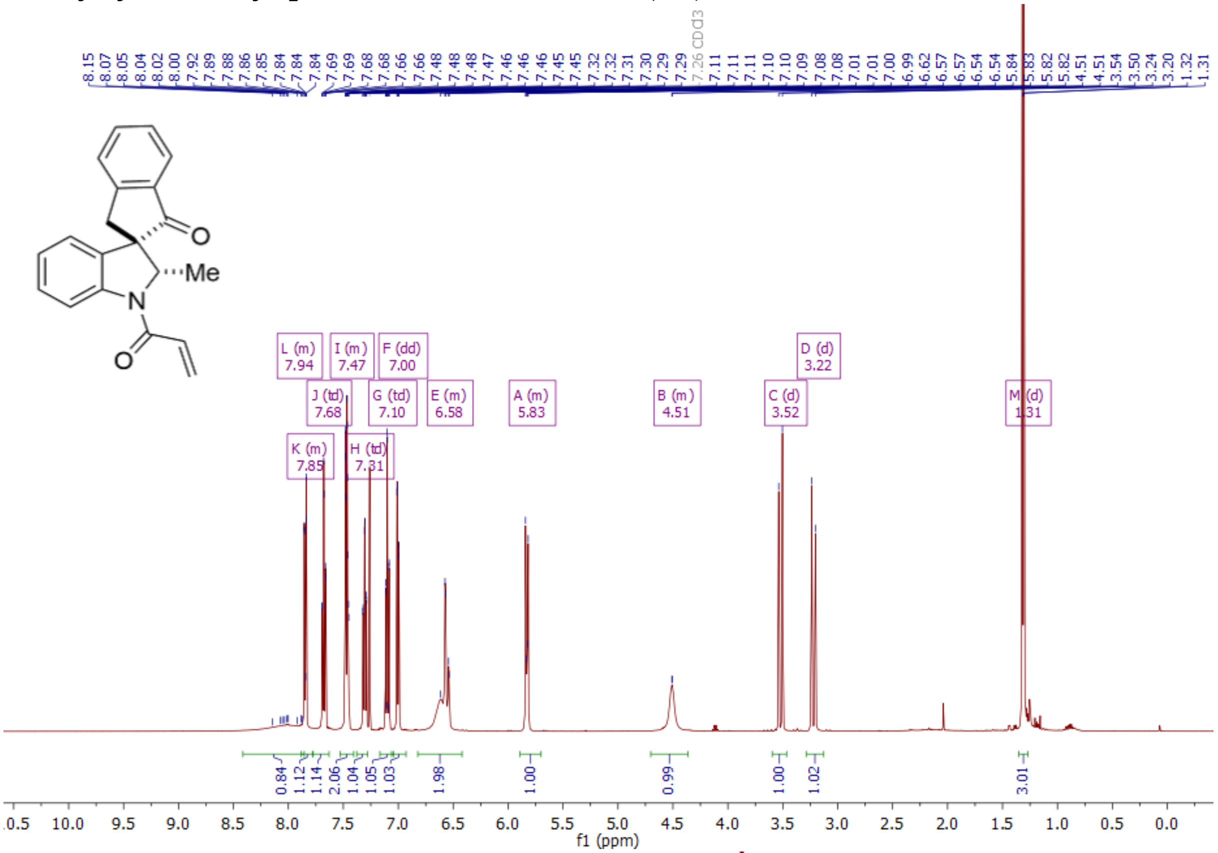

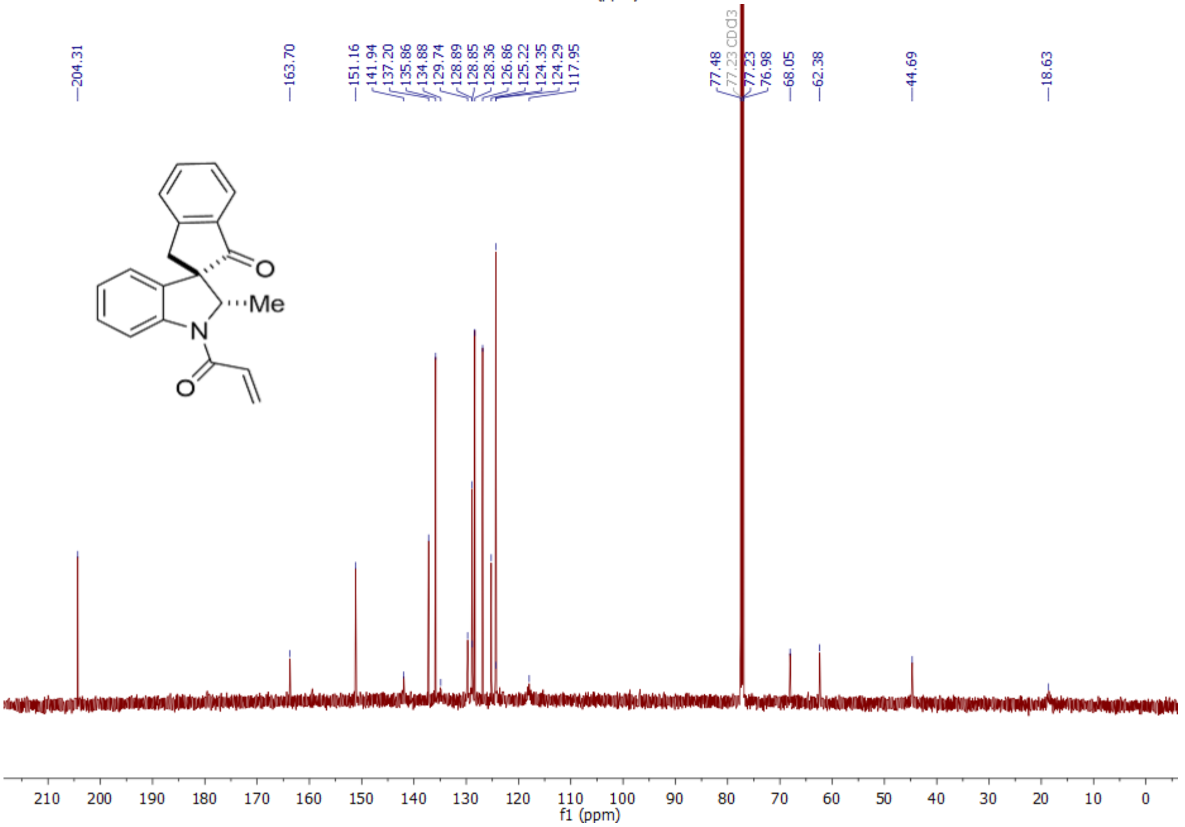

1'-acryloyl-2'-methylspiro[indene-2,3'-indolin]-1(3H)-one **[C3-resynthesized]**:

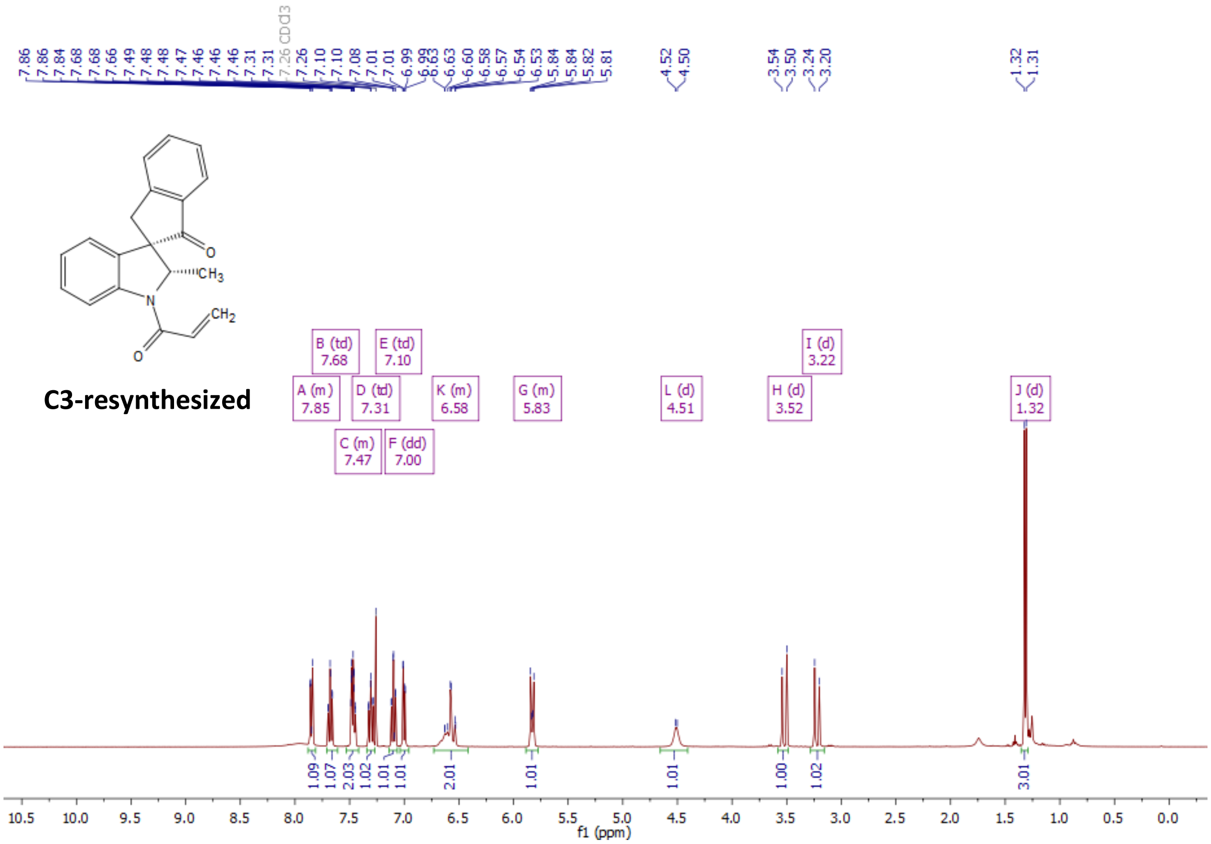

ethyl 2'-methyl-1-oxo-1,3-dihydrospiro[indene-2,3'-indoline]-1'-carboxylate [**C4**]:

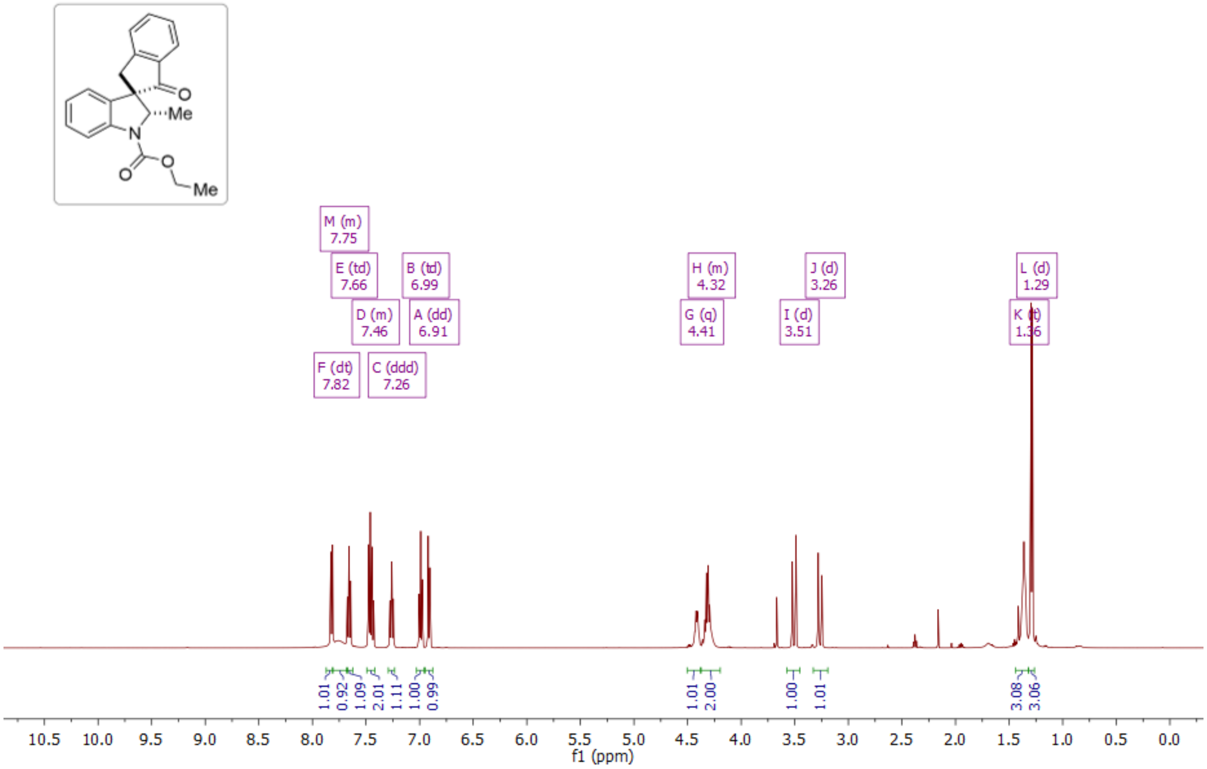

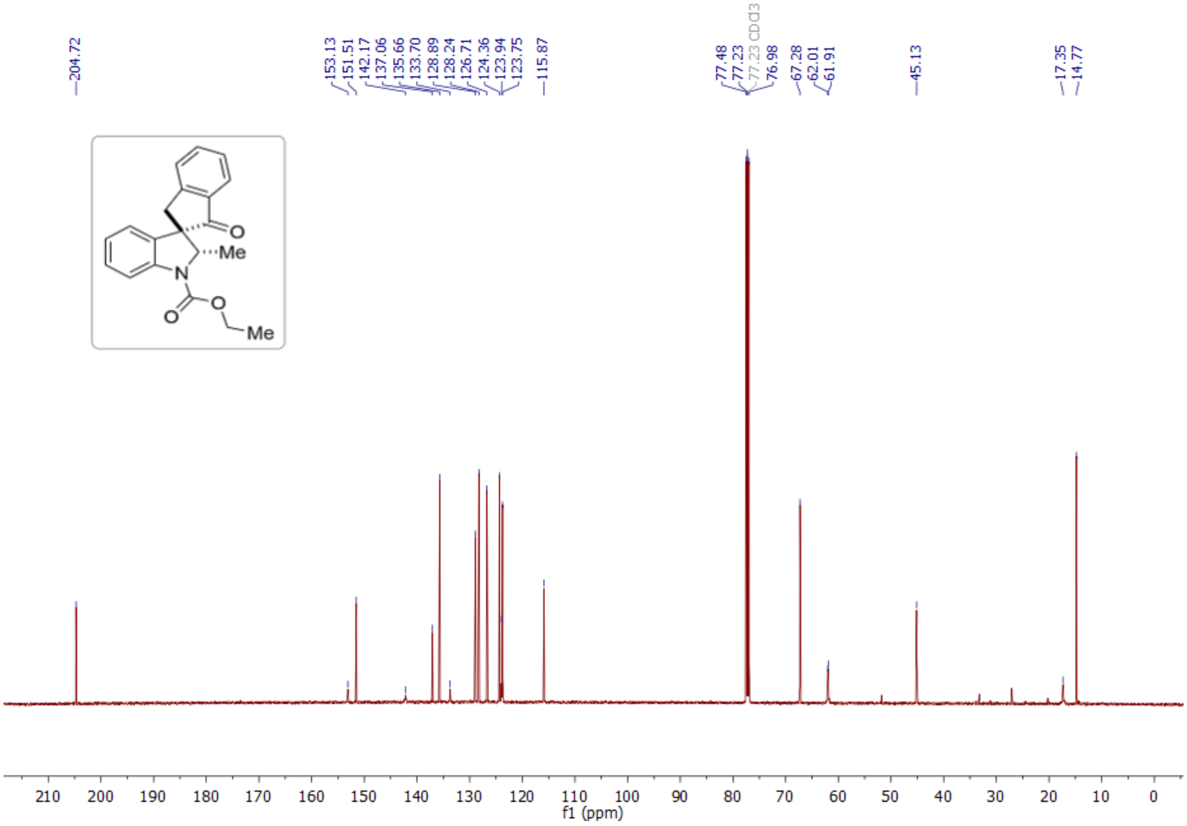

1'-allyl-2'-methylspiro[indene-2,3'-indolin]-1(3H)-one [C5]:

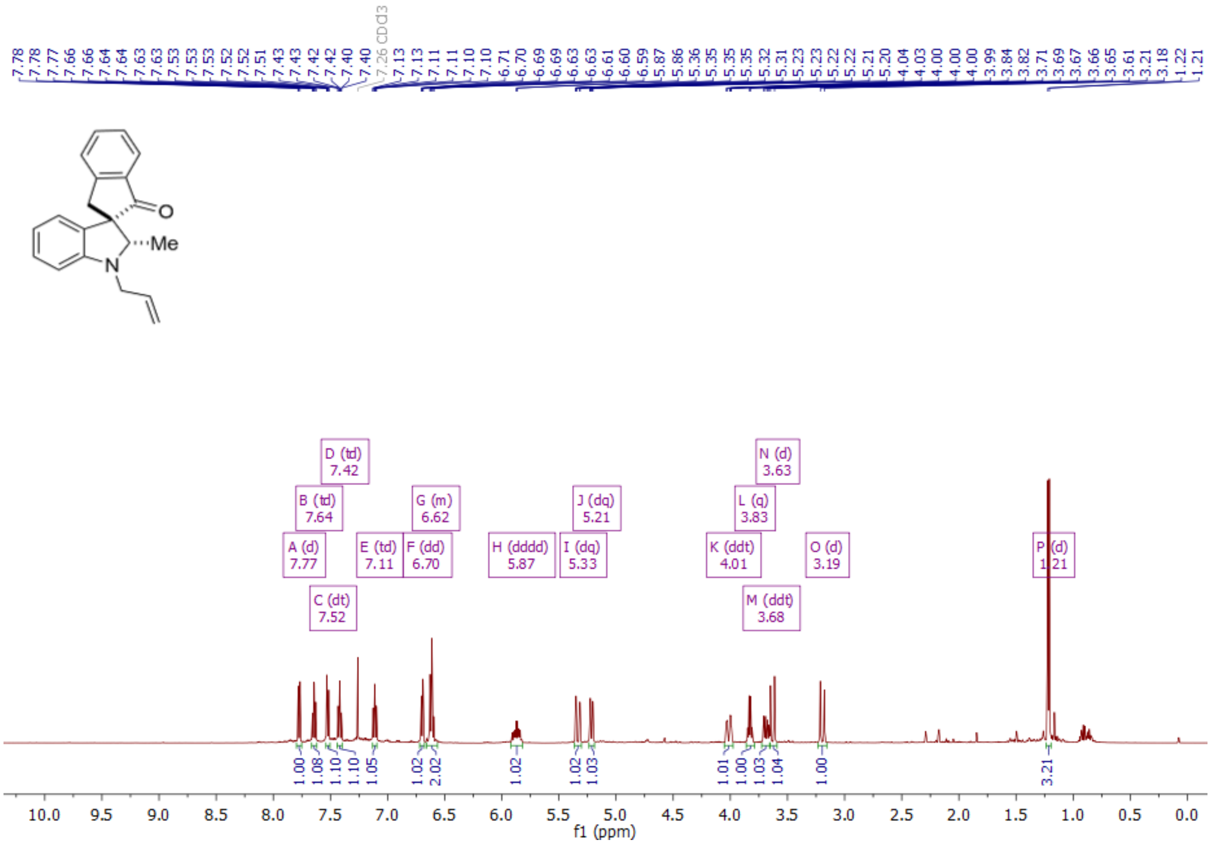

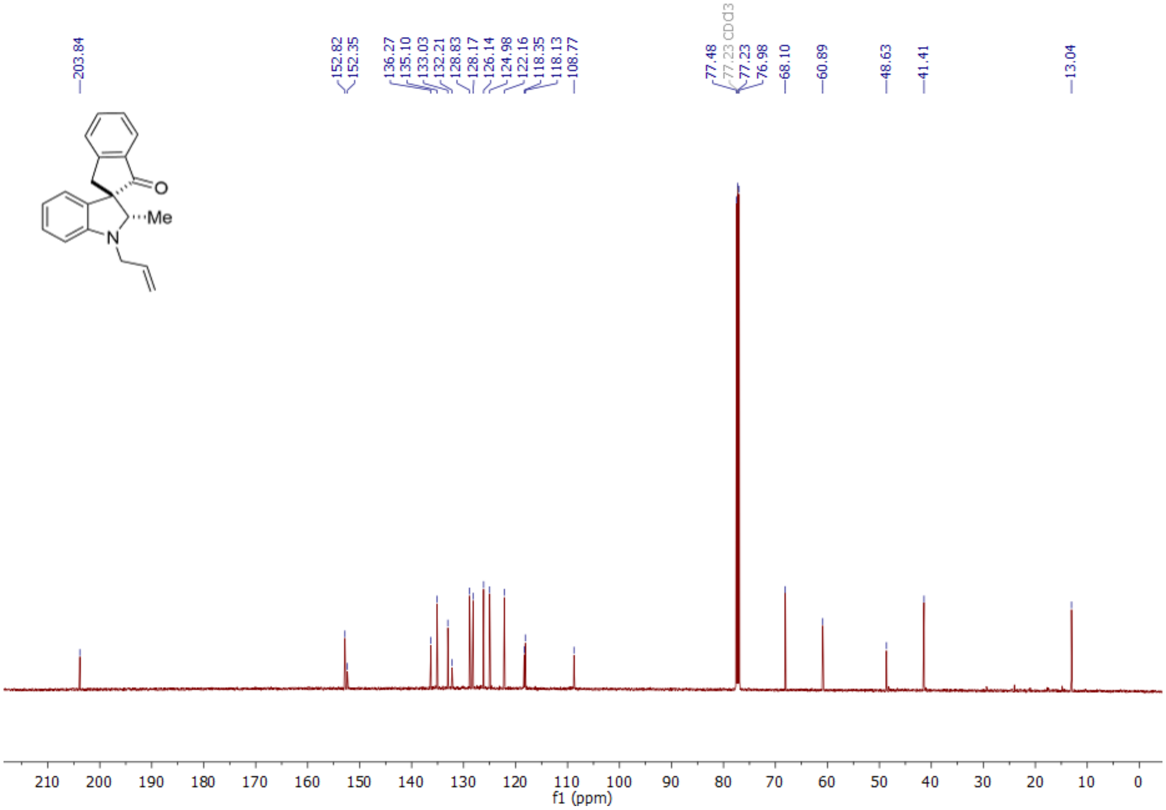

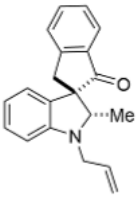

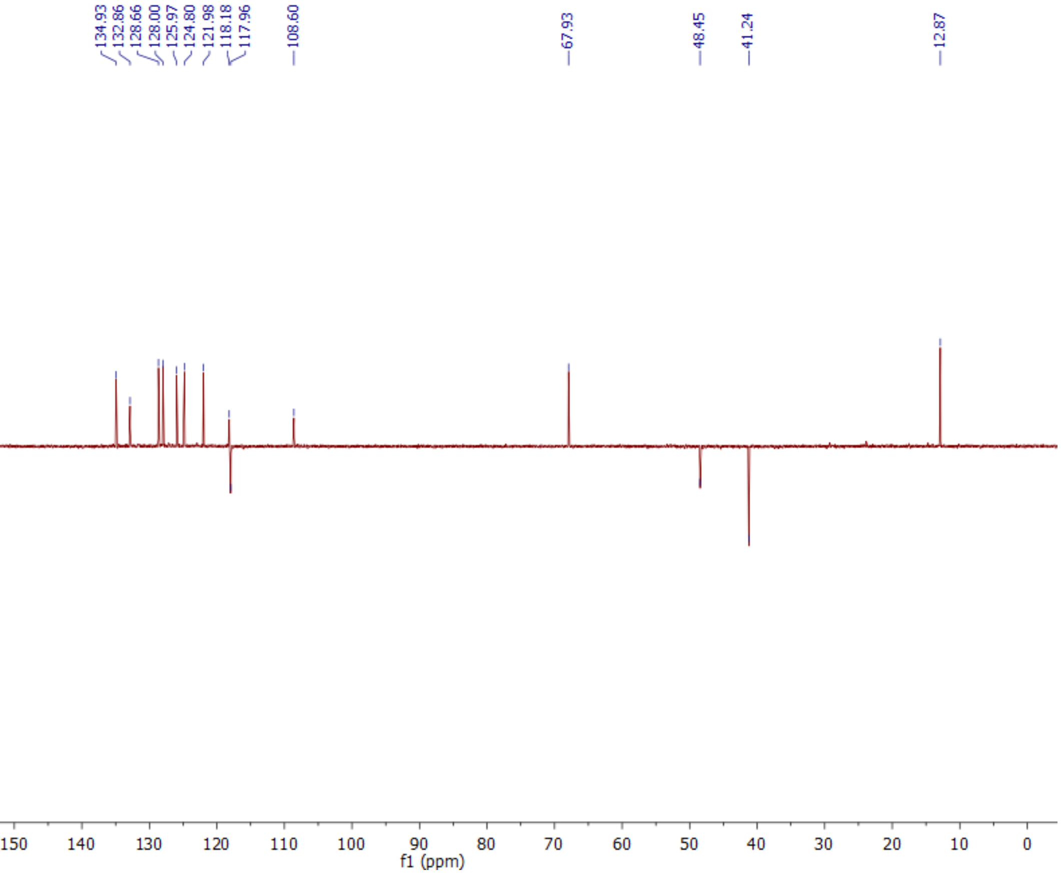

134.93
132.86
128.66
128.00
125.97
124.80
121.98
118.18
117.96
108.60
67.93
48.45
41.24
12.87
30
170
160
150
140
130
120
110
100
90
80
70
60
50
40
30
20
10
0
f1 (ppm)

1'-(3-bromobenzyl)-2'-methylspiro[indene-2,3'-indolin]-1(3H)-one [**C6**]:

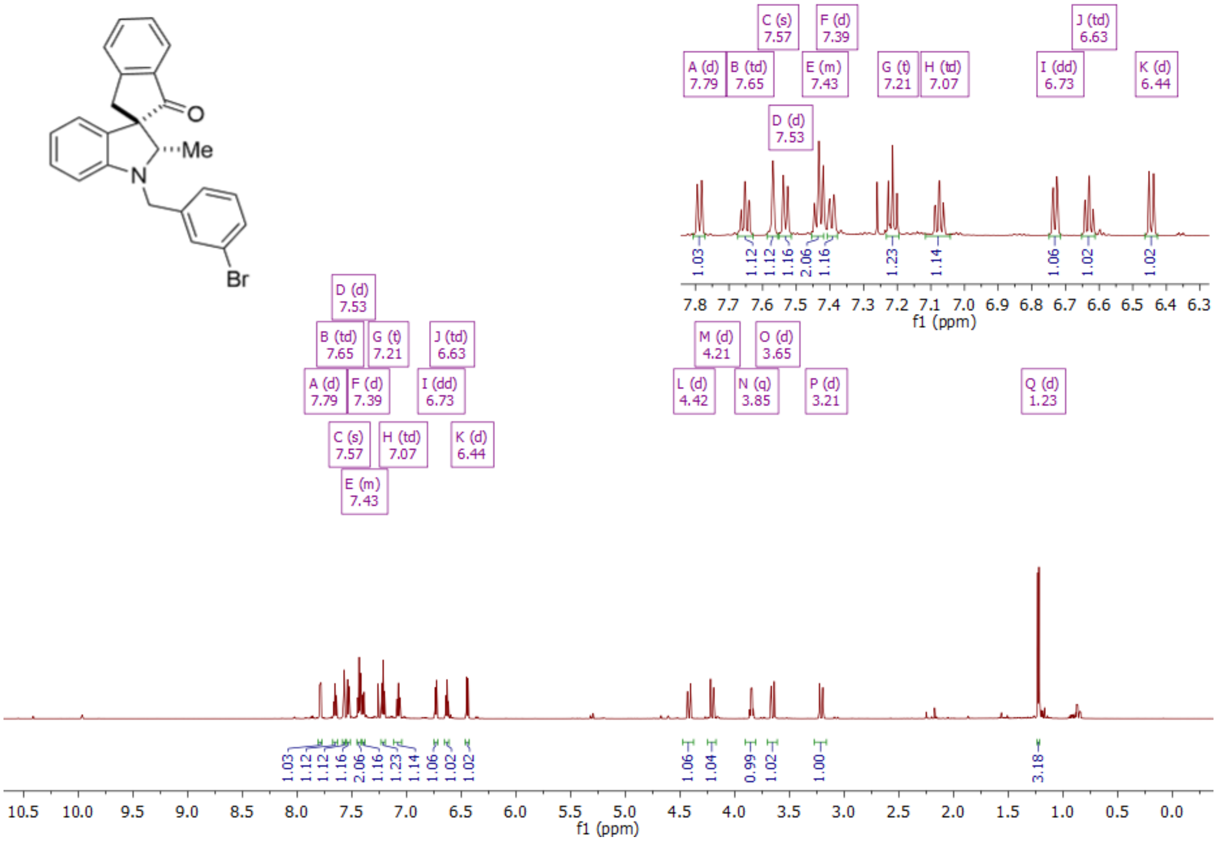

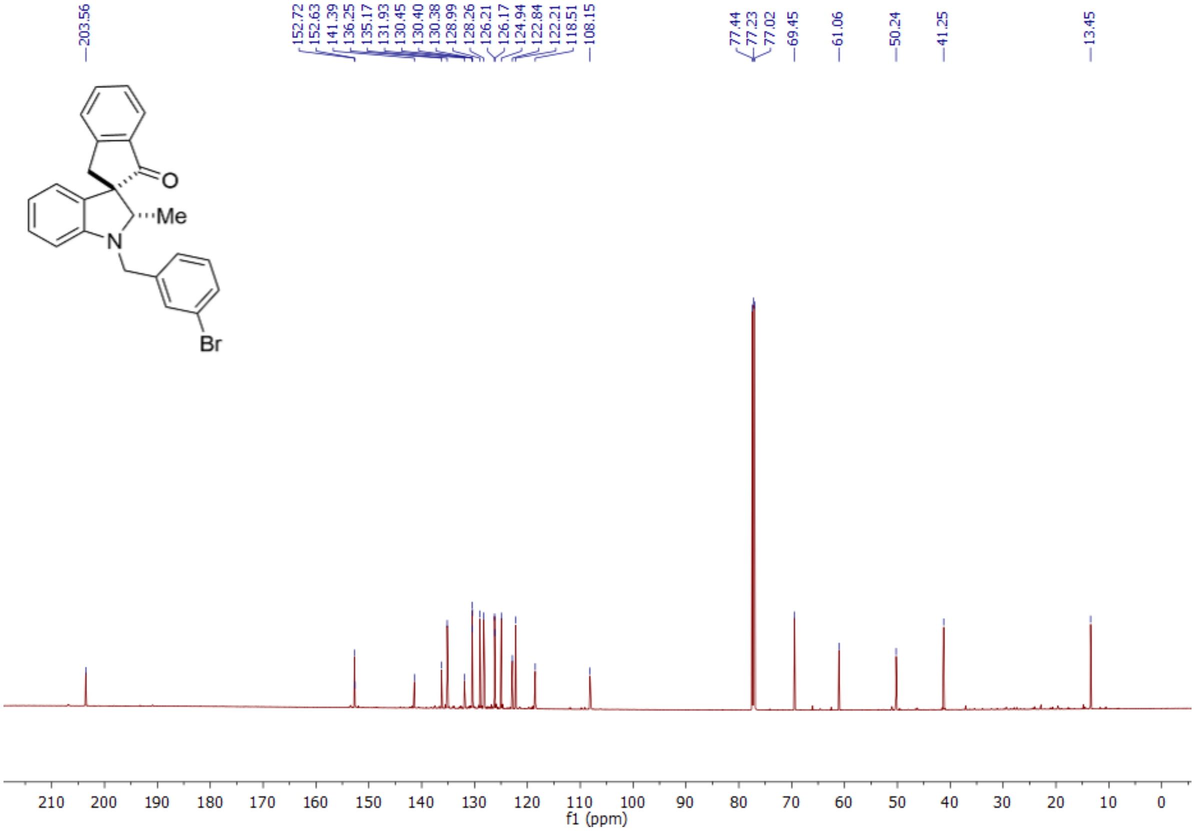

5-fluoro-2'-methyl-1'-(prop-2-yn-1-yl)spiro[indene-2,3'-indolin]-1(3H)-one [C7]:

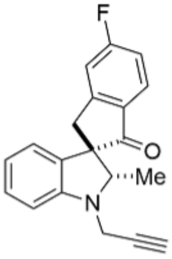

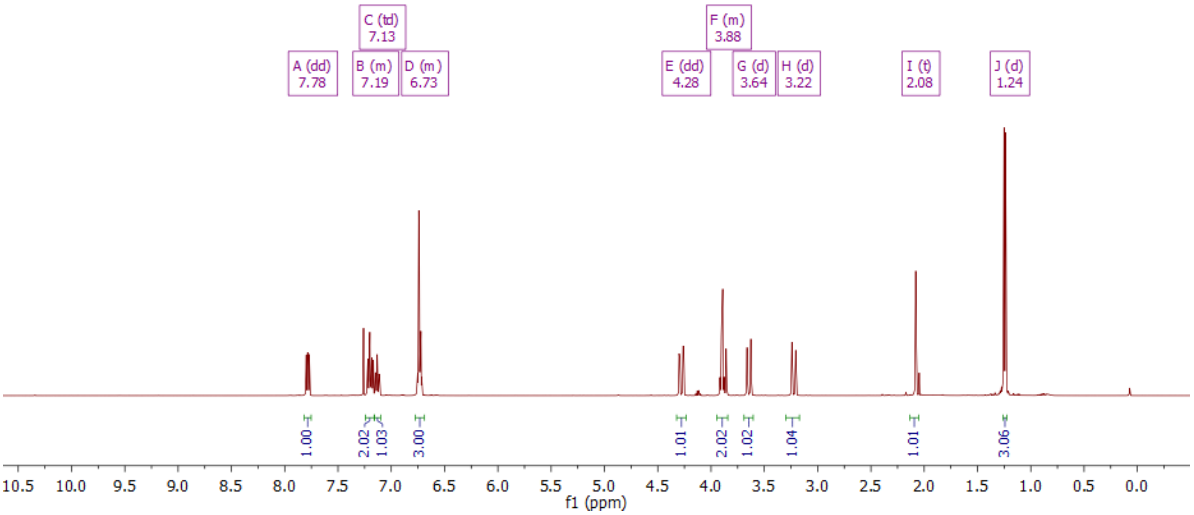

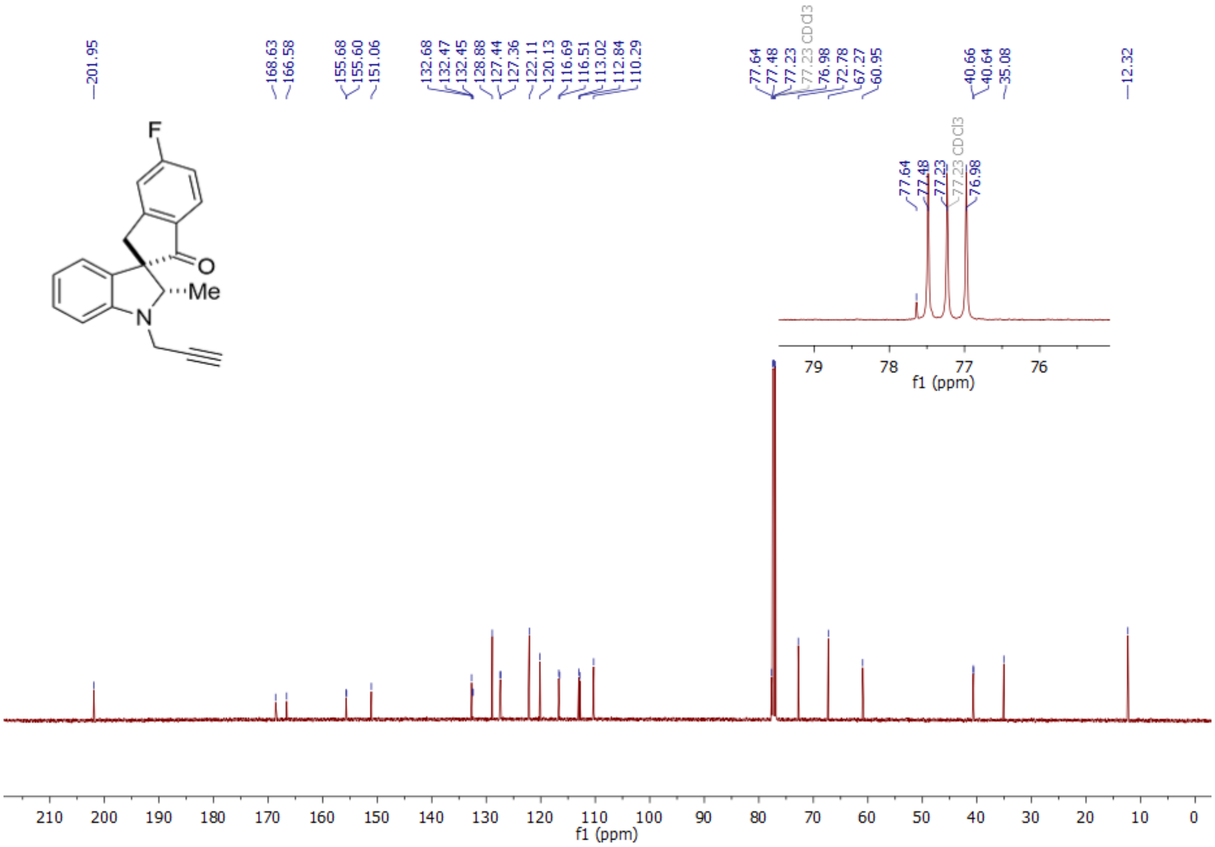

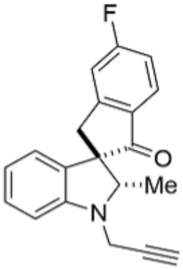

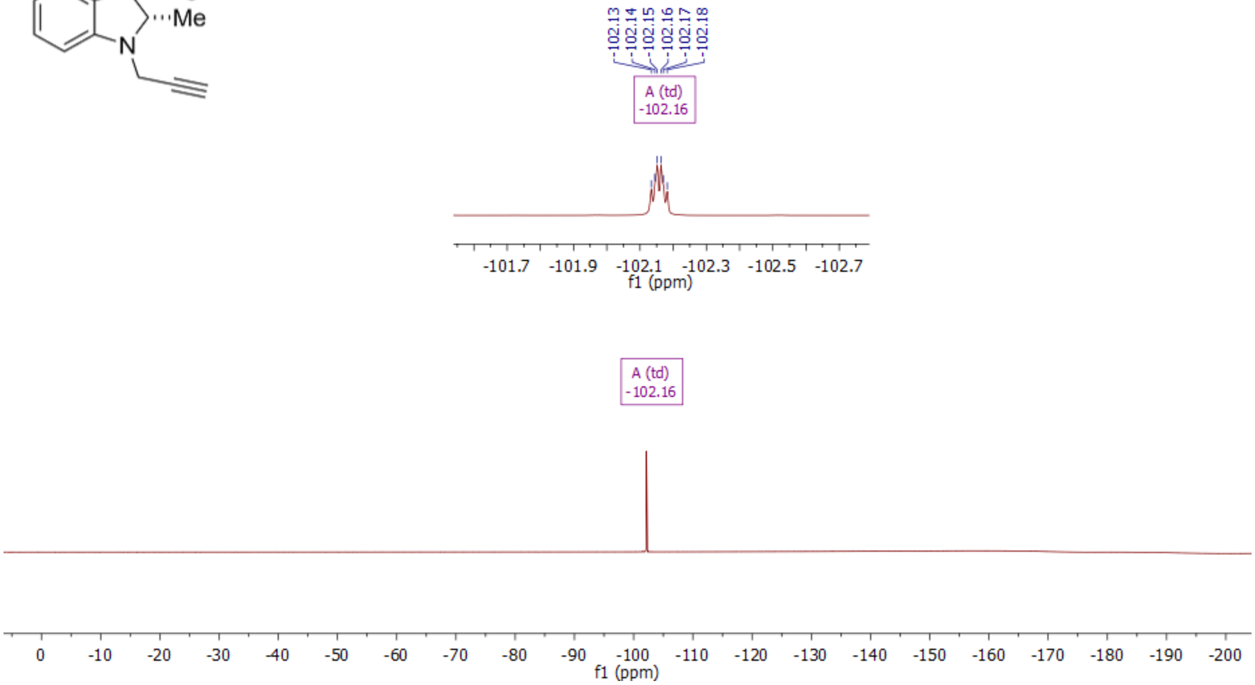
-102.13
-102.14
-102.15
-102.16
-102.17
-102.18
A (td)
-102.16
-101.7
-101.9
-102.1
-102.3
-102.5
-102.7
f1 (ppm)
A (td)
-102.16
0
-10
-20
-30
-40
-50
-60
-70
-80
-90
-100
-110
-120
-130
-140
-150
-160
-170
-180
-190
-200
f1 (ppm)

2'-methyl-1'-tosylspiro[indene-2,3'-indolin]-1(3H)-one [**C8**]:

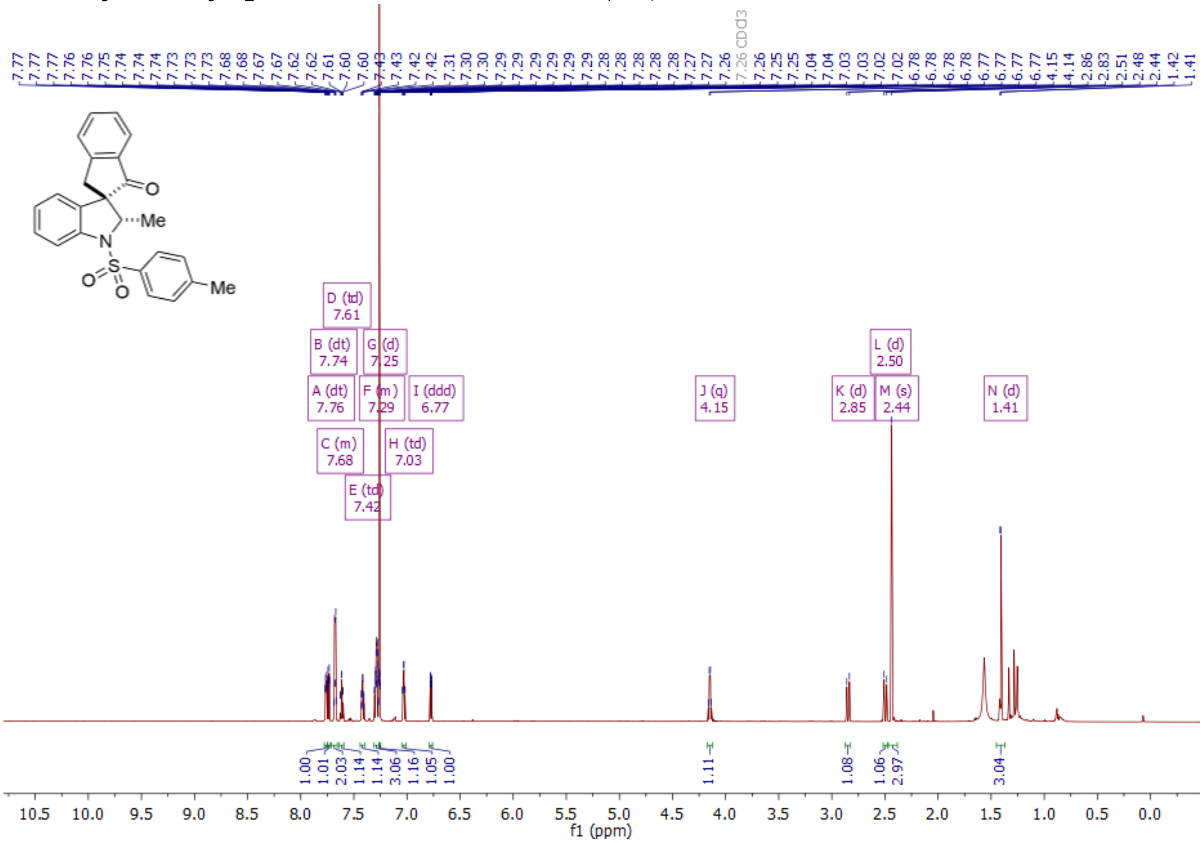

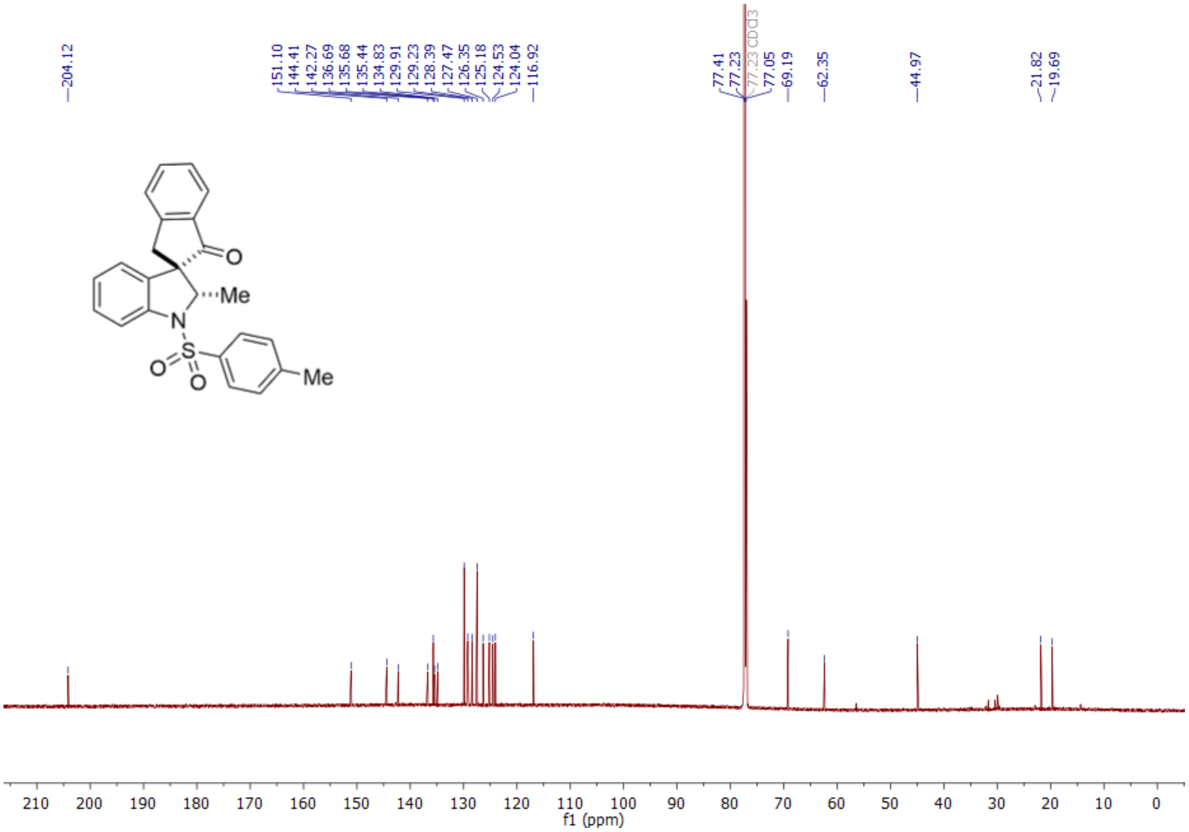

4-fluoro-2'-methyl-1'-tosylspiro[indene-2,3'-indolin]-1(3H)-one [C9]:

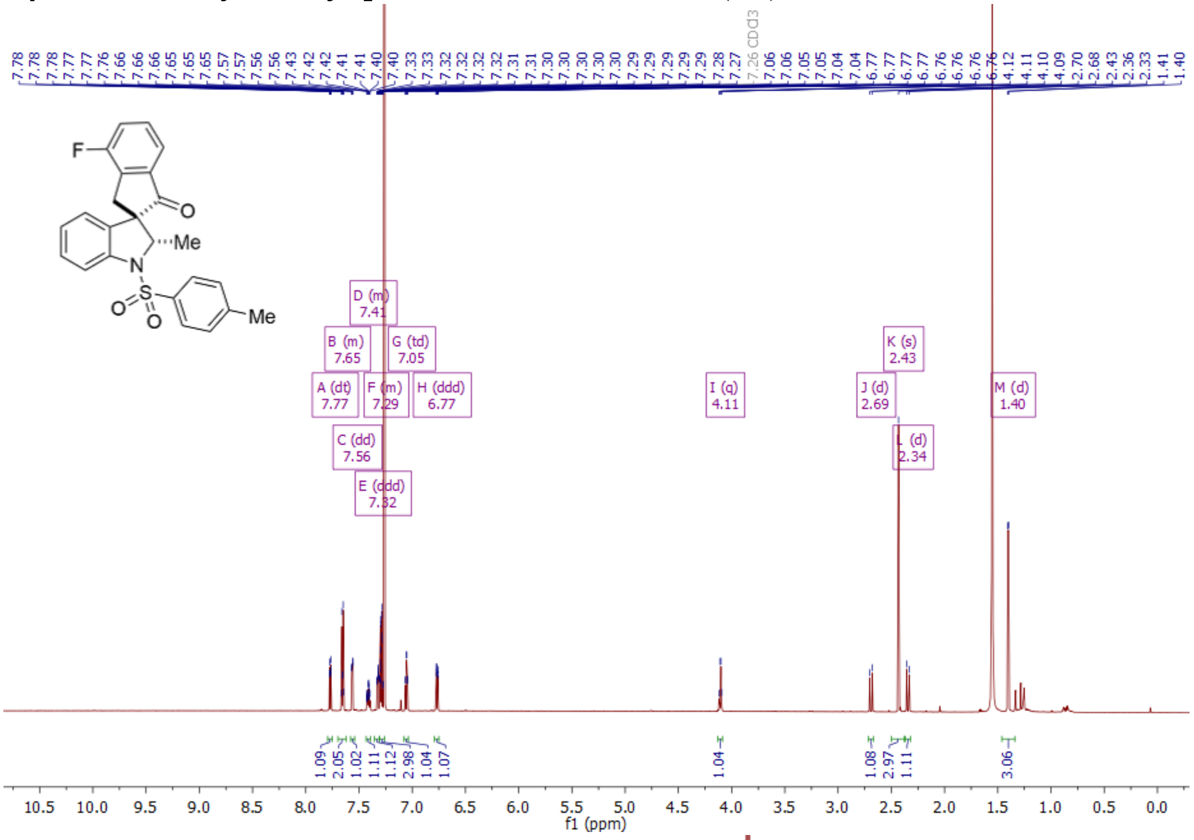

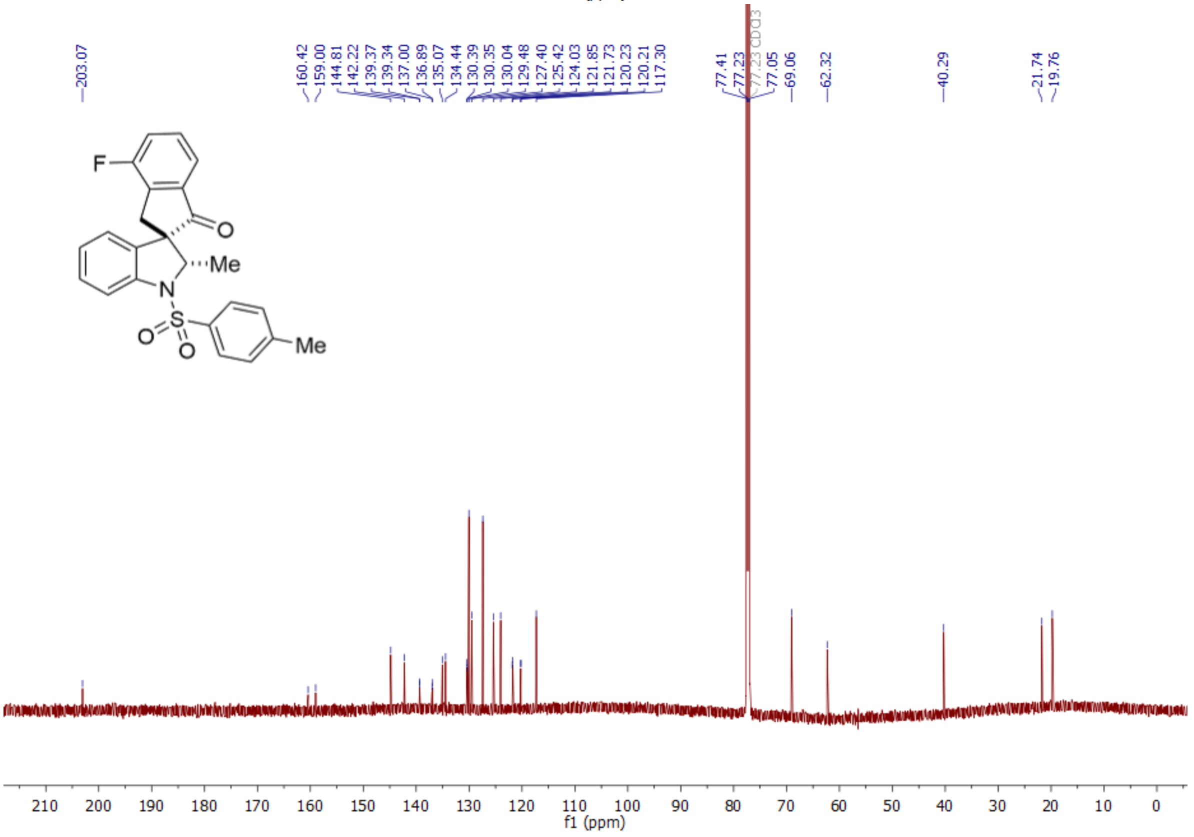

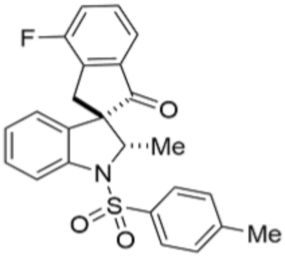
F
O
Me
N
S
O
O
Me

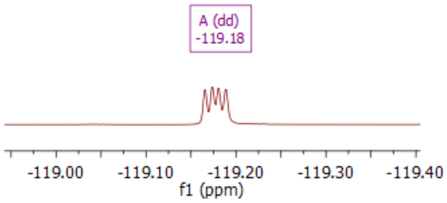
A (dd)
-119.18
-119.00
-119.10
-119.20
-119.30
-119.40
f1 (ppm)

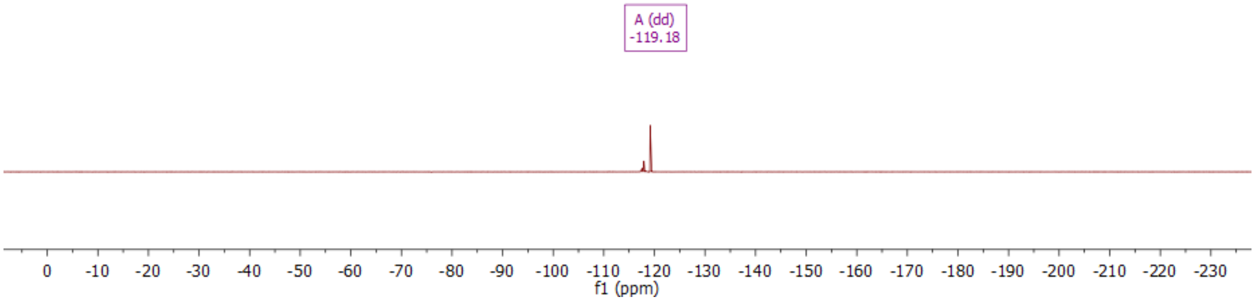
A (dd)
-119.18
0
-10
-20
-30
-40
-50
-60
-70
-80
-90
-100
-110
-120
-130
-140
-150
-160
-170
-180
-190
-200
-210
-220
-230
f1 (ppm)

***1'-((4-(tert-butyl)phenyl)sulfonyl)-6-fluoro-2'-methylspiro[indene-2,3'-indolin]-1(3H)-one* [C10]:**

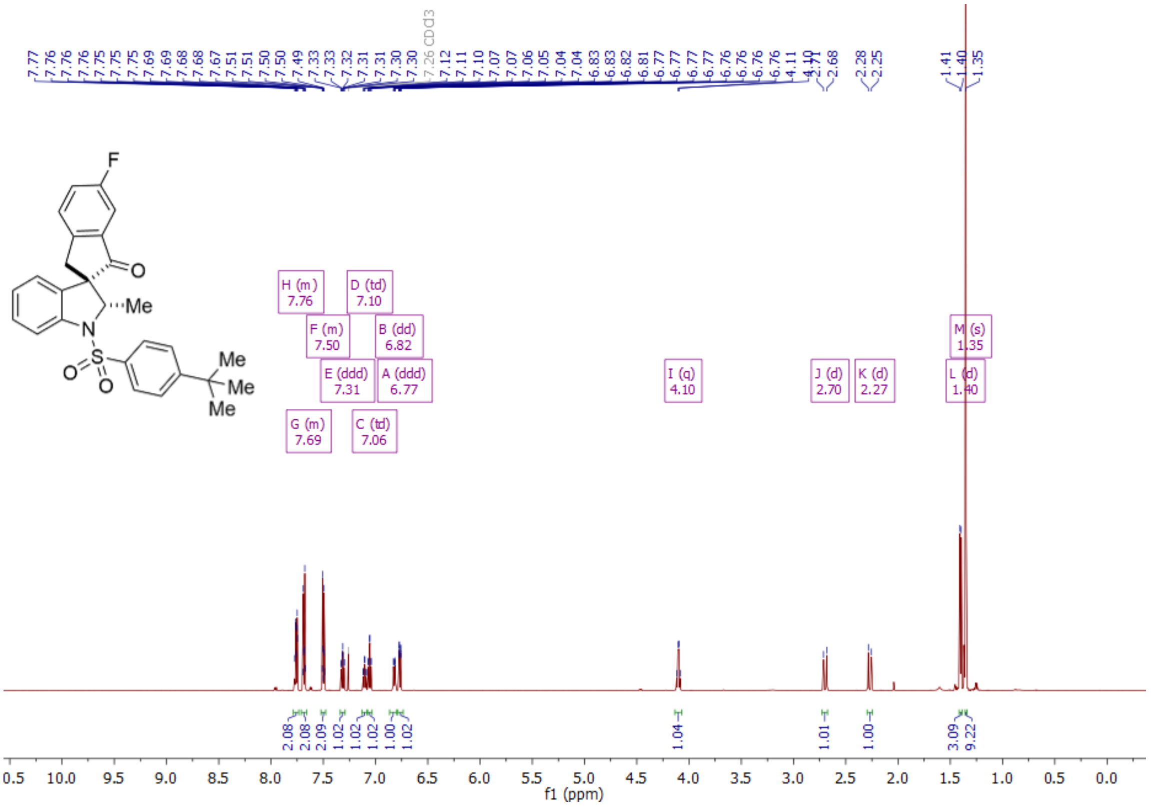

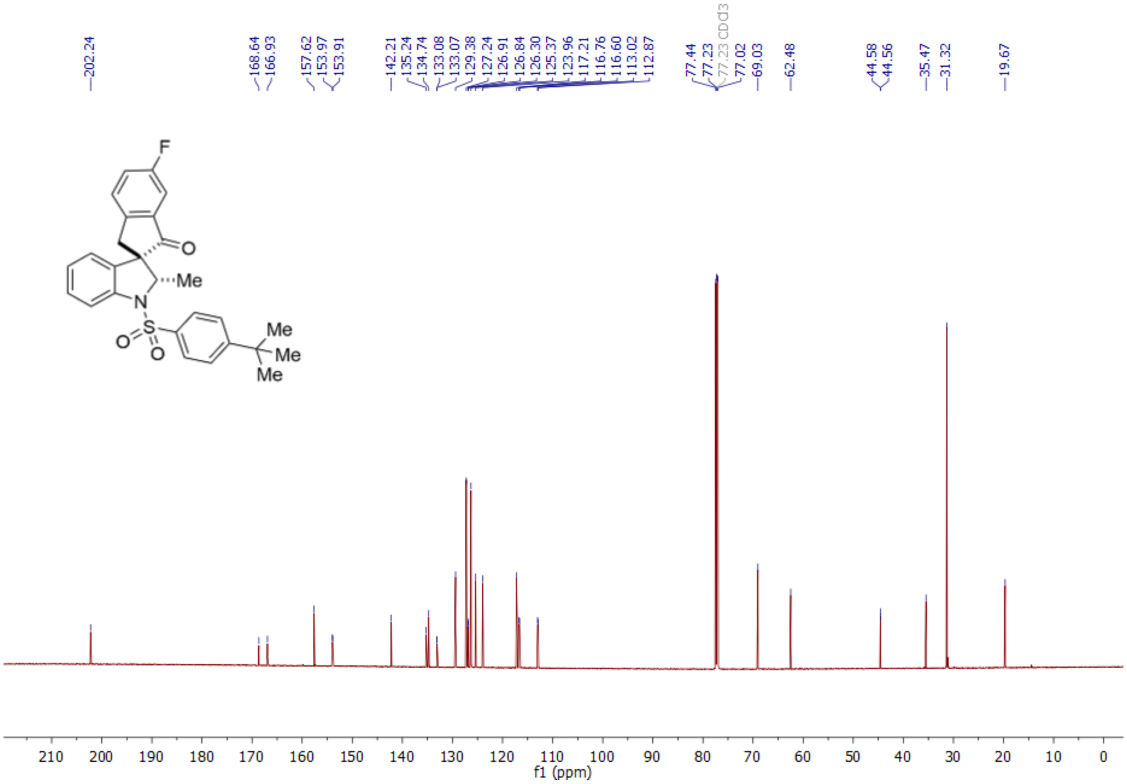

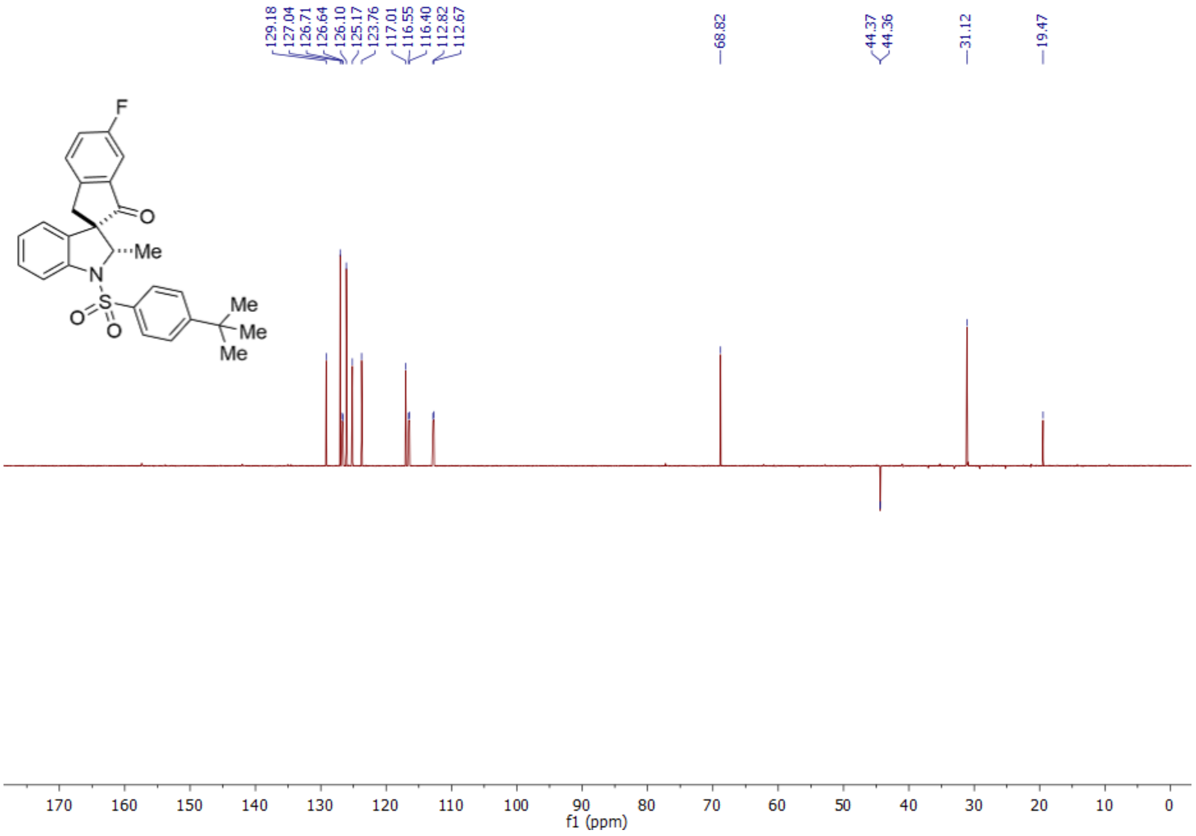
129.18
127.04
126.71
126.64
126.10
125.17
123.76
117.01
116.55
116.40
112.82
112.67
68.82
44.37
44.36
31.12
19.47
F
O
Me
N
S
O
O
Me
Me
Me
170 160 150 140 130 120 110 100 90 80 70 60 50 40 30 20 10 0
f1 (ppm)

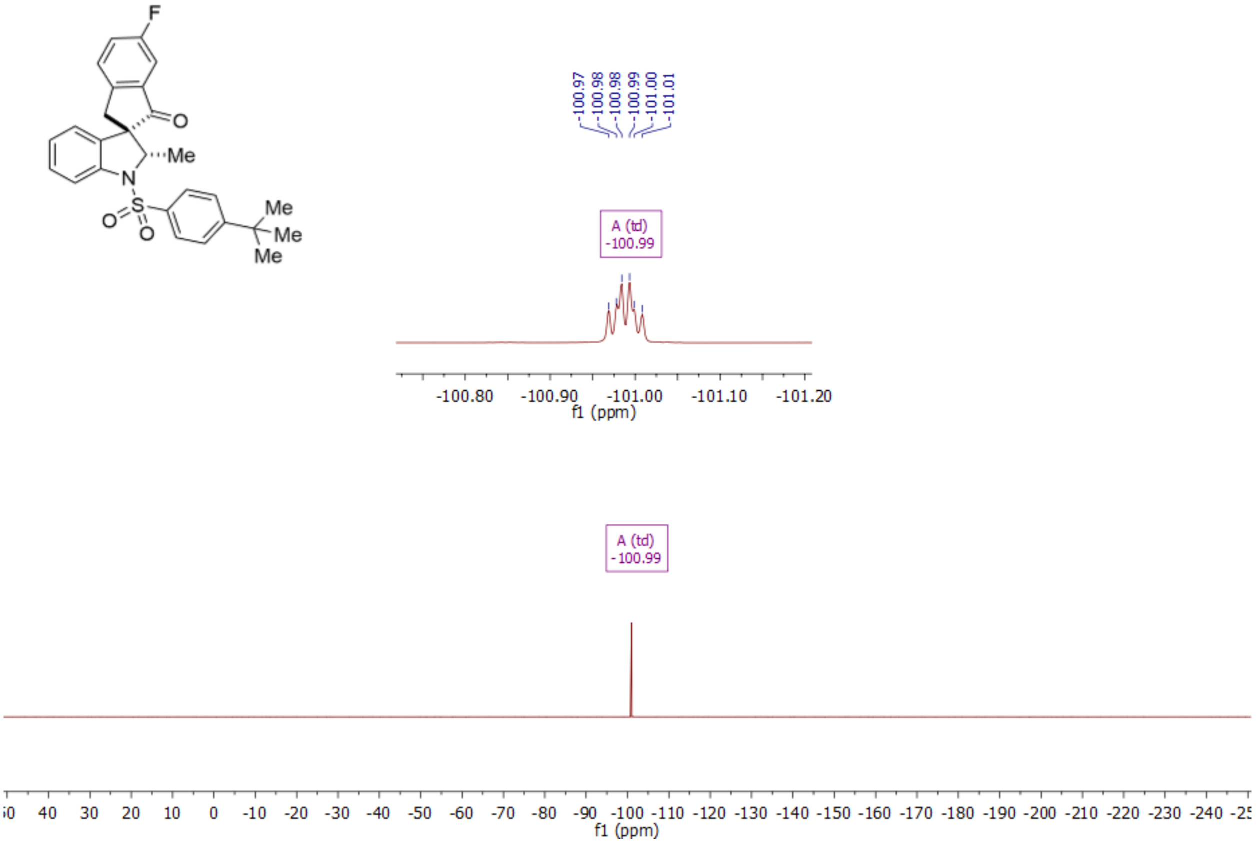
F
O
Me
N
S
O
O
Me
Me
Me
-100.97
-100.98
-100.98
-100.99
-101.00
-101.01
A (td)
-100.99
-100.80 -100.90 -101.00 -101.10 -101.20
f1 (ppm)
A (td)
-100.99
40 30 20 10 0 -10 -20 -30 -40 -50 -60 -70 -80 -90 -100 -110 -120 -130 -140 -150 -160 -170 -180 -190 -200 -210 -220 -230 -240
f1 (ppm)

***1'-((4-(tert-butyl)phenyl)sulfonyl)-5'-chloro-6-fluoro-2'-methylspiro[indene-2,3'-indolin]-1(3H)-one* [C11]:**

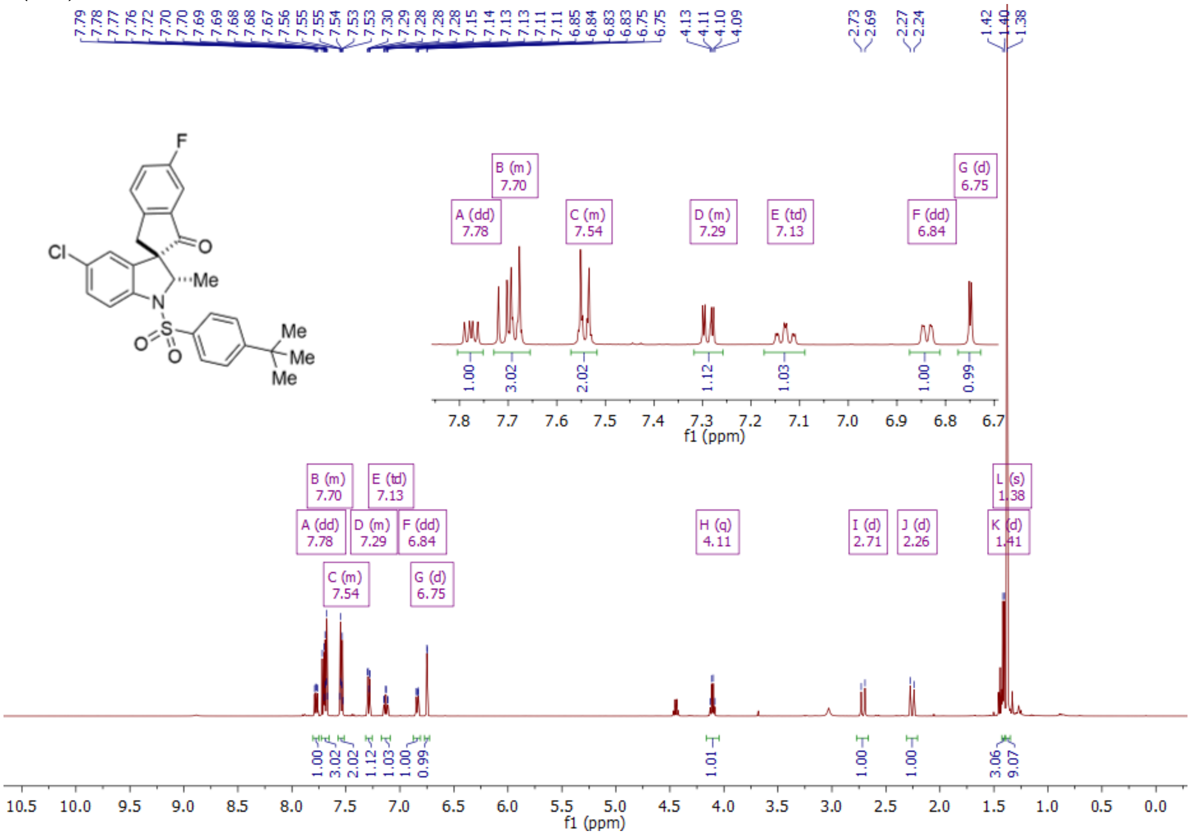

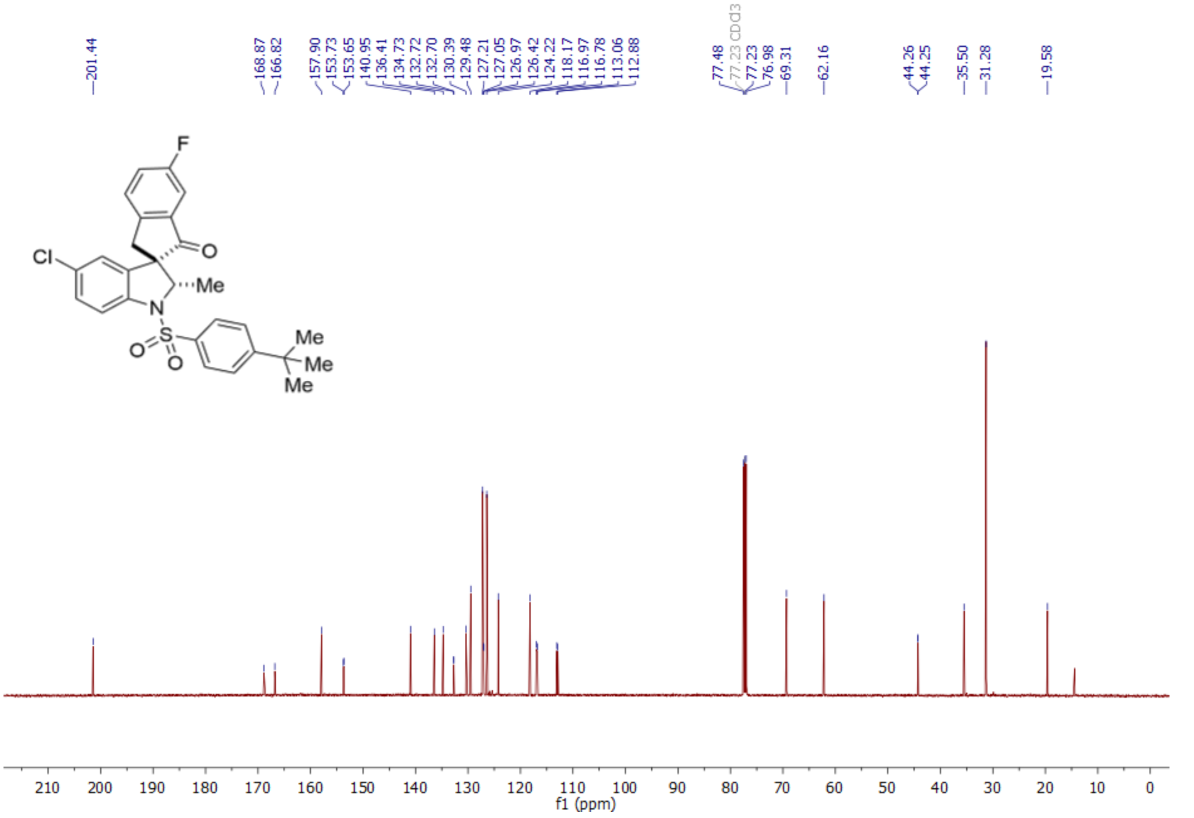

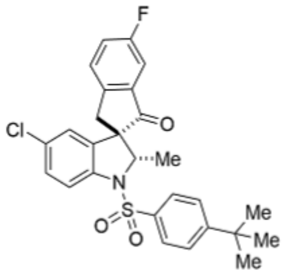
F
Cl
O
Me
N
S
O
O
Me
Me
Me

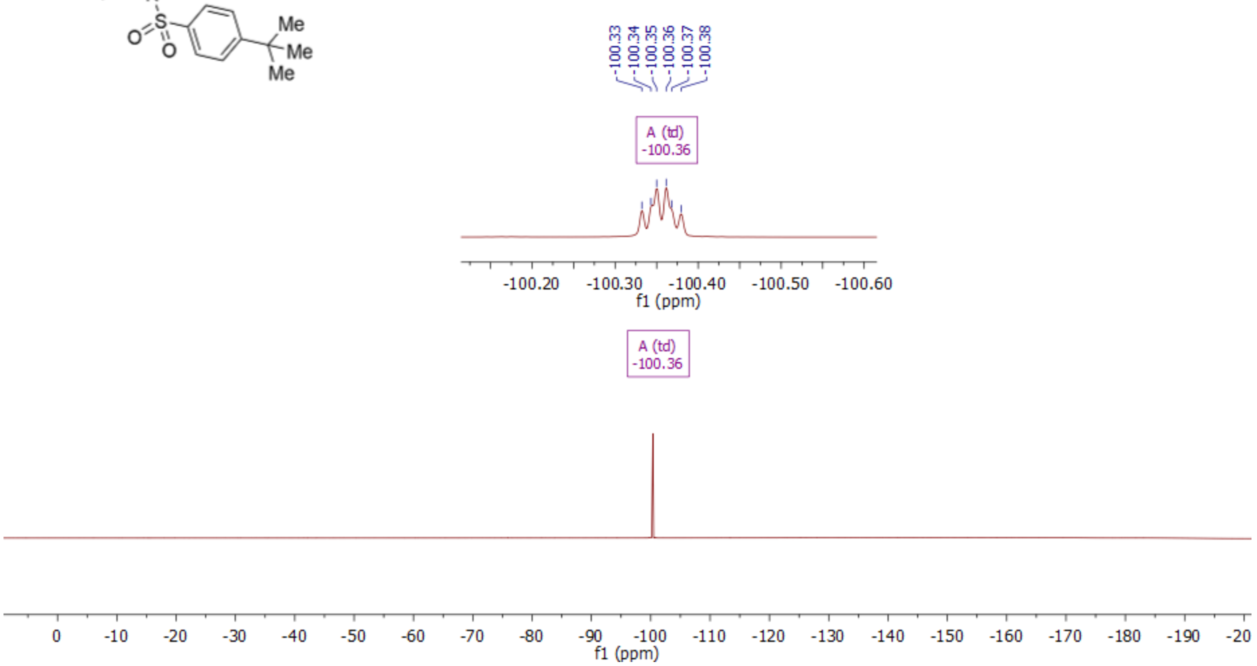
-100.33
-100.34
-100.35
-100.36
-100.37
-100.38
A (td)
-100.36
-100.20
-100.30
-100.40
-100.50
-100.60
f1 (ppm)
A (td)
-100.36
0
-10
-20
-30
-40
-50
-60
-70
-80
-90
-100
-110
-120
-130
-140
-150
-160
-170
-180
-190
-20
f1 (ppm)

2'-methyl-1'-((4-(trifluoromethyl)phenyl)sulfonyl)spiro[indene-2,3'-indolin]-1(3H)-one [**C12**]:

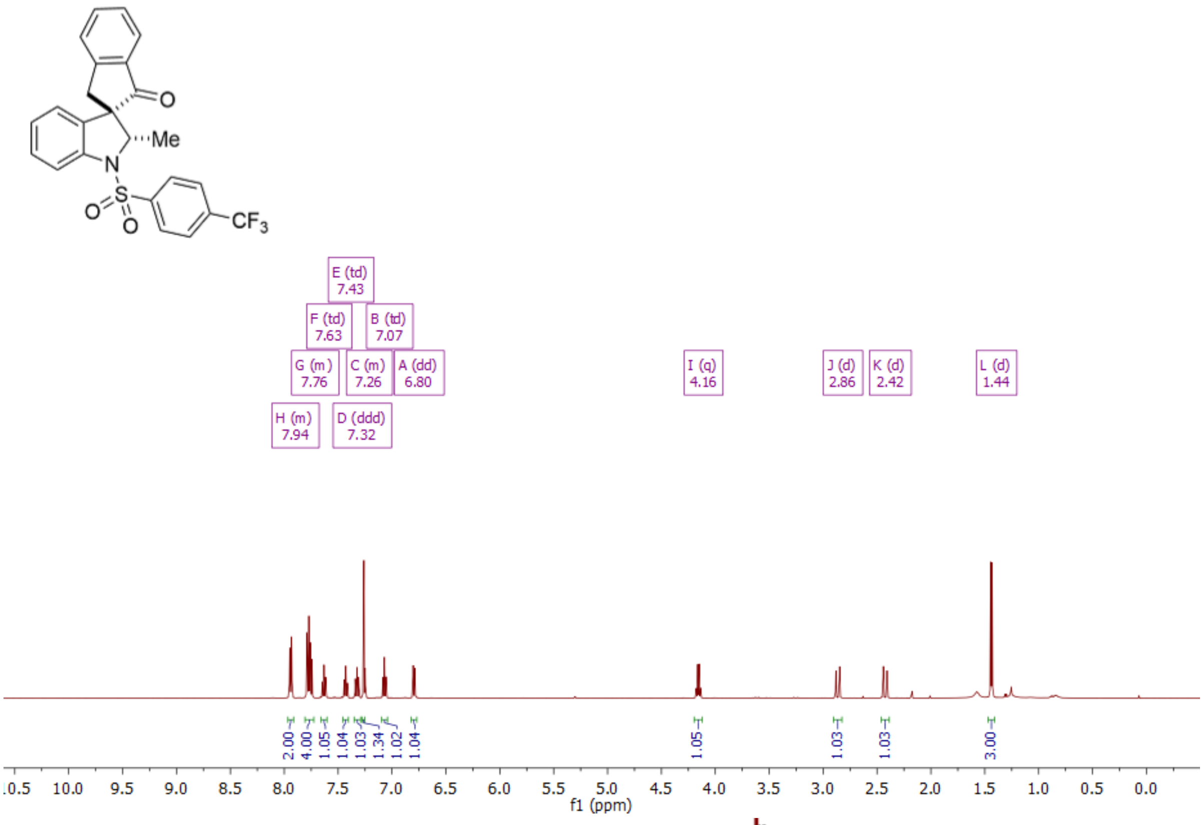

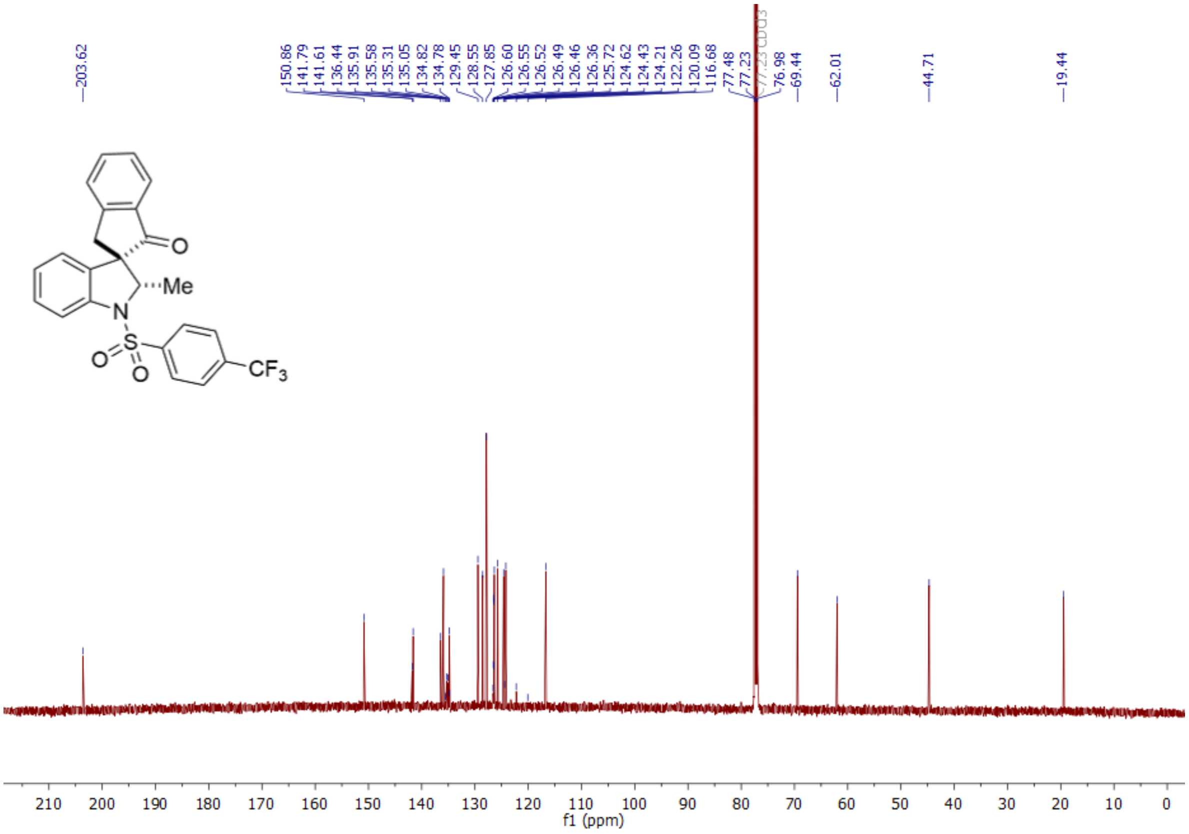

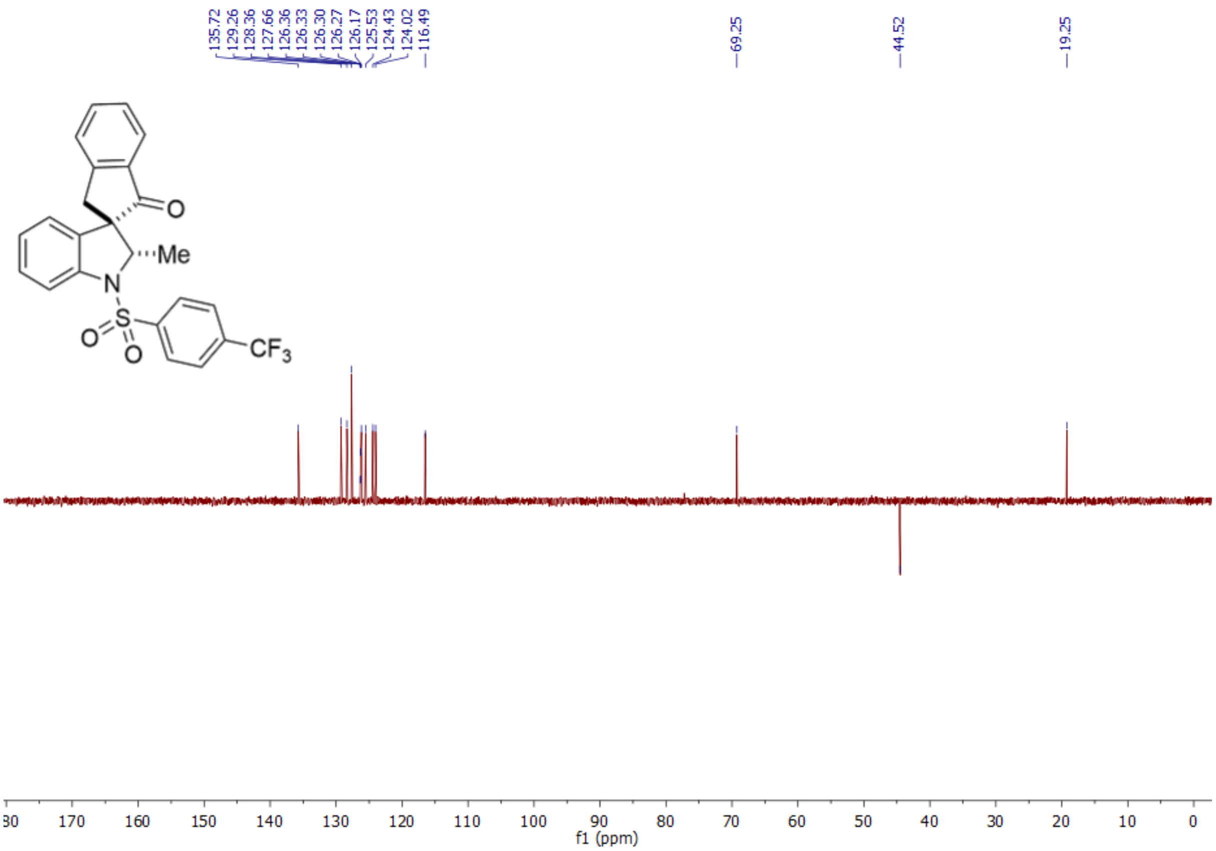
135.72
129.26
128.36
127.66
126.36
126.33
126.30
126.27
126.17
125.53
124.43
124.02
116.49
69.25
44.52
19.25
Me
N
S
O
O
O
CF3
f1 (ppm)

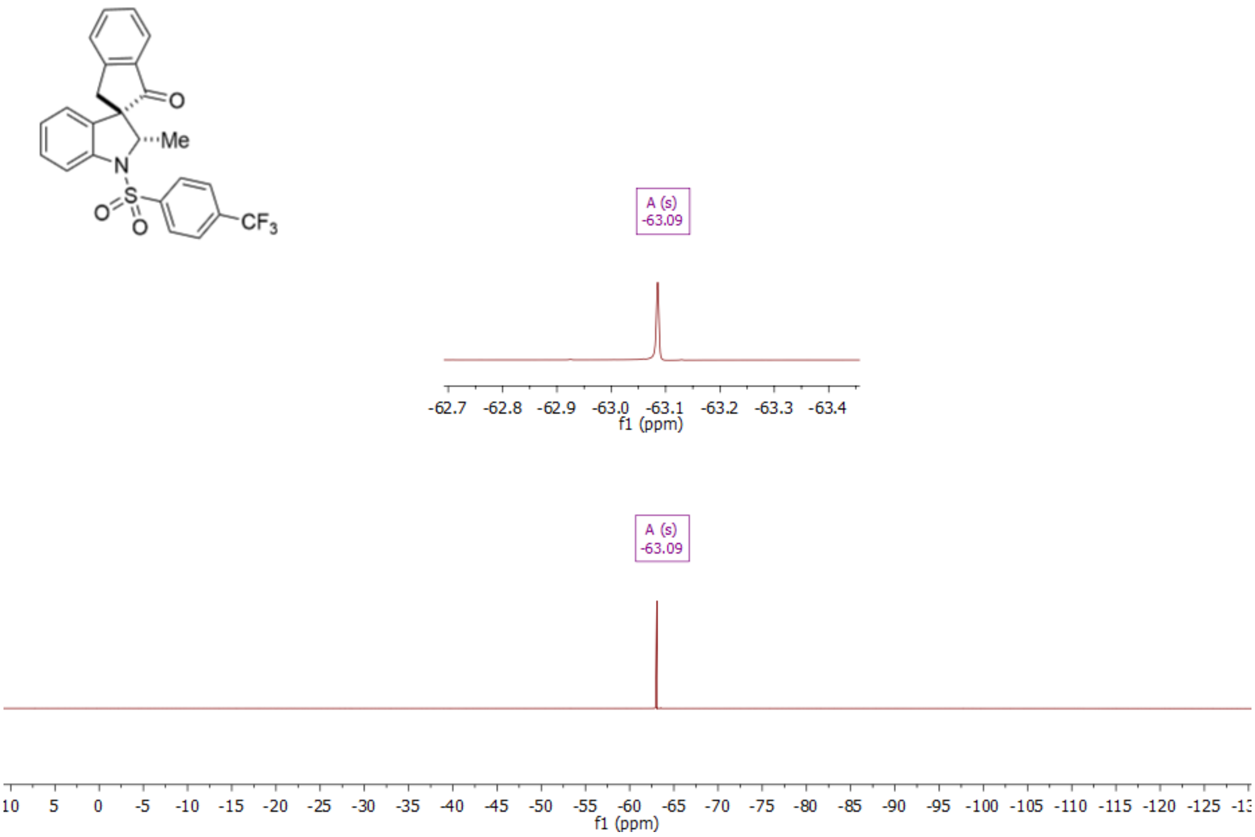
Me
N
S
O
O
O
CF3
A (s)
-63.09
f1 (ppm)
A (s)
-63.09
f1 (ppm)

1'-((4-bromophenyl)sulfonyl)-2'-methylspiro[indene-2,3'-indolin]-1(3H)-one [**C13**]:

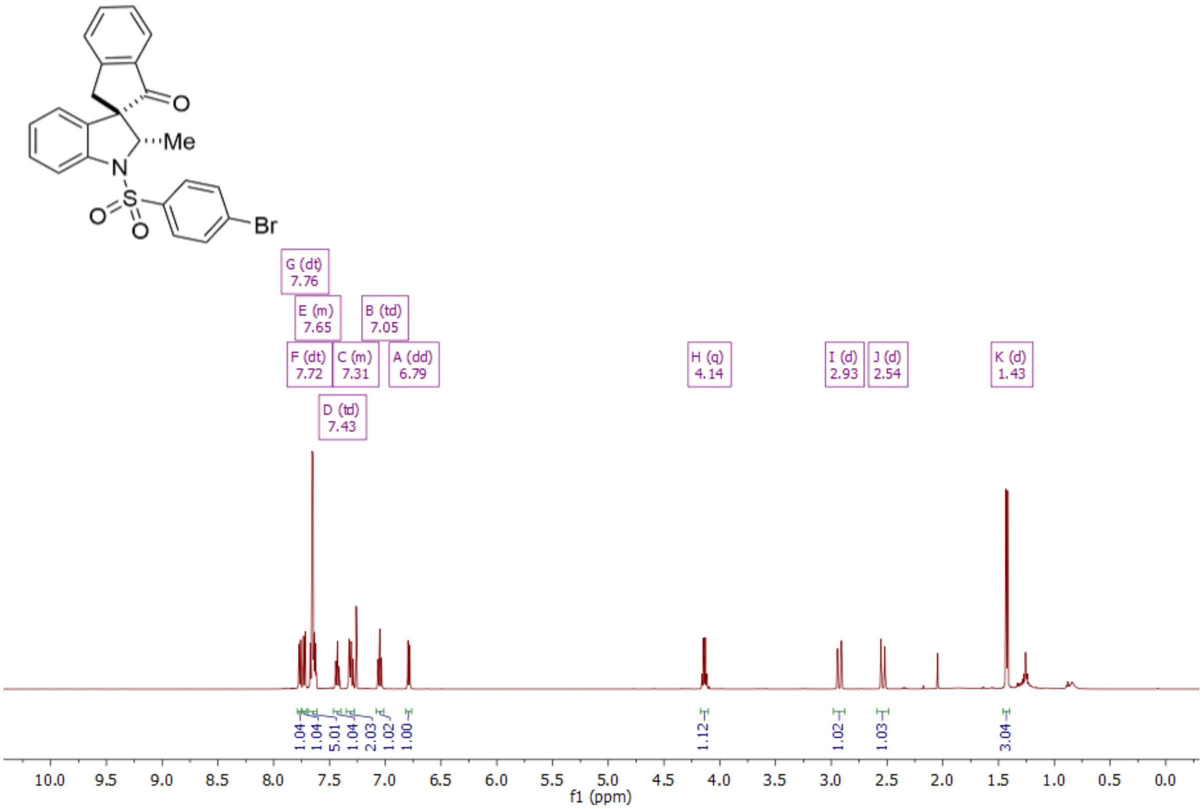

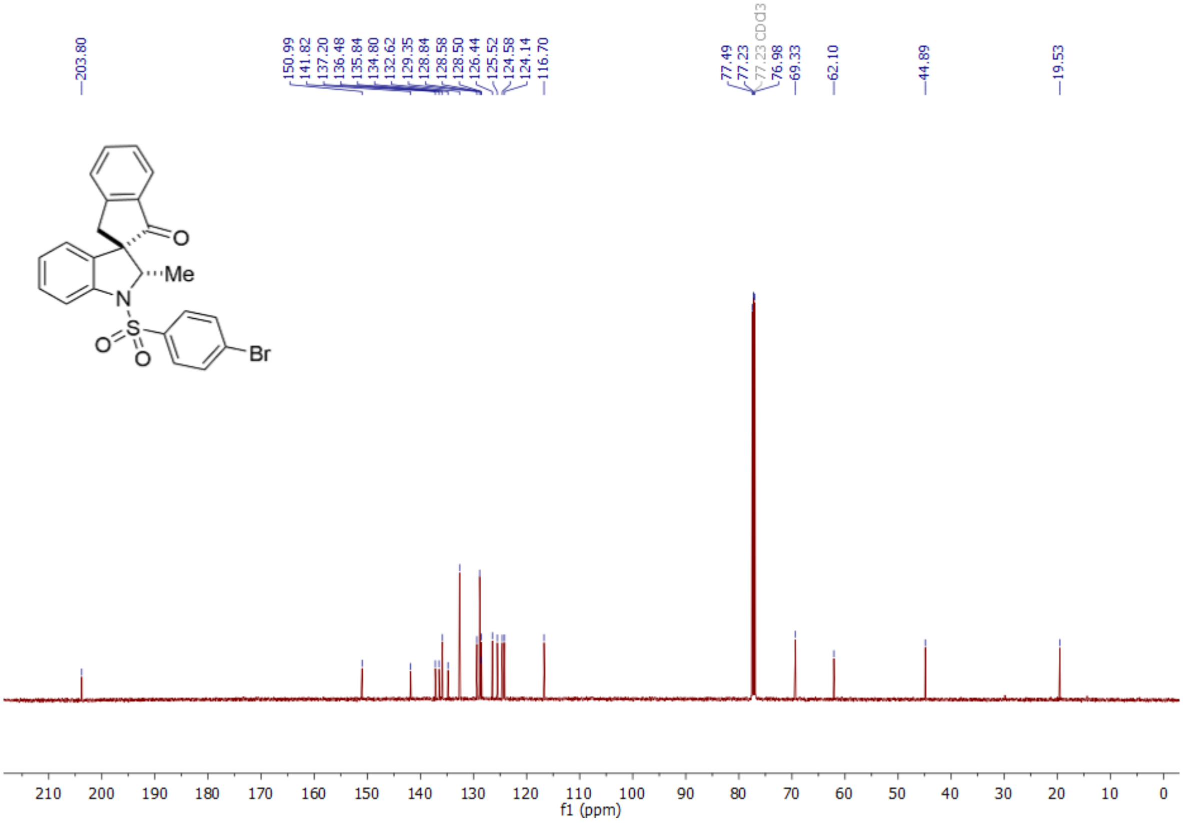

1'-((3,5-difluorophenyl)sulfonyl)-6-fluoro-2'-methylspiro[indene-2,3'-indolin]-1(3H)-one [**C14**]:

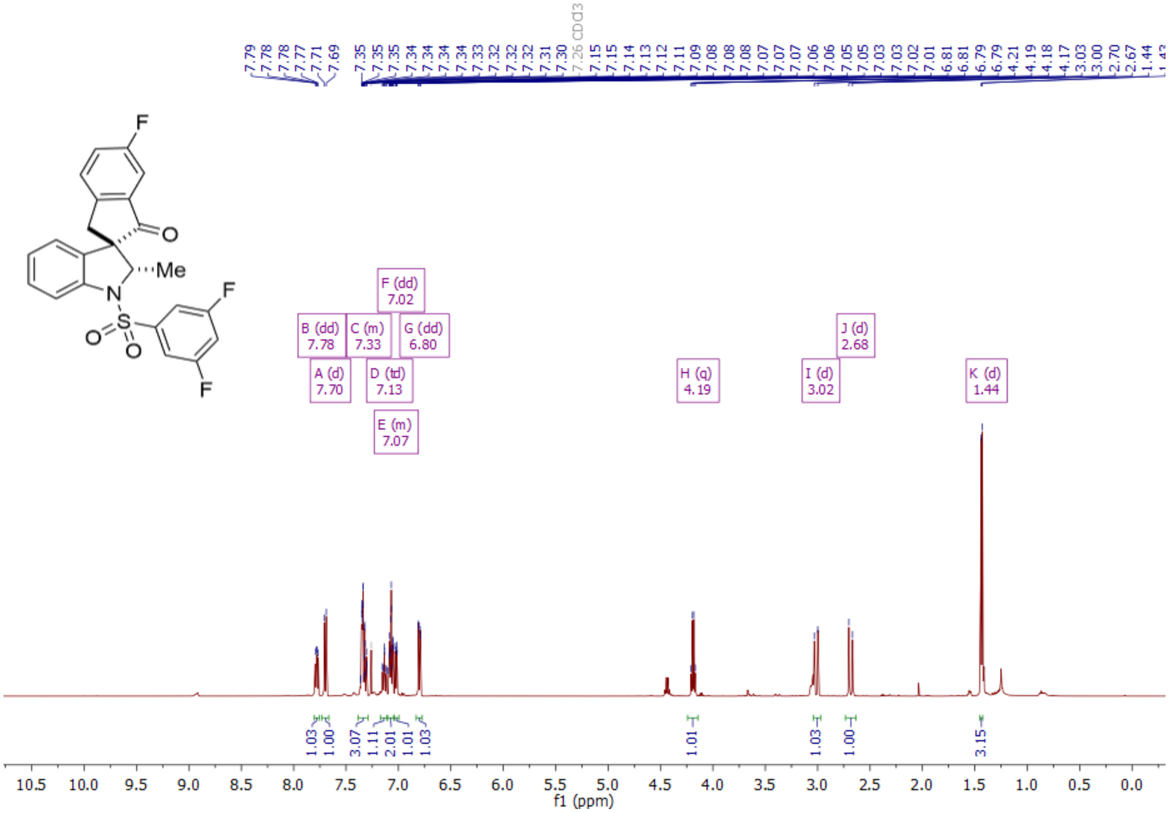

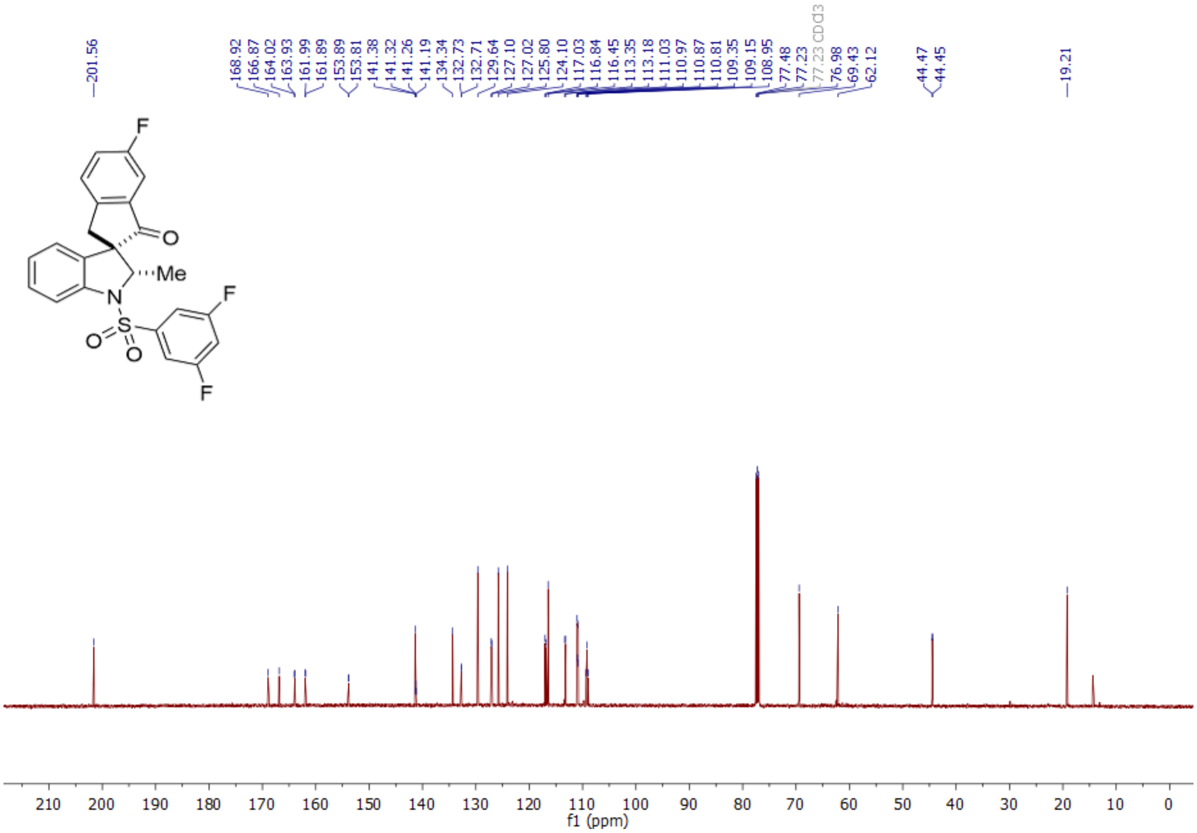

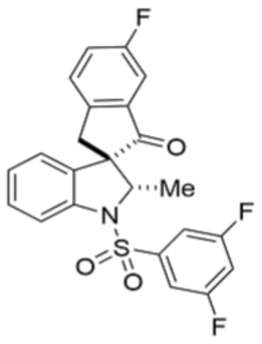

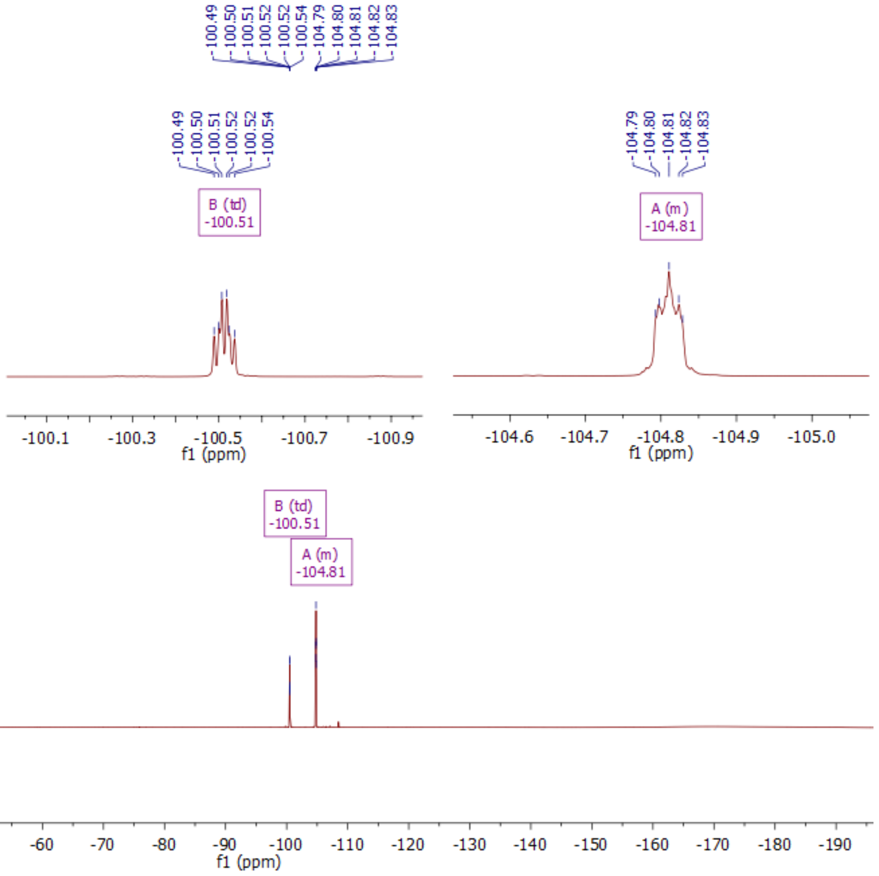

-100.49
-100.50
-100.51
-100.52
-100.52
-100.54
-104.79
-104.80
-104.81
-104.82
-104.83
B (td)
-100.51
A (m)
-104.81
-100.1
-100.3
-100.5
-100.7
-100.9
-104.6
-104.7
-104.8
-104.9
-105.0
f1 (ppm)
0
-10
-20
-30
-40
-50
-60
-70
-80
-90
-100
-110
-120
-130
-140
-150
-160
-170
-180
-190

***1'-((2-chloro-4-fluorophenyl)sulfonyl)-6-fluoro-2'-methylspiro[indene-2,3'-indolin]-1(3H)-one* [C15]:**

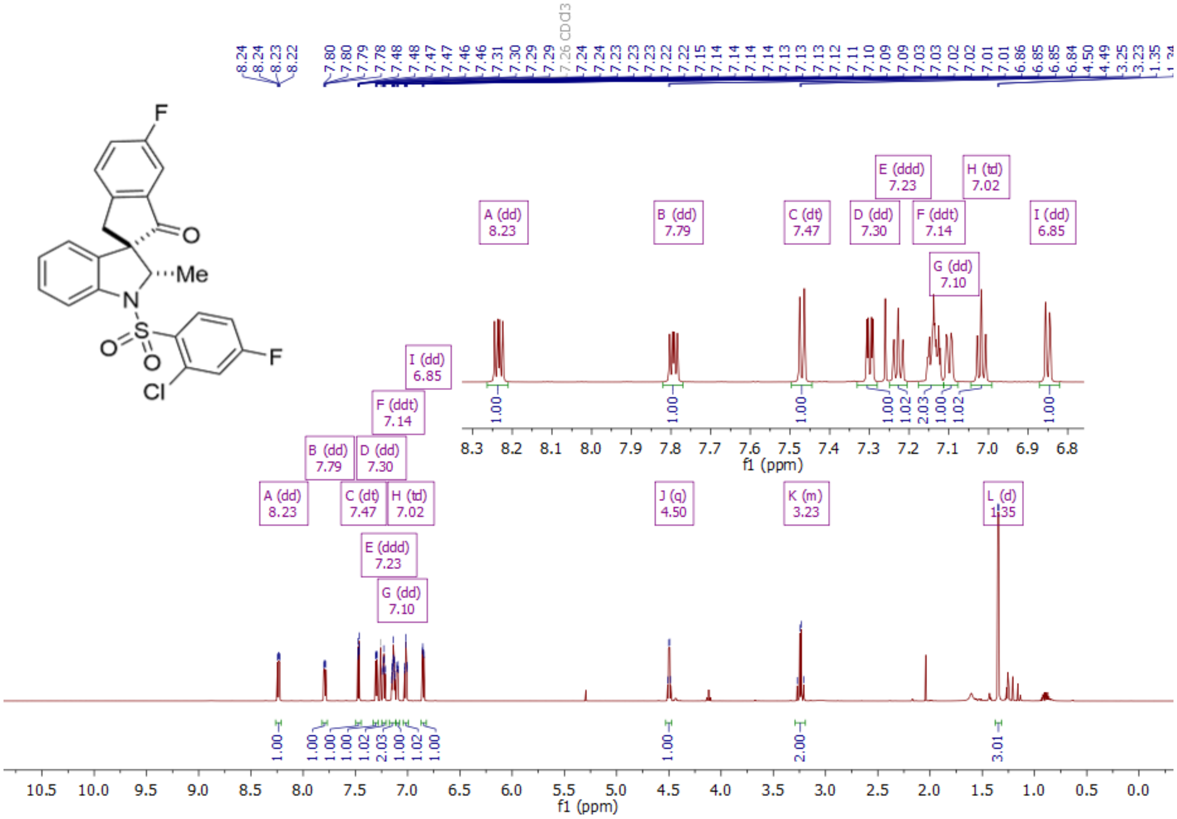

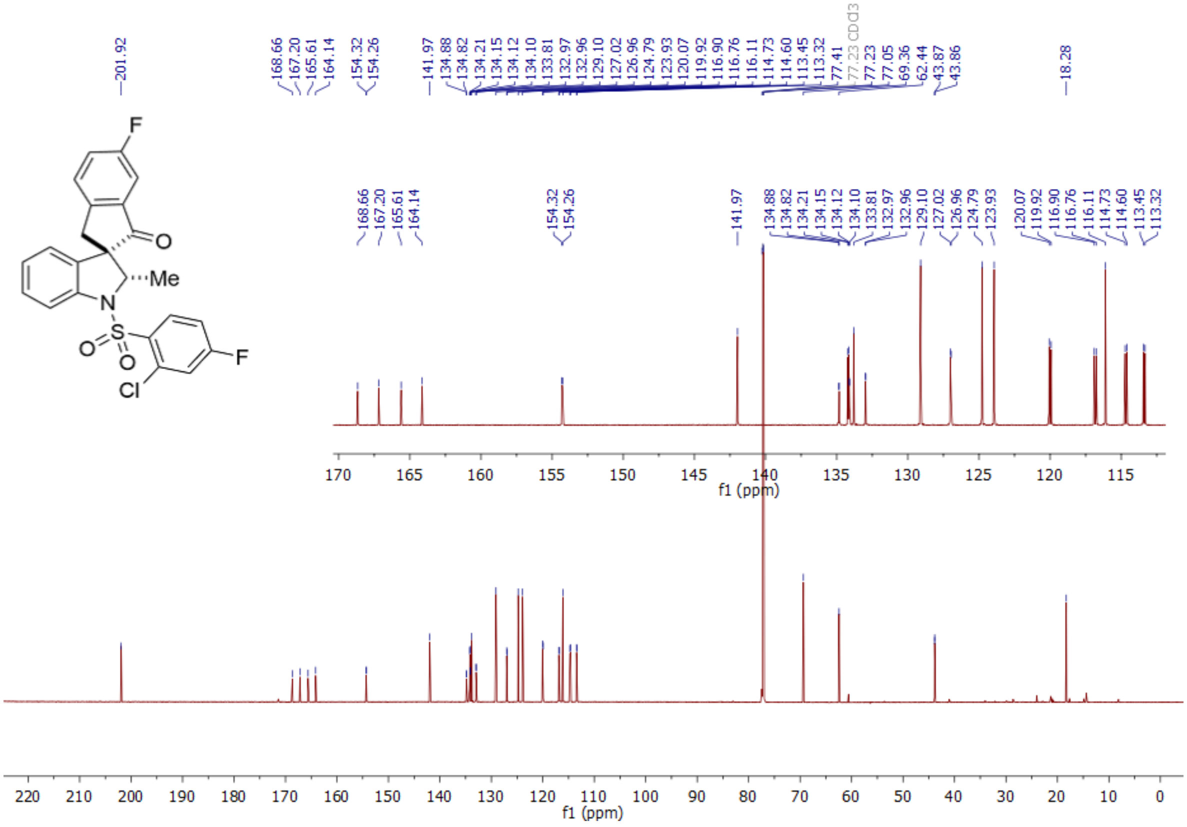

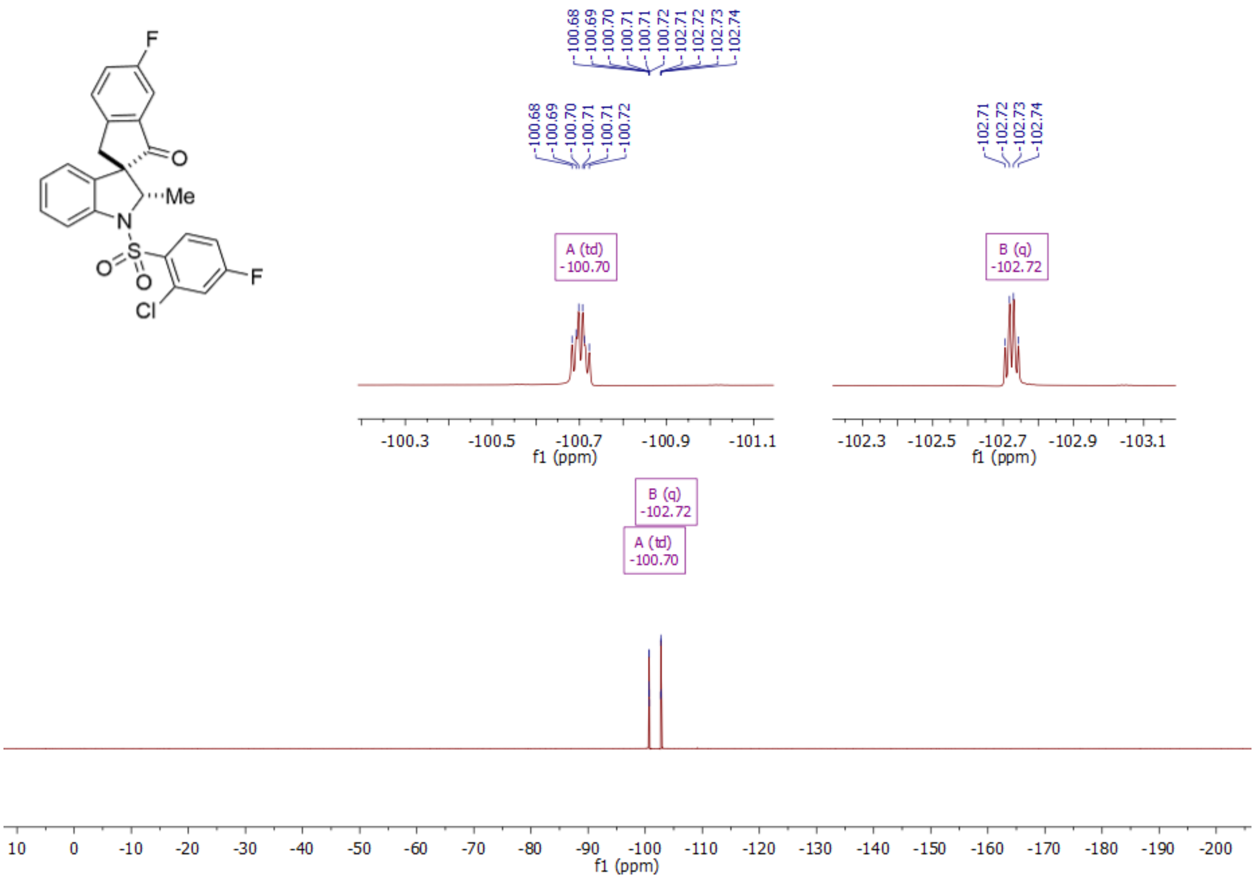
F
O
Me
N
S
O
O
F
Cl
-100.68
-100.69
-100.70
-100.71
-100.71
-100.72
-102.71
-102.72
-102.73
-102.74
A (td)
-100.70
B (q)
-102.72
-100.3 -100.5 -100.7 -100.9 -101.1
f1 (ppm)
-102.3 -102.5 -102.7 -102.9 -103.1
f1 (ppm)
10 0 -10 -20 -30 -40 -50 -60 -70 -80 -90 -100 -110 -120 -130 -140 -150 -160 -170 -180 -190 -200
f1 (ppm)

1'-((3-fluoro-4-methylphenyl)sulfonyl)-2'-methylspiro[indene-2,3'-indolin]-1(3H)-one [**C16**]:

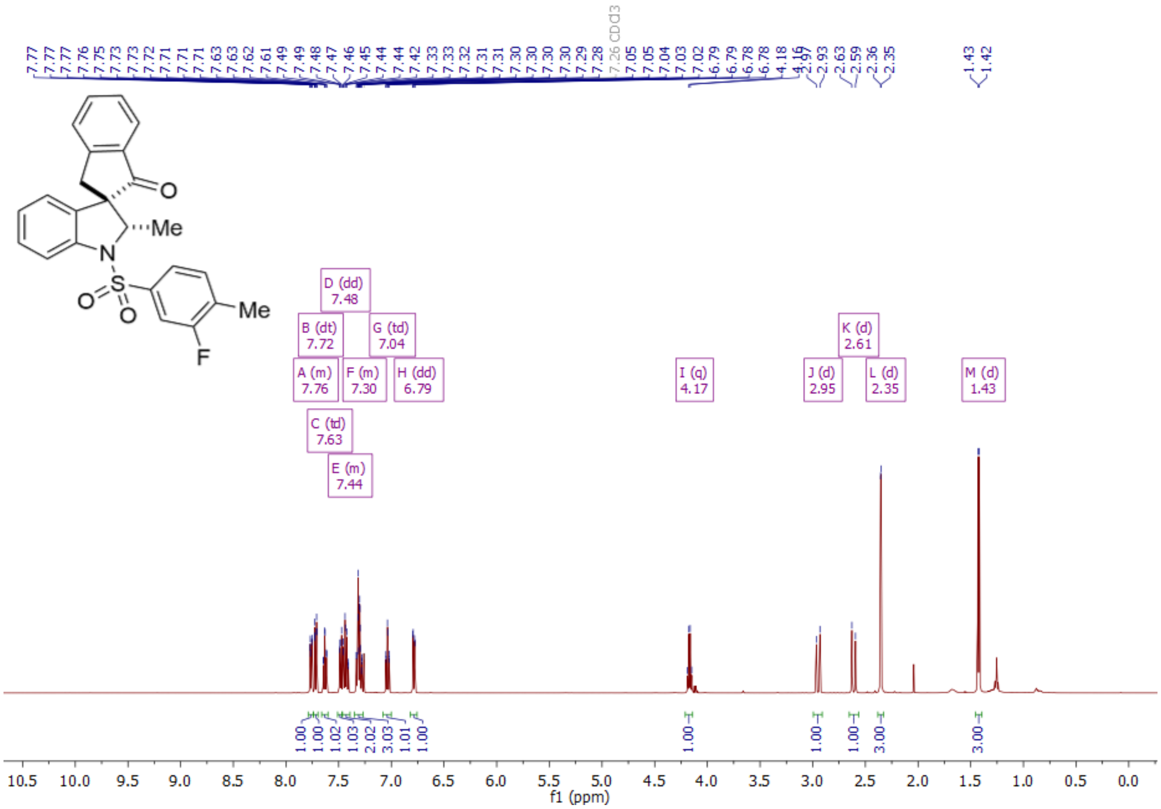

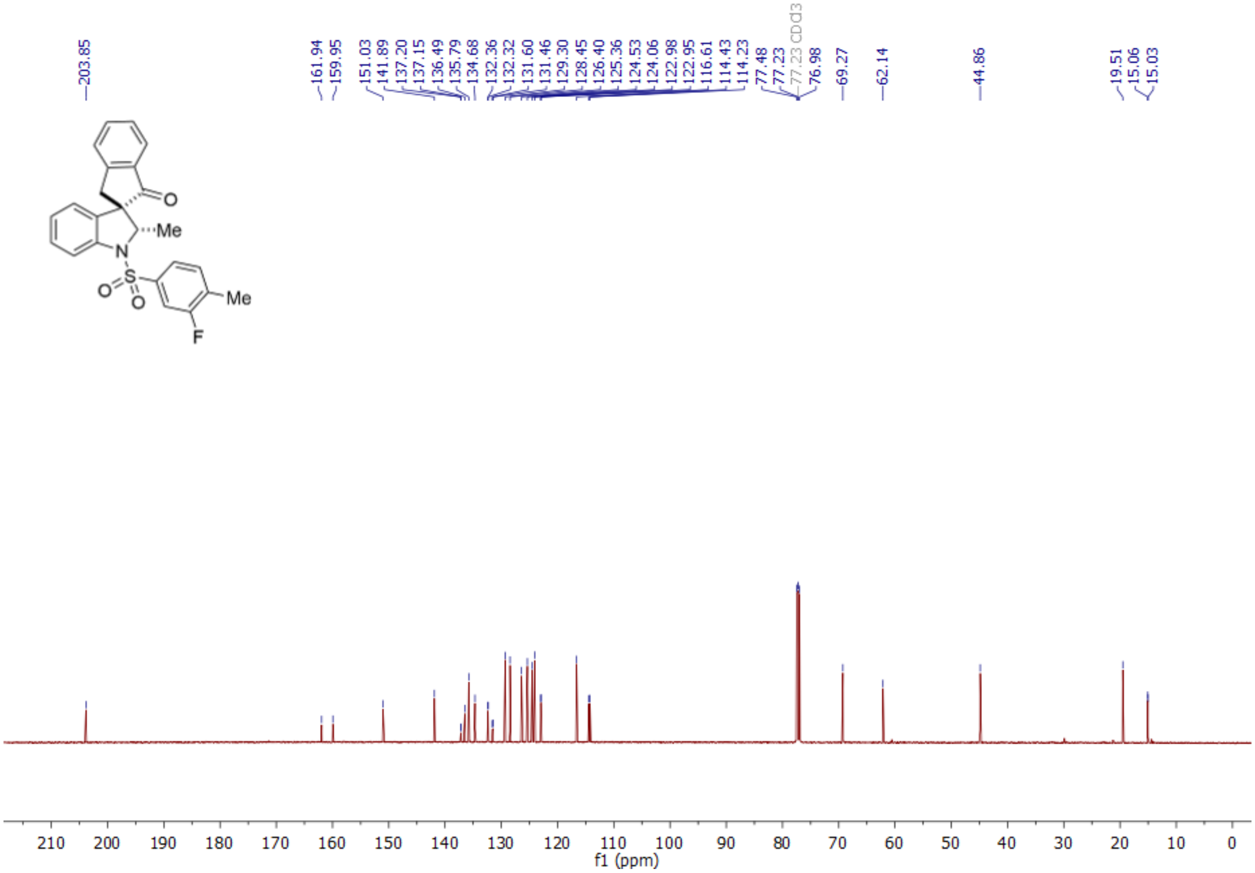

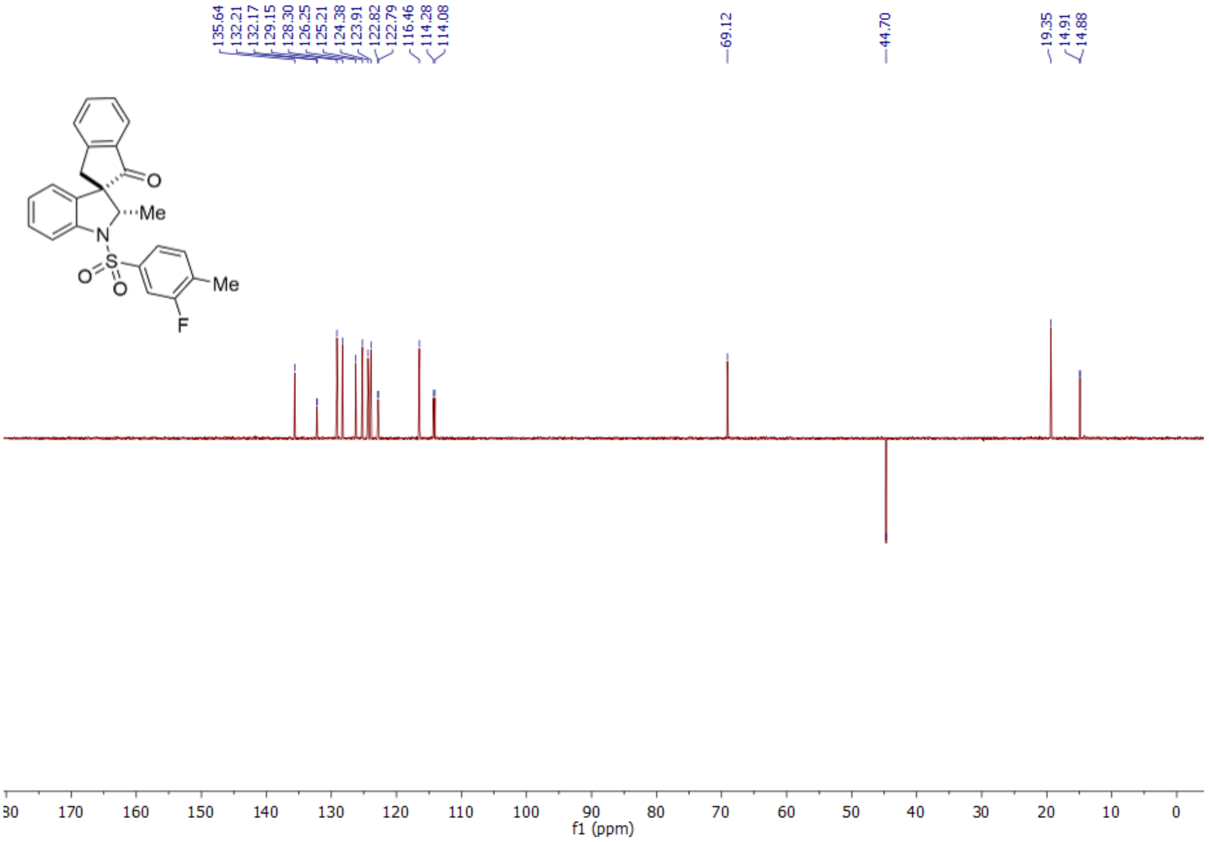
135.64
132.21
132.17
129.15
128.30
126.25
125.21
124.38
123.91
122.82
122.79
116.46
114.28
114.08
69.12
44.70
19.35
14.91
14.88
O
Me
N
S
O
O
Me
F
f1 (ppm)

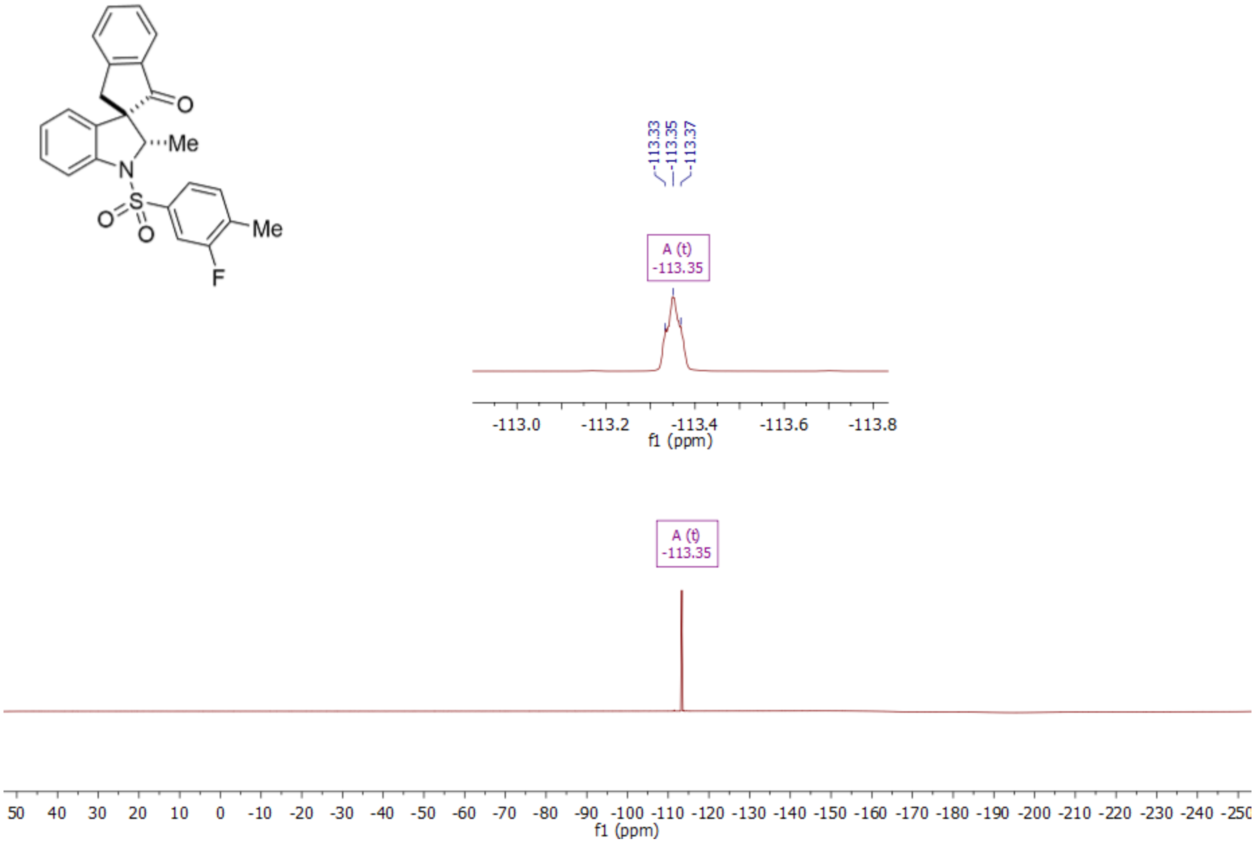
O
Me
N
S
O
O
Me
F
-113.33
-113.35
-113.37
A (t)
-113.35
f1 (ppm)
A (t)
-113.35
f1 (ppm)

2'-methyl-1'-((2,2,2-trifluoroethyl)sulfonyl)spiro[indene-2,3'-indolin]-1(3H)-one [**C17**]:

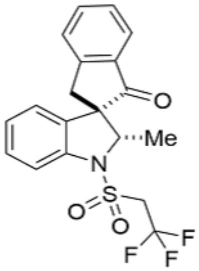

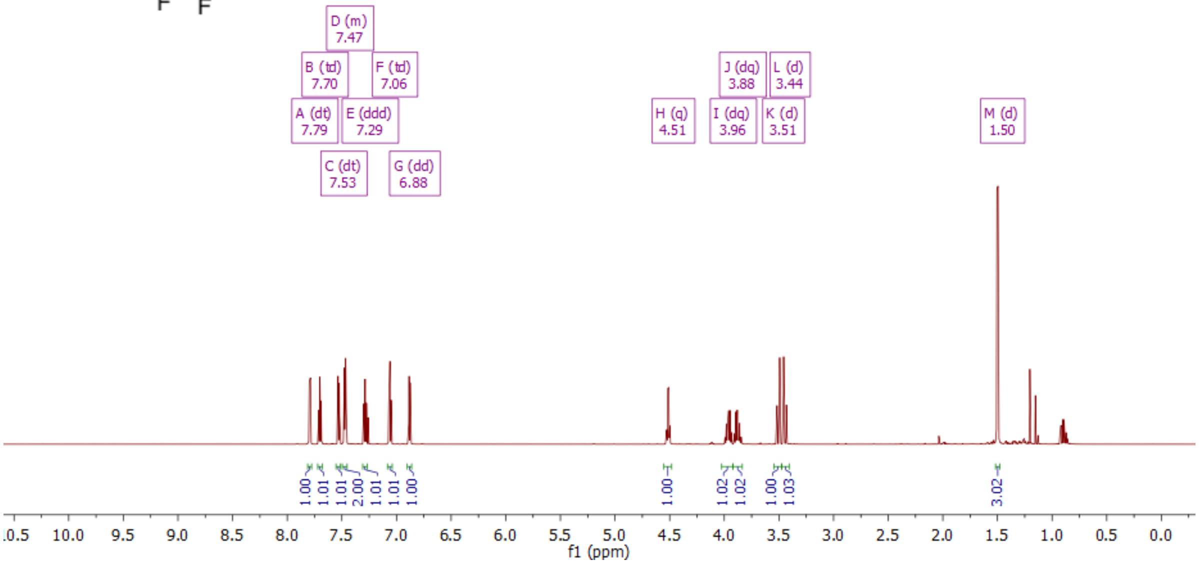

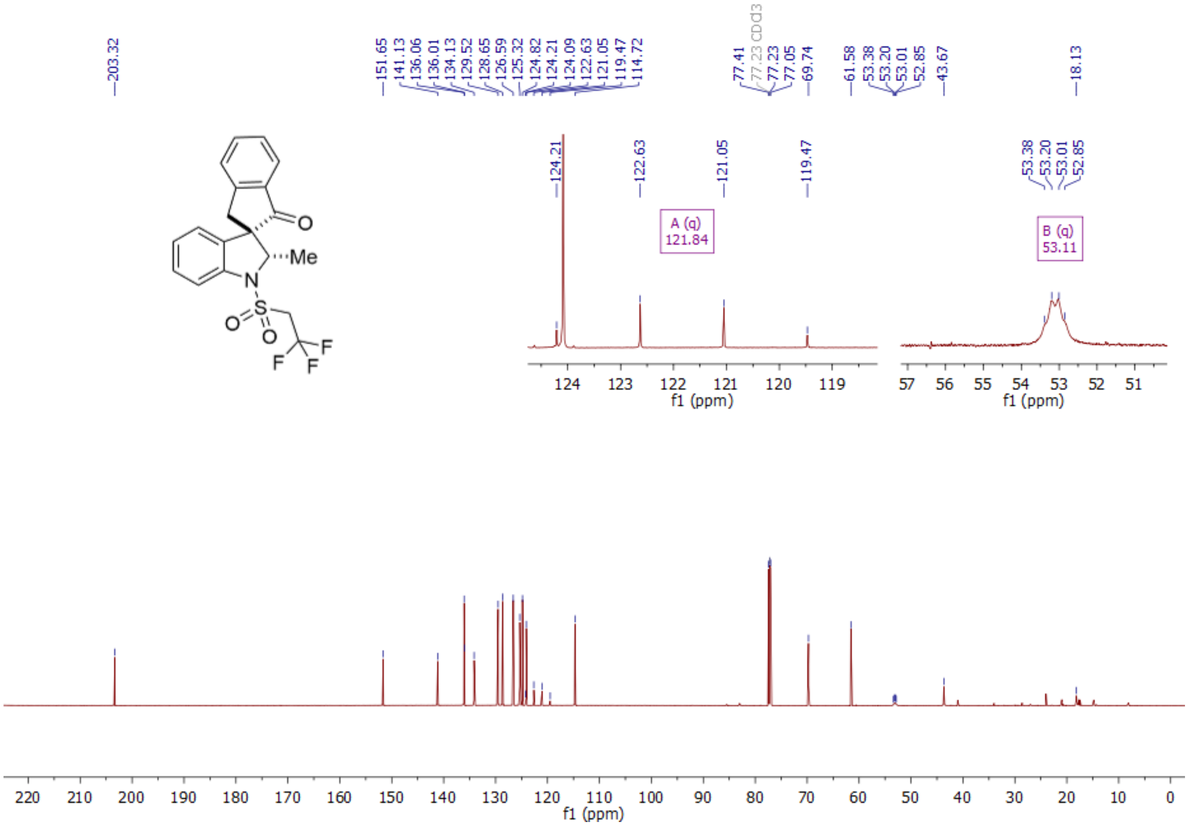

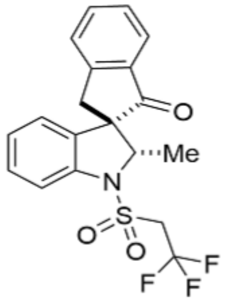

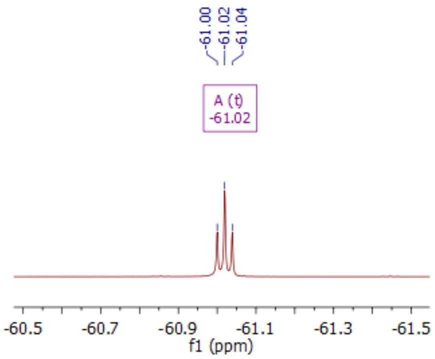
-61.00
-61.02
-61.04
A (t)
-61.02
-60.5
-60.7
-60.9
-61.1
-61.3
-61.5
f1 (ppm)

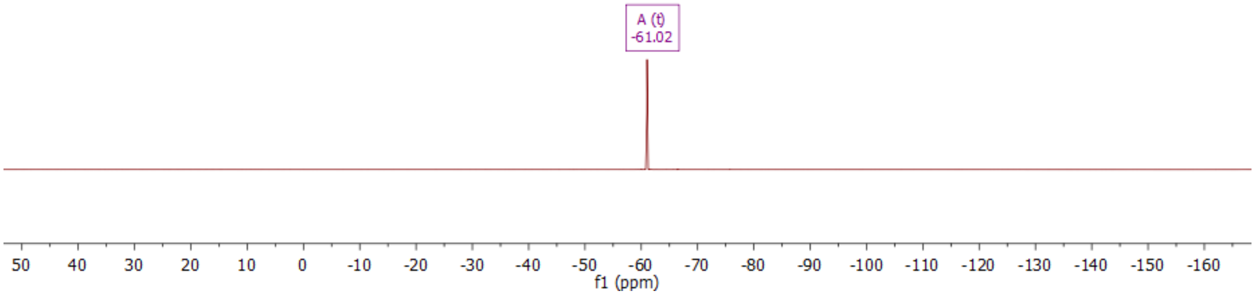
A (t)
-61.02
50
40
30
20
10
0
-10
-20
-30
-40
-50
-60
-70
-80
-90
-100
-110
-120
-130
-140
-150
-160
f1 (ppm)

2'-methyl-1'-propionylspiro[indene-2,3'-indolin]-1(3H)-one [C18]:

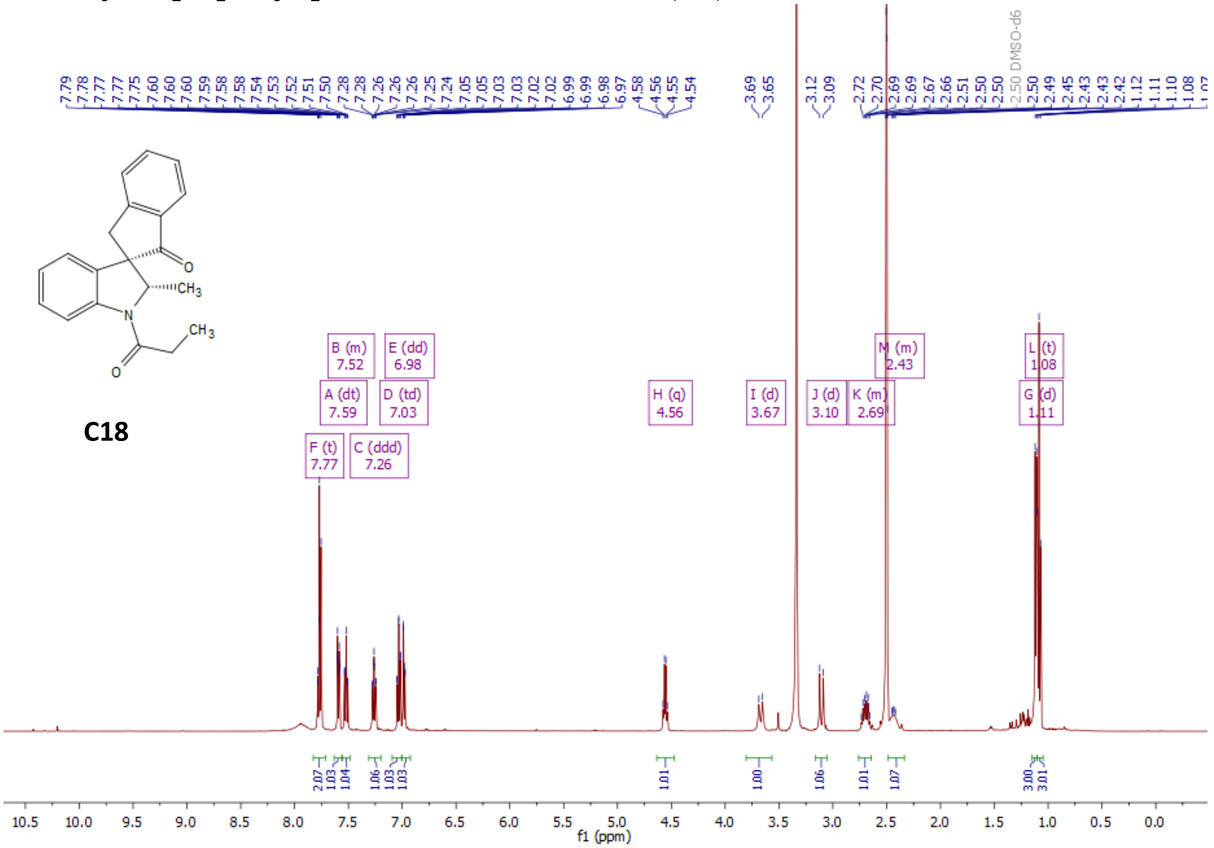

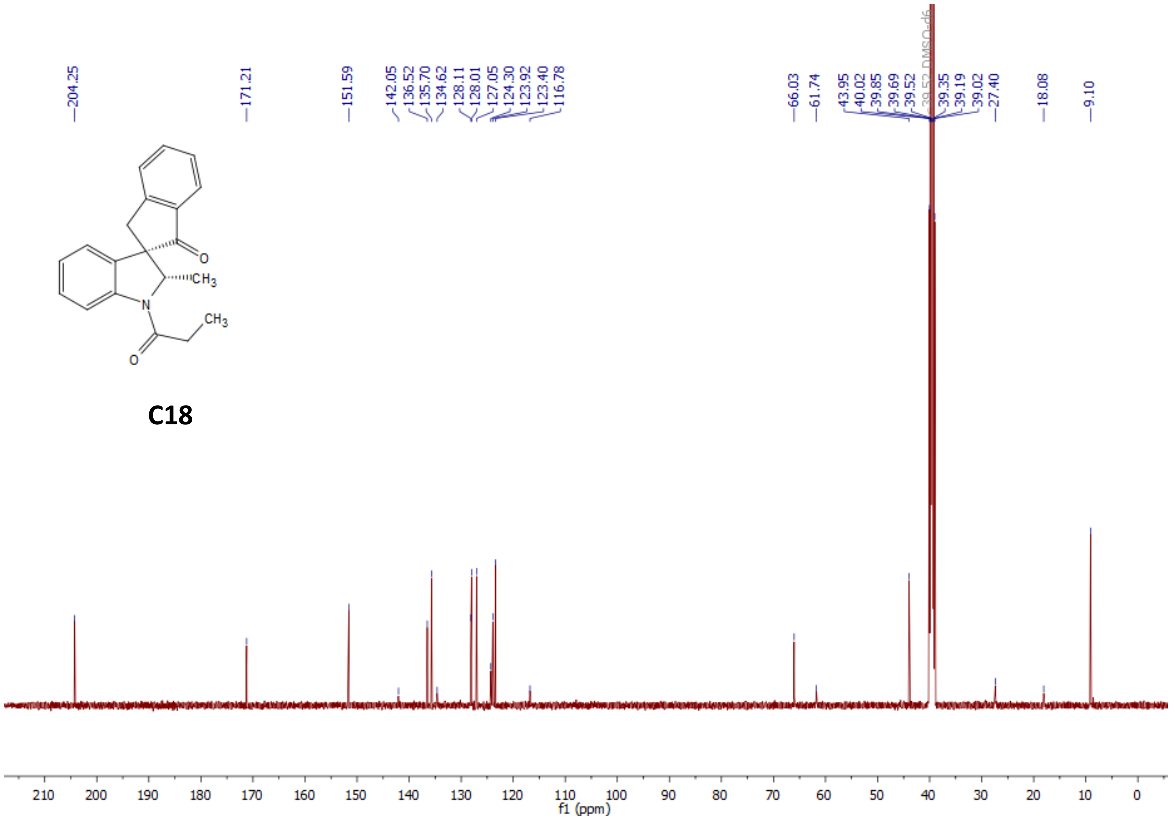

CLASS-D

***1'-(2-chloroacetyl)-2'-methylenespiro[indene-2,3'-indolin]-1(3H)-one* [D1]:**

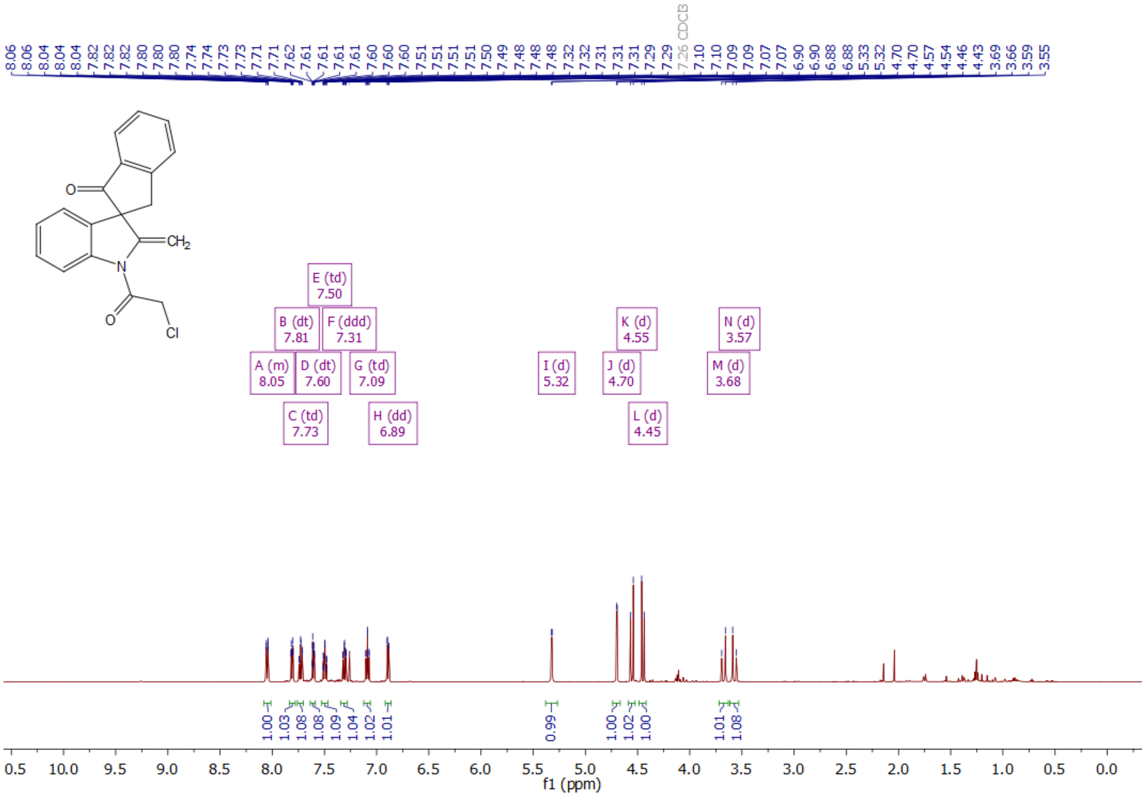

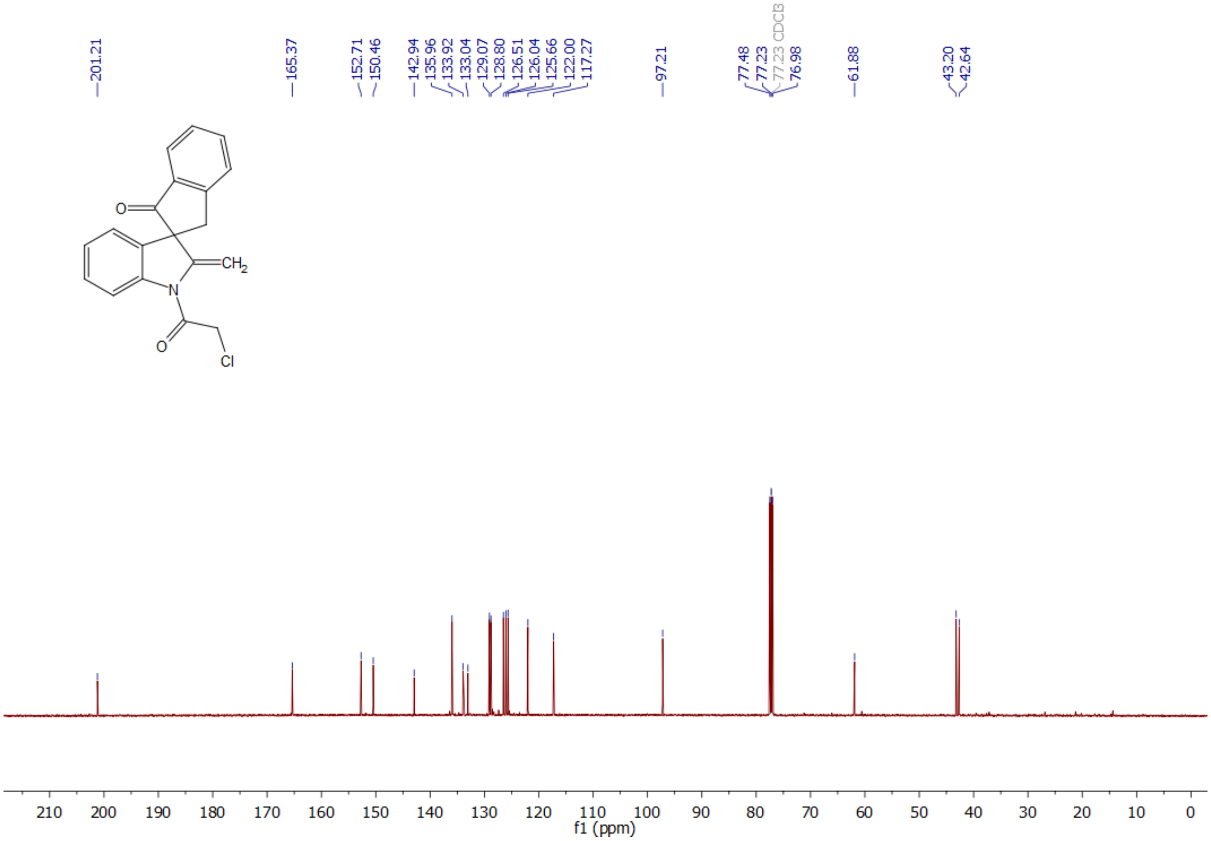

***1'-(2-bromoacetyl)-2'-methylenespiro[indene-2,3'-indolin]-1(3H)-one* [D2]:**

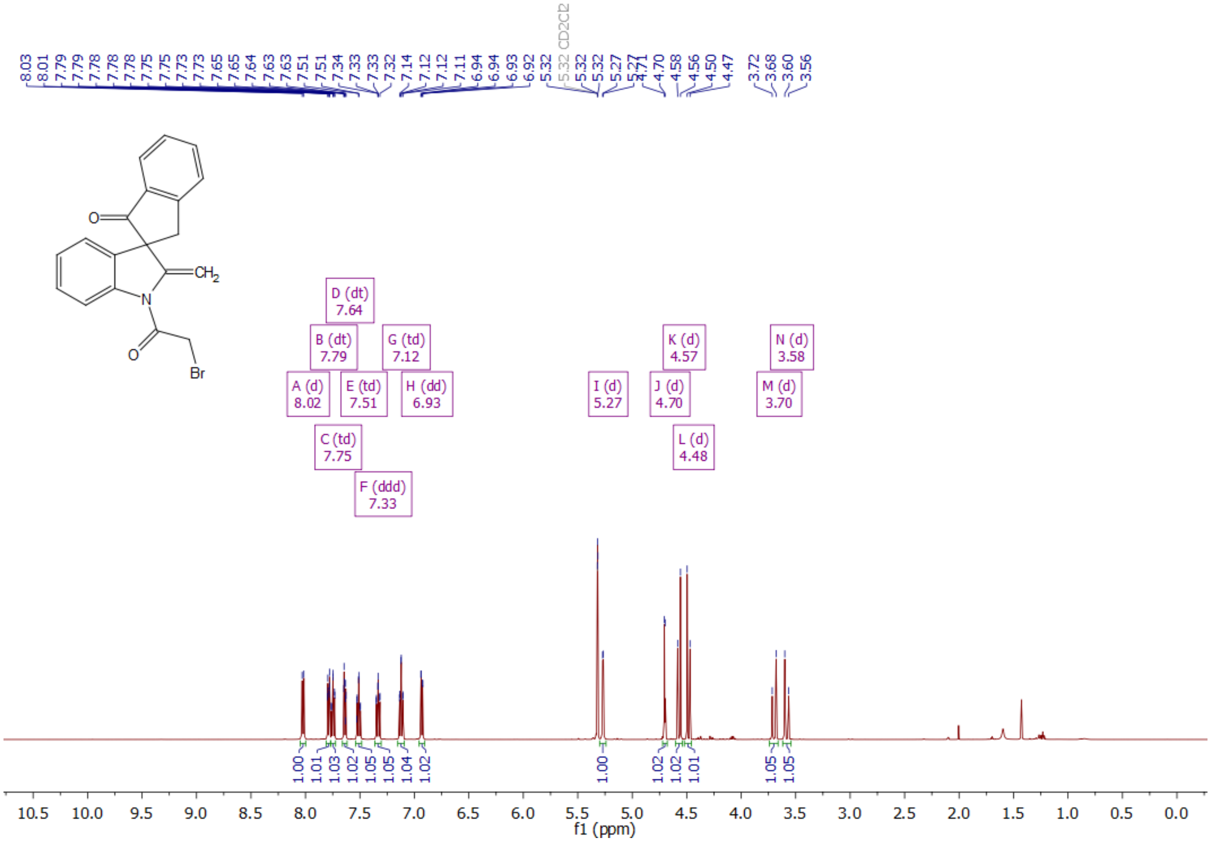

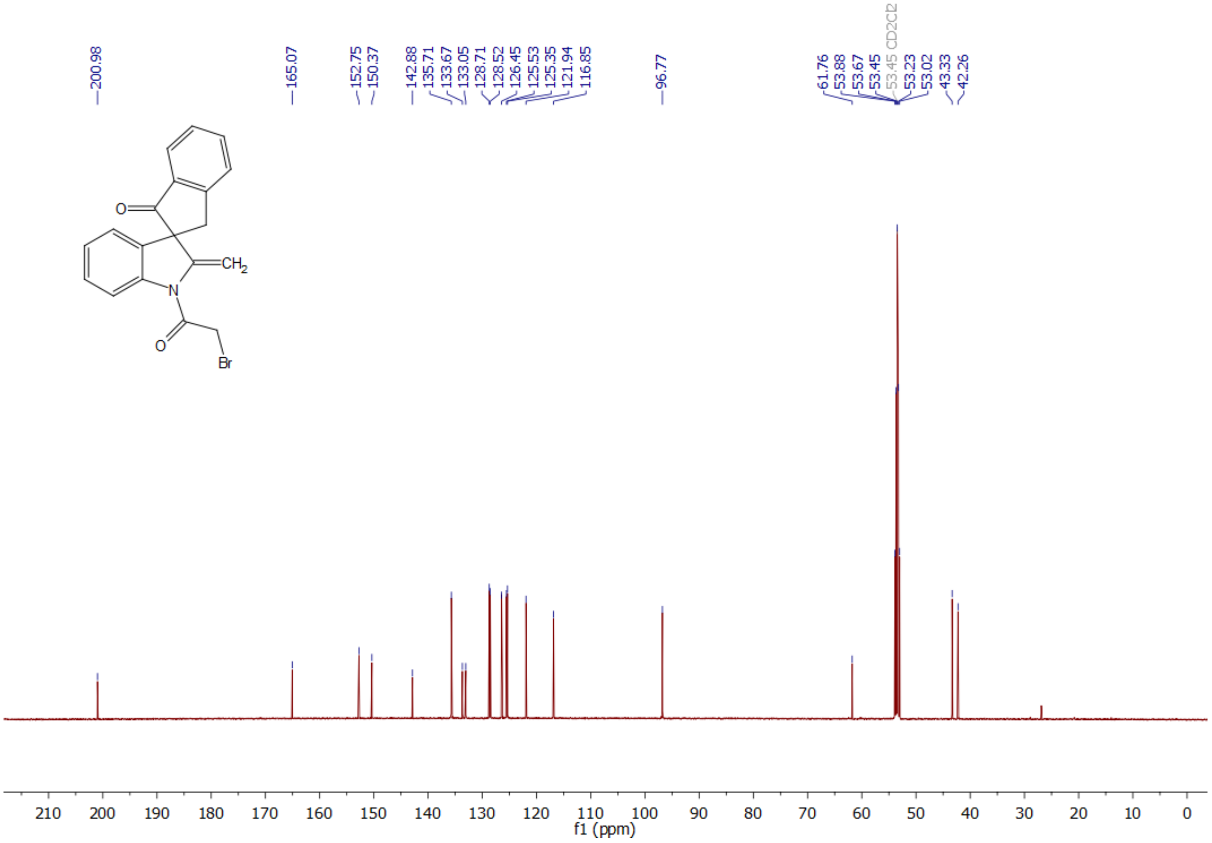

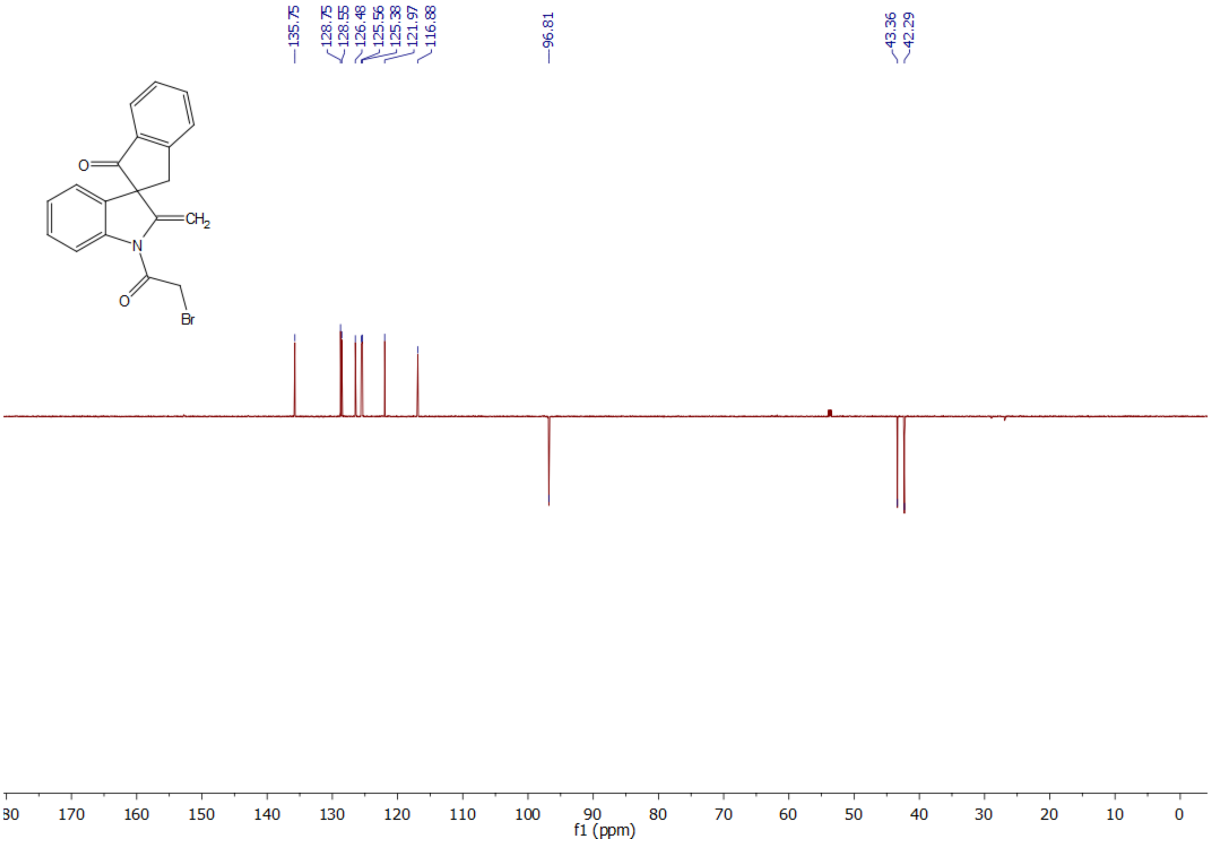
135.75
128.75
128.55
126.48
125.56
125.38
121.97
116.88
96.81
43.36
42.29
O
CH2
N
O
Br
f1 (ppm)

1'-(2-chloroacetyl)-5-fluoro-2'-methylenespiro[indene-2,3'-indolin]-1(3H)-one [**D3**]:

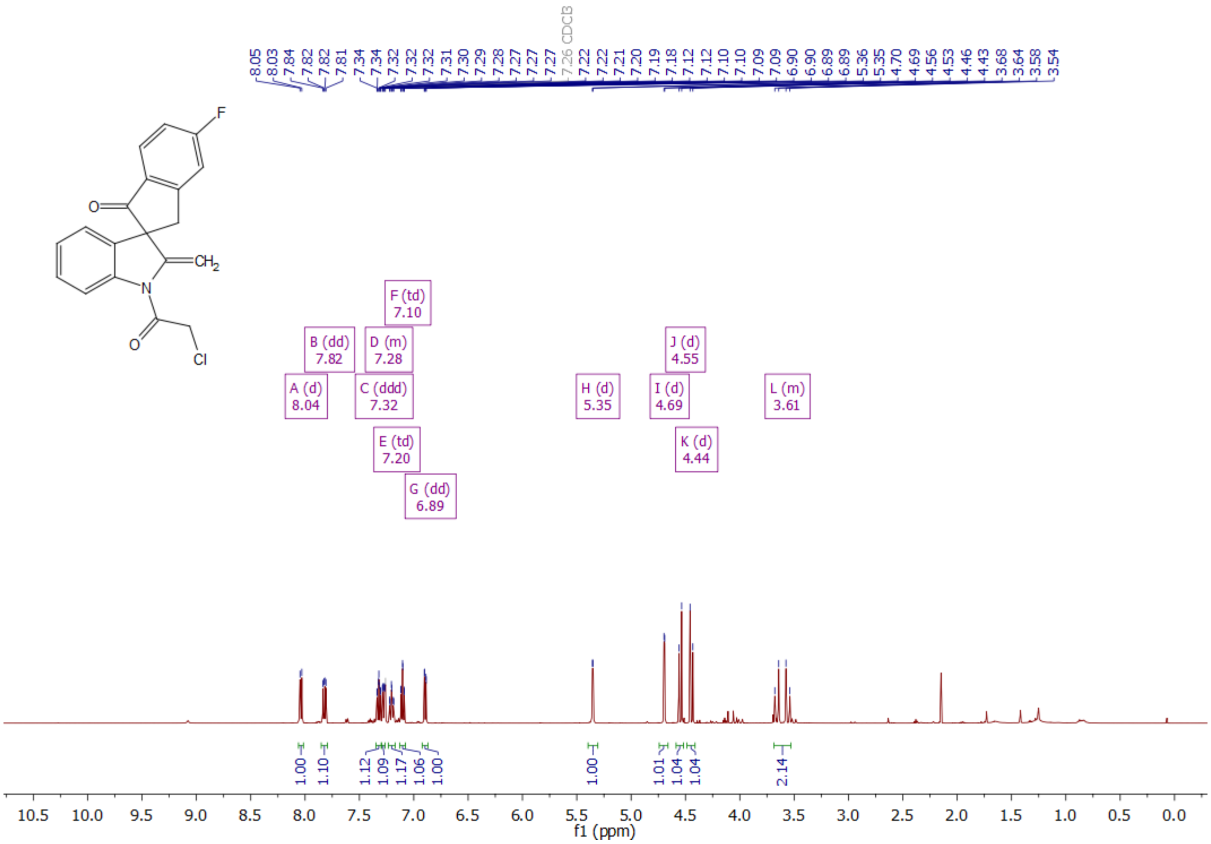

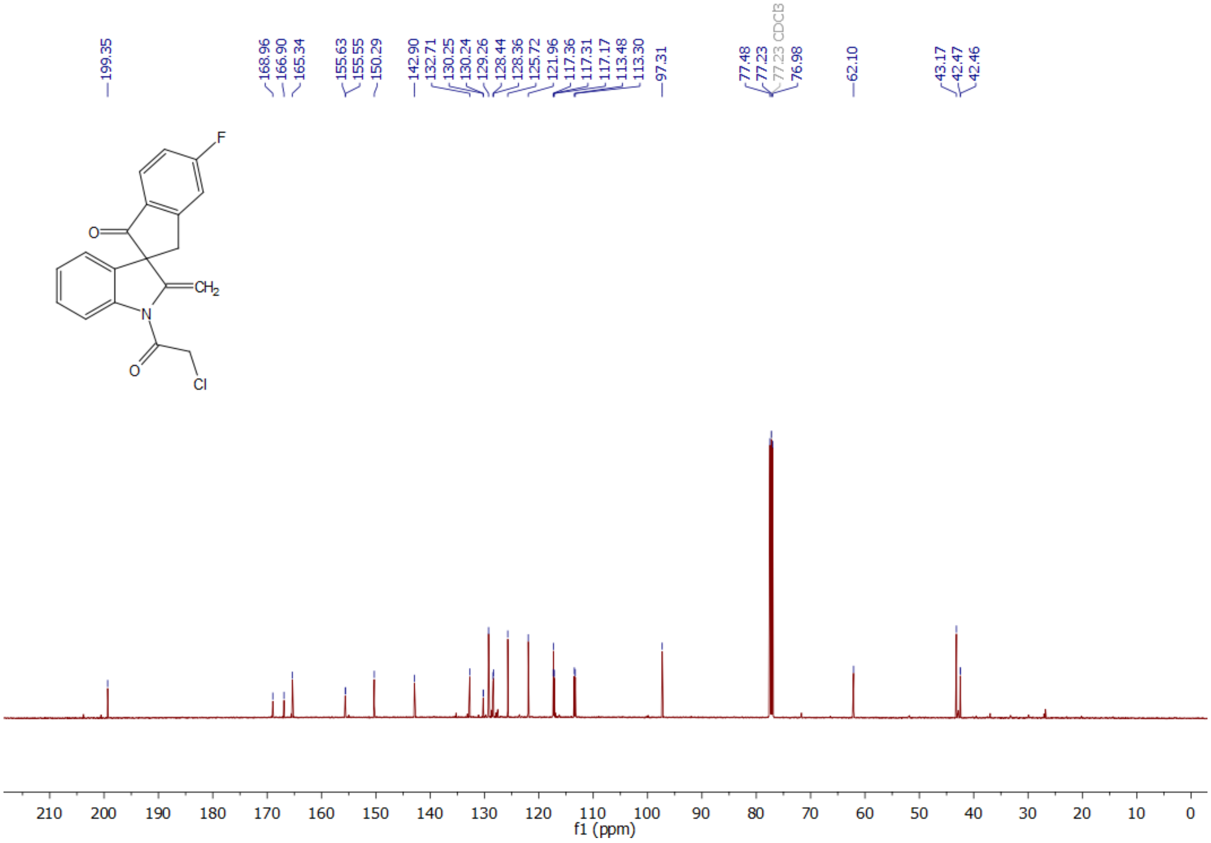

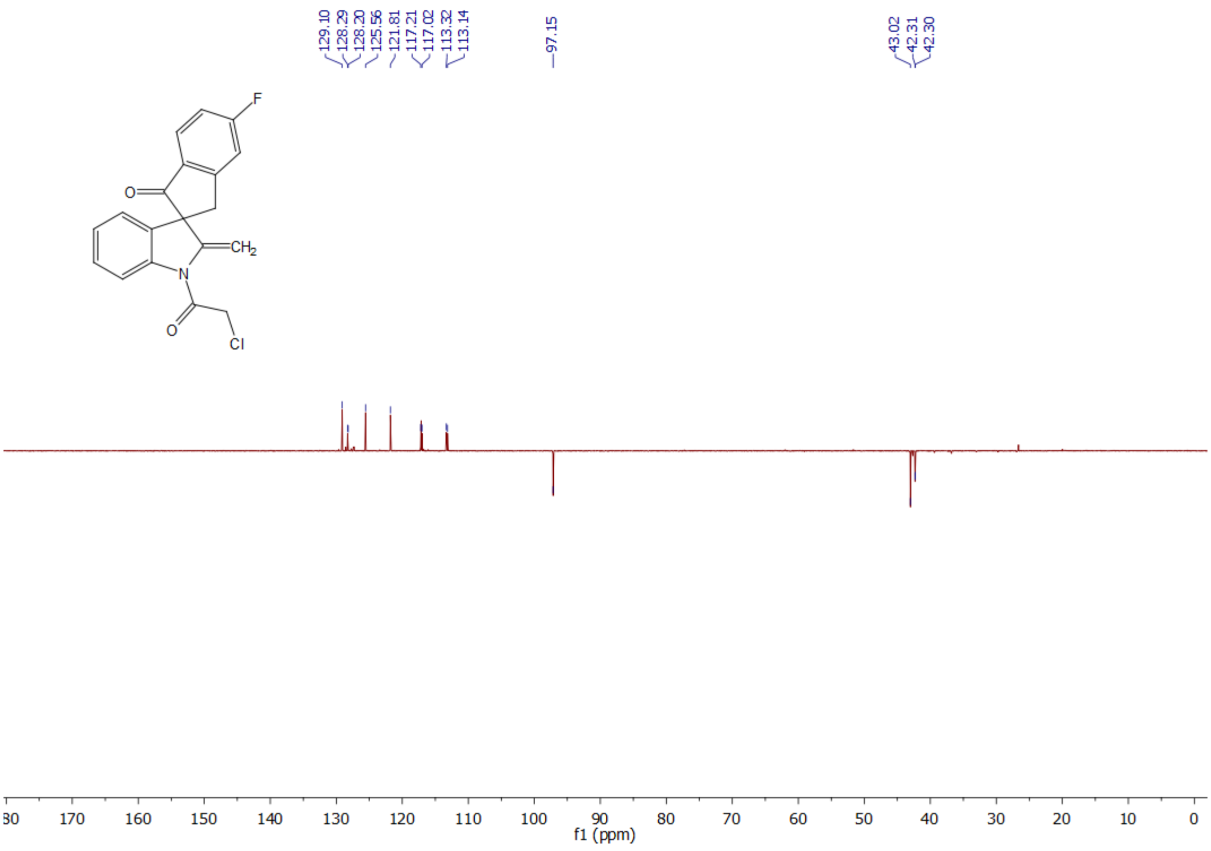
129.10
128.29
128.20
125.56
121.81
117.21
117.02
113.32
113.14
97.15
43.02
42.31
42.30
F
O
CH2
N
O
Cl
f1 (ppm)

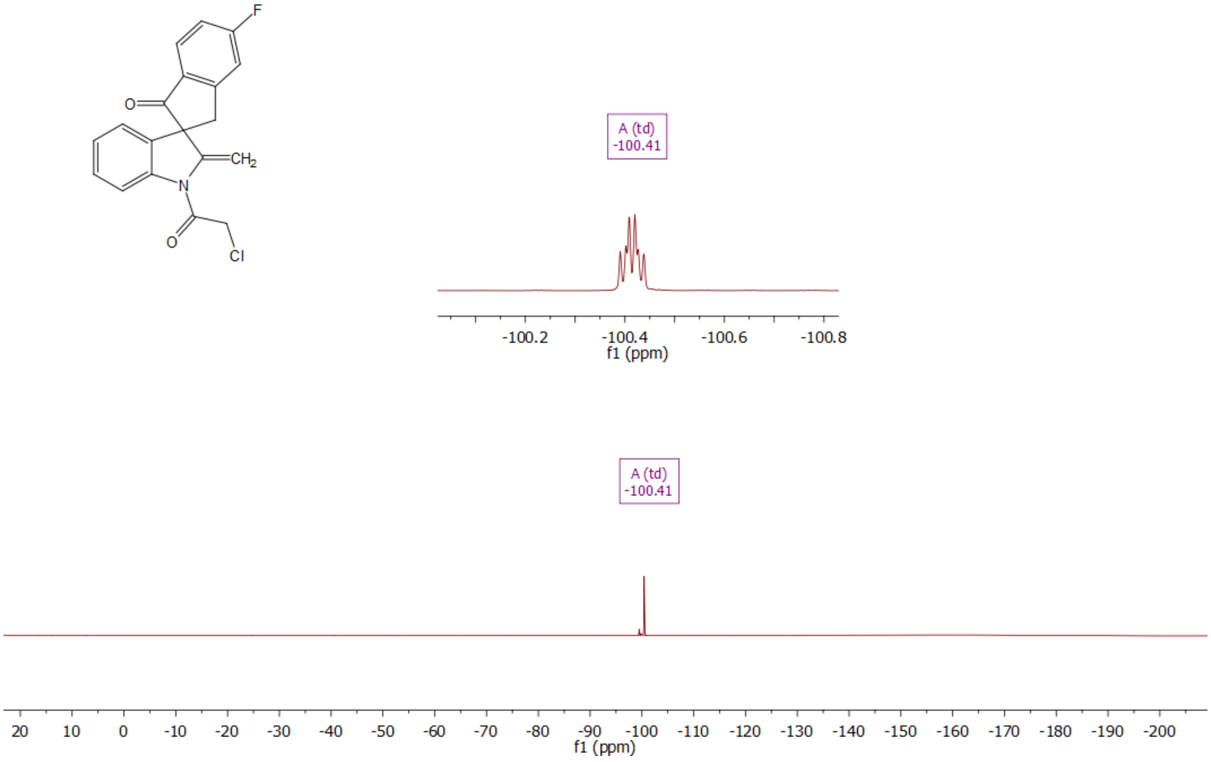
F
O
CH2
N
O
Cl
A (td)
-100.41
f1 (ppm)
A (td)
-100.41
f1 (ppm)

1'-(2-chloroacetyl)-2'-methylenespiro[cyclopenta[a]naphthalene-2,3'-indolin]-1(3H)-one [**D4**]:

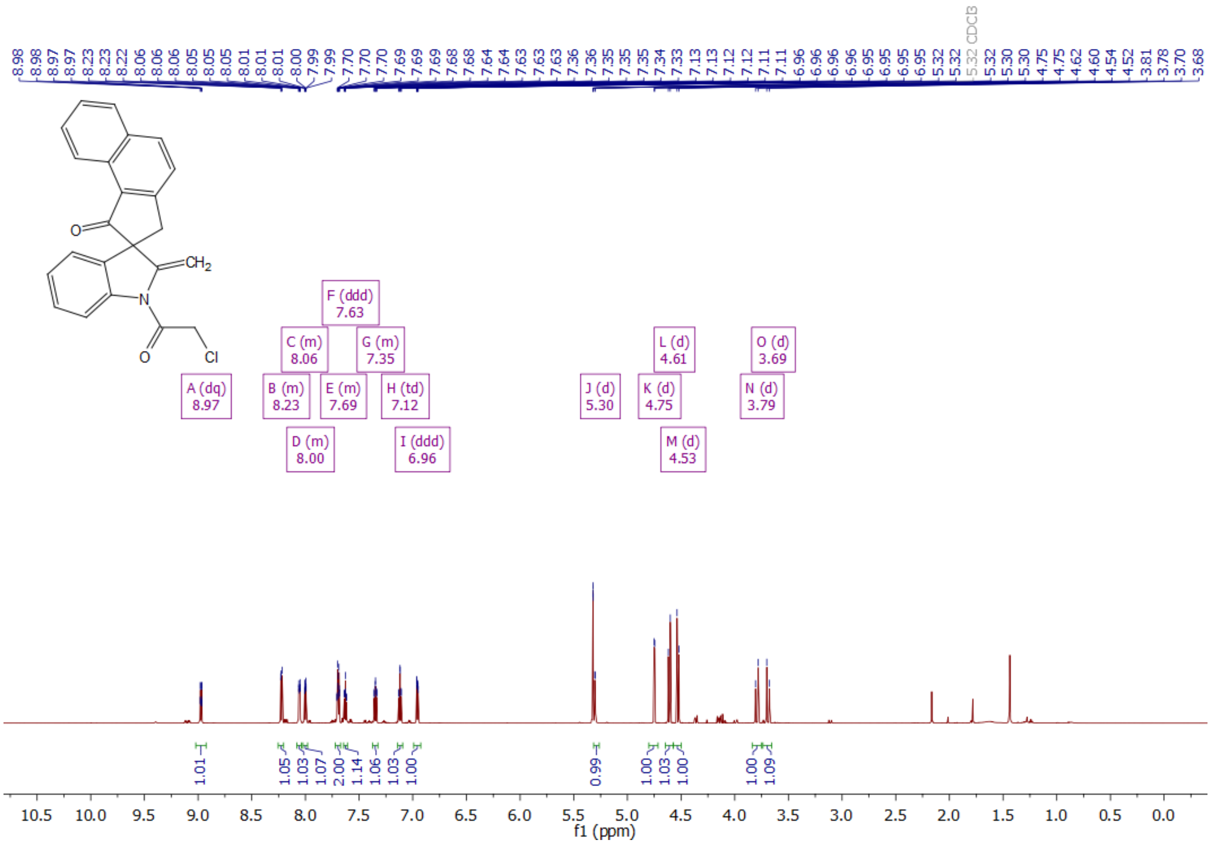

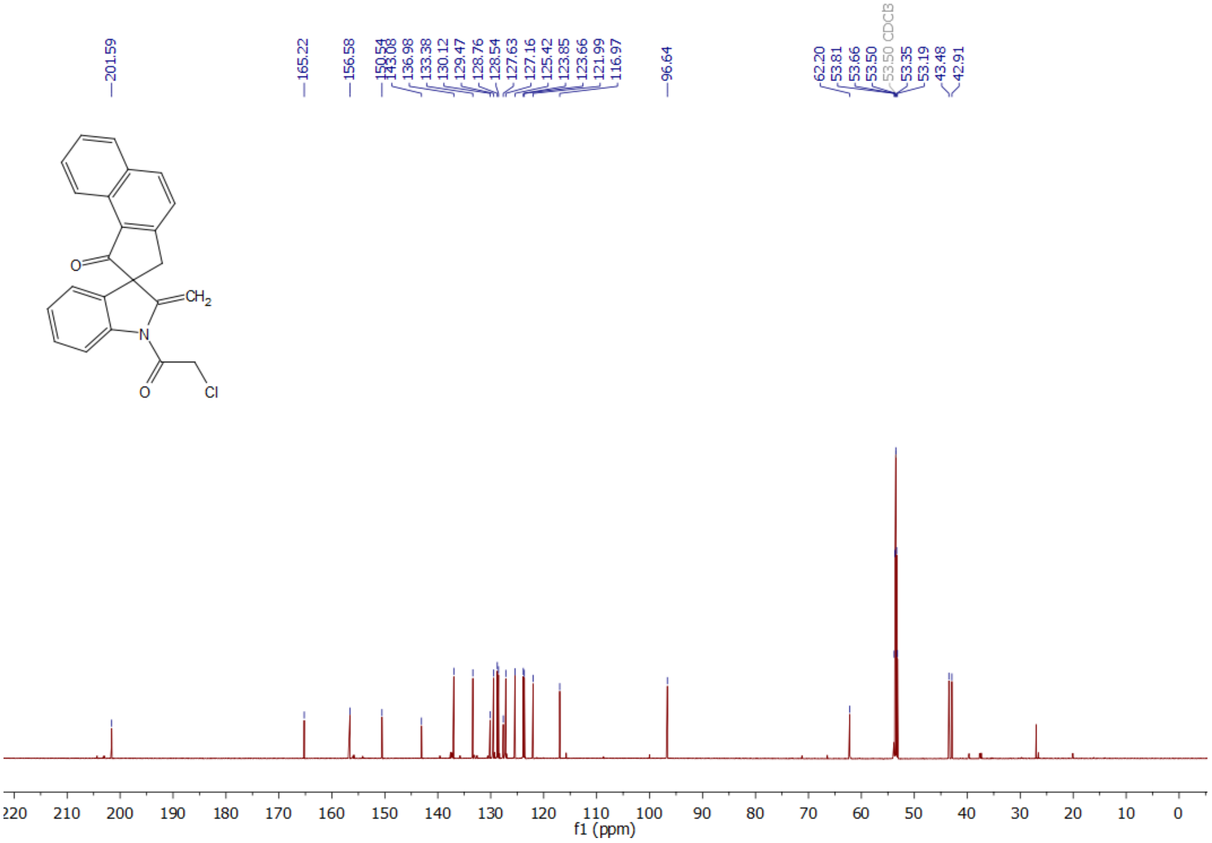

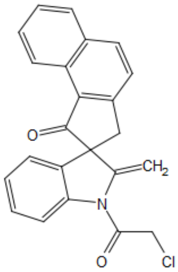
O
CH2
N
O
Cl

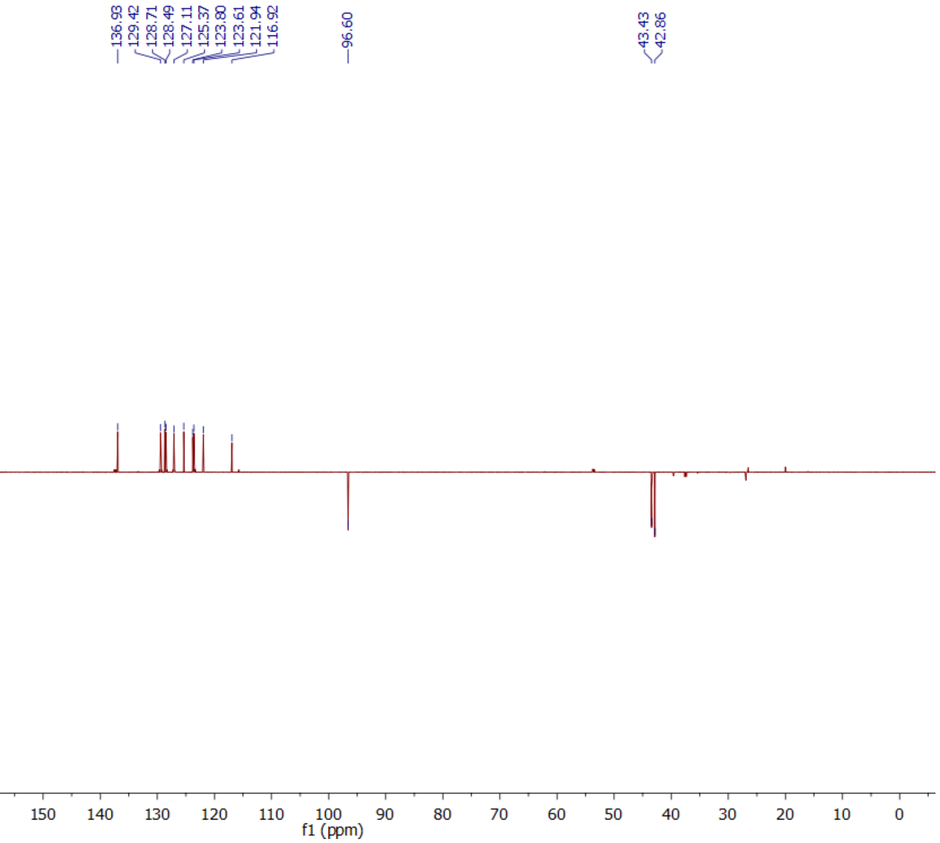
136.93
129.42
128.71
128.49
127.11
125.37
123.80
123.61
121.94
116.92
96.60
43.43
42.86
200 190 180 170 160 150 140 130 120 110 100 90 80 70 60 50 40 30 20 10 0
f1 (ppm)

5'-chloro-1'-(2-chloroacetyl)-5-fluoro-2'-methylenespiro[indene-2,3'-indolin]-1(3H)-one [**D5**]:

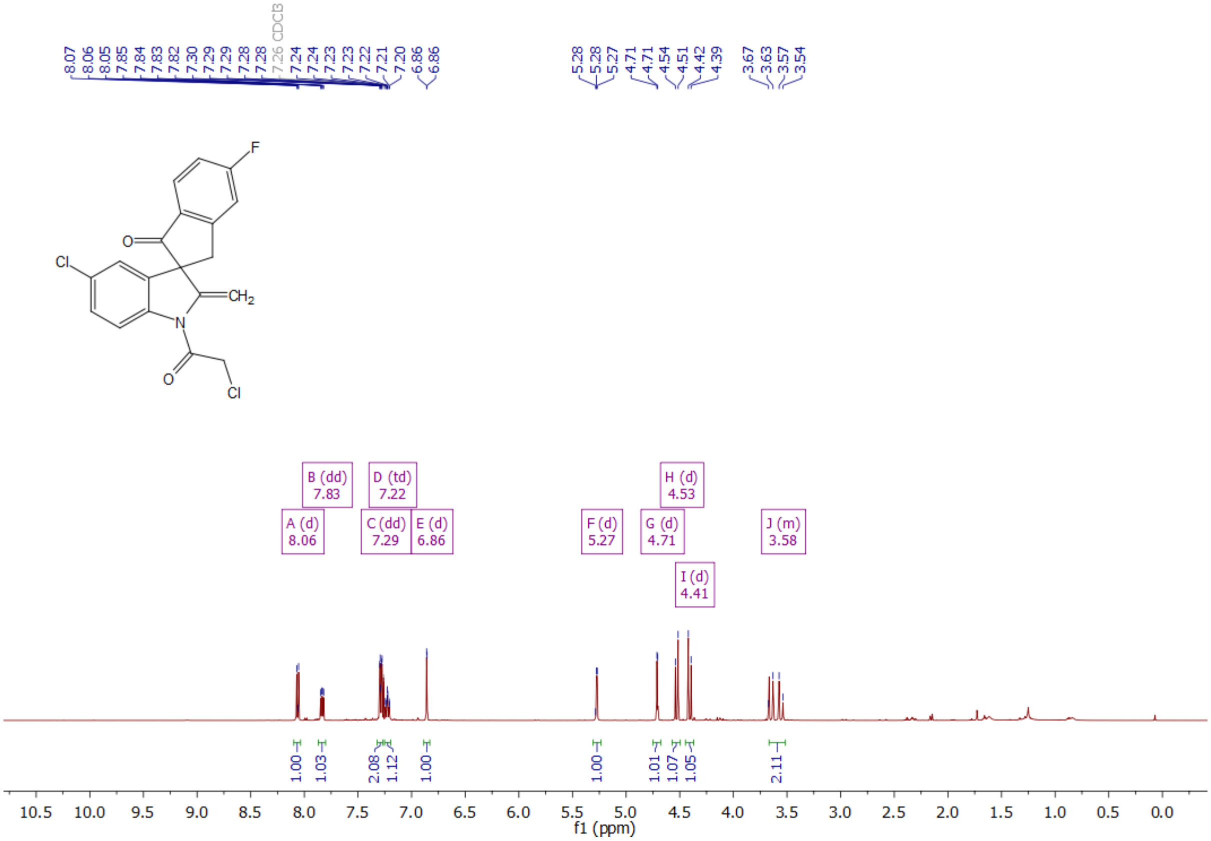

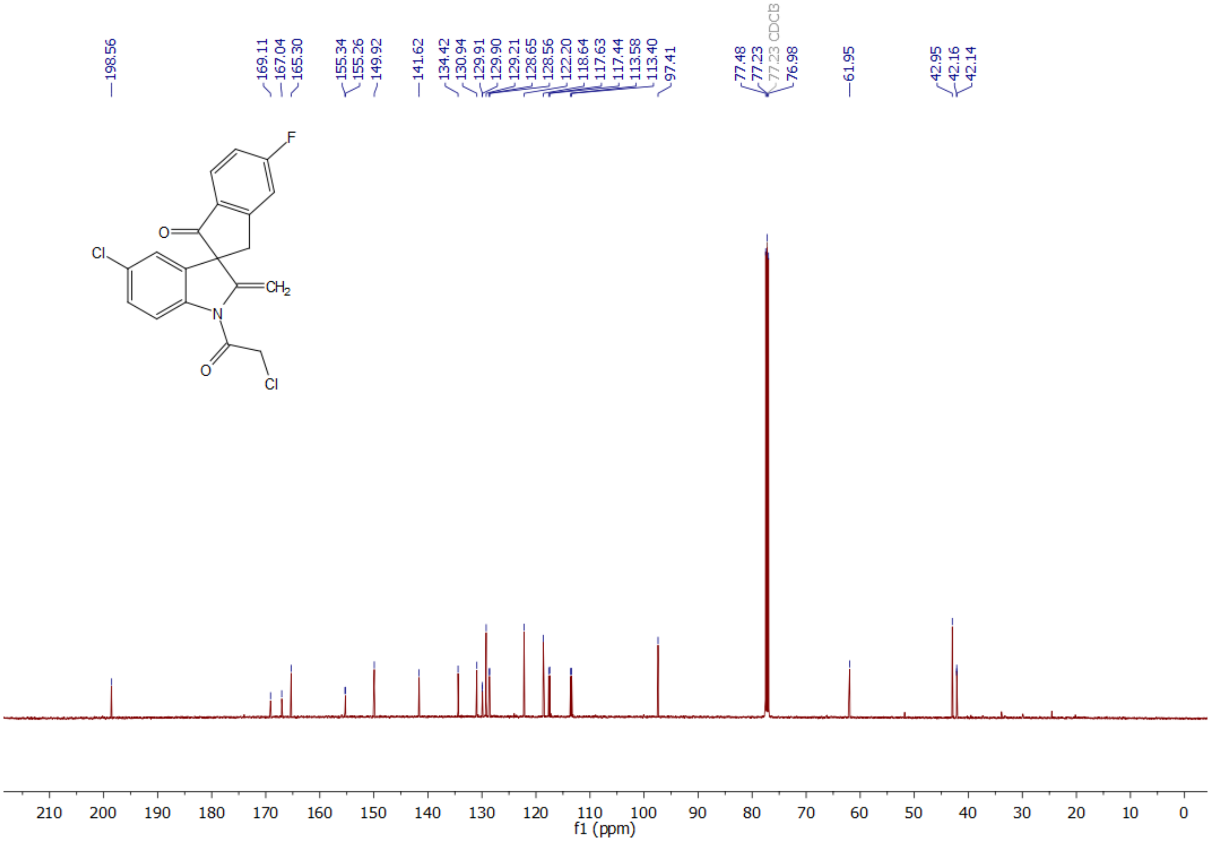

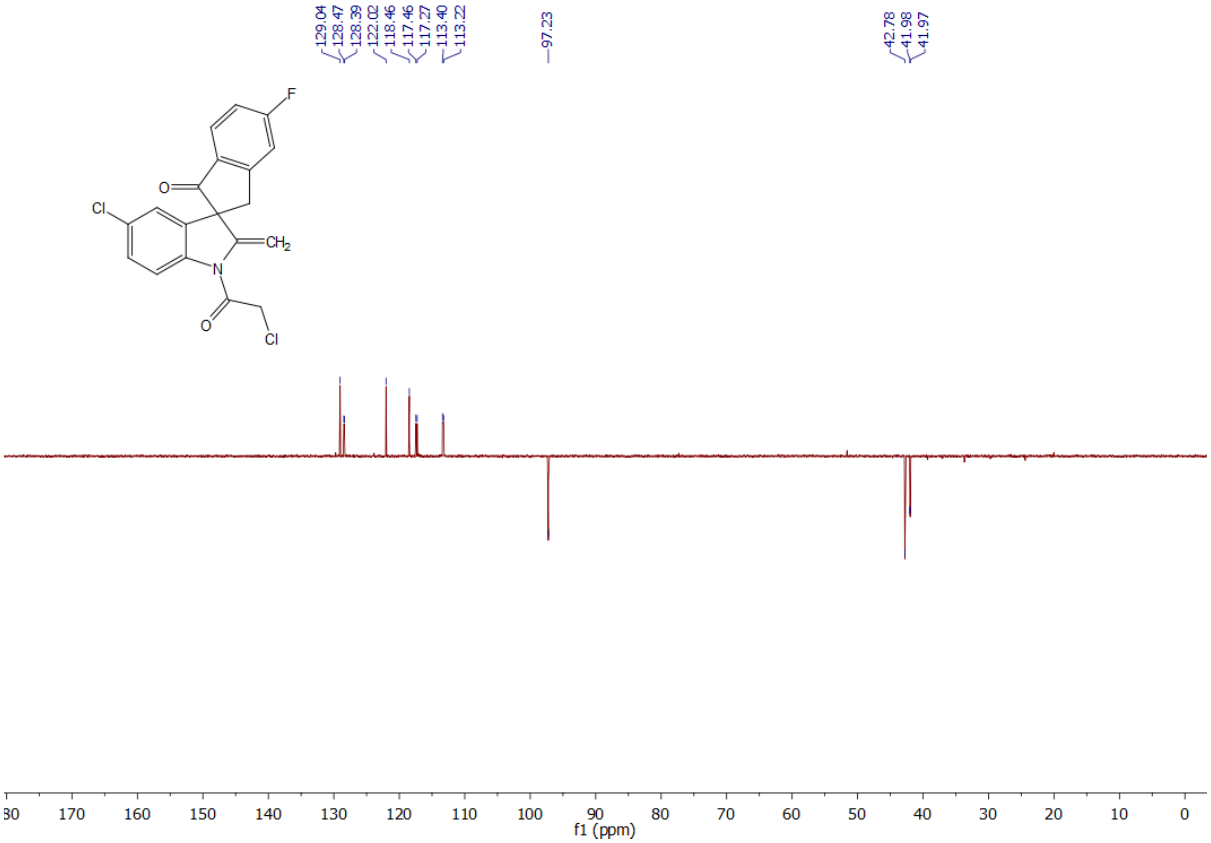
129.04
128.47
128.39
122.02
118.46
117.46
117.27
113.40
113.22
97.23
42.78
41.98
41.97
F
O
Cl
N
CH2
O
Cl
f1 (ppm)

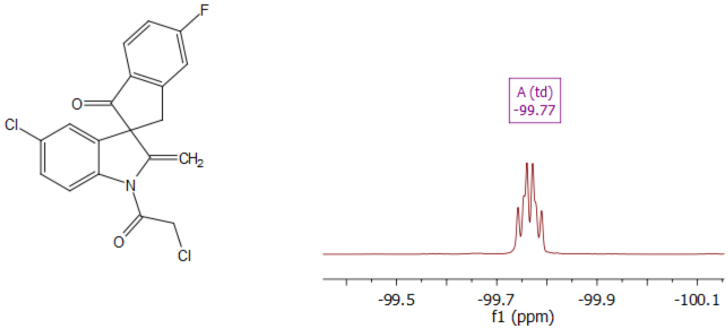
F
O
Cl
N
CH2
O
Cl
A (td)
-99.77
-99.5
-99.7
-99.9
-100.1
f1 (ppm)

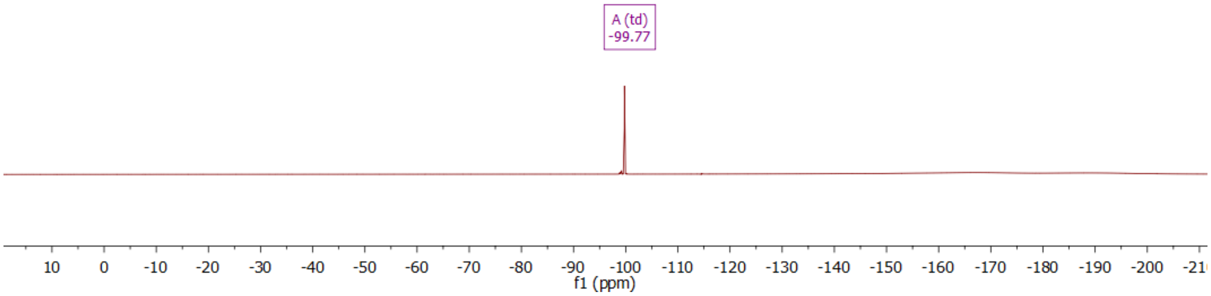
A (td)
-99.77
f1 (ppm)

6-chloro-1'-(2-chloroacetyl)-5'-fluoro-2'-methylenespiro[indene-2,3'-indolin]-1(3H)-one [**D6**]:

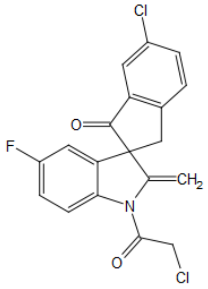

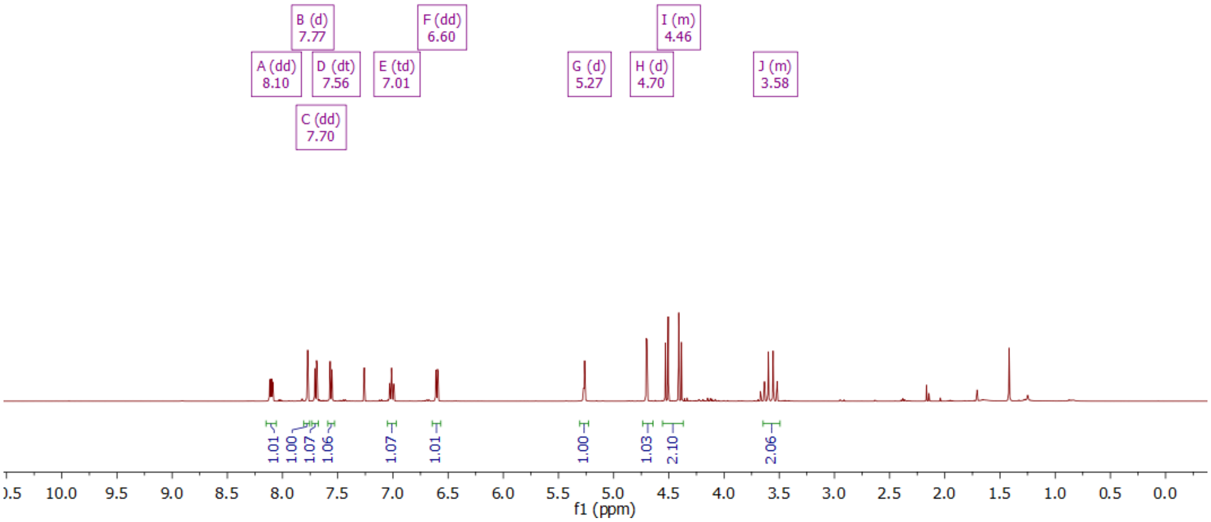

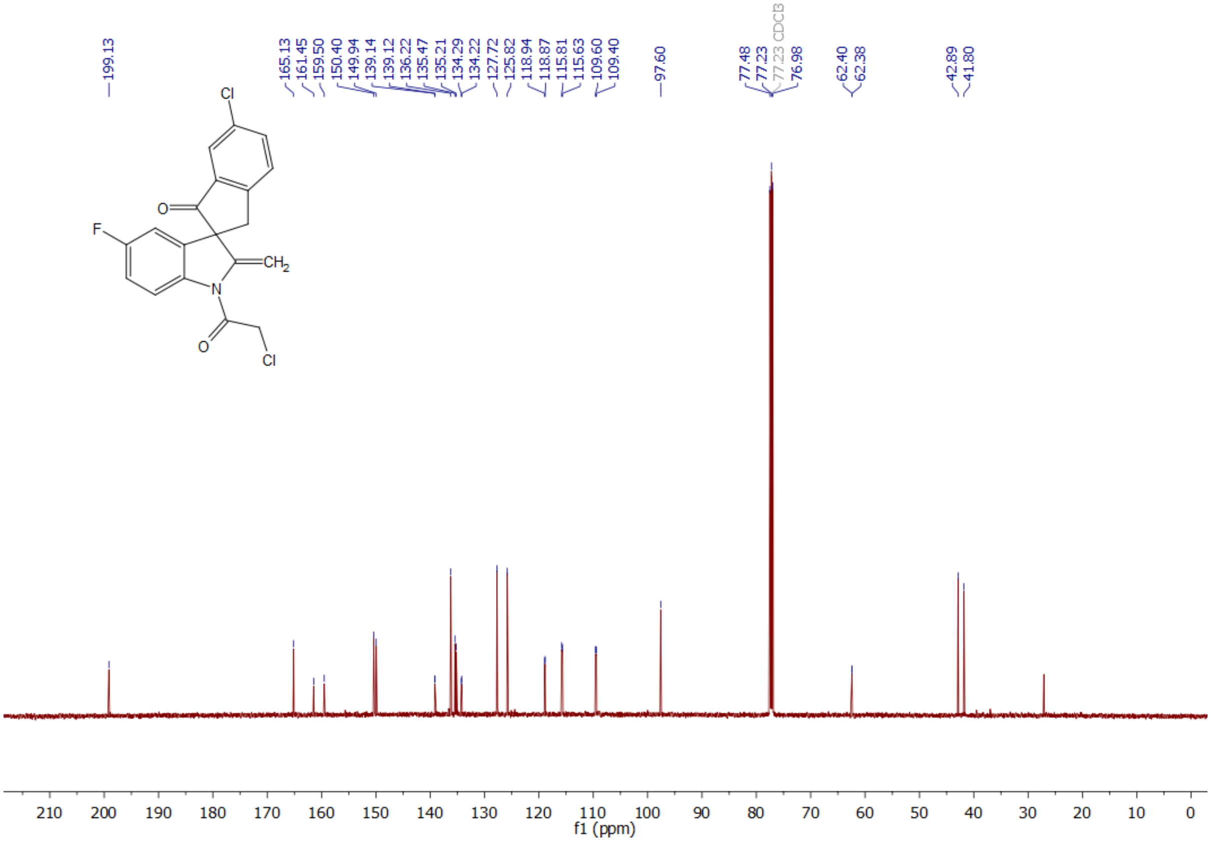

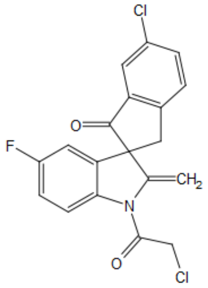

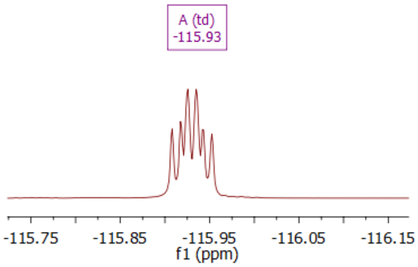
A (td)
-115.93
-115.75
-115.85
-115.95
-116.05
-116.15
f1 (ppm)

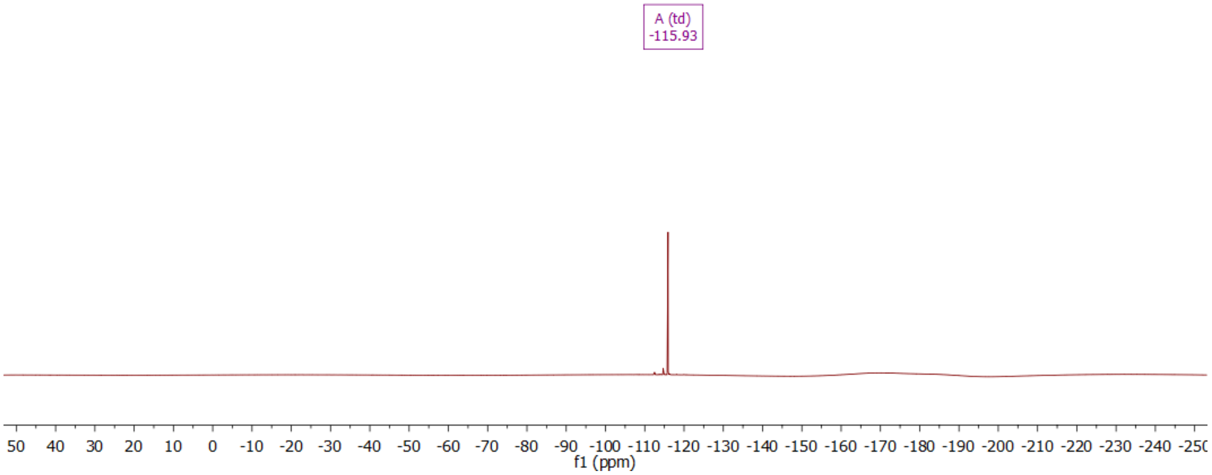
A (td)
-115.93
50
40
30
20
10
0
-10
-20
-30
-40
-50
-60
-70
-80
-90
-100
-110
-120
-130
-140
-150
-160
-170
-180
-190
-200
-210
-220
-230
-240
f1 (ppm)

1'-(2-chloroacetyl)-5'-fluoro-6-methoxy-2'-methylenespiro[indene-2,3'-indolin]-1(3H)-one [**D7**]:

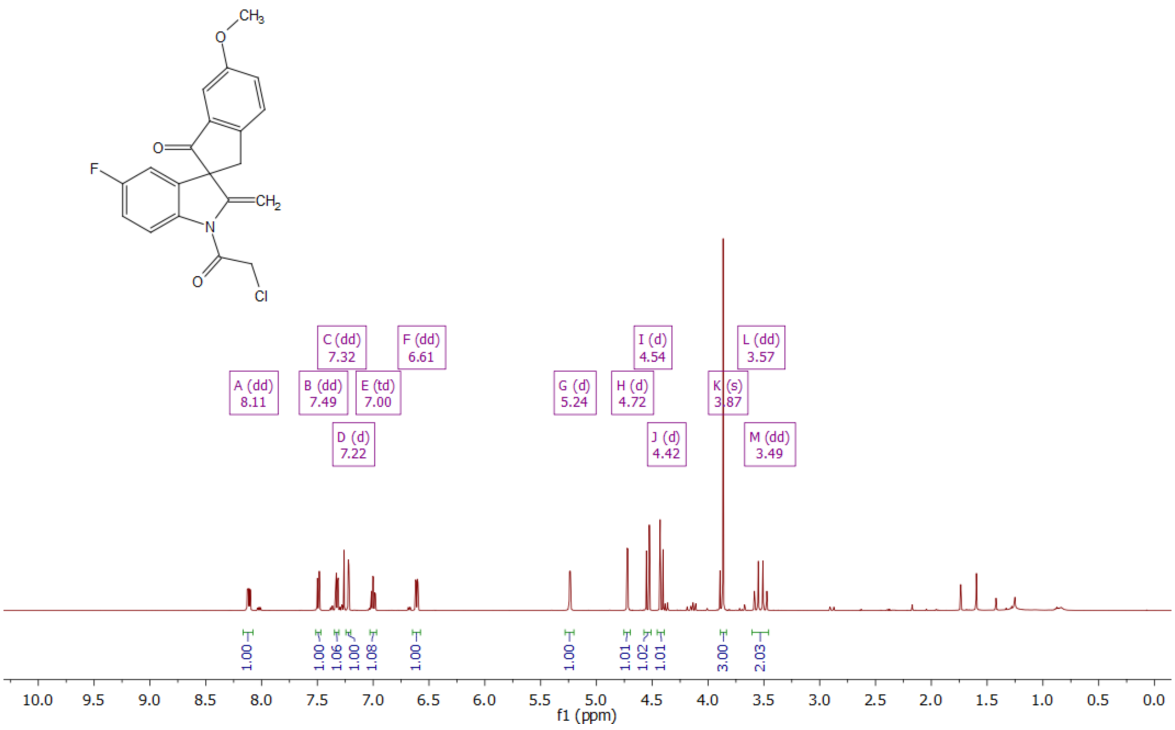

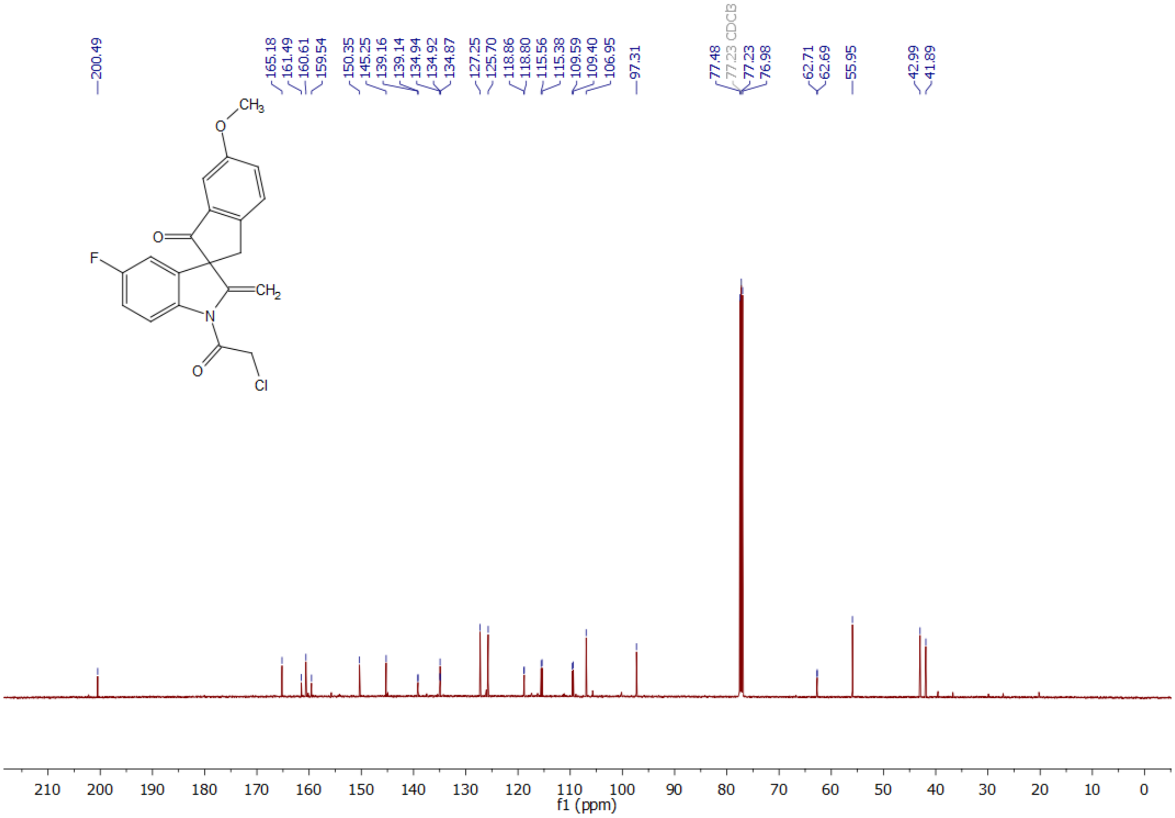

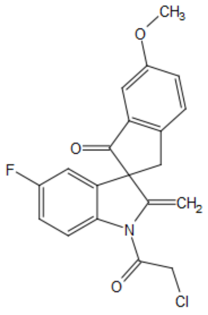
CH3
O
O
F
CH2
N
O
Cl

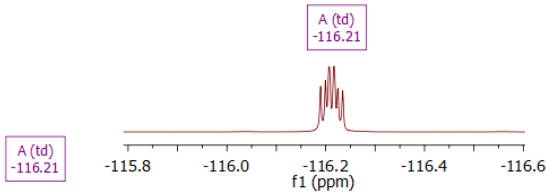
A (td)
-116.21
A (td)
-116.21
-115.8
-116.0
-116.2
-116.4
-116.6
f1 (ppm)

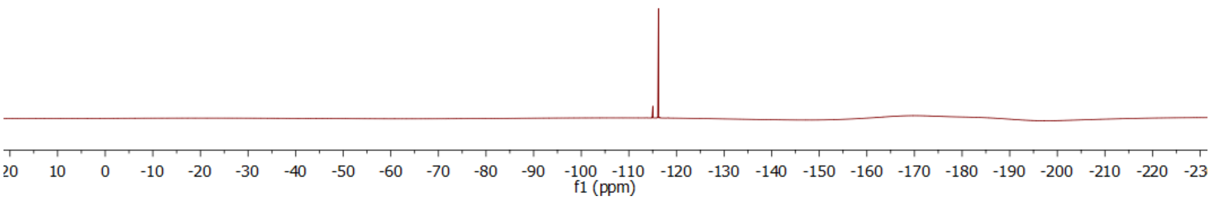
20 10 0 -10 -20 -30 -40 -50 -60 -70 -80 -90 -100 -110 -120 -130 -140 -150 -160 -170 -180 -190 -200 -210 -220 -23
f1 (ppm)

5'-chloro-1'-(2-chloroacetyl)-5,6-dimethoxy-2'-methylenespiro[indene-2,3'-indolin]-1(3H)-one [**D8**]:

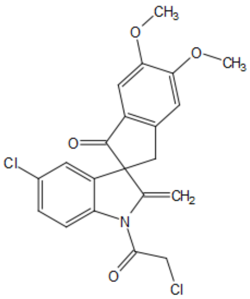

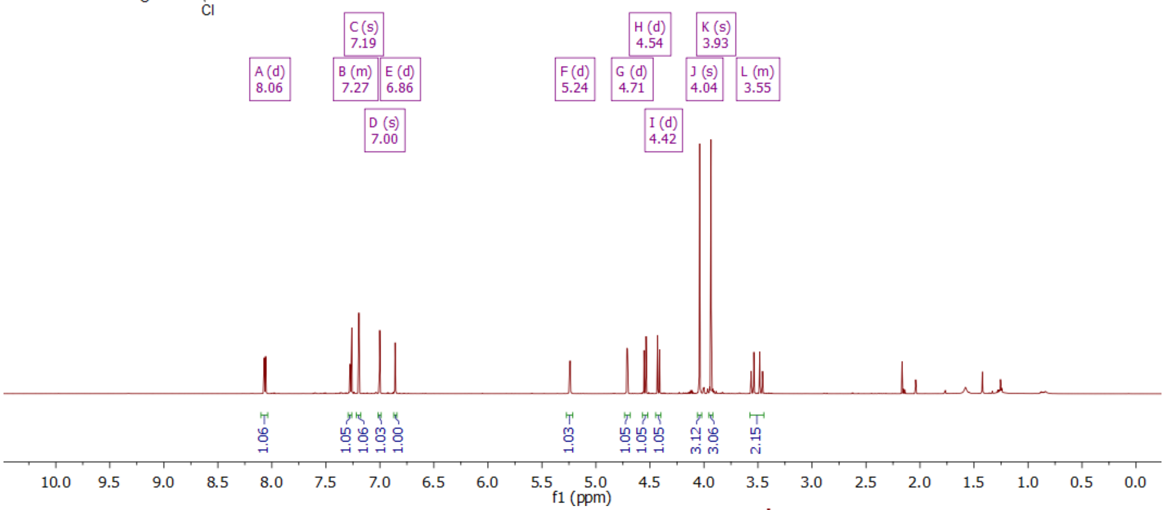

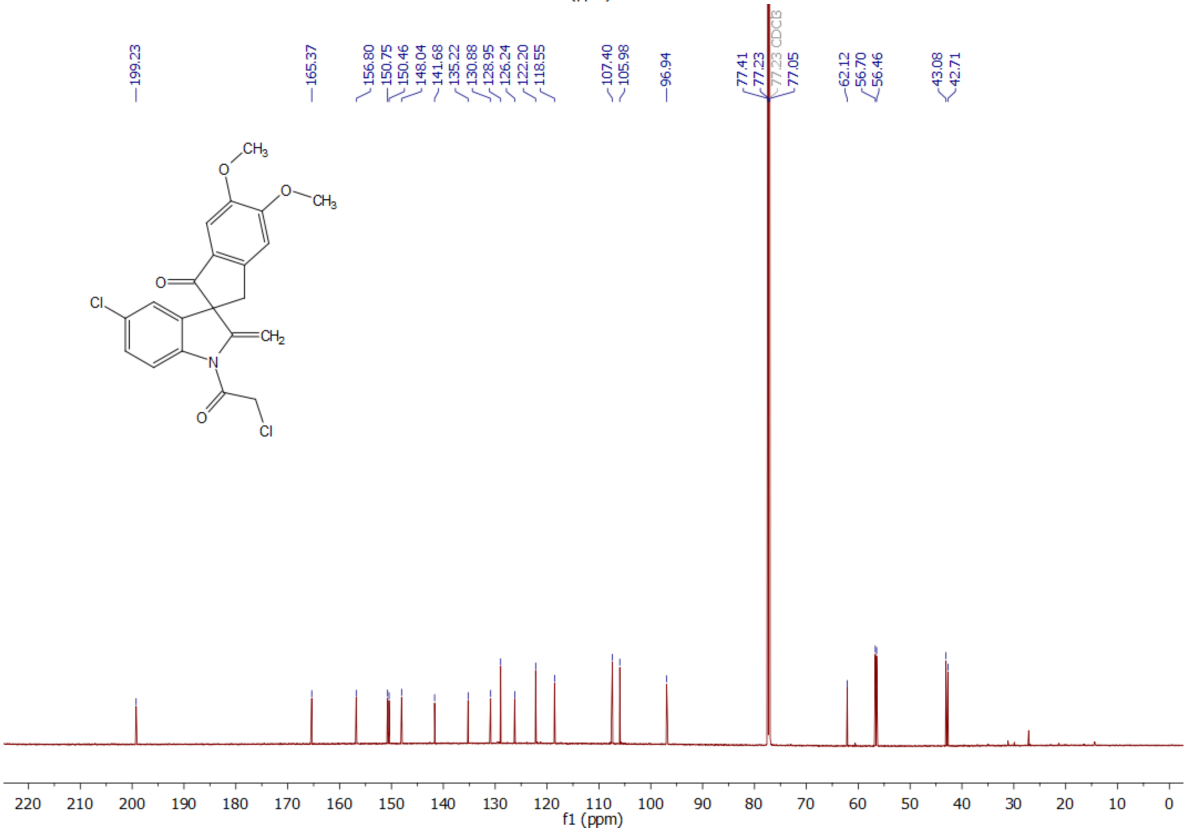

1'-acetyl-2'-methylenespiro[indene-2,3'-indolin]-1(3H)-one **[D9]**:

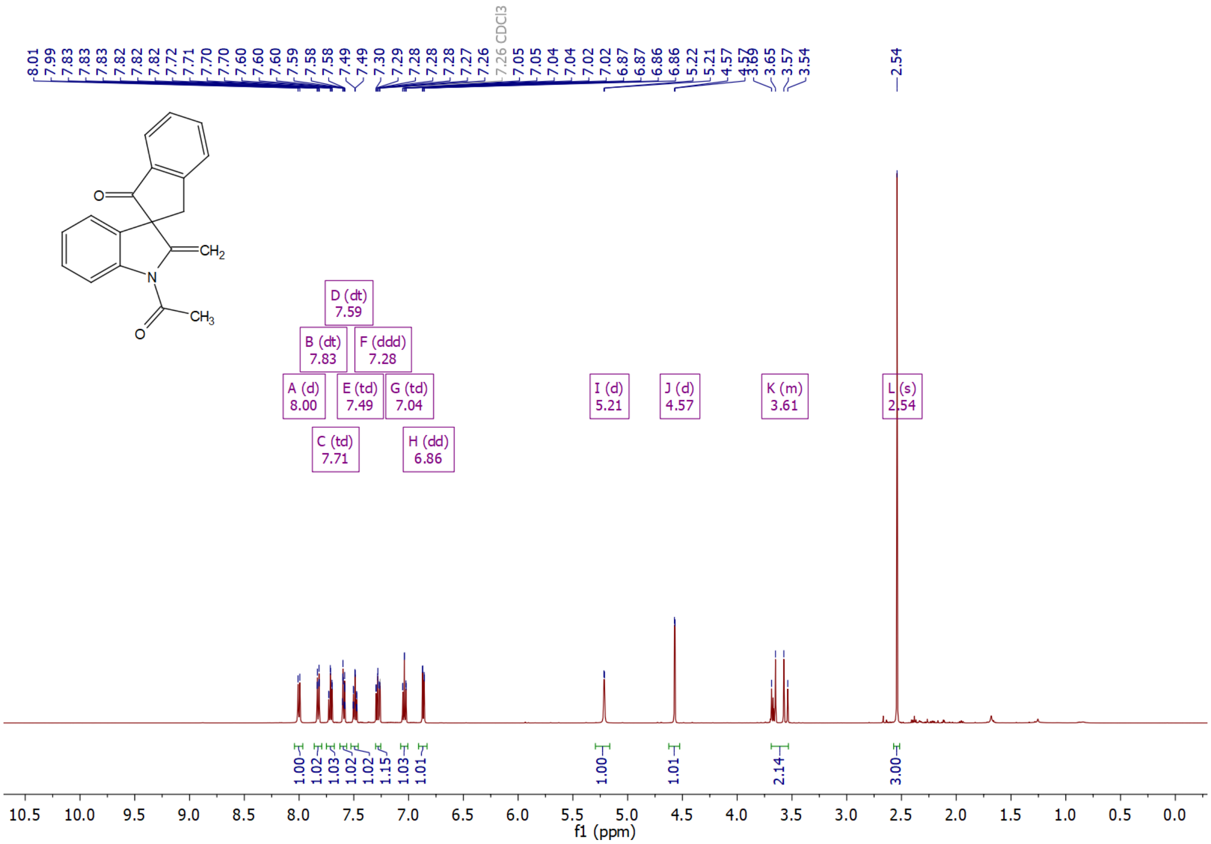

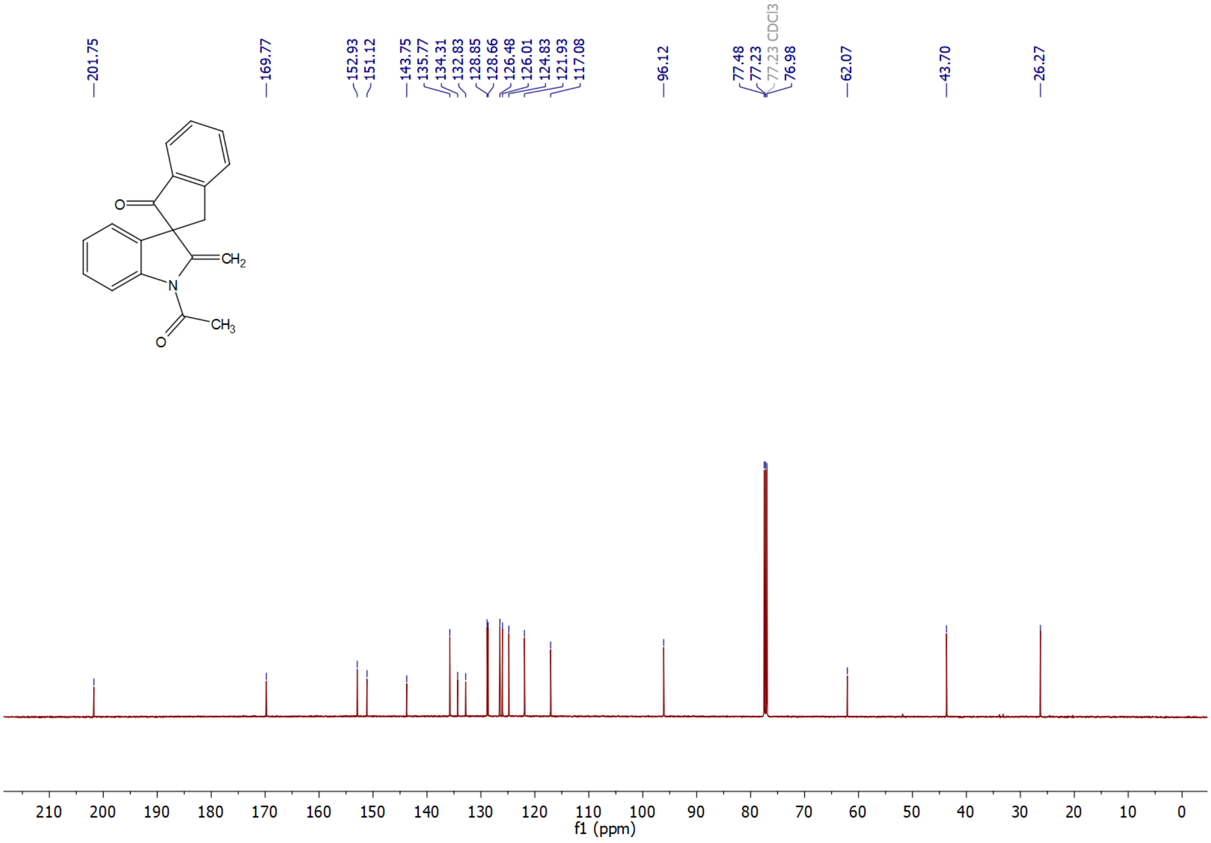

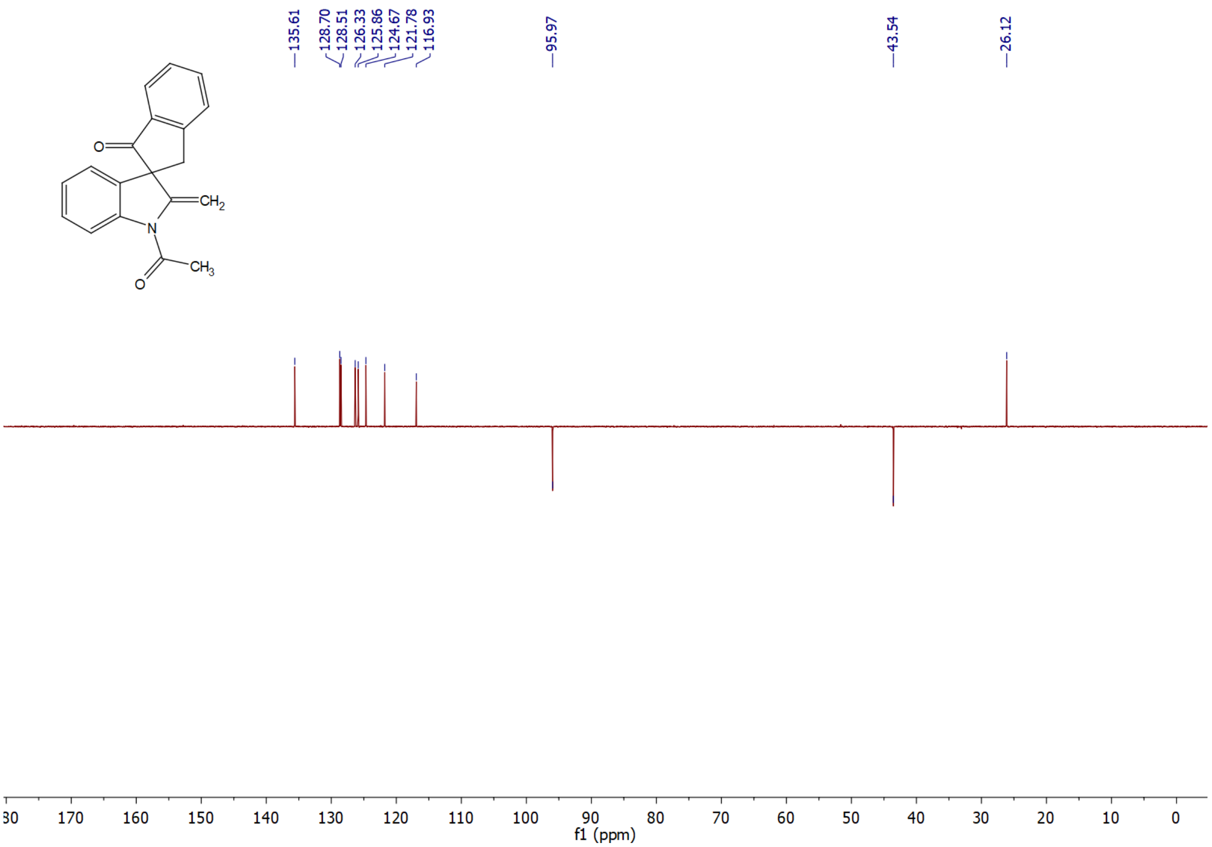
135.61
128.70
128.51
126.33
125.86
124.67
121.78
116.93
95.97
43.54
26.12
O
CH2
N
CH3
O
180 170 160 150 140 130 120 110 100 90 80 70 60 50 40 30 20 10 0
f1 (ppm)

2'-methylene-1'-(2-phenoxyacetyl)spiro[indene-2,3'-indolin]-1(3H)-one [**D10**]:

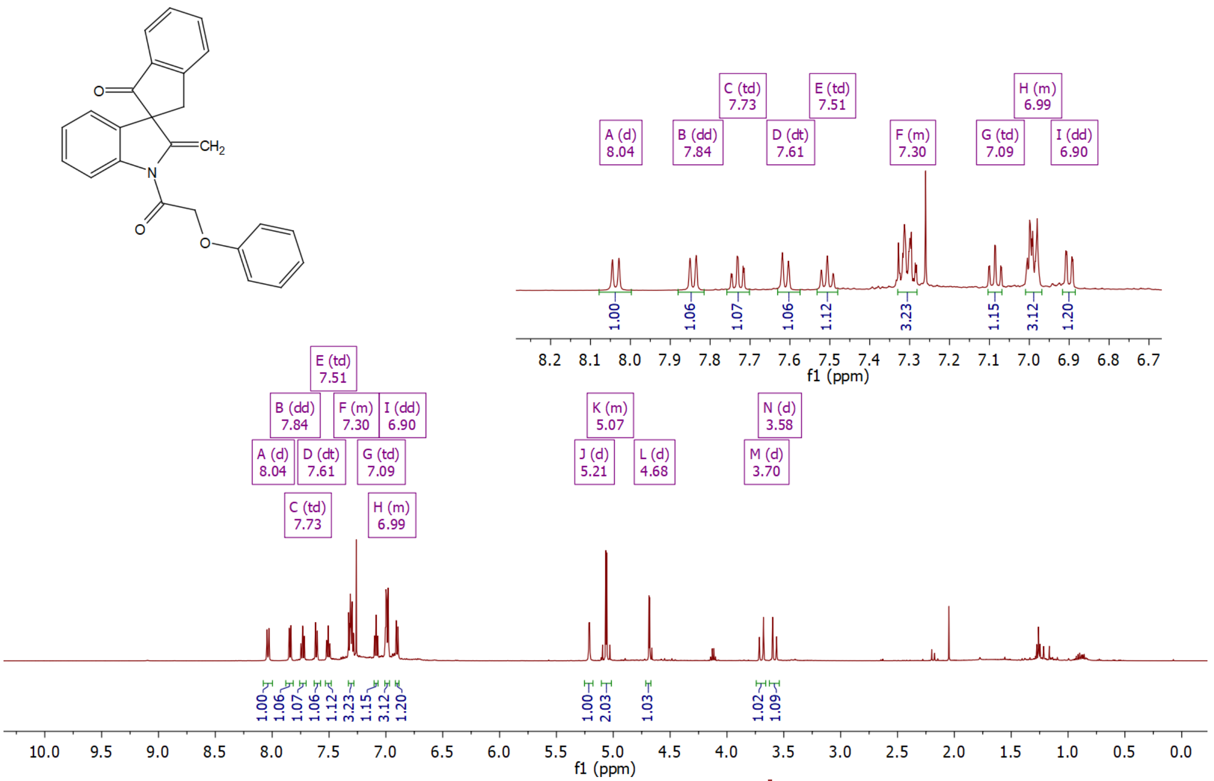

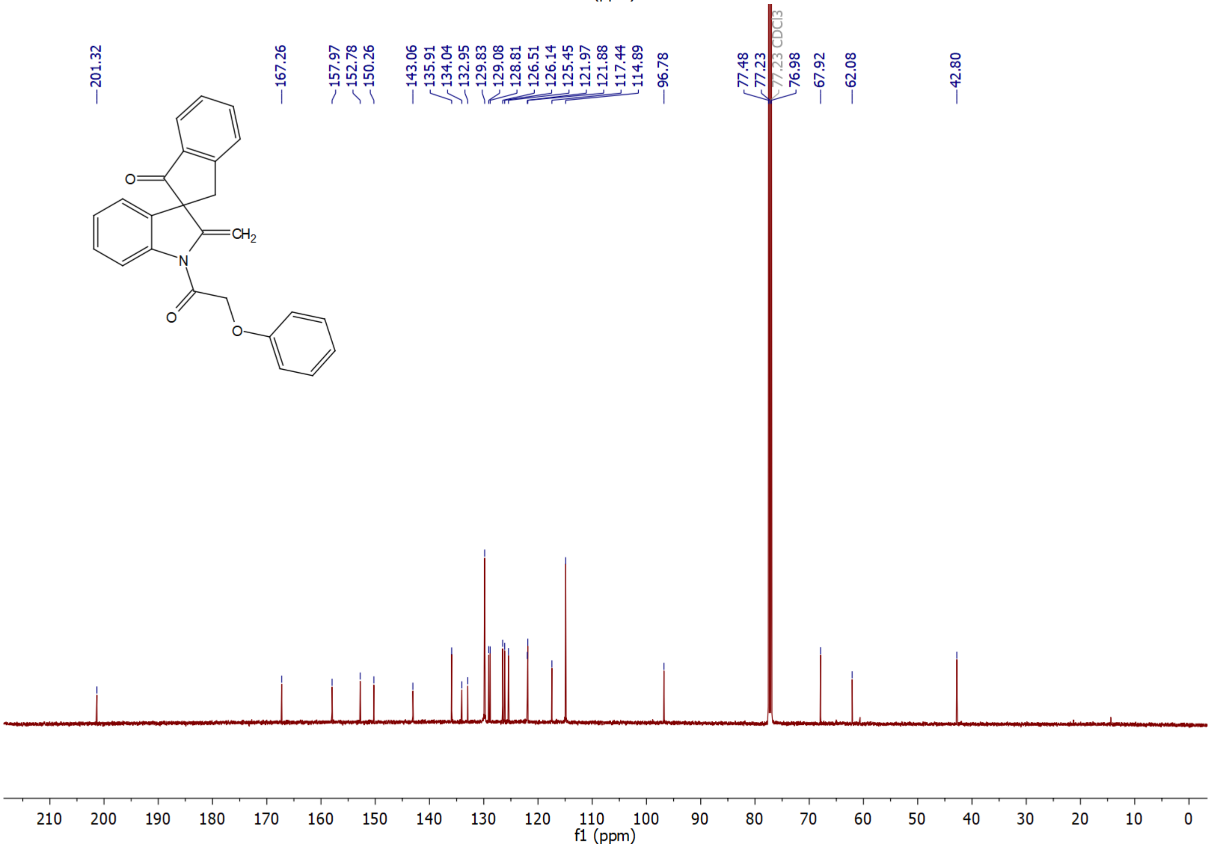

CLASS-E

***6'H-spiro[indene-2,12'-indolo[1,2-b]isoquinoline]-1,6'(3H)-dione* [E1]:**

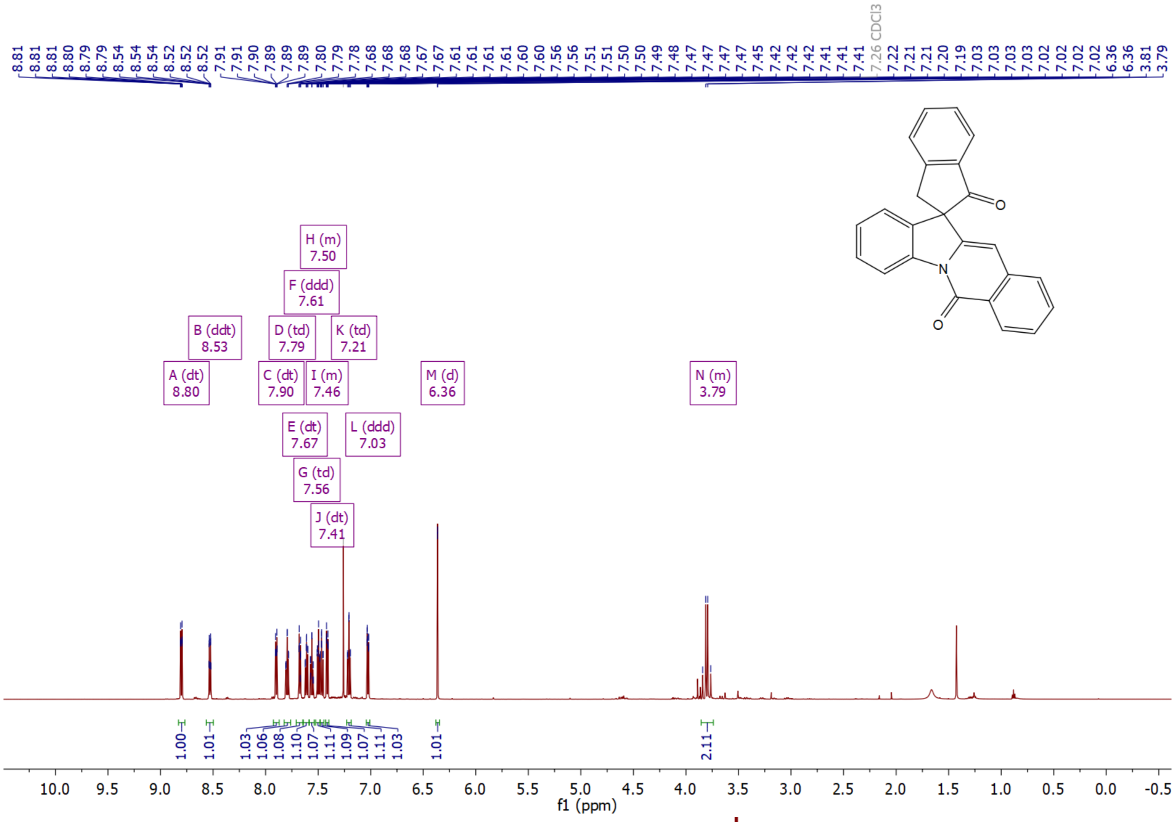

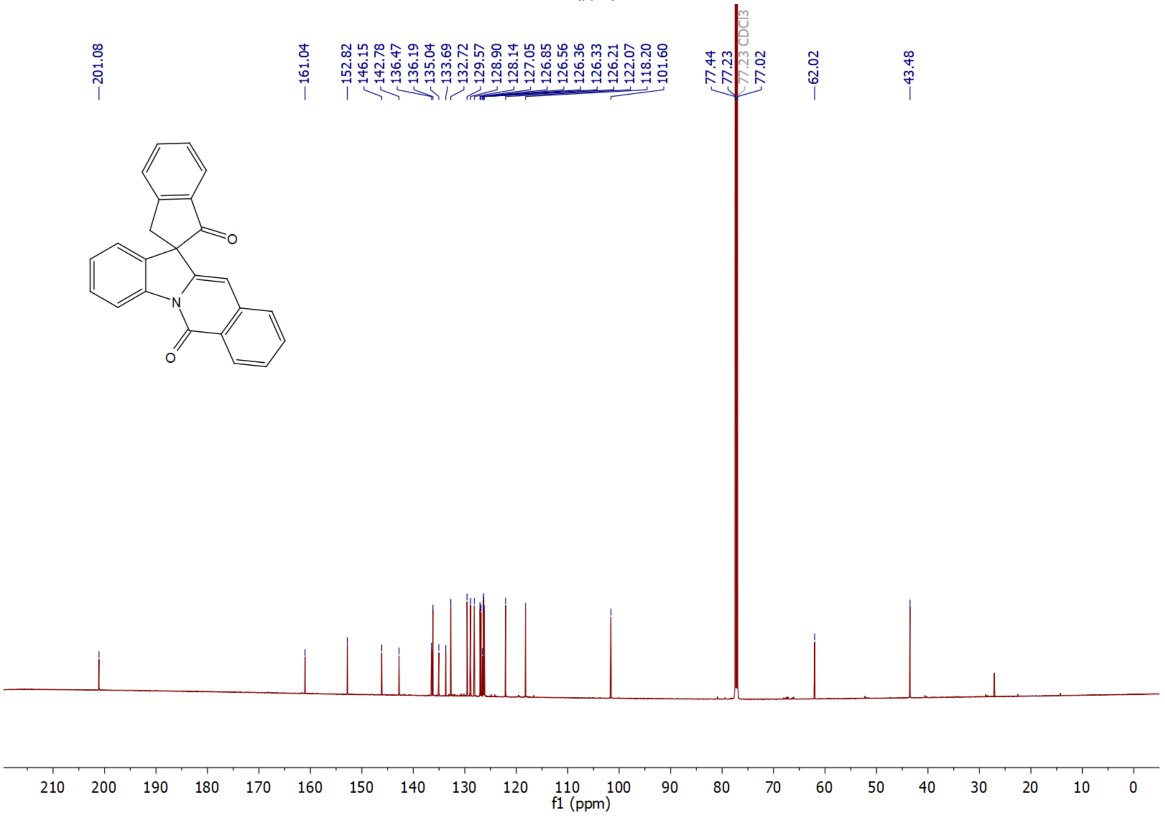

8'-methyl-6'H-spiro[indene-2,12'-indolo[1,2-b]isoquinoline]-1,6'(3H)-dione [**E2**]:

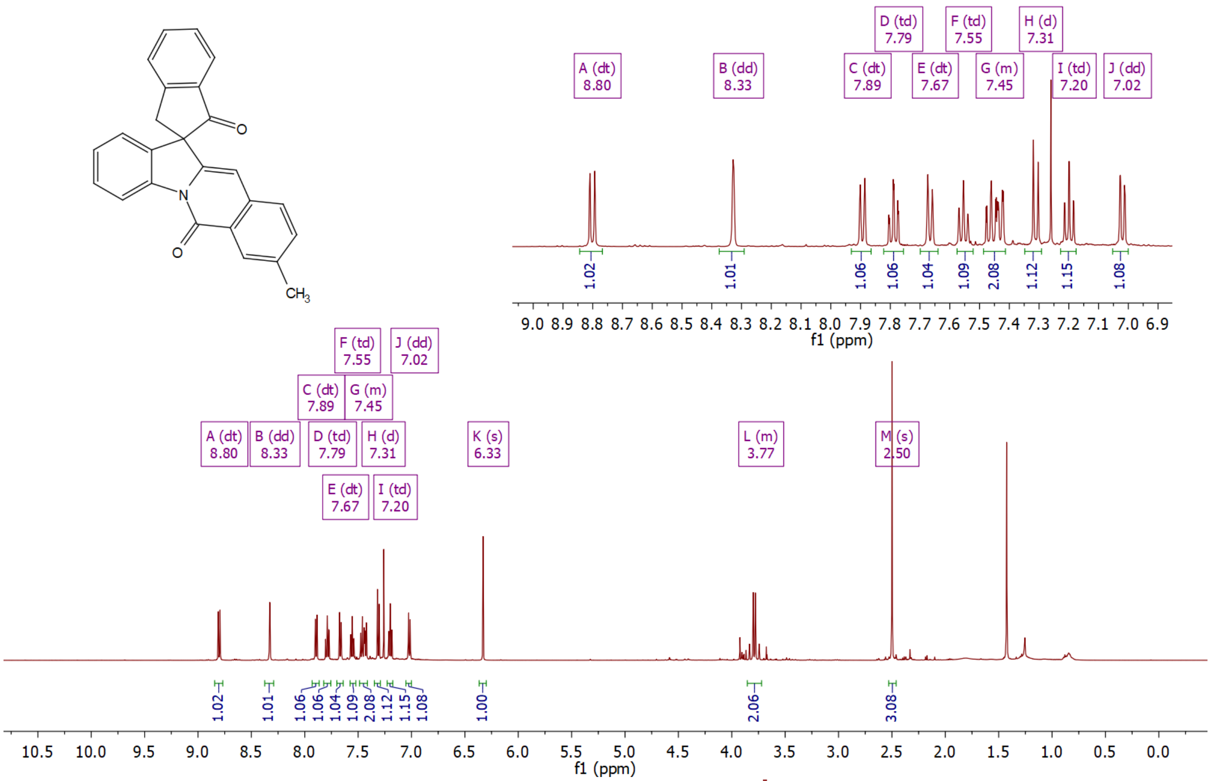

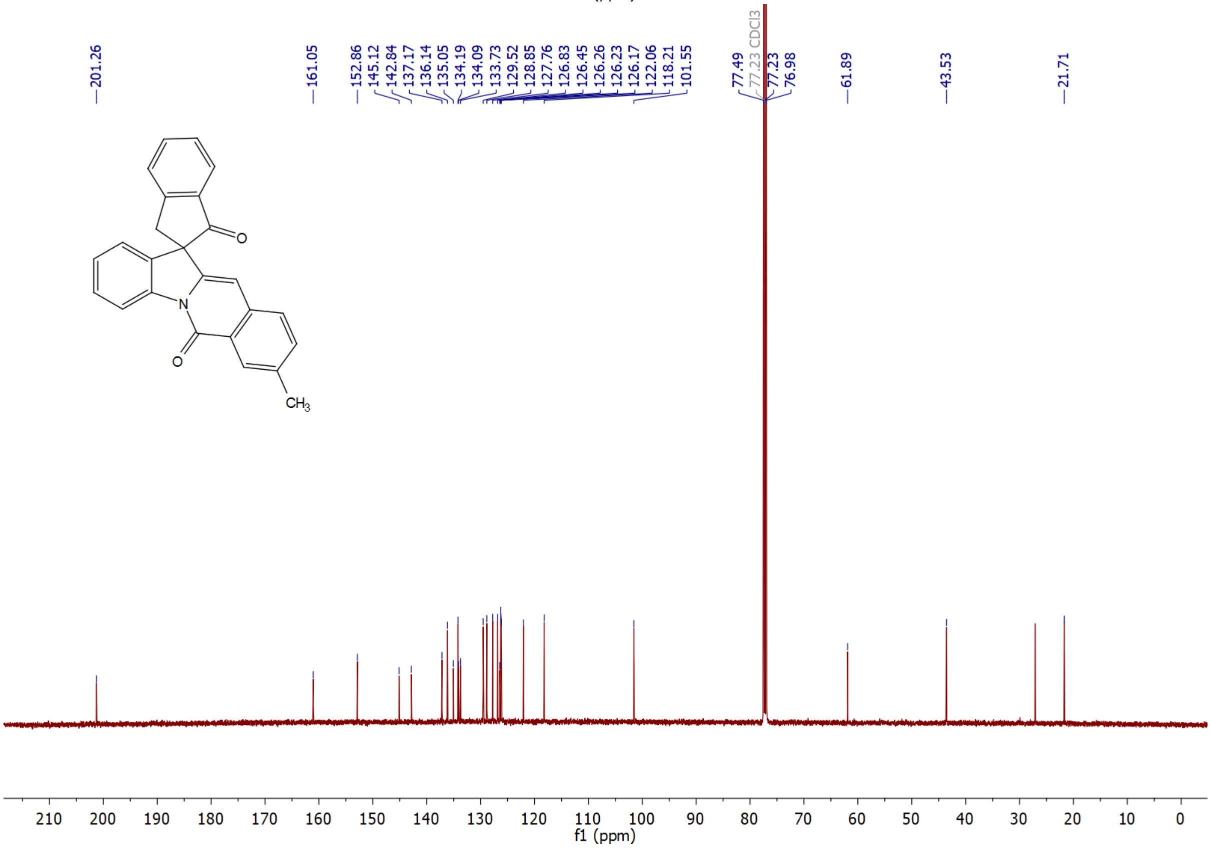

8'-methoxy-6'H-spiro[indene-2,12'-indolo[1,2-b]isoquinoline]-1,6'(3H)-dione [E3]:

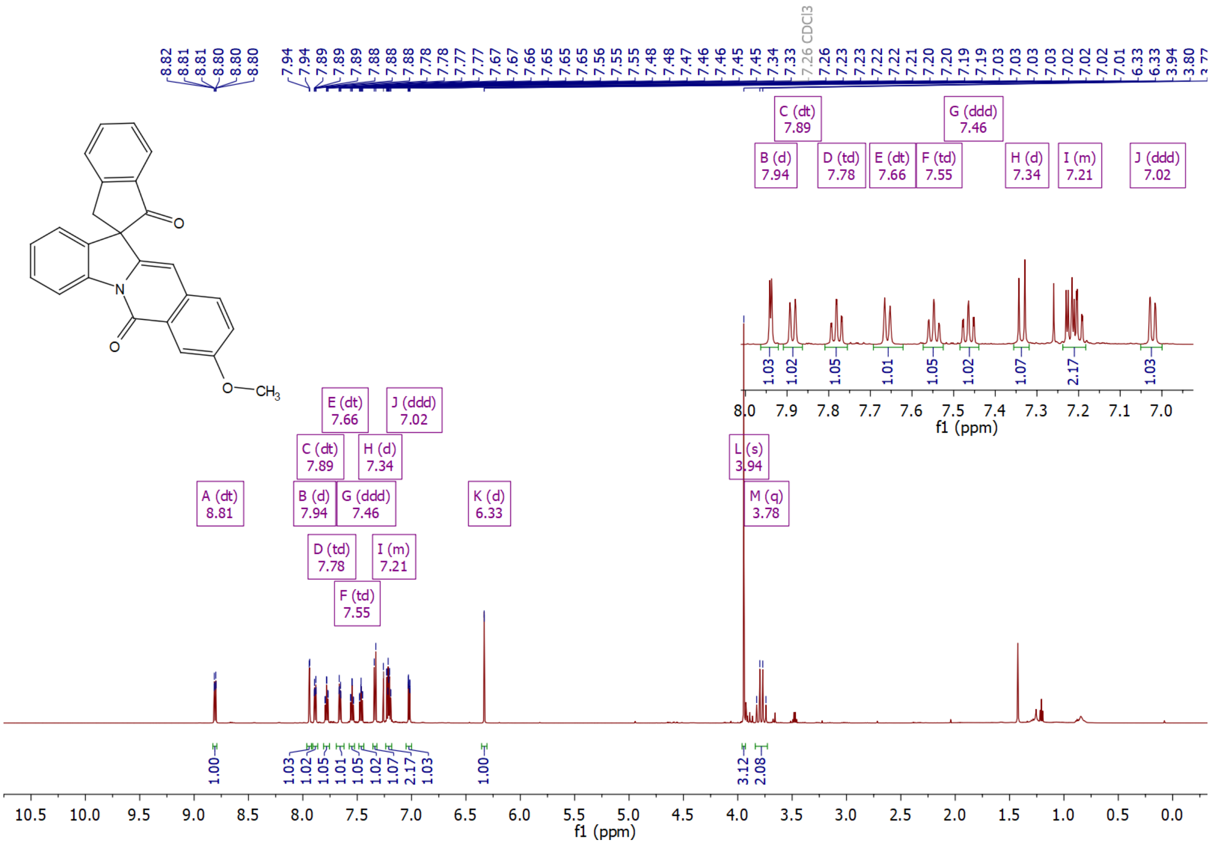

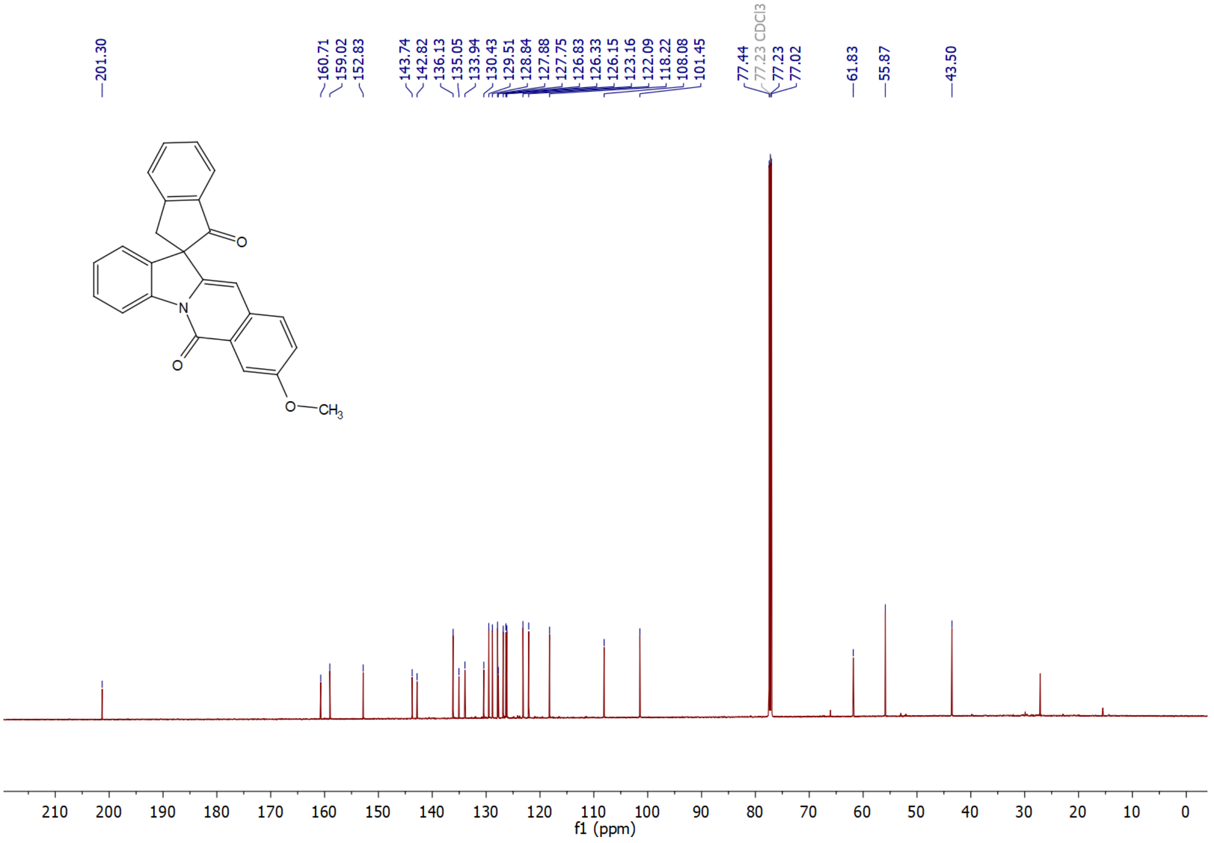

9'-chloro-6'H-spiro[indene-2,12'-indolo[1,2-b]isoquinoline]-1,6'(3H)-dione **[E4]**:

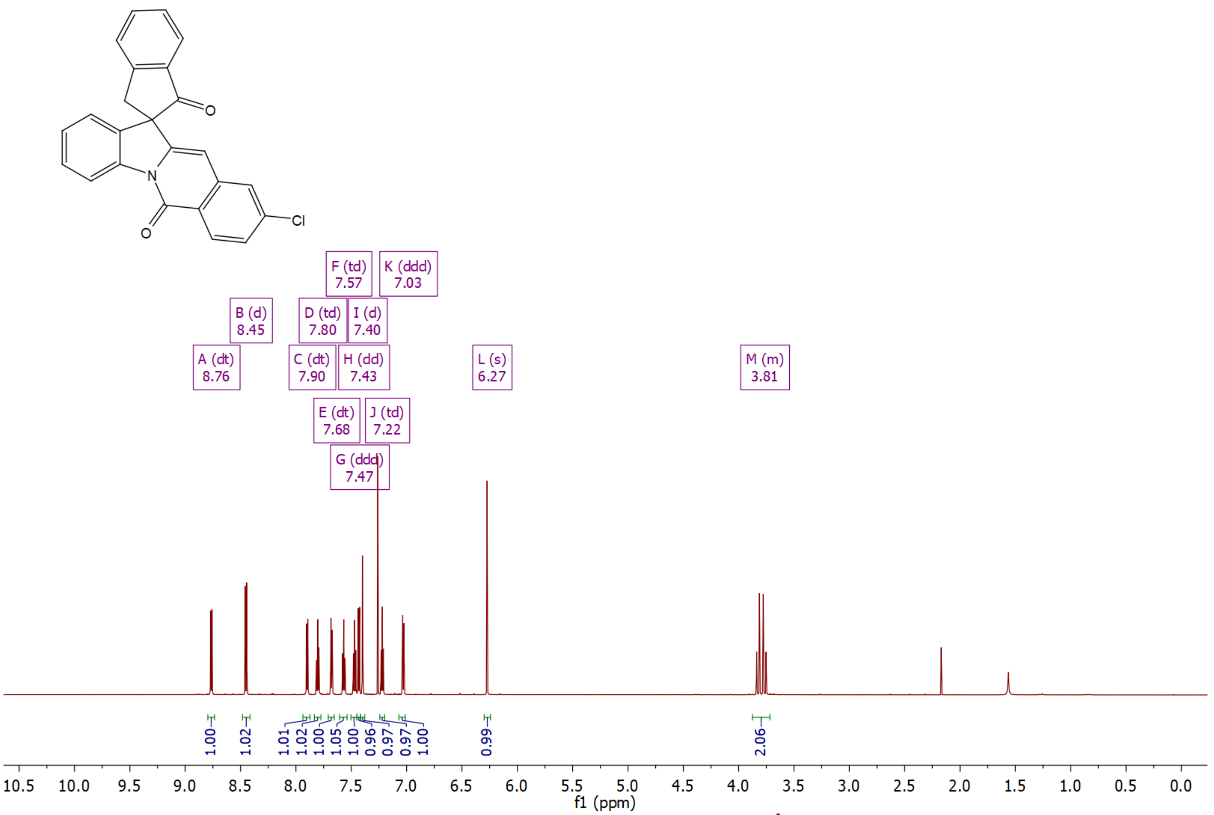

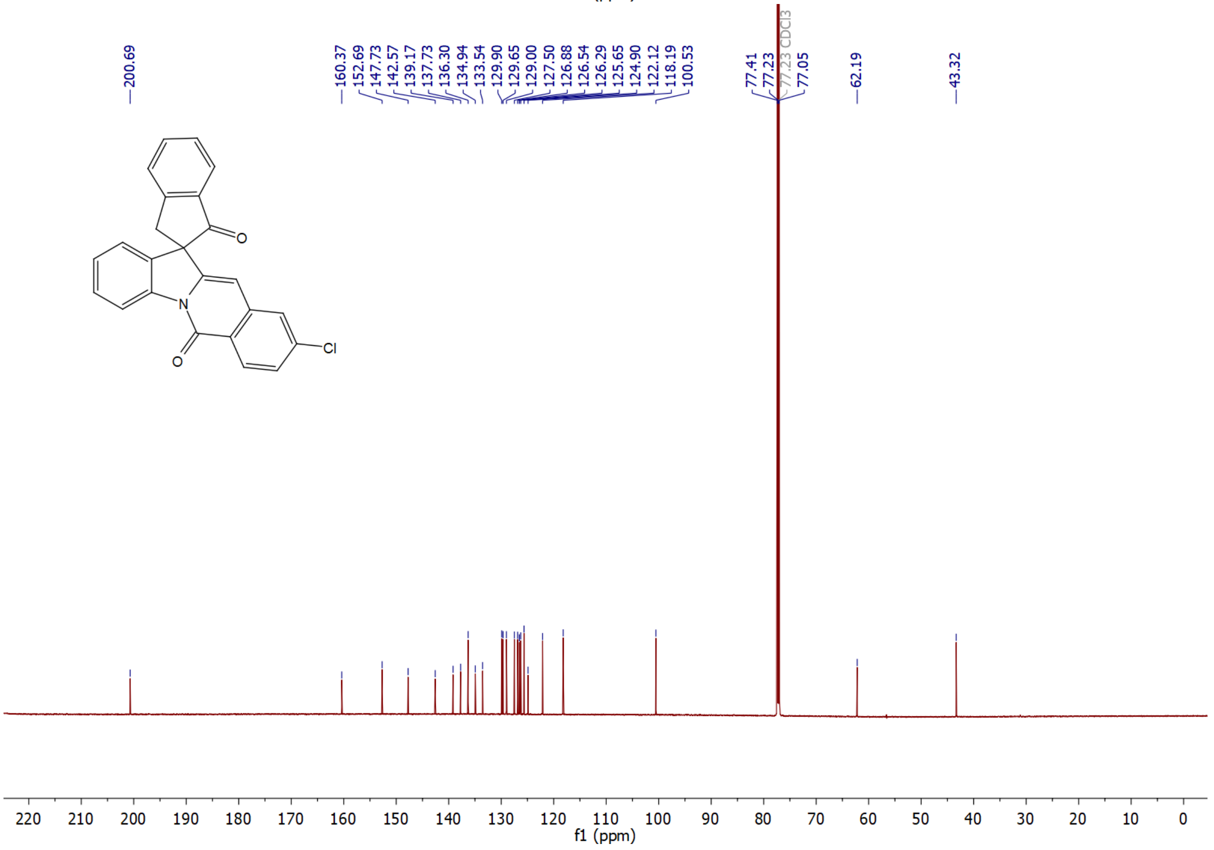

9'-chloro-6'H-spiro[indene-2,12'-indolo[1,2-b]isoquinoline]-1,6'(3H)-dione [**E4-resynthesized**]:

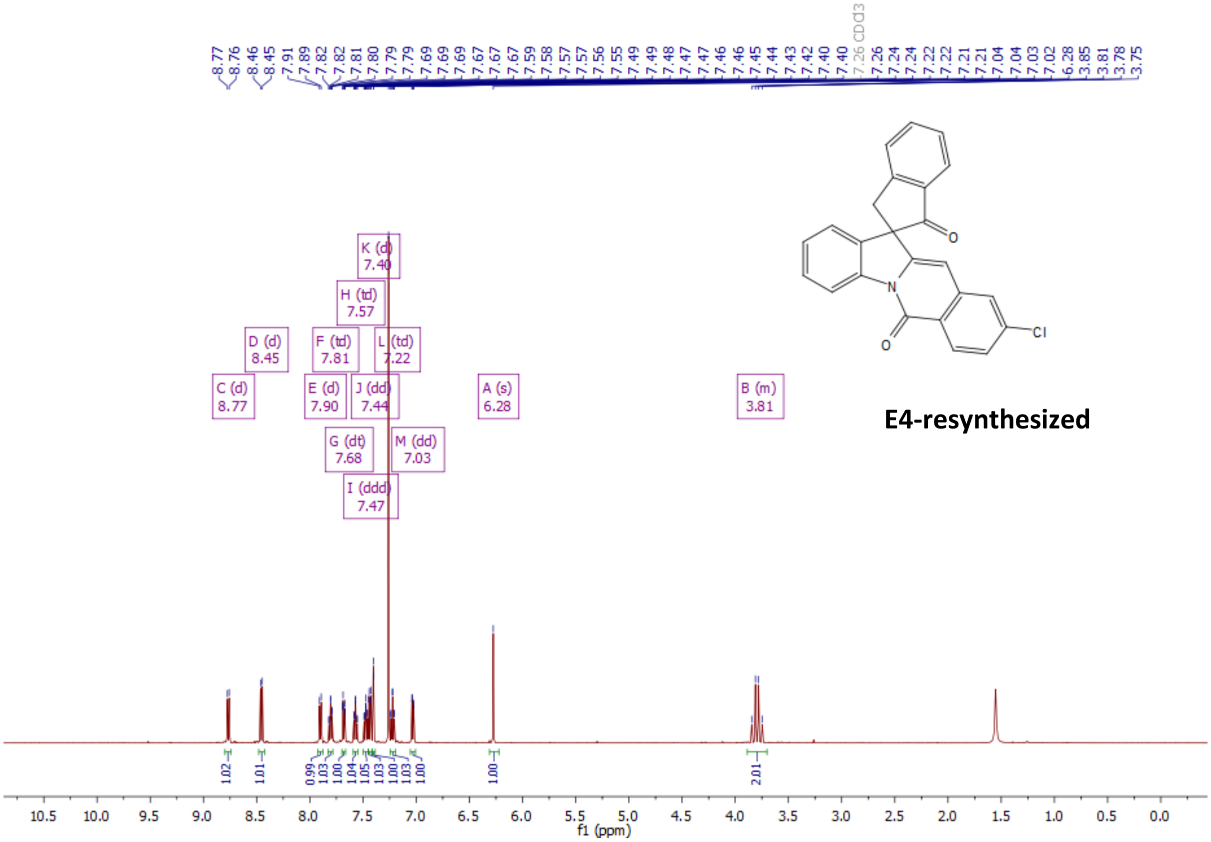

8'-nitro-6'H-spiro[indene-2,12'-indolo[1,2-b]isoquinoline]-1,6'(3H)-dione [E5]:

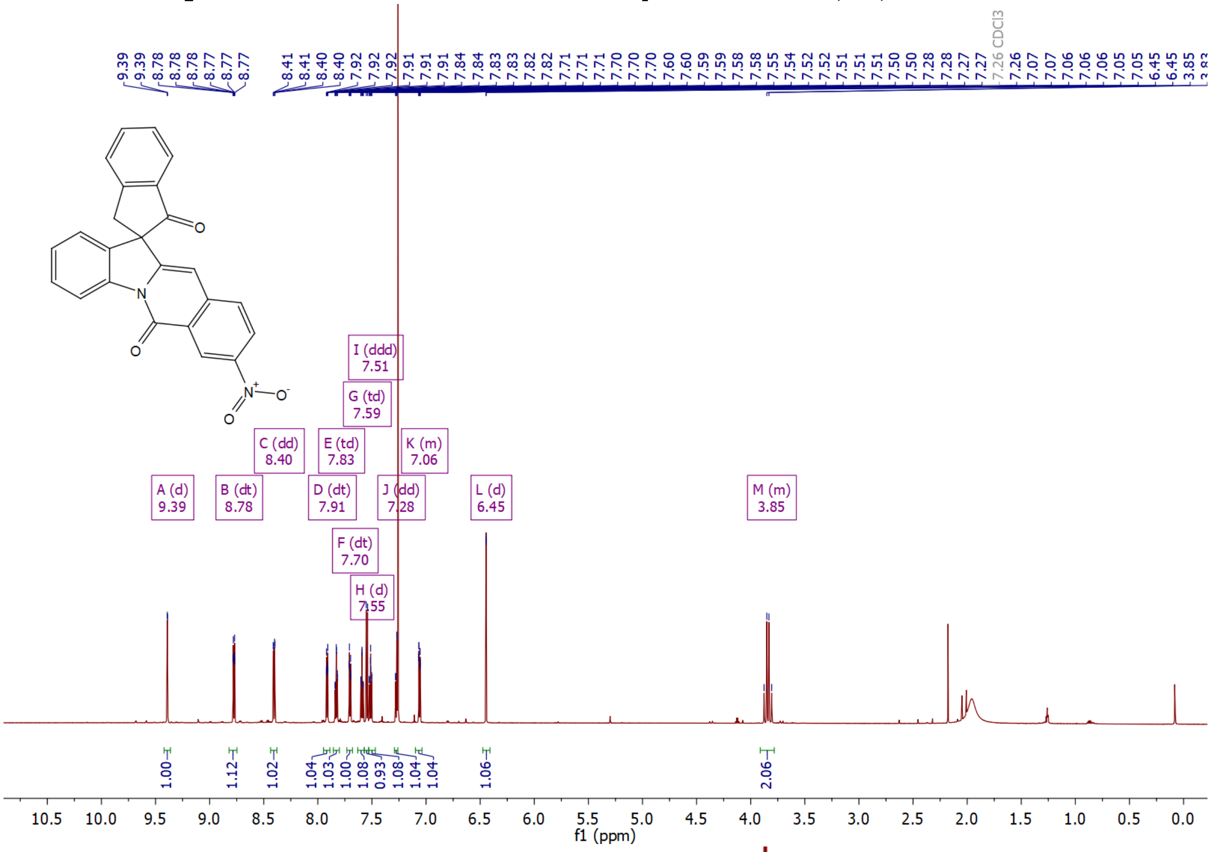

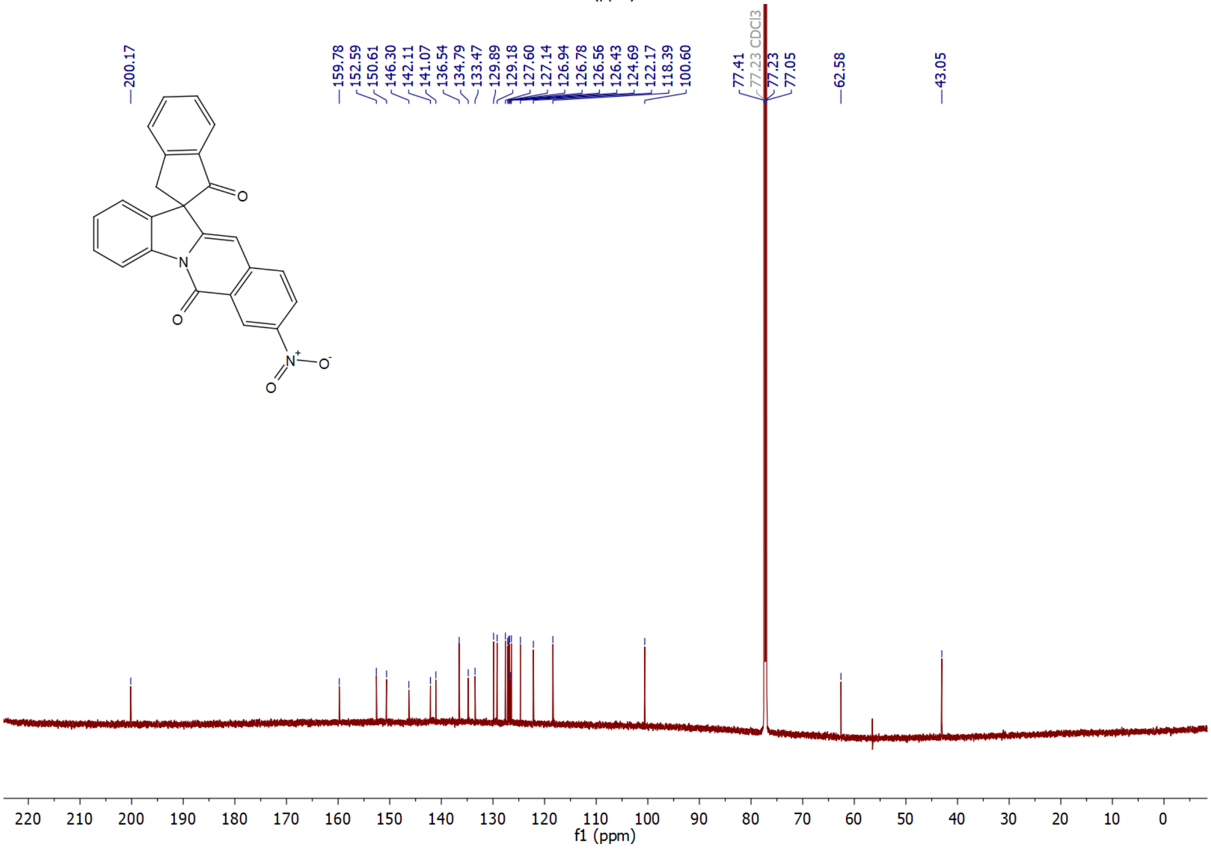

8',9'-difluoro-6'H-spiro[indene-2,12'-indolo[1,2-b]isoquinoline]-1,6'(3H)-dione [E6]:

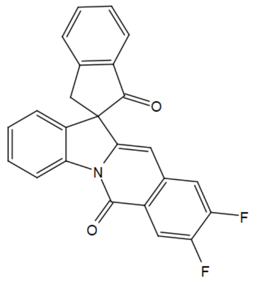

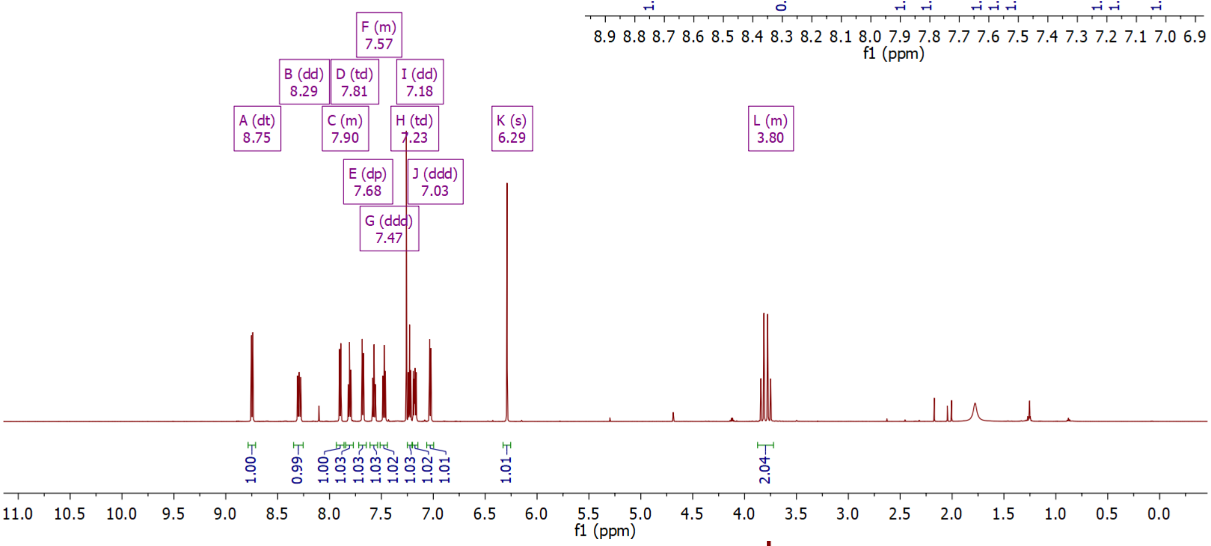

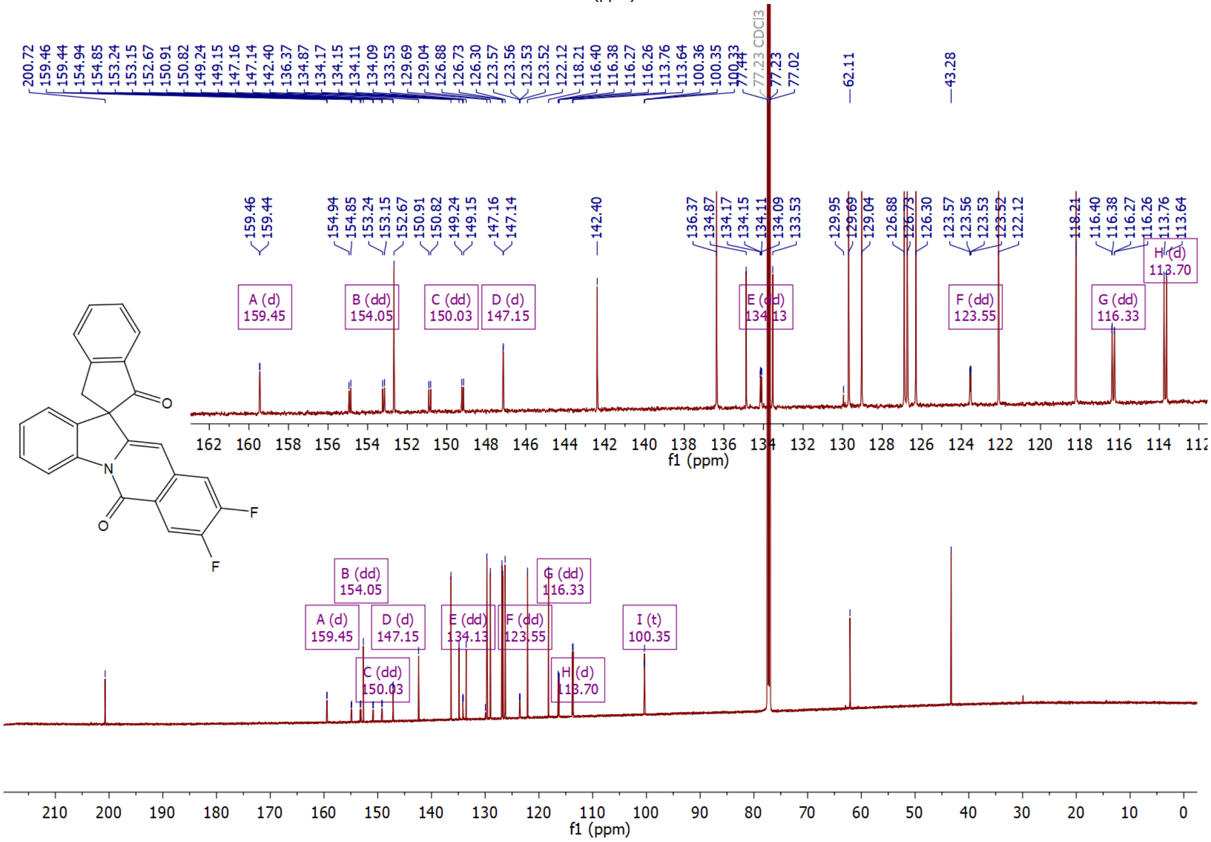

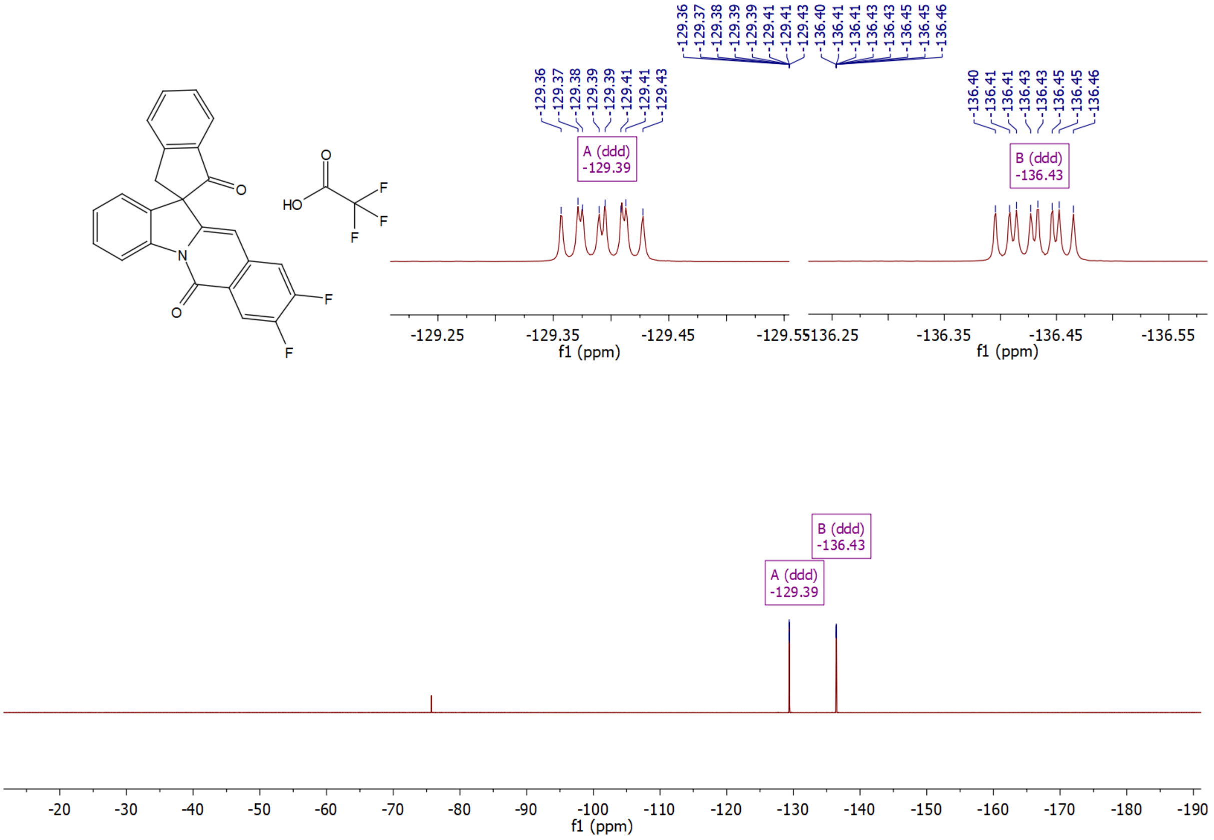
A (ddd)
-129.39
B (ddd)
-136.43
-129.25
-129.35
-129.45
-129.55
136.25
-136.35
-136.45
-136.55
f1 (ppm)
-20
-30
-40
-50
-60
-70
-80
-90
-100
-110
-120
-130
-140
-150
-160
-170
-180
-190
HO
O
F
N

8',9'-dimethoxy-6'H-spiro[indene-2,12'-indolo[1,2-b]isoquinoline]-1,6'(3H)-dione [E7]:

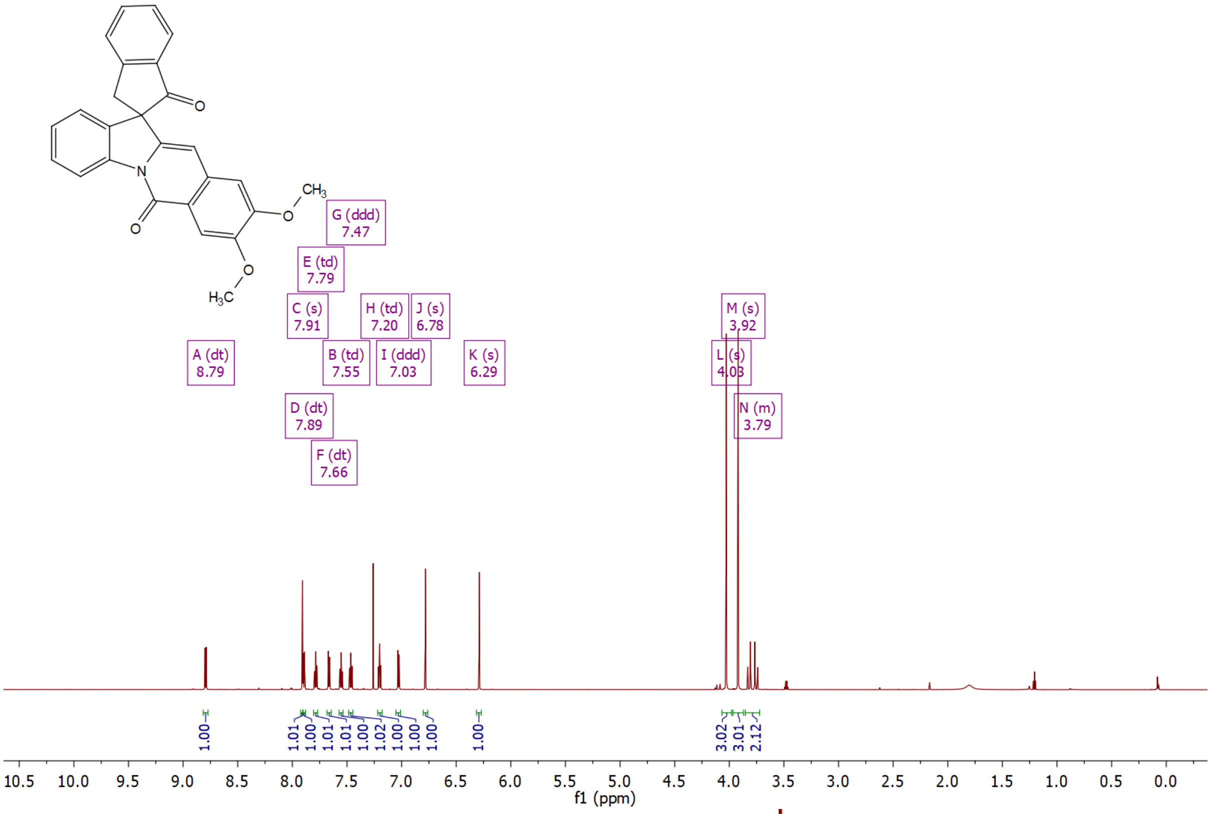

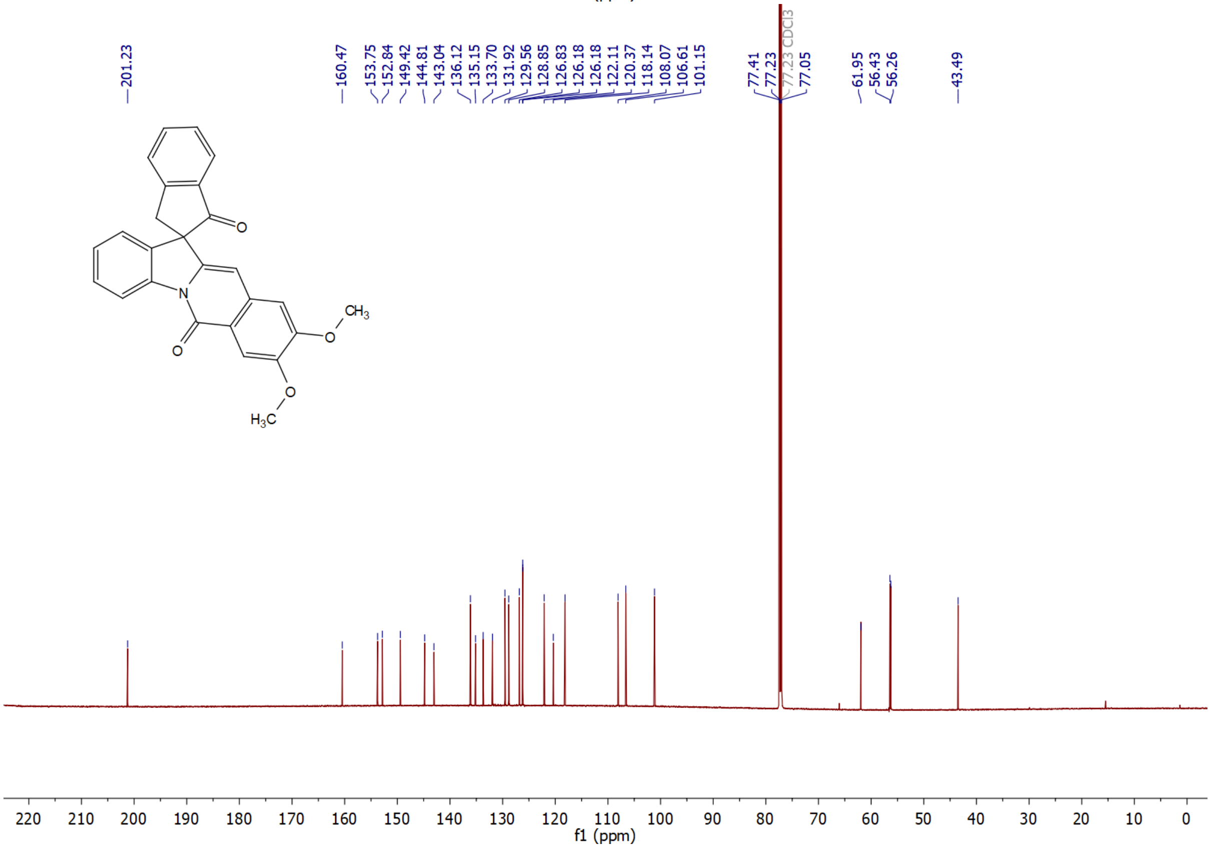

11'H-spiro[indene-2,5'-thieno[3',2':4,5]pyrido[1,2-a]indole]-1,11'(3H)-dione [**E8**]:

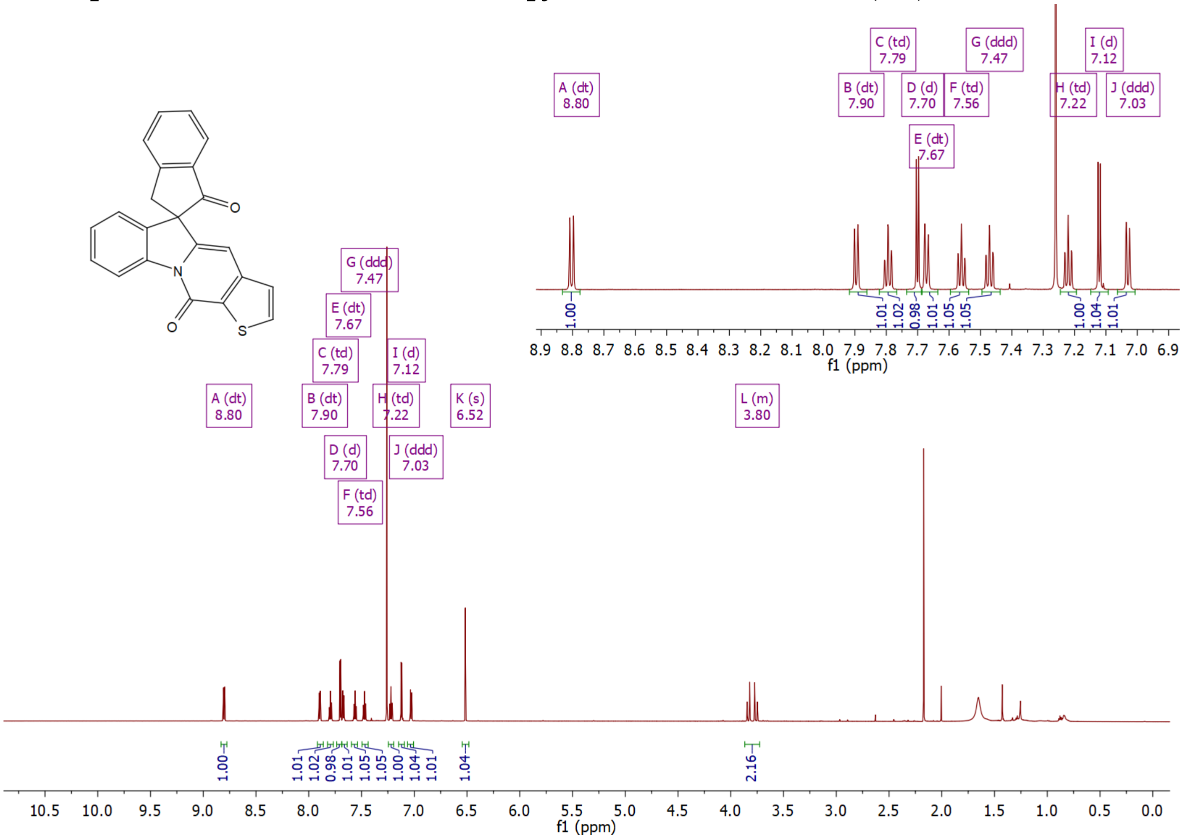

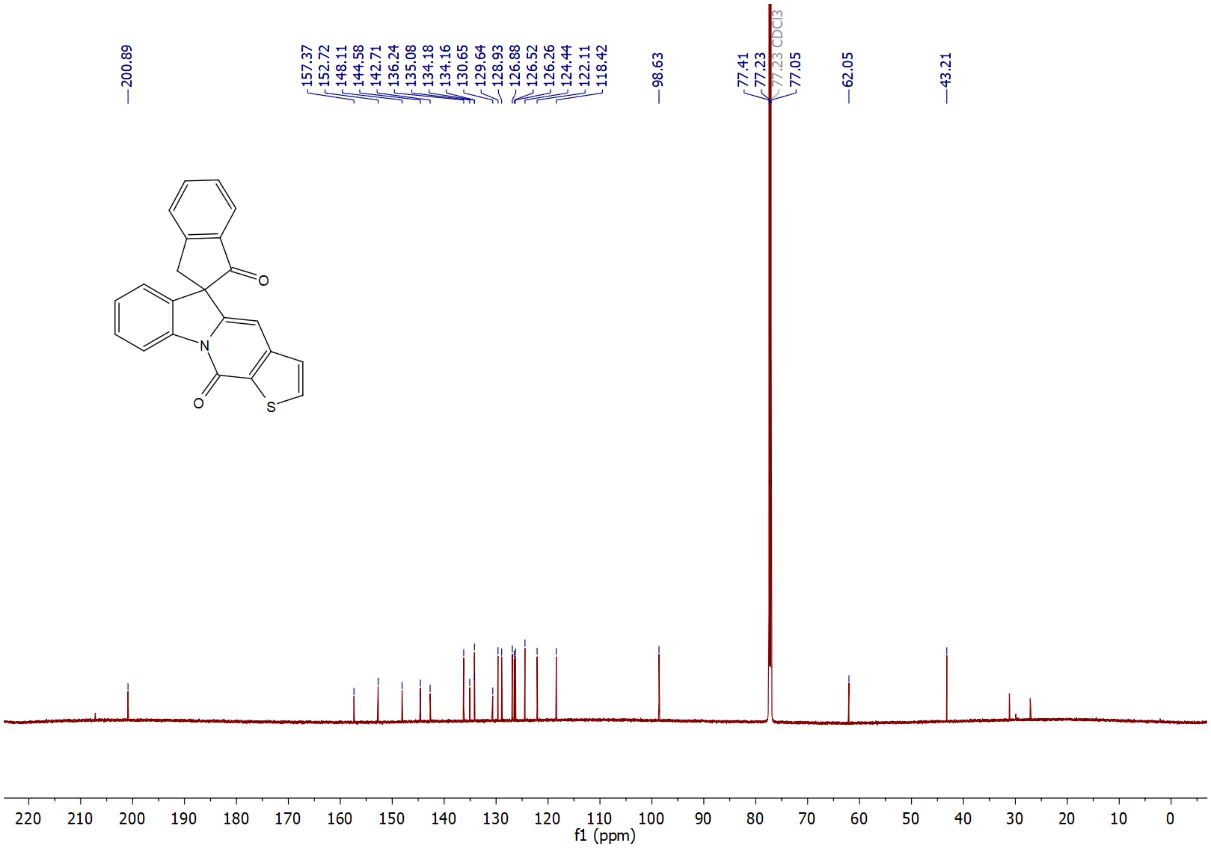

5-fluoro-6'H-spiro[indene-2,12'-indolo[1,2-b]isoquinoline]-1,6'(3H)-dione **[E9]**:

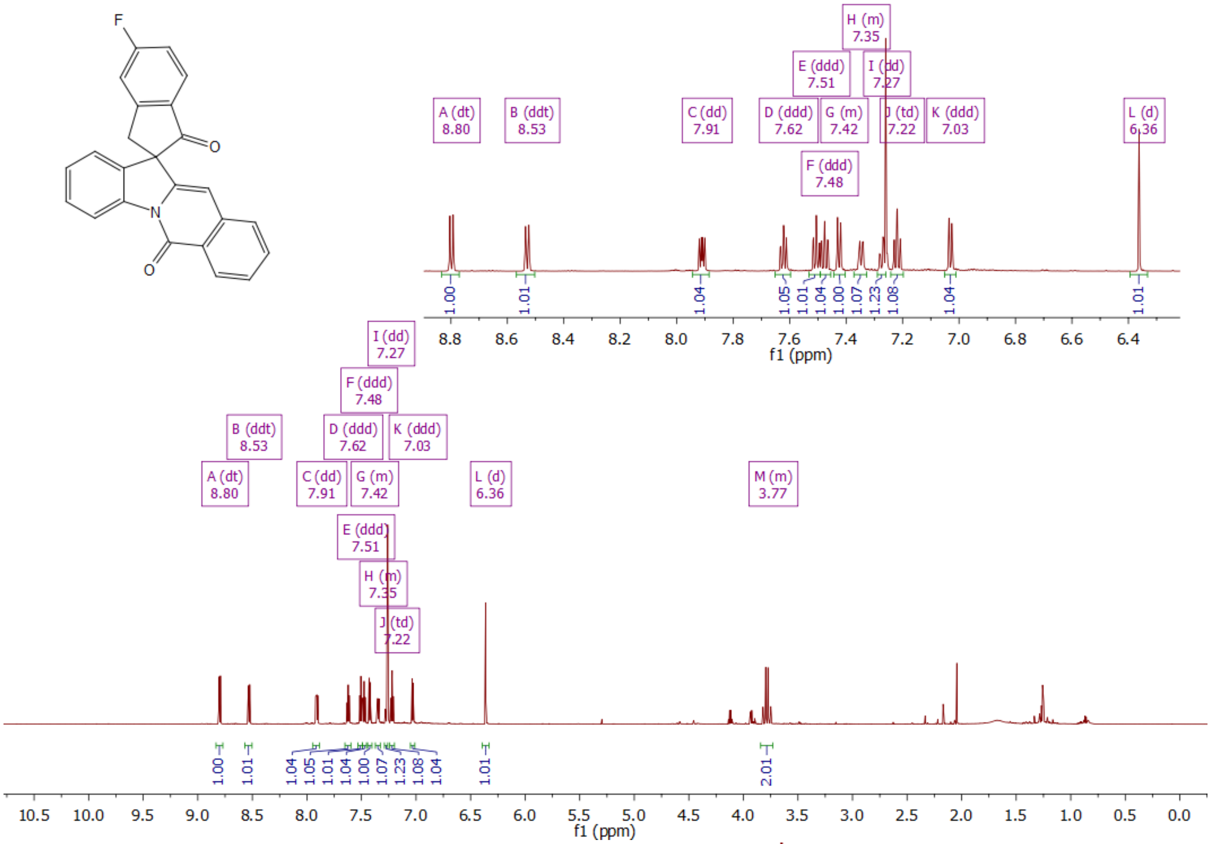

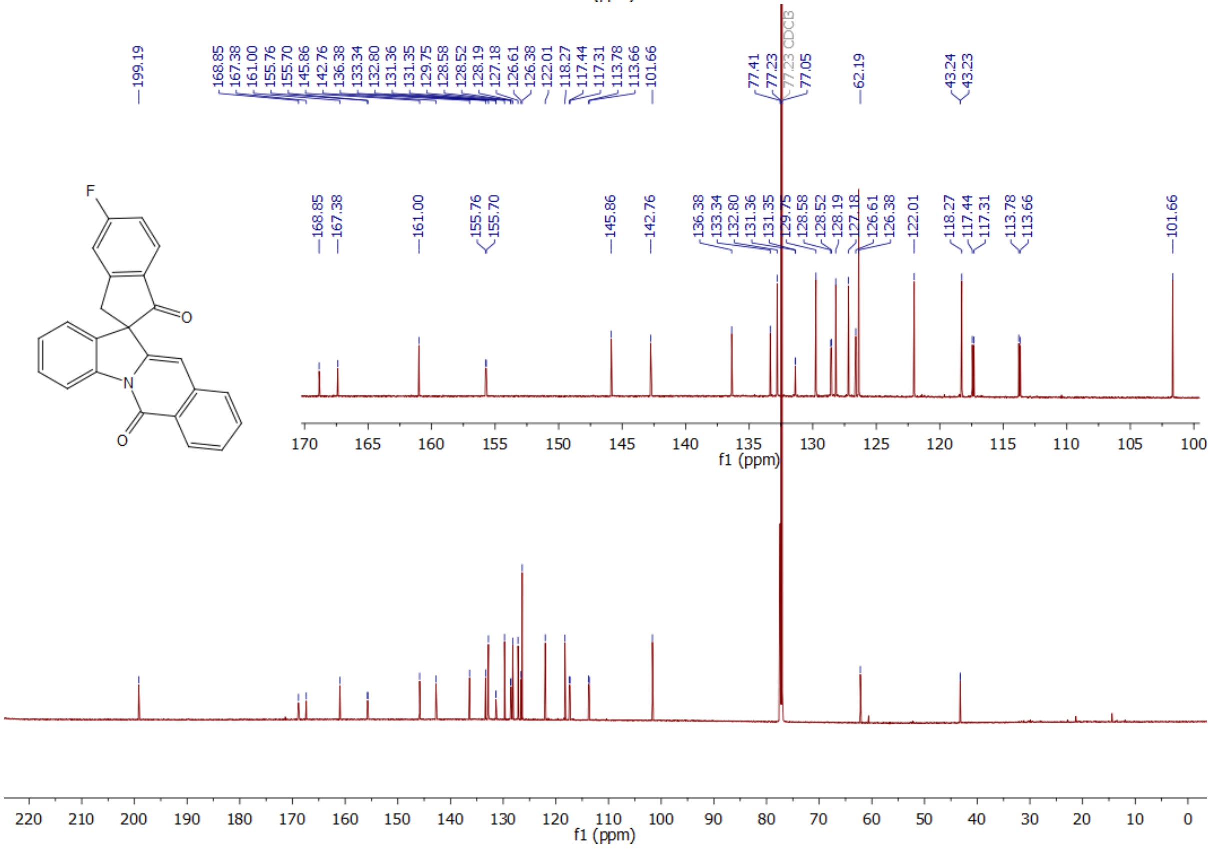

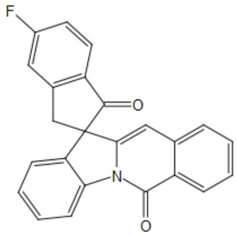
F
O
N
O

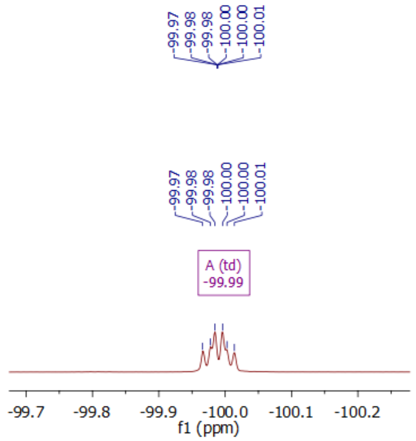
-99.97
-99.98
-99.98
-100.00
-100.00
-100.01
-99.97
-99.98
-99.98
-100.00
-100.00
-100.01
A (td)
-99.99
-99.7 -99.8 -99.9 -100.0 -100.1 -100.2
f1 (ppm)

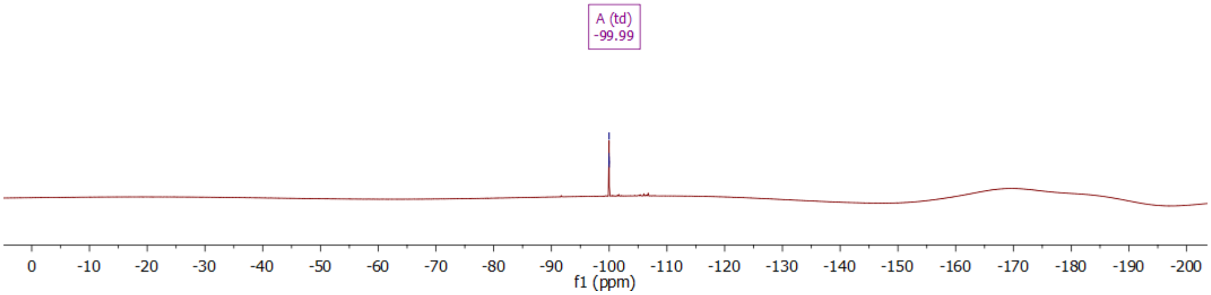
A (td)
-99.99
0 -10 -20 -30 -40 -50 -60 -70 -80 -90 -100 -110 -120 -130 -140 -150 -160 -170 -180 -190 -200
f1 (ppm)

6-chloro-2'-fluoro-6'H-spiro[indene-2,12'-indolo[1,2-b]isoquinoline]-1,6'(3H)-dione [**E10**]:

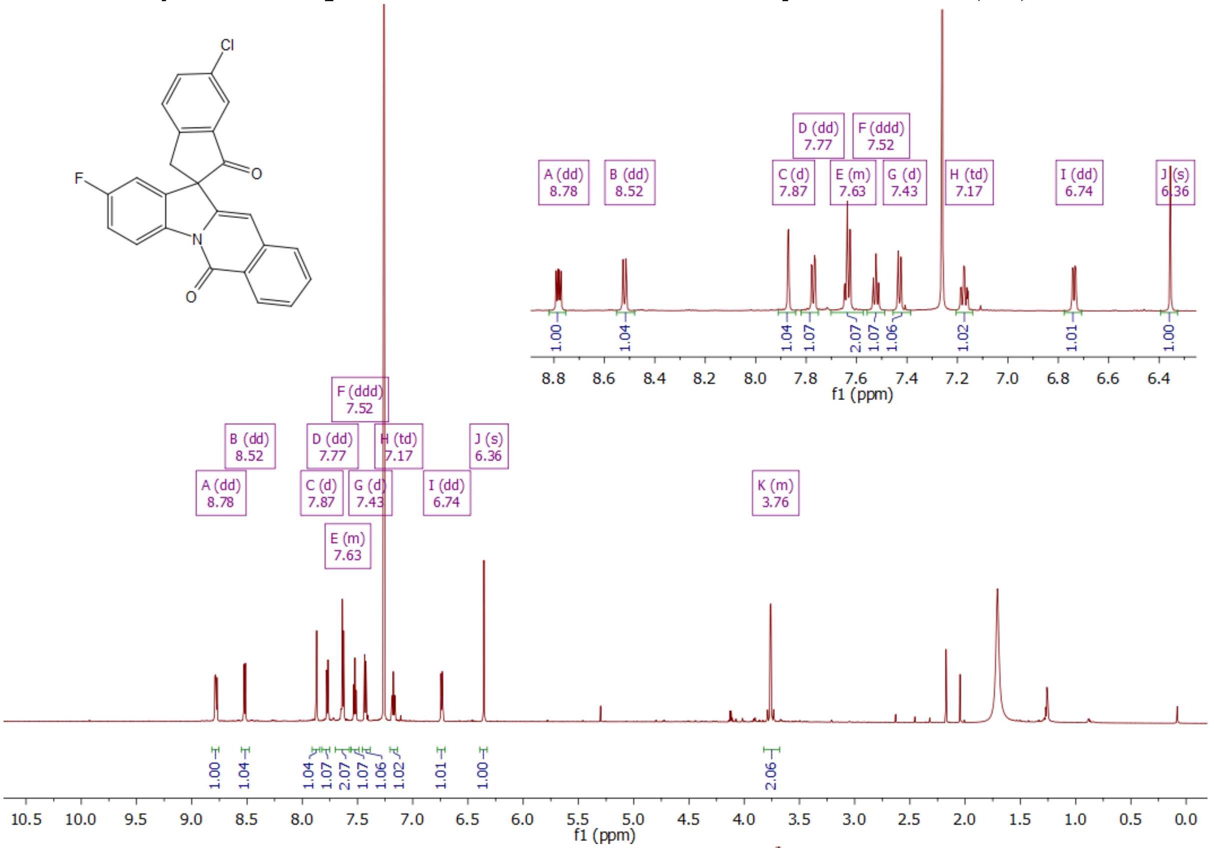

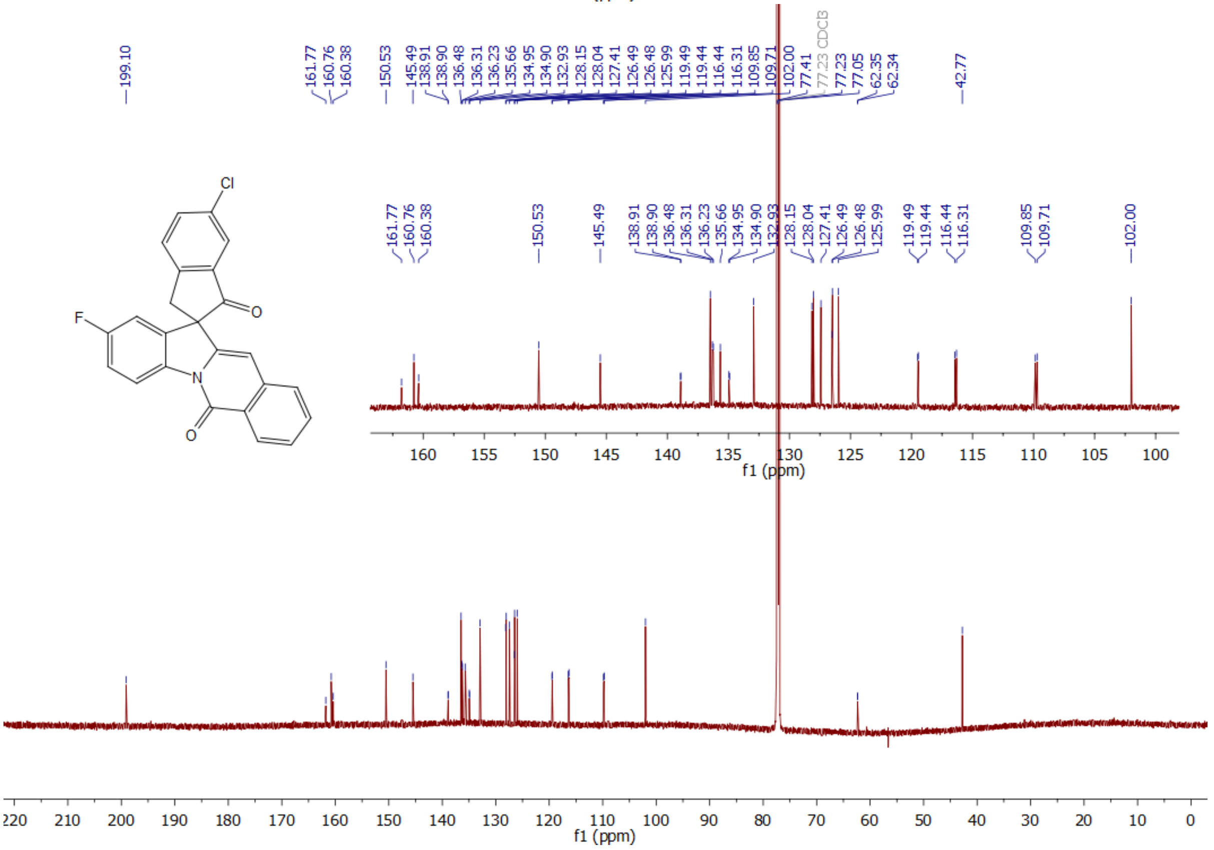

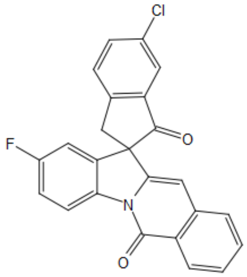
Cl
F
N
O
O

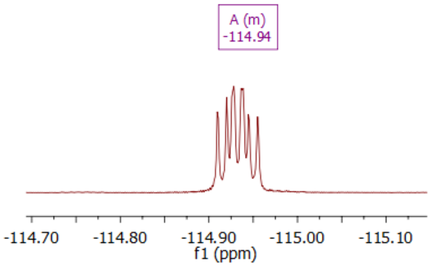
A (m)
-114.94
-114.70
-114.80
-114.90
-115.00
-115.10
f1 (ppm)

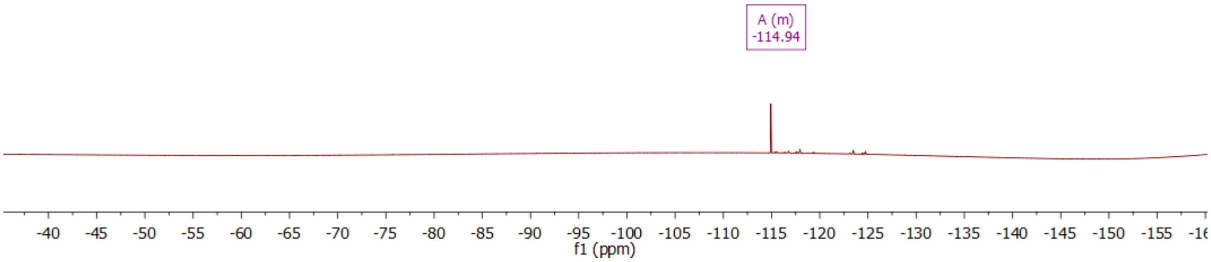
A (m)
-114.94
-40 -45 -50 -55 -60 -65 -70 -75 -80 -85 -90 -95 -100 -105 -110 -115 -120 -125 -130 -135 -140 -145 -150 -155
f1 (ppm)

6-chloro-2'-fluoro-8'-methyl-6'H-spiro[indene-2,12'-indolo[1,2-b]isoquinoline]-1,6'(3H)-dione [**E11**]:

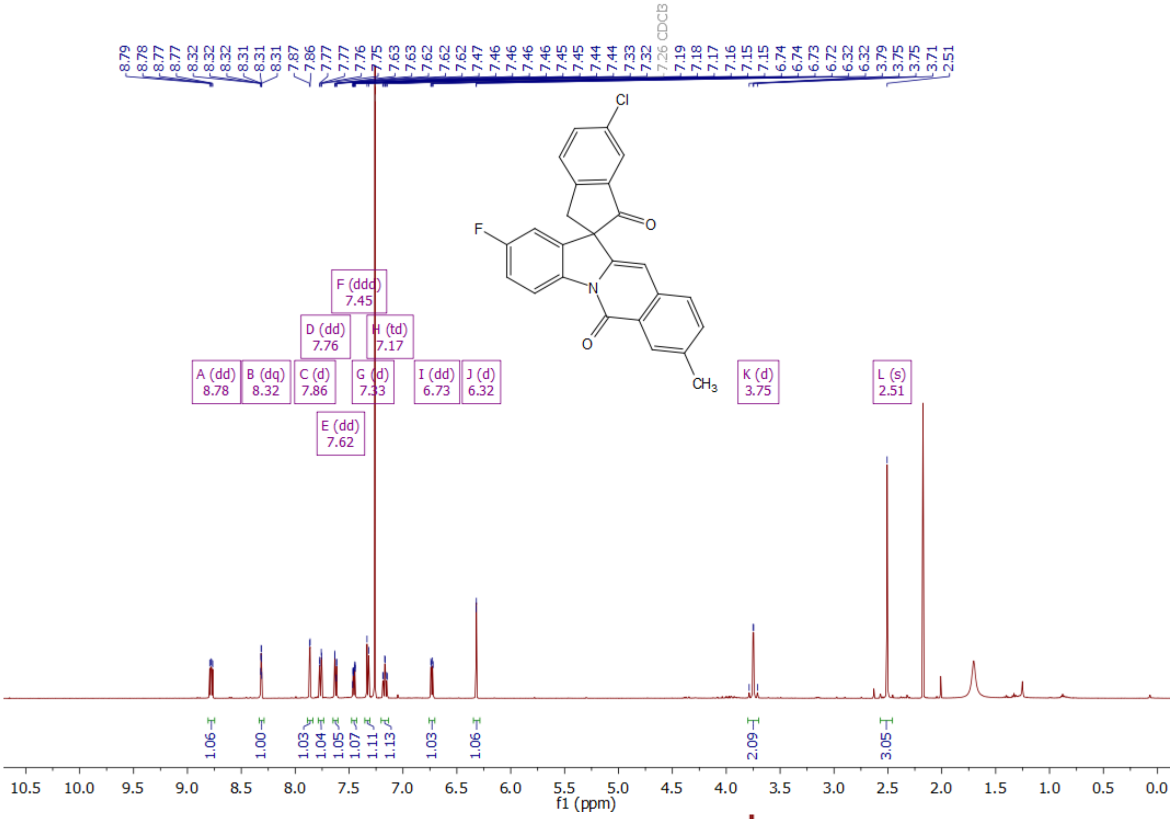

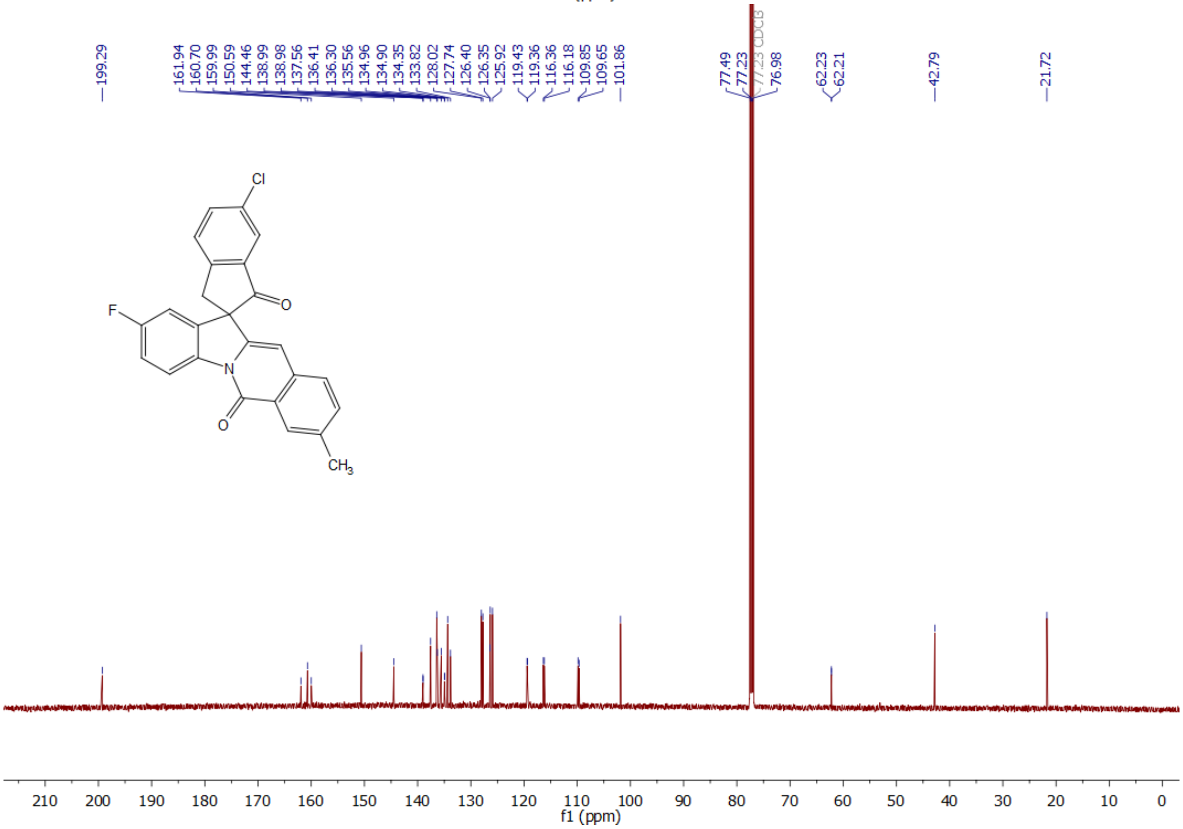

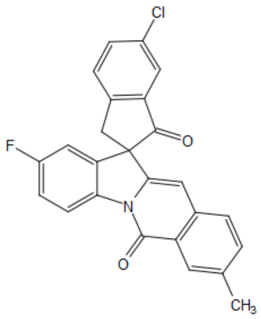
Cl
F
O
N
O
CH3

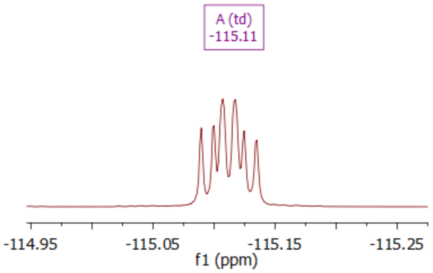
A (td)
-115.11
-114.95
-115.05
-115.15
-115.25
f1 (ppm)

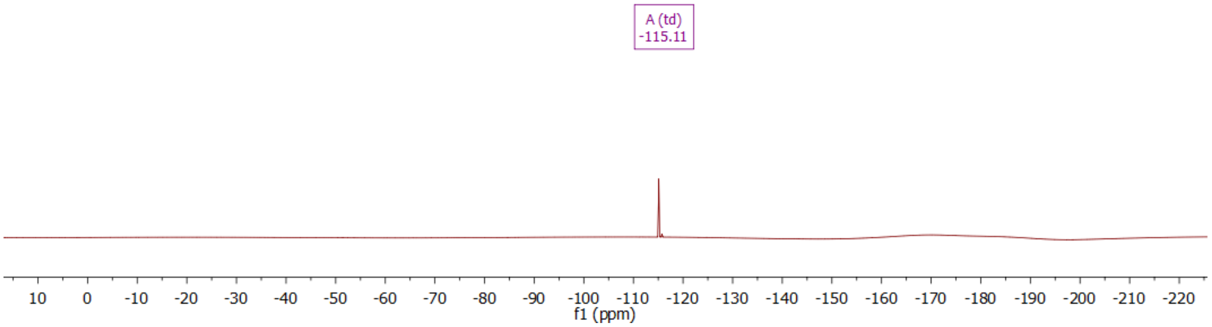
A (td)
-115.11
10
0
-10
-20
-30
-40
-50
-60
-70
-80
-90
-100
-110
-120
-130
-140
-150
-160
-170
-180
-190
-200
-210
-220
f1 (ppm)

2'-chloro-5-fluoro-8'-methyl-6'H-spiro[indene-2,12'-indolo[1,2-b]isoquinoline]-1,6'(3H)-dione **[E12]**:

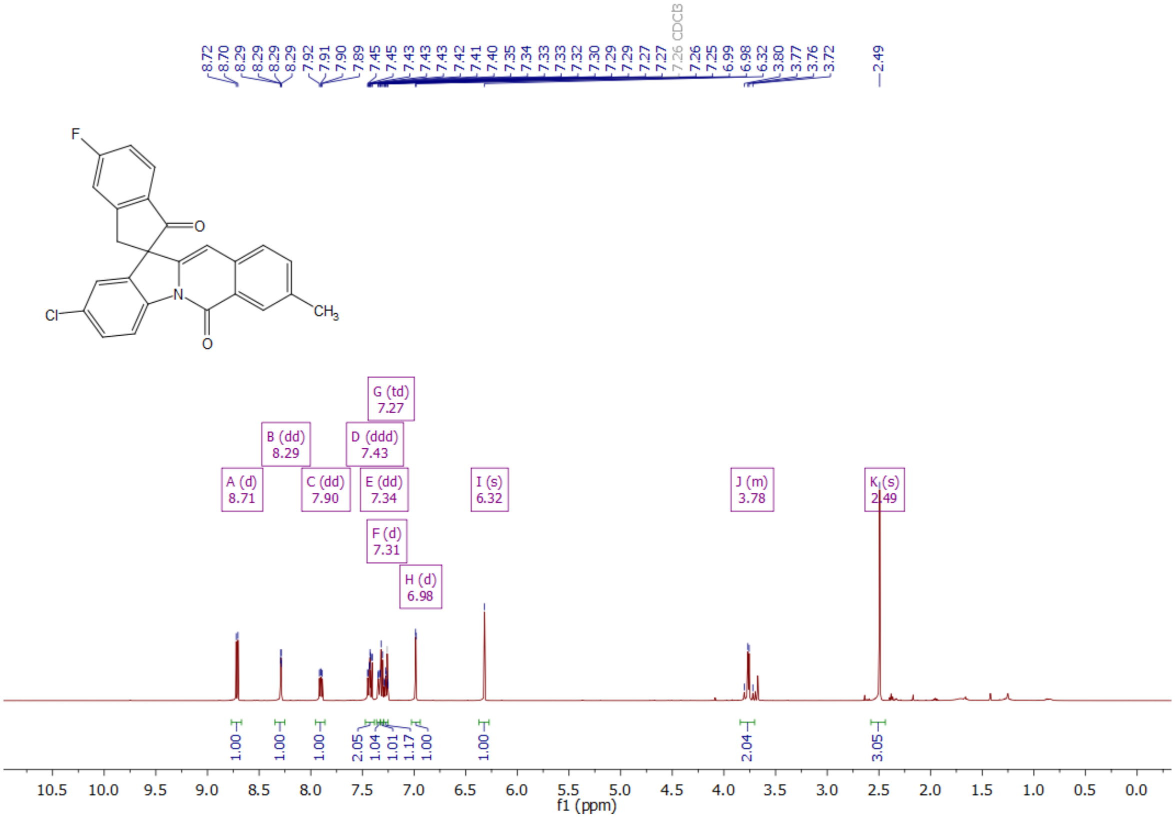

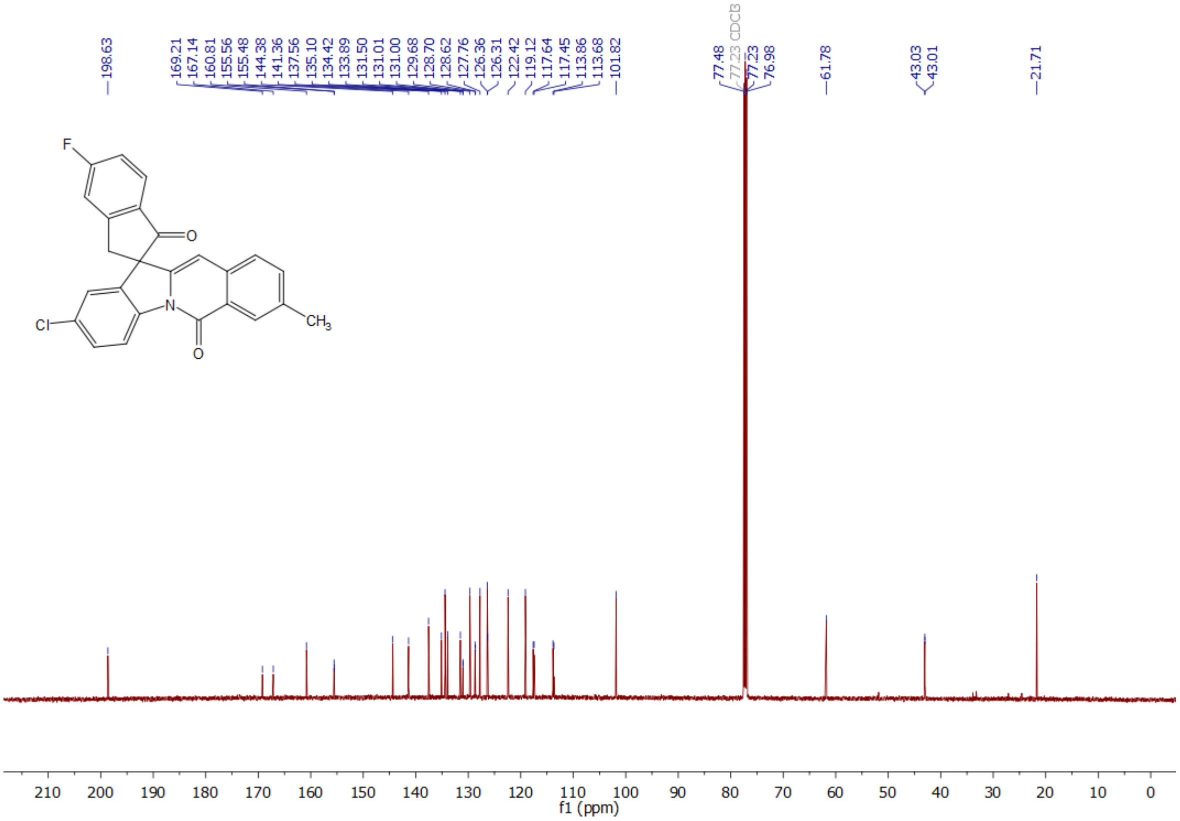

2'-fluoro-6-methoxy-8'-methyl-6'H-spiro[indene-2,12'-indolo[1,2-b]isoquinoline]-1,6'(3H)-dione [**E13**]:

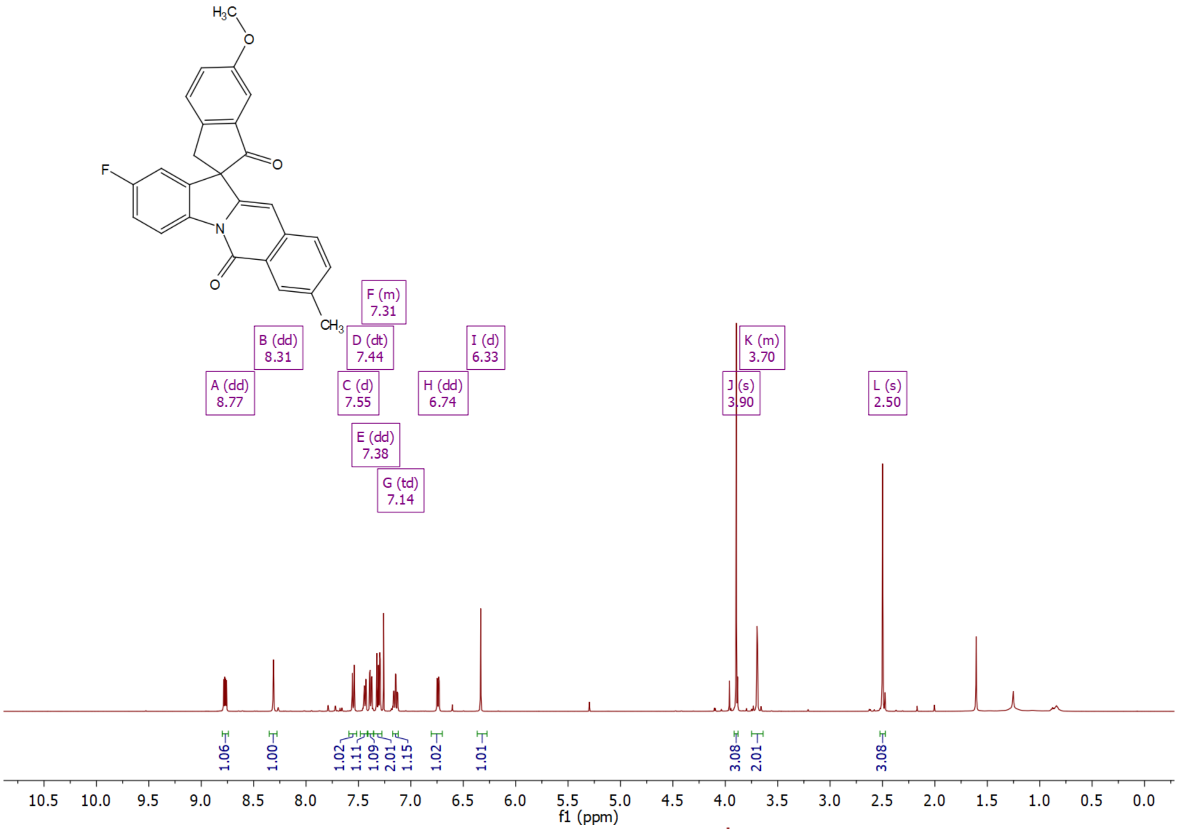

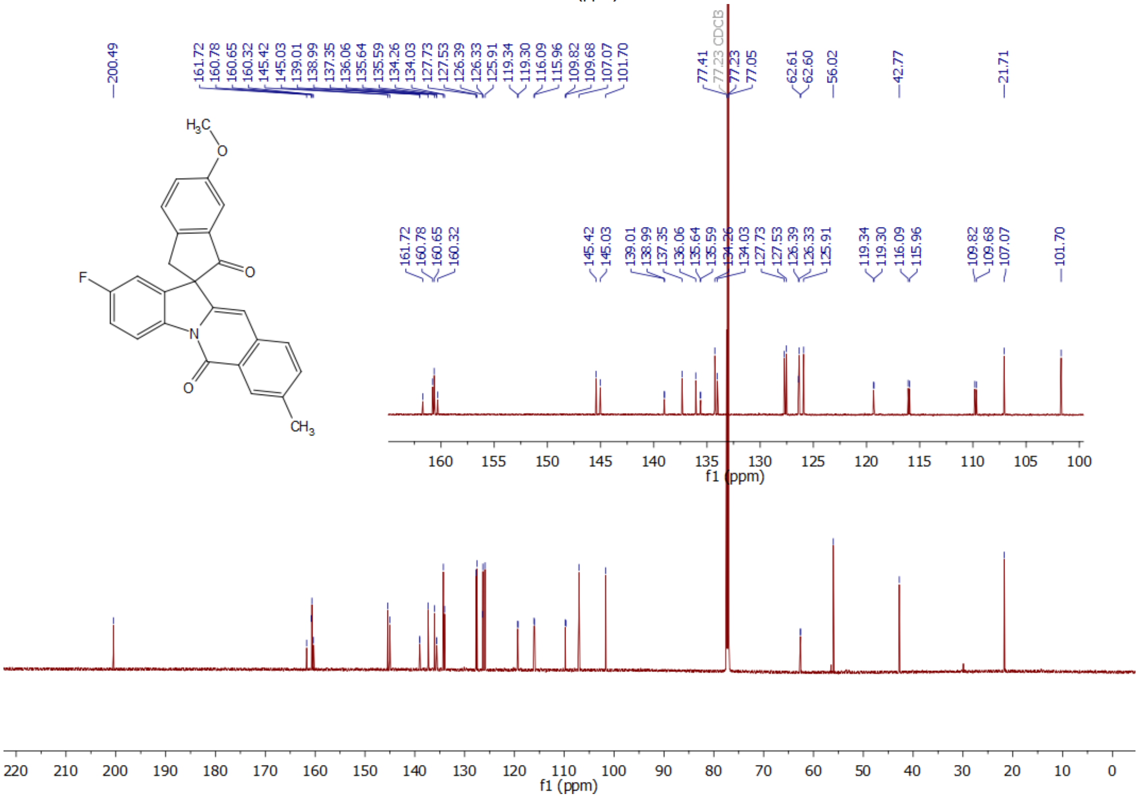

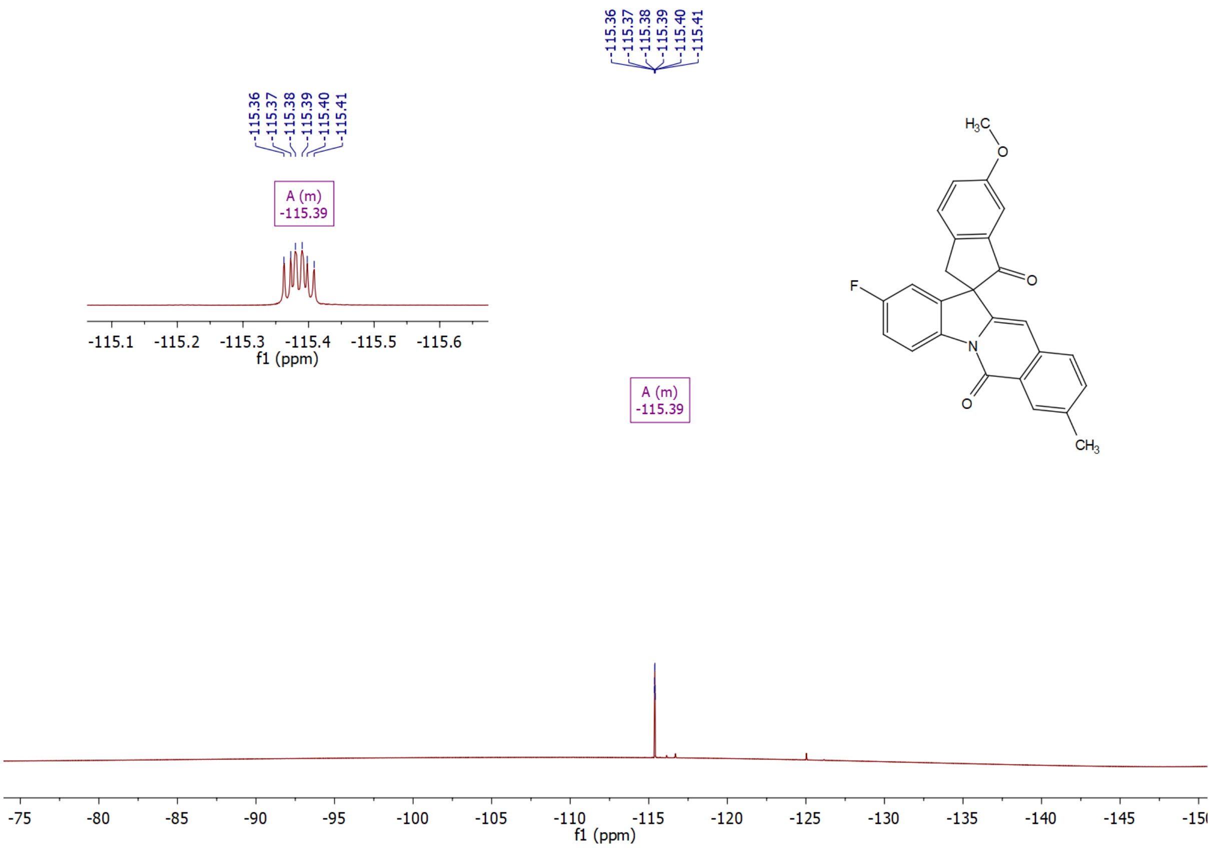

-115.36
-115.37
-115.38
-115.39
-115.40
-115.41
A (m)
-115.39
-115.1 -115.2 -115.3 -115.4 -115.5 -115.6
f1 (ppm)
H3C
O
O
F
N
O
CH3
-75 -80 -85 -90 -95 -100 -105 -110 -115 -120 -125 -130 -135 -140 -145
f1 (ppm)

2'-fluoro-6-methoxy-8'-methyl-6'H-spiro[indene-2,12'-indolo[1,2-b]isoquinoline]-1,6'(3H)-dione **[E13-resynthesized]:**

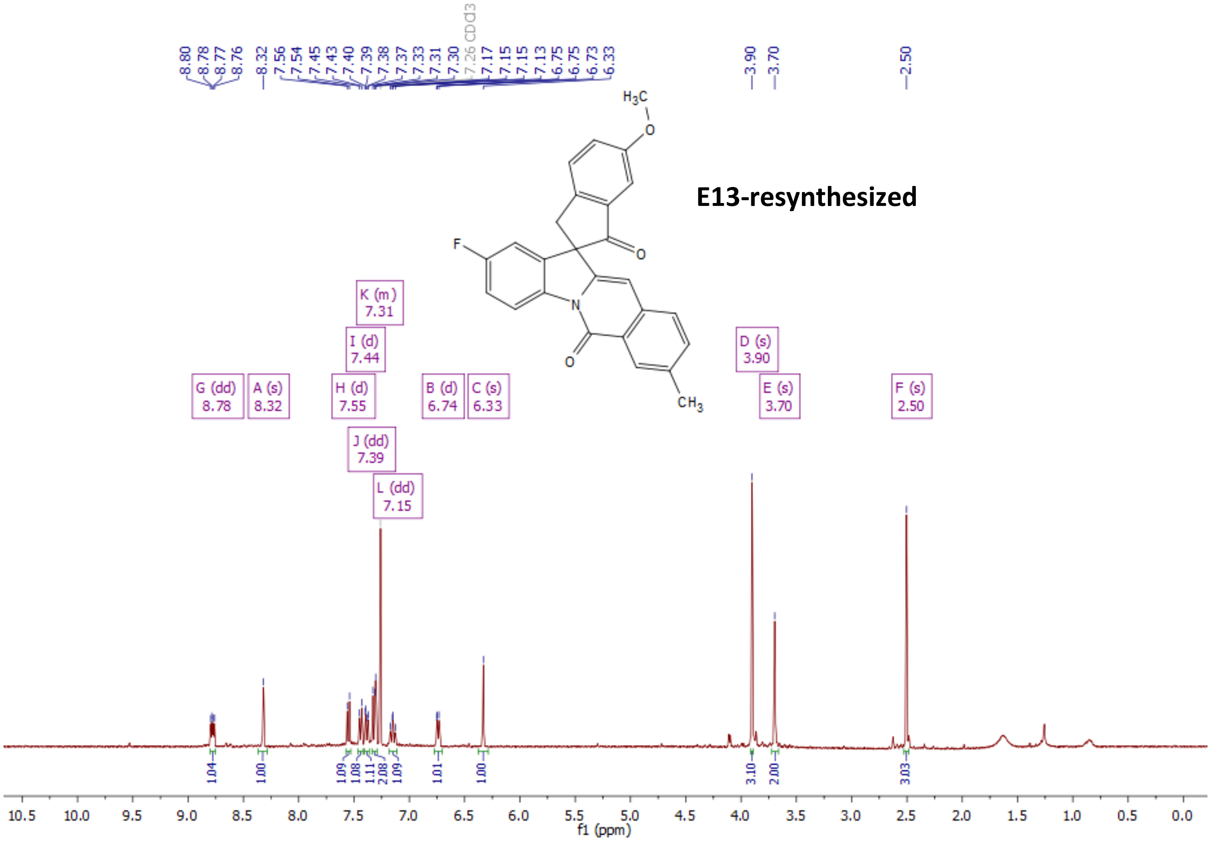

5,6-dimethoxy-6'H-spiro[indene-2,12'-indolo[1,2-b]isoquinoline]-1,6'(3H)-dione [**E14**]:

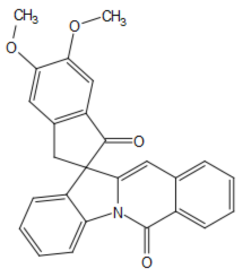

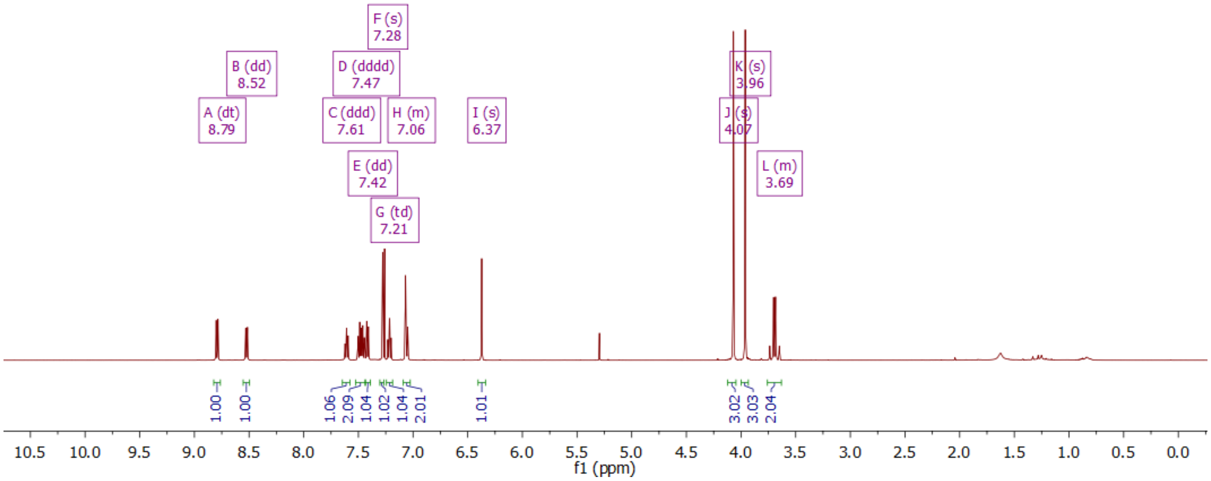

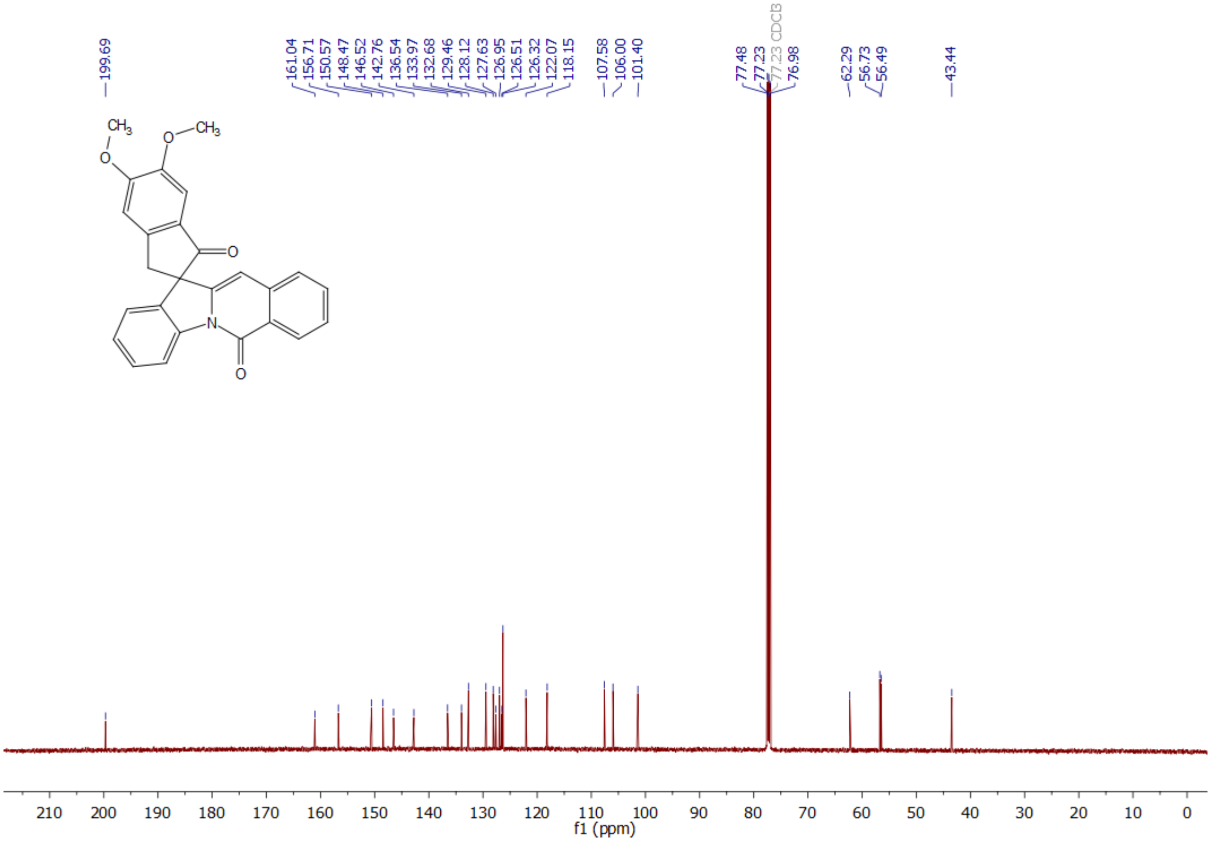

***5,6-dimethoxy-8'-methyl-6'H-spiro[indene-2,12'-indolo[1,2-b]isoquinoline]-1,6'(3H)-dione* [E15]:**

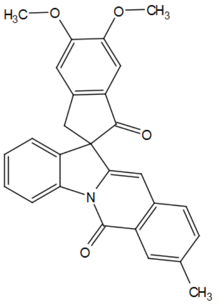

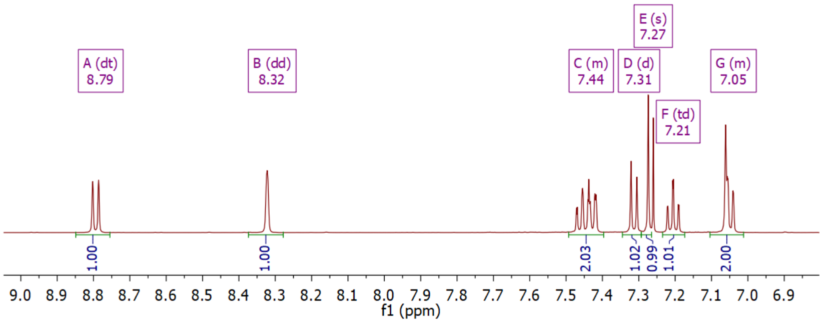

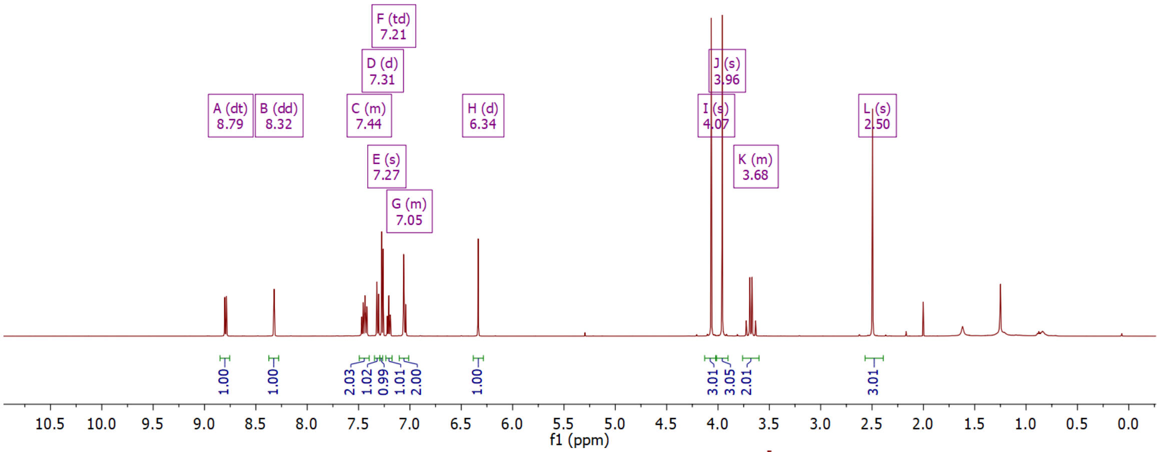

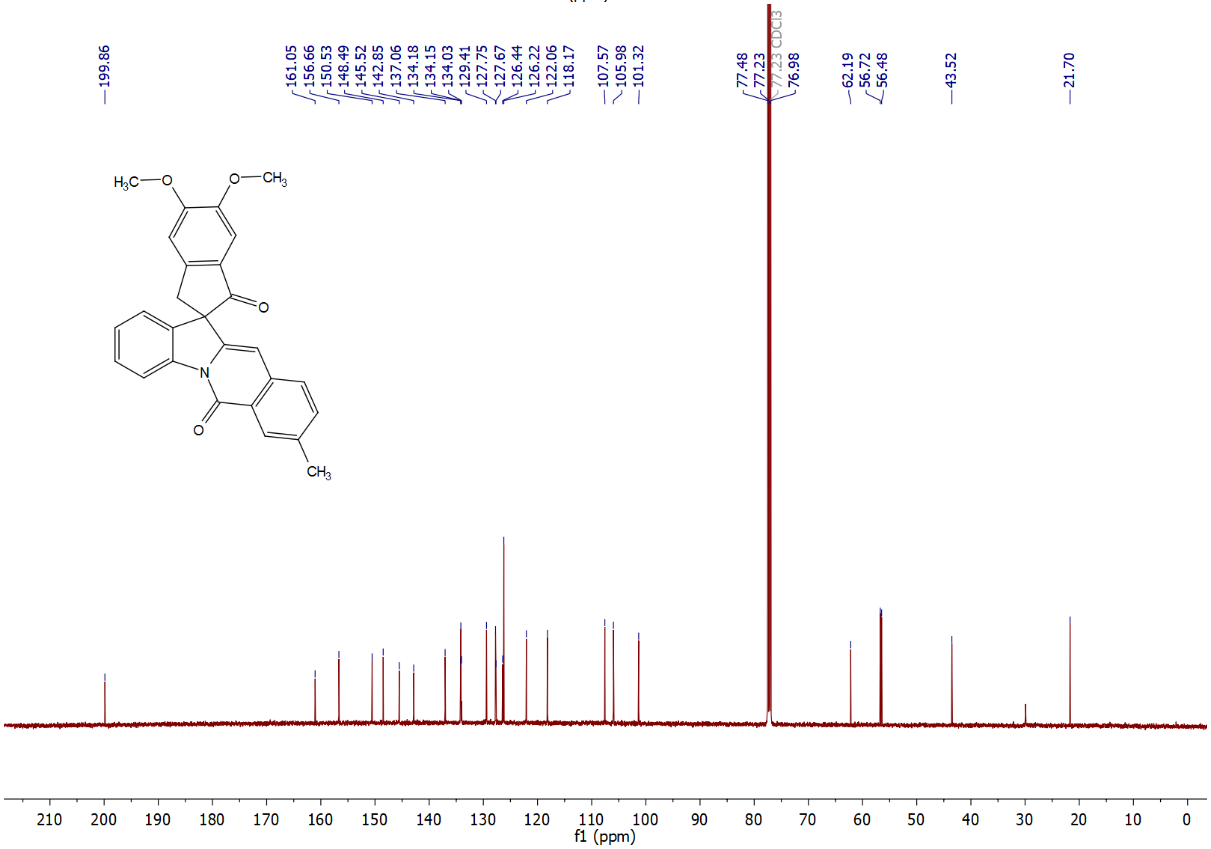

5,6,8'-trimethoxy-6'H-spiro[indene-2,12'-indolo[1,2-b]isoquinoline]-1,6'(3H)-dione [E16]:

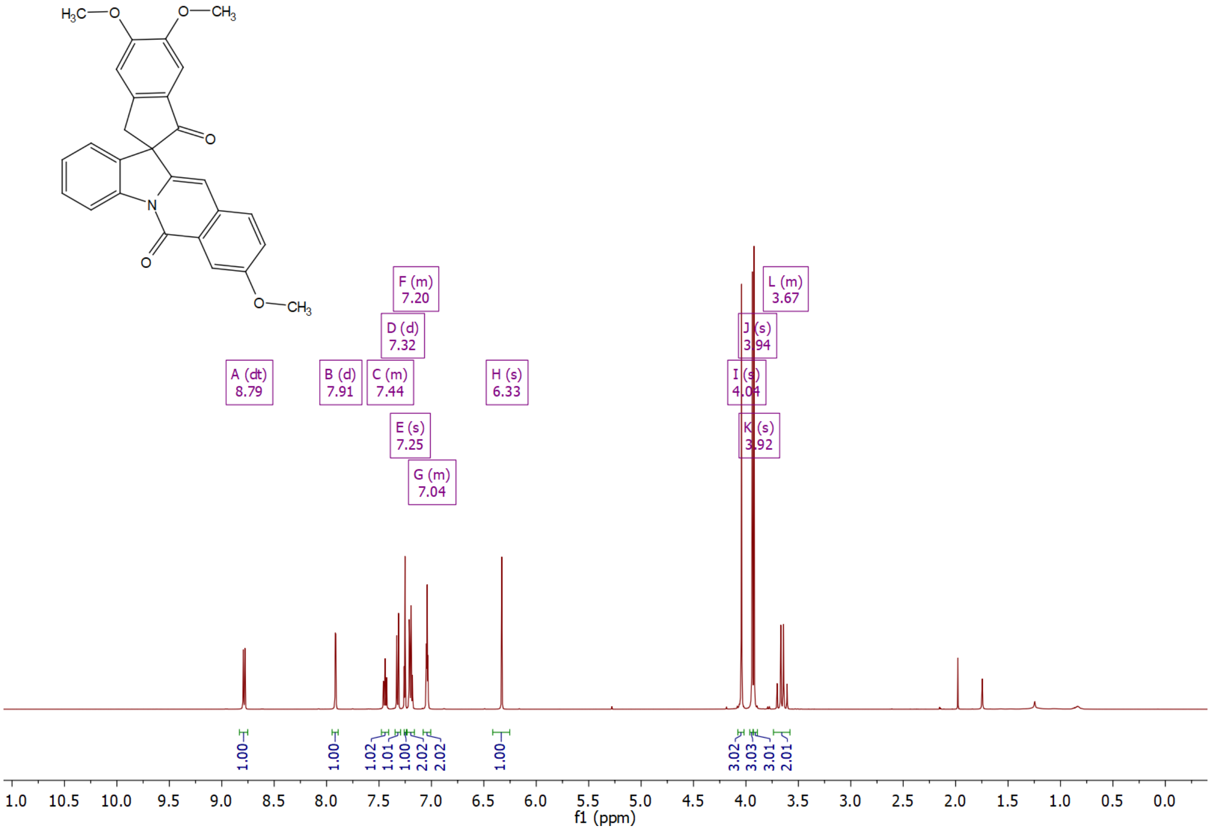

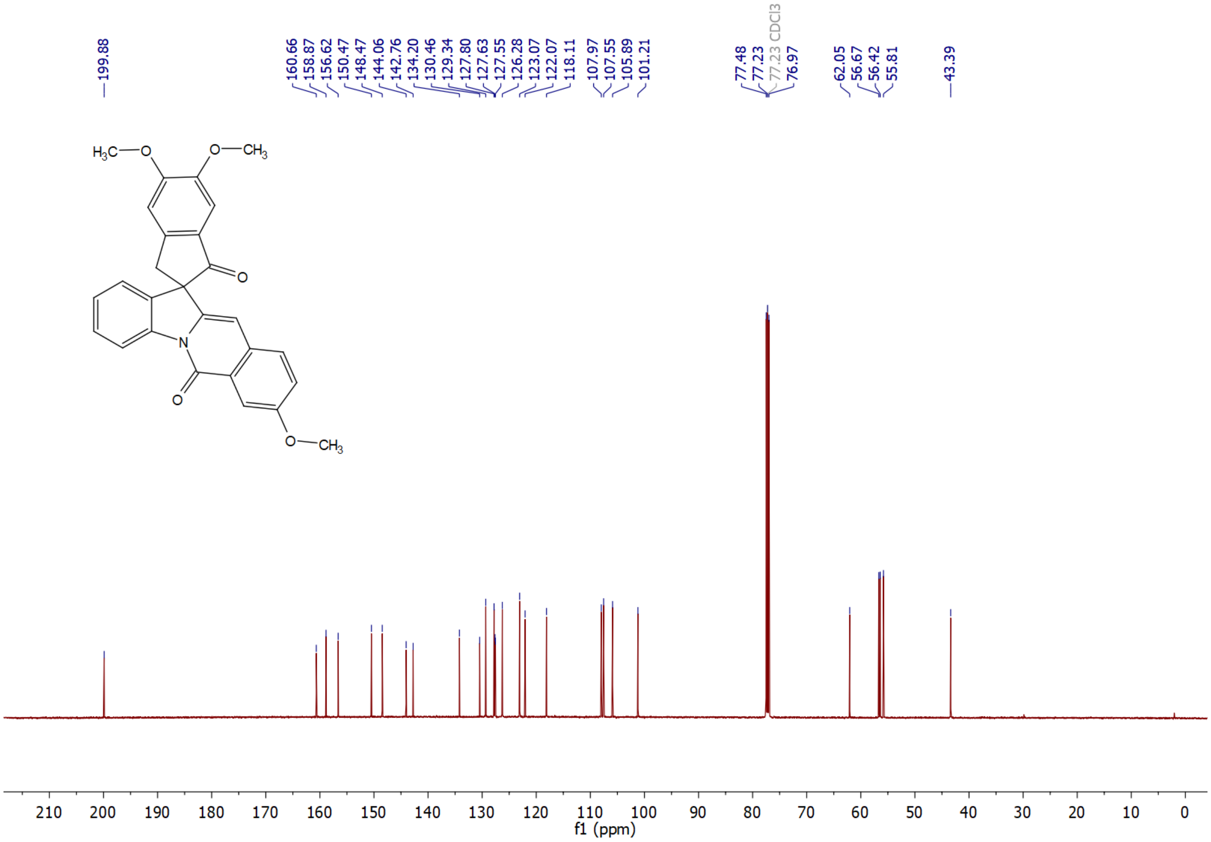

6'H-spiro[cyclopenta[a]naphthalene-2,12'-indolo[1,2-b]isoquinoline]-1,6'(3H)-dione [E17]:

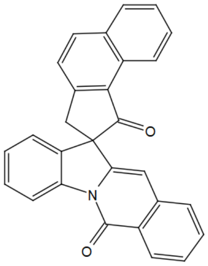

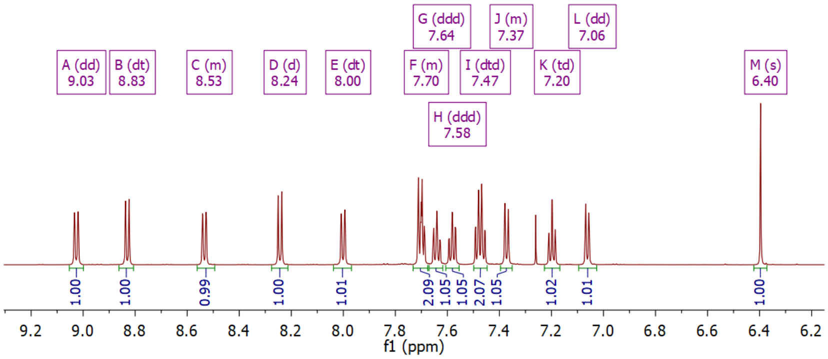

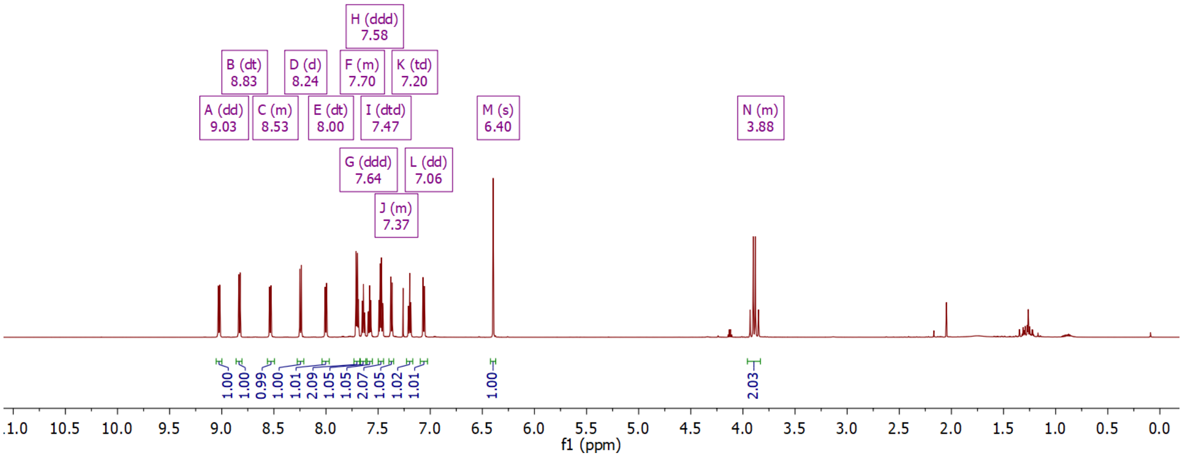

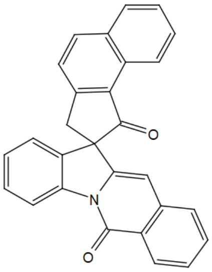

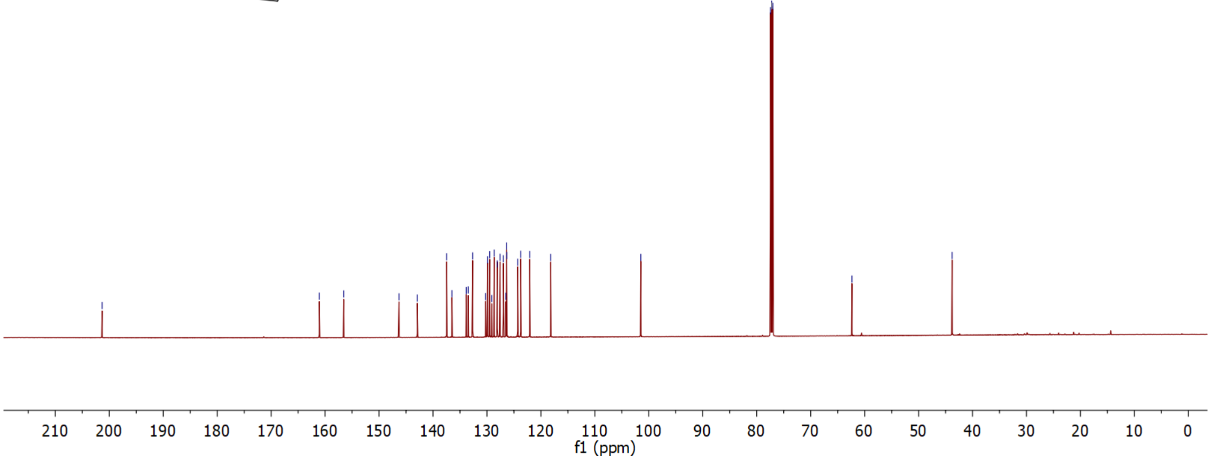

8'-methyl-6'H-spiro[cyclopenta[a]naphthalene-2,12'-indolo[1,2-b]isoquinoline]-1,6'(3H)-dione **[E18]**:

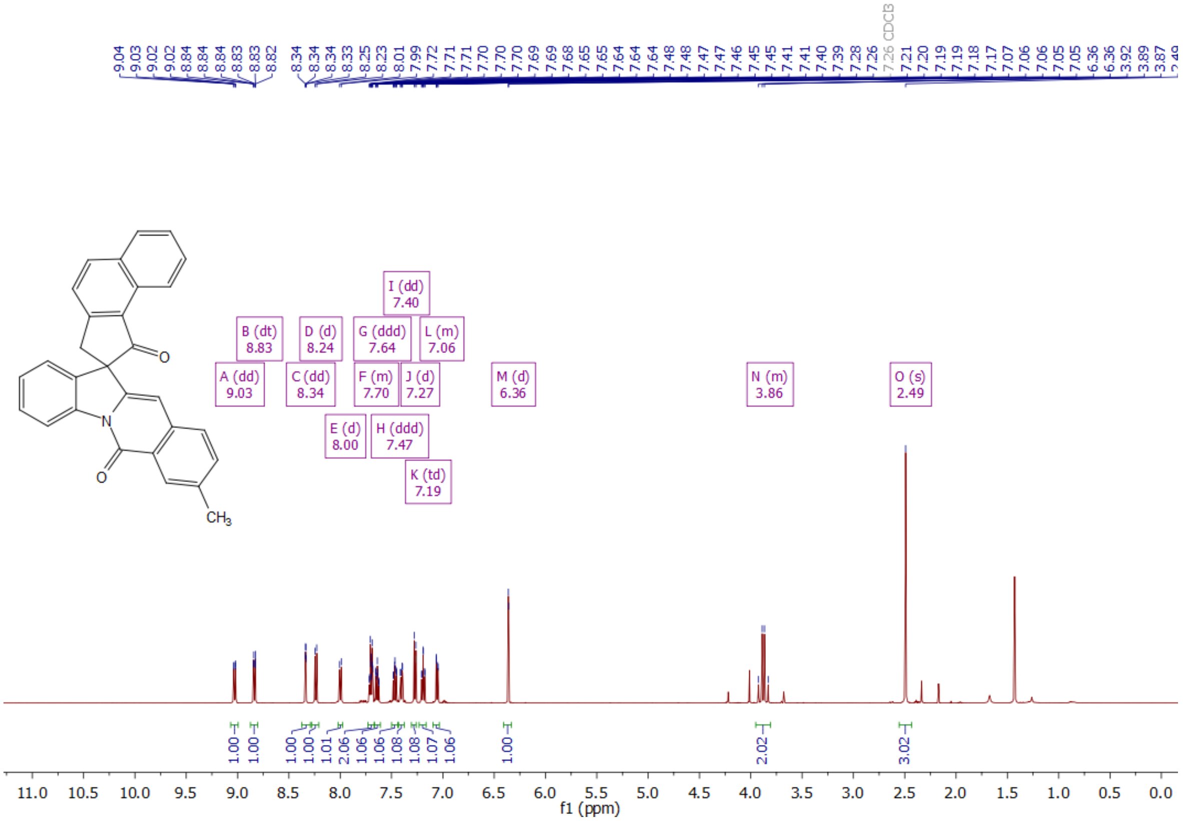

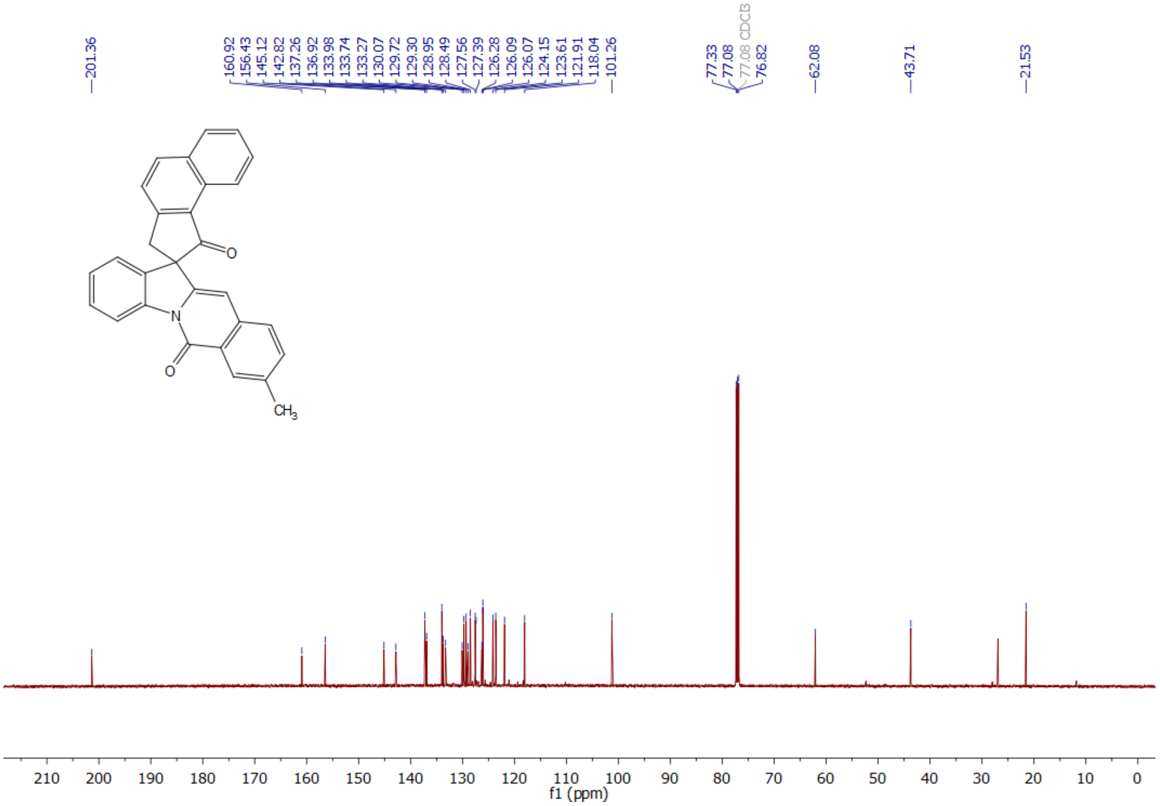

8'-methyl-6'H-spiro[cyclopenta[a]naphthalene-2,12'-indolo[1,2-b]isoquinoline]-1,6'(3H)-dione **[E18-resynthesized]:**

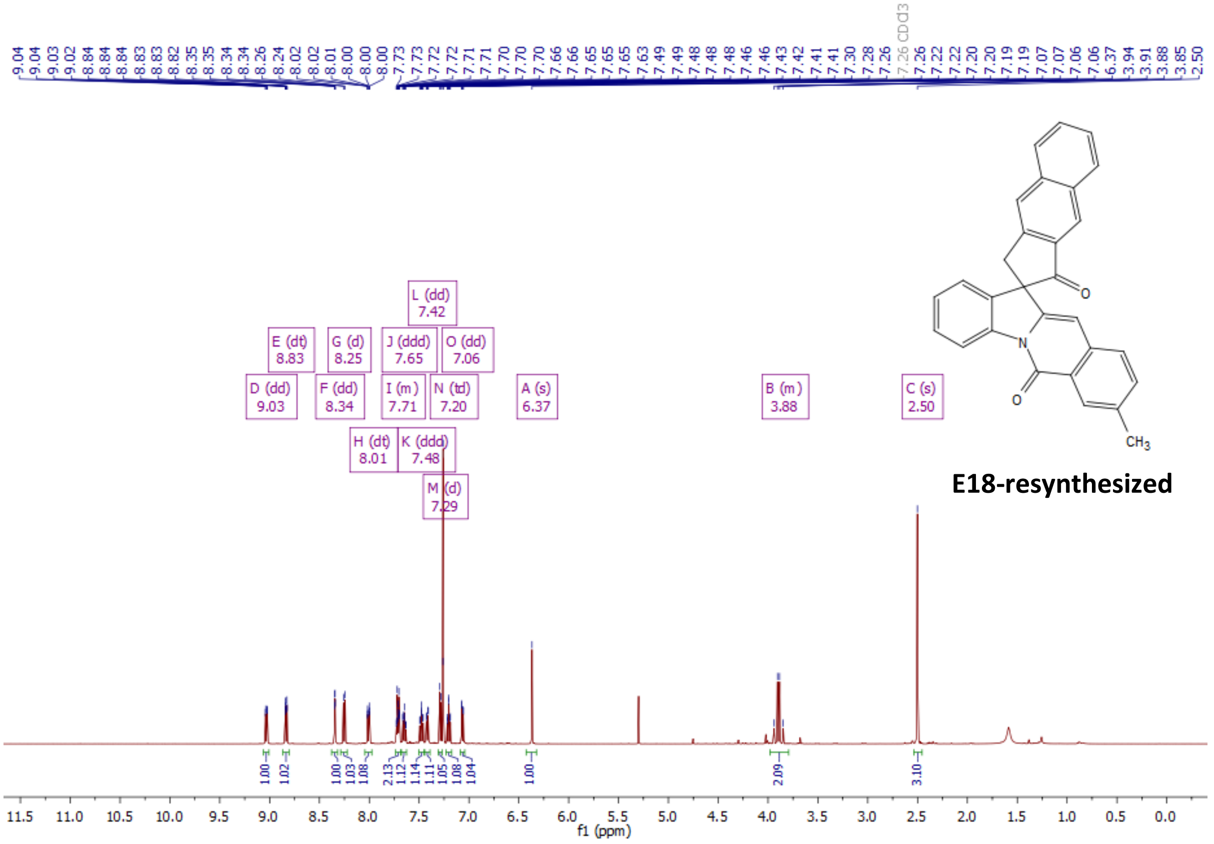

8'-methoxy-6'H-spiro[cyclopenta[a]naphthalene-2,12'-indolo[1,2-b]isoquinoline]-1,6'(3H)-dione [**E19**]:

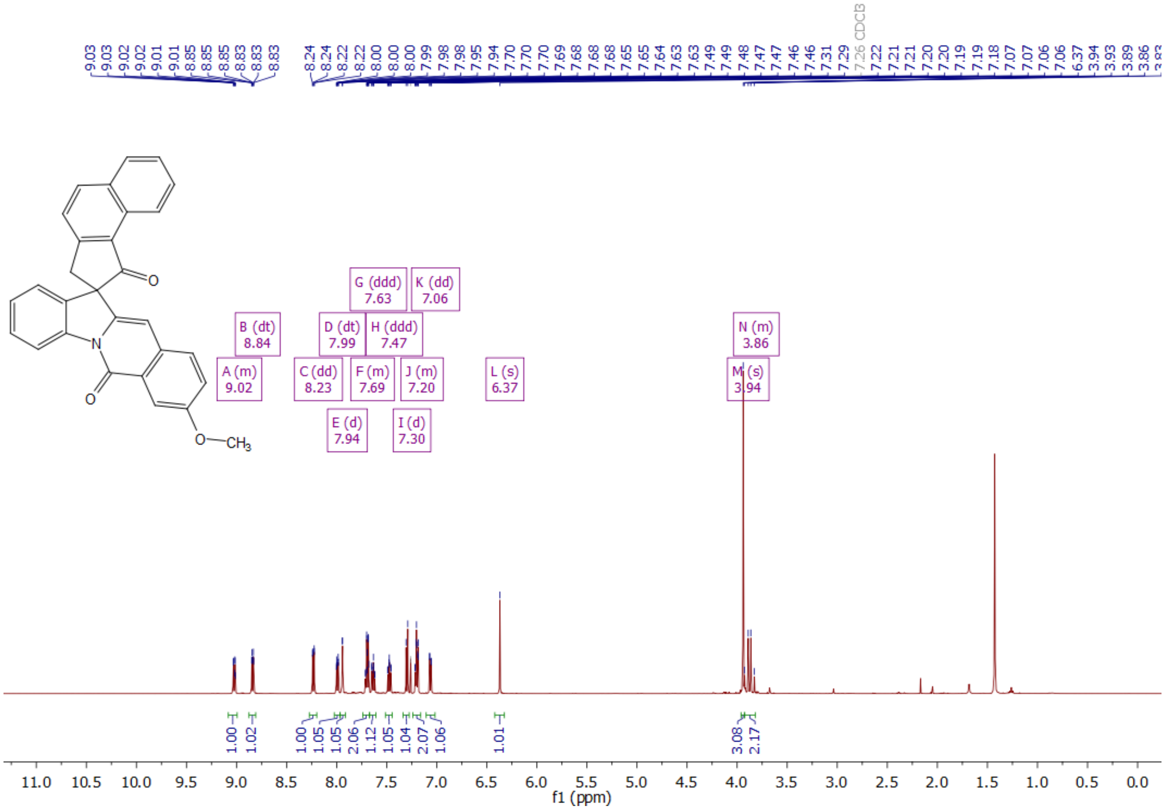

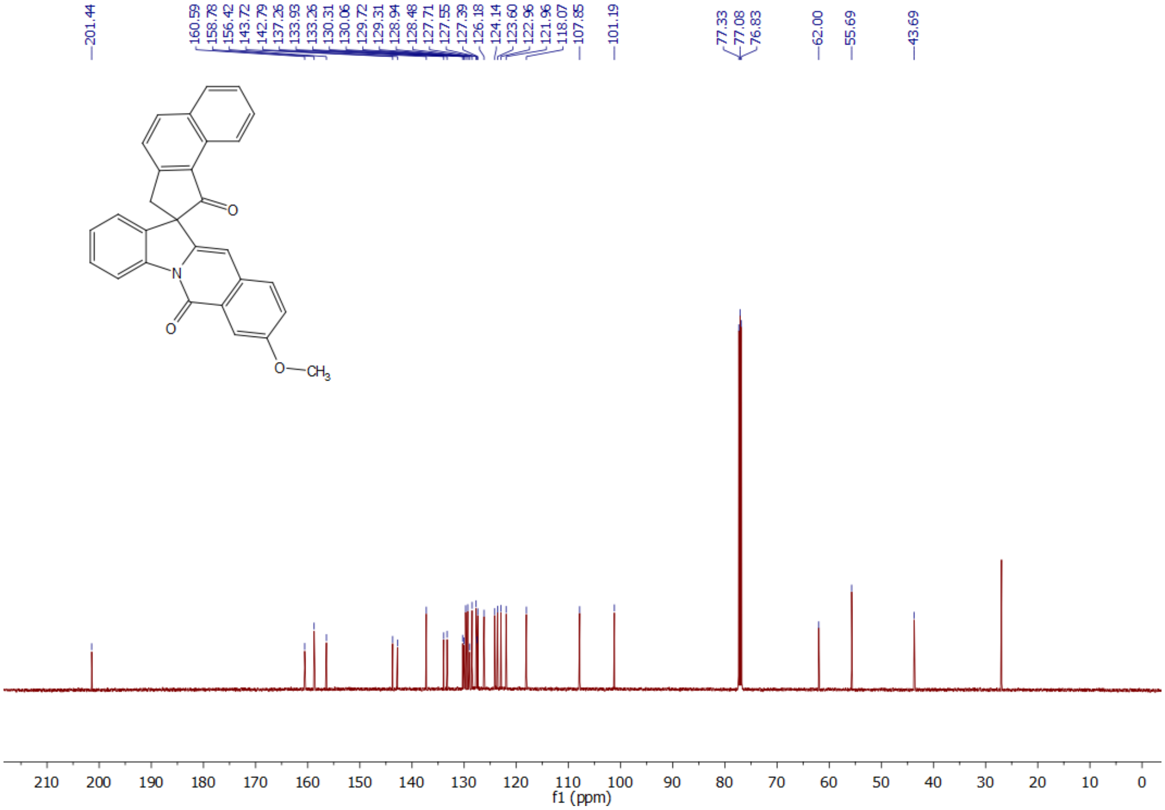

2-(2-bromobenzyl)-2-methylindolin-3-one [**4a**]:

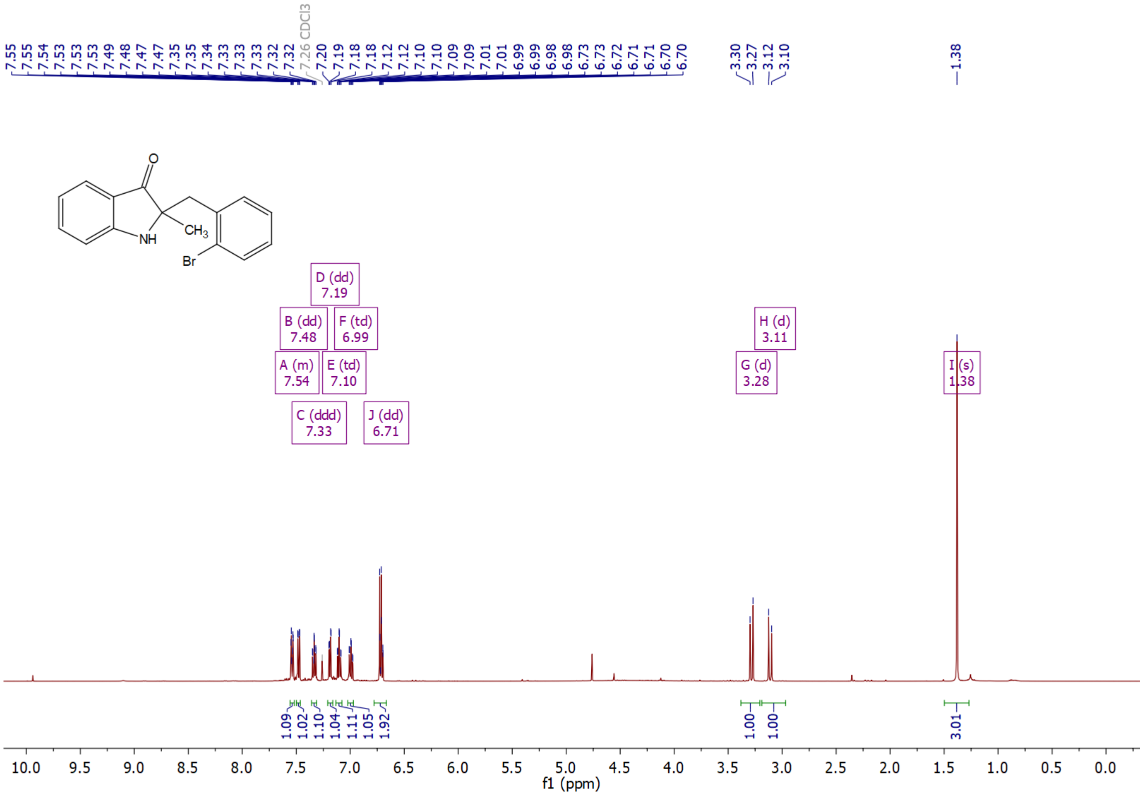

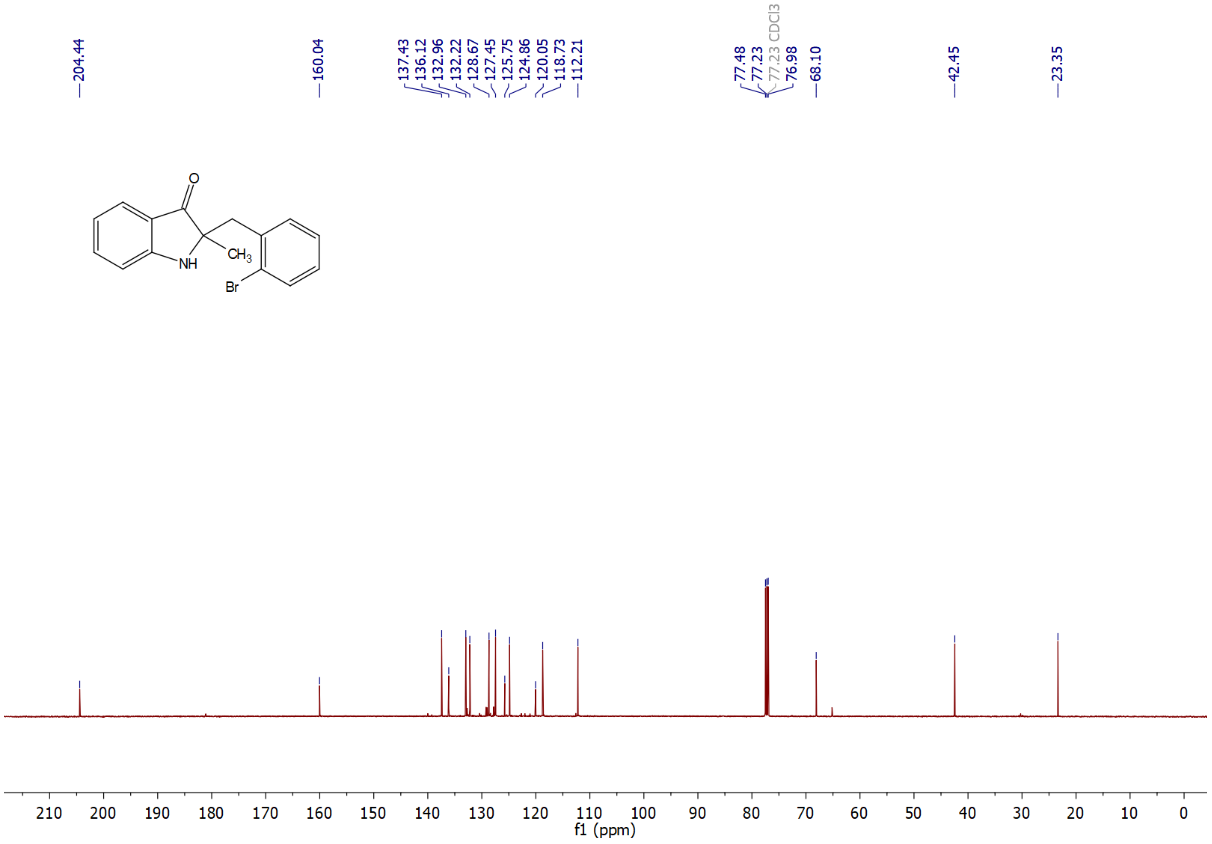

2-(2-bromo-3-fluorobenzyl)-2-methylindolin-3-one [**4b**]:

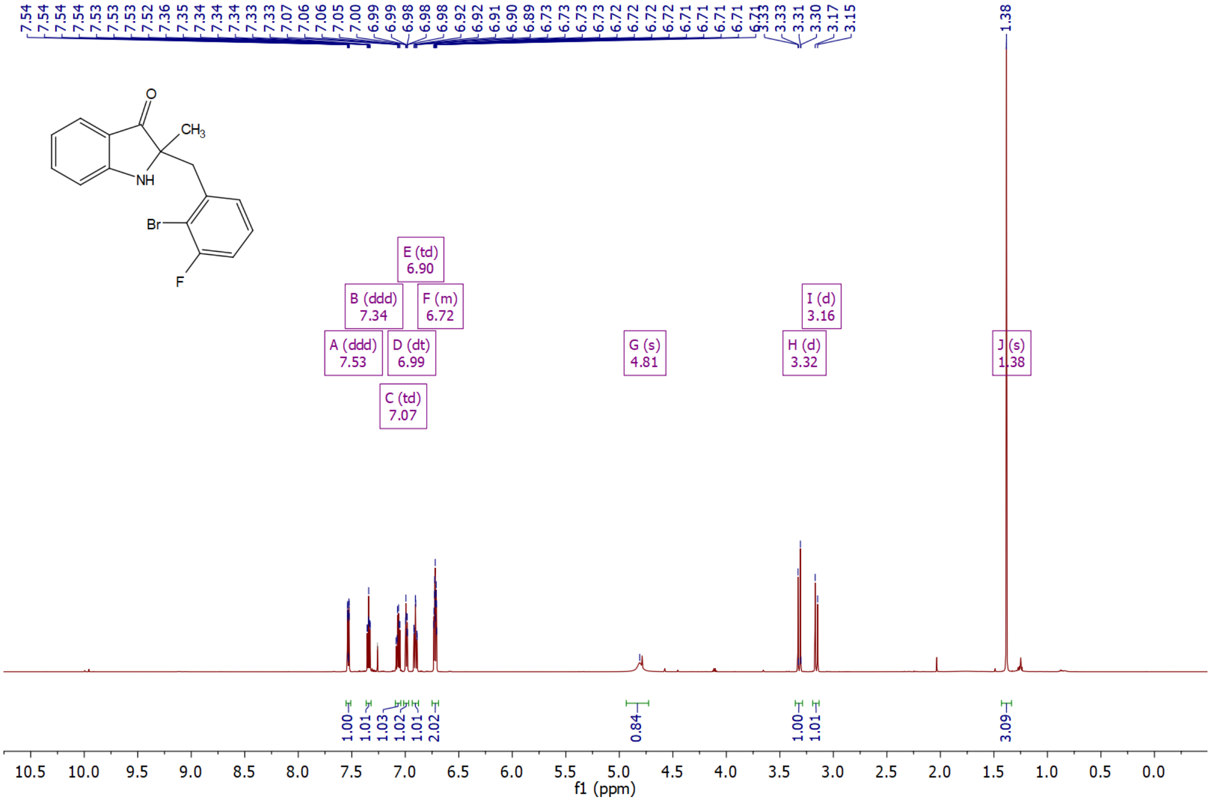

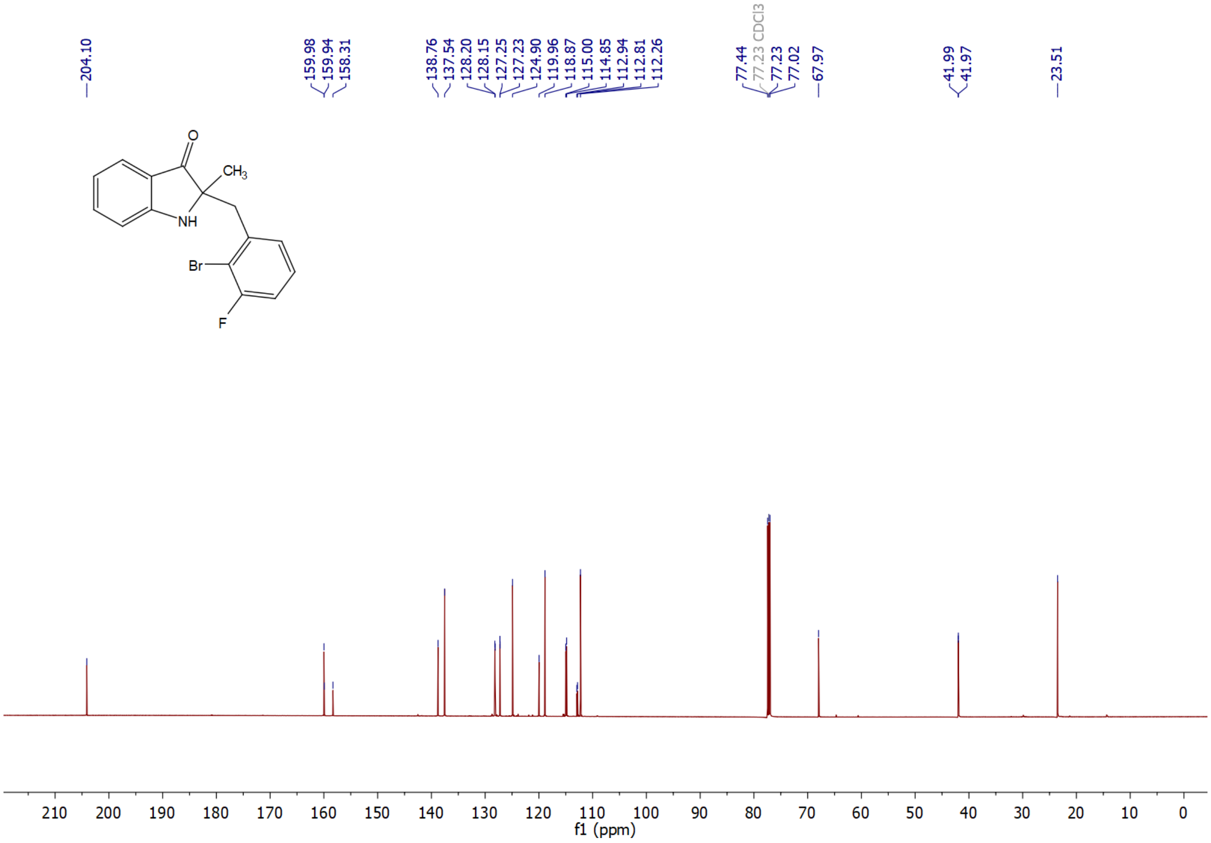

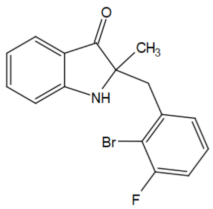
O
CH3
NH
Br
F

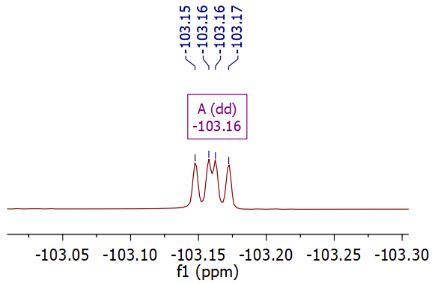
-103.15
-103.16
-103.16
-103.17
A (dd)
-103.16
-103.05 -103.10 -103.15 -103.20 -103.25 -103.30
f1 (ppm)

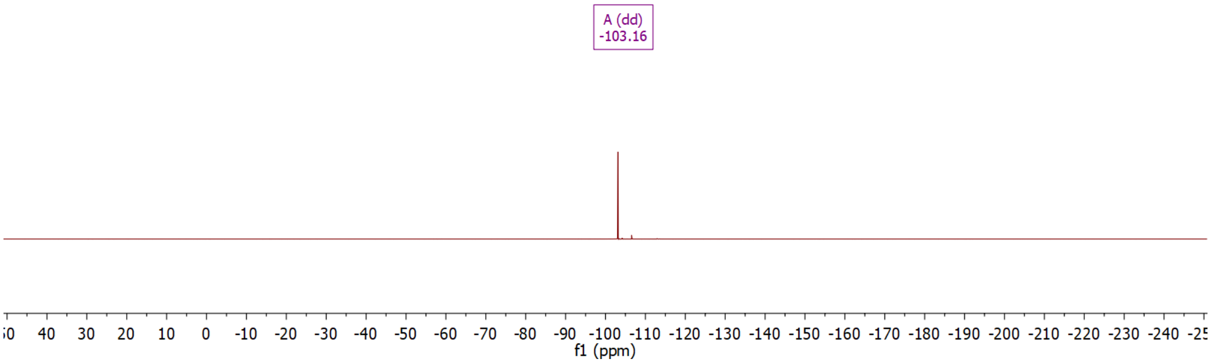
A (dd)
-103.16
40 30 20 10 0 -10 -20 -30 -40 -50 -60 -70 -80 -90 -100 -110 -120 -130 -140 -150 -160 -170 -180 -190 -200 -210 -220 -230 -240
f1 (ppm)

2-(2-bromo-4-chlorobenzyl)-2-methylindolin-3-one [**4c**]:

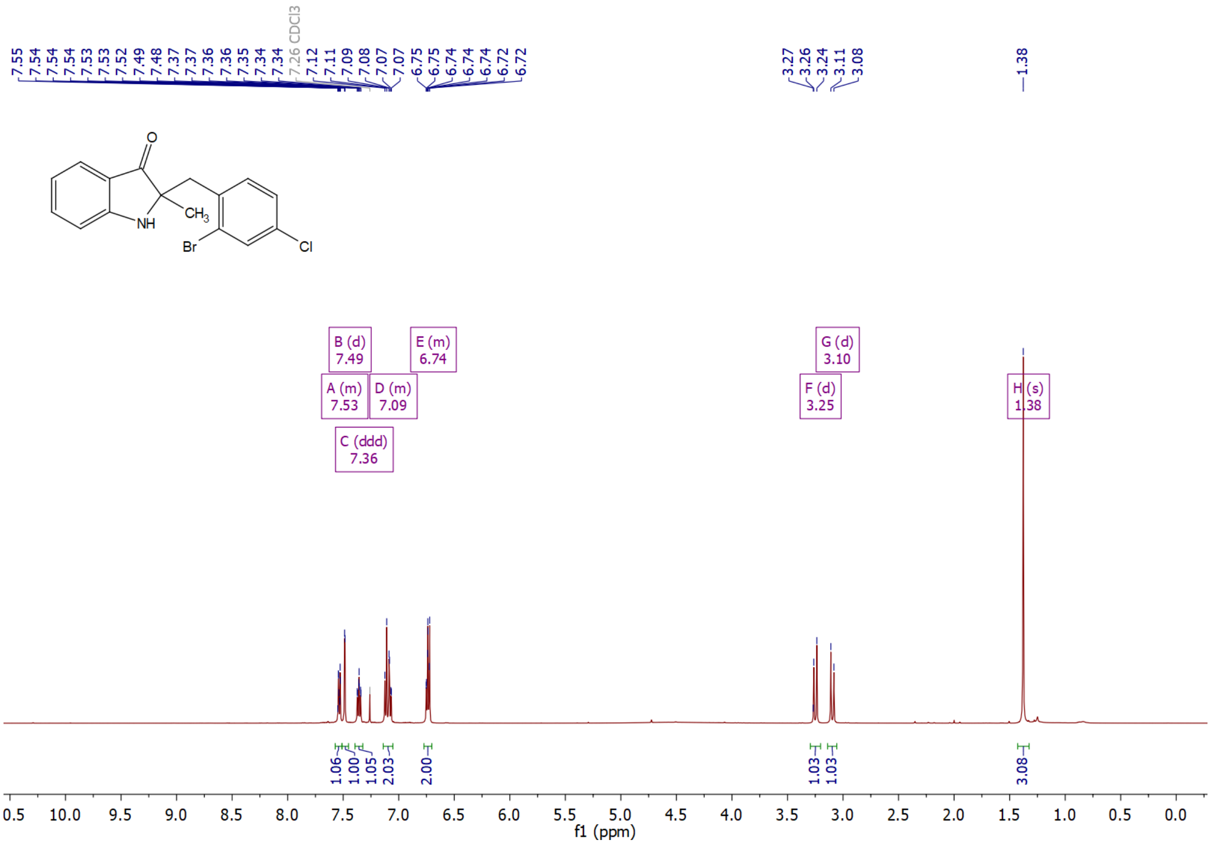

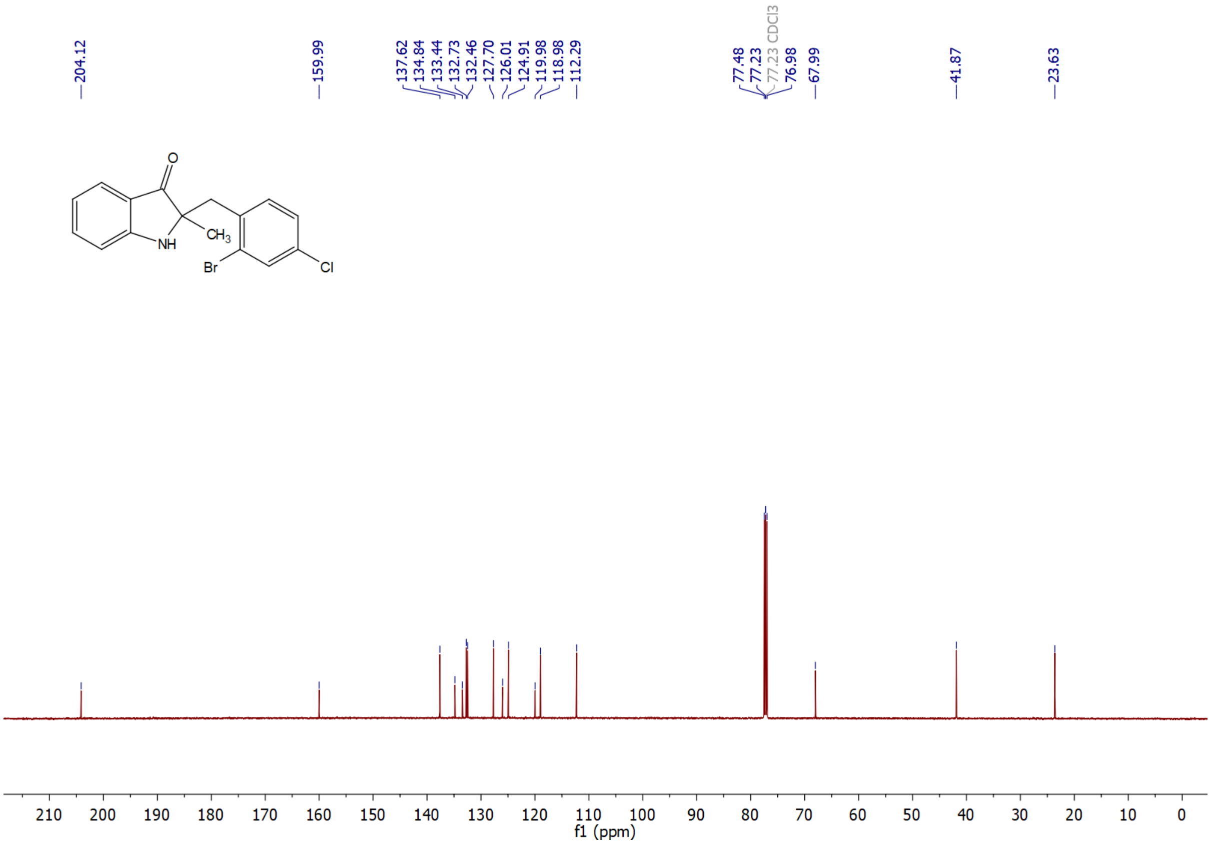

2-(2-bromo-4-methylbenzyl)-2-methylindolin-3-one [**4d**]:

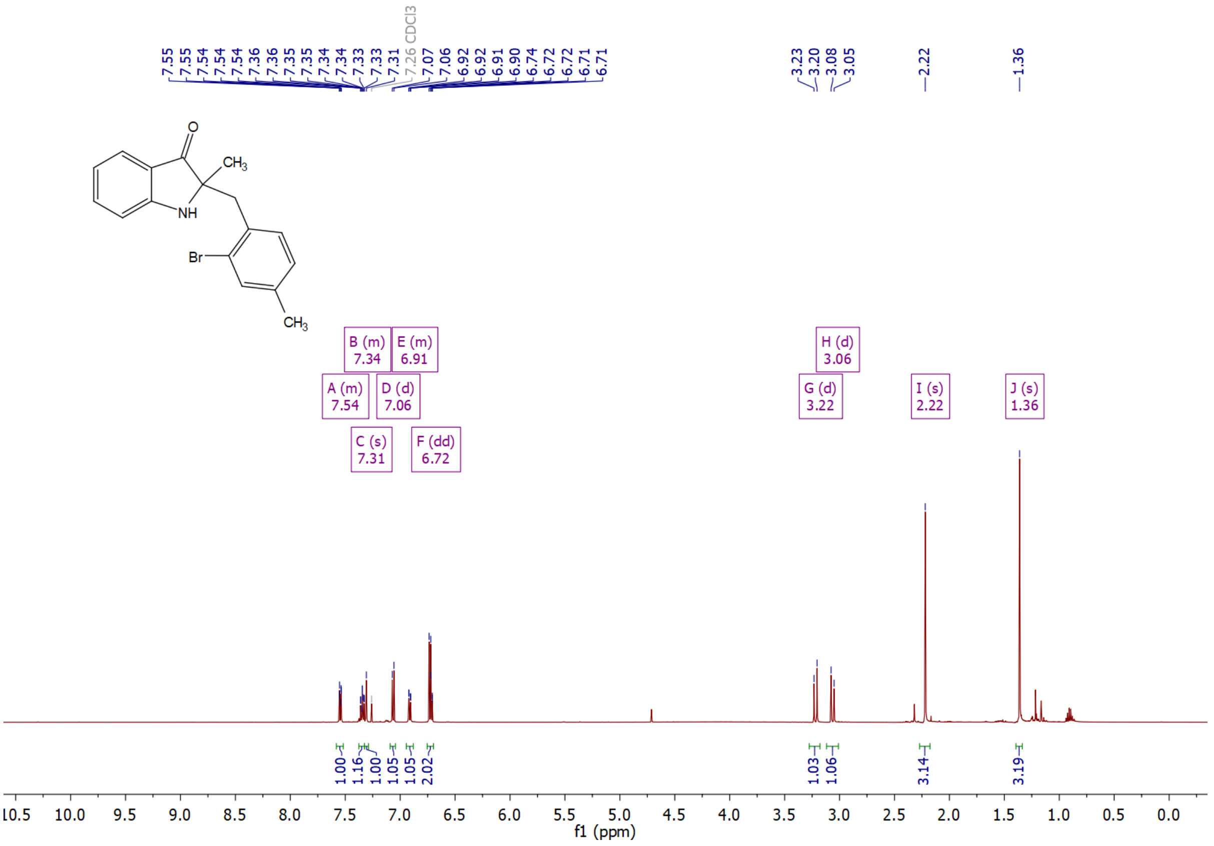

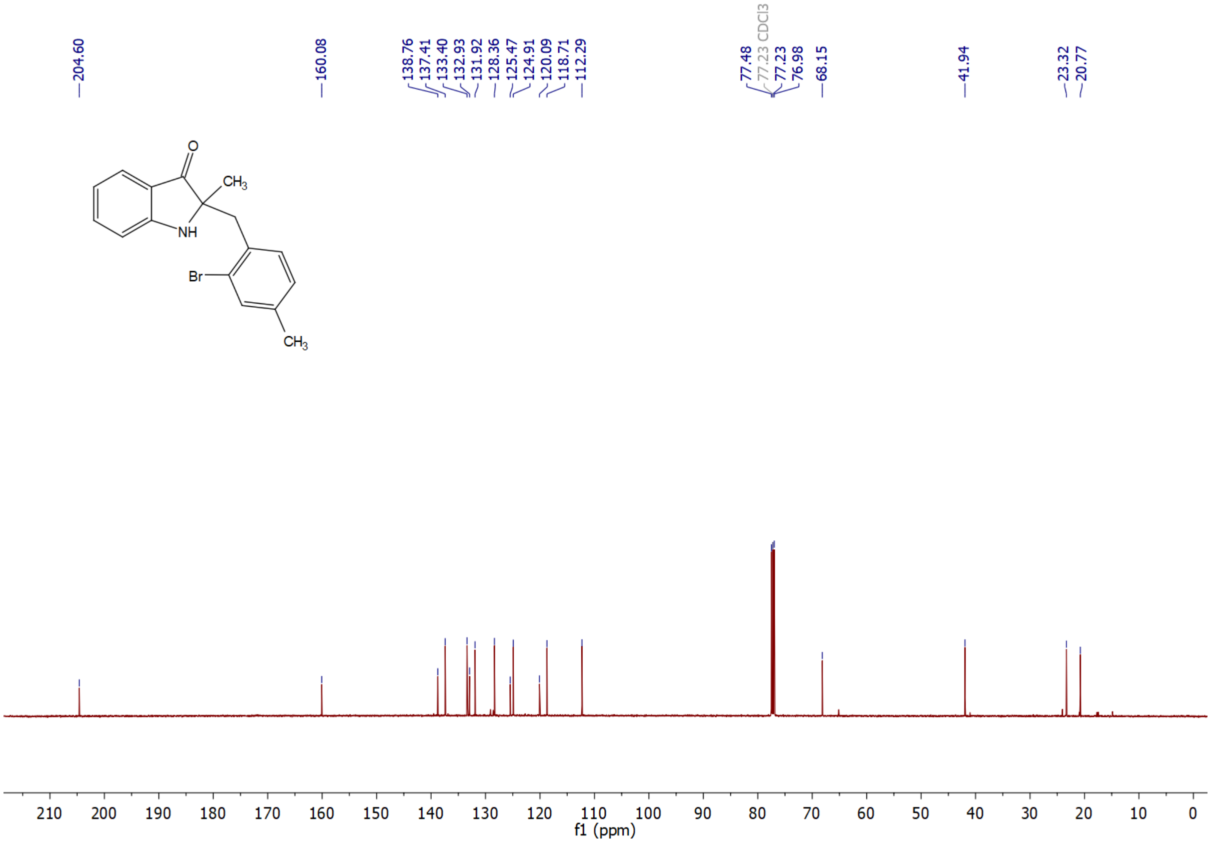

2-(2-bromo-5-fluorobenzyl)-2-methylindolin-3-one [**4e**]:

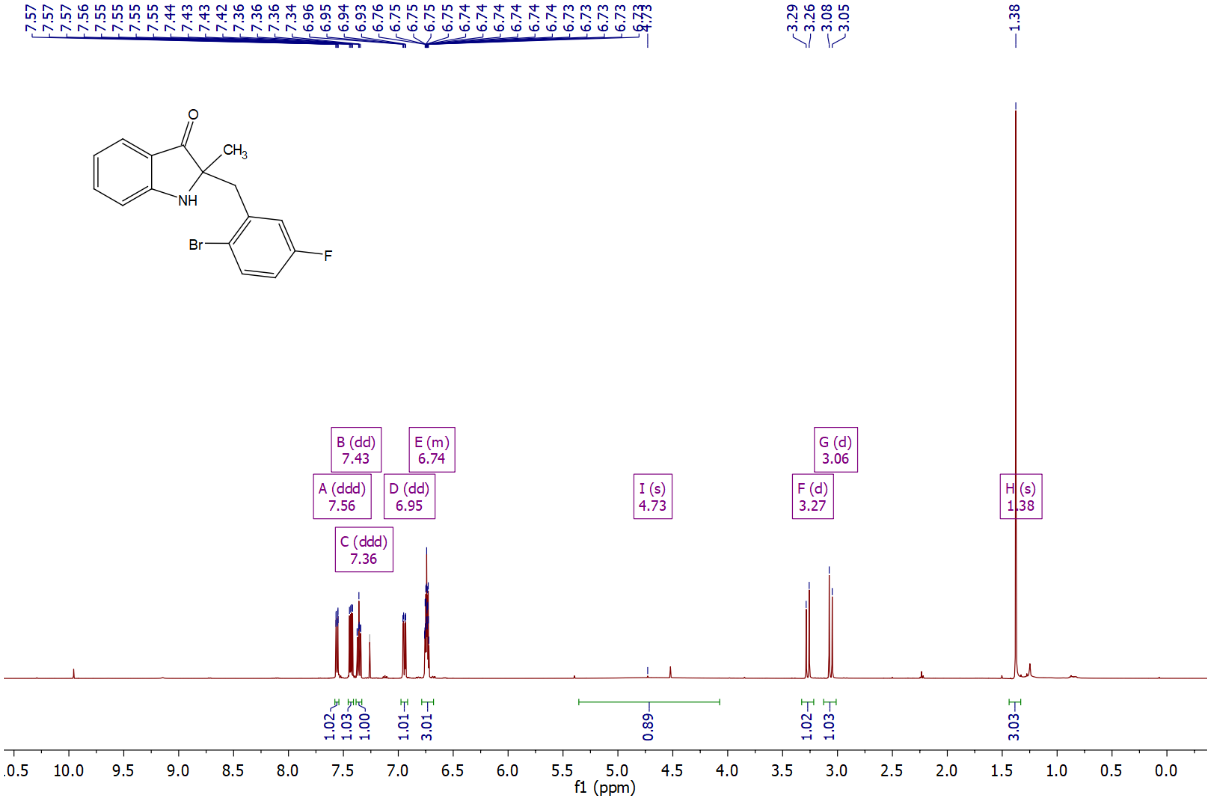

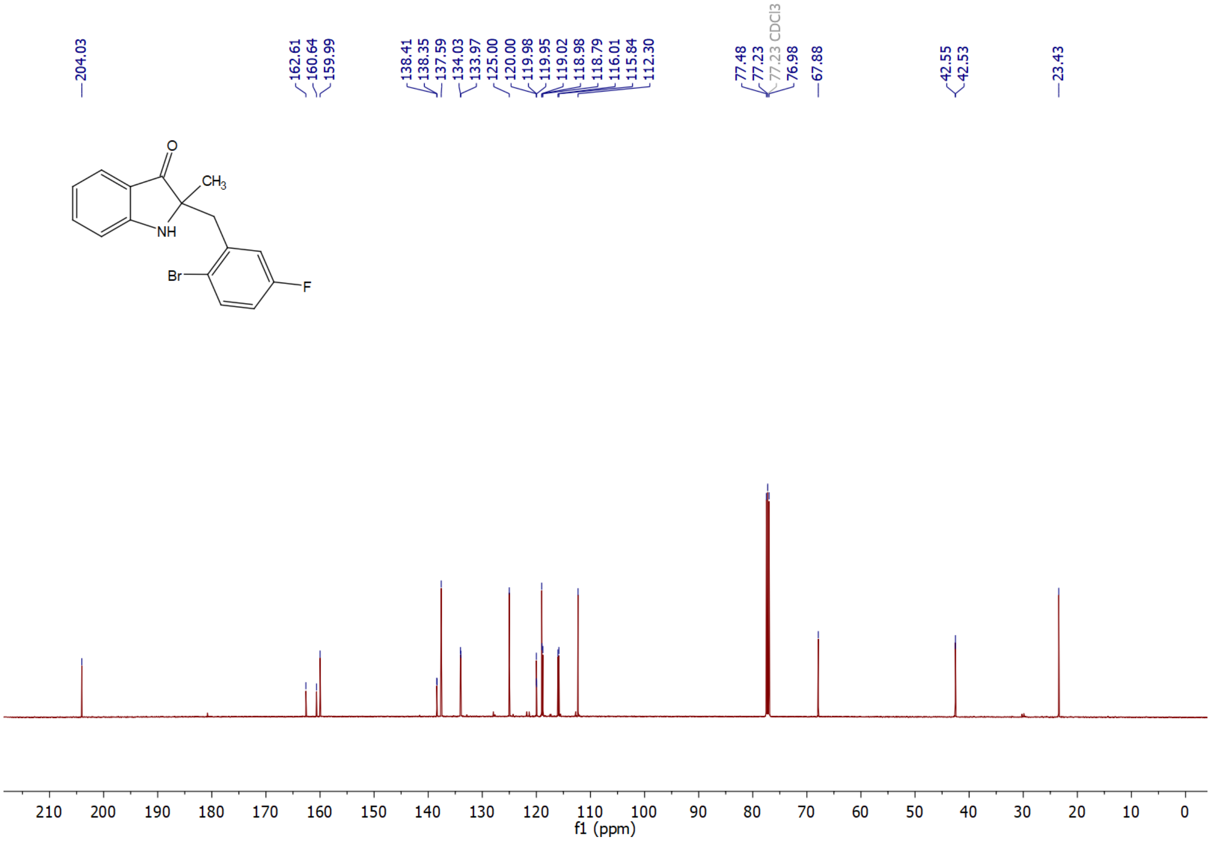

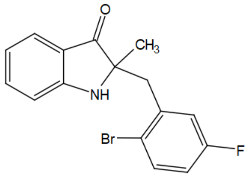
O
CH3
NH
Br
F

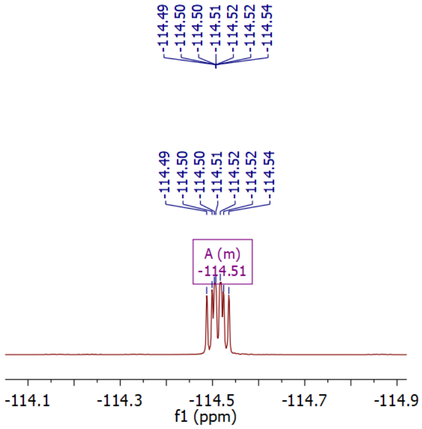
-114.49
-114.50
-114.50
-114.51
-114.52
-114.52
-114.54
A (m)
-114.51
-114.1
-114.3
-114.5
-114.7
-114.9
f1 (ppm)

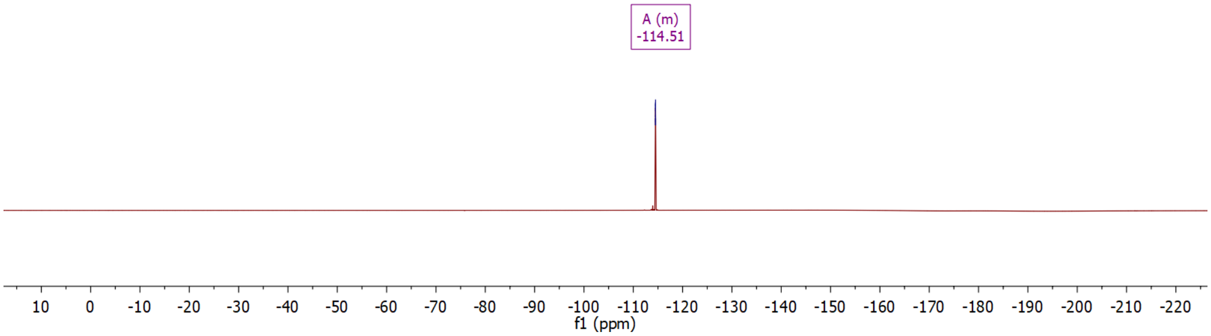
A (m)
-114.51
10 0 -10 -20 -30 -40 -50 -60 -70 -80 -90 -100 -110 -120 -130 -140 -150 -160 -170 -180 -190 -200 -210 -220
f1 (ppm)

2-(2-bromo-5-methoxybenzyl)-2-methylindolin-3-one [**4f**]:

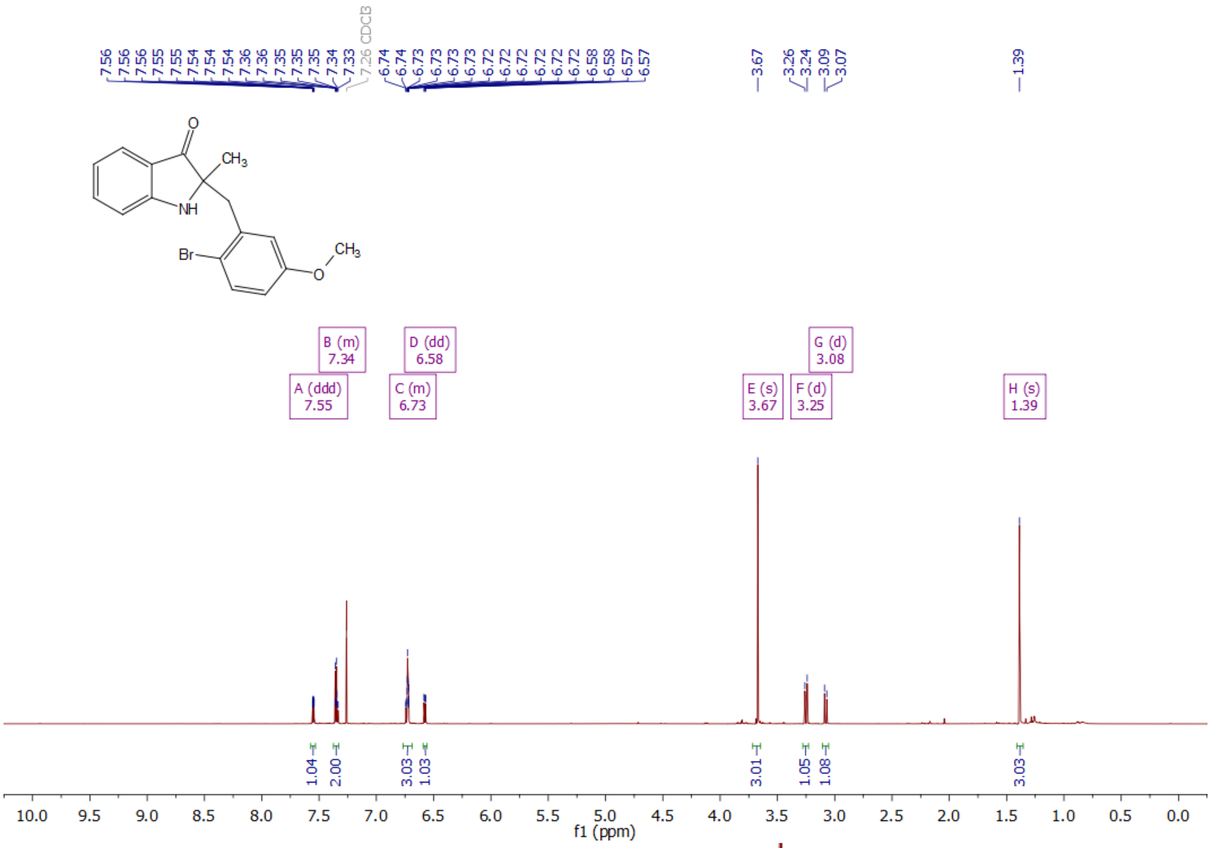

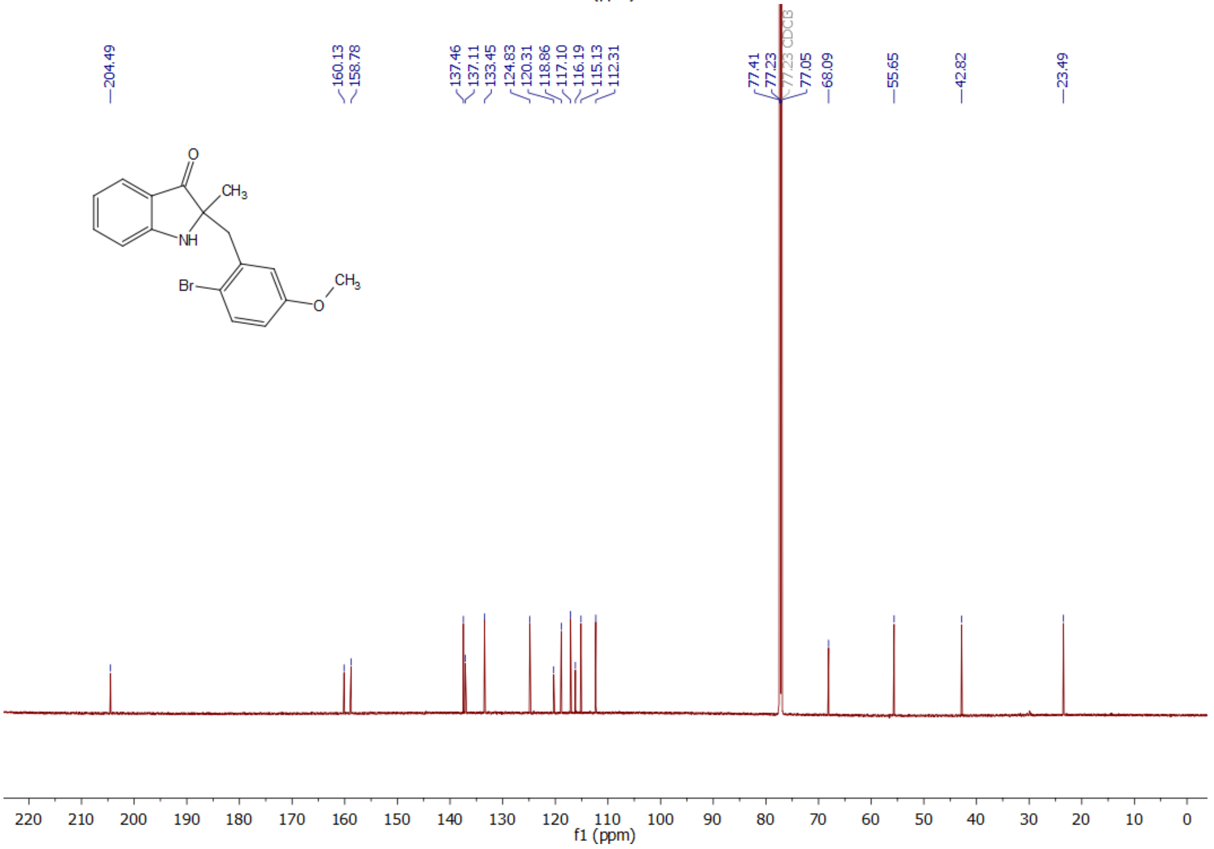

2-((6-bromobenzo[d][1,3]dioxol-5-yl)methyl)-2-methylindolin-3-one [**4g**]:

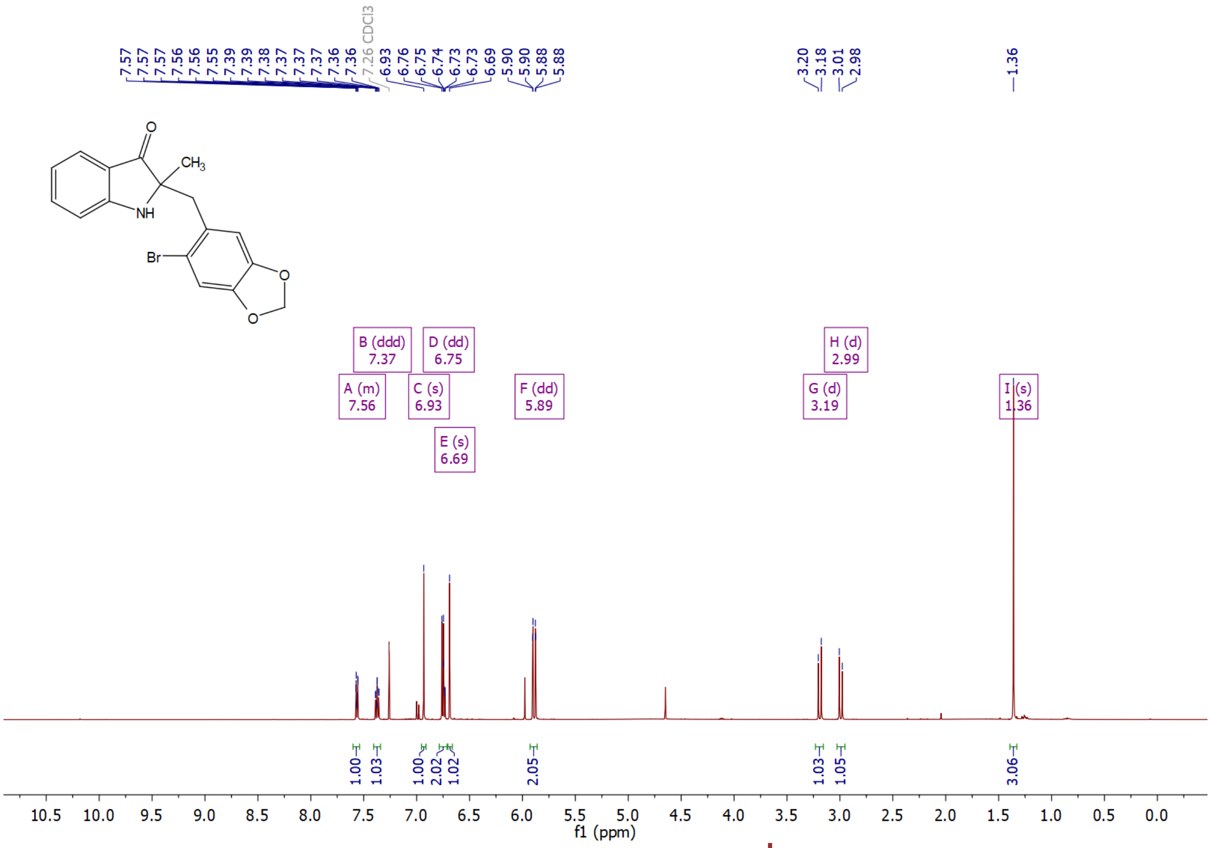

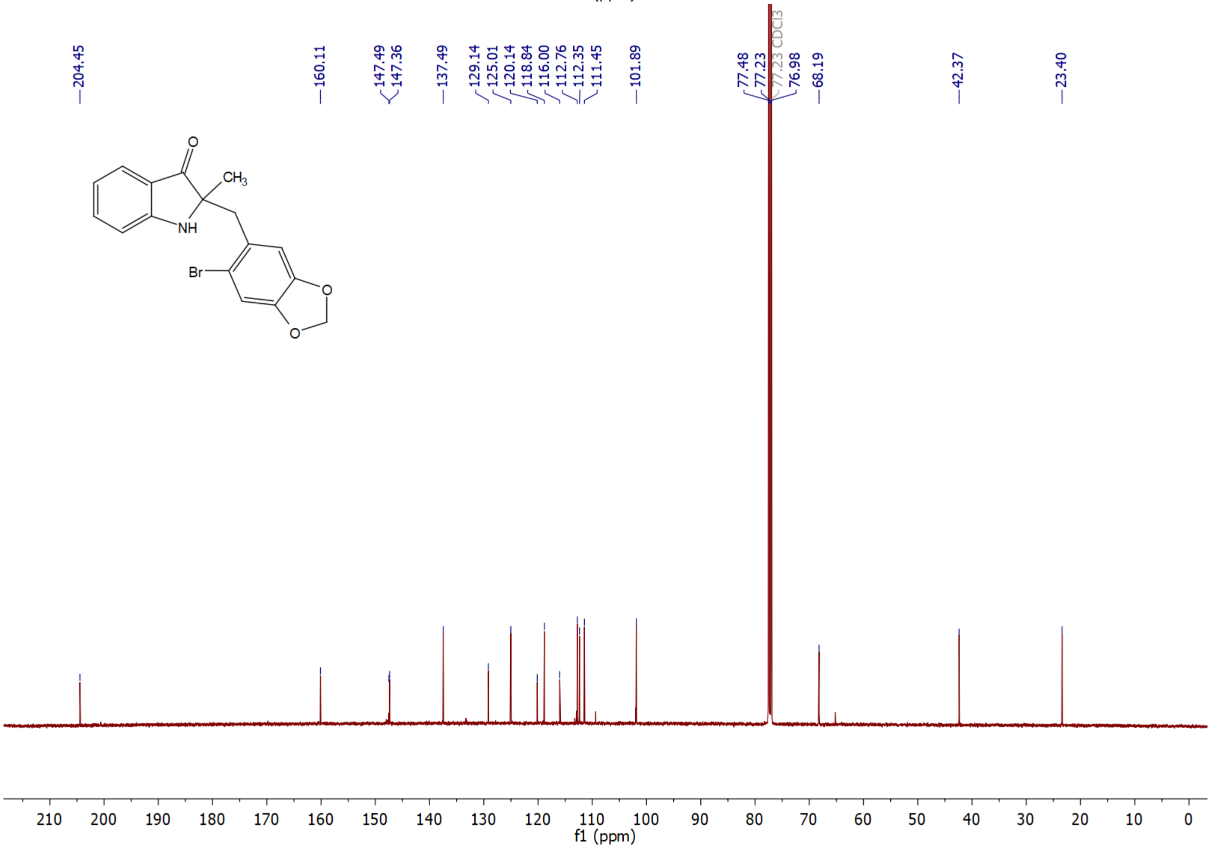

2-((1-bromonaphthalen-2-yl)methyl)-2-methylindolin-3-one [**4h**]:

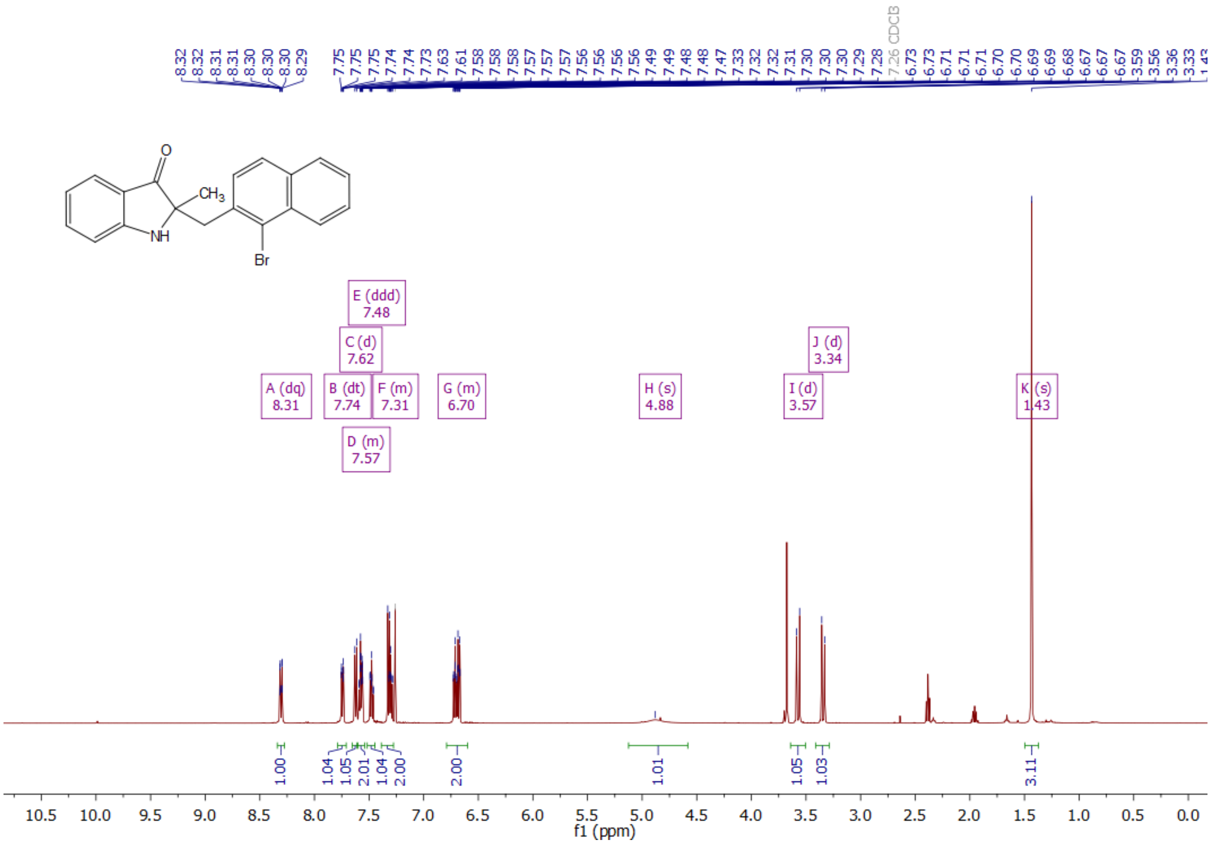

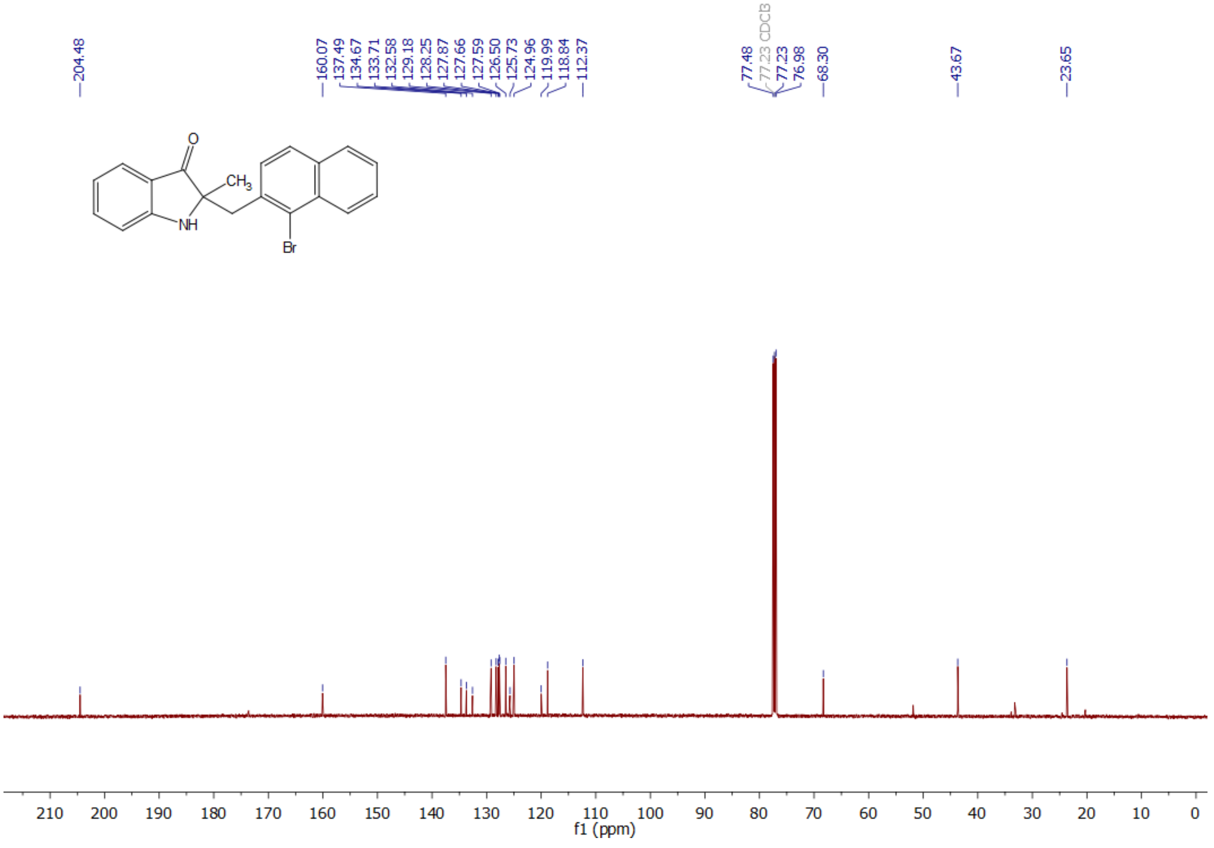

2-(2-bromobenzyl)-5-fluoro-2-methylindolin-3-one [**4i**]:

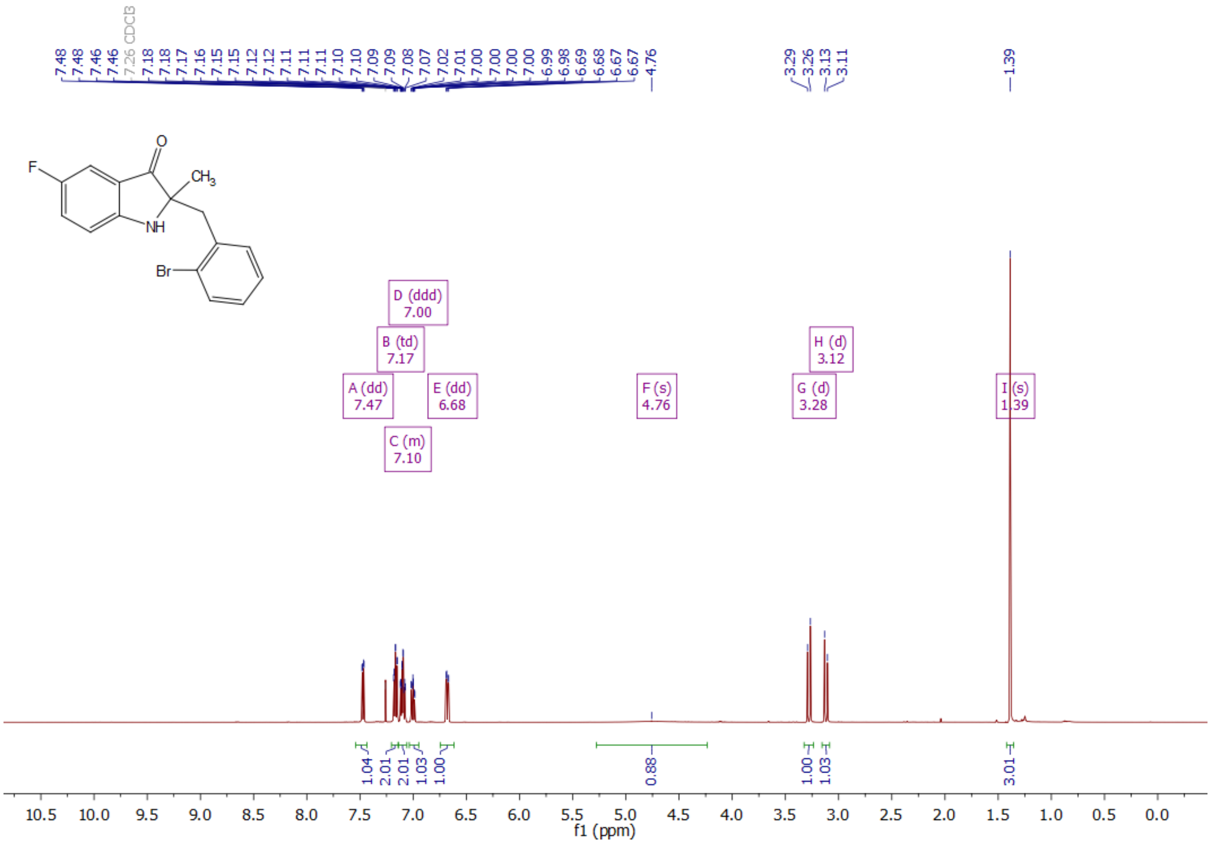

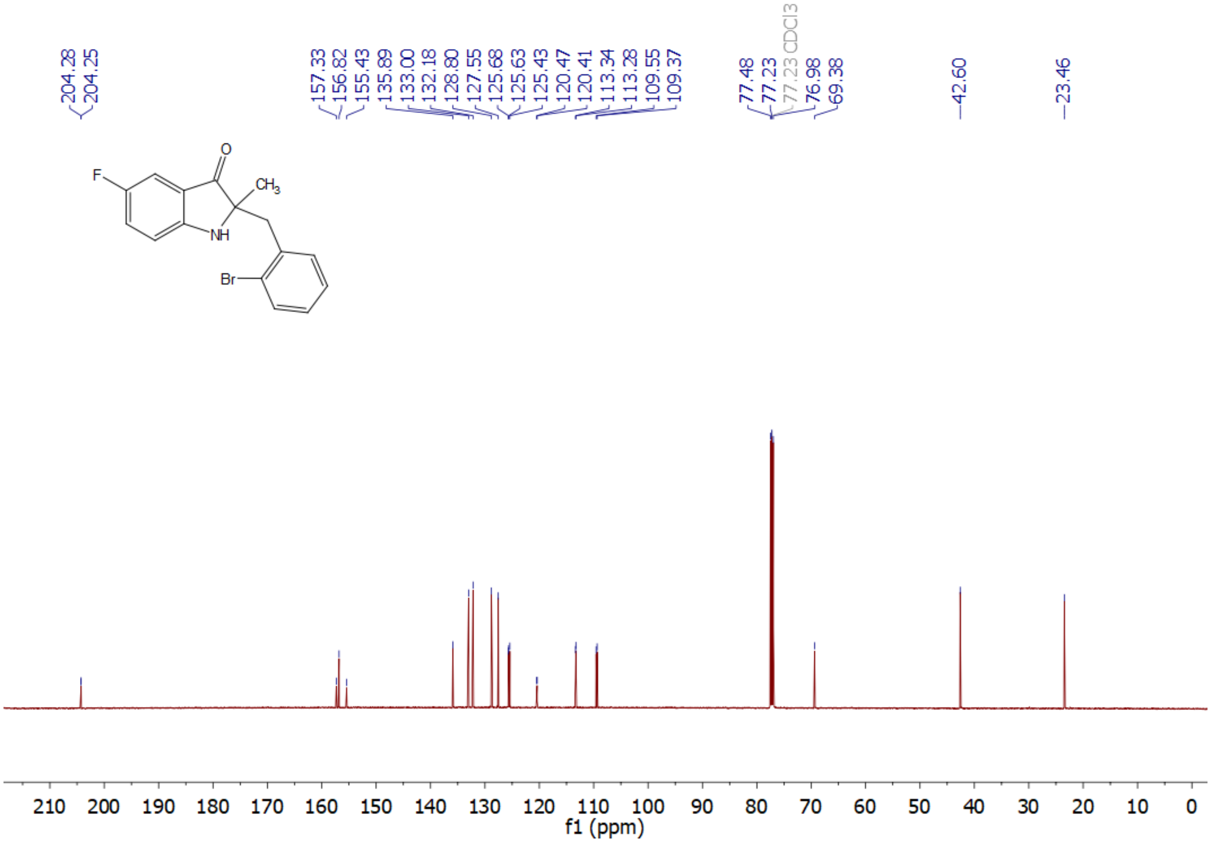

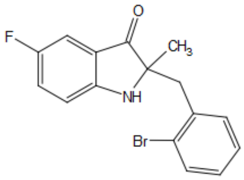
F
O
CH3
NH
Br

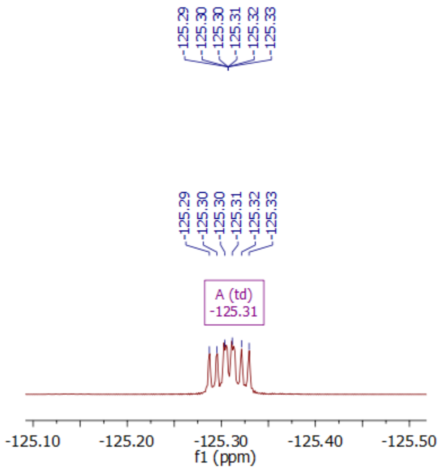
-125.29
-125.30
-125.30
-125.31
-125.32
-125.33
-125.29
-125.30
-125.30
-125.31
-125.32
-125.33
A (td)
-125.31
-125.10
-125.20
-125.30
-125.40
-125.50
f1 (ppm)

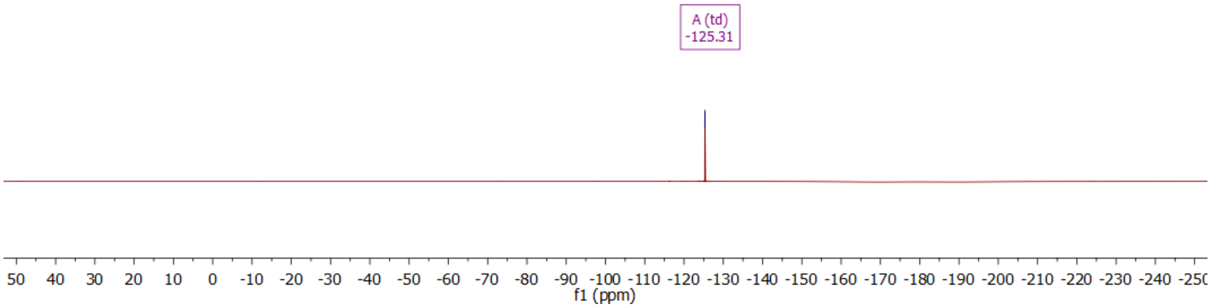
A (td)
-125.31
50 40 30 20 10 0 -10 -20 -30 -40 -50 -60 -70 -80 -90 -100 -110 -120 -130 -140 -150 -160 -170 -180 -190 -200 -210 -220 -230 -240 -250
f1 (ppm)

2-(2-bromobenzyl)-5-chloro-2-methylindolin-3-one [**4j**]:

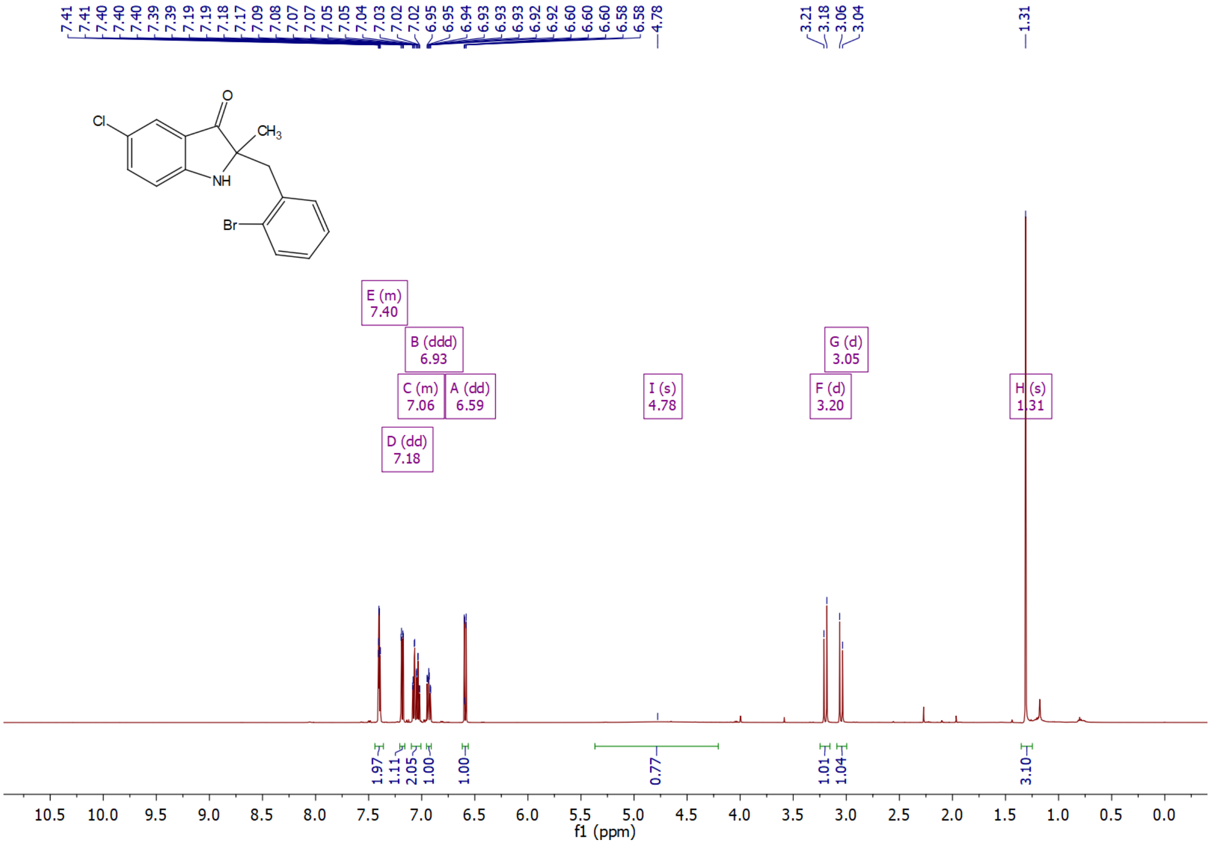

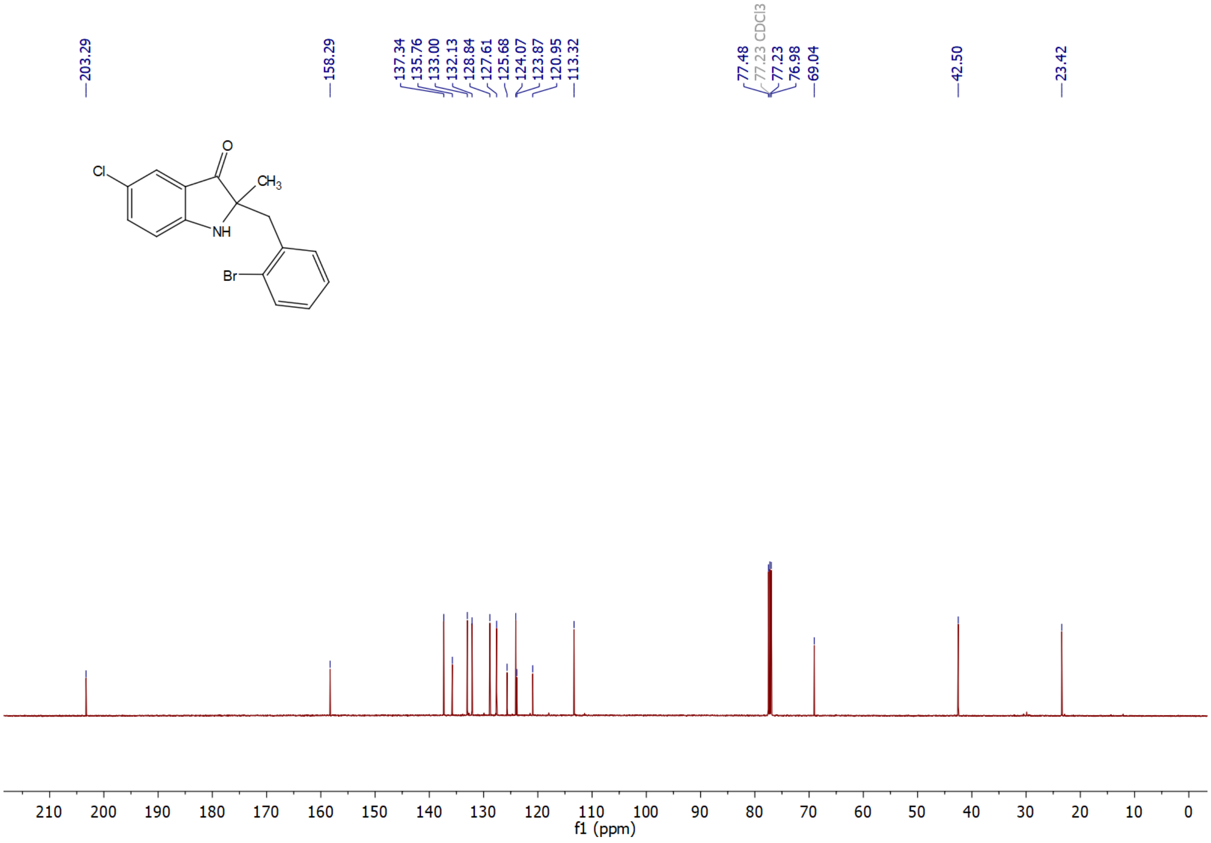

2-(2-bromobenzyl)-2,5-dimethylindolin-3-one [**4k**]:

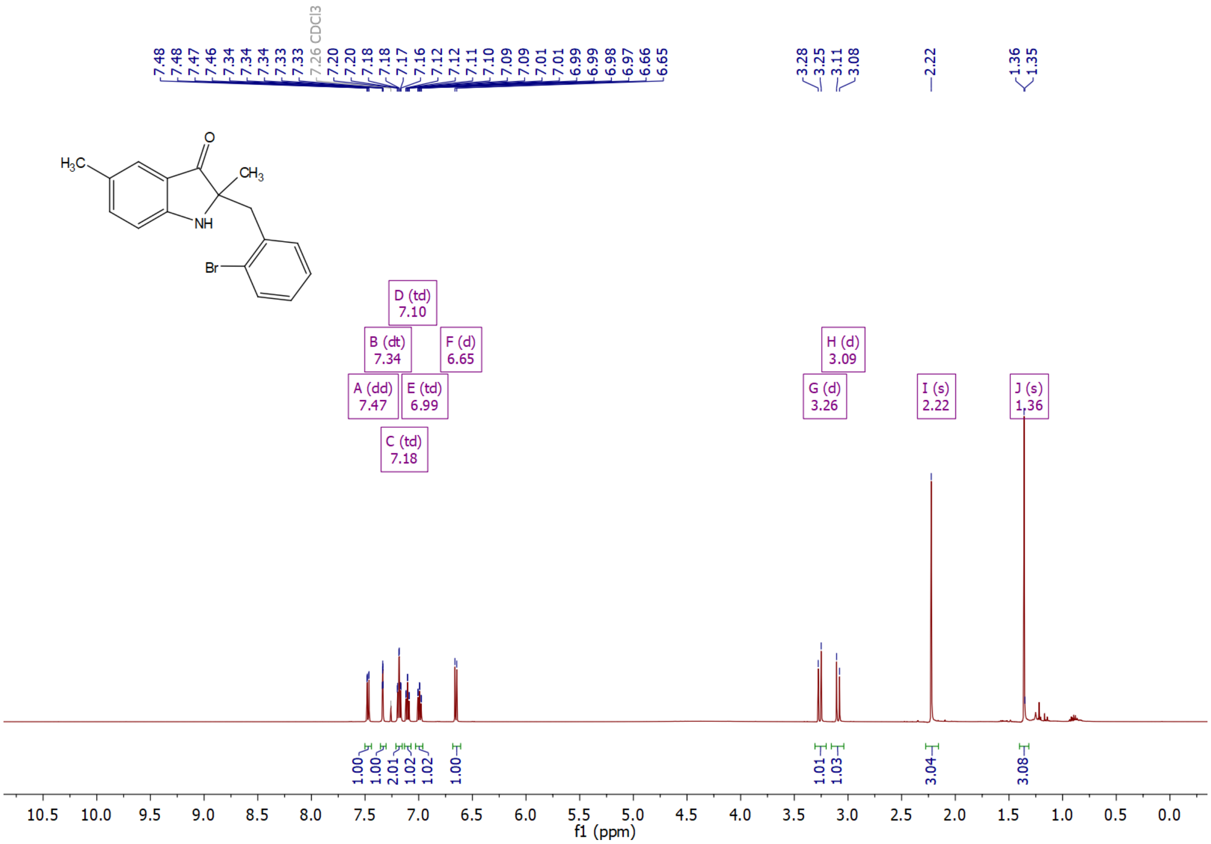

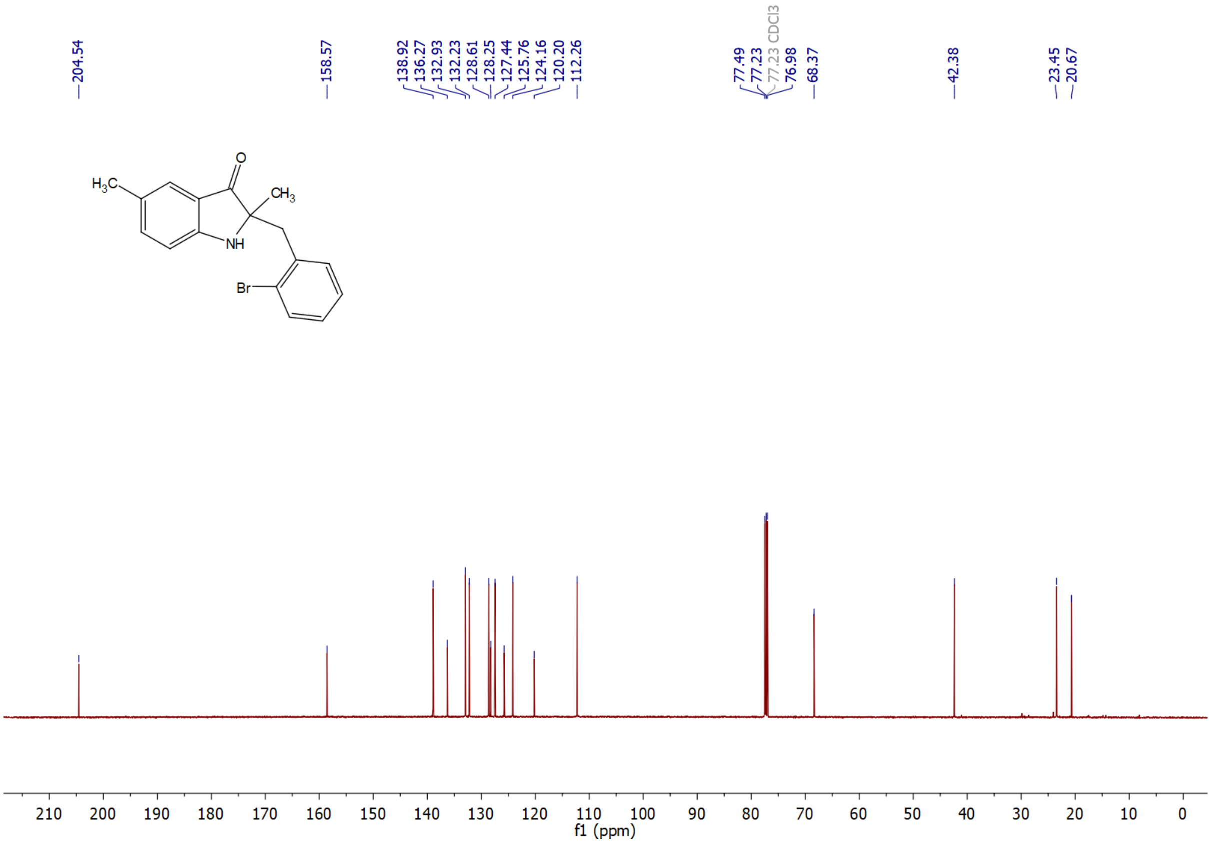

2-(2-bromo-3-fluorobenzyl)-2,5-dimethylindolin-3-one [**4l**]:

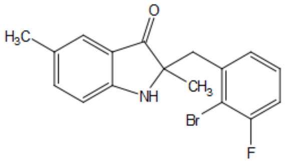

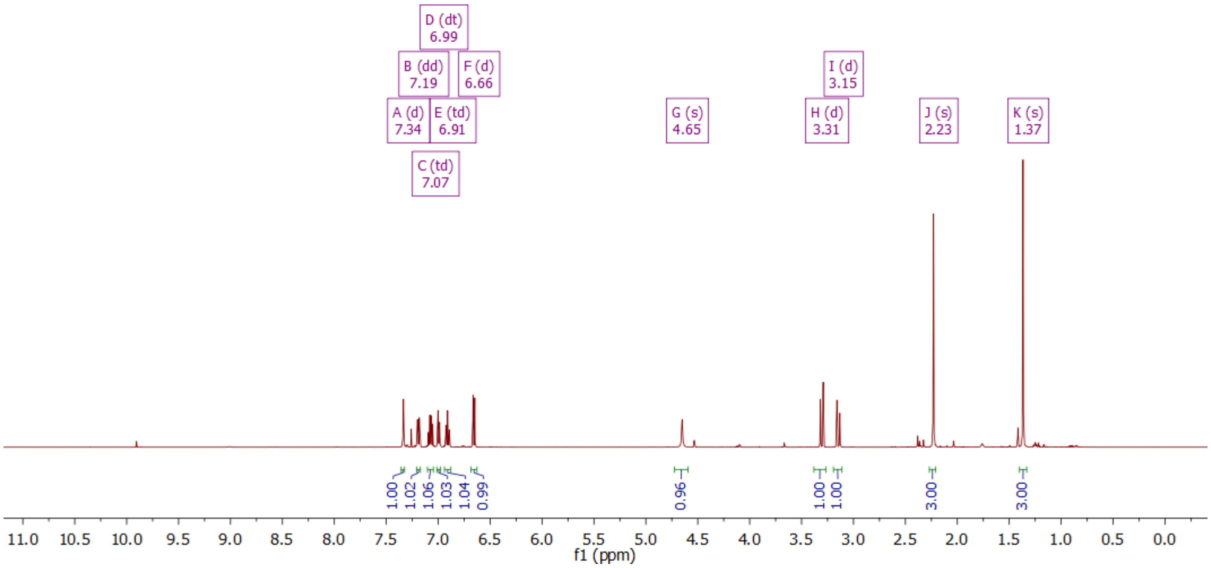

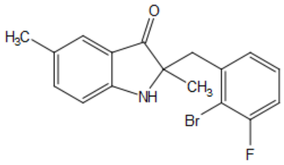

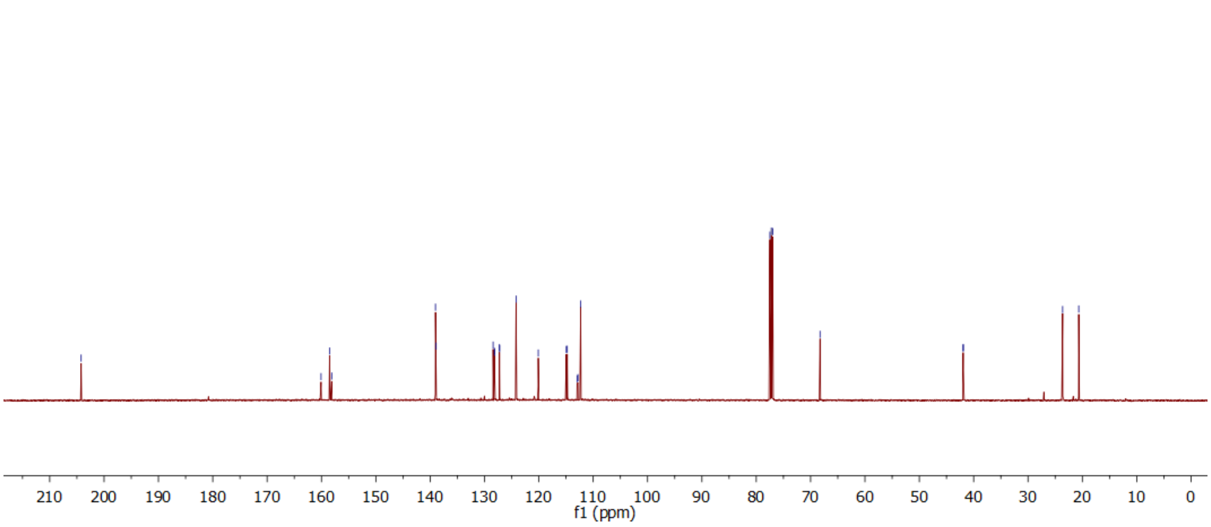

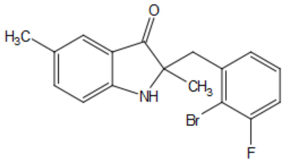
H3C
O
CH3
NH
Br
F

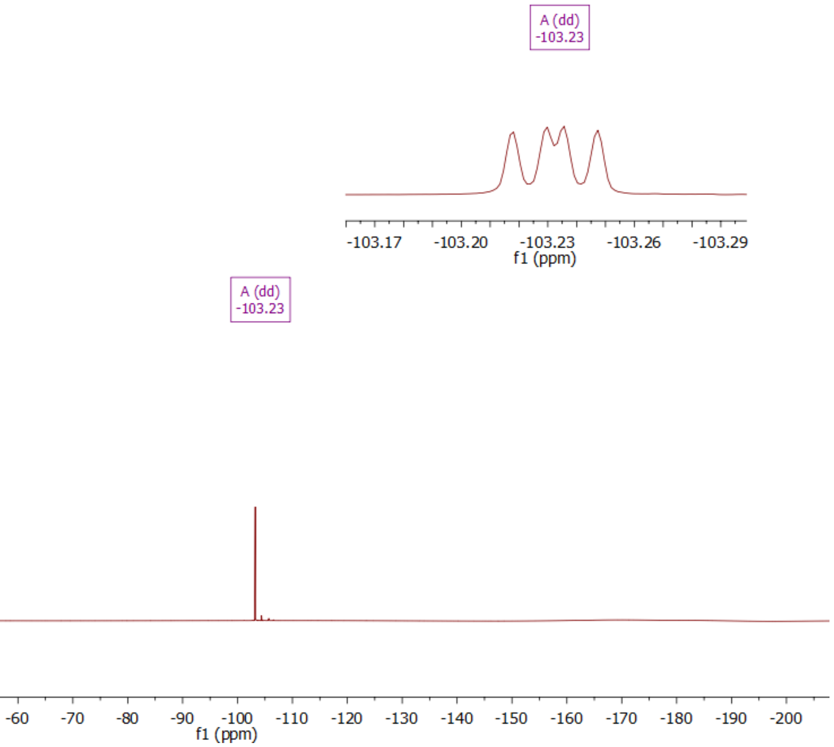
A (dd)
-103.23
-103.17
-103.20
-103.23
-103.26
-103.29
f1 (ppm)
A (dd)
-103.23
10
0
-10
-20
-30
-40
-50
-60
-70
-80
-90
-100
-110
-120
-130
-140
-150
-160
-170
-180
-190
-200
f1 (ppm)

2-(2-bromo-4-methoxybenzyl)-5-fluoro-2-methylindolin-3-one [**4m**]:

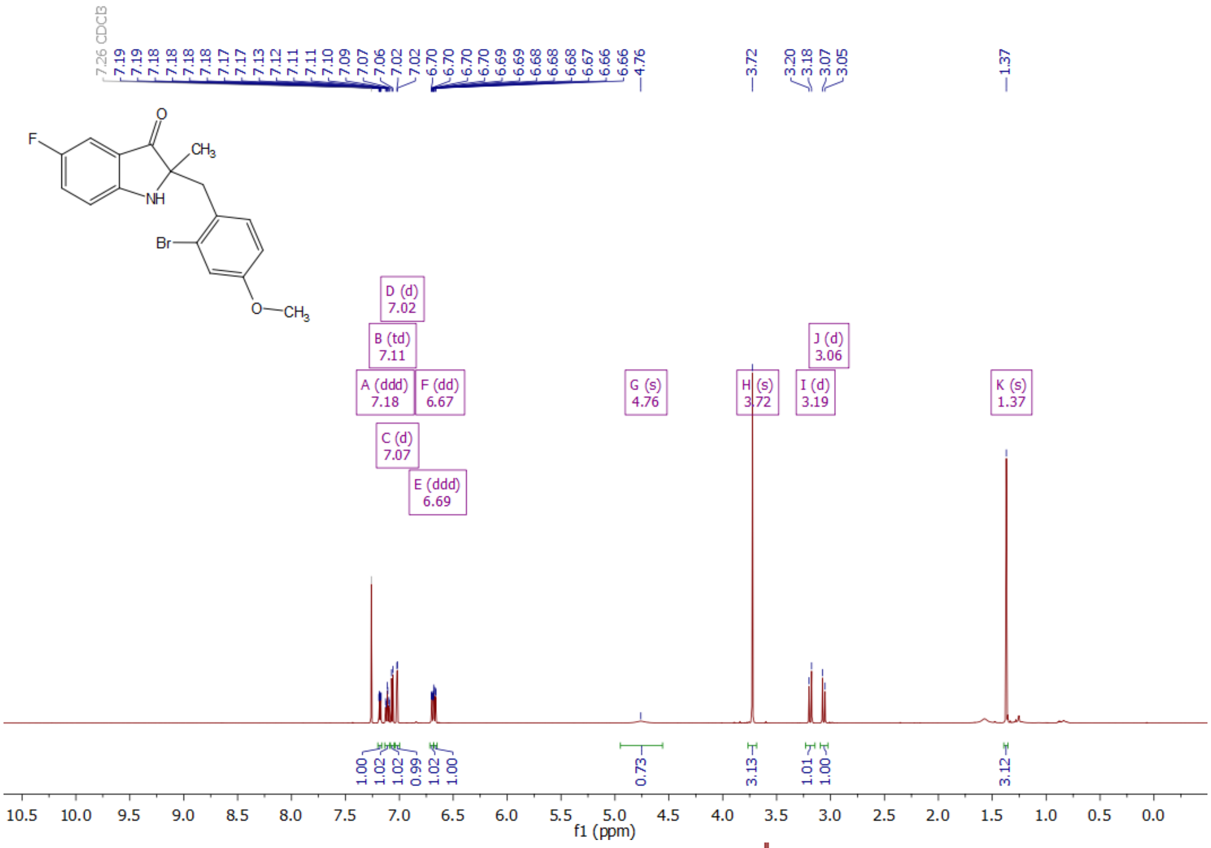

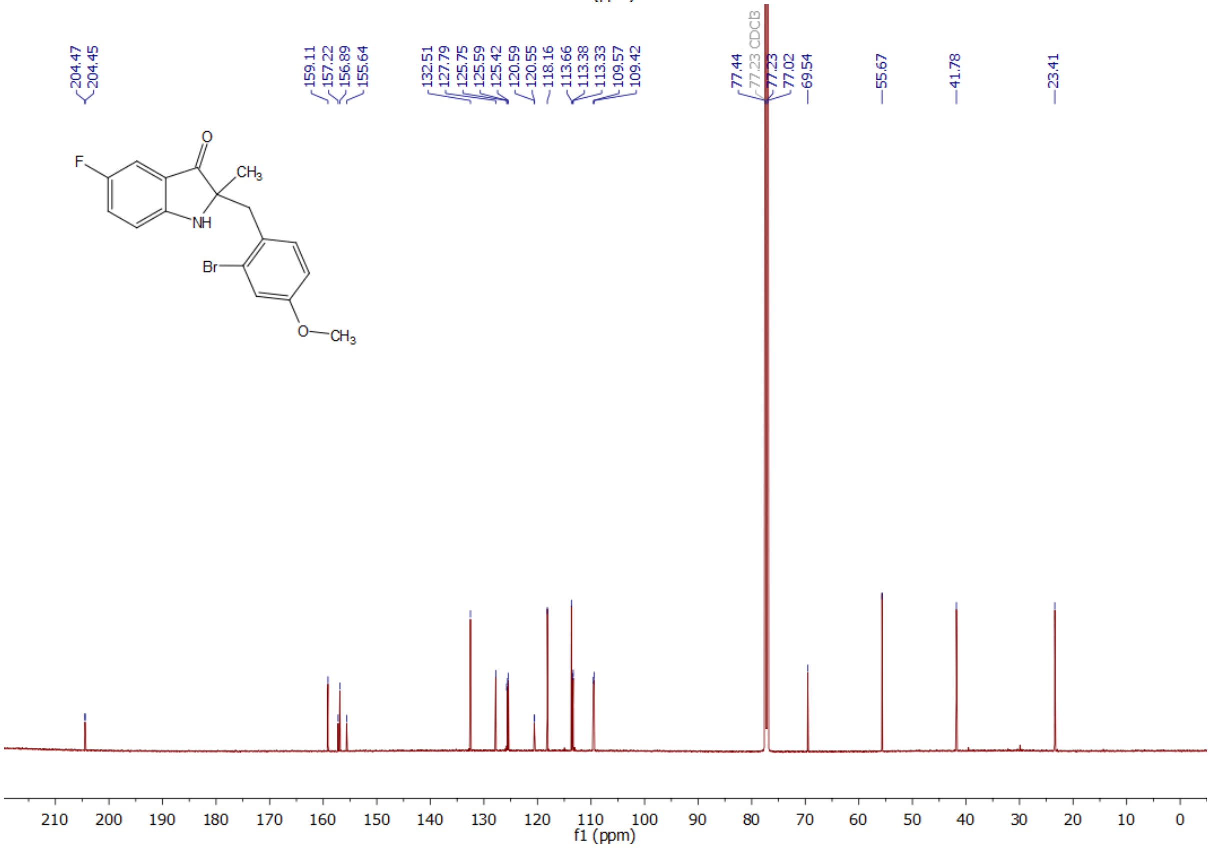

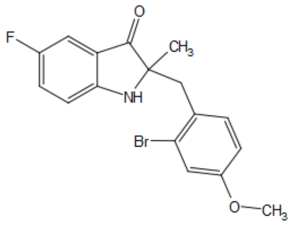
F
O
CH3
NH
Br
O
CH3

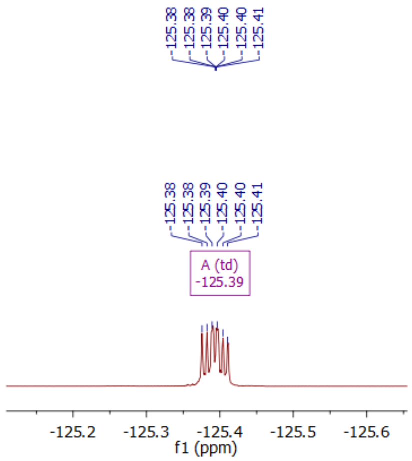
-125.38
-125.38
-125.39
-125.40
-125.40
-125.41
A (td)
-125.39
-125.2
-125.3
-125.4
-125.5
-125.6
f1 (ppm)

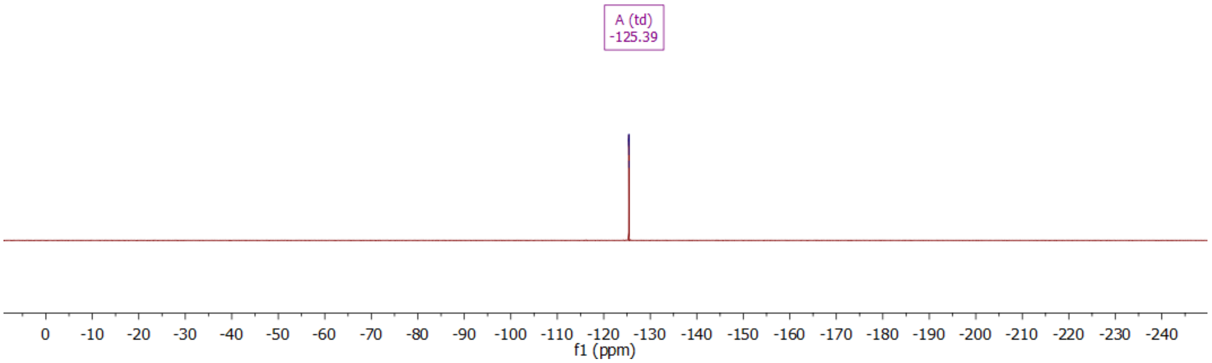
A (td)
-125.39
0 -10 -20 -30 -40 -50 -60 -70 -80 -90 -100 -110 -120 -130 -140 -150 -160 -170 -180 -190 -200 -210 -220 -230 -240
f1 (ppm)

2-(2-bromo-4-methylbenzyl)-5-chloro-2-methylindolin-3-one [**4n**]:

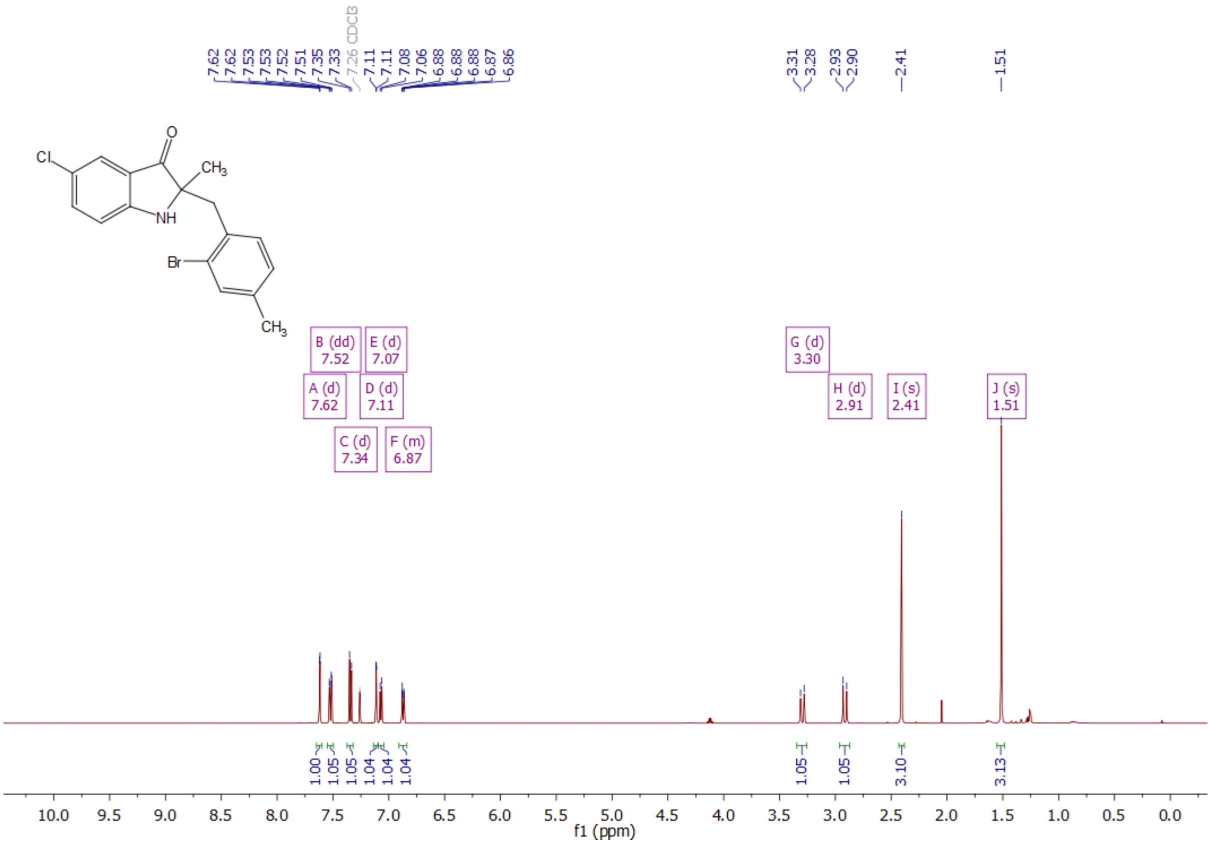

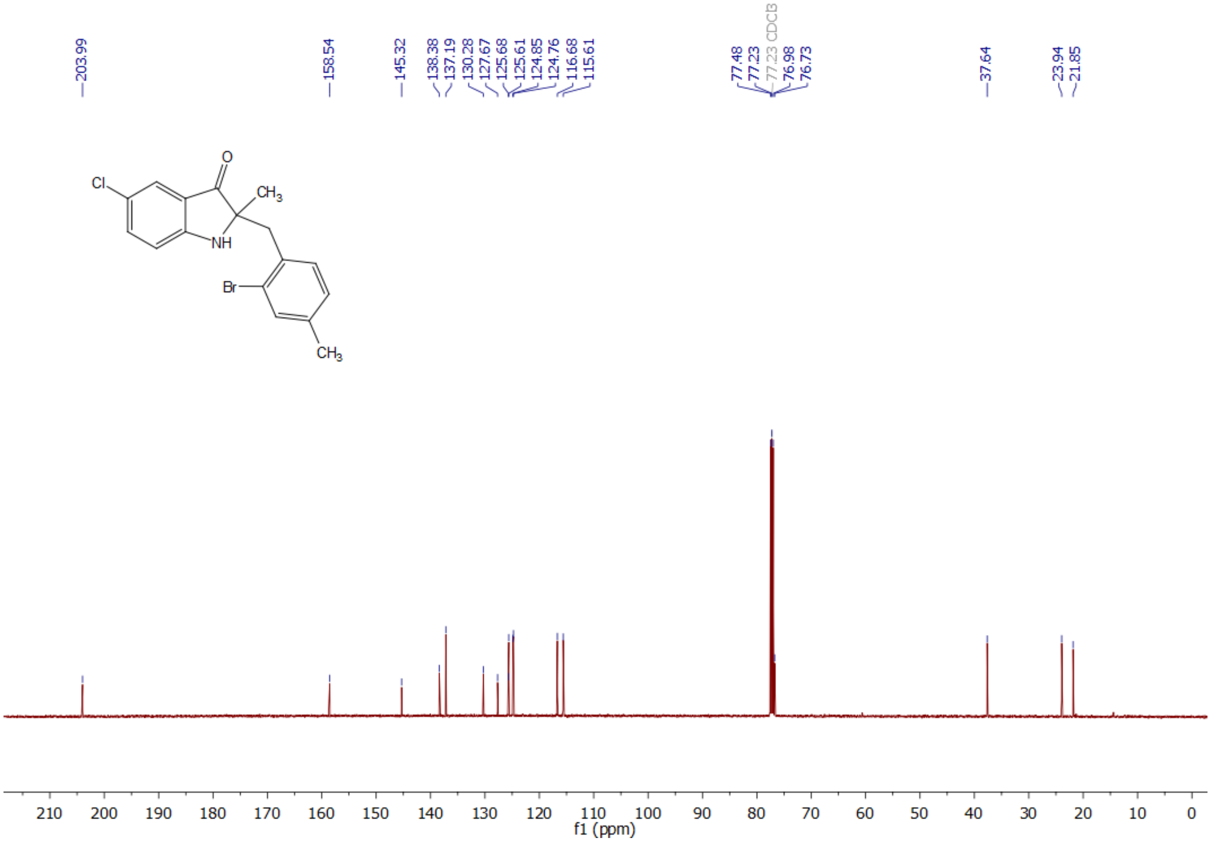

2-(2-bromo-4-chlorobenzyl)-2,5-dimethylindolin-3-one [**4o**]:

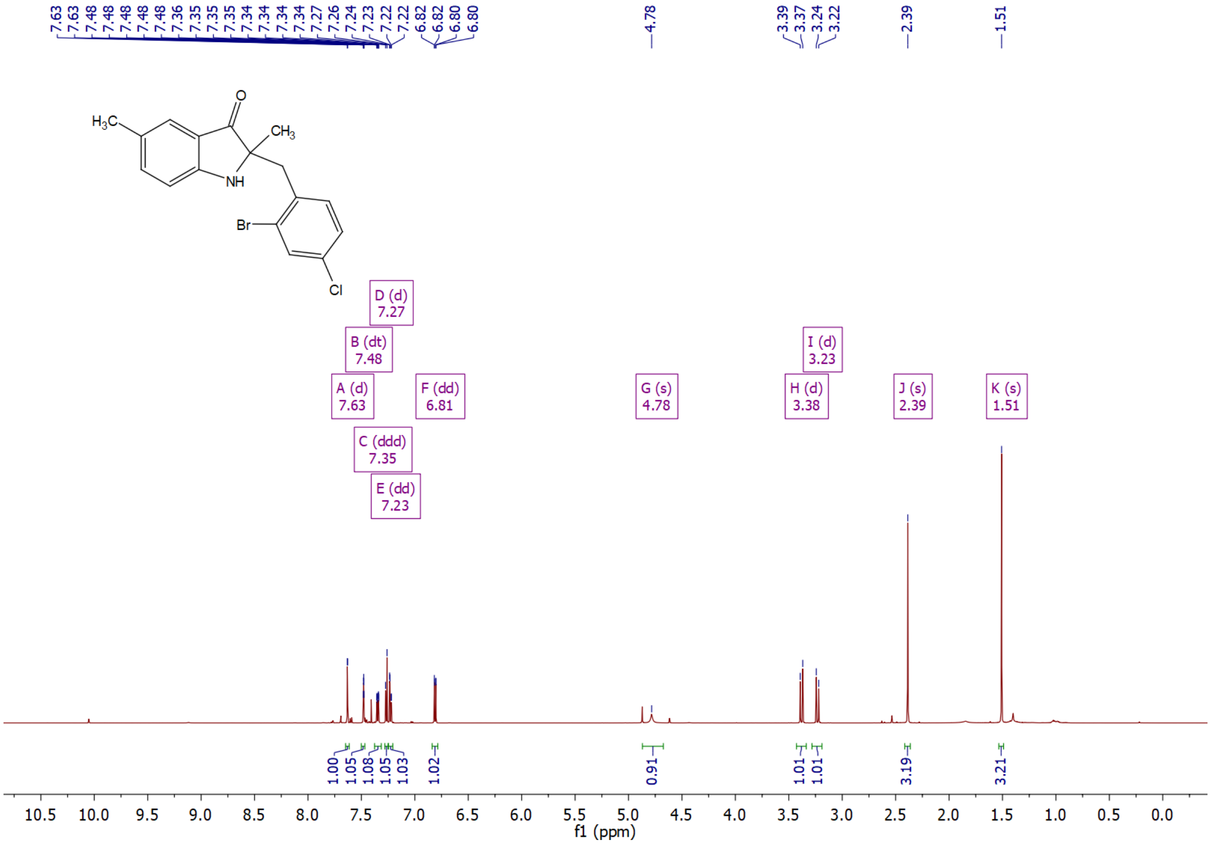

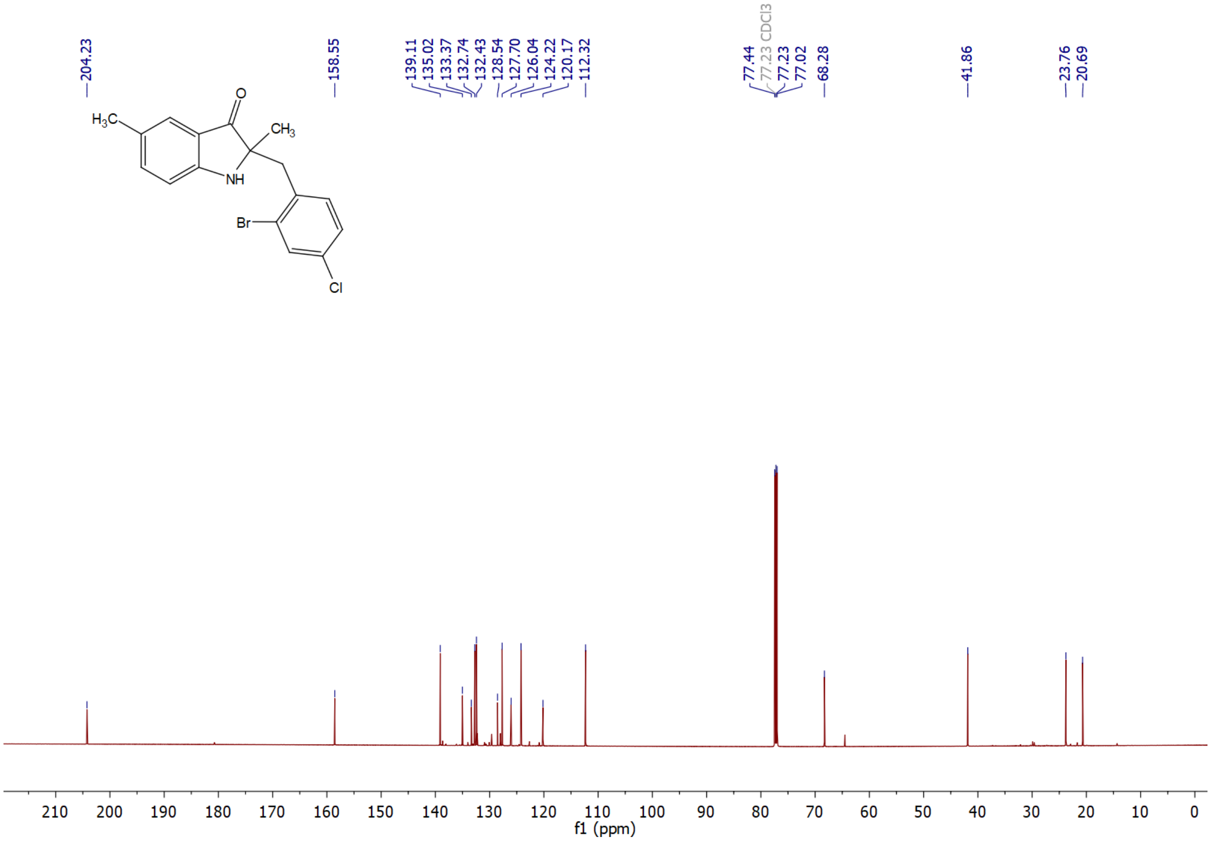

2-(2-bromo-5-fluorobenzyl)-5-chloro-2-methylindolin-3-one [**4p**]:

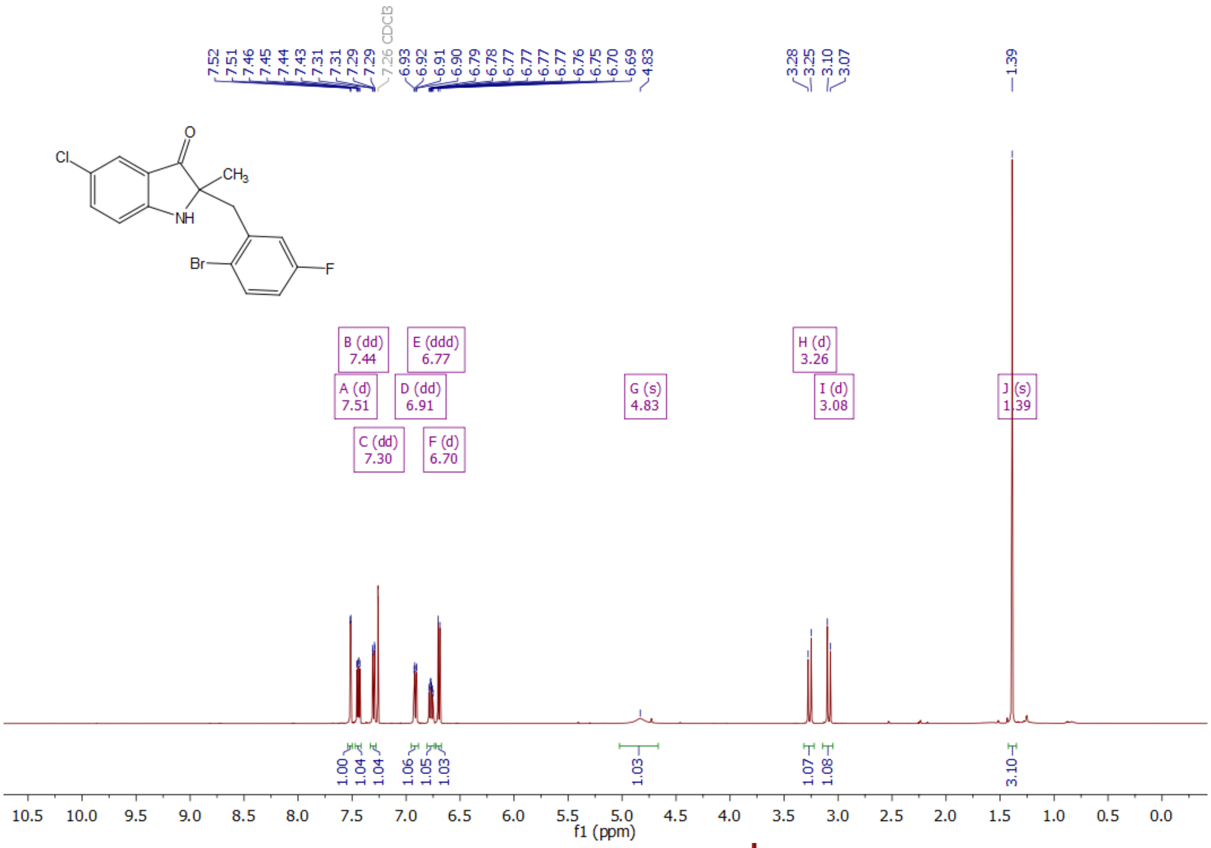

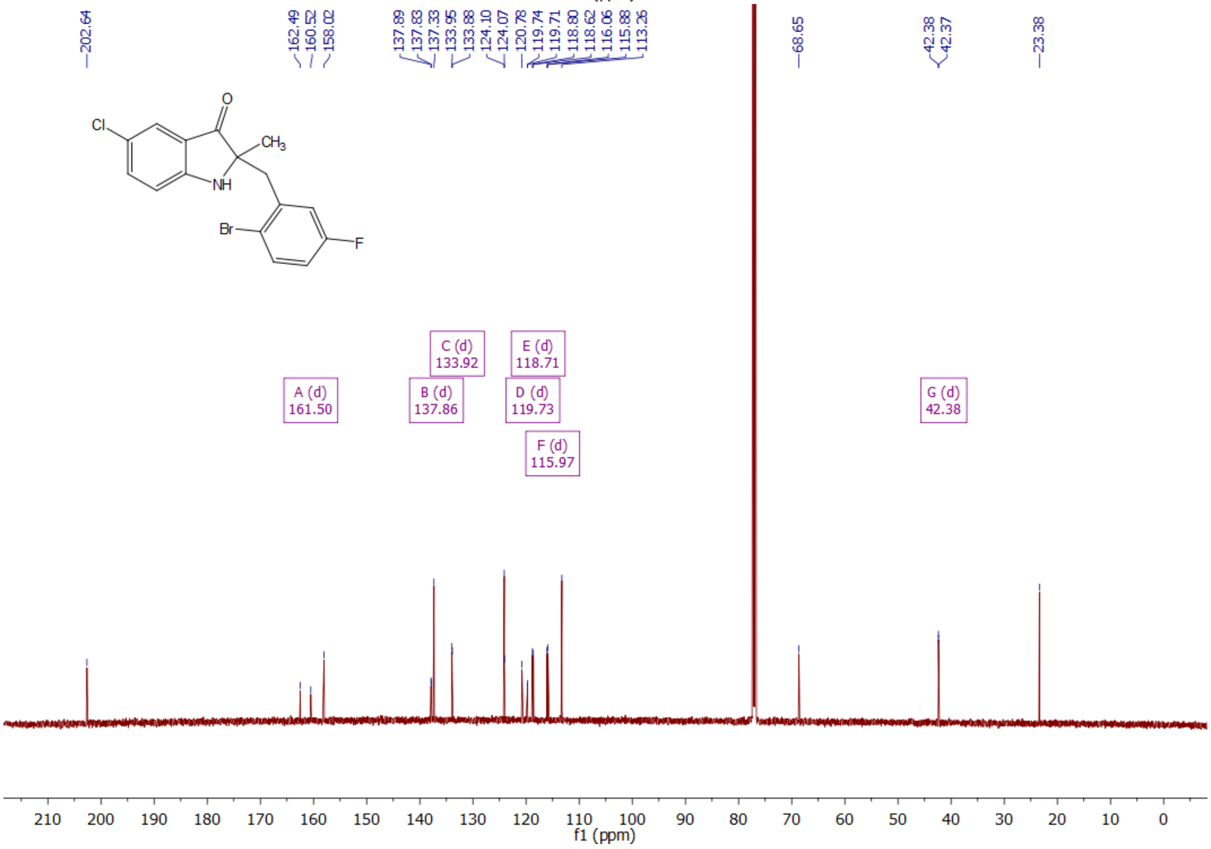

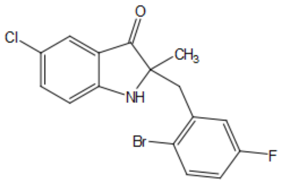

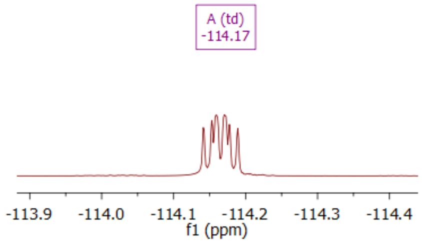
A (td)
-114.17
-113.9
-114.0
-114.1
-114.2
-114.3
-114.4
f1 (ppm)

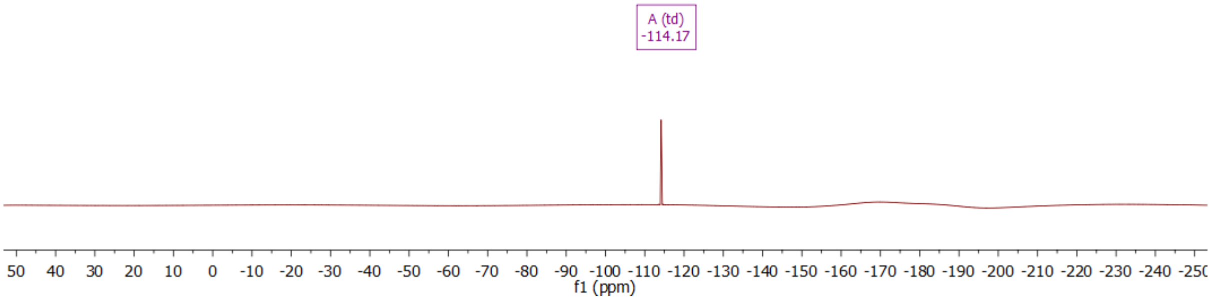
A (td)
-114.17
50 40 30 20 10 0 -10 -20 -30 -40 -50 -60 -70 -80 -90 -100 -110 -120 -130 -140 -150 -160 -170 -180 -190 -200 -210 -220 -230 -240 -250
f1 (ppm)

2-((6-bromobenzo[d][1,3]dioxol-5-yl)methyl)-2,5-dimethylindolin-3-one [**4q**]:

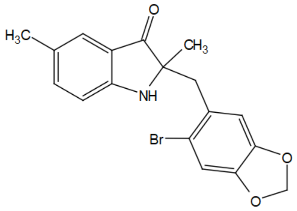

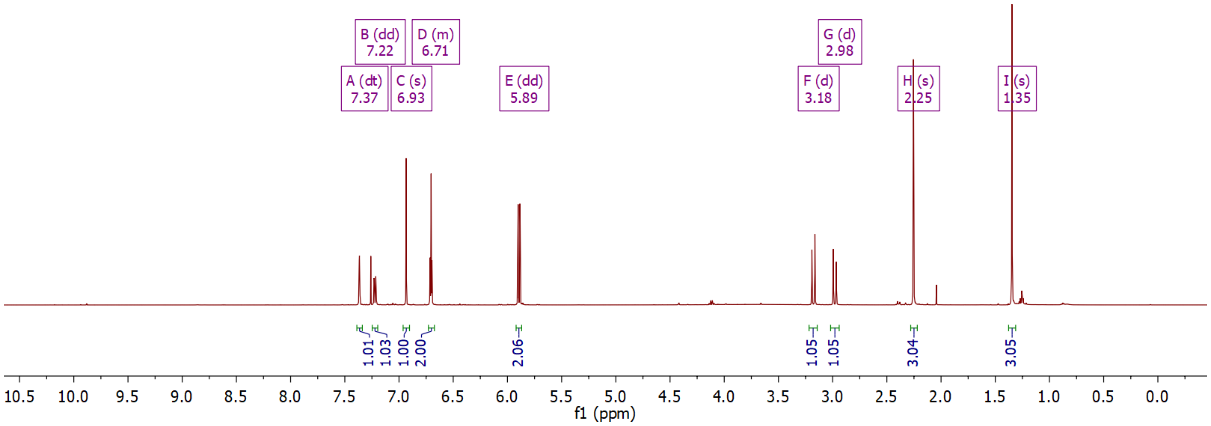

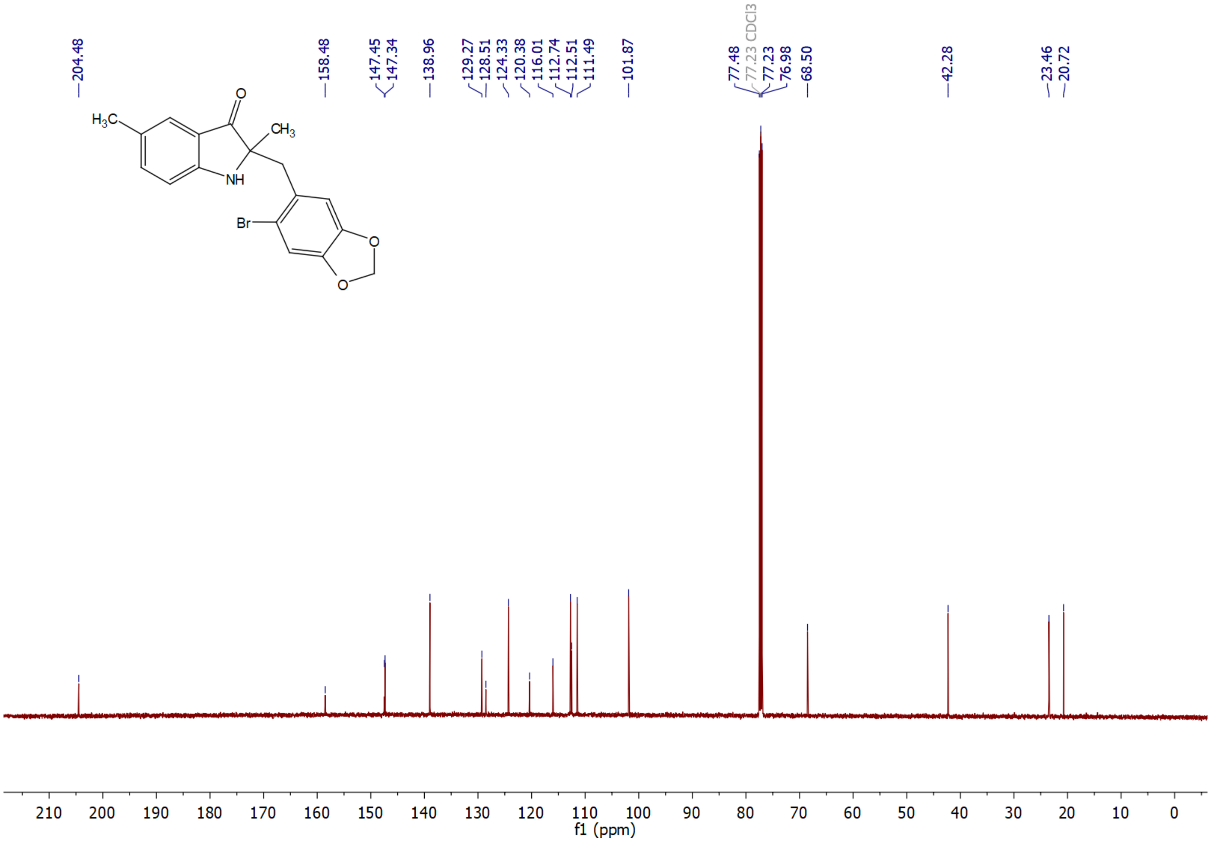

***2-(2-bromobenzyl)-2-phenylindolin-3-one* [4r]:**

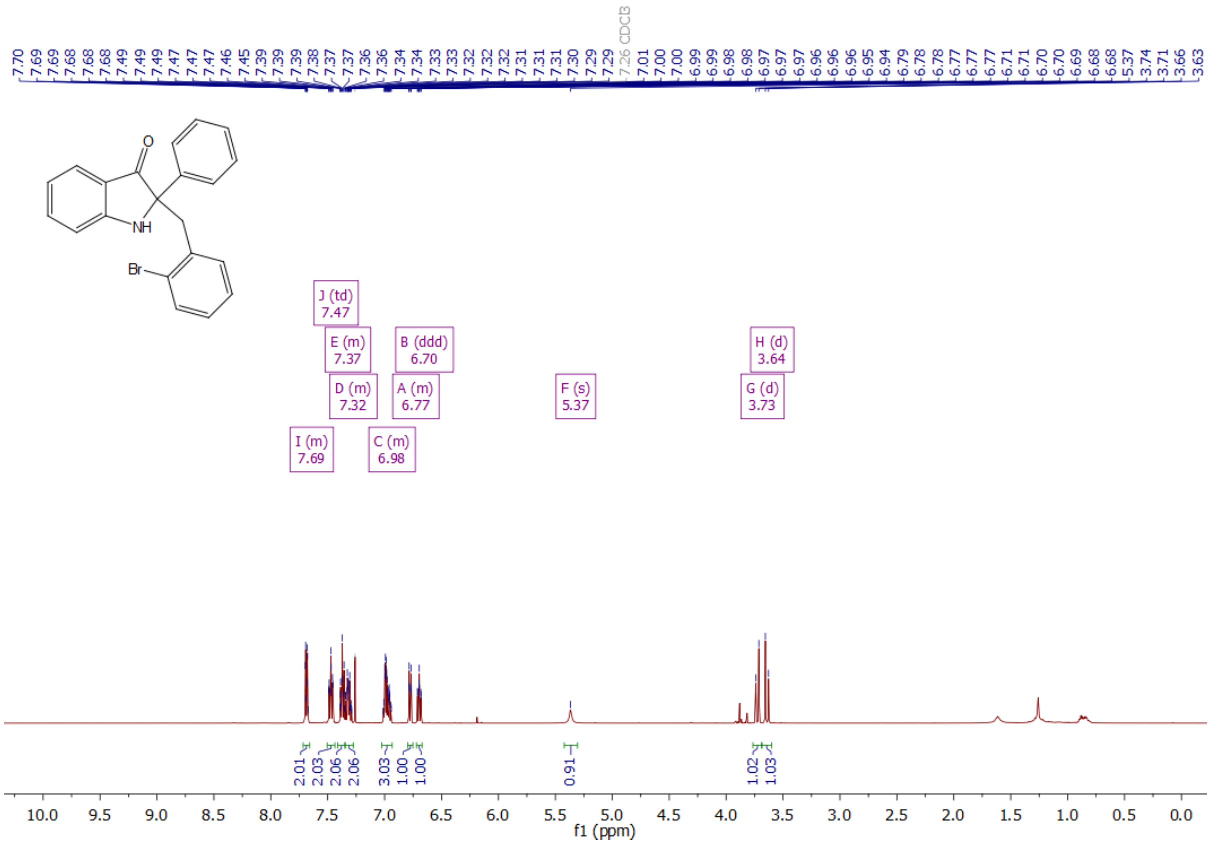

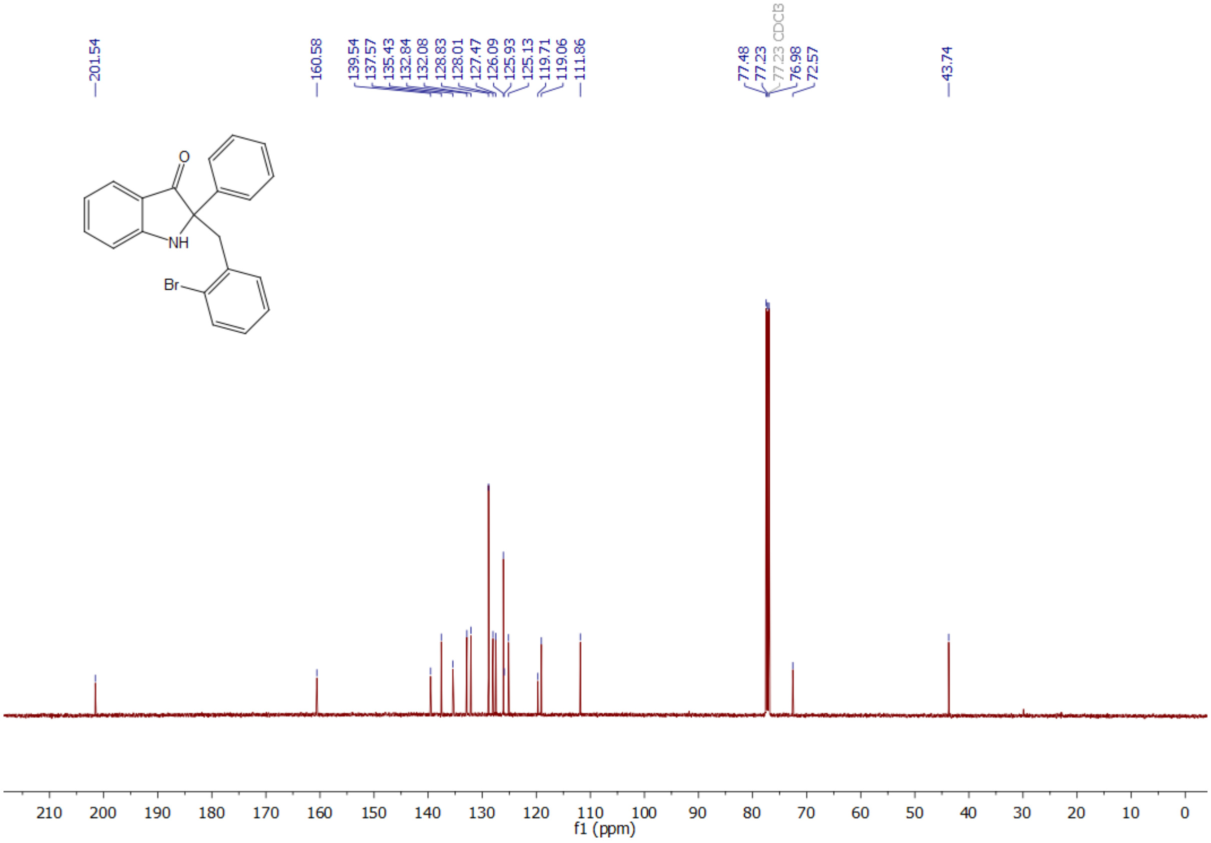

2-(2-bromo-4-fluorobenzyl)-2-phenylindolin-3-one [**4s**]:

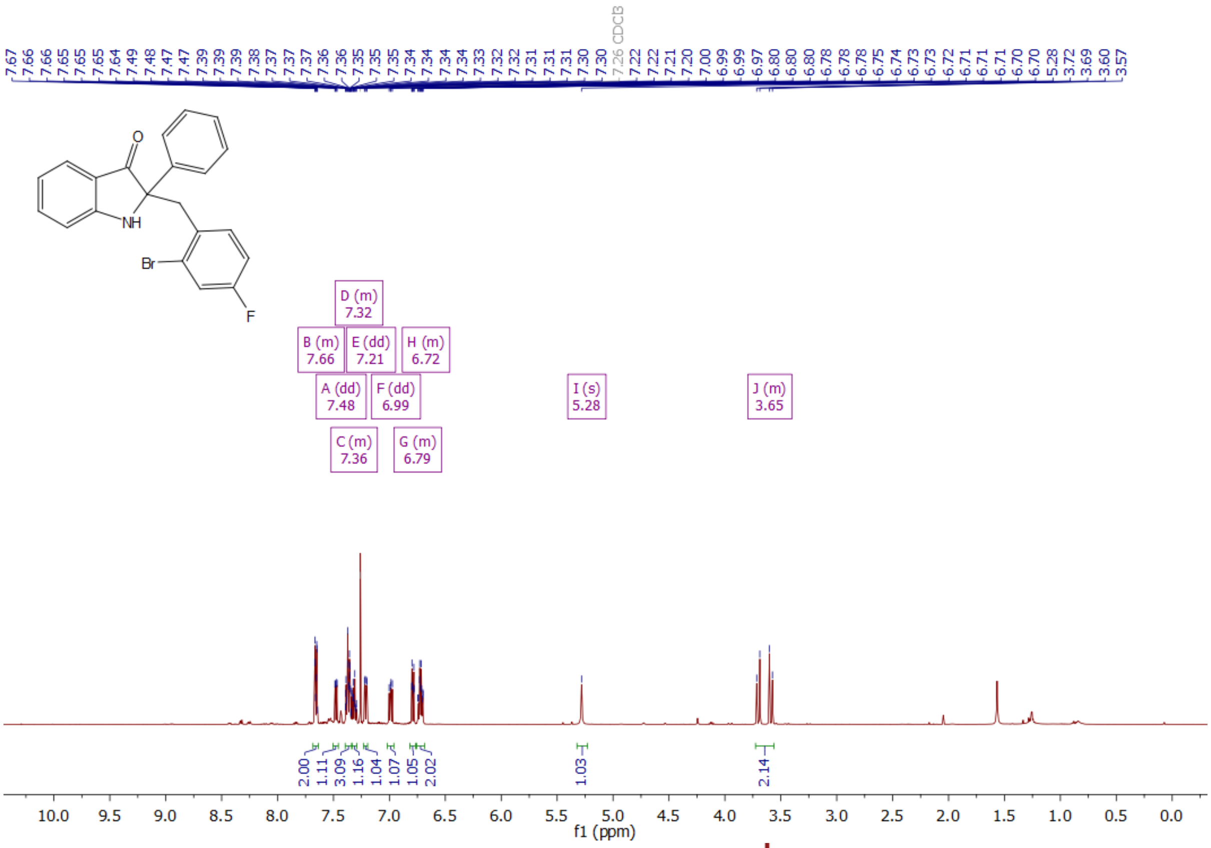

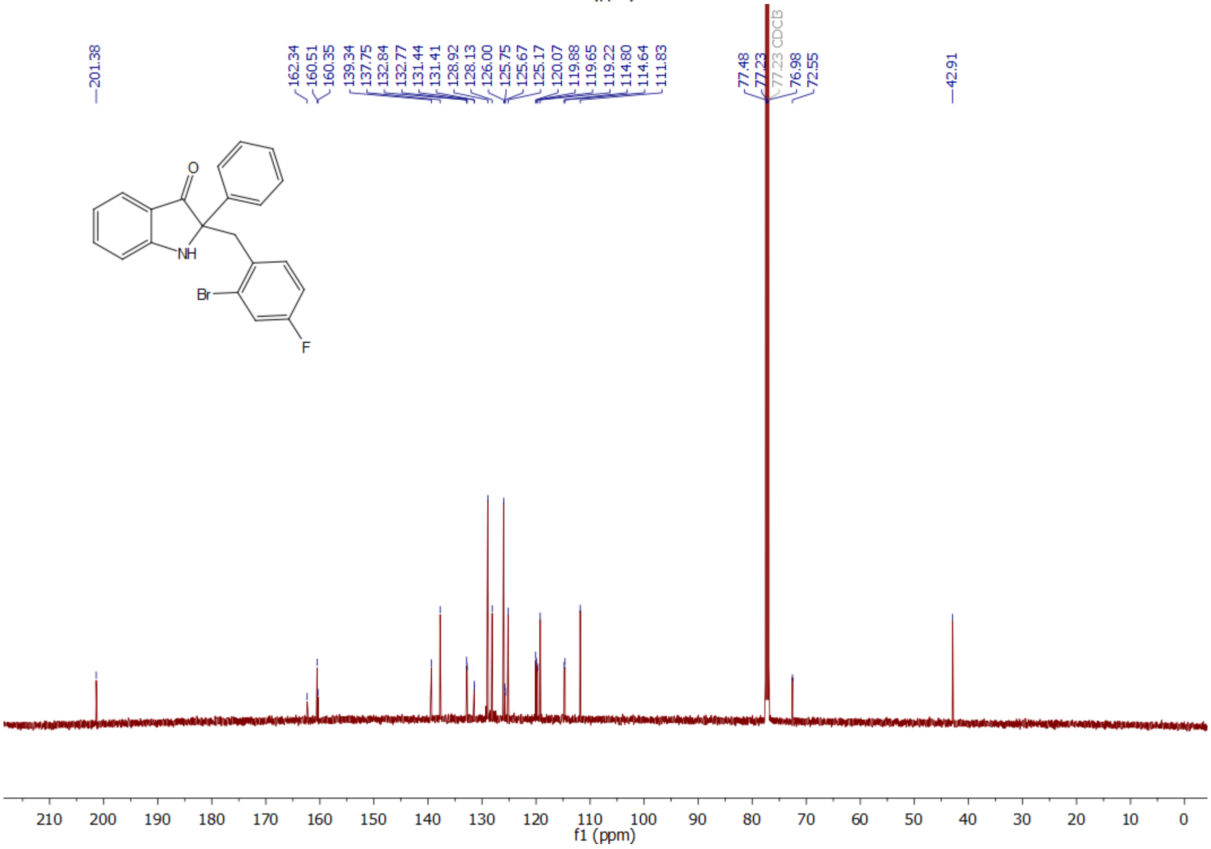

2-(2-bromo-4-chlorobenzyl)-2-phenylindolin-3-one [**4t**]:

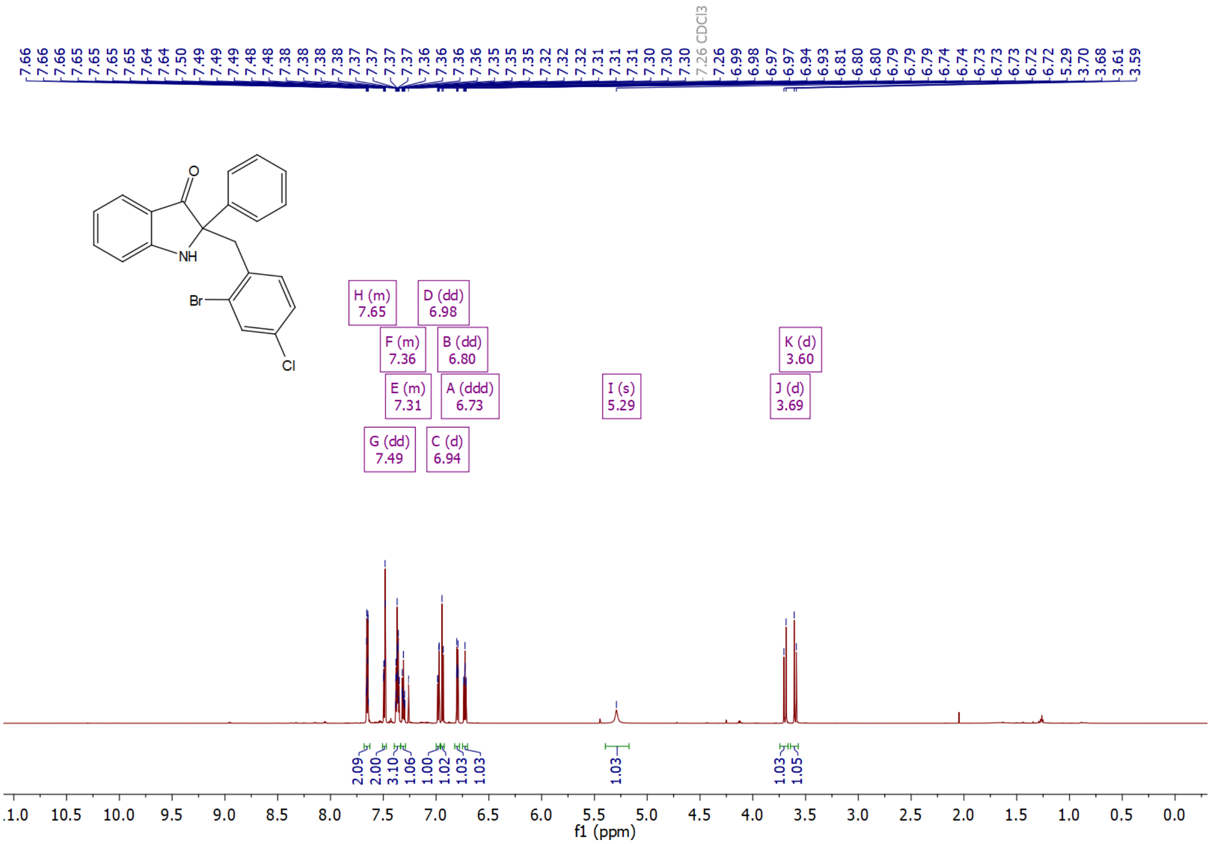

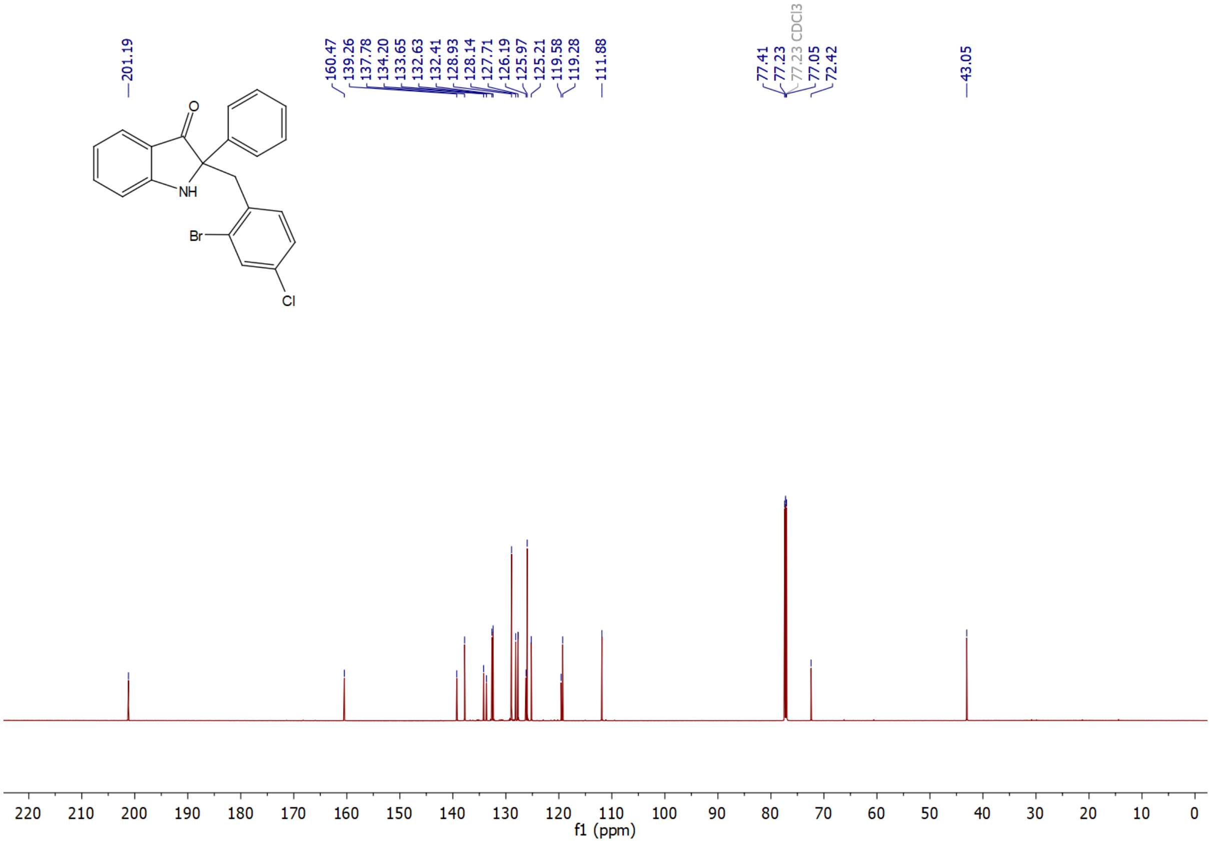

2-(2-bromo-5-fluorobenzyl)-2-phenylindolin-3-one [**4u**]:

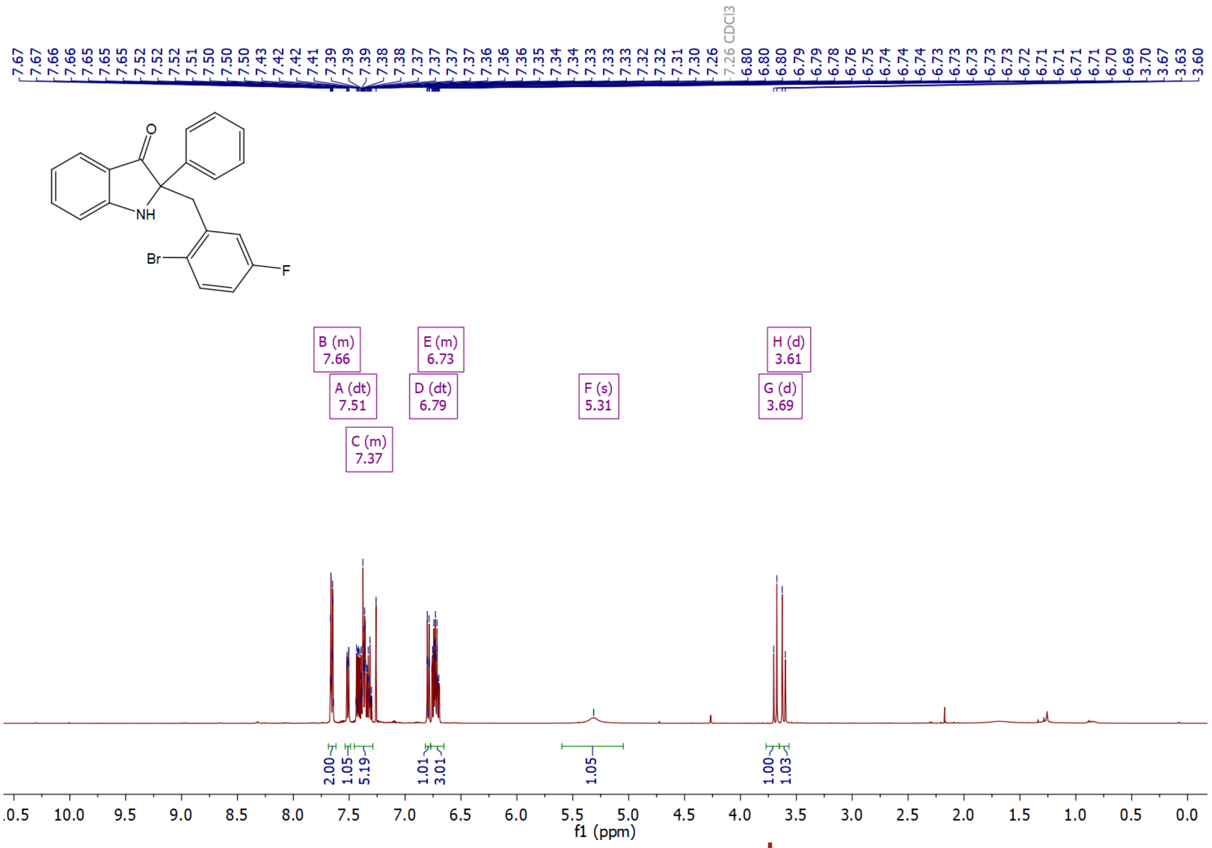

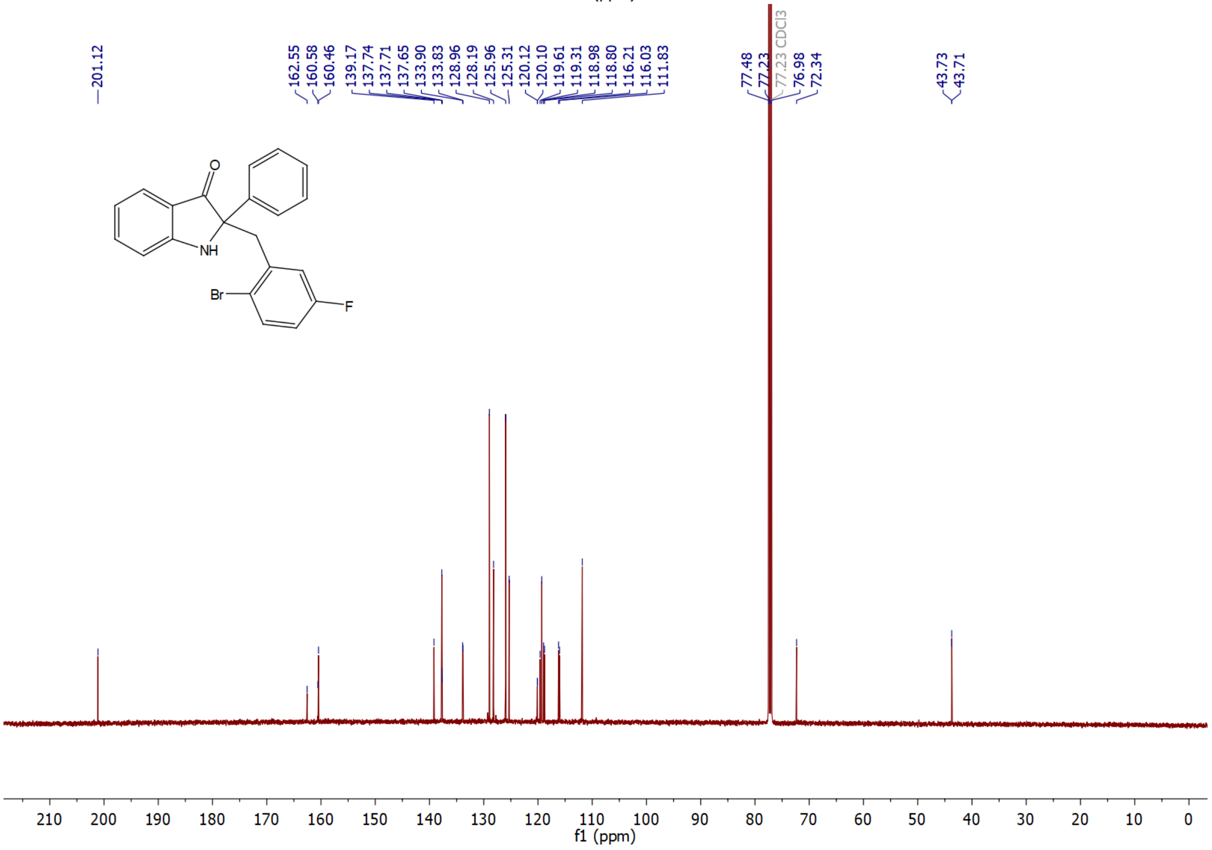

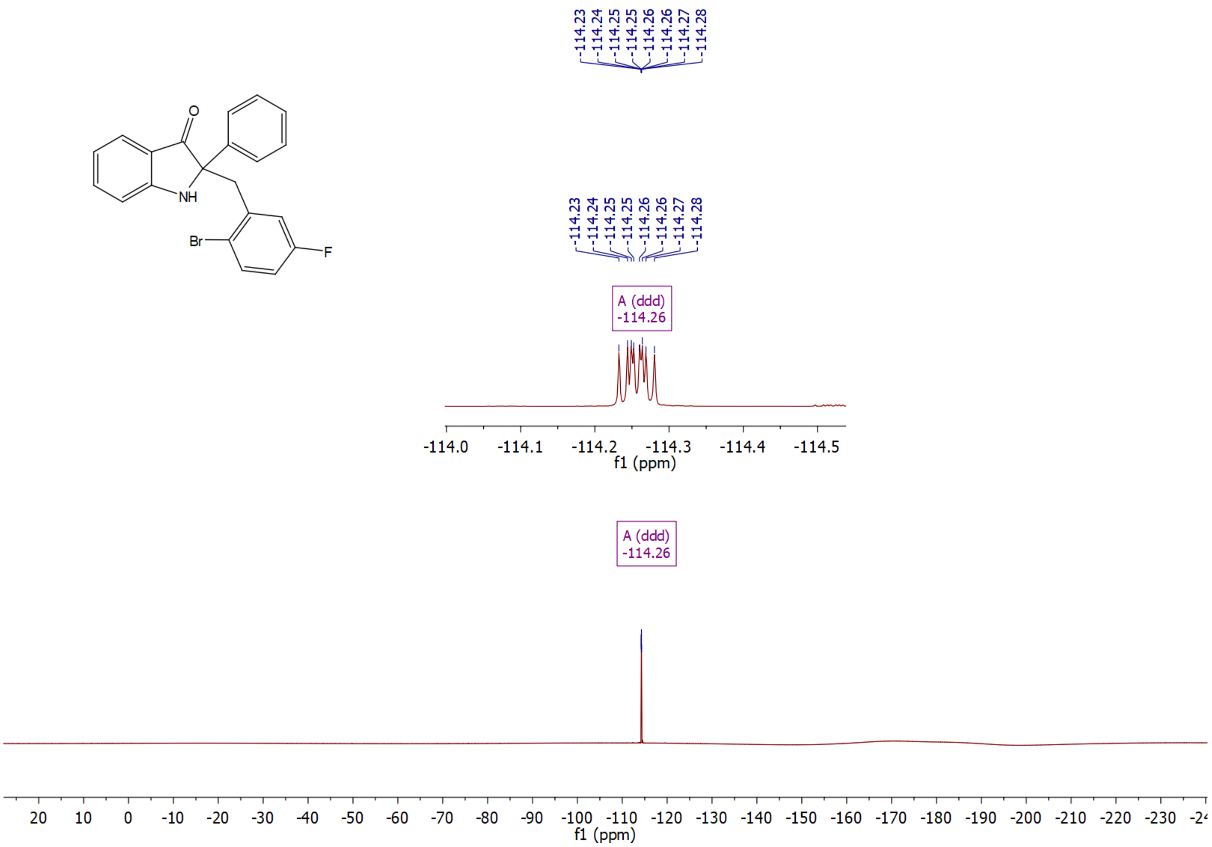
O
NH
Br
F
-114.23
-114.24
-114.25
-114.25
-114.26
-114.26
-114.27
-114.28
A (ddd)
-114.26
-114.0
-114.1
-114.2
-114.3
-114.4
-114.5
f1 (ppm)
20
10
0
-10
-20
-30
-40
-50
-60
-70
-80
-90
-100
-110
-120
-130
-140
-150
-160
-170
-180
-190
-200
-210
-220
-230
f1 (ppm)

***2-(2-bromo-5-fluorobenzyl)-2-(4-chlorophenyl)indolin-3-one* [4v]:**

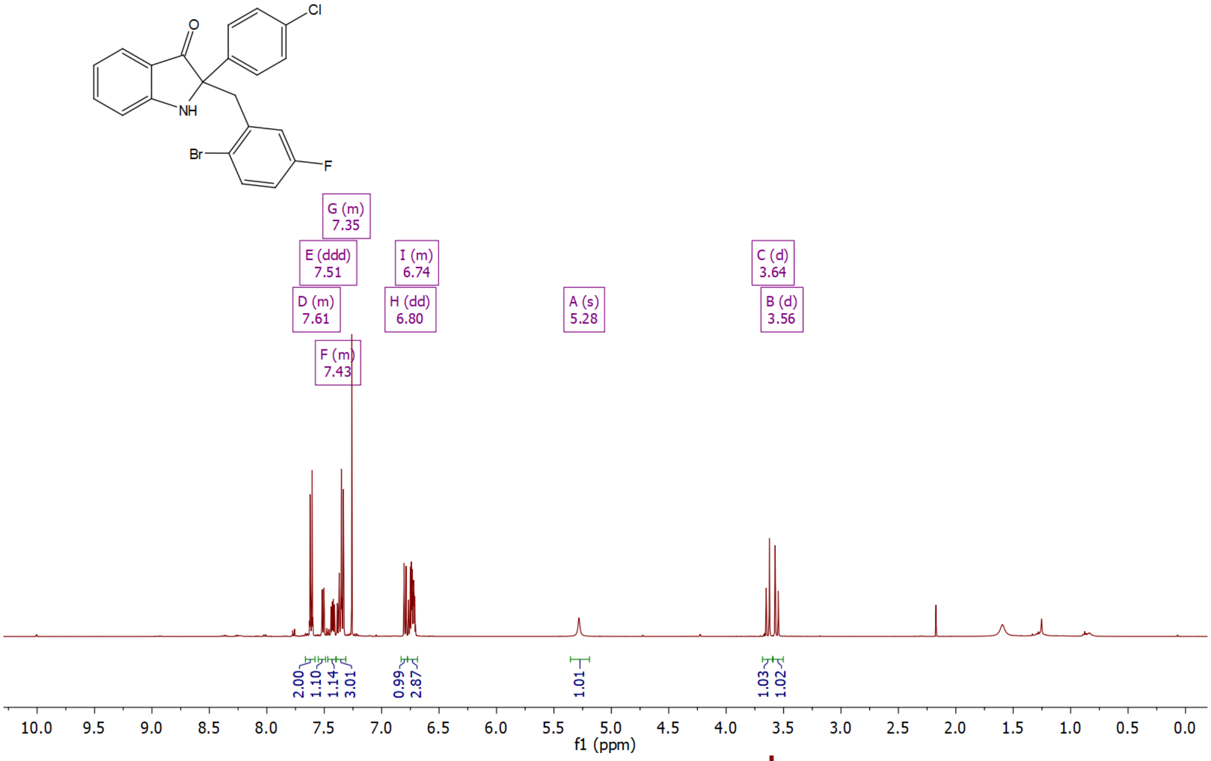

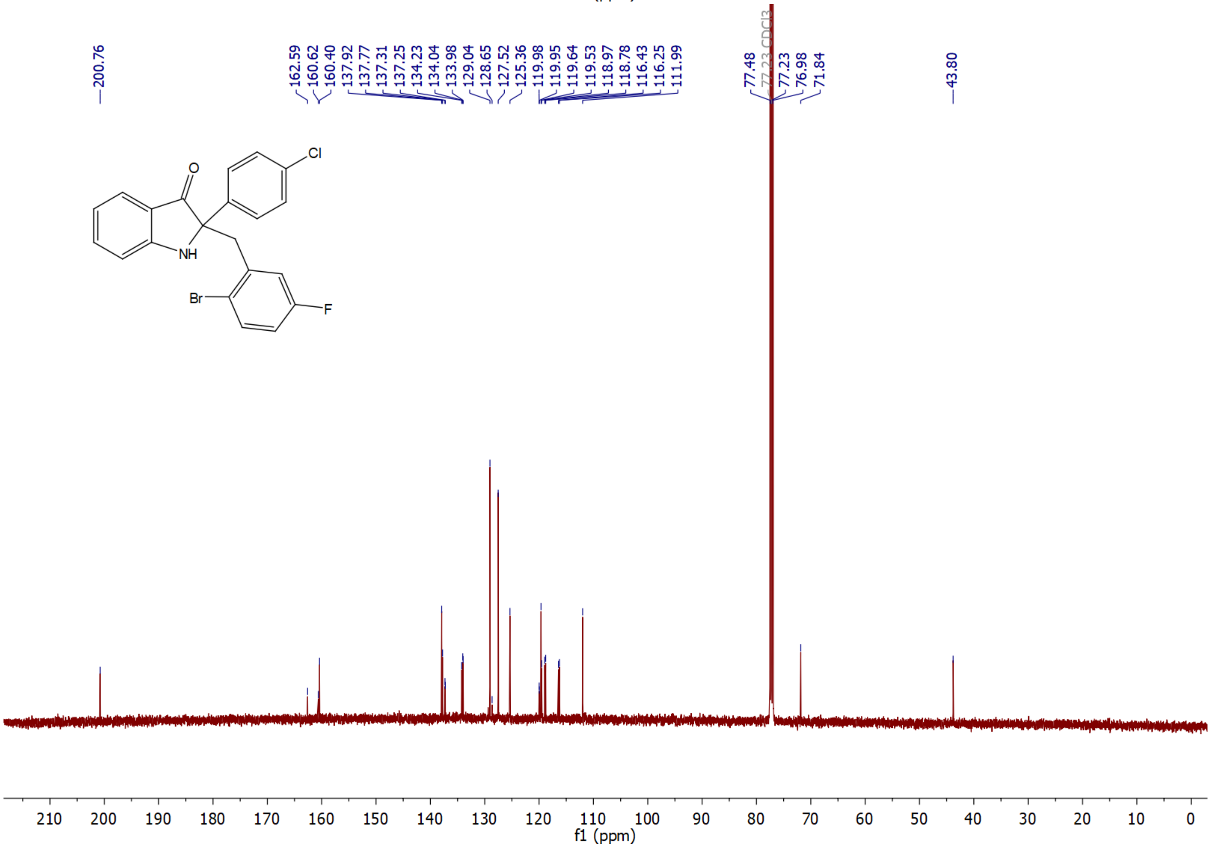

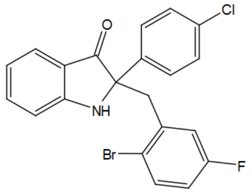
O
Cl
NH
Br
F

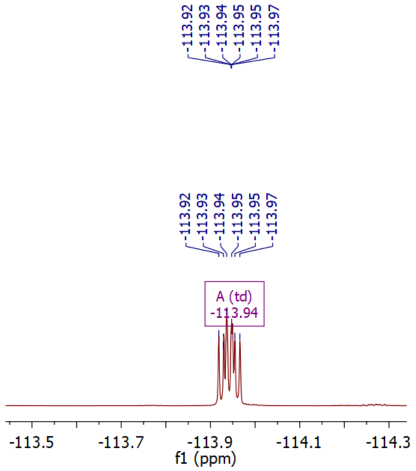
-113.92
-113.93
-113.94
-113.95
-113.95
-113.97
A (td)
-113.94
-113.5
-113.7
-113.9
-114.1
-114.3
f1 (ppm)

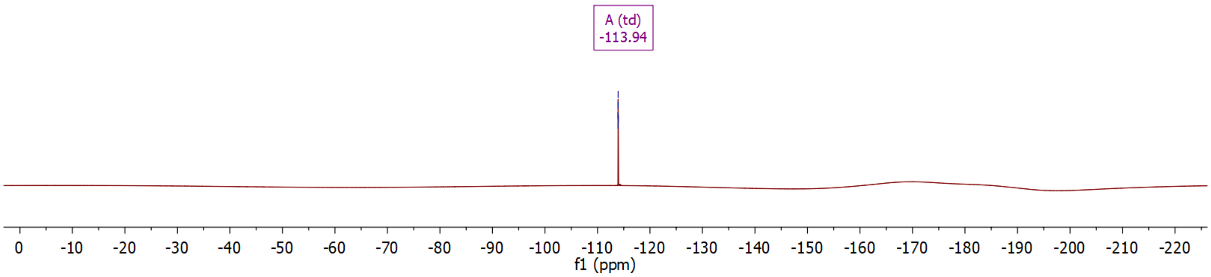
A (td)
-113.94
0
-10
-20
-30
-40
-50
-60
-70
-80
-90
-100
-110
-120
-130
-140
-150
-160
-170
-180
-190
-200
-210
-220
f1 (ppm)

2-(2-bromo-5-methoxybenzyl)-2-(4-chlorophenyl)indolin-3-one [**4w**]:

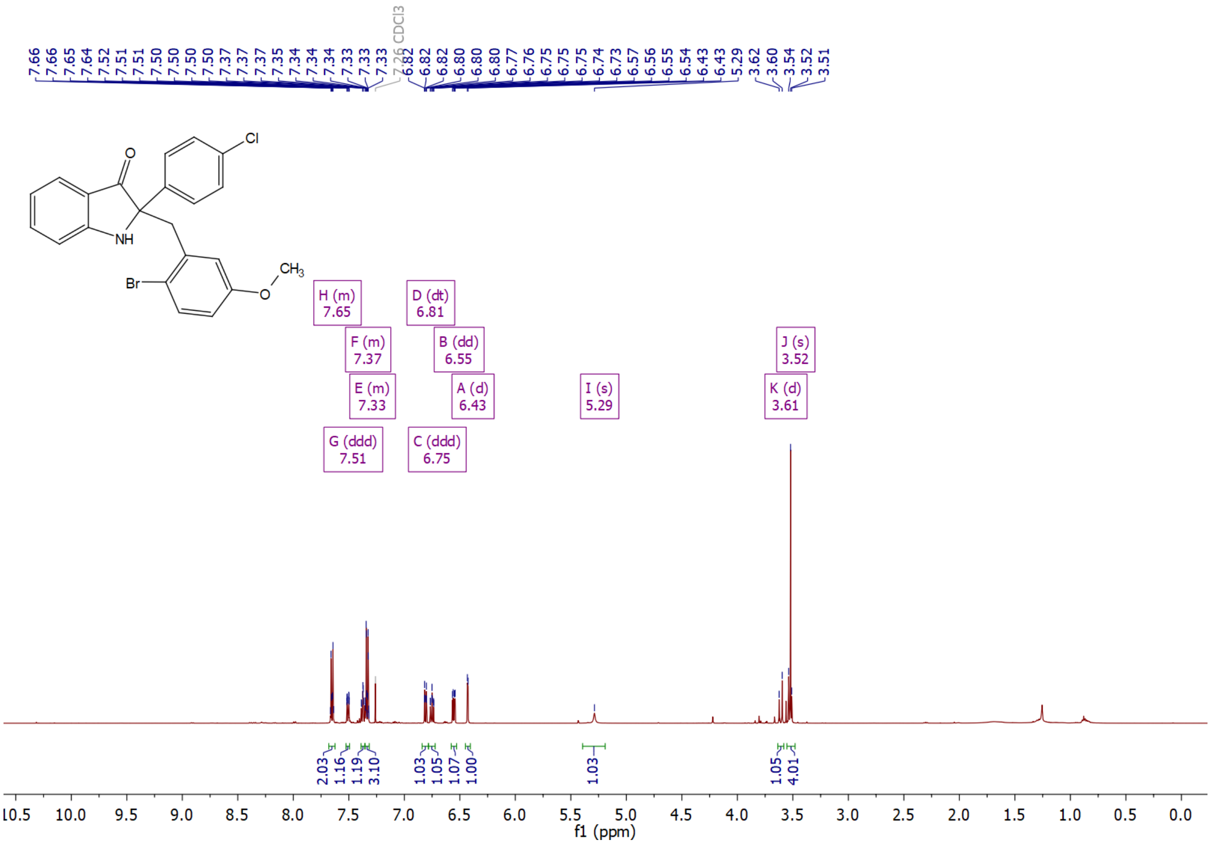

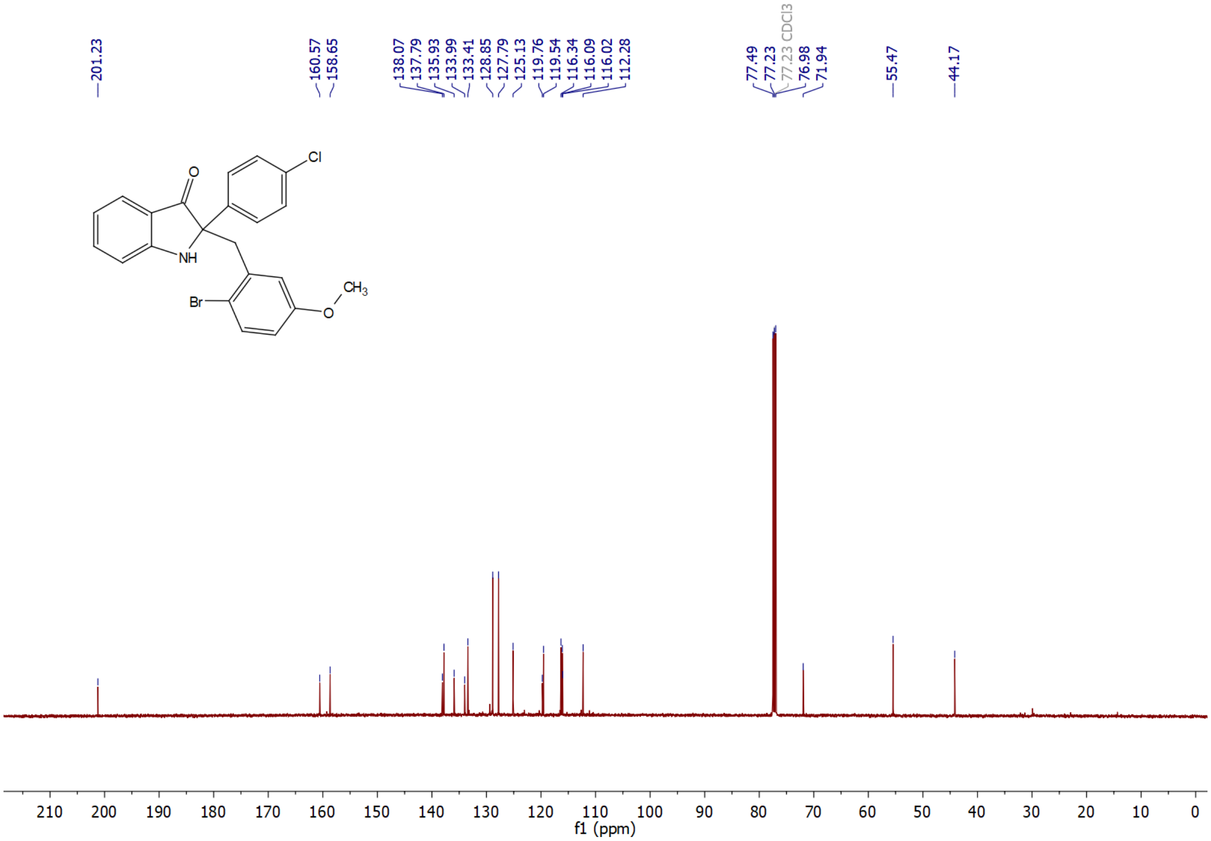

CLASS-F

10a-methyl-10a,11-dihydro-10H-indolo[1,2-a]indol-10-one **[F1]**:

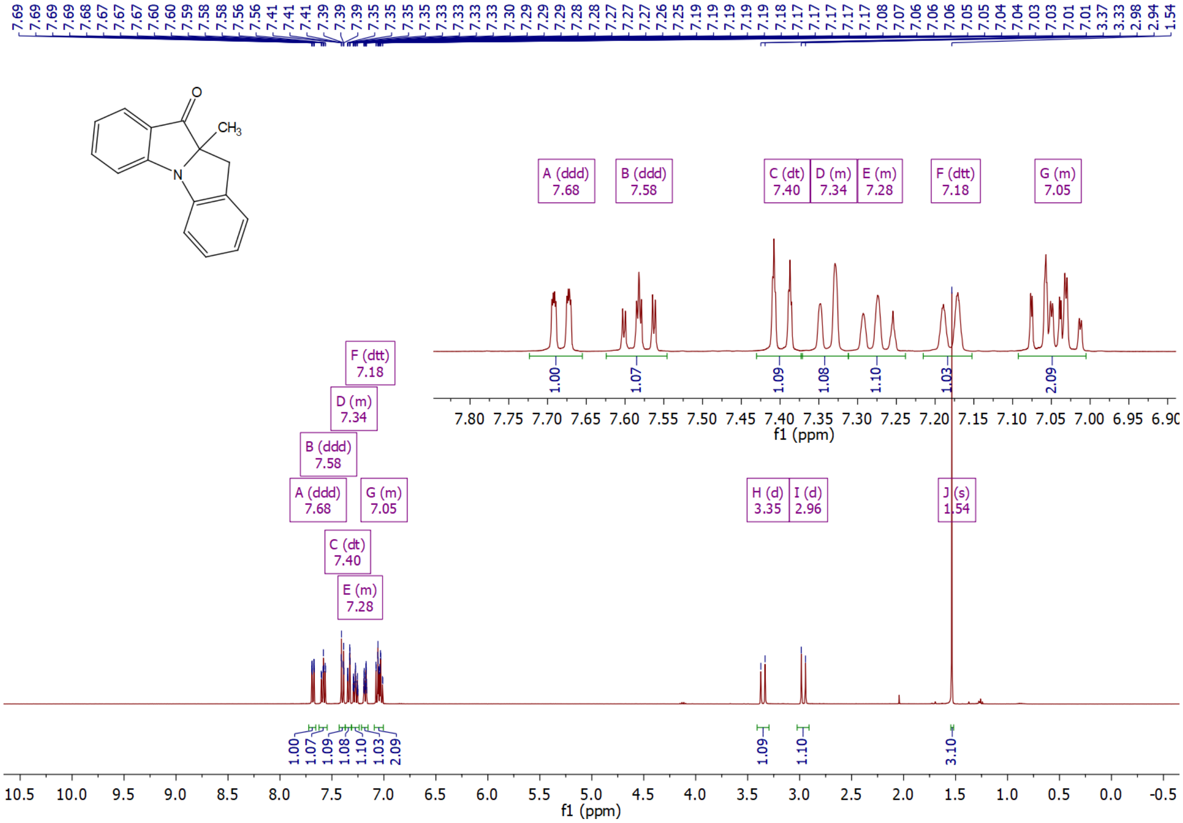

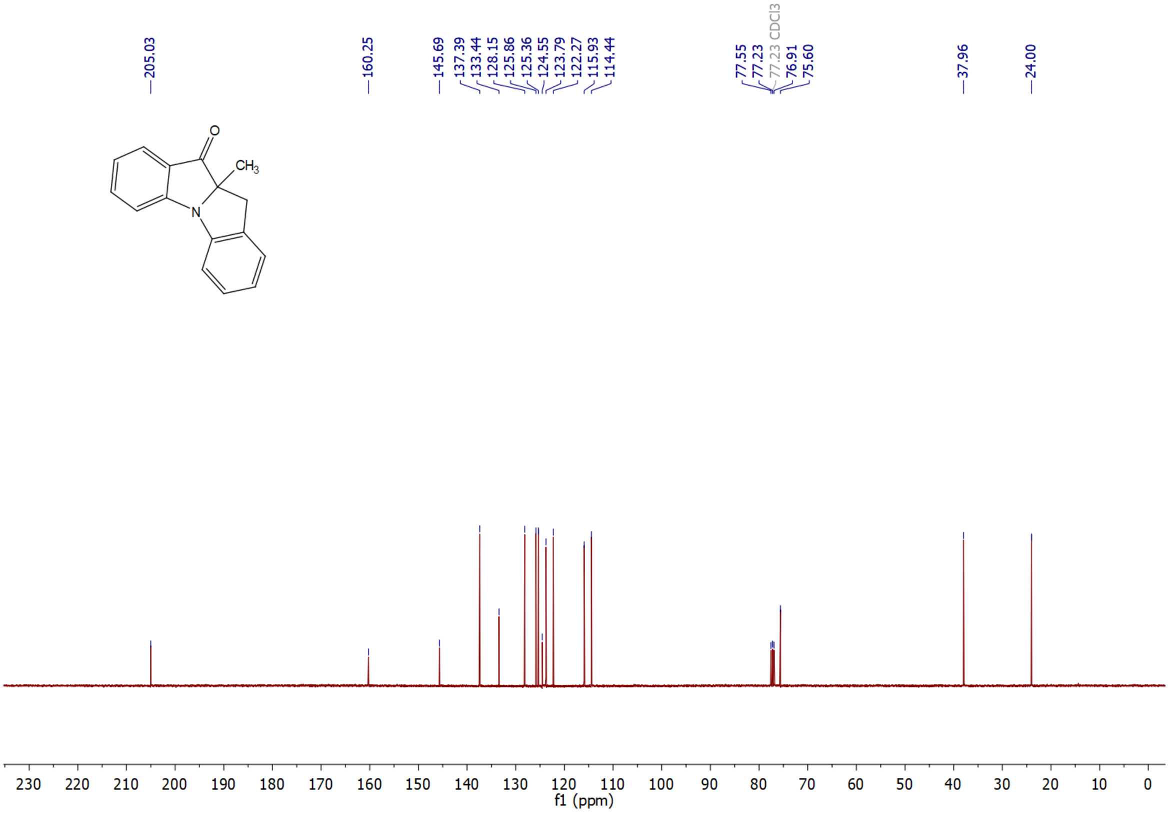

4-fluoro-10a-methyl-10a,11-dihydro-10H-indolo[1,2-a]indol-10-one **[F2]**:

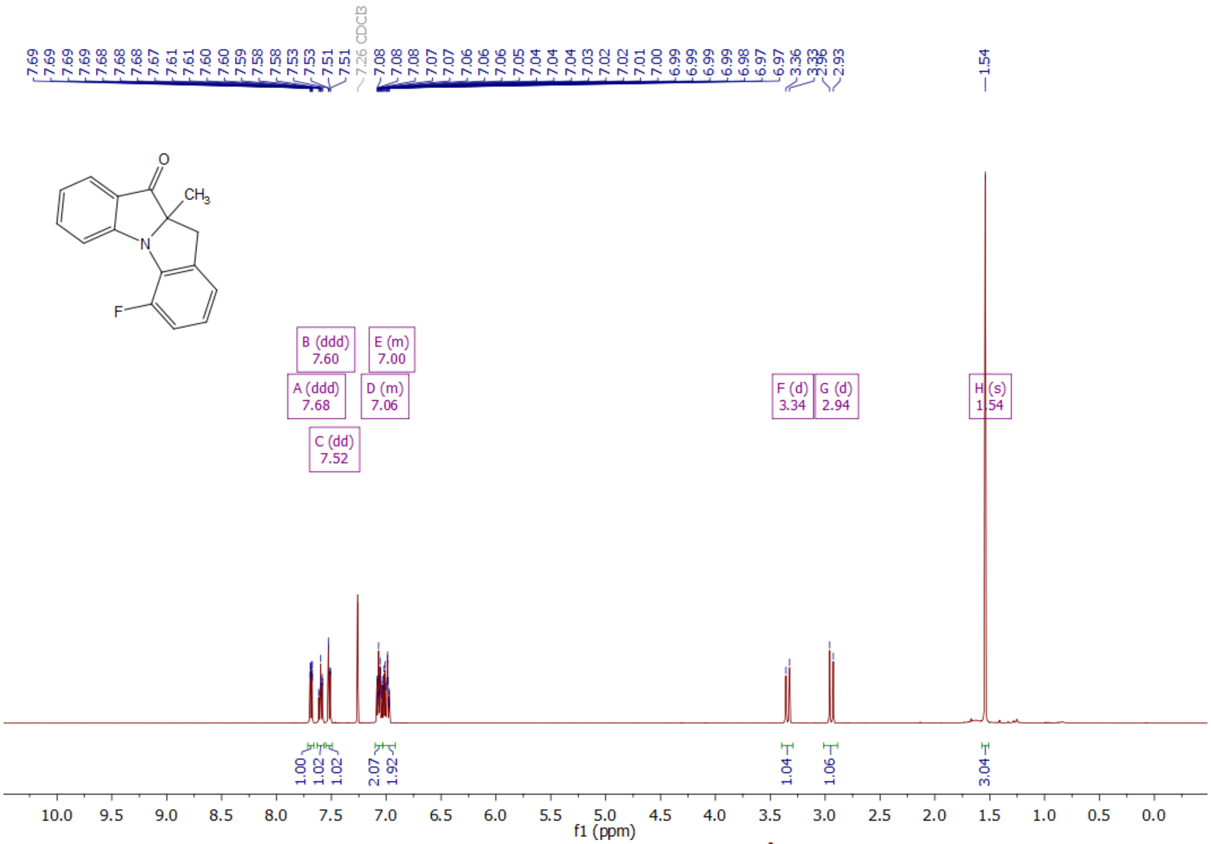

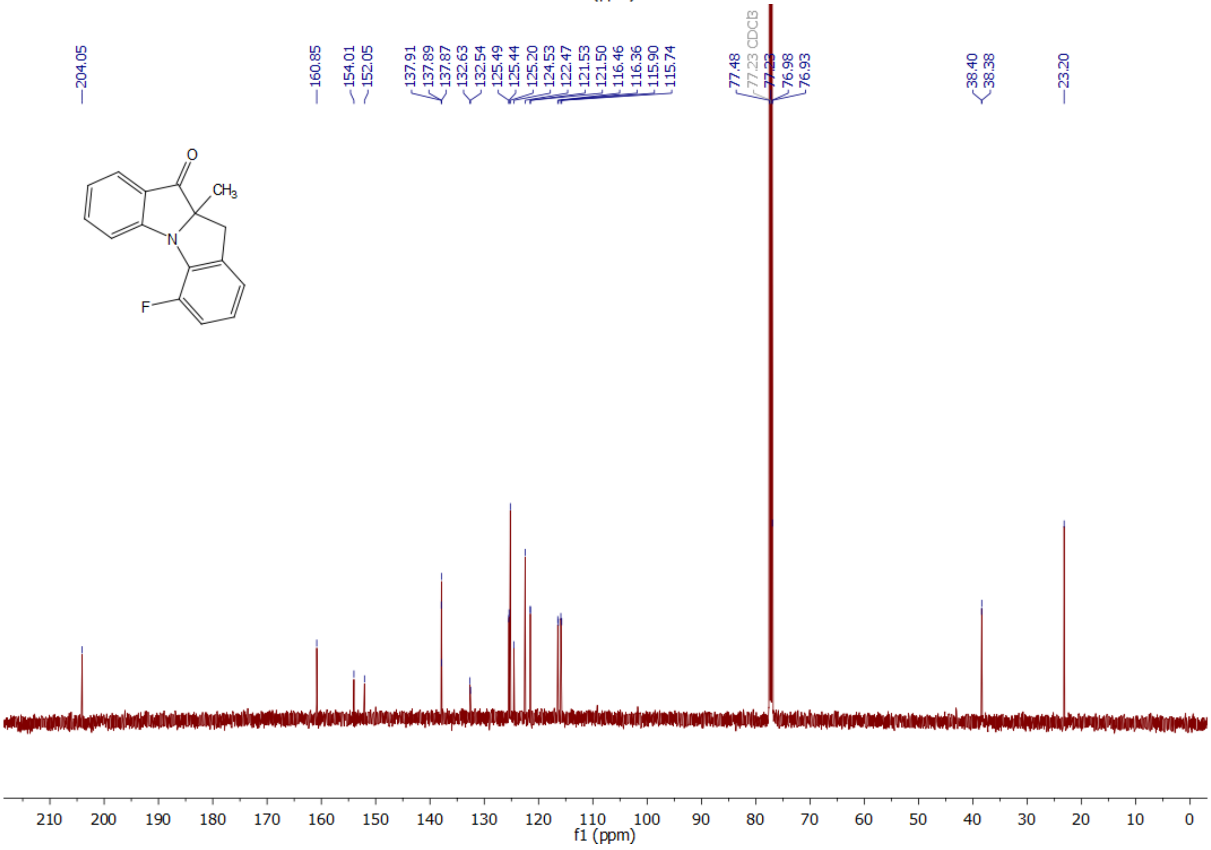

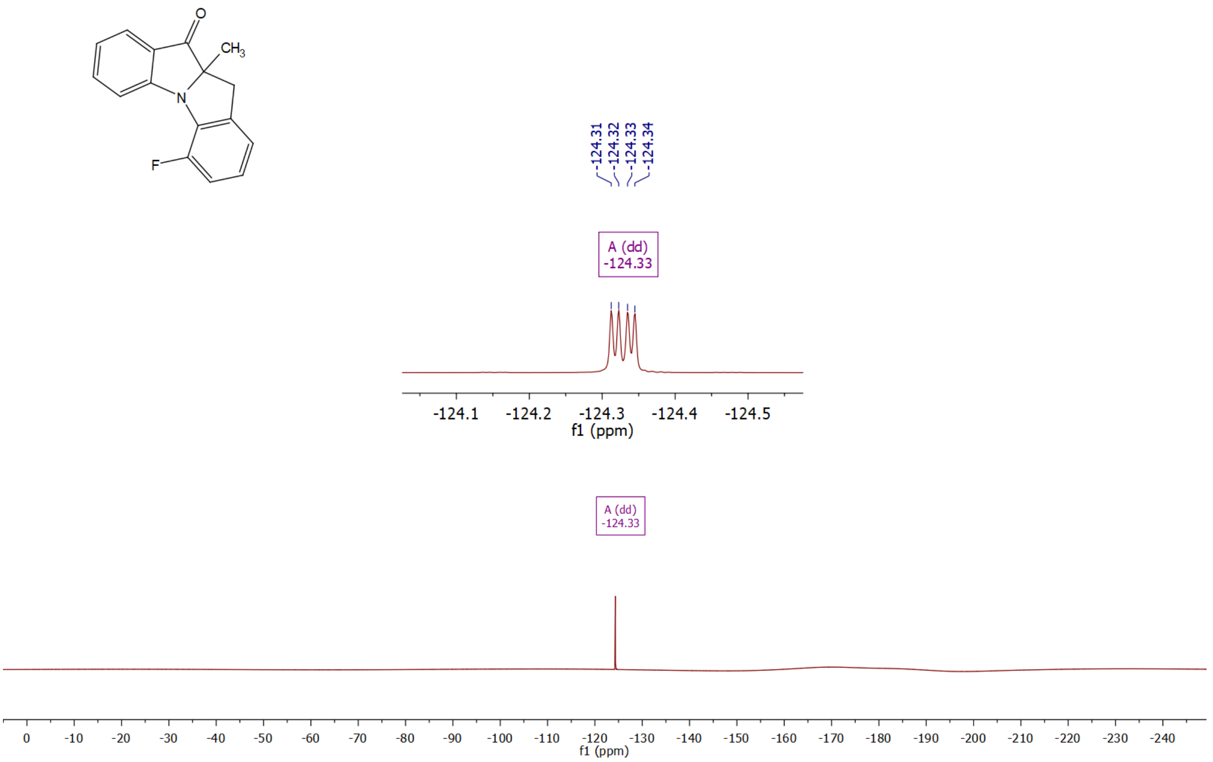
O
CH3
N
F
-124.31
-124.32
-124.33
-124.34
A (dd)
-124.33
-124.1 -124.2 -124.3 -124.4 -124.5
f1 (ppm)
A (dd)
-124.33
0 -10 -20 -30 -40 -50 -60 -70 -80 -90 -100 -110 -120 -130 -140 -150 -160 -170 -180 -190 -200 -210 -220 -230 -240
f1 (ppm)

3,10a-dimethyl-10a,11-dihydro-10H-indolo[1,2-a]indol-10-one [F3]:

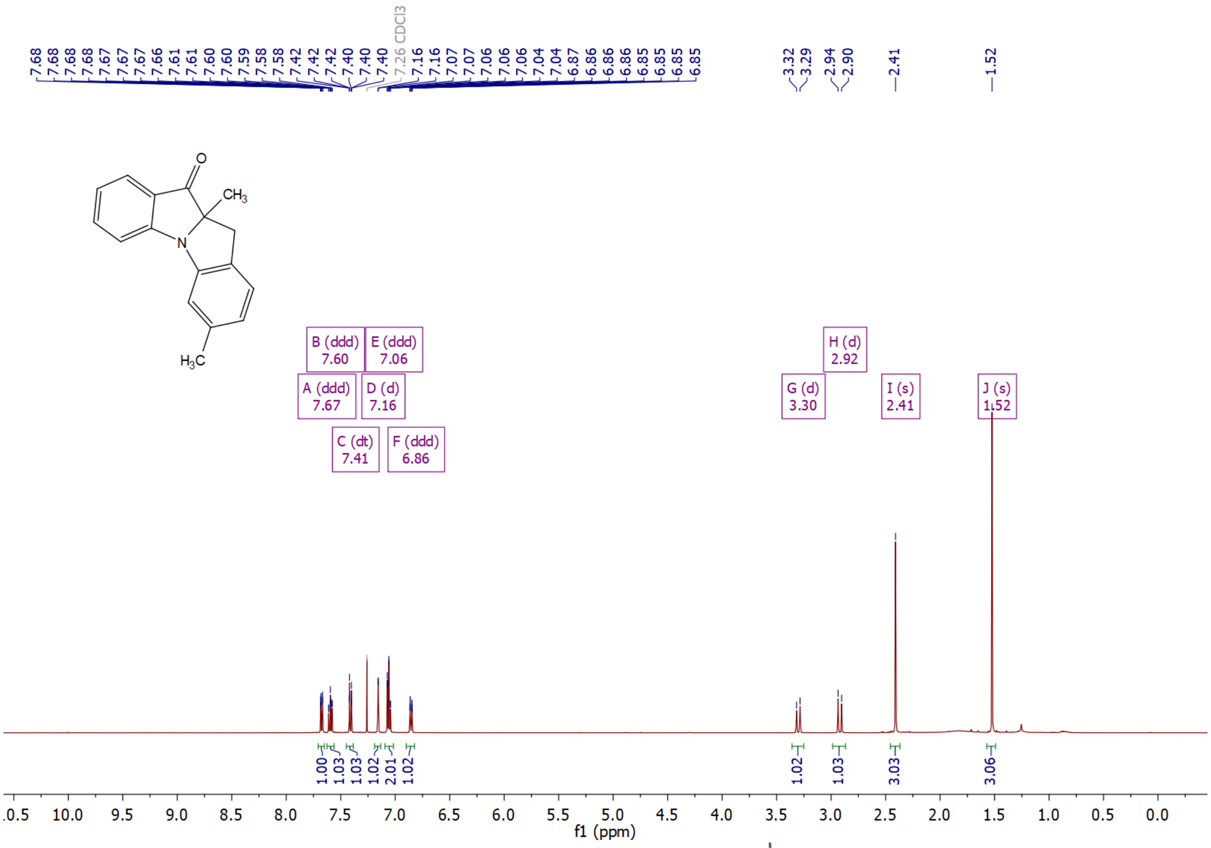

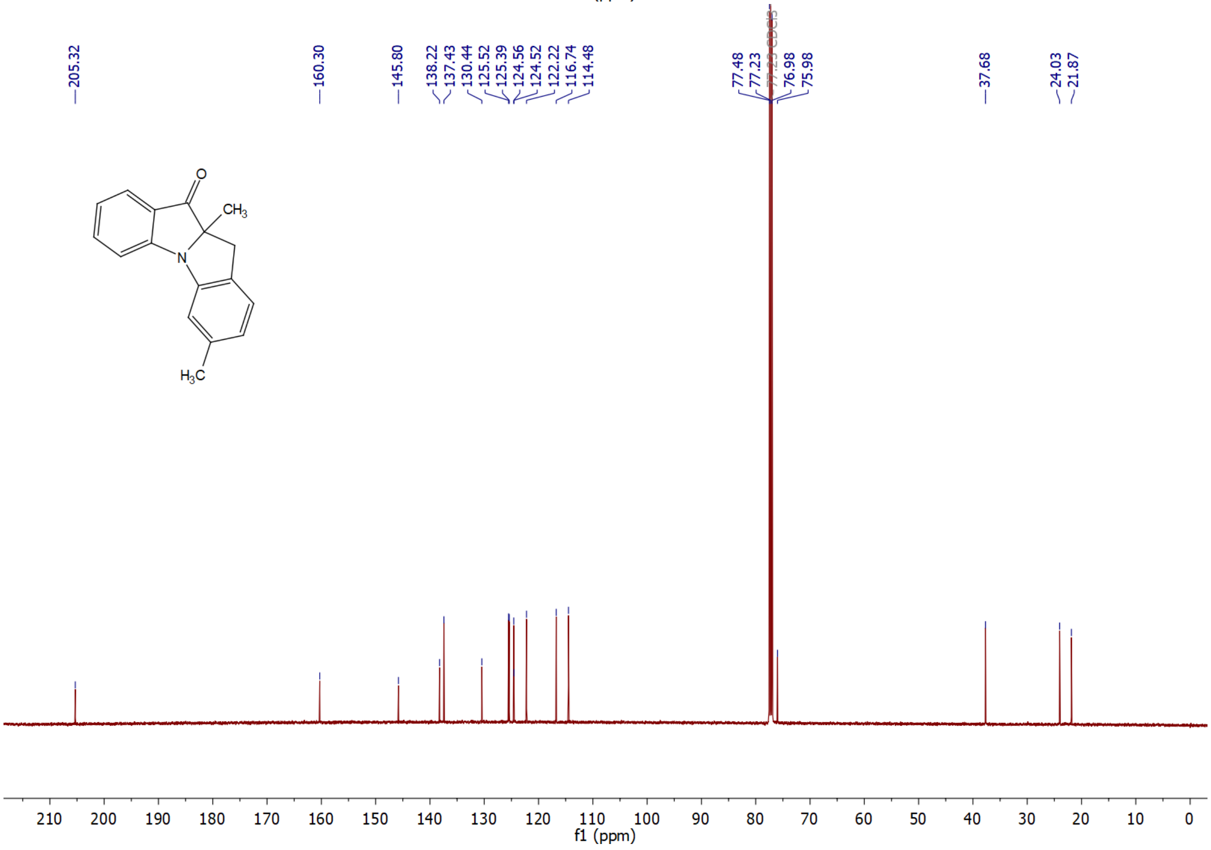

2-fluoro-10a-methyl-10a,11-dihydro-10H-indolo[1,2-a]indol-10-one **[F4]**:

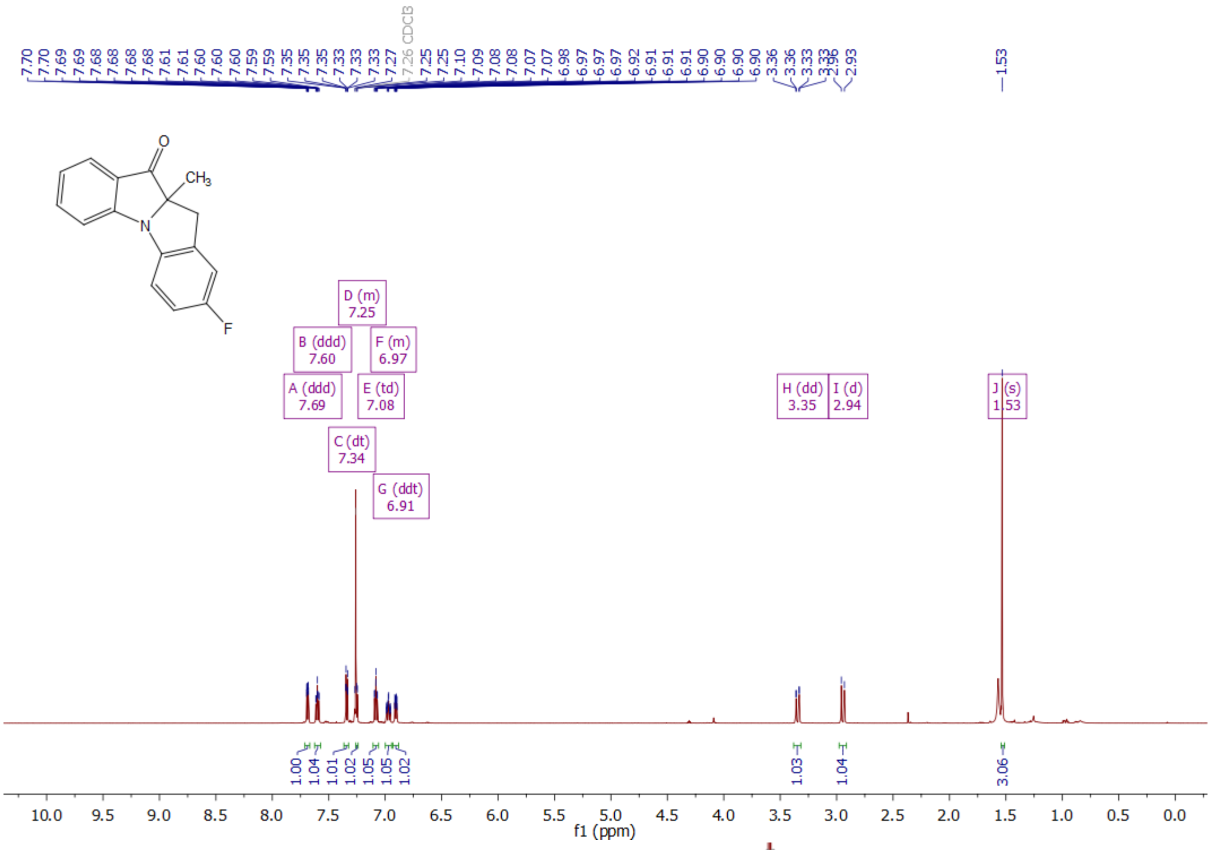

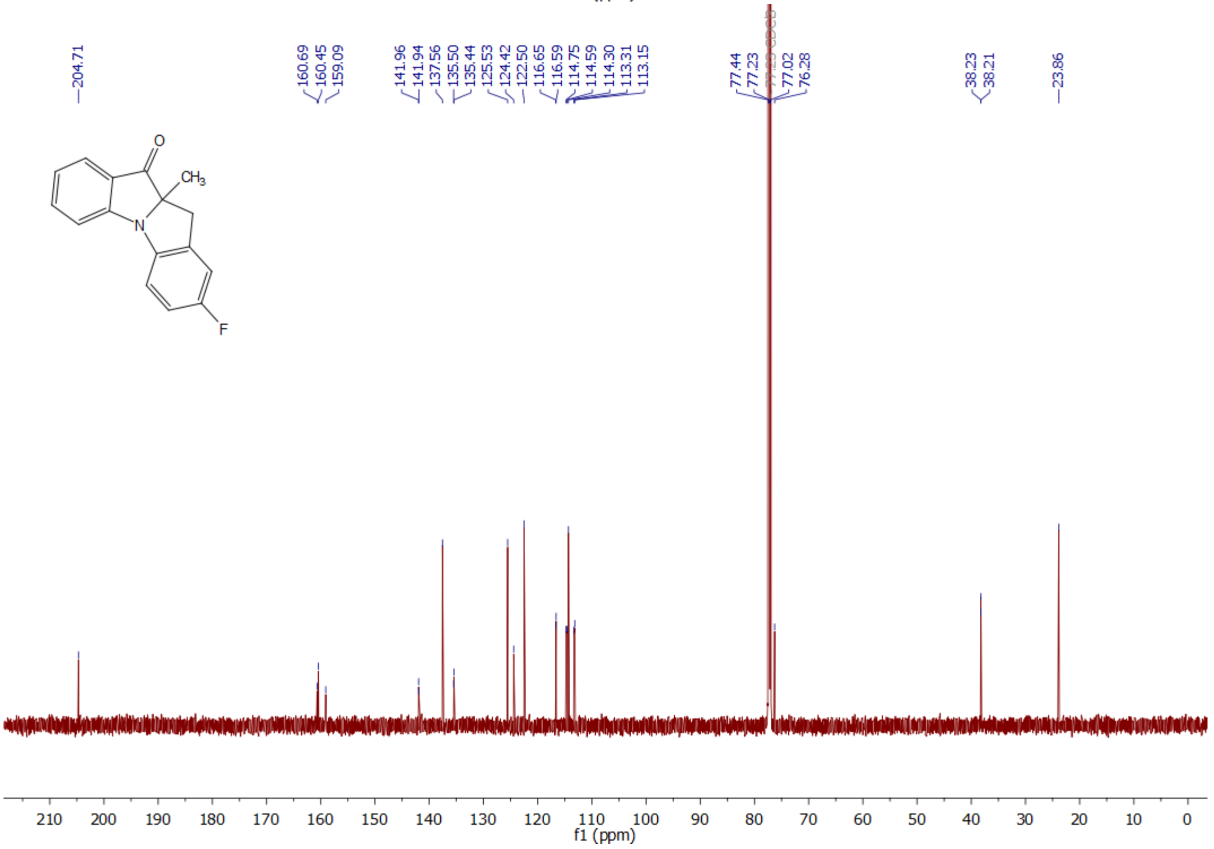

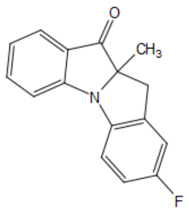

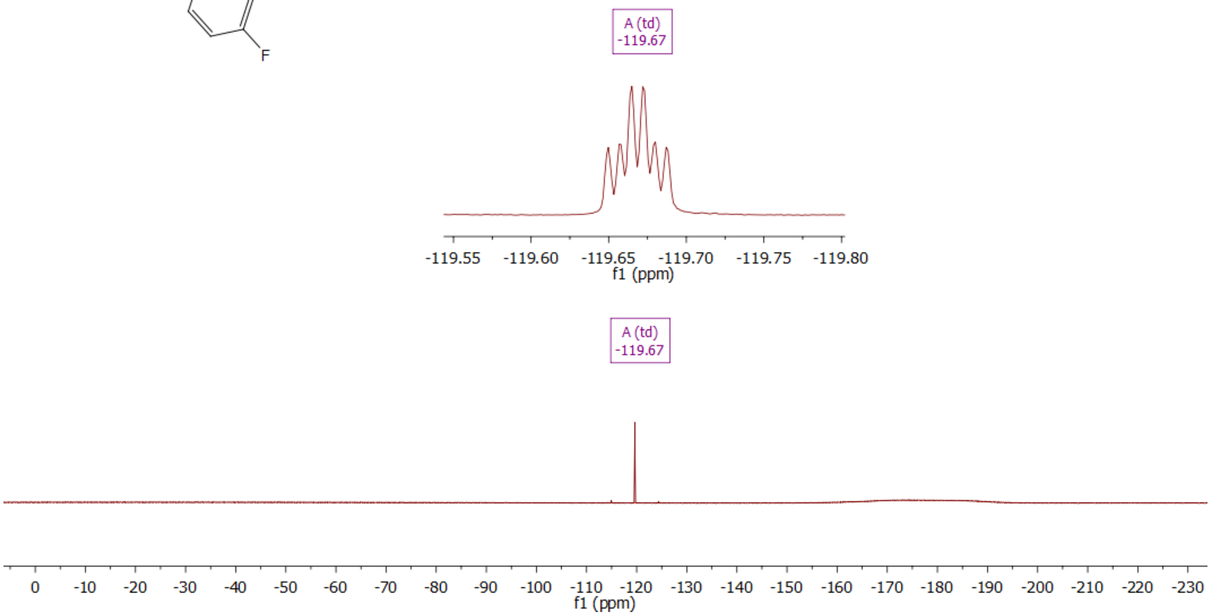

2-fluoro-10a-methyl-10a,11-dihydro-10H-indolo[1,2-a]indol-10-one **[F4-resynthesized]**:

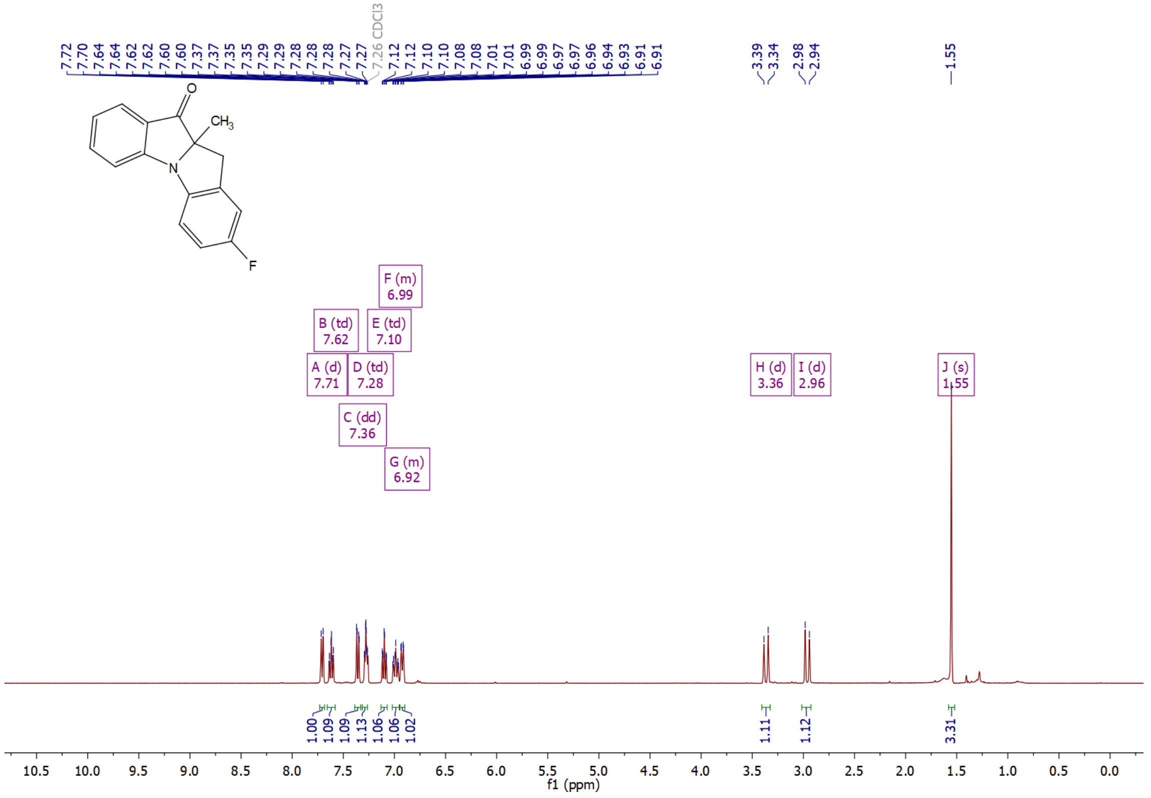

2-methoxy-10a-methyl-10a,11-dihydro-10H-indolo[1,2-a]indol-10-one [**F5**]:

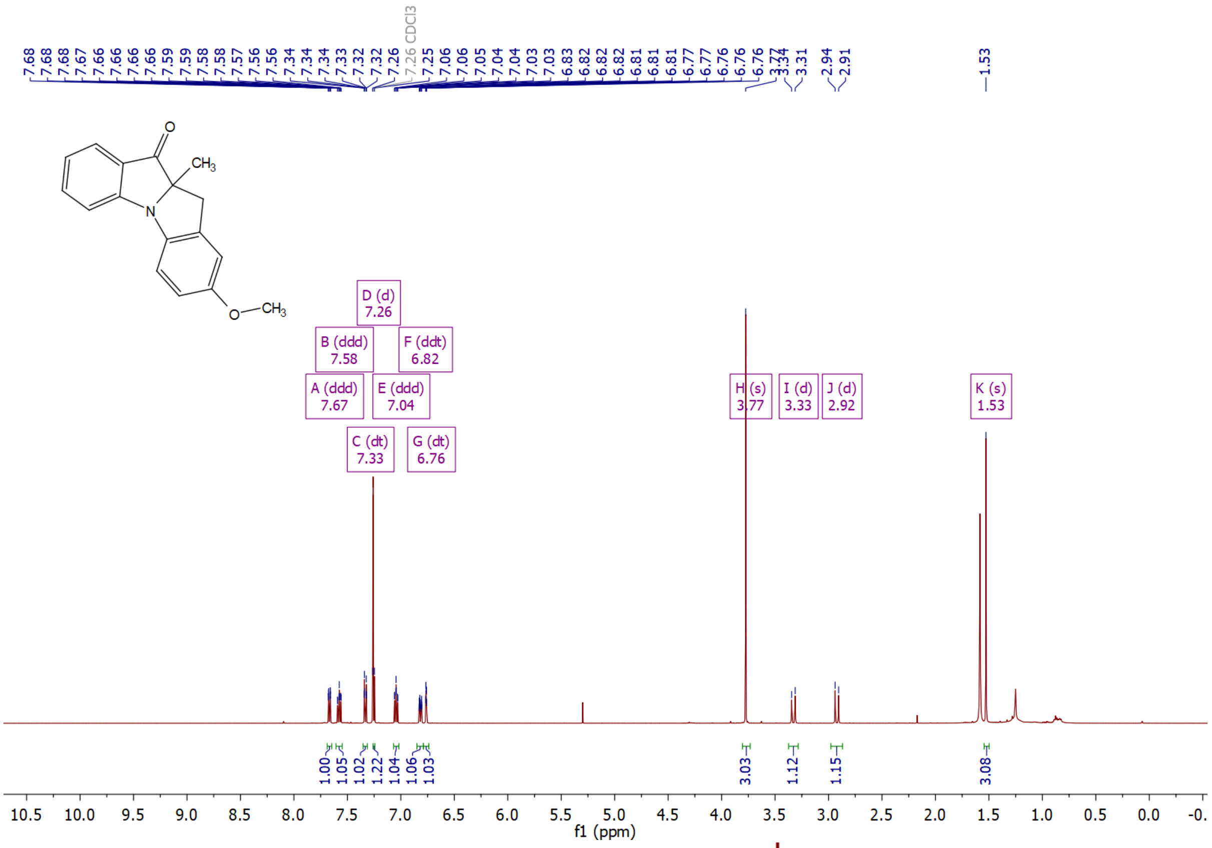

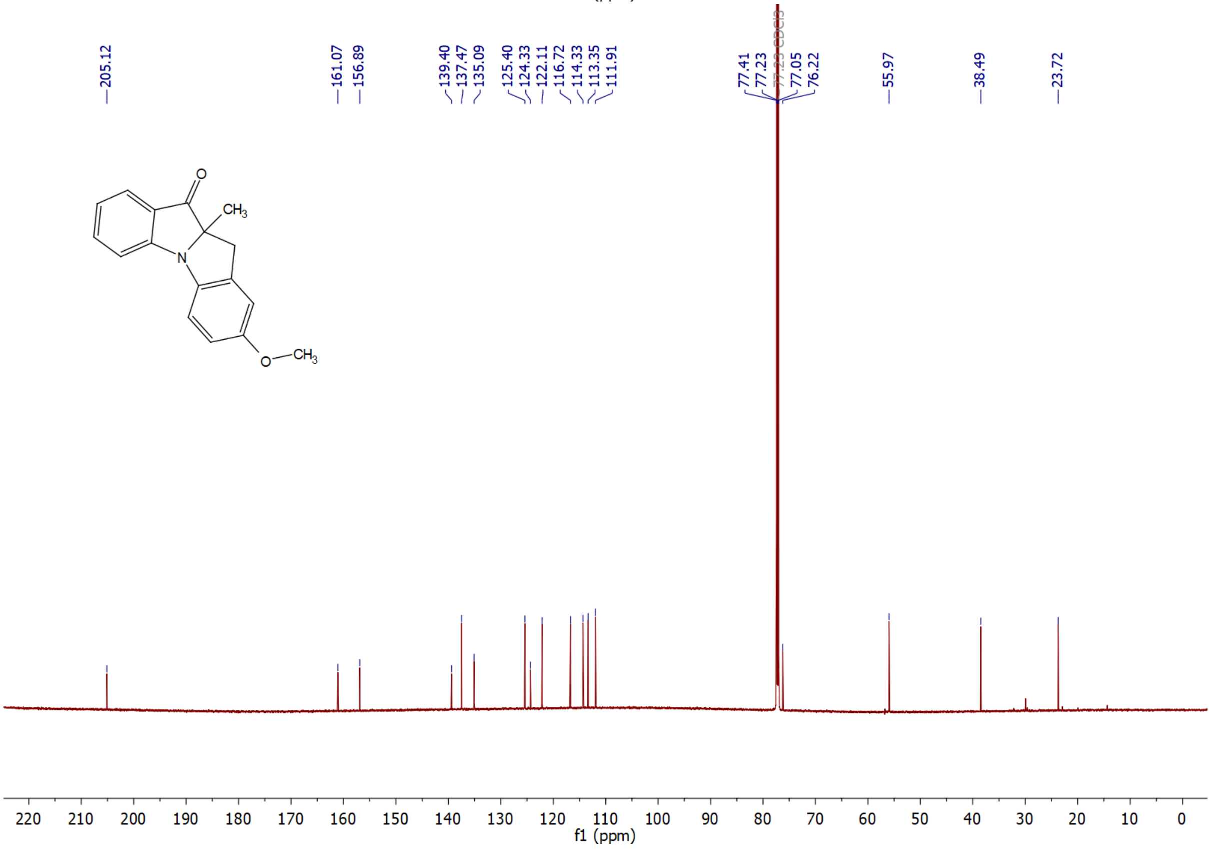

2-methoxy-10a-methyl-10a,11-dihydro-10H-indolo[1,2-a]indol-10-one [**F5-resynthesized**]:

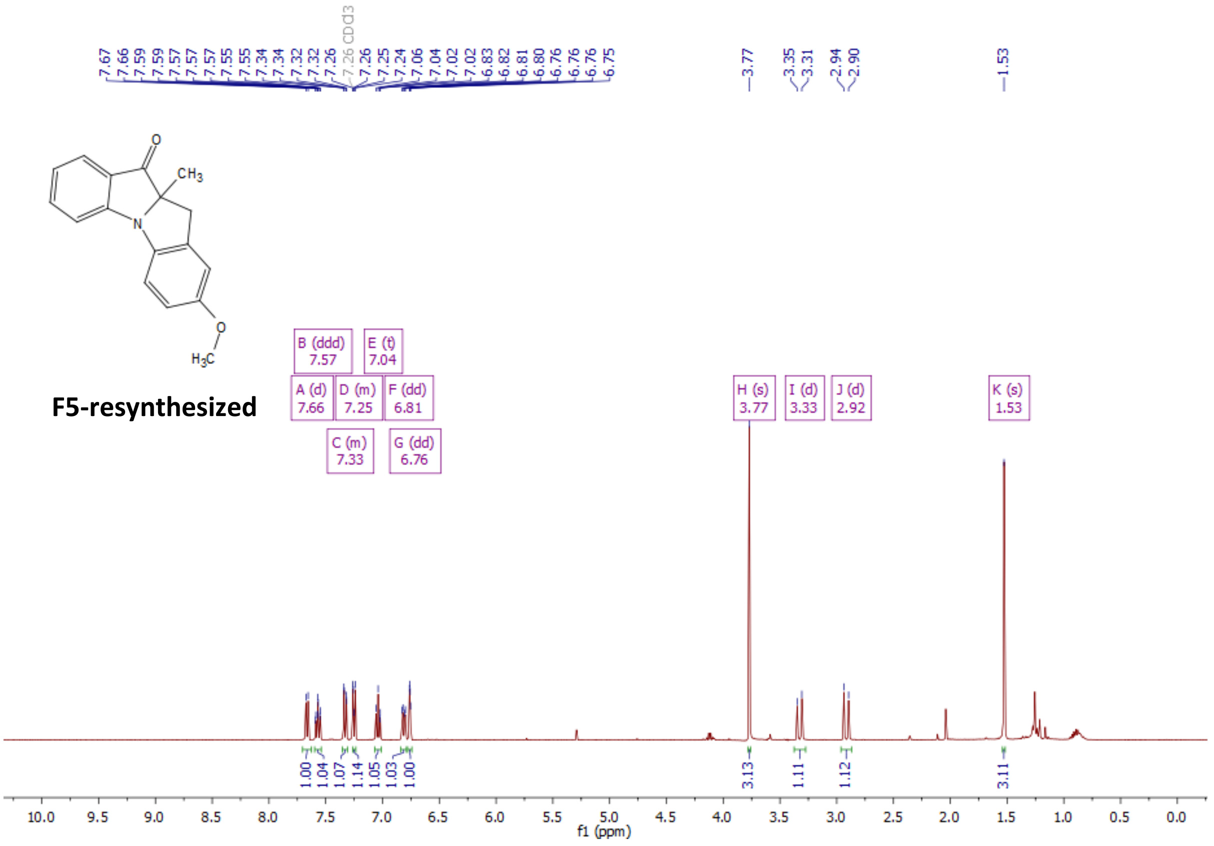

1-fluoro-10a-methyl-10a,11-dihydro-10H-indolo[1,2-a]indol-10-one [**F6**]:

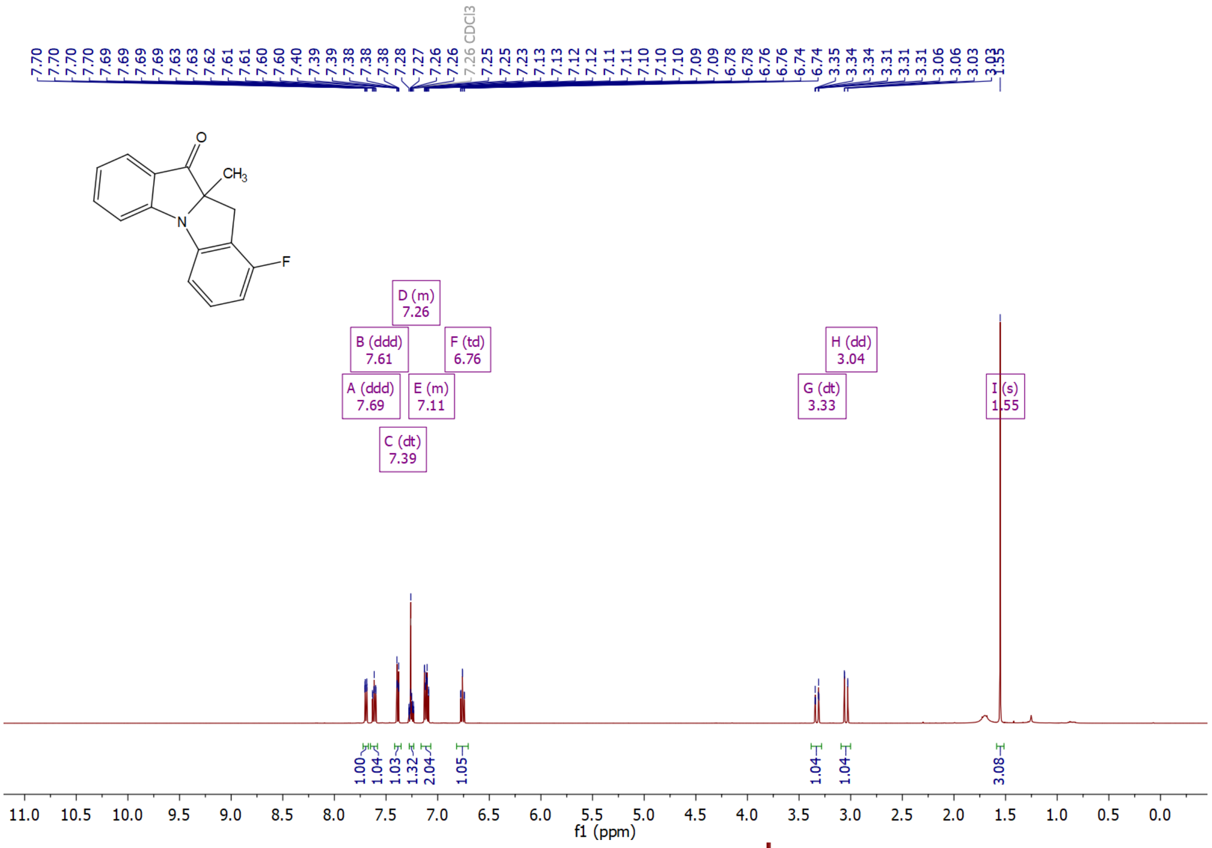

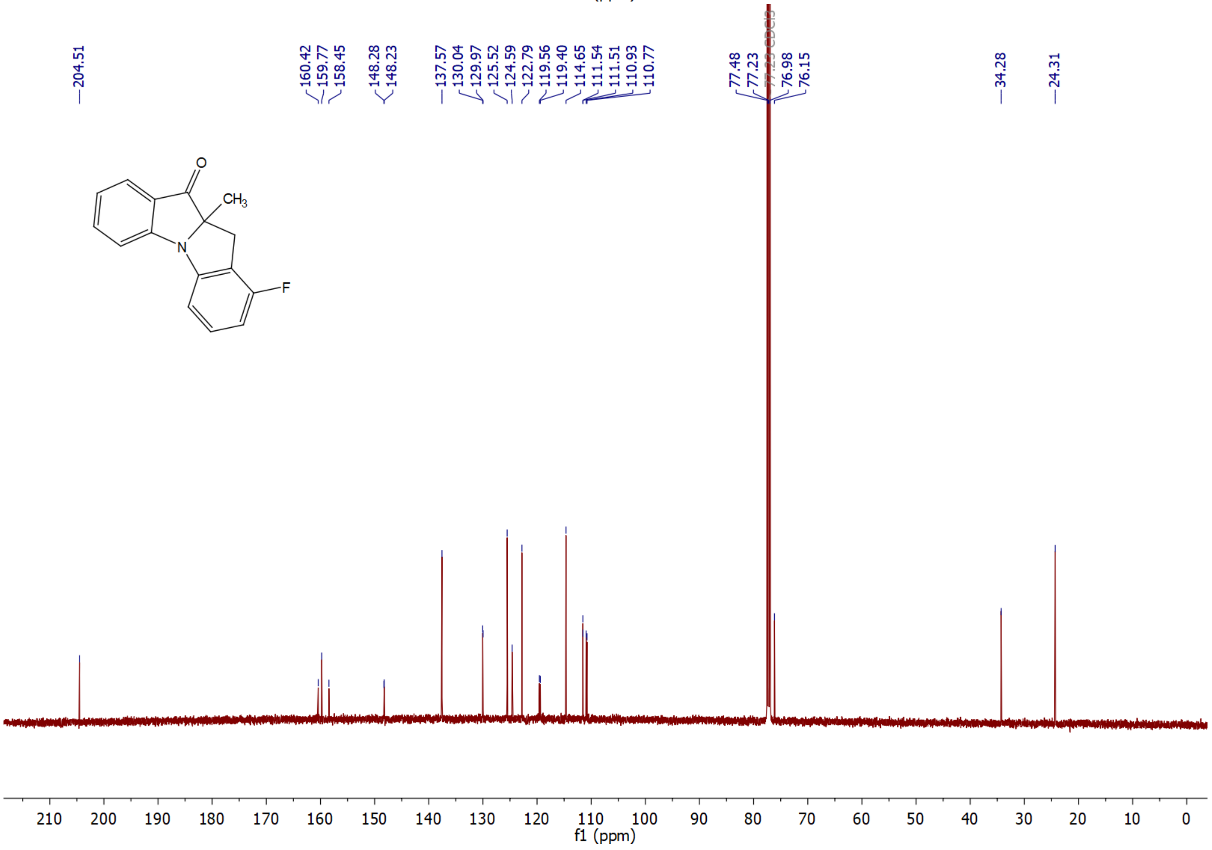

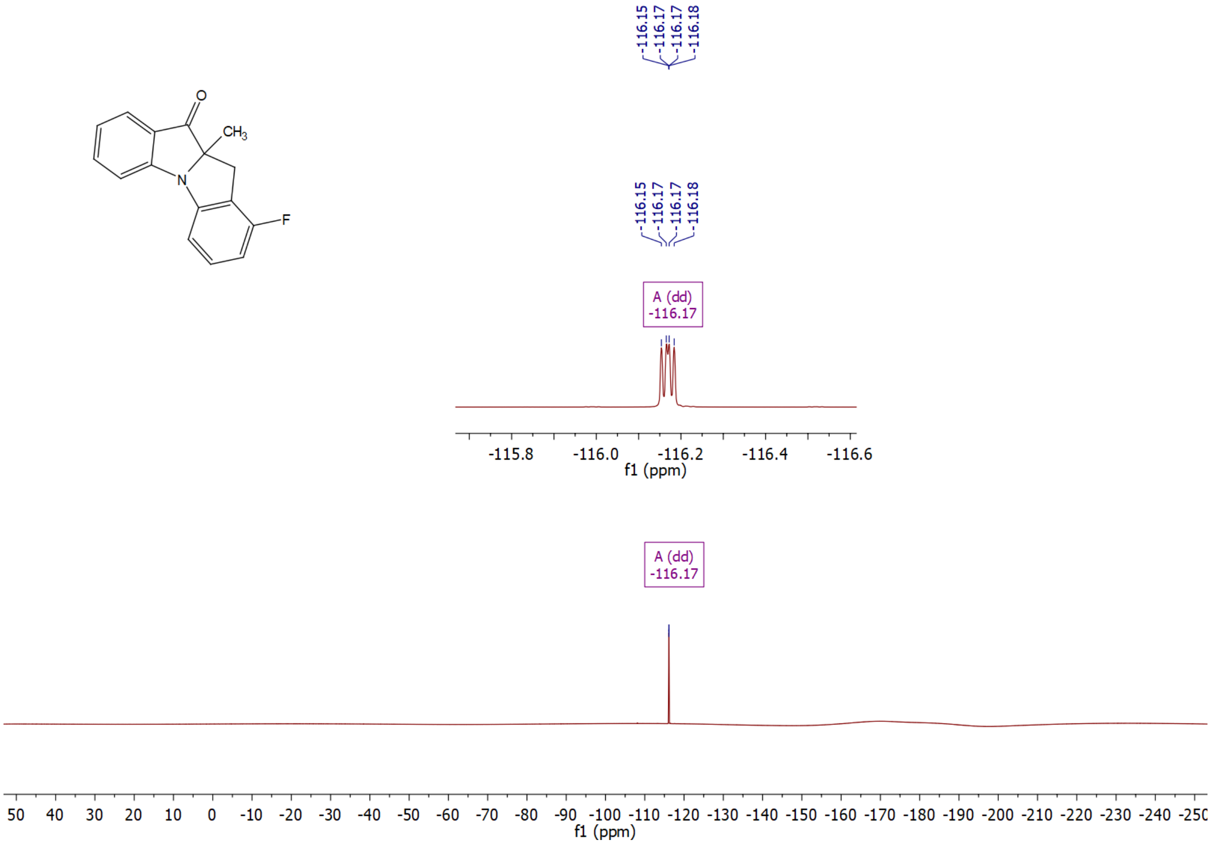
O
CH3
N
F
-116.15
-116.17
-116.17
-116.18
A (dd)
-116.17
-115.8
-116.0
-116.2
-116.4
-116.6
f1 (ppm)
A (dd)
-116.17
50 40 30 20 10 0 -10 -20 -30 -40 -50 -60 -70 -80 -90 -100 -110 -120 -130 -140 -150 -160 -170 -180 -190 -200 -210 -220 -230 -240 -250
f1 (ppm)

10a-methyl-10a,11-dihydro-10H-[1,3]dioxolo[4,5-f]indolo[1,2-a]indol-10-one **[F7]**:

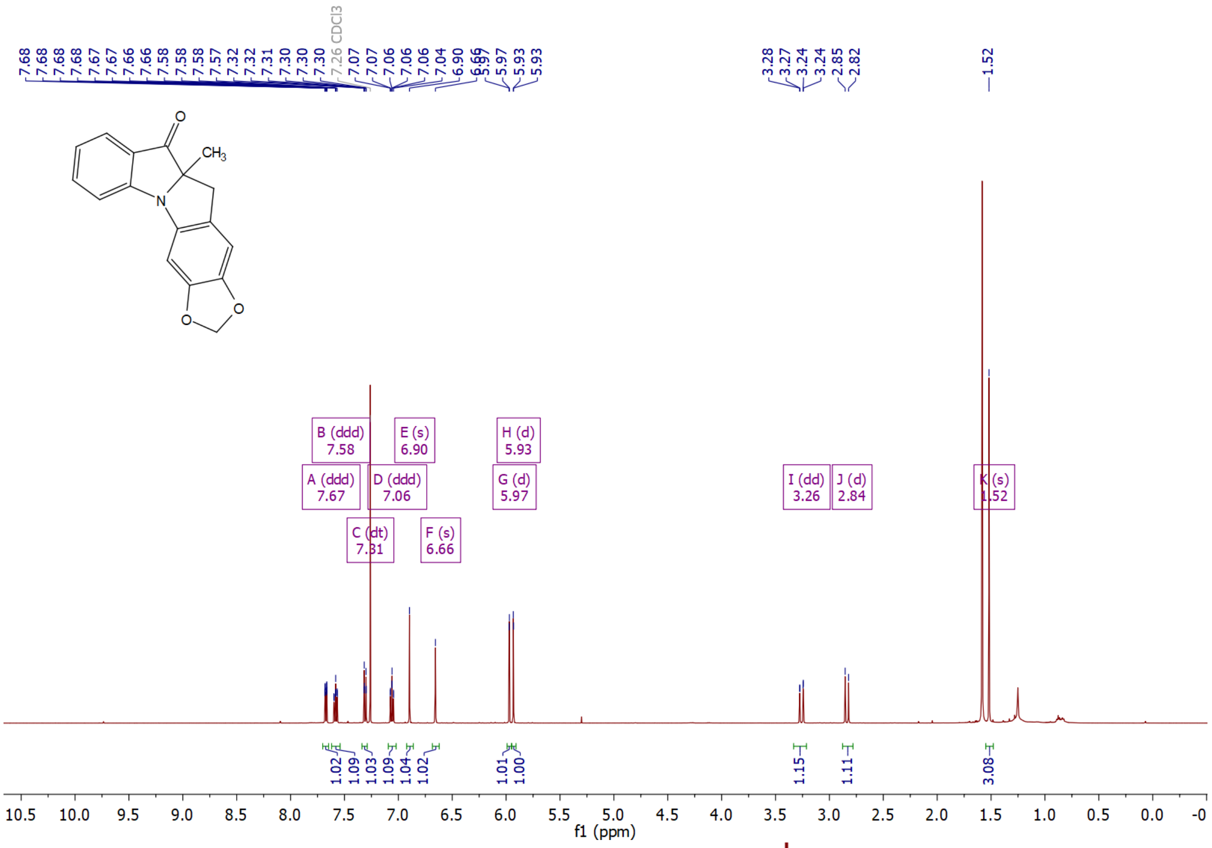

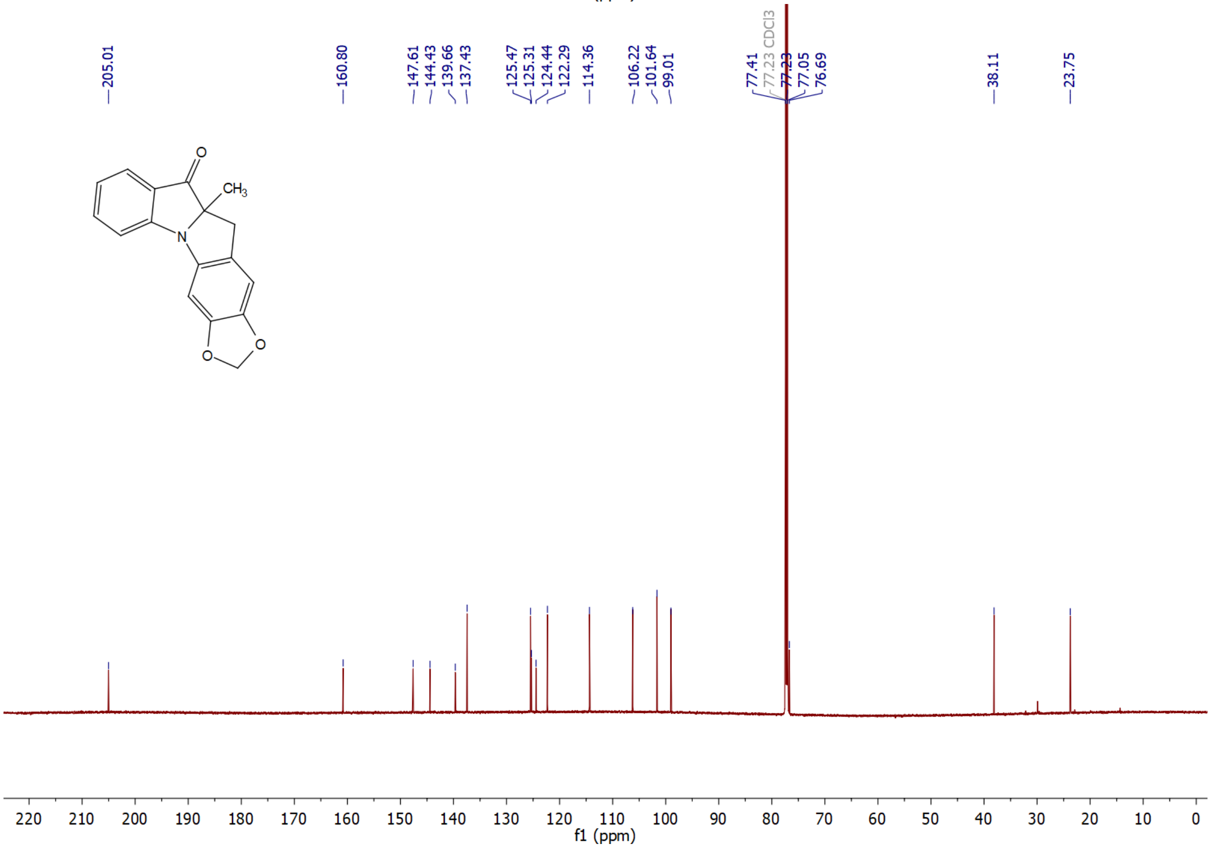

7a-methyl-7,7a-dihydro-8H-benzo[g]indolo[1,2-a]indol-8-one [**F8**]:

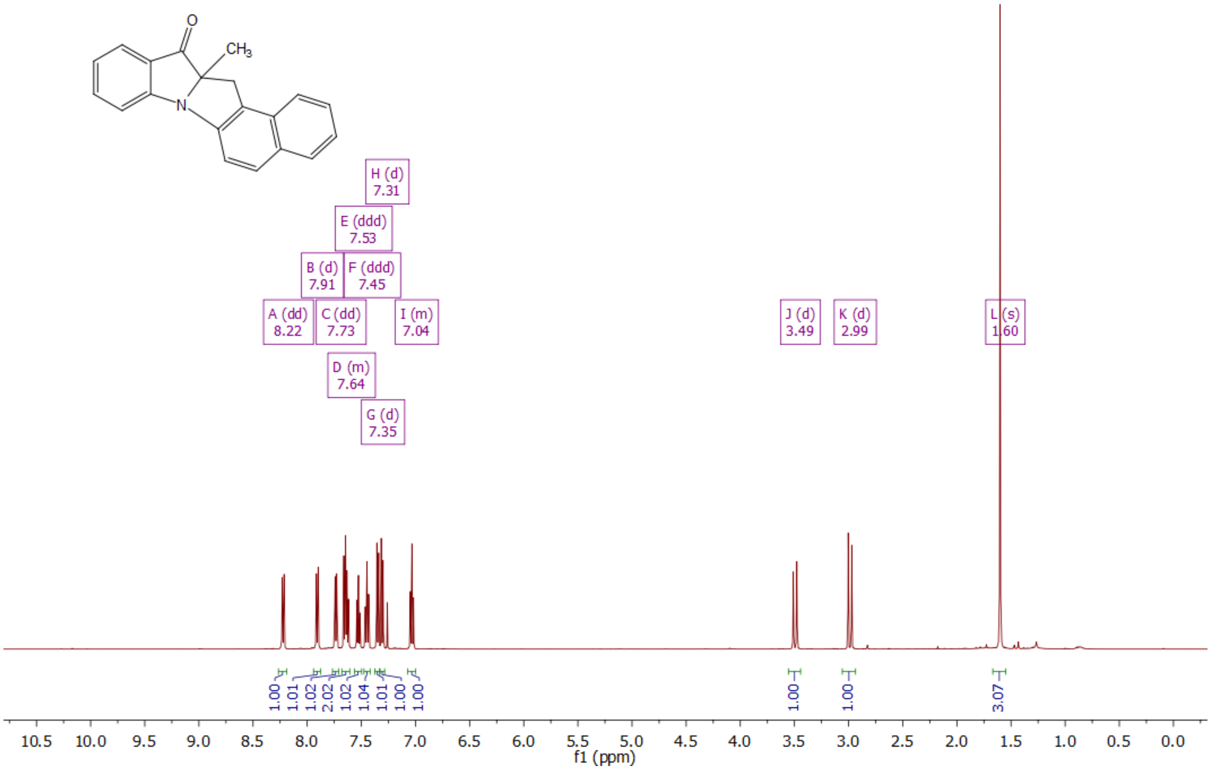

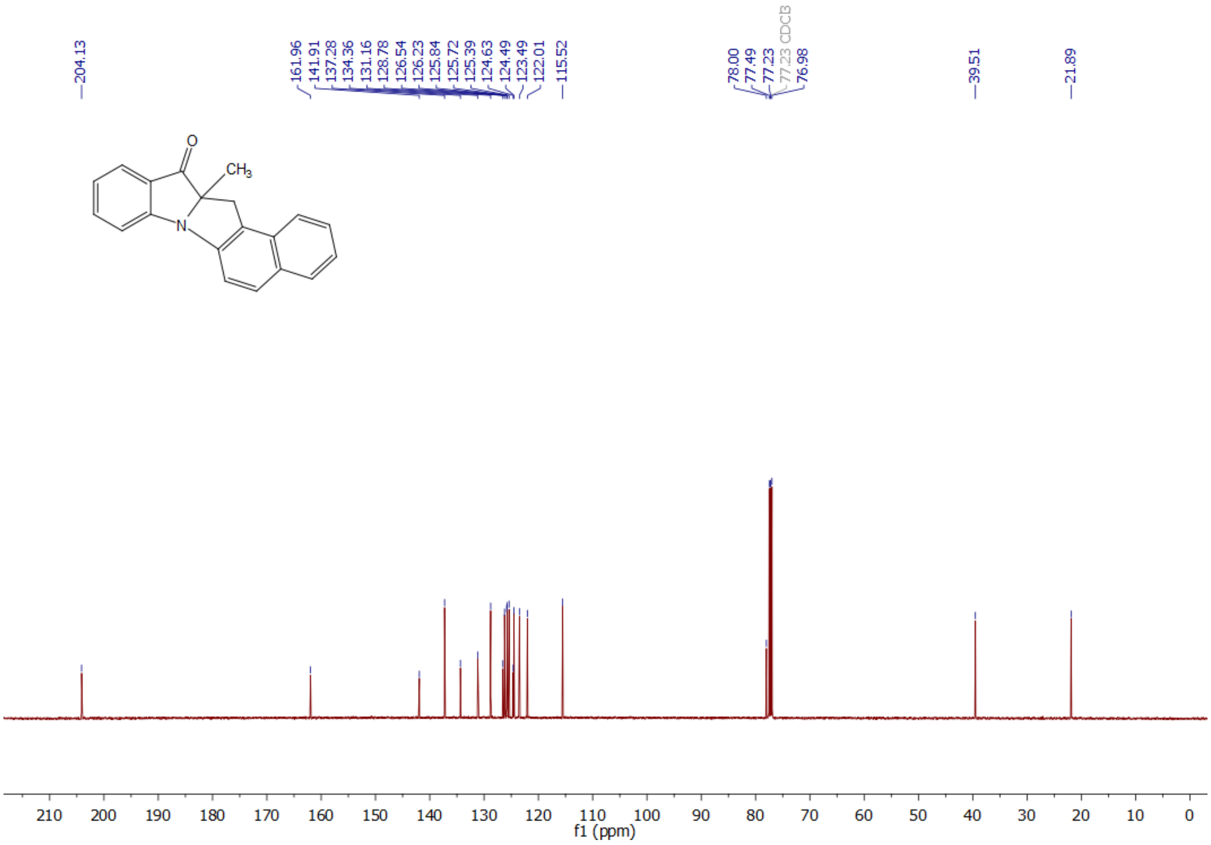

8,10a-dimethyl-10a,11-dihydro-10H-indolo[1,2-a]indol-10-one [F9]:

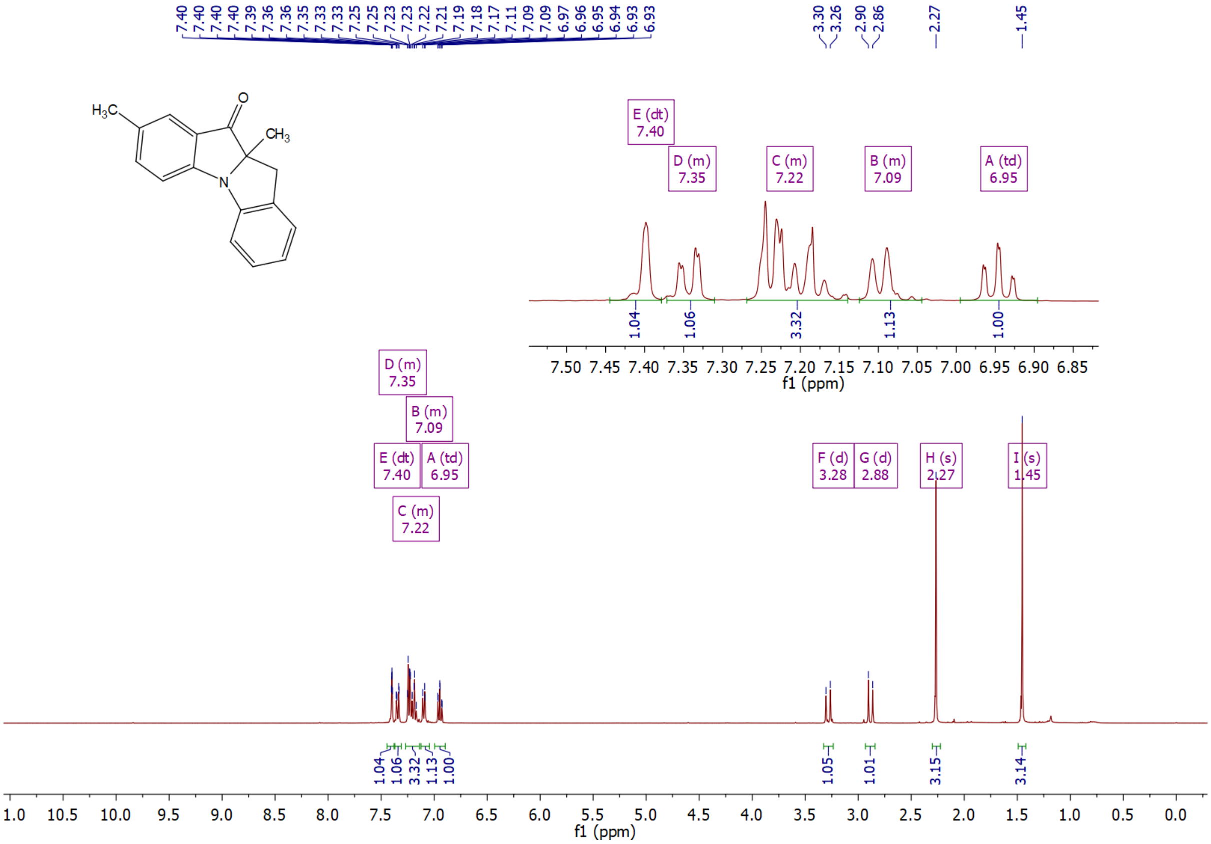

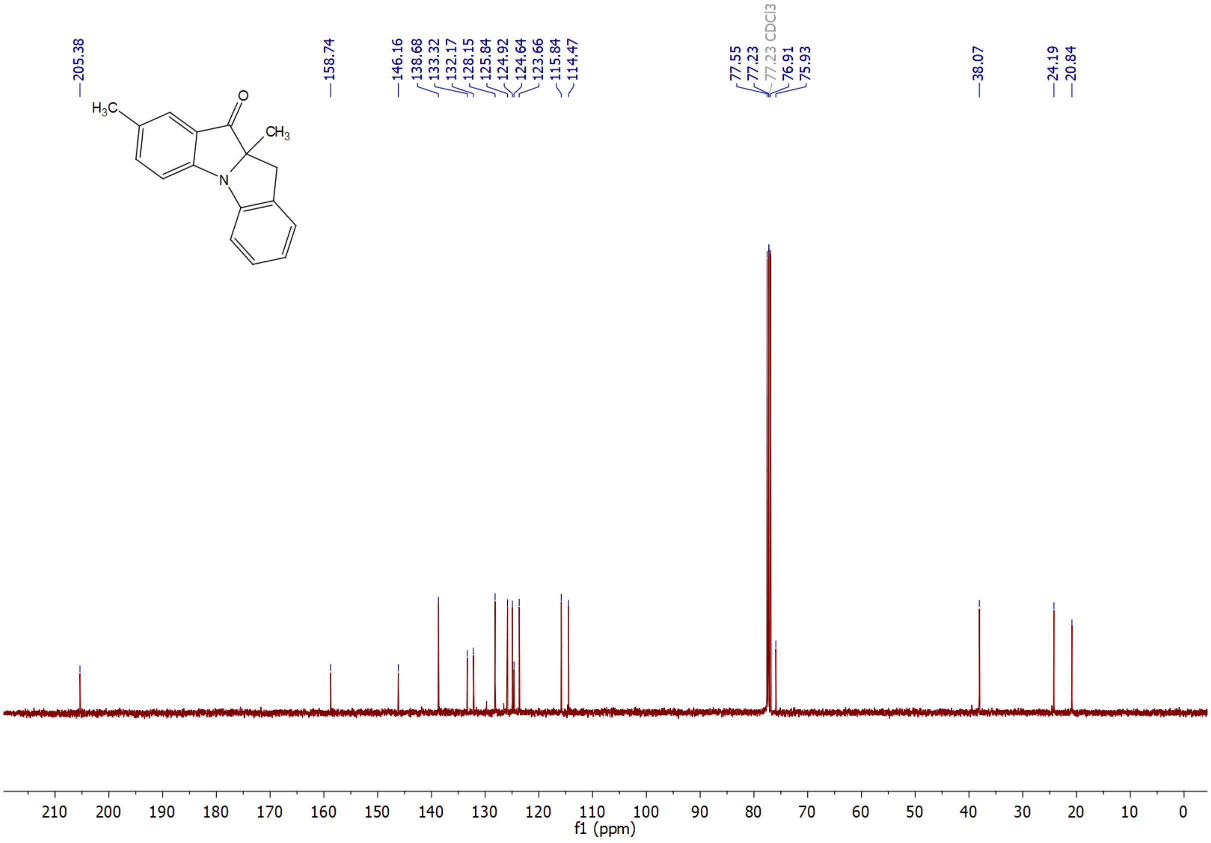

8-chloro-10a-methyl-10a,11-dihydro-10H-indolo[1,2-a]indol-10-one [**F10**]:

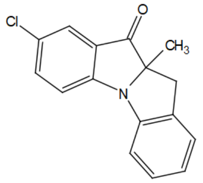

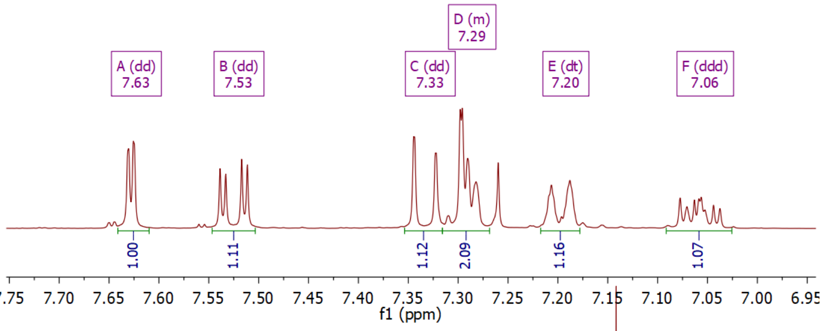

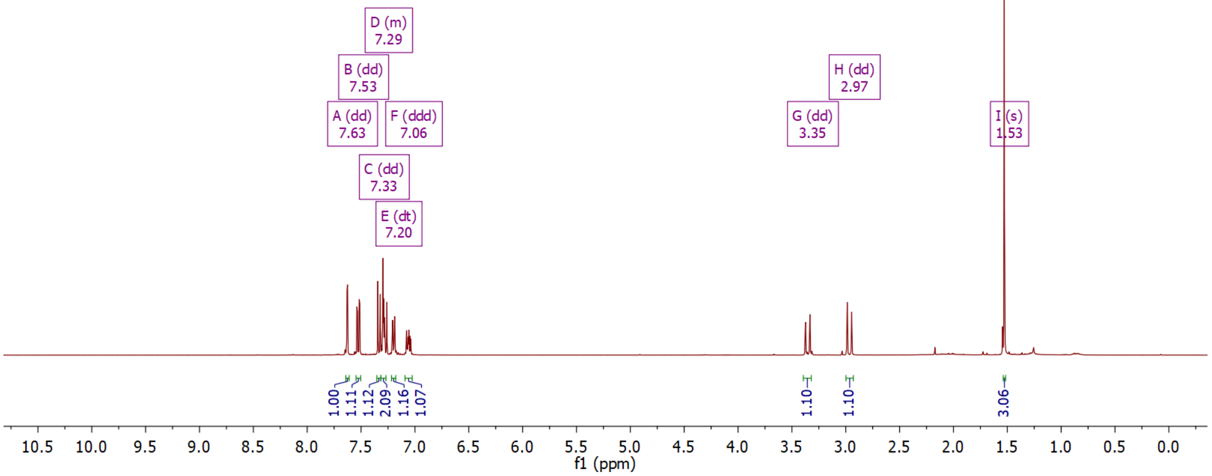

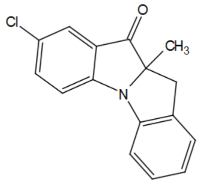

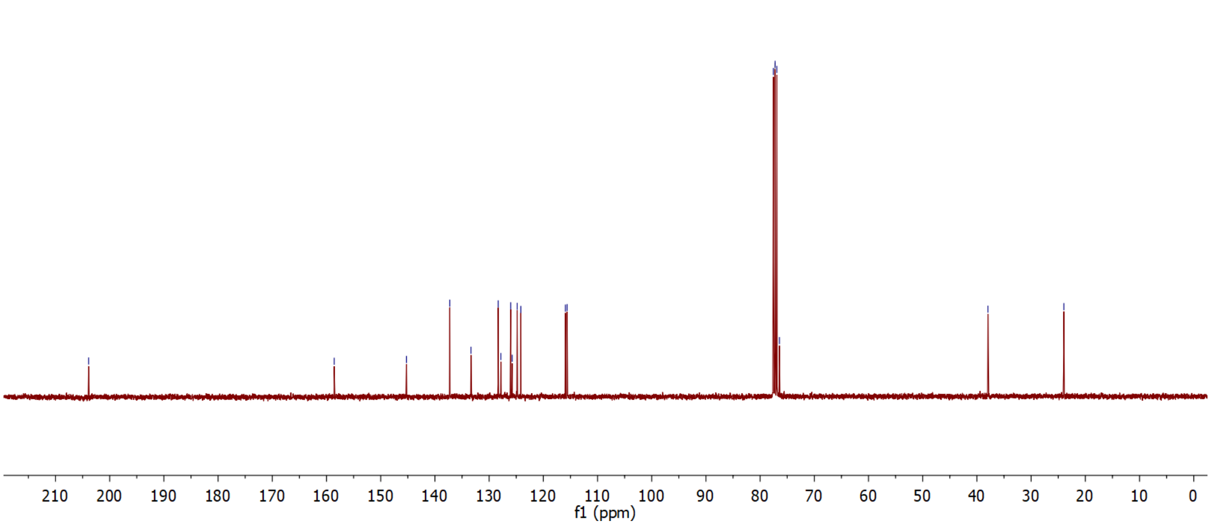

4-fluoro-8,10a-dimethyl-10a,11-dihydro-10H-indolo[1,2-a]indol-10-one [**F11**]:

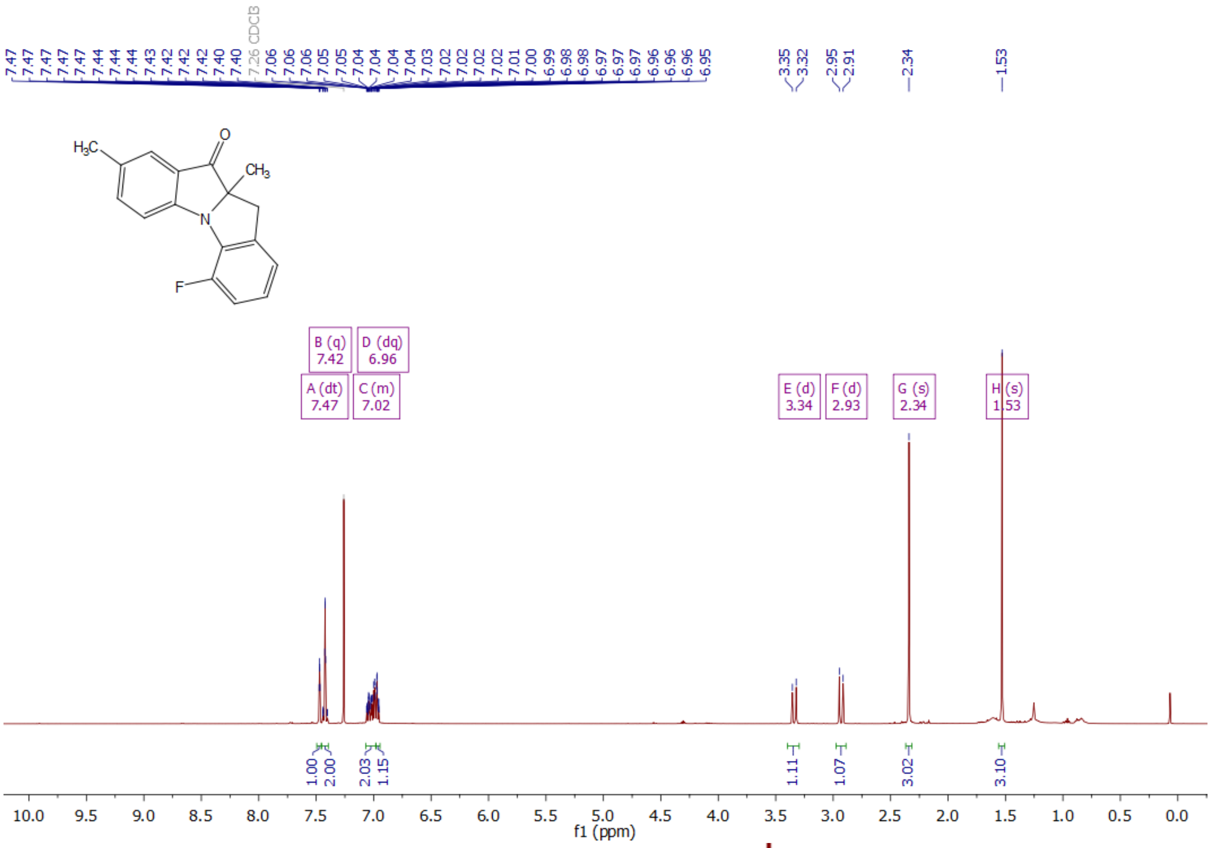

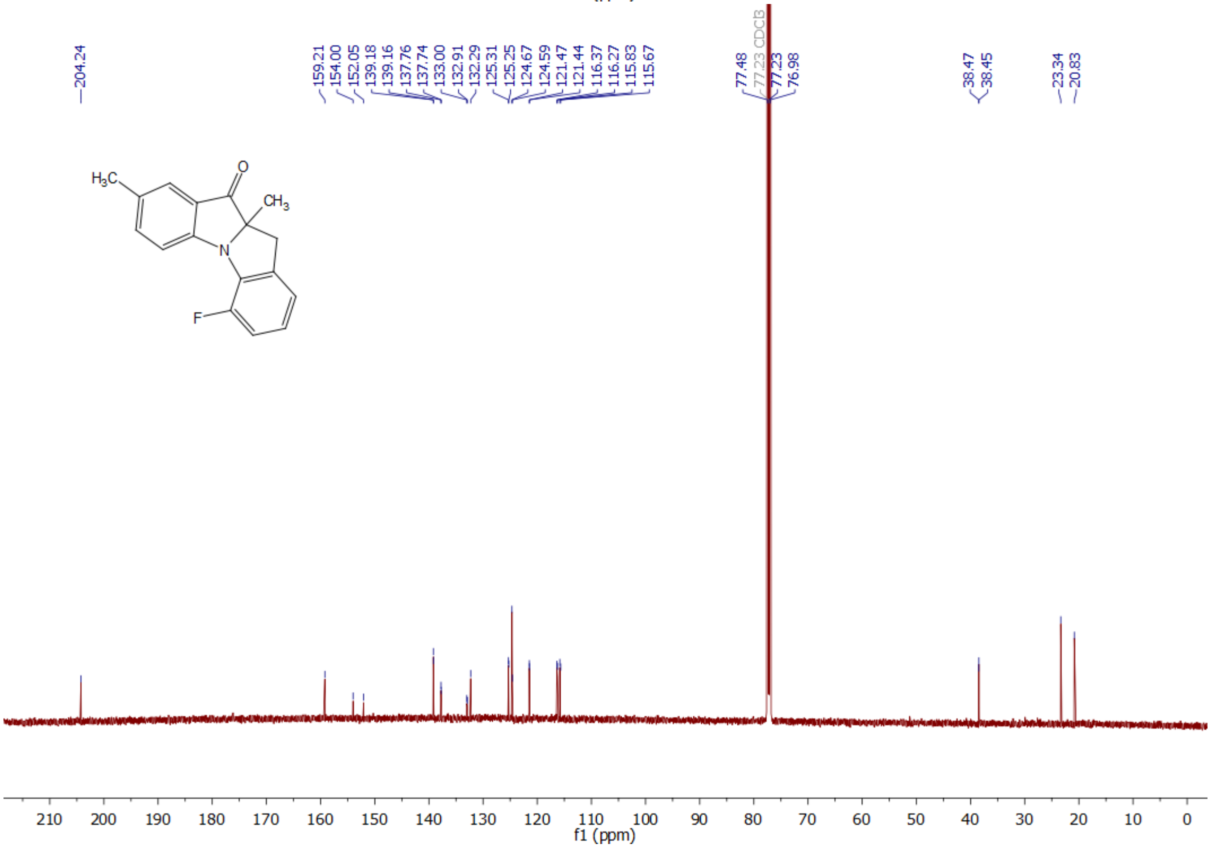

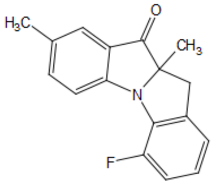
H3C
O
CH3
N
F

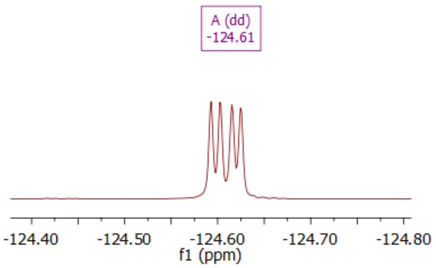
A (dd)
-124.61
-124.40
-124.50
-124.60
-124.70
-124.80
f1 (ppm)

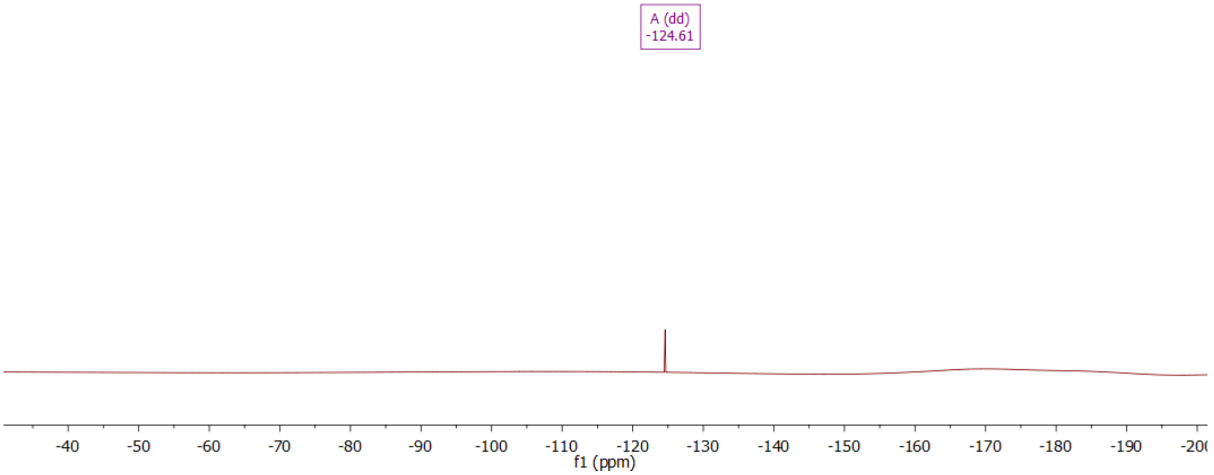
A (dd)
-124.61
-40
-50
-60
-70
-80
-90
-100
-110
-120
-130
-140
-150
-160
-170
-180
-190
f1 (ppm)

8-fluoro-3-methoxy-10a-methyl-10a,11-dihydro-10H-indolo[1,2-a]indol-10-one [**F12**]:

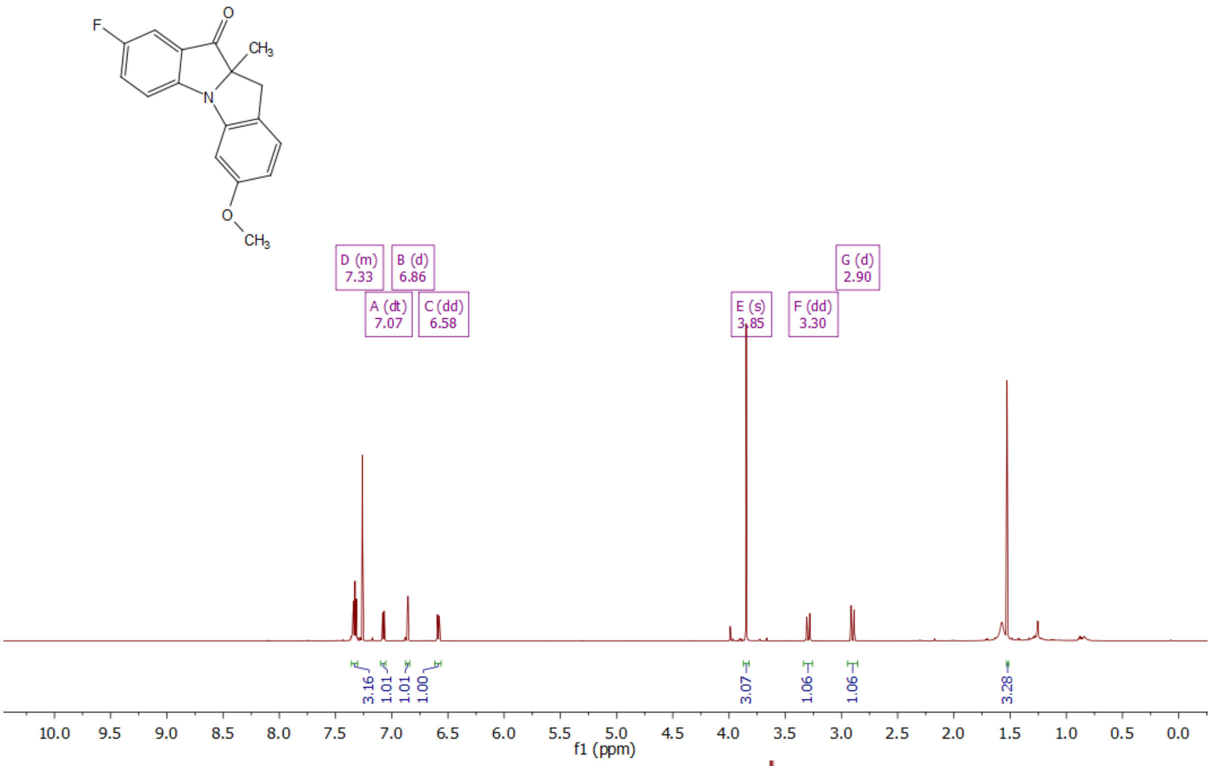

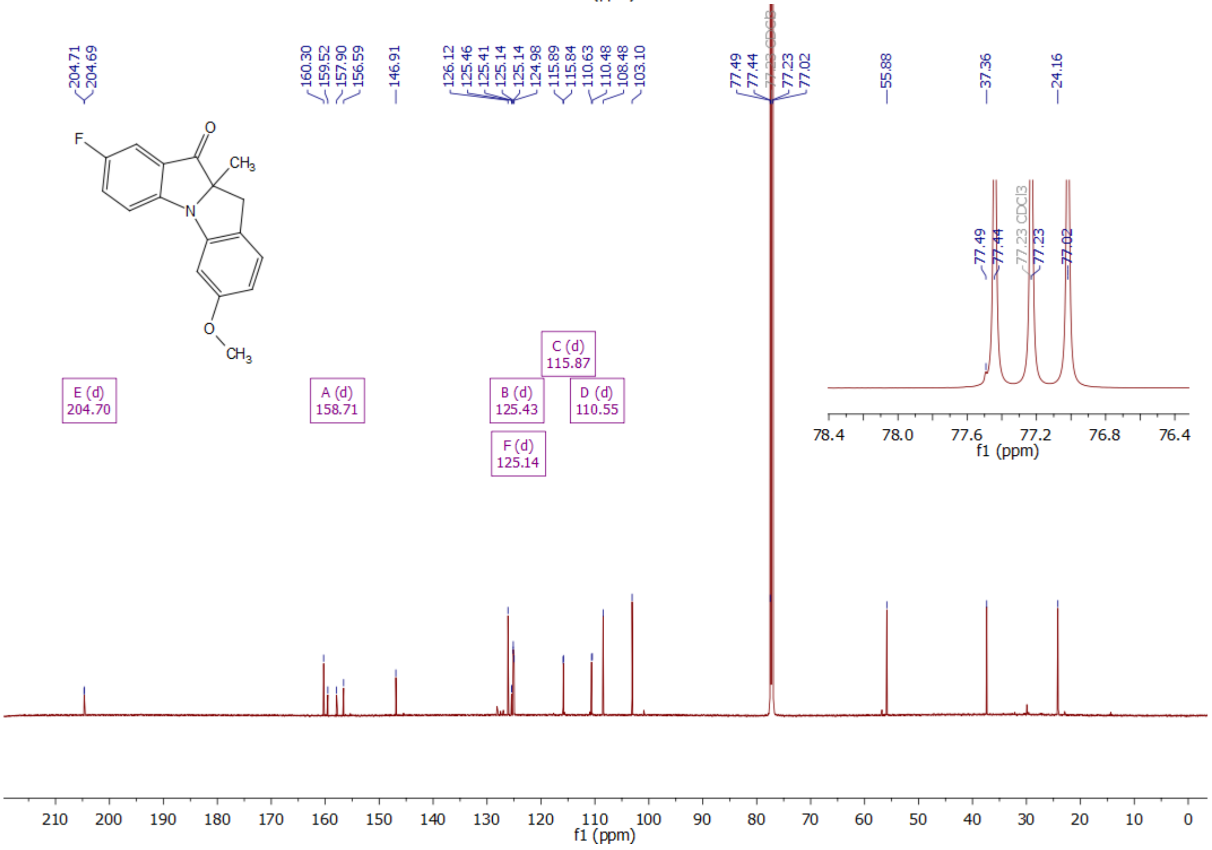

3-chloro-8,10a-dimethyl-10a,11-dihydro-10H-indolo[1,2-a]indol-10-one [F13]:

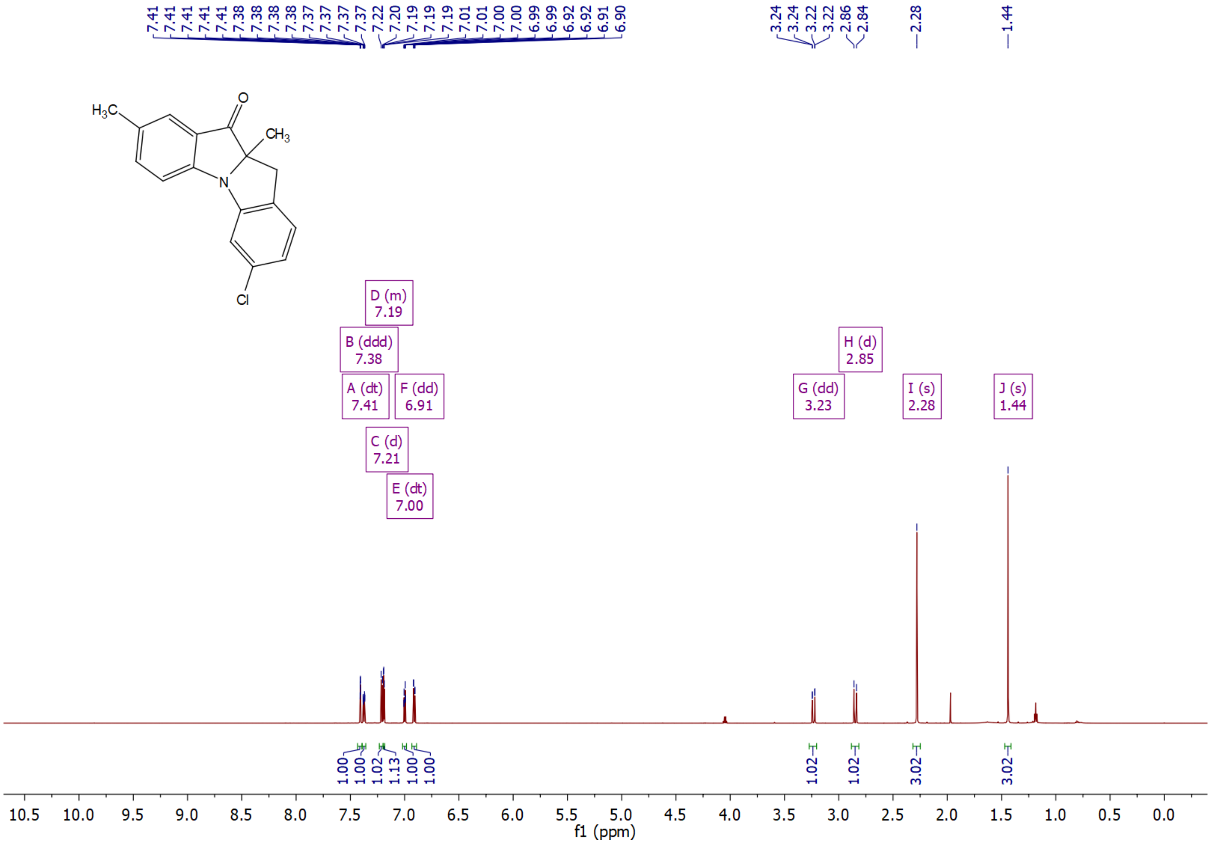

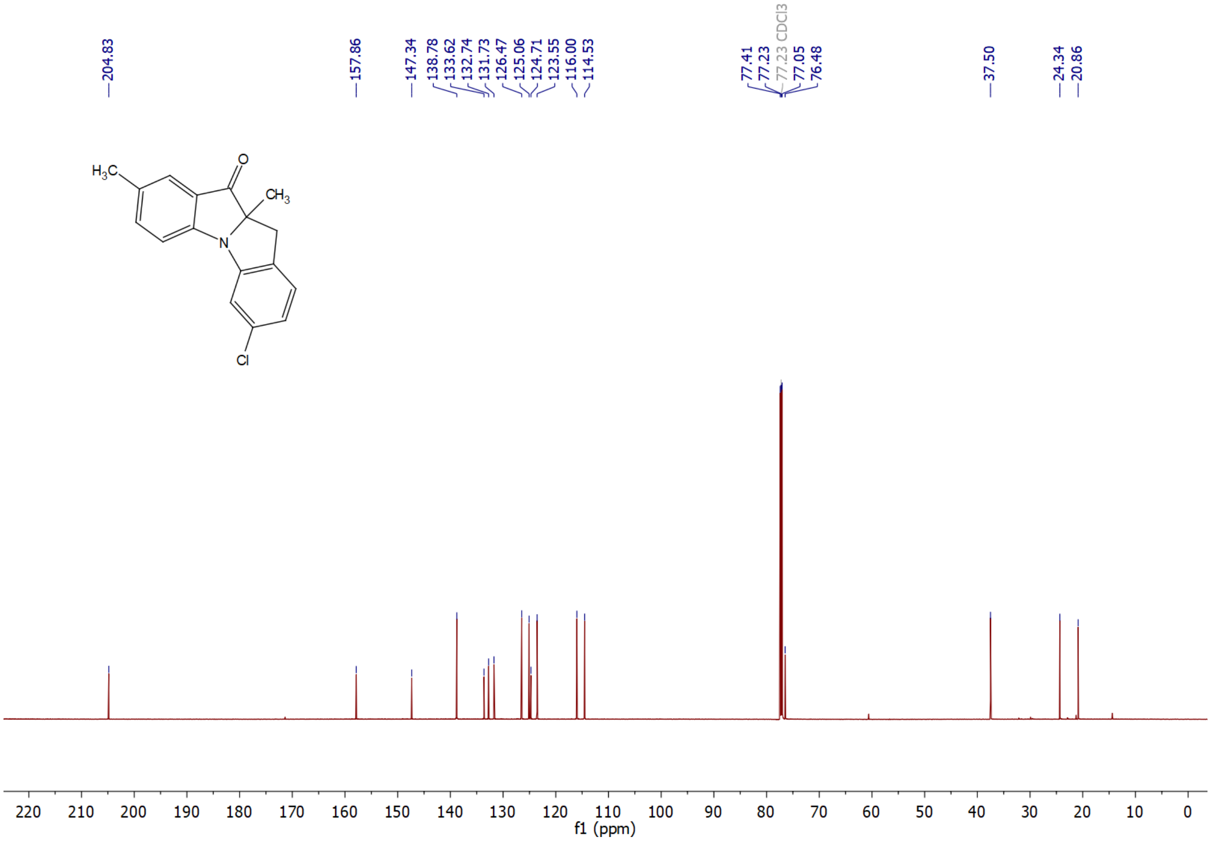

8-chloro-2-fluoro-10a-methyl-10a,11-dihydro-10H-indolo[1,2-a]indol-10-one [**F14**]:

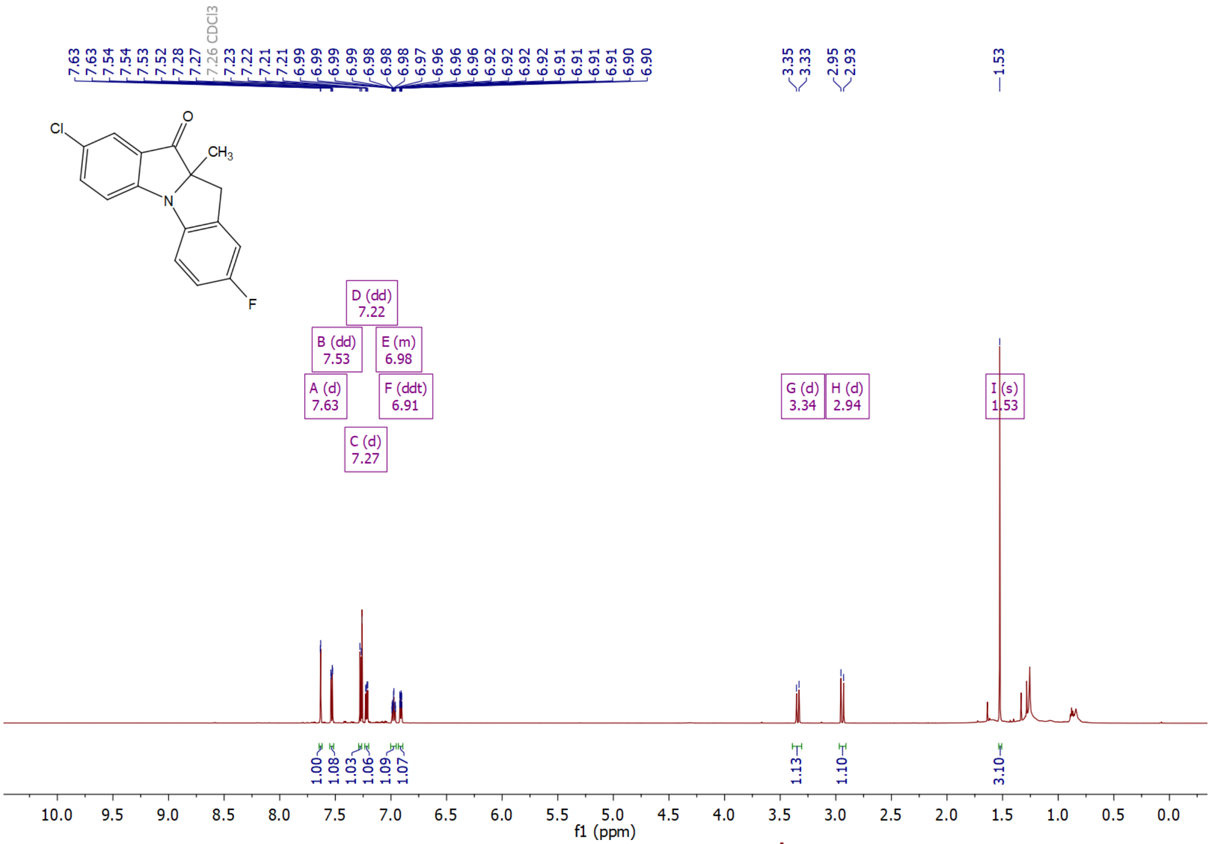

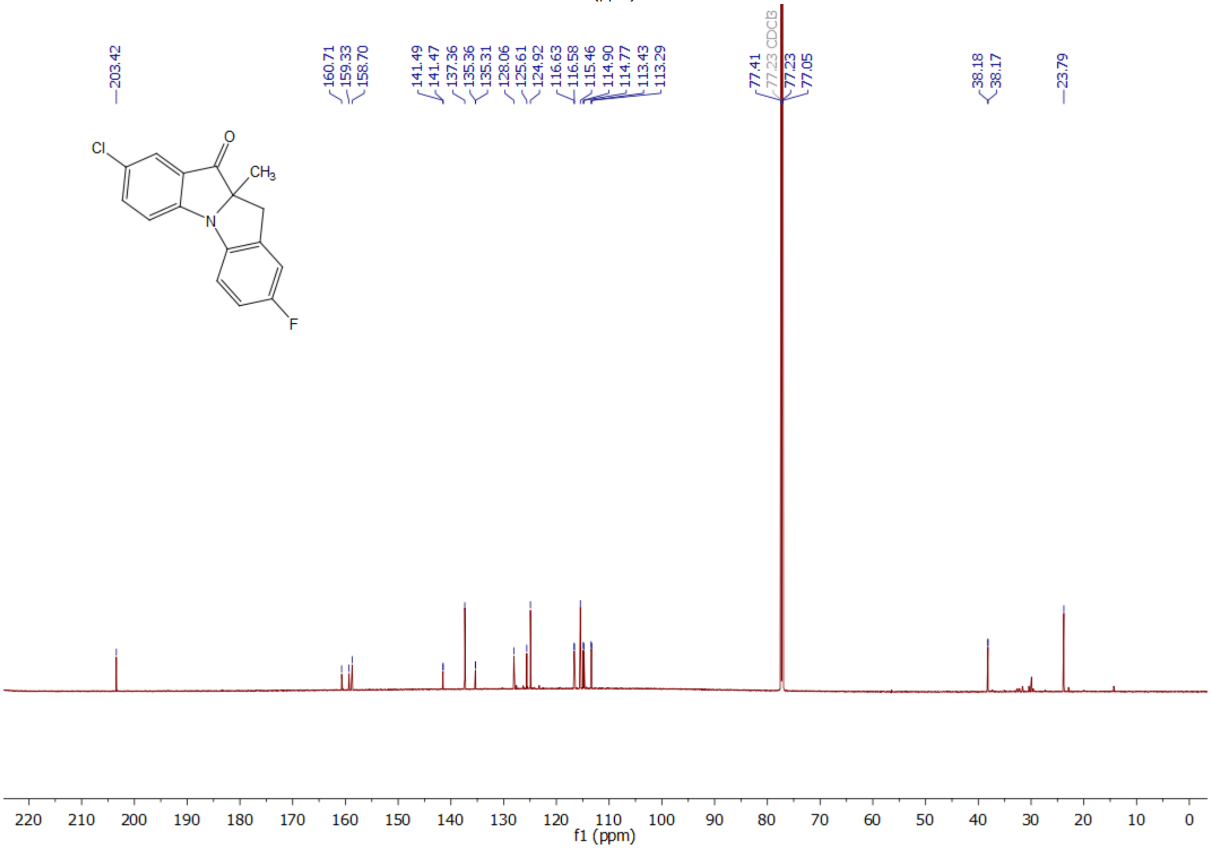

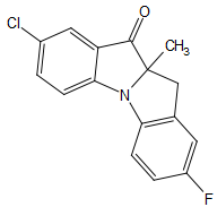

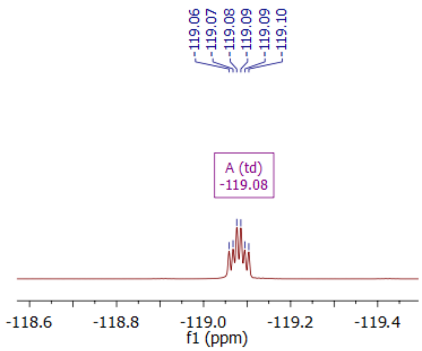

-119.06
-119.07
-119.08
-119.09
-119.09
-119.10
A (td)
-119.08
-118.6
-118.8
-119.0
-119.2
-119.4
f1 (ppm)

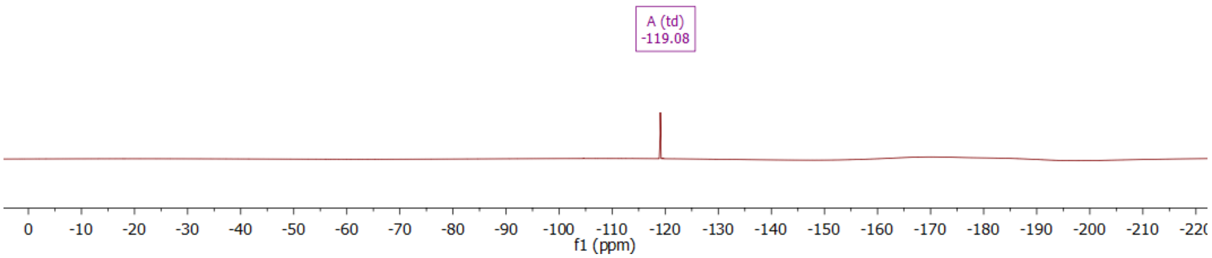

A (td)
-119.08
0
-10
-20
-30
-40
-50
-60
-70
-80
-90
-100
-110
-120
-130
-140
-150
-160
-170
-180
-190
-200
-210
-220
f1 (ppm)

1-fluoro-8,10a-dimethyl-10a,11-dihydro-10H-indolo[1,2-a]indol-10-one [**F15**]:

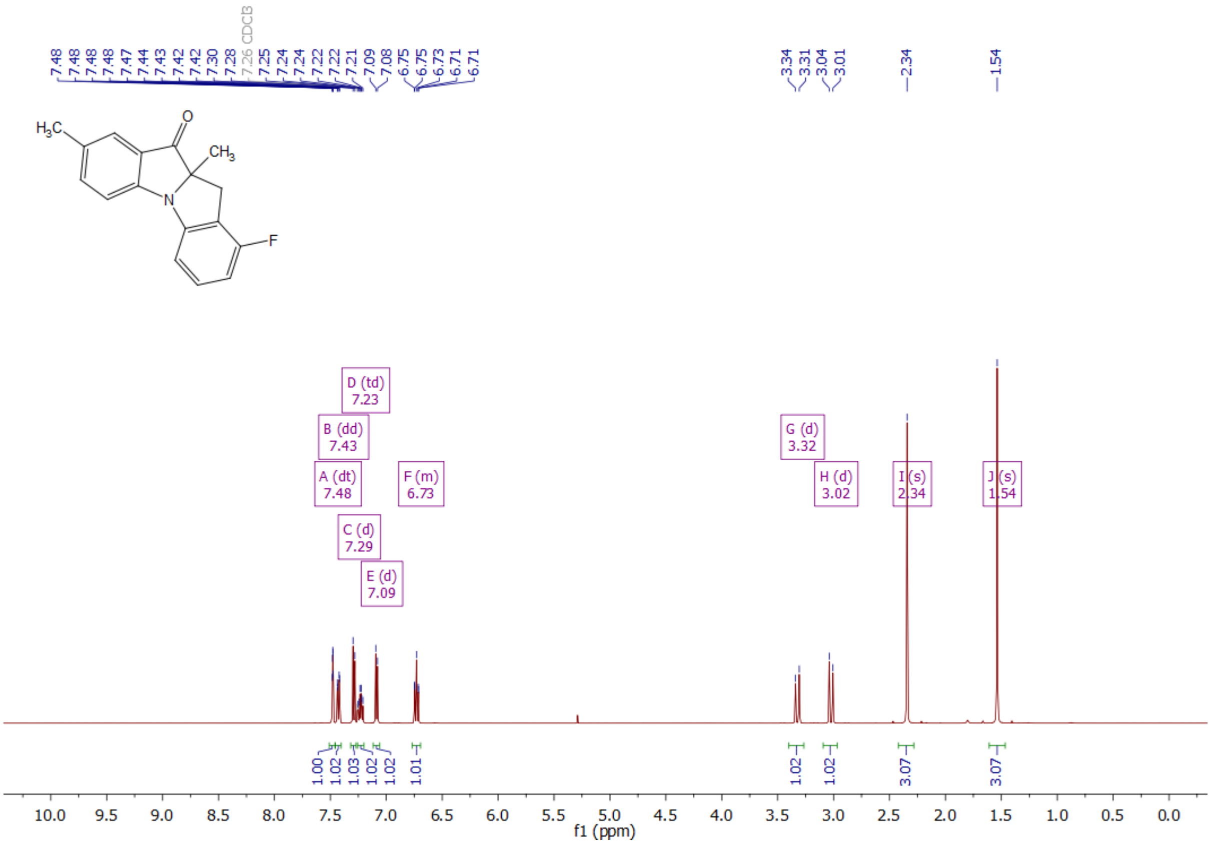

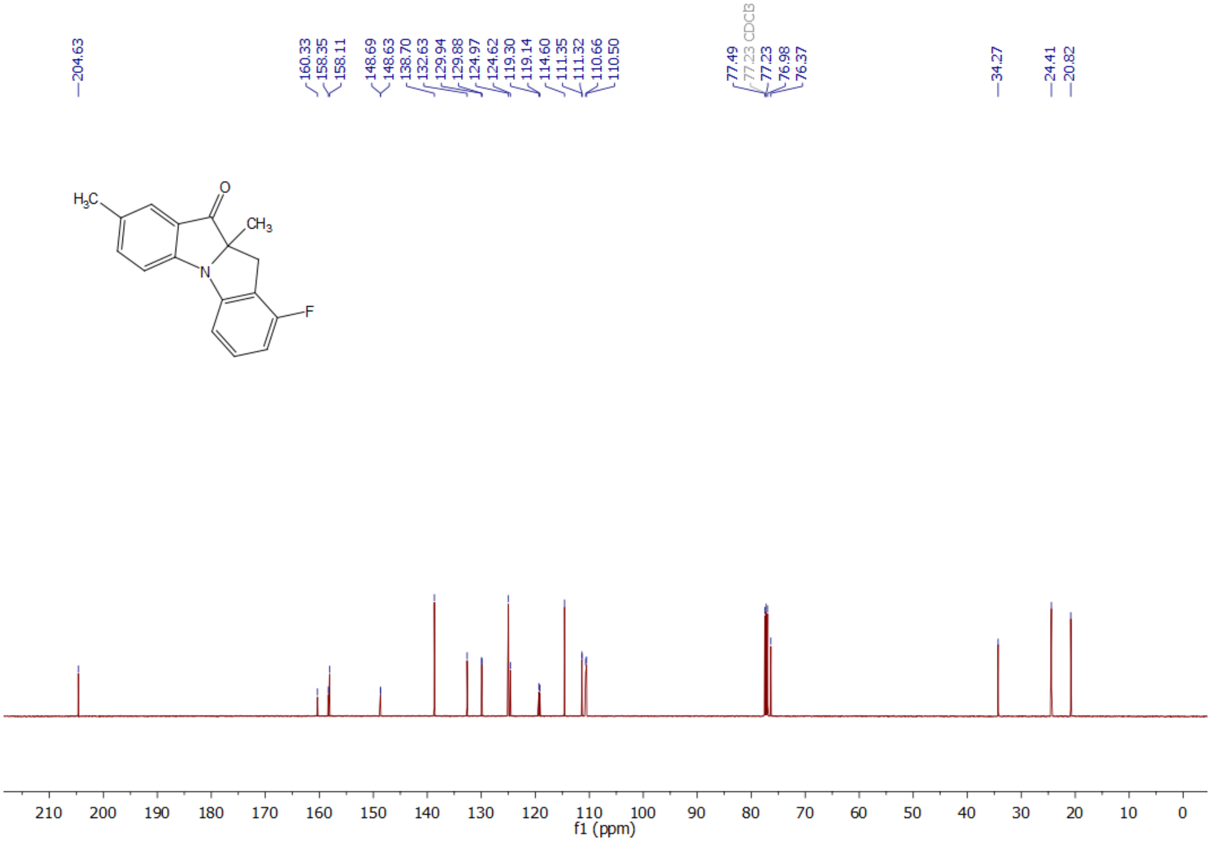

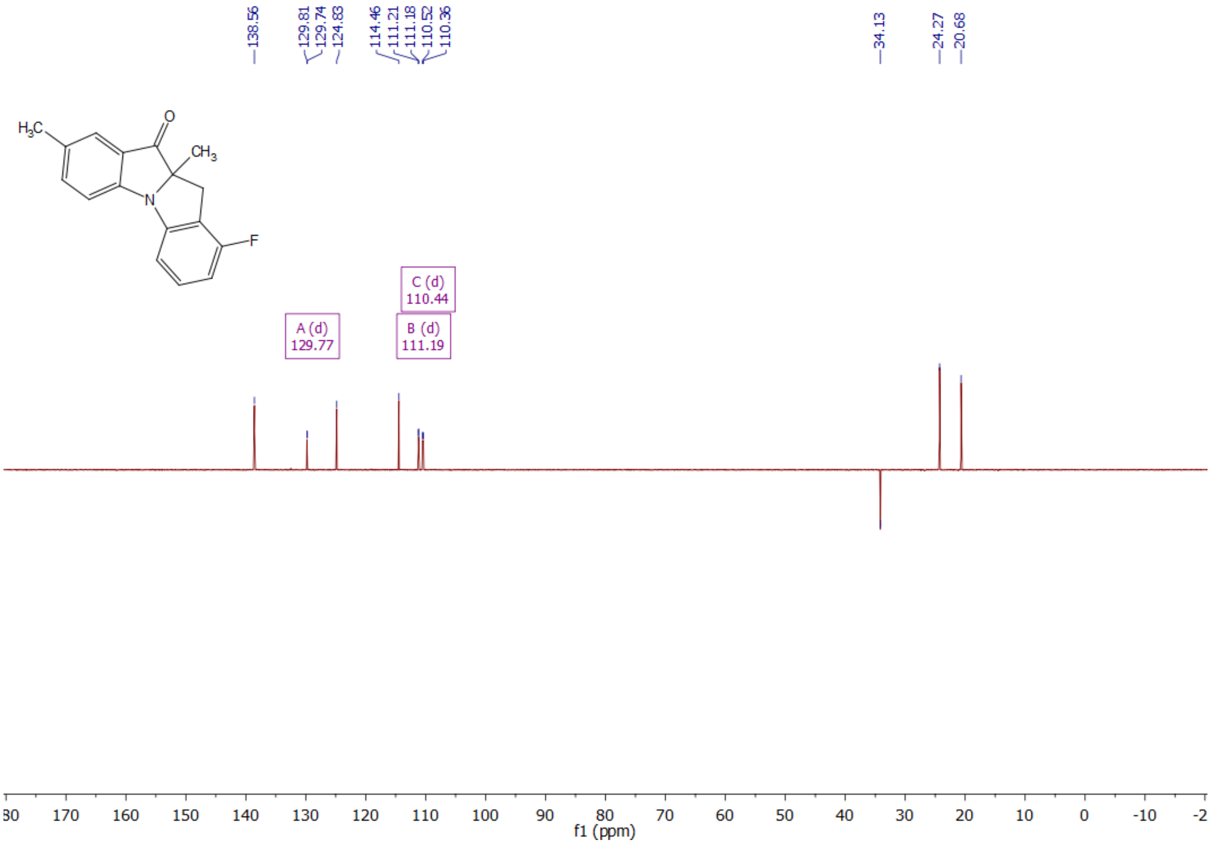
138.56
129.81
129.74
124.83
114.46
111.21
111.18
110.52
110.36
34.13
24.27
20.68
C (d)
110.44
A (d)
129.77
B (d)
111.19
f1 (ppm)

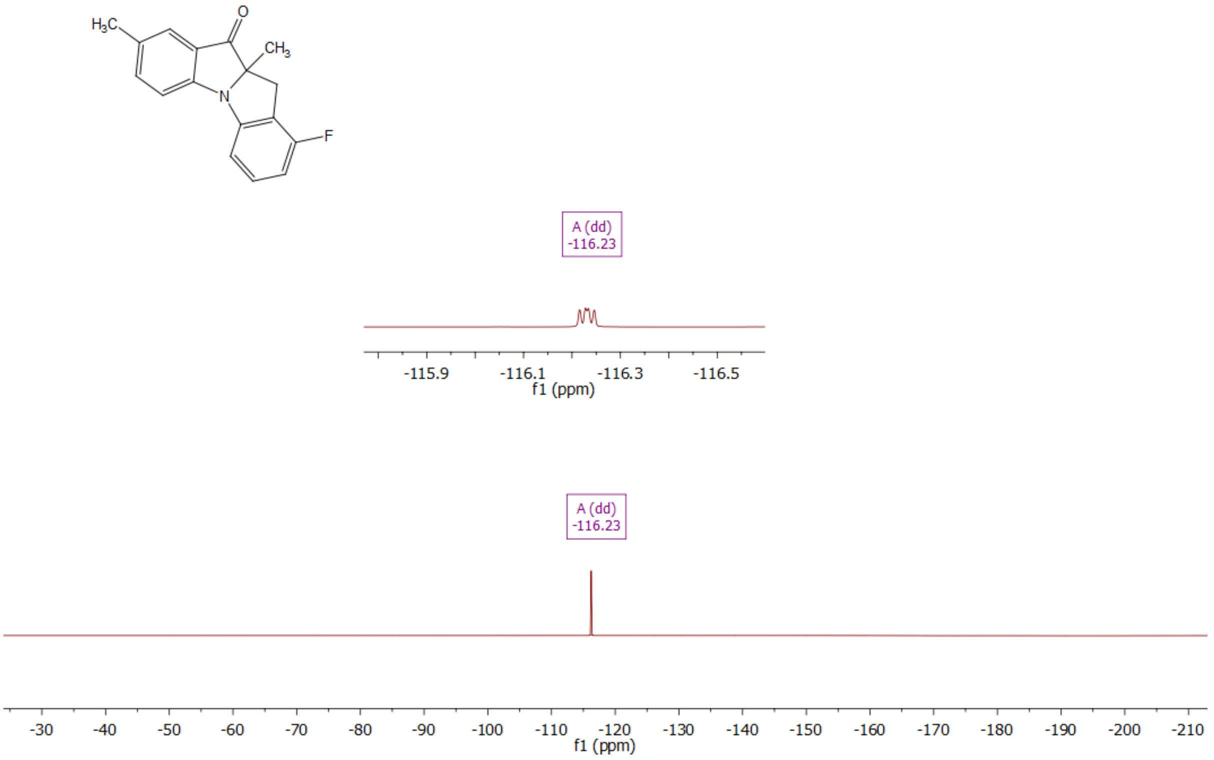
A (dd)
-116.23
-115.9
-116.1
-116.3
-116.5
f1 (ppm)
A (dd)
-116.23
f1 (ppm)

1-fluoro-8,10a-dimethyl-10a,11-dihydro-10H-indolo[1,2-a]indol-10-one [**F15-resynthesized**]:

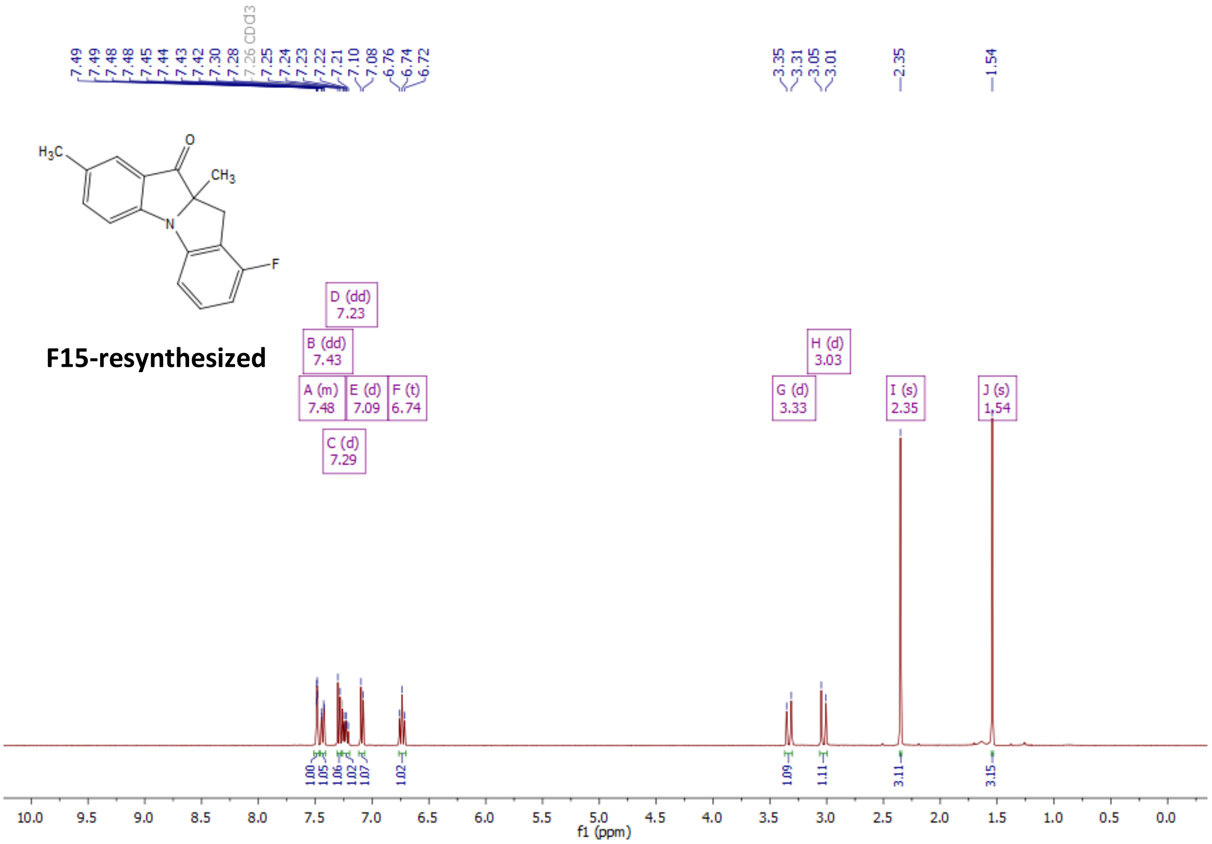

8,10a-dimethyl-10a,11-dihydro-10H-[1,3]dioxolo[4,5-f]indolo[1,2-a]indol-10-one [**F16**]:

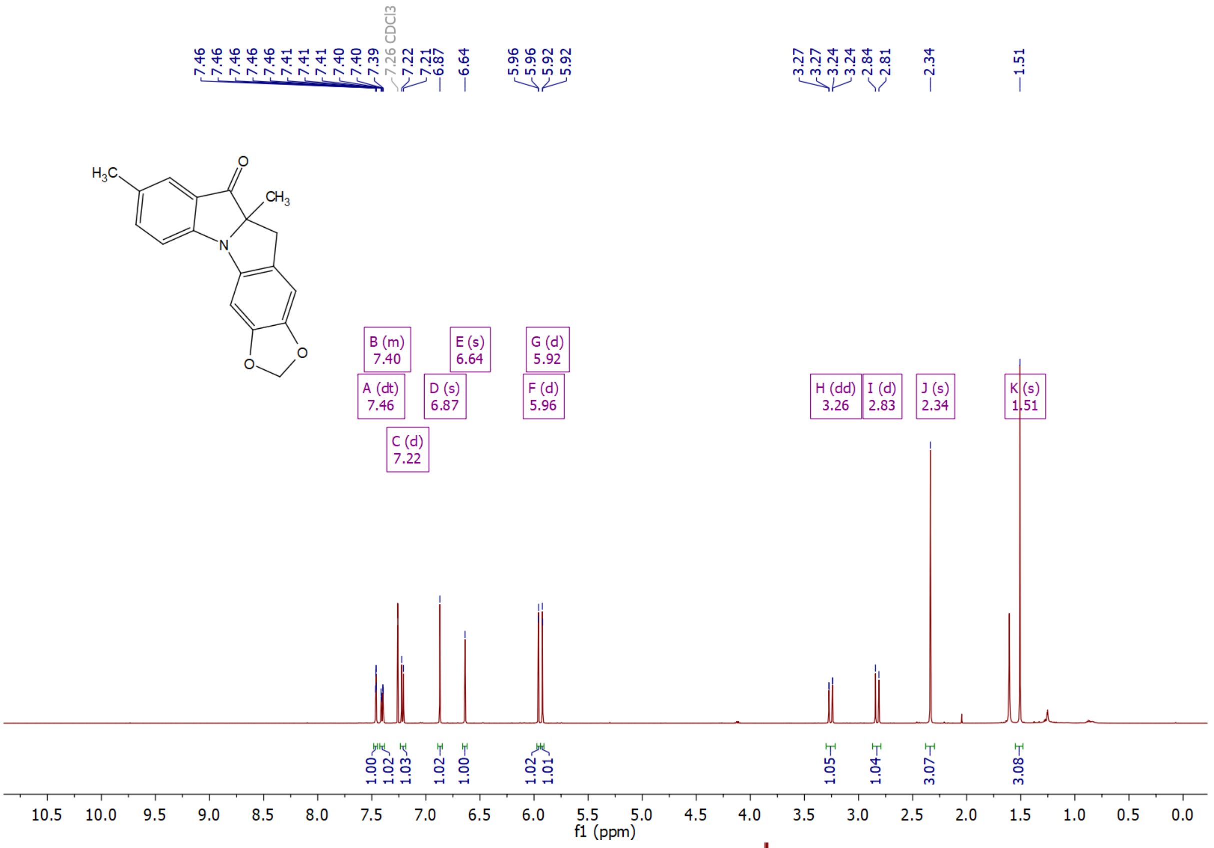

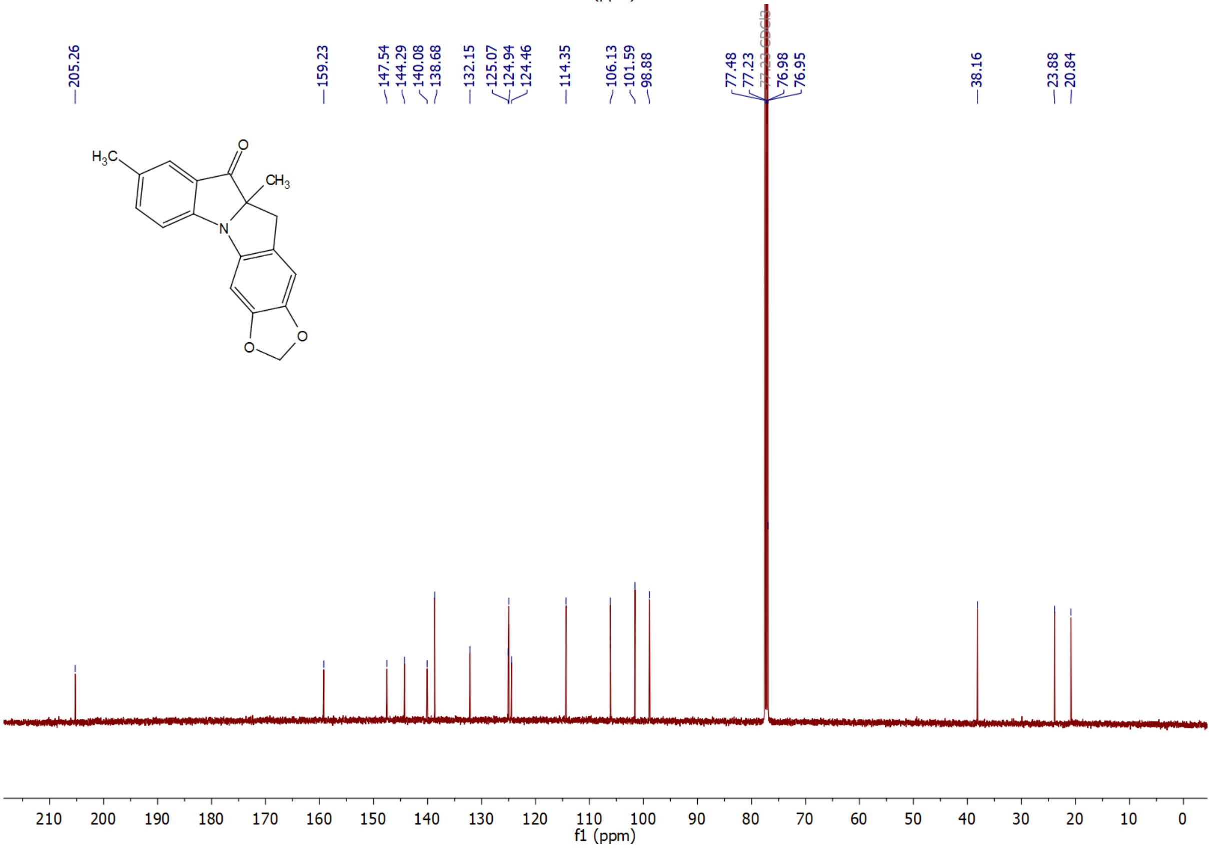

***10a-phenyl-10a,11-dihydro-10H-indolo[1,2-a]indol-10-one* [F17]**:

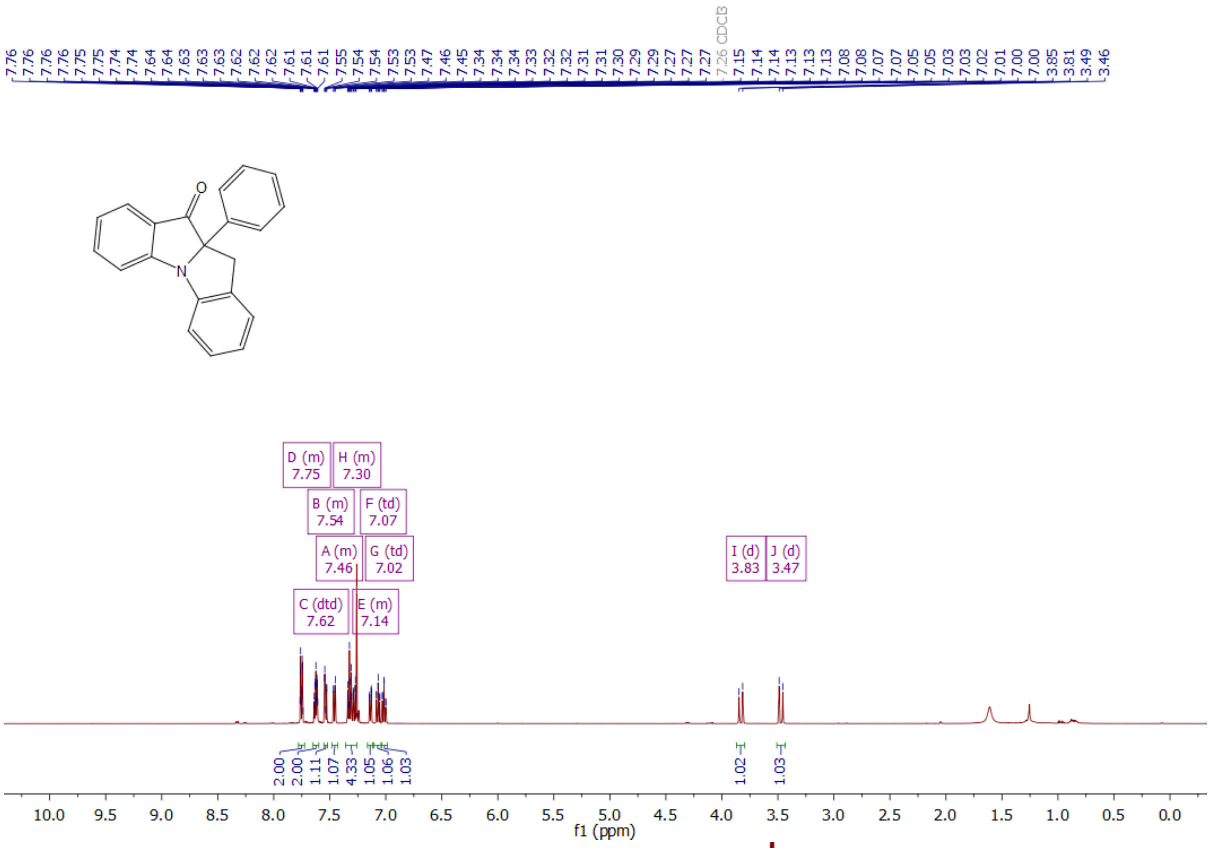

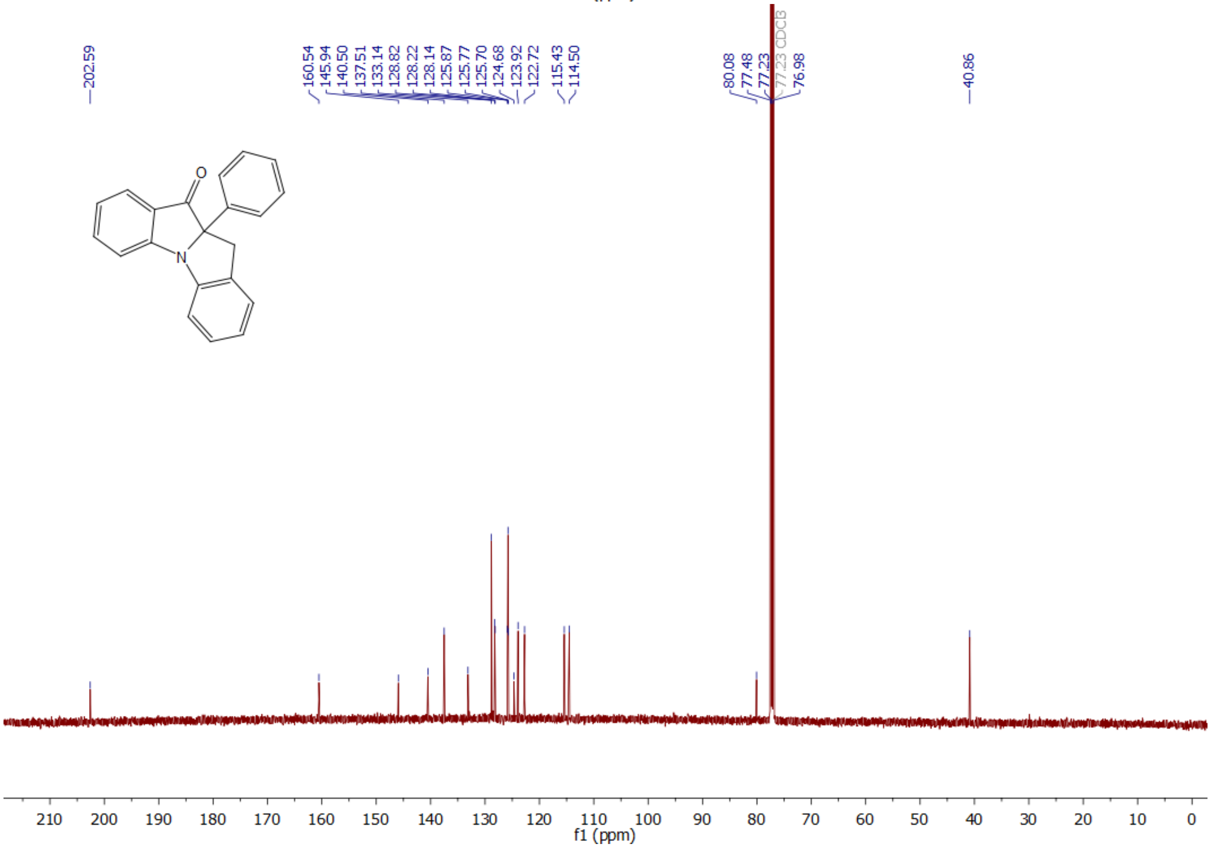

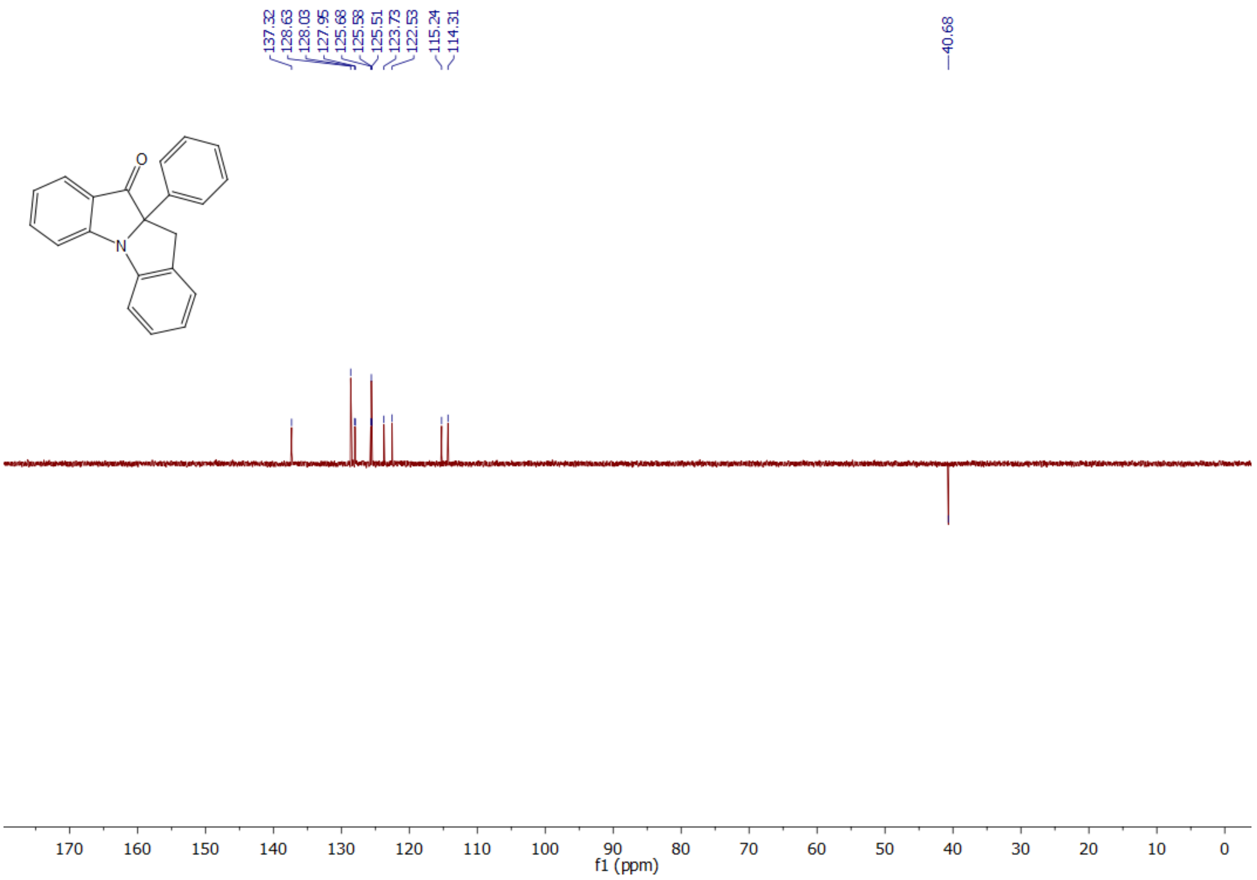
137.32
128.63
128.03
127.95
125.68
125.58
125.51
123.73
122.53
115.24
114.31
40.68
O
N
170
160
150
140
130
120
110
100
90
80
70
60
50
40
30
20
10
0
f1 (ppm)

3-fluoro-10a-phenyl-10a,11-dihydro-10H-indolo[1,2-a]indol-10-one **[F18]**:

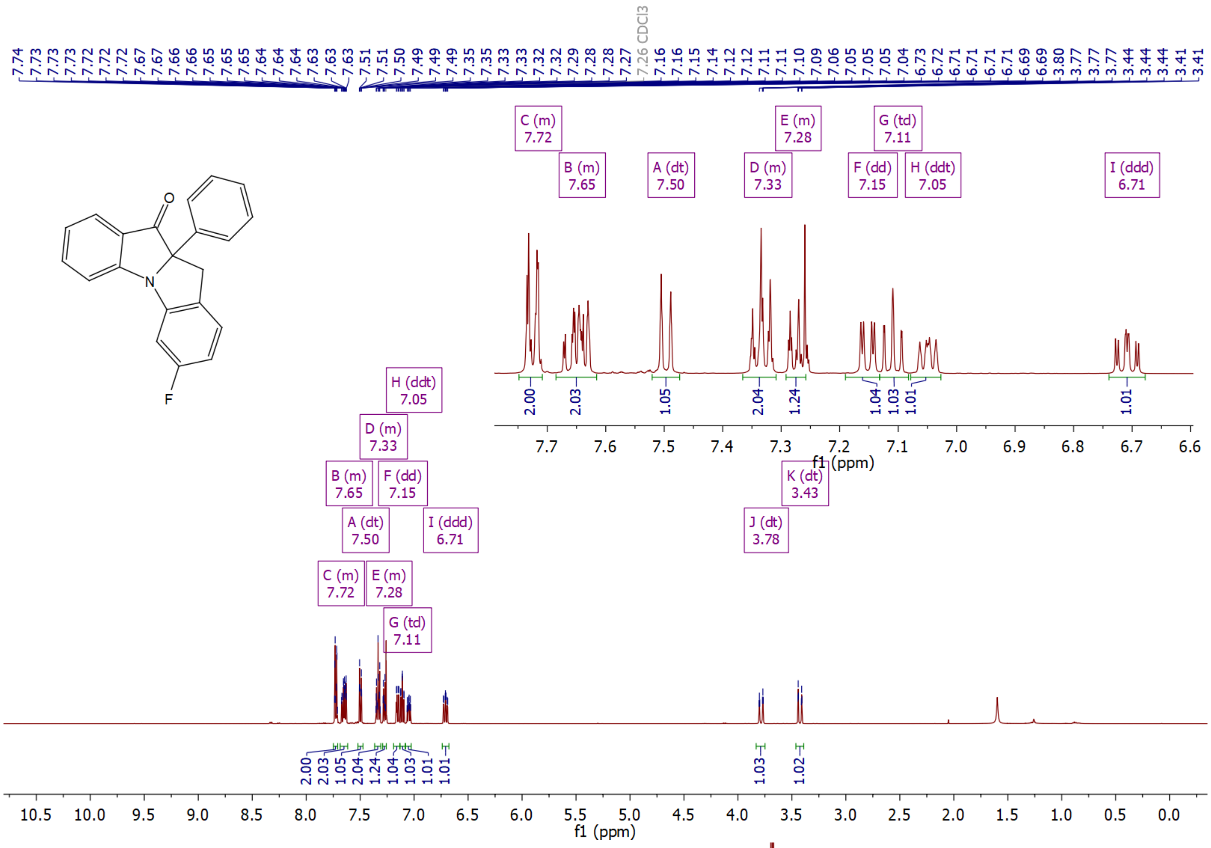

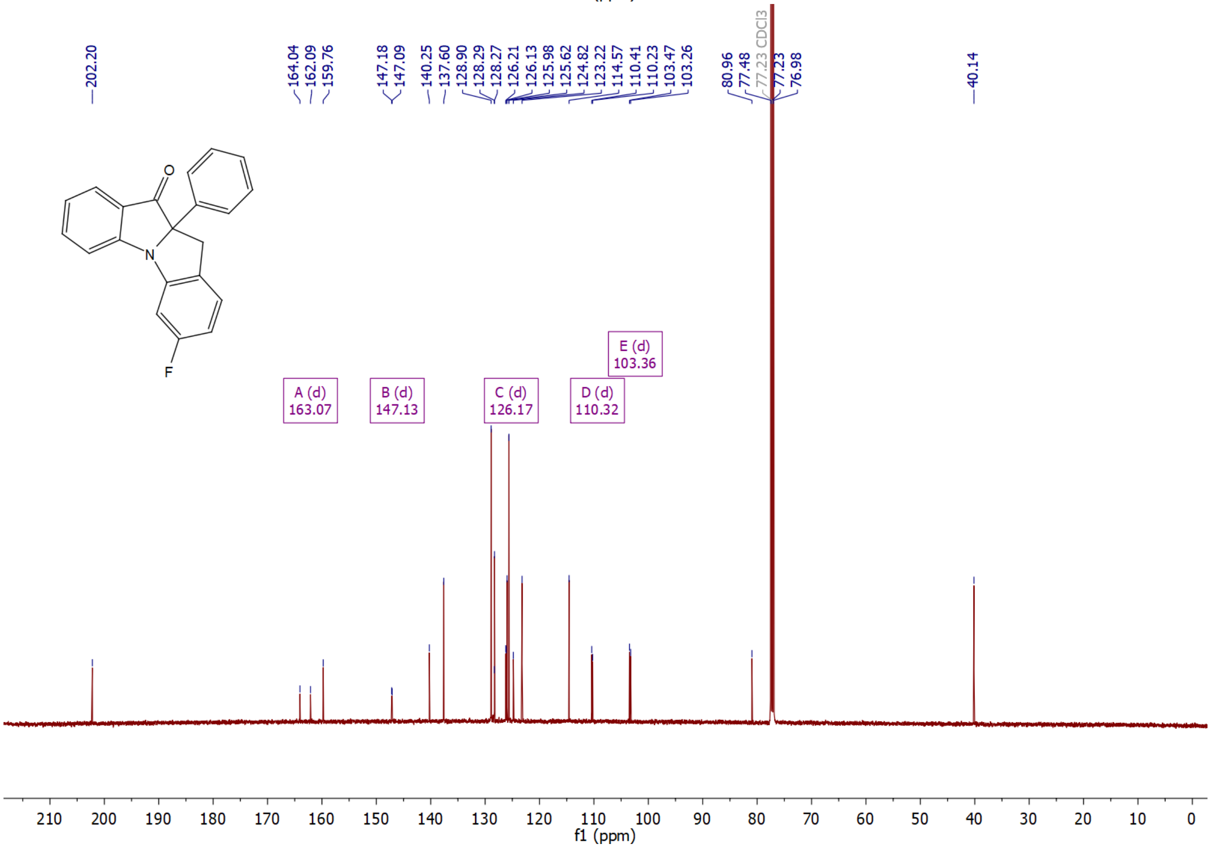

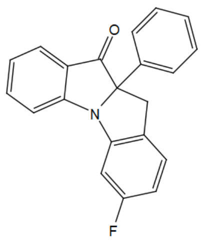

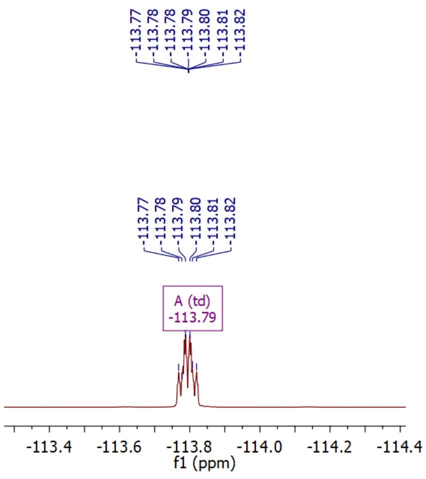

-113.77
-113.78
-113.78
-113.79
-113.80
-113.81
-113.82
-113.77
-113.78
-113.79
-113.80
-113.81
-113.82
A (td)
-113.79
-113.4 -113.6 -113.8 -114.0 -114.2 -114.4
f1 (ppm)

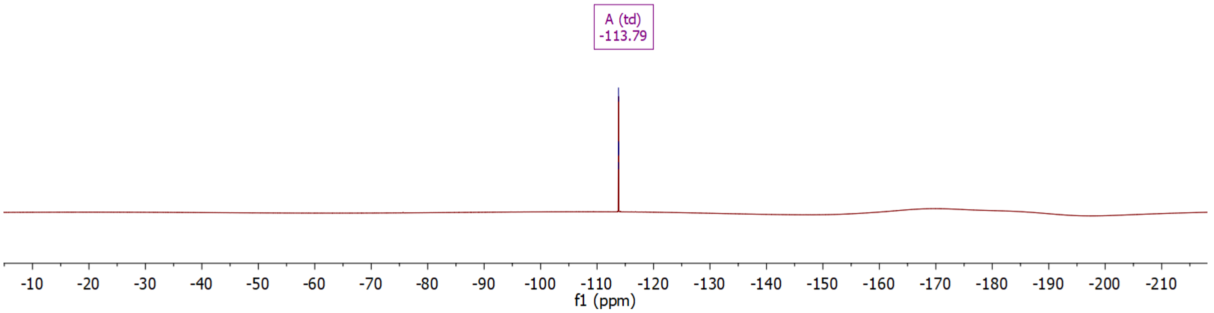

A (td)
-113.79
-10 -20 -30 -40 -50 -60 -70 -80 -90 -100 -110 -120 -130 -140 -150 -160 -170 -180 -190 -200 -210
f1 (ppm)

3-chloro-10a-phenyl-10a,11-dihydro-10H-indolo[1,2-a]indol-10-one [**F19**]:

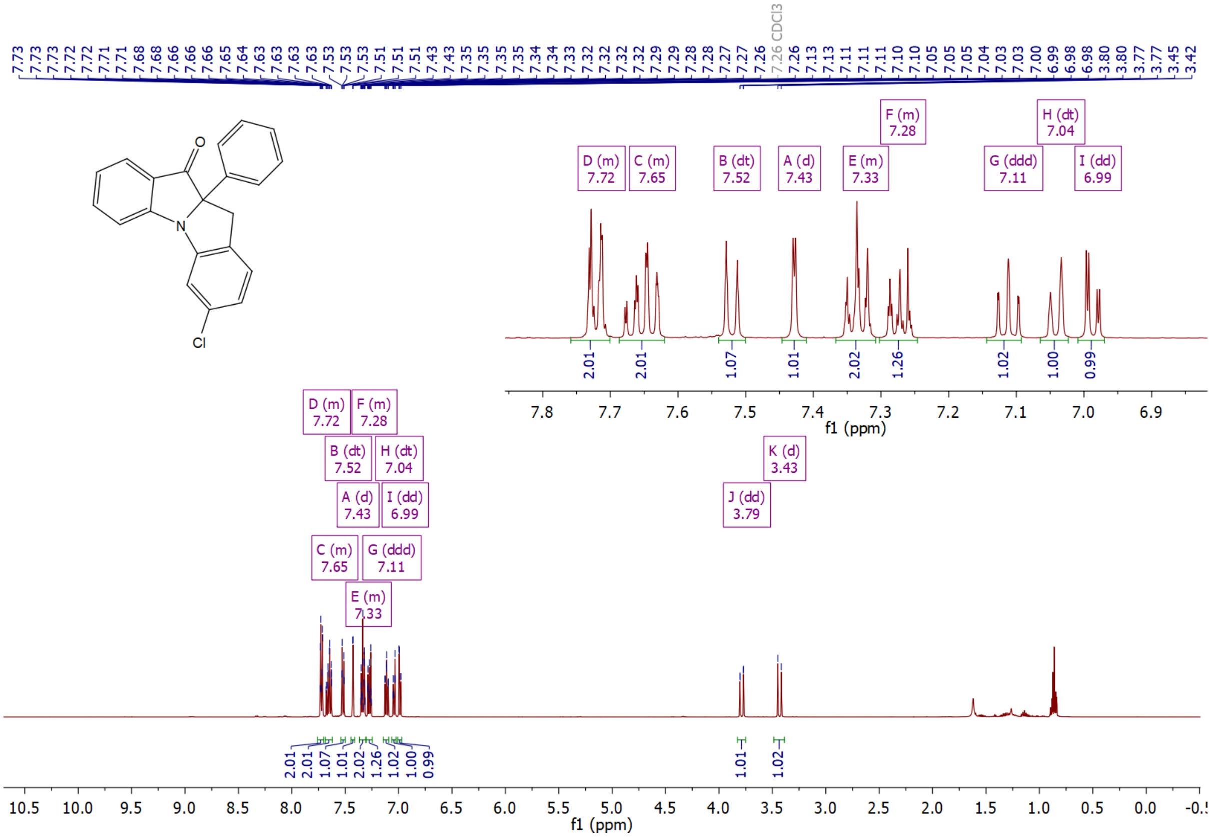

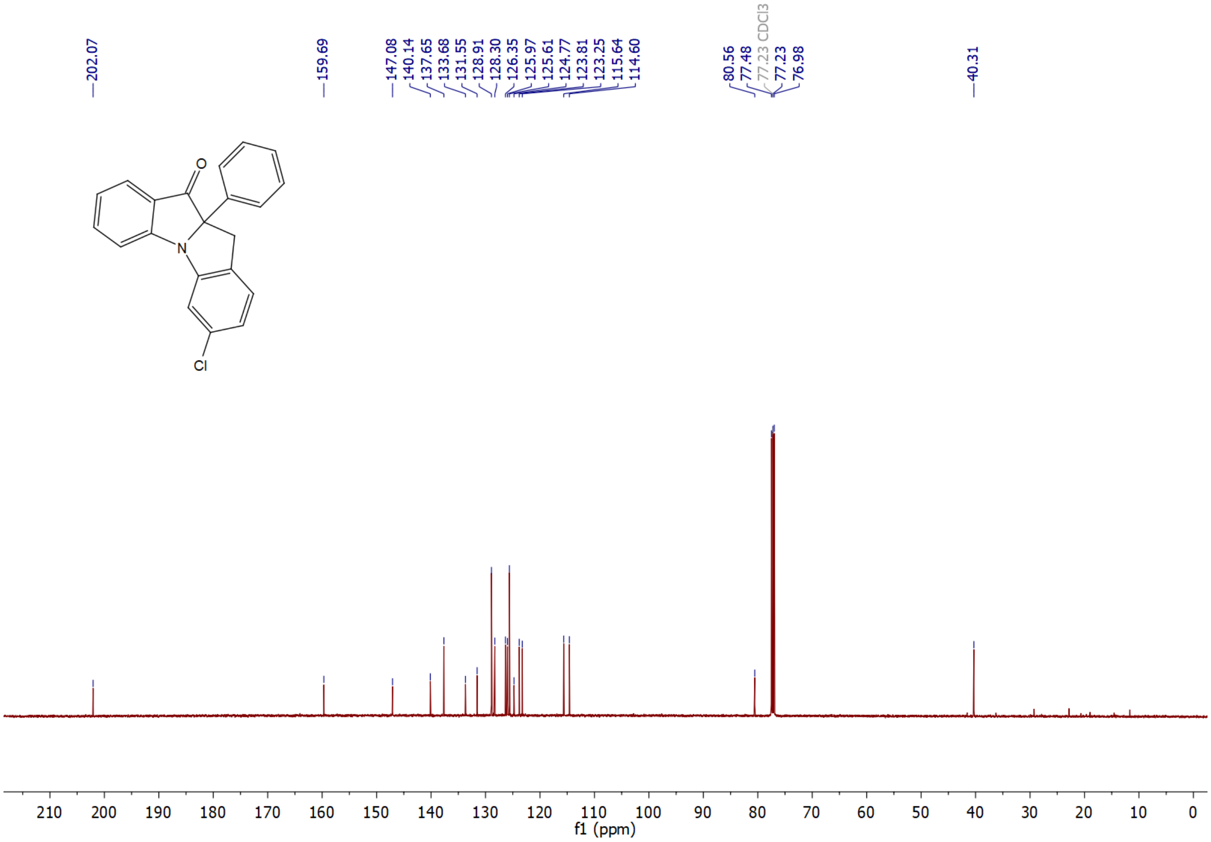

3-methyl-10a-phenyl-10a,11-dihydro-10H-indolo[1,2-a]indol-10-one [**F20**]:

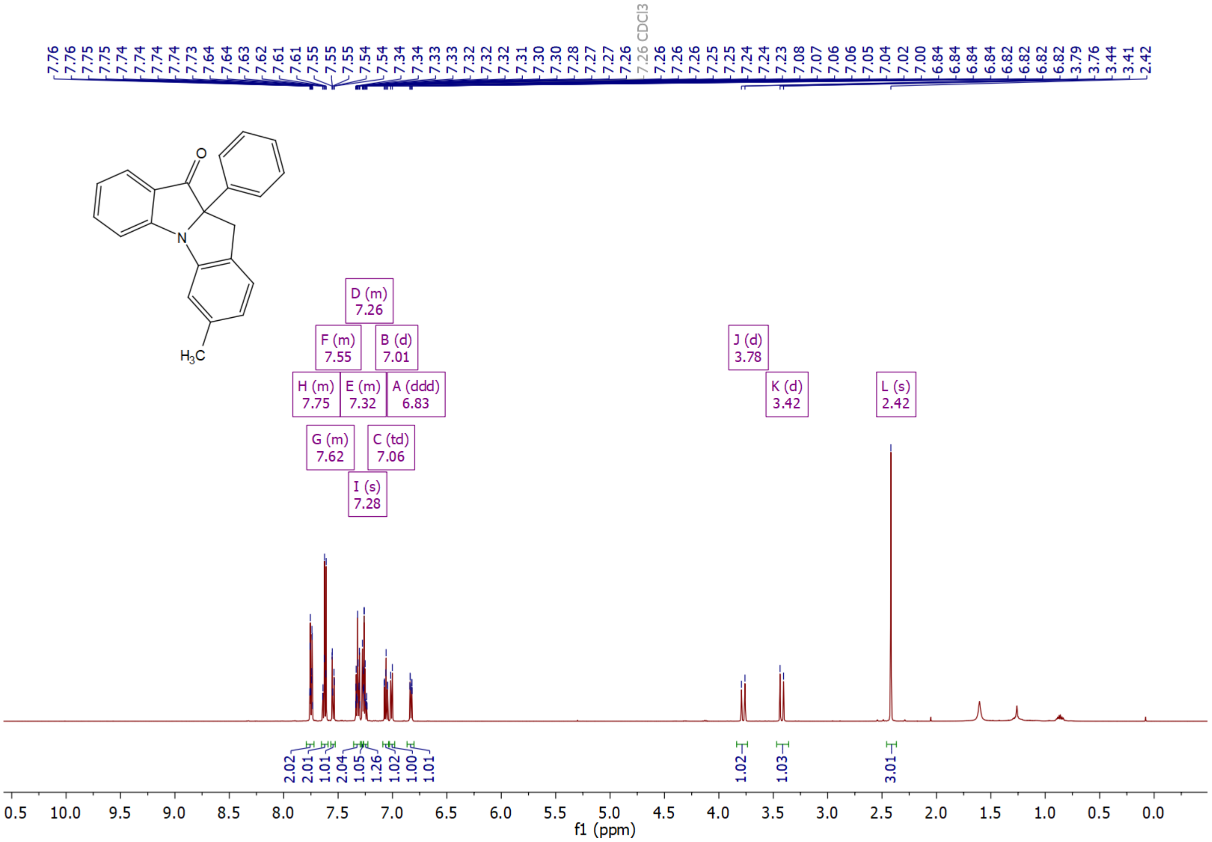

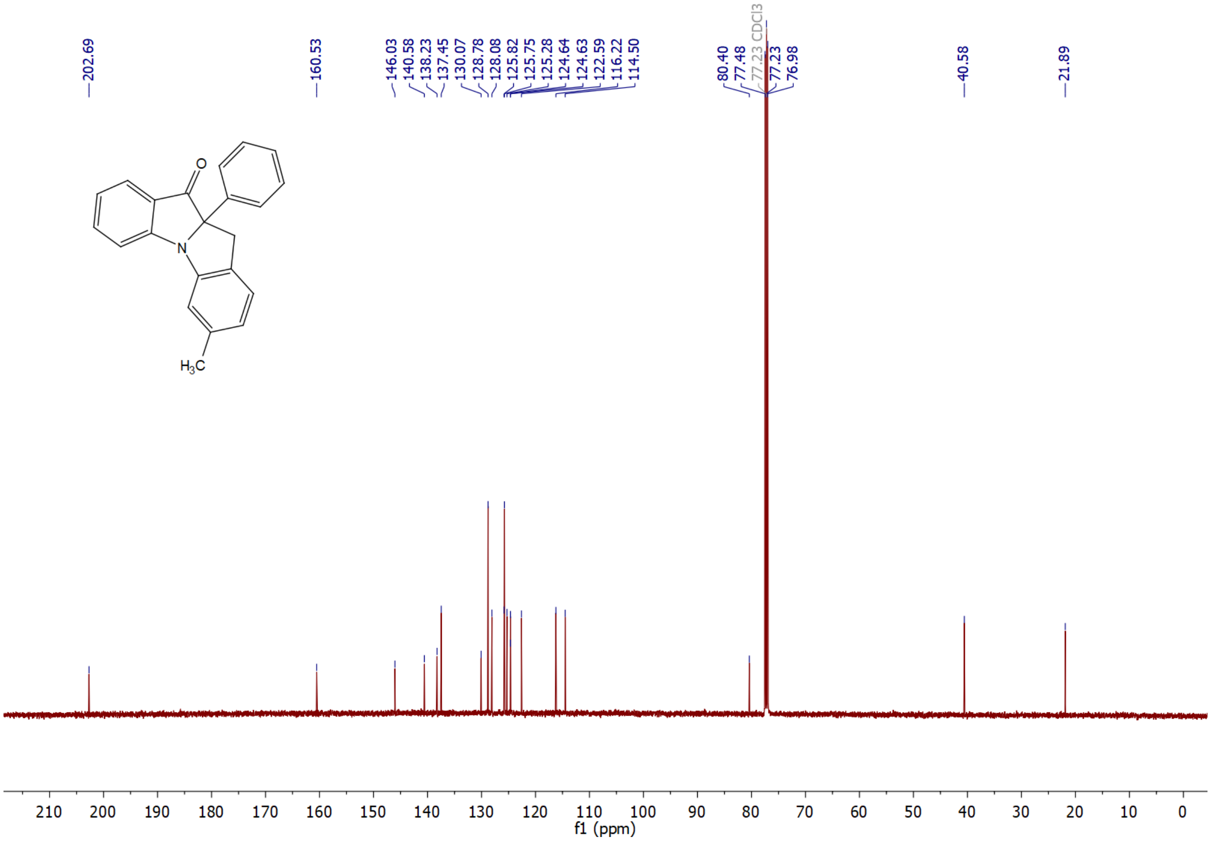

2-fluoro-10a-phenyl-10a,11-dihydro-10H-indolo[1,2-a]indol-10-one [**F21**]:

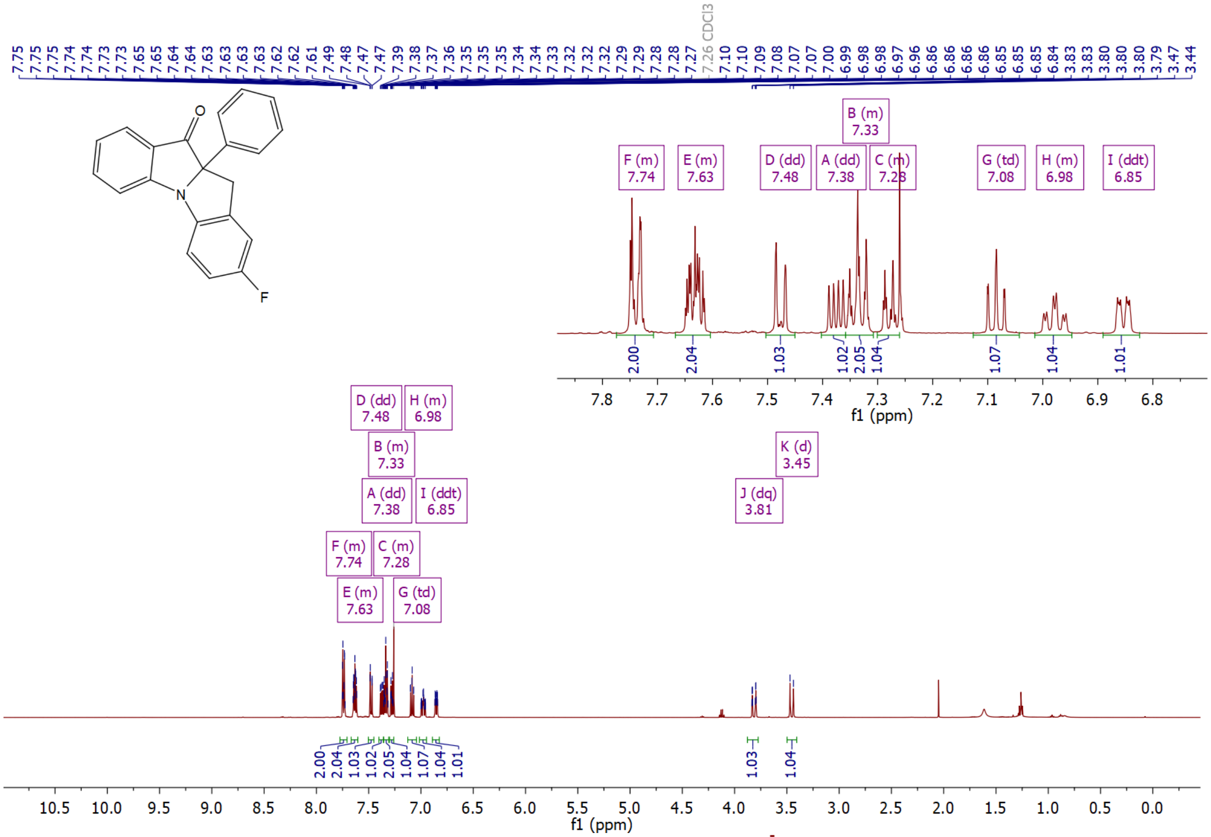

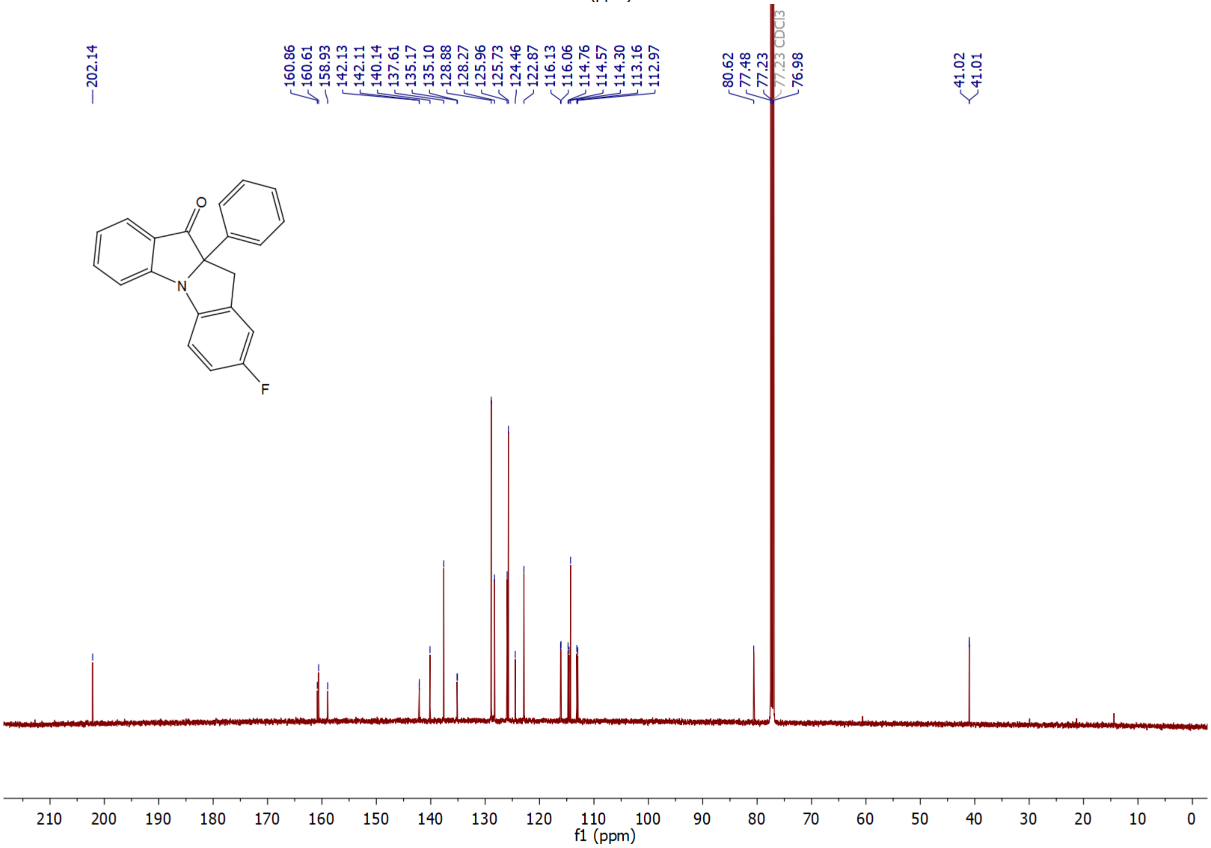

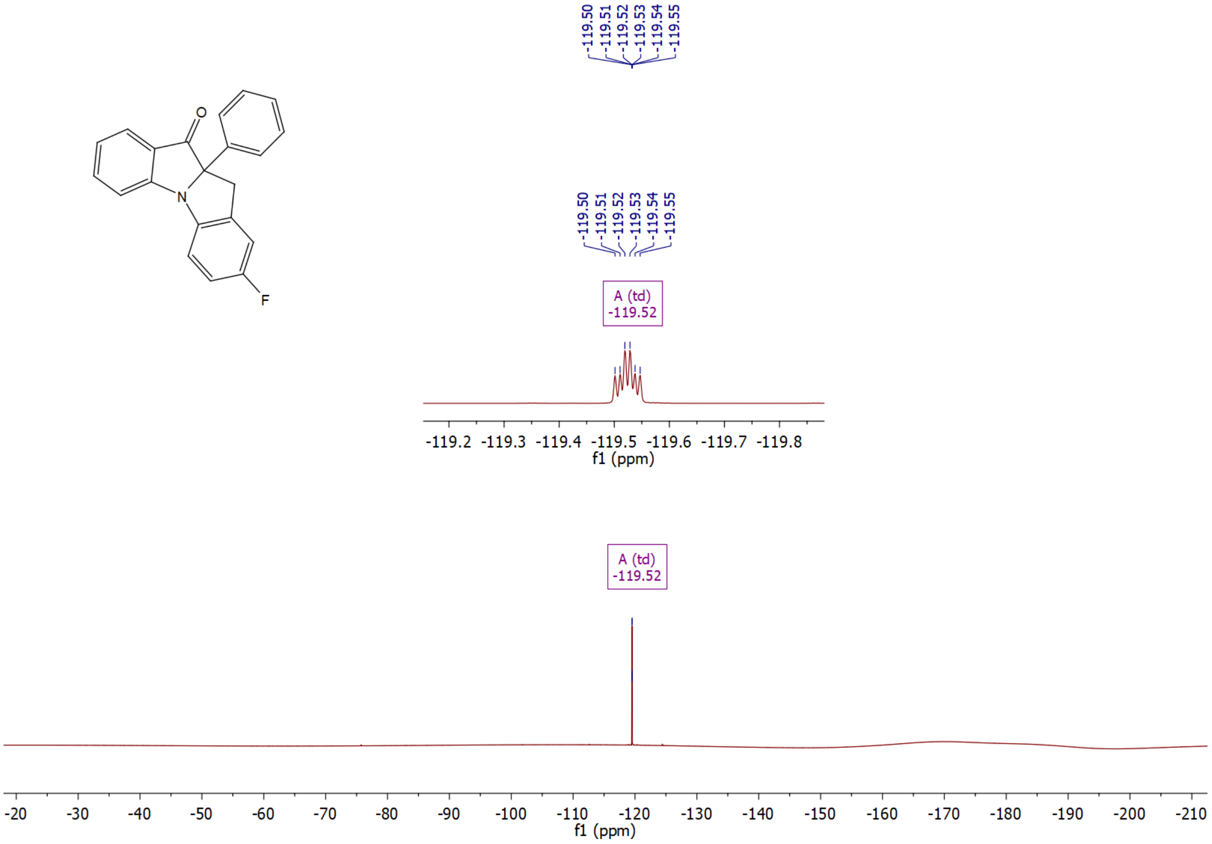
O
N
F
-119.50
-119.51
-119.52
-119.53
-119.54
-119.55
A (td)
-119.52
-119.2 -119.3 -119.4 -119.5 -119.6 -119.7 -119.8
f1 (ppm)
-20 -30 -40 -50 -60 -70 -80 -90 -100 -110 -120 -130 -140 -150 -160 -170 -180 -190 -200 -210
f1 (ppm)

10a-phenyl-10a,11-dihydro-10H-[1,3]dioxolo[4,5-f]indolo[1,2-a]indol-10-one **[F22]**:

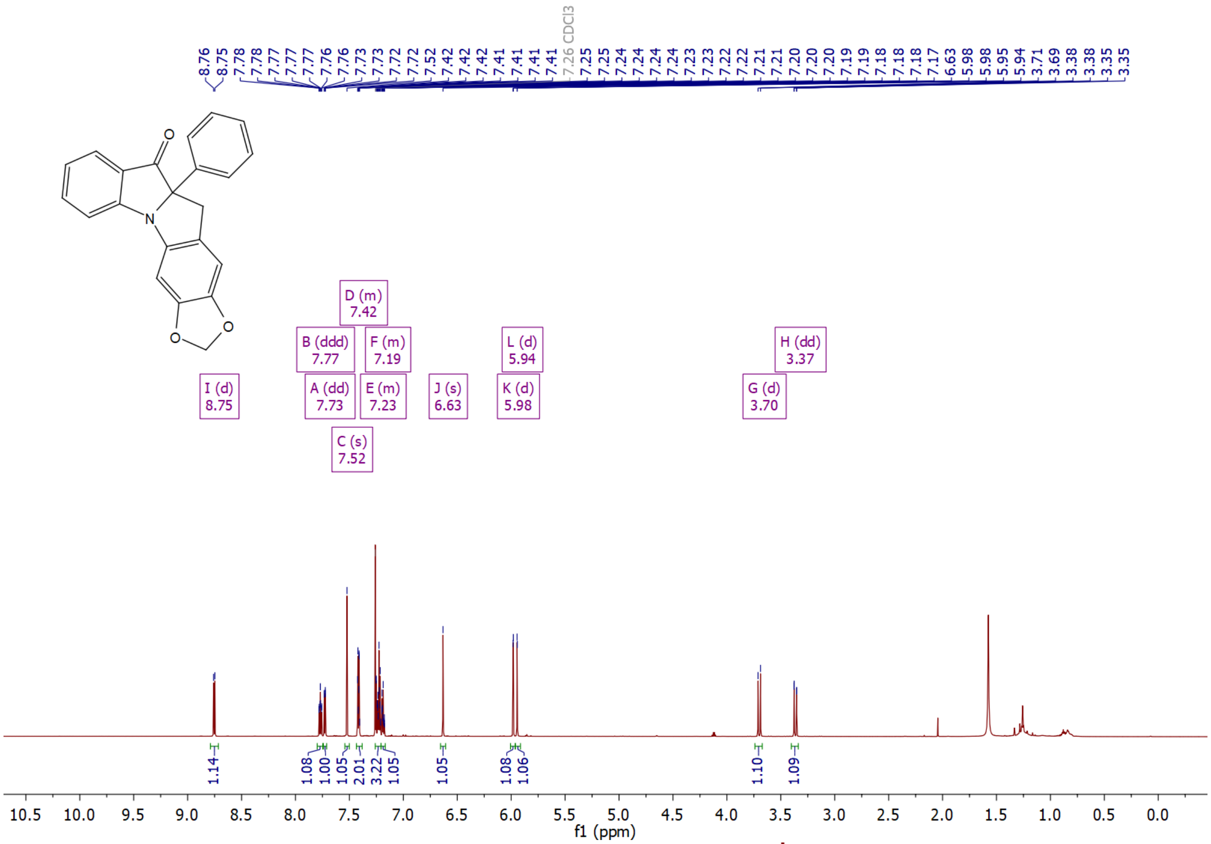

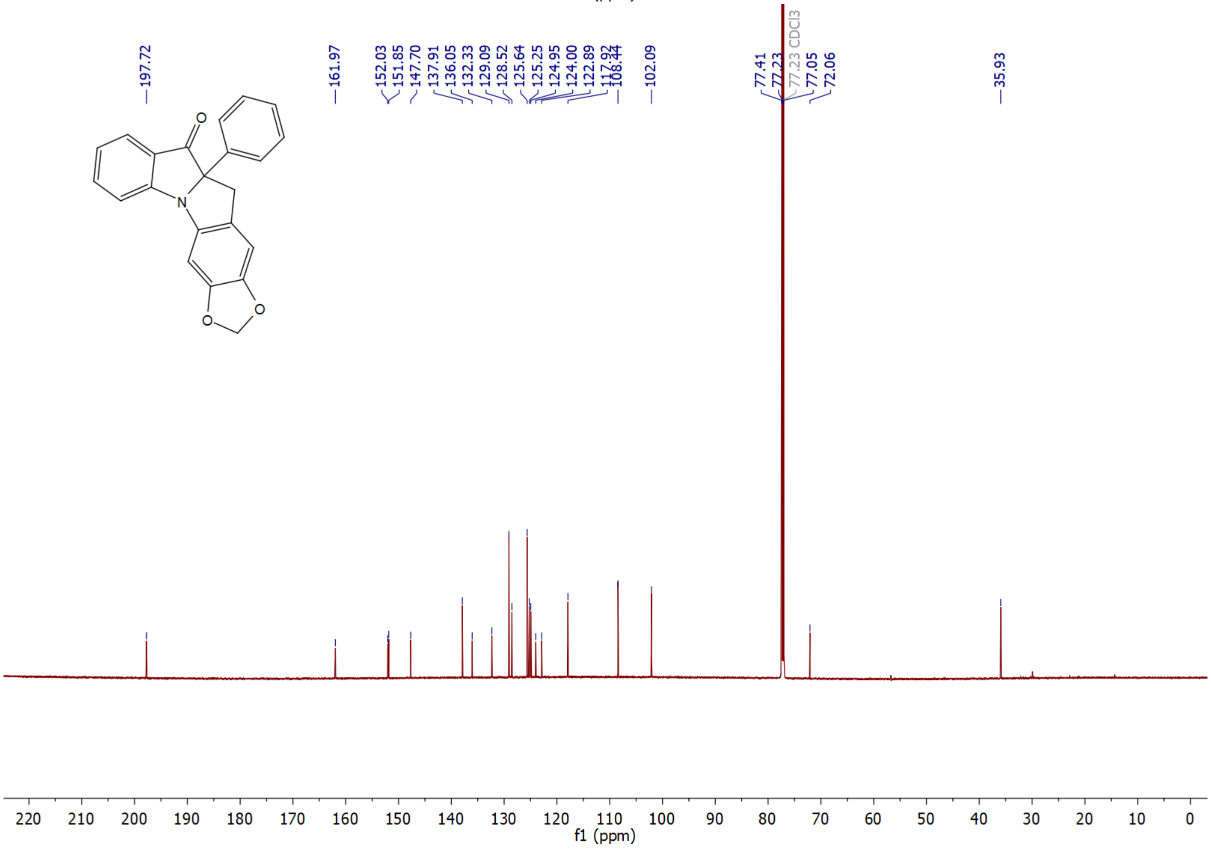

10a-(4-chlorophenyl)-10a,11-dihydro-10H-indolo[1,2-a]indol-10-one [**F23**]:

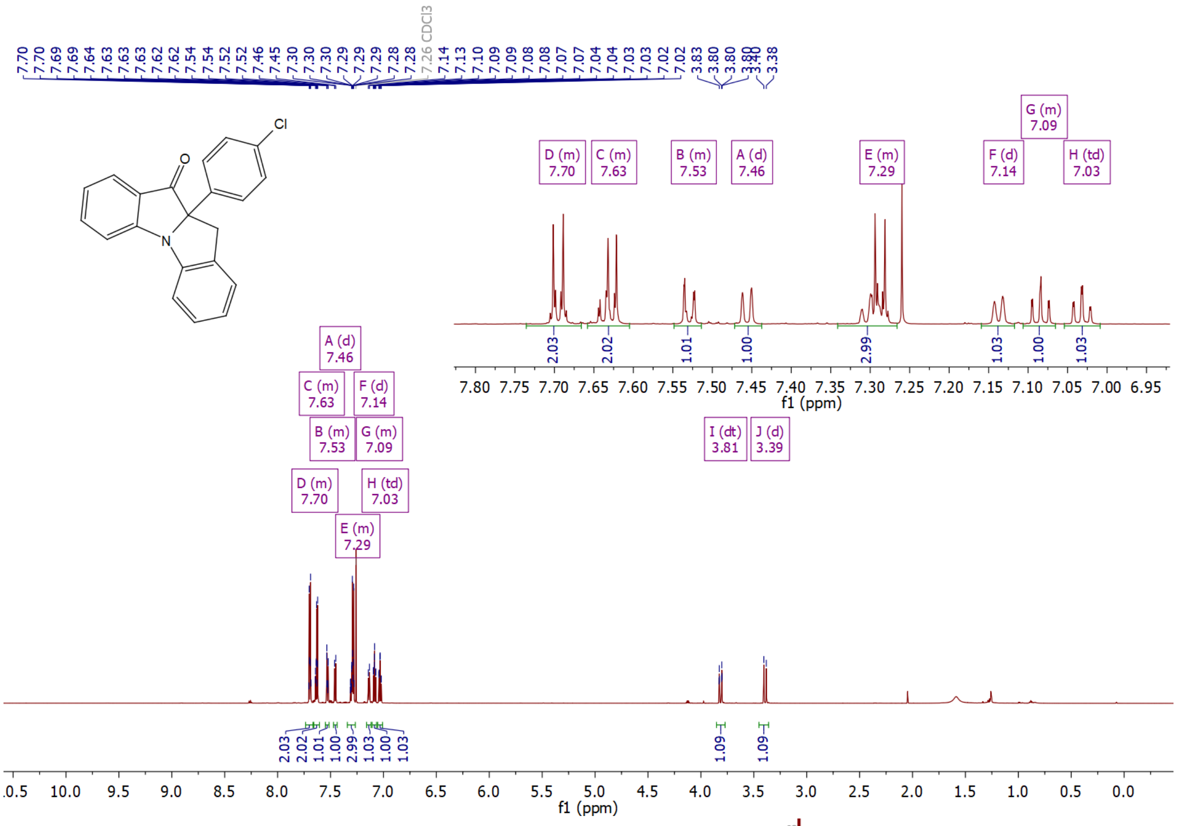

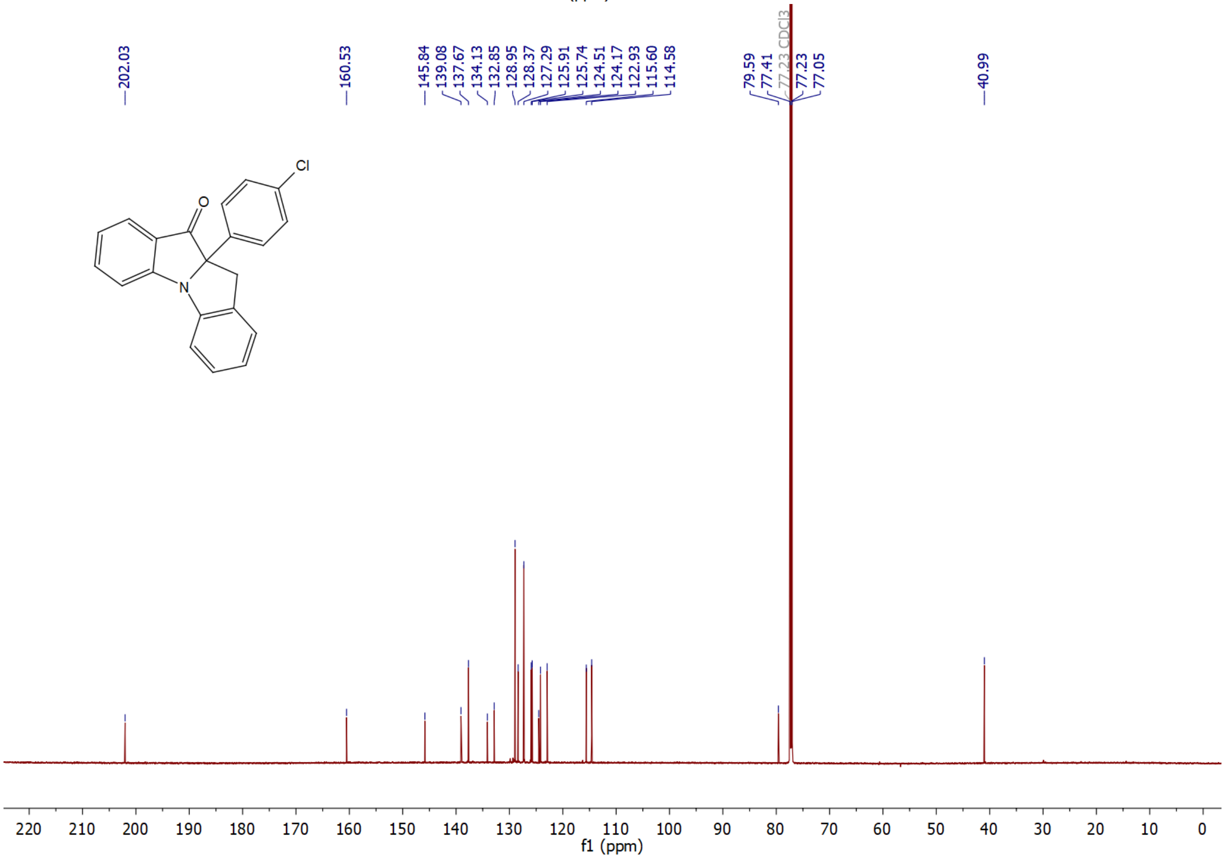

10a-(4-chlorophenyl)-2-fluoro-10a,11-dihydro-10H-indolo[1,2-a]indol-10-one [F24]:

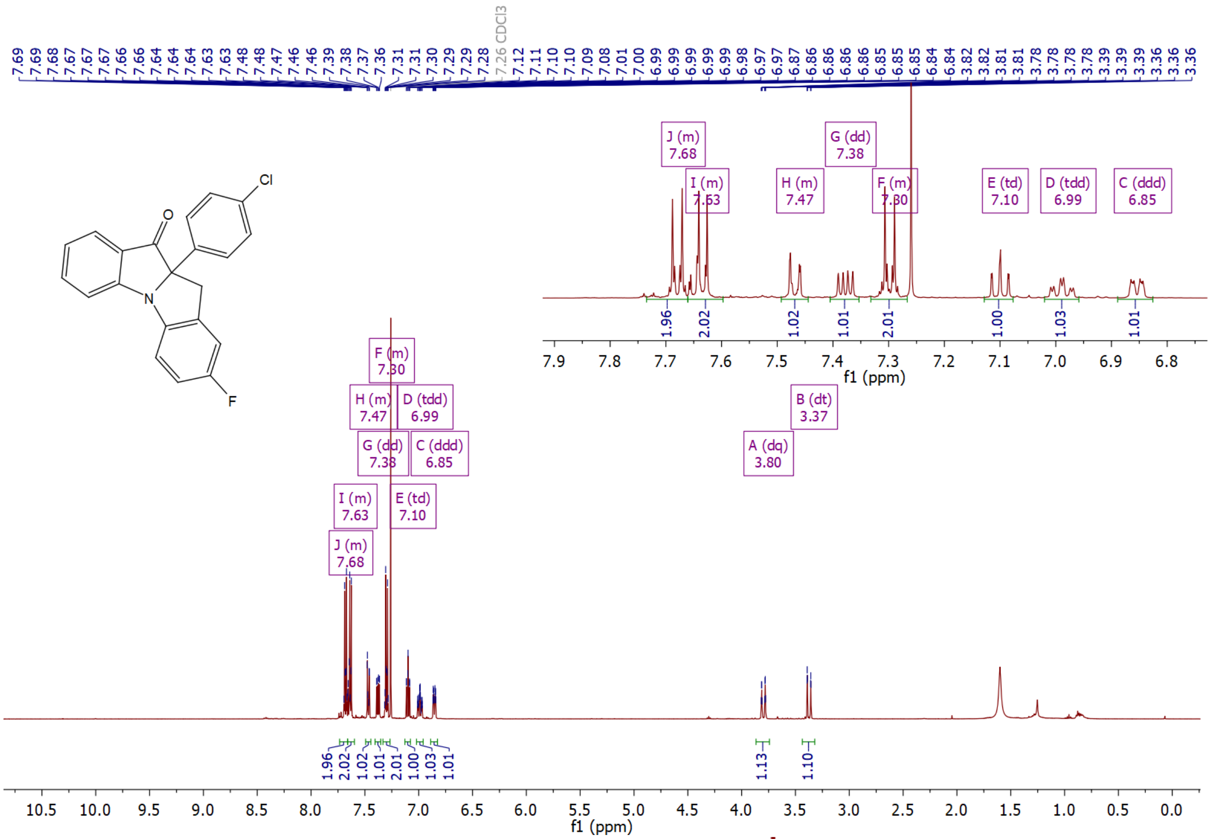

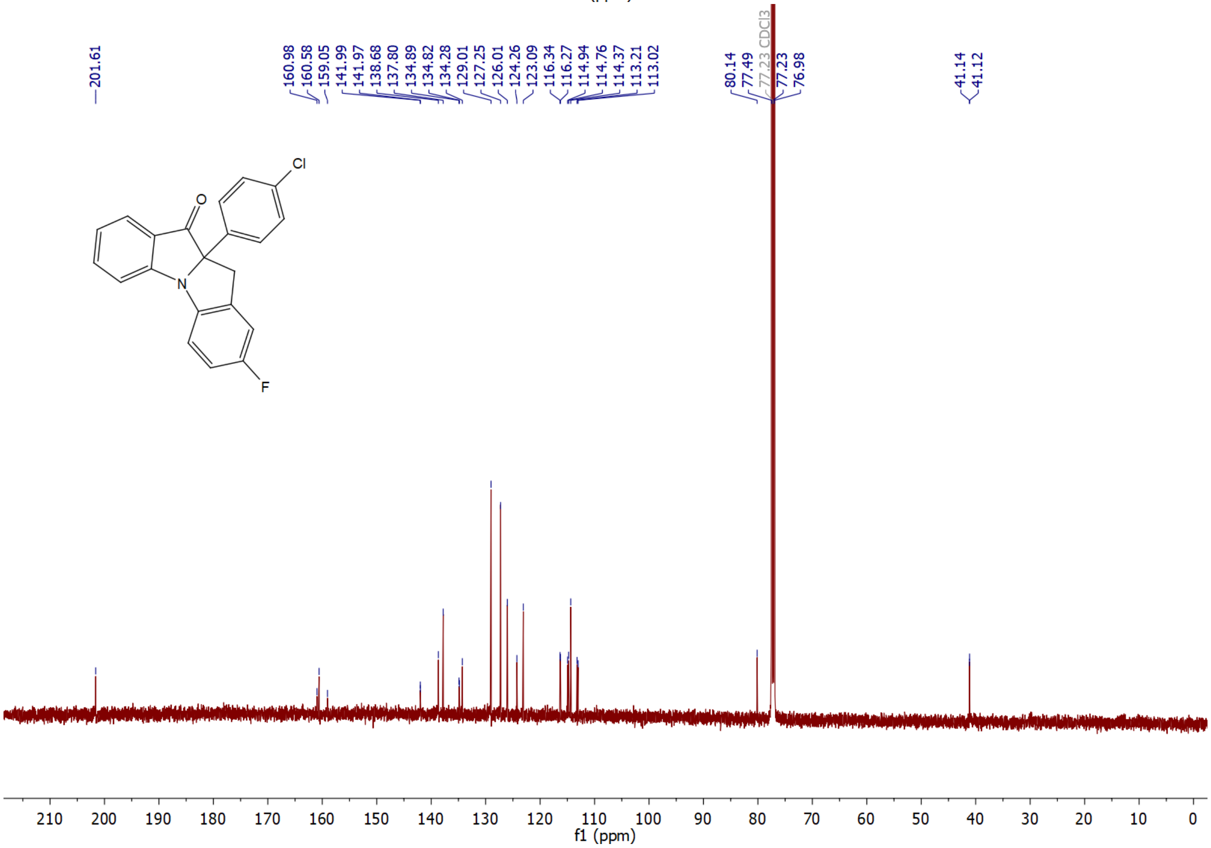

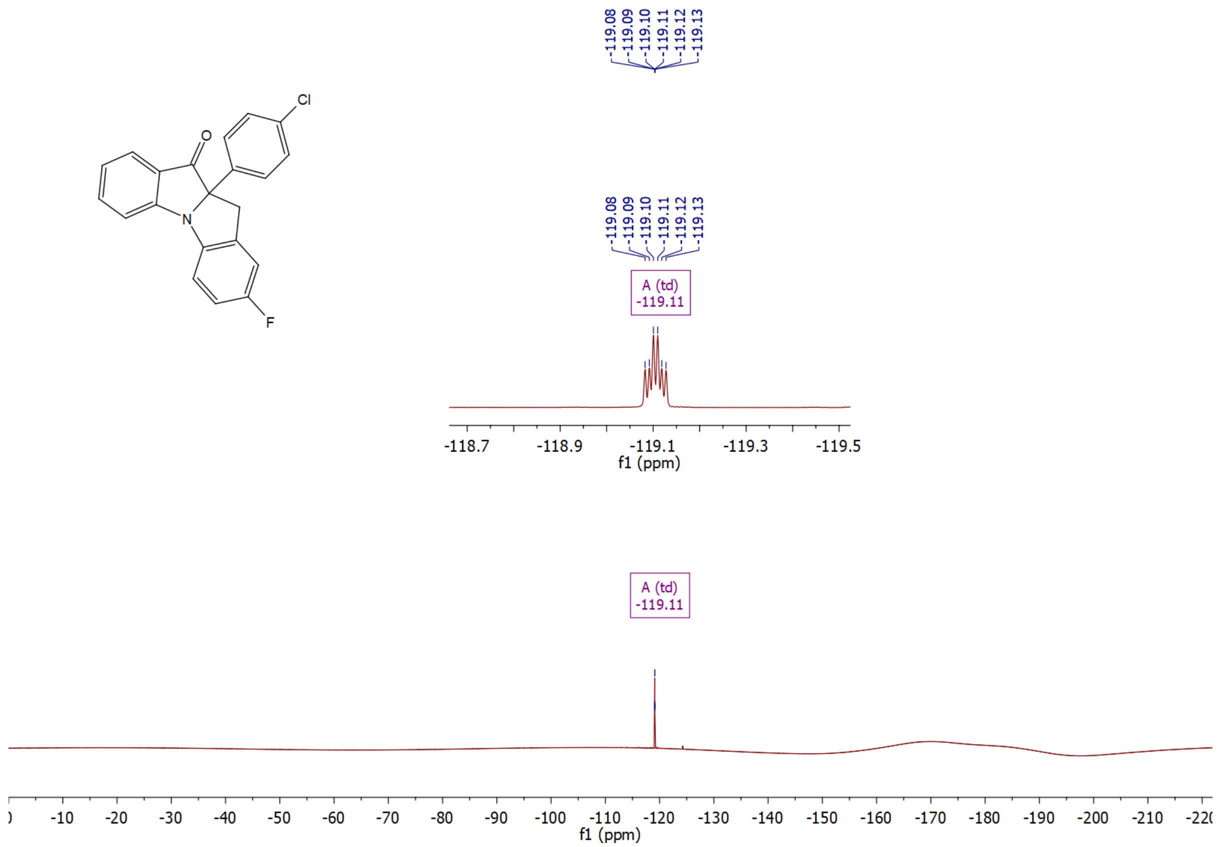
Cl
O
N
F
-119.08
-119.09
-119.10
-119.11
-119.12
-119.13
A (td)
-119.11
-118.7
-118.9
-119.1
-119.3
-119.5
f1 (ppm)
-10
-20
-30
-40
-50
-60
-70
-80
-90
-100
-110
-120
-130
-140
-150
-160
-170
-180
-190
-200
-210
f1 (ppm)

10a-(4-chlorophenyl)-1-fluoro-10a,11-dihydro-10H-indolo[1,2-a]indol-10-one [F25]:

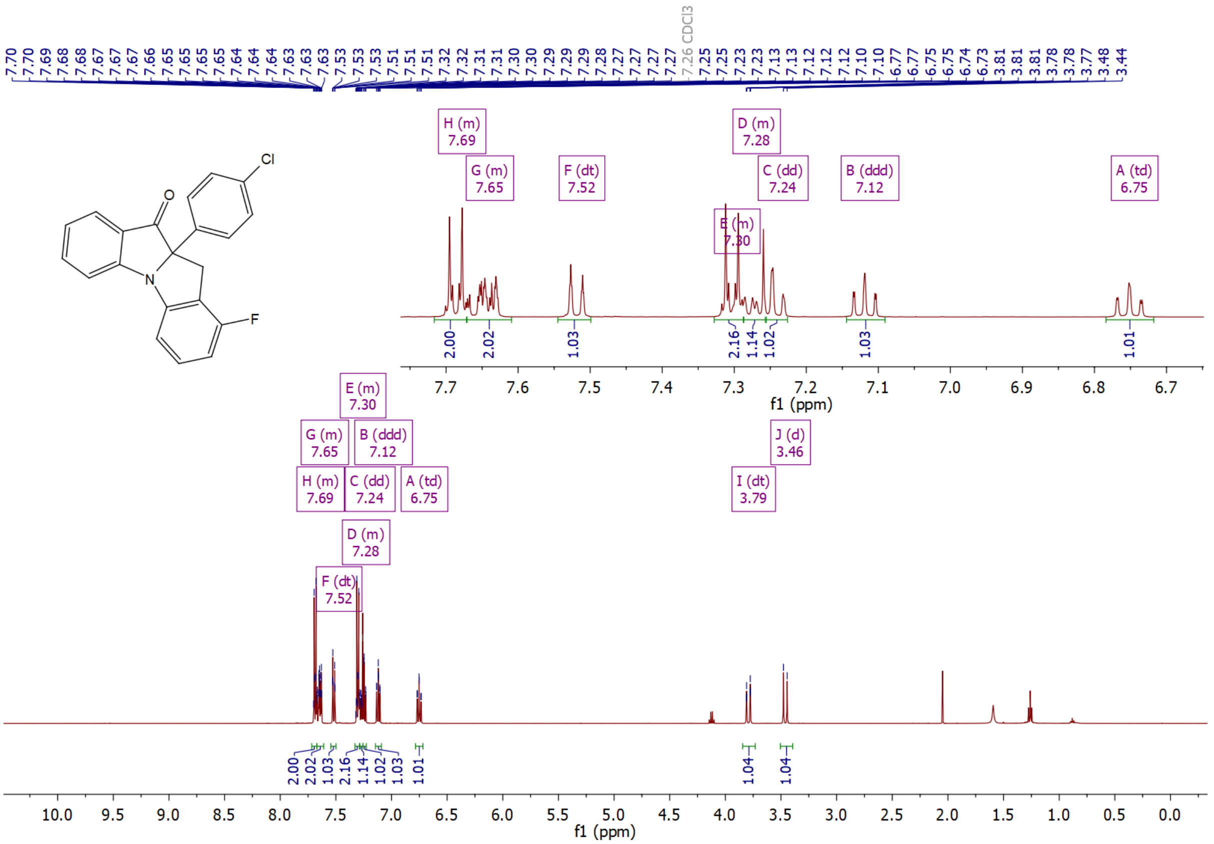

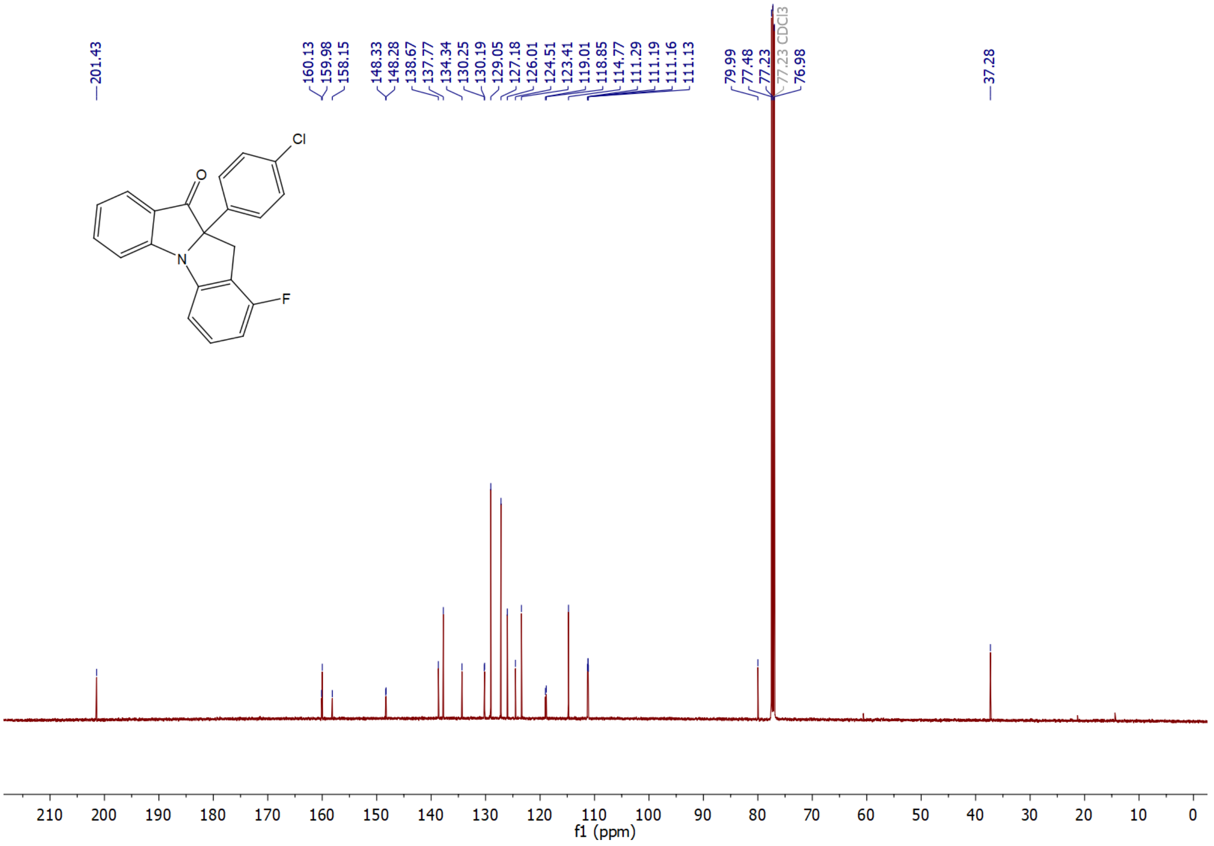

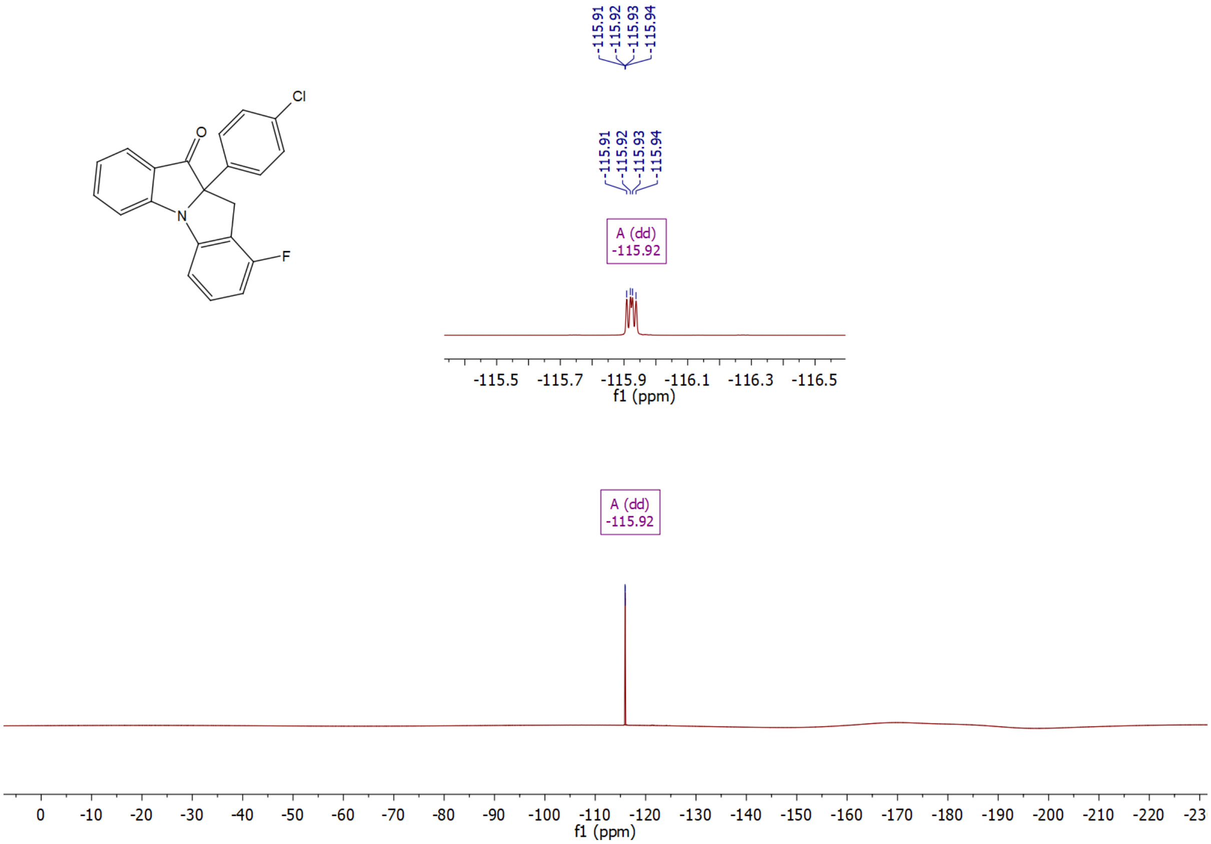
-115.91
-115.92
-115.93
-115.94
A (dd)
-115.92
-115.5 -115.7 -115.9 -116.1 -116.3 -116.5
f1 (ppm)
0 -10 -20 -30 -40 -50 -60 -70 -80 -90 -100 -110 -120 -130 -140 -150 -160 -170 -180 -190 -200 -210 -220 -23
f1 (ppm)
Cl
O
N
F

***2-methoxy-10a-methyl-10a,11-dihydro-10H-indolo[1,2-a]indol-10-ol* [F26]:**

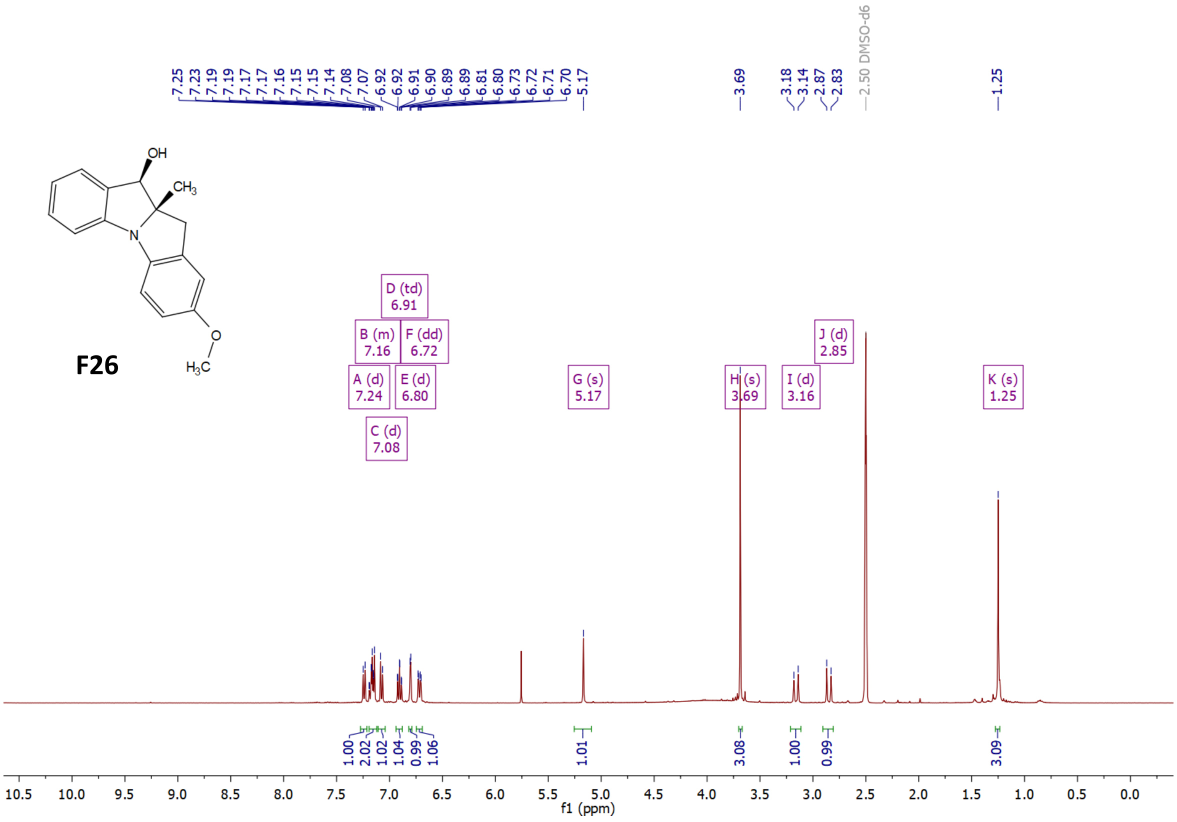

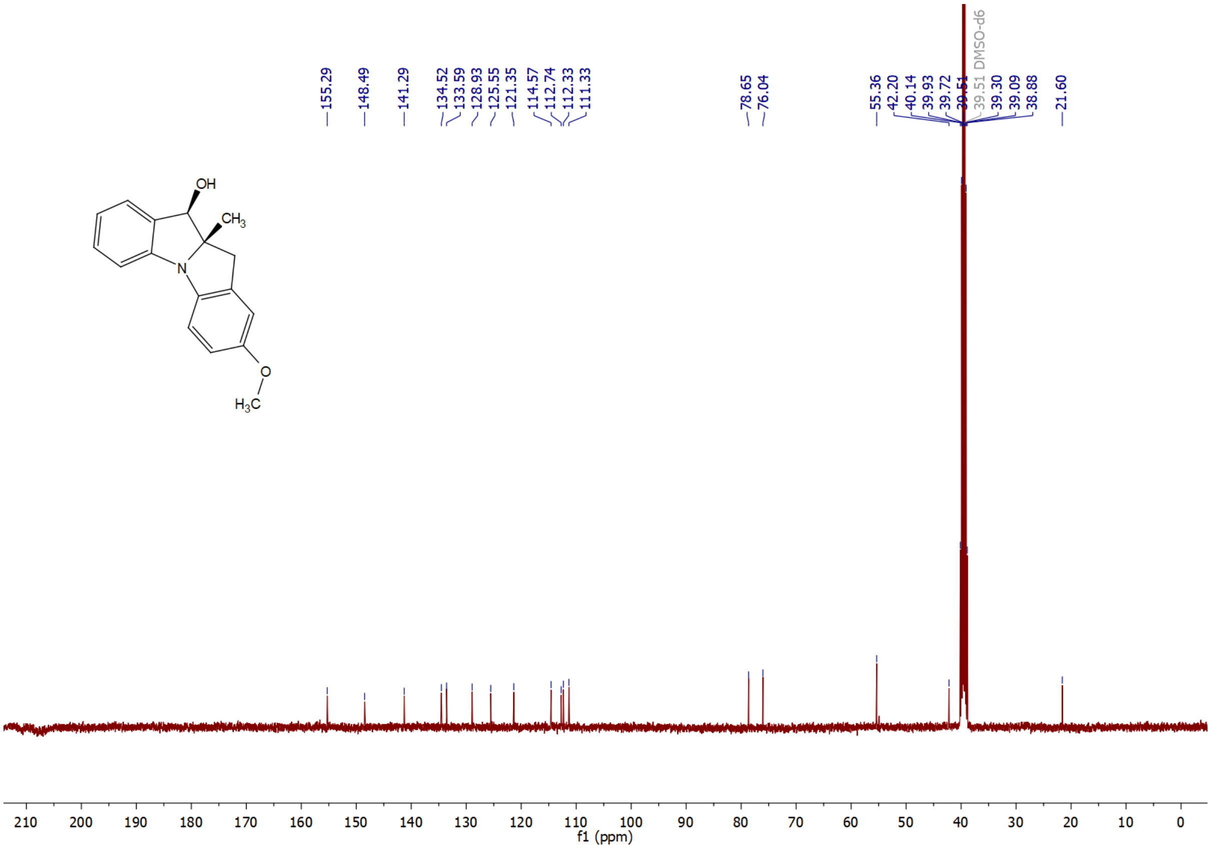

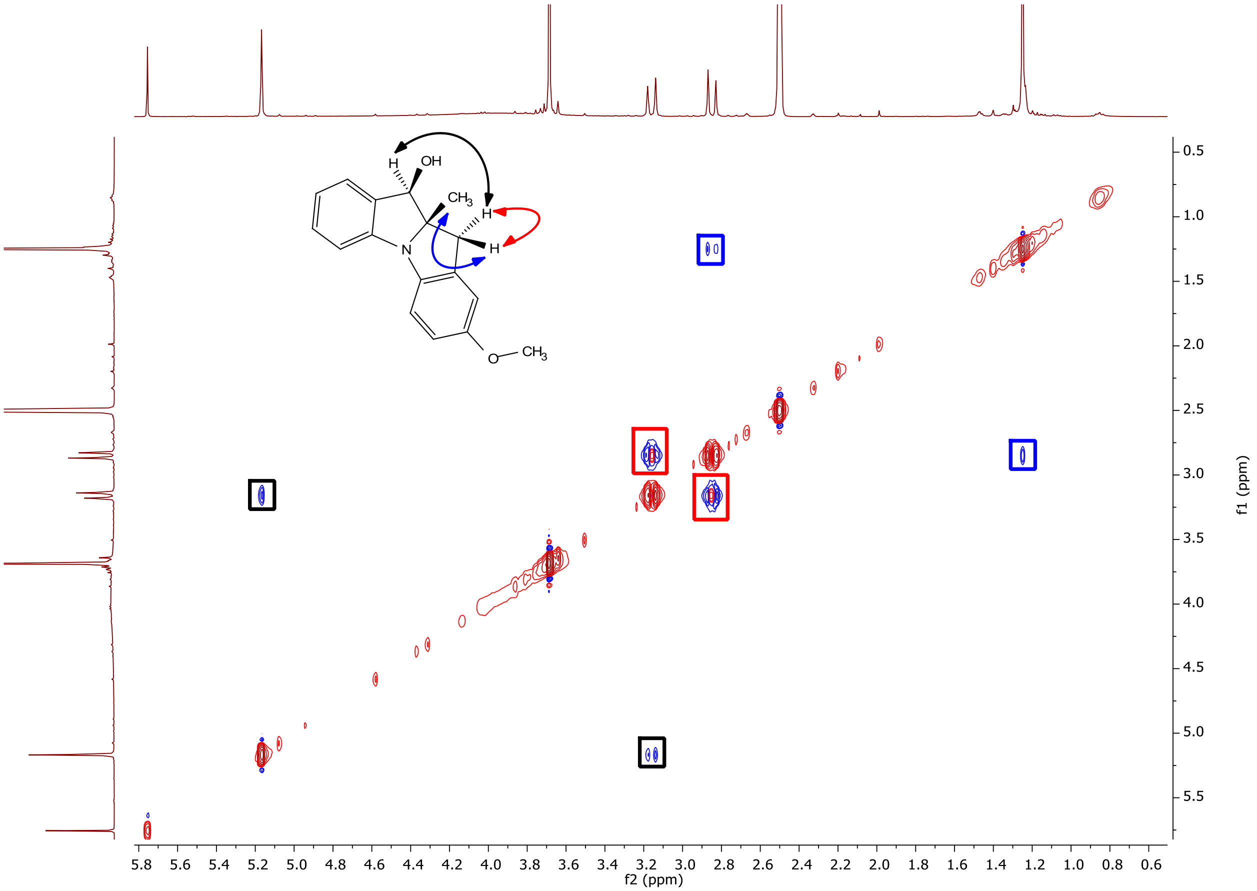

H
OH
CH3
H
H
N
O
CH3
f2 (ppm)
f1 (ppm)

2-hydroxy-10a-methyl-10a,11-dihydro-10H-indolo[1,2-a]indol-10-one **[F27]:**

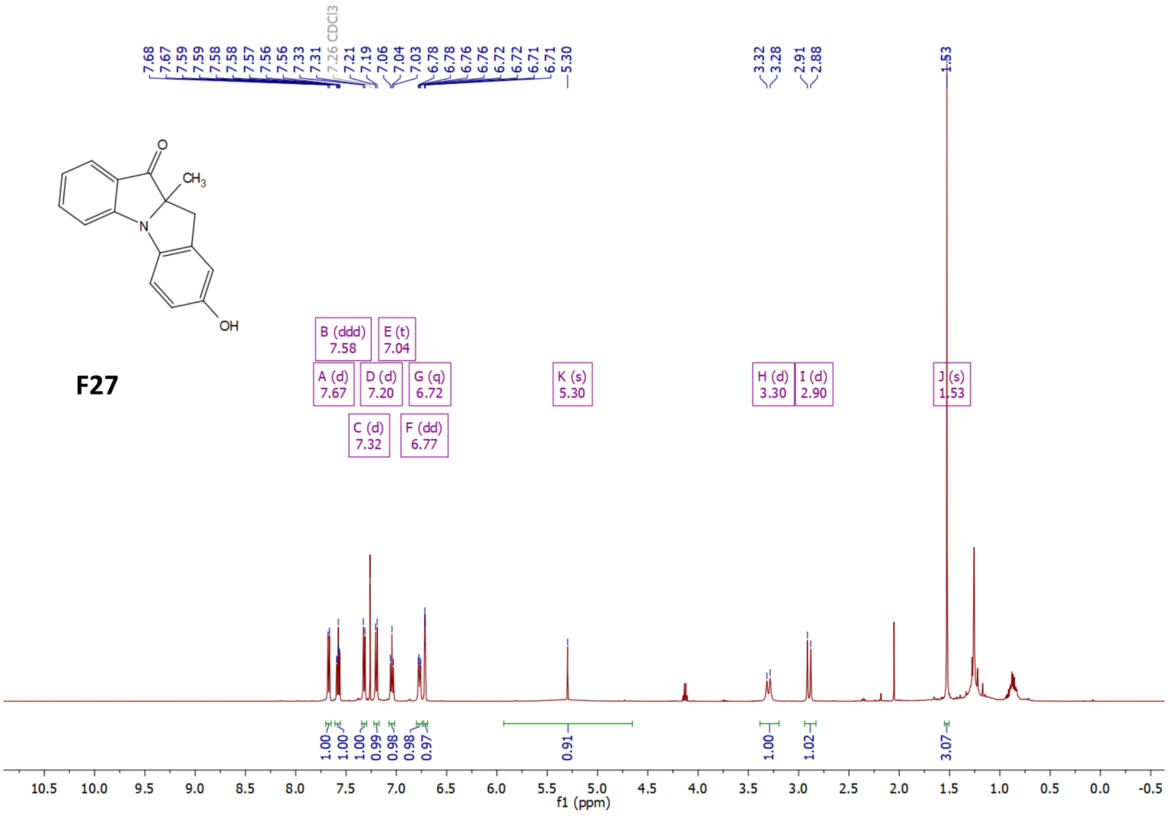

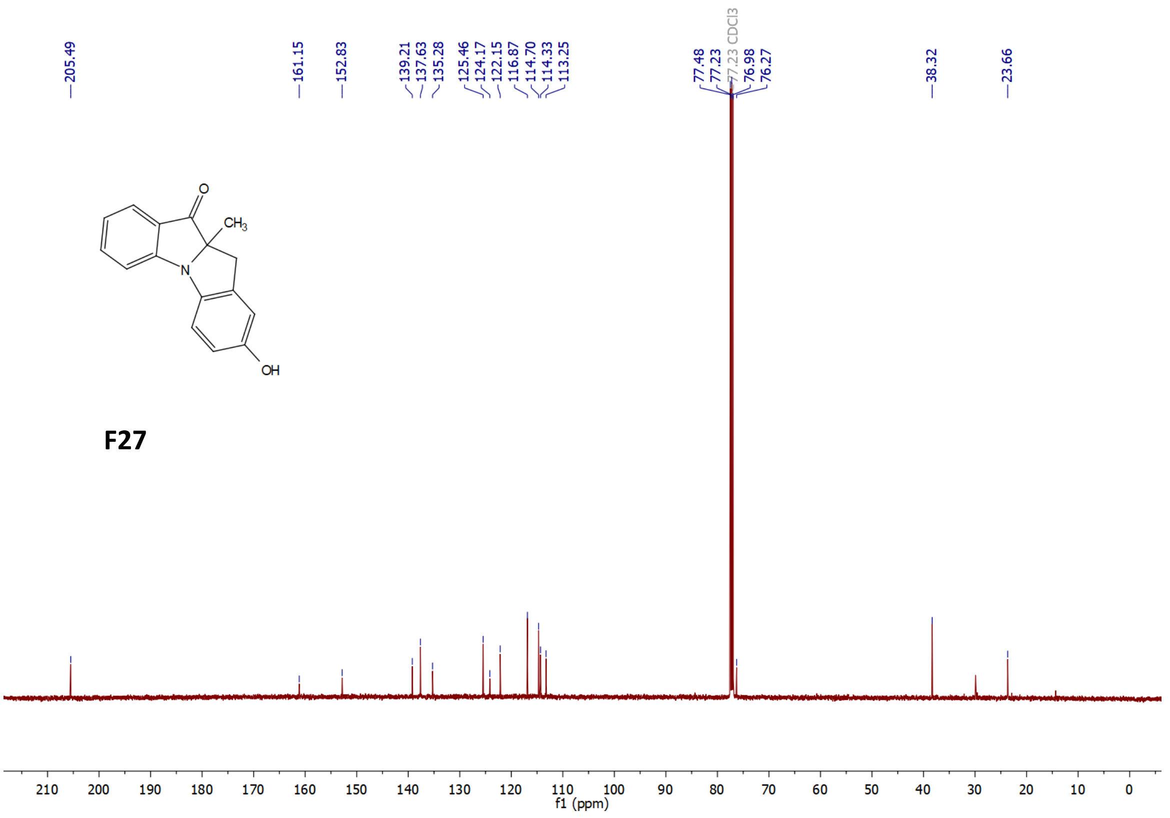

2-(benzyloxy)-10a-methyl-10a,11-dihydro-10H-indolo[1,2-a]indol-10-one **[F28]:**

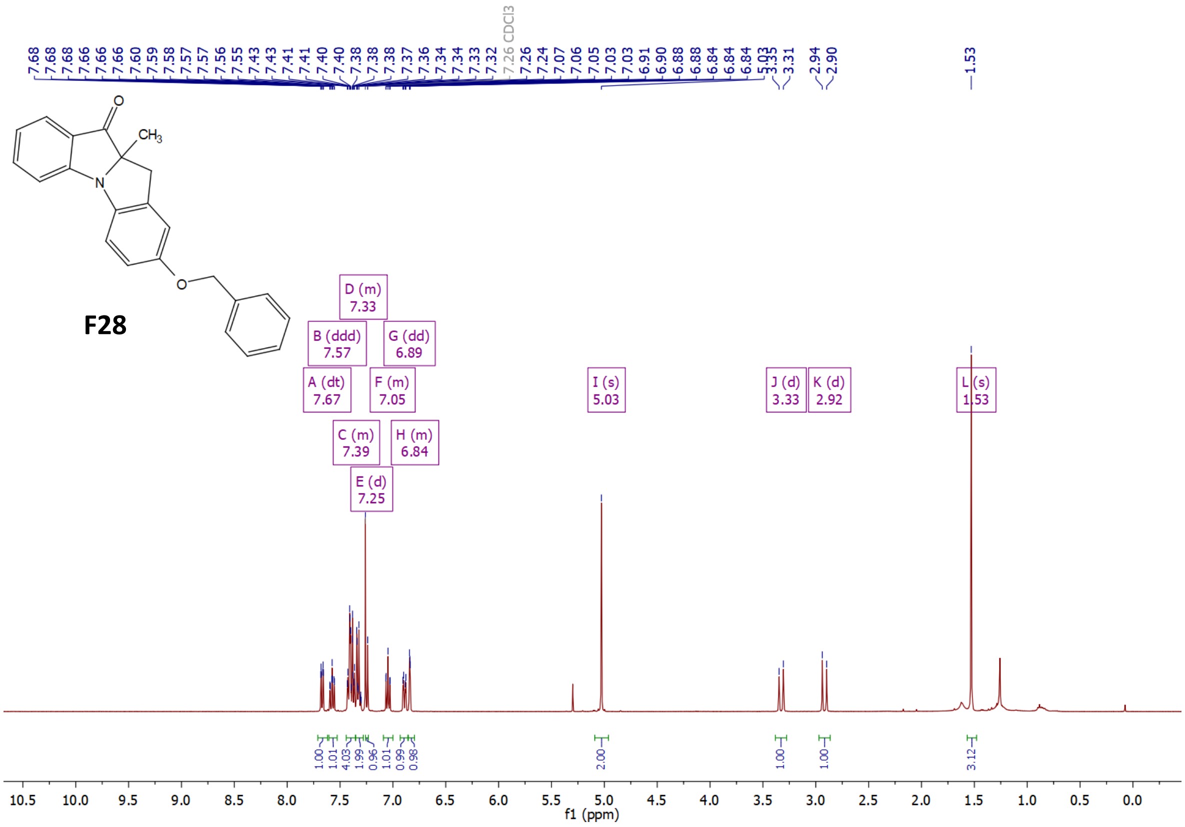

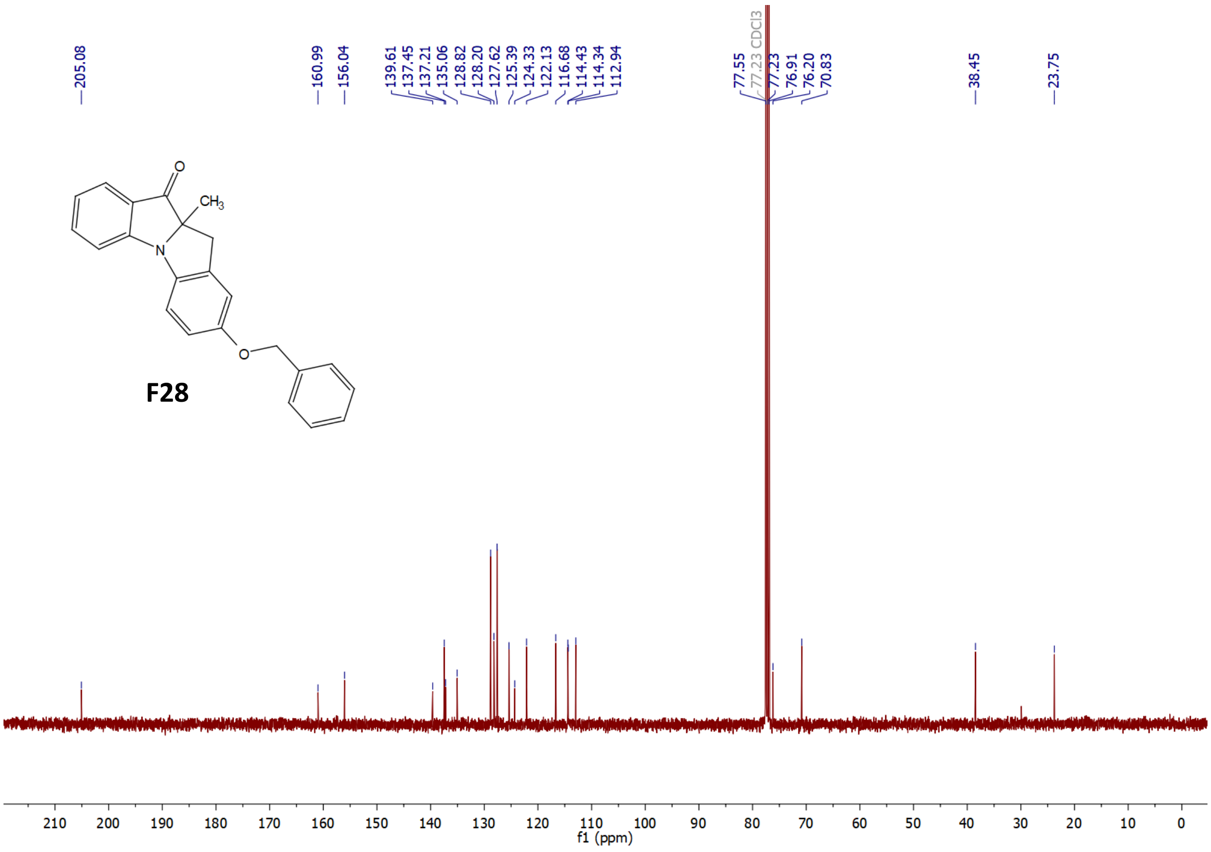

8-bromo-2-methoxy-10a-methyl-10a,11-dihydro-10H-indolo[1,2-a]indol-10-one **[F29]:**

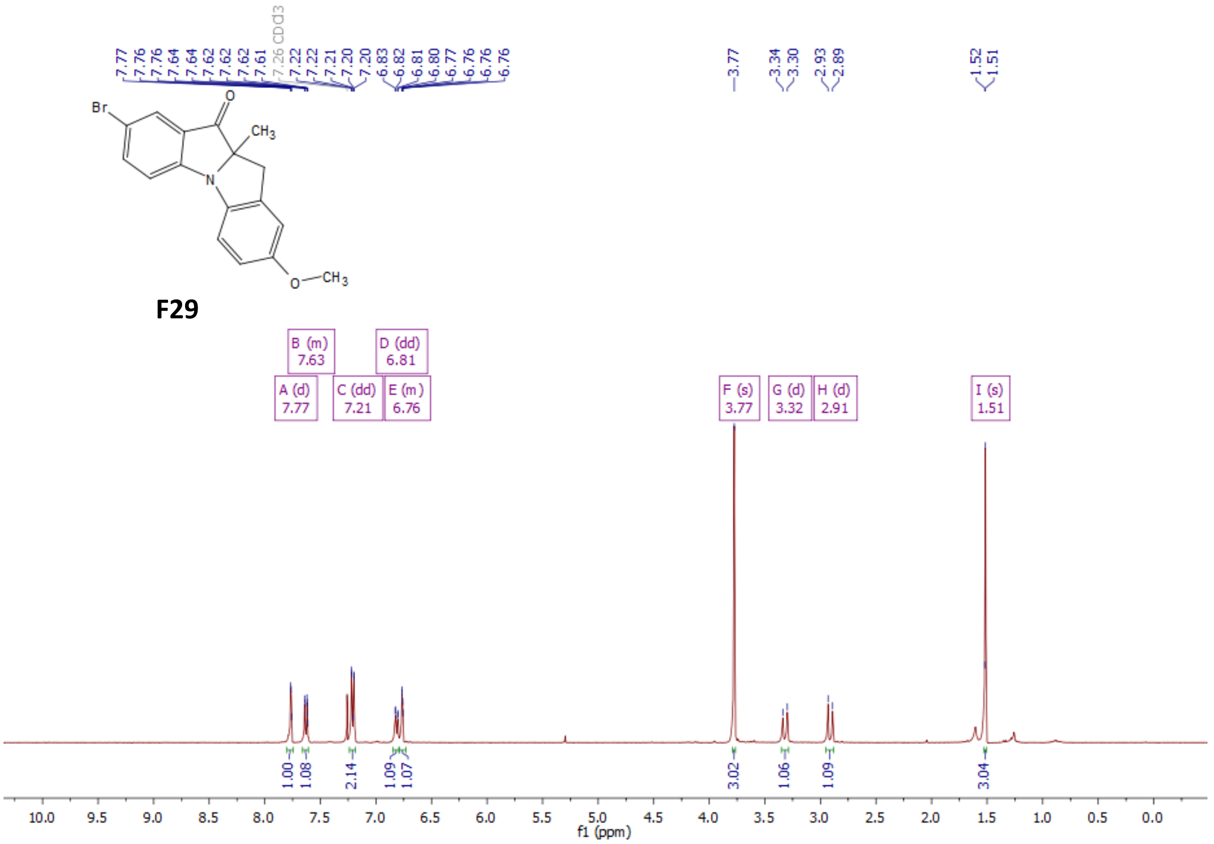

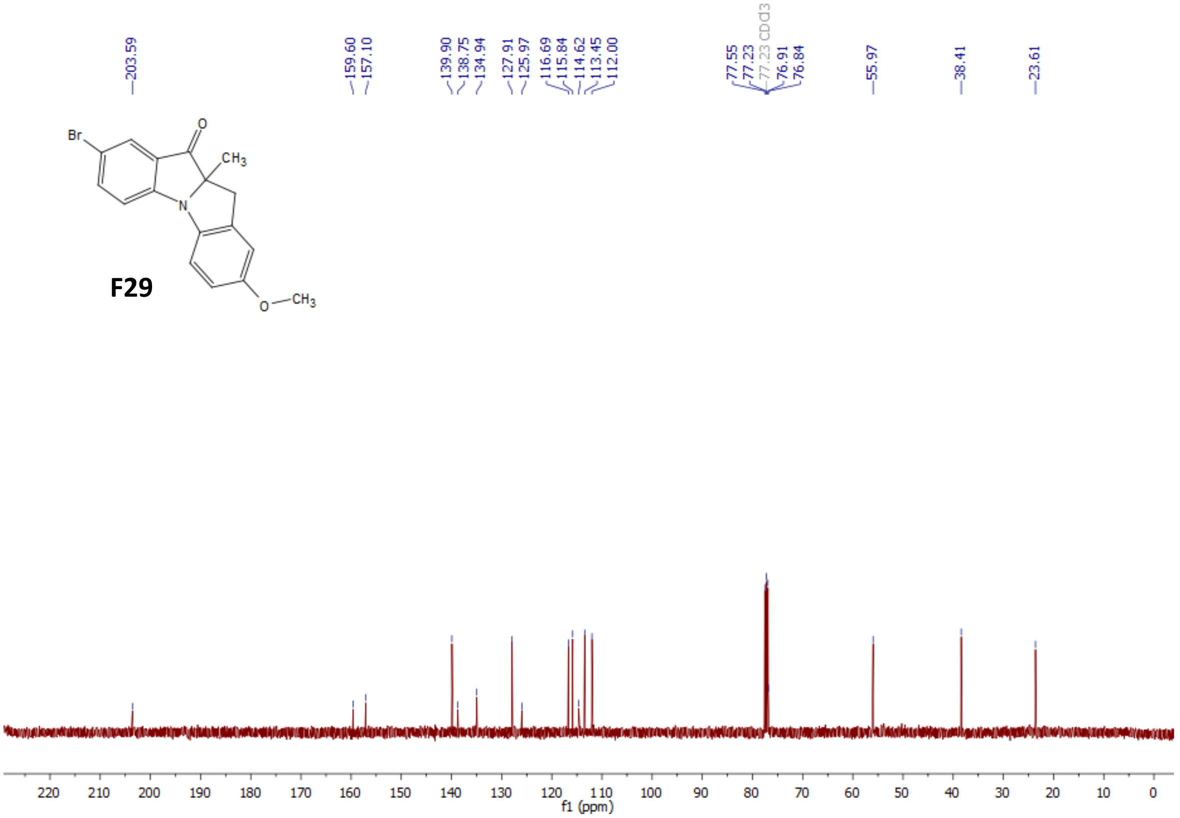

CLASS-G

11a-methyl-11,11a-dihydroindolo[1,2-b]isoquinoline-6,12-dione [G1]:

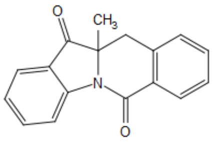

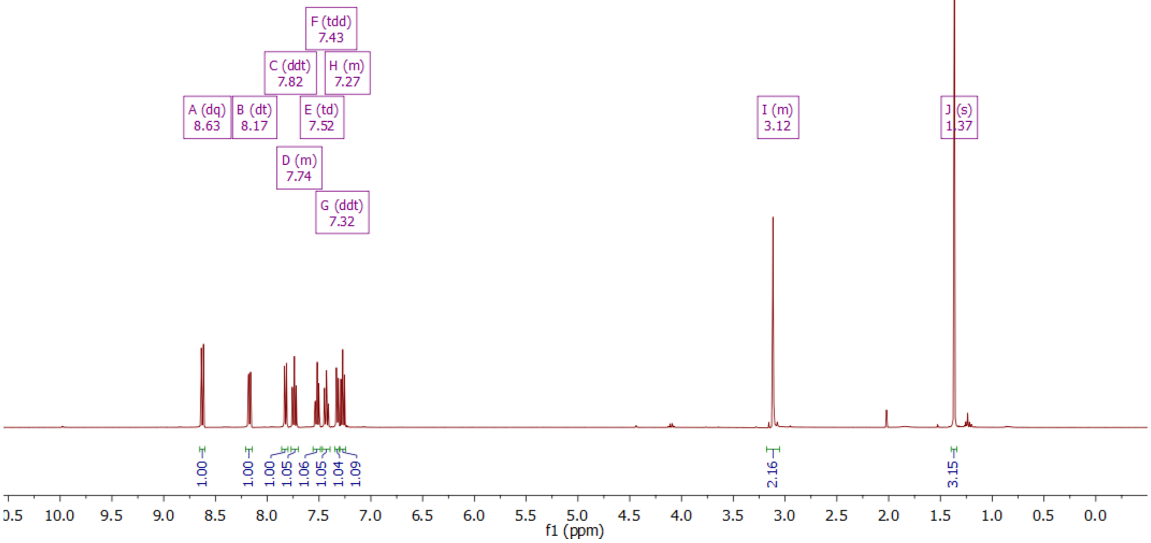

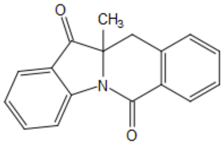

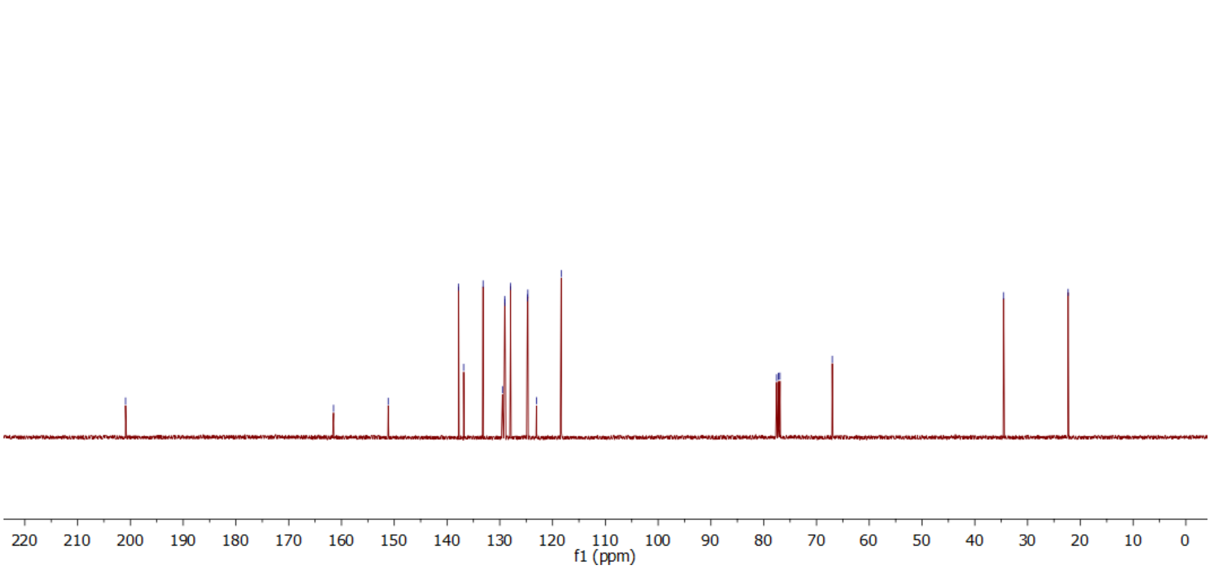

7-fluoro-11a-methyl-11,11a-dihydroindolo[1,2-b]isoquinoline-6,12-dione [G2]:

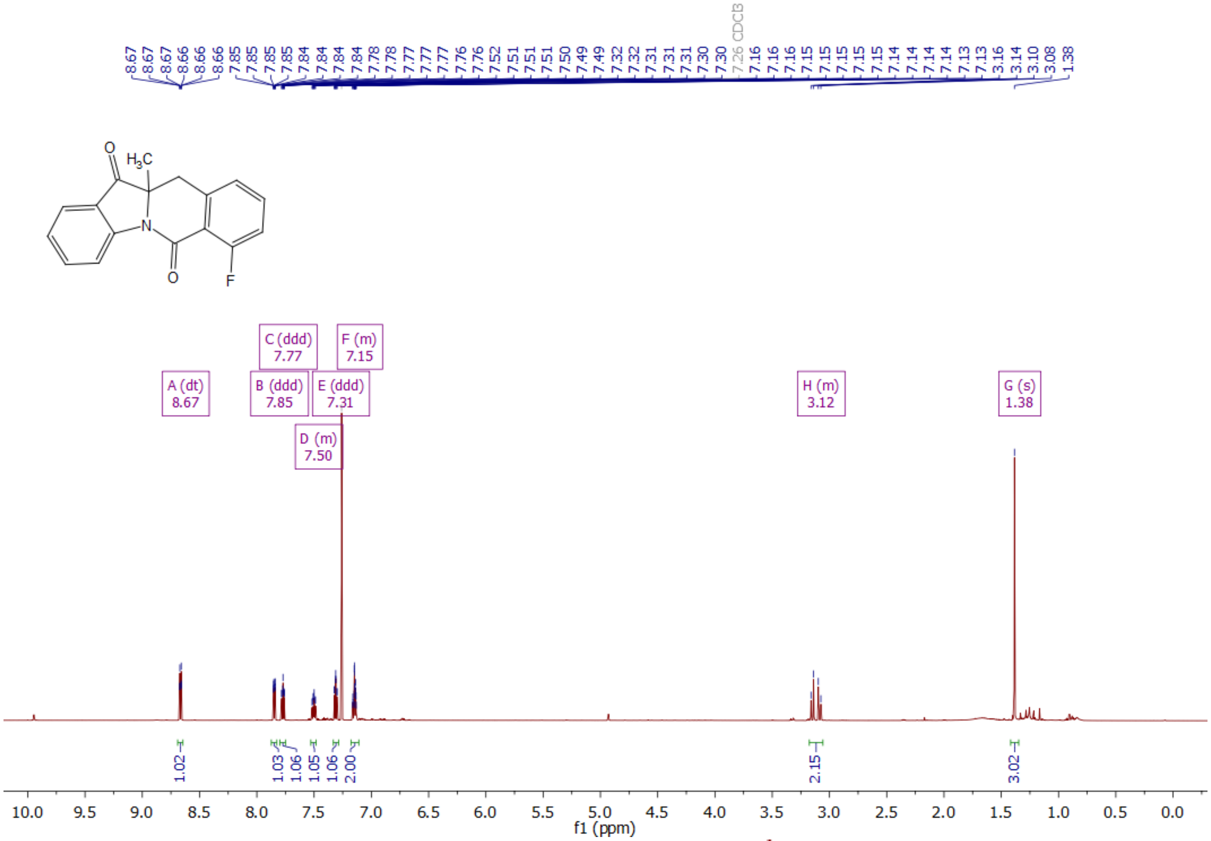

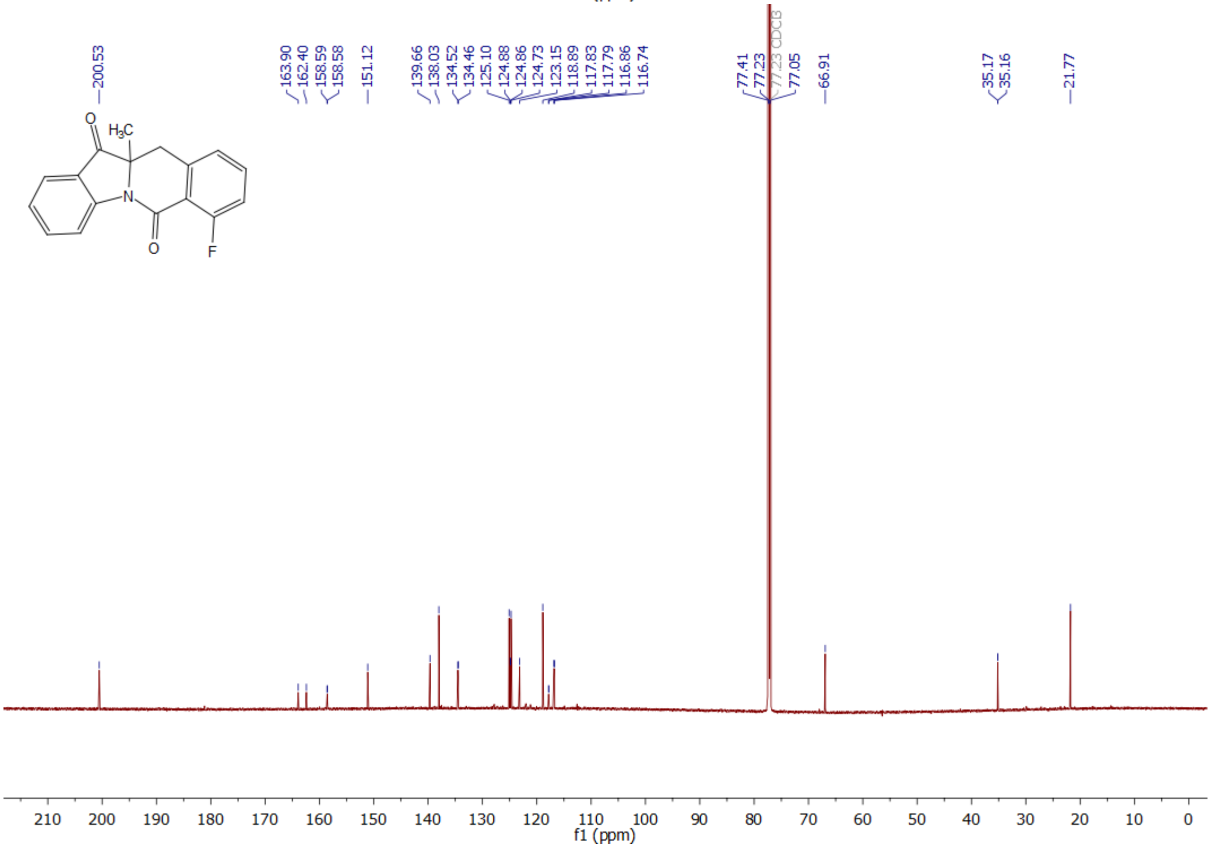

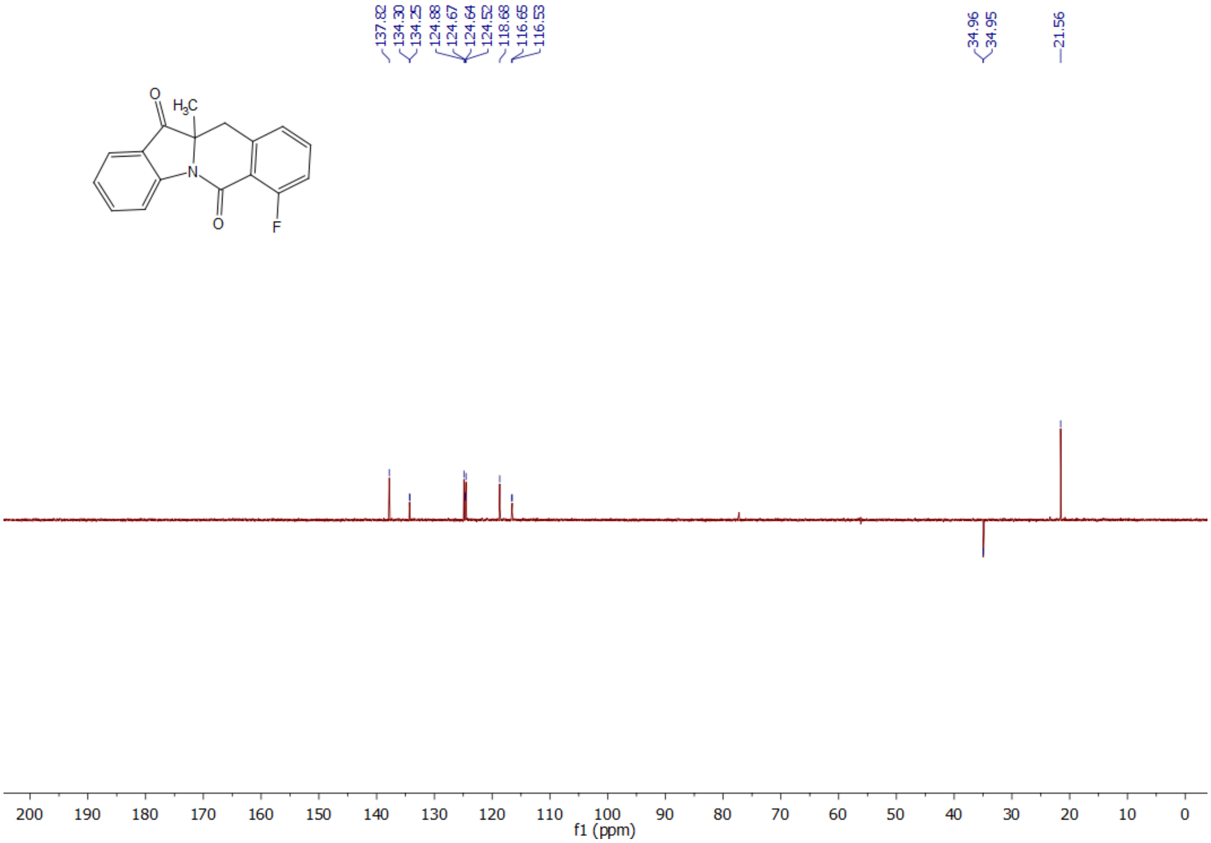

O
H3C
N
O
F
137.82
134.30
134.25
124.88
124.67
124.64
124.52
118.68
116.65
116.53
34.96
34.95
21.56
200 190 180 170 160 150 140 130 120 110 100 90 80 70 60 50 40 30 20 10 0
f1 (ppm)

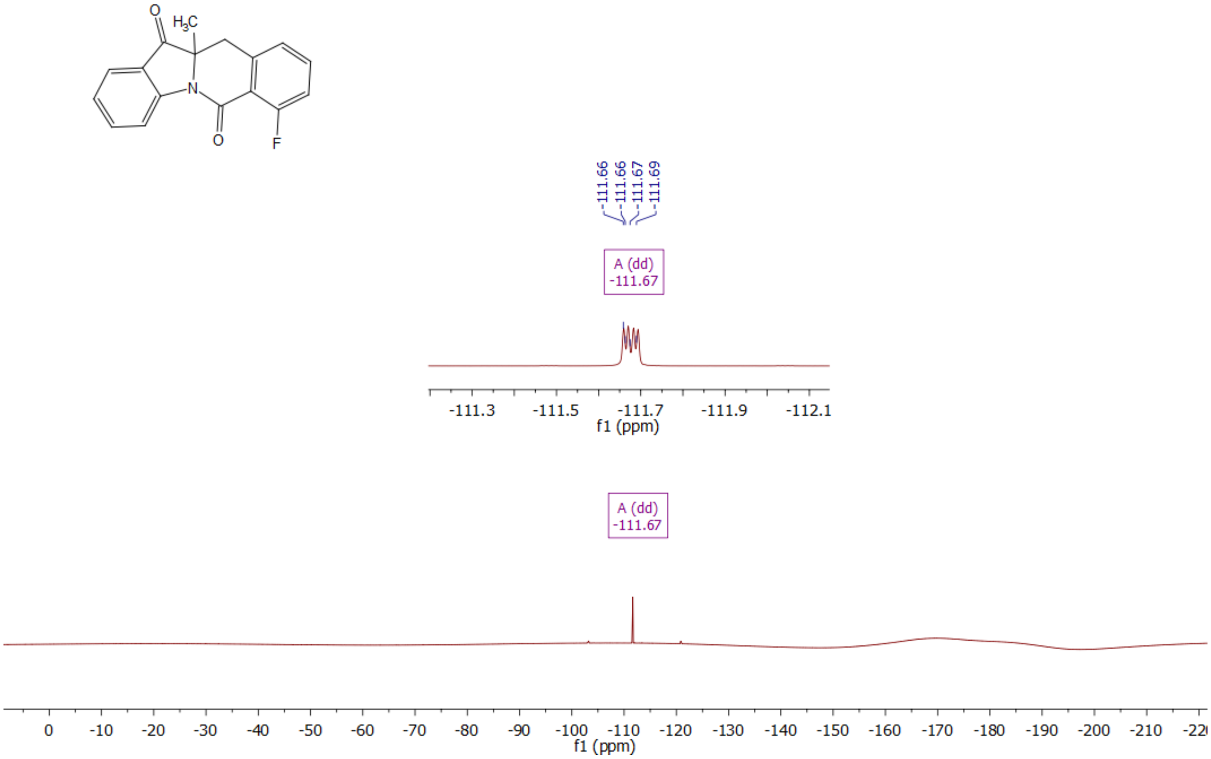

O
H3C
N
O
F
-111.66
-111.66
-111.67
-111.69
A (dd)
-111.67
-111.3 -111.5 -111.7 -111.9 -112.1
f1 (ppm)
A (dd)
-111.67
0 -10 -20 -30 -40 -50 -60 -70 -80 -90 -100 -110 -120 -130 -140 -150 -160 -170 -180 -190 -200 -210
f1 (ppm)

8,11a-dimethyl-11,11a-dihydroindolo[1,2-b]isoquinoline-6,12-dione [G3]:

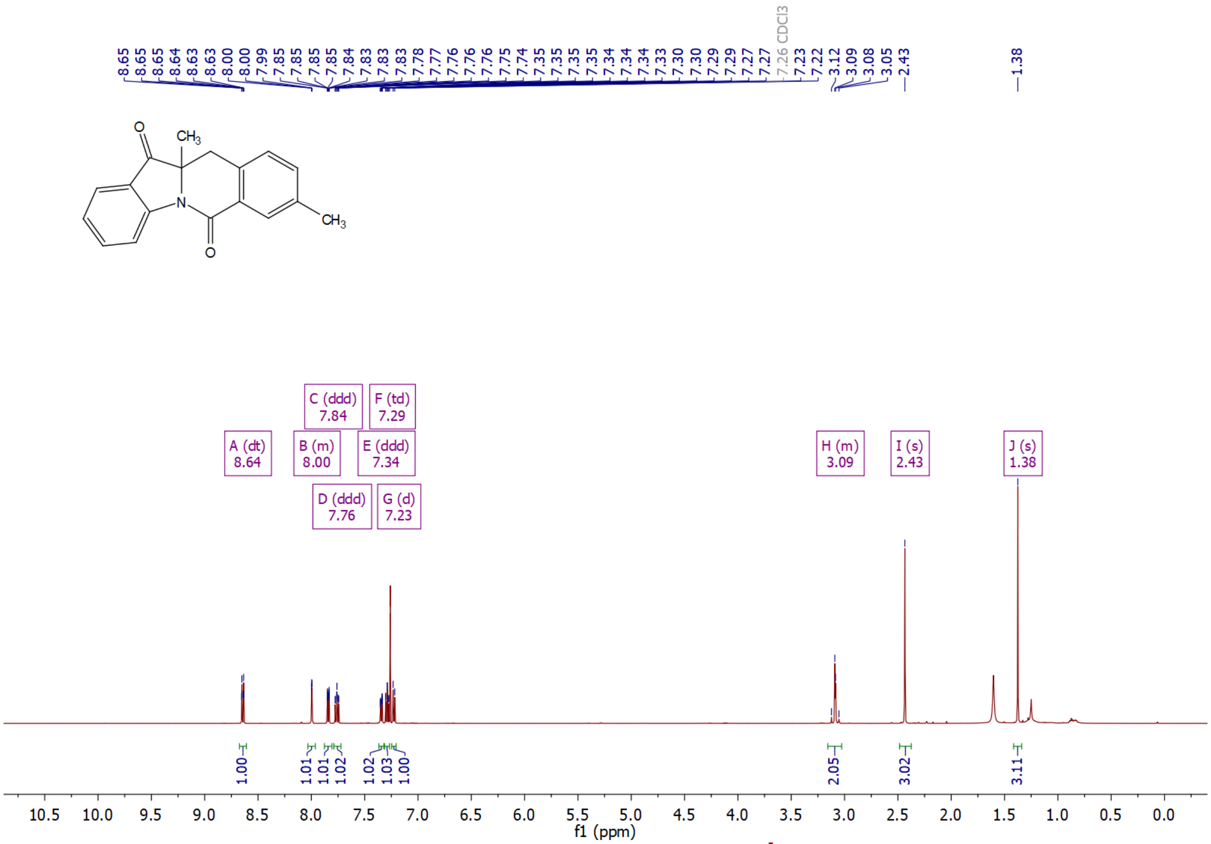

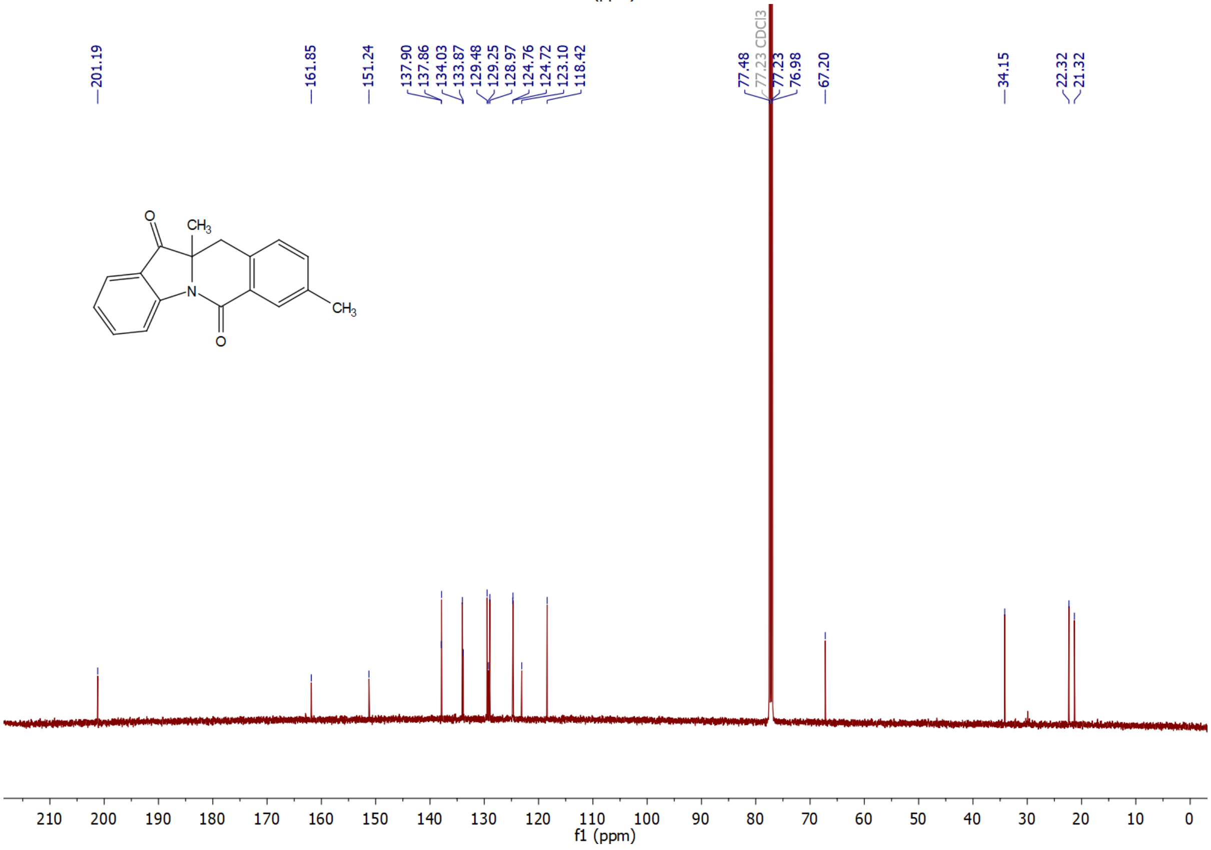

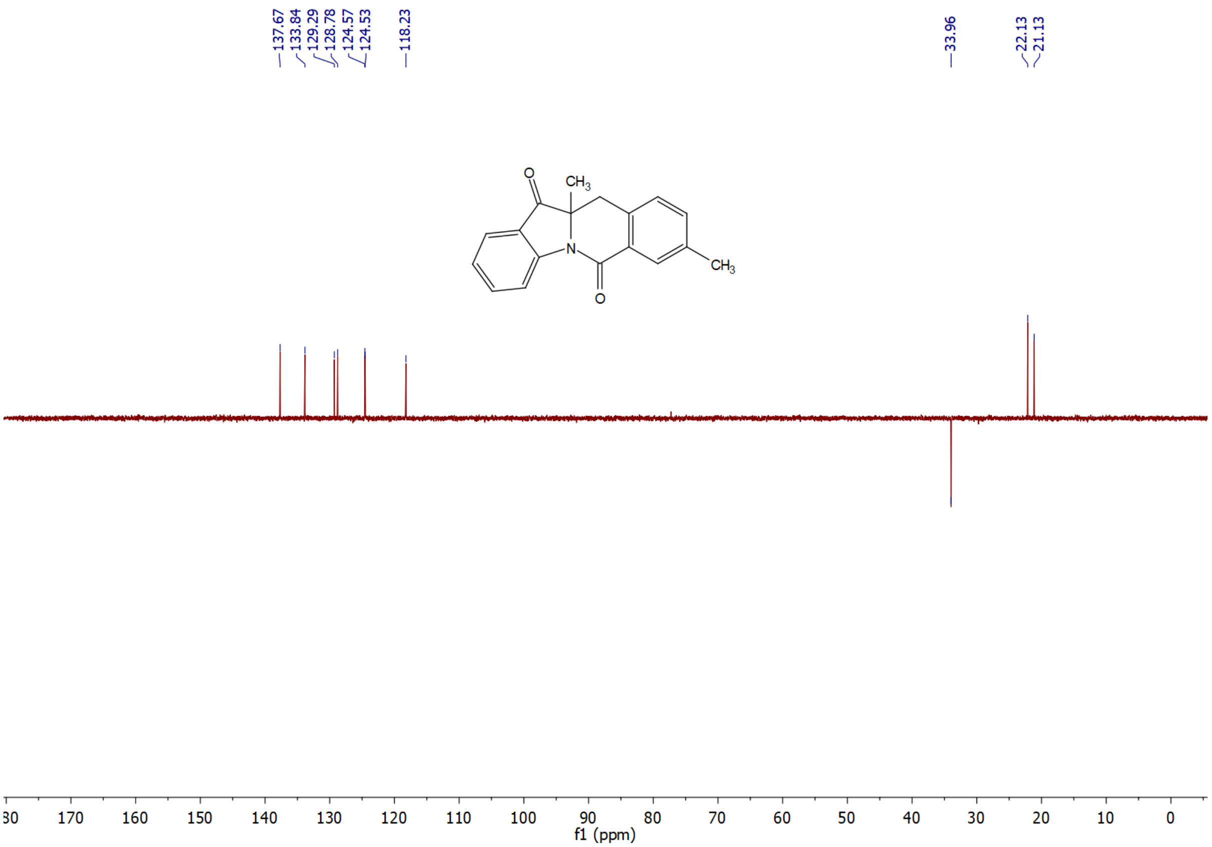
137.67
133.84
129.29
128.78
124.57
124.53
118.23
33.96
22.13
21.13
O
CH3
N
O
CH3
30
170
160
150
140
130
120
110
100
90
80
70
60
50
40
30
20
10
0
f1 (ppm)

9-methoxy-11a-methyl-11,11a-dihydroindolo[1,2-b]isoquinoline-6,12-dione **[G4]**:

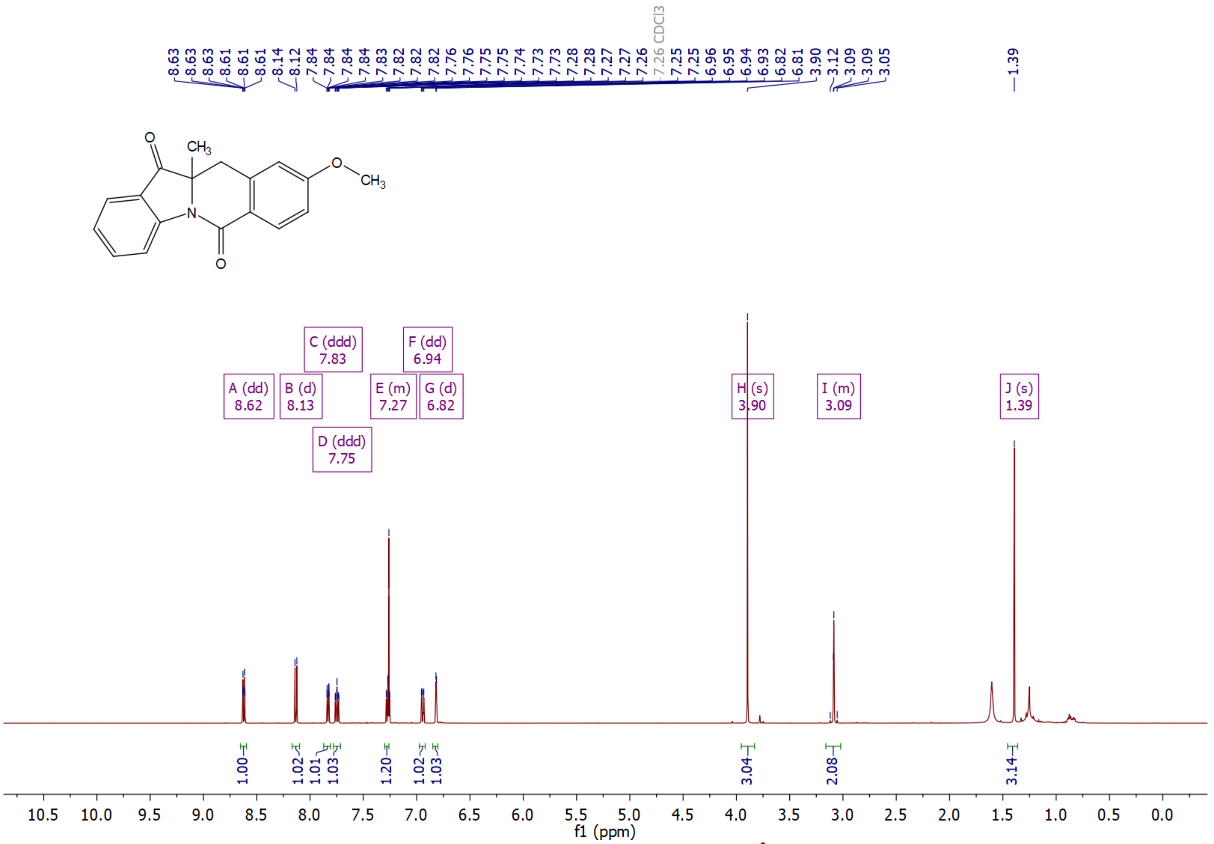

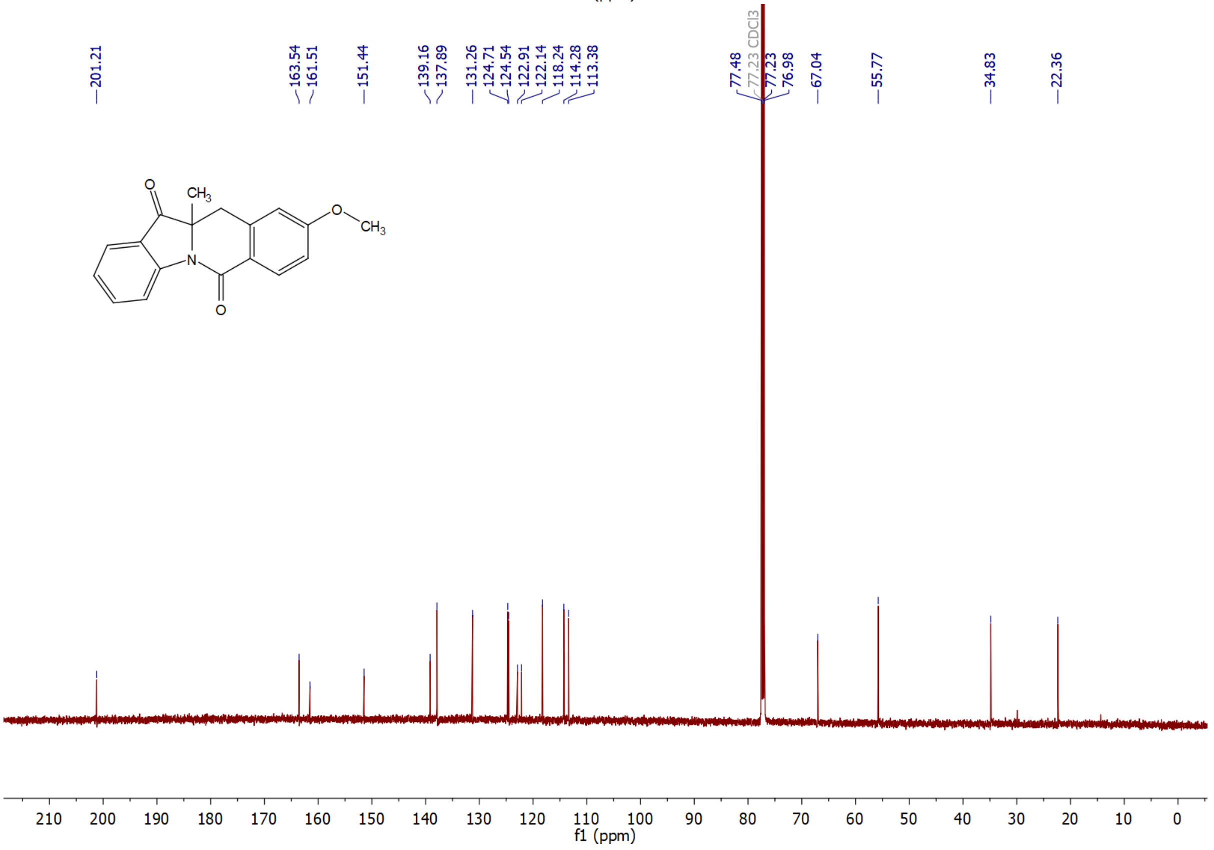

10-fluoro-11a-methyl-11,11a-dihydroindolo[1,2-b]isoquinoline-6,12-dione [G5]:

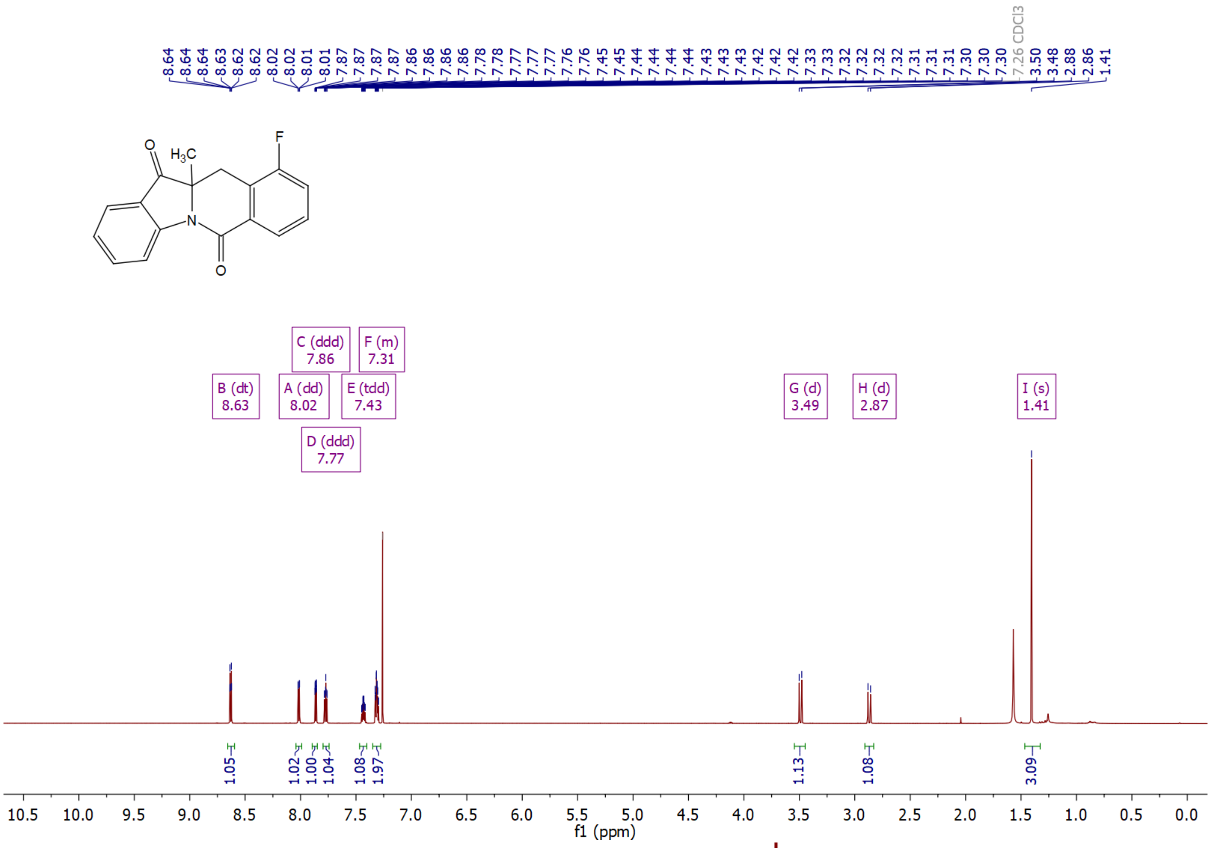

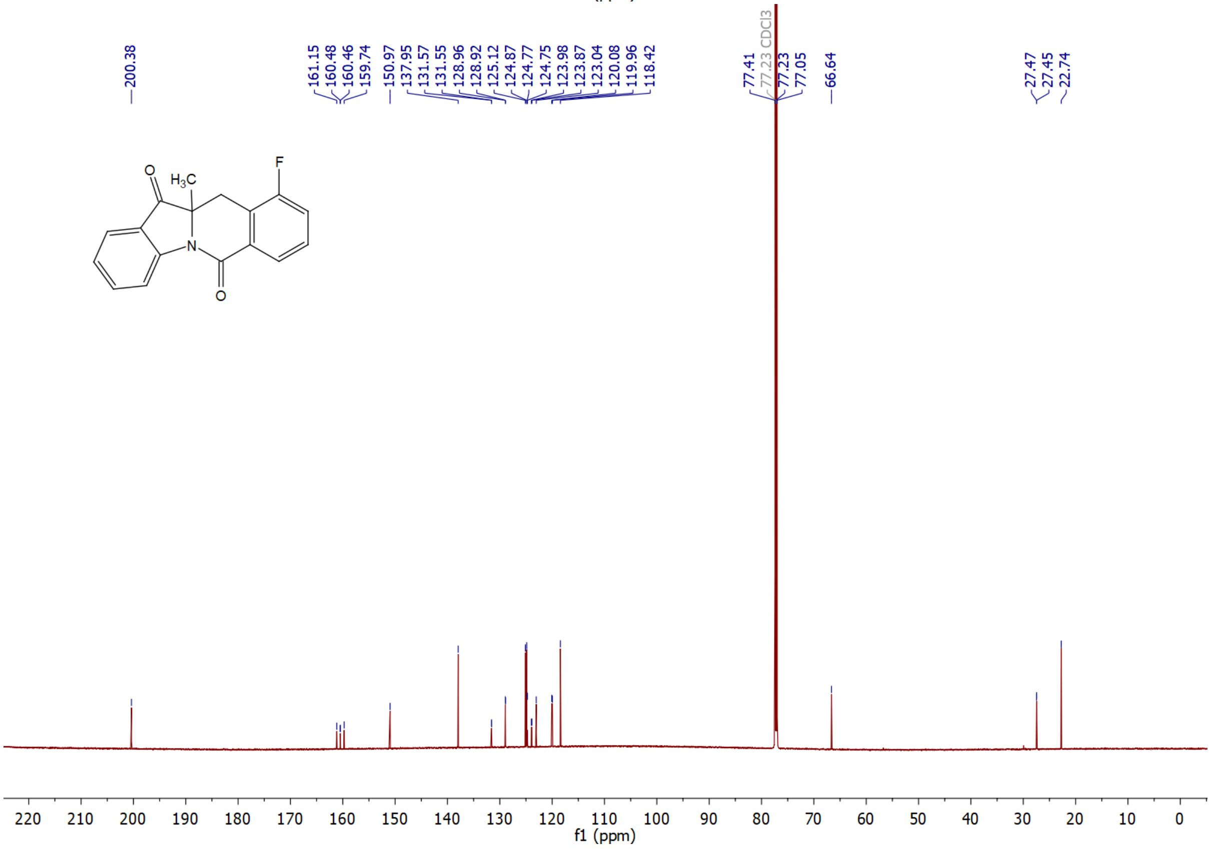

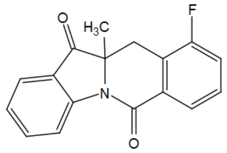
O
H3C
F
N
O

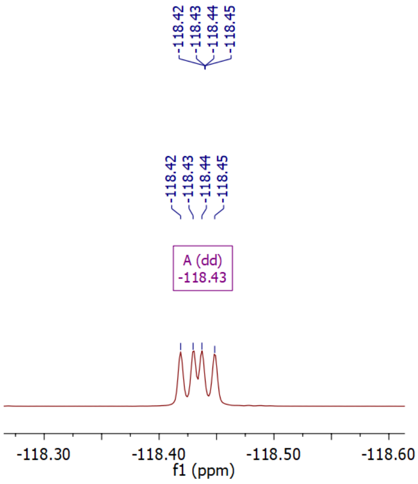
-118.42
-118.43
-118.44
-118.45
-118.42
-118.43
-118.44
-118.45
A (dd)
-118.43
-118.30
-118.40
-118.50
-118.60
f1 (ppm)

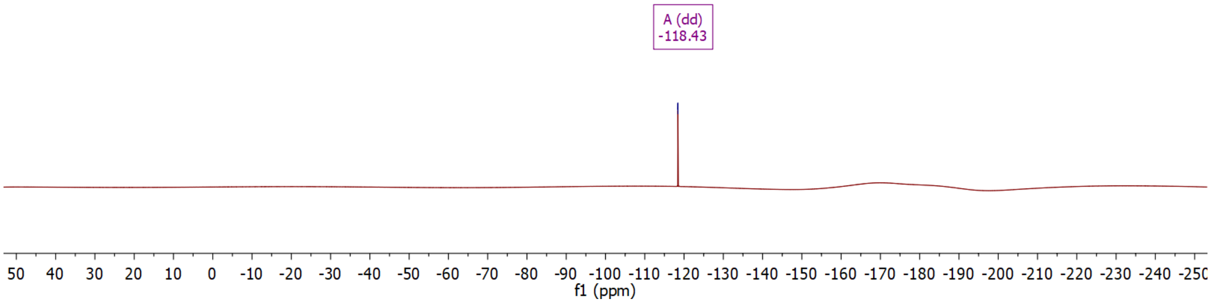
A (dd)
-118.43
50 40 30 20 10 0 -10 -20 -30 -40 -50 -60 -70 -80 -90 -100 -110 -120 -130 -140 -150 -160 -170 -180 -190 -200 -210 -220 -230 -240 -250
f1 (ppm)

11a-methyl-11a,12-dihydro-[1,3]dioxolo[4,5-g]indolo[1,2-b]isoquinoline-5,11-dione [**G6**]:

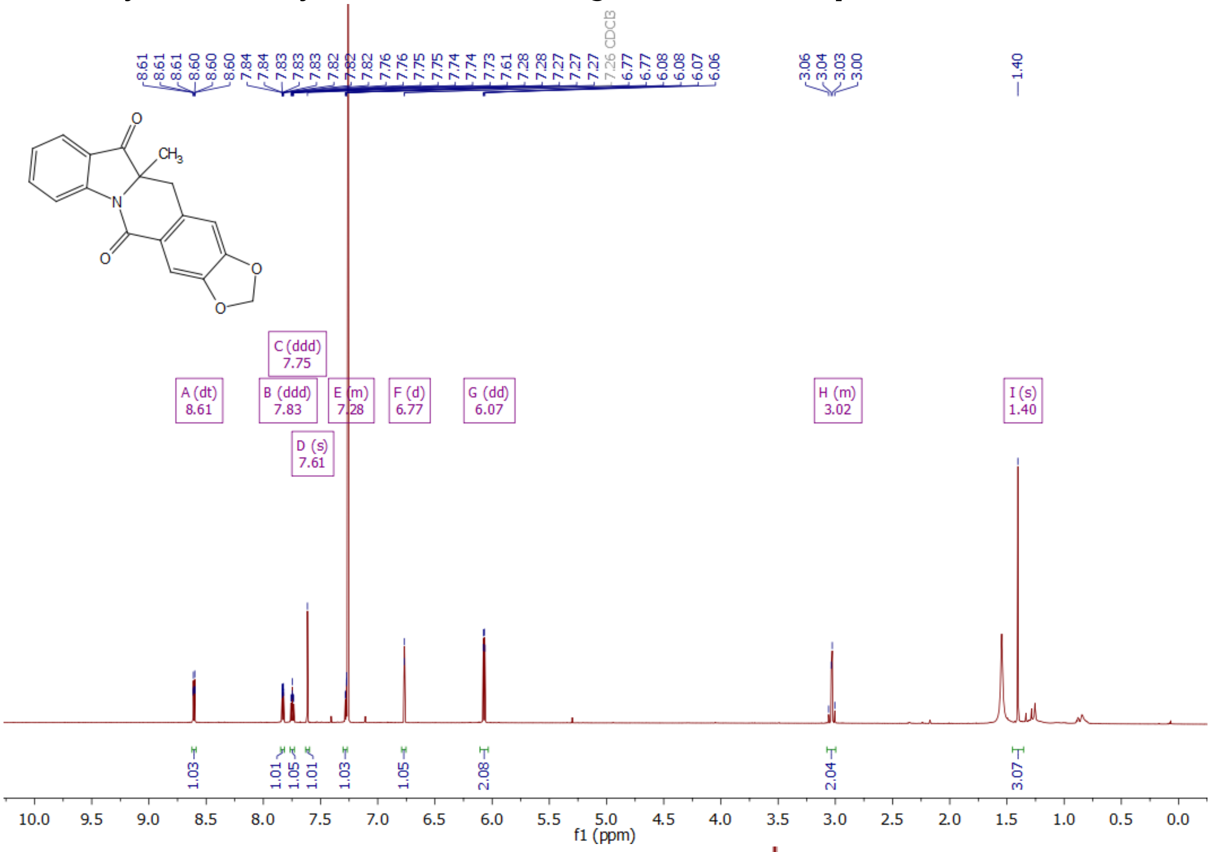

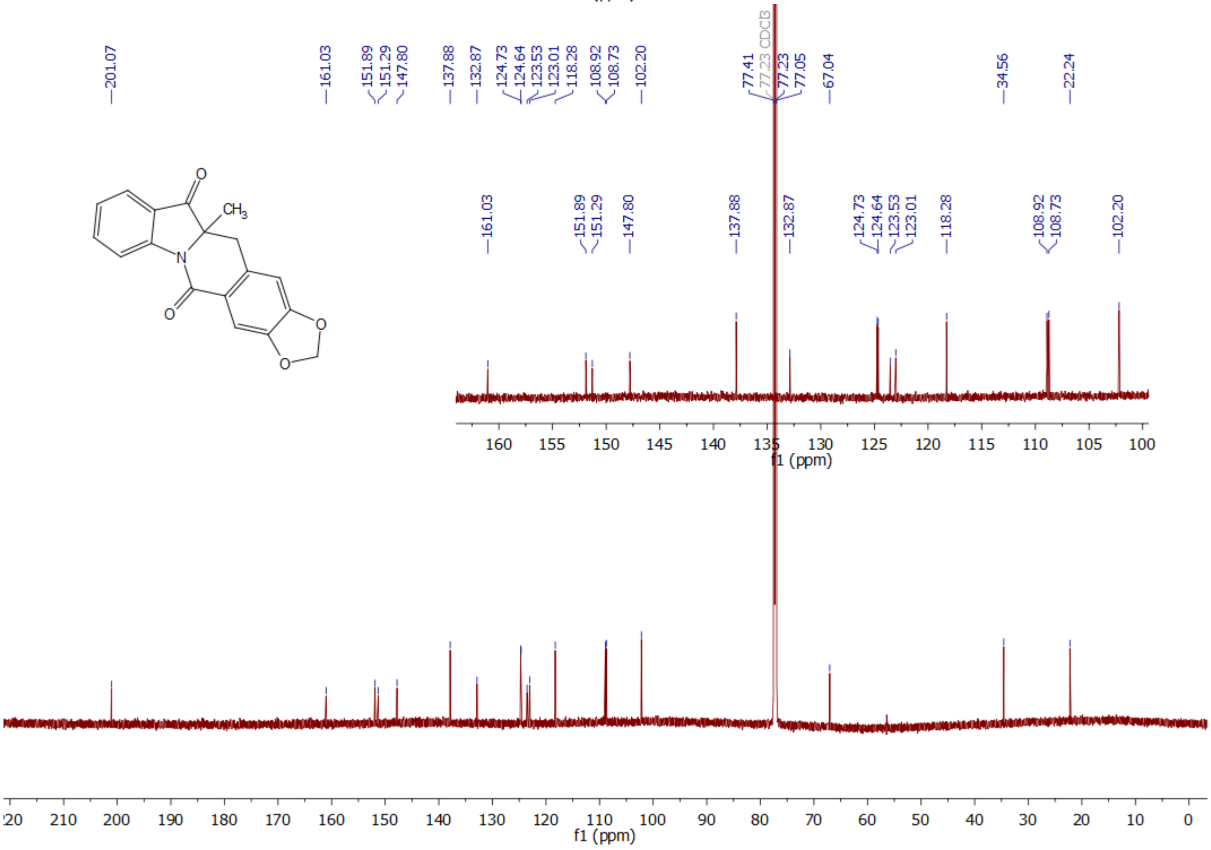

7a-methyl-7,7a-dihydrobenzo[h]indolo[1,2-b]isoquinoline-8,14-dione **[G7]**:

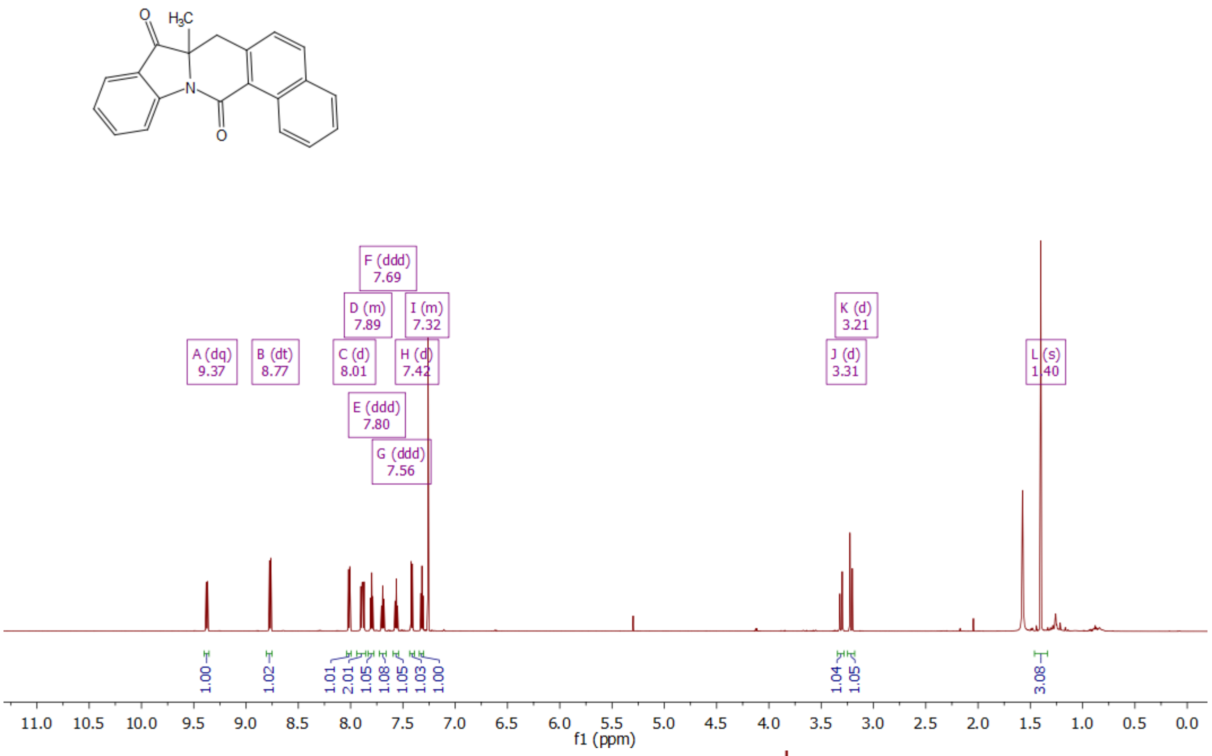

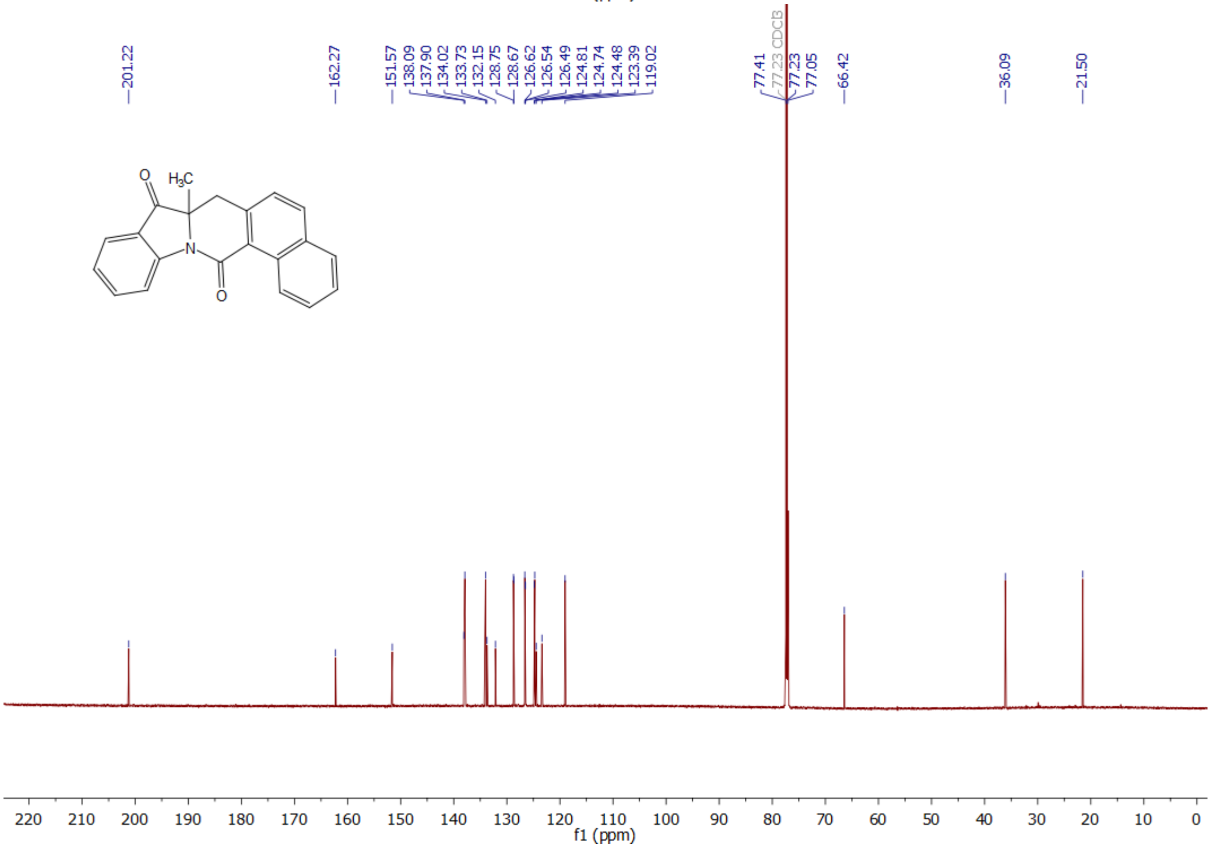

7-fluoro-2,11a-dimethyl-11,11a-dihydroindolo[1,2-b]isoquinoline-6,12-dione [**G8**]:

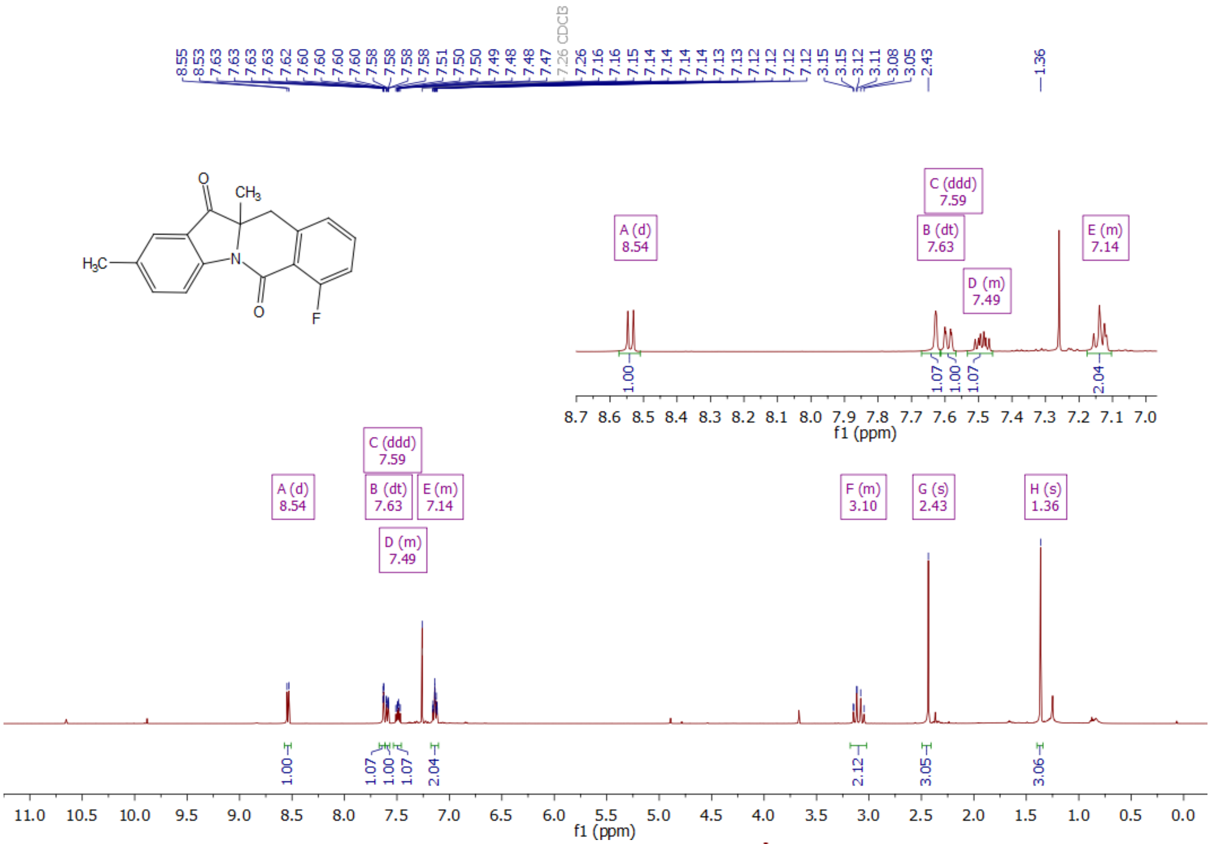

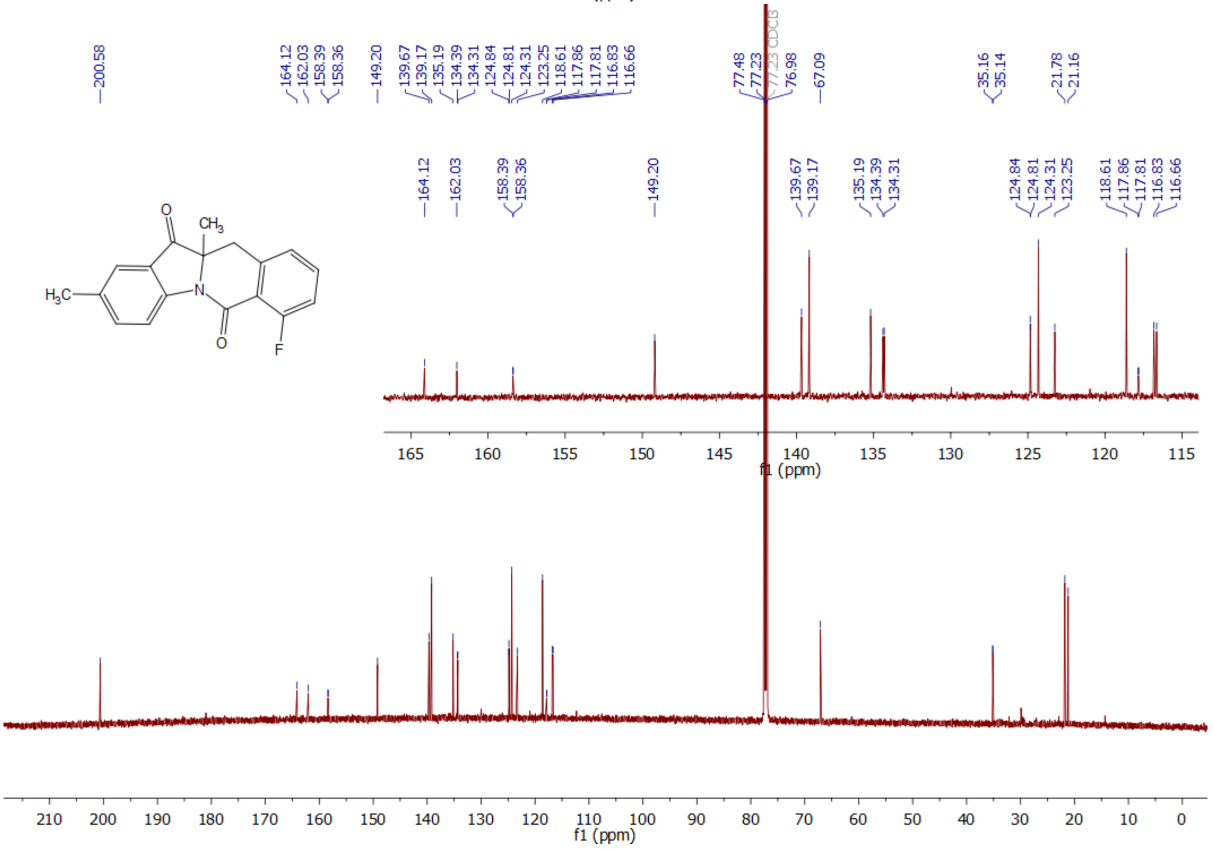

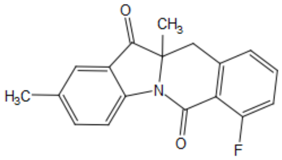

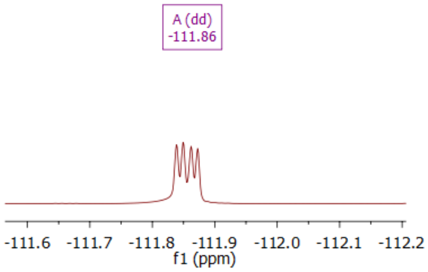
A (dd)
-111.86
-111.6 -111.7 -111.8 -111.9 -112.0 -112.1 -112.2
f1 (ppm)

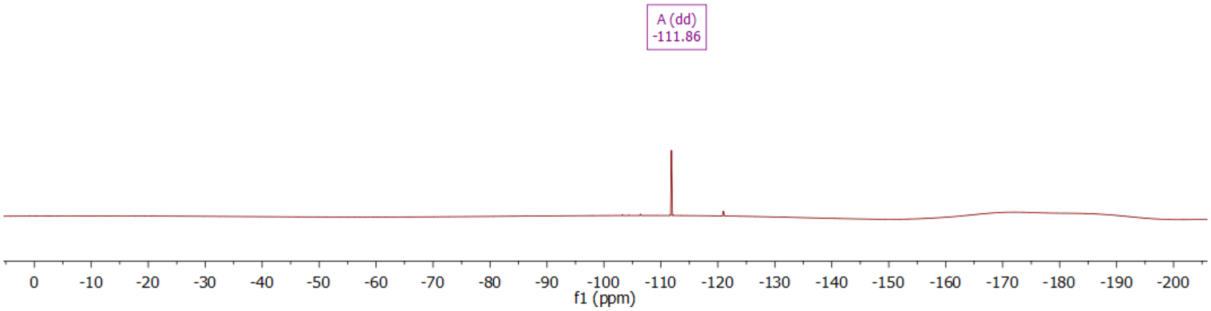
A (dd)
-111.86
0 -10 -20 -30 -40 -50 -60 -70 -80 -90 -100 -110 -120 -130 -140 -150 -160 -170 -180 -190 -200
f1 (ppm)

8-chloro-2-fluoro-11a-methyl-11,11a-dihydroindolo[1,2-b]isoquinoline-6,12-dione [**G9**]:

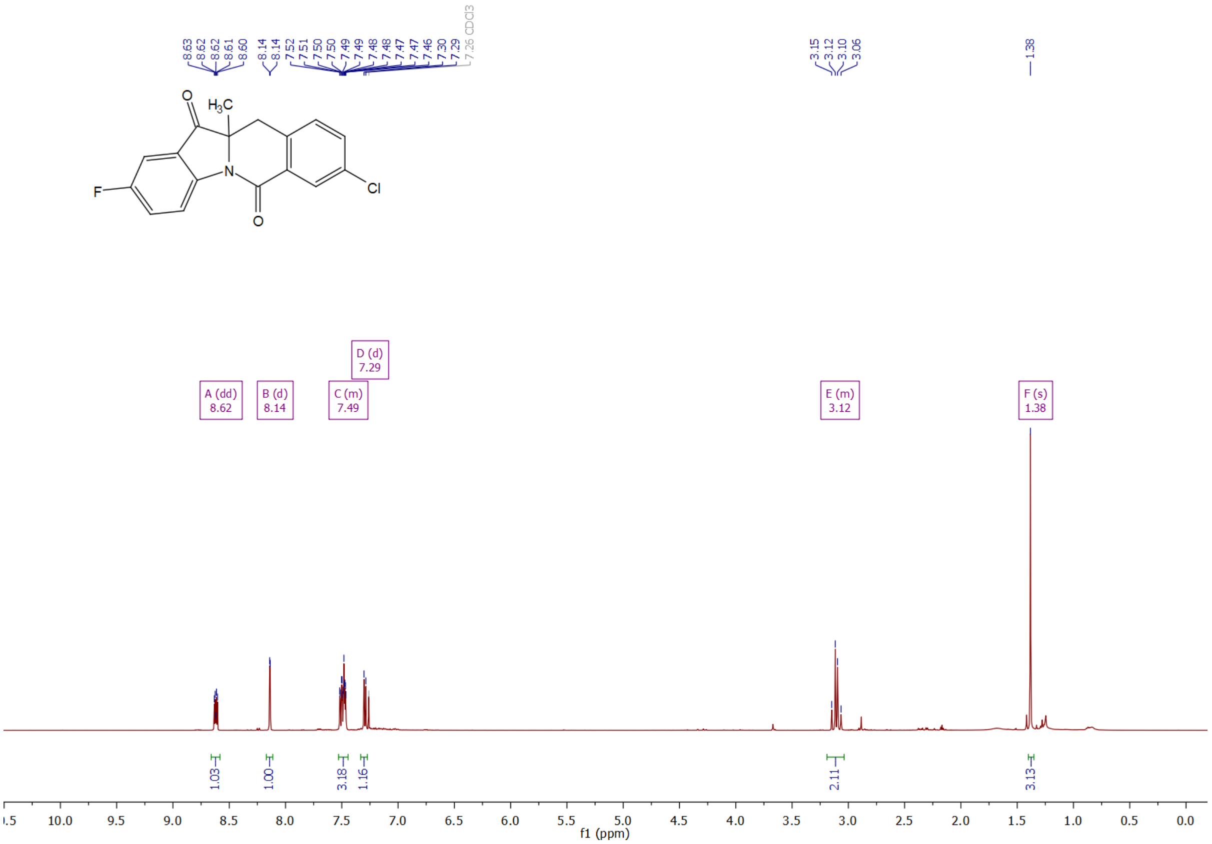

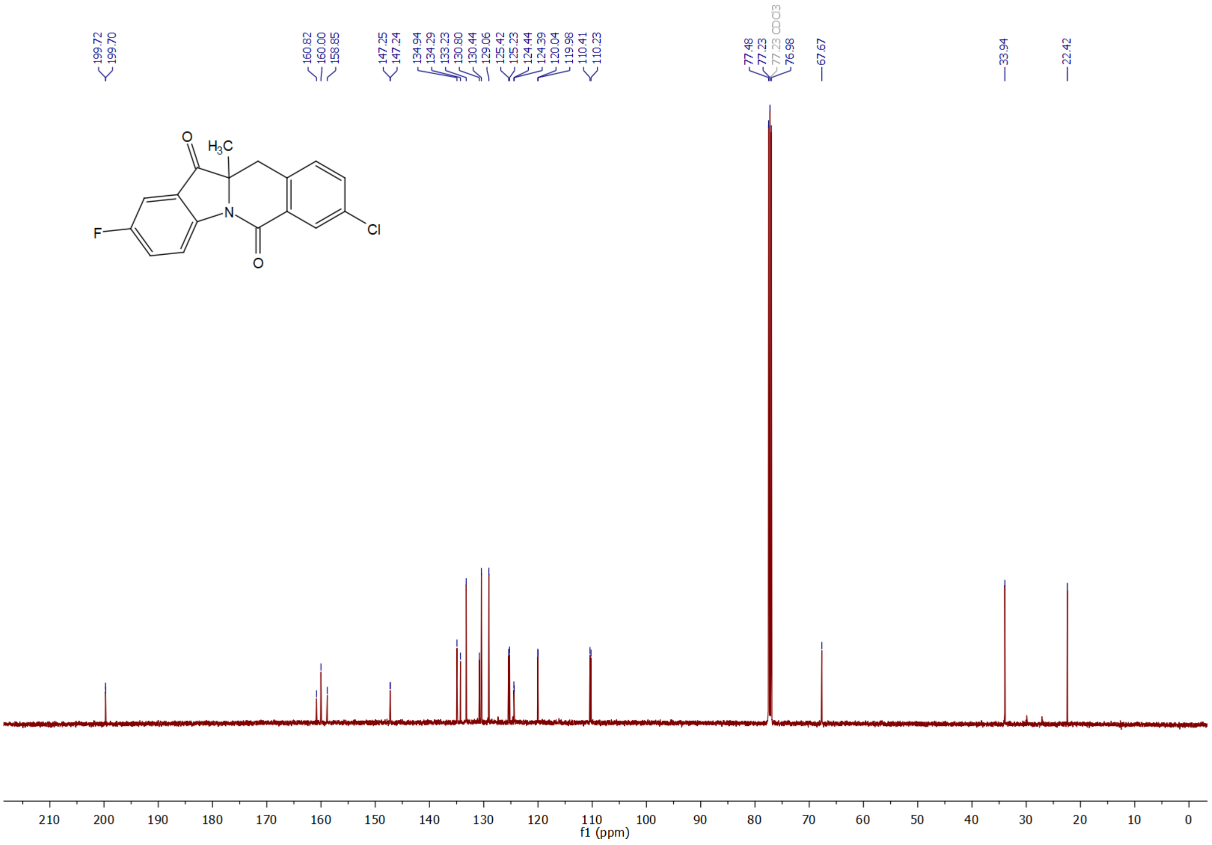

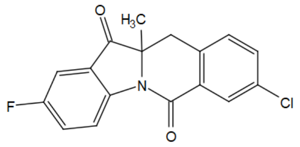
O
H3C
F
N
O
Cl

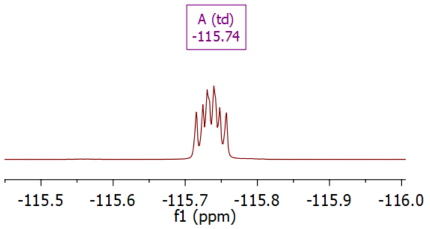
A (td)
-115.74
-115.5
-115.6
-115.7
-115.8
-115.9
-116.0
f1 (ppm)

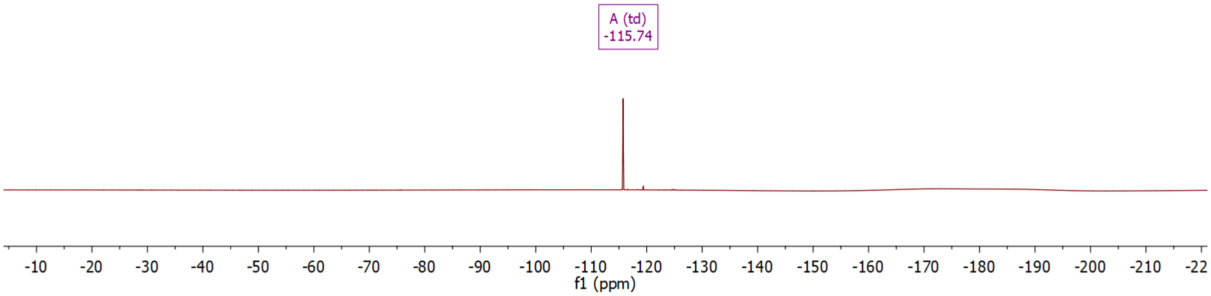
A (td)
-115.74
-10
-20
-30
-40
-50
-60
-70
-80
-90
-100
-110
-120
-130
-140
-150
-160
-170
-180
-190
-200
-210
-22
f1 (ppm)

8-chloro-2,11a-dimethyl-11,11a-dihydroindolo[1,2-b]isoquinoline-6,12-dione [**G10**]:

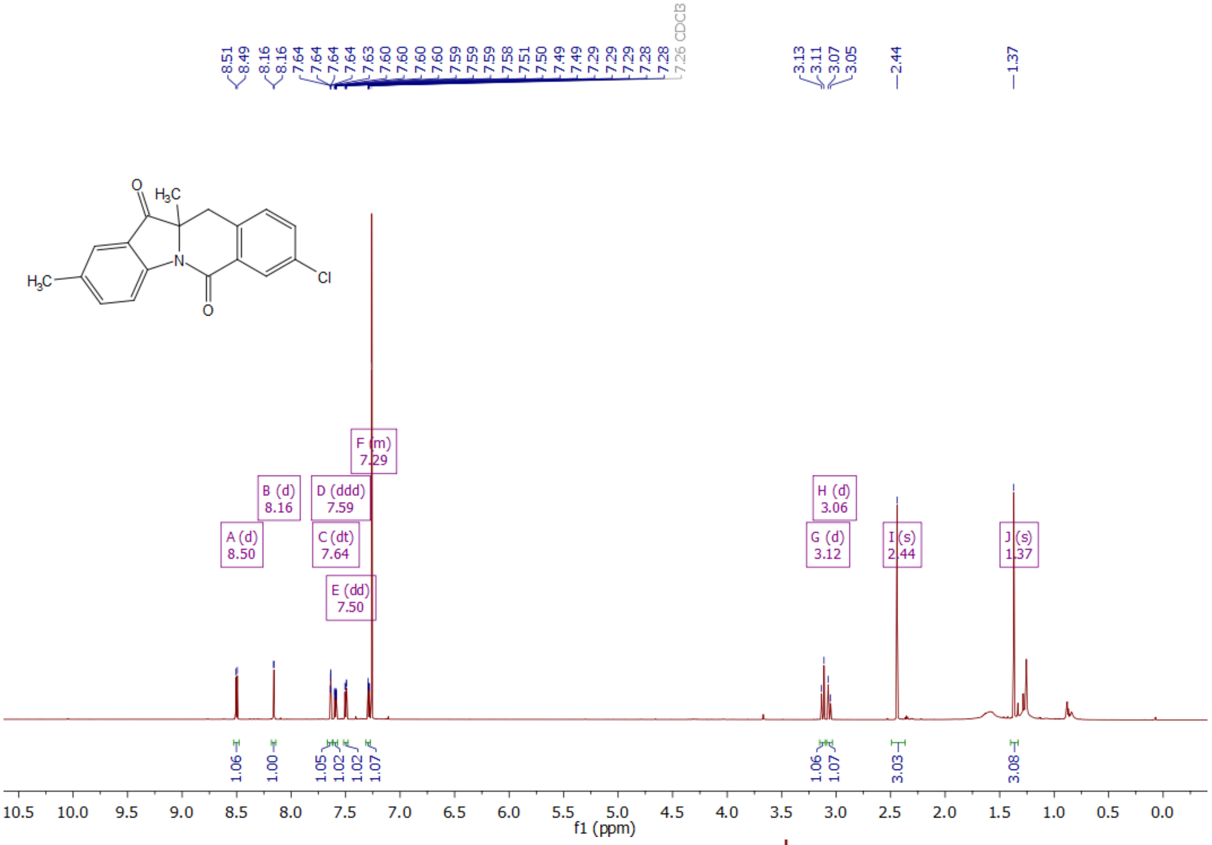

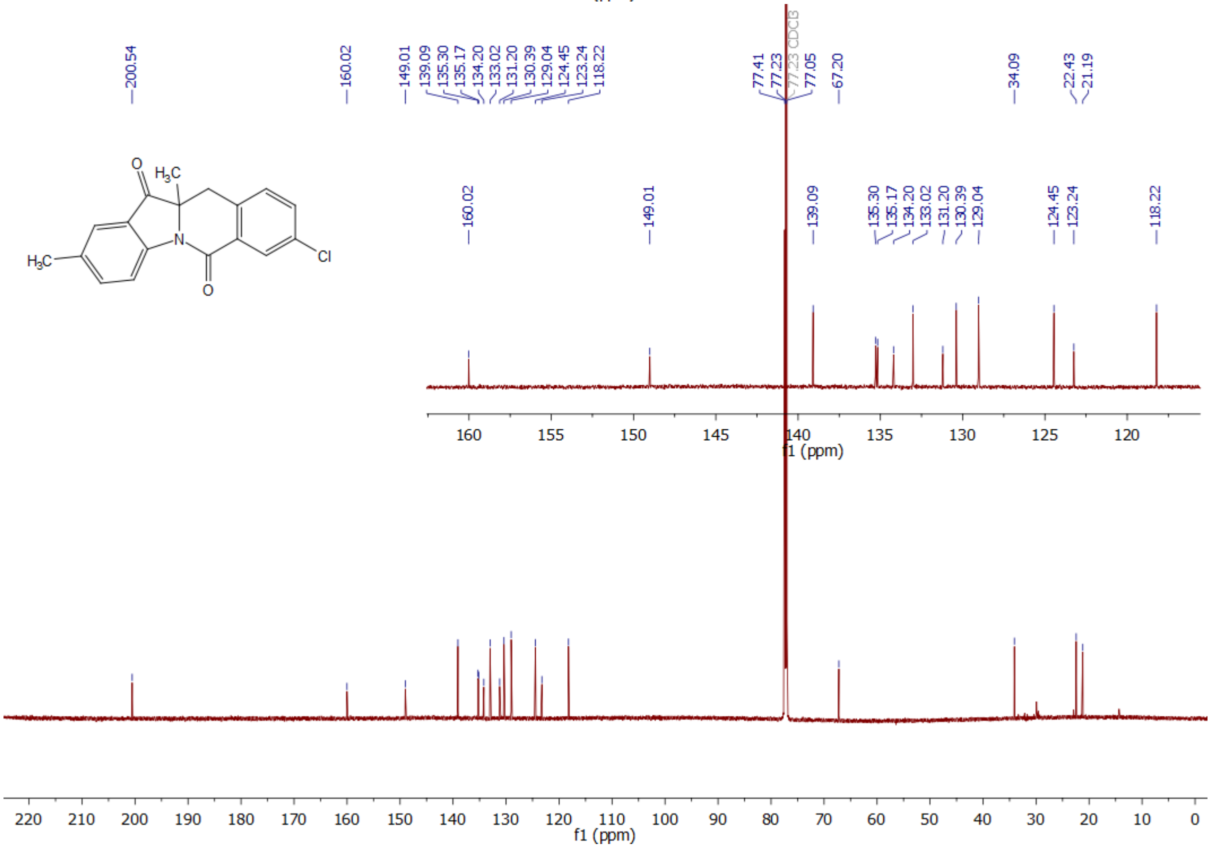

2-chloro-8,11a-dimethyl-11,11a-dihydroindolo[1,2-b]isoquinoline-6,12-dione [G11]:

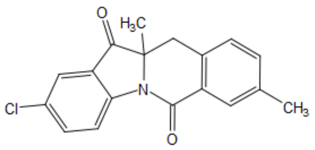

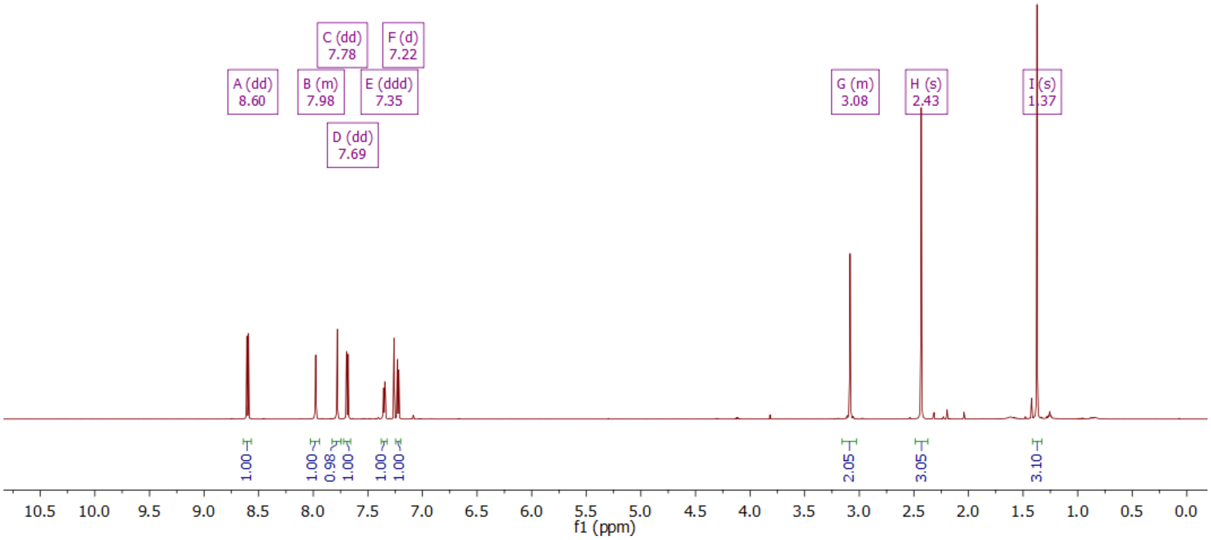

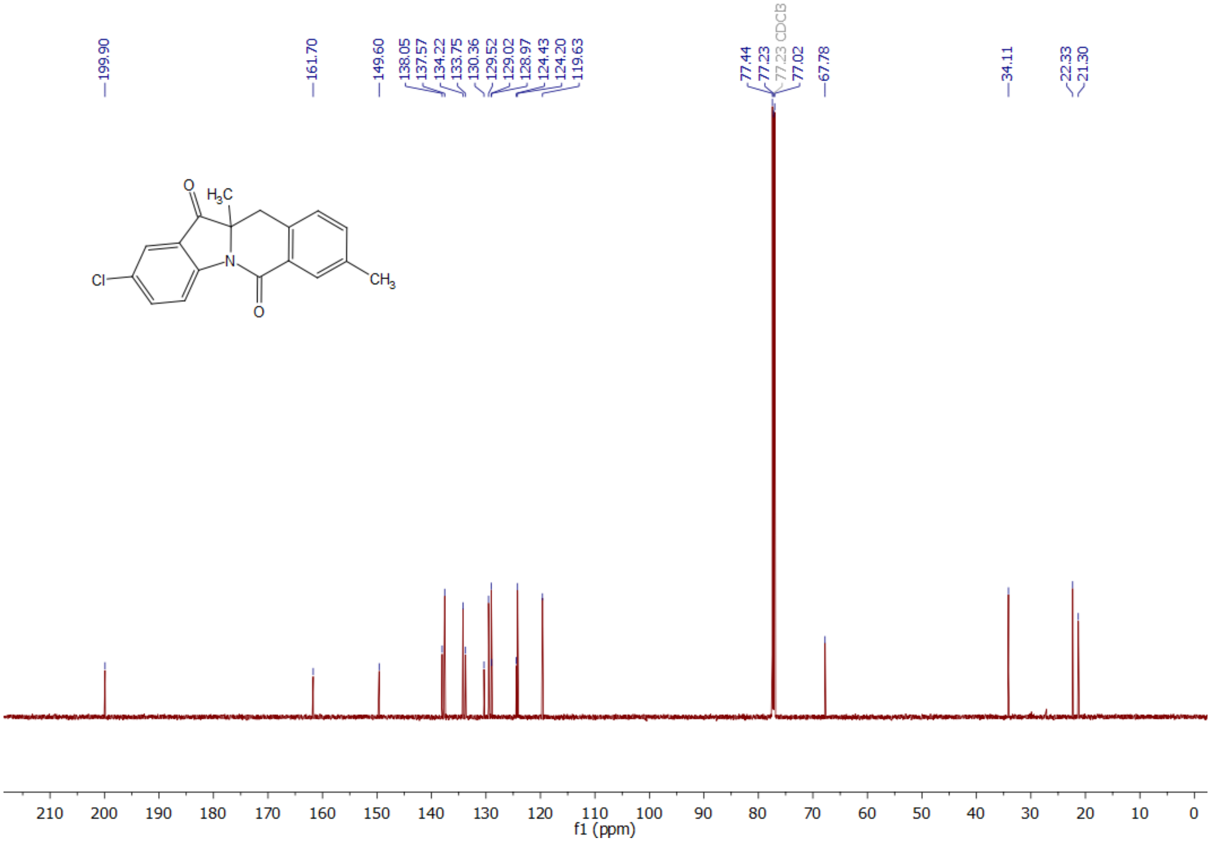

2-fluoro-8-methoxy-11a-methyl-11,11a-dihydroindolo[1,2-b]isoquinoline-6,12-dione [G12]:

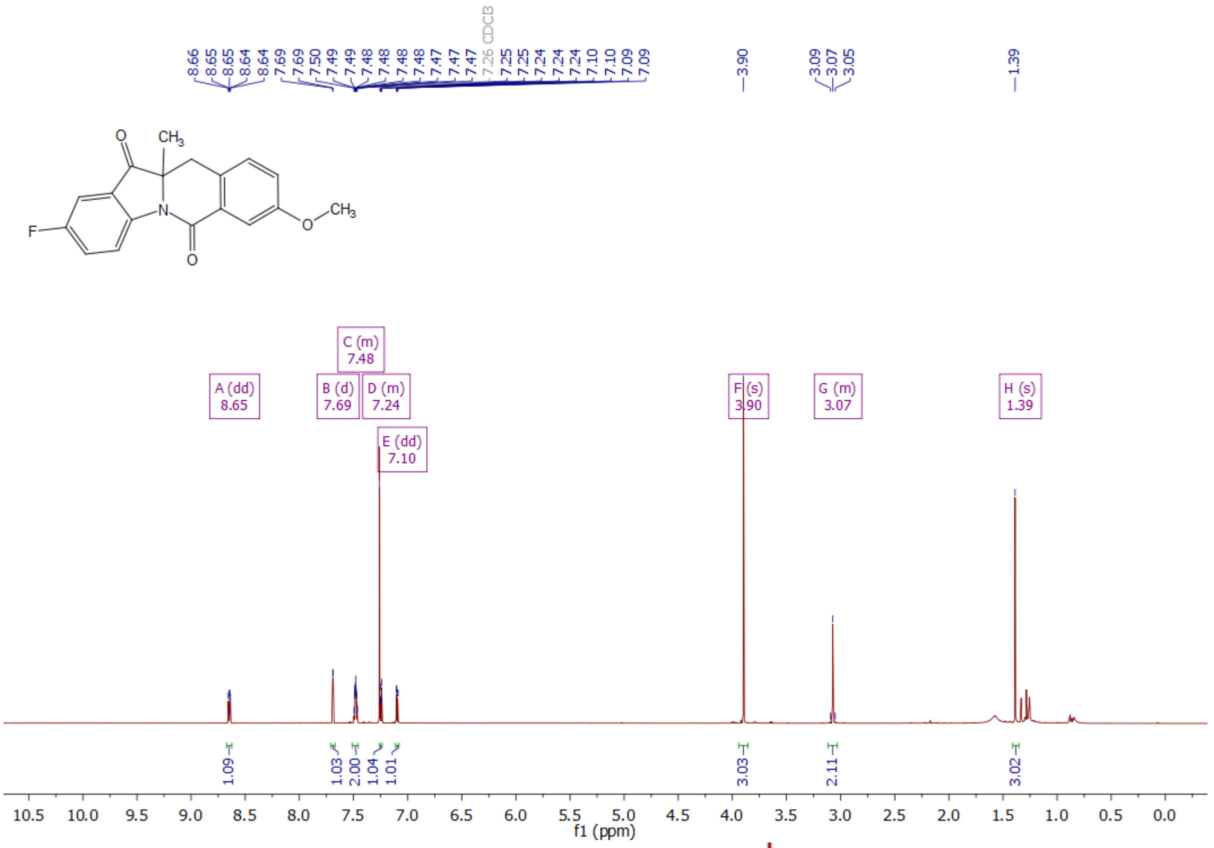

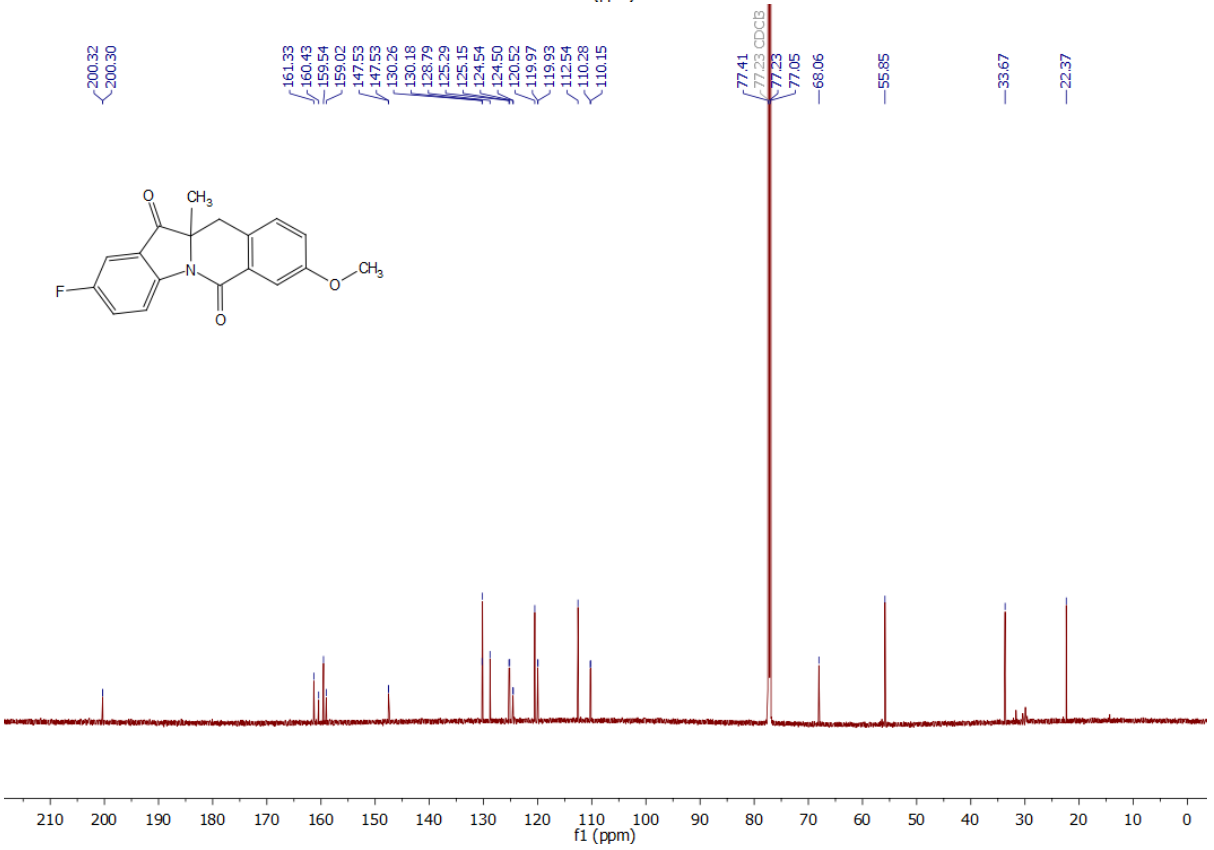

2-chloro-9-fluoro-11a-methyl-11,11a-dihydroindolo[1,2-b]isoquinoline-6,12-dione [**G13**]:

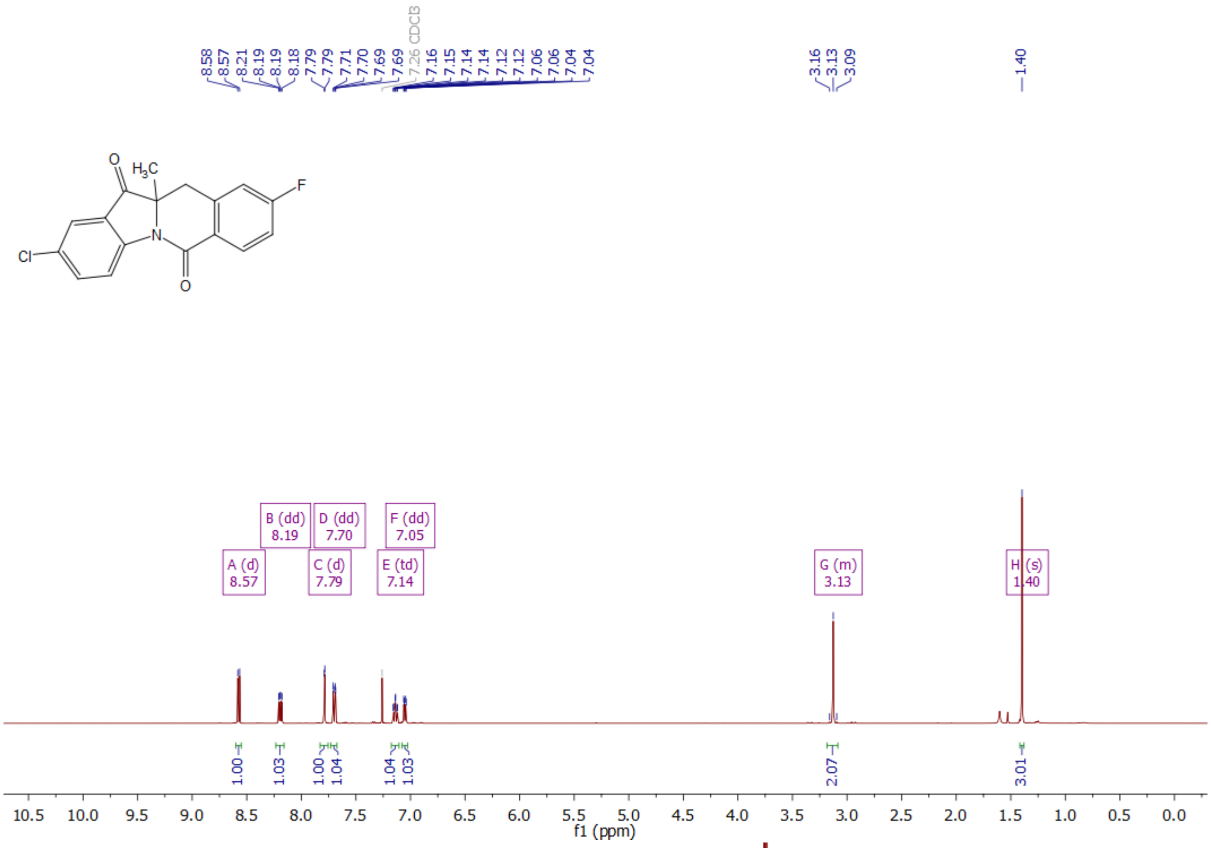

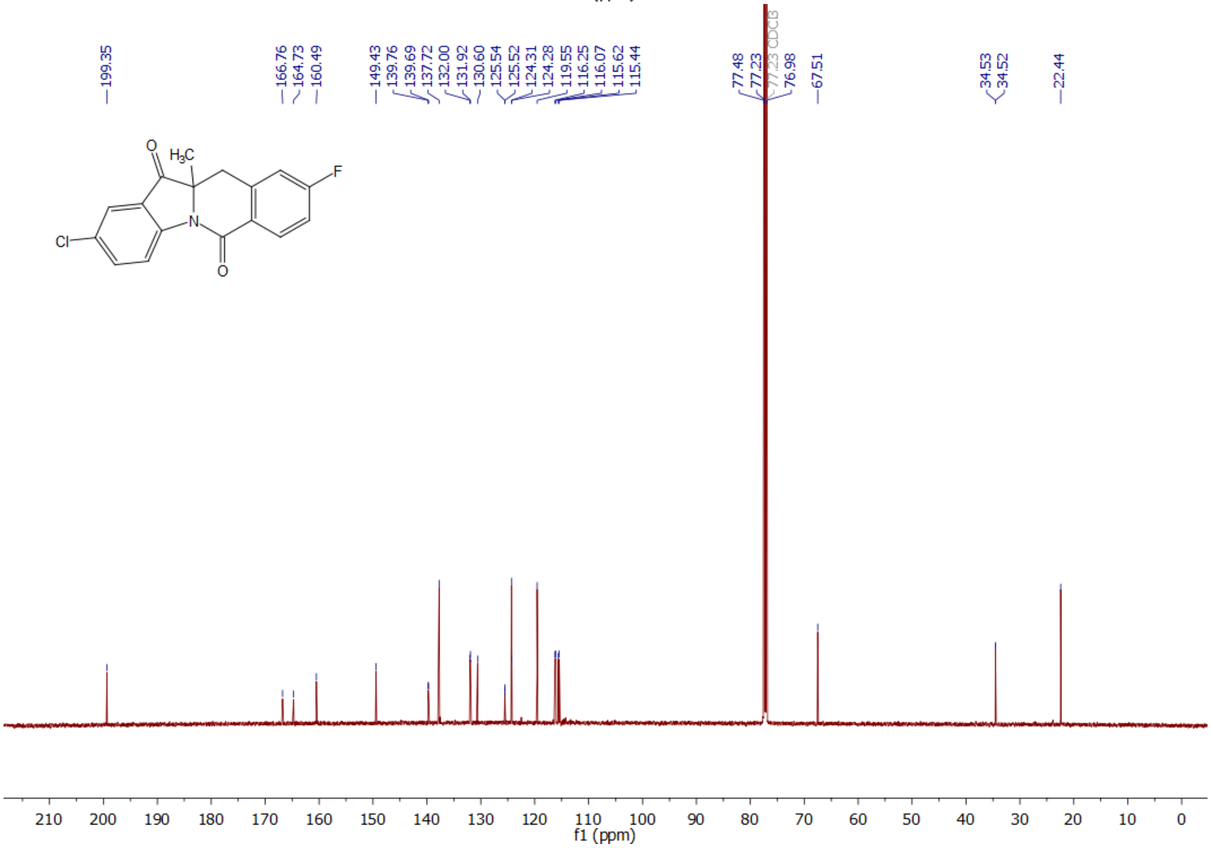

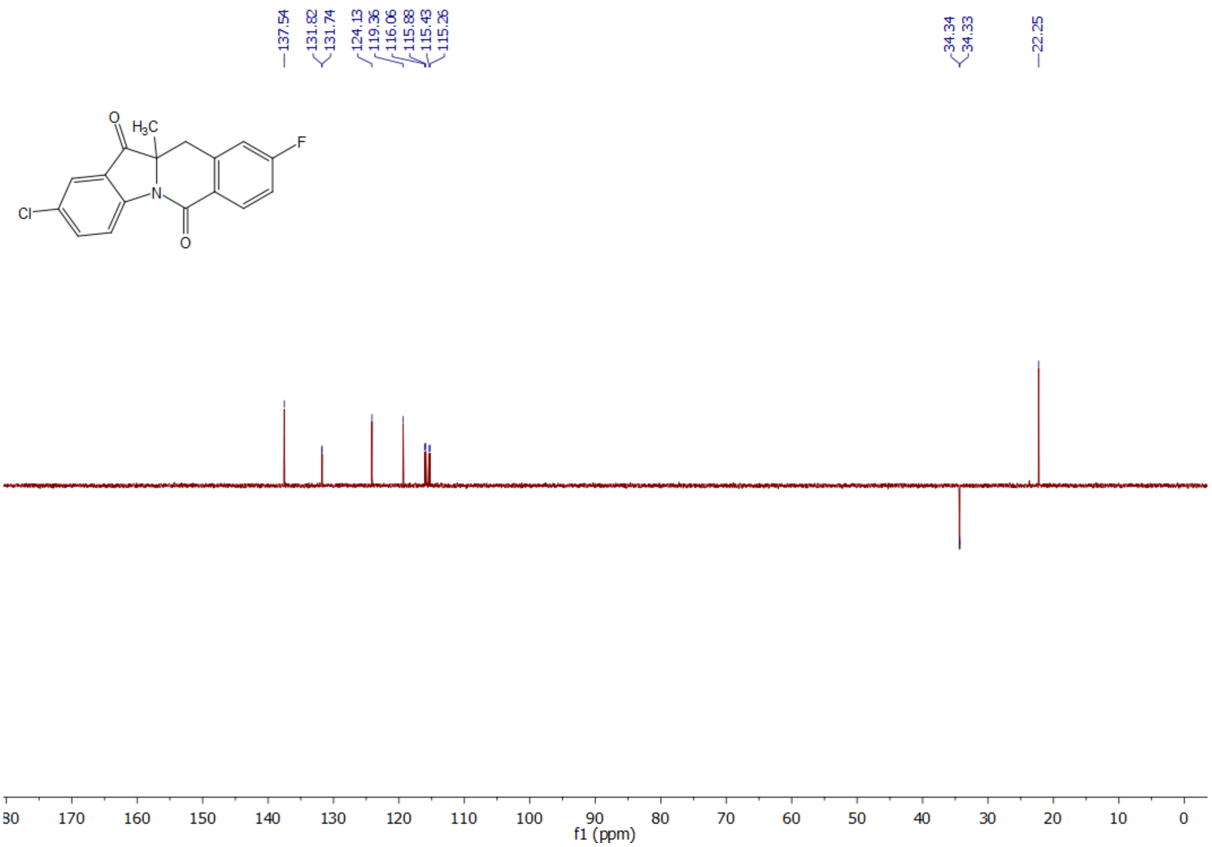
137.54
131.82
131.74
124.13
119.36
116.06
115.88
115.43
115.26
34.34
34.33
22.25
O
H3C
F
Cl
N
O
f1 (ppm)

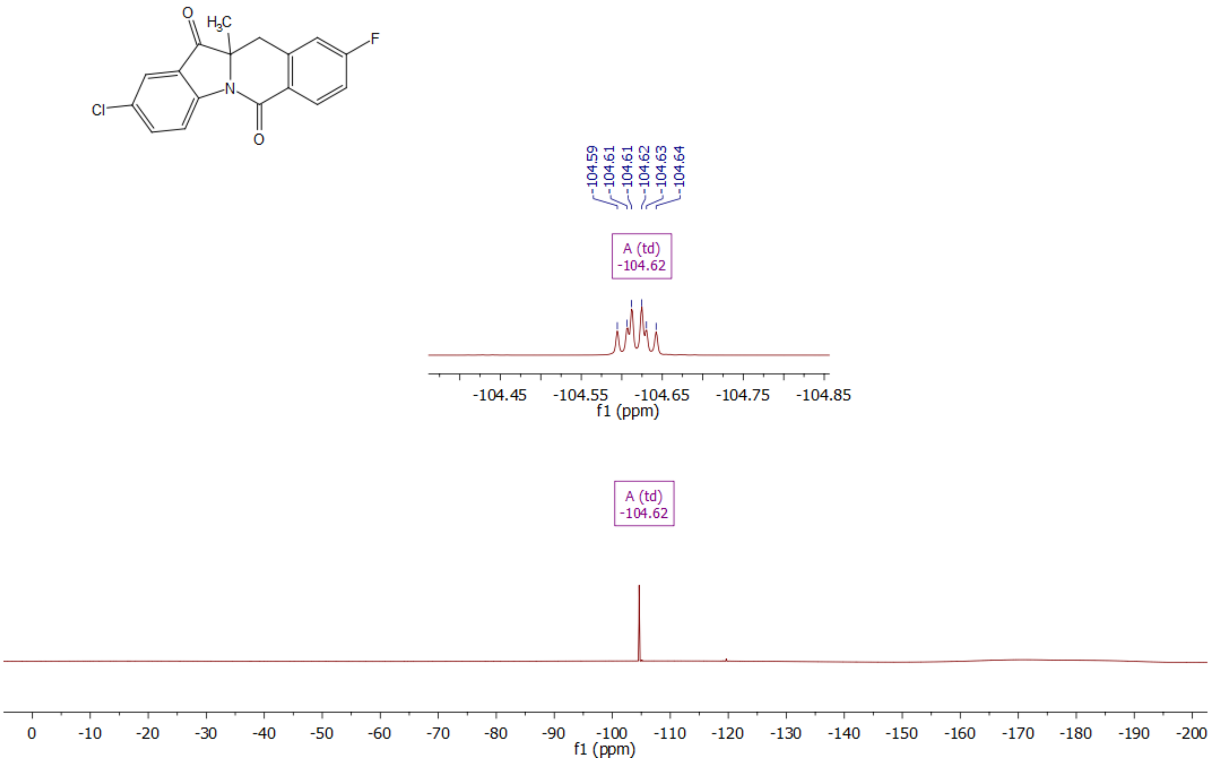
O
H3C
F
Cl
N
O
-104.59
-104.61
-104.61
-104.62
-104.63
-104.64
A (td)
-104.62
f1 (ppm)
A (td)
-104.62
f1 (ppm)

9,11a-dimethyl-11a,12-dihydro-[1,3]dioxolo[4,5-g]indolo[1,2-b]isoquinoline-5,11-dione [G14]:

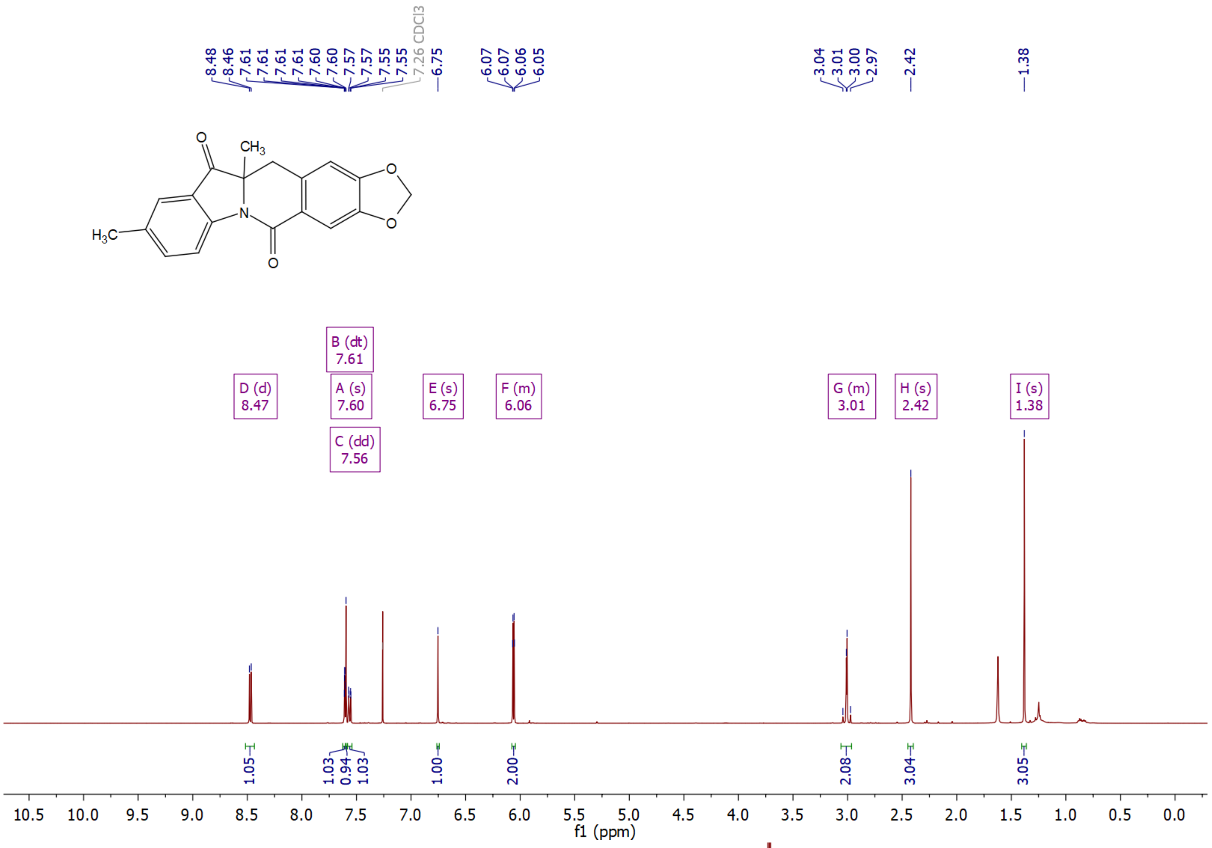

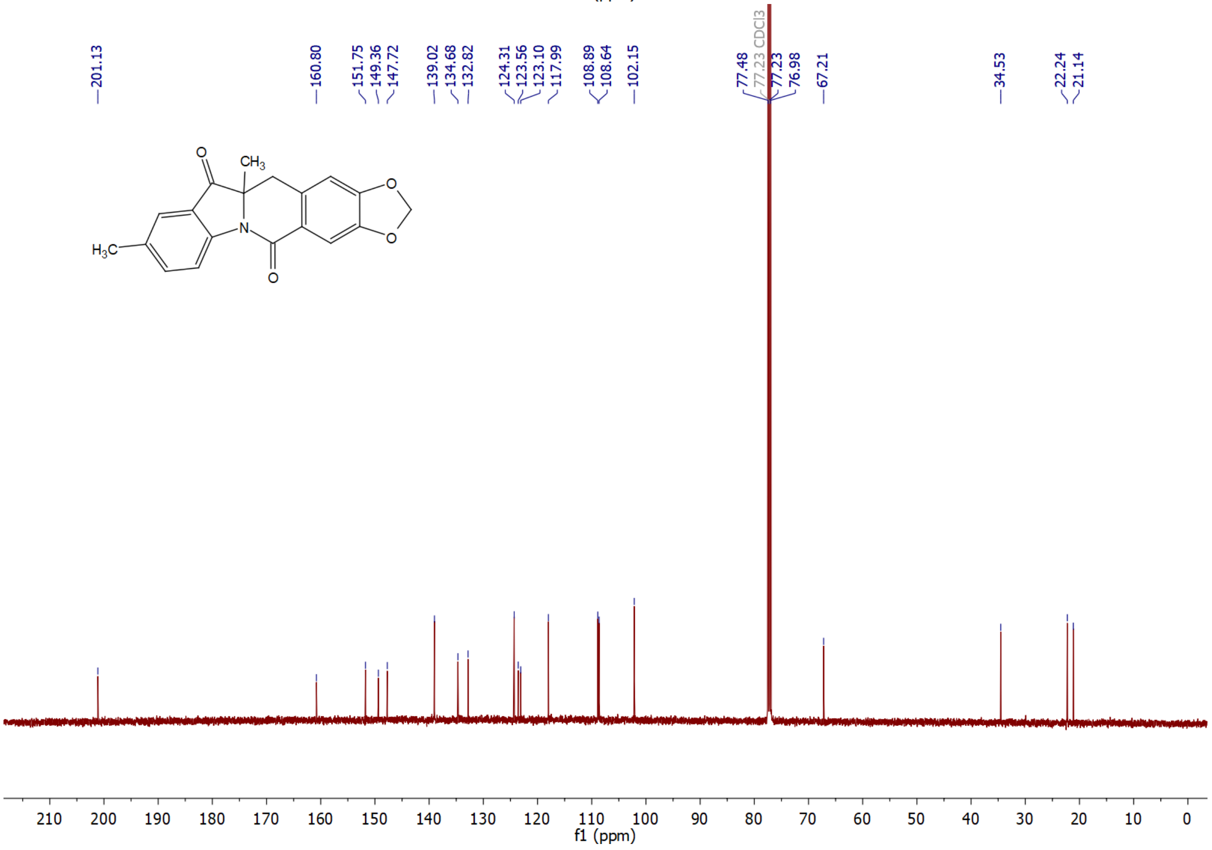

11a-phenyl-11,11a-dihydroindolo[1,2-b]isoquinoline-6,12-dione [G15]:

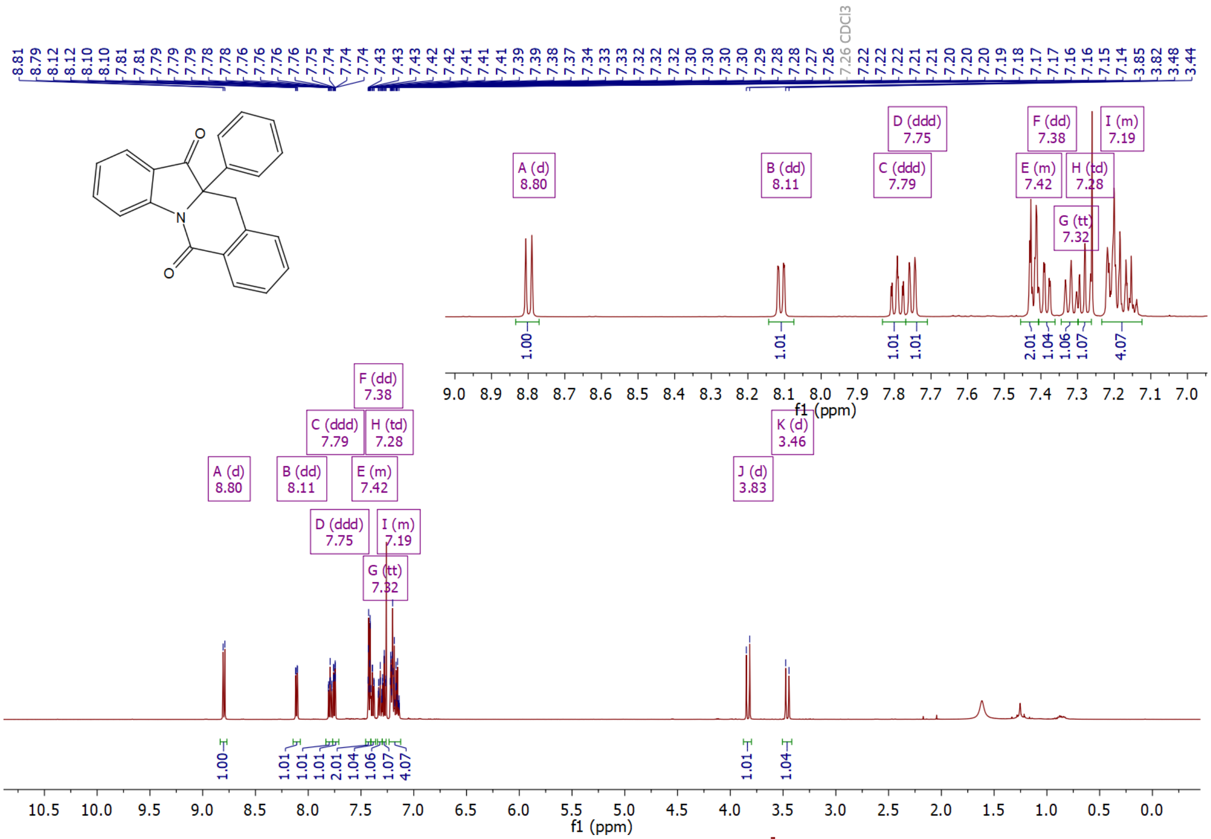

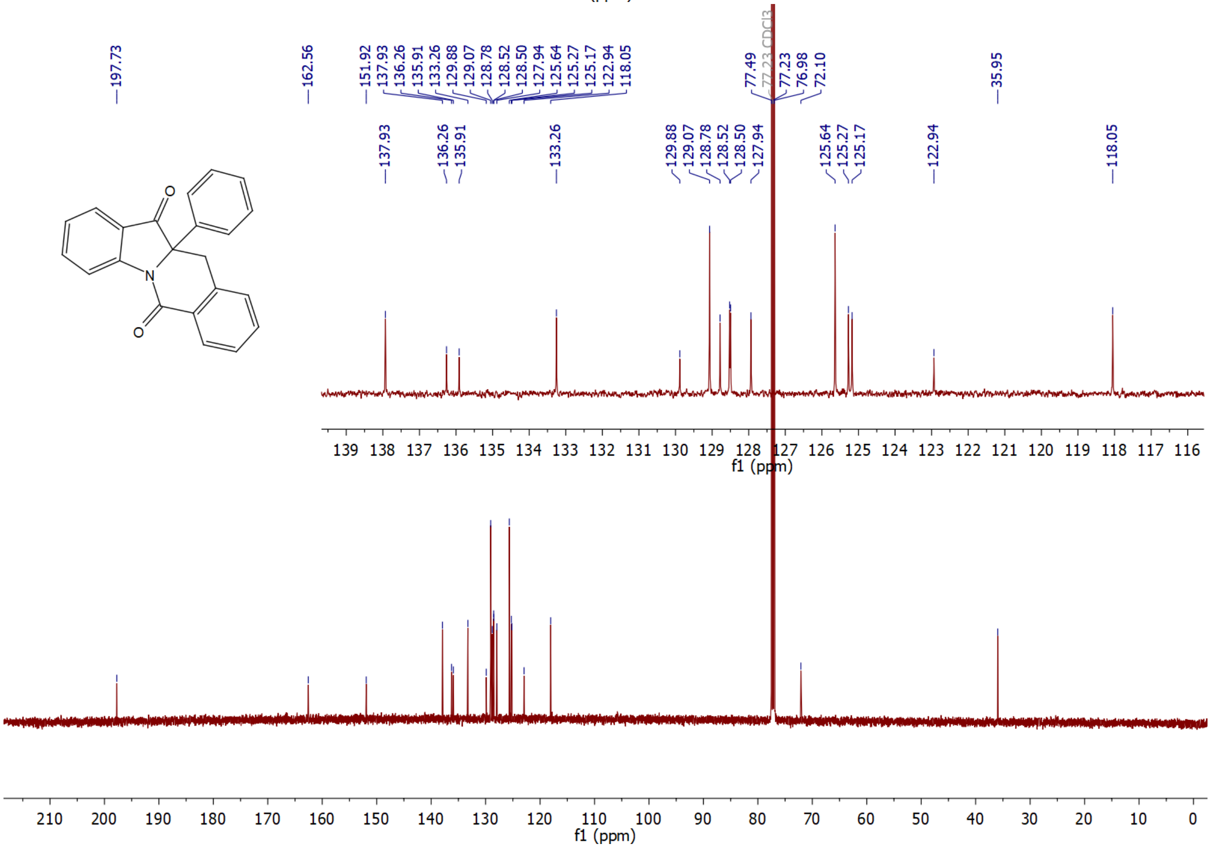

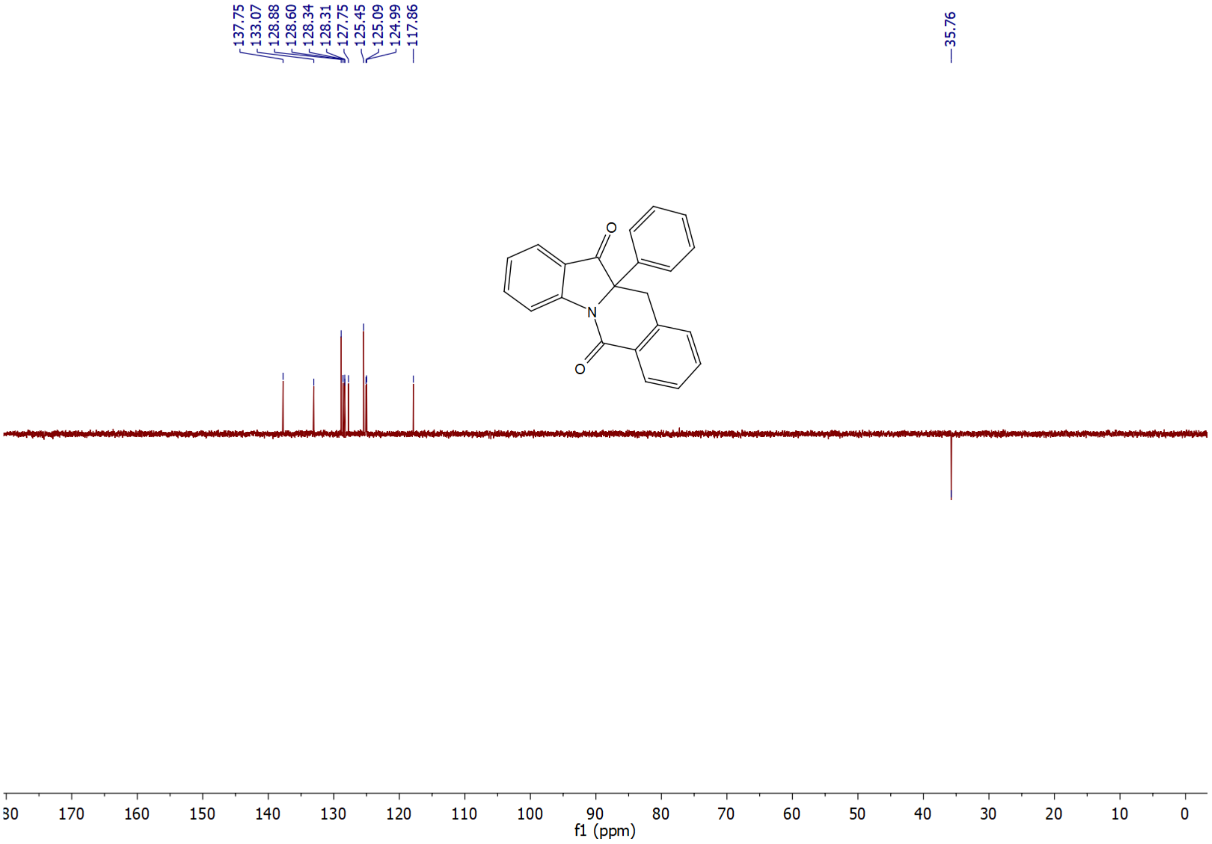
137.75
133.07
128.88
128.60
128.34
128.31
127.75
125.45
125.09
124.99
117.86
35.76
O
N
O
30
170
160
150
140
130
120
110
100
90
80
70
60
50
40
30
20
10
0
f1 (ppm)

8-methyl-11a-phenyl-11,11a-dihydroindolo[1,2-b]isoquinoline-6,12-dione [**G16**]:

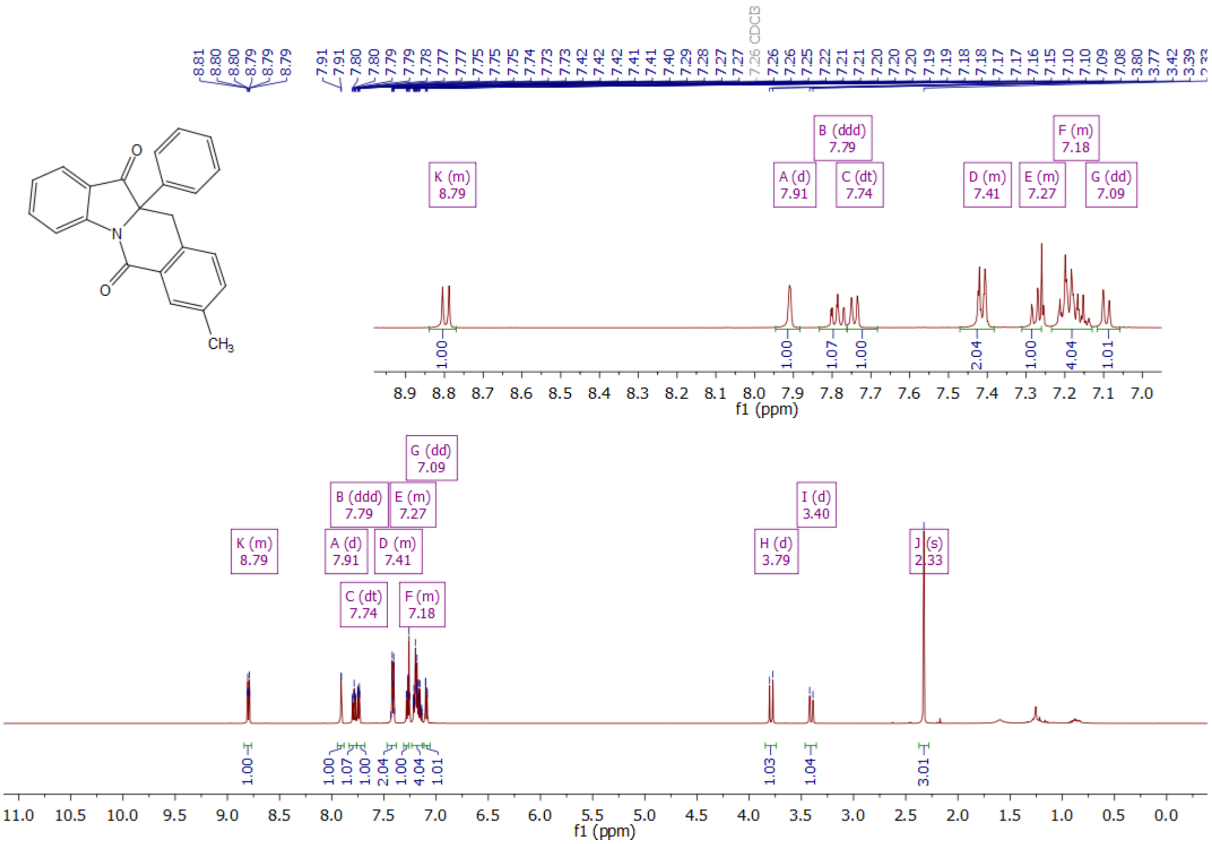

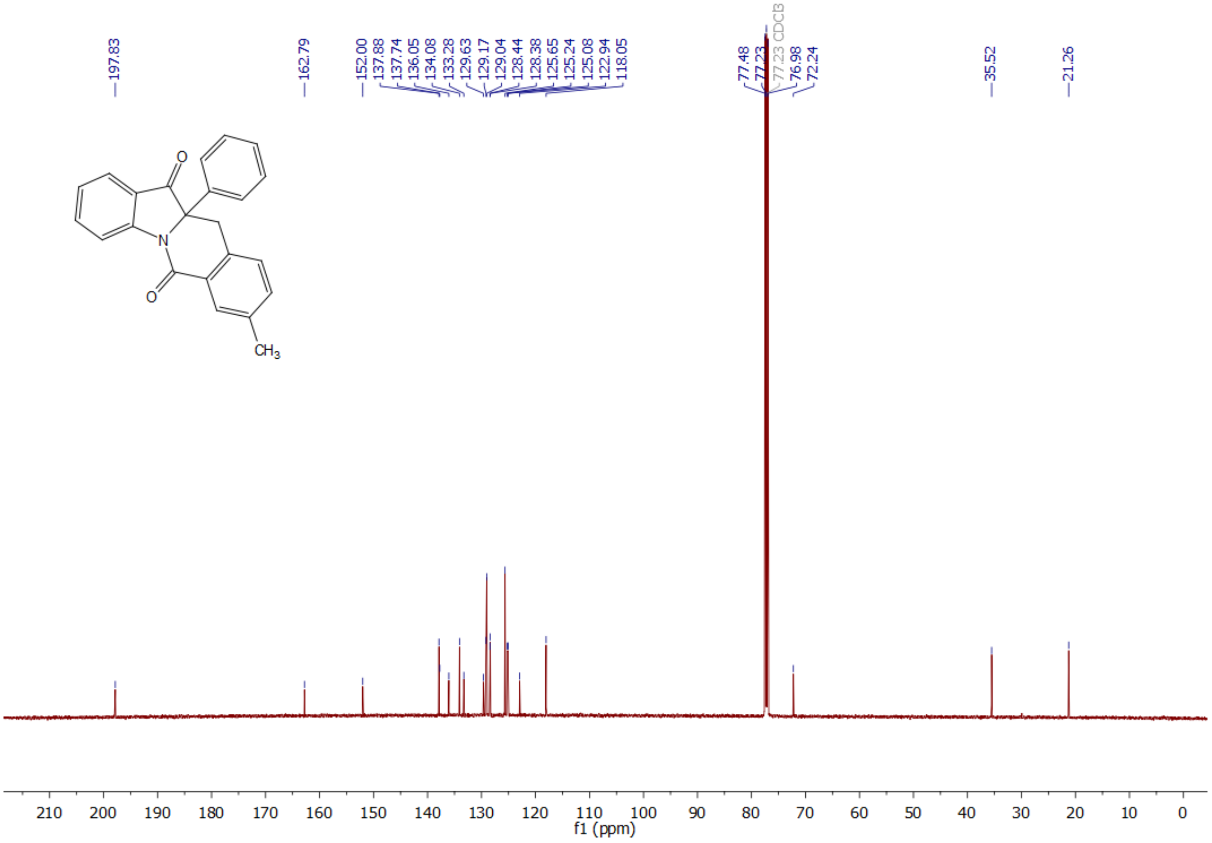

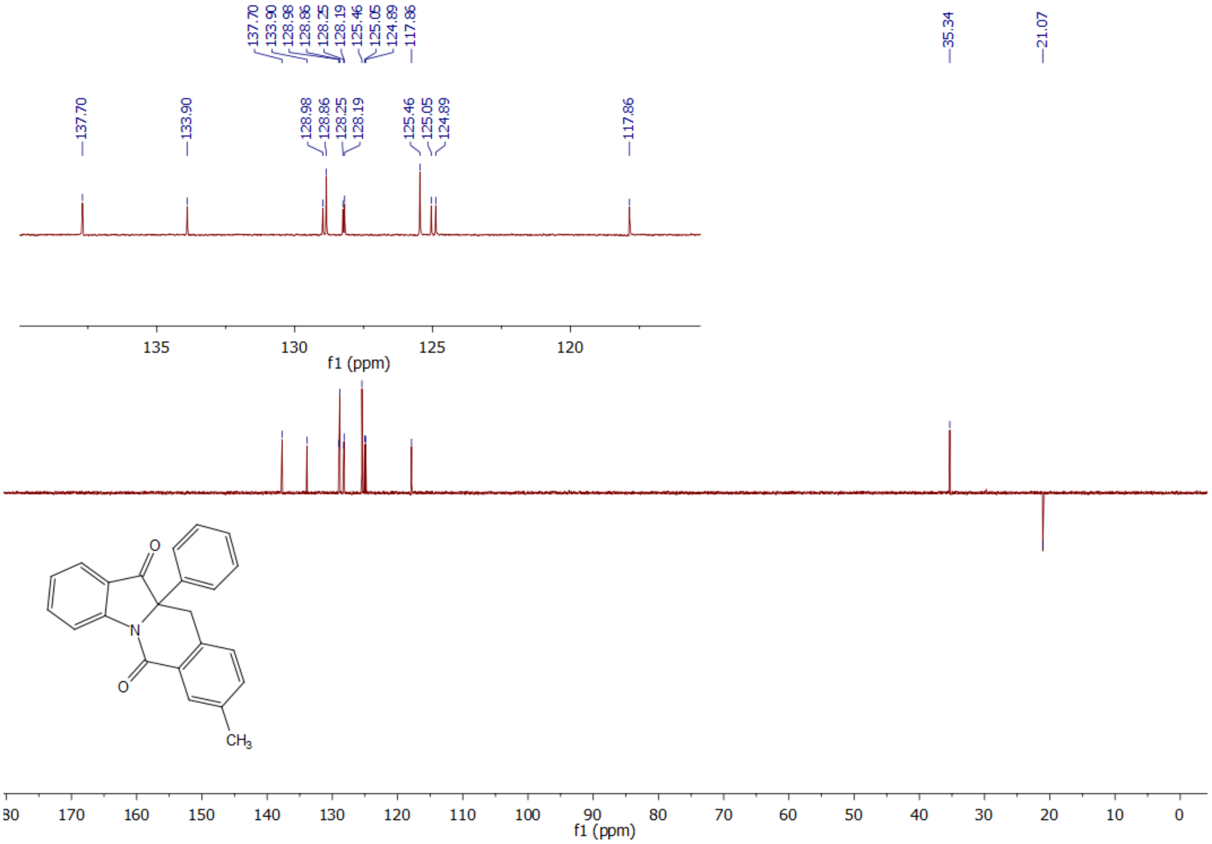
137.70
133.90
128.98
128.86
128.25
128.19
125.46
125.05
124.89
117.86
35.34
21.07
f1 (ppm)
O
N
O
CH3

8-chloro-11a-phenyl-11,11a-dihydroindolo[1,2-b]isoquinoline-6,12-dione [**G17**]:

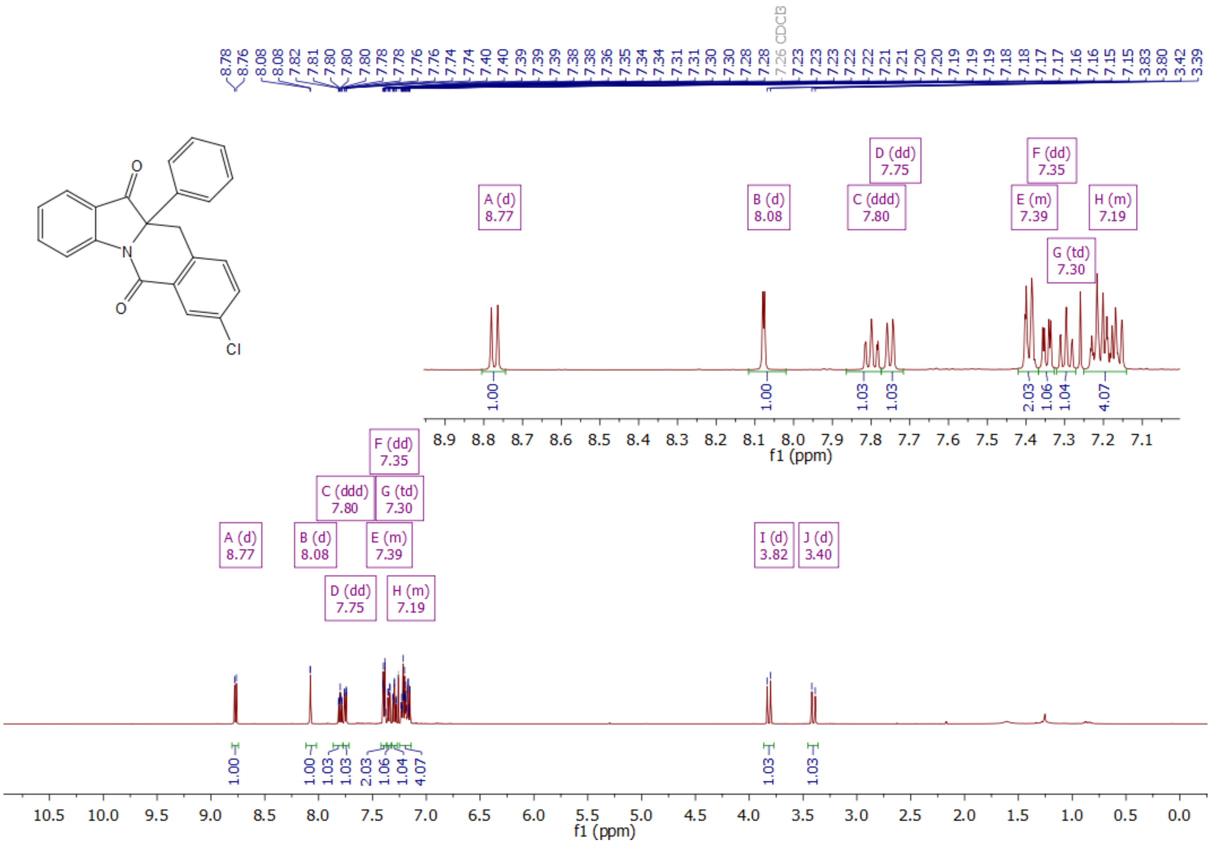

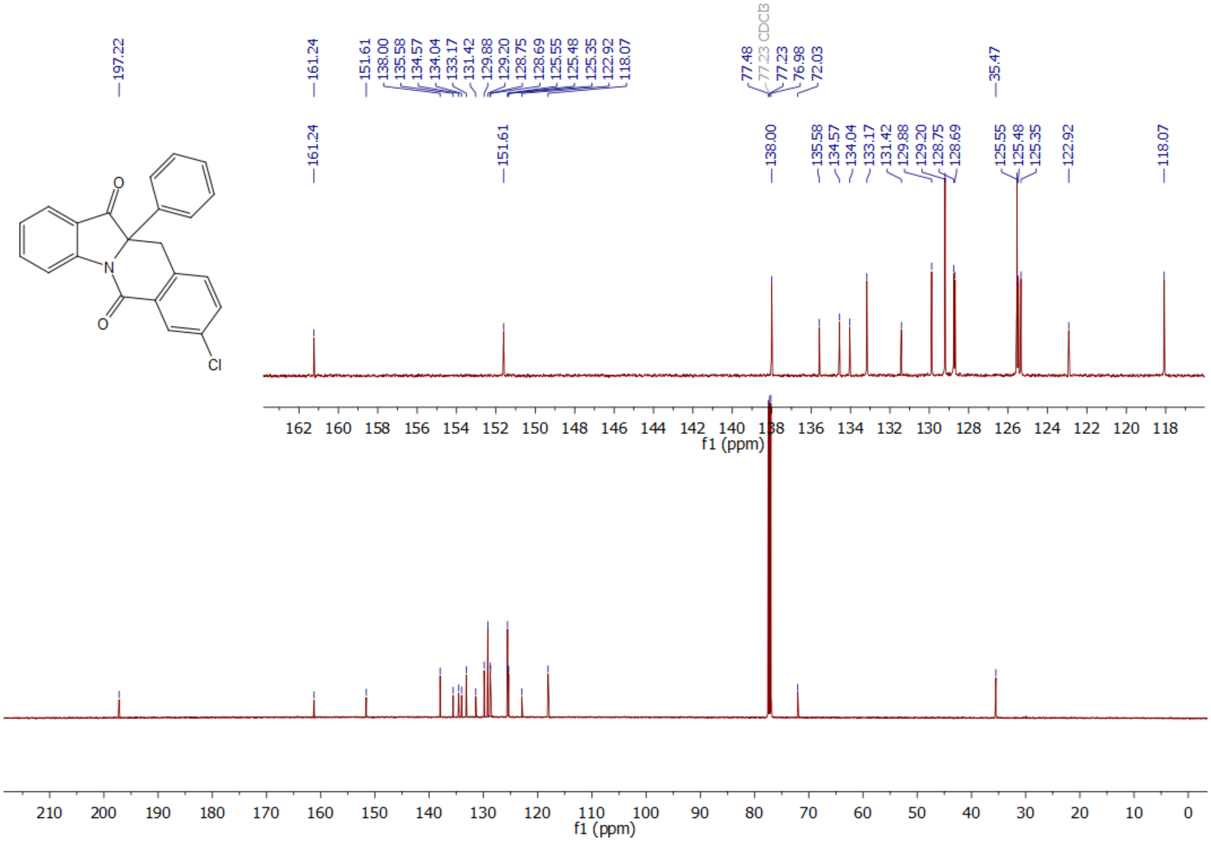

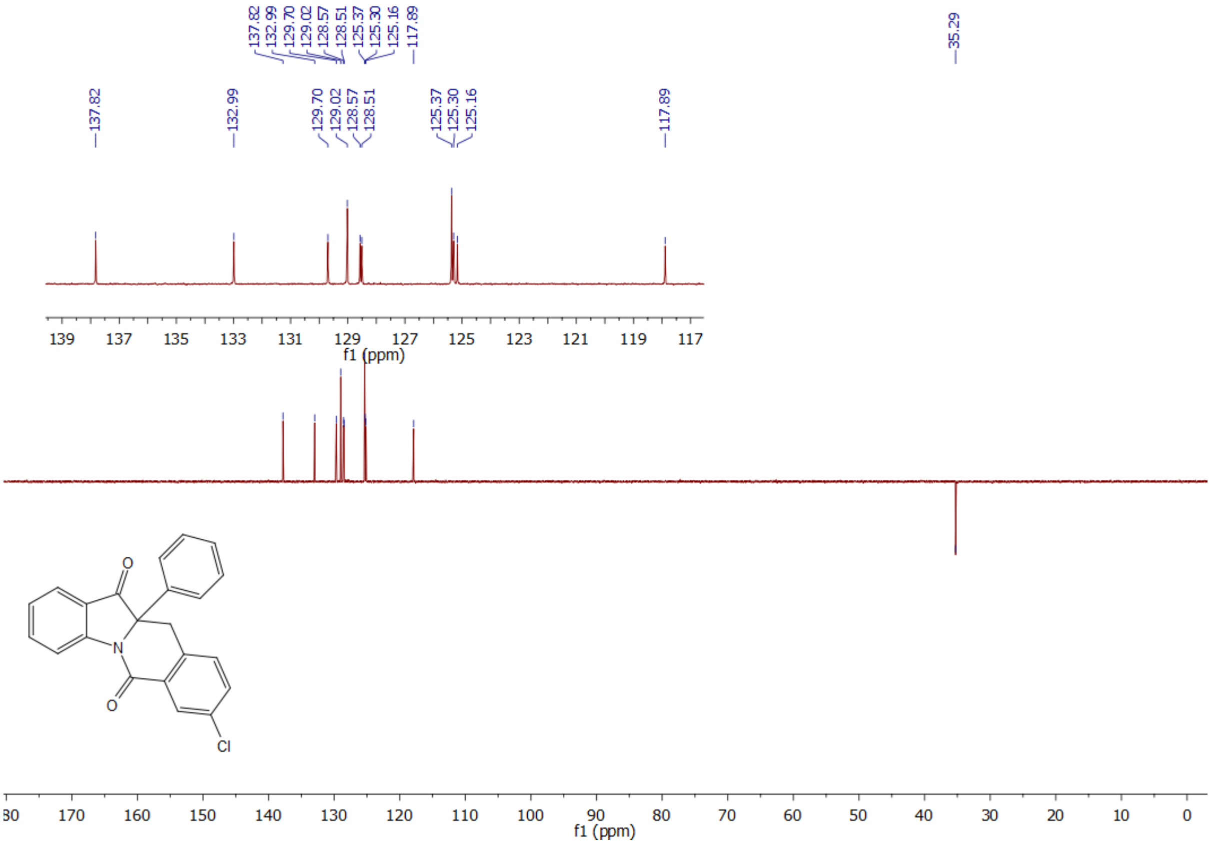
137.82
132.99
129.70
129.02
128.57
128.51
125.37
125.30
125.16
117.89
35.29
f1 (ppm)
O
N
O
Cl

11a-phenyl-11a,12-dihydro-[1,3]dioxolo[4,5-g]indolo[1,2-b]isoquinoline-5,11-dione **[G18]**:

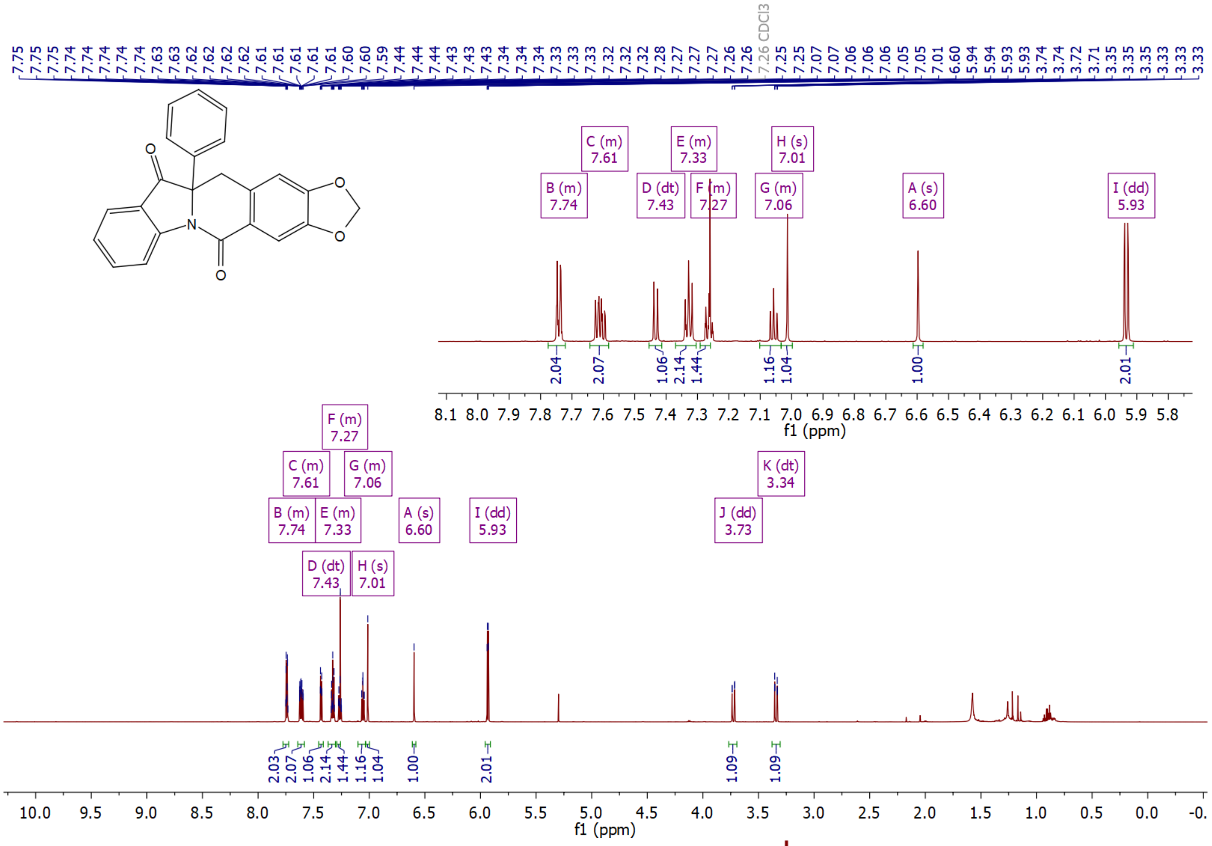

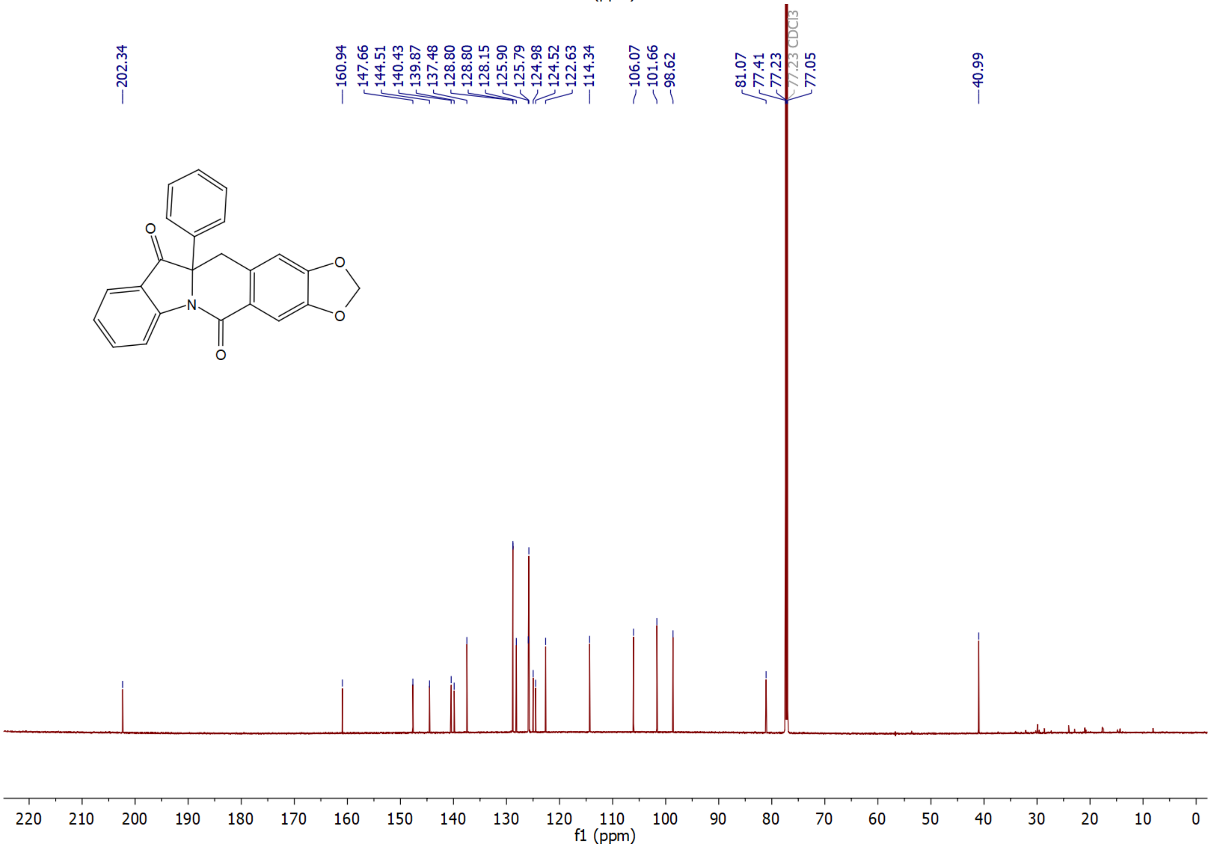

11a-(4-chlorophenyl)-9-methoxy-11,11a-dihydroindolo[1,2-b]isoquinoline-6,12-dione [G19]:

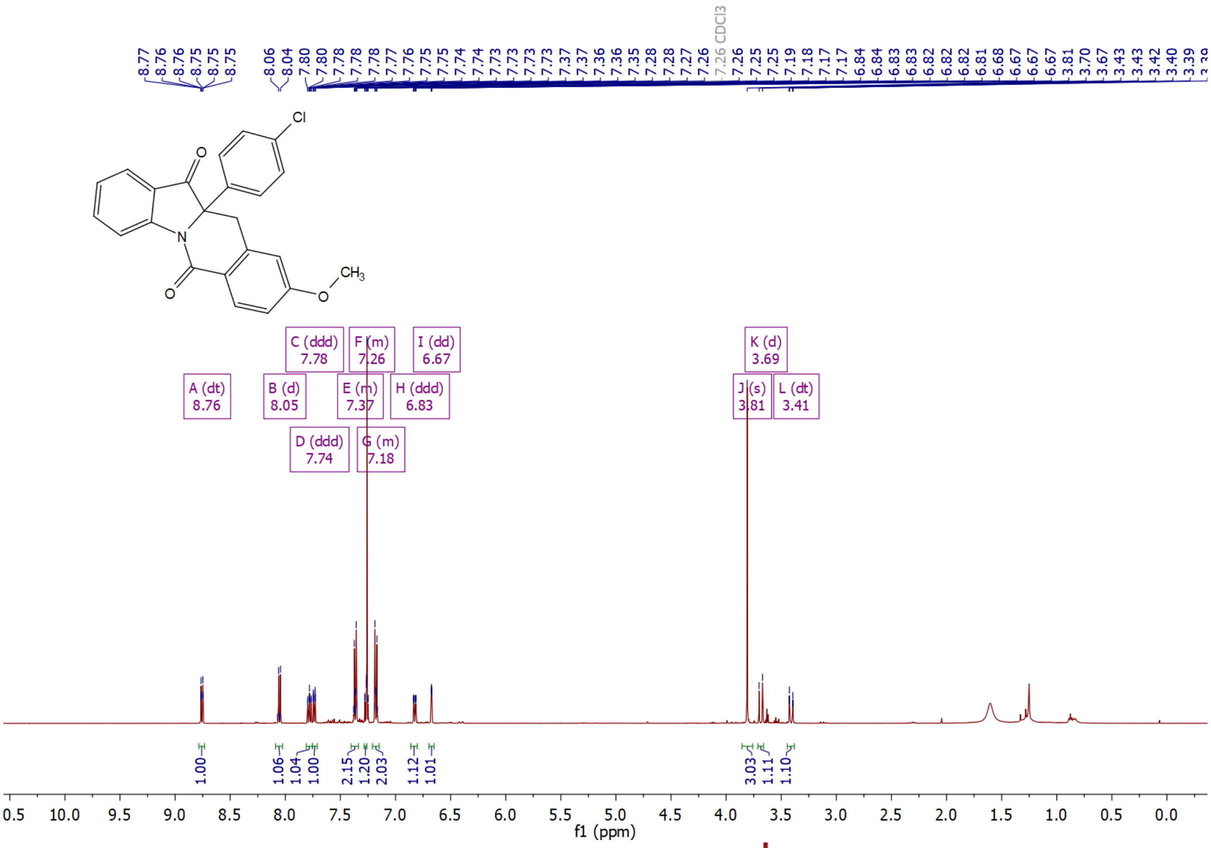

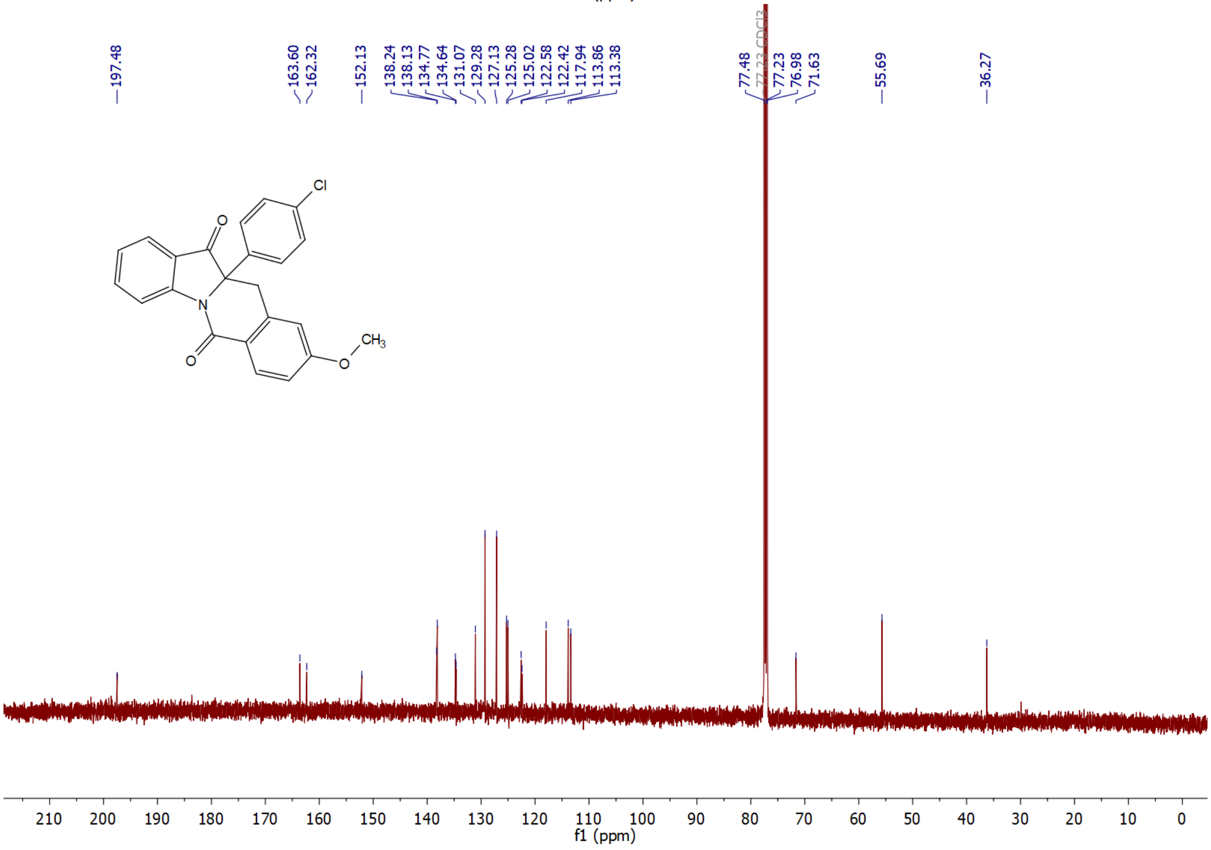

11a-(4-chlorophenyl)-9-fluoro-11,11a-dihydroindolo[1,2-b]isoquinoline-6,12-dione [**G20**]:

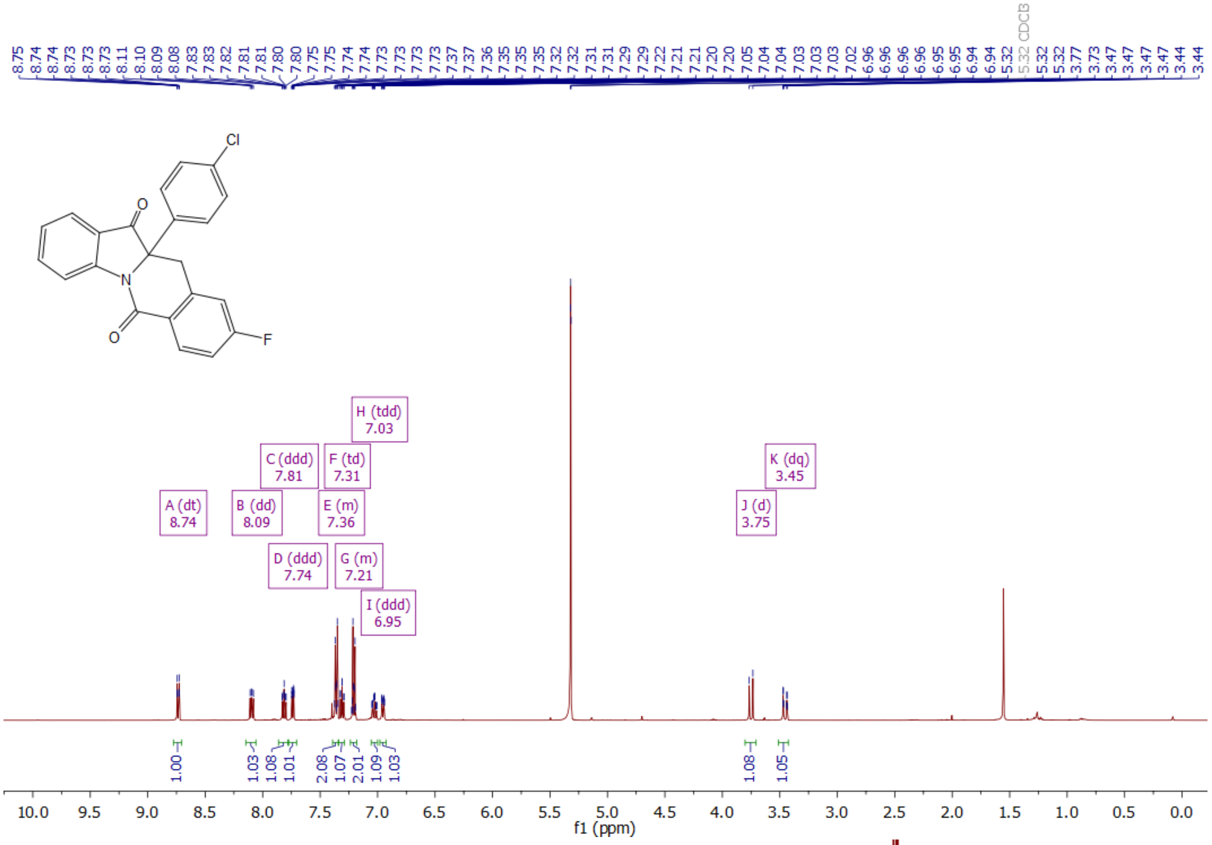

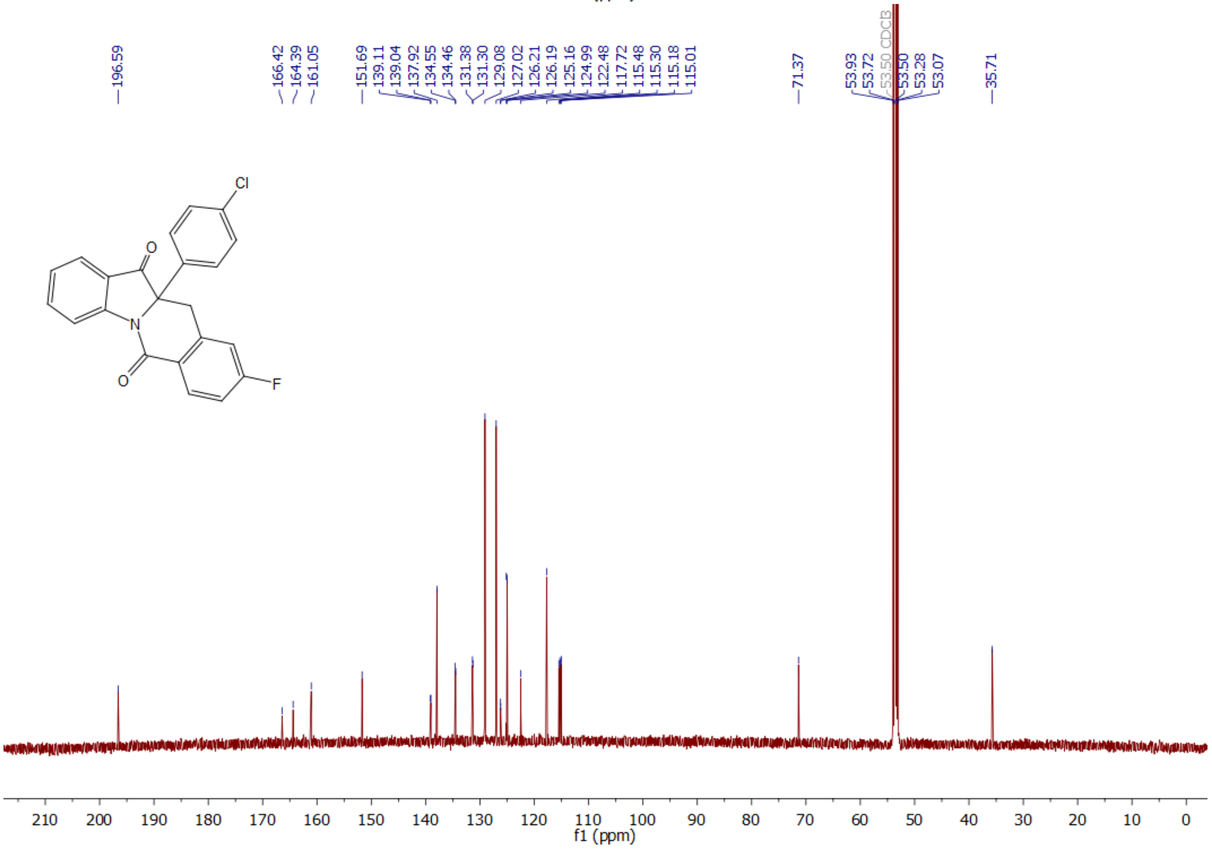

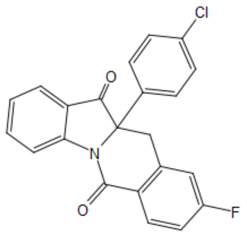

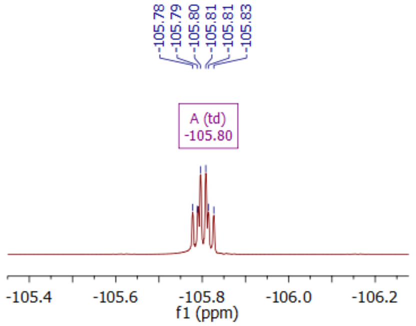

-105.78
-105.79
-105.80
-105.81
-105.81
-105.83
A (td)
-105.80
-105.4
-105.6
-105.8
-106.0
-106.2
f1 (ppm)

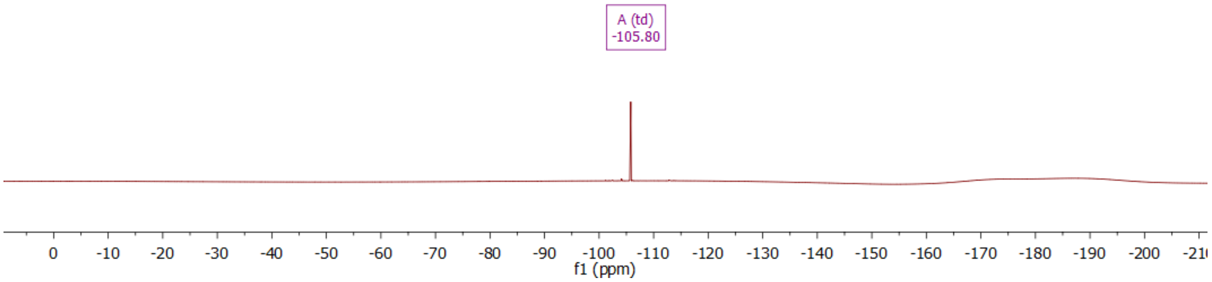

A (td)
-105.80
0
-10
-20
-30
-40
-50
-60
-70
-80
-90
-100
-110
-120
-130
-140
-150
-160
-170
-180
-190
-200
f1 (ppm)

1-(2-bromophenyl)-1,3,4,9-tetrahydropyrano[3,4-b]indole **[5a]:**

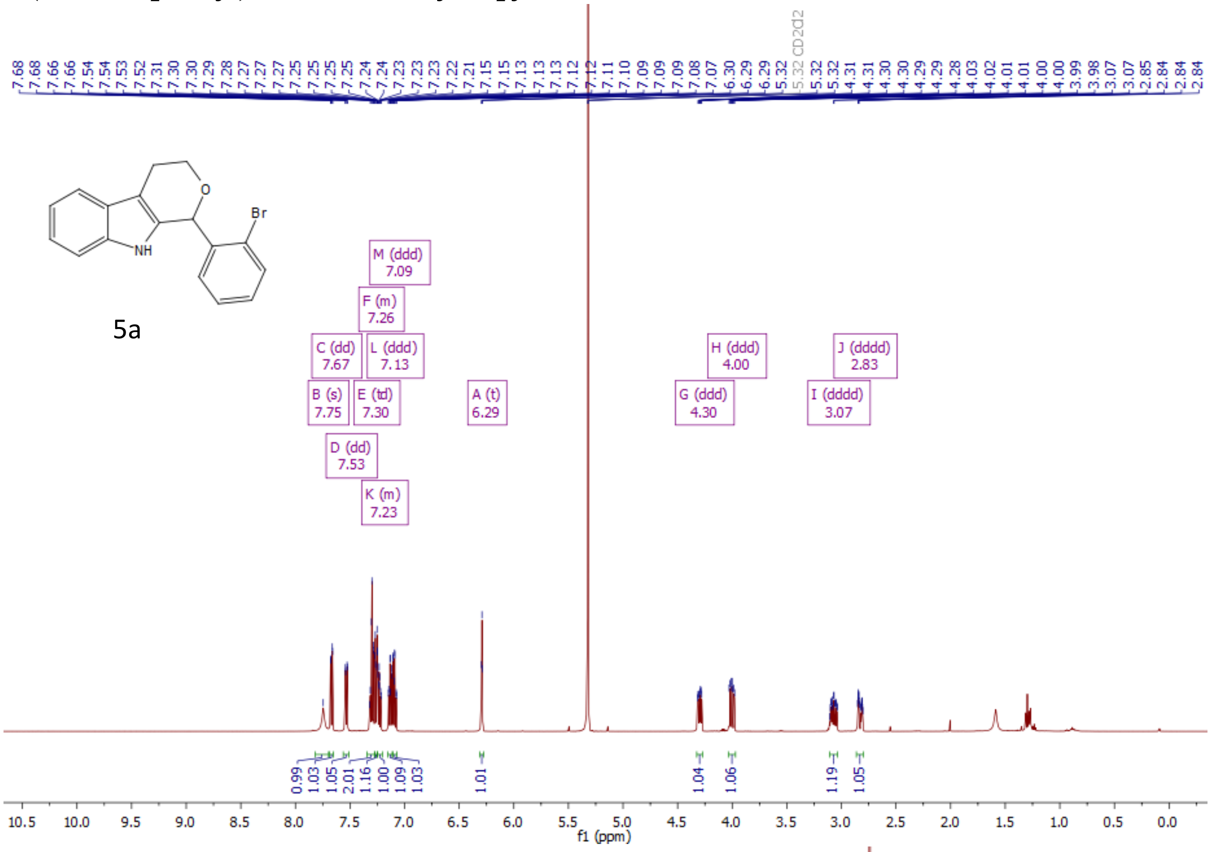

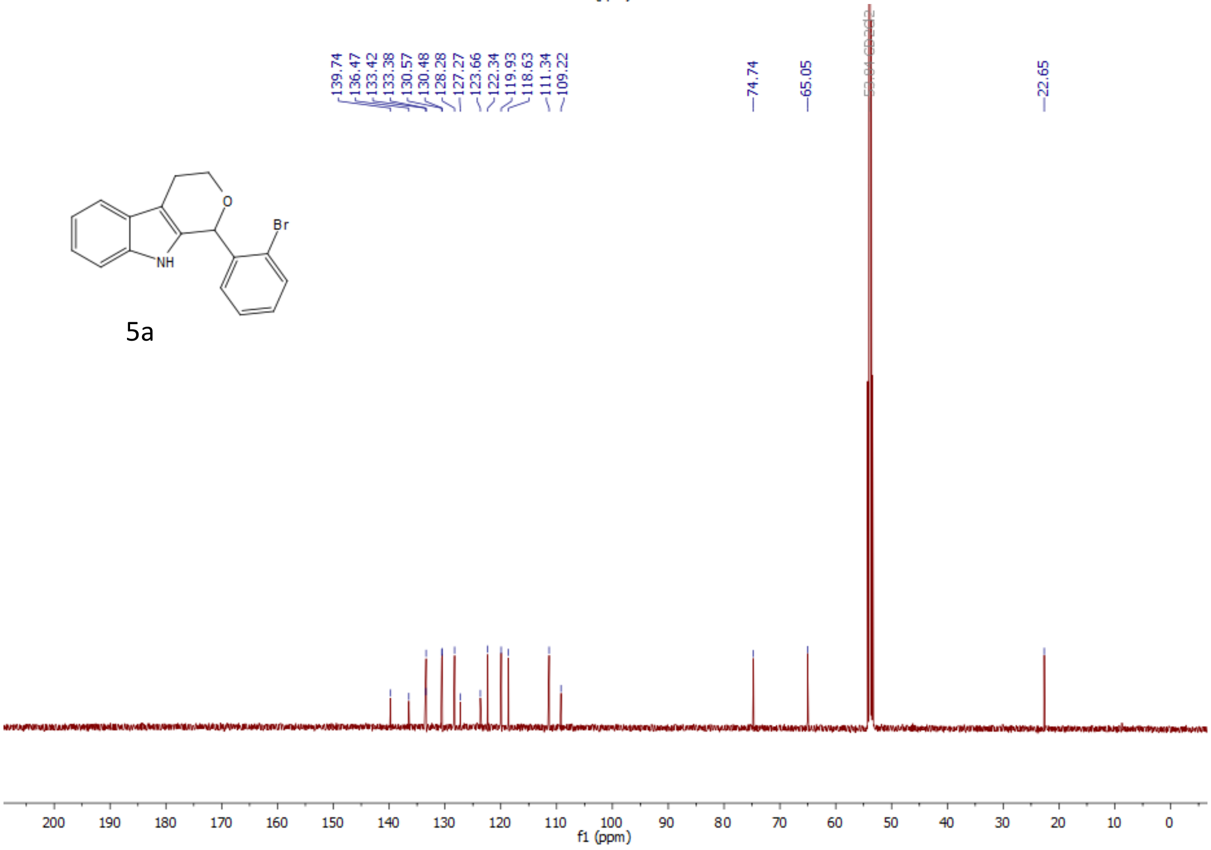

***1-(2-bromo-4-fluorophenyl)-1,3,4,9-tetrahydropyrano[3,4-b]indole* [5b]:**

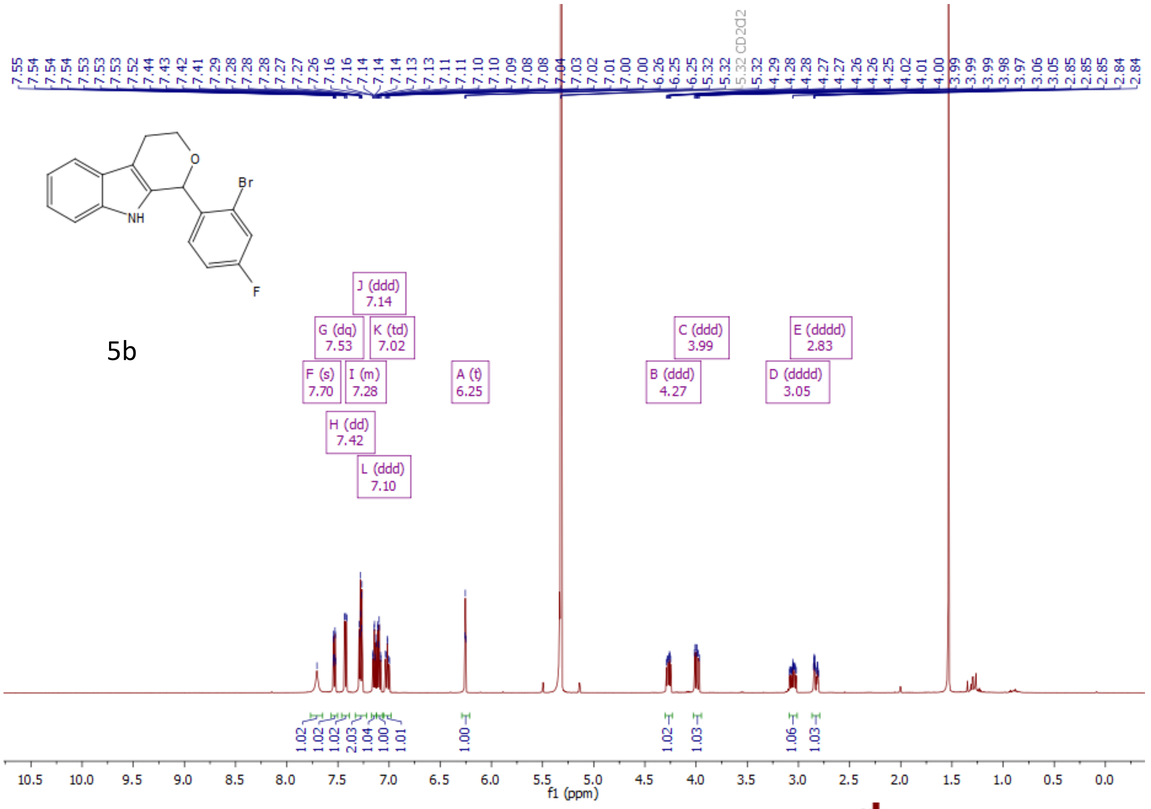

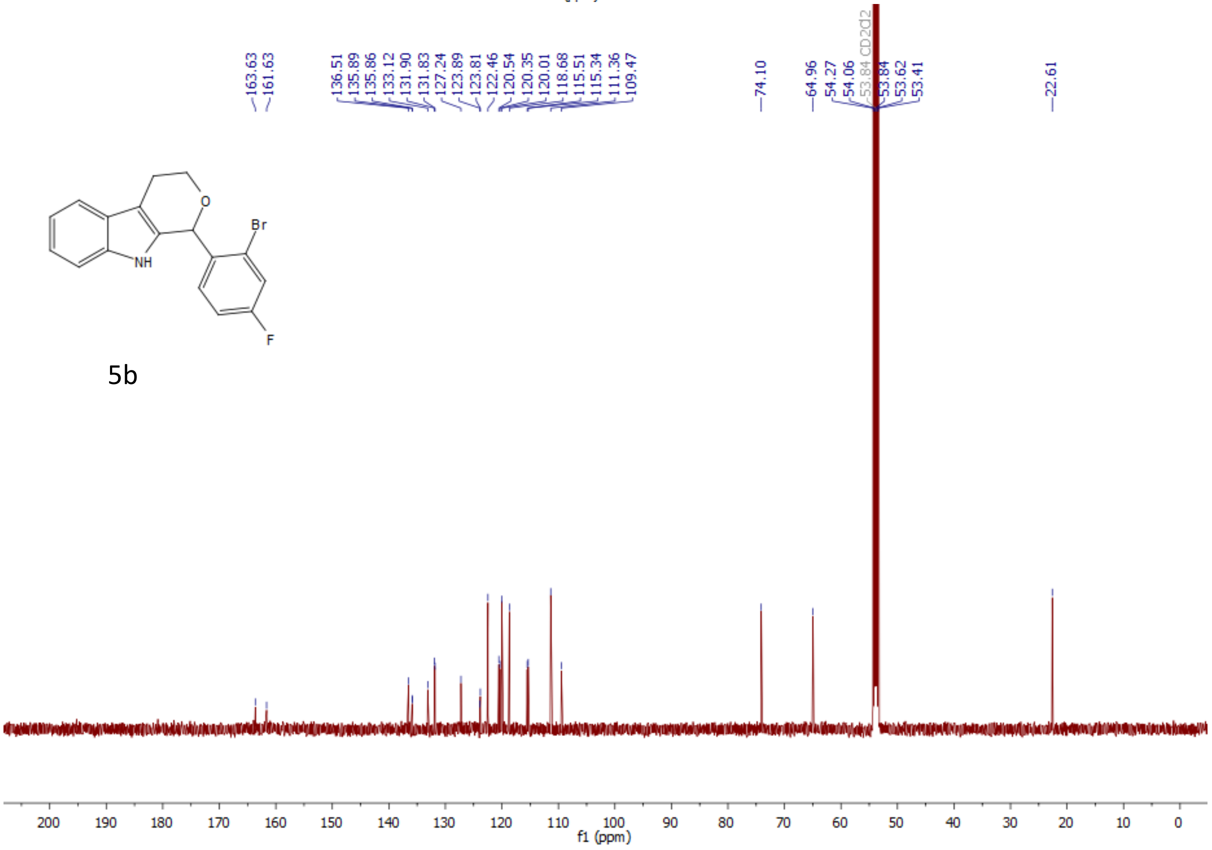

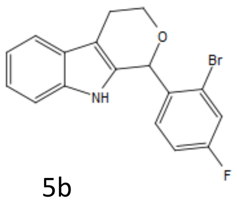
O
Br
NH
F

5b

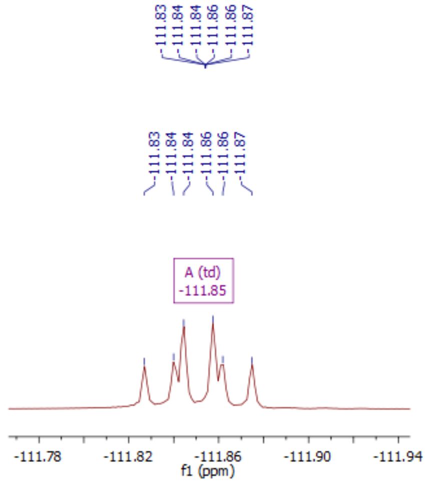
-111.83
-111.84
-111.84
-111.86
-111.86
-111.87
A (td)
-111.85
-111.78
-111.82
-111.86
-111.90
-111.94
f1 (ppm)

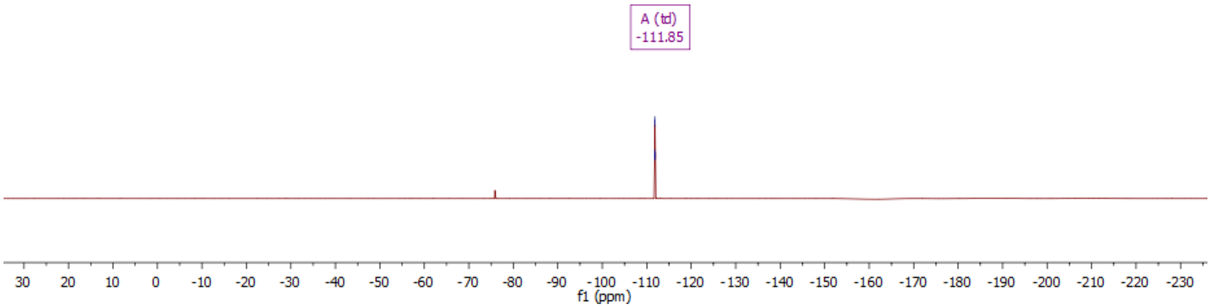
A (td)
-111.85
30 20 10 0 -10 -20 -30 -40 -50 -60 -70 -80 -90 -100 -110 -120 -130 -140 -150 -160 -170 -180 -190 -200 -210 -220 -230
f1 (ppm)

1-(2-bromo-4-methylphenyl)-1,3,4,9-tetrahydropyrano[3,4-b]indole **[5c]:**

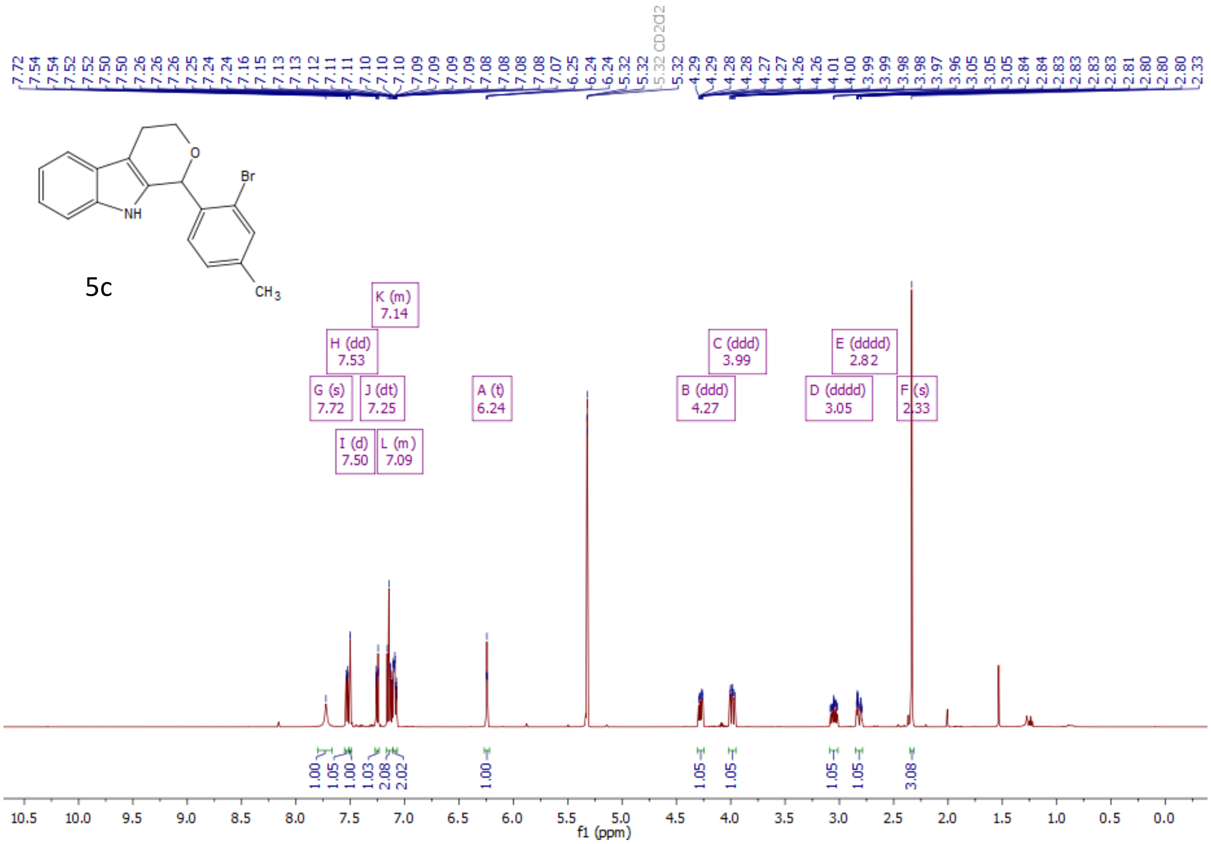

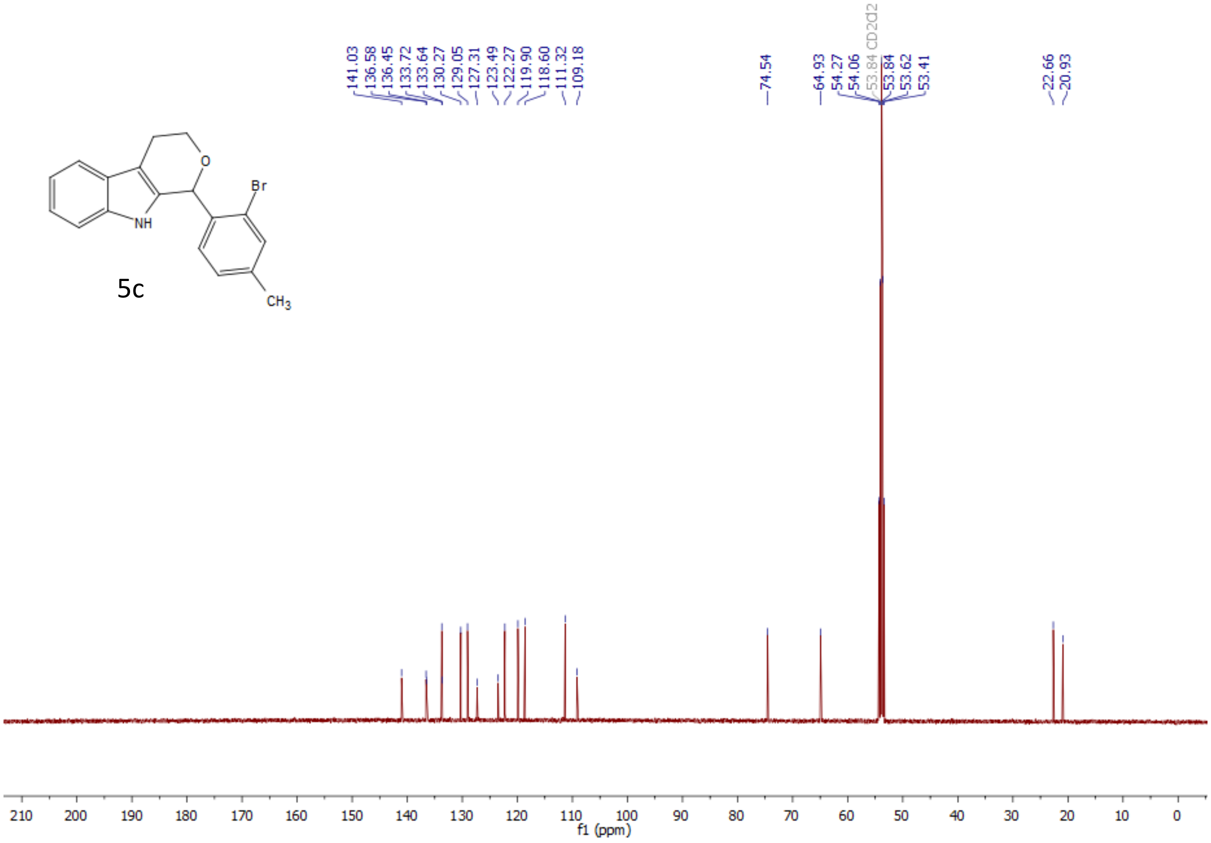

1-(2-bromo-4-methoxyphenyl)-1,3,4,9-tetrahydropyrano[3,4-b]indole [5d]:

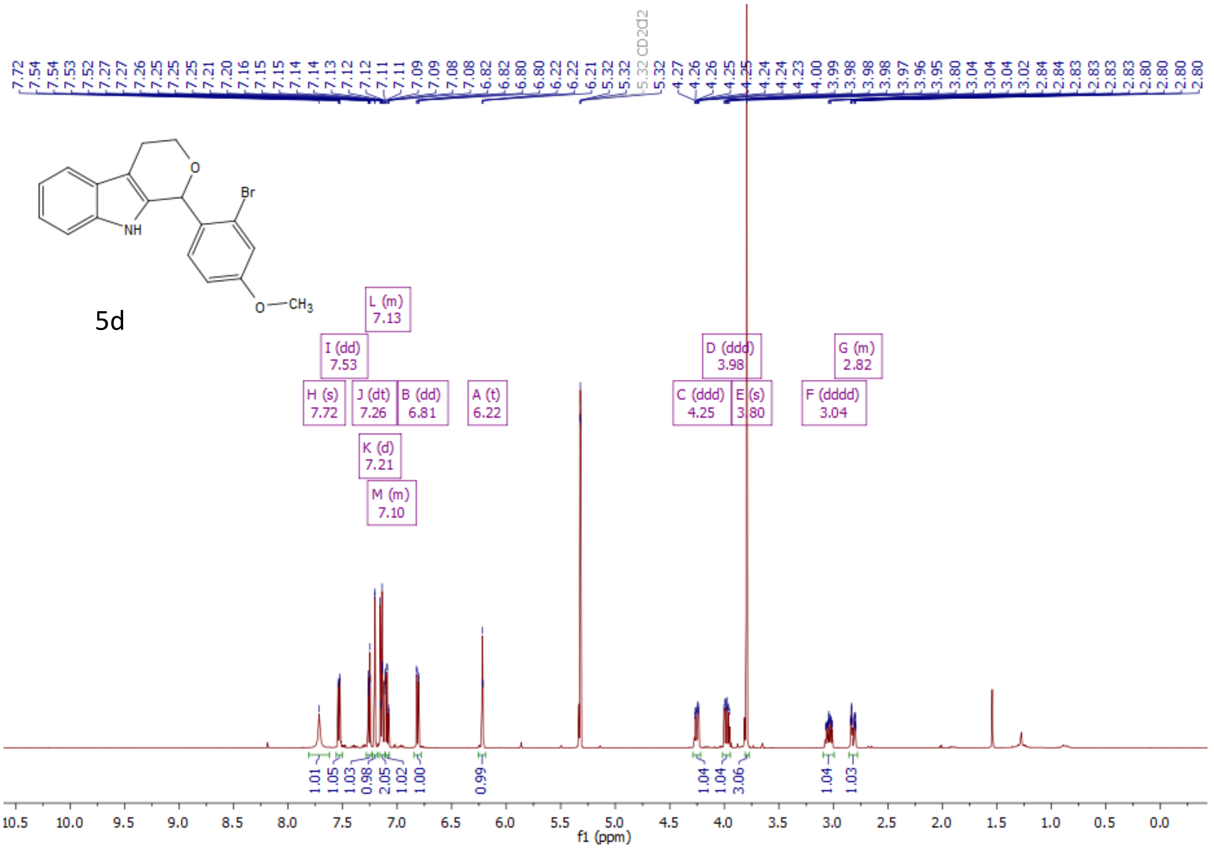

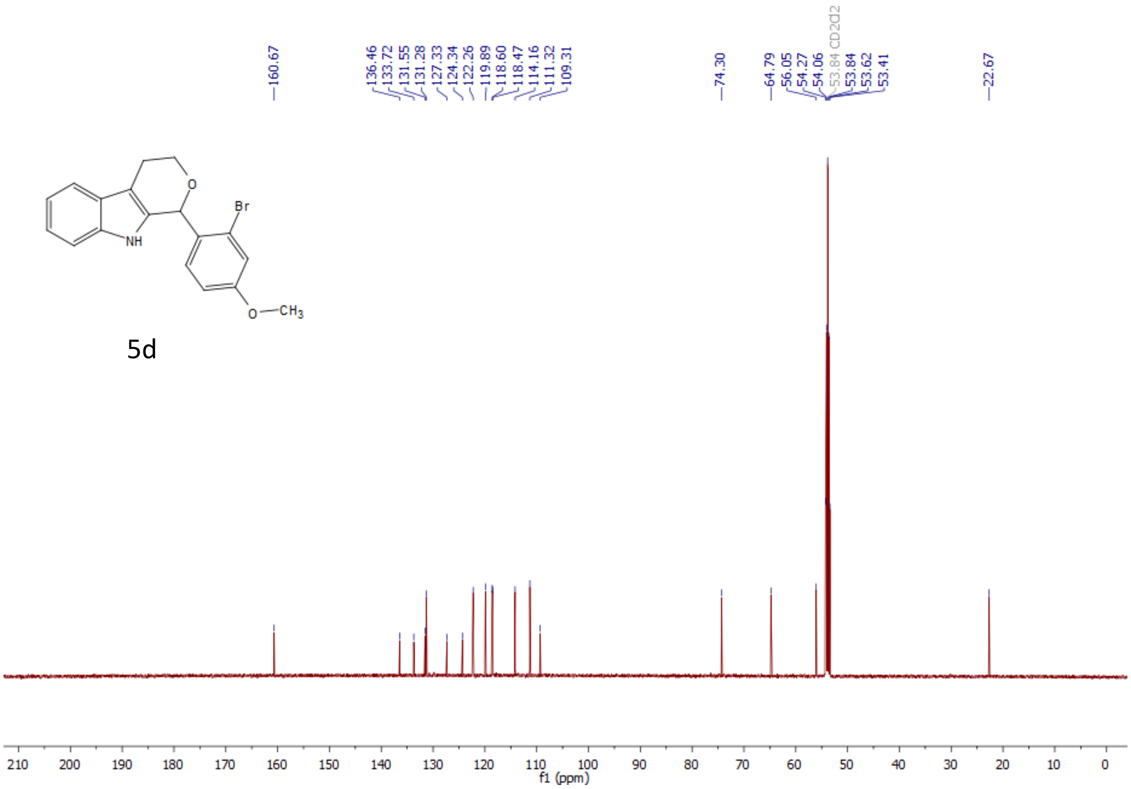

CLASS-H

***1,13b-dihydrodibenzo[b,f]pyrano[2,3,4-hi]indolizin-8(2H)-one* [H1]:**

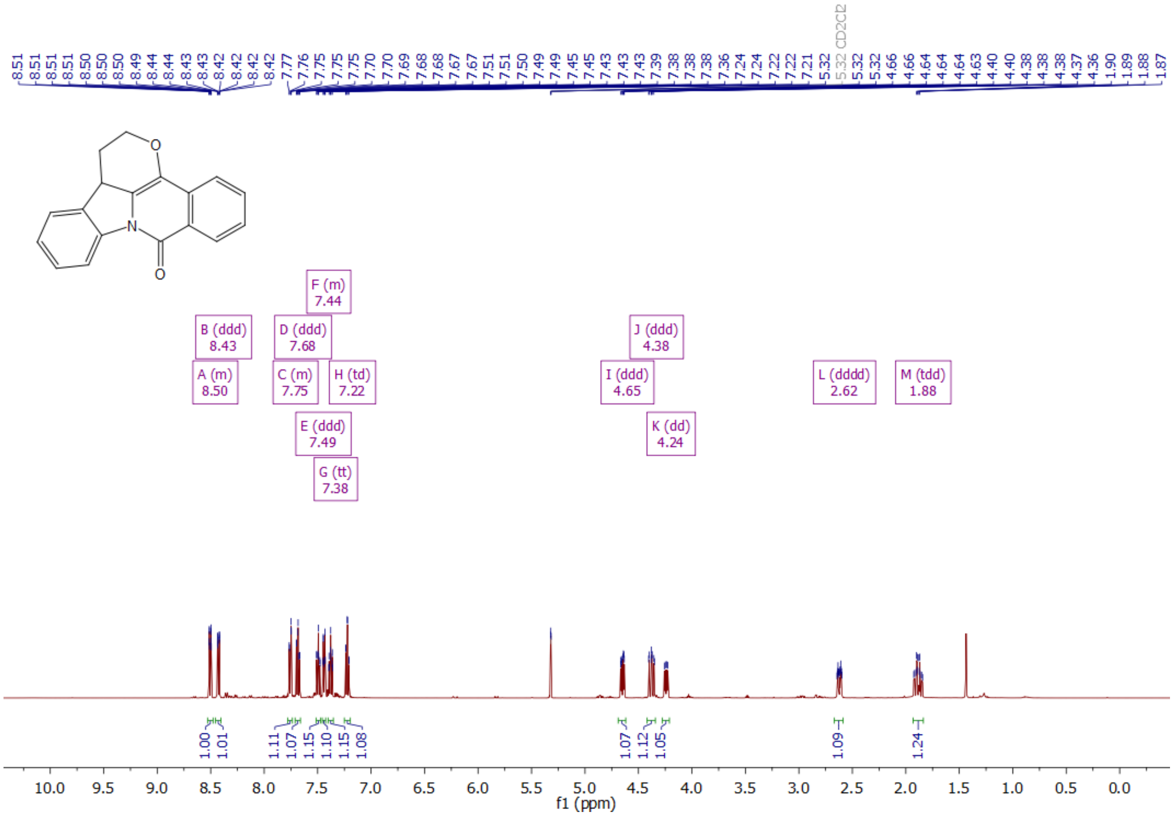

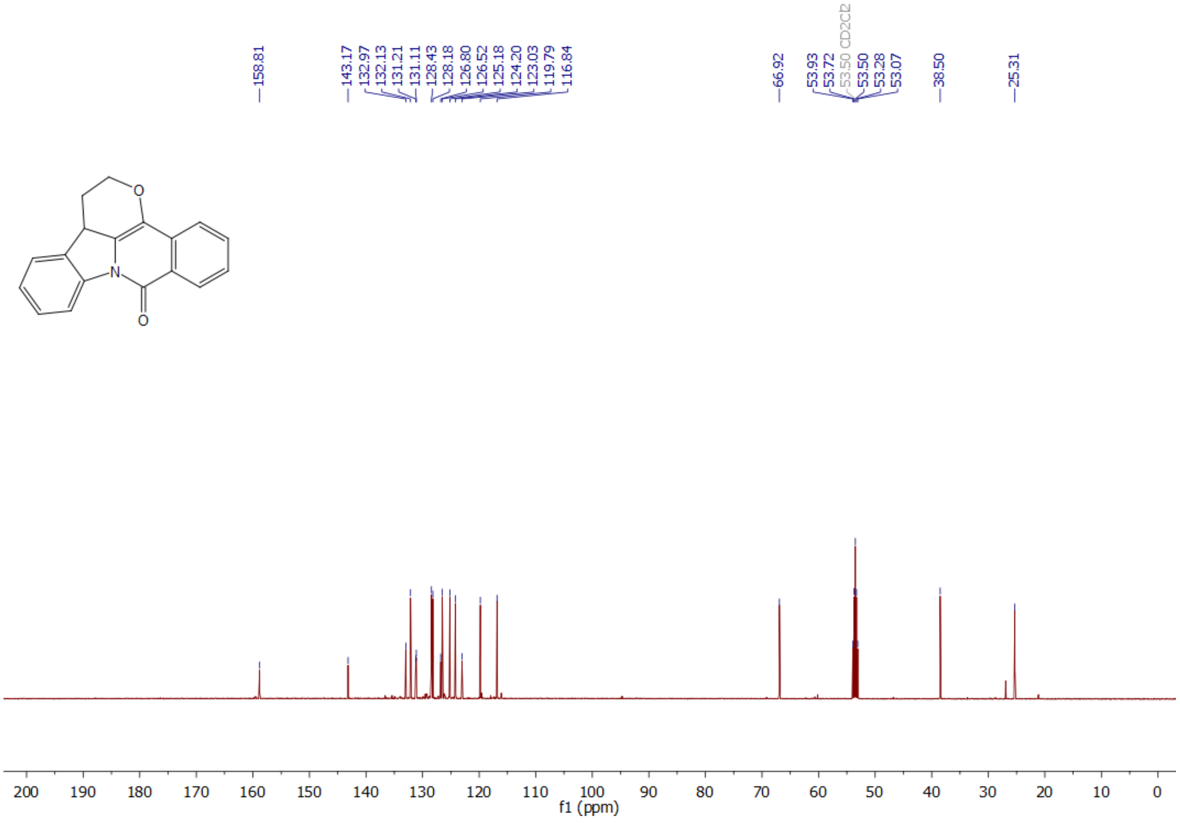

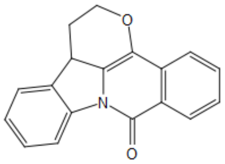
O
N
O

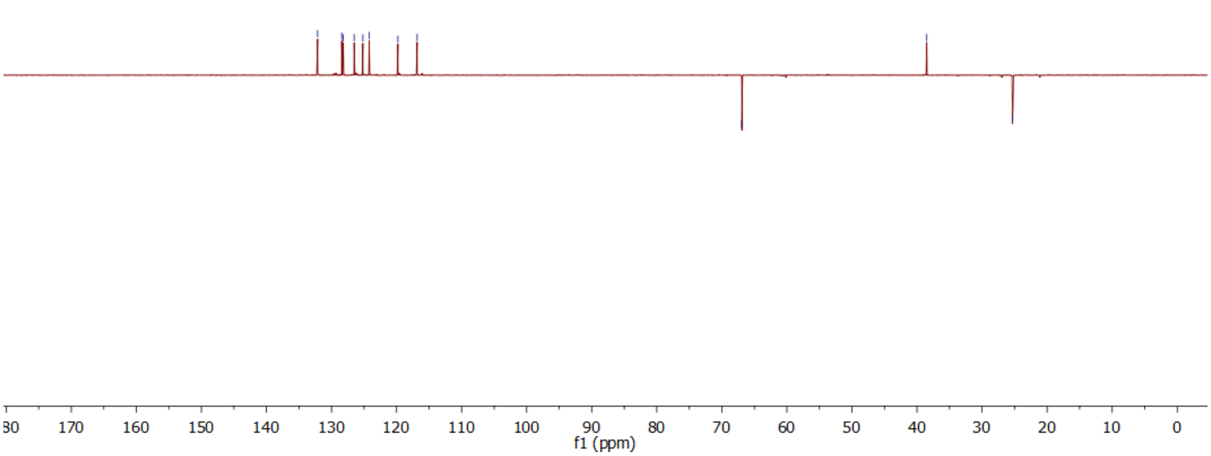
132.13
128.44
128.19
126.52
125.18
124.20
119.79
116.84
66.92
38.50
25.31
80
170
160
150
140
130
120
110
100
90
80
70
60
50
40
30
20
10
0
f1 (ppm)

6-fluoro-1,13b-dihydrodibenzo[b,f]pyrano[2,3,4-hi]indolizin-8(2H)-one [**H2**]:

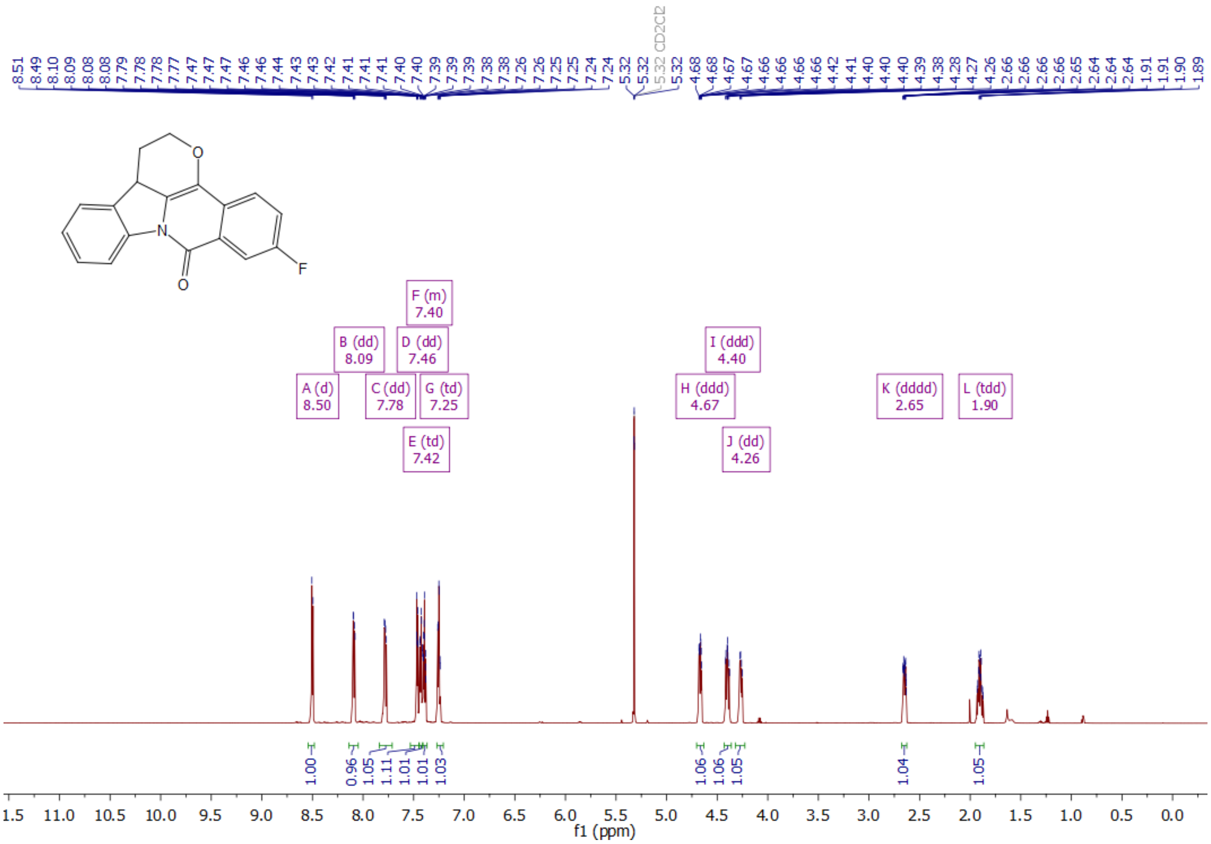

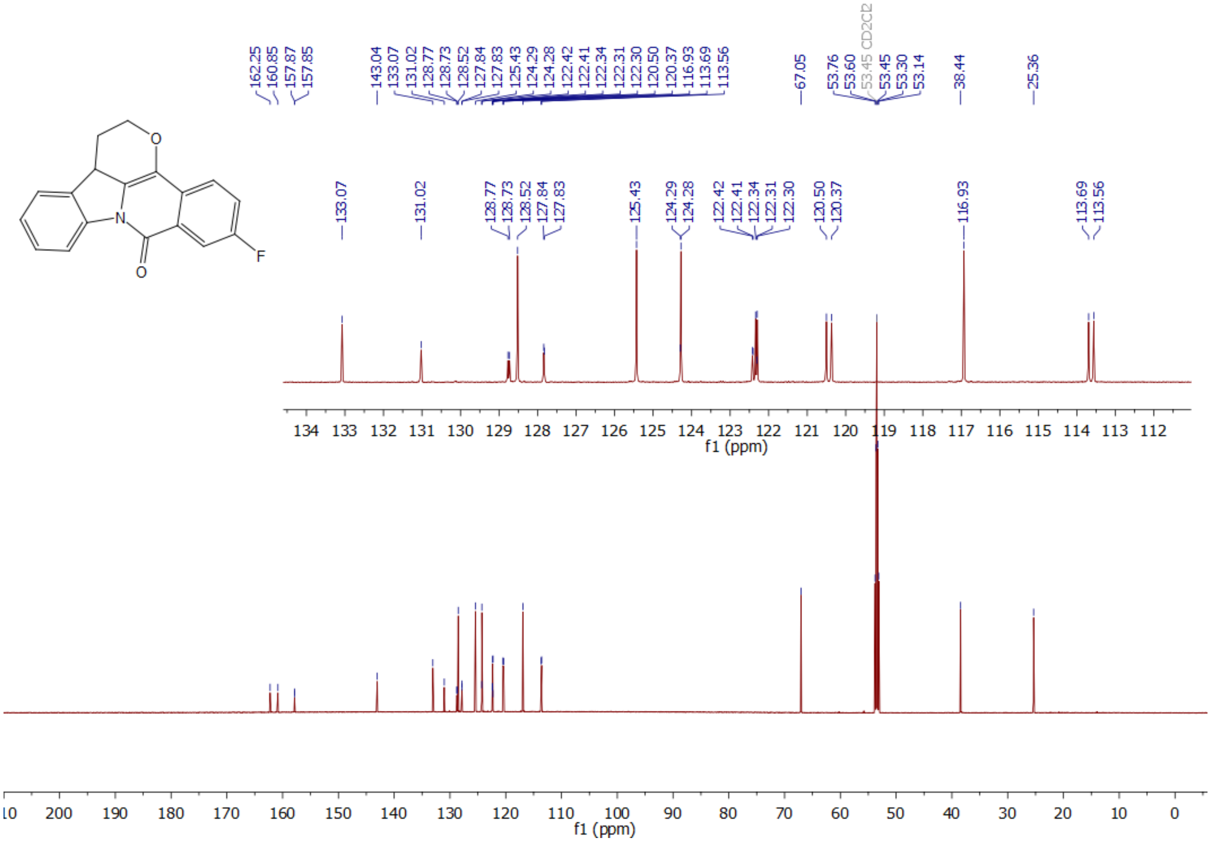

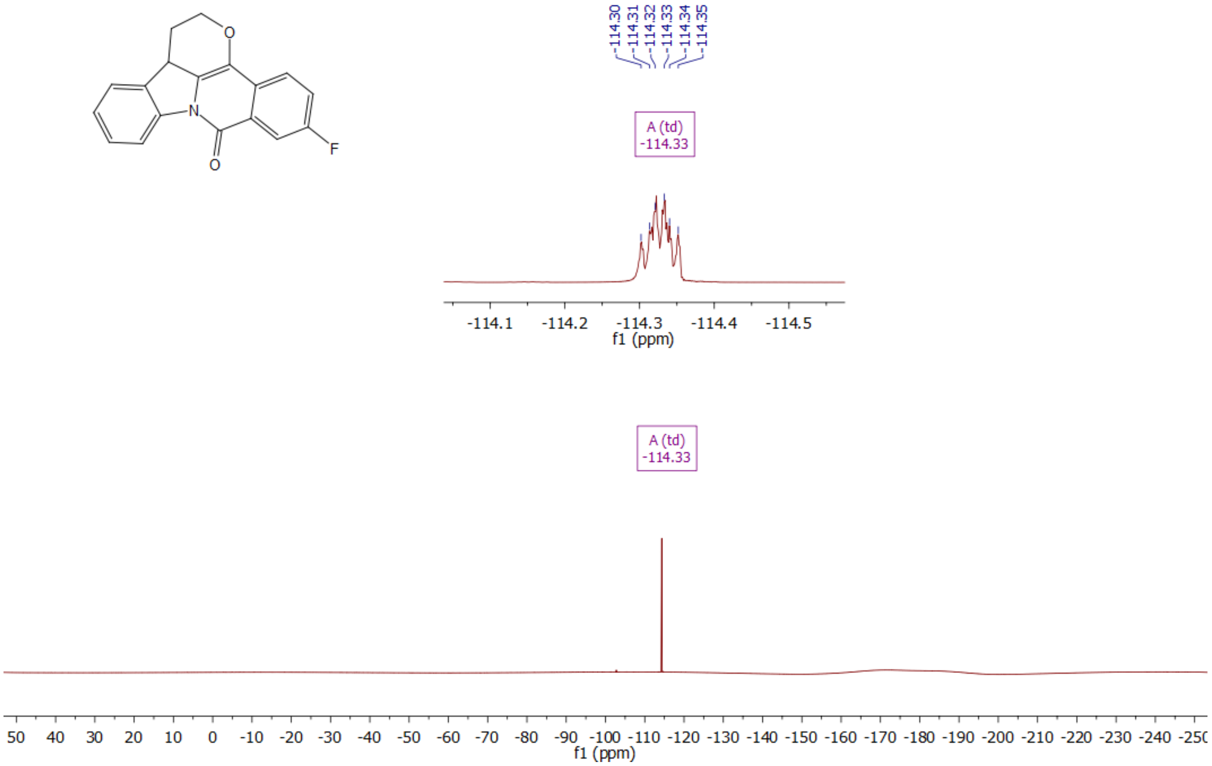
-114.30
-114.31
-114.32
-114.33
-114.34
-114.35
A (td)
-114.33
-114.1 -114.2 -114.3 -114.4 -114.5
f1 (ppm)
A (td)
-114.33
50 40 30 20 10 0 -10 -20 -30 -40 -50 -60 -70 -80 -90 -100 -110 -120 -130 -140 -150 -160 -170 -180 -190 -200 -210 -220 -230 -240 -250
f1 (ppm)

6-methyl-1,13b-dihydrodibenzo[b,f]pyrano[2,3,4-hi]indolizin-8(2H)-one [H3]:

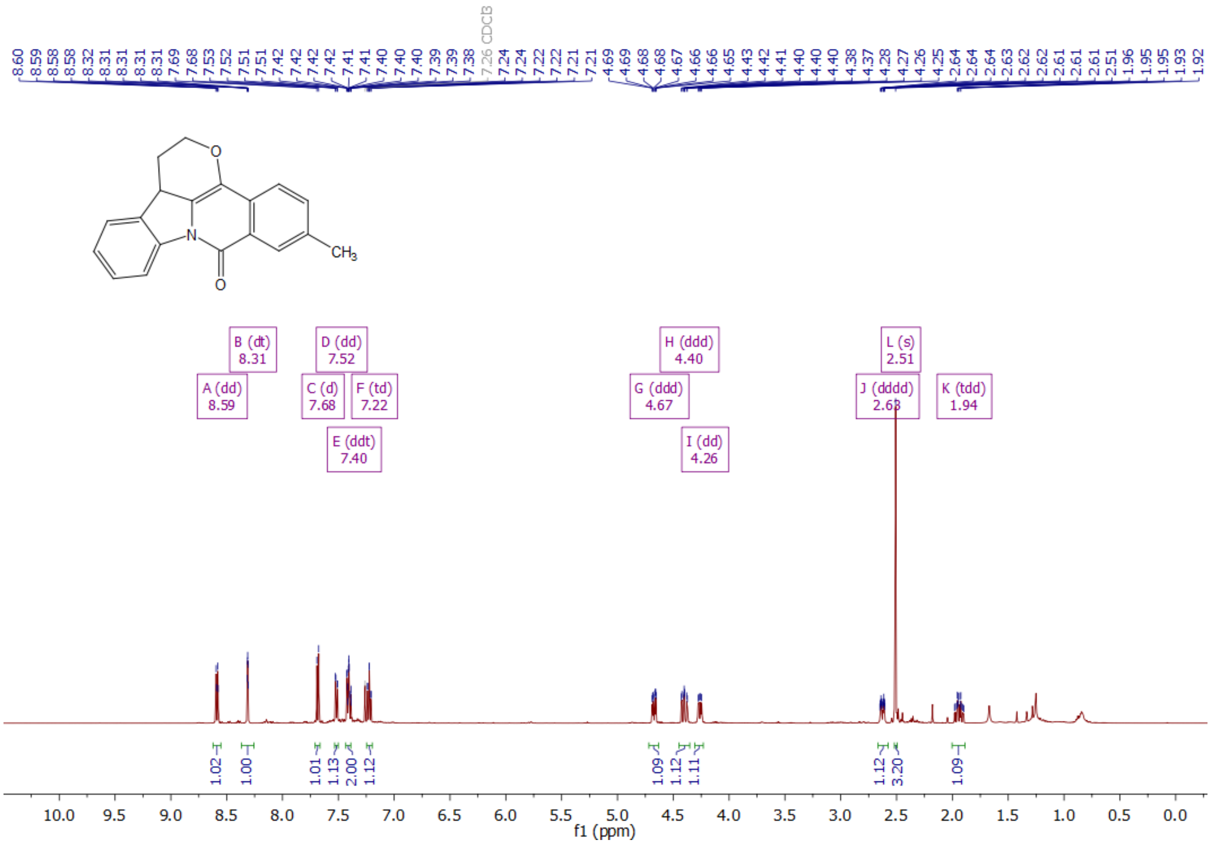

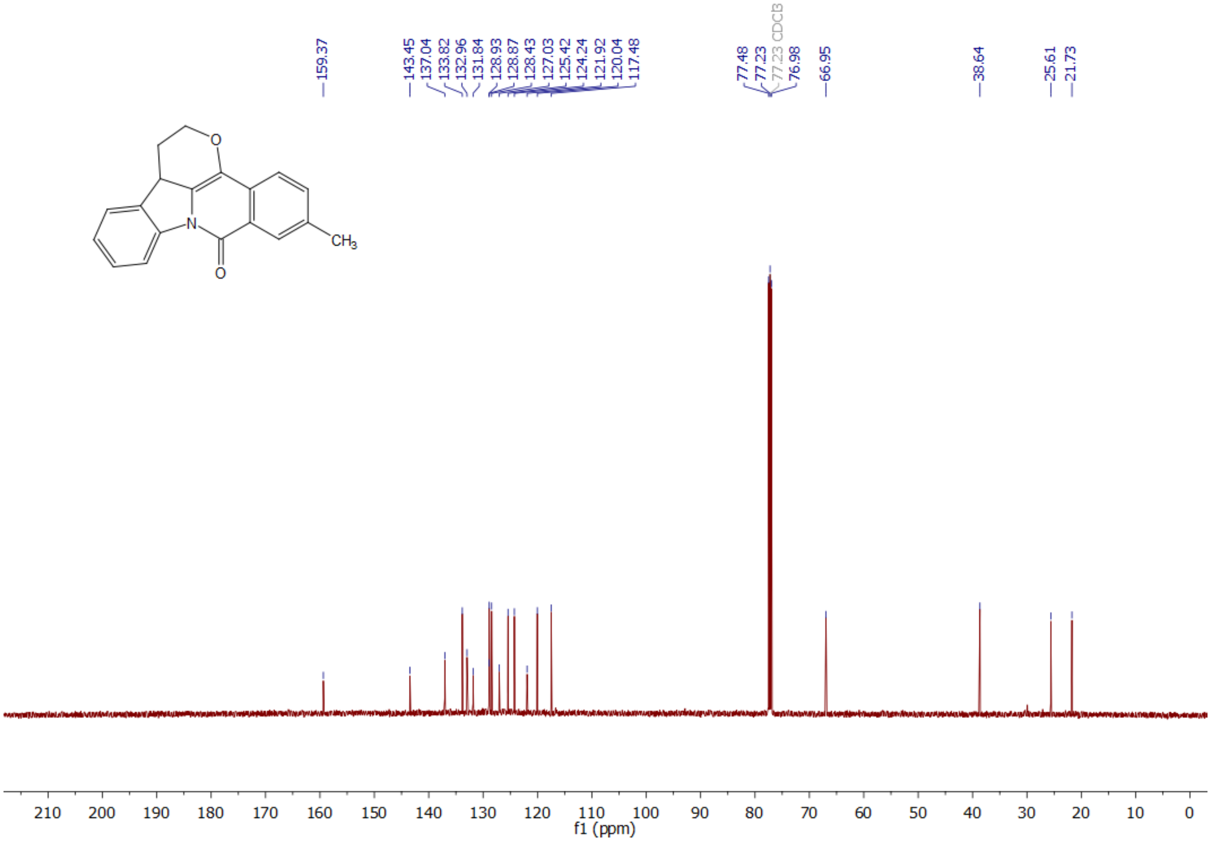

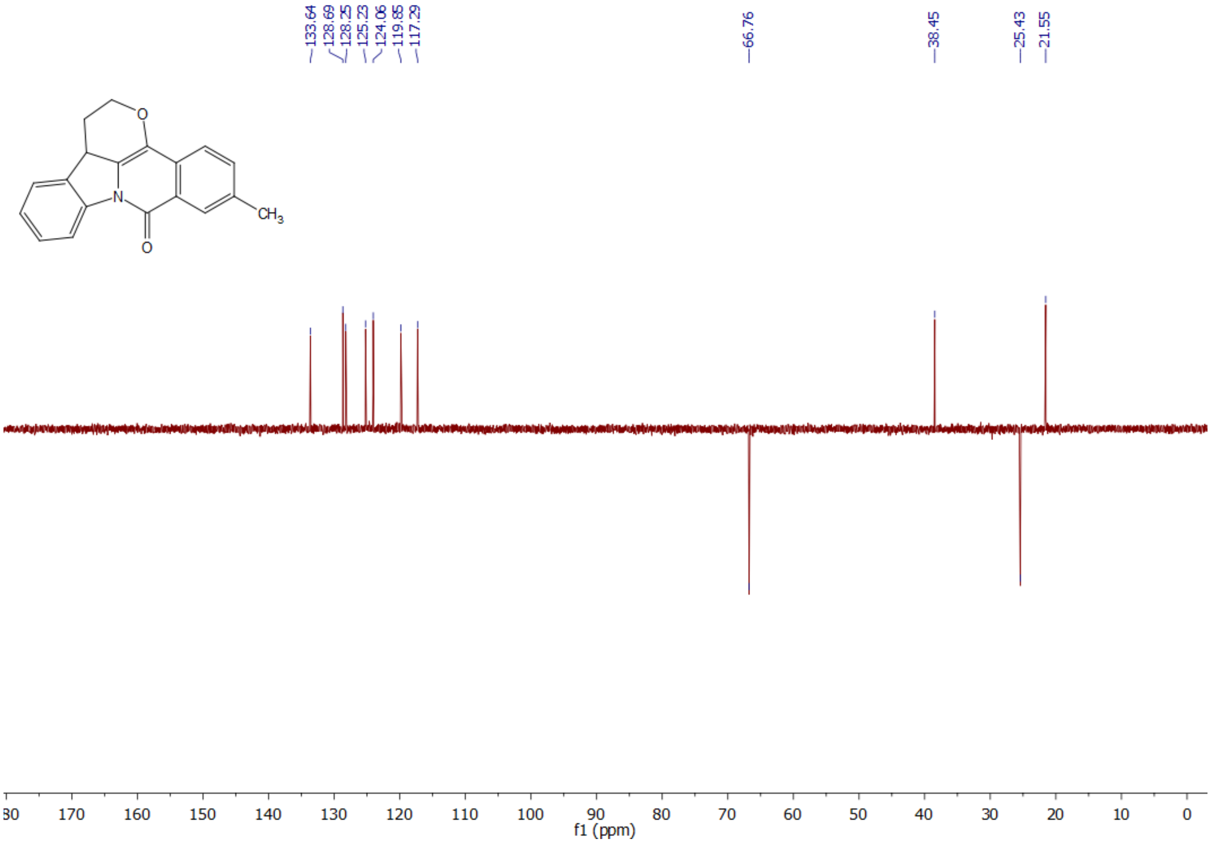
133.64
128.69
128.25
125.23
124.06
119.85
117.29
66.76
38.45
25.43
21.55
O
N
O
CH3
f1 (ppm)

***6-methoxy-1,13b-dihydrodibenzo[b,f]pyrano[2,3,4-hi]indolizin-8(2H)-one* [H4]:**

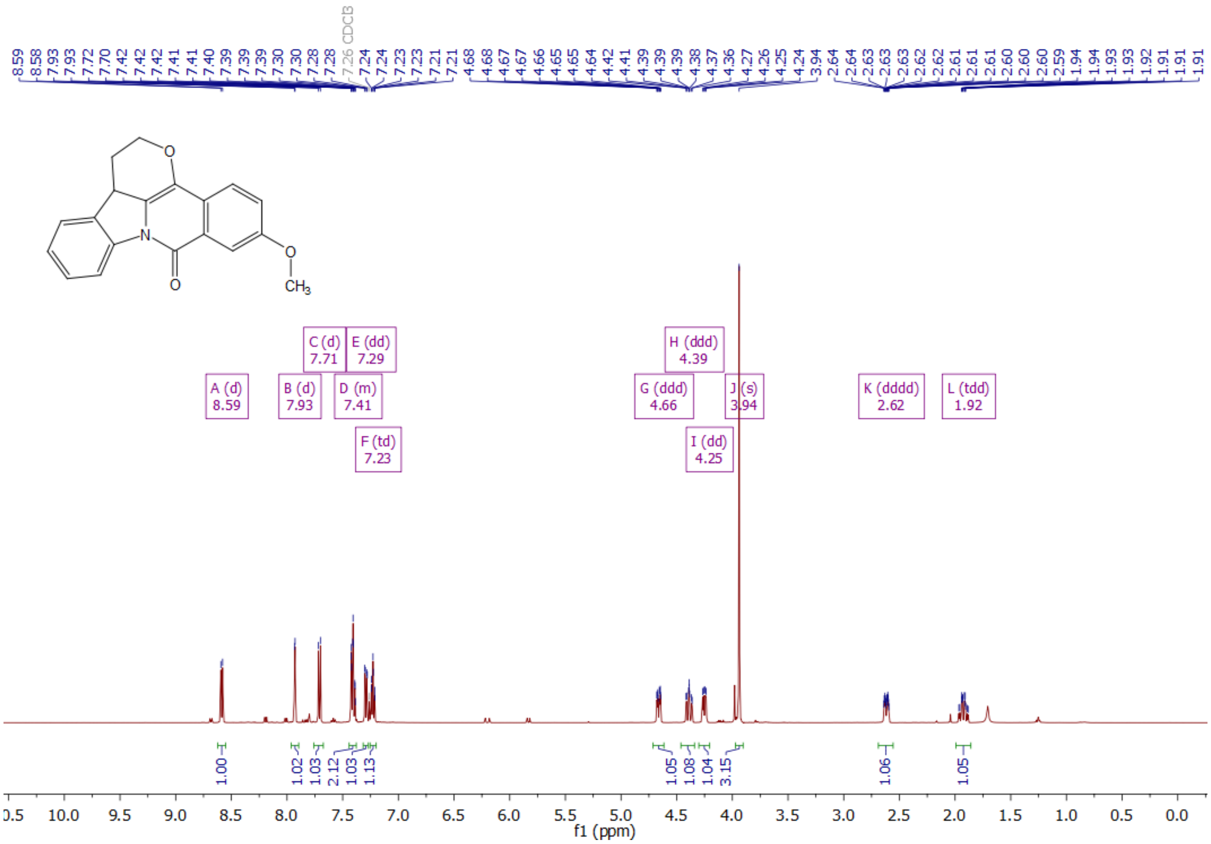

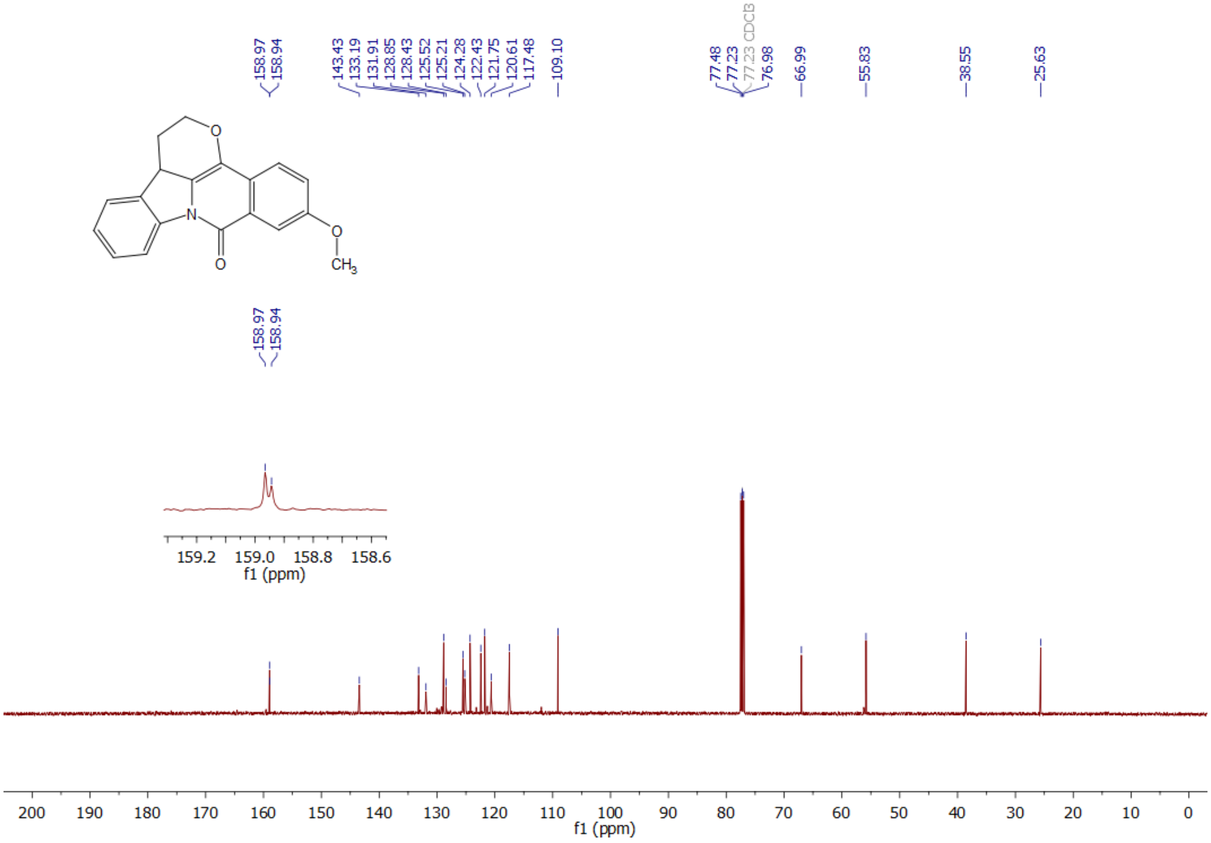

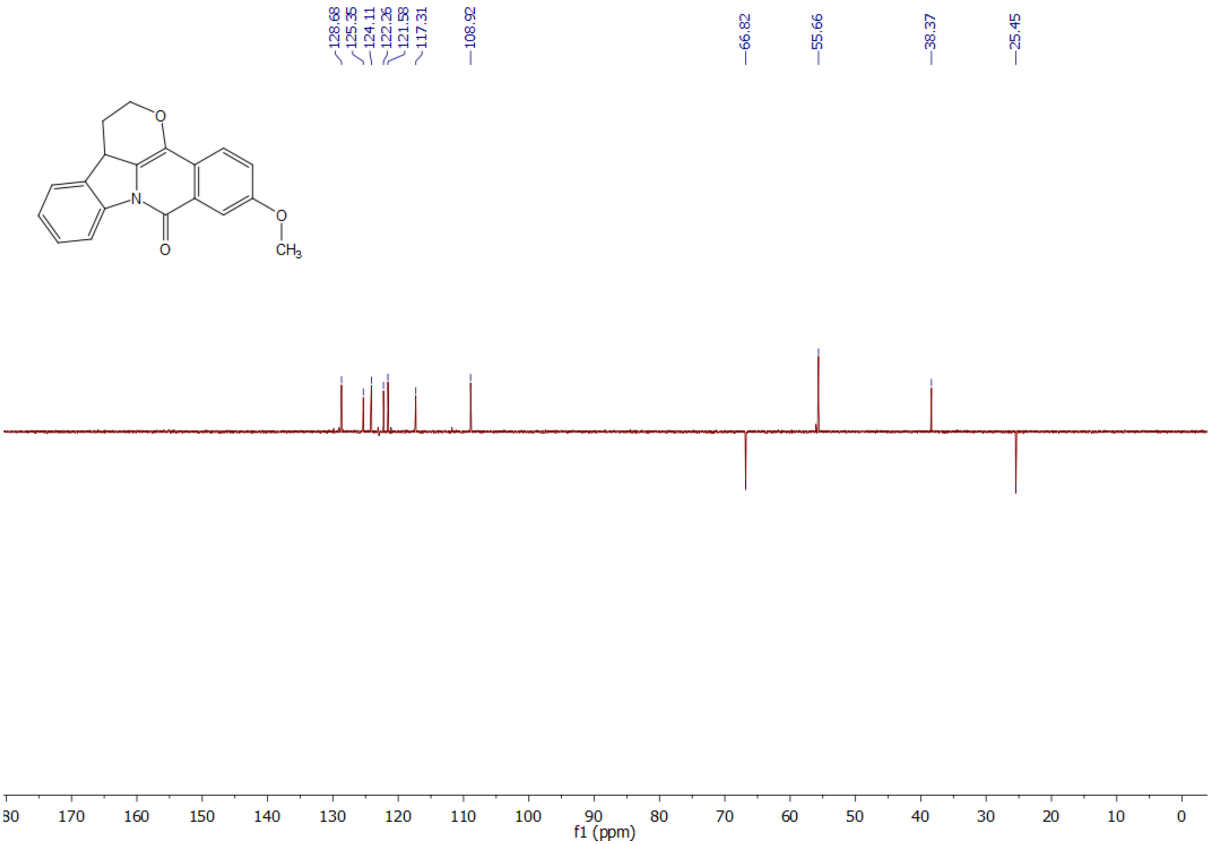
128.68
125.35
124.11
122.26
121.58
117.31
108.92
66.82
55.66
38.37
25.45
O
N
O
O
CH3
170
160
150
140
130
120
110
100
90
80
70
60
50
40
30
20
10
0
f1 (ppm)
