# Supplementary material for: A divergent intermediate strategy yields biologically diverse pseudo-natural products
Source: Nat Chem. 2024 Feb 16;16(6):945–58. doi: 10.1038/s41557-024-01458-4 (PMC11164679; doi:10.1038/s41557-024-01458-4)
Supplement: Supplementary file 2 — Reporting Summary [file 41557_2024_1458_MOESM2_ESM.pdf]

nature portfolio

Corresponding author(s): Herbert Waldmann

Last updated by author(s): Nov 18, 2023

Reporting Summary

Nature Portfolio wishes to improve the reproducibility of the work that we publish. This form provides structure for consistency and transparency in reporting. For further information on Nature Portfolio policies, see our Editorial Policies and the Editorial Policy Checklist.

Statistics

For all statistical analyses, confirm that the following items are present in the figure legend, table legend, main text, or Methods section.

n/a	Confirmed	
☐	☒	The exact sample size (*n*) for each experimental group/condition, given as a discrete number and unit of measurement
☒	☐	A statement on whether measurements were taken from distinct samples or whether the same sample was measured repeatedly
☐	☒	The statistical test(s) used AND whether they are one- or two-sided<br>*Only common tests should be described solely by name; describe more complex techniques in the Methods section.*
☒	☐	A description of all covariates tested
☒	☐	A description of any assumptions or corrections, such as tests of normality and adjustment for multiple comparisons
☐	☒	A full description of the statistical parameters including central tendency (e.g. means) or other basic estimates (e.g. regression coefficient) AND variation (e.g. standard deviation) or associated estimates of uncertainty (e.g. confidence intervals)
☐	☒	For null hypothesis testing, the test statistic (e.g. *F*, *t*, *r*) with confidence intervals, effect sizes, degrees of freedom and *P* value noted<br>*Give P values as exact values whenever suitable.*
☒	☐	For Bayesian analysis, information on the choice of priors and Markov chain Monte Carlo settings
☒	☐	For hierarchical and complex designs, identification of the appropriate level for tests and full reporting of outcomes
☒	☐	Estimates of effect sizes (e.g. Cohen's *d*, Pearson's *r*), indicating how they were calculated

Our web collection on statistics for biologists contains articles on many of the points above.

Software and code

Policy information about availability of computer code

Data collection	All code used in this manuscript has been made available on Github under the MIT license: https://github.com/mpimp-comas/2023_bag_bio_diverse_pnps.<br>Cell Painting Assay microscopy: Micro XL High-Content Screening System (Molecular Devices) in 5 channels (DAPI: Ex350-400/ Em410-480; FITC: Ex470-500/ Em510-540; Spectrum Gold: Ex520-545/ Em560-585; TxRed: Ex535-585/ Em600-650; Cy5: Ex605-650/ Em670-715)<br>Osteoblast differentiation assay: Spark® plate reader (Tecan)<br>RT-qPCR: CFX96 Real-Time PCR Detection System (Bio-Rad, Germany)<br>SMO binding assay: Zeiss Observer Z1 microscope (Carl Zeiss, Germany) was used to acquire the images using a Plan-Apochromat 63x/1.40 Oil DIC M27 objective<br>Immunocytochemistry: Observer Z1 (Carl Zeiss, Germany) using 63X objectives (LD Plan-Neofluar); Axiovert 200M microscope (Carl Zeiss, Germany) equipped with 10X objective was used to detect phospho-histone H3-positive cells and could be quantified by using MetaMorph 7.<br>In Vitro Tubulin Polymerization Assay: Infinite M200 plate reader (Tecan)<br>Flow Cytometry: BD LSRII analyzer (Becton Dickinson, USA)<br>Uridine Rescue Assay: IncuCyte Zoom (Essen BioScience)<br>X-ray analysis: Bruker D8 Venture fourcircle diffractometer by Bruker AXS GmbH using a PHOTON II CPAD detector by Bruker AXS GmbH. X-ray radiation was generated by microfocus sources IµS 3.0 Mo by Incoatec GmbH with HELIOS mirror optics and a single-hole collimator by Bruker AXS GmbH.<br>HRMS: LTQ Orbitrap mass spectrometer coupled to an Accela HPLC-System (HPLC column: Hypersyl GOLD, 50 mm x 1 mm, particle size 1.9 µm, ionization method: electron spray ionization)<br>NMR: Bruker DRX400 (400 MHz), Bruker DRX500 (500 MHz), INOVA500 (500 MHz) or Bruker DRX700
Data analysis	All code used in this manuscript has been made available on Github under the MIT license: https://github.com/mpimp-

March 2021

Data analysis

comas/2023_bag_bio_diverse_pnps
Cell Painting Assay data were analyzed using CellProfiler (version 3.0.0), custom Python (https://www.python.org/) scripts using the Pandas (https://pandas.pydata.org/) and Dask (https://dask.org/) data processing libraries as well as the Scientific Python (https://scipy.org/).
ODA IC50 calculations: GraphPad Prism 9 (GraphPad Software, USA)
RT-qPCR: 2-ΔΔCt method (9. Pfaffl, M. W. A new mathematical model for relative quantification in real-time RT-PCR. Nucleic Acids Res. 29, e45 (2001).)
Immunocytochemistry: CellProfiler (version 3.0.0)
Flow Cytometry: FlowJo 10.7.2
Uridine Rescue Assay: IncuCyte Zoom software (Essen BioScience)
Statistical analysis: GraphPad Prism 9.2.0 software
X-ray analysis: APEX 3 Suite (v.2018.7-2) with the integrated programs SAINT (integration) and SADABS (adsorption correction) by Bruker AXS GmbH
NMR: Mestranova x64

For manuscripts utilizing custom algorithms or software that are central to the research but not yet described in published literature, software must be made available to editors and reviewers. We strongly encourage code deposition in a community repository (e.g. GitHub). See the Nature Portfolio guidelines for submitting code & software for further information.

Data

Policy information about availability of data

All manuscripts must include a data availability statement. This statement should provide the following information, where applicable:

- Accession codes, unique identifiers, or web links for publicly available datasets
- A description of any restrictions on data availability
- For clinical datasets or third party data, please ensure that the statement adheres to our policy

The reference data sets used on the manuscript (Enamine Advanced Screening Collection, https://enamine.net/hit-finding/compound-collections/screening-collection/advanced-collection downloaded on 07-Dec-2020.; Drugbank approved and investigational drugs, v. 5.1.8; ChEMBL v30) are not included, but the steps to generate them are described in the code repository https://zenodo.org/records/8320827. All structures covered in this manuscript and their calculated properties are included in the Github repository https://github.com/mpimp-comas/2023_bag_bio_diverse_pnps. Crystallographic data for compound B10 has been deposited with the Cambridge Crystallographic Data Centre, with deposition number CCDC 2221540.

Human research participants

Policy information about studies involving human research participants and Sex and Gender in Research.

Reporting on sex and gender	N/A
Population characteristics	N/A
Recruitment	N/A
Ethics oversight	N/A

Note that full information on the approval of the study protocol must also be provided in the manuscript.

Field-specific reporting

Please select the one below that is the best fit for your research. If you are not sure, read the appropriate sections before making your selection.

☒ Life sciences ☐ Behavioural & social sciences ☐ Ecological, evolutionary & environmental sciences

For a reference copy of the document with all sections, see nature.com/documents/nr-reporting-summary-flat.pdf

Life sciences study design

All studies must disclose on these points even when the disclosure is negative.

Sample size	A sample size of typically 3 independent experiments was chosen in line with the standard of the field in the molecular biosciences. For non-quantitative experiments, the sample size is indicated in each figure caption.
Data exclusions	No data was excluded.
Replication	Three or more biological replicates were used for Hedgehog-dependent osteoblast differentiation assay, RT-qPCR (Alpl, Ptch1, and Gli1), smoothened binding assay, Immunocytochemistry, tubulin polymerization assay, flow cytometry, and uridine rescue assay. Three technical replicates were used for the cell painting assay. The obtained results were consistent bewteen the independent replicates.

Randomization	Randomization was not applicable as no experiments involving human/animals were performed, and no included experiment were sensitive to the order of measurement/treatment.
Blinding	Blinding was not carried out as no subjective analysis was performed.

Reporting for specific materials, systems and methods

We require information from authors about some types of materials, experimental systems and methods used in many studies. Here, indicate whether each material, system or method listed is relevant to your study. If you are not sure if a list item applies to your research, read the appropriate section before selecting a response.

Materials & experimental systems

n/a	Involved in the study
☐	☒ Antibodies
☐	☒ Eukaryotic cell lines
☒	☐ Palaeontology and archaeology
☒	☐ Animals and other organisms
☒	☐ Clinical data
☒	☐ Dual use research of concern

Methods

n/a	Involved in the study
☒	☐ ChIP-seq
☐	☒ Flow cytometry
☒	☐ MRI-based neuroimaging

Antibodies

Antibodies used	anti-tubulin-FITC antibody (clone TU-01, Thermo Fisher, MA119581) and anti-phospho-histone H3 antibody (Cell Signalling, #8481)
Validation	The antibodies were validated using the control condition for the respective method: the anti-tubulin FITC antibody was validated by the detection of mitotic spindles in the DMSO control(Figure 7c). Nocodazole and Colchicine treatment of cells validated the anti-phospho-histone 3 antibody as upon treatment, the percentageof cells with phosphorylated histone 3 increased as expected (Figure 7d).

Eukaryotic cell lines

Policy information about cell lines and Sex and Gender in Research

Cell line source(s)	U2OS (CLS, 300364; RRID:CVCL_0042; sex: female)<br>C3H10T1/2 cell line (ATCC, CCL-226, RRID:CVCL_0190)<br>HEK293T (ATCC, CRL-11268; RRID:CVCL_1926, sex: female)<br>HCT116 (DSMZ, ACC 581; sex: male)
Authentication	Authentication not performed
Mycoplasma contamination	All cell lines were regularly tested for mycoplasma contamination using the MycoAlert (Mycoplasma Detection Kit, Lonza, LT07-218) according to manufacturer's instructions and were always found to be free of micoplasma.
Commonly misidentified lines (See ICLAC register)	No commonly misidentified cell line was used in this study.

Flow Cytometry

Plots

Confirm that:

☒ The axis labels state the marker and fluorochrome used (e.g. CD4-FITC).

☒ The axis scales are clearly visible. Include numbers along axes only for bottom left plot of group (a 'group' is an analysis of identical markers).

☒ All plots are contour plots with outliers or pseudocolor plots.

☒ A numerical value for number of cells or percentage (with statistics) is provided.

Methodology

Sample preparation	For cell cycle analysis by flow cytometry, the Click-itTM Plus EdU Alexa FluorTM 488 Flow Cytometry Assay Kit (Thermo Fisher Scientific, Cat. No. C10632) was used according to the manufacturer's protocol. For this, 1.25 x 105 U2OS cells were seeded per well in a 6-well plate and incubated overnight. The following day, cells were treated with the compounds or with DMSO as a control and incubated for 22 h. Afterwards, cells were pulsed with 10 µM EdU (5-ethynyl-2´-deoxyuridine) or medium as a control and incubated for another 2 h. Cells were washed with PBS, detached using trypsin, re-suspended in PBS and

	centrifuged at 300 xg for 7 min at room temperature. After another washing step with 1 % BSA in PBS, cells were fixed with 4 % paraformaldehyde (PFA) in PBS, permeabilized and subjected to a click-reaction to label the incorporated EdU. All centrifugation steps after fixation were performed at 900 xg for 7 min at room temperature. The DNA content was stained with a propidium iodide solution (100 µg/mL propidium iodide, 0.1 % (v/v) Triton X-100 and 100 µg/mL DNase-free RNase A in PBS) for 30 min at room temperature. Before analysis, the cell suspensions were filtered into FACS tubes through a nylon mesh.
Instrument	BD LSRII analyzer (Becton Dickinson, USA)
Software	FlowJo 10.7.2
Cell population abundance	10,000 U2OS cells were analyzed per sample.
Gating strategy	SSC-A vs. FSC-A and FSC-W vs. FSC-A was used to separate cells from debris and to select single cells. Cells were finally separated into cell cycle gates (2N, S Phase, 4N) based on EdU-Alexa Fluor 488 and propidium iodide (Supplementary Figure 22).

☒ Tick this box to confirm that a figure exemplifying the gating strategy is provided in the Supplementary Information.
